# Extraordinary expansion of a *Sorangium cellulosum* genome from an alkaline milieu

**DOI:** 10.1038/srep02101

**Published:** 2013-07-01

**Authors:** Kui Han, Zhi-feng Li, Ran Peng, Li-ping Zhu, Tao Zhou, Lu-guang Wang, Shu-guang Li, Xiao-bo Zhang, Wei Hu, Zhi-hong Wu, Nan Qin, Yue-zhong Li

**Affiliations:** 1 State Key Laboratory of Microbial Technology, School of Life Science, Shandong University, Jinan 250100, China; 2 Beijing Genomics Institute, Shenzhen, 518083, China

## Abstract

Complex environmental conditions can significantly affect bacterial genome size by unknown mechanisms. The So0157-2 strain of *Sorangium cellulosum* is an alkaline-adaptive epothilone producer that grows across a wide pH range. Here, we show that the genome of this strain is 14,782,125 base pairs, 1.75-megabases larger than the largest bacterial genome from *S. cellulosum* reported previously. The total 11,599 coding sequences (CDSs) include massive duplications and horizontally transferred genes, regulated by lots of protein kinases, sigma factors and related transcriptional regulation co-factors, providing the So0157-2 strain abundant resources and flexibility for ecological adaptation. The comparative transcriptomics approach, which detected 90.7% of the total CDSs, not only demonstrates complex expression patterns under varying environmental conditions but also suggests an alkaline-improved pathway of the insertion and duplication, which has been genetically testified, in this strain. These results provide insights into and a paradigm for how environmental conditions can affect bacterial genome expansion.

Prokaryotic genomes may be as small and simple as the 140-kilobase (kb) genome of *Hodgkinia cicadicola*^1^ or as large and complex as the 13.03-megabase (Mb) genome of *Sorangium cellulosum* So ce56^2^. Whereas a bacterial genome can be reduced in size to accommodate a host or a simple life cycle, genome expansion may suggest the evolution of complex socialized living patterns and adaptation to variable environments^3^,^4^,^5^. Many studies have focused on estimating the minimum gene set required for life^6^,^7^. However, fewer studies have considered the expansion of prokaryotic genomes^4^, and we know little about the upper limits of bacterial genome size and the effects of the environment on genome variation.

Myxobacteria are well known for their social behavior and as producers of secondary metabolites^8^,^9^. These microorganisms inhabit almost every environment on earth, including soils, river mud, deep-sea sediments, and hydrothermal vents^10^,^11^,^12^. Although the anaerobic myxobacteria, which at present consist of only one genus, *Anaeromyxobacter*, have simple life cycles and relatively small genomes (5–6 Mb)^13^, all of the sequenced aerobic myxobacteria have genomes larger than 9 Mb, including the 13.03 Mb genome of *S. cellulosum* So ce56^2^. The relatively large genome sizes of aerobic myxobacteria seem to be consistent with their complex social activity and comprehensive environmental adaptation. Indeed, these bacteria possess complex regulatory networks consisting of many sigma factors and kinases that respond to fluctuating environments^14^,^15^. Several studies have discussed genome expansion^2^,^14^,^15^, but the underlying mechanisms or the effects of environmental conditions on bacterial genome expansion are still puzzling evolutionary questions.

Myxobacteria, including *S. cellulosum*, are typically found in soil with a neutral pH^10^,^16^. In the laboratory, the optimal pH range for the growth and development of myxobacteria is normally ranged from 6.5 to 8^17^. In our previous studies for the isolation of myxobacteria, we obtained a *S. cellulosum* strain, So0157-2, from an alkaline soil sample^18^,^19^,^20^. So0157-2 produces epothilones, cytotoxic macrolides that stabilize microtubules, mimicking the effects of paclitaxel on cancer cells^21^. More than 20 epothilone derivatives have been identified in So0157-2, including a novel glycosylated epothilone, epoAG^22^, a promising candidate for clinical application. The strain is an alkaline-adaptive epothilone producer that grows across a wide pH range (5.0–14.0). In this study, we sequenced the genome of So0157-2 using next-generation sequencing techniques and measured and compared its transcriptomes under different pH conditions. The data suggest that multiple internal and external factors affect genome expansion. Most notably, the data suggest the existence of an unique alkaline pH-improved pathway formed through the incorporation of exogenous genetic materials and the duplication of internal genes.

## Results

### Characteristics of *Sorangium cellulosum* So0157-2 and its genome

**Figure 1:**
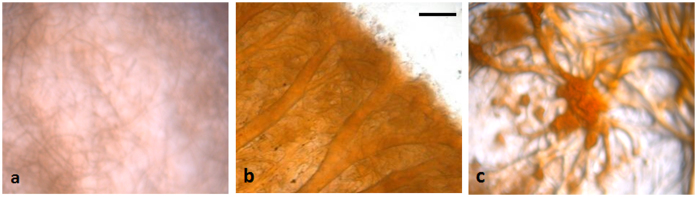
Colony morphologies of *S. cellulosum* strain So0157-2 on CNST medium with different pH values. (a) pH 6.0; (b) pH 9.0; (c) 11.0. Bar = 100 μm.

**Figure 2:**
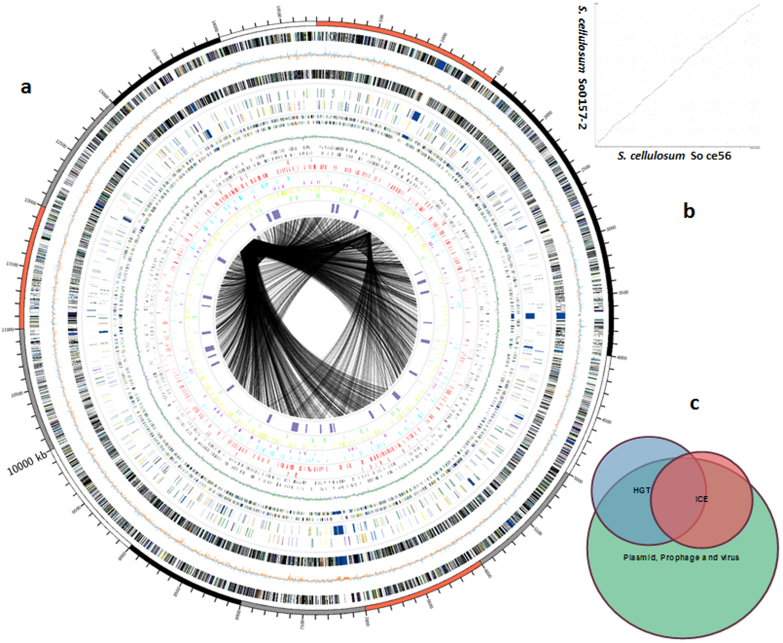
Genomic features of *S. cellulosum* So0157-2. (a) The genomic organization of the *Sorangium cellulosum* So0157-2 strain. Circle 1, genome positions in kb (from *dnaA*); Circles 2 and 4, predicted protein coding sequences (CDSs) on the forward (outer wheel) and the reverse (inner wheel) strands, colored according to COG class (leading strand, 5,825 CDSs, 49.9% of the total CDSs; lagging strand, 5,848 CDSs, 50.1% of the total CDSs); Circle 3, GC skew; Circles 5 and 6, putative ICE (integrative conjugative element)-derived CDSs (leading strand, 456 CDSs; lagging strand, 485 CDSs, 8.06% of the total CDSs); Circles 7 and 8, putative plasmid-derived CDSs (leading strand, 2,434 CDSs; lagging strand, 2,355 CDSs, 41% of the total CDSs); Circle 9, GC content showing deviations from the average (72.1%); Circles 10 and 11, putative HGT (horizontal gene transfer)-related genes (leading strand, 630 CDSs; lagging strand, 613 CDSs, 9.86% of the total CDSs); Circle 12, CDSs with regions showing high identity to virus genes; Circle 13, CDSs with regions showing high identity to prophage genes; Circle 14, putative restriction and modification system genes; Circle 15, two-component system genes in the genome (leading strand, cyan; lagging strand, purple); Circle 16, 109 sigma factor genes and 347 related transcription factors in the genome (leading strand, yellow; lagging strand, green); Circle 17, 55 CDSs with DNA-binding regions (green); Circle 18, secondary metabolite biosynthesis genes (dark purple), 10.6% of the whole genome; Innermost circle, putative paralogous genes in the genome. (b) Syntenic map between *S. cellulosum* So0157-2 and So ce56. (c) HGT (blue), ICE (red) and Plasmid, prophage and virus (green) are the three main mechanisms that have introduced alien genetic materials into the *Sorangium* genome. Generally, most ICEs fall into the green circle (932/941), whereas approximately 3/4 of the HGT genes lie in the green circle (908/1,268). A total of 197 genes are shared between the ICE and HGT groups. 193 genes are common to all three groups. About half of genes could be designated as alien genetic material (5,129/11,599).

The So0157-2 strain was isolated from soil collected from the bank of an alkaline lake (pH 9.0) in Yunnan Province, China. The strain grows well on mineral medium with filter paper as the only carbon source (CNST medium)^23^ in a pH range from 5.0 to 14.0, with optimal growth in alkaline conditions (pH 8.5–10.0). On CNST medium, the alkaline-adaptive *S. cellulosum* So0157-2 cells aggregated to form mound-like structures, but they did not form fruiting bodies. When the pH of the medium was adjusted to 9.0 or higher, myxospores were observed with rare and loosely organized sporangioles (Fig. 1). Assembly of the next-generation sequencing data defined the *S. cellulosum* So0157-2 genome as a circular 14,782,125 bp sequence*,* making it the largest prokaryotic genome described to date. There was no evidence of extra-chromosomal genetic materials in So0157-2. In fact, plasmids have not been identified in the myxobacterial group, with the exception of pMF1 from *Myxococcus fulvus* 124B02^24^. The So0157-2 genome is 1.75 Mb larger than the largest previously reported bacterial genome from *S. cellulosum*. The 11,599 predicted protein coding sequences occupy 89.2% of the So0157-2 genome (Fig. 2A). These CDSs are rich in the genes that encode proteins responsible for polysaccharide degradation, secondary metabolite production, cell motility and chemosensory systems. Even complete aerobic and anaerobic electron transfer chains are both included in the genome (Supporting text). Bioinformatics analysis revealed a total of 5,541 and 4,591 CDSs that are annotated with clear COG classification in the genomes of *S. cellulosum* strains So0157-2 and So ce56, respectively. Generally, except the translation (J) and defense (V) systems, no significant difference was detected in COG number as the CDSs increased in So0157-2 (Table 1). It is known that genome expansion can occur via acquisition of exogenous genetic materials by horizontal gene transfer (HGT) or by genome duplication^25^,^26^. Core genome analysis showed more duplication events occurred in So0157-2 than that in So ce56. In addition, the So0157-2 strain possesses more strain specific genes, which were extremely biased with the increase of CDS. The results suggest that the So0157-2 genome was greatly expanded during the evolution history to its external milieu.

### Genome expansion pathways

Exogenous genetic materials can be categorized based on their probable origin, including phages, prophages, integrated plasmids, integrative conjugative elements (ICE), insertion sequence elements (ISE) and other unclassified sources. In the So0157-2 genome, the primary sources of HGT were plasmids and ICEs, with other sources also yielding minor contributions (Supporting text). The detection of 4,789 putative HGT events from plasmid source CDSs (Fig. 2A, Circles 7 and 8) suggests that plasmids have integrated into the So0157-2 genome frequently over a long evolutionary period, probably by means of transformation or conjugation. Although nearly 50% of the genes in the genome had unknown functions, most of the plasmid-related CDSs (85.8%) were functionally annotated; only 680 plasmid-derived genes were assigned hypothetical functions. 248 of the total 508 serine/threonine phosphatases, 353 of 557 polysaccharide-degrading enzymes (including 180 of the 220 that contain CBM motifs), genes involved in heavy metal metabolism, various types of transporters and even a full set of cytochrome c oxidases were originated from plasmids. In addition, the CDS of a set of 19 DNA repair genes and many biosynthetic gene clusters for secondary metabolites, including 33 NRPS and 27 PKS CDSs, were also found to have been transferred into the So0157-2 genome from putative plasmid sources. ICEs have been found in both Gram-positive and Gram-negative bacteria^27^. They are a diverse group of mobile genetic elements that employ a range of mechanisms to promote their core functions of integration, excision, transfer and regulation^28^. In So0157-2, 941 CDSs were predicted to correspond to ICEs (Fig. 2A, Circles 5 and 6). Integrases, transposases, mobile element proteins, and antibiotic resistance proteins were included in this category.

Duplication can involve either large genome regions or be limited to individual genes. However, the whole-genome alignment showed no obvious evidence of large regions of duplication in So0157-2 (Supplementary Fig. S1). A comparison of all of the predicted 11,599 CDSs to one another using the BLASTP program revealed that 39.5% (4,587) of the CDSs constitute 1,265 families of paralogous genes (two or more members in each family). These families most likely arose via gene duplication or horizontal gene transfer. The largest gene family contains protein kinases (648 members), the second largest family includes members of ion, heavy metal and antibiotic transporter systems (113 members), the third largest family contains three paralogous groups encoding sigma factors (housekeeping sigma factors and ECF sigma factors) and related regulatory proteins (188 members), and the fourth largest family represents secondary metabolism (97 members). Interestingly, many duplicated genes were derived from two core regions (Fig. 2A, the innermost circle). The larger duplication hot spot contained sets of CDSs that were essential to cellular activities, including nearly all of the ribosomal protein-related CDSs and a series of genes responsible for DNA replication and nucleotide metabolism.

### Regulatory network

Upon genome expansion, efficient gene regulation and proper cell functions require the integration of a complex regulatory network into the genome (Supplementary Fig. S2). Since the first report of eukaryote-like protein kinases (ELKs) in *Myxococcus xanthus* DK1622^29^, these kinases have been shown to phosphorylate serine, threonine and tyrosine sites to regulate development, virulence, metabolism, or stress adaptation in many bacterial species^15^. Surprisingly, we identified 508 putative ELKs in *S. cellulosum* So0157-2, including various annotated serine/threonine/tyrosine protein kinases and hypothetical serine/threonine/tyrosine proteins (Supplementary Table S1). This tally exceeded the 317 EKLs annotated in So ce56, which was previously the largest known set of ELKs in prokaryotes^2^,^15^. Of the 306 ORFs encoding two-component system (TCS) proteins in So0157-2, 202 had homologs in *Myxococcus xanthus* DK1622^30^. In addition, So0157-2 encoded 109 sigma factors, 6 anti-sigma factor proteins and 347 sigma factor-related proteins (Fig. 2A, circle 16). These regulatory genes were widely distributed across the So0157-2 genome. So0157-2 cells functioned very well in different environmental conditions, and this was likely supported by, in addition to the large number of ELKs, an abundance of sigma factors and related transcriptional regulatory factors, which allow the initiation of transcription to be specific and flexible.

### Immune systems

Restriction and modification (R&M) systems, having three types^7^, and the clustered regularly interspaced short palindromic repeats (CRISPRs) and their associated (Cas) proteins^31^,^32^ have been established as efficient barriers against both HGT^32^,^33^ and genetic manipulation^34^,^35^. Database searches revealed a diverse collection of R&M CDSs in So0157-2 (Fig. 2A, circle 14). After manual validation, 17 putative type I complex genes were identified in So0157-2. Of these, 6 encoded sequence recognition (S) polypeptides, 7 encoded methylation (M) polypeptides and 4 encoded restriction (R) polypeptides. However, only 3 complete type I RM gene complexes were identified. Of the type II complex, 5 R genes and 3 M genes were identified, but only one complete type III gene complex was identified in the So0157-2 genome. In addition, three mobile element proteins were annotated as homing endonuclease homologs. More interestingly, there were fewer R&M systems, CRISPRs and *cas* genes in the So0157-2 strain than in So ce56 (Supplementary Table S2). For example, So ce56 harbored 12,726 bp CRIPSR sequences and 11 *cas* genes (5 in *cas* cluster and 6 in *cmr* cluster), whereas the So0157-2 genome only contained 6,572 bp CRISPR sequences and 3 *cas* genes (*cas1*, *cas2* and *cas3*). So0157-2 seems to lack a complete array of the three major types of CRISPR/Cas systems^32^. It is possible that the absence of an effective immune system may allow invasive genetic materials to integrate into the host chromosome.

### Comparative transcriptomics analysis

So0157-2 is a soil-dwelling bacterium. The strain grows across a broad pH range (5.0 to 14.0), with optimal growth in alkaline conditions. To investigate the potential relationships between genome expansion and the specific external *milieu*, we performed a comparative transcriptomics analysis to evaluate changes in gene expression and regulation. Expression profiles were obtained from cultures of So0157-2 grown on CNST medium at pH 7.0 and pH 9.0. With a range of 7.9–8.6 million reads per sample, the RNA-Seq analysis detected 10,518 transcripts, accounting for 90.7% of the 11,599 CDSs annotated in the So0157-2 genome. A total of 1,352 genes were silent at pH 9.0 and 744 were silent at pH 7.0; of these, 739 genes were silent in both conditions. Technical validation of the RNA-Seq data was performed by RT-quantitative PCR (qPCR) using eight genes with distinct changes in expression under the two conditions (Supplementary Table S3). There were good correlations between the qPCR results and the RNA-Seq results for the genes of SCE1572_6018, SCE1572_6365, SCE1572_7438 and SCE1572_7815 (Pearson's correlation, R^2^ = 0.998; p = 0.002, two-tailed test). The RNA-Seq reads of these four genes were all greater than 100 at either pH 7.0 or pH 9.0 (Supplementary Table S3). Meanwhile, the expressions of other two tested genes showed similar trends with the RNA-Seq results. However, the qPCR result of SCE1572_8473 was quite unstable, probably because of low RNA-Seq reads of the gene (153/25 at pH 7.0/pH 9.0). Cristino, *et al.* pointed out that low RNA-seq reads often caused poor correlations between the results of qPCR and RNA-Seq^36^. Furtherore, Griffith, *et al.* once validated expressions of 381 genes using qPCR and found that 88% of the tested genes exhibited correlations with the results of RNA-Seq, whereas the other 12% genes had poor correlations between the results of RNA-Seq and qPCR^37^. Accordingly, the qPCR results supported the RNA-Seq results.

**Figure 3:**
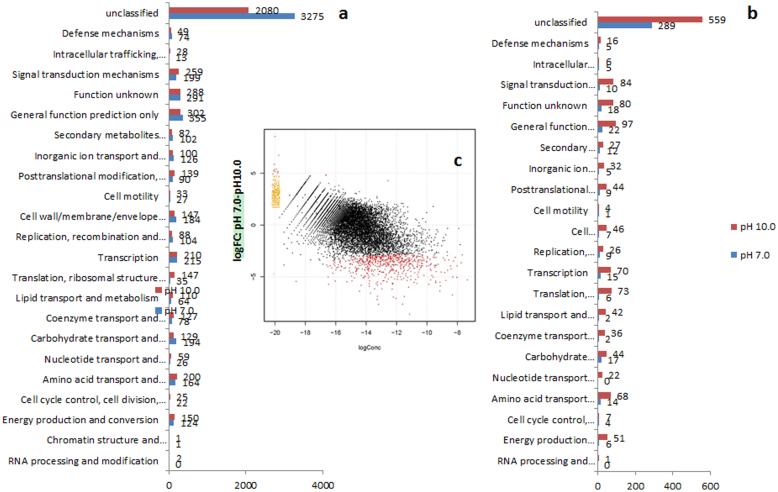
Transcriptomic analysis of *S. cellulosum* So0157-2 in pH 7.0 and pH 9.0 conditions. (a) Categories of genes that are differentially expressed at pH 7.0 and pH 9.0. (b) Categories of significantly differentially expressed genes at pH 7.0 and pH 9.0. All detected transcripts were characterized by clusters of orthologous groups (COG) categories. (c) Statistical analysis of gene expression. Plots of the log2 ratio (fold-change) vs. the mean log expression values under pH 7.0 and pH 9.0 conditions. Red dots indicate the differentially expressed genes at a 5% false discovery rate. The yellow and red dots in the upper left corners of the two panels indicate the genes with the largest log fold changes.

There were 5,763 up-regulated genes at pH 7.0, whereas 4,755 genes were up-regulated at pH 9.0. However, differential expression analysis of the RNA-Seq data revealed that 1,894 genes were differentially expressed (log fold-change > 1.5, P-value < 0.05 and FDR < 0.05). Of these, 1,435 genes were up-regulated at pH 9.0, whereas only 459 were up-regulated at pH 7.0 (Supplementary Table S4). More genes that are important for cell growth (according to their COG category) were up-regulated at pH 9.0 than at pH 7.0 (Fig. 3). For example, genes involved in translation, amino acid and sugar metabolism, and the ribosome were generally up-regulated at pH 9.0, as well as were the genes involved in nucleotide metabolism, cell wall/membrane/envelope biogenesis and replication/recombination/repair processes. Consistent with the up-regulation of the secondary metabolisms at pH 9.0, some drug efflux pump and macrolide export transporters were also significantly up-regulated. Furthermore, the genes encoding electron transfer chain components, including two subunits of the electron transfer flavoprotein and five subunits of the NADH-ubiquinone oxidoreductase complex, were up-regulated at pH 9.0. In addition, at pH 9.0 a whole set of ATP synthase operon-including genes (encoding α-, β-, γ- and δ-chains) was up-regulated. Interestingly, despite being thought to play a dominant role in pH homeostasis in individual bacterial cells^38^, no Na^+^/H^+^ anti transporter, was significantly up-regulated at pH 9.0 (but one was at pH 7.0). However, a total of 56 transporter genes were up-regulated at pH 9.0 whereas 19 transporter genes were up-regulated at pH 7.0.

Sigma factors and transcription factors are a group of proteins that bind to DNA and help initiate or repress gene transcription^39^,^40^. In So0157-2, 16 sigma factors were significantly up-regulated at pH 9.0, including *rpoD*, *rpoE*, *rpoH*, sigma-54 factor *rpoN* and three other ECF sigma factors. In contrast, 2 sigma factors and 7 transcriptional factors were up-regulated at pH 7.0, although the read counts were rather low (Supplementary Table S4). A number of transcriptional regulators were also significantly up-regulated at pH 9.0, and some showed large fold-changes. For example, two transcriptional regulators in the TetR family were among the most highly up-regulated genes, being up-regulated 117-fold and 56-fold at pH 9.0. In addition, at pH 9.0, a total of 41 ELKs were up-regulated by an average of 10-fold over the expression at pH 7.0. By contrast, only 3 ELKs were up-regulated at pH 7.0.

Consistent with the vigorous growth observed at pH 9.0, a large number of significantly up-regulated genes at pH 9.0 were related to DNA replication, expression, post-transcriptional modification, translation, and post-translational modification. For example, two of the four RNA binding proteins were up-regulated 33.1-fold and 39.1-fold at pH 9.0. Moreover, five of the genes involved in homologous recombination were up-regulated at pH 9.0, whereas only the gene encoding RadC was up-regulated at pH 7.0. Similar to the response of *Escherichia coli* to the shift to an alkaline environment^38^, the gene encoding LexA, an SOS-response repressor and protease, was up-regulated 38.2-fold at pH 9.0. Interestingly, whereas the average normalized RNA-Seq counts for pH 7.0 and pH 9.0 were 56.74 and 210.52, respectively, the average counts of *cas1*, *cas2* and *cas3* in the CRISPR system were 33.22 at pH 7.0 but 14.28 at pH 9.0. The expressions of two of the three homing endonucleases were also up-regulated at pH 7.0. In contrast, all of the 36 R&M system CDSs were expressed in both pH conditions; 10 were up-regulated at pH 9.0, and 26 were up-regulated at pH 7.0.

To verify alkaline conditions weaken bacterial repair and immune systems, and thus allow easier gene transfer, we performed a comparative conjugation experiment under alkaline conditions (pH 9.0) and neutral conditions (pH 7.2). The results showed that the conjugative efficiency at pH 9.0 was proximately 10 times higher than that at pH 7.2 with the same insert size.

## Discussion

Environmental pressure is the driving force for natural selection^41^. Complex and fluctuating environments require an omnipotent genome and complex regulation systems, even a collaborative sociality, such as in a biofilm community, for a microorganism to adapt in. The *S. cellulosum* So0157-2 genome is 1.75 Mb larger than that of the So ce56 strain, but it possesses one-third more predicted CDSs, suggesting that the genome of this strain has been shaped by the complex habitat that this strain has encountered. For example, 557 genes in the So0157-2 strain, more than twice the number in the So ce56 strain, were predicted to encode various polysaccharide-degrading enzymes (Supplementary Fig. S3). Transcriptomics analysis showed that most of the identified CDSs were expressed, suggesting that the So0157-2 strain has evolved via HGTs to meet the requirements for survival in complex environmental conditions. Indeed, all of the sequenced large bacterial genomes are from complex milieu (Supplementary Table S5). As noted by Pérez et al., the numbers of eukaryote-like kinases increase exponentially with the genome expansion in the myxobacterial group, whereas the numbers of two-component systems (R^2^ = 0.81), sigma factors and related regulatory factors (R^2^ = 0.96) increase linearly (Supplementary Fig. S2)^15^. This observation underscores the flexibility and adaptability of the *Sorangium* species. The increased gene numbers and functional demands in varying environments also require the proper folding of the produced peptides. In *M. xanthus* DK1622, two copies of the *groEL* chaperone system have divergent functions supporting different cellular processes^42^,^43^. There are also two copies of the *groEL*/*groES* system in So ce56. However, the So0157-2 genome has an additional, third copy of *groEL* accompanied by an HSP20 family protein, possibly derived from *Bradyrhizobium japonicum* or a related Rhizobiales bacterium. Transcriptional analysis showed that all three *groEL* genes expressed at different levels in different conditions.

The So0157-2 strain was isolated from alkaline soils near an alkaline lake (pH 9.0), and tolerant against a broad pH range. A comparative transcriptomics analysis revealed significant differences in genome-wide expression patterns between alkaline and neutral environments. Alkaline adaptation in *E. coli* has resulted in the up-regulation of genes encoding ATP synthase, thereby maximizing proton retention and proton capture by the cell, and the repression of genes involved in cell division and nucleotide biosynthesis, leading to the cessation of growth^38^. The expression profile of the alkaline-adaptive So0157-2 at pH 9.0 showed the up-regulation of genes for ATP synthase, translation, replication, cell division, transporters, energy metabolism and acid production, which was consistent with the vigorous growth of the strain in alkaline conditions. Furthermore, the R&M system and the CRISPR/Cas system were significantly reduced in the So0157-2 genome. Interestingly, these two systems expressed at low levels at pH 9.0, whereas members of the DNA repair system (*recA*, *recR*, *ruvB* and *ruvC*, and *lexA*) were up-regulated at pH 9.0. This pattern of expression may increase the possibility of recombination or the integration of exogenous genetic materials into the genome. Indeed, genetic manipulation became easy under alkaline conditions. Horizontal gene transfer also depends on the uptake of DNA. A group of genes encoding transporters, including a component of the type IV pili, thought to be a transmembrane DNA channel in Gram negative bacteria^44^, was up-regulated at pH 9.0. These findings suggest that the So0157-2 strain has a variety of genetic resources that should enable genome expansion. Accordingly, we suggest that bacteria living in complex and changing environments have more internal and external opportunities to expand their genomes. Experiments performed under natural conditions for extended periods may reveal the mechanisms that underlie bacterial genome expansion.

## Methods

### Strains and culture conditions

So0157-2 is a cellulolytic myxobacterial strain that was isolated from the shoreline of an alkaline lake. The strain has been routinely cultivated on solid CNST agar plate^23^ and in liquid M26 medium^45^ at 30°C. For transcriptome analysis, the strain was plated on solid CNST agar from cryovials stored at −80°C with cellulosum (filter paper) as the only carbon source. The pH of the medium was adjusted to 7 or 10 using PBS buffer (pH 7.0, 61.5 mL 1 mol/L K_2_HPO_4_, 38.5 mL 1 mol/L KH_2_PO_4_) or boric acid buffer (pH 10, 250 mL 0.05 mol/L boric acid, 215 mL 0.2 mol/L NaOH, volume brought to 1 L), respectively.

### DNA extraction, library construction and sequencing

The sorangial bacteria were harvested from 5-day cultures in M26 medium, washed with distilled water and suspended in STE solution containing 0.1 mmol/L NaCl, 10 mmol/L Tris–HCl (pH 8.0) and 1 mmol/L EDTA. Genomic DNA was extracted as previously described^46^ or using the TIANamp Bacteria DNA Kit (TIANGEN BIOTECH CO., LTD., Beijing, China). One shotgun library and seven additional insertion libraries were constructed for paired-end sequencing (Supplementary Table S6).

The genome sequence of *Sorangium cellulosum* So0157-2 was determined with a hybrid next-generation sequencing strategy. Seven Illumina® Solexa® HiSeq® 1000 sequencing runs (performed at the Beijing Genomics Institute, Shenzhen, China) and three 454® whole-genome shotgun sequencing runs (performed at the Genome Institute at Washington University, St. Louis, MO, USA) using different sizes of sequencing libraries were conducted to obtain a fine map of the So0157-2 genome. Synteny-guided gap closure was performed for some contigs via PCR and direct sequencing using primers designed to anneal to each end of the neighboring contigs. Multiplex PCR was carried out from the ends of contigs with no synteny information. One final scaffold was assembled. A total of 87 regions with low coverage (<3 fold) were verified via PCR. A previously constructed fosmid library was used to correct the contigs by end-sequencing.

### Genome assembly and feature annotation

The methods used for genome assembly, general feature annotation and annotation of secondary metabolite pathway, paralogous genes, mobile genetic elements, restriction and modification system, Clustered Regularly Interspaced Short Palindromic Repeats, sigma factors, transcriptional factors and protein kinase were listed in supplementary files (Supplementary methods). The *S*. *cellulosum* So0157-2 genome sequence has been deposited to GenBank under accession number CP003969.

### Genome synteny

The synteny between *S. cellulosum* So0157-2 and *S. cellulosum* So ce56 was analyzed with a Perl script utilizing a bidirectional BLAST search^47^, and a synteny map was drawn by r2cat^48^.

### RNA extraction, library construction and sequencing

The So0157-2 culture was collected from filter paper after 5 days cultivation. Total RNA was prepared using the MICROBExpress Bacterial mRNA Enrichment kit (Life Technologies, Grand Island, NY, USA) following the manufacturer's instructions. rRNA removal was evaluated using the Agilent 2100 Bioanalyzer (Agilent Technologies, Inc., Santa Clara, CA, USA). Enriched mRNAs were chemically fragmented to produce sequences of 200–250 bp with 1× fragmentation solution (Life Technologies, Grand Island, NY, USA) for 5 min at 70°C. cDNA was generated using the SuperScript II Double-Stranded cDNA Synthesis Kit with random hexamer primers (Life Technologies, Grand Island, NY, USA). The Illumina Paired End Sample Prep kit (Illumina, Inc., San Diego, CA, USA) was employed for RNA-Seq library creation according to the manufacturer's instructions. Fragmented cDNA was end-repaired, ligated to Illumina adaptors, and amplified through 10 cycles of PCR. Single or paired-end 90 bp reads were generated by the Illumina Genome Analyzer II instrument (Illumina, Inc., San Diego, CA, USA).

### Transcriptomic data assembly and annotation

We conducted a comparative transcriptome analysis of the cultures on CNST medium (mineral medium with cellulose as the only carbon source) at pH 9.0 and pH 7.0. The raw data were first aligned to the So0157-2 genome. Removal of rRNA and tRNA sequences was conducted with Bowtie 2^49^. RNA-Seq reads were aligned to the reference genome using the Burrows-Wheeler Aligner^50^. Read counts were determined for each library on a per-gene basis. We normalized the raw read counts by dividing by a size factor for each library, as previously suggested^51^,^52^, such that the median fold-change between libraries was approximately 1. Because longer transcripts generate more RNA-Seq reads, the normalized read counts were further divided by the length of the gene in kilobase pairs to allow comparisons across genes and comparisons with qPCR data.

### Differential expression of CDS

Pair-wise differential expression analysis between bacteria cultured at pH 7.0 vs. pH 9.0 was performed using the RSEM (http://deweylab.biostat.wisc.edu/rsem/) and the R package edgeR (http://www.bioconductor.org/packages/2.10/bioc/html/edgeR.html)^53^, available from Bioconductor (www.bioconductor.org). EdgeR normalizes the raw counts using size factors, as described above. Because estimates of the variance per gene based on only two replicates are highly unreliable, edgeR employs an unbiased variance estimator (based on local regression against the mean expression level across the entire dataset), followed by a negative binomial model to test for differential expression. The resulting P values were adjusted for multiple hypotheses testing, controlling the false discovery rate^54^. Genes showing an adjusted P value of <0.05 and a fold-change greater than 1.5 were classified as differentially expressed. EdgeR output tables were included in the supplementary materials (Supplementary Table S4).

### RT-qPCR

cDNA was synthesized using the SuperScript II Double-Stranded cDNA Synthesis Kit with random hexamer primers (Life Technologies, Grand Island, NY, USA) according to the manufacturer's protocol. cDNA samples were used at 1:100 final concentration. Primers were used at 200 nM and are listed in Supplementary Table S3. Reactions in a 20-μL volume were run on the MyiQ™ Single Color Real-time PCR Detection System (Bio-Rad Laboratories, Hercules, CA, USA) using iQ™ SYBR Green SuperMix (Bio-Rad Laboratories, Hercules, CA, USA) mix according to the manufacturer's instructions. Relative transcript abundance was calculated and normalized with respect to the reference, gene encoding Urea ABC transporter, ATPase protein UrtD. Ratio of expression was quantified by the 2^−ΔΔCt^ method^55^.

### Conjugation between So0157-2 strain and *E. coli*

A former established protocol was used to conduct conjugation between So0157-2 strain and *E. coli* DH5α λpir^56^. An alkaline (pH 9.0) culture medium and a combination of low concentration of antibiotics (3 μg/ml chloromycetin and 15 μg/ml gentamicin) were employed to screen the conjugants.

## Author Contributions

Y.Z.L. and K.H. designed the study and wrote the manuscript. K.H., Z.F.L. and L.P.Z. sequenced the genome. K.H., R.P. and L.G.W. performed the transcriptome sequencing and conjugation experiments. T.Z., X.B.Z. and N.Q. performed partly the genome annotation. K.H. analyzed the data of genome and transcriptome. S.G.L. cultivated the strains used in this study. W.H. and Z.H.W. discussed the results and commented on the paper.

## Supplementary Material

Supplementary Information Supporting text

## Figures and Tables

**Table 1:** Comparison of COG assignments between *Sorangium cellulosum* So0157-2 and So ce56

	All features			Homologous genes			Strain specific genes		
	So0157-2	So ce56	P value	So0157-2	So ce56	P value	So0157-2	So ce56	P value
RNA processing and modification	2	3	0.812	2	3	0.6631	0	0	-
Chromatin Structure and dynamics	2	2	0.8315	2	2	0.9917	0	0	-
Energy production and conversion	281	264	0.0835	222	219	0.8352	59	45	0.057
Cell cycle control and mitosis	47	40	0.8967	35	34	0.9654	12	6	0.9514
Amino Acid metabolis and transport	365	310	0.5447	287	280	0.7009	78	30	0.202
Nucleotide metabolism and transport	89	83	0.3888	78	76	0.8839	11	7	0.8507
Carbohydrate metabolism and transport	324	233	0.1794	233	202	0.1159	91	31	0.054
Coenzyme metabolis	205	185	0.2979	174	164	0.5534	31	21	0.4085
Lipid metabolism	218	172	0.8472	167	148	0.2629	51	24	0.8047
Tranlsation	183	184	0.0398*	171	173	0.991	12	11	0.2379
Transcription	427	309	0.1396	283	251	0.1359	144	58	0.1242
Replication and repair	196	170	0.5345	125	136	0.5893	71	34	0.8012
Cell wall/membrane/envelop biogenesis	331	277	0.7	259	238	0.302	72	39	0.8803
Cell motility	61	53	0.7727	50	47	0.7985	11	6	0.9111
Post-translational modification, protein turnover, chaperone functions	231	197	0.614	176	170	0.7111	55	27	0.9271
Inorganic ion transport and metabolism	228	192	0.7129	175	160	0.3838	53	32	0.5509
Secondary Structure	184	139	0.5791	117	107	0.4934	67	32	0.8022
General function prediction only	961	802	0.5076	727	675	0.1004	234	127	0.6719
Signal Transduction	460	405	0.2152	347	343	0.7967	113	62	0.7493
Intracellular trafficing and secretion	41	37	0.7108	34	36	0.9393	7	1	0.3916
Defense mechanisms	123	70	0.0216*	67	61	0.6148	56	9	0.0009***
Function Unknown	582	464	0.8392	413	356	0.0260*	169	108	0.0857
Total	5541	4591	0.0936	4144	3881	<0.0001***	1397	710	0.0030**

Two-tailed statistical analysis was conducted by Chi square test with Yate correction.
