# Supplementary material for: Extraordinary expansion of a Sorangium cellulosum genome from an alkaline milieu
Source: Sci Rep. 2013 Jul 1;3:2101. doi: 10.1038/srep02101 (PMC3696898; doi:10.1038/srep02101)
Supplement: Supplementary Information — Supporting text [file srep02101-s1.pdf]

Supplementary Information for

Extraordinary expansion of a *Sorangium cellulosum* genome from an alkaline milieu

Kui Han, Zhifeng Li, Ran Peng, Liping Zhu, Tao Zhou, Luguang Wang, Shuguang Li, Xiaobo Zhang, Wei Hu, Zhihong Wu, Nan Qin, Yuezhong Li*

*Correspondence to: lilab@sdu.edu.cn

This PDF file includes:

Supporting Text
Supplementary Methods
References
Supplementary Figs. S1 to S3
Supplementary Tables S1 to S3, S5 to S6, S10, S12 to S15

Additiional Data (separate file)

Table S4 (separate file)
Table S7 to S9 (separate files)
Table S11 (separate file)

Supporting Text

Genome sequencing, assembly and annotation

A combined strategy of next-generation and traditional Sanger sequencing methodologies was applied. Seven libraries were constructed from the So0157 2 genomic DNA, with average insertion lengths ranging from 242 to 6000 bp. Assembly of the Solexa® sequenced libraries yielded 5749 Mb of clean data, 400 contigs and 41 scaffolds (Supplementary Table S6). Further co-assembly of an additional 2.5× 454 reads produced one scaffold with a 385-fold average depth of coverage. All of the regions with less than 3-fold coverage were validated by PCR and Sanger sequencing. Ultimately, we obtained the complete 14,782,125 bp circular genome sequence of *S. cellulosum* So0157-2, which is now the largest reported prokaryotic genome, being 1.75 Mb larger than the *S. cellulosum* so ce56 genome (13.03 Mb) [1].

The GC content of So0157-2 genome is 72.1% and it contains 11,599 predicted protein-coding sequences (CDSs). Of these CDSs, 5,175 (44.6%) were annotated for hypothetical proteins through the RAST pipeline. Circles 2 & 4 in Fig. 2A show the final map of the chromosome numbered beginning from the *dnaA* gene. The *S. cellulosum* So0157-2 and So ce56 genomes are largely collinear (Fig. 2B), and approximately two-thirds of the total CDSs in the So0157-2 genome (8224) have homologues in So ce56 (Supplementary Table S7). The remaining one third of the sequences are unique to So0157-2, and approximately 1,000 of its 9,367 CDSs are unique to So ce56 (Supplementary Table S8 & S9). As in So ce56, the GC-skew inversion (Fig. 2A, circle 3), which is the hallmark of a bacterial chromosome replication origin, is absent in So0157-2, and thus, the location of *oriC* cannot be determined.

Genome characteristics

Non-coding RNA. A total of 74 non-coding RNAs, including 62 tRNAs and 12 rRNAs, were identified in the So0157-2 genome. Similar to other myxobacterial genomes, the rRNA genes of So0157-2 are located in four operons. However, only one of the operons presents the typical 5S-16S-23S structure, whereas two are atypical: one either lacks or contains a duplicated 5S rRNA gene, and one is interrupted (Supplementary Table S10). In the genome of the So ce56 strain, the 23S rRNA gene was not interrupted. Two inverted repeat sequences—the "calling cards" of transposons—were identified downstream of the operon containing three consecutive 5S rRNAs. In the interrupted operon, the 23S rRNA gene was replaced with an isoquinoline 1-oxidoreductase beta subunit and a hypothetical protein, which were both encoded by the complementary strand. There were no obvious sequence features suggesting that these two genes resulted from transposition or insertion.

Polysaccharide-degrading enzymes. The So0157-2 strain can effectively degrade a range of polysaccharides, including xylan, starch, mannan, lichenan, avicel, chitin and artificial carboxymethylcellulose. Surprisingly, a total of 557 ORFs, accounting for 7.95% (1,175,488 bp) of the total So0157-2 genome, were predicted to encode proteins involved in polysaccharide degradation, and nearly half of them were predicted to function in cellulose degradation. These polysaccharide degradation related enzymes, which included glycoside hydrolases (GHs), glycosyltransferases (GTs), polysaccharide lyases (PLs) and carbohydrate esterases (CEs), belonged to 112 families, 85 of which had more than one homolog (Supplementary Fig. S3). A total of 220 carbohydrate-binding modules (CBMs) were distributed across 22 families. Five families comprised 161 of the CBMs, with 56,

20, 51, 18 and 14 CBMs identified in the CBM2, CBM6, CBM13, CBM48 and CBM50 families, respectively. These five CBM families were predicted to function in the degradation of amorphous cellulose (CBM6), chitin (CBM2, CBM50), xylan (CBM2, CBM6, CBM13), glycogen (CBM48) and peptidoglycan (CBM13, CBM50). Smaller families contained genes involved in the degradation of various other polysaccharides, such as starch, mannan, arabinofuranose and fucose.

In comparison, only 226 ORFs in the So ce56 genome were predicted to encode proteins responsible for polysaccharide degradation, *i.e.*, 114 GHs, 72 GTs, 11 PLs and 29 CEs. Most of these ORFs had homologues in So0157-2. Although each genome contained similar enzyme families, the number of enzymes was much greater in So0157-2 (Supplementary Fig. S3; Supplementary Table S11). Furthermore, although the composition ratios of the four enzyme classes (GH, GT, PL and CE) were rather consistent in So0157-2 and So ce56, the CBM compositions (161 vs. 40) were significantly divergent in these two strains. The richness and diversity of the ORFs related to polysaccharide degradation may explain the bacteria's excellent ability to degrade various polysaccharides, and also implies that it has adapted to its dwelling environments.

Motility-related genes. Myxobacterial cells move on solid surfaces by gliding in swarms, which are coordinated by two distinct, independent mechanisms: the social motility (S-motility) and adventurous motility (A-motility) systems [2]. In the well-studied myxobacterium *Myxococcus xanthus* DK1622, S-motility is determined by three components: the molecular pulling motor type IV pilus (TFP), exopolysaccharide (EPS) and lipopolysaccharide (LPS). A-motility may be driven by polyelectrolyte secretion at

the lagging cell pole [3] and/or multiple focal adhesion complexes distributed along the cell body [4]. Each system comprises many genes in *M. xanthus* DK1622. All of the related biosynthetic and regulatory genes were extracted from the *S. cellulosum* So0157-2 genome (Supplementary Table S12) for comparison. As previously reported in *S. cellulosum* So ce56 [5], LPS-related gene clusters, including those encoding lipid A, core oligosaccharide and O-polysaccharide, were partially absent in *S. cellulosum* So0157-2 (Supplementary Table S12). Former research shows that a 28-gene cluster is directly responsible for EPS biosynthesis and regulation in *M. xanthus* DK1622 [6]. In the *S. cellulosum* So0157-2 genome, with the exception of two transposases, one glycosyltransferase and nine hypothetical proteins, all of these EPS genes were present. However, unlike their proximal distribution in the DK1622 genome, these EPS biosynthetic and regulatory genes were scattered across the *S. cellulosum* So0157-2 genome, which may, if functional, require much more complicated regulation. Furthermore, some TFP biosynthesis genes that had clear functions in *M. xanthus* DK1622 (*e.g.*, *pilN*) [7] were also atypical in *S. cellulosum* So0157-2. However, *pilT*, a gene responsible for twitching motility [8], was present in five copies in the So0157-2 genome.

More than 35 genes or genome regions are involved in the function and regulation of A-motility in *M. xanthus* DK1622 [9]. In the So0157-2 and So ce56 genomes, with the exception of *mglA* (encoding a Ras-like protein) and the affiliated *mglB* gene, none of the A-motility-related genes have counterparts. These data suggests that the A-motility mechanisms differ between myxobacterial groups.

Chemotaxis systems. In *Myxococcus xanthus* DK1622, there are eight chemotaxis-like

gene clusters defining eight chemosensory pathways. Most of these chemosensory pathways play roles in controlling motility, and at least two pathways regulate gene expression during development [10]. In So0157-2, although all of the defined chemotaxis systems were present, they were each incomplete (Supplementary Table S13). For example, in the well-studied Frz system, only a methyltransferase (CheR) and two regulatory proteins (CheY and CheA) were annotated. A similar result was observed in the Dif system, where only CheA and CheY were annotated. The Che4 and Che5 systems in So0157-2 were structurally similar to the Frz system. The Che3, Che7 and Che8 systems exhibited included MCP (methyl-accepting chemotaxis protein), CheA, CheY, CheR, and an adaptor protein (CheW). In each of the eight chemotaxis systems, at least one histidine kinase (CheA) was annotated in the *S. cellulosum* So0157-2 genome. However, many copies of the components of the chemotaxis system were annotated in So0157-2. For example, 20 CheA proteins were annotated in the Che3 system and 10 CheA proteins were annotated in the Frz system. Notably, in contrast to the clustered chemotaxis systems observed in DK1622, only the Che6 and Che8 systems were organized in clusters, whereas the other systems were scattered throughout the So0157-2 genome.

Secondary metabolism. Myxobacteria can produce diverse bioactive compounds, and *Sorangium* strains contribute nearly half of the compounds produced by myxobacteria [11]. In the So0157-2 genome, we identified, 18 non-ribosomal peptide synthetase (NRPS) and polyketide synthase (PKS) gene clusters (420 kb, approximately 3% of the genome, Fig. 2A, circle 18; Supplementary Table S14) and a number of clusters responsible for the biosynthesis of molecules such as terpenes, indoles, and bacteriocin (approximately 7%

of the genome) (Fig. 2A, circle 18). The 18 NRPS/PKS clusters are organized into three types: 6 with PKS only, 5 with NRPS only and 8 with a mixed distribution of NRPS/PKS. Two PKS clusters exhibited a classical modular structure and may function iteratively; three were single module clusters, with features typical of type II PKS; and one contained 12 modules without an AT domain, which is responsible for substrate identification and specificity. Of the five NRPS clusters, four had typical modular structures that function sequentially or iteratively, and one was atypical in that it contained only one module, which may be functionally irregular. The PKS/NRPS hybrids, including the epothilone biosynthetic gene cluster, represent a significant feature, endowing these compounds with unique bioactivity in myxobacteria [12].

Electron transfer chain. Myxobacteria phylogenetically fall within the δ-proteobacteria, in which most members are strictly anaerobic [13]. An analysis of *M. xanthus* DK1622 shows a chimeric Complex VI electron transfer chain [14]. In *S. cellulosum* So0157-2, two electron transfer systems likely exist (Supplementary Table S15). The genes responsible for electron transfer were divided into several gene clusters surrounded by a series of hypothetical proteins. There was no evidence to suggest that these genes may have resulted from duplication events. *S. cellulosum* So0157-2 and So ce56 shared almost all of their electron transfer chain genes, suggesting that these strains share an early common ancestor. In the *S. cellulosum* genome, the two complete sets of genes responsible for electron transfer to the final acceptor were identified, suggesting that these genes were functional within bacterial cells. In the sequenced myxobacteria, two sets of Complex II succinate dehydrogenase genes. One of these genes arose from the Firmicutes group, but the other was homologous to the corresponding gene in high GC-content Gram-positive

bacteria. Interestingly, *Anaeromyxobacter* and *Sorangium* possess both types of succinate dehydrogenase, whereas *Myxococcus*, *Stigmatella*, and *Corallococcus* have only one type. Two sets of In Complex I NADH dehydrogenases were also identified in the So0157-2 genome. One complete set, comprising 15 components clustered in four groups, was detected; however, the second gene set was incomplete. These two Complex I gene sets originated from proteobacteria and bacteroidetes, respectively. Similar to Complex II, the complete set was common among myxobacteria species, whereas the incomplete set was only present in the anaerobic myxobacterial species and *Sorangium*. The Complex IV is relatively conserved among myxobacteria, and it mainly comprises two sets of heme-copper cytochrome *c* oxidases, one set of cytochrome *bd* quinol oxidases and an alternative cbb_3-type cytochrome *c* oxidase. However, the cbb_3-type cytochrome *c* oxidase was generally missing in the respiratory chain of *S. cellulosum*.

Phage invasion. Microbial genomics has revealed that a substantial number of bacterial genomes include prophage or virus sequences [15]. In So0157-2, 423 putative prophage CDSs and 62 viral CDSs were detected (Fig. 2A, circle 12 & 13). Of the annotated prophage-origin genes, 55 genes were hypothetical, and 367 genes were assigned specific functions. This ratio (367/422) is extremely high. Of the annotated serine/threonine protein kinase CDSs, 63 were predicted to have originated from prophages. Additionally, ATP-dependent proteases (13 CDSs), transcriptional regulators (8 CDSs) and some CDSs related to DNA/RNA metabolism also originated from phages. Putative virus CDSs were scattered throughout the So0157-2 genome. These CDSs included virus structural genes, DNA/RNA metabolism-related genes (*e.g.*, the 3'-to-5' exoribonuclease RNase R, poly(A) polymerase, ribonucleotide reductase, RNA-2',3'-PO4:RNA-5'-OH ligase, and a single-

stranded-DNA-specific exonuclease), and four CDSs of unknown function. Finally, several PKS-related genes had viral homologues. However, none of the prophage or virus sequences was active in the So0157-2 genome.

Insert sequence (IS) elements. IS elements are represented as an open box including the terminal IRs and a single open reading frame encoding the transposase [16]. There were 67 putative IS elements in the So0157-2 genome, like its close relative, So ce56, only one element was longer than 1 kb. The 66 short putative IS sites contained a number of known inverted repeats, but none of the intervening sequences showed significant similarity to any of the available database sequences. Furthermore, 37 predicted transposases were identified in the So0157-2 genome, a finding that is in accordance with presence of the putative IS sites (left and right inverted repeats). These results suggest that there are additional, specific linear insert sequence structures that may contain novel insertion sequence patterns.

Supplementary Methods

Genome assembly and feature annotation

Two independent assemblies of the bacterial genome were obtained using the 454® sequencing reads and the Solexa® reads. The assemblies were generated with SOAPdenovo [17], ABySS [18], and Velvet [19], with the default parameters being employed in all programs. For each assembly, all vs. all alignments were performed using the MUMmer package [20] with a minimum overlap of 100 bp. A new consensus assembly of the hybrid sequences was generated with SOAPdenovo and manually curated. MUMmer alignments of the assembled sequence and the *Sorangium cellulosum* so ce56 genome

were used to assess the orientation and synteny conservation of the newly assembled fragments.

Gene prediction was performed with Glimmer v3.02 [21] and GeneMark.hmm for prokaryotes [22]. This analysis was followed with the application of an automatic annotation pipeline carried out with the RAST [23] and Blast2Go programs [24], which retrieve sequence information from SWISS PROT, InterPro, Pfam, PROSITE, COG, GO, KEGG, NCBI NR, and other databases. Transfer RNAs were detected with tRNAscan-SE [25]. Annotations were checked manually, and frameshift corrections were performed with GenePRIMP [26] using the original reads. Extra annotation of small (<100 bp) ORFs of known function was performed manually. Sequence manipulation, parsing, and statistical features, such as genome size, %GC, codon usage, and Codon Adaptation Index (CAI), were determined using the EMBOSS 6.40 package [27] and related scripts. Schematic features of the genome were constructed by Circos [28].

A whole-genome BLAST search against the CAZy database was conducted to annotate putative polysaccharide-degrading enzymes in the So0157-2 genome [29].

Secondary metabolite pathway analysis

Annotation of PKS/NRPS gene clusters was carried out using ClustScan and related databases [30]. Prediction of the amino acid usage for each module of non-ribosomal peptide synthetase (NRPS) was conducted with NRPSpredictor2 [31]. Additional gene cluster analysis for secondary metabolites was conducted with antiSMASH [32].

Analysis and mapping of paralogous genes

An all-against-all BLAST search of protome was conducted with the BLASTP program (NCBI Blast+ 2.26 package) [33] using an E-value threshold of 1e-10. All of the sequence pairs identified as reciprocal better or best hits were extracted. The average taxa weight based on all paralogous pairs was calculated. All pairs were clustered into independent groups with the MCL program [34]. The paralogous map was drawn by the Circos program [28].

Annotation of mobile genetic elements

The Insertion Sequence (IS) database was downloaded from the IS finder website [35]. The Prophage database from the ACLAME website was also used, which contains prophage, virus and integrated plasmid gene annotations [36]. The ICEberg database was downloaded to annotate integrated and conjugative elements in the So0157-2 genome [37]. The predicted So0157-2 proteome dataset was subjected to BLAST searches against the above dataset (BLASTP). An E-value threshold of 1e-30 was used, and the top 10 hits, when possible, were parsed for subsequent manual validation. The genes were further examined for GC content and codon usage biases, and an additional phylogenetic analysis was applied to identify other sources of horizontally transferred genes.

Restriction and modification system analysis

The full set of protein sequences from all of the experimentally confirmed restriction and modification systems was obtained from REBASE [38]. A BLASTP search was conducted using the predicted So0157-2 proteome dataset against the REBASE database. The E-value cutoff was set to 1e-30. Only the top hit was parsed for follow-up analysis.

Annotation Clustered Regularly Interspaced Short Palindromic Repeats (CRISPR)

Whole-genome scanning for CRISPR sequences was conducted with PILER-CR v.1.06 [39]. The result was filtered using the following parameters: minimum array ≥10, minimum conservation >0.9, repeat length 16-64 bp, and spacer length 8-64 bp. The CRISPR-associated genes were annotated via BLASTP searches against CRISPR/Cas profiles obtained from NCBI (ftp://ftp.ncbi.nih.gov/pub/wolf/_suppl/CRISPRclass/crisprPro.html) with an E-value threshold of ≤ 1e-30.

Annotation sigma factors, transcriptional factors and protein kinase

The sigma factors, transcriptional factors and components of two component system were annotated by P2CS (http://www.p2cs.org/) [40] and P2TF (http://www.p2tf.org/) database. Genes encoding eukaryotic-like kinase in the prokaryotes were identified by BLASTP analysis of complete proteome using the Pfam matrix for protein kinases (Pkinase, PF00069; Pkinase_C, PF00433; Pkinase_tyr, PF07714). All sequences obtained with an E-value≤1e-10 were back-searched against Pfam and Prosite (http://expasy.org/prosite/) to verify unequivocally which proteins matched to the kinase domain.

References

1. Schneiker S, Perlova O, Kaiser O, Gerth K, Alici A, Altmeyer MO, *et al.* Complete genome sequence of the myxobacterium *Sorangium cellulosum*. *Nat. Biotechnol.* 2007, **25**(11)**:** 1281-1289.

2. Hodgkin J, Kaiser D. Cell-to-cell stimulation of movement in nonmotile mutants of Myxococcus. *Proc. Natl. Acad. Sci. U. S. A.* 1977, **74**(7)**:** 2938-2942.

3. Wolgemuth C, Hoiczyk E, Kaiser D, Oster G. How myxobacteria glide. *Curr. Biol.* 2002, **12**(5)**:** 369-377.

4. Mignot T, Shaevitz JW, Hartzell PL, Zusman DR. Evidence that focal adhesion complexes power bacterial gliding motility. *Science* 2007, **315**(5813)**:** 853-856.

5. Keck M, Gisch N, Moll H, Vorholter FJ, Gerth K, Kahmann U, *et al.* Unusual outer membrane lipid composition of the gram-negative, lipopolysaccharide-lacking myxobacterium *Sorangium cellulosum* So ce56. *J. Biol. Chem.* 2011, **286**(15)**:** 12850-12859.

6. Lu A, Cho K, Black WP, Duan XY, Lux R, Yang Z, *et al.* Exopolysaccharide biosynthesis genes required for social motility in *Myxococcus xanthus*. *Mol. Microbiol.* 2005, **55**(1)**:** 206-220.

7. Mattick JS. Type IV pili and twitching motility. *Annu. Rev. Microbiol.* 2002, **56:** 289-314.

8. Wu SS, Wu J, Kaiser D. The *Myxococcus xanthus pilT* locus is required for social gliding motility although pili are still produced. *Mol. Microbiol.* 1997, **23**(1)**:** 109-121.

9. Youderian P, Burke N, White DJ, Hartzell PL. Identification of genes required for adventurous gliding motility in *Myxococcus xanthus* with the transposable element mariner. *Mol. Microbiol.* 2003, **49**(2)**:** 555-570.

10. Zusman DR, Scott AE, Yang Z, Kirby JR. Chemosensory pathways, motility and development in *Myxococcus xanthus*. *Nat. Rev. Microbiol.* 2007, **5**(11)**:** 862-872.

11. Gerth K, Pradella S, Perlova O, Beyer S, Muller R. Myxobacteria: proficient producers of novel natural products with various biological activities--past and future biotechnological aspects with the focus on the genus *Sorangium*. *J. Biotechnol.* 2003, **106**(2-3)**:** 233-253.

12. Wenzel SC, Muller R. Formation of novel secondary metabolites by bacterial multimodular assembly lines: deviations from textbook biosynthetic logic. *Curr. Opin. Chem. Biol.* 2005, **9**(5)**:** 447-458.

13. Brenner D, Krieg N. *The Proteobacteria. Part C: The alpha-, beta-, delta-. and epsilonproteobacteria*. New York: Springer, 2005.

14. Goldman B, Bhat S, Shimkets LJ. Genome evolution and the emergence of fruiting body development in *Myxococcus xanthus*. *PLoS One* 2007, **2**(12)**:** e1329.

15. Canchaya C, Fournous G, Brussow H. The impact of prophages on bacterial chromosomes. *Mol. Microbiol.* 2004, **53**(1)**:** 9-18.

16. Touchon M, Rocha EP. Causes of insertion sequences abundance in prokaryotic genomes. *Mol. Biol. Evol.* 2007, **24**(4)**:** 969-981.

17. Li R, Li Y, Kristiansen K, Wang J. SOAP: short oligonucleotide alignment program. *Bioinformatics* 2008, **24**(5)**:** 713-714.

18. Simpson JT, Wong K, Jackman SD, Schein JE, Jones SJ, Birol I. ABySS: a parallel assembler for short read sequence data. *Genome Res.* 2009, **19**(6)**:** 1117-1123.

19. Zerbino DR, Birney E. Velvet: algorithms for de novo short read assembly using de Bruijn graphs. *Genome Res.* 2008, **18**(5)**:** 821-829.

20. Delcher AL, Salzberg SL, Phillippy AM. Using MUMmer to identify similar regions in large sequence sets. *Curr. Protoc. Bioinformatics* 2003**:** 10.13. 11-10.13. 18.

21. Delcher AL, Bratke KA, Powers EC, Salzberg SL. Identifying bacterial genes and endosymbiont DNA with Glimmer. *Bioinformatics* 2007, **23**(6)**:** 673-679.

22. Borodovsky M, Mills R, Besemer J, Lomsadze A. Prokaryotic gene prediction using GeneMark and GeneMark. hmm. *Curr. Protoc. Bioinformatics* 2003**:** 4.5. 1-4.5. 16.

23. Aziz RK, Bartels D, Best AA, DeJongh M, Disz T, Edwards RA, *et al.* The RAST Server: rapid annotations using subsystems technology. *BMC Genomics* 2008, **9:** 75.

24. Conesa A, Gotz S, Garcia-Gomez JM, Terol J, Talon M, Robles M. Blast2GO: a universal tool for annotation, visualization and analysis in functional genomics research. *Bioinformatics* 2005, **21**(18)**:** 3674-3676.

25. Schattner P, Brooks AN, Lowe TM. The tRNAscan-SE, snoscan and snoGPS web servers for the detection of tRNAs and snoRNAs. *Nucleic Acids Res.* 2005, **33**(Web Server issue)**:** W686-689.

26. Pati A, Ivanova NN, Mikhailova N, Ovchinnikova G, Hooper SD, Lykidis A, *et al.* GenePRIMP: a gene prediction improvement pipeline for prokaryotic genomes. *Nat. Methods* 2010, **7**(6)**:** 455-457.

27. Rice P, Longden I, Bleasby A. EMBOSS: the European Molecular Biology Open Software Suite. *Trends Genet.* 2000, **16**(6)**:** 276-277.

28. Krzywinski M, Schein J, Birol I, Connors J, Gascoyne R, Horsman D, *et al.* Circos: an information aesthetic for comparative genomics. *Genome Res.* 2009, **19**(9)**:** 1639-1645.

29. Cantarel BL, Coutinho PM, Rancurel C, Bernard T, Lombard V, Henrissat B. The Carbohydrate-Active EnZymes database (CAZy): an expert resource for Glycogenomics. *Nucleic Acids Res.* 2009, **37**(Database issue)**:** D233-238.

30. Starcevic A, Zucko J, Simunkovic J, Long PF, Cullum J, Hranueli D. ClustScan: an integrated program package for the semi-automatic annotation of modular biosynthetic gene clusters and in silico prediction of novel chemical structures. *Nucleic Acids Res.* 2008, **36**(21)**:** 6882-6892.

31. Rottig M, Medema MH, Blin K, Weber T, Rausch C, Kohlbacher O. NRPSpredictor2--a web server for predicting NRPS adenylation domain specificity. *Nucleic Acids Res.* 2011, **39**(Web Server issue)**:** W362-367.

32. Medema MH, Blin K, Cimermancic P, de Jager V, Zakrzewski P, Fischbach MA, *et al.* antiSMASH: rapid identification, annotation and analysis of secondary metabolite biosynthesis gene clusters in bacterial and fungal genome sequences. *Nucleic Acids Res.* 2011, **39**(Web Server issue)**:** W339-346.

33. Altschul SF, Madden TL, Schaffer AA, Zhang J, Zhang Z, Miller W, *et al.* Gapped BLAST and PSI-BLAST: a new generation of protein database search programs. *Nucleic Acids Res.* 1997, **25**(17)**:** 3389-3402.

34. Li L, Stoeckert CJ, Jr., Roos DS. OrthoMCL: identification of ortholog groups for eukaryotic genomes. *Genome Res.* 2003, **13**(9)**:** 2178-2189.

35. Varani AM, Siguier P, Gourbeyre E, Charneau V, Chandler M. ISsaga is an ensemble of web-based methods for high throughput identification and semi-automatic annotation of insertion sequences in prokaryotic genomes. *Genome Biol.* 2011, **12**(3)**:** R30.

36. Leplae R, Lima-Mendez G, Toussaint A. ACLAME: a CLAssification of Mobile genetic Elements, update 2010. *Nucleic Acids Res.* 2010, **38**(Database issue)**:** D57-61.

37. Bi D, Xu Z, Harrison EM, Tai C, Wei Y, He X, *et al.* ICEberg: a web-based resource for integrative and conjugative elements found in Bacteria. *Nucleic Acids Res.* 2012, **40**(Database issue)**:** D621-626.

38. Roberts RJ, Vincze T, Posfai J, Macelis D. REBASE--a database for DNA restriction and modification: enzymes, genes and genomes. *Nucleic Acids Res.* 2010, **38**(Database issue)**:** D234-236.

39. Edgar RC. PILER-CR: fast and accurate identification of CRISPR repeats. *BMC Bioinformatics* 2007, **8:** 18.

40. Barakat M, Ortet P, Whitworth DE. P2CS: a database of prokaryotic two-component systems. *Nucleic Acids Res.* 2011, **39**(Database issue)**:** D771-776.

Supplementary Fig. S1.

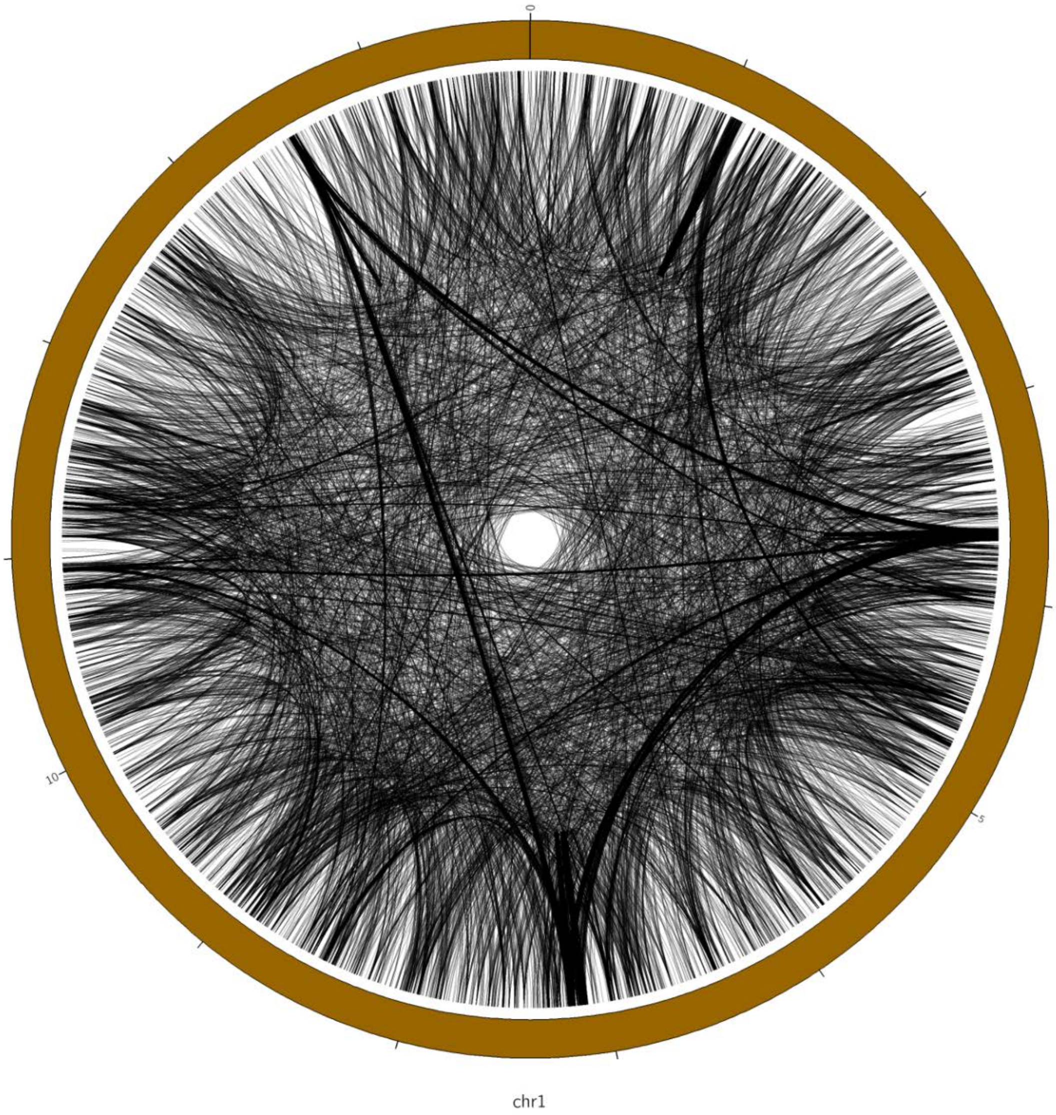

A schematic representation of genome sequence duplication in So0157-2.

Supplementary Fig. S2

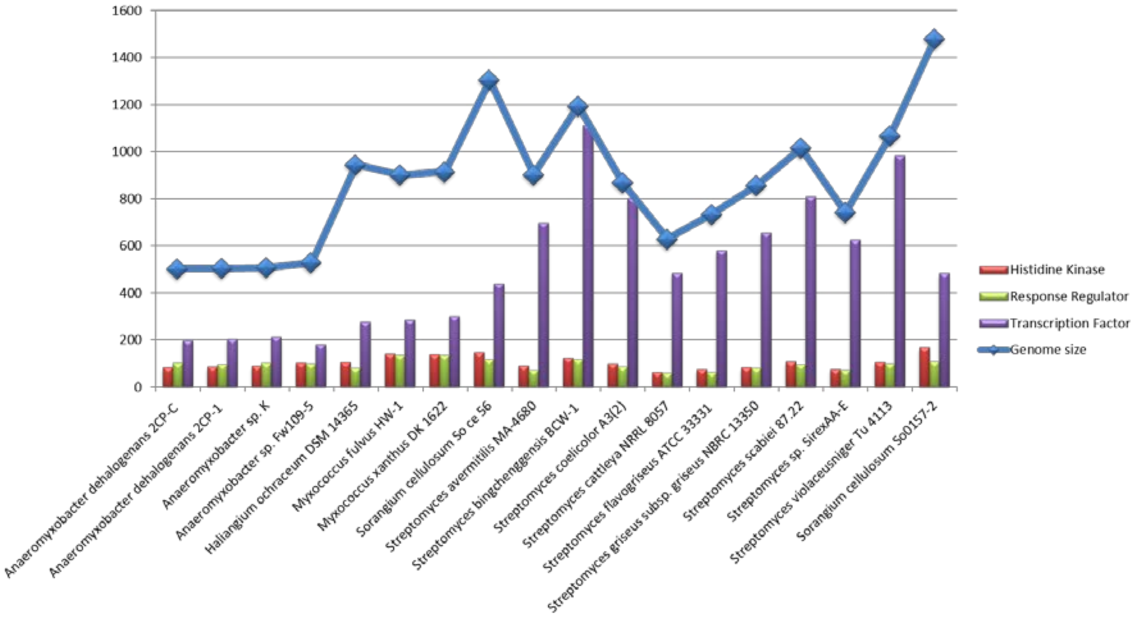

The expansion of the regulatory network as an expansion of the genome.

Supplementary Fig. S3

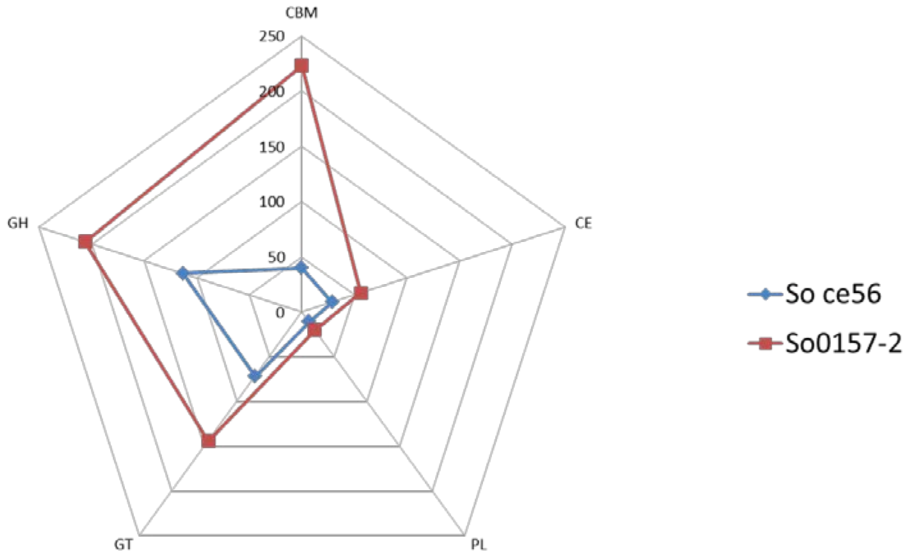

A schematic representation of the abundance of polysaccharide-related families and CMB in two sequenced *Sorangium* genomes, So0157-2 and So ce56.

Supplementary Table S1. Annotations of eukaryotic-like kinases in So0157-2

Locus tag	Database* ID	Identity	Alignment length	Q. start	Q. end	D. start	D. end	E-value	Bit score
SCE1572_2	A9GHT8_SORC5/9-291	96.47	283	9	291	1	283	1.00E-161	566
SCE1572_22	A9GHZ6_SORC5/16-280	100	265	16	280	1	265	6.00E-150	526
SCE1572_38	A9GKW5_SORC5/18-287	97.78	270	18	287	1	270	1.00E-155	545
SCE1572_45	A9GKX5_SORC5/32-304	90.11	273	43	315	1	273	4.00E-142	500
SCE1572_50	B9RTW7_RICCO	36.27	193	5	193	38	225	1.00E-19	93.6
SCE1572_58	A9G3F0_SORC5/15-286	79.04	272	15	286	1	272	4.00E-128	454
SCE1572_72	A9GL24_SORC5/17-279	91.63	263	17	279	1	263	6.00E-144	506
SCE1572_157	A9FXD2_SORC5/32-294	80.23	263	32	294	1	263	1.00E-118	422
SCE1572_159	A9FXC4_SORC5/66-318	92.49	253	66	318	1	253	5.00E-130	461
SCE1572_221	B5DPB6_DROPS	31.68	202	266	457	758	958	2.00E-18	90.9
SCE1572_301	D0LG85_HALO1/9-288	38.23	327	27	351	1	280	4.00E-47	186
SCE1572_324	A6G4E4_9DELT	33.51	385	943	1296	900	1260	1.00E-35	149
SCE1572_368	A9GLX4_SORC5/38-301	83.71	264	24	286	1	264	4.00E-128	455
SCE1572_418	A6G4E4_9DELT	29.12	656	1080	1709	613	1219	1.00E-49	196
SCE1572_430	A9G5P3_SORC5/10-271	86.69	263	27	289	1	262	3.00E-134	474
SCE1572_433	A9GM76_SORC5/21-287	89.89	267	21	284	1	267	1.00E-137	486
SCE1572_498	A9FA64_SORC5/37-294	69.83	232	1	232	29	258	7.00E-89	325
SCE1572_510	A9GQH3_SORC5/6-269	90.53	264	6	268	1	264	4.00E-141	497
SCE1572_515	B9RTW7_RICCO	31.98	197	50	238	38	225	2.00E-12	69.7
SCE1572_535	A9GQL8_SORC5/11-242	94.02	234	11	244	1	232	7.00E-112	401
SCE1572_540	A9GQM9_SORC5/13-268	91.27	252	2	253	5	256	5.00E-130	461
SCE1572_587	A9GQX2_SORC5/13-284	80.51	272	13	282	1	272	4.00E-126	447
SCE1572_596	A9GQY7_SORC5/22-296	92	275	22	296	1	275	4.00E-152	533
SCE1572_602	A9GQZ9_SORC5/222-494	93.04	273	507	779	1	273	1.00E-146	516
SCE1572_648	A9GR88_SORC5/43-313	95.2	271	43	313	1	271	3.00E-150	528

SCE1572_669	B4KV63_DROMO	32.82	262	138	398	701	958	7.00E-31	132
SCE1572_671	A9GRD4_SORC5/21-295	86.79	280	21	300	1	275	5.00E-137	483
SCE1572_686	A9GU97_SORC5/162-331	92.35	170	144	313	1	170	5.00E-87	318
SCE1572_728	A9GUI6_SORC5/43-307	87.55	265	13	277	1	265	1.00E-134	475
SCE1572_743	D8LQ15_ECTSI	35.44	206	110	307	612	816	1.00E-20	97.4
SCE1572_745	A9GUM9_SORC5/30-300	98.89	271	29	299	1	271	5.00E-159	556
SCE1572_805	A9G2M3_SORC5/18-300	72.79	283	19	301	1	283	2.00E-100	362
SCE1572_810	B8LDL2_THAPS	24.32	588	151	718	953	1477	1.00E-35	149
SCE1572_824	A9FS91_SORC5/8-267	60.94	256	20	273	6	260	5.00E-79	293
SCE1572_844	A9GV94_SORC5/20-278	96.15	260	20	279	1	259	2.00E-140	495
SCE1572_872	E9C274_9EUKA	48.11	212	317	528	1539	1748	7.00E-56	214
SCE1572_894	D3BLW9_POLPA	37.04	135	4	137	1564	1697	7.00E-18	88.6
SCE1572_910	A9FP27_SORC5/11-267	84.82	257	11	267	1	257	6.00E-112	402
SCE1572_928	A9GVM0_SORC5/29-281	81.35	252	29	280	1	252	1.00E-103	372
SCE1572_939	A9EMV5_SORC5/25-308	86.01	286	24	309	1	284	1.00E-123	439
SCE1572_940	A9EMV8_SORC5/15-289	93.09	275	17	291	1	275	8.00E-158	553
SCE1572_962	F2UKF9_9EUKA	54.45	696	5	695	9	704	0	766
SCE1572_977	A9EN27_SORC5/21-272	99.6	252	21	272	1	252	2.00E-149	525
SCE1572_993	B9RTW7_RICCO	35.35	198	5	194	43	234	3.00E-16	82.4
SCE1572_1008	D3BJS3_POLPA	26.92	442	7	437	815	1249	5.00E-32	135
SCE1572_1013	A9ENB1_SORC5/68-351	91.9	284	68	351	1	284	1.00E-143	506
SCE1572_1015	D0LZ14_HALO1	38.29	935	103	1019	163	1078	3.00E-148	523
SCE1572_1041	A9EU91_SORC5/223-477	58.1	253	342	594	1	253	7.00E-79	291
SCE1572_1051	B9RTW7_RICCO	32.98	188	65	243	38	219	1.00E-13	73.9
SCE1572_1054	A9FFW3_SORC5/24-336	88.82	313	16	328	1	313	1.00E-165	578
SCE1572_1075	A9VA90_MONBE	29.45	292	72	342	320	591	3.00E-13	73.2
SCE1572_1098	D0LZ14_HALO1	37.95	1199	105	1286	175	1345	1.00E-161	568
SCE1572_1168	F2EE39_HORVD	32.08	293	308	555	23	312	1.00E-24	112

SCE1572_1184	A9F932_SORC5/34-318	71.58	285	68	352	1	285	3.00E-117	419
SCE1572_1197	A9FYH6_SORC5/7-273	74.16	267	16	274	1	267	4.00E-110	396
SCE1572_1229	A9EQJ9_SORC5/6-267	50.38	264	5	266	1	262	7.00E-60	228
SCE1572_1230	A6G4E4_9DELT	30.17	633	1063	1634	589	1209	7.00E-50	196
SCE1572_1255	B8LDL2_THAPS	27.16	567	26	584	997	1484	2.00E-51	201
SCE1572_1256	Q9FF29_ARATH	26.35	296	189	471	14	250	3.00E-14	76.3
SCE1572_1294	A9EPH9_SORC5/19-285	91.79	268	19	286	1	267	1.00E-143	505
SCE1572_1295	A9EPI2_SORC5/10-279	88.52	270	10	279	1	270	9.00E-133	468
SCE1572_1308	F2UKF9_9EUKA	43.1	710	2	703	4	696	1.00E-153	540
SCE1572_1316	A9ETG2_SORC5/740-1011	97.43	272	381	652	1	272	1.00E-159	560
SCE1572_1319	A9ETG8_SORC5/12-270	93.05	259	12	270	1	259	3.00E-139	491
SCE1572_1322	A9FE17_SORC5/11-267	51.89	264	1	264	12	254	4.00E-67	252
SCE1572_1337	A9ETI8_SORC5/40-302	96.2	263	40	301	1	263	3.00E-145	511
SCE1572_1380	Q75J39_ORYSJ	31.4	293	362	624	51	315	3.00E-24	110
SCE1572_1389	E3X4G0_ANODA	35.16	256	39	287	40	283	2.00E-25	113
SCE1572_1402	D8LHV1_ECTSI	31.6	231	103	332	87	306	2.00E-12	71.6
SCE1572_1421	D0LYB2_HALO1	39.25	879	4	821	2	842	1.00E-122	437
SCE1572_1422	D0LYB2_HALO1	38.18	571	7	524	8	557	2.00E-76	283
SCE1572_1447	D7TTD5_VITVI	25.32	233	122	340	688	901	9.00E-12	68.2
SCE1572_1516	F2UKF9_9EUKA	40.94	149	2	145	8	156	8.00E-23	105
SCE1572_1525	A9EUD9_SORC5/28-288	99.6	250	1	250	12	261	4.00E-144	507
SCE1572_1539	A9EUI2_SORC5/20-285	95.49	266	20	285	1	266	2.00E-148	521
SCE1572_1569	B9RTW7_RICCO	32.45	188	19	197	44	219	1.00E-12	70.5
SCE1572_1610	A9EVB6_SORC5/189-453	97.74	265	189	453	1	265	1.00E-151	531
SCE1572_1633	A9EZT1_SORC5/15-309	87.8	295	15	300	1	295	4.00E-135	477
SCE1572_1649	D7FZX4_ECTSI	31.08	399	160	494	106	497	2.00E-28	123
SCE1572_1699	A9F066_SORC5/60-323	96.59	264	59	322	1	264	9.00E-152	532
SCE1572_1705	A9F084_SORC5/63-328	98.5	266	160	425	1	266	1.00E-155	545

SCE1572_1715	A9V4U4_MONBE	34.27	143	4	136	944	1080	4.00E-12	66.6
SCE1572_1733	E9CI33_9EUKA	47.8	205	206	408	70	265	9.00E-39	157
SCE1572_1768	D7FZX4_ECTSI	27.12	413	105	476	106	499	3.00E-18	90.1
SCE1572_1860	D7SRK5_VITVI	28.21	234	75	294	47	276	7.00E-13	72
SCE1572_1869	A9F1G8_SORC5/19-291	92.67	273	19	291	1	273	1.00E-144	508
SCE1572_1882	A9F694_SORC5/14-257	84.43	244	22	265	1	244	4.00E-112	402
SCE1572_1884	A9EV86_SORC5/15-281	67.42	267	11	273	1	267	1.00E-97	352
SCE1572_1889	B8LDL2_THAPS	22.43	437	196	598	1445	1868	2.00E-17	88.2
SCE1572_1903	F2UKF9_9EUKA	29.67	701	9	701	12	700	5.00E-91	332
SCE1572_1906	A9F6G8_SORC5/45-306	87.02	262	41	302	1	262	8.00E-126	446
SCE1572_1928	A9F6S1_SORC5/13-277	83.66	257	1	257	9	265	4.00E-126	446
SCE1572_1936	A9FE17_SORC5/11-267	55.81	258	13	270	1	255	5.00E-73	272
SCE1572_1936	D0LZ14_HALO1	34.97	1384	6	1321	9	1346	2.00E-179	626
SCE1572_1940	D7FKP6_ECTSI	54.43	237	2	233	121	353	4.00E-63	240
SCE1572_1941	D7FKP6_ECTSI	46.47	269	24	278	93	356	6.00E-54	207
SCE1572_2073	A9F816_SORC5/16-240	91.56	225	16	237	1	225	3.00E-109	392
SCE1572_2143	E9C274_9EUKA	29.73	185	412	596	1568	1745	2.00E-20	97.4
SCE1572_2170	A9FRK3_SORC5/74-345	95.22	272	97	368	1	272	5.00E-150	526
SCE1572_2171	A9FRJ9_SORC5/22-280	93.05	259	22	280	1	259	1.00E-139	492
SCE1572_2183	D0LYB2_HALO1	36.51	641	185	779	182	807	2.00E-73	274
SCE1572_2196	A9FUH6_SORC5/44-314	91.88	271	91	361	1	271	1.00E-145	512
SCE1572_2197	A9GRQ7_SORC5/16-276	88.12	261	16	274	1	261	1.00E-129	461
SCE1572_2206	B9RTW7_RICCO	31.98	197	3	192	38	219	3.00E-15	78.6
SCE1572_2220	A9F1G8_SORC5/19-291	75.09	273	19	291	1	273	2.00E-114	407
SCE1572_2238	A9FDG9_SORC5/21-281	96.92	260	20	279	1	260	2.00E-150	529
SCE1572_2239	A9FDH1_SORC5/20-281	88.55	262	20	281	1	262	3.00E-136	481
SCE1572_2241	A9FDG9_SORC5/21-281	60.62	259	7	264	1	259	1.00E-87	320
SCE1572_2242	A9FDJ3_SORC5/13-278	80.83	266	13	278	1	266	9.00E-119	422

SCE1572_2258	D0LZ14_HALO1	39.5	1395	2	1329	7	1346	0	800
SCE1572_2283	B5DPB6_DROPS	38.39	224	166	385	731	950	6.00E-36	149
SCE1572_2326	B9RTW7_RICCO	34.69	196	31	215	43	234	1.00E-20	97.1
SCE1572_2359	A9FIP5_SORC5/20-288	89.22	269	20	287	1	269	3.00E-125	443
SCE1572_2371	E9C274_9EUKA	37.04	162	245	406	1568	1721	8.00E-18	88.6
SCE1572_2410	E3PW85_CLOSD	38.69	168	140	304	1406	1570	1.00E-27	120
SCE1572_2441	A9FJE9_SORC5/231-472	92.56	242	231	472	1	242	2.00E-130	461
SCE1572_2487	A9FNZ9_SORC5/14-281	82.84	268	11	278	1	268	5.00E-131	464
SCE1572_2490	D7TTD5_VITVI	33.8	213	223	432	256	460	4.00E-26	115
SCE1572_2518	A9GT67_SORC5/13-276	71.1	263	13	273	1	263	4.00E-104	374
SCE1572_2519	D7TTD5_VITVI	26.39	341	107	429	121	458	6.00E-25	112
SCE1572_2544	A6G4E4_9DELT	31.65	752	717	1426	537	1260	9.00E-69	259
SCE1572_2588	D0LZ14_HALO1	39.93	1212	103	1290	163	1346	0	670
SCE1572_2590	Q097E7_STIAU/1-261	54.98	291	32	322	1	261	1.00E-77	288
SCE1572_2647	A9G8I3_SORC5/10-268	73.96	265	10	273	1	259	2.00E-111	399
SCE1572_2652	B8HVH1_CYAP4/7-265	54.92	264	7	266	1	259	4.00E-83	306
SCE1572_2679	B9RTW7_RICCO	28.14	231	4	216	36	262	6.00E-15	77.8
SCE1572_2690	A9FJE9_SORC5/231-472	64.56	237	221	456	1	237	2.00E-82	303
SCE1572_2701	D0LZ14_HALO1	38.18	1362	2	1303	7	1346	0	731
SCE1572_2715	A9FQS5_SORC5/56-333	95.68	278	56	333	1	278	6.00E-145	510
SCE1572_2749	A9FUX8_SORC5/6-235	92.14	229	116	344	1	229	4.00E-125	444
SCE1572_2826	B9RTW7_RICCO	33.16	196	7	195	42	229	1.00E-15	79.7
SCE1572_2832	A9VA07_MONBE	45.29	340	13	342	596	930	3.00E-69	259
SCE1572_2934	A9G0Y5_SORC5/28-295	86.94	268	61	328	1	268	6.00E-134	473
SCE1572_2963	A9G147_SORC5/98-359	96.95	262	98	359	1	262	3.00E-144	507
SCE1572_2966	C0WK81_9CORY/14-268	39.31	262	33	290	1	255	7.00E-39	158
SCE1572_2987	A9G170_SORC5/33-336	95.07	304	33	336	1	304	7.00E-163	570
SCE1572_2996	A9G190_SORC5/32-292	84.91	265	44	308	1	261	2.00E-119	426

SCE1572_3033	Q23ZG6_TETTH	24.95	457	13	440	495	944	4.00E-37	153
SCE1572_3041	B4KV63_DROMO	34.03	191	479	661	756	941	3.00E-14	77.4
SCE1572_3114	F2UAL0_9EUKA	40.45	89	28	116	1280	1366	1.00E-10	64.7
SCE1572_3137	D0LZ14_HALO1	37.6	1367	1	1297	12	1344	0	713
SCE1572_3141	A9GTU1_SORC5/18-277	56.15	260	7	266	1	260	1.00E-81	301
SCE1572_3158	D0LZ14_HALO1	36.94	1367	2	1297	7	1344	0	668
SCE1572_3180	A9VA07_MONBE	42.66	354	63	406	596	944	1.00E-69	260
SCE1572_3183	A9V4U4_MONBE	41.98	131	9	137	954	1084	4.00E-28	121
SCE1572_3267	A9G5X1_SORC5/59-323	95.47	265	93	357	1	265	8.00E-146	513
SCE1572_3271	A9G5Y1_SORC5/47-312	98.12	266	26	291	1	266	3.00E-151	531
SCE1572_3291	A9F8N6_SORC5/25-290	55.35	271	19	287	1	266	8.00E-79	290
SCE1572_3371	D3BJS3_POLPA	29.08	447	4	433	830	1268	9.00E-43	171
SCE1572_3400	A9G6S7_SORC5/13-281	96.64	268	13	280	1	268	4.00E-152	534
SCE1572_3426	Q15300_HUMAN	33.33	90	370	459	155	239	2.00E-11	67.8
SCE1572_3444	B2J8F9_NOSP7/7-265	49.8	247	7	253	13	259	3.00E-69	259
SCE1572_3509	A9GA77_SORC5/13-209	90.86	197	13	209	1	197	9.00E-97	351
SCE1572_3555	D0LZ14_HALO1	40.06	1358	2	1297	7	1346	0	799
SCE1572_3557	B9SC01_RICCO	29.83	295	232	509	61	332	4.00E-19	93.2
SCE1572_3564	D0LZ14_HALO1	37.98	1364	2	1294	9	1346	0	655
SCE1572_3565	A9FE17_SORC5/11-267	61.66	253	9	261	1	253	1.00E-84	309
SCE1572_3568	D0LZ14_HALO1	37.21	1360	2	1295	9	1346	0	656
SCE1572_3602	A9GN95_SORC5/15-286	60.44	273	57	329	1	272	2.00E-81	299
SCE1572_3611	D0LYB2_HALO1	37.44	844	7	818	3	808	1.00E-119	428
SCE1572_3648	A9EWS2_SORC5/19-281	90.87	263	24	286	1	263	2.00E-137	484
SCE1572_3687	D7TTD5_VITVI	26.56	384	59	419	95	470	7.00E-23	105
SCE1572_3742	Q7UX63_RHOBA/29-285	38.61	259	48	301	1	257	2.00E-37	154
SCE1572_3748	A9GAS9_SORC5/19-278	93.9	246	1	246	15	260	3.00E-128	456
SCE1572_3762	A9GAU7_SORC5/11-280	97.04	270	11	280	1	270	5.00E-150	528

SCE1572_3765	B4KV63_DROMO	36.52	230	139	367	716	941	3.00E-36	149
SCE1572_3771	A9GAX7_SORC5/14-273	86.54	260	226	485	1	260	9.00E-123	436
SCE1572_3808	A9GB51_SORC5/18-289	93.01	272	18	289	1	272	2.00E-143	504
SCE1572_3832	A9GB92_SORC5/15-298	91.55	284	15	298	1	284	1.00E-151	531
SCE1572_3852	A9GBB7_SORC5/15-282	92.91	268	15	282	1	268	2.00E-139	491
SCE1572_3872	E3PW85_CLOSD	35	160	164	323	1415	1571	2.00E-24	109
SCE1572_3891	A9GEK8_SORC5/15-277	95.44	263	41	303	1	263	2.00E-138	488
SCE1572_3909	A9VA07_MONBE	30.82	717	34	703	601	1247	7.00E-70	262
SCE1572_3923	A9GEU8_SORC5/15-246	82.88	222	15	233	1	222	2.00E-87	320
SCE1572_3948	A9GF00_SORC5/16-284	97.4	269	21	289	1	269	3.00E-157	550
SCE1572_3949	A9GF02_SORC5/17-290	90.15	274	17	290	1	274	2.00E-133	473
SCE1572_3968	A9GF27_SORC5/18-299	93.62	282	18	299	1	282	3.00E-153	538
SCE1572_3971	A9GF34_SORC5/16-292	97.47	277	16	292	1	277	5.00E-158	554
SCE1572_3980	F2E4N3_HORVD	25.66	339	46	358	34	367	4.00E-18	89
SCE1572_4022	D3BJS3_POLPA	26.42	405	37	422	829	1222	9.00E-28	121
SCE1572_4043	A9GFI8_SORC5/49-315	98.13	267	49	315	1	267	4.00E-154	540
SCE1572_4045	A9GFJ3_SORC5/905-1172	96.64	268	898	1165	1	268	1.00E-152	536
SCE1572_4065	A9GFN9_SORC5/14-285	90.16	244	1	244	29	272	9.00E-130	459
SCE1572_4072	A9GFQ7_SORC5/31-302	98.9	272	26	297	1	272	3.00E-162	568
SCE1572_4101	D7TTD5_VITVI	26.14	352	123	446	121	468	3.00E-16	83.2
SCE1572_4129	D3BJS3_POLPA	24.69	401	8	401	829	1223	5.00E-24	108
SCE1572_4162	B9RTW7_RICCO	34.36	195	4	191	38	224	5.00E-13	71.2
SCE1572_4165	B9RTW7_RICCO	31.9	232	5	226	38	258	8.00E-15	77
SCE1572_4185	A9GIL2_SORC5/14-269	93.75	256	14	269	1	256	4.00E-133	472
SCE1572_4342	A9GJC9_SORC5/88-343	92.97	256	83	338	1	256	3.00E-130	462
SCE1572_4349	A9G8J3_SORC5/18-265	42.86	189	80	262	60	248	1.00E-34	145
SCE1572_4350	D0LZ14_HALO1	41	944	2	870	7	930	3.00E-151	533
SCE1572_4364	C3XWU6_BRAFL	33.71	353	92	439	785	1109	9.00E-41	164

SCE1572_4372	D8LQ15_ECTSI	45.68	243	83	318	597	839	3.00E-49	194
SCE1572_4375	A9FDB2_SORC5/22-188	65.81	155	17	170	1	155	4.00E-54	209
SCE1572_4463	A9GQ42_SORC5/62-327	75.57	262	50	308	1	262	3.00E-107	385
SCE1572_4493	A9G1R5_SORC5/6-275	87.78	270	157	426	1	270	9.00E-136	479
SCE1572_4540	A9GJS7_SORC5/31-311	76.16	281	44	322	1	281	1.00E-122	436
SCE1572_4558	A9GN95_SORC5/15-286	93.75	272	13	284	1	272	3.00E-142	500
SCE1572_4582	A9VA07_MONBE	44.32	352	829	1169	596	942	3.00E-77	287
SCE1572_4600	A9GNJ1_SORC5/16-306	91.75	291	16	306	1	291	7.00E-152	532
SCE1572_4662	D0LZ14_HALO1	39.48	1388	10	1337	15	1335	0	723
SCE1572_4675	A9GRN8_SORC5/20-297	88.85	269	20	288	1	269	1.00E-138	489
SCE1572_4692	A9F8N6_SORC5/25-290	56	275	316	588	1	266	2.00E-75	280
SCE1572_4725	B4D5D6_9BACT	30.86	175	412	585	886	1055	6.00E-11	66.2
SCE1572_4741	A9GRY1_SORC5/11-278	94.03	268	11	278	1	268	1.00E-114	409
SCE1572_4758	A6GDW6_9DELT	26.42	318	625	927	580	893	9.00E-12	69.7
SCE1572_4776	A9GS33_SORC5/10-280	95.94	271	10	280	1	271	3.00E-156	547
SCE1572_4809	A9GSA1_SORC5/16-290	93.45	275	16	288	1	275	7.00E-152	532
SCE1572_4846	D3BJS3_POLPA	25.78	419	9	411	815	1197	8.00E-16	82
SCE1572_4847	A9GSI1_SORC5/16-296	90.04	281	16	296	1	281	3.00E-139	490
SCE1572_4851	A6G4E4_9DELT	30.9	631	1089	1658	590	1209	1.00E-52	206
SCE1572_4861	D0LZ14_HALO1	39.69	1018	2	961	7	993	6.00E-163	571
SCE1572_4879	A9GSP6_SORC5/10-277	95.52	268	53	320	1	268	4.00E-145	511
SCE1572_4900	A9FJE9_SORC5/231-472	63.33	240	220	458	1	240	3.00E-82	302
SCE1572_4916	A9GVU5_SORC5/13-275	87.83	263	45	307	1	263	1.00E-132	469
SCE1572_4960	A9GW43_SORC5/19-293	89.09	275	19	292	1	275	4.00E-136	480
SCE1572_4965	A9GJS7_SORC5/31-311	69.74	271	1	269	12	281	1.00E-103	373
SCE1572_4984	A9GW92_SORC5/236-501	95.49	266	236	501	1	266	1.00E-149	525
SCE1572_5016	A9GWG4_SORC5/16-292	88.45	277	16	292	1	277	2.00E-146	515
SCE1572_5032	A9GWK1_SORC5/51-315	94.34	265	51	315	1	265	4.00E-145	510

SCE1572_5053	A9GS33_SORC5/10-280	58.15	270	10	279	1	270	6.00E-90	326
SCE1572_5061	E5SP72_TRISP	56.04	91	19	109	678	768	2.00E-25	109
SCE1572_5065	A9GWP7_SORC5/9-264	94.53	256	30	285	1	256	2.00E-141	497
SCE1572_5069	A6GDW6_9DELT	45.99	274	606	879	660	933	7.00E-50	196
SCE1572_5098	A9GWX6_SORC5/13-270	94.26	244	1	244	15	258	7.00E-139	489
SCE1572_5105	F2UKF9_9EUKA	39.86	148	2	143	8	148	3.00E-19	94
SCE1572_5119	A9GX30_SORC5/18-287	93.7	270	18	287	1	270	1.00E-148	523
SCE1572_5149	A6GDW6_9DELT	37.87	301	1013	1313	657	951	2.00E-29	128
SCE1572_5183	B9RTW7_RICCO	29.29	198	1	192	34	225	2.00E-11	65.9
SCE1572_5199	B9RTW7_RICCO	29.95	197	14	205	35	226	5.00E-12	68.2
SCE1572_5213	A9EPX8_SORC5/14-320	89.25	307	5	311	1	307	5.00E-152	533
SCE1572_5222	A9EPZ6_SORC5/98-351	91.34	254	98	351	1	254	1.00E-127	454
SCE1572_5251	A9EQ70_SORC5/13-281	95.54	269	13	281	1	269	3.00E-152	534
SCE1572_5254	A9EQ76_SORC5/30-377	84.44	347	30	375	1	347	2.00E-158	555
SCE1572_5259	A9EQ83_SORC5/28-318	88.7	292	40	331	1	291	6.00E-138	486
SCE1572_5269	A9EQA5_SORC5/46-335	97.24	290	22	311	1	290	1.00E-165	578
SCE1572_5318	B9RTW7_RICCO	27.89	190	6	186	40	225	1.00E-11	66.6
SCE1572_5332	A9EQJ9_SORC5/6-267	85.88	262	6	267	1	262	2.00E-126	450
SCE1572_5348	A9EQM0_SORC5/13-276	85.98	264	13	276	1	264	1.00E-132	469
SCE1572_5352	E9C1L0_9EUKA	31.53	222	421	641	935	1146	2.00E-20	98.2
SCE1572_5363	A9FX47_SORC5/11-274	59.47	264	42	303	1	264	5.00E-76	282
SCE1572_5365	A9G2X2_SORC5/56-320	66.42	265	31	292	1	265	1.00E-88	322
SCE1572_5389	A9EQW7_SORC5/16-262	91.9	247	9	255	1	247	1.00E-133	474
SCE1572_5405	A9ER29_SORC5/14-284	88.56	271	37	307	1	271	1.00E-126	449
SCE1572_5420	A9EVM0_SORC5/14-277	88.26	264	14	277	1	264	9.00E-141	496
SCE1572_5425	A9GS33_SORC5/10-280	55.56	270	55	322	1	270	6.00E-86	313
SCE1572_5440	A5BDW0_VITVI	26.29	251	3	213	1116	1365	4.00E-16	82
SCE1572_5449	D3BJS3_POLPA	24.01	404	26	420	829	1222	5.00E-27	119

SCE1572_5468	A9GTU1_SORC5/18-277	55.77	260	4	263	1	260	7.00E-81	298
SCE1572_5480	A9GTU1_SORC5/18-277	90	260	7	266	1	260	7.00E-139	491
SCE1572_5495	B4KV63_DROMO	33.16	190	488	668	756	940	8.00E-16	82.4
SCE1572_5590	A9FDG9_SORC5/21-281	48.08	260	20	279	1	259	6.00E-63	237
SCE1572_5597	E3X4G0_ANODA	30.61	330	20	334	7	321	3.00E-35	146
SCE1572_5606	A9EWN7_SORC5/34-297	91.29	264	64	327	1	264	3.00E-129	457
SCE1572_5640	A9F619_SORC5/13-273	79.31	261	9	267	1	261	8.00E-112	401
SCE1572_5644	A9F619_SORC5/13-273	80.25	81	1	81	138	218	5.00E-35	142
SCE1572_5645	A9F619_SORC5/13-273	71.43	84	9	92	1	84	1.00E-28	120
SCE1572_5647	D0LZ14_HALO1	43.91	476	32	506	435	899	7.00E-106	380
SCE1572_5658	A9EWZ2_SORC5/22-300	88.17	279	40	315	1	279	2.00E-140	496
SCE1572_5689	A9EX99_SORC5/14-287	90.91	275	28	302	1	274	2.00E-133	471
SCE1572_5727	A9F1W2_SORC5/19-315	95.29	297	98	394	1	297	5.00E-154	540
SCE1572_5800	A9F2K1_SORC5/24-292	95.91	269	24	292	1	269	6.00E-153	536
SCE1572_5808	A9F2M6_SORC5/7-264	97.29	258	7	264	1	258	1.00E-139	491
SCE1572_5809	C1A3Y3_GEMAT/13-268	30.84	214	71	284	68	256	7.00E-14	75.9
SCE1572_5837	A9F2U6_SORC5/16-277	91.6	262	16	277	1	262	1.00E-137	486
SCE1572_5839	A9FDG9_SORC5/21-281	54.75	263	37	299	1	259	3.00E-74	275
SCE1572_5876	A9F2Y6_SORC5/28-293	78.73	268	23	290	1	266	3.00E-115	411
SCE1572_5963	B5DPB6_DROPS	31.91	257	178	428	700	944	1.00E-20	97.8
SCE1572_5967	A6G4E4_9DELT	32.77	528	47	565	829	1260	2.00E-43	174
SCE1572_5997	A9F3J7_SORC5/16-278	88.97	263	16	278	1	263	2.00E-135	478
SCE1572_6031	D3BJS3_POLPA	25.62	402	78	468	844	1223	2.00E-24	110
SCE1572_6038	A9F865_SORC5/77-280	93.63	204	228	431	1	204	4.00E-104	374
SCE1572_6062	A9F8E9_SORC5/10-280	95.94	271	10	280	1	271	7.00E-153	536
SCE1572_6075	D3BJS3_POLPA	25.81	465	18	473	816	1274	3.00E-36	149
SCE1572_6092	A9EVM0_SORC5/14-277	49.8	255	1	252	12	264	4.00E-63	237
SCE1572_6096	A9GRQ7_SORC5/16-276	46.8	250	10	254	15	261	1.00E-51	201

SCE1572_6098	A9F8N6_SORC5/25-290	90.23	266	95	360	1	266	4.00E-139	491
SCE1572_6122	A9F8W3_SORC5/41-296	97.27	256	41	296	1	256	5.00E-148	520
SCE1572_6127	A9F8Y3_SORC5/32-301	92.96	270	32	301	1	270	5.00E-143	503
SCE1572_6128	B9G748_ORYSJ	35.36	362	47	393	45	404	4.00E-53	205
SCE1572_6133	A9F900_SORC5/96-359	97.73	264	66	329	1	264	2.00E-152	534
SCE1572_6155	A9F950_SORC5/52-318	86.14	267	52	318	1	267	3.00E-133	470
SCE1572_6158	A9F9F8_SORC5/44-308	85.66	265	46	308	1	265	2.00E-118	421
SCE1572_6159	A9F9G1_SORC5/55-317	80.08	266	46	311	1	263	6.00E-100	361
SCE1572_6164	A9F9M5_SORC5/12-288	84.64	280	12	291	1	277	5.00E-122	433
SCE1572_6165	D0LZ14_HALO1	38.07	935	2	867	7	923	8.00E-144	508
SCE1572_6166	A9F9N0_SORC5/11-276	92.59	270	11	280	1	266	2.00E-145	511
SCE1572_6182	A9GBB7_SORC5/15-282	54.28	269	19	281	1	267	8.00E-71	264
SCE1572_6183	C3XWU6_BRAFL	34.08	358	305	653	840	1194	3.00E-49	193
SCE1572_6194	A9GS33_SORC5/10-280	53.33	270	10	279	1	270	1.00E-80	295
SCE1572_6202	D0LZ14_HALO1	42.3	1357	2	1291	7	1346	0	870
SCE1572_6203	B9RTW7_RICCO	40.17	234	8	236	39	266	4.00E-36	148
SCE1572_6277	B9RTW7_RICCO	36.36	198	4	192	38	229	3.00E-18	88.6
SCE1572_6322	E5SP72_TRISP	49.45	91	64	153	678	767	2.00E-15	79
SCE1572_6323	C3ZQY6_BRAFL	27.31	249	50	291	69	316	2.00E-15	80.1
SCE1572_6324	A9FF01_SORC5/27-278	97.22	252	27	278	1	252	1.00E-138	488
SCE1572_6382	D0LZ14_HALO1	39.71	1365	2	1286	7	1346	0	806
SCE1572_6384	A9FFI9_SORC5/13-292	86.43	280	13	292	1	280	9.00E-141	494
SCE1572_6462	A9GQX2_SORC5/13-284	52.81	267	3	266	6	272	4.00E-75	278
SCE1572_6468	E4YNA2_OIKDI	29.39	228	135	349	1287	1511	7.00E-15	79
SCE1572_6475	D7TTD5_VITVI	26.24	343	70	391	121	460	5.00E-22	102
SCE1572_6504	B9RTW7_RICCO	30.48	187	9	194	38	219	3.00E-15	79
SCE1572_6512	D7TTD5_VITVI	24.85	326	67	373	121	441	5.00E-20	95.5
SCE1572_6547	A9FS91_SORC5/8-267	53.19	188	21	208	75	260	2.00E-50	198

SCE1572_6563	B9RTW7_RICCO	33.85	192	17	199	38	225	3.00E-21	99
SCE1572_6585	A9GTY9_SORC5/10-268	47.43	175	1	165	93	259	1.00E-37	155
SCE1572_6594	E9CI33_9EUKA	45.15	206	236	440	36	241	6.00E-39	158
SCE1572_6634	A6G4E4_9DELT	33.76	702	1567	2237	537	1219	8.00E-68	256
SCE1572_6638	A9GS33_SORC5/10-280	57.92	259	1	258	12	269	7.00E-83	303
SCE1572_6657	A6FAX0_9GAMM/20-314	54.55	286	41	326	1	286	3.00E-87	320
SCE1572_6664	B7P9G7_IXOSC	29.79	235	137	362	578	798	1.00E-13	74.3
SCE1572_6688	A9F932_SORC5/34-318	73.33	285	377	661	1	285	8.00E-117	418
SCE1572_6735	D3BJS3_POLPA	28.33	466	74	514	822	1276	2.00E-37	154
SCE1572_6742	A9FL39_SORC5/55-319	89.33	253	69	319	1	253	4.00E-125	444
SCE1572_6800	A9FLI0_SORC5/13-287	94.18	275	35	309	1	275	5.00E-154	540
SCE1572_6833	A9FR17_SORC5/15-284	88.15	270	15	284	1	270	3.00E-137	484
SCE1572_6862	A9FRA7_SORC5/27-289	92.4	263	82	344	1	263	4.00E-144	507
SCE1572_6872	A9FRE2_SORC5/13-279	89.14	267	13	279	1	267	5.00E-130	460
SCE1572_6887	D0LZ14_HALO1	38.34	193	3	192	1157	1346	1.00E-12	68.9
SCE1572_6892	D0LZ14_HALO1	39.38	833	2	775	7	828	1.00E-138	490
SCE1572_6893	D0LZ14_HALO1	39.13	1362	2	1294	7	1346	0	779
SCE1572_6894	D0LZ14_HALO1	38.68	1352	2	1291	7	1346	0	758
SCE1572_6921	A9FP27_SORC5/11-267	95.33	257	11	267	1	257	3.00E-126	449
SCE1572_6948	A9GQV1_SORC5/7-266	57.09	261	7	267	1	260	2.00E-80	297
SCE1572_6974	D0LZ14_HALO1	40.34	932	4	868	9	917	3.00E-156	549
SCE1572_6983	A9FRW7_SORC5/16-282	90.26	267	74	340	1	267	7.00E-139	491
SCE1572_7026	B9G748_ORYSJ	29.65	226	277	498	70	286	3.00E-15	79.7
SCE1572_7035	A9FS85_SORC5/37-352	84.15	347	37	383	1	316	6.00E-137	484
SCE1572_7045	A5D1B0_PELTS/10-265	38.52	257	53	306	1	255	4.00E-49	192
SCE1572_7065	A9GS33_SORC5/10-280	54.44	270	10	279	1	270	2.00E-83	305
SCE1572_7076	A9FSE5_SORC5/14-280	91.39	267	14	280	1	267	6.00E-139	491
SCE1572_7098	A9F7S7_SORC5/16-279	86.74	264	16	279	1	264	2.00E-127	451

SCE1572_7229	A9FX40_SORC5/39-310	90.81	272	121	392	1	272	3.00E-142	501
SCE1572_7235	A9FX47_SORC5/11-274	81.82	264	42	304	1	264	2.00E-115	413
SCE1572_7236	A9FX52_SORC5/61-318	94.19	258	64	321	1	258	1.00E-139	492
SCE1572_7241	A9GRQ7_SORC5/16-276	53.64	261	55	313	1	261	2.00E-72	270
SCE1572_7268	A9FXC4_SORC5/66-318	82.68	254	63	316	1	253	2.00E-112	403
SCE1572_7270	A9FXD2_SORC5/32-294	88.21	263	31	293	1	263	2.00E-131	464
SCE1572_7299	A9GA96_SORC5/7-269	84.03	263	7	266	1	263	9.00E-126	447
SCE1572_7311	A9FYH6_SORC5/7-273	78.65	267	7	265	1	267	2.00E-117	420
SCE1572_7326	A9FYJ4_SORC5/24-284	88.51	261	85	345	1	261	7.00E-130	459
SCE1572_7338	A9FYM0_SORC5/12-281	95.93	246	2	247	25	270	8.00E-138	486
SCE1572_7353	E9CJ13_9EUKA	44.54	119	75	191	315	431	2.00E-23	107
SCE1572_7354	A9VAH9_MONBE	28.92	325	366	674	68	374	9.00E-22	102
SCE1572_7360	A9G2H5_SORC5/75-335	95.02	261	75	335	1	261	8.00E-146	513
SCE1572_7397	A9G2M3_SORC5/18-300	92.58	283	9	291	1	283	3.00E-133	471
SCE1572_7413	D7FZX4_ECTSI	30.58	399	171	505	106	497	5.00E-27	119
SCE1572_7427	A9F932_SORC5/34-318	62.93	294	84	377	1	285	2.00E-101	367
SCE1572_7443	A9FKG8_SORC5/47-312	55.81	267	38	300	1	266	1.00E-69	260
SCE1572_7456	A9G394_SORC5/21-294	90.88	274	21	294	1	274	2.00E-145	511
SCE1572_7473	A9G3H0_SORC5/24-196	96.53	173	24	196	1	173	4.00E-88	321
SCE1572_7501	A9G3U5_SORC5/23-287	93.58	265	23	287	1	265	1.00E-144	509
SCE1572_7543	D7TTD5_VITVI	22.72	471	29	481	34	480	2.00E-17	87.4
SCE1572_7579	A9G7V5_SORC5/54-335	96.45	282	54	335	1	282	3.00E-160	560
SCE1572_7601	A9G814_SORC5/50-310	91.57	261	49	309	1	261	5.00E-132	466
SCE1572_7612	D0LJH9_HALO1	30.22	460	173	613	529	917	3.00E-21	100
SCE1572_7621	B9RTW7_RICCO	32.49	197	23	216	35	224	3.00E-15	79.3
SCE1572_7637	E3X4G0_ANODA	32.77	296	1	291	14	293	5.00E-30	128
SCE1572_7641	A9G8A9_SORC5/19-269	99.2	251	19	269	1	251	4.00E-148	520
SCE1572_7643	A9G8B3_SORC5/33-299	92.88	267	48	314	1	267	5.00E-151	530

SCE1572_7669	B9L1Q1_THERP/10-268	41.73	266	336	592	1	258	3.00E-38	156
SCE1572_7697	A9G8G3_SORC5/22-297	93.12	276	40	315	1	276	2.00E-152	535
SCE1572_7707	A9G8K4_SORC5/15-290	93.09	275	15	289	1	275	9.00E-151	529
SCE1572_7865	A9GC13_SORC5/22-332	89.51	305	1	303	7	311	2.00E-138	488
SCE1572_7872	A9GC29_SORC5/7-277	96.31	271	7	277	1	271	3.00E-151	530
SCE1572_7879	D3BJS3_POLPA	28.68	401	5	397	825	1222	4.00E-42	169
SCE1572_7916	B9S6C8_RICCO	24.15	414	78	474	29	352	4.00E-13	73.2
SCE1572_7931	A9GCF7_SORC5/32-301	93.36	271	32	302	1	270	1.00E-144	508
SCE1572_7938	A9GCH4_SORC5/10-276	93.26	267	10	276	1	267	2.00E-147	518
SCE1572_7942	Q23ZG6_TETTH	23	213	137	334	625	835	4.00E-13	73.9
SCE1572_7955	A9GCK3_SORC5/48-322	85.82	275	15	289	1	275	2.00E-139	491
SCE1572_7984	A9GCS7_SORC5/22-306	92.28	285	22	306	1	285	3.00E-147	518
SCE1572_8190	A6G4E4_9DELT	27.34	578	602	1160	648	1216	4.00E-37	154
SCE1572_8240	A9GGJ5_SORC5/9-279	82.33	283	9	291	1	271	3.00E-124	440
SCE1572_8250	F2UKF9_9EUKA	27.89	484	6	457	9	474	3.00E-38	156
SCE1572_8287	A9GGW8_SORC5/20-305	94.41	286	20	305	1	286	3.00E-150	526
SCE1572_8288	B0VGL8_CLOAI	27.39	376	123	456	251	592	3.00E-16	83.2
SCE1572_8321	A9GH81_SORC5/34-292	94.21	259	38	296	1	259	3.00E-136	481
SCE1572_8378	A9GJX9_SORC5/10-273	87.5	264	5	268	1	264	2.00E-123	437
SCE1572_8383	C8RRI2_CORJE/14-279	30.83	266	9	256	1	264	1.00E-22	103
SCE1572_8391	D0LZ14_HALO1	39.99	1353	2	1278	7	1344	0	758
SCE1572_8403	E4XBZ6_OIKDI	28.65	192	105	292	1678	1869	4.00E-16	80.1
SCE1572_8417	A9GKG4_SORC5/22-288	88.76	267	22	288	1	267	1.00E-128	455
SCE1572_8429	D7TTD5_VITVI	26.49	302	142	426	179	480	3.00E-15	79.7
SCE1572_8434	A9GKK0_SORC5/84-246	92.41	158	84	239	1	158	1.00E-67	254
SCE1572_8448	A9GJS7_SORC5/31-311	79.36	281	104	382	1	281	4.00E-129	458
SCE1572_8495	D0LZ14_HALO1	37.09	1146	24	1103	7	1141	2.00E-171	600
SCE1572_8496	D0LZ14_HALO1	39.91	847	94	932	176	1015	4.00E-142	503

SCE1572_8499	D0LZ14_HALO1	37.51	909	2	802	7	905	7.00E-128	454
SCE1572_8524	A9GNU1_SORC5/8-279	91.57	261	1	261	12	272	4.00E-140	494
SCE1572_8533	A9GNW1_SORC5/30-305	95.65	276	9	284	1	276	3.00E-153	537
SCE1572_8597	D7FZX4_ECTSI	43.19	426	65	476	106	521	2.00E-70	264
SCE1572_8679	B9RTW7_RICCO	30.92	207	2	199	40	242	1.00E-16	83.6
SCE1572_8698	D0LZ14_HALO1	40.28	1003	13	950	7	981	0	630
SCE1572_8739	A9GT67_SORC5/13-276	87.12	264	35	298	1	264	4.00E-136	480
SCE1572_8749	B4CYY7_9BACT	27.67	253	91	302	722	951	3.00E-14	76.6
SCE1572_8762	D0LZ14_HALO1	39.16	835	2	775	7	827	3.00E-135	479
SCE1572_8765	A9F6M3_SORC5	55.01	349	1	336	1	347	4.00E-91	332
SCE1572_8775	D0LZ14_HALO1	28.75	546	9	528	825	1346	4.00E-34	142
SCE1572_8822	B9RTW7_RICCO	32.84	204	1	198	34	224	7.00E-15	77.4
SCE1572_8834	A9GC88_SORC5/32-303	66.18	272	138	408	1	272	4.00E-100	362
SCE1572_8876	B8LDL2_THAPS	26.43	488	77	556	997	1431	1.00E-48	192
SCE1572_8886	A9GTY0_SORC5/11-268	96.9	258	11	268	1	258	1.00E-142	503
SCE1572_8903	E9C7Z1_9EUKA	31.51	219	156	354	271	485	9.00E-14	74.7
SCE1572_8954	A9GX99_SORC5/49-311	90.11	263	9	271	1	263	1.00E-138	489
SCE1572_8968	D0LYB2_HALO1	35.9	493	43	512	349	814	1.00E-44	178
SCE1572_8987	A9GXH6_SORC5/24-319	82.83	297	24	320	1	296	2.00E-135	478
SCE1572_9027	A9GXN7_SORC5/42-300	88.46	260	43	302	1	259	8.00E-127	450
SCE1572_9056	A9VAH9_MONBE	31.42	662	45	678	81	733	1.00E-59	228
SCE1572_9060	A6G4E4_9DELT	27.73	815	523	1227	506	1260	3.00E-38	157
SCE1572_9064	A9VAH9_MONBE	32.48	391	69	446	76	466	4.00E-32	135
SCE1572_9065	Q7PTP9_ANOGA	32.27	220	344	559	818	1030	2.00E-18	90.9
SCE1572_9071	A9GXY7_SORC5/124-394	94.96	258	128	385	14	271	1.00E-142	503
SCE1572_9073	A9VA81_MONBE	34.14	249	130	366	130	353	3.00E-21	100
SCE1572_9075	A9GJS7_SORC5/31-311	80	285	103	387	1	281	9.00E-132	467
SCE1572_9112	A9GYC7_SORC5/46-317	91.94	273	44	316	1	272	1.00E-140	495

SCE1572_9177	A9ERD0_SORC5/20-276	85.99	257	20	276	1	257	5.00E-124	441
SCE1572_9204	A9ERQ0_SORC5/24-283	90	260	47	306	1	260	2.00E-135	478
SCE1572_9234	Q098I7_STIAU/99-355	71.21	257	105	360	1	257	3.00E-100	362
SCE1572_9273	A9ES80_SORC5/13-299	81.47	313	26	338	1	287	3.00E-130	460
SCE1572_9274	A9ES85_SORC5/18-279	91.98	262	18	279	1	262	1.00E-136	482
SCE1572_9286	A9ESD5_SORC5/69-334	92.11	266	11	276	1	266	4.00E-143	503
SCE1572_9290	A9ESE9_SORC5/19-289	98.44	257	1	257	15	271	6.00E-149	524
SCE1572_9346	A9ESY9_SORC5/122-403	95.74	282	122	403	1	282	7.00E-151	530
SCE1572_9383	B4KV63_DROMO	33.91	230	160	388	714	940	5.00E-36	149
SCE1572_9387	A9EXL8_SORC5/33-306	99.27	274	33	306	1	274	4.00E-165	577
SCE1572_9440	A9EY93_SORC5/26-264	94.14	239	14	252	1	239	4.00E-128	454
SCE1572_9453	A9FX52_SORC5/61-318	52.71	258	47	304	1	258	5.00E-75	277
SCE1572_9454	A9FX47_SORC5/11-274	56.32	261	46	301	4	264	4.00E-69	259
SCE1572_9464	A9EYF1_SORC5/26-299	95.26	274	24	297	1	274	2.00E-139	491
SCE1572_9490	A9EYQ5_SORC5/13-322	92.26	310	16	325	1	310	2.00E-166	581
SCE1572_9503	A5BUK4_VITVI	38.36	73	5	77	38	110	4.00E-11	63.5
SCE1572_9529	A9EZ28_SORC5/20-279	93.85	260	20	279	1	260	7.00E-142	500
SCE1572_9534	A9EZ47_SORC5/30-295	86.84	266	26	286	1	266	2.00E-123	439
SCE1572_9543	E9CFQ5_9EUKA	32.68	205	167	344	231	433	5.00E-20	95.5
SCE1572_9561	Q099F0_STIAU/22-311	55.81	301	53	347	1	290	1.00E-76	284
SCE1572_9570	A9GTY9_SORC5/10-268	47.69	260	12	271	1	259	4.00E-61	233
SCE1572_9627	B8LDL2_THAPS	23.81	483	1	472	1038	1467	2.00E-32	139
SCE1572_9635	E5SP72_TRISP	41.23	114	204	313	653	765	8.00E-13	71.2
SCE1572_9698	A9F548_SORC5/73-334	90.84	262	88	349	1	262	8.00E-134	472
SCE1572_9699	A9F553_SORC5/15-290	89.86	276	15	290	1	276	7.00E-142	499
SCE1572_9718	E9C1L0_9EUKA	32.08	212	210	421	955	1155	7.00E-22	101
SCE1572_9733	A9F5E0_SORC5/14-276	93.16	263	14	276	1	263	1.00E-146	515
SCE1572_9768	A9F5P2_SORC5/100-253	81.82	154	100	250	1	154	3.00E-62	234

SCE1572_9776	A9F5R7_SORC5/10-285	89.49	276	37	312	1	276	2.00E-137	485
SCE1572_9812	A9FA64_SORC5/37-294	73.66	262	16	277	1	258	8.00E-110	395
SCE1572_9849	A9FAC8_SORC5/14-277	93.94	264	14	277	1	264	1.00E-149	525
SCE1572_9893	A6G4E4_9DELT	30.84	454	508	955	788	1197	3.00E-36	151
SCE1572_9895	A9FAX2_SORC5/29-287	95.75	259	29	287	1	259	2.00E-143	505
SCE1572_9959	A6GDW6_9DELT	42.6	986	90	1024	29	1004	3.00E-140	496
SCE1572_10034	A9FG39_SORC5/21-290	90	270	25	294	1	270	7.00E-143	503
SCE1572_10048	A9FG77_SORC5/27-293	94.38	267	27	293	1	267	7.00E-151	529
SCE1572_10051	A9FG88_SORC5/13-278	96.62	266	77	342	1	266	2.00E-149	525
SCE1572_10155	A9FH30_SORC5/261-495	94.89	235	256	490	1	235	9.00E-116	413
SCE1572_10170	A9FH69_SORC5/13-277	93.21	265	13	276	1	265	3.00E-139	491
SCE1572_10242	A9F6M3_SORC5	57.64	314	1	303	1	313	2.00E-90	329
SCE1572_10268	Q23ZG6_TETTH	26.18	359	145	466	619	964	1.00E-22	105
SCE1572_10276	E3PW85_CLOSD	39.24	158	141	298	1414	1569	7.00E-28	120
SCE1572_10294	E4YNA2_OIKDI	28.27	329	8	289	1152	1458	1.00E-16	84.7
SCE1572_10347	A9FHU2_SORC5/67-328	94.66	262	66	327	1	262	2.00E-145	511
SCE1572_10352	A6G4E4_9DELT	29.35	293	26	308	926	1213	3.00E-12	69.3
SCE1572_10373	D7SRK5_VITVI	30.12	259	50	289	24	276	7.00E-16	82
SCE1572_10391	D7TV01_VITVI	30.98	410	70	471	488	869	9.00E-39	157
SCE1572_10405	E7F1M5_DANRE	38.58	197	182	325	98	294	3.00E-12	69.7
SCE1572_10476	A9V9Z1_MONBE	31.82	286	1	263	948	1214	3.00E-22	102
SCE1572_10484	A9FMI8_SORC5/61-326	92.08	265	35	298	1	265	6.00E-141	496
SCE1572_10529	B4KV63_DROMO	27.74	274	403	663	677	939	1.00E-13	75.1
SCE1572_10654	A9FNS6_SORC5/28-292	98.11	265	18	282	1	265	2.00E-147	517
SCE1572_10719	E3PW85_CLOSD	35.47	172	541	712	1413	1578	4.00E-26	116
SCE1572_10740	B9RTW7_RICCO	33.5	203	1	198	34	224	1.00E-14	76.6
SCE1572_10785	A9FU56_SORC5/23-293	92.99	271	23	293	1	271	6.00E-152	533
SCE1572_10787	A9FU59_SORC5/20-287	91.04	268	59	326	1	268	2.00E-144	508

SCE1572_10800	A9G0Y5_SORC5/28-295	63.16	266	28	293	1	266	3.00E-93	338
SCE1572_10832	C3ZSQ8_BRAFL	40.32	124	498	621	1640	1761	3.00E-18	90.1
SCE1572_10834	Q23ZG6_TETTH	18.88	466	126	518	578	1032	3.00E-11	67.8
SCE1572_10838	D7FZX4_ECTSI	29.47	397	146	480	106	496	1.00E-19	94.7
SCE1572_10868	E9IEB9_SOLIN	39.81	103	228	329	237	337	1.00E-10	66.6
SCE1572_10892	A9FYY9_SORC5/32-312	93.59	281	32	312	1	281	4.00E-156	548
SCE1572_10919	B9RTW7_RICCO	30.9	233	2	230	34	258	6.00E-15	77.8
SCE1572_10924	A9FZ90_SORC5/7-269	89.74	273	7	279	1	263	5.00E-142	502
SCE1572_10928	A9FZ94_SORC5/32-347	91.77	316	32	347	1	316	2.00E-172	601
SCE1572_10995	A9G0J1_SORC5/93-365	88.64	273	16	288	1	273	4.00E-146	513
SCE1572_11043	A9G4G1_SORC5/11-269	92.66	259	11	269	1	259	3.00E-140	495
SCE1572_11046	A9G4G4_SORC5/74-341	83.08	260	74	333	1	259	1.00E-112	404
SCE1572_11048	A9G4H1_SORC5/35-296	94.44	234	1	234	27	260	3.00E-126	447
SCE1572_11102	B9RTW7_RICCO	30.89	191	3	189	38	224	1.00E-11	67
SCE1572_11141	A9V7M5_MONBE	29.36	327	404	717	71	382	4.00E-13	73.6
SCE1572_11143	B9RTW7_RICCO	32.12	193	6	192	38	224	3.00E-11	65.1
SCE1572_11169	D8LHV1_ECTSI	27.61	326	246	567	31	320	4.00E-13	73.2
SCE1572_11185	B9RTW7_RICCO	30.41	194	6	195	39	225	3.00E-12	69.3
SCE1572_11206	D0LZ14_HALO1	39.67	1220	103	1304	163	1344	0	723
SCE1572_11215	A9GJS7_SORC5/31-311	64.34	286	85	369	1	280	2.00E-97	352
SCE1572_11281	A9G9I7_SORC5/19-323	86.56	305	65	369	1	305	3.00E-141	499
SCE1572_11313	D7U846_VITVI	53.25	169	3	171	657	825	4.00E-45	177
SCE1572_11364	A9GD46_SORC5/12-271	89.62	260	12	271	1	260	3.00E-135	479
SCE1572_11382	A9GD84_SORC5/84-356	95.6	273	60	332	1	273	4.00E-149	523
SCE1572_11384	A9GD87_SORC5/29-299	97.42	271	29	299	1	271	1.00E-158	555
SCE1572_11385	D0LYB2_HALO1	24.13	630	395	986	345	937	9.00E-13	73.2
SCE1572_11393	A9GDB7_SORC5/16-307	93.14	277	31	306	16	292	6.00E-148	521
SCE1572_11400	A9GDD6_SORC5/1-262	93.89	262	27	288	1	262	2.00E-144	508

SCE1572_11413	D3BJS3_POLPA	27.9	405	23	419	822	1222	6.00E-40	162
SCE1572_11432	A9GDN2_SORC5/49-311	92.02	263	49	311	1	263	1.00E-137	485
SCE1572_11467	A9GS33_SORC5/10-280	65.93	270	10	278	1	270	5.00E-104	374
SCE1572_11561	A9GS33_SORC5/10-280	56.83	271	10	279	1	270	1.00E-84	309
SCE1572_11581	A9GHH8_SORC5/49-320	91.54	272	49	320	1	272	8.00E-134	472
SCE1572_11582	A9GHI1_SORC5/382-646	80.68	264	382	645	1	264	2.00E-108	389
SCE1572_11584	A9GHP7_SORC5/10-312	83.61	305	15	315	1	303	5.00E-127	450

* Blasting against three Pfam families, PF00069, PF00433 and PF07714 with a cutoff 1e-10

Supplementary Table S2. CRISPR statistics in Myxobacteria.

Strains	Genome size(bp)	Convincing CRISPR structure	Length (bp)*
Corallococcus coralloides DSM 2259	10,080,619	7	2805
Anaeromyxobacter dehalogenans 2CP-1	5,029,329	2	2131
Anaeromyxobacter dehalogenans 2CP-C	5,013,479	0	0
Anaeromyxobacter sp. Fw109-5	5,277,990	0	0
Anaeromyxobacter sp. K	5,061,632	2	4507
Haliangium ochraceum DSM 14365	9,446,314	3	58594
Myxococcus fulvus HW-1	9,003,593	4	5903
Myxococcus xanthus DK 1622	9,139,763	4	10137
Sorangium cellulosum 'So ce 56'	13,033,779	5	12726
Stigmatella aurantiaca DW4/3-1	10,260,756	1	176
Sorangium cellulosum So0157-2	14,782,435	3	6572

* Just statistics of convincing CRISPR structure.

Supplementary Table S3 Primer and statistical results of qPCR experiment.

Locus tag	Product name	Forward primer (5'-3')	Reverse primer (5'-3')	RNA-Seq Reads* (pH7.0/pH9.0)	logFC (RNA-Seq)**	**ΔΔCt (qPCR) #1**	**ΔΔCt (qPCR) #2**
SCE1572_3207	gliding motility protein MglA	GGCATCGTGTTCGTCAGC	GGAACGGGTTCAACATCG	91/439	-3.54	-1.03	-1.44
SCE1572_3710	RNA-binding protein	CGTCTTTACGTCGGCAATC	GCCTCGTTGACCTTGAGC	89/85	-5.05	-1.20	-1.97
SCE1572_6018	RNA polymerase sigma factor RpoD	GTGCGGAAGGTCCTGAAG	CGAGATCACGCTCTTGTCC	2640/712	0.62	0.3	0.61
SCE1572_6365	Macrolide-specific efflux protein MacA	GGTGGAGAAGGGGACGCT	GACCACGCCCTGGAACAC	108/500	-3.48	-3.54	-2.7
SCE1572_7438	Cell division protein FtsZ	CCTCCCCAAGGACCAGTG	AAGCCGAGGTAGCCGAAG	347/1317	-3.20	-0.47	-2.57
SCE1572_7464 (reference)	Urea ABC transporter, ATPase protein UrtD	GGACGGGCTCGACTTCTC	CACCTCCGTGGAACACCA	412/162	0.08	NA	NA
SCE1572_7815	Heat shock protein 60 family chaperone GroEL	AGGGCAAGGACTCGTTCG	TCGGTGGTGAGCATCAGG	3485/17984	-3.64	-1.62	-3.02
SCE1572_8437	Outer membrane protein A precursor	GTGGAGCACAACATCAAGCTG	TCGTCGTTCGTGAAGCAGA	153/25	1.34	0.04	-2

NA: Not applicable.

*These reads were produced by Illumina Sequencer directly.

** The logarithm values of fold-change (FC) were calculated from the reads after they were normalized by Fragments Per Kilobase of transcript per Million mapped reads (FPKM) using the edgeR program (Reference 53 in the text).

Supplementary Table S5. Statistics of big genome in prokaryote.

Organisms	Genome sizes	Genes	GC content	Niche	Accession
Sorangium cellulosum So0157-2	14.87	11599	0.721	Soil	CP003969
Sorangium cellulosum So ce 56	13.03	9384	0.714	Soil	AM746676
Streptomyces bingchenggensis BCW-1	11.94	10022	0.708	Soil	CP002047
Streptomyces violaceusniger Tü 4113	10.66	8482	0.709	Soil	CP002994
Streptosporangium roseum DSM 43021	10.34	8945	0.7086	Vegetable garden soil	CP001814
Stigmatella aurantiaca DW4/3-1	10.26	8352	0.675	Soil	CP002271
Amycolatopsis mediterranei S699	10.24	9575	0.713	Pine arboretum soil	CP002896
Amycolatopsis mediterranei U32	10.24	9228	0.713	Soil	CP002000
Catenulispora acidiphila DSM 44928	10.17	8914	0.6977	Forest soil, acidophilic	CP001700
Streptomyces hygroscopicus subsp. *jinggangensis* 5008	10.15	8849	0.719	Soil	CP003275
Streptomyces scabiei 87.22	10.15	8809	0.7	Deep-pitted scab lesion on a potato	FN554889
Corallococcus coralloides DSM 2259	10.08	8033	0.699	Soil	CP003389
Solibacter usitatus Ellin6076	9.97	7826	0.619	Soil	CP000473
Streptomyces davawensis JCM 4913	9.47	8503	0.7058	Soil, near Davao Air Port, Mindanao, the Philippines	PRJEB184
Haliangium ochraceum DSM 14365	9.45	6719	0.69	Sea grass samples	CP001804
Nocardia brasiliensis ATCC 700358 HUJEG-1	9.44	8414	0.68	Human mycetoma	AIHV00000000
Saccharothrix espanaensis DSM 44229	9.36	8427	0.7219	Soil	HE804045
Actinoplanes species SE50/110	9.24	8247	0.7132	Soil	CP003170
Bradyrhizobium japonicum USDA 6	9.21	8829	0.64	Glycine max， a soybean plant	AP012206
Myxococcus xanthus DK 1622	9.14	7331	0.689	Soil	CP000113
Chitinophaga pinensis DSM 2588	9.13	7192	0.4523	Infusion of litter from	CP001699

				the base of a pine tree	
Bradyrhizobium japonicum USDA 110	9.11	8317	0.64	Glycine max， a soybean plant	BA000040
Niastella koreensis GR20-10	9.03	7174	0.45	Korean ginseng soil	CP003178
Streptomyces avermitilis MA-4680	9.03	7580	0.707	Soil	BA000030
Myxococcus fulvus HW-1	9	7284	0.706	Marine	CP002830
Frankia species EAN1pec	8.98	7191	0.71	Soybean plant	CP000820
Frankia species EuI1c	8.82	7083	0.72	Soybean plant	CP002299
Kitasatospora setae KM-6054	8.78	7569	0.742	Soil	AP010968
Actinoplanes missouriensis 431	8.77	8125	0.71	Soil	AP012319
Paenibacillus mucilaginosus K02	8.77	7253	0.58	Soil	CP003422
Paenibacillus mucilaginosus 3016	8.74	7057	0.583	Rhizosphere soil	CP003235
Streptomyces coelicolor A3(2)	8.67	7825	0.721	Soil	AL645882
Paenibacillus mucilaginosus KNP414	8.66	7804	0.58	Soil	CP002869
Streptomyces griseus subsp. *griseus* NBRC 13350	8.55	7138	0.722	Soil	AP009493
Haliscomenobacter hydrossis DSM 1100	8.37	6451	0.471	Activated sludge samples	CP002691
Bradyrhizobium species BTAi1	8.26	7394	0.65	Soybean plant	CP000494
Actinosynnema mirum DSM 43827	8.25	6916	0.7371	Grass blade	CP001630
Nostoc punctiforme PCC 73102	8.23	6087	0.41	Symbiotic association with the gymnosperm cycad Macrozamia sp.	CP001037
Streptomyces venezuelae ATCC 10712	8.23	7453	0.724	Soil	FR845719
Saccharopolyspora erythraea NRRL 2338 (UCambridge)	8.21	7198	0.7129	Soil	AM420293
Spirosoma linguale DSM 74	8.08	6524	0.5015	Laboratory water bath	CP001769
Burkholderia xenovorans LB400	9.73	9059	0.63	PCB (polychlorinated biphenyl)-containing landfill	CP000270
Rhodococcus jostii RHA1	9.7	9242	0.67	Lindane-contaminated	CP000431

				soil	
Streptomyces sviceus ATCC 29083	9.31	8275	0.7	Soil	CM000951
Streptomyces clavuligerus ATCC 27064	9.13	7680	0.72	Soil	CM001015
Burkholderia gladioli BSR3	9.05	7493	0.67	Diseased rice sheath	CP002600
Burkholderia phymatum STM815	8.68	7574	0.62	Soybean plant	CP001043
Burkholderia cepacia 383	8.68	7828	0.66	Forest soil	CP000152
Cupriavidus necator N-1, ATCC 43291	8.48	7967	0.65	Soil, described as a nonobligate predator of soil bacteria and fungi and resistance against heavy metal	CP002878
Burkholderia vietnamiensis G4	8.39	7880	0.66	Hazardous environmental contaminants in groundwaters	CP000616
Acaryochloris marina MBIC11017	8.36	8488	0.47	Marine	CP000828
Burkholderia phytofirmans PsJN	8.21	7487	0.62	Glomus vesiculiferum-infected onion roots	CP001053
Streptomyces pristinaespiralis ATCC 25486	8.13	6937	0.71	Soil	CM000950
Streptomyces cattleya DSM 46488	8.09	7650	0.73	Soil	CP003219
Streptomyces cattleya NRRL 8057	8.09	7585	0.73	Soil	FQ859185
Burkholderia cenocepacia J2315	8.05	7229	0.67	Lungs of cystic fibrosis (CF) patients	AM747722

Supplementary Table S6. The sequencing library used for assembly of the So0157-2 genome.

Sample ID	Fragment length(bp)	Raw data(Mb)	Clean Data(Mb)	Pair-end type
So0157-2	274	2,022	869	(75;75)
So0157-2	491	1,862	1,102	(75,75)
So0157-2	242	376	353	(44,44)
So0157-2	6000	1,810	649	(44;44)
So0157-2	250	2,386	973	(70;70)
So0157-2	350	2,830	1,278	(44;44)
So0157-2	2500	679	525	(70;70)

Supplementary Table S10. The structure of the ribosomal RNA operon in So0157-2 and So ce56.

	Sorangium cellulosum So ce56					*S. cellulosum* So0157-2		
RNA operon Stucture	16S rRNA→ 23S rRNA	16S rRNA→ 23S rRNA→ 5S rRNA	16S rRNA →23S rRNA→5S rRNA	16S rRNA→ 23S rRNA→ 5S rRNA	16S rRNA →23S rRNA	16S rRNA→ 23S rRNA→ 5S rRNA(×3)	16SRNA→ 23SRNA→ 5SRNA	16S rRNA→5S rRNA
tRNA	—	tRNA-Ile-GAT,tRNA-Ala-TGC	—	tRNA-Ile-GAT	—	tRNA-Ile-GAT，tRNA-Ala-TGC	—	tRNA-Ile-GAT
interuption or duplication	Absence of 5S rRNA	—	—	—	Absence of 5S rRNA	—	—	Absence of 23S rRNA
repeat						5S rRNA repeat		

Supplementary Table S12. BLAST analysis comparing the *E. coli* K12 strain's LPS gene cluster with the So0157-2 database.

Gene ID	Gene symbol	Source		E-value*	Hits
lipid A					
lpxA	UDP-N-acetylglucosamine acetyltransferase	*E. coli*	+	4.00E-05	1
lpxB	tetraacyldisaccharide-1-P synthase	*E. coli*	-		
lpxC	UDP-3-O-acyl N-acetylglucosamine deacetylase	*E. coli*	-		
lpxD	UDP-3-O-(3-hydroxymyristoyl)-glucosamine N-acyltransferase	*E. coli*	-		
lpxH	UDP-2,3-diacylglucosamine pyrophosphatase	*E. coli*	-		
lpxK	lipid A 4kinase	*E. coli*	-		
lpxL	lauryl-acyl carrier protein (ACP)-dependent acyltransferase carrier	*E. coli*	-		
lpxM	myristoyl-acyl carrier protein (ACP)-dependent acyltransferase carrier	*E. coli*	-		
lpxP	palmitoleoyl-acyl carrier protein (ACP)-dependent acyltransferase carrier	*E. coli*	-		
core oligosaccharide					
rfaB	UDP-D-galactose:(glucosyl)lipopolysaccharide-1,6-D-galactosyltransferase	*E. coli*	+	4.00E-10	7
rfaC	ADP-heptose:LPS heptosyl transferase I	*E. coli*	-		
rfaD	ADP-L-glycero-D-mannoheptose-6-epimerase, NAD(P)-binding	*E. coli*	+	7.00E-13	8
rfaE	fused heptose 7-phosphate kinase and heptose 1-phosphate adenyltransferase	*E. coli*	+	5.00E-09	2
rfaF	ADP-heptose:LPS heptosyltransferase II	*E. coli*	-		
rfaG	glucosyltransferase I	*E. coli*	+	8.00E-12	11
rfaH	DNA-binding transcriptional antiterminator	*E. coli*	-		
rfaI	UDP-D-galactose:(glucosyl)lipopolysaccharide-alpha-1,3-D-galactosyltransferase	*E. coli*	-		
rfaJ	UDP-D-glucose:(galactosyl)lipopolysaccharide glucosyltransferase	*E. coli*	-		
rfaK	lipopolysaccharide core biosynthesis	*E. coli*	-		
rfaL	O-antigen ligase	*E. coli*	-		
rfaP	kinase that phosphorylates core heptose of lipopolysaccharide	*E. coli*	-		
rfaQ	lipopolysaccharide core biosynthesis protein	*E. coli*	-		

rfaS	lipopolysaccharide core biosynthesis protein	*E. coli*	-		
rfaY	lipopolysaccharide core biosynthesis protein	*E. coli*	-		
rfaZ	lipopolysaccharide core biosynthesis protein	*E. coli*	-		
O polysaccharide					
rfbX	predicted polisoprenol-linked O-antigen transporter	*E. coli*	-		
rfbC	dTDP-4-deoxyrhamnose-3,5-epimerase	*E. coli*	-		
rfbA	glucose-1-phosphate thymidylyltransferase	*E. coli*	+	6.00E-14	2
rfbD	dTDP-4-dehydrorhamnose reductase subunit, NAD(P)-binding, of dTDP-L-rhamnose synthase	*E. coli*	+	7.00E-20	1
rfbB	dTDP-glucose 4,6 dehydratase, NAD(P)-binding	*E. coli*	+	3.00E-31	17

*cutoff of Blast is 0.0001

Supplementary Table S13. Chemotaxis in *Sorangium cellulosum* So157-2.

Query ID	Subject ID	E-value*	Bit score	Family
SCE1572_4806	MXAN_2682	1.00E-24	103	Che4
SCE1572_7946	MXAN_2682	1.00E-24	103	Che4
SCE1572_4577	MXAN_2682	7.00E-26	106	Che4
SCE1572_7810	MXAN_2684	1.00E-13	64.7	Che4
SCE1572_9390	MXAN_2684	2.00E-13	65.1	Che4
SCE1572_10471	MXAN_2684	4.00E-14	68.6	Che4
SCE1572_411	MXAN_2684	2.00E-14	69.7	Che4
SCE1572_2795	MXAN_2684	1.00E-18	79.7	Che4
SCE1572_3799	MXAN_2689	1.00E-27	112	Che4
SCE1572_5466	MXAN_2689	1.00E-105	370	Che4
SCE1572_1358	MXAN_2689	2.00E-128	446	Che4
SCE1572_2376	MXAN_4138	2.00E-24	102	Frz (Che1)
SCE1572_7523	MXAN_4140	7.00E-11	58.2	Frz (Che1)
SCE1572_3185	MXAN_4140	4.00E-11	58.5	Frz (Che1)
SCE1572_10862	MXAN_4140	1.00E-12	63.5	Frz (Che1)
SCE1572_6546	MXAN_4140	9.00E-14	66.2	Frz (Che1)
SCE1572_9886	MXAN_4140	1.00E-13	67	Frz (Che1)
SCE1572_6923	MXAN_4140	4.00E-16	75.5	Frz (Che1)
SCE1572_9628	MXAN_4140	1.00E-16	75.9	Frz (Che1)
SCE1572_4879	MXAN_4140	5.00E-17	78.2	Frz (Che1)
SCE1572_4349	MXAN_4140	2.00E-17	80.1	Frz (Che1)
SCE1572_1207	MXAN_4140	8.00E-42	159	Frz (Che1)
SCE1572_1851	MXAN_4144	3.00E-12	59.3	Frz (Che1)
SCE1572_7738	MXAN_4149	6.00E-13	64.7	Frz (Che1)
SCE1572_9885	MXAN_4751	2.00E-15	70.9	Che8
SCE1572_5352	MXAN_4751	2.00E-17	79	Che8

SCE1572_2409	MXAN_4751	8.00E-31	120	Che8
SCE1572_5882	MXAN_4752	2.00E-12	60.5	Che8
SCE1572_2235	MXAN_4752	1.00E-71	258	Che8
SCE1572_5865	MXAN_4753	4.00E-32	128	Che8
SCE1572_2234	MXAN_4753	5.00E-63	229	Che8
SCE1572_2798	MXAN_4756	1.00E-10	54.3	Che8
SCE1572_2232	MXAN_4756	2.00E-12	59.7	Che8
SCE1572_2231	MXAN_4757	4.00E-14	65.5	Che8
SCE1572_1252	MXAN_4758	2.00E-28	115	Che8
SCE1572_2230	MXAN_4758	7.00E-39	150	Che8
SCE1572_2797	MXAN_4758	6.00E-55	202	Che8
SCE1572_2230	MXAN_4758	3.00E-101	357	Che8
SCE1572_1887	MXAN_4759	5.00E-12	60.8	Che8
SCE1572_9096	MXAN_4759	5.00E-19	81.6	Che8
SCE1572_2229	MXAN_4759	3.00E-25	102	Che8
SCE1572_1205	MXAN_5144	2.00E-18	81.3	Che3
SCE1572_5867	MXAN_5145	8.00E-25	102	Che3
SCE1572_4573	MXAN_5145	9.00E-59	215	Che3
SCE1572_9153	MXAN_5147	7.00E-11	54.7	Che3
SCE1572_4252	MXAN_5147	2.00E-11	56.2	Che3
SCE1572_2742	MXAN_5147	1.00E-11	57.8	Che3
SCE1572_10639	MXAN_5147	3.00E-11	57.8	Che3
SCE1572_11082	MXAN_5147	1.00E-11	57.8	Che3
SCE1572_10447	MXAN_5147	3.00E-11	58.5	Che3
SCE1572_9669	MXAN_5147	2.00E-12	60.5	Che3
SCE1572_6975	MXAN_5147	2.00E-12	60.8	Che3
SCE1572_6485	MXAN_5147	6.00E-13	62	Che3
SCE1572_7521	MXAN_5147	3.00E-13	62	Che3
SCE1572_3835	MXAN_5147	2.00E-12	62.4	Che3
SCE1572_6394	MXAN_5147	2.00E-13	62.8	Che3
SCE1572_10286	MXAN_5147	2.00E-13	63.5	Che3
SCE1572_2296	MXAN_5147	5.00E-13	63.9	Che3
SCE1572_933	MXAN_5147	7.00E-13	64.3	Che3
SCE1572_1254	MXAN_5147	7.00E-14	64.3	Che3
SCE1572_9214	MXAN_5147	5.00E-13	64.7	Che3
SCE1572_11146	MXAN_5147	3.00E-13	64.7	Che3
SCE1572_1850	MXAN_5147	2.00E-13	65.9	Che3
SCE1572_8842	MXAN_5147	5.00E-14	66.2	Che3
SCE1572_3496	MXAN_5147	1.00E-13	67	Che3
SCE1572_3496	MXAN_5147	3.00E-14	68.9	Che3
SCE1572_6547	MXAN_5147	5.00E-14	68.9	Che3
SCE1572_3872	MXAN_5147	2.00E-15	71.6	Che3

SCE1572_1238	MXAN_5147	5.00E-16	72.4	Che3
SCE1572_6251	MXAN_5147	2.00E-15	72.8	Che3
SCE1572_6606	MXAN_5147	3.00E-16	74.3	Che3
SCE1572_6664	MXAN_5147	4.00E-17	77	Che3
SCE1572_4572	MXAN_5147	1.00E-17	78.6	Che3
SCE1572_8841	MXAN_5147	5.00E-18	81.3	Che3
SCE1572_1208	MXAN_5148	5.00E-33	130	Che3
SCE1572_9692	MXAN_5153	3.00E-12	59.7	Che3
SCE1572_9926	MXAN_5153	7.00E-13	61.2	Che3
SCE1572_8613	MXAN_5153	6.00E-13	62.4	Che3
SCE1572_8913	MXAN_5153	4.00E-14	65.1	Che3
SCE1572_2647	MXAN_5153	4.00E-14	67.8	Che3
SCE1572_3367	MXAN_5153	8.00E-15	69.7	Che3
SCE1572_430	MXAN_5153	3.00E-16	74.7	Che3
SCE1572_1974	MXAN_5153	2.00E-18	79.7	Che3
SCE1572_10567	MXAN_5153	2.00E-26	108	Che3
SCE1572_3150	MXAN_5153	2.00E-28	114	Che3
SCE1572_3873	MXAN_5153	1.00E-28	115	Che3
SCE1572_1470	MXAN_5153	6.00E-29	117	Che3
SCE1572_1939	MXAN_5153	2.00E-29	118	Che3
SCE1572_11212	MXAN_5153	5.00E-35	136	Che3
SCE1572_9209	MXAN_5153	2.00E-35	138	Che3
SCE1572_6814	MXAN_5153	2.00E-36	141	Che3
SCE1572_3740	MXAN_5153	7.00E-37	143	Che3
SCE1572_2589	MXAN_5153	4.00E-37	144	Che3
SCE1572_8957	MXAN_5153	5.00E-38	146	Che3
SCE1572_4341	MXAN_5153	4.00E-39	150	Che3
SCE1572_7059	MXAN_5153	3.00E-39	150	Che3
SCE1572_8480	MXAN_5153	2.00E-39	151	Che3
SCE1572_8199	MXAN_5153	1.00E-39	152	Che3
SCE1572_10539	MXAN_5153	8.00E-40	153	Che3
SCE1572_6839	MXAN_5153	2.00E-40	154	Che3
SCE1572_5366	MXAN_5153	8.00E-41	155	Che3
SCE1572_8888	MXAN_5153	6.00E-41	155	Che3
SCE1572_9233	MXAN_5153	1.00E-42	162	Che3
SCE1572_432	MXAN_5153	5.00E-43	163	Che3
SCE1572_6162	MXAN_5153	1.00E-43	165	Che3
SCE1572_3179	MXAN_5153	2.00E-44	167	Che3
SCE1572_5537	MXAN_5153	4.00E-45	170	Che3
SCE1572_5925	MXAN_5153	2.00E-45	171	Che3
SCE1572_3259	MXAN_5153	1.00E-45	172	Che3
SCE1572_10720	MXAN_5153	6.00E-47	176	Che3
SCE1572_701	MXAN_5153	3.00E-47	177	Che3

SCE1572_1018	MXAN_5153	3.00E-48	180	Che3
SCE1572_3063	MXAN_5153	3.00E-48	180	Che3
SCE1572_3661	MXAN_5153	1.00E-48	181	Che3
SCE1572_6637	MXAN_5153	2.00E-48	181	Che3
SCE1572_7037	MXAN_5153	1.00E-48	181	Che3
SCE1572_5058	MXAN_5153	7.00E-49	182	Che3
SCE1572_9084	MXAN_5153	1.00E-48	182	Che3
SCE1572_3607	MXAN_5153	2.00E-49	184	Che3
SCE1572_383	MXAN_5153	1.00E-50	188	Che3
SCE1572_4536	MXAN_5153	2.00E-50	188	Che3
SCE1572_11280	MXAN_5153	1.00E-50	188	Che3
SCE1572_2644	MXAN_5153	1.00E-50	189	Che3
SCE1572_3040	MXAN_5153	1.00E-51	192	Che3
SCE1572_8245	MXAN_5153	7.00E-52	192	Che3
SCE1572_5021	MXAN_5153	2.00E-52	194	Che3
SCE1572_5602	MXAN_5153	2.00E-52	194	Che3
SCE1572_4565	MXAN_5153	2.00E-52	195	Che3
SCE1572_7337	MXAN_5153	4.00E-53	196	Che3
SCE1572_2072	MXAN_5153	2.00E-55	204	Che3
SCE1572_11300	MXAN_5153	2.00E-55	204	Che3
SCE1572_3798	MXAN_5153	1.00E-55	205	Che3
SCE1572_9960	MXAN_5153	7.00E-56	206	Che3
SCE1572_3571	MXAN_5153	1.00E-56	208	Che3
SCE1572_7435	MXAN_5153	2.00E-57	210	Che3
SCE1572_1966	MXAN_5153	4.00E-58	213	Che3
SCE1572_1420	MXAN_5153	1.00E-58	215	Che3
SCE1572_9989	MXAN_5153	1.00E-58	215	Che3
SCE1572_4441	MXAN_5153	3.00E-59	217	Che3
SCE1572_5776	MXAN_5153	2.00E-59	218	Che3
SCE1572_5412	MXAN_5153	7.00E-60	219	Che3
SCE1572_990	MXAN_5153	5.00E-60	220	Che3
SCE1572_3609	MXAN_5153	3.00E-60	220	Che3
SCE1572_3923	MXAN_5153	8.00E-60	220	Che3
SCE1572_2120	MXAN_5153	6.00E-61	223	Che3
SCE1572_535	MXAN_5153	5.00E-61	224	Che3
SCE1572_429	MXAN_5153	2.00E-63	231	Che3
SCE1572_6987	MXAN_5153	3.00E-63	231	Che3
SCE1572_7038	MXAN_5153	3.00E-66	240	Che3
SCE1572_8695	MXAN_5153	1.00E-66	241	Che3
SCE1572_9357	MXAN_5153	5.00E-70	253	Che3
SCE1572_4010	MXAN_5153	2.00E-70	254	Che3
SCE1572_4397	MXAN_5153	7.00E-73	262	Che3
SCE1572_7927	MXAN_5153	5.00E-76	272	Che3

SCE1572_9037	MXAN_5153	3.00E-76	273	Che3
SCE1572_10380	MXAN_5153	1.00E-76	275	Che3
SCE1572_11034	MXAN_5153	2.00E-79	284	Che3
SCE1572_9916	MXAN_5153	2.00E-80	287	Che3
SCE1572_7624	MXAN_5153	1.00E-80	288	Che3
SCE1572_6927	MXAN_5153	4.00E-82	293	Che3
SCE1572_2291	MXAN_5153	3.00E-84	300	Che3
SCE1572_3905	MXAN_5153	2.00E-85	304	Che3
SCE1572_2333	MXAN_5153	1.00E-90	321	Che3
SCE1572_4820	MXAN_5153	4.00E-96	339	Che3
SCE1572_3384	MXAN_5153	2.00E-96	340	Che3
SCE1572_11450	MXAN_5153	1.00E-96	341	Che3
SCE1572_6665	MXAN_6029	1.00E-13	65.1	Che5
SCE1572_4205	MXAN_6031	5.00E-16	72.8	Che5
SCE1572_10949	MXAN_6032	2.00E-11	55.8	Che5
SCE1572_7680	MXAN_6032	1.00E-11	57.8	Che5
SCE1572_6666	MXAN_6032	4.00E-11	58.2	Che5
SCE1572_3219	MXAN_6032	4.00E-12	58.9	Che5
SCE1572_5331	MXAN_6032	1.00E-13	65.5	Che5
SCE1572_10791	MXAN_6032	6.00E-15	69.7	Che5
SCE1572_1281	MXAN_6032	4.00E-18	81.6	Che5
SCE1572_3493	MXAN_6033	1.00E-11	60.1	Che5
SCE1572_1276	MXAN_6692	4.00E-21	91.3	Dif (Che2)
SCE1572_12	MXAN_6693	5.00E-11	56.2	Dif (Che2)
SCE1572_6549	MXAN_6693	2.00E-13	63.5	Dif (Che2)
SCE1572_910	MXAN_6693	2.00E-15	73.9	Dif (Che2)
SCE1572_9627	MXAN_6693	5.00E-17	79	Dif (Che2)
SCE1572_4575	MXAN_6950	1.00E-26	108	Che6
SCE1572_2800	MXAN_6950	6.00E-31	123	Che6
SCE1572_10164	MXAN_6951	1.00E-11	56.6	Che6
SCE1572_9924	MXAN_6951	8.00E-13	64.3	Che6
SCE1572_6860	MXAN_6951	1.00E-13	67.4	Che6
SCE1572_1227	MXAN_6951	1.00E-14	68.6	Che6
SCE1572_4574	MXAN_6951	3.00E-32	128	Che6
SCE1572_3503	MXAN_6959	2.00E-79	283	Che6
SCE1572_2799	MXAN_6960	2.00E-19	84.7	Che6
SCE1572_3502	MXAN_6960	5.00E-81	288	Che6
SCE1572_11255	MXAN_6962	7.00E-25	103	Che6
SCE1572_3500	MXAN_6962	3.00E-80	288	Che6

SCE1572_3499	MXAN_6963	8.00E-36	137	Che6
SCE1572_3498	MXAN_6964	3.00E-128	447	Che6
SCE1572_9923	MXAN_6965	9.00E-11	57	Che6
SCE1572_4071	MXAN_6965	4.00E-12	58.5	Che6
SCE1572_6853	MXAN_6965	2.00E-11	58.9	Che6
SCE1572_3987	MXAN_6965	5.00E-14	65.1	Che6
SCE1572_7522	MXAN_6965	1.00E-15	71.6	Che6
SCE1572_198	MXAN_6965	4.00E-16	74.7	Che6
SCE1572_3497	MXAN_6965	4.00E-46	171	Che6

* Blastp analysis against experiment identified Che systems in *Myxococcus xanthus* DK1622. E-value cutoff 1e-5.

Supplementary Table S14. NRPS/PKS statistics in *Sorangium cellulosum* So0157-2.

ID	Type	Function	Modification domain	Number of modules	TE domian	Construction
1	NRPS	Unknown	no	12	yes	linear
2	NRPS-PKShybrid	Epithilones	Glycosylation	10<br>（1 NRPS module）	yes	linear
3	PKS	Unknown	no	1	no	Nonmodular PKS
4	NRPS-PKShybrid	Unknown	no	6	yes	linear
5	PKS	Unknown	no	5	yes	linear
6	NRPS	Unknown	no	4	yes	linear
7	NRPS-PKShybrid	Unknown	Methylation	15	yes	linear
8	NRPS	Unknown	no	2	yes	linear or iterative
9	PKS	Unknown	no	2	no	Type II PKS or nonmodular PKS
10	PKS	Unknown	Methylation	12，absence of AT domain	yes	linear
11	NRPS	Unknown	no	1	no	Unknown
12	NRPS-PKShybrid	Unknown	Epimerization	9	yes	linear
13	NRPS	Unknown	Methylation	3	yes	linear or iterative

**14**	**NRPS-PKShybrid**	**Unknown**	**Epimerization**	**2**	**no**	**Unknown**
**15**	**PKS**	**Unknown**	**no**	**5**	**yes**	**iterative or linear**
**16**	**NRPS-PKShybrid**	**Unknown**	**Epimerization**	**2**	**no**	**Unknown**
**17**	**NRPS-PKShybrid**	**Unknown**	**no**	**3**	**yes**	**iterative or linear**
**18**	**PKS**	**Unknown**	**no**	**1**	**no**	**Unknown**

Supplementary Table S15. BLAST analysis of the electron transfer chain sequences against the *Myxococcus xanthus* DK1622 database.

Gene Symbol	exist or not in 0157-2	E-value*	Hits		
succinate dehydrogenase cytochrome b558 subunit	+	6.00E-20		SCE1572_10673	
NADH dehydrogenase I, N subunit	+	8.00E-95	12	SCE1572_5698	SCE1572_9714
NADH dehydrogenase I, M subunit	+	e-121	13	SCE1572_5699	SCE1572_9713
NADH dehydrogenase I, L subunit	+	e-138	15	SCE1572_5699	SCE1572_9716
NADH dehydrogenase I, K subunit	+	8.00E-24	2	SCE1572_5700	SCE1572_10173
NADH dehydrogenase I, J subunit	+	4.00E-26	2	SCE1572_5701	SCE1572_10174
NADH dehydrogenase I, F subunit	+	e-105		SCE1572_5702	
NADH dehydrogenase I, E subunit	+	1.00E-28		SCE1572_5703	
putative NADH dehydrogenase	+	e-148	3	SCE1572_4082	
NADH dehydrogenase I, I subunit	+	4.00E-43	3	SCE1572_800	
putative NADH dehydrogenase I, H subunit	+	1.00E-73	2	SCE1572_799	SCE1572_9661
putative NADH dehydrogenase I, G subunit	+			SCE1572_798	
NADH dehydrogenase I, D subunit	+	e-127	2	SCE1572_797	SCE1572_11340
NADH dehydrogenase I, C subunit	+	6.00E-30	2	SCE1572_796	SCE1572_11339
sulfatase domain protein	+	5.00E-17	7	SCE1572_4838	
NADH dehydrogenase I, B subunit	+	4.00E-60	2	SCE1572_795	SCE1572_11337
putative NADH dehydrogenase I, A subunit	+	5.00E-29	2	SCE1572_794	SCE1572_11335
succinate dehydrogenase, flavoprotein subunit	+	0	3	SCE1572_10674	
succinate dehydrogenase, iron-sulfur protein	+	2.00E-82	2	SCE1572_10675	SCE1572_8615
putative cytochrome c oxidase, subunit IV	+	2.00E-11	2	SCE1572_6126	SCE1572_9693
cytochrome c oxidase, subunit III	+	1.00E-51	2	SCE1572_6125	SCE1572_9692
cytochrome c oxidase, subunit I	+	0	2	SCE1572_6124	SCE1572_9691
cytochrome c oxidase, subunit II	+	4.00E-93	2	SCE1572_6123	SCE1572_9690
cytochrome c oxidase accessory protein FixG	-				
cytochrome c oxidase, cbb3-type, subunit III	-				
cytochrome c oxidase, cbb3-type, CcoQ subunit	-				
cytochrome c oxidase, cbb3-type, subunit I/II	+	8.00E-09	3	SCE1572_1868	

putative cytochrome oxidase maturation protein, cbb3-type	-				
cytochrome c oxidase, subunit II	+	2.00E-83	2	SCE1572_6123	SCE1572_9690
cytochrome c oxidase, subunit I	+	0	2	SCE1572_6124	SCE1572_9691
cytochrome c oxidase, subunit III	+	2.00E-52	3	SCE1572_6125	SCE1572_9692
putative cytochrome c oxidase, subunit IV	+	7.00E-12	3	SCE1572_6126	SCE1572_9693
cytochrome d ubiquinol oxidase, subunit II	+	e-111		SCE1572_3243	
cytochrome d ubiquinol oxidase, subunit I	+	e-161		SCE1572_3240	

*Cutoff of Blast is 0.0001.

Additiional Dataset 1 Supplementary table S4 (separate file)
Transcripts detected under different pH conditions.

Additiional Dataset 2 Supplementary table S7 (separate file)
Homologous genes in So0157-2 and So ce56.

Additiional Dataset 3 Supplementary table S8 (separate file)
A comparison of KEGG categories for So0157-2 and So ce56.

Additiional Dataset 4 Supplementary table S9 (separate file)
A comparison of sequence similarity for So0157-2 and So ce56.

Additiional Dataset 5 Supplementary table S11 (separate file)
All annotated polysaccharide-degrading enzymes and related CBM motifs in So0157-2.

TableS4 Transcripts detected under different pH conditions (FPKM)

Locus tag	pH 7.0	pH 10.0
SCE1572_5566	71.77	105.81
SCE1572_4403	38.14	132.09
SCE1572_3237	0	0
SCE1572_7422	9.94	0
SCE1572_6423	81.04	28.07
SCE1572_5465	26.48	9.02
SCE1572_5888	23.63	336.49
SCE1572_1430	30.11	7.76
SCE1572_9058	43.32	17.38
SCE1572_8947	36.41	54.77
SCE1572_1415	12.9	3.11
SCE1572_9953	71.59	42.4
SCE1572_8357	29.97	4.24
SCE1572_5736	79.25	843.19
SCE1572_963	269.73	219.45
SCE1572_9431	35.04	34.06
SCE1572_6979	24.38	11
SCE1572_5559	0	0
SCE1572_1203	104.74	41.78
SCE1572_926	57.15	29.56
SCE1572_6927	39.78	63.33
SCE1572_4079	45.01	0
SCE1572_1922	37.01	17.24
SCE1572_10118	78.33	331.47
SCE1572_8912	22.01	0
SCE1572_7208	19.85	5.79
SCE1572_6891	17.94	11.14
SCE1572_10120	119.57	514.25
SCE1572_8105	0	0
SCE1572_5207	54.29	184.85
SCE1572_4631	36.49	16.55
SCE1572_211	42.7	19.92
SCE1572_9167	79.6	4.56
SCE1572_3914	75.5	22.34
SCE1572_7224	84.4	141.1
SCE1572_8249	32.33	48.34
SCE1572_8904	10.14	0.77
SCE1572_5794	118.68	247.38
SCE1572_3433	17.2	117.65
SCE1572_4684	100.06	99.12
SCE1572_9858	4.65	0
SCE1572_9695	0	0
SCE1572_1899	29	9.52
SCE1572_1005	80.17	22.87
SCE1572_7497	0	0
SCE1572_5550	27.01	7.36

SCE1572_10517	42.01	20.52
SCE1572_5396	41.45	20.5
SCE1572_11501	12.43	0
SCE1572_3334	0	0
SCE1572_10265	39.08	11.29
SCE1572_11111	89.02	52.73
SCE1572_6453	0	0
SCE1572_816	84.42	27.99
SCE1572_2624	73.08	29.22
SCE1572_6657	45.53	106.94
SCE1572_1590	32	30.03
SCE1572_4855	38.4	9.48
SCE1572_8348	109.01	92.49
SCE1572_4967	22.74	188.85
SCE1572_7617	140.23	738.5
SCE1572_8843	53.38	31.44
SCE1572_2650	18.43	12.67
SCE1572_1513	44.99	4.45
SCE1572_10034	54.06	37.85
SCE1572_9206	78.58	54.16
SCE1572_1573	33.65	20.93
SCE1572_9449	65.11	39.45
SCE1572_3515	12.87	47.05
SCE1572_7608	3.45	0
SCE1572_6365	39.46	439.67
SCE1572_7454	41.35	77.75
SCE1572_3975	14.44	17.38
SCE1572_3071	74.22	43.87
SCE1572_3042	16.92	3.92
SCE1572_5691	18.81	7.7
SCE1572_1404	86.89	15.87
SCE1572_49	43.2	7.85
SCE1572_8002	67.23	15.37
SCE1572_2278	42.7	283
SCE1572_4137	0	0
SCE1572_1345	63.88	465.15
SCE1572_8495	19.53	10.07
SCE1572_5018	89.68	125.86
SCE1572_830	17.14	19.04
SCE1572_187	45.85	2032.88
SCE1572_6610	12.57	14.24
SCE1572_4114	32.99	47.3
SCE1572_306	19.03	9.6
SCE1572_7613	35.93	397.78
SCE1572_1606	61.55	17.96
SCE1572_10395	22.97	3.69
SCE1572_9894	8.42	0
SCE1572_9491	0	0

SCE1572_11230	157.52	170.44
SCE1572_8955	2.71	0
SCE1572_6436	47.38	21.29
SCE1572_5448	50.73	20.81
SCE1572_3105	47	68.9
SCE1572_1366	64.08	175.7
SCE1572_8601	47.77	149.97
SCE1572_4069	0	0
SCE1572_5880	0	0
SCE1572_8037	0	0
SCE1572_6726	23.23	23.29
SCE1572_3543	0	0
SCE1572_7367	124.16	3591.04
SCE1572_248	95.24	16.45
SCE1572_10623	20.92	24.32
SCE1572_9768	22.51	135.47
SCE1572_10652	15.57	87.97
SCE1572_7802	33.59	221.2
SCE1572_5985	14.85	4.7
SCE1572_9841	0	0
SCE1572_9963	92.7	27.57
SCE1572_7577	17.3	4.9
SCE1572_10612	66.3	105.45
SCE1572_7663	15.74	3.99
SCE1572_11224	66.43	42.03
SCE1572_9408	41.42	32.07
SCE1572_4376	32.88	8.79
SCE1572_9362	53.14	326.55
SCE1572_5731	11.78	205.49
SCE1572_10286	172.62	1300.13
SCE1572_7845	129.01	389.1
SCE1572_4842	19.59	147.61
SCE1572_8798	77.18	17.13
SCE1572_7521	54.58	29.19
SCE1572_2463	82.27	22.93
SCE1572_7211	41.03	33.49
SCE1572_4203	61.18	299.62
SCE1572_782	17.84	17.89
SCE1572_5176	23.13	17.52
SCE1572_1742	31.71	31.73
SCE1572_11206	25.38	28.88
SCE1572_9567	2.11	0
SCE1572_2193	26.87	4.62
SCE1572_3990	35.57	62.58
SCE1572_2460	20.67	0
SCE1572_5137	8.27	11.06
SCE1572_4098	8.85	5.33
SCE1572_282	11.52	4.62

SCE1572_88	56.13	117.43
SCE1572_8438	18.01	12.58
SCE1572_6296	39.48	194.17
SCE1572_1380	112.48	28.3
SCE1572_2438	20.55	74.74
SCE1572_2504	25.79	19.86
SCE1572_6900	13.38	16.1
SCE1572_2791	117.92	226.39
SCE1572_80	79.94	213.65
SCE1572_1472	63.67	47.4
SCE1572_9104	52.91	23.91
SCE1572_1624	45.84	26.87
SCE1572_3362	41.83	52.43
SCE1572_2168	2.37	0
SCE1572_7856	34.27	13.68
SCE1572_4773	21.73	18.91
SCE1572_754	42.67	56.79
SCE1572_6951	26.93	13.12
SCE1572_10444	48.49	101.42
SCE1572_9667	49.17	280.38
SCE1572_10984	82.35	570.29
SCE1572_8110	5.14	0
SCE1572_5079	33.47	80.57
SCE1572_8041	34.11	9.12
SCE1572_2242	36.16	30.95
SCE1572_1372	62.57	628.48
SCE1572_8433	6.83	6.58
SCE1572_5813	35.56	35.4
SCE1572_700	2.92	0
SCE1572_2010	59.77	15.79
SCE1572_11149	7.64	15.33
SCE1572_10976	250.29	0
SCE1572_7217	40.64	50.96
SCE1572_5719	26.64	11.51
SCE1572_11292	86.24	628.48
SCE1572_11053	73.26	228.87
SCE1572_6650	27.16	27.23
SCE1572_8277	40.65	29.5
SCE1572_9473	56.5	125.8
SCE1572_3550	24.25	15.36
SCE1572_5232	30.32	70.9
SCE1572_10529	19.96	29.64
SCE1572_9606	2.14	0
SCE1572_7979	101.62	30.08
SCE1572_8021	50.54	20.7
SCE1572_7030	32.49	14.48
SCE1572_4972	36.82	195.49
SCE1572_7997	43.36	4.15

SCE1572_3219	116.05	1470.95
SCE1572_180	0	0
SCE1572_1340	28.54	30.05
SCE1572_9934	0	0
SCE1572_7895	2.92	0
SCE1572_7063	28.91	12.24
SCE1572_1569	31.07	58.03
SCE1572_8410	97.8	696.42
SCE1572_1140	11.72	16.38
SCE1572_11212	20.49	13.66
SCE1572_5996	89.56	64.5
SCE1572_4989	26.47	52.13
SCE1572_8108	53.61	13.88
SCE1572_2995	29.03	8.59
SCE1572_9626	36.16	10.38
SCE1572_525	70.68	101.52
SCE1572_8183	42.61	5.54
SCE1572_4091	39.43	18.08
SCE1572_5859	25.4	15.28
SCE1572_8465	6.79	0
SCE1572_1858	37.1	7.76
SCE1572_745	99.37	421.31
SCE1572_739	15.46	6.8
SCE1572_3507	27.49	16.04
SCE1572_6096	41.19	22.1
SCE1572_4152	23.33	1306.86
SCE1572_1557	7.46	0
SCE1572_1102	30.1	17.13
SCE1572_8406	37.9	28.88
SCE1572_10274	48.95	71.94
SCE1572_5523	29.46	14.6
SCE1572_1752	13.64	5.05
SCE1572_7397	15.75	9.2
SCE1572_6604	62.25	148.34
SCE1572_520	43.45	20.03
SCE1572_3861	108.86	369
SCE1572_5452	36.88	10.65
SCE1572_1479	45.06	30.41
SCE1572_4183	19.53	13.82
SCE1572_4277	56.44	26.77
SCE1572_4055	20.71	64.08
SCE1572_7277	0	0
SCE1572_273	124.1	360.6
SCE1572_4726	21.74	11.66
SCE1572_4367	61.14	30.31
SCE1572_10239	71.88	17.9
SCE1572_5862	40.9	13.75
SCE1572_5269	57.96	271.71

SCE1572_5003	35.13	211.39
SCE1572_8270	29.15	19.03
SCE1572_5742	84.48	67.08
SCE1572_5688	43.32	21.24
SCE1572_6380	63.75	45.62
SCE1572_4251	11.58	12.67
SCE1572_7555	9.69	2.74
SCE1572_4451	0	0
SCE1572_3700	39.48	35.73
SCE1572_11134	50.1	25.39
SCE1572_5330	32.53	11.36
SCE1572_11	26.06	50.18
SCE1572_6894	25.03	18.63
SCE1572_2819	41.66	19.24
SCE1572_10713	22.83	19.78
SCE1572_10510	20.24	13.81
SCE1572_1136	0	0
SCE1572_1154	12.82	0
SCE1572_9848	23.75	4.4
SCE1572_588	7.28	7.67
SCE1572_2092	54.64	9.26
SCE1572_9341	71.44	694.4
SCE1572_6101	63.96	22.81
SCE1572_4552	40.93	14.07
SCE1572_2039	14.88	69.4
SCE1572_1691	133.09	318.29
SCE1572_9088	11.09	0
SCE1572_10912	32.65	79.69
SCE1572_3654	46	250.12
SCE1572_839	10.77	34.55
SCE1572_3022	35.28	9.18
SCE1572_5237	106.32	174.67
SCE1572_1358	219.3	1779.1
SCE1572_6327	13.04	3.92
SCE1572_10525	28.18	29.61
SCE1572_3380	17.28	20.8
SCE1572_10676	83.55	69.26
SCE1572_4713	24.9	12.93
SCE1572_3785	183.78	453.39
SCE1572_5601	52.44	20.85
SCE1572_973	46.65	104.8
SCE1572_8005	24.05	33.92
SCE1572_10203	33.13	104.59
SCE1572_6265	67.61	118.35
SCE1572_8051	48.73	16.29
SCE1572_6388	18.51	104.71
SCE1572_1686	18.54	9.19
SCE1572_5026	28.43	22.81

SCE1572_5838	17.47	6.78
SCE1572_10160	63.73	102.42
SCE1572_405	36.17	39.64
SCE1572_6510	0	0
SCE1572_4060	16.62	79.98
SCE1572_1251	44.4	20.39
SCE1572_8928	0	0
SCE1572_10836	37.16	19.08
SCE1572_8643	41.94	48.14
SCE1572_7375	12	7.88
SCE1572_335	47.29	23.46
SCE1572_5510	32.6	34.87
SCE1572_4130	107.97	175.55
SCE1572_4475	1210.73	2314.91
SCE1572_9498	291.66	1885.9
SCE1572_2046	13.94	0
SCE1572_11302	26.91	36.91
SCE1572_5889	34.46	71.88
SCE1572_8859	24.96	17.93
SCE1572_5112	19.4	36.32
SCE1572_4954	21.66	0
SCE1572_6232	72.26	19.51
SCE1572_295	8.15	262.9
SCE1572_1892	20.75	21.17
SCE1572_5495	25.1	8.99
SCE1572_5462	30.64	13.41
SCE1572_9142	15.81	6.06
SCE1572_8918	28.79	424.97
SCE1572_3926	58.34	284.55
SCE1572_364	10.63	0.85
SCE1572_3150	18.77	66.43
SCE1572_995	51.58	9.41
SCE1572_8751	38.33	11.99
SCE1572_1718	30.32	46.25
SCE1572_4925	37.45	76.99
SCE1572_4641	37.84	19.11
SCE1572_11128	0	0
SCE1572_1355	14.9	20.62
SCE1572_6588	41.73	41.22
SCE1572_6193	0	0
SCE1572_4588	45.69	391.58
SCE1572_2258	20.9	9.29
SCE1572_2030	58.45	13.19
SCE1572_6967	55.26	34.91
SCE1572_9748	19.58	14.14
SCE1572_6884	46.39	18.98
SCE1572_3659	21.16	25.46
SCE1572_1935	0	0

SCE1572_5064	56.46	89.73
SCE1572_1118	30.44	15.2
SCE1572_11174	55.81	34.93
SCE1572_8966	98.21	138.88
SCE1572_2390	44.31	113.11
SCE1572_4929	34.02	71.84
SCE1572_10200	22.62	50.4
SCE1572_6628	32.34	83.88
SCE1572_4486	43.32	345.37
SCE1572_8117	35.58	11.17
SCE1572_8212	5.53	10.65
SCE1572_8911	14.46	12.35
SCE1572_3389	22.59	12.88
SCE1572_886	1003.33	1367.59
SCE1572_9295	41.49	84.75
SCE1572_8415	33.96	17.92
SCE1572_3294	10.87	4.13
SCE1572_146	44.38	19.28
SCE1572_4930	41.35	48.49
SCE1572_7638	18.36	22.61
SCE1572_7202	26.63	2.62
SCE1572_3557	43.32	15.99
SCE1572_8921	1.26	0
SCE1572_8562	134.36	290.75
SCE1572_6723	26.73	91.54
SCE1572_5457	39.21	12.53
SCE1572_4784	11.03	0
SCE1572_4677	6.93	12.51
SCE1572_1188	52.45	13.84
SCE1572_5655	39	26.44
SCE1572_7814	673.08	5319.02
SCE1572_11271	28.66	90.09
SCE1572_1595	40.99	45.42
SCE1572_474	216.19	1006.92
SCE1572_5185	59.12	2406.04
SCE1572_3746	21.25	10.23
SCE1572_4708	0	0
SCE1572_7138	47.12	85.07
SCE1572_5293	24.44	41.44
SCE1572_4193	532.45	2482.71
SCE1572_3754	22.8	2.74
SCE1572_3251	10.83	0
SCE1572_11044	28.64	37.84
SCE1572_6377	23.94	15.37
SCE1572_7353	61.91	16.61
SCE1572_11193	30.95	16.73
SCE1572_11325	90.2	92.08
SCE1572_6854	64.3	3.82

SCE1572_2692	25.57	68.15
SCE1572_6470	31.73	12.73
SCE1572_2699	2.17	0
SCE1572_9192	41.56	24.14
SCE1572_9930	18.84	35.02
SCE1572_4230	10.93	13.15
SCE1572_7287	26.68	40.87
SCE1572_8364	57.94	31.77
SCE1572_2466	17.96	6.05
SCE1572_5680	127.03	254.29
SCE1572_8608	38.99	146.79
SCE1572_6164	38.73	25.78
SCE1572_2371	57.49	91.23
SCE1572_6829	4.53	0
SCE1572_6398	23.82	89.3
SCE1572_7344	0	0
SCE1572_2395	21.74	79.87
SCE1572_2310	54.12	197.07
SCE1572_1423	121.95	79.94
SCE1572_6673	23.42	19.28
SCE1572_6462	57.12	58.11
SCE1572_11167	53.91	198.68
SCE1572_11454	55.05	109.88
SCE1572_8786	15.88	157.62
SCE1572_6986	0	0
SCE1572_3877	0	0
SCE1572_6944	3.26	0
SCE1572_7420	74.59	52.69
SCE1572_9276	82.81	72.21
SCE1572_2560	10.72	14.74
SCE1572_7449	195.08	765.83
SCE1572_10805	65.96	20.95
SCE1572_9214	35.88	20.3
SCE1572_76	15.27	0
SCE1572_10657	55.29	49.9
SCE1572_9705	48.48	19.86
SCE1572_1218	84.78	26.99
SCE1572_6972	44.82	18.25
SCE1572_2301	8.99	58.76
SCE1572_10659	22.25	26.49
SCE1572_5475	26.4	90.78
SCE1572_3148	26.48	8.73
SCE1572_7037	23.19	51.52
SCE1572_2635	37.56	83.79
SCE1572_9712	0	0
SCE1572_327	3.23	0
SCE1572_10834	44.58	10.65
SCE1572_1000	76.56	146.11

SCE1572_8502	40.87	14.88
SCE1572_2544	35.87	15.24
SCE1572_7769	9.03	8.15
SCE1572_4048	29	72
SCE1572_10216	78.25	35.32
SCE1572_9534	22.24	16.53
SCE1572_2376	40.25	67.54
SCE1572_11510	49.59	25.58
SCE1572_847	287.59	1460.46
SCE1572_6631	2.08	0
SCE1572_8509	95.8	47.64
SCE1572_7626	50.54	20.56
SCE1572_4490	28.64	14.26
SCE1572_581	35.67	37.33
SCE1572_485	25.87	10.38
SCE1572_1261	41.57	57.68
SCE1572_4297	0	0
SCE1572_6486	6.51	0
SCE1572_2598	0	0
SCE1572_1028	2.41	0
SCE1572_122	23.11	7.79
SCE1572_5213	28.05	14.72
SCE1572_387	40.72	104.05
SCE1572_3452	53.09	16.53
SCE1572_4140	19.39	45.62
SCE1572_5841	39.82	16.96
SCE1572_132	174	312.9
SCE1572_10225	8.66	0
SCE1572_6014	15.87	665.29
SCE1572_6912	42.45	8.59
SCE1572_5620	69.23	19.97
SCE1572_11342	21.6	26.96
SCE1572_10406	50.35	62.3
SCE1572_6694	56.98	24.41
SCE1572_7840	25.64	30.86
SCE1572_10968	31.31	26.59
SCE1572_10605	0	0
SCE1572_9668	67.94	405.8
SCE1572_9334	26.98	87.12
SCE1572_9444	29.32	22.18
SCE1572_422	21.09	58.12
SCE1572_576	45.21	189.29
SCE1572_8155	30.32	5.92
SCE1572_3533	65.51	20.27
SCE1572_2657	204.37	66.99
SCE1572_7433	40.92	82.35
SCE1572_5916	194.93	144.55
SCE1572_6281	58.22	126.79

SCE1572_11368	7.22	0
SCE1572_9369	0	0
SCE1572_7400	58.94	57.39
SCE1572_7108	0	0
SCE1572_5275	3.61	4.34
SCE1572_10825	159.45	219.3
SCE1572_6075	63.01	59.55
SCE1572_2478	58.89	64.52
SCE1572_6270	19.56	19.13
SCE1572_3199	75.81	478.68
SCE1572_9190	45.78	24.33
SCE1572_6401	0	0
SCE1572_4153	0	0
SCE1572_4285	32.58	31.06
SCE1572_3442	36.62	36.49
SCE1572_4159	13.12	21.47
SCE1572_9908	33.8	33.26
SCE1572_7128	59.75	36.44
SCE1572_10661	38.52	58.15
SCE1572_4343	103.37	35.73
SCE1572_3613	54.65	135.79
SCE1572_2215	20.02	34.8
SCE1572_2433	15.62	18.11
SCE1572_5403	45.27	17.98
SCE1572_1865	23.26	9.33
SCE1572_9881	52.93	53.98
SCE1572_941	26.27	3.33
SCE1572_6312	20.3	280.64
SCE1572_7884	48.56	28.51
SCE1572_11309	14.38	2.16
SCE1572_3284	12.58	1.68
SCE1572_4383	55.59	22.71
SCE1572_9118	89.79	56.94
SCE1572_5972	34.53	0
SCE1572_5898	10.11	6.95
SCE1572_5586	20.07	0
SCE1572_5391	130.47	112.95
SCE1572_4579	3.3	0
SCE1572_7151	63.58	65.59
SCE1572_1458	0	0
SCE1572_3670	23.41	8.39
SCE1572_11379	19.24	44.46
SCE1572_6415	28.25	10
SCE1572_4236	42.96	166.24
SCE1572_10117	13.93	21.52
SCE1572_10591	46.88	31.29
SCE1572_9516	24.12	111.96
SCE1572_98	41.51	26.07

SCE1572_1098	27.92	17.3
SCE1572_3177	33.17	142.26
SCE1572_1123	28.22	8.69
SCE1572_10692	14.97	64.21
SCE1572_10577	33.93	25.52
SCE1572_4083	18.77	27.8
SCE1572_2595	44.17	32.64
SCE1572_7586	30.1	598.37
SCE1572_2893	18.73	7.34
SCE1572_6222	27.99	11.23
SCE1572_4600	23.65	39.41
SCE1572_643	129.82	307.79
SCE1572_2336	18.29	23.21
SCE1572_8892	99.58	119.06
SCE1572_7056	14.02	14.77
SCE1572_3741	94.98	73.4
SCE1572_9871	11.72	5.81
SCE1572_5596	77.64	76.69
SCE1572_5514	128.49	24.74
SCE1572_5978	30.99	19.49
SCE1572_2864	0	0
SCE1572_2177	31.95	4.71
SCE1572_4654	27.71	42.09
SCE1572_9307	46.48	141.17
SCE1572_6432	20.43	6.47
SCE1572_7483	73.13	49.76
SCE1572_4469	63.22	25.98
SCE1572_8401	67.06	46.01
SCE1572_4848	12.69	9.08
SCE1572_6871	23.19	20.42
SCE1572_1739	33.09	5.79
SCE1572_2453	44.13	39.37
SCE1572_9263	17.66	35.43
SCE1572_4939	54.96	94.59
SCE1572_8763	0	0
SCE1572_3449	38.71	13.2
SCE1572_10729	22.01	38.26
SCE1572_2535	24.07	26.07
SCE1572_9795	47	3.65
SCE1572_5717	134.38	242.87
SCE1572_10197	0	0
SCE1572_1190	65.38	79.59
SCE1572_10580	24.92	27.49
SCE1572_10640	136.2	840.61
SCE1572_4935	69.98	314.39
SCE1572_10865	173.05	437.04
SCE1572_5438	0	0
SCE1572_5141	34.79	22.12

SCE1572_8764	0.04	0.12
SCE1572_7461	381.31	1249.12
SCE1572_9651	45.44	14.31
SCE1572_289	124.94	20.32
SCE1572_4033	0	0
SCE1572_2921	92.95	22.97
SCE1572_9552	15.67	7.14
SCE1572_9434	174.82	677.9
SCE1572_2977	45.05	15.5
SCE1572_10464	14.02	53.74
SCE1572_7671	124.92	7.71
SCE1572_10650	1383.5	2642.43
SCE1572_6239	0	0
SCE1572_9900	126.25	428.14
SCE1572_9241	0	0
SCE1572_8166	8.12	0
SCE1572_3264	24.35	217
SCE1572_10436	43.8	63.52
SCE1572_213	60.56	24.83
SCE1572_4017	17.84	7.16
SCE1572_5956	51.49	34.43
SCE1572_10495	19.87	45.62
SCE1572_8129	0	0
SCE1572_8633	43.26	54.74
SCE1572_5723	3.47	0
SCE1572_6662	51.35	47.22
SCE1572_1224	29.24	30.27
SCE1572_9736	71.71	13.15
SCE1572_7762	38.07	138.25
SCE1572_8826	63.13	15.78
SCE1572_3128	37.32	67.37
SCE1572_7086	43.2	14.27
SCE1572_3479	25.27	0
SCE1572_5942	27.3	9.57
SCE1572_224	35.88	26.46
SCE1572_4323	20.91	9.27
SCE1572_1220	40.9	22.07
SCE1572_1227	44.79	16.81
SCE1572_8933	13.09	10.5
SCE1572_2813	39.47	47.5
SCE1572_7724	56.01	87.18
SCE1572_11578	10.74	4.31
SCE1572_10845	57.74	30.76
SCE1572_1377	19.01	7.81
SCE1572_11406	32.43	10.55
SCE1572_5097	21.2	24.4
SCE1572_4441	19.46	9.09
SCE1572_2801	58.28	553.27

SCE1572_10786	22.11	13.94
SCE1572_10241	17.59	9.15
SCE1572_9923	32.19	105.48
SCE1572_5	64.46	221.03
SCE1572_25	45.68	54.97
SCE1572_2025	37.18	83.34
SCE1572_567	22.98	27.23
SCE1572_3812	9.3	33.58
SCE1572_490	94.96	255.5
SCE1572_4841	41.03	291.31
SCE1572_7331	2.53	0
SCE1572_9992	0	0
SCE1572_9282	901.24	946.41
SCE1572_469	89.44	861.11
SCE1572_2613	56.71	27.86
SCE1572_460	4.44	0
SCE1572_2075	46.96	8.07
SCE1572_5525	37.42	17.58
SCE1572_4430	6.71	4.31
SCE1572_373	27.84	25.13
SCE1572_9389	36.18	166.7
SCE1572_3631	18.33	12.03
SCE1572_6881	69.27	39.32
SCE1572_5253	0	0
SCE1572_6575	18.77	17.38
SCE1572_3030	28.03	9.95
SCE1572_2760	102.04	582.84
SCE1572_10053	19.27	60.48
SCE1572_9017	0	0
SCE1572_824	28.06	16.99
SCE1572_3986	95.51	1422.86
SCE1572_8613	32.22	13.99
SCE1572_4293	30.66	22.08
SCE1572_4008	42.33	16.67
SCE1572_11548	84.75	327.49
SCE1572_1170	55.88	24.25
SCE1572_7849	83.15	70.41
SCE1572_8997	22.7	16.39
SCE1572_516	0	0
SCE1572_8182	45.75	12.96
SCE1572_5534	46.28	15.91
SCE1572_7759	32.2	175.2
SCE1572_241	63.31	43.01
SCE1572_8379	19.44	16.37
SCE1572_9703	20.01	23.36
SCE1572_3937	89.59	73.46
SCE1572_1898	17.47	21.78
SCE1572_8028	5.62	0

SCE1572_1564	20.64	35.98
SCE1572_5419	13.78	46.9
SCE1572_8802	93.43	67.79
SCE1572_4763	28.46	138.05
SCE1572_9370	10.83	6.95
SCE1572_6322	46.01	47.2
SCE1572_8818	41.6	21.08
SCE1572_8073	39.23	18.08
SCE1572_773	13.11	16.83
SCE1572_6206	41.98	31.54
SCE1572_5345	18.01	10.84
SCE1572_2267	33.97	56.14
SCE1572_7927	46.3	46.3
SCE1572_78	78.78	27.43
SCE1572_5801	28.36	87.05
SCE1572_6229	23.63	10.43
SCE1572_6481	16.37	1.71
SCE1572_4456	28.64	61.83
SCE1572_2928	26.73	10.02
SCE1572_8205	12.13	38.93
SCE1572_7876	34.31	27.97
SCE1572_8485	17.37	10.33
SCE1572_6370	35.29	23.89
SCE1572_2723	34.43	42.16
SCE1572_2517	0	0
SCE1572_8536	25.94	12.41
SCE1572_6642	89.14	40.1
SCE1572_4965	29.14	19.46
SCE1572_11461	3.09	0
SCE1572_7444	0	0
SCE1572_8708	21.87	35.89
SCE1572_9950	46.48	21.09
SCE1572_4168	24.66	4.4
SCE1572_9545	43.37	55.43
SCE1572_6509	38.16	12.41
SCE1572_6593	31.59	79.07
SCE1572_987	17.55	15.28
SCE1572_8569	93.22	18.92
SCE1572_7693	148.98	502.43
SCE1572_10000	98.35	456.97
SCE1572_2770	56.86	623.41
SCE1572_1249	11.03	9.95
SCE1572_4439	14.74	5.52
SCE1572_3673	42.73	49.1
SCE1572_5156	95.13	17.89
SCE1572_59	25.87	16.39
SCE1572_876	348.72	235.8
SCE1572_4609	278.18	53.95

SCE1572_10011	354.36	1321.08
SCE1572_5369	15.58	4.85
SCE1572_1917	16.1	5.42
SCE1572_7121	0	0
SCE1572_42	46.95	39.83
SCE1572_6958	56.86	12.67
SCE1572_5592	46.87	37.35
SCE1572_1284	89.6	2903.67
SCE1572_10007	15.92	10.45
SCE1572_8972	29.08	3467.6
SCE1572_8899	4.74	0
SCE1572_6358	39.74	15.59
SCE1572_9234	63.5	98.07
SCE1572_4819	56	249.22
SCE1572_5354	19.16	27.67
SCE1572_10416	6.64	8.72
SCE1572_1169	42.32	19.25
SCE1572_5788	198.75	820.05
SCE1572_120	28.53	5.39
SCE1572_1282	19.69	72.67
SCE1572_8236	64.08	579.97
SCE1572_11492	58.11	159.31
SCE1572_8269	32.79	11.36
SCE1572_5201	10.83	8.41
SCE1572_799	49.6	231.27
SCE1572_2826	18.02	13.46
SCE1572_3346	196.12	142.52
SCE1572_9481	58.04	50.61
SCE1572_2713	36.97	92.84
SCE1572_7036	59.56	189.91
SCE1572_3277	0	0
SCE1572_10917	12.86	17.99
SCE1572_6636	108.15	29.77
SCE1572_3948	78.84	189.76
SCE1572_7775	38.99	13.9
SCE1572_10398	36.54	39.98
SCE1572_2997	18.78	3.23
SCE1572_7476	488.25	0
SCE1572_4514	143.38	425.74
SCE1572_7348	38.11	15.61
SCE1572_2571	47.44	49.65
SCE1572_6502	26.35	24.72
SCE1572_4056	25.9	61.32
SCE1572_1323	12.31	20.8
SCE1572_3575	45.42	15.51
SCE1572_10575	0	0
SCE1572_4412	2.94	0
SCE1572_7650	27.57	0

SCE1572_5117	14.2	34.17
SCE1572_9310	102.87	453.42
SCE1572_4918	43.72	45.09
SCE1572_1957	45.22	33.29
SCE1572_4696	59.18	36.31
SCE1572_1138	26.34	7.73
SCE1572_10372	60.93	23.87
SCE1572_6408	24.33	11.26
SCE1572_2206	58.48	504.37
SCE1572_10148	47.79	59.5
SCE1572_9399	45.85	37.62
SCE1572_4914	24.75	9.58
SCE1572_11408	21.41	12.37
SCE1572_9189	53.19	20.6
SCE1572_8985	34.29	73.35
SCE1572_6768	93.98	153.81
SCE1572_8635	24.5	16.59
SCE1572_6615	46.72	27.54
SCE1572_5614	27.57	7.04
SCE1572_2122	3.97	1.59
SCE1572_3903	69.48	104.36
SCE1572_1970	44.63	18.76
SCE1572_1791	20.31	4.07
SCE1572_11444	27.47	26.44
SCE1572_3957	6.5	0
SCE1572_889	29.09	8.67
SCE1572_10709	20.37	0
SCE1572_4321	103.9	52.89
SCE1572_8395	23.78	530.39
SCE1572_4561	0	0
SCE1572_4024	229.96	201.85
SCE1572_3510	22.55	114.6
SCE1572_2330	86.07	58.92
SCE1572_1822	99.69	22.5
SCE1572_10500	49.67	576.75
SCE1572_6994	11.89	14.31
SCE1572_3299	41.76	43.7
SCE1572_10924	75.72	1698.06
SCE1572_10744	44.5	17.6
SCE1572_3069	21.78	48.31
SCE1572_6627	48.83	80.41
SCE1572_4993	26.37	15.87
SCE1572_611	9.38	46.4
SCE1572_2103	41.41	60.36
SCE1572_11032	0	0
SCE1572_6467	34.48	8.49
SCE1572_10694	39.48	0
SCE1572_1077	55.07	36

SCE1572_6139	24.75	52.13
SCE1572_9983	36.02	15.67
SCE1572_5634	49.33	17.46
SCE1572_8870	39.85	29.77
SCE1572_10928	33.36	33.9
SCE1572_7198	366.5	871.61
SCE1572_9413	20.14	77.69
SCE1572_3589	2.76	0
SCE1572_3272	0	0
SCE1572_1995	67.77	21.43
SCE1572_6788	92.63	34.03
SCE1572_3551	10.42	8.36
SCE1572_9010	23.1	9.98
SCE1572_4827	37.7	22.2
SCE1572_9692	20.16	79.58
SCE1572_6603	23.45	38.56
SCE1572_8519	24.3	334.28
SCE1572_4359	8.74	9.01
SCE1572_10827	38.82	17.68
SCE1572_7752	20.28	14.42
SCE1572_1771	57.19	27.17
SCE1572_5192	45.69	369.92
SCE1572_2369	67.61	14.79
SCE1572_6275	51	147.35
SCE1572_2706	53.57	35.98
SCE1572_10131	19.36	19.97
SCE1572_10380	38.23	34.02
SCE1572_437	22.48	12.3
SCE1572_2935	9.41	2.52
SCE1572_7502	25.95	42.74
SCE1572_5909	87.65	647.17
SCE1572_5347	27.42	15.53
SCE1572_9752	0	0
SCE1572_2677	10.21	166.39
SCE1572_9153	245.89	208.19
SCE1572_860	134.98	854.45
SCE1572_3992	26.82	140.35
SCE1572_6526	26.58	10.18
SCE1572_8624	37.41	0
SCE1572_8497	68.21	26.2
SCE1572_2587	5.7	2.74
SCE1572_11471	25.27	18.88
SCE1572_10550	24.3	19.5
SCE1572_11459	65.38	154
SCE1572_3224	17.75	1.88
SCE1572_2417	15.25	13.63
SCE1572_10811	0	0
SCE1572_3624	35.77	8.2

SCE1572_6114	87.18	15.21
SCE1572_6566	21.35	21.15
SCE1572_4572	45.48	11.52
SCE1572_3845	77.73	24.25
SCE1572_9723	30.17	13.03
SCE1572_4549	35.03	11.47
SCE1572_1068	38.83	0
SCE1572_2855	16.39	5.44
SCE1572_10383	16.13	8.63
SCE1572_2774	33.46	23.86
SCE1572_40	51.57	33.1
SCE1572_1615	44.87	197.77
SCE1572_11434	29.43	18.13
SCE1572_7786	21.07	30.32
SCE1572_7176	51.96	102.31
SCE1572_7737	17.72	28.44
SCE1572_7491	32.34	34.06
SCE1572_6429	14.97	5.48
SCE1572_9155	67.68	116.35
SCE1572_1426	240.32	549.65
SCE1572_3309	16.62	4.8
SCE1572_4542	0	0
SCE1572_7967	22.97	0
SCE1572_5083	42.74	148.17
SCE1572_3411	20.8	34.49
SCE1572_2829	42.91	9.18
SCE1572_1785	61.06	128.64
SCE1572_1643	80.27	25.74
SCE1572_5106	73.64	1454.48
SCE1572_10944	77.29	78.71
SCE1572_3581	40.16	22.96
SCE1572_5770	270.86	4013.51
SCE1572_501	37.23	16.8
SCE1572_3819	48.22	27.26
SCE1572_9092	26.7	17.85
SCE1572_7113	35.21	23.5
SCE1572_5629	13.96	0
SCE1572_8642	6.06	4.87
SCE1572_9878	23.28	8.62
SCE1572_9437	254.1	344.65
SCE1572_6134	33.93	45.93
SCE1572_2666	35.1	51.63
SCE1572_5645	6	3.61
SCE1572_7716	19.74	0
SCE1572_539	2.6	0
SCE1572_4215	382.12	843.07
SCE1572_1827	10.23	2.9
SCE1572_982	55.28	47.43

SCE1572_6140	12.82	6.35
SCE1572_5646	2.61	0
SCE1572_11160	26.16	179.23
SCE1572_7025	2.81	0
SCE1572_4187	98.48	144.99
SCE1572_2404	40.34	299.14
SCE1572_7512	0	0
SCE1572_4799	43.01	48.71
SCE1572_10388	24.4	67.11
SCE1572_8742	27.19	28.84
SCE1572_6149	102.56	93.91
SCE1572_2468	34.64	61.72
SCE1572_862	74.89	267.1
SCE1572_5146	20.67	9.6
SCE1572_11073	3.47	181.42
SCE1572_2019	29.79	14.82
SCE1572_3084	32.11	9.66
SCE1572_8145	17.4	105.69
SCE1572_8884	42.98	3.76
SCE1572_11500	40.29	96.98
SCE1572_321	45.33	3.76
SCE1572_11313	140.73	1973.81
SCE1572_8833	21.81	11.25
SCE1572_8663	53.2	22.41
SCE1572_3045	6.11	0
SCE1572_4864	21.49	9.24
SCE1572_4484	27.14	34.08
SCE1572_2547	21.57	23.32
SCE1572_1849	45.69	27.99
SCE1572_11387	172.74	312.95
SCE1572_11553	40.98	29.59
SCE1572_4266	30.32	32.44
SCE1572_3602	12.25	30.79
SCE1572_10342	20.22	32.44
SCE1572_9526	58.75	637.47
SCE1572_8053	25.89	11.13
SCE1572_9179	46.6	18.96
SCE1572_10934	33.19	16.44
SCE1572_8734	27.57	28.75
SCE1572_8332	129.96	131.78
SCE1572_563	21.5	57.06
SCE1572_1333	12.06	0
SCE1572_11083	8.92	21.47
SCE1572_6518	43.1	9.26
SCE1572_2733	16.97	43.47
SCE1572_1312	683.04	1949.94
SCE1572_2020	26.45	38.82
SCE1572_10304	64.44	97.89

SCE1572_8446	9.72	4.68
SCE1572_4508	14.65	29.84
SCE1572_9464	24.11	13.19
SCE1572_752	20.81	0
SCE1572_8686	65.12	9.8
SCE1572_6742	24.16	5.11
SCE1572_47	20.65	7.91
SCE1572_8292	32.31	17.95
SCE1572_6316	0	0
SCE1572_2235	60.06	184.99
SCE1572_7169	45.32	55.97
SCE1572_7132	34.66	10.43
SCE1572_1526	182.79	2697.39
SCE1572_8534	59.45	255.11
SCE1572_8936	8.27	0
SCE1572_10531	2.23	0
SCE1572_2115	35.67	16.28
SCE1572_1740	19.28	3.2
SCE1572_7328	25.27	4.34
SCE1572_9071	99.07	252.2
SCE1572_4818	7.22	0
SCE1572_2697	72.94	31.35
SCE1572_2135	2.57	0
SCE1572_2683	46.09	328.92
SCE1572_1809	10.47	25.2
SCE1572_2323	23.65	82.13
SCE1572_3494	22.68	2.87
SCE1572_7260	97	1000.09
SCE1572_7377	47.5	72.45
SCE1572_8282	70.95	251.71
SCE1572_9596	15.64	792.11
SCE1572_3885	73.84	22.69
SCE1572_2068	21.66	14.89
SCE1572_2063	40.73	34.25
SCE1572_2101	63.79	240.31
SCE1572_7000	0	0
SCE1572_9688	121.9	229.91
SCE1572_4257	19.14	0
SCE1572_9028	35.7	109.26
SCE1572_11581	29.25	37.68
SCE1572_11232	22.54	12.82
SCE1572_2299	138.29	699.77
SCE1572_8339	47.9	42.79
SCE1572_8957	34.63	114.09
SCE1572_10186	41.86	95.84
SCE1572_2217	5.32	0
SCE1572_9384	14.03	9.8
SCE1572_8749	26.52	28.53

SCE1572_4612	35.5	5.34
SCE1572_3795	26.97	18.69
SCE1572_8487	7.58	3.65
SCE1572_9356	29.62	140.35
SCE1572_4354	27.3	18.18
SCE1572_10322	33.93	505.28
SCE1572_6052	317.8	431.27
SCE1572_3663	21.78	6.31
SCE1572_7624	42.76	66.35
SCE1572_4738	24.89	46.3
SCE1572_2493	83.77	50.24
SCE1572_10218	47.87	70.56
SCE1572_990	43.82	41.74
SCE1572_596	215.32	828.27
SCE1572_11452	27.47	7.35
SCE1572_3857	15.93	41.84
SCE1572_2273	22.4	47.69
SCE1572_11099	30.54	28.37
SCE1572_4881	10.11	4.87
SCE1572_2555	27.34	13.16
SCE1572_263	2.66	0
SCE1572_3116	82.65	45.62
SCE1572_2747	30.78	34.86
SCE1572_4362	2.45	0
SCE1572_1105	37.08	21.51
SCE1572_805	70.25	60.46
SCE1572_3943	568.8	1236.47
SCE1572_11155	72.22	145.97
SCE1572_11479	6.89	0
SCE1572_8504	18.7	5.75
SCE1572_6550	56.98	46.49
SCE1572_8775	35.62	20.58
SCE1572_9000	117.1	223.19
SCE1572_699	14.38	2.88
SCE1572_9009	0	0
SCE1572_7519	38.15	242.5
SCE1572_4311	32.42	14.75
SCE1572_4040	134.98	71.2
SCE1572_3193	85.88	90.31
SCE1572_2455	37.53	80.3
SCE1572_1948	18.58	944.33
SCE1572_11377	20.74	63.69
SCE1572_532	44.27	16.67
SCE1572_4876	25.35	17.09
SCE1572_5264	76.88	370.06
SCE1572_8573	15.68	0
SCE1572_3523	81.98	116.23
SCE1572_1730	23.69	37.06

SCE1572_1852	33.06	89.11
SCE1572_2762	0	0
SCE1572_5605	31.62	9.76
SCE1572_1993	16.78	44.57
SCE1572_3633	28.38	11.71
SCE1572_1121	40.09	19.59
SCE1572_3531	39.28	7.56
SCE1572_1777	61.4	20.12
SCE1572_1754	31.52	22.55
SCE1572_854	211.76	693.65
SCE1572_6001	132.15	387.11
SCE1572_103	55.63	17.66
SCE1572_6348	0	0
SCE1572_4341	20.11	24.2
SCE1572_7308	0	0
SCE1572_4593	78.75	129.32
SCE1572_2443	2.71	0
SCE1572_11592	34.61	19.83
SCE1572_10298	43.96	46.12
SCE1572_8690	53.45	10.5
SCE1572_2866	106.21	31.03
SCE1572_5506	47.13	15.13
SCE1572_446	26.73	13.44
SCE1572_3504	26.76	51.88
SCE1572_767	11.66	3.69
SCE1572_11077	10.17	109.03
SCE1572_9066	80.38	22.02
SCE1572_4106	19.58	12.12
SCE1572_8733	55.06	27.85
SCE1572_6437	97.57	30.71
SCE1572_2522	37.14	68.88
SCE1572_5702	34.6	54.95
SCE1572_4399	39.31	6.76
SCE1572_2004	17.12	0
SCE1572_5982	11.34	3.41
SCE1572_2280	59.69	37.75
SCE1572_5937	25.81	5.18
SCE1572_907	25.27	9.5
SCE1572_1019	0	0
SCE1572_4462	0	0
SCE1572_2914	42.41	12.65
SCE1572_1026	11.66	519.31
SCE1572_6747	0	0
SCE1572_3715	102.14	147.89
SCE1572_10463	85.76	320.38
SCE1572_10456	68.57	13.63
SCE1572_7170	38.06	63.07
SCE1572_10867	26.76	17.89

SCE1572_6781	43.32	466.29
SCE1572_8271	24.97	90.68
SCE1572_7746	56.61	63.16
SCE1572_7501	39.02	226.06
SCE1572_9785	25.27	10.58
SCE1572_10379	0	0
SCE1572_7156	36.96	72.38
SCE1572_10564	66.49	27.18
SCE1572_4873	178.65	472.42
SCE1572_2476	55.42	13.8
SCE1572_3851	30.94	372.37
SCE1572_1722	39.16	38.19
SCE1572_10270	48.81	339.93
SCE1572_1863	29.93	8.61
SCE1572_9554	45.78	107.33
SCE1572_4161	0	0
SCE1572_1982	0	0
SCE1572_6044	11.54	289.56
SCE1572_5967	22.9	5.07
SCE1572_10300	57.4	328.88
SCE1572_7582	181.61	683.01
SCE1572_522	27.63	34.1
SCE1572_7473	36.4	100.07
SCE1572_8194	32.98	6.78
SCE1572_6049	33.63	188.07
SCE1572_2032	50.22	29.18
SCE1572_1920	27.52	29.87
SCE1572_5324	46.04	110.82
SCE1572_2426	323.75	703.78
SCE1572_6490	61.21	24.93
SCE1572_1929	14.03	29.56
SCE1572_3483	89.11	122.71
SCE1572_8518	24.33	169.7
SCE1572_3738	6.42	5.79
SCE1572_3246	136.04	433.01
SCE1572_11563	22.61	12.2
SCE1572_4036	11.36	0.85
SCE1572_11241	61.62	60.82
SCE1572_9560	47.57	37.46
SCE1572_933	47.5	97.6
SCE1572_2196	36.73	574.26
SCE1572_3350	25.2	14.39
SCE1572_7245	67.67	101.02
SCE1572_2355	24.34	29.29
SCE1572_3647	18.21	89.86
SCE1572_9304	57.83	158.76
SCE1572_218	32.91	110.33
SCE1572_1484	71.43	36.6

SCE1572_1038	14.96	4.8
SCE1572_8793	13.1	0
SCE1572_5488	74.43	17.06
SCE1572_36	38.17	65.88
SCE1572_5827	25.12	19.76
SCE1572_5049	31.18	57.39
SCE1572_7939	8.66	29.19
SCE1572_2833	53.16	298.57
SCE1572_3374	97.78	3741.94
SCE1572_114	44.28	9.69
SCE1572_4240	37.78	24.57
SCE1572_2970	30.9	50.47
SCE1572_6536	66.42	134.6
SCE1572_4634	43.88	17.13
SCE1572_1638	15.45	9.3
SCE1572_7781	14.91	0
SCE1572_137	26.21	16.22
SCE1572_6304	40.3	101.62
SCE1572_3829	28.39	237.91
SCE1572_2629	34.09	18
SCE1572_2532	34.37	223.09
SCE1572_8479	53.97	364.92
SCE1572_3863	14.15	6.09
SCE1572_9466	38.75	60.72
SCE1572_913	13.26	21.94
SCE1572_8869	46.92	23.77
SCE1572_174	25.76	12.68
SCE1572_7822	90.97	563.02
SCE1572_10818	43.25	47.15
SCE1572_158	146.17	1011.29
SCE1572_8343	18.27	4.4
SCE1572_6081	26.44	0
SCE1572_3007	20.36	20.27
SCE1572_7142	60.45	85.18
SCE1572_8850	16.79	33.89
SCE1572_1499	30.65	7.76
SCE1572_4970	35.86	9.86
SCE1572_3033	49.44	70.98
SCE1572_11487	20.29	2.76
SCE1572_3895	23.02	42.03
SCE1572_1297	47.88	495.1
SCE1572_8394	96.99	193.84
SCE1572_832	14.54	10
SCE1572_6684	28.45	82.35
SCE1572_2646	0	0
SCE1572_7935	24.35	13.25
SCE1572_2880	23.45	45.15
SCE1572_737	63.95	44.97

SCE1572_11026	17.08	15.42
SCE1572_8584	0	0
SCE1572_9851	37.9	16.89
SCE1572_300	22.74	16.94
SCE1572_1462	19.78	0
SCE1572_9135	10.46	5.03
SCE1572_3843	7.58	0
SCE1572_9223	462.99	95.52
SCE1572_7790	0	0
SCE1572_4905	47.96	932.79
SCE1572_235	5.41	8.69
SCE1572_6411	24.61	19.11
SCE1572_346	62.19	24.58
SCE1572_3217	33.15	21.28
SCE1572_4895	25.95	16.44
SCE1572_5062	55.02	149.61
SCE1572_11600	12.29	1.97
SCE1572_1706	44.96	75.04
SCE1572_1442	40.62	17.42
SCE1572_1395	51.02	3252.69
SCE1572_1762	151.2	27
SCE1572_10141	8.34	29.52
SCE1572_7863	34.24	3.92
SCE1572_9319	20.37	5.45
SCE1572_4398	15.54	14.82
SCE1572_5775	62.53	57.51
SCE1572_11182	33.82	10.85
SCE1572_3597	0	0
SCE1572_333	43.72	50.57
SCE1572_3240	5.14	0
SCE1572_7435	33.3	6.22
SCE1572_3733	0	0
SCE1572_2577	40.55	18.67
SCE1572_6127	41.9	167.14
SCE1572_394	29.39	48.68
SCE1572_11023	55.54	177.11
SCE1572_6499	22.56	24.98
SCE1572_11516	14.8	7.7
SCE1572_8327	61.88	40.62
SCE1572_5040	40.55	160.83
SCE1572_7568	142.17	84.6
SCE1572_499	30.05	18.35
SCE1572_662	367.48	1028.16
SCE1572_1570	69.22	128.25
SCE1572_4042	38.99	31.28
SCE1572_4667	0	0
SCE1572_5851	0	0
SCE1572_1904	10.24	0

SCE1572_5389	47.81	148.82
SCE1572_1111	54.82	8.25
SCE1572_8090	24.92	5.06
SCE1572_1869	34.28	212.68
SCE1572_6325	35.38	152.05
SCE1572_10884	17.73	14.94
SCE1572_2956	9.27	5.82
SCE1572_481	59.04	15.92
SCE1572_9264	77.29	113.05
SCE1572_6309	36	47.26
SCE1572_415	38.53	98.76
SCE1572_6452	54.97	22.5
SCE1572_2108	28.34	138.61
SCE1572_9106	70.18	8.34
SCE1572_8175	11.97	0
SCE1572_2383	19.88	20.64
SCE1572_11103	11.26	21.9
SCE1572_1043	32.41	80.53
SCE1572_9634	33.91	43.19
SCE1572_10138	0	0
SCE1572_10471	13.6	20.66
SCE1572_3076	96.4	88.59
SCE1572_10026	160.71	890.41
SCE1572_7314	34.54	15.75
SCE1572_5623	34.96	118.49
SCE1572_2961	79.47	624.95
SCE1572_9505	10.67	8.56
SCE1572_1966	51.32	416.39
SCE1572_9229	3.53	0
SCE1572_4801	18.31	44.08
SCE1572_8879	27.72	9.1
SCE1572_8308	301.48	872.23
SCE1572_1144	22.32	20.54
SCE1572_7834	2.3	0
SCE1572_9325	0	0
SCE1572_3132	14.52	10.23
SCE1572_476	66.64	182.46
SCE1572_6066	6.46	1.35
SCE1572_5036	7.15	13.77
SCE1572_8367	32.25	10.88
SCE1572_8457	95	167.32
SCE1572_6832	20.03	8.51
SCE1572_4530	19.16	6.15
SCE1572_8350	28.41	0
SCE1572_11485	149.52	184.98
SCE1572_670	35.49	113.15
SCE1572_10991	38.14	45.33
SCE1572_5285	26.66	44.11

SCE1572_7685	19.24	11.11
SCE1572_5289	24.77	4.77
SCE1572_9226	35.2	20.64
SCE1572_2887	15.55	2.34
SCE1572_3146	23.33	8.02
SCE1572_3317	53.85	362.43
SCE1572_1466	89.65	22.21
SCE1572_8346	52.91	19.8
SCE1572_1278	46	26.65
SCE1572_928	45.26	42.66
SCE1572_8133	68.61	12.23
SCE1572_7010	40.07	32.9
SCE1572_2785	10.22	10.82
SCE1572_784	31.75	7.9
SCE1572_6738	15.91	35.89
SCE1572_10751	45.41	18.16
SCE1572_8429	36.79	45.04
SCE1572_6844	28.21	48.6
SCE1572_5382	22.94	8.42
SCE1572_9078	20.19	1.91
SCE1572_7185	35.41	13.11
SCE1572_5166	8.27	8.85
SCE1572_2630	40.26	11.8
SCE1572_6459	35.76	6.89
SCE1572_11262	23.41	55.45
SCE1572_724	85.98	25.59
SCE1572_11189	65.09	22.94
SCE1572_7093	17.29	15.26
SCE1572_11120	18.05	14.48
SCE1572_5728	37.46	46.68
SCE1572_7075	14.67	23.54
SCE1572_1205	14.23	2.94
SCE1572_8470	29.01	34.25
SCE1572_1662	105.95	360.53
SCE1572_11042	323.03	77.61
SCE1572_2558	17.19	8.8
SCE1572_2157	49.07	151.61
SCE1572_9046	226.22	79.1
SCE1572_6447	7.82	15.05
SCE1572_6174	43.21	57.38
SCE1572_7598	16.54	86.25
SCE1572_11258	17.26	47.47
SCE1572_3463	48.57	33.4
SCE1572_10058	256.96	1741.67
SCE1572_10900	46.8	200.48
SCE1572_11197	58.31	59.88
SCE1572_4979	37.9	235.68
SCE1572_10368	15.05	5.57

SCE1572_2806	60.35	24.46
SCE1572_7869	103.88	186.97
SCE1572_5991	49.01	60.21
SCE1572_11046	19.59	9.48
SCE1572_2227	55.33	179.26
SCE1572_6255	37.47	45.09
SCE1572_10762	49.02	48.47
SCE1572_6248	24.53	48.3
SCE1572_1766	10.74	2.07
SCE1572_5698	36.14	106.69
SCE1572_694	9.39	19.11
SCE1572_6195	50.54	12.58
SCE1572_9258	14.11	9.86
SCE1572_1671	36.53	515.66
SCE1572_1239	9.49	3.51
SCE1572_5092	0	0
SCE1572_10963	28.43	22.81
SCE1572_10065	292.4	1985.35
SCE1572_11064	17.01	21.22
SCE1572_1796	32.81	81.26
SCE1572_9126	8.51	7.68
SCE1572_3562	21.09	10.58
SCE1572_5365	14.38	24.83
SCE1572_4529	21.73	11.89
SCE1572_605	37.5	94.17
SCE1572_11141	47.63	17.24
SCE1572_7402	11.8	1131.69
SCE1572_7767	0	0
SCE1572_2584	52.35	10.33
SCE1572_2078	86.33	57.14
SCE1572_7591	65.42	38.06
SCE1572_6023	50.35	61.05
SCE1572_3685	12.36	2.63
SCE1572_1559	23.06	8.41
SCE1572_9817	30.32	28.67
SCE1572_6707	0	0
SCE1572_1271	48.2	26.77
SCE1572_10484	71.14	29.44
SCE1572_9989	21.66	13.43
SCE1572_10752	107.56	103.17
SCE1572_10872	50.39	32.91
SCE1572_2637	24.64	18.9
SCE1572_1652	16.24	0
SCE1572_11118	105.67	1200.19
SCE1572_11411	46.59	22.06
SCE1572_3424	103.35	50.64
SCE1572_3824	34.29	83.44
SCE1572_4838	154.86	122.51

SCE1572_4728	106.07	46.01
SCE1572_1550	36.1	20.85
SCE1572_1308	172.48	2455.07
SCE1572_1055	53.66	22.15
SCE1572_4302	49.36	29.7
SCE1572_425	68.54	914.81
SCE1572_9861	22.26	15.07
SCE1572_554	30.1	69.76
SCE1572_8554	40.91	135.4
SCE1572_4070	220.29	656.86
SCE1572_11060	26.46	41.64
SCE1572_1049	13.1	4.51
SCE1572_3183	21.58	31.44
SCE1572_8659	501.81	1427.4
SCE1572_6233	59.55	58.04
SCE1572_3355	80.67	26.83
SCE1572_2909	30.52	26.07
SCE1572_7327	53.69	25
SCE1572_1199	0	0
SCE1572_7951	82.61	414.05
SCE1572_11350	0	0
SCE1572_4775	0	0
SCE1572_2341	20.94	34.75
SCE1572_6960	58.62	22.24
SCE1572_10535	40.99	331.12
SCE1572_9173	15.73	6.89
SCE1572_7303	45.42	35.46
SCE1572_3290	13.15	4.22
SCE1572_2562	19.26	18.46
SCE1572_6812	44.72	10.5
SCE1572_7294	30.19	60.3
SCE1572_6156	21.85	8.22
SCE1572_10423	31.26	126.03
SCE1572_1798	43.18	191.7
SCE1572_867	34.18	64.31
SCE1572_9426	44.13	42.83
SCE1572_618	80.81	617.19
SCE1572_4790	28.82	54.11
SCE1572_10478	56.01	60.66
SCE1572_1538	19	17.28
SCE1572_7321	31.73	8.81
SCE1572_5318	50.91	20.2
SCE1572_4426	96.02	2123.5
SCE1572_9961	52.31	10.95
SCE1572_3399	22.33	17.5
SCE1572_2271	23.3	21.86
SCE1572_3322	3.37	0
SCE1572_8670	17.6	149.88

SCE1572_2240	20.27	35.85
SCE1572_9774	42.49	89.65
SCE1572_6094	111.22	25.13
SCE1572_1081	34.35	22.81
SCE1572_10555	63.65	12.68
SCE1572_6107	42.39	15.7
SCE1572_2084	38.42	157.8
SCE1572_11575	32.33	208.21
SCE1572_3778	34.18	28
SCE1572_10625	129.22	62.2
SCE1572_6937	25.7	27.83
SCE1572_10455	20.87	8.86
SCE1572_9644	13.89	58.5
SCE1572_3000	50.63	17.8
SCE1572_2608	29.09	17.13
SCE1572_7957	282.1	2485.79
SCE1572_8526	70.36	128.8
SCE1572_5398	59	27.48
SCE1572_9133	5.41	2.61
SCE1572_4520	63.47	161.24
SCE1572_10802	14.72	5.97
SCE1572_8383	32.38	18.73
SCE1572_2984	122.35	85.95
SCE1572_9913	0	0
SCE1572_7941	59.16	167.85
SCE1572_11333	65.87	85.37
SCE1572_5311	69.72	71.92
SCE1572_5915	126.07	159.28
SCE1572_2604	17.79	14.27
SCE1572_11523	10.94	15.05
SCE1572_7730	14.5	2.83
SCE1572_5304	63.96	82.68
SCE1572_5342	12.07	10.89
SCE1572_1301	35.35	18.98
SCE1572_5180	26.42	968.48
SCE1572_10879	59.15	188.32
SCE1572_11359	58.39	38.53
SCE1572_1659	27.53	25.48
SCE1572_2143	17.69	21.29
SCE1572_9587	36.87	78.2
SCE1572_5128	141.46	623.57
SCE1572_10672	6.82	8.69
SCE1572_11172	18.17	10.56
SCE1572_7183	48.09	21.79
SCE1572_884	178.96	197.42
SCE1572_10854	36.54	36.39
SCE1572_10047	66.71	189.76
SCE1572_11214	63.08	24.05

SCE1572_492	0	0
SCE1572_5377	24.96	10.23
SCE1572_10758	47.03	33.17
SCE1572_7893	625.74	24527.11
SCE1572_2937	92.53	952.56
SCE1572_8412	57.64	63.15
SCE1572_7674	57.3	15.04
SCE1572_7385	37.19	17.21
SCE1572_2347	2225.14	24547.25
SCE1572_5239	8.87	13.87
SCE1572_9085	21.66	17.38
SCE1572_5234	205.94	2928.5
SCE1572_4208	43.03	116.82
SCE1572_2254	26.91	4.11
SCE1572_10983	22.8	39.19
SCE1572_6349	39.24	48
SCE1572_154	53.3	76.98
SCE1572_5751	142.82	147.32
SCE1572_251	38.83	3.8
SCE1572_9002	272.06	296.71
SCE1572_10974	3.41	4.1
SCE1572_9471	17.63	12.73
SCE1572_10276	403.8	225.04
SCE1572_8275	53.83	99.23
SCE1572_107	52.99	88.57
SCE1572_2037	35.11	222.79
SCE1572_2027	0	0
SCE1572_9580	57.4	28.53
SCE1572_722	46.65	13.72
SCE1572_790	0	0
SCE1572_10678	44.29	229.49
SCE1572_9628	44.98	20.03
SCE1572_6344	43.19	38.89
SCE1572_4279	47.75	60.48
SCE1572_6372	0	0
SCE1572_7358	32.99	68.25
SCE1572_5332	33.56	37.47
SCE1572_8119	72.83	178.28
SCE1572_3827	69.19	235.12
SCE1572_3017	25.07	30.17
SCE1572_5871	23.54	23.53
SCE1572_8940	50.74	172.03
SCE1572_3039	84.42	12.44
SCE1572_10237	34.72	16.92
SCE1572_6956	45.86	2.25
SCE1572_5857	0	0
SCE1572_2754	52.57	52.46
SCE1572_10904	28.82	439.85

SCE1572_11090	48.17	89.33
SCE1572_10466	36.47	25.63
SCE1572_5725	19.75	0
SCE1572_1181	12.25	0
SCE1572_1583	48.73	4.65
SCE1572_4974	95.83	1450.21
SCE1572_10847	52.21	22.38
SCE1572_6622	43.32	32.41
SCE1572_9086	59.17	14.83
SCE1572_1509	58.9	15.94
SCE1572_7548	53.95	17.91
SCE1572_7427	31.66	19.53
SCE1572_5843	21.84	6.19
SCE1572_1156	0	0
SCE1572_3387	19.52	3.13
SCE1572_1721	9.12	0
SCE1572_8857	63.08	149.17
SCE1572_4982	36.81	6.33
SCE1572_6479	30.58	180.52
SCE1572_1439	23.14	8.64
SCE1572_5552	3.23	11.65
SCE1572_9144	18.58	7.21
SCE1572_819	46.21	27.8
SCE1572_2742	25.16	47.81
SCE1572_5765	45.06	140.64
SCE1572_8185	100.3	5.61
SCE1572_1519	60.42	35.5
SCE1572_5777	54.25	512.23
SCE1572_10680	0	0
SCE1572_3867	49.01	217.48
SCE1572_10169	94.2	874.52
SCE1572_1315	25.71	95.43
SCE1572_9968	21.89	7.08
SCE1572_3736	38.47	50.83
SCE1572_810	33.85	24.49
SCE1572_7909	37.37	72.64
SCE1572_2256	24.47	14.54
SCE1572_8214	53.91	44.75
SCE1572_3020	66.8	20.63
SCE1572_6906	0	0
SCE1572_6577	17.27	15.01
SCE1572_3489	19.69	6.32
SCE1572_6965	21.74	16.97
SCE1572_1831	0	0
SCE1572_8681	44.59	11.45
SCE1572_3756	35.45	9.82
SCE1572_5868	98.83	82.74
SCE1572_3928	85.48	90.1

SCE1572_4724	54.26	26.87
SCE1572_1444	17.04	21.18
SCE1572_7487	42.95	23.42
SCE1572_7528	33.37	30.47
SCE1572_6488	11.43	4.34
SCE1572_7581	556.71	1523.64
SCE1572_11247	22.46	16.89
SCE1572_4786	338.42	4659.99
SCE1572_297	48.31	28.45
SCE1572_6386	31.98	9.5
SCE1572_9944	3.3	0
SCE1572_3357	0	0
SCE1572_10508	74.24	352.34
SCE1572_2044	3.16	0
SCE1572_5255	4.67	0
SCE1572_4405	28.84	7.12
SCE1572_4497	37.54	103.11
SCE1572_6970	10.24	180
SCE1572_6083	38.14	9.36
SCE1572_8916	28.63	130.15
SCE1572_6801	35.38	28.38
SCE1572_1160	23.8	12.63
SCE1572_703	24.68	12.73
SCE1572_658	512	746.69
SCE1572_3891	41.38	54.77
SCE1572_9614	92.99	0
SCE1572_9589	32.37	74.6
SCE1572_5653	54.26	331.89
SCE1572_6562	18.18	15.58
SCE1572_10996	36.73	18.81
SCE1572_8164	16.44	17.59
SCE1572_7116	62.12	129.24
SCE1572_633	19.28	33.75
SCE1572_6992	64.7	22.58
SCE1572_5512	55.88	94.74
SCE1572_1502	57.05	222.51
SCE1572_145	28.24	23.25
SCE1572_433	41.69	32.21
SCE1572_7636	363.24	7582.13
SCE1572_1909	0	0
SCE1572_11261	131.08	57.02
SCE1572_6529	31.7	33.06
SCE1572_4675	22.82	16.53
SCE1572_11512	364.07	1268.22
SCE1572_6191	27.4	76.94
SCE1572_5454	26.22	13.72
SCE1572_6379	21.05	0
SCE1572_8907	42.92	44.27

SCE1572_1482	51.24	21.96
SCE1572_9810	28.25	34.99
SCE1572_8771	0	0
SCE1572_9442	11.66	2.25
SCE1572_4746	37.28	18.98
SCE1572_4706	49.35	46.02
SCE1572_11442	39.52	43.67
SCE1572_9347	37.04	12.44
SCE1572_4991	8.62	6.92
SCE1572_3699	31.41	5.21
SCE1572_4956	35.44	59.42
SCE1572_7881	27.55	26.16
SCE1572_2392	95.58	258.82
SCE1572_8417	22.06	17.02
SCE1572_6319	34.1	31.57
SCE1572_8197	20.26	10.09
SCE1572_10418	36.24	50.49
SCE1572_10515	103.94	195.63
SCE1572_4360	65.52	119.28
SCE1572_8900	161.12	160.07
SCE1572_8949	11.78	22.44
SCE1572_9208	15.16	16.68
SCE1572_527	25.64	58.18
SCE1572_2191	93.5	1067.07
SCE1572_7236	27.59	83.89
SCE1572_366	82.15	279.72
SCE1572_7615	23.92	19.02
SCE1572_5493	0	0
SCE1572_9244	7.34	0
SCE1572_4756	43.85	22.89
SCE1572_3258	21.41	98.83
SCE1572_8621	43.59	155.84
SCE1572_1347	147.92	618.88
SCE1572_6545	64.6	282.42
SCE1572_3973	21.78	72.58
SCE1572_874	160.97	78.09
SCE1572_3435	52.85	69.39
SCE1572_10544	15.55	3.12
SCE1572_4418	19.36	15.53
SCE1572_2618	0	0
SCE1572_732	41.71	21.98
SCE1572_5028	30.13	23.65
SCE1572_3336	40.48	501.97
SCE1572_5055	33.72	19.83
SCE1572_11503	39.74	16.08
SCE1572_6659	60.16	72.39
SCE1572_4969	26.76	8.59
SCE1572_8161	25.72	2.77

SCE1572_10952	0	0
SCE1572_4633	36.23	31.22
SCE1572_5749	25.45	18.71
SCE1572_5678	81.32	256.66
SCE1572_2652	54.9	27.67
SCE1572_7229	25.81	134.42
SCE1572_7105	36.87	17.04
SCE1572_6520	26.12	25.83
SCE1572_1768	70.5	23.28
SCE1572_6860	45.56	22.92
SCE1572_3239	19.12	13.15
SCE1572_7215	34.9	64.32
SCE1572_2622	38.57	3.2
SCE1572_3088	28.64	24.33
SCE1572_10855	46.34	31.76
SCE1572_8845	20.67	9.33
SCE1572_5306	426.08	1492.86
SCE1572_9360	35.57	327.21
SCE1572_278	16.69	6.7
SCE1572_10036	16.74	6.72
SCE1572_4164	48.26	18.77
SCE1572_3242	17.27	0
SCE1572_1413	18.81	14.14
SCE1572_8541	17.89	239.86
SCE1572_5886	41.9	19.74
SCE1572_1843	48.06	22.81
SCE1572_8862	48.88	12
SCE1572_6516	11.87	2.86
SCE1572_3162	47.88	151
SCE1572_11169	27.74	7.03
SCE1572_11019	51.47	147.31
SCE1572_9160	39.51	110.53
SCE1572_5205	47.96	48.97
SCE1572_3871	41.52	94.32
SCE1572_5020	24.36	18.32
SCE1572_4232	16.8	4.49
SCE1572_258	40.66	61.16
SCE1572_1062	120.23	14.71
SCE1572_10598	0	0
SCE1572_9707	196.21	64.4
SCE1572_7718	30.8	12.43
SCE1572_10089	40.98	103.56
SCE1572_10080	275.77	1102.7
SCE1572_3706	58.62	47.03
SCE1572_5803	21.26	24.24
SCE1572_6893	25.62	11.26
SCE1572_6999	57.9	7.34
SCE1572_9679	144.72	484.35

SCE1572_3513	40.06	309.94
SCE1572_9856	28.93	16.81
SCE1572_583	19.37	68.93
SCE1572_2113	54.24	50.33
SCE1572_8355	39.11	15.97
SCE1572_1368	37.23	75.68
SCE1572_801	28.29	24.41
SCE1572_7222	30.39	585.22
SCE1572_7452	33.52	53.78
SCE1572_2659	54.32	40.98
SCE1572_3275	284.77	533.1
SCE1572_7534	5.83	0
SCE1572_404	61.03	65.41
SCE1572_10619	73.31	45.62
SCE1572_3921	48.88	54.47
SCE1572_4488	66.43	32.09
SCE1572_5095	41.23	139.9
SCE1572_8847	0	0
SCE1572_4835	126.98	102.63
SCE1572_2724	36.85	157.02
SCE1572_628	15.16	28.75
SCE1572_9309	0	0
SCE1572_6455	2.14	0
SCE1572_2575	25.17	12.7
SCE1572_7573	30.1	224.57
SCE1572_3003	18.47	57.42
SCE1572_5671	60.19	31.44
SCE1572_202	52.2	45.27
SCE1572_3455	19.27	6.14
SCE1572_11332	33.97	87.74
SCE1572_7804	67.35	58.56
SCE1572_10718	15.29	52.13
SCE1572_4686	49.1	248.77
SCE1572_4249	2.14	0
SCE1572_6869	22.5	15.47
SCE1572_10052	28.7	84.71
SCE1572_1709	56.12	42.78
SCE1572_969	42.35	121.15
SCE1572_5229	31.52	72.26
SCE1572_5143	70.11	10.55
SCE1572_1711	62.9	45.11
SCE1572_7843	80.57	342.01
SCE1572_10230	49.74	16.41
SCE1572_6294	20.44	342.37
SCE1572_3338	203.96	3041.02
SCE1572_1012	37.19	5.51
SCE1572_4844	37.35	68.75
SCE1572_10282	35.11	126.76

SCE1572_6288	34.18	12.66
SCE1572_189	48.78	57.12
SCE1572_1058	5.09	2.45
SCE1572_1728	54.03	32.51
SCE1572_8474	14.17	13.57
SCE1572_2423	127.03	355.06
SCE1572_9406	21.19	51.01
SCE1572_2502	0	0
SCE1572_6413	126.19	77.06
SCE1572_5023	0	0
SCE1572_5220	25.82	73.38
SCE1572_4411	24.52	6.89
SCE1572_4015	90.58	144.57
SCE1572_1094	30.72	14.47
SCE1572_10263	56.94	33.46
SCE1572_5869	19.33	198.38
SCE1572_7228	82.13	1060.98
SCE1572_9169	38.78	18.3
SCE1572_651	69.16	0
SCE1572_198	32.06	80.28
SCE1572_8376	7.64	0
SCE1572_5934	16.11	3.53
SCE1572_5030	133.21	902.2
SCE1572_11284	24.61	36.73
SCE1572_11299	42.57	15.44
SCE1572_9896	9.13	0
SCE1572_5005	42.96	0
SCE1572_6878	186	40.13
SCE1572_10973	50.65	242.2
SCE1572_5136	22.41	21.22
SCE1572_5958	50.86	46.19
SCE1572_459	13.04	3.92
SCE1572_8758	74.79	340.31
SCE1572_7972	16.44	89.4
SCE1572_10646	72.64	34.69
SCE1572_9661	262.85	1816.64
SCE1572_5734	48.49	104.38
SCE1572_3619	38.35	14.27
SCE1572_10393	80.71	32.68
SCE1572_1211	30.51	24.48
SCE1572_86	0	0
SCE1572_7210	4.84	1.94
SCE1572_11560	45.35	24.66
SCE1572_3286	27.03	16.97
SCE1572_10040	28.6	40.6
SCE1572_3521	52.86	60.82
SCE1572_3611	23.63	24.18
SCE1572_11208	84.16	135.29

SCE1572_3545	0	0
SCE1572_3472	37.09	7.81
SCE1572_9902	21.87	24.56
SCE1572_7735	536.4	3430.82
SCE1572_8529	48.51	31.46
SCE1572_7764	10.24	4.93
SCE1572_5218	51.52	24.8
SCE1572_1547	24.63	35.2
SCE1572_3390	59.33	150.73
SCE1572_6109	40.43	6.49
SCE1572_3202	131.4	3207.09
SCE1572_7550	65.64	22.85
SCE1572_5119	28.5	61.68
SCE1572_11180	7.29	2.63
SCE1572_6852	0	0
SCE1572_9921	59.69	82.86
SCE1572_10245	50.26	14.11
SCE1572_10707	0	0
SCE1572_182	119.89	128.74
SCE1572_7084	132.93	71.1
SCE1572_9288	709.78	1088.31
SCE1572_565	105.14	367.59
SCE1572_8684	16.9	28.36
SCE1572_5174	7.19	1.73
SCE1572_6598	51.8	39.53
SCE1572_9036	28.98	18.32
SCE1572_354	24.5	14.74
SCE1572_8789	38.24	95.29
SCE1572_4433	56.42	19.4
SCE1572_2202	54.73	487.75
SCE1572_2051	62.4	19.76
SCE1572_2519	57.96	20.49
SCE1572_8808	16.77	8.77
SCE1572_8126	23.66	10.17
SCE1572_11395	102.3	621.39
SCE1572_4291	16.9	26.73
SCE1572_11162	26.27	57.97
SCE1572_7847	52.97	48.36
SCE1572_621	36.63	22.65
SCE1572_1871	49.98	48.92
SCE1572_5664	13.89	29.51
SCE1572_3668	41.66	18.08
SCE1572_1247	26.36	7.61
SCE1572_3344	102.52	356
SCE1572_10570	87.74	150.83
SCE1572_778	30.67	41.52
SCE1572_6834	0	0
SCE1572_10181	17.98	24.04

SCE1572_3625	46.21	13.9
SCE1572_3676	117.75	183.94
SCE1572_3462	1.67	72.18
SCE1572_8015	18.3	229.65
SCE1572_5240	7.75	23.97
SCE1572_4519	34.33	214.59
SCE1572_52	6.15	0
SCE1572_4220	31.64	38.08
SCE1572_10248	52.02	21.4
SCE1572_2641	65.36	57.47
SCE1572_8560	32.64	22.7
SCE1572_3814	53.78	66.81
SCE1572_1870	5.78	1.07
SCE1572_10720	43.25	17.35
SCE1572_8318	9.97	4.8
SCE1572_4287	25.09	7.49
SCE1572_27	715.39	1257.74
SCE1572_5573	39.33	21.85
SCE1572_271	167.6	1088.58
SCE1572_7575	48.77	115.87
SCE1572_3477	0	0
SCE1572_10788	65.34	45.46
SCE1572_7831	40.43	116.35
SCE1572_6019	34.53	25.29
SCE1572_8926	35.22	57.6
SCE1572_10949	42.51	10.23
SCE1572_6619	31.81	15.31
SCE1572_4002	113.52	1573.84
SCE1572_1375	43.66	43.45
SCE1572_1455	61.79	36.03
SCE1572_11589	15.72	24.41
SCE1572_3806	26.37	0
SCE1572_8656	81.86	917.16
SCE1572_11546	39.03	4.32
SCE1572_1548	51.71	25.11
SCE1572_6700	10.13	4.48
SCE1572_4268	50.25	14.6
SCE1572_2899	71.26	20.72
SCE1572_9243	70.24	57.13
SCE1572_6640	61.97	20.64
SCE1572_3939	12.16	15.49
SCE1572_6596	19.42	3.9
SCE1572_7541	29.11	49.63
SCE1572_826	40.9	12.54
SCE1572_3816	0	0
SCE1572_3748	34.91	61.51
SCE1572_5350	416.44	380.74
SCE1572_7442	19.07	7.84

SCE1572_6919	39.52	29.4
SCE1572_6028	22.17	157.92
SCE1572_7870	71.79	77.45
SCE1572_7692	120.13	199.44
SCE1572_10068	254.23	1070.29
SCE1572_9887	32.75	40.69
SCE1572_4132	65.46	134.18
SCE1572_3136	43.16	17.31
SCE1572_5594	30.74	23.83
SCE1572_1393	22.3	17.89
SCE1572_11191	60.58	88.67
SCE1572_7697	33.04	44.5
SCE1572_9746	51.04	41.91
SCE1572_2483	29.42	16.34
SCE1572_9880	19.19	18.48
SCE1572_3058	32.22	13.68
SCE1572_5360	0	0
SCE1572_4853	20.31	8.73
SCE1572_4477	45.12	12.72
SCE1572_8241	24.44	91
SCE1572_5922	8.78	19.21
SCE1572_10321	35.18	108.87
SCE1572_7701	40.05	6.89
SCE1572_7504	35.24	17.61
SCE1572_5322	72.65	83.63
SCE1572_3839	60.97	25.97
SCE1572_8727	53.45	19.25
SCE1572_10878	59.1	14.43
SCE1572_9496	52.1	160.52
SCE1572_5410	0	0
SCE1572_771	42.41	47.42
SCE1572_9681	138.2	385.91
SCE1572_7838	22.99	6.3
SCE1572_3751	41.06	30.41
SCE1572_6463	0	0
SCE1572_6330	41.89	141.37
SCE1572_6320	13.31	12.46
SCE1572_11550	20.87	18.51
SCE1572_129	5.91	0
SCE1572_7099	31.42	3.29
SCE1572_5918	0	0
SCE1572_6070	10.2	21.05
SCE1572_1566	23.68	8.88
SCE1572_2510	51.8	54.49
SCE1572_5590	67.54	22.48
SCE1572_10261	97.42	91.09
SCE1572_2710	37.44	21.02
SCE1572_9562	11.23	0

SCE1572_7925	22.24	7.3
SCE1572_350	51.23	1825.44
SCE1572_6753	57.65	39.91
SCE1572_8567	48.69	46.77
SCE1572_4212	2.61	0
SCE1572_5584	37.61	15.09
SCE1572_83	27.7	29.89
SCE1572_3126	29.42	177.01
SCE1572_4546	26.64	8.22
SCE1572_9280	15.76	6.54
SCE1572_2144	14.7	49.65
SCE1572_7877	72.46	16.12
SCE1572_11112	21.19	18.13
SCE1572_5536	38.08	37
SCE1572_4629	56.9	136.96
SCE1572_4239	80.18	354.86
SCE1572_9675	38.75	92.72
SCE1572_4943	74.27	430.91
SCE1572_9639	0	0
SCE1572_9236	30.52	14.22
SCE1572_2690	27.92	17.27
SCE1572_9536	84.92	38.37
SCE1572_4765	15.54	10.59
SCE1572_1222	34.51	17.8
SCE1572_6185	37.54	19.95
SCE1572_1174	16.24	0
SCE1572_4948	20.84	12.11
SCE1572_2891	8.79	0
SCE1572_8890	208.85	71.16
SCE1572_8668	15.96	2.4
SCE1572_10820	110.27	201.42
SCE1572_2878	61.3	79.05
SCE1572_2056	33.22	4.51
SCE1572_7258	51.03	29.52
SCE1572_487	59.58	74.46
SCE1572_201	3.94	0
SCE1572_11034	109.43	1709.33
SCE1572_4620	17.96	34.33
SCE1572_392	60.79	7.57
SCE1572_6204	30.72	64.3
SCE1572_845	0	0
SCE1572_57	67.53	20.44
SCE1572_5473	14.03	22.08
SCE1572_441	473.54	202.46
SCE1572_7206	23.33	6.24
SCE1572_5821	28.54	36.26
SCE1572_5599	98.35	51.68
SCE1572_4280	33.69	21.47

SCE1572_4142	0	0
SCE1572_30	180.68	513.49
SCE1572_9216	57.78	95.37
SCE1572_5900	13.65	18.77
SCE1572_2316	15.56	9.55
SCE1572_8546	48.24	17.56
SCE1572_4035	41.44	32.85
SCE1572_4566	14.01	13.8
SCE1572_7285	91.83	84.95
SCE1572_4659	46.98	59.62
SCE1572_6695	12.86	10.67
SCE1572_7588	48.77	392.72
SCE1572_3693	48.48	18.7
SCE1572_10959	28.77	12.22
SCE1572_9959	13.81	17.68
SCE1572_9932	62.98	5.61
SCE1572_7641	98.47	1032.43
SCE1572_11517	21.09	15.31
SCE1572_9714	44.28	1401.56
SCE1572_6942	35.27	16.69
SCE1572_763	41.35	21.11
SCE1572_9828	34.13	19.95
SCE1572_3221	42.29	105.64
SCE1572_11381	18.89	46.59
SCE1572_6166	58.27	196.07
SCE1572_10999	58.48	5.21
SCE1572_1266	927.38	11063.23
SCE1572_8247	29.5	16.51
SCE1572_7600	52.75	79.76
SCE1572_11321	32.25	24.51
SCE1572_4077	25.27	72.59
SCE1572_2870	16.03	8.04
SCE1572_8362	0	0
SCE1572_10940	22.4	13.82
SCE1572_951	111.57	158.36
SCE1572_10832	50.05	283.05
SCE1572_1389	20.34	233.18
SCE1572_2542	71.92	9.36
SCE1572_9514	90.97	724.5
SCE1572_3304	8.16	5.61
SCE1572_3912	11.43	33.01
SCE1572_1370	60.98	220.16
SCE1572_4295	38.41	14.6
SCE1572_10566	60.38	26.35
SCE1572_8421	23.87	28.72
SCE1572_1080	28.02	20.75
SCE1572_5339	45.75	31.46
SCE1572_4656	25.39	69.84

SCE1572_3040	22.31	10.07
SCE1572_10227	29.71	9.86
SCE1572_7147	39.36	109.8
SCE1572_6168	14.53	17.02
SCE1572_1674	90.97	257.27
SCE1572_8000	57.69	25.84
SCE1572_6925	12.06	8.29
SCE1572_11373	121.19	754.67
SCE1572_10654	33.06	212.19
SCE1572_11526	84.61	31.44
SCE1572_9522	7.29	3.51
SCE1572_3414	87.28	128.66
SCE1572_1896	0	0
SCE1572_4602	35.91	103.23
SCE1572_6798	27.52	30.22
SCE1572_4080	37.73	206
SCE1572_4046	0	0
SCE1572_5246	305.03	1077.49
SCE1572_3212	18.4	30.11
SCE1572_4694	32.58	30.41
SCE1572_7983	168.22	209.1
SCE1572_6665	31.31	141.8
SCE1572_5568	18.12	15.17
SCE1572_3144	15.16	2.81
SCE1572_1125	54.28	19.71
SCE1572_8778	41.17	16.32
SCE1572_2334	0	0
SCE1572_518	0	0
SCE1572_10208	37.04	16.08
SCE1572_7369	8.29	2.85
SCE1572_10494	61.94	490.61
SCE1572_9621	35.79	16.25
SCE1572_8071	62.8	38.87
SCE1572_4448	22.13	10.65
SCE1572_6161	39.63	21.77
SCE1572_4937	33.57	80.8
SCE1572_8953	28.74	9.15
SCE1572_5974	0	0
SCE1572_5370	0	0
SCE1572_3855	45.53	26.5
SCE1572_3029	17.27	0
SCE1572_9805	27.09	18.49
SCE1572_10892	30.68	74.46
SCE1572_2766	79.86	68.25
SCE1572_11187	5.23	3.15
SCE1572_10505	47.44	21.19
SCE1572_1289	0	0
SCE1572_1035	28.57	22.74

SCE1572_6310	25.43	302.53
SCE1572_5179	5.86	94.76
SCE1572_2537	8.14	24.92
SCE1572_6910	43.03	15.26
SCE1572_3403	17.15	42.62
SCE1572_5182	12.9	432.21
SCE1572_9377	14.73	16.25
SCE1572_6223	29.64	16.46
SCE1572_6314	17.18	0
SCE1572_7551	106.22	86.59
SCE1572_5715	42.15	39.37
SCE1572_3608	73.85	858.83
SCE1572_8766	48.27	16.42
SCE1572_5000	100.39	118.35
SCE1572_10593	3.09	0
SCE1572_8597	56.63	18.53
SCE1572_3179	38.12	6.19
SCE1572_3	101.32	282.07
SCE1572_1100	40.98	58.08
SCE1572_11340	29.9	11.36
SCE1572_8296	22.74	68.01
SCE1572_389	11.03	8.29
SCE1572_381	40.53	10.84
SCE1572_11253	93.61	1304.73
SCE1572_2597	1.63	10.47
SCE1572_9019	4.8	3.85
SCE1572_4089	33.56	93.78
SCE1572_3529	83.35	823.04
SCE1572_4481	36.81	10.49
SCE1572_2321	79.47	420.29
SCE1572_9332	0	0
SCE1572_10122	62.4	22.32
SCE1572_6896	55.77	202.01
SCE1572_3372	23.57	34.71
SCE1572_3247	67.38	238.6
SCE1572_10434	49.09	48.06
SCE1572_10137	43.74	19.09
SCE1572_9031	58.11	40.29
SCE1572_7481	31.01	0
SCE1572_9653	64.68	22.86
SCE1572_7057	29.58	0
SCE1572_10190	67.21	194.73
SCE1572_11014	53.86	33.82
SCE1572_130	42.4	15.41
SCE1572_2927	25.07	5.75
SCE1572_215	41.63	33.75
SCE1572_7903	9.72	11.7
SCE1572_5295	15.38	3.53

SCE1572_8261	78.55	334.15
SCE1572_8648	54.93	8.26
SCE1572_6591	32.37	14.27
SCE1572_191	44.61	21.41
SCE1572_9062	53.07	29.86
SCE1572_10663	86.64	130.69
SCE1572_4467	21.72	6.54
SCE1572_4201	21.32	19.96
SCE1572_7106	19.78	7.93
SCE1572_11226	42.92	21.47
SCE1572_9212	42.33	54.06
SCE1572_483	20.43	12.29
SCE1572_3536	19.98	5.66
SCE1572_2471	19.48	5.7
SCE1572_2431	72.15	542.99
SCE1572_1981	0	0
SCE1572_3743	20.86	24.49
SCE1572_1737	40.01	15.21
SCE1572_8103	28.7	38.97
SCE1572_5541	40.95	159.39
SCE1572_10124	123.56	47.4
SCE1572_9576	30.99	7.99
SCE1572_4814	176.18	476.43
SCE1572_287	40.99	61.22
SCE1572_939	34.52	13.71
SCE1572_10096	38.39	95.98
SCE1572_5471	40.06	17.02
SCE1572_3832	31.79	98.1
SCE1572_3170	39.34	90.43
SCE1572_2094	17.08	33.41
SCE1572_7854	27.44	66.05
SCE1572_10582	12.4	13.27
SCE1572_9196	47.08	21.13
SCE1572_6709	43.7	88.01
SCE1572_4933	37.9	136.85
SCE1572_11276	59.64	31.63
SCE1572_8615	47.05	35.46
SCE1572_5393	155.3	73.49
SCE1572_5405	11.72	19.39
SCE1572_10589	16.85	4.51
SCE1572_2451	0	0
SCE1572_8320	10.46	16.78
SCE1572_1091	53.2	10.47
SCE1572_5467	41.45	16.18
SCE1572_7463	87.23	322.93
SCE1572_11039	27.46	22.26
SCE1572_7653	14.56	16.49
SCE1572_641	54.05	87.26

SCE1572_5148	31.43	2.97
SCE1572_10288	90.25	70.3
SCE1572_9654	24.51	7.51
SCE1572_10579	26.5	7.09
SCE1572_578	5.27	0
SCE1572_8086	19.22	0
SCE1572_711	67.27	313.52
SCE1572_6873	46.1	368.62
SCE1572_3209	43.38	41.77
SCE1572_3922	46.36	21.11
SCE1572_9978	42.06	6.25
SCE1572_7478	58.22	163.49
SCE1572_11264	33.81	118.35
SCE1572_5499	18.99	5.9
SCE1572_7404	8.75	152.99
SCE1572_6332	65.51	40.69
SCE1572_1334	6.05	0
SCE1572_3315	21.08	37.08
SCE1572_7994	58.31	0
SCE1572_6977	8.01	0
SCE1572_7278	28.03	9.2
SCE1572_11421	38.79	40.46
SCE1572_11450	35.09	60.43
SCE1572_9619	76.64	46.17
SCE1572_6744	15.47	2.98
SCE1572_7390	14.47	13.63
SCE1572_3313	33.69	10.46
SCE1572_11234	20.73	9.78
SCE1572_11398	47.64	267.36
SCE1572_3609	26.52	6.01
SCE1572_7167	0	0
SCE1572_5902	53.57	15.27
SCE1572_10184	13.78	64.4
SCE1572_6649	80.37	4.4
SCE1572_3427	43.07	35.72
SCE1572_8532	36.65	0
SCE1572_6125	21.28	61.46
SCE1572_8938	10.83	19.55
SCE1572_4767	109.93	195
SCE1572_1234	16.73	7.43
SCE1572_10344	799.46	6126.12
SCE1572_10932	21.03	37.97
SCE1572_7134	46.24	20.7
SCE1572_543	24.45	16.05
SCE1572_8986	2.33	0
SCE1572_8489	83.46	97.47
SCE1572_997	18.55	24.24
SCE1572_9488	55.13	70.63

SCE1572_10993	0	0
SCE1572_9594	22.55	445.76
SCE1572_3883	20.73	12.48
SCE1572_9994	82.56	29.47
SCE1572_11081	36.86	134.86
SCE1572_769	31.19	8.34
SCE1572_594	15.01	3.61
SCE1572_7655	23.55	8.86
SCE1572_7399	21.46	26.66
SCE1572_127	28.1	15.73
SCE1572_1979	1404.97	4309.83
SCE1572_6129	152.42	390.44
SCE1572_265	42.06	21.69
SCE1572_6030	21.46	19.78
SCE1572_1946	47.43	31.19
SCE1572_1436	31.08	18.13
SCE1572_992	128.55	262.62
SCE1572_891	34.1	84.65
SCE1572_7262	31.35	32.05
SCE1572_10310	11.22	6.14
SCE1572_9761	10.66	19.96
SCE1572_6003	52	726.83
SCE1572_6242	18.57	13.68
SCE1572_7526	0	0
SCE1572_5928	68.16	73.65
SCE1572_11303	33.06	33.66
SCE1572_413	27.92	11.77
SCE1572_1641	53.22	16.92
SCE1572_2061	27.24	15.3
SCE1572_7240	50.69	83.13
SCE1572_9710	52.88	132.13
SCE1572_7050	0	0
SCE1572_2769	17.44	16.32
SCE1572_760	1124.53	486.8
SCE1572_311	27.44	24.48
SCE1572_10401	34.42	74.96
SCE1572_9201	63.17	25.73
SCE1572_11401	46.78	25.23
SCE1572_6779	9.17	6.79
SCE1572_7985	32.95	72.69
SCE1572_4812	64.32	60.82
SCE1572_4155	17.71	51.94
SCE1572_2358	65.87	95.63
SCE1572_8550	38.45	117.3
SCE1572_807	49.35	39.19
SCE1572_9738	30.86	6.4
SCE1572_8444	24.68	19.09
SCE1572_2745	31.88	70.82

SCE1572_4334	28.57	7.93
SCE1572_4191	36.01	39.6
SCE1572_5248	26.34	53.43
SCE1572_3195	53.86	0
SCE1572_10492	63.37	229.77
SCE1572_9528	97.7	184.03
SCE1572_10296	221.97	4072.53
SCE1572_1469	6.52	11.77
SCE1572_11108	34.93	36.32
SCE1572_10211	47.62	40.94
SCE1572_6176	95.09	146.22
SCE1572_8250	57.96	125.08
SCE1572_8777	29.56	22.4
SCE1572_3502	37.77	39.52
SCE1572_9683	51.04	270.98
SCE1572_2219	0	0
SCE1572_10483	47.77	31.66
SCE1572_5786	40.58	39.71
SCE1572_7447	3.53	0
SCE1572_10272	43.65	509.6
SCE1572_1596	55.02	8.83
SCE1572_9116	97.72	297.03
SCE1572_6500	29.83	59.82
SCE1572_3302	101.08	121.03
SCE1572_9358	39.7	110.8
SCE1572_7955	41.86	71.86
SCE1572_7171	29.42	0
SCE1572_1807	12.92	37.84
SCE1572_105	36.48	0
SCE1572_1756	7.13	0
SCE1572_11477	41.78	8.69
SCE1572_494	41.86	12.71
SCE1572_6346	20.1	25.24
SCE1572_7313	39.98	23.87
SCE1572_1293	19.98	11.03
SCE1572_2688	12.88	19.73
SCE1572_9911	24.87	17.27
SCE1572_5190	59.69	189.8
SCE1572_10507	129.96	186.81
SCE1572_5266	33.47	107.11
SCE1572_8701	24.2	30.75
SCE1572_11210	61.77	29.73
SCE1572_7269	202.72	1120.45
SCE1572_4506	34.52	13.03
SCE1572_7009	73.58	24.82
SCE1572_9754	21.37	28.57
SCE1572_529	10.03	0
SCE1572_10361	25.84	901.94

SCE1572_10256	58.8	24.26
SCE1572_8571	15.59	6.82
SCE1572_2843	7.82	7.52
SCE1572_11203	34.57	62.4
SCE1572_677	48.25	27.72
SCE1572_9640	541.37	1262.76
SCE1572_5508	1.64	0
SCE1572_8228	21.24	6.1
SCE1572_9462	22.42	43.9
SCE1572_3950	0	0
SCE1572_1201	37.9	15.46
SCE1572_11085	52.67	138.29
SCE1572_1773	0	0
SCE1572_9068	16.44	16.95
SCE1572_721	46.73	34.03
SCE1572_1811	34.33	24.59
SCE1572_5554	89.53	92.97
SCE1572_3666	41.47	3.27
SCE1572_7163	49.59	80.71
SCE1572_10533	36.23	81.84
SCE1572_4583	0	0
SCE1572_2681	95.6	16.22
SCE1572_10353	2042.3	2793.71
SCE1572_9061	26.72	13.74
SCE1572_2907	53.07	549.04
SCE1572_208	10.64	3.2
SCE1572_6667	100.73	100.2
SCE1572_6091	31.59	8.69
SCE1572_11278	19.69	0
SCE1572_909	66.44	32.63
SCE1572_9352	29.96	175.1
SCE1572_3711	91.96	0
SCE1572_2182	13.69	6.34
SCE1572_4111	31.52	9.03
SCE1572_9379	15.36	5.55
SCE1572_4591	13.87	13.35
SCE1572_7188	43.75	113.48
SCE1572_3725	261.64	4918.72
SCE1572_2282	30.94	74.47
SCE1572_6761	6.08	5.57
SCE1572_1732	48.75	28.37
SCE1572_7905	62.26	22.92
SCE1572_3717	65.33	89.4
SCE1572_2524	26.01	27.97
SCE1572_8294	35.26	30.55
SCE1572_4533	23.11	5.85
SCE1572_10665	71.07	323.58
SCE1572_14	23.39	24.5

SCE1572_5578	22.74	0
SCE1572_9847	22	11.07
SCE1572_10377	57.59	12.73
SCE1572_9556	28.36	60.7
SCE1572_1473	45.62	21.53
SCE1572_11281	20.36	5.17
SCE1572_361	3.99	0
SCE1572_6749	10.7	4.29
SCE1572_3443	30.99	6.05
SCE1572_6552	0	0
SCE1572_4369	54.58	92.45
SCE1572_5603	37.43	58.02
SCE1572_2066	70	59.73
SCE1572_4568	12.78	2.12
SCE1572_10094	59.65	0
SCE1572_900	40.61	22.51
SCE1572_4871	31.59	30.41
SCE1572_10177	44.07	140.03
SCE1572_7046	12.63	7.6
SCE1572_1854	0	0
SCE1572_8528	48.15	82.05
SCE1572_8280	13.21	3.61
SCE1572_5700	7.43	0
SCE1572_5199	50.54	48.38
SCE1572_4364	49.52	19.98
SCE1572_2949	13.96	0
SCE1572_11079	178.17	1896.77
SCE1572_1182	0	0
SCE1572_7820	108.69	525.8
SCE1572_11286	59.68	126.45
SCE1572_5636	30.51	71.15
SCE1572_561	109.44	966.68
SCE1572_10396	16.32	14.73
SCE1572_1280	61.33	19.68
SCE1572_9521	0	0
SCE1572_11552	46.52	24.88
SCE1572_4920	30.8	5.7
SCE1572_6984	13.54	13.03
SCE1572_8267	71.32	85.34
SCE1572_7722	117.43	110.91
SCE1572_10926	0	0
SCE1572_10919	20.22	14.31
SCE1572_5662	14.15	34.06
SCE1572_556	13.24	4.78
SCE1572_11245	33.28	14.83
SCE1572_820	60.94	60.82
SCE1572_911	20.83	15.16
SCE1572_7259	0	0

SCE1572_5811	31.07	18.81
SCE1572_1902	108.97	32.87
SCE1572_1072	21.35	9.12
SCE1572_989	63.36	155.23
SCE1572_9543	46.16	19.46
SCE1572_3959	9.72	6.18
SCE1572_8238	63.33	465.39
SCE1572_2552	28.22	30.51
SCE1572_1529	17.12	13.96
SCE1572_10829	31.15	15
SCE1572_8221	25.7	7.17
SCE1572_3640	14.33	99.85
SCE1572_6613	65.56	45.37
SCE1572_11154	50.54	129.75
SCE1572_4916	50.44	479.74
SCE1572_280	61.97	31.86
SCE1572_4893	57.76	57.92
SCE1572_3086	49.38	15.07
SCE1572_10942	138.01	60.15
SCE1572_5113	5.67	6.21
SCE1572_9800	36.12	28.7
SCE1572_5769	331.14	2884.68
SCE1572_2675	15.98	100.17
SCE1572_2246	38.51	38.72
SCE1572_5313	20.67	9.22
SCE1572_1079	0	0
SCE1572_237	97.14	30.59
SCE1572_6783	13.55	53.01
SCE1572_3013	21.39	22.39
SCE1572_2925	60.98	31.64
SCE1572_6057	116.06	271.8
SCE1572_5387	36.41	52.44
SCE1572_9719	32.07	445.63
SCE1572_3905	124.23	675.19
SCE1572_1554	71.37	42.02
SCE1572_7750	31.82	4.51
SCE1572_10742	46.97	33.58
SCE1572_8964	41.59	82.35
SCE1572_5672	0	0
SCE1572_4792	25.27	4.05
SCE1572_8289	21.35	28.27
SCE1572_11357	50.54	26.44
SCE1572_10800	28.74	30.05
SCE1572_8860	40.64	11.29
SCE1572_8490	17.33	39.39
SCE1572_1325	32.82	7.52
SCE1572_5216	17.43	40.55
SCE1572_4308	0	0

SCE1572_2772	11.06	7.6
SCE1572_8720	10.16	10.19
SCE1572_3369	90.91	197.47
SCE1572_9137	13.54	16.29
SCE1572_5709	33.69	45.75
SCE1572_243	10.91	2.63
SCE1572_3226	53.58	20.52
SCE1572_11056	8.25	24.82
SCE1572_5548	32.72	13.31
SCE1572_2821	100.94	87.13
SCE1572_390	35.28	13.77
SCE1572_5538	45.25	60.51
SCE1572_5500	44.92	25.68
SCE1572_6469	31.18	7.5
SCE1572_2233	12.78	9.38
SCE1572_10157	27.34	42.54
SCE1572_616	33.78	125.55
SCE1572_8797	64.18	14.6
SCE1572_3270	25.2	172.86
SCE1572_7969	8.52	2.05
SCE1572_8257	65.49	183.32
SCE1572_8831	110.81	386.13
SCE1572_8665	42.36	16.1
SCE1572_669	28.94	17.86
SCE1572_5792	19.88	37.89
SCE1572_4820	27.51	33.11
SCE1572_9075	37.68	14.28
SCE1572_8341	29.16	24.56
SCE1572_4869	11.72	16.93
SCE1572_8729	11.54	15.87
SCE1572_2137	29.11	16.54
SCE1572_4544	38.12	31.28
SCE1572_2415	65.83	14.4
SCE1572_8699	44.46	21.4
SCE1572_5648	33.92	15.83
SCE1572_10813	38.93	54.25
SCE1572_9351	329.55	1299.85
SCE1572_439	251.3	56.14
SCE1572_2912	42.5	13.66
SCE1572_9411	55.31	295.59
SCE1572_3051	32.87	1.61
SCE1572_7699	17.84	15.74
SCE1572_8124	50.82	30.58
SCE1572_1919	45.36	34.37
SCE1572_2857	24.64	6.84
SCE1572_6772	15.47	0
SCE1572_5995	67.97	58.72
SCE1572_4181	12.95	14.79

SCE1572_8499	19.89	7.68
SCE1572_4550	40.18	14.63
SCE1572_11534	34.82	13.52
SCE1572_1824	22.31	33.84
SCE1572_4744	14.3	192.79
SCE1572_1829	236.36	860.14
SCE1572_10078	415.02	971.06
SCE1572_2402	29.71	120.82
SCE1572_5879	16.67	5.2
SCE1572_5920	28.61	62.54
SCE1572_3092	66.33	0
SCE1572_9458	60.29	70.42
SCE1572_5625	3.39	4.08
SCE1572_5273	9.6	4.62
SCE1572_10523	41.96	4.59
SCE1572_9469	104.21	188.11
SCE1572_8740	35.64	22.96
SCE1572_7115	24.5	4.91
SCE1572_5846	14.82	29.72
SCE1572_6638	36.27	17.72
SCE1572_507	41.16	13.21
SCE1572_4570	40.28	16.62
SCE1572_8738	52.88	40.55
SCE1572_9124	29.56	20.92
SCE1572_9699	29.32	23.15
SCE1572_140	10.11	8.69
SCE1572_7733	38.88	188.31
SCE1572_8886	26.66	10.15
SCE1572_11436	67.93	66.58
SCE1572_7629	27.52	82.45
SCE1572_6187	72.78	87.58
SCE1572_6151	22.52	4.54
SCE1572_8888	7.86	3.15
SCE1572_6608	16.85	62.66
SCE1572_5352	68.61	156.6
SCE1572_2672	44.9	58.95
SCE1572_10107	156.98	939.76
SCE1572_2381	54.98	165.57
SCE1572_3850	28.43	76.57
SCE1572_9076	36.98	19.07
SCE1572_11040	57.07	42.98
SCE1572_2719	39.73	27.68
SCE1572_10385	18.38	6.45
SCE1572_9885	33.97	58.06
SCE1572_1778	39.63	22.71
SCE1572_864	122.32	204.5
SCE1572_6147	21.87	7.26
SCE1572_7729	49.13	121.64

SCE1572_4512	0	0
SCE1572_6564	0	0
SCE1572_6116	50.28	19.96
SCE1572_3043	0	0
SCE1572_7023	0	0
SCE1572_150	28.57	20.32
SCE1572_9312	110.5	168.54
SCE1572_9151	32.05	11.87
SCE1572_1613	533.81	129.24
SCE1572_6031	41.3	40.7
SCE1572_2668	16.36	26.25
SCE1572_3698	42.28	42.11
SCE1572_2125	46.4	25.61
SCE1572_2150	21.06	4.34
SCE1572_9301	36.03	177.04
SCE1572_4754	49.44	70.52
SCE1572_6506	2.81	0
SCE1572_10499	22.39	484.93
SCE1572_4577	16.91	7.72
SCE1572_4356	24.92	20
SCE1572_2704	75.81	268.33
SCE1572_9604	10.34	1.46
SCE1572_2155	41.17	23.36
SCE1572_2958	74.34	49.01
SCE1572_8378	23.87	36.41
SCE1572_5429	46.59	90.72
SCE1572_1626	35.7	95.97
SCE1572_2275	126.99	825.49
SCE1572_4121	81.38	220.09
SCE1572_2397	56.6	1771.09
SCE1572_7065	60.65	59.14
SCE1572_893	35.41	72.11
SCE1572_4104	43.44	11.84
SCE1572_9819	54.25	16.74
SCE1572_1273	30.51	17.12
SCE1572_3453	31.42	13.7
SCE1572_11179	29.57	34.83
SCE1572_5380	15.04	4.22
SCE1572_1744	56.05	62.26
SCE1572_1758	67.02	20.33
SCE1572_2412	21.35	13.19
SCE1572_8135	20.71	6.15
SCE1572_60	47.34	39.29
SCE1572_9048	8.27	9.95
SCE1572_8925	1.26	0
SCE1572_6917	21.79	31.93
SCE1572_1783	59.82	141.5
SCE1572_8472	32.36	875.53

SCE1572_3343	140.48	330.66
SCE1572_1237	14.59	15.42
SCE1572_2425	164.33	310.57
SCE1572_1937	2.53	0
SCE1572_8467	33.14	8.49
SCE1572_1017	20.79	402.46
SCE1572_5168	48.05	135.6
SCE1572_5090	36.73	52.13
SCE1572_6021	76.83	101.09
SCE1572_7293	0	0
SCE1572_9772	28.04	72.48
SCE1572_4527	58.73	208.21
SCE1572_4862	3.32	0
SCE1572_10366	44.14	9.24
SCE1572_3971	26.32	21.32
SCE1572_7122	91.21	113.44
SCE1572_8650	32.15	12.55
SCE1572_2041	27.78	170.04
SCE1572_10961	41.92	100.89
SCE1572_3185	47.6	16.97
SCE1572_67	60.5	23.68
SCE1572_10252	0	0
SCE1572_10038	66.65	140.15
SCE1572_4428	16.95	29.47
SCE1572_9210	40.04	27.66
SCE1572_4770	53.51	161
SCE1572_8552	102.84	176.11
SCE1572_2632	48.22	20.99
SCE1572_10063	625.56	4775.6
SCE1572_2804	44.48	35.82
SCE1572_5108	132.3	126.81
SCE1572_10898	9.52	64.13
SCE1572_5420	33.43	70.4
SCE1572_3364	36.44	67.35
SCE1572_8442	9.78	10.46
SCE1572_10906	2.9	0
SCE1572_6514	11.02	2.79
SCE1572_3994	89.93	36.67
SCE1572_11201	53.39	123.66
SCE1572_6054	22.06	70.39
SCE1572_5039	18.19	48.66
SCE1572_2910	48.88	18.52
SCE1572_6449	190.51	57.12
SCE1572_11480	82.97	107.78
SCE1572_8130	43.47	21.69
SCE1572_10902	35.11	30.73
SCE1572_11066	23.8	21.92
SCE1572_4808	24.82	143.36

SCE1572_3687	62.8	15.03
SCE1572_8334	0	0
SCE1572_3793	54.66	135.27
SCE1572_7593	46.63	47.01
SCE1572_6258	7.11	8.55
SCE1572_3426	69.84	22.99
SCE1572_1535	22.49	44.11
SCE1572_11356	31.59	66.52
SCE1572_8676	57.12	62.96
SCE1572_3822	20.99	44.91
SCE1572_10642	52.91	217.95
SCE1572_9052	55.69	56.77
SCE1572_3232	30.53	91.02
SCE1572_1468	0	0
SCE1572_921	29.72	15.23
SCE1572_4643	31.94	25.62
SCE1572_2850	44.83	206
SCE1572_2986	0	0
SCE1572_10005	12.2	12.58
SCE1572_4829	13.82	60.38
SCE1572_9428	152.45	257.65
SCE1572_5799	29.82	24.33
SCE1572_1698	24.32	175.58
SCE1572_3765	32.71	38.74
SCE1572_3587	310.28	277.23
SCE1572_9251	57.15	28.07
SCE1572_4028	84.81	158.98
SCE1572_8158	72.47	18.36
SCE1572_10453	22.8	15.55
SCE1572_7796	44.99	29.16
SCE1572_2602	67.25	21.02
SCE1572_4836	39.65	123.51
SCE1572_256	23.58	4.05
SCE1572_4420	0	0
SCE1572_11143	37.14	43.97
SCE1572_9987	78.79	25.63
SCE1572_6814	27.49	36.09
SCE1572_3945	396.81	795.06
SCE1572_8206	38.46	71.05
SCE1572_2963	65.37	812.15
SCE1572_7852	0	0
SCE1572_6158	12.42	10.73
SCE1572_1794	20.09	568.63
SCE1572_3577	39.68	81.25
SCE1572_2606	43.89	6.4
SCE1572_10793	8.64	5.67
SCE1572_3134	19.05	7.64
SCE1572_9397	28.23	56.29

SCE1572_1649	38.34	11.99
SCE1572_6373	46.92	30.3
SCE1572_1664	25.35	70.59
SCE1572_8714	92.37	150.1
SCE1572_7688	76.63	18.21
SCE1572_1053	41.17	85.11
SCE1572_7937	8.21	5.2
SCE1572_3841	70.11	34.8
SCE1572_9439	31.46	39.37
SCE1572_2082	36.86	40.32
SCE1572_10822	46.8	46.55
SCE1572_10537	0	0
SCE1572_11062	49.35	28.8
SCE1572_3984	31.15	96.23
SCE1572_6842	28.84	21.03
SCE1572_6559	50.01	74.26
SCE1572_2290	311.92	1957.1
SCE1572_11310	36.26	11.9
SCE1572_3002	7.26	3.88
SCE1572_1883	0	0
SCE1572_5379	42.47	14.23
SCE1572_4777	35.47	139.48
SCE1572_7459	172.01	420.41
SCE1572_5980	440.23	298.97
SCE1572_5763	0	0
SCE1572_11463	91.29	169.23
SCE1572_11573	0	0
SCE1572_325	21.34	5.41
SCE1572_7539	68.21	20.79
SCE1572_2445	20.05	7.62
SCE1572_3967	118.49	78.39
SCE1572_4559	11.58	34.21
SCE1572_1536	62.4	34.25
SCE1572_4636	111.72	22.41
SCE1572_7683	17.84	4.91
SCE1572_7144	48.84	105.31
SCE1572_9646	98.91	132.36
SCE1572_7783	0	0
SCE1572_5187	18.78	1332.13
SCE1572_2353	6.51	20.15
SCE1572_4242	18.01	3.61
SCE1572_1984	0	0
SCE1572_10408	38.97	56.27
SCE1572_9822	12.86	8.85
SCE1572_10685	12.68	9.46
SCE1572_11135	38.48	45.38
SCE1572_6530	25.77	33.76
SCE1572_8196	27.39	15.33

SCE1572_2648	49.15	24.06
SCE1572_2378	39.83	507.18
SCE1572_1402	55.96	17.68
SCE1572_1708	36.1	0
SCE1572_2171	111.94	58.48
SCE1572_7247	0	0
SCE1572_1496	20.13	9.69
SCE1572_7252	33.11	10.19
SCE1572_1147	52.64	69.72
SCE1572_2953	20.09	0
SCE1572_2204	29.13	205.51
SCE1572_8657	9.78	133.41
SCE1572_4171	52.51	60.25
SCE1572_5486	77.65	19.01
SCE1572_11122	37.24	10.73
SCE1572_2189	29.03	80.23
SCE1572_2835	12.23	0
SCE1572_2210	42.34	227.11
SCE1572_4396	15.29	21.47
SCE1572_1491	0	0
SCE1572_1497	32.78	13.12
SCE1572_11489	0	0
SCE1572_681	16.01	7.23
SCE1572_3297	8.94	6.59
SCE1572_4139	12.48	4.51
SCE1572_5158	7.84	0
SCE1572_9829	22.01	0
SCE1572_4903	46.72	70.53
SCE1572_3215	147.14	480.98
SCE1572_302	16.53	5.19
SCE1572_8791	80.27	400.7
SCE1572_4326	24.68	15.76
SCE1572_4271	30.44	25.78
SCE1572_735	79.37	40.41
SCE1572_6882	0	0
SCE1572_1955	15.08	18.15
SCE1572_3009	15.9	23.54
SCE1572_4038	48.83	58.35
SCE1572_5070	23.13	35.05
SCE1572_7619	69.98	338.13
SCE1572_5016	73.45	1054.07
SCE1572_9981	51.01	29.72
SCE1572_1464	45.53	19.57
SCE1572_5290	52.43	21.51
SCE1572_4668	7.98	4.8
SCE1572_2589	41.43	70.46
SCE1572_1295	65.84	37.53
SCE1572_10199	73.69	73.1

SCE1572_663	0	0
SCE1572_3352	21.09	8.92
SCE1572_1764	39.71	12.16
SCE1572_2106	87.86	241.13
SCE1572_11315	259.58	460.76
SCE1572_8867	47.15	24.5
SCE1572_10697	75.16	167.47
SCE1572_9838	0	0
SCE1572_787	66.41	280.74
SCE1572_1900	13.35	6.89
SCE1572_135	33.55	18.88
SCE1572_8516	83.27	13.24
SCE1572_11583	44.84	118.44
SCE1572_7865	28.34	17.05
SCE1572_1023	23.69	17.11
SCE1572_8097	25.08	15.32
SCE1572_11101	37.49	44.11
SCE1572_717	18.14	6.98
SCE1572_8392	0	0
SCE1572_7194	0	0
SCE1572_2780	49.17	6.58
SCE1572_10882	40.11	303.6
SCE1572_10324	21.1	32.65
SCE1572_5282	94.71	113.49
SCE1572_3645	209.13	424.24
SCE1572_1802	43.82	44.3
SCE1572_8877	3.09	0
SCE1572_2885	25.93	15.01
SCE1572_8306	124.05	8.29
SCE1572_6497	40.91	7.72
SCE1572_8173	22.26	5.79
SCE1572_4605	121.41	819.98
SCE1572_9266	26.87	47.79
SCE1572_1258	37.9	3.65
SCE1572_10336	11.84	9.5
SCE1572_3074	27.5	11.88
SCE1572_6306	16.3	9.51
SCE1572_3491	29.41	54.01
SCE1572_1517	3.3	0
SCE1572_3773	59.26	24.59
SCE1572_7948	128.14	207.18
SCE1572_440	173.68	56.55
SCE1572_9897	9.33	7.67
SCE1572_10446	11.81	7.48
SCE1572_10772	32.88	30.78
SCE1572_8068	42.78	11.8
SCE1572_7643	92.65	115.85
SCE1572_5561	47.15	23.29

SCE1572_11006	0	0
SCE1572_7005	10.78	3.24
SCE1572_8544	76.47	60.29
SCE1572_1041	57.61	24.52
SCE1572_6676	48.27	31.09
SCE1572_4226	29.96	48.56
SCE1572_11561	35.09	58.67
SCE1572_3595	20.29	2.76
SCE1572_9292	22.12	42.03
SCE1572_7915	2.53	0
SCE1572_4661	12.63	6.61
SCE1572_3103	20.04	8.39
SCE1572_2360	69.56	450.79
SCE1572_9812	52.02	17.54
SCE1572_2735	26.56	20.66
SCE1572_3709	0	0
SCE1572_7784	13.75	66.79
SCE1572_7103	0	0
SCE1572_10087	0	0
SCE1572_11021	34.77	26.34
SCE1572_9503	582.37	1943.03
SCE1572_3731	25.15	11.08
SCE1572_2327	63.75	34.27
SCE1572_7437	43.76	14.09
SCE1572_168	20.2	8.38
SCE1572_9867	37.38	75.81
SCE1572_6718	0	0
SCE1572_8838	0	0
SCE1572_6218	37.35	12.84
SCE1572_9323	26.86	42.4
SCE1572_8995	22.56	9.77
SCE1572_3897	27.21	42.63
SCE1572_6803	38.45	61.11
SCE1572_9394	11.74	2.42
SCE1572_8746	25.66	28.07
SCE1572_3062	18.53	4.05
SCE1572_230	109.04	47.49
SCE1572_10307	20.22	4.42
SCE1572_11418	69.19	62.95
SCE1572_4644	49.39	36.24
SCE1572_456	35.13	11.87
SCE1572_9569	0	0
SCE1572_8459	43.32	49.46
SCE1572_8365	39.2	17.8
SCE1572_4198	20.67	0
SCE1572_10020	46.39	19.64
SCE1572_9633	45.4	14.28
SCE1572_5580	31.66	32.08

SCE1572_5772	24.43	15.87
SCE1572_2488	4.27	0
SCE1572_10761	0	0
SCE1572_371	19.69	194.31
SCE1572_2300	26.87	139.73
SCE1572_8258	37.38	224.05
SCE1572_9448	42.05	28.51
SCE1572_4622	79.68	1877.88
SCE1572_6355	0	0
SCE1572_11495	120.27	22.81
SCE1572_937	13.46	10.23
SCE1572_11432	24.29	19.15
SCE1572_9012	32.8	28.47
SCE1572_2314	35.92	59.76
SCE1572_917	17.91	46.34
SCE1572_5479	45.24	21.54
SCE1572_4888	22.24	14.15
SCE1572_2591	40.73	23.97
SCE1572_3379	68.72	19.61
SCE1572_9513	18.21	16.31
SCE1572_384	0	0
SCE1572_10785	48.08	76.46
SCE1572_7800	52.27	101.65
SCE1572_10808	8.6	0
SCE1572_843	55.34	299.42
SCE1572_603	11.66	4.16
SCE1572_4960	27.09	47.87
SCE1572_1488	0	0
SCE1572_6395	0	0
SCE1572_9656	41.09	22.29
SCE1572_6000	37.9	125.2
SCE1572_8688	154.01	241.36
SCE1572_3370	17.44	271.27
SCE1572_11256	28.71	48.58
SCE1572_9716	20.37	187.91
SCE1572_414	43.22	31.37
SCE1572_6691	2.33	0
SCE1572_8628	47.46	132.02
SCE1572_8342	29.62	16.42
SCE1572_9709	51.73	83.49
SCE1572_1639	25.95	39.45
SCE1572_464	40.53	415.51
SCE1572_6209	27.69	4.57
SCE1572_7871	19.51	13.09
SCE1572_7765	35.91	32.01
SCE1572_5363	16.18	4.71
SCE1572_3073	16.7	6.43
SCE1572_55	73.31	23.11

SCE1572_7602	63.91	56.22
SCE1572_1427	3.45	0
SCE1572_5297	53.16	61.61
SCE1572_7922	33.33	19.57
SCE1572_6071	0	0
SCE1572_7480	45.41	66.99
SCE1572_3906	87.52	94.55
SCE1572_3487	27.17	6.99
SCE1572_260	57.68	14.93
SCE1572_10541	56.17	58.63
SCE1572_6202	28.99	19.77
SCE1572_5845	45.71	42.18
SCE1572_573	207.66	389.75
SCE1572_4478	26.63	32.04
SCE1572_8694	24.87	10.69
SCE1572_3873	12.71	10.67
SCE1572_10433	20.05	15.08
SCE1572_10972	22.25	93.91
SCE1572_4166	40.61	19.55
SCE1572_9271	28.43	45.62
SCE1572_1265	305.7	11633.01
SCE1572_10077	380.89	893.02
SCE1572_7523	34.41	15.31
SCE1572_9338	28.31	41.77
SCE1572_4282	31.04	2.87
SCE1572_2540	46.95	25.11
SCE1572_7904	14.52	5.18
SCE1572_8543	52.96	1087.57
SCE1572_6697	16.22	74.16
SCE1572_10313	48.29	22.98
SCE1572_9199	19.84	3.67
SCE1572_9670	43.79	154.11
SCE1572_7559	42.46	28.81
SCE1572_10994	37.25	53.48
SCE1572_6862	24.74	7.58
SCE1572_6976	42.94	13.54
SCE1572_10339	40.56	25.57
SCE1572_10725	17.1	7.55
SCE1572_2247	38.55	12.37
SCE1572_11279	31.22	128.8
SCE1572_5328	50.64	42.85
SCE1572_2	28.63	53.01
SCE1572_519	41.96	43.61
SCE1572_1306	220.18	8947.52
SCE1572_7465	125.05	207.41
SCE1572_468	22.44	71.66
SCE1572_1609	20.1	18.67
SCE1572_9821	65.51	27.97

SCE1572_5498	25.34	23.72
SCE1572_6235	30.96	32.12
SCE1572_2260	26.41	26.65
SCE1572_1004	18.65	18.51
SCE1572_10341	35.93	13.19
SCE1572_1645	63.69	116.67
SCE1572_10050	63.33	124.05
SCE1572_7411	40.08	29.36
SCE1572_2539	21.06	25.8
SCE1572_6203	31.98	160.57
SCE1572_4010	30.06	14.47
SCE1572_3249	43.2	61.99
SCE1572_8263	40.78	124.28
SCE1572_2188	184.21	334.23
SCE1572_10032	23.15	68.95
SCE1572_8102	15.39	77.34
SCE1572_118	184.04	849.19
SCE1572_7340	121.62	258.6
SCE1572_5461	24.59	5.92
SCE1572_8599	0	0
SCE1572_3172	20.73	11.75
SCE1572_624	3.3	0
SCE1572_4825	100.61	111.5
SCE1572_3475	19.06	46.89
SCE1572_10522	13.56	20.77
SCE1572_11397	6.19	0
SCE1572_4584	88.74	32.1
SCE1572_11138	9.6	0
SCE1572_11392	65.21	133.41
SCE1572_3206	72.41	60.66
SCE1572_9392	33.21	128.18
SCE1572_1963	45.43	17.8
SCE1572_10947	3.79	3.04
SCE1572_3482	39.63	9.98
SCE1572_1681	32.16	296.73
SCE1572_5780	35.77	51.4
SCE1572_11190	0	0
SCE1572_3801	107.83	70.25
SCE1572_8760	82.8	84.83
SCE1572_8159	5.88	0
SCE1572_7380	45.55	18.79
SCE1572_5032	79.32	791.51
SCE1572_10823	54.23	40.02
SCE1572_6825	0	0
SCE1572_9572	12.79	17.59
SCE1572_8610	48.65	27.32
SCE1572_3405	53.99	24.95
SCE1572_4358	54.62	18.93

SCE1572_5684	58.45	178.06
SCE1572_10774	130.94	497.62
SCE1572_8704	11.44	41.31
SCE1572_7976	19.85	5.08
SCE1572_10584	157.31	660.38
SCE1572_9865	27.12	25.54
SCE1572_10957	40.11	49.09
SCE1572_4495	35.07	24.62
SCE1572_227	13.09	6.52
SCE1572_5430	107.68	134.11
SCE1572_9630	44.22	15.21
SCE1572_4515	51.98	45.18
SCE1572_252	0	0
SCE1572_8174	10.53	2.53
SCE1572_6859	17.69	4.56
SCE1572_1943	41.95	24.33
SCE1572_220	42.96	93.66
SCE1572_2726	73.68	1023.15
SCE1572_6163	30.4	10.73
SCE1572_3593	37.15	15.99
SCE1572_7003	67.61	16.91
SCE1572_10015	26.62	67.59
SCE1572_3982	38.54	97.86
SCE1572_11475	32.76	19.36
SCE1572_1288	104.57	104.04
SCE1572_21	46.93	26.07
SCE1572_1941	104.6	17.8
SCE1572_7013	20.92	0
SCE1572_2053	61.81	58.11
SCE1572_6690	36.45	63.81
SCE1572_3830	30.07	144.76
SCE1572_2530	0	0
SCE1572_2268	21.9	10.67
SCE1572_3037	30.61	12.87
SCE1572_11593	69.05	514.87
SCE1572_9956	42.62	25.23
SCE1572_3194	51.06	63.79
SCE1572_10183	69.98	282.19
SCE1572_8730	78.97	54.3
SCE1572_10876	51.14	46.07
SCE1572_11164	21.16	28.29
SCE1572_5374	54.09	53.23
SCE1572_9574	15.96	3.84
SCE1572_10010	31.3	21.19
SCE1572_3524	27.97	23.28
SCE1572_3307	111.16	2481.68
SCE1572_6078	2.78	0
SCE1572_4347	51.94	20.27

SCE1572_2512	45.21	989.31
SCE1572_972	63.17	170.95
SCE1572_9232	3.49	0
SCE1572_3125	42.05	47.95
SCE1572_8216	25.57	13.03
SCE1572_6010	165.24	1795.91
SCE1572_7415	11.23	3.38
SCE1572_5530	99.89	52.13
SCE1572_3766	22.92	86.15
SCE1572_7079	20.22	236.33
SCE1572_8209	38.64	40.24
SCE1572_2389	13.71	23.29
SCE1572_5577	28.43	0
SCE1572_777	11.71	2.25
SCE1572_1733	70.87	31.16
SCE1572_7676	37.9	5.37
SCE1572_10756	28.2	23.52
SCE1572_9799	6.89	6.63
SCE1572_504	14.15	6.49
SCE1572_7307	17.63	3.39
SCE1572_7082	28.45	16.34
SCE1572_5123	23.92	7.43
SCE1572_1267	0	0
SCE1572_10049	55.33	48.83
SCE1572_958	19.43	22.81
SCE1572_6458	51.4	47.82
SCE1572_5518	15.23	5.72
SCE1572_8645	0	0
SCE1572_11151	28.21	19.8
SCE1572_4388	24.97	17.17
SCE1572_6092	41.4	4.8
SCE1572_1223	21.66	52.13
SCE1572_6553	0	0
SCE1572_10682	11.87	11.64
SCE1572_8229	9.71	5.56
SCE1572_5408	31.92	24.33
SCE1572_1913	0	0
SCE1572_8125	186.4	30.05
SCE1572_7878	29.16	14.04
SCE1572_6594	68.82	106.61
SCE1572_9252	5.95	3.58
SCE1572_2749	59.96	186.91
SCE1572_91	258.42	2622.77
SCE1572_1877	0	0
SCE1572_6268	63.22	34.64
SCE1572_6180	35.4	19.18
SCE1572_6816	64.16	20.59
SCE1572_9097	10	4.01

SCE1572_7253	44.9	15.94
SCE1572_5349	77.69	15.08
SCE1572_4650	58.77	152.15
SCE1572_2930	49.17	14.25
SCE1572_5639	11.46	5.3
SCE1572_10950	41.92	18.5
SCE1572_11018	0	0
SCE1572_3234	47.3	27.66
SCE1572_2475	50.13	42.45
SCE1572_9538	82.77	21.7
SCE1572_3782	22.03	51.25
SCE1572_8935	9.76	5.87
SCE1572_6317	0	0
SCE1572_6940	17.1	21.95
SCE1572_6017	86.3	296.43
SCE1572_7621	24.83	16.01
SCE1572_3418	32.07	26.51
SCE1572_6259	14.95	12.85
SCE1572_9375	0	0
SCE1572_6261	15.92	5.47
SCE1572_7243	38.42	62.6
SCE1572_1620	54.58	300.7
SCE1572_8809	81.85	12.92
SCE1572_2185	81.59	24.55
SCE1572_3692	24.41	23.73
SCE1572_10083	192.46	868.37
SCE1572_1676	20.33	46.52
SCE1572_10460	45.35	141.26
SCE1572_2939	17.84	54.64
SCE1572_9790	99.41	175.11
SCE1572_8013	23.4	64.9
SCE1572_714	30.86	3.23
SCE1572_6067	13.66	11.75
SCE1572_4003	53.7	144.45
SCE1572_9531	22.53	33.33
SCE1572_690	41.78	30.17
SCE1572_2639	67.02	31.32
SCE1572_5912	37.37	67.45
SCE1572_11580	36.98	1.78
SCE1572_8523	38.44	0
SCE1572_9794	20.12	22.46
SCE1572_6823	55.86	180.54
SCE1572_2058	6.15	0
SCE1572_9108	47.51	65.69
SCE1572_4435	24.51	39.33
SCE1572_3933	103.28	262.14
SCE1572_9616	2.92	0
SCE1572_1939	28.08	96.65

SCE1572_705	20.57	21.3
SCE1572_7734	16.58	0
SCE1572_5784	56.48	145.97
SCE1572_4436	34.47	25.31
SCE1572_4945	24.11	56.45
SCE1572_3995	17.11	16.84
SCE1572_11587	1.97	4.75
SCE1572_9926	78.39	779.49
SCE1572_5903	17.19	13.79
SCE1572_5271	24.95	13.86
SCE1572_9802	23.42	20.03
SCE1572_7705	0	0
SCE1572_1543	539.61	367.8
SCE1572_8349	77.2	6.72
SCE1572_10017	66.61	282.37
SCE1572_3918	25.91	73.91
SCE1572_1634	129.32	692.95
SCE1572_2580	0	0
SCE1572_6482	79.17	65.5
SCE1572_8895	44.21	48.36
SCE1572_6982	12.43	0
SCE1572_73	34.21	142.09
SCE1572_11338	70.04	8.54
SCE1572_6750	22.04	16.97
SCE1572_1560	2.49	0
SCE1572_1546	64.88	31.01
SCE1572_2873	90.31	65.81
SCE1572_9756	13.78	8.29
SCE1572_3753	78.19	292.92
SCE1572_550	0	0
SCE1572_11365	15.79	7.6
SCE1572_9246	0	0
SCE1572_11268	40.64	47.98
SCE1572_851	60.74	183.33
SCE1572_3635	27.39	16.48
SCE1572_9	26.32	33.05
SCE1572_6505	36.21	30.22
SCE1572_4681	21.31	13.81
SCE1572_5572	28.03	14.47
SCE1572_1376	26.83	15.31
SCE1572_6791	6.67	6.89
SCE1572_11514	53.27	36.99
SCE1572_6955	47.77	12.35
SCE1572_1931	46.31	16.46
SCE1572_10042	474.9	1500.24
SCE1572_1162	38.3	12.8
SCE1572_5732	65.16	208.09
SCE1572_10988	59.85	787.46

SCE1572_5953	49.51	31.64
SCE1572_7719	65.32	28.85
SCE1572_430	56.43	39.82
SCE1572_11036	13.38	136.85
SCE1572_10037	21.23	45.25
SCE1572_10631	36.03	0
SCE1572_1687	38.61	13.6
SCE1572_1714	70.05	57.74
SCE1572_5308	31.15	44.99
SCE1572_8874	110.44	25.95
SCE1572_5211	47.63	68.97
SCE1572_9383	592.45	2352.03
SCE1572_1576	35.96	16.95
SCE1572_11557	35.02	20.9
SCE1572_8435	70.97	3.88
SCE1572_6888	39.76	11.48
SCE1572_11205	3.74	13.52
SCE1572_1344	11.13	6.35
SCE1572_3721	2.57	0
SCE1572_10449	20.57	97
SCE1572_368	45.46	50.97
SCE1572_9970	8.58	4.92
SCE1572_6990	2.3	0
SCE1572_1159	44.13	18.75
SCE1572_7974	29.97	28.85
SCE1572_5058	22.66	9.99
SCE1572_2346	37.9	687.41
SCE1572_10367	16.51	8.64
SCE1572_276	43.49	22.63
SCE1572_9320	34.43	45.77
SCE1572_7537	7.46	1.99
SCE1572_45	27.06	120.86
SCE1572_1840	20.67	42.39
SCE1572_1360	62.14	194.21
SCE1572_9698	21.75	8.47
SCE1572_8950	59.03	24.53
SCE1572_10700	0	0
SCE1572_2362	36.63	58.57
SCE1572_11438	52.54	33.24
SCE1572_5022	50.7	242.89
SCE1572_3331	33.69	337.89
SCE1572_4207	49.4	135.31
SCE1572_10602	75.31	36.97
SCE1572_183	149.84	366.14
SCE1572_160	68.57	37.7
SCE1572_11057	35.08	18.1
SCE1572_6374	40.16	11.24
SCE1572_6914	14.93	11.06

SCE1572_5562	94.99	48.36
SCE1572_7485	105.29	284.5
SCE1572_7450	9.86	50.44
SCE1572_6892	36.27	37.43
SCE1572_6522	72.27	122.78
SCE1572_6422	35.21	44.73
SCE1572_9611	53.44	8.97
SCE1572_4264	31.33	10.56
SCE1572_5167	0	0
SCE1572_6675	45.31	16.78
SCE1572_10135	19.87	16.36
SCE1572_1245	6.04	4.85
SCE1572_8829	0	0
SCE1572_7196	2.68	0
SCE1572_8323	36.03	42.55
SCE1572_7810	113.28	209.31
SCE1572_5160	28.11	20.5
SCE1572_3547	0	0
SCE1572_4921	7.07	9.28
SCE1572_9327	265.63	580.96
SCE1572_4692	19.18	18.65
SCE1572_4373	23.94	25.99
SCE1572_5611	6.67	4.59
SCE1572_4064	0	0
SCE1572_7806	38.14	34.43
SCE1572_4289	43.62	66.58
SCE1572_2292	102.28	124.54
SCE1572_9764	35.43	33.96
SCE1572_443	29.82	8.09
SCE1572_10656	74.74	143.06
SCE1572_3155	91.57	184.27
SCE1572_2382	9.34	3.46
SCE1572_4908	59.65	105.12
SCE1572_1213	0	0
SCE1572_6733	37.9	231.59
SCE1572_6427	12.26	10.73
SCE1572_5433	47.38	10.53
SCE1572_6997	23.23	11.77
SCE1572_5086	163.28	50.53
SCE1572_8419	8.92	0
SCE1572_5286	89.09	35.6
SCE1572_1320	15.09	36.33
SCE1572_812	0	0
SCE1572_7072	58.44	87.61
SCE1572_3256	31.14	16.29
SCE1572_4688	0	0
SCE1572_6297	12.2	88.08
SCE1572_11287	80.79	466.14

SCE1572_10411	27.73	28.93
SCE1572_10206	70.75	31.47
SCE1572_5134	20.22	43.79
SCE1572_8605	75.42	102.1
SCE1572_6363	39.7	333.07
SCE1572_10269	11.53	6.94
SCE1572_3519	15.72	11.13
SCE1572_2654	24.26	9.73
SCE1572_429	41.11	66.35
SCE1572_2758	61.09	183.23
SCE1572_653	527.66	1742.14
SCE1572_6600	36.93	33.99
SCE1572_9999	24.5	238.09
SCE1572_8006	5.14	0
SCE1572_7667	190.47	40.24
SCE1572_786	12.79	4.4
SCE1572_9967	36.44	24.19
SCE1572_403	19.61	34.47
SCE1572_6439	26.8	20.37
SCE1572_1974	0	0.69
SCE1572_8924	0	0
SCE1572_7387	54.4	98.2
SCE1572_10628	22.8	67.84
SCE1572_8908	22.22	12.58
SCE1572_8469	136.63	163.37
SCE1572_448	20.14	21.15
SCE1572_10488	97.42	1263.39
SCE1572_10108	23.04	68.32
SCE1572_4147	23.18	1385.31
SCE1572_5939	32.53	8.41
SCE1572_10192	17.58	5.29
SCE1572_1489	36.87	10
SCE1572_10097	18.08	118.85
SCE1572_7571	26.26	101.05
SCE1572_943	112.48	34.09
SCE1572_9807	297.4	1178.98
SCE1572_1926	100.79	762.23
SCE1572_113	35.65	28.29
SCE1572_8109	45.05	26.44
SCE1572_3288	31.93	72.98
SCE1572_4228	16.41	9.41
SCE1572_6729	8.42	28.38
SCE1572_637	14.19	34.16
SCE1572_6713	28.14	48.38
SCE1572_5767	112.2	681.88
SCE1572_8959	0	0
SCE1572_2164	6.45	3.88
SCE1572_2795	44.38	62.3

SCE1572_955	0	0
SCE1572_11505	191.13	1191.82
SCE1572_7290	13.54	6.52
SCE1572_6020	118.79	160.91
SCE1572_8273	24.7	43.05
SCE1572_3459	3913.89	1122.72
SCE1572_932	0	0
SCE1572_3837	52.12	21.99
SCE1572_11297	55.55	77.55
SCE1572_3979	32.74	47.69
SCE1572_10090	49.26	98.68
SCE1572_10503	9.83	28.72
SCE1572_377	85.7	33.28
SCE1572_9898	36.62	8.81
SCE1572_3586	28.04	22.5
SCE1572_457	13.9	17.53
SCE1572_10126	4.15	0
SCE1572_8430	51.93	98.53
SCE1572_10044	675.22	2732.67
SCE1572_4059	11.08	12.63
SCE1572_10668	0	0
SCE1572_9110	16.36	14.6
SCE1572_1096	52.89	99.72
SCE1572_7220	45.08	1134.22
SCE1572_4094	10.06	5.85
SCE1572_8491	24.62	19.96
SCE1572_343	47.11	49.45
SCE1572_10616	34.99	53.02
SCE1572_7651	76.85	24.99
SCE1572_5945	24.67	18.1
SCE1572_7779	14.52	7.76
SCE1572_3016	1.7	0
SCE1572_9404	22.89	216.89
SCE1572_7227	117.63	3768.77
SCE1572_5540	15.11	34.03
SCE1572_4417	15.55	12.48
SCE1572_9700	44.43	49.8
SCE1572_5184	17.7	1257.56
SCE1572_1024	37.49	10.03
SCE1572_1349	18.72	279.32
SCE1572_5547	39.92	11.24
SCE1572_3094	23.04	48.8
SCE1572_11012	51.74	62.81
SCE1572_1046	20.9	14.49
SCE1572_6384	34.37	27.98
SCE1572_8697	2.57	0
SCE1572_3027	54.06	106.19
SCE1572_9845	66.92	12.58

SCE1572_3384	66.02	270.65
SCE1572_968	99.52	510.89
SCE1572_2035	0	0
SCE1572_4563	7.22	13.03
SCE1572_10852	84.28	52.92
SCE1572_8083	13.99	9.43
SCE1572_1131	14.8	3.69
SCE1572_7891	98.96	3730.07
SCE1572_524	16.8	21.28
SCE1572_4716	37.37	17.99
SCE1572_3503	23.59	38.91
SCE1572_11124	25.63	3.43
SCE1572_7906	50.54	22.22
SCE1572_8316	49.22	65.92
SCE1572_4073	51.07	552.8
SCE1572_4718	30.18	22.48
SCE1572_6850	10.21	7.56
SCE1572_1581	29.92	38.41
SCE1572_9346	46.35	56.57
SCE1572_8480	24.38	90.02
SCE1572_1015	13.8	5.07
SCE1572_10326	31.73	11.32
SCE1572_2752	33.33	65.57
SCE1572_5962	0	0
SCE1572_3758	51.8	26.32
SCE1572_8066	154.01	56.69
SCE1572_6285	43.22	48.02
SCE1572_2456	431.95	357.47
SCE1572_1150	121.29	1426.57
SCE1572_3662	20.14	0.95
SCE1572_1185	41.65	9.01
SCE1572_740	8.86	2.37
SCE1572_3970	31.22	76.66
SCE1572_10440	52.64	5.07
SCE1572_7159	37.17	15.62
SCE1572_8246	36.24	89.51
SCE1572_706	10.42	2.28
SCE1572_3109	70.38	16.73
SCE1572_7123	11.54	3.97
SCE1572_5650	2.92	0
SCE1572_4647	0	0
SCE1572_10110	35.67	118.6
SCE1572_1507	67.23	24.77
SCE1572_750	27.6	9.02
SCE1572_10167	21.35	28.98
SCE1572_7564	22.79	7.62
SCE1572_5335	38.39	83.7
SCE1572_1433	0	0

SCE1572_8147	39.55	18.63
SCE1572_4995	35.89	72.28
SCE1572_3262	11.66	15.49
SCE1572_7282	45.6	31.55
SCE1572_8653	22.09	16.31
SCE1572_3610	0	0
SCE1572_7496	0	0
SCE1572_9946	34.99	0
SCE1572_2991	30.36	22.14
SCE1572_5226	24.43	59.81
SCE1572_5383	45.2	16.62
SCE1572_6686	9.48	9.12
SCE1572_4050	74.9	195.29
SCE1572_7446	21.16	0
SCE1572_4363	86.45	19.21
SCE1572_3658	8.6	2.3
SCE1572_9942	36.74	20.15
SCE1572_7324	0	0
SCE1572_152	36.22	38.41
SCE1572_7354	51.87	15.14
SCE1572_6807	21.57	14.83
SCE1572_8815	4.46	0
SCE1572_3429	25.4	5.15
SCE1572_5303	14.94	9.32
SCE1572_7187	36.04	24.14
SCE1572_3437	25.99	9.12
SCE1572_7273	1.78	0
SCE1572_5569	40.86	29.62
SCE1572_9904	24.9	19.39
SCE1572_858	806.81	26391.7
SCE1572_4985	9.02	0
SCE1572_2786	16.93	59.28
SCE1572_4722	52.52	122.01
SCE1572_6654	84.9	390.17
SCE1572_11145	70.61	91.23
SCE1572_8386	86.21	40.16
SCE1572_792	138.88	205.46
SCE1572_1408	15.29	1.99
SCE1572_10235	32.88	16.96
SCE1572_6625	22.61	25.61
SCE1572_5746	24.78	51.83
SCE1572_4134	64.49	21.89
SCE1572_8009	364.94	21246.12
SCE1572_2951	0	0
SCE1572_4702	101.27	49.73
SCE1572_1986	45.44	628.86
SCE1572_9084	20.57	47.38
SCE1572_5412	14.31	8.79

SCE1572_7403	67.76	2009.33
SCE1572_3250	178.09	379.4
SCE1572_1018	26.86	139.73
SCE1572_9494	649.64	725.01
SCE1572_8477	61.51	29.39
SCE1572_683	49.31	35.6
SCE1572_584	26.47	126.49
SCE1572_5444	38.21	8.76
SCE1572_5189	16.51	261.95
SCE1572_514	69.92	56.72
SCE1572_1319	21.79	8.36
SCE1572_5132	42.94	85.04
SCE1572_8640	42.94	216.91
SCE1572_5659	38.41	46.98
SCE1572_1418	68.68	96.69
SCE1572_2496	17.07	4.83
SCE1572_4704	85.66	22.68
SCE1572_1833	38.32	28.62
SCE1572_6337	45.33	68.97
SCE1572_9743	153.51	1043.45
SCE1572_3026	17.68	22.75
SCE1572_6755	55.02	35.32
SCE1572_10679	182.32	954.27
SCE1572_7993	21.97	7.05
SCE1572_5006	76.64	496.25
SCE1572_11457	122.63	137.47
SCE1572_1746	58.2	36.67
SCE1572_5712	26.76	4.77
SCE1572_6899	25.89	126.09
SCE1572_8962	31.1	22.46
SCE1572_8150	27.96	8.92
SCE1572_5320	34.36	90.57
SCE1572_10767	31.07	13.82
SCE1572_8062	0	0
SCE1572_6273	13.88	21.62
SCE1572_999	41.84	37.07
SCE1572_10862	20.55	128.42
SCE1572_749	64.03	88.82
SCE1572_1351	61.16	105.15
SCE1572_2846	27.85	7.45
SCE1572_8725	48.7	18.43
SCE1572_9331	288.61	2595.21
SCE1572_10987	18.32	106.01
SCE1572_11170	62.46	55.08
SCE1572_11556	1.56	0
SCE1572_3490	19.14	56.69
SCE1572_7135	24.09	19.33
SCE1572_9928	22.94	43.21

SCE1572_8120	55.13	15.31
SCE1572_10546	4.74	3.8
SCE1572_2013	60.23	17.23
SCE1572_5157	10.04	4.16
SCE1572_8780	54.55	75.3
SCE1572_11569	17.78	168.95
SCE1572_10981	54.54	228.23
SCE1572_7703	16.02	63.79
SCE1572_1859	26.37	3.97
SCE1572_1195	2.71	0
SCE1572_3925	103.44	107.4
SCE1572_2252	42.36	41.89
SCE1572_8033	0	0
SCE1572_7034	85.34	83.75
SCE1572_81	5.05	8.11
SCE1572_11535	46.45	52.03
SCE1572_10674	62.64	178.94
SCE1572_4788	68.1	72.16
SCE1572_9349	135.18	402.29
SCE1572_8114	42.25	17.95
SCE1572_4406	71.52	18.36
SCE1572_8856	27.43	113.69
SCE1572_4022	19.73	58.33
SCE1572_1176	51.07	39.37
SCE1572_11389	218.67	377.66
SCE1572_8915	23.94	543.27
SCE1572_5276	16.85	0
SCE1572_1906	21.17	153.97
SCE1572_835	42.34	13.15
SCE1572_3214	0	0
SCE1572_5491	37.11	24.25
SCE1572_6635	3.05	0
SCE1572_7125	50.79	40.75
SCE1572_10891	67.11	129.62
SCE1572_10719	30.98	14.91
SCE1572_3010	18.05	5.21
SCE1572_8224	12.06	8.29
SCE1572_6715	21.45	12.91
SCE1572_2550	55.24	31.12
SCE1572_733	29.06	11.08
SCE1572_9315	14.78	13.24
SCE1572_8744	56.31	29.75
SCE1572_5385	76.73	50.4
SCE1572_509	35.5	19.1
SCE1572_3456	57.02	14.89
SCE1572_1800	7.88	9.48
SCE1572_6576	30.02	9.63
SCE1572_7028	62.57	101.37

SCE1572_632	117.98	344.4
SCE1572_4638	10.61	2.43
SCE1572_4759	70.97	45.29
SCE1572_7682	87.14	14.68
SCE1572_8952	20.78	9.09
SCE1572_2567	130.01	262.67
SCE1572_2702	46.99	75.13
SCE1572_6540	10.93	0
SCE1572_4850	78.87	66.35
SCE1572_9255	17.93	11.26
SCE1572_4899	13.63	10.93
SCE1572_10777	183.81	200.93
SCE1572_2644	28.48	22.23
SCE1572_8390	2.92	0
SCE1572_3735	55.39	20.44
SCE1572_7059	32.7	50.92
SCE1572_2153	38.53	50.4
SCE1572_1115	15.96	19.21
SCE1572_3048	7.44	1.05
SCE1572_3643	0	0
SCE1572_3359	51.63	23.59
SCE1572_178	33.86	18.52
SCE1572_2823	20.15	7.27
SCE1572_5484	24.29	4.38
SCE1572_6085	35.76	35.57
SCE1572_9940	0	0
SCE1572_10112	75.81	0
SCE1572_5582	19.56	11.77
SCE1572_9523	82.47	174.44
SCE1572_3960	60.34	57.72
SCE1572_9581	26.2	41.08
SCE1572_2250	152.16	147.3
SCE1572_2043	22.02	278.61
SCE1572_5668	4.71	0
SCE1572_7826	39.53	44.25
SCE1572_4890	275.46	450.47
SCE1572_8711	65.56	39.45
SCE1572_4177	67.25	21.02
SCE1572_11518	59.31	29.79
SCE1572_8047	34.73	12.23
SCE1572_7770	21.89	25.79
SCE1572_11022	41.14	30.86
SCE1572_1446	33.51	12.1
SCE1572_8326	39.78	39.54
SCE1572_9139	0	0
SCE1572_728	28	81.9
SCE1572_5088	35.41	58.75
SCE1572_9365	58.05	76.29

SCE1572_2326	11.9	52.31
SCE1572_4097	24.26	10.95
SCE1572_8111	49.62	13.72
SCE1572_6064	35.75	11.15
SCE1572_8040	72.84	44.85
SCE1572_10573	27.99	39.3
SCE1572_10146	160.67	192.97
SCE1572_6532	37.62	14.78
SCE1572_1908	33.82	54.26
SCE1572_8375	28.78	143.49
SCE1572_5516	2.71	0
SCE1572_9300	78.41	161.43
SCE1572_10152	289.85	1348.78
SCE1572_11572	46.34	195.19
SCE1572_101	6.59	0
SCE1572_674	215.97	435.81
SCE1572_4503	28.61	19.97
SCE1572_9583	28.61	17.22
SCE1572_8865	92.09	97.39
SCE1572_6494	9.36	9.01
SCE1572_11376	40.65	54.11
SCE1572_3004	7.08	0
SCE1572_454	34.94	14.92
SCE1572_1305	31.37	15.1
SCE1572_5214	94.46	42.03
SCE1572_5564	11.84	0
SCE1572_9065	41.86	14.86
SCE1572_6466	27.63	3.24
SCE1572_9551	42.55	23.08
SCE1572_10420	78.32	132.49
SCE1572_10695	21.42	11.9
SCE1572_2212	12.94	22.25
SCE1572_1882	33.94	16.08
SCE1572_4901	31.35	18.26
SCE1572_1033	42.78	19.55
SCE1572_10275	30.32	94.04
SCE1572_8153	74.43	13.27
SCE1572_5042	24.3	57.27
SCE1572_8372	78.8	91.23
SCE1572_3142	33.31	29.46
SCE1572_2628	49.65	17.07
SCE1572_4458	18.31	16.28
SCE1572_5435	31.45	14.42
SCE1572_10609	42.79	237.75
SCE1572_6443	2.92	10.53
SCE1572_4136	44.16	15.34
SCE1572_6513	0	0
SCE1572_1087	11.23	22.53

SCE1572_2836	14.6	40.55
SCE1572_9197	100.21	54.82
SCE1572_2952	13.46	4.05
SCE1572_4575	37.36	12.63
SCE1572_5159	38.51	44.41
SCE1572_1862	45.76	10.3
SCE1572_6702	84.1	20.44
SCE1572_4547	87.24	16.09
SCE1572_31	11.47	16.87
SCE1572_4272	39.67	74.69
SCE1572_4076	21.23	0
SCE1572_5076	35.59	147.55
SCE1572_10022	15.15	4.51
SCE1572_7642	19.2	9.43
SCE1572_1208	37.83	24.14
SCE1572_3899	65.61	26.44
SCE1572_2429	106.24	157.16
SCE1572_4752	16.39	59.18
SCE1572_5694	19.74	3.59
SCE1572_8514	51.85	0
SCE1572_1533	5.19	8.03
SCE1572_9649	39.31	7.72
SCE1572_4663	0	0
SCE1572_11423	33.86	14.82
SCE1572_5169	29.26	0
SCE1572_7830	82.43	60.23
SCE1572_4173	46.52	54
SCE1572_10498	19.87	28.52
SCE1572_11107	42.03	76.43
SCE1572_6308	50.36	20.2
SCE1572_5529	29.86	29.55
SCE1572_1490	30.63	7.37
SCE1572_8820	30.31	13.15
SCE1572_5876	38.14	334.32
SCE1572_1384	78.57	28.41
SCE1572_2306	48.93	149.29
SCE1572_3060	51.06	7.56
SCE1572_3707	21.38	28.07
SCE1572_8578	86.21	40.16
SCE1572_9777	36.8	687.33
SCE1572_8475	315.38	255.98
SCE1572_3445	79.39	51.29
SCE1572_8941	39.34	91.23
SCE1572_7368	326.84	2636.53
SCE1572_398	13.24	3.98
SCE1572_7049	14.42	4.79
SCE1572_93	83.07	41.52
SCE1572_10027	32.69	26.68

SCE1572_8922	35.22	57.6
SCE1572_6539	49.05	7.16
SCE1572_1126	0	0
SCE1572_7913	23.69	10.69
SCE1572_10880	83.47	406.31
SCE1572_9884	27.97	19.12
SCE1572_9038	28.97	48.81
SCE1572_7119	35.53	74.12
SCE1572_3229	252.69	141.5
SCE1572_8354	55.54	10.84
SCE1572_4087	14.53	6.56
SCE1572_10250	49.07	13.22
SCE1572_4244	80.03	915.69
SCE1572_4394	37.1	13.23
SCE1572_5926	133.97	19.6
SCE1572_5067	137.66	355.65
SCE1572_8187	4.74	2.28
SCE1572_688	96.51	249.6
SCE1572_10796	0	0
SCE1572_3157	28.06	36.93
SCE1572_8451	40.31	346.1
SCE1572_6025	31.19	37.53
SCE1572_10699	51.1	1273.17
SCE1572_4538	75.81	26.53
SCE1572_15	24.32	79.18
SCE1572_7431	33.69	6.54
SCE1572_9322	111.95	95.47
SCE1572_6155	33.61	33.26
SCE1572_6389	29.46	40.34
SCE1572_9422	152.89	466.12
SCE1572_9186	117.21	43.16
SCE1572_7661	46.97	8.6
SCE1572_5741	32.26	19.97
SCE1572_541	100.86	51.68
SCE1572_3420	35.18	146.9
SCE1572_7639	27.63	40.75
SCE1572_1813	16.75	8.06
SCE1572_1354	23.66	4.81
SCE1572_4328	20.45	8.56
SCE1572_9329	0	0
SCE1572_11254	29.61	64.94
SCE1572_8253	14.28	13.09
SCE1572_6677	44.7	68.85
SCE1572_11228	19.06	207.39
SCE1572_2088	52.88	35.8
SCE1572_9813	22.68	11.49
SCE1572_7097	27.61	20.04
SCE1572_4524	64.11	14.6

SCE1572_5101	35.95	23.51
SCE1572_3526	18.05	2111.34
SCE1572_9225	2.61	0
SCE1572_7594	23.58	32.44
SCE1572_10849	40.84	16.73
SCE1572_10428	43.04	20.01
SCE1572_8440	177.4	233.35
SCE1572_7744	35.23	1162.87
SCE1572_5361	44.29	139.41
SCE1572_6376	23.29	1.25
SCE1572_5911	35.4	60.28
SCE1572_9745	20.75	15.37
SCE1572_9045	60.31	25.65
SCE1572_5254	15.54	3.68
SCE1572_357	57.91	64.44
SCE1572_1660	117.82	418.38
SCE1572_2765	52.5	70.72
SCE1572_8756	162.83	74.03
SCE1572_6849	12.24	1.69
SCE1572_1254	55.35	23.17
SCE1572_2789	37.13	37.24
SCE1572_3681	7.32	5.29
SCE1572_232	6.79	0
SCE1572_7052	19.71	0
SCE1572_6722	56.99	191.52
SCE1572_5056	37.15	28.79
SCE1572_4557	26.82	12.16
SCE1572_2348	22.84	8.19
SCE1572_6012	36.74	76.12
SCE1572_3808	43.54	107.55
SCE1572_9770	43.32	23.97
SCE1572_8137	35.64	15.99
SCE1572_3140	72.1	136.5
SCE1572_4735	174.58	8302.36
SCE1572_3342	167.91	416.63
SCE1572_10921	45.84	50.92
SCE1572_3186	51.73	157.42
SCE1572_6080	57.48	31.76
SCE1572_4526	34.39	17.56
SCE1572_11098	39.68	39.97
SCE1572_5904	20.47	23.72
SCE1572_4332	18.23	20.79
SCE1572_11272	17.65	11.58
SCE1572_8449	24.03	39.88
SCE1572_1326	2.33	5.61
SCE1572_1232	28.61	13.77
SCE1572_239	14.21	0
SCE1572_5657	13.74	23.7

SCE1572_411	39.51	21.52
SCE1572_3392	161.48	224.74
SCE1572_5959	67.69	59.02
SCE1572_11441	24.82	38.75
SCE1572_6802	178.17	805.1
SCE1572_5120	107.91	1085.06
SCE1572_7817	33.6	62.2
SCE1572_9418	281.42	3254.31
SCE1572_9854	149.3	51.79
SCE1572_8203	19.25	0
SCE1572_3468	21.06	3.38
SCE1572_8559	27.61	28.48
SCE1572_1164	119.25	192.71
SCE1572_6170	20.72	68
SCE1572_961	135.09	481.88
SCE1572_895	10.71	2.06
SCE1572_175	20.15	2.42
SCE1572_6680	26.58	26.17
SCE1572_4470	22.9	5.7
SCE1572_10645	64.84	92.07
SCE1572_9427	15.72	0
SCE1572_6661	32.17	43.4
SCE1572_65	31.1	220.56
SCE1572_2686	0	0
SCE1572_5161	505.39	5279.97
SCE1572_5855	50	27.18
SCE1572_7894	1682.69	0
SCE1572_7953	20.97	11.54
SCE1572_11403	19.56	35.32
SCE1572_9147	25.49	157.29
SCE1572_10474	29.71	36.76
SCE1572_4446	0	0
SCE1572_3969	64.86	266.6
SCE1572_536	163.54	413.12
SCE1572_601	53.44	83.49
SCE1572_11185	30.96	14.25
SCE1572_1291	45.39	32.54
SCE1572_2988	17.55	13.52
SCE1572_10332	95.91	27.83
SCE1572_8078	0	0
SCE1572_2507	0	0
SCE1572_8303	18.02	76.56
SCE1572_1391	47.41	60.62
SCE1572_923	91.65	159.58
SCE1572_10749	62.9	589.49
SCE1572_1694	44.46	14.94
SCE1572_1654	23.85	12.94
SCE1572_6840	18.57	50.27

SCE1572_8587	0	0
SCE1572_675	79.01	10.28
SCE1572_5761	44.54	31.4
SCE1572_10351	48.16	34.29
SCE1572_4223	53.83	69.1
SCE1572_8409	0	0
SCE1572_4423	1.33	0
SCE1572_7180	15.64	5.79
SCE1572_417	8.03	4.29
SCE1572_7918	43.71	15.51
SCE1572_11215	31.79	14.8
SCE1572_11318	24.83	32.6
SCE1572_3584	6	0
SCE1572_10481	62.36	64.73
SCE1572_2388	0	0
SCE1572_7007	0	0
SCE1572_11067	33.17	45.62
SCE1572_9549	47.97	22.81
SCE1572_11559	41.58	21.39
SCE1572_8132	14.49	5.68
SCE1572_1667	16.45	22.38
SCE1572_4419	47.27	14.16
SCE1572_7044	943.07	7059.24
SCE1572_423	44.45	177.68
SCE1572_8841	35.04	18.25
SCE1572_11177	33.99	67.53
SCE1572_2400	74.32	757.67
SCE1572_8425	23.84	158.36
SCE1572_1148	16.99	40.88
SCE1572_259	15.55	12.48
SCE1572_2520	76.3	36.73
SCE1572_5482	59.26	84.08
SCE1572_2783	24.79	48.2
SCE1572_10840	34.51	71.2
SCE1572_11115	61.9	64.45
SCE1572_809	74.51	168.43
SCE1572_7647	83.26	24.95
SCE1572_5969	42.54	15.4
SCE1572_472	44.66	251.77
SCE1572_2447	49.44	15.87
SCE1572_6153	0	0
SCE1572_2965	27.24	6.19
SCE1572_983	66.89	269.46
SCE1572_7346	0	0
SCE1572_4205	34.36	64.32
SCE1572_1421	18.72	6.19
SCE1572_5884	43.75	43.15
SCE1572_6571	26.43	7.49

SCE1572_11465	34.08	20.37
SCE1572_10155	13.74	10.67
SCE1572_7777	18.14	15.71
SCE1572_2969	52.28	6
SCE1572_5358	45.94	137.54
SCE1572_5805	53.2	685.03
SCE1572_9565	41.4	19.36
SCE1572_7795	19.37	23.87
SCE1572_4598	52.25	142.14
SCE1572_8170	46.65	9.36
SCE1572_3868	116.29	55.68
SCE1572_11348	33.3	42.19
SCE1572_9601	91.3	254.26
SCE1572_5754	26.94	54.67
SCE1572_3267	28.63	94
SCE1572_6382	18.36	9.07
SCE1572_3430	51.08	148.31
SCE1572_7457	9.2	1.17
SCE1572_1275	15.49	29.3
SCE1572_3567	32.02	7.93
SCE1572_3761	77.04	45.08
SCE1572_8232	19.16	29.07
SCE1572_5670	168.62	78.38
SCE1572_9678	26.44	12.99
SCE1572_7460	21.49	19.57
SCE1572_4870	10.83	98.25
SCE1572_11355	46.11	27.99
SCE1572_4510	97.99	97.05
SCE1572_599	26.14	16.36
SCE1572_11585	18.66	0
SCE1572_5933	19.49	7.9
SCE1572_5895	104.41	20.99
SCE1572_4020	29.42	72.62
SCE1572_7021	48.2	18.91
SCE1572_6036	16.85	0
SCE1572_3325	69.77	199.5
SCE1572_1241	17.81	22.08
SCE1572_3080	43.42	223.93
SCE1572_1009	22.85	13.57
SCE1572_2993	124.86	123.43
SCE1572_2922	44.89	16.81
SCE1572_9354	93.65	98.74
SCE1572_629	42.95	129.4
SCE1572_4365	71.89	18.04
SCE1572_6283	11.2	27.92
SCE1572_7532	7.59	4.81
SCE1572_4124	38.47	32.68
SCE1572_4794	327.2	728.67

SCE1572_5575	11.66	21.05
SCE1572_5649	120.78	157.44
SCE1572_6271	5.35	15.03
SCE1572_7174	0	0
SCE1572_7110	0	0
SCE1572_3535	29.24	7.58
SCE1572_8335	0	0
SCE1572_11595	362.1	1311.64
SCE1572_10281	76.14	364.92
SCE1572_5840	30.82	2.97
SCE1572_163	37.18	19.35
SCE1572_2869	13.69	68.42
SCE1572_7712	15.05	18.56
SCE1572_6404	8.32	0
SCE1572_821	30.32	12.16
SCE1572_4386	22.48	8.49
SCE1572_6800	233.94	467.08
SCE1572_3900	71.97	95.37
SCE1572_7363	89.29	80.67
SCE1572_192	3.61	0
SCE1572_7756	16.06	6.19
SCE1572_2778	23.68	32.99
SCE1572_2434	43.19	32.79
SCE1572_2662	42.18	170.11
SCE1572_4053	42.94	177.62
SCE1572_622	0	0
SCE1572_2852	0	0
SCE1572_9130	17.8	8.29
SCE1572_6059	102.76	874.44
SCE1572_1602	19.03	11.45
SCE1572_8024	11.34	10.23
SCE1572_5686	34.91	64.82
SCE1572_4832	0	0
SCE1572_2919	34.39	32.6
SCE1572_10578	0	0
SCE1572_382	20.22	6.08
SCE1572_718	16.49	10.69
SCE1572_4531	26.77	17.77
SCE1572_10909	62.49	67.62
SCE1572_8795	28.47	30.28
SCE1572_5630	77.63	263.17
SCE1572_11566	53.75	44.41
SCE1572_4742	24.96	71.2
SCE1572_3164	55.64	21.58
SCE1572_7041	31.92	6.78
SCE1572_8616	67.58	46.68
SCE1572_4867	0	0
SCE1572_9129	20.17	12.55

SCE1572_7963	36.88	147.08
SCE1572_4026	54.16	157.03
SCE1572_7506	43.51	13.56
SCE1572_6606	35.05	17.15
SCE1572_5618	69.29	23.54
SCE1572_2118	19.56	8.83
SCE1572_9150	51.71	330.98
SCE1572_2419	293.81	1695.41
SCE1572_3907	12.06	4.15
SCE1572_11094	55.98	28.07
SCE1572_2947	0	0
SCE1572_6872	23.7	38.03
SCE1572_4315	35.38	9.73
SCE1572_3997	56.18	210.81
SCE1572_11049	53.05	47.64
SCE1572_2669	58.73	28.64
SCE1572_7720	34.87	194.54
SCE1572_7999	7.86	2.7
SCE1572_5759	9.5	0
SCE1572_3113	61.71	15.82
SCE1572_11430	100.56	366.42
SCE1572_6145	26.79	8.51
SCE1572_10621	17.41	17.21
SCE1572_3064	74.85	26.18
SCE1572_6764	31.03	65.09
SCE1572_9159	23.69	7.6
SCE1572_9486	10.73	17.5
SCE1572_5442	30.62	15.71
SCE1572_2008	55.57	9.28
SCE1572_557	36.56	49.25
SCE1572_9456	0	0
SCE1572_6793	43.83	142.84
SCE1572_4262	43.86	22.12
SCE1572_6618	16.59	12.2
SCE1572_1256	46.58	37.88
SCE1572_8662	19.27	14.95
SCE1572_762	102.04	32.43
SCE1572_7237	103.21	1123.89
SCE1572_668	26.47	26.65
SCE1572_7162	43.05	28.16
SCE1572_8168	26.52	8.33
SCE1572_7192	58.48	5.21
SCE1572_1073	48.45	27.59
SCE1572_1588	10.94	5.18
SCE1572_9119	36.84	11.94
SCE1572_8095	9.73	1.38
SCE1572_4337	0	0
SCE1572_4305	21.69	13.36

SCE1572_10387	26.24	19.09
SCE1572_10705	29.23	6.59
SCE1572_9122	103.59	46.24
SCE1572_2943	5.76	0
SCE1572_3119	10.05	56.45
SCE1572_4445	35.97	113.52
SCE1572_4339	36.36	20.48
SCE1572_11008	22.91	49.88
SCE1572_1611	10.11	164.68
SCE1572_7632	345.12	3112.79
SCE1572_11416	22.87	47.86
SCE1572_10734	31.55	18.52
SCE1572_6646	22.7	11.71
SCE1572_548	52.42	18.12
SCE1572_337	54.46	26.7
SCE1572_7634	94.71	860.11
SCE1572_11544	57.44	17.44
SCE1572_517	61.58	40.55
SCE1572_11266	305.88	1693.75
SCE1572_7518	100.51	555.61
SCE1572_10371	17.21	16.75
SCE1572_1510	42.17	20.56
SCE1572_11248	21.95	11.56
SCE1572_7946	62.85	38.65
SCE1572_9918	58.21	28.24
SCE1572_8508	32.31	43.07
SCE1572_11322	12.79	12.54
SCE1572_592	24.78	65.32
SCE1572_2482	13.66	21.81
SCE1572_9831	15.23	10
SCE1572_9511	2.49	0
SCE1572_2897	5.99	0
SCE1572_4860	13.13	0
SCE1572_3775	26.76	27.32
SCE1572_1636	27.42	308.63
SCE1572_3274	23.82	16.38
SCE1572_8521	0	0
SCE1572_1805	12.85	12.37
SCE1572_9975	21.11	13.48
SCE1572_1618	21.97	24.93
SCE1572_7805	47.68	61.39
SCE1572_8981	0	0
SCE1572_6774	45.7	27.94
SCE1572_2676	16.5	191.15
SCE1572_3305	67.42	97.05
SCE1572_10105	24.45	23.54
SCE1572_7727	41.96	100.5
SCE1572_4565	25.33	58.2

SCE1572_841	47.12	80.31
SCE1572_11383	71.63	77.58
SCE1572_6426	33.08	8.85
SCE1572_2527	186.22	36.34
SCE1572_9090	23.45	4.03
SCE1572_6179	65.86	1384.42
SCE1572_11087	20.96	90.81
SCE1572_6244	27.88	6.99
SCE1572_5351	22.15	12.3
SCE1572_142	19.41	3.78
SCE1572_341	62.37	40.64
SCE1572_4823	29.35	50.45
SCE1572_348	22.86	183.43
SCE1572_315	52.03	20.77
SCE1572_6118	33.61	7.19
SCE1572_8493	9.51	4.29
SCE1572_7392	6.22	0
SCE1572_8286	24.74	69.14
SCE1572_6226	28.39	34.42
SCE1572_1521	27.86	9.36
SCE1572_7130	45.72	28.77
SCE1572_3878	92.57	74.91
SCE1572_10316	0	0
SCE1572_2127	12.63	3.38
SCE1572_5661	19.61	15.73
SCE1572_1456	26.95	13.52
SCE1572_2410	87.26	163.1
SCE1572_11345	121.95	341.12
SCE1572_4877	15.98	13.81
SCE1572_4213	285.6	1054.03
SCE1572_7136	37.25	10.87
SCE1572_8988	11.44	0
SCE1572_205	20.6	10.34
SCE1572_9181	34.27	15.66
SCE1572_298	26.84	32.3
SCE1572_6033	166.75	260.19
SCE1572_882	81.71	88.24
SCE1572_8638	69.89	191.52
SCE1572_7990	0	0
SCE1572_9024	79.25	725.7
SCE1572_3605	238.67	130.66
SCE1572_2717	62.49	32.05
SCE1572_9697	11.98	17.75
SCE1572_125	33.77	13.84
SCE1572_10294	62.09	198.8
SCE1572_1924	79.42	170.88
SCE1572_6334	22.89	8.58
SCE1572_3881	16.17	4.87

SCE1572_6131	129.47	881.55
SCE1572_6113	21.5	46.44
SCE1572_4805	32.63	26.18
SCE1572_1575	21.08	20.49
SCE1572_43	10	6.68
SCE1572_3789	695.72	6152.73
SCE1572_10966	40.82	18.05
SCE1572_10795	6.69	21.47
SCE1572_7698	27.8	16.95
SCE1572_199	32.56	56.08
SCE1572_7177	29.53	24.97
SCE1572_6433	14.21	9.12
SCE1572_11236	20.05	7.62
SCE1572_9237	116.13	46.59
SCE1572_6343	31.01	103.67
SCE1572_5677	31.59	46.78
SCE1572_1789	18.01	0
SCE1572_4761	0	0
SCE1572_8979	0	0
SCE1572_7019	114.77	75.28
SCE1572_2368	14.19	3.9
SCE1572_2370	13.32	32.07
SCE1572_7987	27.09	42.64
SCE1572_9020	0	0
SCE1572_312	35.63	17.95
SCE1572_3178	6.59	0
SCE1572_8881	61.82	30.16
SCE1572_8898	3.18	0
SCE1572_6789	71.72	34.83
SCE1572_5993	68.16	62.86
SCE1572_9470	71.6	448.04
SCE1572_7241	31.55	26.75
SCE1572_2130	5.77	0.99
SCE1572_5425	53.47	84.58
SCE1572_5877	52.48	87.02
SCE1572_3555	22.53	10.11
SCE1572_267	47.48	27.12
SCE1572_6048	102.03	225.68
SCE1572_3890	13.47	8.24
SCE1572_9684	125.22	403.05
SCE1572_9726	13.02	11.19
SCE1572_3791	40.09	33.37
SCE1572_1316	23.39	20.13
SCE1572_9290	39.33	159.84
SCE1572_2067	32.02	34
SCE1572_9111	37.15	5.47
SCE1572_9175	622.11	944.12
SCE1572_1769	38.64	14.09

SCE1572_5609	11.09	2.85
SCE1572_695	6.27	22.65
SCE1572_609	0	0
SCE1572_3230	41.01	121.14
SCE1572_6477	20.76	10.86
SCE1572_7230	57.83	52.67
SCE1572_3395	30.63	7.37
SCE1572_8667	34.69	24.74
SCE1572_4517	14.96	7.55
SCE1572_9299	158.38	1676.74
SCE1572_3570	31.5	13.43
SCE1572_1449	24.59	27.62
SCE1572_2862	5.95	0
SCE1572_6930	15.59	0
SCE1572_3947	142.99	329.19
SCE1572_2696	40.49	27.2
SCE1572_7426	40.89	131.21
SCE1572_7477	13.71	52.41
SCE1572_5831	31.95	34.68
SCE1572_1552	58.17	44.68
SCE1572_8276	58.71	63.67
SCE1572_7264	63.33	29.26
SCE1572_6743	10.83	3.72
SCE1572_6005	27.2	57.62
SCE1572_10690	38.48	62.68
SCE1572_323	24.96	28.46
SCE1572_5125	46.62	9.44
SCE1572_2111	32.06	144.53
SCE1572_9054	24.33	12.78
SCE1572_4997	22.97	34.88
SCE1572_9874	34.72	15.3
SCE1572_8770	4.1	0
SCE1572_3886	40.68	27.97
SCE1572_5333	11.23	11.58
SCE1572_743	20.12	15.77
SCE1572_3719	30.51	492.87
SCE1572_106	72.47	18.56
SCE1572_3509	18.23	10.24
SCE1572_977	88.44	1141.35
SCE1572_6974	16.47	12.26
SCE1572_3826	68.41	44.29
SCE1572_7546	49.95	47.81
SCE1572_9781	9.86	0
SCE1572_1696	26.91	12.45
SCE1572_9203	31.49	36.49
SCE1572_2139	23.3	6.43
SCE1572_8297	101.2	205.67
SCE1572_3181	55.25	379.7

SCE1572_8530	29.48	44.98
SCE1572_2902	19.58	9.43
SCE1572_8679	25.63	21
SCE1572_4102	1.88	0
SCE1572_10346	41.96	50.92
SCE1572_5260	29.82	415.54
SCE1572_3727	35.86	13.33
SCE1572_5247	91.89	42.02
SCE1572_5196	14.38	3.15
SCE1572_1998	62.52	21.32
SCE1572_6581	29.37	32.65
SCE1572_11329	199.23	491.59
SCE1572_1945	28.3	17.52
SCE1572_3415	57.28	450.07
SCE1572_2859	15.63	7.52
SCE1572_9218	38.65	32.2
SCE1572_6939	34.13	8.21
SCE1572_10888	24.89	0
SCE1572_1173	48.82	13.56
SCE1572_11455	130.76	86.82
SCE1572_10358	72.51	32.95
SCE1572_1647	67.18	114.44
SCE1572_9985	16.71	11.49
SCE1572_903	41.35	12.95
SCE1572_2128	85.19	29.54
SCE1572_2221	10.11	0
SCE1572_7407	0	0
SCE1572_10560	2.14	0
SCE1572_5115	27.23	51.37
SCE1572_7332	73.3	36.17
SCE1572_5951	28.36	15.75
SCE1572_1438	16.46	10.8
SCE1572_4911	69.98	162.11
SCE1572_1598	67.28	26.89
SCE1572_11312	0	0
SCE1572_4615	35.4	29.27
SCE1572_11428	59.27	29.86
SCE1572_9642	13.46	30.23
SCE1572_5706	38.96	129.93
SCE1572_9622	15.37	12.09
SCE1572_10175	533.77	1039.78
SCE1572_7334	100.76	602.78
SCE1572_1889	43.73	386.06
SCE1572_10254	0	0
SCE1572_1133	22.68	28.73
SCE1572_3664	23.88	5.13
SCE1572_4150	26.37	31.07
SCE1572_1651	60.8	7.23

SCE1572_5208	7.09	0
SCE1572_1328	51.7	9.79
SCE1572_2895	19.02	27.47
SCE1572_2974	0	0
SCE1572_9702	28.85	14.98
SCE1572_2454	73.36	238.64
SCE1572_7382	0	0
SCE1572_4006	35.17	32.52
SCE1572_4235	40.65	15.87
SCE1572_9936	73.15	203.27
SCE1572_837	26.39	27.03
SCE1572_4652	46.2	51.82
SCE1572_1420	15.09	24.97
SCE1572_7080	22.81	112.22
SCE1572_8652	40.25	137.67
SCE1572_4188	37.43	134.82
SCE1572_7204	22.3	0
SCE1572_9373	54.95	17.53
SCE1572_849	45.83	108.96
SCE1572_1875	56.13	37.79
SCE1572_9284	0	0
SCE1572_5372	109.82	1473.72
SCE1572_5178	16.68	13.73
SCE1572_2073	9.79	1.68
SCE1572_8706	35.67	54.49
SCE1572_6592	55.52	149.05
SCE1572_7836	17.78	38.53
SCE1572_11591	52.51	45.81
SCE1572_8539	15.12	15.87
SCE1572_3835	34.41	14.05
SCE1572_6830	7	0
SCE1572_11030	47.38	98.07
SCE1572_3266	43.9	105.66
SCE1572_5948	58.03	36.04
SCE1572_148	16.95	9.07
SCE1572_23	21.04	66.23
SCE1572_5947	28.54	19.24
SCE1572_948	38.14	15.71
SCE1572_2338	21.97	88.85
SCE1572_4224	15.16	0
SCE1572_7888	13.62	15.05
SCE1572_1562	42.73	16.59
SCE1572_5532	44.98	22.06
SCE1572_10132	29.35	23.32
SCE1572_775	163.48	317.32
SCE1572_2070	75.81	13.68
SCE1572_9183	27.44	13.46
SCE1572_6949	36.87	16.95

SCE1572_5121	14.08	7.09
SCE1572_1874	14.1	11.88
SCE1572_8937	832.84	7173.44
SCE1572_6818	16.28	7.35
SCE1572_10945	34.55	32.25
SCE1572_3281	18.06	27.45
SCE1572_5527	38.09	26.59
SCE1572_4618	32.31	24.8
SCE1572_3833	33.6	11.55
SCE1572_11166	31.13	86.03
SCE1572_1477	51.12	25.81
SCE1572_8127	46.1	22.19
SCE1572_8824	7.32	102.91
SCE1572_7061	0	0
SCE1572_2817	0	0
SCE1572_9804	0	0
SCE1572_871	88.44	175.16
SCE1572_6585	66.64	137.14
SCE1572_4607	11.59	16.27
SCE1572_9906	117.52	94.29
SCE1572_8011	35.12	102.42
SCE1572_7417	19.19	24.02
SCE1572_7349	36.37	17.71
SCE1572_9032	35.1	21.47
SCE1572_7874	29.99	46.12
SCE1572_6923	24.57	14.79
SCE1572_6015	153.66	44.38
SCE1572_7429	29.86	11.06
SCE1572_4005	25.92	43.67
SCE1572_1787	31.7	14.93
SCE1572_2514	17.58	0
SCE1572_5459	26.47	9.1
SCE1572_3630	34.14	59.96
SCE1572_8769	151.17	51.68
SCE1572_3935	13.39	9.91
SCE1572_5414	23.28	10.25
SCE1572_11016	7.11	0
SCE1572_7544	15.06	0
SCE1572_6671	25.5	9.95
SCE1572_7073	138.98	45.62
SCE1572_6329	34.53	28.66
SCE1572_64	50.22	83.68
SCE1572_5977	27.98	17.23
SCE1572_8807	41.18	4.51
SCE1572_6839	13.6	4.71
SCE1572_7760	71.71	68.33
SCE1572_4711	40.25	34.45
SCE1572_3348	32.75	20.44

SCE1572_1029	20.22	120.12
SCE1572_462	38.33	50.19
SCE1572_11038	0	0
SCE1572_10520	32.73	23.81
SCE1572_5154	28.83	20.15
SCE1572_6555	49.64	22.45
SCE1572_6123	19.45	29.68
SCE1572_5496	62.1	37.36
SCE1572_3450	26.93	10.97
SCE1572_4001	43.17	708.73
SCE1572_1269	41.28	35.29
SCE1572_4463	20.26	10.39
SCE1572_2265	53.86	108.04
SCE1572_1933	29	108.47
SCE1572_8218	37.43	21.73
SCE1572_1243	33.82	84.21
SCE1572_7233	30.99	19.98
SCE1572_5140	29.32	18.93
SCE1572_3120	32.31	17.95
SCE1572_8811	18.16	4.37
SCE1572_7929	55.78	142.87
SCE1572_8672	47.43	52.4
SCE1572_10059	627.87	4216.06
SCE1572_2486	48.68	77.79
SCE1572_5570	32.59	18.14
SCE1572_1716	0	0
SCE1572_7296	27.7	35.09
SCE1572_1286	49.97	835.53
SCE1572_4143	42.72	24.19
SCE1572_10838	27.76	28.64
SCE1572_11467	60.08	160.29
SCE1572_3123	67.22	34.16
SCE1572_3810	65.47	38.7
SCE1572_1374	28.18	59.25
SCE1572_5194	13.96	14.4
SCE1572_11300	22.65	45.8
SCE1572_8122	30.32	10.07
SCE1572_3637	0	0
SCE1572_2420	367.54	1325.91
SCE1572_8093	31.23	12.53
SCE1572_7376	30.55	26.11
SCE1572_5072	27.77	23.01
SCE1572_6105	35.88	0
SCE1572_9149	127.01	361.1
SCE1572_6795	80.01	135.94
SCE1572_11116	11.93	11.83
SCE1572_9809	19.84	34.1
SCE1572_1541	0	0

SCE1572_8590	202.74	708.63
SCE1572_4120	89.32	965.34
SCE1572_4617	36.81	4.22
SCE1572_1565	84.94	84.07
SCE1572_9165	0	0
SCE1572_4976	138.8	1498.24
SCE1572_3465	0	0
SCE1572_11582	42.76	45.68
SCE1572_10816	21.84	7.22
SCE1572_2694	17.77	98.36
SCE1572_6704	60.03	188.94
SCE1572_2207	33.22	16.4
SCE1572_3780	18.74	49.2
SCE1572_7	156.05	608.2
SCE1572_5340	26.74	34.53
SCE1572_11554	52.74	7.93
SCE1572_71	28.11	16.64
SCE1572_7096	62.97	29.66
SCE1572_3671	35.33	46.06
SCE1572_8200	56.15	9.01
SCE1572_1544	99.77	40.02
SCE1572_970	71.72	91.78
SCE1572_6418	83.7	1053.18
SCE1572_1379	33.01	13.47
SCE1572_10013	33.78	11.34
SCE1572_7317	9.19	3.69
SCE1572_915	5	4.01
SCE1572_8692	0	0
SCE1572_5477	23.35	286.56
SCE1572_795	170.33	322.57
SCE1572_11497	28.54	0
SCE1572_1571	27.44	10.43
SCE1572_4127	0	0
SCE1572_9636	96.15	130.84
SCE1572_8368	23.04	4.03
SCE1572_4962	22.8	100.61
SCE1572_4857	33.93	21.69
SCE1572_11183	62.43	119.4
SCE1572_9518	56.03	115.03
SCE1572_11306	36.71	89.45
SCE1572_8265	50.91	78.58
SCE1572_4350	18.09	8.71
SCE1572_4031	1147.07	1218.61
SCE1572_6200	68.83	110.06
SCE1572_4401	10.9	0
SCE1572_1229	18.95	19.63
SCE1572_2408	122.99	718.67
SCE1572_2593	0	0

SCE1572_6038	8.76	7.3
SCE1572_10076	321.83	846.9
SCE1572_10543	0	0
SCE1572_9672	39.9	108.84
SCE1572_575	2.76	0
SCE1572_8018	12.2	12.58
SCE1572_10896	5.12	0
SCE1572_5710	12.25	1.4
SCE1572_2223	48.33	197.16
SCE1572_10586	0	0
SCE1572_1362	113.78	519.02
SCE1572_8990	94.62	1865.86
SCE1572_10806	111.86	27.72
SCE1572_3669	0	0
SCE1572_1578	39.74	16.19
SCE1572_8503	62.27	10.86
SCE1572_9446	86.97	104.67
SCE1572_7700	35.02	10.67
SCE1572_570	74.73	224.33
SCE1572_9127	28.64	47.3
SCE1572_5203	33.82	214.34
SCE1572_788	56.49	69.22
SCE1572_10755	34.47	16.85
SCE1572_4690	34.65	53.67
SCE1572_5820	76.42	71.44
SCE1572_195	2.23	8.05
SCE1572_7850	47.92	73.81
SCE1572_5356	0	0
SCE1572_4298	101.08	78.2
SCE1572_7842	157.74	261.71
SCE1572_9792	12.69	3.39
SCE1572_4389	98.39	27.18
SCE1572_2190	52.09	158.95
SCE1572_9862	37.9	30.98
SCE1572_9955	51.23	20.05
SCE1572_4771	28.21	14.4
SCE1572_2841	30.32	19.46
SCE1572_4815	22.4	103.78
SCE1572_4105	20.07	7.51
SCE1572_648	64.18	98.53
SCE1572_2865	5.76	0
SCE1572_10221	82.13	29.28
SCE1572_8152	13.78	0
SCE1572_4625	18.47	8.27
SCE1572_11307	34.85	74.66
SCE1572_4678	7.15	0
SCE1572_3680	27.86	8.78
SCE1572_4846	19.09	43.21

SCE1572_7342	50	282.84
SCE1572_10731	49.22	76.42
SCE1572_10431	46.31	32.36
SCE1572_6886	16.85	14.1
SCE1572_339	17.09	18.58
SCE1572_351	30.79	82.49
SCE1572_2303	21.25	64.8
SCE1572_11009	37.39	36.62
SCE1572_2477	86.27	20.56
SCE1572_386	4.1	4.93
SCE1572_3916	94.09	98.35
SCE1572_10223	37.74	56.26
SCE1572_9273	56.12	69.56
SCE1572_10315	13.34	37.95
SCE1572_2197	41.85	38.34
SCE1572_7677	20.44	6.05
SCE1572_6353	53.96	37.11
SCE1572_5847	50.54	63.77
SCE1572_7645	26.46	21.47
SCE1572_396	0	0
SCE1572_1263	47.79	759.36
SCE1572_8930	166.23	164.88
SCE1572_8718	40.23	55.04
SCE1572_646	0	0
SCE1572_116	42.06	72.32
SCE1572_4107	16.48	9.7
SCE1572_7978	45.01	35.42
SCE1572_5941	32.78	11.66
SCE1572_1961	64.68	19.56
SCE1572_1002	24.02	5.42
SCE1572_9336	14.73	29.19
SCE1572_3473	138.2	90.72
SCE1572_10099	44.2	76.83
SCE1572_9650	38.64	16.91
SCE1572_5825	30.94	29.49
SCE1572_9924	120.05	354.9
SCE1572_5598	39.74	116.55
SCE1572_5299	34.68	13.12
SCE1572_3541	0	0
SCE1572_10403	30.26	62.59
SCE1572_7289	60.52	17.82
SCE1572_6165	18.06	35.1
SCE1572_3066	21.18	10.19
SCE1572_8619	39.83	25.23
SCE1572_4157	5.8	3.99
SCE1572_1084	15.5	11.66
SCE1572_2081	77.69	35.44
SCE1572_4493	29.79	52.46

SCE1572_8762	36.76	19.25
SCE1572_10438	20.64	25.7
SCE1572_7493	58.17	22.05
SCE1572_2465	30.78	40.75
SCE1572_466	49.01	36.86
SCE1572_3197	11.2	7.19
SCE1572_6827	41.6	220.55
SCE1572_6934	63.96	148.47
SCE1572_7467	45.67	36.94
SCE1572_2244	48.22	32.41
SCE1572_7579	62.31	72.21
SCE1572_5545	25.27	21.96
SCE1572_10693	32.4	21.83
SCE1572_8314	89.65	99.64
SCE1572_2311	136.42	262.98
SCE1572_8612	40.4	11.03
SCE1572_11147	35.92	12.64
SCE1572_3204	75.37	102.18
SCE1572_6189	42.24	34.82
SCE1572_11522	50.77	20.37
SCE1572_626	40.49	43.07
SCE1572_7092	13.62	50.26
SCE1572_8149	30.01	15.21
SCE1572_157	73.23	439.46
SCE1572_6805	50.41	47.64
SCE1572_5015	223.58	6096.05
SCE1572_246	7.98	7.68
SCE1572_6876	17.36	39
SCE1572_6947	32.37	18.18
SCE1572_4648	57.17	29.91
SCE1572_3300	35.64	14.98
SCE1572_4322	31	11.76
SCE1572_7413	36.53	57.73
SCE1572_345	25.7	9.9
SCE1572_3174	46.61	77.06
SCE1572_1032	39.14	32.56
SCE1572_3024	42.13	20.68
SCE1572_4045	16.41	13.37
SCE1572_10955	142.7	391.27
SCE1572_10092	74.6	59.02
SCE1572_6989	14.88	15.67
SCE1572_7126	165.09	75.69
SCE1572_6902	63.63	11.34
SCE1572_11542	75.53	18.44
SCE1572_10244	8.03	0
SCE1572_1039	24.28	4.77
SCE1572_11361	28.89	39.85
SCE1572_5394	212.95	99.52

SCE1572_7001	40.88	38.74
SCE1572_1928	107.28	247.67
SCE1572_9659	62.12	86.39
SCE1572_10292	153.1	228.97
SCE1572_11372	41.48	161.7
SCE1572_10501	39.31	70.96
SCE1572_10469	31.78	113.14
SCE1572_7153	91.92	26.23
SCE1572_5337	63.83	28.23
SCE1572_4586	168.22	323.39
SCE1572_10442	19.1	6.11
SCE1572_1867	85.28	56.42
SCE1572_4092	61.59	24.71
SCE1572_3522	0	0
SCE1572_9718	63.12	212.18
SCE1572_8100	26.34	34.75
SCE1572_11051	42.96	336.03
SCE1572_5416	24.24	6.78
SCE1572_4826	68.01	88.36
SCE1572_1192	44.51	27.62
SCE1572_3500	51.32	47.07
SCE1572_10115	39.22	57.14
SCE1572_3036	38.52	8.88
SCE1572_3078	43.16	47.36
SCE1572_2318	15.11	0
SCE1572_9823	79.16	20.19
SCE1572_1406	72.97	119.45
SCE1572_7068	16.91	10.18
SCE1572_5314	0	0
SCE1572_10715	42.59	31.51
SCE1572_2090	14.74	0
SCE1572_11243	16.33	37.43
SCE1572_4940	179.18	2116.55
SCE1572_4118	28.61	3541.81
SCE1572_1558	40.45	15.89
SCE1572_5224	43.63	70.88
SCE1572_10858	275.39	449.06
SCE1572_7747	52.42	70.54
SCE1572_11219	48.41	28
SCE1572_6498	60.49	222.58
SCE1572_10173	74.3	75.12
SCE1572_4019	31.62	38.51
SCE1572_8116	0	0
SCE1572_9140	30.84	13.61
SCE1572_2259	116.72	35.8
SCE1572_10910	88.82	19.31
SCE1572_10689	14.15	14.6
SCE1572_7190	45.53	55.96

SCE1572_9344	31.69	91.75
SCE1572_3439	24.2	18.69
SCE1572_10231	80.64	29.08
SCE1572_8189	34.24	26.16
SCE1572_10129	27.46	2.87
SCE1572_5468	43.07	18.18
SCE1572_8592	64.54	65.67
SCE1572_6688	27.4	8.78
SCE1572_2262	17.4	11.39
SCE1572_1587	51.32	0
SCE1572_8244	354.59	1253.68
SCE1572_4709	37.61	6.32
SCE1572_3382	9.45	27.69
SCE1572_8088	56.78	14.04
SCE1572_8813	0	0
SCE1572_7356	100	102.66
SCE1572_8655	211.69	2161.99
SCE1572_7819	120.41	442.48
SCE1572_8227	21.41	77.31
SCE1572_7669	23.11	6.89
SCE1572_3496	34.95	48.31
SCE1572_3695	26.87	110.86
SCE1572_2549	0	0
SCE1572_8482	18.27	131.9
SCE1572_975	340.72	5457.79
SCE1572_7695	17.3	22.04
SCE1572_1331	50.54	34.17
SCE1572_3107	15.08	13.1
SCE1572_9343	140.27	1218.32
SCE1572_5259	13.59	11.15
SCE1572_7280	19.64	7.27
SCE1572_4987	68.86	87.23
SCE1572_5451	49.15	21.58
SCE1572_2033	78.43	160.9
SCE1572_11425	51.27	72.01
SCE1572_7365	66.5	148.99
SCE1572_1748	49.09	43.8
SCE1572_8081	4.97	0
SCE1572_10527	42.64	40.73
SCE1572_8038	66.83	18.41
SCE1572_407	51.02	383.95
SCE1572_9948	73.64	20.85
SCE1572_4473	41.35	88.47
SCE1572_7898	37.04	93.91
SCE1572_1451	23.98	5.87
SCE1572_6098	31.29	32.22
SCE1572_6719	49.55	364.92
SCE1572_10233	21.66	6.13

SCE1572_3295	12.89	14.77
SCE1572_3787	76.85	121.64
SCE1572_586	38.48	31.25
SCE1572_8388	26.59	10.89
SCE1572_11126	27.07	21.26
SCE1572_2579	66.74	35.7
SCE1572_1505	38.13	10.67
SCE1572_11598	85.7	1010.9
SCE1572_3401	26.44	12.73
SCE1572_856	0	0
SCE1572_11157	87.16	35.66
SCE1572_1363	177.76	926.99
SCE1572_4700	26.97	12.98
SCE1572_5008	195.95	629.54
SCE1572_4923	14.17	6.82
SCE1572_6864	2.11	0
SCE1572_4952	9.85	0.85
SCE1572_1629	6.42	3.09
SCE1572_8056	0	0
SCE1572_2998	85.55	75.59
SCE1572_1338	24.35	17.45
SCE1572_534	0	0
SCE1572_5084	83.57	1490.47
SCE1572_10864	25.27	79.53
SCE1572_4670	31.37	9.56
SCE1572_4408	45.91	12.85
SCE1572_7960	41.14	77.02
SCE1572_7920	66.8	32.93
SCE1572_6928	34.36	37
SCE1572_11482	40.68	151.31
SCE1572_5130	51.9	95.34
SCE1572_8999	164.86	784.03
SCE1572_6207	40.64	8.87
SCE1572_747	34.06	12.06
SCE1572_7318	22.99	19.25
SCE1572_5697	43.71	89.03
SCE1572_2626	25.72	28.37
SCE1572_8713	62.36	39.4
SCE1572_3408	91.3	143.84
SCE1572_6211	50.54	9.5
SCE1572_4720	54.12	20.88
SCE1572_5782	17.76	32.88
SCE1572_8210	13.04	20.17
SCE1572_8460	16.01	70.26
SCE1572_9741	41.77	214.73
SCE1572_1894	46.65	19.81
SCE1572_10738	60.03	29.1
SCE1572_9990	24.76	21.9

SCE1572_1915	88.7	41.38
SCE1572_7209	33.27	45.62
SCE1572_6968	42.87	223.99
SCE1572_5729	32.19	35.68
SCE1572_8852	39.89	19.69
SCE1572_3618	0	0
SCE1572_9891	43.77	1.88
SCE1572_1847	61.77	25.34
SCE1572_781	10.75	11.62
SCE1572_1724	58.18	277.39
SCE1572_5230	64.35	356.64
SCE1572_4683	76.41	49.63
SCE1572_9081	75.81	10.58
SCE1572_5280	66.56	97.91
SCE1572_7658	0	0
SCE1572_6961	0	0
SCE1572_8854	25.96	116.23
SCE1572_5807	24.5	18.43
SCE1572_1152	17.65	17.5
SCE1572_10165	24.12	11.85
SCE1572_5520	24.12	35.94
SCE1572_11499	24.24	13.79
SCE1572_4959	0	0
SCE1572_2116	24.45	5.89
SCE1572_11538	43.03	650.94
SCE1572_8060	70.26	4.45
SCE1572_7558	17.97	0.76
SCE1572_5864	18.01	18.07
SCE1572_3367	63.89	1156.57
SCE1572_6339	15.82	45.94
SCE1572_8893	0	0
SCE1572_11490	0	13.81
SCE1572_11323	35.72	103.9
SCE1572_1167	12.4	56.4
SCE1572_5367	12.63	22.12
SCE1572_2151	41.81	8.13
SCE1572_9082	24.68	0
SCE1572_8945	161.13	811.67
SCE1572_10487	251.38	8122.2
SCE1572_9834	51.79	22.67
SCE1572_8804	27.71	172.27
SCE1572_8902	31.31	16.99
SCE1572_1310	233.26	2150.94
SCE1572_8191	51.46	21.52
SCE1572_8064	30.32	0
SCE1572_3333	70.02	148.08
SCE1572_8031	73.36	55.99
SCE1572_8256	21.96	40.93

SCE1572_708	59.7	100.21
SCE1572_10649	27.27	3.86
SCE1572_5833	46.74	97.42
SCE1572_4727	25.09	13.01
SCE1572_7808	122.85	99.32
SCE1572_10039	11.54	15.15
SCE1572_254	0	0
SCE1572_6367	27.34	130.12
SCE1572_7707	34.64	23.59
SCE1572_11059	67.23	15.37
SCE1572_2556	0	0
SCE1572_89	98.94	119.59
SCE1572_823	32.44	10.89
SCE1572_10365	41.9	122.86
SCE1572_1486	61.64	90.38
SCE1572_5720	22.66	11.69
SCE1572_274	0	0
SCE1572_8359	0	0
SCE1572_5250	44.55	220.51
SCE1572_9204	36.67	61.61
SCE1572_6460	62.71	28.16
SCE1572_7225	110.77	92.65
SCE1572_3279	41.94	68.77
SCE1572_3254	9.28	20.61
SCE1572_1215	44.63	3.41
SCE1572_6980	24.4	11.53
SCE1572_10061	1251.91	7965.73
SCE1572_4971	0	0
SCE1572_10259	20.81	7.16
SCE1572_8835	50.75	16.8
SCE1572_5955	0	0
SCE1572_1198	99.48	33.9
SCE1572_8437	1.56	22.57
SCE1572_4927	18.1	0
SCE1572_2096	20.86	65.28
SCE1572_6905	30.93	9.08
SCE1572_5882	41.2	37.02
SCE1572_6445	26.1	12.03
SCE1572_8555	15.73	192.79
SCE1572_9163	12.26	1.55
SCE1572_9120	18	15.97
SCE1572_10711	9.63	0
SCE1572_2277	55.46	447.14
SCE1572_5730	0	0
SCE1572_8432	6.65	1.69
SCE1572_1342	195.25	673.4
SCE1572_1108	55.22	15.25
SCE1572_7530	39.19	30.49

SCE1572_111	39.55	23.8
SCE1572_3770	0	0
SCE1572_5024	52.75	34.82
SCE1572_6420	43.65	58.31
SCE1572_11221	9.94	0
SCE1572_5053	64.33	47.34
SCE1572_965	6.37	0
SCE1572_10626	17.44	45.21
SCE1572_2393	49.52	290.04
SCE1572_5914	30.23	92.08
SCE1572_9533	28.17	29.88
SCE1572_9014	130.89	528.85
SCE1572_10970	99.98	270.71
SCE1572_10600	16.5	8.99
SCE1572_10085	86.82	245.84
SCE1572_3100	18.22	6.45
SCE1572_6821	45.75	14.16
SCE1572_7628	17.53	15.82
SCE1572_5037	33.54	241.44
SCE1572_7740	38.41	71.04
SCE1572_2737	17.04	23.63
SCE1572_6735	42.44	149.35
SCE1572_1425	47.79	57.51
SCE1572_4733	28.93	39
SCE1572_2700	7.98	2.95
SCE1572_1842	49.71	7.49
SCE1572_9618	15.16	15.78
SCE1572_5865	36.27	22.79
SCE1572_5756	44.65	16.25
SCE1572_162	36.26	20.82
SCE1572_10519	25.02	13.21
SCE1572_7513	10.17	3.16
SCE1572_432	34.15	155.06
SCE1572_10985	2945.68	8445.35
SCE1572_11399	34.54	56.53
SCE1572_8397	26.31	14.48
SCE1572_3236	74.8	559.55
SCE1572_569	73.36	115.36
SCE1572_6712	58.11	247.57
SCE1572_980	21.17	17.9
SCE1572_945	74.53	14.72
SCE1572_4483	42.72	66.66
SCE1572_5744	42.18	32.53
SCE1572_11289	0	0
SCE1572_9402	297.4	1405.3
SCE1572_6728	12.29	32.41
SCE1572_5674	33.28	13.35
SCE1572_503	12.57	3.78

SCE1572_1048	18.49	32.64
SCE1572_1604	65.42	42.61
SCE1572_10512	164.34	387.89
SCE1572_3977	59.9	40.83
SCE1572_10201	20.55	43.48
SCE1572_7535	527.12	153.08
SCE1572_10684	49.17	14.79
SCE1572_6602	10.22	6.83
SCE1572_2793	15.55	1.65
SCE1572_4253	1.46	0
SCE1572_10871	48.13	18.42
SCE1572_1622	31.24	196.21
SCE1572_8321	31.55	23.2
SCE1572_185	31.99	6.7
SCE1572_650	756.41	1310.03
SCE1572_9797	25.57	30.78
SCE1572_1637	10.47	8.94
SCE1572_6215	44.59	16.36
SCE1572_2615	20.35	20.54
SCE1572_7611	35.35	98.51
SCE1572_475	25.49	268.97
SCE1572_2656	47.6	26.16
SCE1572_9436	587.4	1828.88
SCE1572_635	153.03	966.7
SCE1572_10267	31.69	52.8
SCE1572_1632	36.53	48.36
SCE1572_5284	27.32	65.76
SCE1572_4057	1.85	0
SCE1572_1470	56.74	49.76
SCE1572_2756	3.37	0
SCE1572_400	26.85	22.81
SCE1572_5013	43.54	93.7
SCE1572_6953	22.58	14.23
SCE1572_5556	60.58	94.96
SCE1572_10859	141.54	405.11
SCE1572_1658	24.78	8.77
SCE1572_4932	232.98	956.72
SCE1572_9965	0	0
SCE1572_6542	54.65	87.68
SCE1572_359	46.33	27.44
SCE1572_7774	7.71	0
SCE1572_401	49.62	42.02
SCE1572_11549	26.67	15.83
SCE1572_8023	16.33	0
SCE1572_8404	20.34	4.45
SCE1572_3422	38.04	49.4
SCE1572_6475	97.54	560.57
SCE1572_10727	80.37	53.5

SCE1572_11507	24.09	9.9
SCE1572_953	43.18	43.5
SCE1572_5930	0	0
SCE1572_7214	32.23	104.88
SCE1572_6073	2.92	0
SCE1572_3803	46.29	16.15
SCE1572_2166	62.72	23.02
SCE1572_6391	0	0
SCE1572_1683	10.22	434.63
SCE1572_206	0	0
SCE1572_4345	20.89	22.99
SCE1572_11295	13.42	32.29
SCE1572_11150	28.99	41.44
SCE1572_1022	43.89	20.58
SCE1572_284	14.76	16.15
SCE1572_8043	0	0
SCE1572_1013	19.17	9.74
SCE1572_6360	68.75	253.86
SCE1572_1702	22.05	23.7
SCE1572_5589	53.8	42.56
SCE1572_3554	6.97	2.1
SCE1572_6361	86.74	48.43
SCE1572_9962	3.09	0
SCE1572_375	44.02	14.13
SCE1572_3360	15.18	12.42
SCE1572_1382	3.61	0
SCE1572_496	33.51	32.26
SCE1572_4906	30.12	39.99
SCE1572_10279	9.32	0
SCE1572_7742	43.19	73.72
SCE1572_1958	71.95	89.07
SCE1572_2484	143.1	262.42
SCE1572_5543	58.2	7.37
SCE1572_11096	43.62	24.96
SCE1572_3840	35.18	12.1
SCE1572_11175	52.15	114.5
SCE1572_9050	45.79	52.39
SCE1572_4536	19.88	84.95
SCE1572_2141	23.78	1.43
SCE1572_363	28.39	9.62
SCE1572_2979	54	20
SCE1572_6042	85.32	3260.6
SCE1572_139	12.63	30.41
SCE1572_10363	42.26	140.8
SCE1572_2086	44.94	60.67
SCE1572_7149	11.95	50.87
SCE1572_2884	39.53	56.04
SCE1572_7256	41.23	84.47

SCE1572_7012	0	0
SCE1572_10071	851.58	1529.63
SCE1572_9429	11.96	8.06
SCE1572_6087	32.22	36.49
SCE1572_1454	22.3	12.38
SCE1572_5163	0	0
SCE1572_2811	114.59	8.49
SCE1572_6963	46.4	123.63
SCE1572_11528	20.61	4.41
SCE1572_2771	3.49	0
SCE1572_8139	17.63	53.75
SCE1572_8427	67.11	8.97
SCE1572_6847	11.39	0.68
SCE1572_18	55.2	188.91
SCE1572_9852	47.96	57.72
SCE1572_8582	43.08	29.91
SCE1572_3980	53.8	93.19
SCE1572_1717	17.81	55.42
SCE1572_713	47.21	61.62
SCE1572_4522	46.4	20.22
SCE1572_3892	48.29	89.2
SCE1572_1887	54.15	126.24
SCE1572_2458	48.76	160.76
SCE1572_3340	97.22	5100.43
SCE1572_2148	58.18	16.94
SCE1572_559	37.9	1148.83
SCE1572_2023	46.52	48.33
SCE1572_4729	36.93	23.39
SCE1572_10328	59.23	17.04
SCE1572_11069	56.94	54.03
SCE1572_5035	27.06	19.64
SCE1572_8484	27.72	56.61
SCE1572_10964	34.38	19.14
SCE1572_3188	13.33	2.76
SCE1572_10046	59.71	427.35
SCE1572_660	343.25	992.62
SCE1572_5654	37.87	434.93
SCE1572_7637	56.04	795.44
SCE1572_10703	36.14	173.95
SCE1572_3683	33.32	7.02
SCE1572_2729	22.59	16.15
SCE1572_7670	17.33	5.21
SCE1572_6172	3.23	0
SCE1572_10213	11.66	0
SCE1572_5060	67.26	179.78
SCE1572_3517	57.93	21.82
SCE1572_2945	0	0
SCE1572_2730	30.92	22.43

SCE1572_10334	25.42	24.04
SCE1572_8557	18.46	9
SCE1572_2343	54.15	39.1
SCE1572_7160	17.37	9.04
SCE1572_2076	38.12	41.99
SCE1572_7855	61.19	105.52
SCE1572_4309	23.11	9.14
SCE1572_1238	22.37	11.96
SCE1572_7792	24.48	9.07
SCE1572_1398	74.42	35.29
SCE1572_7799	2.57	0
SCE1572_552	22.88	110.14
SCE1572_11352	21.66	0
SCE1572_8589	17.38	4.65
SCE1572_4555	50.45	23.38
SCE1572_8548	42.54	17.73
SCE1572_8291	57.95	23.7
SCE1572_6757	32.52	43.37
SCE1572_685	0	0
SCE1572_5480	39.88	19.24
SCE1572_7732	11	28.07
SCE1572_9613	15.35	0
SCE1572_8301	89.89	1300.69
SCE1572_171	39.24	68.69
SCE1572_1880	44.62	30.03
SCE1572_3629	15.9	5.1
SCE1572_6786	91.57	160.78
SCE1572_6227	9.69	3.01
SCE1572_4580	53.27	24.28
SCE1572_11419	0	0
SCE1572_2279	81.56	122.16
SCE1572_2386	78.92	104.26
SCE1572_9171	16.98	10.97
SCE1572_1656	12.55	53.46
SCE1572_10893	34.36	58.93
SCE1572_10557	25.41	10.87
SCE1572_4317	18.8	2.89
SCE1572_1303	18.89	89.69
SCE1572_925	68.56	128.8
SCE1572_5437	0	0
SCE1572_8974	34.51	22.25
SCE1572_6549	42.62	92.49
SCE1572_1493	4.46	0
SCE1572_234	66.33	43.08
SCE1572_6573	50.07	15.17
SCE1572_8782	43.09	231.87
SCE1572_7749	82.7	28.75
SCE1572_759	1398.15	940.17

SCE1572_7301	0	0
SCE1572_4425	16.07	79.22
SCE1572_3091	151.62	102.8
SCE1572_3569	11.17	3.84
SCE1572_2509	42.45	79.24
SCE1572_8045	36.53	11.99
SCE1572_1040	8.64	0
SCE1572_2867	0	0
SCE1572_7311	38.49	15.35
SCE1572_1412	15.76	61.38
SCE1572_8723	18.27	13.19
SCE1572_10277	11.38	10.47
SCE1572_613	59.49	41.9
SCE1572_8076	84.34	26.99
SCE1572_10633	42.71	259.39
SCE1572_3763	29.9	35.98
SCE1572_7561	25.52	18.07
SCE1572_5892	39.13	150.09
SCE1572_470	67.38	187.43
SCE1572_2449	24.97	6.44
SCE1572_11092	37.65	146.21
SCE1572_11217	20.01	25.93
SCE1572_1277	0	0
SCE1572_3432	70.22	225.83
SCE1572_8180	20.78	5.86
SCE1572_2967	0.92	0
SCE1572_10009	41.4	30.01
SCE1572_9257	67.83	9.6
SCE1572_10056	17.93	141.26
SCE1572_9388	89.72	135.59
SCE1572_4596	97.79	574.75
SCE1572_9424	74.58	43.4
SCE1572_7857	37.83	29.61
SCE1572_10178	75.81	243.28
SCE1572_9815	38.36	21.29
SCE1572_2440	31.05	25.39
SCE1572_6435	36.6	8.99
SCE1572_3582	27.66	12.33
SCE1572_1524	128.29	135.68
SCE1572_9381	27.19	50.24
SCE1572_5893	17.54	9.38
SCE1572_3866	75.01	875.81
SCE1572_2238	69.3	479.21
SCE1572_10002	69.96	233.89
SCE1572_6324	57.78	195.22
SCE1572_10873	100.92	24.86
SCE1572_10781	10.46	0
SCE1572_8134	52.57	15.82

SCE1572_6157	48.54	28.81
SCE1572_5316	40.99	19.02
SCE1572_3320	33.46	47.01
SCE1572_11047	26.51	23.51
SCE1572_8178	1.52	0
SCE1572_11448	9.24	4.45
SCE1572_7680	31.76	21.64
SCE1572_3006	2.41	0
SCE1572_3728	55.27	14.13
SCE1572_6511	11.37	7.82
SCE1572_2838	67.23	15.37
SCE1572_930	78.84	639.02
SCE1572_2214	0	0
SCE1572_630	69.88	689.3
SCE1572_607	73.19	767.6
SCE1572_4392	35.75	13.35
SCE1572_1670	109.92	691.07
SCE1572_1760	0	0
SCE1572_7772	18.42	17.09
SCE1572_11335	0	0
SCE1572_672	26.15	75.72
SCE1572_2364	5.89	0
SCE1572_8352	19.36	7.76
SCE1572_5257	26.51	14.04
SCE1572_4202	26.86	31.97
SCE1572_9274	49.4	181.09
SCE1572_4757	33.57	18.25
SCE1572_2529	42.45	36.49
SCE1572_2799	32.75	12.96
SCE1572_7911	47.78	6.63
SCE1572_5853	27.96	15.73
SCE1572_7885	31.34	34.89
SCE1572_7140	0	0
SCE1572_3909	21.26	8.27
SCE1572_3622	80.88	17.24
SCE1572_3549	82.01	28.57
SCE1572_10842	4.13	6.63
SCE1572_10422	34.74	66.89
SCE1572_1704	16.85	0
SCE1572_2200	67.33	100.86
SCE1572_9269	63.55	72.26
SCE1572_7607	266.03	43.93
SCE1572_6278	40.15	92.08
SCE1572_11270	113.13	178.76
SCE1572_3641	34.72	20.1
SCE1572_1448	10.99	7.05
SCE1572_8222	28.08	21.83
SCE1572_6668	34.67	31.91

SCE1572_10391	44.65	17.78
SCE1572_3723	19.58	346.29
SCE1572_5401	19.88	0
SCE1572_2209	17.41	8.11
SCE1572_5051	53.45	31.5
SCE1572_2294	42.16	40
SCE1572_2739	27.86	41.42
SCE1572_9260	15.14	16.54
SCE1572_6238	36.57	11.87
SCE1572_2175	14.44	22.59
SCE1572_10144	32.13	16.34
SCE1572_1793	2.66	0
SCE1572_8299	28.27	6.19
SCE1572_5044	14.3	14.4
SCE1572_11579	61.01	36.16
SCE1572_3989	9.08	14.13
SCE1572_3448	31.5	29.53
SCE1572_2473	131.4	756.04
SCE1572_3614	24.69	14.6
SCE1572_4179	39.16	14.33
SCE1572_217	21.01	20.88
SCE1572_3012	27.69	7.93
SCE1572_7604	13.32	9.16
SCE1572_5446	52.99	19.49
SCE1572_6629	23.17	124.87
SCE1572_11570	23.73	577.53
SCE1572_3537	58.71	23.29
SCE1572_2564	25.27	13.22
SCE1572_7053	68.38	28.62
SCE1572_3293	44.4	30.24
SCE1572_6213	27.69	17.45
SCE1572_5011	20.18	44.84
SCE1572_3067	38.61	31.44
SCE1572_10740	24.78	14.2
SCE1572_9132	69.81	58.86
SCE1572_3161	26.37	63.46
SCE1572_2882	33.24	36.49
SCE1572_726	18.59	7.3
SCE1572_4371	37.66	16.27
SCE1572_7824	16.77	6.34
SCE1572_8143	10.03	0
SCE1572_2954	52.31	23.61
SCE1572_5103	60.52	414.99
SCE1572_9826	42.96	15.21
SCE1572_3579	46.65	158.08
SCE1572_9507	33.28	28.19
SCE1572_1085	75.81	30.6
SCE1572_7861	5.32	0

SCE1572_6231	25.48	14.07
SCE1572_11414	33.91	43.11
SCE1572_6492	26.41	26.7
SCE1572_1886	7.36	3.54
SCE1572_4664	94.76	15.61
SCE1572_9500	19.27	13.43
SCE1572_838	17.96	23.12
SCE1572_4803	31.73	30.54
SCE1572_5874	37.56	43.52
SCE1572_11531	24.23	11.66
SCE1572_8455	16.39	87.78
SCE1572_6926	31.98	5.7
SCE1572_9780	57.14	37.9
SCE1572_3260	143.26	224.13
SCE1572_3777	16.28	12.25
SCE1572_6220	40.57	8.57
SCE1572_1142	32.23	10.34
SCE1572_8822	21.83	5.68
SCE1572_7566	10.83	37.91
SCE1572_3604	95.2	64.86
SCE1572_10374	53.4	57.38
SCE1572_9914	1.92	0
SCE1572_4802	17.71	49.35
SCE1572_8785	16.19	130.19
SCE1572_4750	45.76	80.1
SCE1572_4672	41.03	22.9
SCE1572_3768	32.68	45.09
SCE1572_8251	32.59	23.87
SCE1572_2809	30.25	19.04
SCE1572_9608	51.69	42.85
SCE1572_11139	19.33	10.18
SCE1572_2017	44.65	17
SCE1572_7047	23.94	15.37
SCE1572_7038	32.61	26.96
SCE1572_4149	38.57	17.14
SCE1572_7596	77.74	190.5
SCE1572_10029	16.46	26.19
SCE1572_5074	490.01	179.82
SCE1572_2225	39.73	14.04
SCE1572_11002	15.44	13.52
SCE1572_9094	62.61	632.7
SCE1572_3159	319.19	1263.45
SCE1572_2634	69.43	28.26
SCE1572_11028	33.23	5
SCE1572_6136	75.17	59.28
SCE1572_4496	29.36	38.55
SCE1572_3130	39.21	19.92
SCE1572_8453	19.2	60.82

SCE1572_2427	388.3	511.32
SCE1572_8943	89.69	25.7
SCE1572_6301	55.26	85.96
SCE1572_4897	51.52	51.37
SCE1572_8863	20.79	7.3
SCE1572_3705	0	0
SCE1572_10023	25.75	19.85
SCE1572_7336	43.39	31.57
SCE1572_1113	7.4	22.25
SCE1572_2889	26.03	10.94
SCE1572_5818	55.63	18.4
SCE1572_7916	122.41	324.75
SCE1572_5727	26.99	99.99
SCE1572_2352	45.63	280.45
SCE1572_764	30.55	12.49
SCE1572_2372	49.74	137.01
SCE1572_9220	54.16	23.71
SCE1572_8603	36.43	50.95
SCE1572_10779	81.57	27.74
SCE1572_2831	31.72	21.38
SCE1572_7868	77.13	261.93
SCE1572_4175	88.48	20.64
SCE1572_1591	54.42	69.65
SCE1572_10318	77.93	52.55
SCE1572_1416	37.99	24.27
SCE1572_3031	5.83	0
SCE1572_9916	36.76	26.07
SCE1572_7640	28.02	85.28
SCE1572_229	100.22	510.27
SCE1572_5600	74.17	46.27
SCE1572_8512	118.23	52.62
SCE1572_1206	6.83	1.17
SCE1572_8369	19.17	20.39
SCE1572_4246	77.26	55.78
SCE1572_10798	28.43	11.4
SCE1572_9317	238.32	1187.64
SCE1572_7389	2.71	0
SCE1572_7409	31.95	16.94
SCE1572_6745	71.27	299.42
SCE1572_4875	54.56	30.41
SCE1572_3319	40.36	54.47
SCE1572_865	36.21	81.7
SCE1572_4211	203.23	528.84
SCE1572_3632	41.01	29.1
SCE1572_9026	15.01	326.02
SCE1572_803	3.09	0
SCE1572_222	31.78	18.81
SCE1572_1836	20.49	49.31

SCE1572_313	32.25	7.14
SCE1572_3591	0	0
SCE1572_7031	54.58	33.36
SCE1572_9686	86.33	133.76
SCE1572_9177	23.79	6.42
SCE1572_5427	31.18	62.11
SCE1572_4749	62.47	34.84
SCE1572_7017	0	0
SCE1572_3530	35.47	30.94
SCE1572_11195	17.59	13.1
SCE1572_994	52.12	27.37
SCE1572_5607	26.07	42.62
SCE1572_8574	6.32	10.14
SCE1572_9624	44.47	41.59
SCE1572_4453	34.38	48.8
SCE1572_3702	43.44	24.8
SCE1572_11274	69.11	242.42
SCE1572_9889	14.74	26.36
SCE1572_1850	57.6	43.25
SCE1572_6557	35.4	6.89
SCE1572_2001	33.37	19.21
SCE1572_11105	10.18	16.34
SCE1572_1432	5.51	4.42
SCE1572_2121	8.33	0
SCE1572_697	61.88	47.88
SCE1572_6143	6.74	0
SCE1572_2065	22.18	21
SCE1572_6776	228.46	69.22
SCE1572_5970	7.58	0
SCE1572_2751	54.23	40.44
SCE1572_8576	24.4	6.37
SCE1572_6740	21.21	76.86
SCE1572_8716	39.2	80.1
SCE1572_11251	33.49	11.52
SCE1572_4275	40.49	46.56
SCE1572_10552	0	0
SCE1572_269	48.07	22.25
SCE1572_293	4.53	0
SCE1572_5558	44.28	59.22
SCE1572_2491	113.33	33.17
SCE1572_6291	9.26	9.39
SCE1572_11385	106.53	867.44
SCE1572_10764	139.15	0
SCE1572_9238	27.07	18.25
SCE1572_9193	11.12	1.41
SCE1572_10936	0	0
SCE1572_11469	35.14	86.14
SCE1572_8617	38.04	6.73

SCE1572_9540	36.05	12.98
SCE1572_1968	29.63	46.14
SCE1572_3853	2.76	0
SCE1572_6246	29.76	6.78
SCE1572_9721	77.57	796.52
SCE1572_8165	0	0
SCE1572_6336	69.47	39.81
SCE1572_3572	0	0
SCE1572_1815	14.85	3.14
SCE1572_6007	44.24	89.61
SCE1572_4255	151.62	350.78
SCE1572_2981	24.72	54.21
SCE1572_2170	60.58	82.48
SCE1572_5696	77.48	55.32
SCE1572_1751	29.68	42.48
SCE1572_6198	83.59	16.65
SCE1572_7944	46.03	26.07
SCE1572_5924	24.73	18.79
SCE1572_10451	39.03	379.37
SCE1572_5522	21.96	19.31
SCE1572_4883	23.16	38.01
SCE1572_9188	25.85	18.23
SCE1572_10162	39	12.66
SCE1572_108	116.13	336.19
SCE1572_9728	37.3	47.8
SCE1572_11393	30.35	32.24
SCE1572_3129	21.14	14.04
SCE1572_3652	22.54	19.15
SCE1572_3190	9.57	0
SCE1572_7274	24.44	8.02
SCE1572_11370	31.19	9.5
SCE1572_5704	97.59	104.86
SCE1572_1988	61.39	20.9
SCE1572_4608	80.24	140.45
SCE1572_1075	39.21	32.36
SCE1572_10405	55.96	69.7
SCE1572_4731	24.26	7.3
SCE1572_3712	25.61	1848.48
SCE1572_10747	22.48	11.51
SCE1572_6932	74	1116.49
SCE1572_9057	2.81	0
SCE1572_10886	134.56	34.21
SCE1572_310	14.28	8.18
SCE1572_757	115.52	26.86
SCE1572_11405	22.57	9.73
SCE1572_7090	0	0
SCE1572_9731	64.42	57.18
SCE1572_9585	26.23	21.7

SCE1572_8284	44.47	4.87
SCE1572_10150	21.29	122.34
SCE1572_7339	39.65	800.02
SCE1572_10291	23.23	35.32
SCE1572_7901	43.48	8.94
SCE1572_1171	45.86	24.67
SCE1572_2132	43.54	14.71
SCE1572_11238	15.16	7.3
SCE1572_10496	55.55	80.78
SCE1572_2048	17.78	35.23
SCE1572_905	38.29	12.1
SCE1572_8631	111.02	75.78
SCE1572_7424	13.83	33.29
SCE1572_11132	10.31	4.38
SCE1572_526	43.85	110.46
SCE1572_11075	50.54	444.69
SCE1572_8669	14.61	0
SCE1572_7989	95.65	168.17
SCE1572_10938	20.64	12.16
SCE1572_9286	50.12	87.18
SCE1572_798	143.53	149.21
SCE1572_10348	49.45	17.5
SCE1572_9787	26.37	9.92
SCE1572_9547	49.33	19.67
SCE1572_7350	13.86	2.09
SCE1572_9007	24.92	32.49
SCE1572_7471	0	0
SCE1572_9758	61.03	127.23
SCE1572_8772	78.6	163.68
SCE1572_11453	20.14	22.81
SCE1572_3397	23.44	16.97
SCE1572_6121	11.14	7.31
SCE1572_10302	36.29	7.18
SCE1572_10472	67.06	173.44
SCE1572_1059	42.06	18.83
SCE1572_1186	12.09	12.12
SCE1572_8319	62.4	99.37
SCE1572_2287	44.09	144.93
SCE1572_9113	14.36	55.7
SCE1572_10458	87.14	61.52
SCE1572_5613	45.02	14.32
SCE1572_2900	7.68	9.24
SCE1572_7933	0	0
SCE1572_2289	23.49	179.89
SCE1572_7330	43.4	19.5
SCE1572_7266	0	0
SCE1572_1135	6.79	0
SCE1572_2972	46.52	30.65

SCE1572_10562	74.45	32.39
SCE1572_2848	27.72	10.04
SCE1572_8564	36.1	61.79
SCE1572_985	54.26	49.09
SCE1572_8058	38.51	0
SCE1572_5245	24.68	12.73
SCE1572_4999	108.74	583.33
SCE1572_10538	25.45	5.33
SCE1572_4304	26.11	9.92
SCE1572_8977	11.2	3.6
SCE1572_421	10.83	39.1
SCE1572_4115	57.76	63.72
SCE1572_1952	25.2	12.34
SCE1572_10717	14.57	8.35
SCE1572_8677	34.51	60.4
SCE1572_2002	29.74	66.53
SCE1572_480	28.01	21.98
SCE1572_1991	51.3	15.3
SCE1572_53	67	26.31
SCE1572_2160	43.71	18.65
SCE1572_3055	16.02	0
SCE1572_5262	0	0
SCE1572_1052	105.64	535.24
SCE1572_7370	43.93	24.33
SCE1572_6484	18.95	7.84
SCE1572_8753	28.39	7.74
SCE1572_11089	22.19	42.52
SCE1572_9475	55.45	116.92
SCE1572_5081	3337.38	4102.08
SCE1572_6583	34.9	31.67
SCE1572_8969	18.65	70.96
SCE1572_7158	36.41	39.24
SCE1572_4491	31.72	172.11
SCE1572_11343	31.66	156.4
SCE1572_10356	52.63	357.36
SCE1572_5222	39.43	27.77
SCE1572_1514	29.27	7.36
SCE1572_8982	29.01	17.13
SCE1572_6069	86.29	390.16
SCE1572_7323	4.94	0
SCE1572_4100	23.74	24.12
SCE1572_5965	21.4	20.39
SCE1572_9041	247.77	1838.83
SCE1572_3329	85.79	570.82
SCE1572_4500	21.16	5.66
SCE1572_6568	20.89	70.4
SCE1572_11446	94.34	131.08
SCE1572_9590	50.32	402.73

SCE1572_1972	28.41	105.2
SCE1572_4980	27.64	98.83
SCE1572_5643	0	0
SCE1572_4284	53.53	22.53
SCE1572_9468	23.84	54.79
SCE1572_6148	42.33	12.97
SCE1572_9306	40.6	78.9
SCE1572_8337	45.13	44.72
SCE1572_5632	18.28	40.79
SCE1572_9599	40.32	22
SCE1572_11353	3.37	0
SCE1572_7109	30.98	19.62
SCE1572_9570	58.97	20.03
SCE1572_3955	63.58	28.98
SCE1572_3798	22.57	31.57
SCE1572_2689	82.51	237.62
SCE1572_5344	45.75	21.59
SCE1572_3999	255.23	525.36
SCE1572_590	37.25	18.11
SCE1572_4501	52.34	232.46
SCE1572_3817	60.09	694.53
SCE1572_878	83.62	485.31
SCE1572_731	6.46	4.79
SCE1572_3940	335.21	662.17
SCE1572_2815	29.16	44.66
SCE1572_4126	62.77	23.24
SCE1572_8960	55.89	32.91
SCE1572_11282	34.28	80.27
SCE1572_4414	35.48	19.41
SCE1572_2231	13.55	93.78
SCE1572_165	21.91	7.54
SCE1572_3744	52.07	114.27
SCE1572_4195	35.62	74.14
SCE1572_307	0	0
SCE1572_7379	32.08	13.63
SCE1572_3327	289.1	2603.94
SCE1572_10635	65.08	168.69
SCE1572_5796	27.42	27.18
SCE1572_3902	20.42	98.31
SCE1572_7754	44.15	114.65
SCE1572_5504	31.72	27
SCE1572_546	0	0
SCE1572_10082	134.06	1006.42
SCE1572_7516	18.95	30.41
SCE1572_7231	52.48	94.42
SCE1572_3082	24.21	19.42
SCE1572_8537	26.59	26.75
SCE1572_8141	28.17	8.65

SCE1572_3560	53.47	13.68
SCE1572_3847	25.07	9.73
SCE1572_2740	14.08	9.97
SCE1572_7965	14.56	23.36
SCE1572_8234	0	0
SCE1572_6760	112.7	120.79
SCE1572_3166	48.93	30.99
SCE1572_6766	0	0
SCE1572_9459	0	0
SCE1572_34	58.78	217.83
SCE1572_7554	10.38	2
SCE1572_303	33.19	13.7
SCE1572_6874	116.57	1750.22
SCE1572_10915	0	0
SCE1572_6341	57.15	22.74
SCE1572_2105	157.27	812.91
SCE1572_2664	201.98	1877.65
SCE1572_9443	21.85	42.74
SCE1572_2920	44.66	11.27
SCE1572_2183	14.75	8.13
SCE1572_2854	0	0
SCE1572_6406	14.63	105.64
SCE1572_5666	11.73	7.6
SCE1572_10594	52.4	23.69
SCE1572_2917	10.4	4.81
SCE1572_10354	15.64	11.58
SCE1572_10143	39.11	159.29
SCE1572_1066	37.74	14.1
SCE1572_9553	42.35	17.73
SCE1572_4886	4.27	0
SCE1572_6352	55.82	54.47
SCE1572_9157	96.06	76.05
SCE1572_3310	18.31	6.15
SCE1572_9478	103.59	778.58
SCE1572_2776	0	0
SCE1572_2331	46.27	30.84
SCE1572_2714	44.56	58.86
SCE1572_2941	33.6	22.81
SCE1572_9484	179.73	778.94
SCE1572_3096	3.39	2.72
SCE1572_5987	15.34	12.21
SCE1572_10614	27.05	5.92
SCE1572_4465	42.08	61.85
SCE1572_193	31.7	46.2
SCE1572_2093	31.26	33.86
SCE1572_6253	38.08	221.37
SCE1572_9454	21.08	12.55
SCE1572_1820	21.78	30.24

SCE1572_1810	27.31	22.81
SCE1572_10923	239.67	19913.2
SCE1572_2498	0	0
SCE1572_8754	72.03	21.67
SCE1572_1803	20.53	15.21
SCE1572_7630	50.94	297.78
SCE1572_10548	40.91	14.45
SCE1572_489	16.04	8.18
SCE1572_10189	20.34	49.92
SCE1572_4865	34.04	81.92
SCE1572_7981	27.27	39.38
SCE1572_2674	22.97	125.33
SCE1572_3115	32.96	15.87
SCE1572_5906	57.3	335.37
SCE1572_11071	69.94	75.81
SCE1572_10102	31.15	115.98
SCE1572_5099	21.52	4.78
SCE1572_3962	33.01	24.54
SCE1572_7714	50.28	179.71
SCE1572_2871	0	0
SCE1572_8872	35.42	16.45
SCE1572_6663	15.57	9.71
SCE1572_11473	47.58	32.06
SCE1572_4831	62.25	114.64
SCE1572_8580	202.8	88.75
SCE1572_8506	19.51	7.23
SCE1572_4740	52.47	80.13
SCE1572_9775	0	0
SCE1572_4067	41.75	50.24
SCE1572_10732	62.5	32.02
SCE1572_7508	40.78	15.18
SCE1572_9665	22.89	91.8
SCE1572_10647	23.94	65.3
SCE1572_5069	24.24	20.97
SCE1572_2406	31.03	229.73
SCE1572_10074	154.06	0
SCE1572_2825	17.49	14.04
SCE1572_9558	0	0
SCE1572_6182	26.77	8.36
SCE1572_156	62.12	191.33
SCE1572_1825	14.59	9.16
SCE1572_7739	6.41	0
SCE1572_4260	34.84	33.13
SCE1572_5621	65.42	27.39
SCE1572_1774	27.47	7.93
SCE1572_2860	42.22	13.86
SCE1572_7088	38.78	14.68
SCE1572_6424	0	0

SCE1572_6175	53.09	88.47
SCE1572_6616	25.31	10.05
SCE1572_8736	21.55	16.67
SCE1572_2720	90.17	20.54
SCE1572_2055	19.16	25.78
SCE1572_6778	260.44	111.83
SCE1572_9517	62.25	103.44
SCE1572_1616	9.88	40.24
SCE1572_7100	10.71	28.37
SCE1572_449	19.28	16.01
SCE1572_4443	28.43	108.34
SCE1572_9766	27.67	69.26
SCE1572_6644	2.53	0
SCE1572_331	55.57	157.72
SCE1572_10569	122.34	211.37
SCE1572_4335	42.39	23.35
SCE1572_4796	148.17	77.41
SCE1572_10791	56.95	78.73
SCE1572_2679	24.19	638.97
SCE1572_4217	32.49	45.79
SCE1572_9525	9.05	25.42
SCE1572_9072	31.5	47.6
SCE1572_41	59.81	45.75
SCE1572_1128	30.04	26.07
SCE1572_1523	32.59	11.27
SCE1572_5212	40.15	81.38
SCE1572_9882	74.59	60.5
SCE1572_3480	44.13	50.01
SCE1572_9973	62.4	239.05
SCE1572_11074	7	0
SCE1572_6681	13.61	74.57
SCE1572_1122	71.3	15.05
SCE1572_5346	3.42	5.49
SCE1572_11532	0	0
SCE1572_4573	20.67	15.55
SCE1572_9154	39.34	138.92
SCE1572_2707	31.17	10.14
SCE1572_10551	29.48	11.26
SCE1572_8774	129.51	297.39
SCE1572_1851	59.42	36.99
SCE1572_8666	19.11	1.48
SCE1572_2665	58.14	439.32
SCE1572_5604	41.09	48.86
SCE1572_8623	72.09	228.59
SCE1572_8259	125.04	368.07
SCE1572_861	49.98	743.36
SCE1572_2416	114.57	137.19
SCE1572_8975	14.66	9.07

SCE1572_7268	37.14	76.18
SCE1572_1614	46.92	126
SCE1572_7725	33.69	31.81
SCE1572_2596	82.69	94.85
SCE1572_9387	73.1	152.74
SCE1572_7112	0	0
SCE1572_6111	18.63	51.03
SCE1572_167	73	21.63
SCE1572_2678	20.59	272.57
SCE1572_6643	172.05	176.15
SCE1572_6637	35	40.08
SCE1572_8288	13.24	38.69
SCE1572_2929	0	0
SCE1572_3951	32.87	44.9
SCE1572_9480	34.17	41.12
SCE1572_7250	44.21	17.69
SCE1572_4312	41.27	14.49
SCE1572_9416	109.5	2275.13
SCE1572_3621	20.91	3.15
SCE1572_2024	14.16	5.38
SCE1572_5793	127.43	220.52
SCE1572_6115	71.31	20.19
SCE1572_9350	114.93	509.88
SCE1572_5673	25.57	31.48
SCE1572_2856	23.43	8.85
SCE1572_3499	35.48	49.17
SCE1572_2469	10.69	7.45
SCE1572_804	0	0
SCE1572_10588	13.18	3.53
SCE1572_4913	19.87	10.07
SCE1572_1784	29.08	10
SCE1572_9077	33.97	16.22
SCE1572_8235	30.32	44.6
SCE1572_10176	126.83	77.2
SCE1572_4817	164.5	1485.93
SCE1572_10596	51.71	19.8
SCE1572_6146	59.76	35.96
SCE1572_1779	52.71	72.04
SCE1572_7045	24.79	124.32
SCE1572_3412	25.73	85.37
SCE1572_3708	0	0
SCE1572_11508	20.67	9.22
SCE1572_11472	8.53	0
SCE1572_5983	6.67	8.76
SCE1572_7511	46.69	23.17
SCE1572_8462	67.01	667.34
SCE1572_5626	11.52	4.38
SCE1572_1916	7	0

SCE1572_10664	50.77	369.88
SCE1572_3580	37.46	16.33
SCE1572_2405	29.55	239.61
SCE1572_10352	28.24	19.87
SCE1572_6056	148.87	465.05
SCE1572_1248	82.91	181.78
SCE1572_9995	41.57	21.34
SCE1572_564	59.87	1338.82
SCE1572_797	97.04	157.99
SCE1572_7026	43.62	18.83
SCE1572_8144	35.05	7.85
SCE1572_3413	50.09	58.65
SCE1572_8743	4.33	0
SCE1572_2098	46.65	110.2
SCE1572_4455	122.5	126.36
SCE1572_9751	39.14	24.73
SCE1572_11358	45.73	28
SCE1572_9520	51	147.35
SCE1572_9879	32.88	63.02
SCE1572_2996	18.1	9.14
SCE1572_5082	82.81	159.75
SCE1572_9776	19.99	28.07
SCE1572_3796	8.93	1.57
SCE1572_6769	324.79	4879.58
SCE1572_2572	50.28	17.16
SCE1572_8883	56.86	25.78
SCE1572_4798	60.35	683.79
SCE1572_10929	55.24	39.6
SCE1572_179	15.3	11.54
SCE1572_10389	36.73	49.41
SCE1572_4016	112.63	93.84
SCE1572_6138	32.2	48.44
SCE1572_2731	61.2	58.32
SCE1572_2712	267.07	865.74
SCE1572_10072	402.87	1115.62
SCE1572_10194	27	14.04
SCE1572_4329	22.28	24.48
SCE1572_6407	17.14	55.59
SCE1572_7776	47.68	245
SCE1572_9769	11.04	19.33
SCE1572_6501	35.11	19.21
SCE1572_10399	58.06	70.72
SCE1572_10992	6.42	12.37
SCE1572_9398	28.91	71.13
SCE1572_10415	18.83	38.53
SCE1572_5310	22.66	33.56
SCE1572_1335	26.49	10.99
SCE1572_4267	0	0

SCE1572_2142	34.44	9.4
SCE1572_6177	51.47	68.68
SCE1572_1014	45.35	32.58
SCE1572_9544	65.8	18.93
SCE1572_9324	644.51	1002.74
SCE1572_4324	30.16	21.58
SCE1572_26	39.32	58.14
SCE1572_2978	21.42	3.97
SCE1572_9034	27.09	18.04
SCE1572_10100	47.83	26.94
SCE1572_11460	187.27	464.54
SCE1572_1322	10.85	3.07
SCE1572_10149	27.36	7.12
SCE1572_5118	54.15	161.61
SCE1572_10916	39.01	31.46
SCE1572_988	33.46	85.51
SCE1572_8428	21.96	17.05
SCE1572_3011	37.05	8.76
SCE1572_484	38.08	24.84
SCE1572_7809	0	0
SCE1572_7723	40.86	53.06
SCE1572_10305	43.32	29.41
SCE1572_6737	116.01	728.37
SCE1572_6717	86.92	254.82
SCE1572_544	53.51	52.13
SCE1572_6710	109.15	91.89
SCE1572_11584	31.47	16.75
SCE1572_9489	39.08	26.52
SCE1572_11244	25.17	57.62
SCE1572_10290	25.87	13.84
SCE1572_5615	51.04	12.16
SCE1572_10069	136.01	21.47
SCE1572_10708	47.6	29.7
SCE1572_6	18.37	24.81
SCE1572_6441	11.66	0
SCE1572_7982	33.35	20.81
SCE1572_9735	92.13	161.08
SCE1572_3511	39.47	65.84
SCE1572_9452	153.4	84.43
SCE1572_9305	26.79	48.02
SCE1572_2708	109.44	27.68
SCE1572_2915	35.69	10.9
SCE1572_3956	34.63	12.04
SCE1572_766	13.02	3.69
SCE1572_1106	38.21	7.83
SCE1572_3949	27.24	142.37
SCE1572_1396	15.67	187.33
SCE1572_3068	46.78	108.53

SCE1572_4440	55.86	32.57
SCE1572_4413	9.7	0
SCE1572_9837	106.5	238.65
SCE1572_102	21.79	7.98
SCE1572_10925	0	0
SCE1572_2380	21.19	19.12
SCE1572_4904	28.93	402.53
SCE1572_4333	7	3.74
SCE1572_11140	30.63	24.99
SCE1572_6648	4.56	0
SCE1572_8330	33.55	210.2
SCE1572_8223	10.01	13.77
SCE1572_5191	51.14	182.94
SCE1572_6614	77.79	42.03
SCE1572_11443	117.02	78.37
SCE1572_1947	71.48	45.62
SCE1572_3059	10.46	1.8
SCE1572_3271	40.92	443.79
SCE1572_1838	81.58	401.32
SCE1572_8965	37.9	66.26
SCE1572_691	13.39	4.2
SCE1572_10574	105.75	129.87
SCE1572_11233	109.92	290.11
SCE1572_3598	75.57	23.52
SCE1572_8510	37.47	16.78
SCE1572_4747	91.06	30.41
SCE1572_9693	44.46	856.97
SCE1572_4081	16.68	22.81
SCE1572_9482	15.26	29.39
SCE1572_1078	31.57	19.44
SCE1572_853	42.33	199.05
SCE1572_7931	64.56	54.14
SCE1572_7300	125.13	57.16
SCE1572_6196	16.72	6.94
SCE1572_3634	31.17	17.8
SCE1572_1994	47.28	17.4
SCE1572_7958	99.73	292.72
SCE1572_6350	35.06	43.24
SCE1572_9487	36.18	55.98
SCE1572_10210	104.24	19.77
SCE1572_10728	26.44	63.63
SCE1572_3321	54.31	98.48
SCE1572_3552	53.07	20.85
SCE1572_10375	67.31	17.05
SCE1572_6785	0	0
SCE1572_6347	38.96	42.81
SCE1572_6126	21.9	95.11
SCE1572_9117	59.41	34.14

SCE1572_1992	0	0
SCE1572_5265	0	0
SCE1572_8832	9.72	9.36
SCE1572_10721	92.9	41.14
SCE1572_1452	0	0
SCE1572_9722	576.27	5983.35
SCE1572_7961	32.16	0
SCE1572_10299	28.4	89.68
SCE1572_9069	45.43	250.12
SCE1572_7864	57.55	72.98
SCE1572_1776	23.81	12.06
SCE1572_3052	35.8	10.14
SCE1572_10792	41.35	65.02
SCE1572_8700	35.16	27.5
SCE1572_1025	44.44	1113.64
SCE1572_7309	54.26	17.29
SCE1572_2283	21.02	10.84
SCE1572_2775	39.47	118.76
SCE1572_5505	1.6	7.68
SCE1572_7166	27.42	7.95
SCE1572_3093	159.48	129.75
SCE1572_680	11.06	22.81
SCE1572_5535	39.3	12.43
SCE1572_3846	26.99	6.91
SCE1572_4351	34.95	11.65
SCE1572_11084	15.07	47.6
SCE1572_1219	15.79	12.35
SCE1572_209	9.9	5.62
SCE1572_2003	124.3	56.35
SCE1572_219	29.11	105.1
SCE1572_2523	2.57	0
SCE1572_5317	5.62	0
SCE1572_8583	85.65	35.05
SCE1572_8252	36.54	107.08
SCE1572_5936	18.07	12.66
SCE1572_128	9.48	0
SCE1572_7787	80.27	0
SCE1572_3486	84.07	42.11
SCE1572_1137	76.39	35.87
SCE1572_8272	15.16	22.59
SCE1572_9391	40.34	25.11
SCE1572_6400	30.56	111.46
SCE1572_4145	50.79	155.38
SCE1572_3398	178.37	42.93
SCE1572_9008	9.83	3.64
SCE1572_9888	35.95	20.78
SCE1572_7155	26.49	13.42
SCE1572_8527	20.44	0

SCE1572_7583	190.36	1212.19
SCE1572_6741	61.48	29.55
SCE1572_9788	74.01	64.78
SCE1572_7745	1308.39	6305.05
SCE1572_7322	18.76	3.66
SCE1572_5966	14.84	8.69
SCE1572_4513	3.79	4.56
SCE1572_6135	36.4	66.35
SCE1572_8735	0	0
SCE1572_223	16.28	3.67
SCE1572_6921	38.33	32.44
SCE1572_11078	22.01	11.77
SCE1572_3316	43.36	44.44
SCE1572_1866	17.71	17.05
SCE1572_4730	27.96	16.53
SCE1572_8279	26.71	8.29
SCE1572_7474	102.54	221.48
SCE1572_9979	51.69	20.14
SCE1572_3714	80.07	153.76
SCE1572_908	12.09	6.72
SCE1572_1630	109.82	44.45
SCE1572_6032	34.24	58.86
SCE1572_11080	34.51	400.52
SCE1572_8535	65.55	40.95
SCE1572_8293	20.28	12.2
SCE1572_912	47.03	144.23
SCE1572_1313	836.9	3508.26
SCE1572_998	39.11	70.89
SCE1572_8054	11.03	6.63
SCE1572_2492	61.54	35.58
SCE1572_10933	42.12	0
SCE1572_3046	12.2	2.26
SCE1572_11451	13.86	7.85
SCE1572_3223	9.68	2.59
SCE1572_4307	29.5	25.96
SCE1572_7788	0	0
SCE1572_4699	24.64	11.4
SCE1572_10343	21.31	121.25
SCE1572_6041	85.38	133.48
SCE1572_5665	15.78	8.44
SCE1572_897	68.14	32.8
SCE1572_9027	35.79	59.84
SCE1572_4286	0	0
SCE1572_10669	77.23	332.12
SCE1572_2546	48.52	26.27
SCE1572_2671	111.35	205.52
SCE1572_7345	18.74	4.1
SCE1572_10565	64.31	27.21

SCE1572_3559	26.04	31.34
SCE1572_8242	51.6	198.7
SCE1572_1504	52.53	73.62
SCE1572_4861	10	4.89
SCE1572_5771	3.3	0
SCE1572_314	26.28	10.95
SCE1572_2324	19.72	38.57
SCE1572_6762	0	0
SCE1572_890	20.05	39.21
SCE1572_10301	30.19	12.78
SCE1572_11388	297.79	529.61
SCE1572_6519	28.64	7.66
SCE1572_2069	42.5	18.5
SCE1572_2746	25.75	2.3
SCE1572_11196	47.72	46.83
SCE1572_1821	13.92	17.63
SCE1572_2114	194.39	176.14
SCE1572_9211	9.36	15.03
SCE1572_11349	0	0
SCE1572_2554	27.16	12.06
SCE1572_3858	22.18	64.27
SCE1572_9070	27.78	22.05
SCE1572_10187	11.82	5.02
SCE1572_5923	61.9	76.38
SCE1572_5708	39.78	34.19
SCE1572_9711	54.53	220.56
SCE1572_5421	22.3	119.4
SCE1572_597	91.92	285.9
SCE1572_10639	43.27	259.97
SCE1572_2583	13.78	3.32
SCE1572_6089	7.82	47.65
SCE1572_9597	35.03	1735.56
SCE1572_2134	0	0
SCE1572_7261	0	0
SCE1572_2060	18.95	22.81
SCE1572_5979	12.8	37.3
SCE1572_1770	44.12	21.24
SCE1572_9335	33.44	150.91
SCE1572_4256	35.74	216.52
SCE1572_4992	34.99	116.29
SCE1572_10969	74.48	150.81
SCE1572_9281	147.21	553.35
SCE1572_8675	34.31	26.64
SCE1572_2687	28.22	30.19
SCE1572_689	12.78	19.57
SCE1572_4192	25.41	68.42
SCE1572_9689	137.05	240.63
SCE1572_4611	16.26	10.58

SCE1572_7971	6.05	8.32
SCE1572_7479	45.69	38.09
SCE1572_1071	30.43	13.65
SCE1572_6228	67.7	25.53
SCE1572_9172	38.53	14.11
SCE1572_8447	20.34	0
SCE1572_7751	42.47	20.56
SCE1572_3231	31.94	115.87
SCE1572_319	40.18	14.4
SCE1572_4349	40.46	19.29
SCE1572_7623	33.04	45.75
SCE1572_11025	38.84	64.72
SCE1572_4122	35.73	34.87
SCE1572_9586	33.72	61.03
SCE1572_6002	17.33	96.97
SCE1572_1410	41.19	20.11
SCE1572_372	32.54	160.08
SCE1572_10142	31.34	64.4
SCE1572_10883	25.49	280.96
SCE1572_9053	66.45	26.52
SCE1572_1112	7.15	8.61
SCE1572_6833	18.4	19.69
SCE1572_8752	34.07	12.3
SCE1572_4129	63.34	191.15
SCE1572_8594	17.38	29.13
SCE1572_2888	29.29	13.17
SCE1572_8728	48.48	22.28
SCE1572_8069	24.91	3.07
SCE1572_10323	26.7	53.94
SCE1572_9265	31.38	43.2
SCE1572_8305	35.08	22.62
SCE1572_10479	56.47	57.72
SCE1572_6326	18.01	10.84
SCE1572_3686	19.05	0
SCE1572_5835	475.64	703.96
SCE1572_4669	72.81	14.43
SCE1572_4828	84.83	34.75
SCE1572_262	65.06	25.87
SCE1572_8142	14.95	2.57
SCE1572_5288	18.73	15.62
SCE1572_9477	0	0
SCE1572_2442	83.58	458.49
SCE1572_8581	101.3	25.59
SCE1572_9227	20.48	23.67
SCE1572_734	25.08	11.79
SCE1572_8091	6.65	0
SCE1572_11486	35.99	64.51
SCE1572_4043	51.07	305.37

SCE1572_8393	44.13	38.28
SCE1572_2342	38.19	139.28
SCE1572_3732	16.55	15.93
SCE1572_3772	59.44	78.78
SCE1572_6517	12.57	62.4
SCE1572_3354	40.9	42.76
SCE1572_4528	0	0
SCE1572_11181	35.35	54
SCE1572_3077	38.04	17.11
SCE1572_8553	32.3	420.45
SCE1572_11102	44.48	84.44
SCE1572_7609	32.75	43.79
SCE1572_4553	17.6	1.63
SCE1572_5388	106.6	443.8
SCE1572_8283	17	3.23
SCE1572_661	0	0
SCE1572_8098	16.29	678.57
SCE1572_3301	22.34	20.59
SCE1572_1983	0	0
SCE1572_6855	17.07	38.67
SCE1572_7914	28.05	17.61
SCE1572_1145	17.05	4.32
SCE1572_3147	33.5	4.24
SCE1572_7329	19.17	2.1
SCE1572_6250	0	0
SCE1572_7315	12.22	6.92
SCE1572_7938	20.14	62.72
SCE1572_11420	39.52	39.08
SCE1572_9869	120.41	540.51
SCE1572_1089	44.64	30.19
SCE1572_7606	61.39	51.43
SCE1572_6451	37.04	18.6
SCE1572_4199	16.44	46.16
SCE1572_4800	52.77	1243.24
SCE1572_9860	48.93	34.75
SCE1572_6247	14.41	9.08
SCE1572_3133	71.39	44.77
SCE1572_8456	40.08	434.65
SCE1572_11417	50.98	41.43
SCE1572_1204	0	0
SCE1572_1030	37.11	188.84
SCE1572_1146	139.39	94.17
SCE1572_9504	12.36	3.22
SCE1572_11484	8.15	0
SCE1572_8195	52.34	48.99
SCE1572_1921	0	0
SCE1572_1699	43.75	111.35
SCE1572_3245	35.72	26.52

SCE1572_3852	38.61	67.58
SCE1572_10844	13.33	8.02
SCE1572_4227	23.15	26.19
SCE1572_10073	161.95	682.85
SCE1572_4666	37.33	5.5
SCE1572_8839	22.46	0
SCE1572_8366	39.51	8.99
SCE1572_7833	43.72	64.92
SCE1572_7436	6.38	4.72
SCE1572_8213	37.11	49.23
SCE1572_9311	81.67	371.97
SCE1572_7567	17.94	8.22
SCE1572_1082	20.52	3.61
SCE1572_5537	38.52	26.49
SCE1572_6535	30.78	22.12
SCE1572_934	42.58	177.96
SCE1572_11564	6.47	5.19
SCE1572_11263	22.61	53.35
SCE1572_11020	9.72	6.93
SCE1572_2533	19.22	336.65
SCE1572_5489	0	0
SCE1572_4809	56.5	52.24
SCE1572_33	408.2	2198.17
SCE1572_6300	75.06	277.27
SCE1572_9607	14.86	4.77
SCE1572_11524	23.42	11.87
SCE1572_10753	35.33	7.79
SCE1572_115	31.15	11.66
SCE1572_6102	28.11	8.2
SCE1572_6410	0	0
SCE1572_8792	46.54	56.01
SCE1572_610	44.78	72.37
SCE1572_1973	0	0
SCE1572_2828	0	0
SCE1572_136	36.48	11.64
SCE1572_3375	20.24	67.08
SCE1572_5690	26.65	41.34
SCE1572_2172	58.62	4.87
SCE1572_6082	60.92	9.77
SCE1572_5041	126.86	441.26
SCE1572_10895	93.23	179.06
SCE1572_5325	27.83	33.49
SCE1572_9645	74.04	1005.66
SCE1572_7780	14.89	6.52
SCE1572_1037	2.61	0
SCE1572_1903	90.24	29.03
SCE1572_5816	21.1	10.33
SCE1572_7143	31.59	9.68

SCE1572_11435	39.94	30.86
SCE1572_2732	45.87	90.82
SCE1572_11223	21.66	13.9
SCE1572_4830	34.99	52.93
SCE1572_452	9.7	67.15
SCE1572_1556	43.7	70.12
SCE1572_159	39.1	70.43
SCE1572_4894	55.32	21.37
SCE1572_4109	19.42	8.09
SCE1572_5852	112	118.35
SCE1572_6473	124.78	248.31
SCE1572_3648	28.55	137.42
SCE1572_1719	68.57	89.86
SCE1572_2205	20.31	202.01
SCE1572_606	60.38	108.44
SCE1572_2179	22.71	68.75
SCE1572_4041	123.12	1594.3
SCE1572_2563	34.19	17.33
SCE1572_12	94.23	335.78
SCE1572_1252	23.77	10.44
SCE1572_1460	15.47	11.17
SCE1572_10773	22.92	27.58
SCE1572_2216	40.26	216.44
SCE1572_2645	5.27	0
SCE1572_147	21.45	19.8
SCE1572_3844	52.82	293.99
SCE1572_4030	182.21	422.31
SCE1572_1461	44.74	25.03
SCE1572_2588	27.24	10.6
SCE1572_7646	37.47	51.7
SCE1572_725	24.48	23.78
SCE1572_4884	12.09	27.77
SCE1572_7828	45.94	118.88
SCE1572_6491	8.92	0
SCE1572_4037	11.21	17.04
SCE1572_1414	57.34	21.05
SCE1572_4502	96.71	39.45
SCE1572_1298	153.66	64.11
SCE1572_1705	82.83	97.96
SCE1572_664	84.12	279.17
SCE1572_3423	8.58	0
SCE1572_3008	50.54	27.31
SCE1572_3034	69.22	174.53
SCE1572_3823	24.16	44.11
SCE1572_10890	45.75	34.6
SCE1572_1498	88.95	19.48
SCE1572_7798	9.02	11.99
SCE1572_2603	20.29	2.76

SCE1572_2181	45.41	29.64
SCE1572_2079	19.44	13.1
SCE1572_4839	59.3	126.51
SCE1572_10006	17.84	21.47
SCE1572_9960	24.17	12.21
SCE1572_6292	40.86	24.59
SCE1572_1231	44.07	182.13
SCE1572_617	175.56	775.94
SCE1572_6587	44.65	29.44
SCE1572_3757	38.97	12.49
SCE1572_1551	25	10.68
SCE1572_5046	0	0
SCE1572_6159	8.58	0.32
SCE1572_1801	33.23	284.94
SCE1572_4739	139.32	32.05
SCE1572_6496	25.46	10.1
SCE1572_5907	1111.85	1060.98
SCE1572_7900	40.31	28.96
SCE1572_3864	8.13	3.15
SCE1572_6811	89.24	62.46
SCE1572_10812	21.86	9.19
SCE1572_10554	43.19	15.91
SCE1572_1799	20.58	234.42
SCE1572_2241	35.52	24.17
SCE1572_1585	63.17	330.93
SCE1572_10534	55.38	253.48
SCE1572_8384	17.12	7.85
SCE1572_1270	51.85	185.9
SCE1572_2499	59.91	29.43
SCE1572_8329	36.79	24.28
SCE1572_1592	83.89	97.58
SCE1572_3563	74.82	43.33
SCE1572_9465	6.69	0
SCE1572_3991	199.47	1821.29
SCE1572_2879	66.04	125.61
SCE1572_3182	51.7	247.67
SCE1572_3568	35.27	9.23
SCE1572_553	71.07	117.84
SCE1572_1365	104.39	2627.74
SCE1572_4509	42	113.42
SCE1572_10424	64.32	24.33
SCE1572_5073	58.24	596.62
SCE1572_4429	24.78	47.46
SCE1572_9101	23.73	59.68
SCE1572_9859	46.55	44.01
SCE1572_6936	8.78	4.6
SCE1572_10057	56.86	47.58
SCE1572_9773	28.93	30.85

SCE1572_5078	43.66	291.36
SCE1572_10819	34.82	66.9
SCE1572_7599	1892.17	4688.52
SCE1572_2049	27.47	69.93
SCE1572_8939	27.34	340.99
SCE1572_3985	42.44	127.92
SCE1572_334	27.57	62.2
SCE1572_1884	30.16	74.55
SCE1572_10746	10.55	3.17
SCE1572_6106	20.11	6.54
SCE1572_5105	120.08	1460.3
SCE1572_11334	126.22	277.86
SCE1572_11412	59.29	209.36
SCE1572_7394	16.02	1.71
SCE1572_13	50.09	30.41
SCE1572_9423	41.65	56.14
SCE1572_10803	17.2	10.35
SCE1572_10429	44.26	15.87
SCE1572_1812	31.22	14.57
SCE1572_11576	25.89	51.18
SCE1572_2607	28.67	18.29
SCE1572_1525	86.64	428.42
SCE1572_8651	15.05	2.59
SCE1572_5999	34.51	315.85
SCE1572_11259	57.26	556.62
SCE1572_4917	10.63	3.34
SCE1572_10130	24.67	20.11
SCE1572_4485	25.48	110.4
SCE1572_1530	0	0
SCE1572_2805	20.44	20.83
SCE1572_428	36.28	92.53
SCE1572_8471	160.93	458.62
SCE1572_870	12.88	33.39
SCE1572_4449	2.23	0
SCE1572_11041	64.51	78.1
SCE1572_3616	35.63	17.95
SCE1572_3464	57.35	27.32
SCE1572_5397	31.36	15.98
SCE1572_6024	76.28	73.44
SCE1572_3335	27.97	61.28
SCE1572_5644	25.6	2.37
SCE1572_11121	53.25	26.07
SCE1572_2195	79.64	81.09
SCE1572_4103	39.9	16.01
SCE1572_2973	2.97	0
SCE1572_785	39.04	48.35
SCE1572_5699	19.76	96.04
SCE1572_6767	16.68	7.3

SCE1572_4685	59.58	80.18
SCE1572_7768	97.11	93.97
SCE1572_1465	28.9	13.43
SCE1572_9093	9.23	1.06
SCE1572_2489	6.93	0
SCE1572_4170	0	0
SCE1572_5381	27.44	9.91
SCE1572_6448	95.43	44.71
SCE1572_9476	15.58	4.33
SCE1572_10901	16.22	25.37
SCE1572_4534	29.13	30.56
SCE1572_2834	336.38	3757.74
SCE1572_8658	60.34	352.21
SCE1572_8707	54.66	43.44
SCE1572_7018	31.54	9.8
SCE1572_5990	77.92	55.92
SCE1572_9156	56.53	144.84
SCE1572_2959	69.89	31.06
SCE1572_7094	0	0
SCE1572_11529	33.13	10.27
SCE1572_2226	46.85	16.47
SCE1572_5235	31.76	322.4
SCE1572_7076	18.29	20.69
SCE1572_2296	48.09	130.8
SCE1572_9566	0	0
SCE1572_5890	29.58	21.73
SCE1572_4942	15.96	90.79
SCE1572_4978	50.4	720.59
SCE1572_1441	39.7	15.05
SCE1572_10905	159.95	232.59
SCE1572_11109	92.91	105.64
SCE1572_10066	180.66	847.51
SCE1572_11275	80.21	403.53
SCE1572_7592	32.73	27.07
SCE1572_7821	33.22	187.55
SCE1572_1492	52.61	21.5
SCE1572_8871	67.76	52.48
SCE1572_8300	64.64	8.49
SCE1572_10641	17.01	19.88
SCE1572_1763	47.51	3.4
SCE1572_1797	45.27	35.48
SCE1572_2559	40.95	25.3
SCE1572_4138	37.02	12.73
SCE1572_4270	56.02	20.52
SCE1572_9125	12.88	10.73
SCE1572_11171	30.59	3.2
SCE1572_7791	14.18	2.35
SCE1572_927	19.65	7.72

SCE1572_10962	100.86	37.59
SCE1572_9003	186.76	775.76
SCE1572_9136	19.04	5.09
SCE1572_6706	565.79	1973.7
SCE1572_292	10.11	82.11
SCE1572_10485	55.9	257.48
SCE1572_5093	97.59	1157.68
SCE1572_1272	7.71	12.37
SCE1572_5100	27.26	47.15
SCE1572_9178	162.76	456.15
SCE1572_11063	26.34	19.73
SCE1572_305	42.51	22.17
SCE1572_2112	2.45	0
SCE1572_10394	0	0
SCE1572_10835	89.66	27.88
SCE1572_10624	44.59	136.31
SCE1572_9740	77.7	403.88
SCE1572_7372	16.93	22.49
SCE1572_1677	3.72	2.24
SCE1572_9363	26.03	65.78
SCE1572_8905	9.1	3.65
SCE1572_11129	54.25	62.61
SCE1572_5860	0	0
SCE1572_5283	33.08	18.58
SCE1572_4071	31.13	140.17
SCE1572_3936	0	0
SCE1572_7455	36.93	240.94
SCE1572_6725	34.73	57.87
SCE1572_1844	31.93	19.36
SCE1572_2505	13.98	9.06
SCE1572_6563	30.55	32.08
SCE1572_1296	0	0
SCE1572_652	560.76	2315.25
SCE1572_6730	356.06	5485.43
SCE1572_7453	50.59	368.95
SCE1572_1672	15.52	16.19
SCE1572_5689	30.03	14.22
SCE1572_8439	35.93	29.53
SCE1572_1736	58.43	28.48
SCE1572_6290	0	0
SCE1572_79	49.78	28.59
SCE1572_3291	14.06	16.62
SCE1572_4084	36.95	10.73
SCE1572_5366	33.95	542.12
SCE1572_5844	30.96	24.84
SCE1572_6293	54.09	60.82
SCE1572_4200	37.03	107.19
SCE1572_4776	69.19	363.72

SCE1572_2158	0	0
SCE1572_6797	49	135.73
SCE1572_1960	59.75	12.16
SCE1572_10613	13.27	12.29
SCE1572_8382	15.41	2.97
SCE1572_5135	49.93	389.99
SCE1572_2011	27.01	1.14
SCE1572_10528	8.81	0
SCE1572_1934	82.81	151.58
SCE1572_11207	0	0
SCE1572_1743	53.46	30.73
SCE1572_5718	21.06	26.19
SCE1572_9134	27.63	13.48
SCE1572_1625	56.22	27.37
SCE1572_1381	20.06	15.87
SCE1572_2085	24.6	22.32
SCE1572_4014	0	0
SCE1572_4560	12.13	4.87
SCE1572_4649	43.98	17.38
SCE1572_3779	15.98	13.92
SCE1572_7107	51.29	32.3
SCE1572_11562	0	0
SCE1572_3755	27.74	7.57
SCE1572_5138	0	0
SCE1572_11283	30.12	41.78
SCE1572_10088	0	0
SCE1572_2807	8.91	0
SCE1572_10266	60.85	167.57
SCE1572_9441	50.87	429.18
SCE1572_8799	7	0
SCE1572_4843	28.12	33.49
SCE1572_3612	46.78	19.67
SCE1572_1710	25	38.82
SCE1572_3363	40.16	74.96
SCE1572_7352	40.35	14.71
SCE1572_1471	0	0
SCE1572_309	37.06	11.58
SCE1572_9103	44.81	56.3
SCE1572_5305	0	0
SCE1572_1350	38.5	42.76
SCE1572_8020	43.16	23.67
SCE1572_5984	44.75	41.83
SCE1572_3079	0	0
SCE1572_9249	38.37	12.43
SCE1572_6950	11.66	0
SCE1572_7522	34.46	12.44
SCE1572_701	34.99	352.09
SCE1572_6820	22.92	5.66

SCE1572_8389	57.97	21.47
SCE1572_11293	119.89	409.55
SCE1572_5739	117.59	97.91
SCE1572_2790	16.32	7.6
SCE1572_5209	8.99	6.19
SCE1572_7219	132.43	2315.68
SCE1572_8163	30.63	17.82
SCE1572_1300	49.7	13
SCE1572_491	366.86	346.86
SCE1572_5455	26.16	8.36
SCE1572_7184	0	0
SCE1572_5233	18.76	127.91
SCE1572_8913	62.23	24.51
SCE1572_4278	65.13	39.06
SCE1572_8414	44.35	29.19
SCE1572_3238	36.47	22.77
SCE1572_8464	170.83	426.6
SCE1572_8496	39.46	14.72
SCE1572_10606	31.12	24.7
SCE1572_5404	11.49	11.06
SCE1572_744	98.21	103.67
SCE1572_10856	118.46	452.16
SCE1572_964	97.19	534.91
SCE1572_10846	15.28	12.51
SCE1572_4640	154.05	91.52
SCE1572_947	64.32	8.58
SCE1572_6924	2.86	0
SCE1572_4090	25.64	13.42
SCE1572_8405	30.76	134.86
SCE1572_7549	71.87	21.24
SCE1572_6454	81.68	88.47
SCE1572_9207	45.64	37.83
SCE1572_8087	61.51	12.8
SCE1572_3098	0	0
SCE1572_9972	72.16	340.11
SCE1572_210	30.23	10.86
SCE1572_5524	31.54	20.82
SCE1572_5357	51.99	29.1
SCE1572_5795	48.59	37.43
SCE1572_10257	31.74	27.14
SCE1572_3514	60.28	395.7
SCE1572_5887	19.56	14.39
SCE1572_8946	118.93	489.73
SCE1572_11502	18.78	1.67
SCE1572_7205	74.16	94.09
SCE1572_9694	716.09	4340.34
SCE1572_1141	15.26	8.29
SCE1572_2623	3.45	0

SCE1572_7419	13.78	3.9
SCE1572_6656	49.02	136.27
SCE1572_2270	109.58	614.95
SCE1572_3151	36.9	422.85
SCE1572_7614	20.3	15.76
SCE1572_4679	50.43	15.17
SCE1572_5206	54.75	136.12
SCE1572_6611	28.51	9.36
SCE1572_6287	44.16	72.63
SCE1572_11290	331.89	518.12
SCE1572_196	17.58	0
SCE1572_6478	34.49	13.84
SCE1572_3070	23.8	21.27
SCE1572_9515	38.89	113.44
SCE1572_10283	30.21	136.18
SCE1572_11540	25.14	11.34
SCE1572_10033	64.45	473.58
SCE1572_5802	66.89	128.8
SCE1572_9492	39.43	21.52
SCE1572_7064	42.92	18.56
SCE1572_8345	0	0
SCE1572_582	12.96	6.93
SCE1572_1346	19.7	35.92
SCE1572_4569	37.9	31.73
SCE1572_655	1052.09	1472.02
SCE1572_3021	11.74	2.35
SCE1572_5587	21.03	9.49
SCE1572_8858	50.03	42.11
SCE1572_4983	246.42	511.21
SCE1572_11173	25.58	30.03
SCE1572_6366	14.8	206.45
SCE1572_11004	24.88	41.97
SCE1572_6897	0	0
SCE1572_2818	45.99	31.63
SCE1572_9818	20.54	21.19
SCE1572_5567	45.05	148.07
SCE1572_11298	62.59	201.29
SCE1572_3929	41.83	71.1
SCE1572_831	0	0
SCE1572_772	13.46	8.64
SCE1572_5837	29.07	60.68
SCE1572_3974	13.22	53.04
SCE1572_8074	17.28	5.67
SCE1572_7618	157.08	300.81
SCE1572_6387	26.25	73.55
SCE1572_4377	44.91	29.4
SCE1572_7664	39.79	13.34
SCE1572_6885	46.78	98.99

SCE1572_5511	87.54	73.85
SCE1572_1891	53.07	7.3
SCE1572_4955	23.65	8.4
SCE1572_3104	21.46	9.89
SCE1572_10163	51.71	22.63
SCE1572_9407	23.38	32.23
SCE1572_2576	25.37	7.45
SCE1572_1057	23.31	9.8
SCE1572_3041	13.78	1.56
SCE1572_3894	16.79	7.35
SCE1572_9509	16.85	10.81
SCE1572_1729	21.81	94.98
SCE1572_10171	10.15	3.7
SCE1572_11043	31.15	25.83
SCE1572_1117	11.62	19.46
SCE1572_2373	41.14	14.14
SCE1572_188	23.15	288.44
SCE1572_6957	0	0
SCE1572_9168	29.53	17.77
SCE1572_7074	48.88	67.22
SCE1572_2983	14.41	38.85
SCE1572_6866	55.16	30.35
SCE1572_7234	61.93	60.82
SCE1572_6806	23.35	17.34
SCE1572_8920	62.19	93.8
SCE1572_8919	29.73	787.09
SCE1572_117	37.35	286.91
SCE1572_7702	48.77	16.36
SCE1572_1503	38.22	102.63
SCE1572_5774	35.56	3
SCE1572_1512	10.01	13.77
SCE1572_5065	27.14	90.1
SCE1572_3749	37.59	286.51
SCE1572_7921	2.61	0
SCE1572_11396	215.8	660.44
SCE1572_2257	53.71	43.3
SCE1572_4926	45.05	13.99
SCE1572_9141	14.23	4.72
SCE1572_6890	42.88	7.37
SCE1572_7139	0	0
SCE1572_10736	28.92	53.69
SCE1572_4587	92.51	217.45
SCE1572_473	33.33	259.54
SCE1572_9660	351.78	834.95
SCE1572_5186	37.65	1252.14
SCE1572_4194	499.81	939.36
SCE1572_8894	38.94	29.12
SCE1572_8906	70.19	67.58

SCE1572_3388	11.1	0.92
SCE1572_6857	24.32	6.83
SCE1572_11148	22.02	37.86
SCE1572_10658	1164.39	996.69
SCE1572_9615	0	0
SCE1572_10048	35.5	155.48
SCE1572_4370	27.07	14.89
SCE1572_1483	80.18	30.79
SCE1572_8118	33.13	104.59
SCE1572_11491	25.97	18.38
SCE1572_5830	41.78	22.71
SCE1572_996	55.42	27.63
SCE1572_5897	30.07	15.08
SCE1572_702	63.68	32.84
SCE1572_2960	40.03	31.44
SCE1572_7942	29.21	16.91
SCE1572_4707	17.84	37.28
SCE1572_1967	15.86	19.08
SCE1572_4676	45.48	29.47
SCE1572_815	30.32	14.79
SCE1572_3168	64.03	34.02
SCE1572_4990	98.91	670.76
SCE1572_6378	34.35	9.98
SCE1572_11371	207.4	225.23
SCE1572_1594	38.43	60.99
SCE1572_9191	23.53	18.88
SCE1572_9059	18.69	15
SCE1572_10443	22.97	6.63
SCE1572_3127	16.28	41.64
SCE1572_7996	56.42	25.54
SCE1572_6471	16.18	10.25
SCE1572_3243	43.62	22.5
SCE1572_528	46.45	164.53
SCE1572_2255	21.23	5.47
SCE1572_5711	230.03	532.5
SCE1572_5870	33.14	57.83
SCE1572_3001	19.12	8.22
SCE1572_181	28.9	16.36
SCE1572_4973	239.89	2367.1
SCE1572_9432	27.76	20.97
SCE1572_2263	128.9	807.41
SCE1572_5494	85.92	20.27
SCE1572_738	18.19	15.99
SCE1572_10712	22.68	30.66
SCE1572_10975	491.55	6726.09
SCE1572_1753	33.94	108.93
SCE1572_4250	30.72	77.07
SCE1572_5439	48.89	15.9

SCE1572_4551	32.83	5.75
SCE1572_8107	35.08	13.57
SCE1572_7499	55.33	26.64
SCE1572_2026	63.39	50.15
SCE1572_3377	48.63	25.64
SCE1572_6978	16.78	1.5
SCE1572_1855	46.69	16.53
SCE1572_3220	140.88	258.35
SCE1572_3600	66.75	52.79
SCE1572_498	49.81	25.4
SCE1572_7276	39.07	5.6
SCE1572_6901	129.47	1312.08
SCE1572_10	28.75	83.89
SCE1572_10204	2.81	0
SCE1572_3869	28.43	30.41
SCE1572_8184	2.17	0
SCE1572_434	254.32	235.43
SCE1572_5268	14.86	50.09
SCE1572_5027	26.42	212.6
SCE1572_35	15.03	65.75
SCE1572_4988	63.73	126.17
SCE1572_10524	49.21	20.81
SCE1572_6861	12.49	6.44
SCE1572_4630	71.08	20.48
SCE1572_6095	24.06	16.39
SCE1572_8115	20.18	2.49
SCE1572_8121	62.74	24.5
SCE1572_7359	7.38	4.74
SCE1572_5463	29.32	11.14
SCE1572_9340	631.12	14657.71
SCE1572_10913	71.45	230.7
SCE1572_8039	53.62	19.21
SCE1572_3651	44.81	48.91
SCE1572_7556	0	0
SCE1572_3259	42.96	142.05
SCE1572_478	18.78	20.95
SCE1572_1403	2.97	0
SCE1572_9836	92.59	30.64
SCE1572_1179	16.97	7.99
SCE1572_5228	52.64	150.1
SCE1572_9089	43.53	17.34
SCE1572_6589	118.02	30.59
SCE1572_7533	0	0
SCE1572_3383	50.89	505.08
SCE1572_4498	25.44	45.92
SCE1572_10670	11.49	0
SCE1572_6100	32.76	6.87
SCE1572_5861	38.56	0

SCE1572_7527	49.48	16.2
SCE1572_1050	26.44	14.85
SCE1572_9842	0	0
SCE1572_8036	16.85	4.51
SCE1572_10675	74.37	61.44
SCE1572_10599	25.67	3.59
SCE1572_4113	35.42	31.83
SCE1572_7859	74.4	149.6
SCE1572_8602	43.74	26.58
SCE1572_8967	23.02	74.51
SCE1572_1357	53.4	149.22
SCE1572_3544	53.75	18.37
SCE1572_4402	12.89	57.62
SCE1572_1155	41.48	30.54
SCE1572_2031	56.98	13.95
SCE1572_5019	14.71	36.61
SCE1572_5449	39.12	25.54
SCE1572_8004	9.19	3.69
SCE1572_1692	7.03	0
SCE1572_5122	14.13	131.46
SCE1572_8358	54.22	23.8
SCE1572_7223	184.53	715.64
SCE1572_3218	8.05	0
SCE1572_5223	117.44	199.84
SCE1572_8721	53.1	47.09
SCE1572_589	6.64	2.66
SCE1572_9849	37.73	38.61
SCE1572_936	18.81	1.33
SCE1572_8819	21.94	12
SCE1572_3626	33.74	10.51
SCE1572_3821	51.32	42.06
SCE1572_6371	9.19	5.53
SCE1572_7542	47.78	247.7
SCE1572_4382	16.84	3.55
SCE1572_97	20.99	11.23
SCE1572_6282	7.34	0
SCE1572_3198	12.78	139.41
SCE1572_8156	37.2	15.35
SCE1572_10159	6.74	8.11
SCE1572_2432	48.44	121.25
SCE1572_7672	6.63	0
SCE1572_9825	7.61	19.48
SCE1572_2479	12.63	43.44
SCE1572_4578	7.4	8.9
SCE1572_7152	20.17	10.3
SCE1572_10378	37.51	16.07
SCE1572_10430	54.69	43.15
SCE1572_9909	52.61	29.93

SCE1572_11367	18.38	7.74
SCE1572_1191	25.1	10.07
SCE1572_7552	10.86	0
SCE1572_6414	14	2.81
SCE1572_6544	68.97	190.32
SCE1572_9016	0	0
SCE1572_8260	83.58	340.59
SCE1572_4507	26.84	12.47
SCE1572_6240	90.69	26.46
SCE1572_2080	34.26	27.76
SCE1572_9806	50.16	74.76
SCE1572_4154	2.17	0
SCE1572_5144	0	0
SCE1572_577	0	0
SCE1572_288	20.5	83.11
SCE1572_2057	21.17	17.67
SCE1572_1605	66.48	26.01
SCE1572_5973	37.33	8.87
SCE1572_10826	0	0
SCE1572_7926	17.1	49.39
SCE1572_7883	47.38	9.77
SCE1572_10437	27.52	24.99
SCE1572_8956	31.22	0
SCE1572_7875	46.82	48.3
SCE1572_10012	34.39	110.37
SCE1572_2932	28.3	15.35
SCE1572_4964	28.33	68.19
SCE1572_6759	40.76	15.49
SCE1572_10590	54.57	23.8
SCE1572_10191	66.33	63.35
SCE1572_5542	32.62	14.81
SCE1572_7055	65.88	16.59
SCE1572_6392	55.2	100.9
SCE1572_9870	22.97	25.8
SCE1572_10136	24.83	34.6
SCE1572_5327	82.87	1921.6
SCE1572_482	34.72	21.17
SCE1572_9720	20.33	157.99
SCE1572_9262	31.04	34.48
SCE1572_8741	24.72	7.93
SCE1572_10726	12.98	3.35
SCE1572_9895	28.05	39.31
SCE1572_6782	4.48	46.74
SCE1572_9674	33.48	76.94
SCE1572_2691	109.32	201.26
SCE1572_10691	55.59	0
SCE1572_8501	2.25	0.39
SCE1572_7585	343.99	753.39

SCE1572_1457	18.82	7.55
SCE1572_6708	24.86	95.62
SCE1572_7853	68.45	219.66
SCE1572_10581	47.38	22.81
SCE1572_1097	36.52	31.96
SCE1572_3805	40.75	196.16
SCE1572_3044	15.59	0.94
SCE1572_4032	1331.03	311.14
SCE1572_5716	183.33	505.87
SCE1572_6186	3.74	0
SCE1572_6402	21.03	7.56
SCE1572_4628	1.96	0
SCE1572_5155	67.84	75.84
SCE1572_887	156.12	363.72
SCE1572_5533	40.18	16.49
SCE1572_10770	35.28	56
SCE1572_8765	51.51	22.41
SCE1572_5789	56.22	202.99
SCE1572_8547	27.13	6.72
SCE1572_2178	30.69	24.13
SCE1572_10545	24.57	15.21
SCE1572_4093	17.62	3.35
SCE1572_2797	56.76	21.81
SCE1572_2534	0	0
SCE1572_1918	39.83	30.93
SCE1572_7257	20.15	25.41
SCE1572_5513	41.51	56.48
SCE1572_2863	36.12	13.47
SCE1572_7494	35.67	56.59
SCE1572_10196	53.37	5.84
SCE1572_212	42.63	13.01
SCE1572_7129	41.45	46.38
SCE1572_2452	35.1	19.43
SCE1572_3740	44.14	17.71
SCE1572_2074	14.38	11.01
SCE1572_4627	10.19	1.97
SCE1572_9080	13.48	0
SCE1572_9555	49.51	469.19
SCE1572_7462	84.17	267.28
SCE1572_2335	37.72	385
SCE1572_502	19.06	4.17
SCE1572_2021	9.78	0
SCE1572_942	73.61	21.12
SCE1572_7024	46.97	27.58
SCE1572_10943	65.77	19.32
SCE1572_8787	29.09	122.3
SCE1572_8312	89.32	211.37
SCE1572_3964	41.59	25.74

SCE1572_5822	0	0
SCE1572_1217	7.71	0
SCE1572_5474	12.45	10.89
SCE1572_3368	106.69	370.16
SCE1572_4543	38.6	23.56
SCE1572_3089	44.51	354.88
SCE1572_8634	0	0
SCE1572_11341	40.8	17.59
SCE1572_249	0	0
SCE1572_4269	31.28	27.19
SCE1572_255	30.68	13.03
SCE1572_2894	59.53	25.7
SCE1572_5703	40.01	281.86
SCE1572_7286	49.22	214.72
SCE1572_6689	6.69	0
SCE1572_9296	43.59	108.56
SCE1572_4849	68	92.12
SCE1572_9215	16.22	13.66
SCE1572_123	15.42	0
SCE1572_6315	30.11	16.59
SCE1572_5292	26.16	376.23
SCE1572_8649	47.09	24.93
SCE1572_9463	11.66	0
SCE1572_2684	57.93	575.27
SCE1572_4601	12.01	12.04
SCE1572_9935	19.04	6.55
SCE1572_6630	19.05	645.49
SCE1572_10530	21.66	56.96
SCE1572_2636	56.29	20.42
SCE1572_8044	26.29	14.77
SCE1572_1378	37.48	17.43
SCE1572_8685	60.01	26.07
SCE1572_486	39.08	11.29
SCE1572_365	32.49	15.4
SCE1572_2302	67.31	198.19
SCE1572_121	11	11.14
SCE1572_4296	36.83	31.84
SCE1572_6971	72.45	14.38
SCE1572_3694	34.74	20.49
SCE1572_4725	54.07	55.38
SCE1572_6397	28.79	88.69
SCE1572_5241	23.48	29.06
SCE1572_10462	54.5	519.64
SCE1572_11380	11.16	96.27
SCE1572_9357	65.18	592.65
SCE1572_9527	46.96	96.88
SCE1572_8625	48.73	56.79
SCE1572_5551	41.12	12.05

SCE1572_846	61.1	91.23
SCE1572_9993	75.01	27.63
SCE1572_90	61.46	133.03
SCE1572_2350	957.45	1778.64
SCE1572_3225	17.73	10.83
SCE1572_3534	0	0
SCE1572_2545	62.24	11.52
SCE1572_410	17.45	595.95
SCE1572_9283	61.25	251.34
SCE1572_8748	6.41	1.84
SCE1572_2722	42.32	107.47
SCE1572_5560	30	18.5
SCE1572_133	80.23	143.15
SCE1572_10953	14.69	26.87
SCE1572_3674	64.31	546.64
SCE1572_7200	33.76	36.49
SCE1572_328	9.78	1.96
SCE1572_10226	45.94	12.9
SCE1572_2561	240.62	575.41
SCE1572_3145	5.69	4.56
SCE1572_9123	66.19	48.16
SCE1572_5681	155.99	80.7
SCE1572_6693	0	0
SCE1572_10637	13.07	6.29
SCE1572_8691	31.17	20.36
SCE1572_8927	9.63	5.79
SCE1572_9958	32.83	9.3
SCE1572_10833	0	0
SCE1572_1262	49.25	195.86
SCE1572_9669	29.2	106.19
SCE1572_6487	26.49	2.71
SCE1572_6995	93.56	20.11
SCE1572_69	98.45	41.07
SCE1572_3623	81.57	18.48
SCE1572_5392	74.44	59.28
SCE1572_9704	50.54	76.96
SCE1572_7443	27.32	19.73
SCE1572_6913	50.4	14.51
SCE1572_2264	43	727.12
SCE1572_5908	133.52	331.66
SCE1572_9749	54.46	79.72
SCE1572_8709	17.02	7.45
SCE1572_11324	79.65	1121.67
SCE1572_4344	7.46	0
SCE1572_4460	26.04	11.06
SCE1572_5200	24.34	17.41
SCE1572_9011	26.17	33.34
SCE1572_388	42.71	43.69

SCE1572_5274	29.16	18.71
SCE1572_1817	108.59	98.63
SCE1572_5745	20.49	167.67
SCE1572_4712	67.74	55.29
SCE1572_6918	49.08	65.63
SCE1572_3283	62.73	21.67
SCE1572_6264	47.59	9.45
SCE1572_6525	37.64	30.2
SCE1572_8486	46.12	16.61
SCE1572_11462	107.87	186.07
SCE1572_7570	12.26	18.63
SCE1572_3874	49.67	57.41
SCE1572_8016	255.71	49.02
SCE1572_3149	25.14	22.09
SCE1572_4682	53.67	12.92
SCE1572_242	31.43	45.24
SCE1572_8810	73.26	26.94
SCE1572_7272	65.54	37.67
SCE1572_8240	14.32	10.7
SCE1572_8561	32.17	30.49
SCE1572_1914	52.11	30.48
SCE1572_2516	30.8	19.01
SCE1572_5258	6.89	0
SCE1572_8204	2.61	0
SCE1572_4438	16.86	8.44
SCE1572_5809	20.34	25.66
SCE1572_9378	0	0
SCE1572_4764	18.85	12.55
SCE1572_3913	53.88	138.17
SCE1572_5323	35.56	51.81
SCE1572_8400	25.11	50.36
SCE1572_2095	34.57	35.09
SCE1572_5652	27.76	317.2
SCE1572_2594	40.34	43.38
SCE1572_8568	9.96	4.37
SCE1572_1872	18.04	92.18
SCE1572_4160	25.81	27.18
SCE1572_62	29.99	0
SCE1572_7837	70.64	62.2
SCE1572_2926	14.13	4.53
SCE1572_6074	85.22	317.18
SCE1572_6508	1.97	0
SCE1572_1283	0	0
SCE1572_4167	16.91	10.5
SCE1572_4476	18.29	17.38
SCE1572_2711	60.26	177.38
SCE1572_6835	26.3	260.66
SCE1572_5628	36.57	10.73

SCE1572_6987	21.13	5.3
SCE1572_10093	436.13	1265.97
SCE1572_1950	16.07	16.73
SCE1572_9931	50.54	207.05
SCE1572_70	84.05	45.53
SCE1572_8973	11.18	17.94
SCE1572_9579	70.87	27.18
SCE1572_892	0	0
SCE1572_9037	42.13	77.59
SCE1572_10413	3.03	0
SCE1572_612	36.57	232.08
SCE1572_10019	47.47	81.49
SCE1572_1281	40.4	10.1
SCE1572_5173	13.07	0
SCE1572_10262	31.51	20.52
SCE1572_2511	70.89	360.18
SCE1572_5595	0	0
SCE1572_10001	14.52	5.14
SCE1572_3831	22.9	28.66
SCE1572_9877	0	0
SCE1572_8805	24.07	7.72
SCE1572_6141	36.76	19.9
SCE1572_825	52.28	16.52
SCE1572_880	216.7	841.12
SCE1572_1225	19.46	8.07
SCE1572_9901	1.67	0
SCE1572_8551	56	59.6
SCE1572_3786	203.86	303.99
SCE1572_4697	0	0
SCE1572_7890	662.62	28158.49
SCE1572_8891	109.16	46.04
SCE1572_5591	37.38	10.1
SCE1572_9240	0	0
SCE1572_10866	13.91	13.39
SCE1572_2877	132.88	229.61
SCE1572_4655	0	0
SCE1572_4186	22.89	16.63
SCE1572_8825	35.91	48.02
SCE1572_5526	25.66	23.39
SCE1572_11054	10.73	7.5
SCE1572_2167	69.34	19.67
SCE1572_7503	47.73	64.2
SCE1572_9629	30.06	10.34
SCE1572_8844	0	0
SCE1572_7384	203.37	160.5
SCE1572_3987	122.93	3866.2
SCE1572_11577	15.98	5.98
SCE1572_8317	15.25	92.34

SCE1572_10787	41.35	61.85
SCE1572_3263	311.97	3461.2
SCE1572_1008	55.81	30.6
SCE1572_875	93.17	72.32
SCE1572_5096	74.69	86.76
SCE1572_5943	34.63	11.91
SCE1572_710	118.4	107.74
SCE1572_2802	32.82	26.86
SCE1572_5378	49.12	11.56
SCE1572_2976	22.32	3.88
SCE1572_755	17.84	10.02
SCE1572_2236	49.98	48.2
SCE1572_7813	6.45	0
SCE1572_51	52.88	16.46
SCE1572_10242	35.52	9.04
SCE1572_1388	30.95	51.86
SCE1572_6108	57.2	17.79
SCE1572_3811	30.64	10.17
SCE1572_6359	23.03	9.24
SCE1572_1890	33.45	65.63
SCE1572_3053	28.98	11.35
SCE1572_11192	18.12	15.1
SCE1572_2761	44.27	256.18
SCE1572_9706	126.35	111.5
SCE1572_10247	0	0
SCE1572_3471	7.58	0
SCE1572_976	0	0
SCE1572_761	20.05	16.48
SCE1572_7364	42.98	82.42
SCE1572_10054	54.5	269.66
SCE1572_1221	49.29	8.99
SCE1572_6311	26.09	1091.21
SCE1572_10215	2.61	0
SCE1572_959	32.96	65.23
SCE1572_1330	54.27	16.59
SCE1572_5145	18.79	22.23
SCE1572_9951	15.11	13.02
SCE1572_7085	25.99	14.99
SCE1572_2162	17.58	5.29
SCE1572_9798	21.97	0
SCE1572_11547	22.06	3.53
SCE1572_5635	102.81	74.41
SCE1572_3451	28.91	5.68
SCE1572_9499	399.18	3232.98
SCE1572_353	22.69	9.3
SCE1572_4594	60.92	53.22
SCE1572_568	15.72	111.26
SCE1572_11407	30.09	16.71

SCE1572_3137	27.47	51.89
SCE1572_855	69.64	450.88
SCE1572_11031	35.28	151.48
SCE1572_8422	105.05	844.53
SCE1572_7761	16.62	5
SCE1572_9733	155.33	65.29
SCE1572_7696	147.85	86.7
SCE1572_3235	603.78	487.46
SCE1572_4007	53.14	131.24
SCE1572_10745	32.99	24.58
SCE1572_7709	237.11	3596.25
SCE1572_4292	30.79	16.78
SCE1572_5750	20.24	2.87
SCE1572_4854	15.45	11.02
SCE1572_981	57.04	20.42
SCE1572_1808	12.04	31.62
SCE1572_9022	26.08	43.93
SCE1572_379	25.38	115.67
SCE1572_3993	24.04	66.75
SCE1572_104	22.52	2.41
SCE1572_1780	33.42	65.54
SCE1572_11427	30.61	27.8
SCE1572_7302	116.11	32.33
SCE1572_9641	71.27	51.53
SCE1572_5423	31.96	13.77
SCE1572_3505	85.65	30.81
SCE1572_493	35.25	5.14
SCE1572_5997	15.95	31.68
SCE1572_6565	17.27	10.39
SCE1572_1450	8.03	21.6
SCE1572_7448	29.64	44.12
SCE1572_360	108.91	133.63
SCE1572_7164	30.43	10.1
SCE1572_9278	3.46	0
SCE1572_1755	36.5	157.68
SCE1572_6911	57.55	18.15
SCE1572_2904	41.89	4.8
SCE1572_5507	3.3	0
SCE1572_9457	52.03	17.61
SCE1572_10643	73.04	247.72
SCE1572_9461	51.01	77.3
SCE1572_3393	21.02	3.61
SCE1572_5994	45.53	46.17
SCE1572_9724	30.64	18.81
SCE1572_3303	20.8	30.81
SCE1572_9648	61.82	102.75
SCE1572_8230	20.75	39.37
SCE1572_7175	70.61	212.28

SCE1572_1597	56.34	26.7
SCE1572_1772	64.02	18.92
SCE1572_9230	38.75	10.49
SCE1572_2218	33.69	49.42
SCE1572_3856	59.95	30.53
SCE1572_131	10.99	26.44
SCE1572_2322	11.37	39.53
SCE1572_10255	37.98	19.93
SCE1572_7968	46.07	51.58
SCE1572_8636	8.35	13.39
SCE1572_4582	35.09	18.94
SCE1572_6194	39.29	70.49
SCE1572_9359	36.06	46.73
SCE1572_11153	148.16	1377.82
SCE1572_5701	15.49	69.26
SCE1572_10506	0	0
SCE1572_3061	4.3	0
SCE1572_5502	22.33	8.49
SCE1572_7785	2.81	0
SCE1572_8732	0	0
SCE1572_6043	0	0
SCE1572_3710	2516.82	83355.21
SCE1572_6345	18.1	9.08
SCE1572_5267	41.74	143.08
SCE1572_9886	33.04	101.21
SCE1572_471	17.14	34.91
SCE1572_10559	12.63	11.4
SCE1572_7470	71.37	70.14
SCE1572_5319	72.02	27.37
SCE1572_322	37.74	21.56
SCE1572_2285	13.1	189.22
SCE1572_3656	21.57	29.67
SCE1572_5509	10.17	5.83
SCE1572_6039	11.86	41.04
SCE1572_3446	0	0
SCE1572_9213	96.22	23.06
SCE1572_8647	63.68	18.25
SCE1572_1183	35.03	23.1
SCE1572_549	0	0
SCE1572_5579	34.61	21.47
SCE1572_9783	33.17	45.62
SCE1572_11086	36.29	74.95
SCE1572_4567	68.47	58.86
SCE1572_521	10.98	6.6
SCE1572_9592	31.06	381.39
SCE1572_8281	17.72	23.7
SCE1572_4119	44.59	8840.42
SCE1572_48	30.32	8.42

SCE1572_2892	29.57	15.51
SCE1572_3724	22.83	438.19
SCE1572_1474	45.48	12.16
SCE1572_506	8.66	2.61
SCE1572_7475	107.21	503.72
SCE1572_1911	50.39	30.32
SCE1572_3323	0	0
SCE1572_11347	31.14	48.33
SCE1572_9730	49.87	90.27
SCE1572_9786	71.09	19.14
SCE1572_2948	36.22	20.27
SCE1572_4450	17.33	20.85
SCE1572_5165	83.33	577.07
SCE1572_4889	14.81	13.17
SCE1572_901	36.23	18.78
SCE1572_9353	48.06	150.63
SCE1572_7189	54.93	32.38
SCE1572_8641	35.2	15.73
SCE1572_1814	15.89	4.78
SCE1572_11280	30.82	218.06
SCE1572_6133	61.11	441.27
SCE1572_10760	18.74	393.62
SCE1572_7355	89.55	76.78
SCE1572_9941	20.26	23.59
SCE1572_9029	62.56	21.71
SCE1572_8331	57.54	251.71
SCE1572_3312	29.98	36.6
SCE1572_7168	87.65	224.82
SCE1572_10801	0	0
SCE1572_5901	43.67	26.27
SCE1572_11265	91.53	274.89
SCE1572_3314	20.08	18.21
SCE1572_6845	93.24	417.49
SCE1572_8800	47.54	3.09
SCE1572_5637	47.36	22.97
SCE1572_6046	97.17	201.9
SCE1572_3087	24.64	18.25
SCE1572_1642	25.27	22.41
SCE1572_2744	59.43	502.13
SCE1572_1090	21.77	8.27
SCE1572_3961	116.3	167.65
SCE1572_2570	40.98	0
SCE1572_5773	0	0
SCE1572_9200	0	0
SCE1572_7656	4.08	4.21
SCE1572_2844	8.29	0
SCE1572_6331	70.54	544.85
SCE1572_10931	19.83	14.04

SCE1572_3942	696.98	870.64
SCE1572_6051	21.89	56.51
SCE1572_10185	76.27	60.45
SCE1572_6058	10.74	38.31
SCE1572_9595	23.94	689.03
SCE1572_8739	35.41	11
SCE1572_3923	14.06	5.89
SCE1572_595	42.22	12.7
SCE1572_899	34.4	19.87
SCE1572_8987	20.22	40.55
SCE1572_4892	62.26	97.13
SCE1572_11327	10.47	19.98
SCE1572_1435	30.75	35.06
SCE1572_3601	13.27	12.29
SCE1572_5929	78.36	25.11
SCE1572_9060	37.69	19.73
SCE1572_11363	28.99	17.52
SCE1572_141	32.6	5.12
SCE1572_8533	51.1	26.7
SCE1572_126	13.96	7.25
SCE1572_6276	44.5	201.52
SCE1572_8026	2.49	0
SCE1572_7299	84.26	50.4
SCE1572_4599	11.37	0
SCE1572_991	0	0
SCE1572_1139	34.41	23.29
SCE1572_10070	476.72	1198.25
SCE1572_806	0	0
SCE1572_4994	48.41	25.28
SCE1572_8445	31.29	26.07
SCE1572_10567	38.4	25.33
SCE1572_8520	5.82	0.5
SCE1572_10369	51.06	21.17
SCE1572_10311	5.62	1.35
SCE1572_2005	82.03	576.47
SCE1572_4882	46.34	32.29
SCE1572_1555	0	0
SCE1572_317	28.49	23.22
SCE1572_445	68.82	28.47
SCE1572_979	0	0
SCE1572_7172	82.97	50.83
SCE1572_1996	34.39	8.28
SCE1572_6128	200.69	2087.29
SCE1572_7133	48.37	21.11
SCE1572_4811	25.27	60.16
SCE1572_9762	12.79	21.62
SCE1572_9982	32.07	19.49
SCE1572_6171	23.01	16.29

SCE1572_8673	32.63	7.7
SCE1572_3457	0	0
SCE1572_5921	0	0
SCE1572_9030	71.18	34.82
SCE1572_7051	20	2.63
SCE1572_2298	60.65	105.7
SCE1572_264	37.9	44.5
SCE1572_4590	9.19	0
SCE1572_10212	48	23.75
SCE1572_6004	38.54	122.05
SCE1572_753	35.75	40.05
SCE1572_7410	0	0
SCE1572_5249	28.3	55.47
SCE1572_9385	17.06	36.49
SCE1572_9115	257.24	656.04
SCE1572_7995	65.75	13.03
SCE1572_6124	18.27	48.36
SCE1572_11409	20.7	13.9
SCE1572_2768	6.36	2.55
SCE1572_7263	18.87	12.53
SCE1572_50	19.47	4.46
SCE1572_11533	24.22	10.14
SCE1572_1765	15.38	1.72
SCE1572_11082	44.41	76.34
SCE1572_1741	72.8	79.84
SCE1572_11488	67.25	21.02
SCE1572_4447	39.03	24.06
SCE1572_9371	36.43	42.65
SCE1572_5386	36.82	24.77
SCE1572_6626	31.81	43.38
SCE1572_11525	24.71	14.87
SCE1572_2136	39.25	5.56
SCE1572_6787	52.25	51.78
SCE1572_4190	20.36	34.02
SCE1572_6664	34.44	26.82
SCE1572_6784	96.54	61.07
SCE1572_6752	10.63	9.43
SCE1572_2705	10.48	11.65
SCE1572_6641	34.99	24.33
SCE1572_10376	58.43	12.12
SCE1572_4902	16.41	10.4
SCE1572_5791	32.88	38.58
SCE1572_3153	0	0
SCE1572_2163	15.62	8.85
SCE1572_10382	30.32	28.89
SCE1572_370	31.76	30.29
SCE1572_2667	61.75	27.35
SCE1572_2414	94.13	69.94

SCE1572_10814	11.66	14.04
SCE1572_3057	46.88	12
SCE1572_1897	11.09	8.9
SCE1572_9753	58.1	13.64
SCE1572_5089	13.71	21.35
SCE1572_1294	42.35	24.9
SCE1572_6144	0	0
SCE1572_10140	26.31	28.98
SCE1572_7114	52.15	32.54
SCE1572_1279	78.84	29.19
SCE1572_8887	22.61	0
SCE1572_1823	30.66	77.9
SCE1572_2123	32.94	47.76
SCE1572_3187	11.23	0
SCE1572_11415	20.99	11.23
SCE1572_10345	25.33	17.78
SCE1572_2913	25.47	7.53
SCE1572_8498	0	0
SCE1572_4505	2.41	0
SCE1572_6241	23.24	23.77
SCE1572_4745	20.12	49
SCE1572_5622	30.37	52.73
SCE1572_4859	32.7	9.52
SCE1572_1619	224.93	3085.56
SCE1572_9074	95.68	23.21
SCE1572_4182	9.15	5.87
SCE1572_6428	24.41	4.45
SCE1572_1200	38.13	11.74
SCE1572_2403	26.31	24.99
SCE1572_169	50.96	119.63
SCE1572_2147	77.84	27.96
SCE1572_2858	77.83	195.57
SCE1572_4319	43.09	6.6
SCE1572_6771	10.22	16.4
SCE1572_1600	31.06	32.04
SCE1572_4320	49.28	30.41
SCE1572_6639	38.36	13.19
SCE1572_4314	36.51	11.68
SCE1572_10287	272.38	459.82
SCE1572_8750	0	0
SCE1572_9152	30.37	39.14
SCE1572_10723	30.86	35.43
SCE1572_10154	82.83	564.28
SCE1572_4874	679.2	2181.1
SCE1572_7924	21.82	20.61
SCE1572_9638	28.94	122.75
SCE1572_8698	22.04	19.47
SCE1572_2124	58.37	18.08

SCE1572_10576	80.88	31.65
SCE1572_7635	603.14	6212.34
SCE1572_3774	28.87	10.53
SCE1572_3716	154.35	54.79
SCE1572_6299	22.22	22.02
SCE1572_7686	10.11	0
SCE1572_1274	14.21	3.8
SCE1572_6252	64.94	92.87
SCE1572_6609	43.54	37.43
SCE1572_8885	1.74	0
SCE1572_9557	32.06	273.36
SCE1572_1612	14.07	24.04
SCE1572_4571	27.41	21.05
SCE1572_9830	0	0
SCE1572_3278	17.91	61.24
SCE1572_7391	51.86	21.67
SCE1572_1209	50.02	9.41
SCE1572_4821	32.96	5.05
SCE1572_8572	0	0
SCE1572_10330	0	0
SCE1572_5988	0	0
SCE1572_1259	17.04	4.1
SCE1572_10106	195.86	924.38
SCE1572_6438	2.45	0
SCE1572_10783	45.7	27.94
SCE1572_5617	49.39	20.73
SCE1572_11470	25.08	20.58
SCE1572_5981	41.83	69.21
SCE1572_863	40.41	123.18
SCE1572_531	27.24	200.01
SCE1572_5798	58.72	21.09
SCE1572_1124	37.43	12.87
SCE1572_4062	58.87	434.43
SCE1572_2718	46.15	16.66
SCE1572_5647	24.63	12
SCE1572_6612	21.32	11.4
SCE1572_1527	32.23	91.95
SCE1572_10927	47.32	168.75
SCE1572_9542	11.12	7.3
SCE1572_10666	53.92	168.6
SCE1572_6060	166.06	178.12
SCE1572_6409	9.13	0
SCE1572_1104	49.1	124.41
SCE1572_11551	5.14	0
SCE1572_7721	41.62	243.28
SCE1572_7035	16.72	5.15
SCE1572_1782	16.54	102.84
SCE1572_8268	3.7	0

SCE1572_2600	10.18	12.25
SCE1572_555	44.84	34.49
SCE1572_3953	35.05	47.54
SCE1572_6219	23.02	11.82
SCE1572_3345	260.59	1772.86
SCE1572_3497	291.39	1485.35
SCE1572_3958	19.34	27.33
SCE1572_10381	30.14	19.05
SCE1572_3882	22.31	3.98
SCE1572_8984	11.34	0.68
SCE1572_10397	35.15	65.66
SCE1572_281	13.31	10.89
SCE1572_5312	64.66	35.51
SCE1572_7361	4.33	9.12
SCE1572_3675	0	0
SCE1572_9396	53.78	129.15
SCE1572_7738	47.35	48.06
SCE1572_562	85.85	495.42
SCE1572_6150	19.49	0
SCE1572_7984	42.76	122.63
SCE1572_8237	47.92	293.62
SCE1572_542	38.16	3.67
SCE1572_4936	20.7	36.64
SCE1572_5663	26.49	6.26
SCE1572_7736	22.83	60.59
SCE1572_4300	32.07	17.72
SCE1572_2611	19.24	11.23
SCE1572_6117	19.01	16.34
SCE1572_2682	37.27	25.46
SCE1572_3574	27.14	14.2
SCE1572_6739	2.76	0
SCE1572_391	0	0
SCE1572_8796	13.63	0.8
SCE1572_7199	33.63	152.28
SCE1572_9737	23.18	33.17
SCE1572_3351	18.93	21.35
SCE1572_3110	31.22	11.3
SCE1572_9145	18.71	46.19
SCE1572_3117	52.84	171.26
SCE1572_4361	60.65	15.64
SCE1572_9450	42.83	24.88
SCE1572_4265	27.79	7.04
SCE1572_5693	13.78	6.32
SCE1572_9495	34.01	60.97
SCE1572_9180	32.49	9.31
SCE1572_10743	54.71	60.19
SCE1572_2245	85.62	69.88
SCE1572_3425	20.11	11.77

SCE1572_6412	39.93	14.66
SCE1572_2924	61.81	28.36
SCE1572_4915	170.83	1989.08
SCE1572_9529	30.18	45.27
SCE1572_9691	24.59	140.19
SCE1572_1324	15.87	8.49
SCE1572_4025	52.13	81.01
SCE1572_2232	8.37	15.1
SCE1572_8014	23.48	14.3
SCE1572_9414	126.05	1997.66
SCE1572_4791	42.52	32.47
SCE1572_1212	25.86	21.47
SCE1572_1130	0	0
SCE1572_9997	92.28	192.17
SCE1572_11055	41.06	137.61
SCE1572_2436	13.66	70.48
SCE1572_10706	26.9	27.82
SCE1572_9771	26.47	20.27
SCE1572_3241	45.57	14.62
SCE1572_3565	0	0
SCE1572_6551	8.12	1.09
SCE1572_7797	49.67	41.53
SCE1572_2047	0	0
SCE1572_2638	64.29	35.27
SCE1572_4737	32.82	170.96
SCE1572_7326	26.89	51.78
SCE1572_11410	18.3	8.39
SCE1572_2962	3.7	0
SCE1572_7866	170.13	841.27
SCE1572_10452	27.35	92.65
SCE1572_3192	62.74	69.69
SCE1572_4075	43.58	124.55
SCE1572_4837	103.01	253.5
SCE1572_10618	65.55	63.83
SCE1572_10698	36.3	155.91
SCE1572_7161	31.12	17.29
SCE1572_4645	68.24	119.97
SCE1572_20	35.98	307.71
SCE1572_3596	67.25	21.02
SCE1572_2803	22.46	69.27
SCE1572_1042	0	0
SCE1572_4039	44.76	6.95
SCE1572_257	54.64	19.73
SCE1572_6813	111.55	45.72
SCE1572_9420	38.12	224.25
SCE1572_4325	21.54	10.16
SCE1572_4781	184.12	240.13
SCE1572_9250	66.48	19.54

SCE1572_7950	65.08	99.15
SCE1572_6192	29.77	27.87
SCE1572_10920	17	40.91
SCE1572_1731	74.94	29.89
SCE1572_2107	69.07	140.11
SCE1572_2605	11.63	4.66
SCE1572_9055	35.57	11.21
SCE1572_9209	30.44	100.3
SCE1572_8759	70.27	232.29
SCE1572_9245	12.2	0
SCE1572_11065	2.81	13.52
SCE1572_5198	0	0
SCE1572_6931	12.63	0
SCE1572_4783	178.16	85.2
SCE1572_11061	534.74	1520.14
SCE1572_9485	36.98	66.75
SCE1572_8830	52.43	178.71
SCE1572_8374	38.65	83.48
SCE1572_68	58.63	126.3
SCE1572_11142	0	16.78
SCE1572_6652	26.47	10.8
SCE1572_7077	0	0
SCE1572_2640	214.79	129.52
SCE1572_2725	39.31	42.93
SCE1572_3014	31.29	8.97
SCE1572_450	29.28	19.31
SCE1572_1795	46.45	180.13
SCE1572_4101	110.55	1968.3
SCE1572_8747	20.22	0
SCE1572_5301	19.44	24.95
SCE1572_1337	30.26	86.39
SCE1572_11123	43.76	21.76
SCE1572_2601	0	0
SCE1572_3983	13.97	14.95
SCE1572_3966	10.57	34.33
SCE1572_4421	0	0
SCE1572_5048	26.84	5.24
SCE1572_5111	343.34	1015.94
SCE1572_7320	14.03	4.12
SCE1572_7500	12.15	16.71
SCE1572_6916	20.77	21.25
SCE1572_9912	56.68	17.05
SCE1572_10536	93.04	453.39
SCE1572_4331	10.04	3.81
SCE1572_1539	35.79	44.64
SCE1572_8220	95.87	85.86
SCE1572_4487	50.35	78.58
SCE1572_11000	27.7	10.53

SCE1572_8702	54.09	229.22
SCE1572_9568	33.14	20.84
SCE1572_10051	71.12	109.73
SCE1572_10004	34.07	14.35
SCE1572_9977	79.78	37.36
SCE1572_336	25.61	16.22
SCE1572_11574	31.81	36.49
SCE1572_4822	0	0
SCE1572_4353	131.55	258.67
SCE1572_4919	43.23	10.87
SCE1572_6558	29.62	19.33
SCE1572_10821	0	0
SCE1572_2291	123.77	386.72
SCE1572_7956	27.29	120.95
SCE1572_9425	33.37	22.7
SCE1572_7896	3031.15	118712
SCE1572_2971	52.71	23.95
SCE1572_4511	67.32	39.41
SCE1572_426	31.3	266.04
SCE1572_6790	15.77	2.92
SCE1572_5428	19.19	36.95
SCE1572_7525	96.48	117.62
SCE1572_9049	33.33	21.36
SCE1572_512	22.85	0
SCE1572_3862	50.93	94.4
SCE1572_6090	33.19	718.95
SCE1572_9185	21.46	0
SCE1572_4637	24.7	18.75
SCE1572_7396	120.65	94.72
SCE1572_10907	98.68	717.17
SCE1572_3257	26.48	12.5
SCE1572_929	36.85	73.49
SCE1572_5896	35.56	8.56
SCE1572_11331	36.45	67.8
SCE1572_10064	0	0
SCE1572_3484	23.73	15.87
SCE1572_8715	95.65	97.1
SCE1572_9419	69.61	789.52
SCE1572_5091	122.69	285.2
SCE1572_8637	33.6	33.3
SCE1572_6507	59.1	29.79
SCE1572_10407	43.18	62.83
SCE1572_9222	66.71	62.04
SCE1572_1467	277.67	480.97
SCE1572_2398	72.97	1120.35
SCE1572_7251	0	0
SCE1572_5399	0	0
SCE1572_10400	36.55	299.55

SCE1572_4243	24.27	13.66
SCE1572_2424	136.03	243.91
SCE1572_294	0	0
SCE1572_8473	135.66	53.35
SCE1572_8162	3.23	11.65
SCE1572_4395	0	0
SCE1572_8868	43.23	17.82
SCE1572_2685	0	0
SCE1572_7221	10.16	85.62
SCE1572_1197	41.12	11.67
SCE1572_11198	24.43	21.47
SCE1572_2631	41.3	24.14
SCE1572_10139	0	0
SCE1572_9005	24.21	9.2
SCE1572_8391	16.64	6.82
SCE1572_8136	16.13	7.76
SCE1572_10769	16.05	12.88
SCE1572_4632	34.41	15.47
SCE1572_5107	84.66	129.19
SCE1572_10960	8.39	6.21
SCE1572_10903	78.2	30.73
SCE1572_4613	46.19	25.38
SCE1572_5029	51.48	202.89
SCE1572_10967	34.99	16.04
SCE1572_4385	19.58	8.78
SCE1572_7560	32.18	13.41
SCE1572_7401	13.32	1109.56
SCE1572_8207	22.54	28
SCE1572_4532	0	0
SCE1572_3216	47.15	75.11
SCE1572_6303	1.8	0
SCE1572_2040	49.77	315.71
SCE1572_7043	92.86	1446
SCE1572_6022	0	0
SCE1572_8344	36.5	9.01
SCE1572_1011	17.35	5.97
SCE1572_10174	0	0
SCE1572_1063	13.15	8.33
SCE1572_8443	6.11	2.94
SCE1572_9138	39.31	5.41
SCE1572_615	4.21	0
SCE1572_10350	7.98	5.24
SCE1572_3165	38.58	14.39
SCE1572_3794	43.51	25.39
SCE1572_5150	39.82	5.53
SCE1572_6257	35.18	20.16
SCE1572_9988	24.08	11.86
SCE1572_894	57.4	90.86

SCE1572_9474	92.16	1144.85
SCE1572_8585	19.23	12.46
SCE1572_10426	12.06	16.59
SCE1572_5071	60.1	98.39
SCE1572_2272	36.69	62.98
SCE1572_10750	15.93	21.47
SCE1572_8515	15.84	12.53
SCE1572_6029	40.43	130.29
SCE1572_5278	21.58	11.69
SCE1572_2156	7.78	21.83
SCE1572_2015	5.57	5.37
SCE1572_3849	29.26	2.13
SCE1572_4604	43.25	58.21
SCE1572_2987	36.98	110.66
SCE1572_2329	47.11	28.14
SCE1572_3356	14.48	3.67
SCE1572_10308	36.3	5.14
SCE1572_7715	16.34	1.23
SCE1572_7644	37.35	32.16
SCE1572_10325	39.22	55.37
SCE1572_7438	132.86	1213.64
SCE1572_2526	19.06	4.27
SCE1572_8878	20.07	8.59
SCE1572_8837	18.95	13.03
SCE1572_6249	14.52	3.88
SCE1572_9174	77.57	83.65
SCE1572_11119	69.75	271.13
SCE1572_2585	46.72	12.73
SCE1572_10735	8.61	9.56
SCE1572_3646	63.69	119.38
SCE1572_416	29.21	14.51
SCE1572_6683	47.83	265.92
SCE1572_9588	32.77	33.94
SCE1572_3135	9.48	14.66
SCE1572_9400	11.09	35.6
SCE1572_8328	0	0
SCE1572_3492	0	0
SCE1572_393	23.76	10.89
SCE1572_9850	0	0
SCE1572_1697	111.82	118.6
SCE1572_11007	16.68	31.04
SCE1572_6234	26.04	18.66
SCE1572_10360	25.53	32.64
SCE1572_3075	43.7	27.44
SCE1572_9632	193.25	508.5
SCE1572_8067	23.65	13.39
SCE1572_3639	18.95	32.2
SCE1572_11024	138.53	372.8

SCE1572_4518	47.54	70.12
SCE1572_9313	78.39	68.42
SCE1572_9105	44.15	76.9
SCE1572_5778	40.5	50.06
SCE1572_8545	22.85	17.19
SCE1572_2328	26.29	24.86
SCE1572_4660	66.39	1075.33
SCE1572_2886	31.11	24.72
SCE1572_6515	13.61	2.98
SCE1572_716	40.3	17.86
SCE1572_11100	58.16	145.65
SCE1572_10532	0	0
SCE1572_10470	37.9	45.62
SCE1572_7912	111.53	13.37
SCE1572_10337	20.49	0
SCE1572_7947	27	0
SCE1572_2083	3.16	0
SCE1572_1110	3.37	0
SCE1572_8827	16.31	0
SCE1572_2194	35.26	21.22
SCE1572_10030	28.88	9.27
SCE1572_3730	0	0
SCE1572_8507	17.87	3.91
SCE1572_2461	55.64	0
SCE1572_6034	51.74	92.38
SCE1572_95	16.34	3.15
SCE1572_2784	38.47	9.34
SCE1572_8157	41.14	3.67
SCE1572_10079	120.4	21.47
SCE1572_9866	21.46	35.8
SCE1572_8146	63.58	61.8
SCE1572_10516	79.06	39.59
SCE1572_4218	43.17	182.46
SCE1572_9367	28.33	10.85
SCE1572_4778	26.26	87.66
SCE1572_1635	19.36	20.7
SCE1572_10025	24.09	39.6
SCE1572_5114	82.6	245.13
SCE1572_10477	34.89	106.57
SCE1572_455	34.94	27.08
SCE1572_420	13.56	10.49
SCE1572_8500	29.56	2.3
SCE1572_2734	749.92	1406.35
SCE1572_1309	1818.76	13320.88
SCE1572_3739	3.74	0
SCE1572_10881	0	0
SCE1572_5343	88.61	82.15
SCE1572_10119	34.21	30.04

SCE1572_4197	31.46	30.68
SCE1572_6065	0	0
SCE1572_10447	39.08	49.74
SCE1572_919	69.81	115.51
SCE1572_7846	2.31	1.85
SCE1572_10622	22.97	17.27
SCE1572_8994	37.54	62.19
SCE1572_7248	31.1	9.36
SCE1572_7569	24.07	21.49
SCE1572_11422	37.9	37.63
SCE1572_7070	2.81	0
SCE1572_11586	19.89	46.58
SCE1572_910	48.18	28.96
SCE1572_1202	34.76	11.54
SCE1572_11136	42.1	57.4
SCE1572_7782	0	0
SCE1572_1236	82.54	91.75
SCE1572_9647	148.17	181.31
SCE1572_1985	161.07	7565.41
SCE1572_7882	32.36	51.96
SCE1572_7145	55.49	37.62
SCE1572_2384	61.9	113.83
SCE1572_236	7.71	0
SCE1572_2647	52.89	47.52
SCE1572_1819	37.15	20.14
SCE1572_2377	23.01	136.85
SCE1572_7936	30.21	12.58
SCE1572_2356	55.92	198.91
SCE1572_9303	117.92	683.06
SCE1572_5814	15.57	7.14
SCE1572_1463	35.94	52.26
SCE1572_10889	27.36	42.34
SCE1572_1495	0	0
SCE1572_1663	0	0
SCE1572_11029	4.79	0
SCE1572_1707	591.8	139.55
SCE1572_9228	0	0
SCE1572_153	33.39	19.55
SCE1572_2861	0	0
SCE1572_723	36.31	12.27
SCE1572_2308	37.5	99.6
SCE1572_604	53.17	154.91
SCE1572_679	88.27	89.98
SCE1572_10686	24.71	119.68
SCE1572_1971	15.27	21.79
SCE1572_10373	49.89	60.82
SCE1572_4806	38.58	124.07
SCE1572_5487	61.77	43.25

SCE1572_1965	40.31	13.03
SCE1572_5539	43.13	102.72
SCE1572_10759	5.76	13.86
SCE1572_173	11.66	0
SCE1572_6537	29.43	20.05
SCE1572_10794	26.6	33.61
SCE1572_3764	33.16	17.05
SCE1572_6084	39.23	45.93
SCE1572_693	56.12	41.07
SCE1572_666	85.4	351.06
SCE1572_1978	47.03	501.52
SCE1572_3896	88.44	585.59
SCE1572_10251	2.3	0
SCE1572_5291	10.34	11.06
SCE1572_1394	23.17	83.29
SCE1572_4340	44.1	16.64
SCE1572_10897	18.54	39.67
SCE1572_682	36.47	8.73
SCE1572_7292	13.16	6.03
SCE1572_9259	30.85	19.09
SCE1572_8790	63.69	79.15
SCE1572_7469	38.68	42.45
SCE1572_6260	85.84	43.79
SCE1572_1956	6.63	2.28
SCE1572_2918	14.88	13.43
SCE1572_2174	38.14	11.33
SCE1572_6393	189.98	353.2
SCE1572_8176	57.58	15.09
SCE1572_3842	23.27	24.01
SCE1572_9839	0	0
SCE1572_9291	23.69	114.04
SCE1572_2569	16.58	17.11
SCE1572_5878	42.62	30.57
SCE1572_6621	25.27	16.97
SCE1572_1250	20.95	21.61
SCE1572_2481	220.87	1254.7
SCE1572_1443	57.24	24.19
SCE1572_9605	6.54	0
SCE1572_2851	22.58	5.18
SCE1572_4807	70.04	106.39
SCE1572_5850	54.33	28.38
SCE1572_11390	47.79	40.99
SCE1572_3588	109.57	24.53
SCE1572_11314	432.17	594.79
SCE1572_9561	40.88	14.73
SCE1572_10297	16.18	34.62
SCE1572_2444	7.22	8.69
SCE1572_5692	15.45	13.34

SCE1572_134	0	0
SCE1572_2911	46.95	21.77
SCE1572_6578	38.81	21.72
SCE1572_8029	45.94	84.78
SCE1572_10161	32.68	43.7
SCE1572_7405	52.8	67.17
SCE1572_9112	18.44	20.66
SCE1572_4674	54.35	34.43
SCE1572_7949	63.98	141.86
SCE1572_2574	13.57	13.44
SCE1572_8803	14.61	54.96
SCE1572_4714	42.24	18.16
SCE1572_1901	10.07	59.25
SCE1572_4723	73.46	75.24
SCE1572_6809	97.52	593
SCE1572_10509	22.93	70.53
SCE1572_2391	99.75	2855.36
SCE1572_2743	27.93	45.62
SCE1572_6907	27.07	11.17
SCE1572_5152	0	0
SCE1572_5867	37.21	13.27
SCE1572_1846	33.17	17.11
SCE1572_5743	19.09	147.25
SCE1572_1734	15.04	6.03
SCE1572_9620	12.38	7.45
SCE1572_8600	6.59	5.29
SCE1572_6169	0	0
SCE1572_828	18.45	4.16
SCE1572_8034	33.06	21.95
SCE1572_742	38.45	79.76
SCE1572_1727	66.03	52.97
SCE1572_11246	73.1	17.11
SCE1572_5776	25.46	58.5
SCE1572_8817	0	0
SCE1572_10285	8.37	18.88
SCE1572_8917	33.48	97.31
SCE1572_2543	30.28	14.01
SCE1572_8198	2.49	0
SCE1572_7195	61.07	20.56
SCE1572_10630	71.79	28.37
SCE1572_1356	112.39	642.7
SCE1572_1508	66.09	18.71
SCE1572_6883	96.38	78.68
SCE1572_7908	0	0
SCE1572_7578	43.52	253.42
SCE1572_3102	14.1	38.19
SCE1572_7803	2.81	0
SCE1572_9840	92.41	0

SCE1572_11005	37.41	42.34
SCE1572_9430	20.43	8.85
SCE1572_7844	176.89	5392.11
SCE1572_11045	41.73	16.74
SCE1572_1832	31.37	34.6
SCE1572_8052	55.28	5.7
SCE1572_7489	14.21	125.44
SCE1572_6724	38.1	49.24
SCE1572_6093	24.31	9.75
SCE1572_1386	0	0
SCE1572_2617	58.96	552.45
SCE1572_6804	42.62	19.05
SCE1572_8992	42.59	68.34
SCE1572_87	37.19	39.59
SCE1572_9864	11.66	6.6
SCE1572_2985	19.07	17.66
SCE1572_1161	31.15	6.67
SCE1572_10306	34.83	115.89
SCE1572_2006	16.03	67.36
SCE1572_3636	73.45	16.22
SCE1572_5221	40.43	52.13
SCE1572_9747	23.65	42.33
SCE1572_6651	6.5	20.85
SCE1572_6908	58.07	11.09
SCE1572_7666	8.36	11.49
SCE1572_10489	58.6	639.05
SCE1572_214	68.51	13.52
SCE1572_6837	51.49	105.58
SCE1572_4950	48.21	33.59
SCE1572_5054	16.46	14.6
SCE1572_5492	26.03	20.67
SCE1572_11260	134.66	150.05
SCE1572_7117	74.68	44.26
SCE1572_4705	11.31	10.89
SCE1572_7616	72.65	104.75
SCE1572_8172	27.26	10.93
SCE1572_1056	32.01	23.07
SCE1572_3252	156.56	110.5
SCE1572_7940	135.8	240.3
SCE1572_5188	28.54	761.07
SCE1572_1119	26.01	6.85
SCE1572_7815	973.08	12086.12
SCE1572_7580	14.18	53.11
SCE1572_3920	93.74	29.59
SCE1572_9293	18.48	47.9
SCE1572_5832	22.81	39.64
SCE1572_1481	39.84	21.17
SCE1572_3558	9.24	0

SCE1572_3828	65.08	224.95
SCE1572_1419	6.79	0
SCE1572_419	20.71	40.26
SCE1572_1501	0	0
SCE1572_144	41.35	29.49
SCE1572_5131	11.04	7.25
SCE1572_6385	0	0
SCE1572_4172	58.41	56.81
SCE1572_10688	98.52	1160.35
SCE1572_4452	20.11	9.31
SCE1572_8416	89.96	218.04
SCE1572_3628	28.27	18.56
SCE1572_3546	64.98	9.07
SCE1572_5752	225.17	528.92
SCE1572_5127	20.24	15.53
SCE1572_620	46.83	73.53
SCE1572_10809	4.94	11.9
SCE1572_5722	18.66	17.97
SCE1572_4957	3.07	2.47
SCE1572_2253	200.97	258.98
SCE1572_5957	31.33	41.36
SCE1572_538	24.96	52.9
SCE1572_4404	0	0
SCE1572_11568	138.51	935.97
SCE1572_8646	116.02	63.46
SCE1572_10264	61.34	28.79
SCE1572_5960	20.14	10.77
SCE1572_3436	25.57	58.67
SCE1572_9409	41.48	431.19
SCE1572_10671	17.3	3.97
SCE1572_9235	24.04	4.82
SCE1572_8910	41.51	21.72
SCE1572_1180	15.62	23.49
SCE1572_1006	99.04	248.68
SCE1572_8278	26.2	18.61
SCE1572_10454	0	0
SCE1572_8689	40.76	36.62
SCE1572_8458	80.51	635.34
SCE1572_2038	20.77	33.74
SCE1572_3222	44.74	110.07
SCE1572_1189	37.99	59.53
SCE1572_347	39.97	90.09
SCE1572_8846	15.74	25.25
SCE1572_10979	31.22	39.02
SCE1572_1401	20.43	16.39
SCE1572_10677	16.04	48.27
SCE1572_4966	16.39	26.3
SCE1572_3038	65.62	20.83

SCE1572_957	51.71	169.43
SCE1572_7710	22.63	65.36
SCE1572_272	0	0
SCE1572_4163	56.9	24.33
SCE1572_5331	37.33	77.64
SCE1572_1157	17.11	8.01
SCE1572_2781	6.63	9.12
SCE1572_920	3.3	0
SCE1572_6623	36.91	24.61
SCE1572_1314	416.94	3959.41
SCE1572_9289	15.87	25.46
SCE1572_4184	53	221.03
SCE1572_1720	18.95	53.67
SCE1572_5858	3.7	0
SCE1572_3386	2.61	0
SCE1572_1582	76.67	22.03
SCE1572_3888	46.41	12.86
SCE1572_9063	91.15	20.64
SCE1572_8540	18.24	10.79
SCE1572_3381	33.91	41.28
SCE1572_8614	86.92	130.55
SCE1572_10445	23.33	19.75
SCE1572_2994	2.53	0
SCE1572_3607	23.14	15
SCE1572_3653	11.97	0
SCE1572_9497	49.05	75.64
SCE1572_2494	27.18	16.35
SCE1572_3280	24.68	39.26
SCE1572_5348	160.57	225.94
SCE1572_8186	0	0
SCE1572_10651	69.58	177.47
SCE1572_2649	27.83	18.51
SCE1572_9087	61.99	22.81
SCE1572_4975	346.74	2597.64
SCE1572_10757	32.75	32.31
SCE1572_6868	11.23	3.38
SCE1572_1518	31.44	23.4
SCE1572_5017	38.67	41.25
SCE1572_818	12.13	4.87
SCE1572_791	61.63	280.86
SCE1572_6863	55.75	20.27
SCE1572_9922	0	0
SCE1572_7428	18.02	8.23
SCE1572_4034	217.26	205.03
SCE1572_10238	73.83	31.73
SCE1572_6815	51.9	22.71
SCE1572_5764	79.86	41.96
SCE1572_1690	14.21	7.6

SCE1572_2755	102.49	72.76
SCE1572_7694	25.16	33.31
SCE1572_10853	82.07	36.83
SCE1572_1712	17.39	8.19
SCE1572_2500	33.41	8.25
SCE1572_627	29	36.63
SCE1572_1093	16.28	22.04
SCE1572_10611	17.95	43.21
SCE1572_4248	24.87	69.56
SCE1572_7066	58.04	14.25
SCE1572_1044	14.64	22.65
SCE1572_2109	58.42	462.01
SCE1572_1679	40.88	127.76
SCE1572_8848	54.4	30.31
SCE1572_3688	63.63	21.56
SCE1572_203	44.26	42.86
SCE1572_2045	25.18	30.3
SCE1572_9969	16.2	26.51
SCE1572_4029	166.86	183.43
SCE1572_7212	2.45	0
SCE1572_5431	23.07	15.87
SCE1572_2059	69.09	15.71
SCE1572_2503	64.86	25.37
SCE1572_3784	26.14	1184.42
SCE1572_6732	38.18	162.77
SCE1572_2309	14.27	49.37
SCE1572_657	848.88	707.17
SCE1572_5021	68.24	271.68
SCE1572_8215	18.14	3.12
SCE1572_190	29.23	18.76
SCE1572_7492	20.09	12.09
SCE1572_4131	41.69	54.74
SCE1572_7851	43.4	78.68
SCE1572_10848	26.14	15.49
SCE1572_4642	91.15	31.97
SCE1572_4834	0	0
SCE1572_9966	9.23	6.35
SCE1572_2169	25.27	18.8
SCE1572_10410	38.94	55.65
SCE1572_654	0	0
SCE1572_11296	19.85	20.09
SCE1572_9302	70.89	113.74
SCE1572_8085	0	0
SCE1572_6190	51.36	19.47
SCE1572_11209	20.62	22.06
SCE1572_5737	118.49	143.27
SCE1572_11285	47.29	1103.14
SCE1572_3339	266.23	4016.62

SCE1572_4082	0	0
SCE1572_4785	35.55	41.32
SCE1572_1210	2.23	0
SCE1572_8377	0	0
SCE1572_7706	16.8	17.75
SCE1572_5872	81.68	78.87
SCE1572_3520	13.58	0
SCE1572_4410	0	0
SCE1572_9490	48.6	72.49
SCE1572_10860	446.87	746.31
SCE1572_340	36.39	11.35
SCE1572_6879	73.66	136.91
SCE1572_11213	13.78	12.44
SCE1572_4258	57.99	218.08
SCE1572_4845	20.96	26.33
SCE1572_8954	21.77	27.74
SCE1572_4687	16.1	10
SCE1572_6528	15.42	9.28
SCE1572_10392	14.44	13.9
SCE1572_1745	56.92	23.71
SCE1572_4099	26.95	38.93
SCE1572_197	53.84	233.62
SCE1572_1627	189.78	168.95
SCE1572_2875	25.8	36.75
SCE1572_9368	9.59	1.44
SCE1572_9294	39.57	348.87
SCE1572_1923	23.74	17.51
SCE1572_380	25.91	4.62
SCE1572_7520	9.97	12.63
SCE1572_11231	32.04	16.28
SCE1572_3337	332.73	5135.07
SCE1572_8876	55.9	17.42
SCE1572_7182	52.27	15.87
SCE1572_7246	46.29	41.78
SCE1572_885	161.38	42.06
SCE1572_5133	13.78	16.59
SCE1572_8059	0	0
SCE1572_6870	73.32	230.94
SCE1572_3163	82.37	44.41
SCE1572_3876	27.71	15.7
SCE1572_639	46.04	45.17
SCE1572_9447	0	0
SCE1572_5748	27.4	34.32
SCE1572_9472	136.51	107.59
SCE1572_7104	49.41	23.78
SCE1572_5464	33.54	14.53
SCE1572_8622	44.22	40.55
SCE1572_8517	30.42	14.64

SCE1572_11504	10.34	3.32
SCE1572_10604	30.92	26.9
SCE1572_4468	9.75	10.56
SCE1572_8003	95.68	36.92
SCE1572_1572	55.22	33.83
SCE1572_949	147.43	149.77
SCE1572_8434	16.17	13.26
SCE1572_6546	101.96	67.39
SCE1572_7498	26.89	8.93
SCE1572_7218	50.93	80.31
SCE1572_7235	16.72	23.43
SCE1572_8407	14.33	31.61
SCE1572_10121	104.29	225.9
SCE1572_3919	18.59	6.14
SCE1572_510	24.9	24.68
SCE1572_5307	39.9	43.21
SCE1572_1103	131.76	114.69
SCE1572_9013	2.49	0
SCE1572_10273	83.14	184.42
SCE1572_3972	129.96	238.94
SCE1572_4635	43.86	20.66
SCE1572_2651	56.9	19.3
SCE1572_1936	36.83	31.64
SCE1572_250	18.01	21.68
SCE1572_10209	0	0
SCE1572_10514	30.12	37.46
SCE1572_5679	98.82	265
SCE1572_4755	25.22	21.79
SCE1572_2621	42.65	28.17
SCE1572_8106	90.54	16.45
SCE1572_4793	13.1	31.54
SCE1572_3184	7.11	0
SCE1572_1685	14.66	2.58
SCE1572_2192	0	0
SCE1572_8466	88.21	200.71
SCE1572_2513	61.37	198.1
SCE1572_1976	42.29	315.08
SCE1572_4241	43.07	31.1
SCE1572_1767	49.85	38.06
SCE1572_5406	4.7	4.24
SCE1572_3276	19.29	65.86
SCE1572_6605	18.36	198.84
SCE1572_8948	11.26	10.43
SCE1572_1669	14.15	104.61
SCE1572_1759	27.38	10.14
SCE1572_5687	0	0
SCE1572_3018	46.43	16.81
SCE1572_3512	15.09	14.52

SCE1572_5204	90.97	214.01
SCE1572_9161	11.94	24.99
SCE1572_4968	21.17	5.99
SCE1572_10035	18.95	8.29
SCE1572_296	28.92	67.72
SCE1572_4054	50.95	155.54
SCE1572_3454	34.08	24.91
SCE1572_1061	33.43	100.57
SCE1572_5885	33.79	16.49
SCE1572_6456	21.41	8.25
SCE1572_833	126.49	225.21
SCE1572_4078	28.83	15.55
SCE1572_7601	41.35	39.4
SCE1572_3269	31.05	172.94
SCE1572_2599	42.65	24.82
SCE1572_8901	12.59	17.21
SCE1572_817	0	0
SCE1572_7083	30.15	153.65
SCE1572_2199	49.73	28.29
SCE1572_9857	59.68	126.17
SCE1572_3701	42.17	26.58
SCE1572_6670	36.15	2.42
SCE1572_6658	371.67	293.97
SCE1572_10168	24.66	10.99
SCE1572_5804	32.35	53.44
SCE1572_7717	10.69	2.03
SCE1572_38	94.09	323.66
SCE1572_8169	57.59	14.14
SCE1572_3365	7.25	2.42
SCE1572_1367	43.32	91.8
SCE1572_279	0	0
SCE1572_4061	17.56	43.3
SCE1572_7510	3.94	0
SCE1572_4397	21.85	12.75
SCE1572_1348	195.46	885.68
SCE1572_4852	16.24	24.2
SCE1572_5272	40.86	16.39
SCE1572_11252	0	0
SCE1572_6027	11.05	10.34
SCE1572_1680	88.86	628.25
SCE1572_8123	10.64	0
SCE1572_1895	26.76	11.56
SCE1572_9617	30.32	20.01
SCE1572_7691	23.33	62.75
SCE1572_6076	0	0
SCE1572_2145	19.98	82.24
SCE1572_3285	43.05	49.5
SCE1572_4947	20	41.81

SCE1572_8660	16.48	15.87
SCE1572_10870	48.1	8.79
SCE1572_2773	145.02	66.31
SCE1572_4342	16.57	10.46
SCE1572_1653	70.22	0
SCE1572_5418	19.68	21.77
SCE1572_9043	11.9	16.63
SCE1572_5658	55.57	24.46
SCE1572_7704	17.29	19.21
SCE1572_1673	48.43	180.81
SCE1572_4592	17.69	41.97
SCE1572_8970	35.52	535.91
SCE1572_8861	54.72	18.97
SCE1572_1429	23.2	50.65
SCE1572_5147	19.46	0.76
SCE1572_4009	45.29	21.27
SCE1572_1476	53.73	16.65
SCE1572_3800	0	0
SCE1572_6561	27.77	5.42
SCE1572_10320	13.48	2.03
SCE1572_6480	32.91	37.6
SCE1572_1860	44.78	68.52
SCE1572_3911	147.23	1628.44
SCE1572_704	57.31	233.97
SCE1572_6224	41.48	154.92
SCE1572_9405	25.92	10.26
SCE1572_10067	335.4	884.71
SCE1572_5102	45.32	178.05
SCE1572_644	83.27	158.71
SCE1572_9563	31.07	13.97
SCE1572_3441	19.07	55.52
SCE1572_5585	57.34	10.62
SCE1572_4407	24.26	9.73
SCE1572_5353	75.51	139.91
SCE1572_9099	356.26	4709.09
SCE1572_10918	12.31	65.5
SCE1572_873	20.22	0
SCE1572_3330	42.64	590.15
SCE1572_10877	75.59	39.28
SCE1572_834	101.08	281.03
SCE1572_1424	23.57	100.13
SCE1572_2366	51.3	34.96
SCE1572_4162	72.2	50.68
SCE1572_7374	10.36	11.33
SCE1572_7347	24.45	8.83
SCE1572_8566	45.7	143.18
SCE1572_200	20.75	18.35
SCE1572_10095	21.79	395.51

SCE1572_9680	104.71	327.77
SCE1572_5376	42.8	15.97
SCE1572_7098	34.46	24.88
SCE1572_10260	23.43	12.53
SCE1572_6966	27.23	20.55
SCE1572_3201	47.73	0
SCE1572_4589	20.94	12.1
SCE1572_7758	13.28	1.33
SCE1572_5944	48.6	23.57
SCE1572_11326	25.71	7.58
SCE1572_5031	400.75	3340.84
SCE1572_2228	94.91	125.6
SCE1572_8696	0	0
SCE1572_7015	18.28	18.12
SCE1572_6009	137.18	369.27
SCE1572_9577	2.23	1.79
SCE1572_5917	68.83	230.54
SCE1572_7441	118.29	18.05
SCE1572_8402	68.25	35.11
SCE1572_10156	68.63	169.02
SCE1572_2266	29.1	30.69
SCE1572_1392	19.02	5.09
SCE1572_2203	31.37	207.14
SCE1572_5593	21.9	8.69
SCE1572_11493	25.22	9.74
SCE1572_7386	53.36	22.43
SCE1572_4294	36.73	22.67
SCE1572_9195	47.98	27.72
SCE1572_4766	59.51	73.33
SCE1572_2518	58.15	54.97
SCE1572_3697	14.84	12.42
SCE1572_2050	45.05	29.15
SCE1572_1549	37.9	71.88
SCE1572_9662	116.39	653.67
SCE1572_10246	0	0
SCE1572_974	0	0
SCE1572_2234	43	25.05
SCE1572_7973	8.68	0
SCE1572_10465	63.99	30.53
SCE1572_9903	23.69	27.56
SCE1572_5219	5.29	4.24
SCE1572_6062	59.09	155.4
SCE1572_7305	62.67	24.33
SCE1572_6945	9.36	0
SCE1572_3931	435.54	1413.22
SCE1572_6985	22.49	1.87
SCE1572_4238	0	0
SCE1572_1568	44.76	32.79

SCE1572_1246	13.38	2.3
SCE1572_1839	11.15	5.37
SCE1572_9277	30.23	18.74
SCE1572_4206	41.47	114.06
SCE1572_3665	0	0
SCE1572_11161	42.21	24.88
SCE1572_436	31.7	25.17
SCE1572_5171	48.59	252.31
SCE1572_6461	36.9	29.06
SCE1572_566	48.23	35.27
SCE1572_8932	26.93	39.21
SCE1572_2698	23.96	6.96
SCE1572_1857	0	0
SCE1572_7679	66.83	248.74
SCE1572_5236	47.05	77.07
SCE1572_9535	0	0
SCE1572_4657	33.11	157.29
SCE1572_75	10.46	5.59
SCE1572_10868	13.46	3.36
SCE1572_2028	102.47	405.02
SCE1572_3815	441.13	3721.49
SCE1572_9242	11.66	5.61
SCE1572_1158	15.35	5.6
SCE1572_2820	73.22	189.53
SCE1572_3461	42.92	13.33
SCE1572_1879	25.4	29.64
SCE1572_3813	0	0
SCE1572_3478	26.92	92.08
SCE1572_6799	72.04	20.4
SCE1572_3391	13.4	20.16
SCE1572_2767	23.94	34.57
SCE1572_7451	31.26	69.6
SCE1572_10217	17.59	114.3
SCE1572_6266	22.05	33.17
SCE1572_29	47.51	77.36
SCE1572_9708	27.55	60.74
SCE1572_2375	8.99	18.56
SCE1572_5935	38.58	12.43
SCE1572_11374	79.6	991.68
SCE1572_1528	55.25	54.47
SCE1572_10869	48.46	11.73
SCE1572_4221	81.41	631.87
SCE1572_9361	0	0
SCE1572_10240	0	0
SCE1572_2658	249.19	189.45
SCE1572_4290	19.09	38.26
SCE1572_8488	67.93	31.97
SCE1572_6013	20.07	38.52

SCE1572_9376	5.41	0
SCE1572_2248	80.87	28.38
SCE1572_5910	44.06	114.36
SCE1572_2800	79.2	29.38
SCE1572_9713	16.05	290.51
SCE1572_244	39.13	17.21
SCE1572_11033	56.1	25.54
SCE1572_7763	0	0
SCE1572_11545	43.14	22.18
SCE1572_5094	47.11	239.16
SCE1572_4	129.85	501.53
SCE1572_3677	26.93	16.81
SCE1572_10789	49.73	125.53
SCE1572_5002	199.22	1413.85
SCE1572_5338	24.59	6.58
SCE1572_8420	137.74	51.4
SCE1572_1332	26.7	6.89
SCE1572_7540	60.03	149.44
SCE1572_4695	7.62	6.67
SCE1572_3139	17.42	983.81
SCE1572_4288	17.41	33.4
SCE1572_6701	60.41	19.11
SCE1572_8411	44.8	78.79
SCE1572_2642	42.52	24.81
SCE1572_355	48.86	16.97
SCE1572_827	23.77	9.28
SCE1572_1665	97.07	182.98
SCE1572_1567	8.61	0
SCE1572_3737	51.15	13.19
SCE1572_7464	176.46	167
SCE1572_5470	75.81	13.34
SCE1572_11478	119.76	162.08
SCE1572_3248	6.55	2.25
SCE1572_4938	3.94	0
SCE1572_11188	1.94	0
SCE1572_5975	15.96	20.04
SCE1572_5472	47.2	19.42
SCE1572_9938	4.59	0
SCE1572_4480	16.8	13.48
SCE1572_2450	22.95	1.45
SCE1572_5787	25.91	154.75
SCE1572_11113	12.42	2.03
SCE1572_5043	30.54	131.27
SCE1572_5553	40.31	17.38
SCE1572_2614	23.91	26.94
SCE1572_9018	65.94	44.77
SCE1572_286	29.66	0
SCE1572_7649	41.45	19.57

SCE1572_5411	22.86	11.58
SCE1572_7484	28.23	28.94
SCE1572_77	18.81	8.31
SCE1572_3938	177.23	190.01
SCE1572_3904	0	0
SCE1572_579	20.59	216.25
SCE1572_10435	23.54	38.41
SCE1572_1036	52.63	15.13
SCE1572_7825	15.74	11.36
SCE1572_1099	34.67	143.76
SCE1572_4237	28.94	27.03
SCE1572_7366	117.74	1821.63
SCE1572_6590	8.2	16.44
SCE1572_2430	41.2	67.43
SCE1572_6633	71.34	20.17
SCE1572_7587	139.99	1619.34
SCE1572_5899	31.52	16.26
SCE1572_6205	91.52	23.84
SCE1572_4375	14.9	19.27
SCE1572_1607	29.92	8.54
SCE1572_3649	17.94	10.8
SCE1572_3742	16.97	13.05
SCE1572_6313	36.39	14.6
SCE1572_11304	47.47	116.81
SCE1572_8776	78.62	13.52
SCE1572_10592	25.97	4.36
SCE1572_3404	20.86	28.46
SCE1572_4384	28.4	11.57
SCE1572_9933	30.95	22.69
SCE1572_9333	33.32	368.93
SCE1572_2934	39.69	137.85
SCE1572_4545	50.11	8.32
SCE1572_8596	20.67	4.15
SCE1572_11211	72.93	29.79
SCE1572_5515	49.85	20.7
SCE1572_6464	22.48	7.34
SCE1572_7902	30.16	7.64
SCE1572_3208	68.39	442.57
SCE1572_11277	14.62	34.36
SCE1572_9652	45.7	37.01
SCE1572_5294	41.89	15.34
SCE1572_7505	31.48	18.61
SCE1572_3528	25.51	3442.32
SCE1572_9676	9.36	13.52
SCE1572_1980	169.51	1086.39
SCE1572_11369	36.24	4.59
SCE1572_4012	27.22	20.78
SCE1572_9876	9.33	2.81

SCE1572_11225	33.91	22.81
SCE1572_8295	62.05	159.21
SCE1572_8767	18.12	4.59
SCE1572_4540	44.38	20.36
SCE1572_4209	49.53	157.09
SCE1572_5210	6.94	2.79
SCE1572_1644	19.35	18.41
SCE1572_2536	74.74	73.24
SCE1572_5640	44.95	16.63
SCE1572_6431	95.79	50.59
SCE1572_5829	22.72	3.77
SCE1572_3444	54.06	18.59
SCE1572_10583	13.27	0
SCE1572_500	27.32	0
SCE1572_5602	16.88	57.37
SCE1572_9796	122.79	59.11
SCE1572_5714	17.76	29.01
SCE1572_6184	26.76	8.35
SCE1572_6357	16.02	8.46
SCE1572_10271	41.38	18.89
SCE1572_1713	27.07	40.96
SCE1572_4934	227.88	439.86
SCE1572_3171	97.74	453.84
SCE1572_8683	31.12	65.36
SCE1572_8262	16.76	12.53
SCE1572_10998	26.12	81.26
SCE1572_1878	33.11	41.95
SCE1572_4432	51.74	12.11
SCE1572_844	67.12	230.69
SCE1572_4621	48.22	230.92
SCE1572_8363	87.59	28.9
SCE1572_11511	34.2	32.05
SCE1572_8042	47.7	16.4
SCE1572_7547	49.1	23.84
SCE1572_1092	0	0
SCE1572_10941	51.26	11.69
SCE1572_8315	44.77	34.48
SCE1572_10547	19.38	11.66
SCE1572_4603	106.66	556.86
SCE1572_11320	27.15	3.19
SCE1572_2525	18.38	11.8
SCE1572_10571	67.28	150.82
SCE1572_11013	27.96	31.91
SCE1572_1361	38.34	146.81
SCE1572_5355	26.37	31.73
SCE1572_291	0	0
SCE1572_8049	49.83	5.14
SCE1572_3227	24.05	4.14

SCE1572_8072	32.78	14.09
SCE1572_768	58.18	69.2
SCE1572_2315	99.97	497.26
SCE1572_4049	35.39	263.62
SCE1572_4281	33.9	20.4
SCE1572_952	12.63	34.75
SCE1572_9603	16.34	3.15
SCE1572_10831	53.09	34.85
SCE1572_2906	66.44	15.38
SCE1572_28	29.88	53.93
SCE1572_5440	63.17	41.36
SCE1572_4431	13.42	5.7
SCE1572_9827	38.84	37.7
SCE1572_9217	48.24	91.72
SCE1572_634	49.48	171.9
SCE1572_4944	17.37	40.19
SCE1572_6167	49.6	15.77
SCE1572_11168	21.63	15.58
SCE1572_2814	40.71	227.23
SCE1572_8310	36.23	16.09
SCE1572_3176	38.53	49.41
SCE1572_4231	80.41	38.95
SCE1572_6527	0	0
SCE1572_9945	764.19	1400.89
SCE1572_225	0	0
SCE1572_11382	38.31	241.66
SCE1572_367	32.7	71.55
SCE1572_488	93.15	72.33
SCE1572_770	47.25	100.14
SCE1572_7848	39.25	45.21
SCE1572_4169	53.66	150.34
SCE1572_10951	33.38	37.94
SCE1572_2439	11.08	21.34
SCE1572_2333	50.65	116.36
SCE1572_4141	18.84	10.55
SCE1572_572	22.11	22.81
SCE1572_3660	37.4	48.66
SCE1572_6574	40.22	20.05
SCE1572_10828	46.2	28.51
SCE1572_515	78.11	20.89
SCE1572_4768	28.46	15.72
SCE1572_8079	22.63	0
SCE1572_5243	10.53	0
SCE1572_7954	184.73	16.78
SCE1572_7832	41.83	264.25
SCE1572_7312	25.04	16.29
SCE1572_9143	45.68	32.28
SCE1572_7284	28.16	1.74

SCE1572_7150	15.48	11.73
SCE1572_6399	47.83	427.57
SCE1572_3211	3.63	0
SCE1572_10493	79.28	410.24
SCE1572_4156	14.66	16.88
SCE1572_6489	24.75	29.79
SCE1572_10228	31.52	0
SCE1572_6280	14.71	298.71
SCE1572_1260	49.14	19.18
SCE1572_5432	57.61	14.6
SCE1572_5824	16.77	20.18
SCE1572_8779	30.58	162.53
SCE1572_7146	62.84	38.41
SCE1572_11400	47.99	164.8
SCE1572_6993	17.48	7.21
SCE1572_10958	31.31	23.68
SCE1572_8607	97.13	350.26
SCE1572_3143	34	18.78
SCE1572_5252	41.58	399.29
SCE1572_58	36.8	34.01
SCE1572_1861	67.28	59.3
SCE1572_10475	29.89	46.99
SCE1572_11424	17.1	100.71
SCE1572_2881	44.72	32.82
SCE1572_2787	35.21	166.51
SCE1572_602	54.43	67.28
SCE1572_2955	2.49	0
SCE1572_2839	42.71	27.24
SCE1572_585	23.36	30.46
SCE1572_11199	10.43	0
SCE1572_2792	29.55	11.85
SCE1572_3776	50	50.47
SCE1572_9198	27.93	16.81
SCE1572_1987	14.83	7.93
SCE1572_8450	30.03	10.02
SCE1572_5012	18.51	83.95
SCE1572_10687	10.96	17.59
SCE1572_8304	46.28	32.65
SCE1572_1701	73.42	122.21
SCE1572_3769	47.63	406.9
SCE1572_8505	15.72	14.29
SCE1572_1494	37.67	20.4
SCE1572_1881	36.88	7.89
SCE1572_4958	23.02	3.61
SCE1572_6216	57.8	27.82
SCE1572_8710	28.21	25.46
SCE1572_10894	18.95	36.49
SCE1572_6547	35.9	42.16

SCE1572_2506	40.85	17.02
SCE1572_2016	48.9	22.47
SCE1572_5434	34.48	17.84
SCE1572_6465	3.73	2.69
SCE1572_10608	24.23	111.59
SCE1572_4665	8.71	2.1
SCE1572_8513	15.15	4.51
SCE1572_2305	16.01	67.24
SCE1572_4548	43.62	0
SCE1572_2883	78.84	531.33
SCE1572_10409	24.19	58.23
SCE1572_5624	0	0
SCE1572_2470	8.57	0.94
SCE1572_6909	54.87	0
SCE1572_4872	6.7	4.36
SCE1572_369	17.92	3.32
SCE1572_730	13.84	13.44
SCE1572_1276	19.89	9.77
SCE1572_8171	48.5	17.2
SCE1572_5875	7.02	6.76
SCE1572_1149	616.74	2322.51
SCE1572_3358	14.53	6.08
SCE1572_5823	32.39	4.98
SCE1572_2428	120.18	244.76
SCE1572_9564	14.84	10.87
SCE1572_5077	145.41	638.08
SCE1572_1257	31.45	104.38
SCE1572_5441	58.18	33.95
SCE1572_6756	32.98	25.77
SCE1572_5321	39.36	38.6
SCE1572_8199	32.29	15.24
SCE1572_7648	45.77	24.48
SCE1572_7662	23.45	5.64
SCE1572_10193	29.36	9.68
SCE1572_8309	38.5	105.33
SCE1572_32	21.29	10.87
SCE1572_1194	41.96	57.23
SCE1572_10317	20.04	13.61
SCE1572_8724	34.35	19.96
SCE1572_1907	26.4	76.57
SCE1572_4174	9.28	9.31
SCE1572_2441	48.61	39.54
SCE1572_6154	54.15	7.45
SCE1572_10733	15.55	152.83
SCE1572_684	27.94	12.86
SCE1572_9039	23.05	29.88
SCE1572_10797	17.22	14.72
SCE1572_11413	45.71	23.52

SCE1572_9508	18.09	9.01
SCE1572_9890	54.25	24.48
SCE1572_9501	135.27	395.33
SCE1572_10790	214.86	771.95
SCE1572_4216	215.66	406.2
SCE1572_7406	25.11	17.91
SCE1572_530	70.53	40.18
SCE1572_10776	137.95	197.95
SCE1572_2201	64.54	539.57
SCE1572_10062	433.82	1307.86
SCE1572_1453	86.21	40.16
SCE1572_2736	15.41	30.08
SCE1572_4804	53.49	22.05
SCE1572_6225	41.35	26.86
SCE1572_7432	43.46	55.78
SCE1572_10491	68.92	213.02
SCE1572_11449	118.49	391.87
SCE1572_4273	64.48	42.99
SCE1572_5581	43.69	170.89
SCE1572_2553	10.52	4.68
SCE1572_100	23.29	14.45
SCE1572_1445	39.93	30.3
SCE1572_2251	73.5	60.65
SCE1572_7687	19.99	14.08
SCE1572_7148	66.48	294.74
SCE1572_2131	20.95	11.92
SCE1572_5485	37.41	16.6
SCE1572_9899	84.46	453.09
SCE1572_2104	100.62	926.68
SCE1572_8361	30.73	4.93
SCE1572_4909	4.97	0
SCE1572_10636	13.38	0
SCE1572_5707	11.81	16.5
SCE1572_7910	10.42	2.28
SCE1572_2916	49.46	12.94
SCE1572_7778	21.66	16.82
SCE1572_7249	42.32	14.09
SCE1572_9321	13.43	6.93
SCE1572_7124	42.07	65.05
SCE1572_2446	42.54	27.3
SCE1572_4023	61.76	645.82
SCE1572_10364	20.38	72.59
SCE1572_4898	32.1	14.79
SCE1572_3428	24.63	33.39
SCE1572_3880	30.43	21.91
SCE1572_6086	2.37	0
SCE1572_6531	33.18	30.33
SCE1572_9268	41.09	62.53

SCE1572_11048	27.19	41.11
SCE1572_3729	11.66	4.68
SCE1572_9100	49.9	680.65
SCE1572_3207	32.01	371.7
SCE1572_2154	28.43	13.68
SCE1572_6617	43.8	5.41
SCE1572_2159	0	0
SCE1572_2568	48.73	36.49
SCE1572_614	20.33	15.38
SCE1572_8179	0	0
SCE1572_4013	38.53	97.26
SCE1572_5812	72.99	50.89
SCE1572_10280	28.96	263.1
SCE1572_715	0	0
SCE1572_11567	0	0
SCE1572_10696	16	18.95
SCE1572_665	28.16	29.54
SCE1572_9855	61.7	27.92
SCE1572_5007	633.92	1507.1
SCE1572_8923	9.63	5.79
SCE1572_10476	40.58	96.32
SCE1572_3527	26.47	10309.6
SCE1572_9064	69.59	22.66
SCE1572_4851	35.87	20.85
SCE1572_2401	91.91	340.34
SCE1572_4721	86.57	231.52
SCE1572_2357	38.36	101.12
SCE1572_1792	16.48	38.08
SCE1572_9582	62.63	45.04
SCE1572_216	76.77	217.11
SCE1572_8325	53.93	122.6
SCE1572_10327	26.14	5.03
SCE1572_729	26.04	86.62
SCE1572_7325	137.8	770.71
SCE1572_1695	0	0
SCE1572_8070	20.14	9.69
SCE1572_11601	33.27	137.83
SCE1572_1074	2.81	0
SCE1572_4047	31.46	11.86
SCE1572_1132	22.74	5.61
SCE1572_6716	103.07	254.01
SCE1572_5856	24.97	4.29
SCE1572_10147	25.59	74.46
SCE1572_7563	66.08	31.23
SCE1572_4178	20.29	2.76
SCE1572_5563	57.98	63
SCE1572_4891	3.09	0
SCE1572_9364	89.8	86.08

SCE1572_2176	23.17	8.12
SCE1572_10572	27.12	41.85
SCE1572_10899	9.6	0
SCE1572_453	35.58	12.07
SCE1572_9131	7.67	8.2
SCE1572_6836	21.84	157.72
SCE1572_8084	0	0
SCE1572_8371	21	15.8
SCE1572_9682	130.97	342.2
SCE1572_11330	84.33	386.52
SCE1572_362	39.51	22.54
SCE1572_8077	49.57	9.77
SCE1572_7562	21.18	4.02
SCE1572_3860	24.95	48.5
SCE1572_3627	35.38	10.43
SCE1572_11429	30.64	21.89
SCE1572_5109	43.07	124.41
SCE1572_2521	17.03	5.52
SCE1572_720	6.32	3.8
SCE1572_537	62.13	142.96
SCE1572_10482	42.08	15.71
SCE1572_5104	61.64	179.05
SCE1572_2399	531.19	3524.18
SCE1572_8558	10.7	2.92
SCE1572_2365	160.17	353.95
SCE1572_8993	75.17	240.73
SCE1572_2029	52.45	23.96
SCE1572_5952	5.41	0
SCE1572_4471	43.28	32
SCE1572_6444	25.27	18.25
SCE1572_9455	23.42	69.72
SCE1572_2345	37.68	99.14
SCE1572_5407	0	0
SCE1572_1666	17.15	12.8
SCE1572_11402	7.49	4.51
SCE1572_4259	2.41	0
SCE1572_435	50.91	146.68
SCE1572_2653	47.43	28.54
SCE1572_1693	7.64	3.07
SCE1572_3720	26.52	23.08
SCE1572_6011	32.78	68.33
SCE1572_4148	64.98	440.75
SCE1572_4671	0	0
SCE1572_3585	40.82	14.04
SCE1572_5961	32.99	37.82
SCE1572_3460	47.41	52.75
SCE1572_11114	78.22	273.91
SCE1572_7919	48.9	12.39

SCE1572_11202	53.56	1031.35
SCE1572_896	0	0
SCE1572_2964	74.16	1983.27
SCE1572_2764	11.49	0
SCE1572_4222	74.57	47.86
SCE1572_3576	26.89	9.64
SCE1572_37	15.21	23.19
SCE1572_1537	13.6	30.74
SCE1572_11558	44.66	21.37
SCE1572_6178	38.97	218.27
SCE1572_2413	46.91	217.74
SCE1572_1116	0	0
SCE1572_10539	46.08	28.84
SCE1572_3760	75.93	3.58
SCE1572_2485	49.23	126.38
SCE1572_10459	17.18	70.55
SCE1572_9732	74.49	22.17
SCE1572_8586	34.83	10.16
SCE1572_7610	72.05	232.22
SCE1572_9779	0	0
SCE1572_8639	24.24	82.97
SCE1572_6026	38.32	80.2
SCE1572_6560	0	0
SCE1572_1735	66.33	40.47
SCE1572_3968	26.04	12.9
SCE1572_9537	79.35	242.14
SCE1572_5359	19.23	205.6
SCE1572_3019	23.44	12.66
SCE1572_1655	0	0
SCE1572_1304	54.84	109.29
SCE1572_11255	72.87	139.43
SCE1572_5968	4.16	1.43
SCE1572_9254	15	6.08
SCE1572_5151	1.02	0
SCE1572_5753	0	0
SCE1572_10425	43.32	52.13
SCE1572_5894	21.19	3.19
SCE1572_7458	18.83	14.5
SCE1572_2091	14.01	0
SCE1572_3820	44.5	29.05
SCE1572_6570	31.79	27.96
SCE1572_2387	21.23	13.93
SCE1572_7595	66.81	361.83
SCE1572_2968	24	52.96
SCE1572_5483	37.76	7.87
SCE1572_9919	28.54	12.88
SCE1572_5362	25.45	46.61
SCE1572_1977	861.59	20858.47

SCE1572_4327	17.72	7.9
SCE1572_16	52.23	107.92
SCE1572_10289	140.56	157.75
SCE1572_3564	29.34	10.17
SCE1572_11366	23.79	9.32
SCE1572_9382	0	0
SCE1572_4422	11	5.67
SCE1572_4204	22.11	391.53
SCE1572_3538	16.24	7.82
SCE1572_3470	30.13	12.48
SCE1572_11519	57.45	28.17
SCE1572_631	80.56	135.49
SCE1572_10172	53.19	57.39
SCE1572_11527	46	25.63
SCE1572_11537	70.97	241.8
SCE1572_8757	17.41	13.52
SCE1572_5631	27.57	17.69
SCE1572_7048	0	0
SCE1572_4523	13.96	0
SCE1572_66	32.89	54.06
SCE1572_2703	218.37	122.55
SCE1572_7743	134.12	643.08
SCE1572_5819	31.98	15.21
SCE1572_6375	53.1	10.15
SCE1572_3893	14.55	5.53
SCE1572_3210	11.46	30.82
SCE1572_3138	78.42	1029.33
SCE1572_330	37.41	33.46
SCE1572_751	65.3	68.65
SCE1572_10219	45.81	40.15
SCE1572_8481	34.21	28.93
SCE1572_356	56.22	12.3
SCE1572_11229	12.63	13.03
SCE1572_2643	73.81	35.32
SCE1572_17	18	57.02
SCE1572_6848	11.96	0.71
SCE1572_3734	52.66	6.81
SCE1572_5066	193.01	354.85
SCE1572_7398	40.64	187.14
SCE1572_3141	36.5	13.77
SCE1572_2822	22.64	20.27
SCE1572_5919	36.94	83.74
SCE1572_2089	18.25	9.76
SCE1572_8441	52.33	108.72
SCE1572_8661	14.27	6.25
SCE1572_673	135.25	1081.64
SCE1572_9051	72.78	29.19
SCE1572_3495	61.33	36.9

SCE1572_3981	25.58	23.73
SCE1572_2495	112.86	50.73
SCE1572_10667	13.54	7.24
SCE1572_6666	39.91	189.36
SCE1572_9541	14.36	5.9
SCE1572_1888	66.89	762.04
SCE1572_3287	35.67	0
SCE1572_10134	26.57	11.29
SCE1572_5162	0	0
SCE1572_779	34.56	37.69
SCE1572_2293	32.34	41.84
SCE1572_4525	27.66	12.8
SCE1572_5768	131.18	917.47
SCE1572_2007	19.7	27.67
SCE1572_9808	405.36	1193.6
SCE1572_5244	20.97	43.86
SCE1572_1387	54.64	31.42
SCE1572_5656	20.86	6.03
SCE1572_5038	243.09	2406.68
SCE1572_10939	186.45	244.1
SCE1572_9417	194.11	2963.59
SCE1572_6053	111.96	565.63
SCE1572_3807	47.07	70.73
SCE1572_2759	34.67	181.75
SCE1572_2980	117.04	1702.97
SCE1572_3682	63.17	3.04
SCE1572_7089	30.66	27.29
SCE1572_4662	14.41	3.79
SCE1572_10402	41.14	85.64
SCE1572_8138	34.85	15.25
SCE1572_4736	77.58	629.54
SCE1572_5610	30.06	1.61
SCE1572_7507	32.52	10.93
SCE1572_9121	28.12	7.96
SCE1572_2340	0	0
SCE1572_11093	41.3	24.25
SCE1572_5217	82.82	27.47
SCE1572_170	20.89	7.45
SCE1572_10739	28.83	12.85
SCE1572_7945	11.4	0
SCE1572_11097	26.96	24.43
SCE1572_9148	58.49	310.23
SCE1572_231	1.68	0
SCE1572_8875	20.66	0
SCE1572_8333	242.27	402.54
SCE1572_4558	26.44	68.18
SCE1572_176	21.37	4.9
SCE1572_765	17.63	101.84

SCE1572_2670	38.83	10.07
SCE1572_10384	13.46	1.71
SCE1572_1938	12.09	15.87
SCE1572_6061	676.97	1874.17
SCE1572_10704	34.93	145.2
SCE1572_8298	161.28	101.02
SCE1572_9182	24.92	12.76
SCE1572_719	37.79	42.79
SCE1572_3112	5.17	0
SCE1572_2876	32.19	44.15
SCE1572_4306	15.62	9.4
SCE1572_10103	23.33	50.88
SCE1572_3097	26.02	8.64
SCE1572_1868	21.04	102.45
SCE1572_9980	16.7	41.8
SCE1572_1516	84.59	239.31
SCE1572_11070	31.59	76.03
SCE1572_5848	36.83	23.16
SCE1572_9875	42.81	90.16
SCE1572_1648	28.7	9.28
SCE1572_10648	37.42	93.54
SCE1572_3944	149.72	387.73
SCE1572_2989	100.11	60.94
SCE1572_4338	22.43	7.6
SCE1572_6773	26.23	24.09
SCE1572_9107	15.78	48.52
SCE1572_904	29.8	30.2
SCE1572_9832	13.55	3.11
SCE1572_9412	34.49	121.06
SCE1572_8096	6.67	4.59
SCE1572_7757	14.84	8.93
SCE1572_2409	93.86	747.22
SCE1572_3952	18.51	12.04
SCE1572_9548	20.91	0
SCE1572_7755	39.05	49.76
SCE1572_7468	29.57	32.76
SCE1572_5758	14.64	22.81
SCE1572_3228	62.43	126.65
SCE1572_7118	133.61	67.11
SCE1572_2138	25.7	0
SCE1572_9739	0	0
SCE1572_3118	0	0
SCE1572_2942	39.99	38.1
SCE1572_4263	41.38	21.59
SCE1572_7288	63.86	116.93
SCE1572_7631	67.77	724.32
SCE1572_4066	43.75	117.01
SCE1572_6068	16.22	1.59

SCE1572_8889	27.71	22.23
SCE1572_7022	84.06	26.1
SCE1572_290	0	0
SCE1572_5195	60.38	46.68
SCE1572_6763	57.48	84.05
SCE1572_6647	31.92	4.8
SCE1572_1633	27.51	65.46
SCE1572_8167	0	0
SCE1572_1826	35.83	11.25
SCE1572_7986	61.37	36.2
SCE1572_1775	34.66	0
SCE1572_2903	0	0
SCE1572_5797	16.99	4.54
SCE1572_9637	92.03	1287.21
SCE1572_8057	0	0
SCE1572_7102	58.82	69.21
SCE1572_4330	70.66	38.97
SCE1572_7633	439.08	712.33
SCE1572_6112	54.86	37.21
SCE1572_2126	63.34	24.01
SCE1572_9690	129.96	281.06
SCE1572_808	29.21	10.86
SCE1572_7517	62.48	119.64
SCE1572_3111	24.74	8.87
SCE1572_1127	28.71	12.96
SCE1572_2716	38.84	52.22
SCE1572_4858	42.82	25.34
SCE1572_9073	56.7	38.66
SCE1572_9623	28.45	14.61
SCE1572_2673	31.88	167.63
SCE1572_1511	89.93	670.81
SCE1572_3416	25.92	20.8
SCE1572_9974	45.88	36.81
SCE1572_9957	18.13	3.97
SCE1572_7728	0	0
SCE1572_1207	17.45	11.13
SCE1572_2249	34.82	25.61
SCE1572_6632	45.3	182.91
SCE1572_2528	1.64	1.97
SCE1572_9765	26.01	31.31
SCE1572_9042	43.2	186.39
SCE1572_9091	28.51	9.36
SCE1572_8160	13.83	4.44
SCE1572_9750	16.55	23.9
SCE1572_11001	17.63	4.24
SCE1572_4444	33.45	85.4
SCE1572_10104	38.81	105.1
SCE1572_2992	57.76	52.13

SCE1572_1617	11.45	10.86
SCE1572_9755	22.02	10.19
SCE1572_9524	30.67	34.82
SCE1572_11464	179.9	60.91
SCE1572_3879	24.56	68.21
SCE1572_984	42.36	106.26
SCE1572_6119	201.39	147.44
SCE1572_9598	107.5	1940.22
SCE1572_1235	76.02	23.38
SCE1572_3324	40.5	155.86
SCE1572_4753	89.91	258.84
SCE1572_1287	33.54	452.37
SCE1572_7531	33.9	172.08
SCE1572_9811	0	0
SCE1572_776	9.63	0
SCE1572_11588	15.62	15.72
SCE1572_7042	25.94	11.1
SCE1572_7371	21.56	8.37
SCE1572_3353	0	0
SCE1572_11476	10.55	41.25
SCE1572_7684	0	0
SCE1572_9445	30.81	20
SCE1572_11354	10.33	6.54
SCE1572_3620	18.41	5.21
SCE1572_4123	89.31	29.99
SCE1572_2798	49.17	11.84
SCE1572_8731	19.13	6.82
SCE1572_9814	40.91	217.22
SCE1572_6403	82.58	61.91
SCE1572_866	43.32	26.07
SCE1572_3005	6.89	0
SCE1572_4052	44.74	305.1
SCE1572_10295	31.46	148.28
SCE1572_4390	52.57	26.59
SCE1572_10568	82.63	205.02
SCE1572_4196	41.8	54.3
SCE1572_696	8.31	0
SCE1572_1240	61.32	18.11
SCE1572_3606	24.09	3.41
SCE1572_7029	39.37	25.18
SCE1572_1603	0	0
SCE1572_8336	86.21	40.16
SCE1572_6342	26.66	43.31
SCE1572_8880	29.06	9.12
SCE1572_10839	3.94	0
SCE1572_5676	11.45	4.75
SCE1572_1292	26.21	12.76
SCE1572_4833	88.22	942.18

SCE1572_560	215.71	3594.96
SCE1572_5050	12.9	0
SCE1572_5619	18.46	28.28
SCE1572_2779	85.91	302.53
SCE1572_5375	27.98	16.29
SCE1572_4868	19.14	12.65
SCE1572_2418	20.3	67.78
SCE1572_8025	0	0
SCE1572_1534	28	14.51
SCE1572_4313	0	0
SCE1572_1010	45.94	20.73
SCE1572_8961	10.39	5.89
SCE1572_7238	42.77	179.18
SCE1572_5057	113.5	484.5
SCE1572_2186	2.41	0
SCE1572_1788	4.59	3.69
SCE1572_8356	15.31	5.26
SCE1572_3081	9.53	45.85
SCE1572_6181	51.69	59.71
SCE1572_2847	45.97	49.54
SCE1572_2946	23.38	11.58
SCE1572_3200	101.27	488.86
SCE1572_2435	24.23	45.62
SCE1572_3996	3.35	2.02
SCE1572_6607	0	0
SCE1572_5932	18.29	18.79
SCE1572_204	56.86	34.21
SCE1572_5616	0	0
SCE1572_800	0	0
SCE1572_3469	33.57	12.57
SCE1572_320	38.03	12.2
SCE1572_3049	7.44	2.11
SCE1572_1700	90.69	145.53
SCE1572_1268	76.31	71.52
SCE1572_8794	25.03	11.37
SCE1572_109	37.28	41.88
SCE1572_5641	7.31	0
SCE1572_301	16.39	15.78
SCE1572_1640	26.33	15.37
SCE1572_4316	56.13	14.83
SCE1572_7962	62.78	617.87
SCE1572_2837	70.48	61.81
SCE1572_11316	357.31	715.02
SCE1572_6217	7.31	13.19
SCE1572_4576	2.38	0
SCE1572_8231	44.57	32.49
SCE1572_10198	54.88	21.79
SCE1572_5695	49.33	13.97

SCE1572_7425	7.87	130.82
SCE1572_10922	300.57	32336.89
SCE1572_2715	45.39	104.13
SCE1572_2110	37.06	386.93
SCE1572_922	10.23	15.67
SCE1572_9483	89.1	230.67
SCE1572_10561	0	0
SCE1572_2741	25.27	13.03
SCE1572_11131	18.27	7.69
SCE1572_11200	41.9	151.29
SCE1572_7858	55.9	63.28
SCE1572_6554	19.88	0
SCE1572_9202	17.76	22.19
SCE1572_7265	14.63	12.14
SCE1572_6975	34.71	13.19
SCE1572_2840	40.17	29.28
SCE1572_6122	60.75	172.52
SCE1572_6045	256.73	1042.1
SCE1572_2680	33.69	9.54
SCE1572_3792	4.89	0
SCE1572_6580	18.8	3.02
SCE1572_326	33.55	35.78
SCE1572_8570	5.05	0
SCE1572_5126	25.14	43.49
SCE1572_3898	30.69	94.35
SCE1572_5950	73.19	17.62
SCE1572_5986	9.83	0
SCE1572_3180	27.59	60.61
SCE1572_4539	26.62	25.29
SCE1572_978	37.35	54.21
SCE1572_7934	49.47	3.84
SCE1572_8050	48.32	9.36
SCE1572_10887	46.34	68.85
SCE1572_11237	21.39	6.43
SCE1572_9395	27.76	25.7
SCE1572_3889	12.15	6.36
SCE1572_1553	21.42	13.22
SCE1572_3825	24.72	11.9
SCE1572_3713	134.71	237.77
SCE1572_8773	72.27	78.47
SCE1572_8048	33.44	10.94
SCE1572_1054	62.08	76.86
SCE1572_10359	28.36	28.88
SCE1572_3788	53.3	98.36
SCE1572_9986	14.83	10.71
SCE1572_3311	24.68	10.61
SCE1572_9663	32.12	213.72
SCE1572_3781	77.09	675.42

SCE1572_7304	59.81	15.62
SCE1572_3678	19.87	47.82
SCE1572_3056	7.8	2.61
SCE1572_2220	32.96	92.93
SCE1572_9784	29.4	4.42
SCE1572_10521	23.02	23.35
SCE1572_9355	95.7	292.46
SCE1572_10220	39.27	47.63
SCE1572_3594	12.72	2.45
SCE1572_1437	39.02	16.1
SCE1572_10253	0	0
SCE1572_8630	74.86	428.78
SCE1572_1997	17.08	5.14
SCE1572_5261	68.69	786.3
SCE1572_155	52.06	102.24
SCE1572_5242	33.1	7.71
SCE1572_5762	10.7	4.48
SCE1572_7393	22.99	2.13
SCE1572_2036	225.44	2511.52
SCE1572_10370	26.03	9.31
SCE1572_11088	35.82	52.13
SCE1572_5116	29.45	32.54
SCE1572_6130	17.46	115.01
SCE1572_9021	7.35	7.74
SCE1572_2042	63.05	248.1
SCE1572_4303	60.65	10.68
SCE1572_11249	15.65	69.45
SCE1572_3273	26.39	137.64
SCE1572_3063	49.29	95.74
SCE1572_6938	34.58	10.24
SCE1572_11267	403.05	2452.46
SCE1572_1953	46.93	65.16
SCE1572_1329	37.63	122.53
SCE1572_6538	43.57	25.17
SCE1572_5927	124.86	36.06
SCE1572_6152	59.22	53.58
SCE1572_4996	8.92	10.73
SCE1572_9677	29.67	14.51
SCE1572_4614	61.88	25.53
SCE1572_11339	19.18	12.98
SCE1572_3394	92.11	162.84
SCE1572_4393	16.05	9.04
SCE1572_2239	61.86	54.01
SCE1572_10182	30.32	86.02
SCE1572_9643	62.2	82.66
SCE1572_1599	0	0
SCE1572_4910	3.09	0
SCE1572_8745	6.23	0

SCE1572_508	31.27	6.39
SCE1572_6279	47.7	1976.32
SCE1572_1726	38.5	22.93
SCE1572_11384	197.35	400.96
SCE1572_9910	27.99	17.97
SCE1572_1954	22.01	25.51
SCE1572_9298	86.45	177.03
SCE1572_3930	230.69	957.38
SCE1572_6035	22.91	36.37
SCE1572_3726	80.14	1866.32
SCE1572_3865	28.88	10.43
SCE1572_5781	73.77	256.85
SCE1572_8522	88.38	170.78
SCE1572_299	44.62	30.82
SCE1572_9760	25.94	56.69
SCE1572_10028	12.98	2.23
SCE1572_11375	0	0
SCE1572_7991	51.39	203.28
SCE1572_7335	34	108.37
SCE1572_8971	73.09	203.98
SCE1572_4479	10.78	19.25
SCE1572_4887	57.76	20.85
SCE1572_8579	0	0
SCE1572_6746	40.17	65.5
SCE1572_4639	18.73	2.91
SCE1572_5608	53.9	22
SCE1572_9023	548.85	1260.84
SCE1572_2229	33.6	680.08
SCE1572_11481	31.57	14.98
SCE1572_4245	26.27	92.06
SCE1572_1475	0	0
SCE1572_1925	0	0
SCE1572_1500	24.39	21.87
SCE1572_4597	0	0
SCE1572_3556	12.85	0
SCE1572_4880	122.83	320.58
SCE1572_3571	24.36	22.37
SCE1572_6843	22.66	20.78
SCE1572_1781	11.16	12.2
SCE1572_8287	36.29	112.58
SCE1572_593	51.37	92.39
SCE1572_399	28.64	16.22
SCE1572_6254	35.19	42.35
SCE1572_318	25.04	3.35
SCE1572_11596	48.58	528.23
SCE1572_9725	5.27	17.45
SCE1572_2274	43.18	9.24
SCE1572_1520	45.48	16.42

SCE1572_4355	28.93	28.81
SCE1572_3167	23.49	34.27
SCE1572_3872	63.44	157.49
SCE1572_6333	20.25	72.55
SCE1572_4366	36.27	32.3
SCE1572_6243	26.86	7.36
SCE1572_2064	32.93	29.49
SCE1572_8264	44.74	144.77
SCE1572_5424	28.13	28.9
SCE1572_678	31.59	45.62
SCE1572_338	36.86	23.8
SCE1572_9602	40.18	12.96
SCE1572_9187	27.09	47.65
SCE1572_7178	74.51	48.48
SCE1572_2320	55.47	748.92
SCE1572_1969	41.12	26.8
SCE1572_9098	37.9	12
SCE1572_7006	0	0
SCE1572_5501	18.37	14.34
SCE1572_1317	34.58	63.93
SCE1572_5061	57.89	265.4
SCE1572_7016	0	0
SCE1572_1610	78.97	60.94
SCE1572_4879	60.04	105.86
SCE1572_2661	45.48	60.82
SCE1572_6305	24.65	13.35
SCE1572_5669	24.24	8.01
SCE1572_8989	97.78	241.3
SCE1572_5992	39.17	62.85
SCE1572_6142	23.35	16.93
SCE1572_3298	12.58	10.09
SCE1572_5549	31.94	13.13
SCE1572_3189	9.58	0
SCE1572_10965	50.31	26.82
SCE1572_11106	41.71	53.14
SCE1572_6132	0	0
SCE1572_8531	24.52	30.86
SCE1572_143	12.63	30.41
SCE1572_349	21.54	74.78
SCE1572_5839	39.13	14.66
SCE1572_9685	57.4	123.4
SCE1572_3508	26.46	17.14
SCE1572_238	30.32	4.87
SCE1572_1065	71.79	18.13
SCE1572_5296	40.13	17.89
SCE1572_10153	20.18	95.74
SCE1572_8978	30.08	28
SCE1572_9593	46.86	843.54

SCE1572_8866	48.37	28.13
SCE1572_2728	14.53	11.65
SCE1572_2284	10.42	9.12
SCE1572_2149	43.15	70.68
SCE1572_1845	6.74	4.05
SCE1572_6476	79.32	902.17
SCE1572_5329	67.56	19.48
SCE1572_7242	52.66	68.49
SCE1572_10930	50.54	40.21
SCE1572_6251	42.72	18.94
SCE1572_5940	25.16	10.5
SCE1572_4516	18.59	14.13
SCE1572_6160	16.11	17.63
SCE1572_7574	57.33	316.81
SCE1572_11391	54.44	36.74
SCE1572_7466	77.74	439.3
SCE1572_6236	47.74	17.68
SCE1572_8695	14.74	21.6
SCE1572_4494	34.43	24.72
SCE1572_266	40.96	13.84
SCE1572_9393	20.52	12.35
SCE1572_7545	63.54	48.3
SCE1572_3173	38.13	22.12
SCE1572_8687	24.3	13.1
SCE1572_1101	30.72	11.25
SCE1572_11137	44.41	311.47
SCE1572_8598	0	0
SCE1572_11017	66.39	178.88
SCE1572_9996	28.84	21.99
SCE1572_2695	20.06	110.06
SCE1572_4769	22.74	100.35
SCE1572_8897	43.73	85.75
SCE1572_1747	68.34	56.61
SCE1572_7829	26.25	104.45
SCE1572_8313	14.85	10.43
SCE1572_7360	30.83	56.42
SCE1572_7482	0	0
SCE1572_8101	60.47	547.91
SCE1572_2319	36.97	246.29
SCE1572_10031	68.71	62.87
SCE1572_1003	60.23	26.99
SCE1572_10125	0.87	0
SCE1572_2422	23.46	13.03
SCE1572_5785	46.03	128.16
SCE1572_1601	5.41	0
SCE1572_3718	0	0
SCE1572_623	176.46	1527.49
SCE1572_352	24.29	33.66

SCE1572_8217	68.46	21.97
SCE1572_5546	37.47	12.39
SCE1572_1031	52.93	289.35
SCE1572_2394	12.49	12.19
SCE1572_8148	40.72	21
SCE1572_5001	115.82	587.93
SCE1572_3783	15.55	9.36
SCE1572_5413	3.33	0
SCE1572_4787	240.94	1721.36
SCE1572_6991	41.77	10.24
SCE1572_10347	40.47	116.61
SCE1572_9573	72.28	10.61
SCE1572_10997	53.69	234.46
SCE1572_7381	26.95	18.92
SCE1572_285	83.47	22.12
SCE1572_2339	25.36	147.87
SCE1572_7412	29.99	105.72
SCE1572_1175	25.87	145.28
SCE1572_4658	40.28	82.14
SCE1572_6483	36.78	12.96
SCE1572_245	18.42	4.95
SCE1572_3205	43.95	120.33
SCE1572_11513	55.07	112.41
SCE1572_7430	58.11	21.3
SCE1572_8591	77.46	20.94
SCE1572_5517	39.25	16.82
SCE1572_4946	2.92	0
SCE1572_9219	23.89	4.55
SCE1572_6006	13.42	49.42
SCE1572_2173	9.65	6.63
SCE1572_226	28.61	13.77
SCE1572_467	33.57	71.23
SCE1572_342	141.65	853.2
SCE1572_1608	17.96	12.04
SCE1572_2464	66.81	18.33
SCE1572_10585	186.47	71.31
SCE1572_9824	16.42	5.61
SCE1572_9440	22.08	28.13
SCE1572_6875	239.22	97.31
SCE1572_7977	0	0
SCE1572_10775	43.67	20.94
SCE1572_5478	31.43	26.54
SCE1572_6858	40.41	23.15
SCE1572_1307	696.27	10545.56
SCE1572_3406	54.46	23.62
SCE1572_2538	12.18	18.84
SCE1572_3466	20.32	27.59
SCE1572_9701	37.28	0

SCE1572_3213	39.16	63.44
SCE1572_4623	31.64	193.95
SCE1572_5422	11.23	0
SCE1572_2515	16.13	23.29
SCE1572_3650	18.15	11.27
SCE1572_3690	2.89	0
SCE1572_85	41.12	89.55
SCE1572_6356	32.92	17.72
SCE1572_836	30.73	32.11
SCE1572_4824	41.4	82.11
SCE1572_2313	21.72	36.11
SCE1572_7899	42.65	65.77
SCE1572_11494	13.12	4.28
SCE1572_9954	46.39	28.53
SCE1572_9671	43.41	358.68
SCE1572_8104	34.29	24.98
SCE1572_2590	18.61	21.14
SCE1572_9267	16.62	0
SCE1572_574	37.36	139.45
SCE1572_872	32.77	30.18
SCE1572_1021	57.35	16.81
SCE1572_383	35.33	39.51
SCE1572_2890	43.19	16.22
SCE1572_4624	121.72	408.61
SCE1572_1574	41.83	17.62
SCE1572_11431	44.11	27.49
SCE1572_7004	5.79	1.44
SCE1572_10982	37.29	32.37
SCE1572_842	10.19	6.13
SCE1572_7659	25.97	16.42
SCE1572_918	28.17	31.81
SCE1572_7490	60.41	62.72
SCE1572_3152	78.82	68.42
SCE1572_6521	29.86	0
SCE1572_8248	8.81	10.6
SCE1572_9512	70.1	277.34
SCE1572_7708	37.62	13.72
SCE1572_4646	23.87	15.96
SCE1572_9502	24.2	48.83
SCE1572_2354	55.72	728.29
SCE1572_3617	33.69	9.67
SCE1572_1478	37.9	15.21
SCE1572_2097	18.64	565.33
SCE1572_4357	47.81	20.55
SCE1572_7589	49.6	419.19
SCE1572_4489	34.51	50.44
SCE1572_10166	0	0
SCE1572_3072	25.42	14.77

SCE1572_2211	75.05	123.27
SCE1572_10312	85.46	60.82
SCE1572_6915	29.98	11.61
SCE1572_7627	25.1	23.17
SCE1572_3870	99.08	76.83
SCE1572_8542	82.26	533.8
SCE1572_3488	16.75	11.98
SCE1572_7889	35.31	1837.08
SCE1572_10971	2.14	10.28
SCE1572_9270	0	0
SCE1572_2312	164.52	2703.92
SCE1572_7524	15.42	58.76
SCE1572_2187	25.86	14.01
SCE1572_10331	58.7	27.05
SCE1572_5779	20.75	16.32
SCE1572_6696	21.97	7.74
SCE1572_3696	117.92	229.77
SCE1572_9905	0	0
SCE1572_56	70.17	6.03
SCE1572_7283	17.4	9.31
SCE1572_5638	24.77	19.87
SCE1572_11305	6.79	0
SCE1572_10540	73.04	25.45
SCE1572_3378	62.32	24.63
SCE1572_3498	30.32	51.34
SCE1572_10782	33.44	12.13
SCE1572_2853	48.21	26.57
SCE1572_1621	129.64	1012.79
SCE1572_8001	151.82	53.52
SCE1572_9015	0	0
SCE1572_3790	15.8	5.34
SCE1572_9939	31.25	9.6
SCE1572_7081	19.27	80.02
SCE1572_6933	12.25	44.23
SCE1572_1675	33.57	78.1
SCE1572_10338	54.19	270.06
SCE1572_7988	30.1	5.37
SCE1572_3349	32.49	22.81
SCE1572_7622	74.7	160.08
SCE1572_5172	42.88	75.56
SCE1572_6964	36.37	35.01
SCE1572_10417	1.8	0
SCE1572_3417	56.52	328.11
SCE1572_7071	22.97	24.33
SCE1572_11328	119.25	196.81
SCE1572_5976	39.98	24.64
SCE1572_5826	21.96	16.78
SCE1572_5571	6.45	2.96

SCE1572_9763	22.33	32.53
SCE1572_2541	0	0
SCE1572_4693	71.01	138.06
SCE1572_5300	43.32	17.66
SCE1572_6504	51.71	60.82
SCE1572_5497	56.63	113.88
SCE1572_9374	49.65	18.14
SCE1572_9287	205.05	886.35
SCE1572_4949	16.91	10.18
SCE1572_10018	45.61	31.51
SCE1572_4760	45.54	21.64
SCE1572_1589	0	0
SCE1572_4434	58.05	39
SCE1572_5149	25.66	12.98
SCE1572_8012	2.37	0
SCE1572_8027	12.21	14.69
SCE1572_8094	41.56	12.68
SCE1572_4409	16.67	12.94
SCE1572_6018	625.42	405.98
SCE1572_1910	55.42	22.65
SCE1572_1373	41.21	177.46
SCE1572_3481	15.68	6.29
SCE1572_11364	51.37	29.09
SCE1572_5270	39.9	8023.49
SCE1572_1244	0	0
SCE1572_879	100.56	548.01
SCE1572_3809	26.08	51.09
SCE1572_950	7.12	25.12
SCE1572_10243	23.63	0
SCE1572_8761	21.66	30.95
SCE1572_5033	122.14	2489.29
SCE1572_5946	21.74	11.16
SCE1572_667	6.68	7.78
SCE1572_2180	48.06	20.42
SCE1572_6751	83.19	84.7
SCE1572_3767	13.32	48.1
SCE1572_8201	111.84	35.38
SCE1572_74	28.98	55.14
SCE1572_1522	32.28	30.61
SCE1572_6794	17.03	26.44
SCE1572_8565	26.13	53.03
SCE1572_6983	26.54	13.17
SCE1572_4299	68.07	28.31
SCE1572_2936	36.98	13.35
SCE1572_9927	64.6	140.7
SCE1572_7835	39.73	183.44
SCE1572_9530	35.79	35.5
SCE1572_2052	0	0

SCE1572_2437	6.76	9.03
SCE1572_6831	60.21	97.61
SCE1572_7181	31.43	27.6
SCE1572_2581	26.76	55.81
SCE1572_5685	47.8	238.15
SCE1572_7291	9.72	0
SCE1572_9631	69.24	42.19
SCE1572_3124	18.52	5.57
SCE1572_11050	40.29	71.98
SCE1572_10617	118.59	43.36
SCE1572_6440	40.49	14.51
SCE1572_6419	39.5	41.91
SCE1572_2874	29.77	16.86
SCE1572_8674	20.57	86.19
SCE1572_11543	84.79	36.19
SCE1572_124	50.14	5.75
SCE1572_463	53.29	1958.15
SCE1572_2269	76.86	649.12
SCE1572_2014	39.38	7.97
SCE1572_11362	26.49	6.94
SCE1572_8089	18.34	8.61
SCE1572_10875	61.27	32.49
SCE1572_2905	25.5	239.93
SCE1572_1343	13.86	9.81
SCE1572_1942	0	0
SCE1572_3306	20.91	296.13
SCE1572_1228	18.55	32.79
SCE1572_1940	96.51	25.11
SCE1572_1682	247.37	2473.79
SCE1572_698	22.46	105.42
SCE1572_3802	41.57	35.32
SCE1572_9575	1.08	0
SCE1572_6582	15.29	46
SCE1572_1327	0	0
SCE1572_11011	0	0
SCE1572_4689	61.2	336.7
SCE1572_1876	66.12	95.49
SCE1572_4210	5.51	0
SCE1572_5576	49.31	20.77
SCE1572_2071	6.93	7.41
SCE1572_8403	66.91	47.28
SCE1572_4074	125.89	997.37
SCE1572_8413	23.37	7.21
SCE1572_971	54.6	42.11
SCE1572_4779	0	0
SCE1572_5177	47.74	26.32
SCE1572_11035	17.64	185.61
SCE1572_2898	20.37	5.45

SCE1572_6077	3.89	18.71
SCE1572_6599	33.05	59.66
SCE1572_8208	60.21	47.6
SCE1572_10807	43.41	34.31
SCE1572_9801	61.25	24.17
SCE1572_10016	9.43	90.76
SCE1572_8705	0	0
SCE1572_6104	39.82	9.22
SCE1572_149	38.81	122.74
SCE1572_4348	43.85	12.72
SCE1572_4459	45.54	18.71
SCE1572_6653	23.94	0
SCE1572_22	39.34	76.22
SCE1572_3838	0	0
SCE1572_859	265.77	7725.62
SCE1572_4472	18.28	3.88
SCE1572_883	0	0
SCE1572_8703	21.66	52.13
SCE1572_852	84.98	309.89
SCE1572_10841	22.64	5.84
SCE1572_4651	46.16	63.2
SCE1572_10180	38.03	122.47
SCE1572_6267	21.66	22.34
SCE1572_9253	12.26	3.97
SCE1572_10558	49.02	19.21
SCE1572_9033	20.72	12.16
SCE1572_6099	39.66	57.79
SCE1572_270	35.84	28.2
SCE1572_4144	51.53	234.93
SCE1572_10722	115.71	38.41
SCE1572_4387	23.38	23.87
SCE1572_7440	22.03	15.59
SCE1572_4606	21.06	162.19
SCE1572_7298	60.1	13.95
SCE1572_2146	39.72	11.23
SCE1572_5409	2.23	0
SCE1572_10003	10.83	10.43
SCE1572_11163	31	211.03
SCE1572_8801	0	0
SCE1572_5724	0	0
SCE1572_8768	21.09	15.87
SCE1572_8717	18.38	5.53
SCE1572_5373	30.61	478
SCE1572_7416	79.38	415.37
SCE1572_6597	96.06	61.28
SCE1572_9793	24.46	18.59
SCE1572_8644	29.45	21.41
SCE1572_3691	118.05	24.59

SCE1572_7388	20.01	12.97
SCE1572_5004	20.49	66.57
SCE1572_5238	74.3	79.24
SCE1572_7254	10.87	0.67
SCE1572_3476	42.1	115.24
SCE1572_4108	64.72	32.99
SCE1572_10084	43.9	99.52
SCE1572_10610	16.62	5
SCE1572_2612	16.63	10.3
SCE1572_4900	28.55	9.56
SCE1572_6579	32.54	24.99
SCE1572_8909	30.94	14.89
SCE1572_4133	7.24	5.45
SCE1572_8468	25.43	55.38
SCE1572_11178	0	0
SCE1572_8008	92.65	1943.36
SCE1572_11440	44.22	30.41
SCE1572_10681	24.26	26.27
SCE1572_10109	40.37	125.59
SCE1572_4743	30.93	32.11
SCE1572_659	68.23	0
SCE1572_11288	45.6	374.65
SCE1572_8408	38.9	35.11
SCE1572_11317	110.01	143.42
SCE1572_10412	10.51	7.23
SCE1572_9435	175.54	856.5
SCE1572_458	31.4	17.51
SCE1572_8424	58.1	85.26
SCE1572_1975	37.18	136.26
SCE1572_3518	12	3.94
SCE1572_1964	23.82	6.99
SCE1572_402	47.75	14.11
SCE1572_505	7.89	2.71
SCE1572_1064	45.48	17.58
SCE1572_5014	62.31	367.28
SCE1572_2931	0	0
SCE1572_6421	27.28	4.31
SCE1572_11152	21.66	14.48
SCE1572_7357	15.39	19.09
SCE1572_7060	71.19	25.39
SCE1572_4051	158.14	829.9
SCE1572_6721	79.05	377.4
SCE1572_5139	40.27	19.96
SCE1572_9366	9.15	1.38
SCE1572_8998	99.25	597.7
SCE1572_3050	21.34	2.7
SCE1572_9162	177.1	6.13
SCE1572_4088	47.18	56.59

SCE1572_3638	59.73	24.88
SCE1572_112	10.11	0
SCE1572_944	82.87	26.54
SCE1572_8781	42.68	903.01
SCE1572_8255	56.86	80.17
SCE1572_10114	21.13	2.76
SCE1572_378	36.5	36.04
SCE1572_6474	30.52	92.42
SCE1572_1151	13.78	47.39
SCE1572_3373	45.16	19.41
SCE1572_707	67.17	518.51
SCE1572_10043	53.42	1212.94
SCE1572_5806	44.14	120.1
SCE1572_8629	25.75	94.67
SCE1572_789	0	0
SCE1572_7654	19.14	12.9
SCE1572_11227	52.62	47.23
SCE1572_9610	25.43	13.6
SCE1572_3759	25.87	29.07
SCE1572_8431	48.37	22.59
SCE1572_6394	636.02	241.22
SCE1572_4813	34.42	241.52
SCE1572_956	0	0
SCE1572_9964	51.72	18.99
SCE1572_3095	40.97	75.56
SCE1572_1045	34.41	42.7
SCE1572_9929	23.49	0
SCE1572_4715	41.19	107.84
SCE1572_4466	30.51	15.3
SCE1572_5790	22.3	60.1
SCE1572_10673	12.67	22.45
SCE1572_5555	44.12	51.6
SCE1572_4416	43.62	13.22
SCE1572_1095	34.92	234.43
SCE1572_1561	24.78	12.87
SCE1572_11536	29.69	59.56
SCE1572_931	320.25	6326.56
SCE1572_10824	143.36	273.07
SCE1572_10989	14.39	32.94
SCE1572_10632	0	0
SCE1572_8849	25.91	16.36
SCE1572_1369	51.29	97.46
SCE1572_8873	42.46	10.98
SCE1572_7841	26.46	14.69
SCE1572_6954	25.18	10.32
SCE1572_6734	32.45	78.11
SCE1572_277	48.05	28.02
SCE1572_6792	26.28	158.96

SCE1572_1930	72.5	131.33
SCE1572_6904	61.19	10.99
SCE1572_8436	83.03	61.39
SCE1572_5740	23.75	43.97
SCE1572_2501	21.41	55.67
SCE1572_6364	26.72	321.55
SCE1572_6853	46.81	47.9
SCE1572_6998	48.38	35.5
SCE1572_5197	8.83	0
SCE1572_2307	18.76	13.55
SCE1572_442	40.82	39.77
SCE1572_5755	60.84	197.51
SCE1572_1506	46.07	15.07
SCE1572_5989	0	4.65
SCE1572_5735	33.8	70.89
SCE1572_748	8.1	92.12
SCE1572_2480	15.16	5.21
SCE1572_4219	38.23	36.6
SCE1572_84	14.67	87.11
SCE1572_1353	38.42	24.66
SCE1572_9971	54.55	403.17
SCE1572_3015	44.66	15.92
SCE1572_1688	11.08	16.84
SCE1572_5883	42.07	53.5
SCE1572_46	82.91	167.58
SCE1572_8942	47.2	36.72
SCE1572_6298	25.01	53.51
SCE1572_10480	27.44	86.89
SCE1572_358	41.99	29.76
SCE1572_9328	54.04	70.06
SCE1572_11506	33.47	34.12
SCE1572_1422	18.11	17.56
SCE1572_7771	31.89	148.12
SCE1572_10041	27.8	0
SCE1572_3400	36.15	209.88
SCE1572_4564	32.49	29.79
SCE1572_7216	58.81	20.78
SCE1572_8620	32.21	168.28
SCE1572_7538	75.62	40.35
SCE1572_3101	10.6	63.8
SCE1572_7310	23.01	18.79
SCE1572_1841	127.3	929.52
SCE1572_8951	0	0
SCE1572_1109	0	0
SCE1572_4233	11.4	6.04
SCE1572_5651	0	0
SCE1572_6824	141.07	341.12
SCE1572_4772	26.8	6.53

SCE1572_10701	0	0
SCE1572_2548	25.46	9.29
SCE1572_8226	13.66	18.79
SCE1572_6678	173.63	680.63
SCE1572_9976	0	0
SCE1572_10309	72.58	2.59
SCE1572_10850	35.4	15.04
SCE1572_2619	18.11	8.96
SCE1572_10448	23.53	39.85
SCE1572_9314	64.15	76.61
SCE1572_10513	0	0
SCE1572_3255	13	88.62
SCE1572_447	29.98	23.15
SCE1572_5309	26.59	35.4
SCE1572_4922	60.65	12.16
SCE1572_10111	63.78	462.44
SCE1572_10948	45.01	45.28
SCE1572_10603	0	0
SCE1572_9947	10.95	12.16
SCE1572_8828	14.77	6.89
SCE1572_4063	49.51	75.54
SCE1572_7816	29.81	198.86
SCE1572_11439	80.5	39.33
SCE1572_5087	63.28	521.75
SCE1572_10390	50.14	24.99
SCE1572_4984	63.45	21.82
SCE1572_962	270.34	133.7
SCE1572_6731	0	0
SCE1572_6274	112.52	651.81
SCE1572_9403	78.4	45.72
SCE1572_811	14.52	7.76
SCE1572_2950	3.03	0
SCE1572_7992	58.01	5.78
SCE1572_4011	87.27	246.3
SCE1572_6865	40.12	30.23
SCE1572_184	17.84	0
SCE1572_3282	13.3	6.4
SCE1572_902	58.07	15.53
SCE1572_4283	13	20.85
SCE1572_6898	50.44	126.98
SCE1572_3025	37.28	13.16
SCE1572_6173	32.72	145.97
SCE1572_10123	182.83	168.66
SCE1572_4703	36.39	10.7
SCE1572_8151	37.46	15.03
SCE1572_5490	73.74	31.91
SCE1572_8063	8.75	0
SCE1572_2627	60.01	23.57

SCE1572_7197	49.63	26.13
SCE1572_1417	133.83	65.33
SCE1572_1725	33.98	68.69
SCE1572_1628	53.67	94.73
SCE1572_1088	34.99	5.61
SCE1572_8112	40.87	12.58
SCE1572_4165	45.91	13.08
SCE1572_8032	0	0
SCE1572_4701	62.29	46.95
SCE1572_9146	0	0
SCE1572_7488	45.24	79.46
SCE1572_11458	73.33	126.41
SCE1572_8606	1.99	0
SCE1572_6672	0	0
SCE1572_2497	51.5	36.41
SCE1572_1834	9.87	8.64
SCE1572_5445	36.26	17.45
SCE1572_6338	29.13	80.89
SCE1572_4951	13.46	6.82
SCE1572_1853	62.95	19.4
SCE1572_8353	336.53	1797.9
SCE1572_3978	35.29	26.32
SCE1572_6754	109.25	30.72
SCE1572_1385	24.36	20.22
SCE1572_8476	118.2	98.43
SCE1572_10644	87.78	1943.24
SCE1572_9231	29.48	4.05
SCE1572_8190	39.93	13.57
SCE1572_2845	42.42	0
SCE1572_11599	11.54	4.51
SCE1572_10978	349.64	1176.69
SCE1572_1715	37.35	44.95
SCE1572_8492	134.28	252.83
SCE1572_10207	24.02	28.9
SCE1572_7058	38.55	12.37
SCE1572_10278	10.61	1.82
SCE1572_3458	91.54	20.66
SCE1572_9348	56.93	51.73
SCE1572_4112	37.04	11.14
SCE1572_8726	48.4	22.27
SCE1572_4380	18.06	6.72
SCE1572_1848	69.72	34.37
SCE1572_2087	0	0
SCE1572_3332	0	0
SCE1572_649	0	0
SCE1572_7681	20.42	15.13
SCE1572_10768	6.79	0
SCE1572_3366	21.43	373.95

SCE1572_642	68.64	146.85
SCE1572_495	0	0
SCE1572_8682	19.84	51.16
SCE1572_10830	14.26	7.63
SCE1572_7033	49.34	17.38
SCE1572_9004	19.01	5.58
SCE1572_6210	47.36	26.45
SCE1572_1318	11.72	3.53
SCE1572_10861	373.22	1467.88
SCE1572_5279	86.3	66.97
SCE1572_1757	47.32	24.64
SCE1572_11216	29.87	27.23
SCE1572_1196	24.73	37.53
SCE1572_10504	39.69	46.99
SCE1572_5466	45.41	21.02
SCE1572_3924	33.69	243.28
SCE1572_8855	103.99	252.64
SCE1572_2551	23.11	13.35
SCE1572_5458	27.97	7.81
SCE1572_6620	31.88	20.59
SCE1572_8188	0	0
SCE1572_7975	15.94	2.07
SCE1572_7279	3.16	0
SCE1572_4931	62.08	39.32
SCE1572_1311	219.41	2356.21
SCE1572_11144	28.74	20.75
SCE1572_5963	34.14	17.72
SCE1572_6383	32.75	0
SCE1572_829	30.82	14.04
SCE1572_5384	14.24	15.12
SCE1572_1487	123.09	61.42
SCE1572_8840	66.75	27.54
SCE1572_523	39.98	63.74
SCE1572_3667	10.01	1.85
SCE1572_7557	25.41	10.19
SCE1572_1166	43.41	23.31
SCE1572_11125	17.99	16.49
SCE1572_1184	40.03	18.19
SCE1572_7067	85.18	4.1
SCE1572_5834	11.87	237.99
SCE1572_1163	14.01	22.88
SCE1572_4018	27.76	23.38
SCE1572_7445	69.34	36.78
SCE1572_1434	67.94	37.89
SCE1572_9655	19.52	2.56
SCE1572_7673	52.99	14.22
SCE1572_8245	24.34	37.83
SCE1572_4717	18.95	10.95

SCE1572_1580	30.43	13.32
SCE1572_5583	41.59	24.76
SCE1572_6541	16.61	11.61
SCE1572_9920	58.05	25.93
SCE1572_9846	37.56	6.7
SCE1572_1577	28.43	9.65
SCE1572_5334	128.04	398.19
SCE1572_6943	10.83	2.61
SCE1572_3108	34.73	18.58
SCE1572_9279	13.71	10.68
SCE1572_6286	7.13	0
SCE1572_8816	38.41	10.95
SCE1572_8082	0	0
SCE1572_3655	17.58	0
SCE1572_10716	55.13	12.06
SCE1572_11235	19.83	8.95
SCE1572_9715	9	5.41
SCE1572_11294	18.55	3.88
SCE1572_7203	0	0
SCE1572_8035	23.03	15.44
SCE1572_6584	68.23	173.34
SCE1572_8461	104.05	110.91
SCE1572_1336	27.66	91.23
SCE1572_793	206.04	275.1
SCE1572_7665	72.43	8.86
SCE1572_2794	6.41	0
SCE1572_6889	67.87	20.85
SCE1572_9744	34.46	147.21
SCE1572_3750	31.81	11.04
SCE1572_4096	18.49	0
SCE1572_2359	24.16	58.16
SCE1572_8788	0	0
SCE1572_6826	16.85	83.12
SCE1572_3548	30.32	15.81
SCE1572_11159	19.53	33.58
SCE1572_408	20.99	20.03
SCE1572_5227	4.1	0
SCE1572_11130	39.46	22.46
SCE1572_10467	67.08	141.11
SCE1572_3438	0	0
SCE1572_6817	71.65	10
SCE1572_7186	23.94	11.82
SCE1572_96	27.62	26.87
SCE1572_6495	9.05	0
SCE1572_8418	29.69	16.24
SCE1572_7341	46.34	125.53
SCE1572_7078	0	0
SCE1572_10851	23.71	48.6

SCE1572_7495	48.77	17.86
SCE1572_1364	604.14	3379.26
SCE1572_8373	62.77	128.4
SCE1572_1409	113.54	33.02
SCE1572_8385	39.63	17.03
SCE1572_3261	28.98	47.15
SCE1572_10236	132.6	82.75
SCE1572_511	44.03	37.68
SCE1572_4374	34.24	0
SCE1572_5181	34.21	1118.16
SCE1572_151	16.73	7.55
SCE1572_9345	23.92	47.83
SCE1572_9339	179.35	1426.31
SCE1572_3679	29.91	14.89
SCE1572_2753	22.82	7.85
SCE1572_409	25.75	13.77
SCE1572_8963	50.79	34.16
SCE1572_5866	40.03	37.95
SCE1572_9943	151.62	513.51
SCE1572_6922	24.21	205.46
SCE1572_7576	10.88	10.97
SCE1572_10653	318.25	2652.83
SCE1572_3540	113.26	39.57
SCE1572_6624	21.73	24.96
SCE1572_1407	13.74	12.8
SCE1572_7668	15.37	11.31
SCE1572_11058	59.77	64.33
SCE1572_11351	72.08	31.48
SCE1572_4301	60.45	18.64
SCE1572_11186	28.18	4.31
SCE1572_4581	26.93	12
SCE1572_7793	49.5	29.08
SCE1572_10556	0	0
SCE1572_4424	31.42	11.11
SCE1572_6548	0	0
SCE1572_11520	36.18	12.44
SCE1572_9256	610.73	806.94
SCE1572_2808	72.05	27.6
SCE1572_5481	10.83	4.34
SCE1572_1399	16.14	12.49
SCE1572_3976	45.11	60.32
SCE1572_11220	35.25	13.95
SCE1572_5675	17.91	25.06
SCE1572_8046	7.78	0
SCE1572_3532	37.03	13.45
SCE1572_497	25.27	3.5
SCE1572_5045	36.01	52.46
SCE1572_7862	53.92	269.3

SCE1572_10457	0	0
SCE1572_3988	33.32	42.11
SCE1572_4427	40.37	7.91
SCE1572_2966	48.29	24.91
SCE1572_1302	17.42	135.1
SCE1572_2224	20.72	8.31
SCE1572_3722	69.24	3187.94
SCE1572_8483	9.79	12.04
SCE1572_868	45.34	99.76
SCE1572_6323	26.9	34.33
SCE1572_2738	12.93	5.19
SCE1572_551	33.82	53.33
SCE1572_2777	28.51	10.72
SCE1572_10497	58.36	40.55
SCE1572_3054	14.21	0
SCE1572_9102	0	0
SCE1572_8755	87.42	19.73
SCE1572_418	102.88	25.6
SCE1572_2763	32.92	13.36
SCE1572_2782	28.3	22.7
SCE1572_8075	22.32	28.02
SCE1572_8351	20.81	9.54
SCE1572_3440	87.04	34.46
SCE1572_5891	50.87	76.83
SCE1572_687	42.45	12.51
SCE1572_8834	43.26	26.51
SCE1572_8177	26.21	12.9
SCE1572_207	0	0
SCE1572_3032	16.92	17.92
SCE1572_10055	246.13	500.8
SCE1572_3818	19.67	62.63
SCE1572_5068	140.69	163.56
SCE1572_8131	24.63	14.82
SCE1572_2508	37.24	20.63
SCE1572_10008	16.29	9.31
SCE1572_374	35.85	14.83
SCE1572_1856	30.28	12.33
SCE1572_4912	25.27	30.41
SCE1572_9600	125.52	696.94
SCE1572_7917	200.63	339.93
SCE1572_10874	33.9	22.67
SCE1572_11091	39.13	57.55
SCE1572_186	52.74	34.17
SCE1572_9095	46.3	95.52
SCE1572_9559	29.07	39.71
SCE1572_6572	27.19	36.17
SCE1572_9421	51.59	392.16
SCE1572_1864	0	0

SCE1572_332	33.23	84.98
SCE1572_7794	5.69	10.28
SCE1572_1172	24.61	22.21
SCE1572_275	147.88	62.92
SCE1572_2385	41.77	65.66
SCE1572_3762	35.55	89.51
SCE1572_3689	11.93	18.52
SCE1572_1411	39.04	11.8
SCE1572_7456	29.04	15.46
SCE1572_8588	34.66	24.76
SCE1572_3566	33.65	12.68
SCE1572_8290	57.83	19.57
SCE1572_10780	42.59	49.2
SCE1572_7008	0	0
SCE1572_7731	23.78	10.73
SCE1572_324	24.82	20.66
SCE1572_2609	48.58	40.32
SCE1572_9401	28.99	56.35
SCE1572_8525	80.77	98.9
SCE1572_9917	59.75	27.83
SCE1572_9467	22.48	26.59
SCE1572_1069	46.82	10.34
SCE1572_3292	46.34	162.41
SCE1572_3169	33.29	73.72
SCE1572_5164	13.63	295.22
SCE1572_10170	34.1	46.11
SCE1572_4247	51.48	52.89
SCE1572_6962	33.3	56.27
SCE1572_6846	41.49	314.99
SCE1572_7255	100.66	631.85
SCE1572_2472	58.42	1710.78
SCE1572_9380	6.45	3.88
SCE1572_608	66.49	107.77
SCE1572_1253	22.02	10.19
SCE1572_10490	20.86	28.17
SCE1572_3421	20.99	27
SCE1572_7373	38.56	20.45
SCE1572_10214	61.22	75.98
SCE1572_2349	43.73	53.77
SCE1572_2276	67.09	368.74
SCE1572_2721	307.56	243.28
SCE1572_7020	35.7	6.52
SCE1572_19	49.86	238.81
SCE1572_7011	95.48	64.11
SCE1572_2701	26.91	12.51
SCE1572_7597	58.75	260.01
SCE1572_2824	34.7	25.85
SCE1572_5544	31.6	7.7

SCE1572_1255	43	32.12
SCE1572_8202	76.27	20.46
SCE1572_11095	33.06	13.72
SCE1572_6634	22.17	8.36
SCE1572_11539	33.1	7.97
SCE1572_7590	59.2	77.71
SCE1572_2982	0	0
SCE1572_3642	70.68	28.23
SCE1572_4673	23.48	28.61
SCE1572_8381	32.45	31.06
SCE1572_3684	34	4.42
SCE1572_11068	46.49	78.83
SCE1572_5215	83.52	203.59
SCE1572_9176	0	0
SCE1572_7660	37.83	10.93
SCE1572_2295	13.78	0
SCE1572_1951	41.84	14.39
SCE1572_10914	20.14	19.24
SCE1572_3771	31.98	42.05
SCE1572_4734	32.67	45.29
SCE1572_9248	32.85	44.31
SCE1572_9044	37.18	28.23
SCE1572_7753	16.85	10.14
SCE1572_2459	47.09	43.07
SCE1572_619	10.8	23
SCE1572_2208	18.38	11.06
SCE1572_6685	37.1	21.47
SCE1572_8140	20.99	117.1
SCE1572_2100	1.58	3.8
SCE1572_7943	31.8	131.73
SCE1572_3561	24.79	8.95
SCE1572_2812	48.32	52.28
SCE1572_2616	29.81	87.09
SCE1572_11378	20.79	169.95
SCE1572_7741	46.32	40.06
SCE1572_2788	47.69	32.37
SCE1572_10737	36.96	63.93
SCE1572_7952	174.17	512.03
SCE1572_8556	49.82	129.22
SCE1572_6446	8.83	3.54
SCE1572_7823	103.13	466.8
SCE1572_2620	55.94	34.43
SCE1572_3539	38.48	16.7
SCE1572_4863	43.32	44.68
SCE1572_758	8.92	8.35
SCE1572_2022	130.46	1284.31
SCE1572_1531	18.05	14.22
SCE1572_3493	36.88	15.3

SCE1572_9001	555.93	932.58
SCE1572_3160	78.97	65.89
SCE1572_6856	68.77	45.86
SCE1572_8396	43.01	183.76
SCE1572_7565	18.95	8.4
SCE1572_10607	36.47	68.85
SCE1572_4457	43.32	56.36
SCE1572_6302	78.25	1121.52
SCE1572_686	20.33	6.22
SCE1572_9609	88.78	339.95
SCE1572_6534	42.62	9.86
SCE1572_1067	39.81	11.62
SCE1572_8549	36.26	15.87
SCE1572_10335	109.19	266.16
SCE1572_6230	24.22	28.07
SCE1572_8511	41.64	34.69
SCE1572_3376	19.44	163.75
SCE1572_9056	45.62	18.77
SCE1572_6493	24.75	18.62
SCE1572_6929	22.38	5.77
SCE1572_7439	11.97	0
SCE1572_1885	14.26	61.8
SCE1572_1143	35.5	60.52
SCE1572_5873	36.36	25.9
SCE1572_10362	35.24	368.92
SCE1572_935	55.34	187.43
SCE1572_8154	31.74	6.84
SCE1572_5588	25.36	9.29
SCE1572_5557	41.17	94.97
SCE1572_10329	39.77	49.85
SCE1572_9506	24.59	9.35
SCE1572_1129	33.5	15.51
SCE1572_10473	73.97	491.81
SCE1572_9261	53.01	15.31
SCE1572_11027	47.53	31.56
SCE1572_10021	95.36	40.64
SCE1572_5766	232.33	1234.76
SCE1572_8864	51.02	0
SCE1572_8784	23.71	120.71
SCE1572_3341	217.9	478.59
SCE1572_8426	19.72	0
SCE1572_3308	11.44	1.38
SCE1572_5817	7.78	0
SCE1572_8452	27.04	230.11
SCE1572_11319	65.98	16.89
SCE1572_11590	14.09	10.53
SCE1572_638	33.88	47
SCE1572_676	21	7.62

SCE1572_924	152.75	620.91
SCE1572_477	13.65	12.11
SCE1572_5854	7.4	0
SCE1572_8399	53.38	65.42
SCE1572_9778	57.71	241.71
SCE1572_10799	77.25	68.95
SCE1572_6307	68.88	78.39
SCE1572_9318	257.91	396.74
SCE1572_8454	18.08	41.29
SCE1572_10319	52.91	85.53
SCE1572_11003	106.45	236.81
SCE1572_2102	24.64	84.39
SCE1572_9742	46.85	67.24
SCE1572_11176	22.61	144.05
SCE1572_9915	110.61	57.58
SCE1572_3158	25.95	14.56
SCE1572_1959	1.92	0
SCE1572_11222	64.34	27.61
SCE1572_1390	54.9	173.82
SCE1572_8976	10.99	2.64
SCE1572_9109	7.9	7.6
SCE1572_8307	848.7	43.62
SCE1572_5075	4.17	1.67
SCE1572_5368	19.93	10.28
SCE1572_4535	55.25	24.74
SCE1572_1060	0	0
SCE1572_10024	29.24	19.56
SCE1572_7131	19.65	3.38
SCE1572_3131	25.98	13.49
SCE1572_9820	116.44	97.24
SCE1572_9221	51.99	36.99
SCE1572_2830	21.9	4.05
SCE1572_8737	50.22	23.24
SCE1572_397	40.43	30.41
SCE1572_8722	28.66	9.08
SCE1572_6430	33.59	34.75
SCE1572_10990	57.68	63.46
SCE1572_2957	145.5	81.49
SCE1572_2633	45.96	3.5
SCE1572_2152	25.63	34.27
SCE1572_8609	37.41	21.69
SCE1572_2351	36.14	450.55
SCE1572_4214	0	0
SCE1572_10179	60.75	280.34
SCE1572_4310	68.39	21.15
SCE1572_2901	0	0
SCE1572_4780	40.27	67.51
SCE1572_2325	13.1	69.1

SCE1572_5726	32.24	250.12
SCE1572_4085	37.34	12.58
SCE1572_10771	28.77	18.65
SCE1572_4758	77.5	42.65
SCE1572_5256	41.35	16.59
SCE1572_1299	48.8	23.49
SCE1572_5810	5204.42	6531.47
SCE1572_6758	26.84	14.84
SCE1572_4391	33.01	13.05
SCE1572_9224	49.19	10.6
SCE1572_5469	78.37	16.48
SCE1572_9835	17.62	30.84
SCE1572_8593	0	0
SCE1572_11483	217.75	302.81
SCE1572_1668	35.31	28.99
SCE1572_1459	47.25	17.79
SCE1572_3583	30.05	16.94
SCE1572_412	15.64	16.36
SCE1572_600	1.65	0
SCE1572_9438	27.02	52.39
SCE1572_2566	27.99	0
SCE1572_9883	42.41	51.04
SCE1572_6088	25.65	65.96
SCE1572_2140	41.26	19.86
SCE1572_10421	30.32	13.84
SCE1572_177	14.1	4.24
SCE1572_10303	85.02	50.68
SCE1572_8851	10.7	103.04
SCE1572_9275	49.25	59.03
SCE1572_7141	27.49	8.02
SCE1572_5063	7.34	5.89
SCE1572_1703	80.79	89.02
SCE1572_10427	7.22	4.34
SCE1572_4378	179.18	20.42
SCE1572_9816	13.91	21.2
SCE1572_5436	64.98	25.32
SCE1572_7434	20.61	88.57
SCE1572_8193	25.51	8.77
SCE1572_2565	41.77	116.95
SCE1572_2288	37.46	54.38
SCE1572_3407	98.32	255.81
SCE1572_451	13.45	25.01
SCE1572_10258	25.27	4.34
SCE1572_10908	47.69	85.34
SCE1572_6533	86	10.67
SCE1572_9584	23.34	24.8
SCE1572_11239	30.59	11.91
SCE1572_6703	25.2	16.11

SCE1572_11426	44.96	65.43
SCE1572_11433	74.79	148.25
SCE1572_7054	24.09	23.87
SCE1572_10145	192.69	309.49
SCE1572_6669	15.66	16.59
SCE1572_424	30.44	171.4
SCE1572_7603	23.74	7.37
SCE1572_5010	86.45	506.08
SCE1572_8712	33.18	13.52
SCE1572_1532	37.38	134.97
SCE1572_4541	79.68	24.22
SCE1572_11156	43.14	44.5
SCE1572_2923	73.76	35.75
SCE1572_8324	66.21	23.9
SCE1572_4907	38.29	63.89
SCE1572_1086	40.6	51.18
SCE1572_138	30.32	38.41
SCE1572_3447	48.87	78.41
SCE1572_11404	26.57	18.81
SCE1572_3467	49.52	25.01
SCE1572_2077	30.88	41.04
SCE1572_1034	52.4	18.06
SCE1572_9949	69.89	20.7
SCE1572_6714	100.06	244.5
SCE1572_10060	0	0
SCE1572_1650	12.38	7.45
SCE1572_3244	108.91	241.57
SCE1572_2213	32.17	71.37
SCE1572_1321	36.61	81.41
SCE1572_11242	95.72	48.77
SCE1572_727	38.21	8.9
SCE1572_1447	34.18	13.13
SCE1572_3859	52.83	83.66
SCE1572_7886	24.99	112.28
SCE1572_10741	21.76	32.1
SCE1572_5400	13.51	7.23
SCE1572_11565	31.42	65.75
SCE1572_6214	13.24	4.4
SCE1572_6468	29.98	9.71
SCE1572_10937	31.05	99.92
SCE1572_8931	17.01	7.23
SCE1572_5964	13.78	12.76
SCE1572_8632	23.24	90.56
SCE1572_3887	32.88	116.37
SCE1572_7678	35.2	420.31
SCE1572_2009	49.6	13.57
SCE1572_4732	16.93	12.97
SCE1572_9734	29.6	86.89

SCE1572_7472	71.02	121
SCE1572_3191	0	0
SCE1572_5760	18.34	9.81
SCE1572_558	44.47	324.38
SCE1572_645	5.41	18.25
SCE1572_11076	13.28	251.53
SCE1572_7111	24.08	23.19
SCE1572_8934	12.09	5.6
SCE1572_6047	9.94	149.56
SCE1572_7091	34.49	28.54
SCE1572_9453	21.31	42.5
SCE1572_2237	90.44	70.42
SCE1572_8524	44.49	262.15
SCE1572_10151	39	271.04
SCE1572_3396	79.17	74.06
SCE1572_9789	12.52	9.77
SCE1572_10660	6.87	0
SCE1572_906	43.21	0
SCE1572_7351	15.49	6.07
SCE1572_1804	2.27	0
SCE1572_7529	34.66	15.64
SCE1572_7789	0	0
SCE1572_10748	21.14	43.86
SCE1572_1989	0	4.62
SCE1572_1949	4.81	0
SCE1572_6237	27.07	11.73
SCE1572_9782	36.05	25.52
SCE1572_7306	6.69	0
SCE1572_2281	36.07	16.19
SCE1572_7964	31.73	29.7
SCE1572_7423	83.99	133.89
SCE1572_5667	3.89	0
SCE1572_10563	38.42	28.45
SCE1572_7275	71.78	27.18
SCE1572_11133	38.35	24.69
SCE1572_9114	3.65	0
SCE1572_7239	0	0
SCE1572_4027	41.66	111.12
SCE1572_5905	0	0
SCE1572_7267	58.09	18.76
SCE1572_2133	61.57	16.08
SCE1572_10810	84.47	185.07
SCE1572_4116	12.84	1.72
SCE1572_5080	164.77	1102.68
SCE1572_8448	36.22	12.35
SCE1572_7157	60.19	22.88
SCE1572_2129	23.78	16.1
SCE1572_794	170.99	241.14

SCE1572_4368	31.92	120.04
SCE1572_11311	47.72	22.16
SCE1572_11337	18.08	10.04
SCE1572_9843	33.13	104.59
SCE1572_2222	30.09	25.07
SCE1572_5124	42.18	4.12
SCE1572_1070	19.47	1.12
SCE1572_1187	41.94	2.59
SCE1572_545	21.62	4.79
SCE1572_2849	0	0
SCE1572_2230	50.74	171.07
SCE1572_1076	25.27	53.67
SCE1572_3932	765.83	4832.6
SCE1572_7165	80.59	19.73
SCE1572_10357	37.04	350.66
SCE1572_1051	24.24	18.03
SCE1572_7137	35.56	8.07
SCE1572_3023	37.9	16.19
SCE1572_7337	58.24	234.93
SCE1572_11344	71.16	275.42
SCE1572_11394	82.43	308.24
SCE1572_11594	80.74	505.02
SCE1572_3525	57.76	793.34
SCE1572_3917	123.47	76.08
SCE1572_7408	23.87	128.4
SCE1572_7932	18.08	12.76
SCE1572_3065	18.19	20.44
SCE1572_1835	42.64	38.77
SCE1572_5417	72.31	97.69
SCE1572_6263	20.14	18.71
SCE1572_2908	33.43	46.78
SCE1572_4998	16.8	34.96
SCE1572_5705	53.7	66.52
SCE1572_11269	33.33	41.68
SCE1572_10778	50.97	44.26
SCE1572_1114	38.53	158.6
SCE1572_1515	18.09	49.76
SCE1572_10885	30.99	16.84
SCE1572_6780	0	0
SCE1572_4885	39.74	20.08
SCE1572_329	24.48	52.72
SCE1572_7039	36.83	15.46
SCE1572_2000	4.07	0
SCE1572_9025	63.17	243.28
SCE1572_4176	90.69	39.69
SCE1572_3506	90.21	266.39
SCE1572_6256	28.55	12.21
SCE1572_5642	9.89	10.58

SCE1572_2374	149.17	1268.4
SCE1572_5660	33.34	8.51
SCE1572_7032	37.31	17.92
SCE1572_4616	8.61	4.15
SCE1572_10293	0	0
SCE1572_8577	0	0
SCE1572_3910	81.13	155.25
SCE1572_1431	4.97	0
SCE1572_4795	45.1	122.41
SCE1572_316	49.19	26.02
SCE1572_5633	30.72	22.02
SCE1572_3703	26.91	11.88
SCE1572_4595	12.42	11.21
SCE1572_10587	39.43	12.03
SCE1572_10763	2.71	0
SCE1572_10935	15.98	13.42
SCE1572_4963	109.81	427.13
SCE1572_6137	10.78	13.84
SCE1572_6748	37.67	22.12
SCE1572_5606	12.38	10.43
SCE1572_9687	138.22	301.61
SCE1572_2832	20.02	35.44
SCE1572_1818	19.23	24.51
SCE1572_4454	22.24	14.6
SCE1572_221	54.64	58.54
SCE1572_6037	9.63	0
SCE1572_11273	54.75	236.95
SCE1572_6277	56.93	65.54
SCE1572_3590	16.08	0
SCE1572_9591	45.94	606.7
SCE1572_6556	31.63	10.07
SCE1572_9625	20.85	15.37
SCE1572_4274	53	272.95
SCE1572_3501	15.43	13
SCE1572_10414	37.03	50.45
SCE1572_5925	27.6	41.2
SCE1572_11104	10.83	72.98
SCE1572_3573	11.14	0.96
SCE1572_9635	68.39	69.07
SCE1572_9194	31.22	49.37
SCE1572_2062	94.25	64.11
SCE1572_1990	41.88	22.4
SCE1572_9006	48.42	68.47
SCE1572_4254	8.92	3.07
SCE1572_6660	38.97	37.26
SCE1572_6434	136.98	37.03
SCE1572_9096	37.9	101.96
SCE1572_11386	102.18	417.2

SCE1572_5521	42.93	47.56
SCE1572_2842	8.29	0
SCE1572_10730	17.97	4.05
SCE1572_2286	14.28	34.36
SCE1572_11466	32.62	22.69
SCE1572_1440	38.96	46.88
SCE1572_9727	25.97	15.12
SCE1572_5503	41.54	37.99
SCE1572_10188	49.73	86.83
SCE1572_3854	36.37	37.78
SCE1572_9729	45.94	51.04
SCE1572_888	36.71	3.84
SCE1572_8618	44.06	31.19
SCE1572_7014	14.44	2.9
SCE1572_7244	0	0
SCE1572_1233	43.54	14.86
SCE1572_9239	55.13	24.88
SCE1572_2490	42.53	17.19
SCE1572_61	35.39	73.49
SCE1572_6335	26.43	4.73
SCE1572_6765	97.37	30.13
SCE1572_9170	7.84	0
SCE1572_268	0	0
SCE1572_3927	174.8	279.06
SCE1572_993	18.34	37.66
SCE1572_8575	95.49	16.07
SCE1572_11250	21.06	0
SCE1572_2748	35.56	50.1
SCE1572_6245	15.46	3.54
SCE1572_10450	23.85	4.1
SCE1572_44	32.3	47.5
SCE1572_4878	9.52	4.58
SCE1572_1646	0	0
SCE1572_7509	0	0
SCE1572_6008	60.29	597.53
SCE1572_8285	44.09	83.78
SCE1572_5281	49.24	41.38
SCE1572_7812	140.01	1230.68
SCE1572_10549	39.02	20.63
SCE1572_172	143.42	59.18
SCE1572_2944	70.19	28.72
SCE1572_2332	76.18	70.21
SCE1572_9460	24.33	8.28
SCE1572_194	0	0
SCE1572_4556	30.1	12.37
SCE1572_11456	46.76	23.87
SCE1572_869	0	0
SCE1572_1905	7.29	0

SCE1572_7101	141.39	405.31
SCE1572_11474	51.13	14.6
SCE1572_5193	34.62	174.96
SCE1572_5443	53.02	17.91
SCE1572_4741	24.76	13.39
SCE1572_10553	0	0
SCE1572_3114	68.32	22.36
SCE1572_4068	28.91	43.69
SCE1572_11072	57.88	130.68
SCE1572_9767	69.36	584.26
SCE1572_756	0	0
SCE1572_4464	84.71	27.41
SCE1572_7980	38.62	30.98
SCE1572_6512	36.27	12.58
SCE1572_9297	112.18	426.09
SCE1572_5574	58.44	40.19
SCE1572_6775	0	0
SCE1572_9184	29.76	16.22
SCE1572_3090	86.98	84.21
SCE1572_2940	19.85	40.18
SCE1572_1007	63.26	163.65
SCE1572_10101	14.53	7.65
SCE1572_9664	32.31	167.51
SCE1572_4521	12.94	4.45
SCE1572_9451	0	0
SCE1572_10349	0	0
SCE1572_6063	9.19	0
SCE1572_7711	32.27	84
SCE1572_6040	145.2	224.62
SCE1572_7295	83.18	95.55
SCE1572_5612	56.76	110.41
SCE1572_914	78.11	213.79
SCE1572_7338	23.01	200.71
SCE1572_9546	34.48	57.49
SCE1572_2361	59.52	104.84
SCE1572_10081	574.3	3500.73
SCE1572_6425	40.68	10.01
SCE1572_7087	40.32	17.28
SCE1572_3122	62.01	24.28
SCE1572_10629	37.57	146.47
SCE1572_6183	44.99	13.54
SCE1572_2407	77.91	313.22
SCE1572_9479	109.09	121.27
SCE1572_3946	330.25	346.86
SCE1572_2018	14.29	13.37
SCE1572_7193	36.12	44.33
SCE1572_8678	16.04	10.53
SCE1572_6295	40.55	237.62

SCE1572_4225	36.82	7.82
SCE1572_9757	21.45	16.81
SCE1572_2709	59.3	21.68
SCE1572_1134	23.83	20.85
SCE1572_5682	22.26	95.42
SCE1572_6996	32.54	21.36
SCE1572_4442	44.67	27.34
SCE1572_10956	19.92	65.5
SCE1572_3745	12.69	3.92
SCE1572_4336	91.79	17.38
SCE1572_3099	17.26	3.93
SCE1572_11336	9.05	5.45
SCE1572_1216	3.89	0
SCE1572_535	24.19	64.43
SCE1572_9158	58.69	31.39
SCE1572_166	25.08	65.85
SCE1572_3410	9.94	0
SCE1572_2161	32.18	11.79
SCE1572_3908	17.92	3.32
SCE1572_6777	6.85	5.5
SCE1572_6645	26.86	16.44
SCE1572_3965	25.16	21.2
SCE1572_4261	48.13	25.43
SCE1572_2344	67.43	382.21
SCE1572_6770	2.79	4.48
SCE1572_1828	33.28	11.87
SCE1572_5426	69.7	52.29
SCE1572_7726	20.56	507.18
SCE1572_598	23.45	13.17
SCE1572_3901	35.71	81.43
SCE1572_8478	6.13	0
SCE1572_10284	40.07	147.9
SCE1572_11240	32.66	31.81
SCE1572_4461	201.41	21.55
SCE1572_10638	19.56	0
SCE1572_3799	64.44	220.36
SCE1572_1786	39.15	73.91
SCE1572_10158	21.02	12.04
SCE1572_8338	74.65	51.95
SCE1572_8980	73.3	24.17
SCE1572_3954	30.96	21.42
SCE1572_6417	0	0
SCE1572_9079	0	0
SCE1572_3083	33.21	7.36
SCE1572_10595	55.11	14.81
SCE1572_3328	137.1	392.1
SCE1572_3434	73.01	136.28
SCE1572_9984	72.32	35.93

SCE1572_8968	21.47	14.09
SCE1572_39	34.71	29.24
SCE1572_2411	62.39	244.78
SCE1572_1999	10.86	5.23
SCE1572_3318	9.93	30.2
SCE1572_10817	27.66	20.49
SCE1572_9696	50.39	125.01
SCE1572_6524	120.64	122.81
SCE1572_6569	15.99	95.57
SCE1572_7689	0	0
SCE1572_6110	46.96	19.38
SCE1572_7027	37.87	52.19
SCE1572_2816	83.72	36.49
SCE1572_5263	0	0
SCE1572_2933	0	0
SCE1572_6272	35.59	58.97
SCE1572_7271	27.57	9.76
SCE1572_4318	44.97	8.62
SCE1572_8239	40.9	111.64
SCE1572_2660	0	0
SCE1572_4415	29.23	6.59
SCE1572_11571	27.71	1391.15
SCE1572_308	30.14	69.19
SCE1572_3326	31.48	65.81
SCE1572_4125	18.45	7.67
SCE1572_7515	26.55	30.93
SCE1572_6405	30.11	39.42
SCE1572_802	32.25	45.18
SCE1572_9326	409.36	583.88
SCE1572_4981	50.93	188.16
SCE1572_7173	38.98	160.3
SCE1572_8233	13.81	23.75
SCE1572_10355	25.11	167.91
SCE1572_8626	76.35	49.88
SCE1572_822	5.83	0
SCE1572_2586	9.18	3.22
SCE1572_1120	35.94	13.71
SCE1572_10702	12.85	142.26
SCE1572_9415	3.3	0
SCE1572_6120	64	57.66
SCE1572_3047	24.02	12.85
SCE1572_1540	21.04	46.23
SCE1572_7232	55.31	22.03
SCE1572_3848	15.45	9.64
SCE1572_7998	25.73	12.25
SCE1572_8882	0	0
SCE1572_4751	44.2	178.74
SCE1572_6050	0	0

SCE1572_10386	57.77	26.78
SCE1572_9410	91.01	1580.21
SCE1572_3578	9.99	32.78
SCE1572_9550	160.39	108.57
SCE1572_6199	4.15	0
SCE1572_4504	56.07	30.41
SCE1572_7179	25.63	15.42
SCE1572_7040	12.55	0
SCE1572_4185	29.75	50.92
SCE1572_2868	37.18	33.7
SCE1572_304	78.61	22.36
SCE1572_11445	58.57	83.87
SCE1572_4021	31.03	45.09
SCE1572_4574	23.51	14.28
SCE1572_2367	0	0
SCE1572_1750	39.93	37.61
SCE1572_986	99.98	372.81
SCE1572_7378	8.59	7.39
SCE1572_1790	21.1	5.9
SCE1572_2796	23.16	22.81
SCE1572_2184	55.55	18.09
SCE1572_2119	54.1	111.36
SCE1572_6351	64.54	19.32
SCE1572_8494	6.49	0
SCE1572_8812	41.85	16.51
SCE1572_898	18.28	11.65
SCE1572_11468	51.52	152.35
SCE1572_6079	0	0
SCE1572_8680	21.04	17.87
SCE1572_3347	159.06	85.59
SCE1572_6055	117.56	171.88
SCE1572_5153	36.18	10.76
SCE1572_119	82.41	223.79
SCE1572_8896	65.51	322.12
SCE1572_1242	59.85	64.75
SCE1572_3419	63.59	79.36
SCE1572_9666	2.31	16.67
SCE1572_2487	34.28	133.9
SCE1572_1264	0	0
SCE1572_5098	59.33	254.61
SCE1572_2474	43.16	295.06
SCE1572_3592	16.01	13.6
SCE1572_1830	37.72	38.07
SCE1572_5597	82.17	300.16
SCE1572_7095	61.94	22.69
SCE1572_10249	0	0
SCE1572_4482	75.5	182.21
SCE1572_161	34.44	9.91

SCE1572_4585	30.55	40.1
SCE1572_63	28.54	25.34
SCE1572_2872	25.86	18.76
SCE1572_2379	22.92	100.42
SCE1572_6262	24.11	14.95
SCE1572_5456	11.43	4.23
SCE1572_8360	16.51	3.61
SCE1572_7880	34.24	35.32
SCE1572_6935	48.48	48.41
SCE1572_6679	48.52	40.14
SCE1572_3121	38.44	64.86
SCE1572_4352	0	0
SCE1572_1623	0	0
SCE1572_8671	13.78	4.74
SCE1572_10766	62.82	3112.93
SCE1572_4866	23.94	220
SCE1572_1285	93.23	1921.58
SCE1572_9791	0	0
SCE1572_24	35.91	47.06
SCE1572_9539	41.18	9.01
SCE1572_3941	648.03	891.46
SCE1572_6796	66.96	127.72
SCE1572_9330	334.31	1053.97
SCE1572_1657	39.51	33.17
SCE1572_3963	63.31	19.25
SCE1572_7879	53.99	46.69
SCE1572_5175	33.88	17.97
SCE1572_8423	92.88	60.82
SCE1572_4437	0	0
SCE1572_2421	20.39	33.01
SCE1572_6988	22.28	3.92
SCE1572_11010	29.03	144.07
SCE1572_5170	54.88	683.28
SCE1572_8563	74.48	464.16
SCE1572_3747	0	0
SCE1572_3998	28.74	52.28
SCE1572_4977	59.38	991.07
SCE1572_6959	2.57	0
SCE1572_1684	48.48	63.3
SCE1572_7333	0	0
SCE1572_3804	18.48	7.6
SCE1572_9627	52.96	19.9
SCE1572_7297	48.88	24.01
SCE1572_3672	28.19	17.38
SCE1572_1107	63.63	14.18
SCE1572_11117	33.19	21.79
SCE1572_11541	24.26	2.43
SCE1572_10815	13.48	9.93

SCE1572_11555	33.59	14.63
SCE1572_7690	33.13	104.59
SCE1572_6318	13.15	8.33
SCE1572_6941	24.49	10.4
SCE1572_8	75.18	78.85
SCE1572_9759	44.12	17.38
SCE1572_7657	39.68	26.94
SCE1572_6851	7.26	4.37
SCE1572_7002	0	0
SCE1572_9166	55.81	15.67
SCE1572_2975	35.64	7.09
SCE1572_4128	23.33	25.52
SCE1572_2261	37.23	119.46
SCE1572_4234	40.5	12.5
SCE1572_461	16.68	7.3
SCE1572_9873	18.38	14.74
SCE1572_9372	41.12	25.77
SCE1572_72	42.15	57.19
SCE1572_228	24.86	0
SCE1572_82	0	0
SCE1572_5341	39.42	44.14
SCE1572_10486	18.95	34.21
SCE1572_6682	46.65	303.68
SCE1572_9571	17.2	18.58
SCE1572_9040	26.68	171.25
SCE1572_11052	59.33	684.75
SCE1572_11360	125.74	569.24
SCE1572_5202	16.77	26.07
SCE1572_6880	2.26	0
SCE1572_4610	16.04	14.48
SCE1572_7675	58.7	15.1
SCE1572_1661	52.43	155.75
SCE1572_7892	492.21	22628.64
SCE1572_9047	5.14	0
SCE1572_5277	0	0
SCE1572_5998	30.12	58.98
SCE1572_8266	156.42	118.06
SCE1572_938	20.22	20.85
SCE1572_7383	140.14	51.29
SCE1572_2457	2.81	0
SCE1572_2054	100	143.64
SCE1572_571	0	0
SCE1572_4856	44.2	12.77
SCE1572_591	103.15	156.24
SCE1572_4653	71.56	33.37
SCE1572_3661	28.72	5.67
SCE1572_10229	14.21	11.4
SCE1572_6595	0	0

SCE1572_10091	80.53	462.5
SCE1572_4346	0	0
SCE1572_2896	42.03	10.84
SCE1572_692	20.22	24.33
SCE1572_881	75.1	109.82
SCE1572_4474	0	0
SCE1572_7801	68.98	112.6
SCE1572_6981	105.01	9.46
SCE1572_2582	52.61	28.47
SCE1572_5949	37.77	20.52
SCE1572_6269	13.87	40.05
SCE1572_11015	10.11	9.73
SCE1572_5129	0	0
SCE1572_1584	44.8	215.64
SCE1572_427	29.45	338.67
SCE1572_857	85.62	1850.37
SCE1572_3371	28.11	15.31
SCE1572_6197	3.76	3.02
SCE1572_640	9.88	16.64
SCE1572_9128	33.69	192.6
SCE1572_1193	35.91	62.74
SCE1572_5713	118.13	62.69
SCE1572_7201	25.96	22.09
SCE1572_11346	25.45	105.89
SCE1572_11037	34.96	166.78
SCE1572_7343	42.74	48.98
SCE1572_8128	31.22	0
SCE1572_1226	46.75	14.72
SCE1572_513	35.22	17.76
SCE1572_5913	7.37	3.73
SCE1572_1944	54.87	18.15
SCE1572_2663	29.78	19.55
SCE1572_4762	66.68	1070.27
SCE1572_3875	49.53	100.32
SCE1572_9233	39.69	174.56
SCE1572_3797	38.64	55.42
SCE1572_9285	13.43	16.16
SCE1572_11165	33.69	5.59
SCE1572_9532	62.53	973.13
SCE1572_8340	0	0
SCE1572_9803	22.16	35.16
SCE1572_8719	29.54	16.73
SCE1572_7418	0	0
SCE1572_4619	0	0
SCE1572_3836	16.85	0
SCE1572_1873	17.89	8.2
SCE1572_7970	24.24	37.24
SCE1572_3265	0	0

SCE1572_7069	54.44	207.34
SCE1572_8463	111.48	491.29
SCE1572_7553	25.08	13.72
SCE1572_11110	58.96	170.52
SCE1572_3915	8.39	0
SCE1572_5528	52.99	27.33
SCE1572_6920	43.32	0
SCE1572_9578	44.36	16.22
SCE1572_8019	0	0.01
SCE1572_8538	33.65	28.58
SCE1572_10597	64.98	17.38
SCE1572_3834	3.7	0
SCE1572_261	33.17	10.26
SCE1572_877	222.04	449.68
SCE1572_7620	21.32	19.86
SCE1572_8823	40.3	25.41
SCE1572_10432	38.98	24.38
SCE1572_3485	44.59	33.6
SCE1572_9925	222.8	704.88
SCE1572_736	36.15	26.58
SCE1572_7536	65.42	17.24
SCE1572_9893	22.83	12.7
SCE1572_3474	24.22	10.6
SCE1572_4680	4.15	0
SCE1572_5298	24.42	23.23
SCE1572_940	53.04	14.8
SCE1572_8983	4.94	0
SCE1572_6162	31.72	43.52
SCE1572_11509	27.99	19.34
SCE1572_9247	35.26	7.64
SCE1572_8387	112.31	23.65
SCE1572_10404	30.85	25.17
SCE1572_7127	3.16	0
SCE1572_7652	68.11	27.32
SCE1572_1001	0	0
SCE1572_3203	32.87	391.72
SCE1572_2243	61.24	34.79
SCE1572_10340	29.61	9.38
SCE1572_4698	6.77	3.26
SCE1572_92	89.36	198.98
SCE1572_547	13.91	14.62
SCE1572_8219	28.43	30.41
SCE1572_4004	0	0
SCE1572_4774	41.81	0
SCE1572_5326	0	0
SCE1572_6895	0	0
SCE1572_6208	0	0
SCE1572_6946	29.09	16.38

SCE1572_406	3.37	0
SCE1572_6188	14.88	10.07
SCE1572_5364	12.43	0
SCE1572_3196	48.42	136.93
SCE1572_3934	0	0
SCE1572_8611	32.87	12.92
SCE1572_6810	197.42	543.58
SCE1572_8806	0	0
SCE1572_4158	64.98	34.09
SCE1572_625	0	0
SCE1572_9337	83.96	251.67
SCE1572_9717	95.36	1145.26
SCE1572_848	59.17	42.73
SCE1572_9658	16.98	35.54
SCE1572_6828	16.68	13.21
SCE1572_7207	9.24	28.19
SCE1572_5783	24.59	46.62
SCE1572_7362	18.49	4.45
SCE1572_6948	29.55	24.36
SCE1572_6328	37.74	67.35
SCE1572_5971	82.64	157.8
SCE1572_11515	6.2	6.78
SCE1572_9998	16.6	3.77
SCE1572_7827	159.43	466.5
SCE1572_11498	0	0
SCE1572_8627	23.22	14.82
SCE1572_6396	13.27	44.85
SCE1572_1816	18.66	5.61
SCE1572_1689	34.99	47.72
SCE1572_540	29.26	18.76
SCE1572_4847	41.19	69.61
SCE1572_54	28.73	14.89
SCE1572_2120	51.89	85.78
SCE1572_1480	64.59	20.95
SCE1572_3175	47.89	87.95
SCE1572_6472	15.5	2.33
SCE1572_5395	0	0
SCE1572_7930	109.01	50.42
SCE1572_6485	17.57	23.49
SCE1572_2317	14.21	9.12
SCE1572_11521	27.57	10.3
SCE1572_6877	77.71	187.42
SCE1572_2337	17.4	9.97
SCE1572_9657	26.5	26.27
SCE1572_99	29.08	15
SCE1572_10461	44	198.87
SCE1572_2610	23.85	41
SCE1572_2693	13.36	5.8

SCE1572_5047	36.94	48.16
SCE1572_465	41.95	99.2
SCE1572_1806	21.83	14.6
SCE1572_1545	37.9	28.17
SCE1572_10098	21.79	88.16
SCE1572_783	51.08	20.7
SCE1572_9872	51.27	243.28
SCE1572_1542	11.37	29.65
SCE1572_6838	89.19	47.7
SCE1572_283	41.09	32.97
SCE1572_4492	33.69	27.47
SCE1572_850	52.08	176.1
SCE1572_3599	25.27	44.82
SCE1572_9433	136.62	180.46
SCE1572_1837	73.88	139.06
SCE1572_10439	194.26	713.55
SCE1572_10075	901.55	1765.8
SCE1572_10995	42.06	28.49
SCE1572_5183	23.82	457.2
SCE1572_10468	12.81	5.14
SCE1572_10502	43.55	33.89
SCE1572_1563	20.77	0
SCE1572_8311	7.17	1.26
SCE1572_3752	75.25	145.97
SCE1572_4381	18.06	8.21
SCE1572_164	21.6	13.48
SCE1572_2810	4.79	0
SCE1572_4276	43.84	24.43
SCE1572_5476	32.92	843.63
SCE1572_10133	13.3	10.06
SCE1572_11496	72.29	20.02
SCE1572_3154	11.05	11.82
SCE1572_10954	76.64	22.72
SCE1572_11204	63.75	141.64
SCE1572_6699	23.72	11.58
SCE1572_6692	6.23	0
SCE1572_2592	0	0
SCE1572_11437	39.98	61.73
SCE1572_4044	23.58	61.63
SCE1572_4691	38.81	65.69
SCE1572_5415	44.23	12.45
SCE1572_4748	86.18	42.25
SCE1572_7421	23.33	9.36
SCE1572_5627	43.8	6.08
SCE1572_6284	40.96	81.29
SCE1572_6442	71.5	40.55
SCE1572_916	0	0
SCE1572_240	19.77	0

SCE1572_11184	20.96	4.45
SCE1572_6705	567.87	2153.41
SCE1572_4810	0	0
SCE1572_2750	41.87	62.59
SCE1572_5828	16.52	9.23
SCE1572_7923	9.37	2.05
SCE1572_7154	14.56	9.67
SCE1572_9510	9.3	0
SCE1572_7887	79.1	393.68
SCE1572_9937	17.33	0
SCE1572_4961	22.81	96.88
SCE1572_8370	30.89	10.3
SCE1572_2727	26.49	13.19
SCE1572_8092	16.07	2.42
SCE1572_8693	45.12	9.78
SCE1572_2531	20.49	0
SCE1572_746	35.05	51.33
SCE1572_2099	20.11	12.41
SCE1572_5565	78.24	58.54
SCE1572_10542	80.2	53.54
SCE1572_9272	37.06	35.68
SCE1572_7316	0	0
SCE1572_8991	11.17	23.05
SCE1572_9863	56.46	21.82
SCE1572_5231	61.79	235.7
SCE1572_840	40.85	102.1
SCE1572_11308	24.93	5.75
SCE1572_2198	0	0
SCE1572_10222	21.92	36.64
SCE1572_4626	146.64	260.35
SCE1572_11257	47.93	77.69
SCE1572_10224	144.92	96.68
SCE1572_6698	19.53	20.15
SCE1572_580	16.48	37.02
SCE1572_6523	2.37	0
SCE1572_5519	0	0
SCE1572_8347	51.48	54.21
SCE1572_10511	0	0
SCE1572_444	27.27	7.88
SCE1572_5371	63.77	83.72
SCE1572_7543	17.02	72.24
SCE1572_7928	93.1	73.17
SCE1572_6072	34.19	14.31
SCE1572_6289	11.35	25.72
SCE1572_7872	94.81	299.73
SCE1572_7766	19.86	7.59
SCE1572_7584	20.13	0
SCE1572_2304	67.56	194.56

SCE1572_10314	30.72	4.77
SCE1572_6450	55.03	42.87
SCE1572_9907	38.53	62.5
SCE1572_385	2.76	0
SCE1572_10765	32.54	270.58
SCE1572_647	0	0
SCE1572_8929	32.26	116.46
SCE1572_6354	10.16	3.76
SCE1572_5287	38.29	243.28
SCE1572_6201	45.24	20.74
SCE1572_9991	52.67	15.39
SCE1572_395	38.2	54.41
SCE1572_9673	36.31	119.6
SCE1572_774	63.97	153.09
SCE1572_10195	16.68	5.47
SCE1572_796	83.72	163.41
SCE1572_1428	23.47	7.17
SCE1572_1400	15.32	15.53
SCE1572_2573	34.13	9.39
SCE1572_5836	27.07	26.07
SCE1572_11301	21.02	57.81
SCE1572_2655	29.85	21.43
SCE1572_4710	14.94	8.46
SCE1572_479	36.74	11.18
SCE1572_5447	54.65	19.8
SCE1572_6103	15.09	6.92
SCE1572_3603	168.43	104.26
SCE1572_4110	20.33	4.98
SCE1572_813	26.3	12.1
SCE1572_8604	14.18	29.49
SCE1572_8055	20.14	17.11
SCE1572_5747	6.1	1.83
SCE1572_1893	3.74	38.29
SCE1572_2990	8.42	0
SCE1572_3542	0	0
SCE1572_5938	14.21	3.8
SCE1572_1912	27.13	0
SCE1572_10333	0	0
SCE1572_967	0	0
SCE1572_11447	64.18	61.45
SCE1572_8007	54.01	1130.54
SCE1572_780	23.58	8.11
SCE1572_10863	31.11	362.81
SCE1572_6841	90.88	116.16
SCE1572_9493	0	0
SCE1572_10754	35.69	14.81
SCE1572_6212	67.53	26.98
SCE1572_1405	17.15	11.56

SCE1572_5390	0	0
SCE1572_7572	77.12	177.72
SCE1572_8821	9.52	22.91
SCE1572_1339	21.25	105.18
SCE1572_4924	32.34	51.9
SCE1572_7839	30.96	42.4
SCE1572_533	14.13	49.48
SCE1572_6340	27.77	23.34
SCE1572_1485	43.58	36.99
SCE1572_6969	49.29	442.22
SCE1572_4180	8.37	17.62
SCE1572_6543	94.08	115.67
SCE1572_7713	22.74	10.14
SCE1572_1020	28.36	100.03
SCE1572_1352	43.37	62.15
SCE1572_6221	59.17	31.15
SCE1572_4928	3.94	0
SCE1572_11194	33.49	168.54
SCE1572_8274	18.84	32.07
SCE1572_7612	43.5	88.07
SCE1572_8192	28.25	13.88
SCE1572_2938	44.56	84.21
SCE1572_1723	9.92	6.82
SCE1572_10164	44.66	14.14
SCE1572_10441	14.39	42.62
SCE1572_10980	9.48	16.67
SCE1572_3704	41.35	193.94
SCE1572_2165	85.65	47.38
SCE1572_2117	62.68	105.05
SCE1572_4941	15.33	23.92
SCE1572_8030	61.42	51.71
SCE1572_6903	51.13	12.09
SCE1572_4896	5.93	4.08
SCE1572_7270	118.57	95.13
SCE1572_7226	40.89	149.66
SCE1572_6720	5434.99	20079.31
SCE1572_9067	26.33	17.48
SCE1572_8853	5.83	3.51
SCE1572_3409	30.98	18.31
SCE1572_4072	50.16	134.85
SCE1572_1230	31.96	39.31
SCE1572_9833	31.07	10.32
SCE1572_4537	24.51	10.34
SCE1572_5721	66.41	68.36
SCE1572_11218	30.09	22.5
SCE1572_4782	59.03	47.86
SCE1572_10205	60.04	14.7
SCE1572_5110	34.42	311.44

SCE1572_4953	41.05	25.11
SCE1572_4499	57.15	43.94
SCE1572_5453	44.98	14.6
SCE1572_1383	0	0
SCE1572_10128	2.08	0
SCE1572_3035	15.05	6.19
SCE1572_3385	39.14	40.74
SCE1572_2578	22.33	4.09
SCE1572_8254	37.3	1859.59
SCE1572_10620	28.12	22.56
SCE1572_4986	16.9	43.07
SCE1572_6819	33.41	4.64
SCE1572_7748	11.73	1.13
SCE1572_1165	41.5	46.29
SCE1572_6381	24.19	139.76
SCE1572_3296	0	0
SCE1572_5450	46.61	44.38
SCE1572_7191	183.26	425.21
SCE1572_2557	58.89	28.13
SCE1572_3615	43.49	14.14
SCE1572_10857	105.29	66.69
SCE1572_4117	14.63	2131.91
SCE1572_6867	191.38	182.46
SCE1572_8380	17.84	18.4
SCE1572_1761	61.48	16.82
SCE1572_10234	15.04	0
SCE1572_10232	58.31	12.96
SCE1572_1579	23.18	25.57
SCE1572_5315	21.94	33.61
SCE1572_4151	27.96	251.34
SCE1572_3657	13.24	14.29
SCE1572_10615	54.18	32.83
SCE1572_1341	54.93	342.45
SCE1572_8243	113.07	261.01
SCE1572_7414	10.29	1.65
SCE1572_10911	3.92	0
SCE1572_5225	204.22	610.69
SCE1572_2396	9.83	25.64
SCE1572_4400	18.31	9.04
SCE1572_8842	38.6	55.97
SCE1572_10419	88.97	43.7
SCE1572_7959	14.01	24.53
SCE1572_1153	43.96	133.98
SCE1572_5336	25.93	16.01
SCE1572_4719	28.43	9.02
SCE1572_6586	241.25	55.08
SCE1572_7897	17.56	38.41
SCE1572_11158	50.28	47.83

SCE1572_3884	38.88	14.04
SCE1572_8080	25.54	3.84
SCE1572_3106	56.57	103.49
SCE1572_8814	23.07	11.38
SCE1572_5302	48.38	12.48
SCE1572_8654	411.33	2273.81
SCE1572_10843	19.25	0
SCE1572_7605	39.96	22.63
SCE1572_1593	45.39	83.68
SCE1572_11127	43.76	29.67
SCE1572_6369	43.48	42.04
SCE1572_741	17.2	27.23
SCE1572_1749	41.06	32.31
SCE1572_11146	35.84	75.75
SCE1572_6097	32.07	54.39
SCE1572_7486	18.42	14.43
SCE1572_10655	77.86	601.63
SCE1572_4816	2.61	0
SCE1572_2034	64.65	113.42
SCE1572_1214	21.41	12.37
SCE1572_10804	56.12	20.16
SCE1572_4252	62.5	22.29
SCE1572_11597	68.88	813.26
SCE1572_6887	16.41	3.76
SCE1572_7811	18.44	4.93
SCE1572_1359	61.74	193.19
SCE1572_10714	37.78	10.36
SCE1572_5251	65.33	273.36
SCE1572_7395	21.56	3.46
SCE1572_587	28.34	12.87
SCE1572_6808	97.31	380.45
SCE1572_2363	20.07	10.73
SCE1572_9844	13.24	28.96
SCE1572_10526	24.72	11.9
SCE1572_9342	211.46	2837.75
SCE1572_4146	25.94	9.76
SCE1572_7281	14.52	67.11
SCE1572_4372	49.84	18.75
SCE1572_814	16.4	6.4
SCE1572_5025	23.6	16.71
SCE1572_1083	31.13	4.83
SCE1572_6457	23.66	12.27
SCE1572_3233	31.11	20.49
SCE1572_671	23.92	79.72
SCE1572_4379	16.24	6.17
SCE1572_10683	88.3	196.98
SCE1572_1027	29.71	746.96
SCE1572_1631	39.24	8.59

SCE1572_8958	22	39.82
SCE1572_7873	75.81	264.33
SCE1572_233	14.36	34.57
SCE1572_10045	590.27	6756.38
SCE1572_344	59.95	18.04
SCE1572_5842	65.03	17.92
SCE1572_5402	31.45	20.34
SCE1572_10627	38.24	122.28
SCE1572_1	78.06	479.35
SCE1572_5460	25.62	1.26
SCE1572_5738	178.92	188.51
SCE1572_5815	13	13.21
SCE1572_6362	114.77	84.32
SCE1572_247	92.48	22.52
SCE1572_2757	32.34	20.42
SCE1572_8113	32.41	9.75
SCE1572_6567	32.64	57.67
SCE1572_3516	20.66	84.43
SCE1572_10268	42.08	28.88
SCE1572_4095	24.93	20.61
SCE1572_6711	55.01	96.88
SCE1572_7319	30.99	40.32
SCE1572_8996	22.93	32.49
SCE1572_10202	78.2	30.23
SCE1572_4229	37.35	25.9
SCE1572_10113	21.02	45.77
SCE1572_3289	44.63	26.07
SCE1572_5800	55.13	154.08
SCE1572_2827	32.9	6.89
SCE1572_10116	25.56	49.5
SCE1572_8211	49.29	63.61
SCE1572_6952	45.21	11.72
SCE1572_1962	54.18	20.22
SCE1572_9390	33.3	61.39
SCE1572_960	52.66	96.15
SCE1572_10946	51.87	23.63
SCE1572_2999	13.86	5.89
SCE1572_1927	30.61	57.39
SCE1572_9035	25.99	12.74
SCE1572_10724	0	0
SCE1572_4058	16.13	10.35
SCE1572_6655	24.99	12.3
SCE1572_438	0	0
SCE1572_5849	22.07	4.62
SCE1572_6601	100.27	115.95
SCE1572_8322	24.83	91.23
SCE1572_10662	44.4	34
SCE1572_10127	12.03	5.79

SCE1572_3361	22.58	0
SCE1572_5931	11.76	6.53
SCE1572_7120	60.96	32.95
SCE1572_8914	22.13	7.99
SCE1572_7213	112.71	16.15
SCE1572_5808	56.18	23.15
SCE1572_1738	32.49	0
SCE1572_1178	43.75	16.68
SCE1572_5531	55.79	0
SCE1572_3085	0	0
SCE1572_4189	26.14	63.28
SCE1572_1290	22.64	16.45
SCE1572_6503	6.85	0
SCE1572_7966	34.33	15.49
SCE1572_7867	11.73	91.23
SCE1572_10986	743.08	13966.3
SCE1572_8302	99.79	282.65
SCE1572_712	5.36	1.84
SCE1572_1371	69.58	63.46
SCE1572_10014	17.8	6.9
SCE1572_8836	0	0
SCE1572_4135	31.84	10.95
SCE1572_9308	351.96	2140.32
SCE1572_7860	61.5	92.95
SCE1572_11530	47.38	28.17
SCE1572_1168	86.01	38.63
SCE1572_3268	127.94	1735.08
SCE1572_5142	71.15	24.57
SCE1572_954	4.36	2.63
SCE1572_6321	6.62	6.67
SCE1572_9892	0	0
SCE1572_8065	140.33	32.25
SCE1572_8022	22.81	25.84
SCE1572_1397	40.05	77.46
SCE1572_6727	63.14	137.49
SCE1572_946	55.84	19.09
SCE1572_2467	37.38	27.75
SCE1572_1047	50.72	16.7
SCE1572_4000	24.5	51.84
SCE1572_7773	88.75	133.51
SCE1572_2072	27.61	12.82
SCE1572_6390	56.2	142.31
SCE1572_1177	47.09	48.57
SCE1572_8664	41.12	8.25
SCE1572_5863	29.48	16.69
SCE1572_10634	32.74	77.59
SCE1572_3028	0	0
SCE1572_1678	13.93	36.75

SCE1572_376	32.34	11.68
SCE1572_4086	45.87	23.39
SCE1572_4789	40.95	355.16
SCE1572_9083	10.67	0.89
SCE1572_8181	27	5
SCE1572_94	180.22	130.82
SCE1572_8595	0	0
SCE1572_4562	75.81	98.53
SCE1572_6368	23.87	139.98
SCE1572_7514	0	0
SCE1572_709	0	0
SCE1572_10710	21.95	10
SCE1572_3553	3.37	0
SCE1572_253	52.15	20.92
SCE1572_8944	9.78	0
SCE1572_6674	13.9	24.33
SCE1572_6736	60.55	312.13
SCE1572_2448	14.8	7.7
SCE1572_11291	125.25	105.77
SCE1572_5059	54.63	75.13
SCE1572_3156	16.71	16.68
SCE1572_1016	0	0
SCE1572_10518	45.57	28.36
SCE1572_7907	224.67	1334.9
SCE1572_1586	26.82	46.35
SCE1572_10977	115.94	182.46
SCE1572_6973	3.09	0
SCE1572_9853	76.43	29.83
SCE1572_1932	69.88	19.79
SCE1572_9952	9.97	8
SCE1572_5881	51.04	45.5
SCE1572_5954	39	27.26
SCE1572_110	100.98	331.62
SCE1572_6687	11.15	18.78
SCE1572_8903	56.1	11.86
SCE1572_5683	101.19	315.56
SCE1572_8061	6.5	0
SCE1572_2297	29.8	109.16
SCE1572_9316	29.77	65.05
SCE1572_7818	15.37	19.14
SCE1572_9386	7.16	9.4
SCE1572_4554	29.06	6.08
SCE1572_2012	39.76	17.88
SCE1572_3431	172.24	451.81
SCE1572_8225	63.79	33.38
SCE1572_9164	53.91	16.22
SCE1572_3644	44.12	22.2
SCE1572_966	28.46	53.54

SCE1572_9205	31.4	36.86
SCE1572_2462	29.31	45.41
SCE1572_5733	104.04	870.41
SCE1572_636	151.27	443.3
SCE1572_10837	37.16	56.54
SCE1572_8783	45.07	264.02
SCE1572_5757	59.86	64.91
SCE1572_5085	0	0
SCE1572_9612	8.42	0
SCE1572_5009	256.41	397.12
SCE1572_5052	34.94	19.72
SCE1572_6416	34.75	7.6
SCE1572_2625	0	0
SCE1572_5034	39.51	13.27
SCE1572_7625	57.32	98.87
SCE1572_3253	26.1	19.44
SCE1572_8099	21.2	176.7
SCE1572_4840	58.67	436.32
SCE1572_431	44.38	26.7
SCE1572_4065	22.45	11.77
SCE1572_10086	294.71	1283.38
SCE1572_8010	45.01	245.18
SCE1572_7807	220.41	173.2
SCE1572_4797	70.36	244.47
SCE1572_10784	50.13	51.11
SCE1572_3402	24.56	92.52
SCE1572_10601	31.1	10.92
SCE1572_6016	153.25	459.1
SCE1572_6822	131.67	1569.17
SCE1572_656	642.29	1382.7
SCE1572_8398	52.6	32.02
SCE1572_7062	0	0
SCE1572_9868	33.55	71.79

TableS3 Transcripts detected under different pH conditions(Original reads)

Locus tag	pH 7.0	pH 10.0
SCE1572_5566	240	147
SCE1572_4403	41	59
SCE1572_3237	0	0
SCE1572_7422	4	0
SCE1572_6423	271	39
SCE1572_5465	106	15
SCE1572_5888	12	71
SCE1572_1430	56	6
SCE1572_9058	138	23
SCE1572_8947	128	80
SCE1572_1415	20	2
SCE1572_9953	382	94
SCE1572_8357	17	1
SCE1572_5736	69	305
SCE1572_963	781	264
SCE1572_9431	52	21
SCE1572_6979	32	6
SCE1572_5559	0	0
SCE1572_1203	181	30
SCE1572_926	121	26
SCE1572_6927	127	84
SCE1572_4079	19	0
SCE1572_1922	93	18
SCE1572_10118	124	218
SCE1572_8912	9	0
SCE1572_7208	33	4
SCE1572_6891	31	8
SCE1572_10120	291	520
SCE1572_8105	0	0
SCE1572_5207	82	116
SCE1572_4631	207	39
SCE1572_211	98	19
SCE1572_9167	42	1
SCE1572_3914	122	15
SCE1572_7224	167	116
SCE1572_8249	132	82
SCE1572_8904	63	2
SCE1572_5794	209	181
SCE1572_3433	38	108
SCE1572_4684	328	135
SCE1572_9858	13	0
SCE1572_9695	0	0
SCE1572_1899	66	9
SCE1572_1005	294.01	34.85
SCE1572_7497	0	0
SCE1572_5550	106	12

SCE1572_10517	69	14
SCE1572_5396	73	15
SCE1572_11501	5	0
SCE1572_3334	0	0
SCE1572_10265	50	6
SCE1572_11111	256	63
SCE1572_6453	0	0
SCE1572_816	196	27
SCE1572_2624	295	49
SCE1572_6657	415	405
SCE1572_1590	118	46
SCE1572_4855	39	4
SCE1572_8348	261	92
SCE1572_4967	60	207
SCE1572_7617	234	512
SCE1572_8843	94	23
SCE1572_2650	35	10
SCE1572_1513	73	3
SCE1572_10034	220	64
SCE1572_9206	227	65
SCE1572_1573	89	23
SCE1572_9449	143	36
SCE1572_3515	27	41
SCE1572_7608	1	0
SCE1572_6365	108	500
SCE1572_7454	96	75
SCE1572_3975	16	8
SCE1572_3071	281	69
SCE1572_3042	104	10
SCE1572_5691	100	17
SCE1572_1404	145	11
SCE1572_49	53	4
SCE1572_8002	205.3	19.5
SCE1572_2278	69	190
SCE1572_4137	0	0
SCE1572_1345	158	478
SCE1572_8495	168	36
SCE1572_5018	307	179
SCE1572_830	39	18
SCE1572_187	62	1142
SCE1572_6610	17	8
SCE1572_4114	47	28
SCE1572_306	62	13
SCE1572_7613	50	230
SCE1572_1606	231	28
SCE1572_10395	15	1
SCE1572_9894	3	0
SCE1572_9491	0	0

SCE1572_11230	347	156
SCE1572_8955	1	0
SCE1572_6436	75	14
SCE1572_5448	88	15
SCE1572_3105	133	81
SCE1572_1366	194	221
SCE1572_8601	46	60
SCE1572_4069	0	0
SCE1572_5880	0	0
SCE1572_8037	0	0
SCE1572_6726	36	15
SCE1572_3543	0	0
SCE1572_7367	416	4999
SCE1572_248	223	16
SCE1572_10623	368.73	178.08
SCE1572_9768	64	160
SCE1572_10652	23	54
SCE1572_7802	72	197
SCE1572_5985	38	5
SCE1572_9841	0	0
SCE1572_9963	601.01	74.26
SCE1572_7577	17	2
SCE1572_10612	115	76
SCE1572_7663	19	2
SCE1572_11224	83.68	22
SCE1572_9408	115	37
SCE1572_4376	18	2
SCE1572_9362	170	434
SCE1572_5731	8	58
SCE1572_10286	263	823
SCE1572_7845	411	515
SCE1572_4842	23	72
SCE1572_8798	141	13
SCE1572_7521	45	10
SCE1572_2463	106.9	12.38
SCE1572_7211	115	39
SCE1572_4203	115	234
SCE1572_782	24	10
SCE1572_5176	54	17
SCE1572_1742	178	74
SCE1572_11206	220	104
SCE1572_9567	1	0
SCE1572_2193	28	2
SCE1572_3990	355.64	259.98
SCE1572_2460	18	0
SCE1572_5137	18	10
SCE1572_4098	8	2
SCE1572_282	6	1

SCE1572_88	107	93
SCE1572_8438	31	9
SCE1572_6296	69	141
SCE1572_1380	641	67
SCE1572_2438	45	68
SCE1572_2504	25	8
SCE1572_6900	6	3
SCE1572_2791	252	201
SCE1572_80	145	161
SCE1572_1472	236	73
SCE1572_9104	245	46
SCE1572_1624	78	19
SCE1572_3362	96	50
SCE1572_2168	1	0
SCE1572_7856	205	34
SCE1572_4773	47	17
SCE1572_754	85	47
SCE1572_6951	84	17
SCE1572_10444	252	219
SCE1572_9667	84	199
SCE1572_10984	359	1033
SCE1572_8110	2	0
SCE1572_5079	34	34
SCE1572_8041	63	7
SCE1572_2242	135	48
SCE1572_1372	52	217
SCE1572_8433	5	2
SCE1572_5813	133	55
SCE1572_700	1	0
SCE1572_2010	82	9
SCE1572_11149	6	5
SCE1572_10976	104	0
SCE1572_7217	119	62
SCE1572_5719	39	7
SCE1572_11292	215	651
SCE1572_11053	359	466
SCE1572_6650	60	25
SCE1572_8277	63	19
SCE1572_9473	120	111
SCE1572_3550	95	25
SCE1572_5232	35	34
SCE1572_10529	94	58
SCE1572_9606	5	0
SCE1572_7979	374	46
SCE1572_8021	47	8
SCE1572_7030	27	5
SCE1572_4972	34	75
SCE1572_7997	77.79	3.09

SCE1572_3219	124	653
SCE1572_180	0	0
SCE1572_1340	48	21
SCE1572_9934	0	0
SCE1572_7895	1	0
SCE1572_7063	290	51
SCE1572_1569	58	45
SCE1572_8410	169	500
SCE1572_1140	31	18
SCE1572_11212	65	18
SCE1572_5996	127	38
SCE1572_4989	33	27
SCE1572_8108	186	20
SCE1572_2995	179	22
SCE1572_9626	87.06	10.38
SCE1572_525	62	37
SCE1572_8183	111	6
SCE1572_4091	84	16
SCE1572_5859	96	24
SCE1572_8465	3	0
SCE1572_1858	46	4
SCE1572_745	369	650
SCE1572_739	93	17
SCE1572_3507	33	8
SCE1572_6096	480	107
SCE1572_4152	36	838
SCE1572_1557	3	0
SCE1572_1102	55	13
SCE1572_8406	199	63
SCE1572_10274	113	69
SCE1572_5523	68	14
SCE1572_1752	26	4
SCE1572_7397	70	17
SCE1572_6604	101	100
SCE1572_520	47	9
SCE1572_3861	578	814
SCE1572_5452	225	27
SCE1572_1479	107	30
SCE1572_4183	17	5
SCE1572_4277	274	54
SCE1572_4055	84	108
SCE1572_7277	0	0
SCE1572_273	415	501
SCE1572_4726	157	35
SCE1572_4367	369	76
SCE1572_10239	174	18
SCE1572_5862	222	31
SCE1572_5269	229	446

SCE1572_5003	38	95
SCE1572_8270	129	35
SCE1572_5742	344.91	113.79
SCE1572_5688	54	11
SCE1572_6380	37	11
SCE1572_4251	11	5
SCE1572_7555	17	2
SCE1572_4451	0	0
SCE1572_3700	125	47
SCE1572_11134	114	24
SCE1572_5330	62	9
SCE1572_11	55	44
SCE1572_6894	213.43	66
SCE1572_2819	172	33
SCE1572_10713	25	9
SCE1572_10510	67	19
SCE1572_1136	0	0
SCE1572_1154	12.85	0
SCE1572_9848	13	1
SCE1572_588	16	7
SCE1572_2092	71	5
SCE1572_9341	131	529
SCE1572_6101	189	28
SCE1572_4552	98	14
SCE1572_2039	16	31
SCE1572_1691	158	157
SCE1572_9088	9	0
SCE1572_10912	143	145
SCE1572_3654	54	122
SCE1572_839	12	16
SCE1572_3022	37	4
SCE1572_5237	230	157
SCE1572_1358	661	2228
SCE1572_6327	8	1
SCE1572_10525	71	31
SCE1572_3380	22	11
SCE1572_10676	151	52
SCE1572_4713	102	22
SCE1572_3785	120	123
SCE1572_5601	230	38
SCE1572_973	60	56
SCE1572_8005	99	58
SCE1572_10203	46.55	61.05
SCE1572_6265	99	72
SCE1572_8051	72	10
SCE1572_6388	57	134
SCE1572_1686	34	7
SCE1572_5026	45	15

SCE1572_5838	31	5
SCE1572_10160	641	428
SCE1572_405	224	102
SCE1572_6510	0	0
SCE1572_4060	16	32
SCE1572_1251	152	29
SCE1572_8928	0	0
SCE1572_10836	75	16
SCE1572_8643	65	31
SCE1572_7375	22	6
SCE1572_335	131	27
SCE1572_5510	63	28
SCE1572_4130	188	127
SCE1572_4475	4416	3508
SCE1572_9498	252	677
SCE1572_2046	24	0
SCE1572_11302	93	53
SCE1572_5889	30	26
SCE1572_8859	67	20
SCE1572_5112	54	42
SCE1572_4954	12	0
SCE1572_6232	214	24
SCE1572_295	5	67
SCE1572_1892	59	25
SCE1572_5495	121	18
SCE1572_5462	77	14
SCE1572_9142	31.38	5
SCE1572_8918	30	184
SCE1572_3926	152	308
SCE1572_364	30	1
SCE1572_3150	51	75
SCE1572_995	66	5
SCE1572_8751	585	76
SCE1572_1718	101	64
SCE1572_4925	144	123
SCE1572_4641	143	30
SCE1572_11128	0	0
SCE1572_1355	40	23
SCE1572_6588	480	197
SCE1572_6193	0	0
SCE1572_4588	66	235
SCE1572_2258	184	34
SCE1572_2030	64	6
SCE1572_6967	160	42
SCE1572_9748	70	21
SCE1572_6884	153	26
SCE1572_3659	18	9
SCE1572_1935	0	0

SCE1572_5064	159	105
SCE1572_1118	429	89
SCE1572_11174	300	78
SCE1572_8966	160	94
SCE1572_2390	346	367
SCE1572_4929	57	50
SCE1572_10200	27	25
SCE1572_6628	90	97
SCE1572_4486	48	159
SCE1572_8117	23	3
SCE1572_8212	5	4
SCE1572_8911	31	11
SCE1572_3389	38	9
SCE1572_886	3461	1960
SCE1572_9295	185	157
SCE1572_8415	114	25
SCE1572_3294	19	3
SCE1572_146	216	39
SCE1572_4930	78	38
SCE1572_7638	43	22
SCE1572_7202	49	2
SCE1572_3557	150	23
SCE1572_8921	0.5	0
SCE1572_8562	327	294
SCE1572_6723	104	148
SCE1572_5457	113	15
SCE1572_4784	4	0
SCE1572_4677	8	6
SCE1572_1188	73	8
SCE1572_5655	142	40
SCE1572_7814	586	1924
SCE1572_11271	121	158
SCE1572_1595	63	29
SCE1572_474	154	298
SCE1572_5185	163	2756
SCE1572_3746	30	6
SCE1572_4708	0	0
SCE1572_7138	92	69
SCE1572_5293	44	31
SCE1572_4193	1036	2007
SCE1572_3754	20	1
SCE1572_3251	4	0
SCE1572_11044	51	28
SCE1572_6377	45	12
SCE1572_7353	269.08	30
SCE1572_11193	49	11
SCE1572_11325	257	109
SCE1572_6854	81	2

SCE1572_2692	28	31
SCE1572_6470	54	9
SCE1572_2699	1	0
SCE1572_9192	145	35
SCE1572_9930	123	95
SCE1572_4230	16	8
SCE1572_7287	22	14
SCE1572_8364	180	41
SCE1572_2466	50	7
SCE1572_5680	713	593
SCE1572_8608	296	463
SCE1572_6164	94	26
SCE1572_2371	182	120
SCE1572_6829	2	0
SCE1572_6398	52	81
SCE1572_7344	0	0
SCE1572_2395	76	116
SCE1572_2310	156	236
SCE1572_1423	481	131
SCE1572_6673	38	13
SCE1572_6462	220	93
SCE1572_11167	32	49
SCE1572_11454	293	243
SCE1572_8786	40	165
SCE1572_6986	0	0
SCE1572_3877	0	0
SCE1572_6944	2	0
SCE1572_7420	184	54
SCE1572_9276	207	75
SCE1572_2560	7	4
SCE1572_7449	561	915
SCE1572_10805	144	19
SCE1572_9214	217	51
SCE1572_76	28	0
SCE1572_10657	128	48
SCE1572_9705	47	8
SCE1572_1218	189	25
SCE1572_6972	201	34
SCE1572_2301	7	19
SCE1572_10659	91	45
SCE1572_5475	35	50
SCE1572_3148	73	10
SCE1572_7037	78	72
SCE1572_2635	82	76
SCE1572_9712	0	0
SCE1572_327	2	0
SCE1572_10834	252	25
SCE1572_1000	512	406

SCE1572_8502	238	36
SCE1572_2544	340	60
SCE1572_7769	24	9
SCE1572_4048	127	131
SCE1572_10216	48	9
SCE1572_9534	136	42
SCE1572_2376	274	191
SCE1572_11510	70	15
SCE1572_847	901	1901
SCE1572_6631	1	0
SCE1572_8509	271.07	56
SCE1572_7626	71	12
SCE1572_4490	58	12
SCE1572_581	92	40
SCE1572_485	114	19
SCE1572_1261	85	49
SCE1572_4297	0	0
SCE1572_6486	10	0
SCE1572_2598	0	0
SCE1572_1028	1	0
SCE1572_122	50	7
SCE1572_5213	133	29
SCE1572_387	65	69
SCE1572_3452	201	26
SCE1572_4140	44	43
SCE1572_5841	130	23
SCE1572_132	1586	1185
SCE1572_10225	4	0
SCE1572_6014	29	505
SCE1572_6912	119	10
SCE1572_5620	242.01	29
SCE1572_11342	54	28
SCE1572_10406	177	91
SCE1572_6694	118	21
SCE1572_7840	114	57
SCE1572_10968	51	18
SCE1572_10605	0	0
SCE1572_9668	164	407
SCE1572_9334	79	106
SCE1572_9444	105	33
SCE1572_422	69	79
SCE1572_576	215	374
SCE1572_8155	74	6
SCE1572_3533	210	27
SCE1572_2657	492	67
SCE1572_7433	61	51
SCE1572_5916	198	61
SCE1572_6281	299.53	271

SCE1572_11368	3	0
SCE1572_9369	0	0
SCE1572_7400	131	53
SCE1572_7108	0	0
SCE1572_5275	2	1
SCE1572_10825	224	128
SCE1572_6075	219	86
SCE1572_2478	134	61
SCE1572_6270	32	13
SCE1572_3199	162	425
SCE1572_9190	77	17
SCE1572_6401	0	0
SCE1572_4153	0	0
SCE1572_4285	101	40
SCE1572_3442	128	53
SCE1572_4159	25	17
SCE1572_9908	181	74
SCE1572_7128	292	74
SCE1572_10661	110	69
SCE1572_4343	195	28
SCE1572_3613	62	64
SCE1572_2215	54	39
SCE1572_2433	27	13
SCE1572_5403	103	17
SCE1572_1865	54	9
SCE1572_9881	118	50
SCE1572_941	57	3
SCE1572_6312	51	293
SCE1572_7884	123	30
SCE1572_11309	48	3
SCE1572_3284	36	2
SCE1572_4383	165	28
SCE1572_9118	167	44
SCE1572_5972	23	0
SCE1572_5898	7	2
SCE1572_5586	18	0
SCE1572_5391	253	91
SCE1572_4579	1	0
SCE1572_7151	91	39
SCE1572_1458	0	0
SCE1572_3670	40.3	6
SCE1572_11379	50	48
SCE1572_6415	68	10
SCE1572_4236	102	164
SCE1572_10117	67	43
SCE1572_10591	256	71
SCE1572_9516	28	54
SCE1572_98	23	6

SCE1572_1098	237	61
SCE1572_3177	142	253
SCE1572_1123	86	11
SCE1572_10692	23	41
SCE1572_10577	32	10
SCE1572_4083	39	24
SCE1572_2595	215	66
SCE1572_7586	54	446
SCE1572_2893	43	7
SCE1572_6222	108	18
SCE1572_4600	78	54
SCE1572_643	268	264
SCE1572_2336	55	29
SCE1572_8892	155	77
SCE1572_7056	16	7
SCE1572_3741	109	35
SCE1572_9871	34	7
SCE1572_5596	212	87
SCE1572_5514	100	8
SCE1572_5978	111	29
SCE1572_2864	0	0
SCE1572_2177	98	6
SCE1572_4654	122.99	77.63
SCE1572_9307	126	159
SCE1572_6432	38	5
SCE1572_7483	382	108
SCE1572_4469	369	63
SCE1572_8401	207	59
SCE1572_4848	74	22
SCE1572_6871	41	15
SCE1572_1739	55	4
SCE1572_2453	170	63
SCE1572_9263	12	10
SCE1572_4939	207	148
SCE1572_8763	0	0
SCE1572_3449	120	17
SCE1572_10729	36	26
SCE1572_2535	20	9
SCE1572_9795	31	1
SCE1572_5717	265	199
SCE1572_10197	0	0
SCE1572_1190	348	176
SCE1572_10580	24	11
SCE1572_10640	583	1495
SCE1572_4935	120	224
SCE1572_10865	670	703
SCE1572_5438	0	0
SCE1572_5141	159	42

SCE1572_8764	0.15	0.18
SCE1572_7461	1094	1489
SCE1572_9651	107	14
SCE1572_289	651	44
SCE1572_4033	0	0
SCE1572_2921	263	27
SCE1572_9552	37	7
SCE1572_9434	324	522
SCE1572_2977	76.95	11
SCE1572_10464	27	43
SCE1572_7671	117	3
SCE1572_10650	1022	811
SCE1572_6239	0	0
SCE1572_9900	423	596
SCE1572_9241	0	0
SCE1572_8166	3	0
SCE1572_3264	84	311
SCE1572_10436	78	47
SCE1572_213	135	23
SCE1572_4017	30	5
SCE1572_5956	54	15
SCE1572_10495	130	124
SCE1572_8129	0	0
SCE1572_8633	97	51
SCE1572_5723	3	0
SCE1572_6662	212	81
SCE1572_1224	86	37
SCE1572_9736	105	8
SCE1572_7762	57	86
SCE1572_8826	148.64	15.44
SCE1572_3128	32	24
SCE1572_7086	51	7
SCE1572_3479	16	0
SCE1572_5942	103	15
SCE1572_224	62	19
SCE1572_4323	76	14
SCE1572_1220	58	13
SCE1572_1227	109	17
SCE1572_8933	24	8
SCE1572_2813	44	22
SCE1572_7724	133	86
SCE1572_11578	18	3
SCE1572_10845	131	29
SCE1572_1377	41	7
SCE1572_11406	37	5
SCE1572_5097	46	22
SCE1572_4441	67	13
SCE1572_2801	143	564

SCE1572_10786	42	11
SCE1572_10241	37	8
SCE1572_9923	155	211
SCE1572_5	179	255
SCE1572_25	94	47
SCE1572_2025	102	95
SCE1572_567	132	65
SCE1572_3812	10	15
SCE1572_490	86.43	96.62
SCE1572_4841	220	649
SCE1572_7331	1	0
SCE1572_9992	0	0
SCE1572_9282	1813	791
SCE1572_469	164	656
SCE1572_2613	49	10
SCE1572_460	7	0
SCE1572_2075	70	5
SCE1572_5525	292	57
SCE1572_4430	15	4
SCE1572_373	56	21
SCE1572_9389	105	201
SCE1572_3631	44	12
SCE1572_6881	318	75
SCE1572_5253	0	0
SCE1572_6575	13	5
SCE1572_3030	61	9
SCE1572_2760	142	337
SCE1572_10053	46	60
SCE1572_9017	0	0
SCE1572_824	318	80
SCE1572_3986	206	1275
SCE1572_8613	61	11
SCE1572_4293	127	38
SCE1572_4008	55	9
SCE1572_11548	109	175
SCE1572_1170	122	22
SCE1572_7849	793	279
SCE1572_8997	50	15
SCE1572_516	0	0
SCE1572_8182	102	12
SCE1572_5534	406	58
SCE1572_7759	96	217
SCE1572_241	489	138
SCE1572_8379	40	14
SCE1572_9703	33	16
SCE1572_3937	182	62
SCE1572_1898	56	29
SCE1572_8028	2	0

SCE1572_1564	29	21
SCE1572_5419	29	41
SCE1572_8802	554	167
SCE1572_4763	64	129
SCE1572_9370	30	8
SCE1572_6322	61	26
SCE1572_8818	76	16
SCE1572_8073	141	27
SCE1572_773	30	16
SCE1572_6206	157	49
SCE1572_5345	24	6
SCE1572_2267	67	46
SCE1572_7927	142	59
SCE1572_78	159	23
SCE1572_5801	98	125
SCE1572_6229	60	11
SCE1572_6481	23	1
SCE1572_4456	68	61
SCE1572_2928	122	19
SCE1572_8205	12	16
SCE1572_7876	62	21
SCE1572_8485	89	22
SCE1572_6370	64	18
SCE1572_2723	57	29
SCE1572_2517	0	0
SCE1572_8536	85.73	17.04
SCE1572_6642	107	20
SCE1572_4965	173	48
SCE1572_11461	1	0
SCE1572_7444	0	0
SCE1572_8708	44	30
SCE1572_9950	122	23
SCE1572_4168	27	2
SCE1572_9545	113	60
SCE1572_6509	37	5
SCE1572_6593	25	26
SCE1572_987	47	17
SCE1572_8569	83	7
SCE1572_7693	339	475
SCE1572_10000	72	139
SCE1572_2770	36	164
SCE1572_1249	8	3
SCE1572_4439	45	7
SCE1572_3673	155	74
SCE1572_5156	64	5
SCE1572_59	57	15
SCE1572_876	299	84
SCE1572_4609	211	17

SCE1572_10011	603	934
SCE1572_5369	85	11
SCE1572_1917	50	7
SCE1572_7121	0	0
SCE1572_42	122	43
SCE1572_6958	54	5
SCE1572_5592	145	48
SCE1572_1284	302	4066
SCE1572_10007	44	12
SCE1572_8972	84	4162
SCE1572_8899	2	0
SCE1572_6358	135	22
SCE1572_9234	508	326
SCE1572_4819	106	196
SCE1572_5354	35	21
SCE1572_10416	11	6
SCE1572_1169	127	24
SCE1572_5788	350	600
SCE1572_120	51	4
SCE1572_1282	30	46
SCE1572_8236	71	267
SCE1572_11492	151	172
SCE1572_8269	125	18
SCE1572_5201	31	10
SCE1572_799	159	308
SCE1572_2826	29	9
SCE1572_3346	520	157
SCE1572_9481	276	100
SCE1572_2713	69	72
SCE1572_7036	77	102
SCE1572_3277	0	0
SCE1572_10917	43	25
SCE1572_6636	306	35
SCE1572_3948	286	286
SCE1572_7775	27	4
SCE1572_10398	121	55
SCE1572_2997	14	1
SCE1572_7476	190	0
SCE1572_4514	261	322
SCE1572_7348	94	16
SCE1572_2571	138	60
SCE1572_6502	118	46
SCE1572_4056	62	61
SCE1572_1323	47	33
SCE1572_3575	148	21
SCE1572_10575	0	0
SCE1572_4412	2	0
SCE1572_7650	12	0

SCE1572_5117	25	25
SCE1572_9310	249	456
SCE1572_4918	126	54
SCE1572_1957	85	26
SCE1572_4696	153	39
SCE1572_1138	41	5
SCE1572_10372	86	14
SCE1572_6408	52	10
SCE1572_2206	108	387
SCE1572_10148	29	15
SCE1572_9399	88	30
SCE1572_4914	56	9
SCE1572_11408	25	6
SCE1572_9189	174	28
SCE1572_8985	45	40
SCE1572_6768	75	51
SCE1572_8635	32	9
SCE1572_6615	49	12
SCE1572_5614	66	7
SCE1572_2122	6	1
SCE1572_3903	258	161
SCE1572_1970	126	22
SCE1572_1791	60	5
SCE1572_11444	25	10
SCE1572_3957	3	0
SCE1572_889	113	14
SCE1572_10709	18	0
SCE1572_4321	331	70
SCE1572_8395	64	593
SCE1572_4561	0	0
SCE1572_4024	1042	380
SCE1572_3510	36	76
SCE1572_2330	218	62
SCE1572_1822	192	18
SCE1572_10500	57	275
SCE1572_6994	24	12
SCE1572_3299	92	40
SCE1572_10924	828	7715
SCE1572_10744	140	23
SCE1572_3069	51	47
SCE1572_6627	38	26
SCE1572_4993	48	12
SCE1572_611	18	37
SCE1572_2103	180	109
SCE1572_11032	0	0
SCE1572_6467	88	9
SCE1572_10694	25	0
SCE1572_1077	162	44

SCE1572_6139	120	105
SCE1572_9983	177	32
SCE1572_5634	68	10
SCE1572_8870	87	27
SCE1572_10928	180	76
SCE1572_7198	336	332
SCE1572_9413	68	109
SCE1572_3589	1	0
SCE1572_3272	0	0
SCE1572_1995	236	31
SCE1572_6788	190	29
SCE1572_3551	9	3
SCE1572_9010	39	7
SCE1572_4827	139	34
SCE1572_9692	25	41
SCE1572_6603	60	41
SCE1572_8519	21	120
SCE1572_4359	14	6
SCE1572_10827	74	14
SCE1572_7752	44	13
SCE1572_1771	152	30
SCE1572_5192	66	222
SCE1572_2369	33	3
SCE1572_6275	239.5	287.5
SCE1572_2706	430	120
SCE1572_10131	42	18
SCE1572_10380	119	44
SCE1572_437	110	25
SCE1572_2935	9	1
SCE1572_7502	57	39
SCE1572_5909	74	227
SCE1572_5347	51	12
SCE1572_9752	0	0
SCE1572_2677	13	88
SCE1572_9153	253	89
SCE1572_860	73	192
SCE1572_3992	23	50
SCE1572_6526	44	7
SCE1572_8624	19	0
SCE1572_8497	539	86
SCE1572_2587	5	1
SCE1572_11471	58	18
SCE1572_10550	21	7
SCE1572_11459	47	46
SCE1572_3224	68	3
SCE1572_2417	35	13
SCE1572_10811	0	0
SCE1572_3624	21	2

SCE1572_6114	138	10
SCE1572_6566	68	28
SCE1572_4572	114	12
SCE1572_3845	162	21
SCE1572_9723	39	7
SCE1572_4549	125	17
SCE1572_1068	21	0
SCE1572_2855	28.64	3.95
SCE1572_10383	45	10
SCE1572_2774	81	24
SCE1572_40	150	40
SCE1572_1615	261	478
SCE1572_11434	125	32
SCE1572_7786	40.16	24.01
SCE1572_7176	110	90
SCE1572_7737	18	12
SCE1572_7491	32	14
SCE1572_6429	46	7
SCE1572_9155	154	110
SCE1572_1426	382	363
SCE1572_3309	50	6
SCE1572_4542	0	0
SCE1572_7967	20	0
SCE1572_5083	84	121
SCE1572_3411	45	31
SCE1572_2829	45	4
SCE1572_1785	329.01	288
SCE1572_1643	728	97
SCE1572_5106	34	279
SCE1572_10944	52	22
SCE1572_3581	80	19
SCE1572_5770	1029	6335
SCE1572_501	96	18
SCE1572_3819	166	39
SCE1572_9092	252	70
SCE1572_7113	137	38
SCE1572_5629	7	0
SCE1572_8642	3	1
SCE1572_9878	39	6
SCE1572_9437	181	102
SCE1572_6134	32	18
SCE1572_2666	72	44
SCE1572_5645	4	1
SCE1572_7716	47	0
SCE1572_539	3	0
SCE1572_4215	1056	968
SCE1572_1827	17	2
SCE1572_982	101	36

SCE1572_6140	68	14
SCE1572_5646	1	0
SCE1572_11160	39	111
SCE1572_7025	1	0
SCE1572_4187	291	178
SCE1572_2404	62	191
SCE1572_7512	0	0
SCE1572_4799	238	112
SCE1572_10388	14	16
SCE1572_8742	59	26
SCE1572_6149	92	35
SCE1572_2468	77	57
SCE1572_862	164	243
SCE1572_5146	57	11
SCE1572_11073	4	87
SCE1572_2019	179	37
SCE1572_3084	72	9
SCE1572_8145	21	53
SCE1572_8884	55	2
SCE1572_11500	97	97
SCE1572_321	29	1
SCE1572_11313	168	979
SCE1572_8833	42	9
SCE1572_8663	80	14
SCE1572_3045	5	0
SCE1572_4864	56	10
SCE1572_4484	46	24
SCE1572_2547	69	31
SCE1572_1849	220	56
SCE1572_11387	368	277
SCE1572_11553	140	42
SCE1572_4266	27	12
SCE1572_3602	45	47
SCE1572_10342	48	32
SCE1572_9526	124	559
SCE1572_8053	28	5
SCE1572_9179	71	12
SCE1572_10934	102	21
SCE1572_8734	30	13
SCE1572_8332	216	91
SCE1572_563	39	43
SCE1572_1333	7	0
SCE1572_11083	7	7
SCE1572_6518	56	5
SCE1572_2733	31	33
SCE1572_1312	446	529
SCE1572_2020	41	25
SCE1572_10304	431	272

SCE1572_8446	10	2
SCE1572_4508	26	22
SCE1572_9464	66	15
SCE1572_752	14	0
SCE1572_8686	64	4
SCE1572_6742	91	8
SCE1572_47	44	7
SCE1572_8292	91	21
SCE1572_6316	0	0
SCE1572_2235	143	183
SCE1572_7169	191	98
SCE1572_7132	40	5
SCE1572_1526	985	6039
SCE1572_8534	129	230
SCE1572_8936	3	0
SCE1572_10531	6	0
SCE1572_2115	116	22
SCE1572_1740	29	2
SCE1572_7328	28	2
SCE1572_9071	624	660
SCE1572_4818	5	0
SCE1572_2697	140	25
SCE1572_2135	1	0
SCE1572_2683	114	338
SCE1572_1809	25	25
SCE1572_2323	61	88
SCE1572_3494	19	1
SCE1572_7260	254	1088
SCE1572_7377	172	109
SCE1572_8282	270	398
SCE1572_9596	26	547
SCE1572_3885	94	12
SCE1572_2068	14	4
SCE1572_2063	83	29
SCE1572_2101	69	108
SCE1572_7000	0	0
SCE1572_9688	439	344
SCE1572_4257	13	0
SCE1572_9028	81	103
SCE1572_11581	71	38
SCE1572_11232	55	13
SCE1572_2299	166	349
SCE1572_8339	97	36
SCE1572_8957	103	141
SCE1572_10186	82	78
SCE1572_2217	2	0
SCE1572_9384	31	9
SCE1572_8749	85	38

SCE1572_4612	48	3
SCE1572_3795	66	19
SCE1572_8487	5	1
SCE1572_9356	160	315
SCE1572_4354	47	13
SCE1572_10322	32	198
SCE1572_6052	807	455
SCE1572_3663	133	16
SCE1572_7624	152	98
SCE1572_4738	22	17
SCE1572_2493	305	76
SCE1572_10218	209	128
SCE1572_990	187	74
SCE1572_596	774	1237
SCE1572_11452	27	3
SCE1572_3857	66	72
SCE1572_2273	26	23
SCE1572_11099	57	22
SCE1572_4881	5	1
SCE1572_2555	55	11
SCE1572_263	1	0
SCE1572_3116	157	36
SCE1572_2747	68	32
SCE1572_4362	1	0
SCE1572_1105	112	27
SCE1572_805	316	113
SCE1572_3943	1797	1623
SCE1572_11155	131	110
SCE1572_11479	3	0
SCE1572_8504	47	6
SCE1572_6550	118	40
SCE1572_8775	125	30
SCE1572_9000	173	137
SCE1572_699	48	4
SCE1572_9009	0	0
SCE1572_7519	39	103
SCE1572_4311	164	31
SCE1572_4040	73	16
SCE1572_3193	618	270
SCE1572_2455	126	112
SCE1572_1948	44	929
SCE1572_11377	29	37
SCE1572_532	294	46
SCE1572_4876	150	42
SCE1572_5264	108	216
SCE1572_8573	6	0
SCE1572_3523	73	43
SCE1572_1730	20	13

SCE1572_1852	75	84
SCE1572_2762	0	0
SCE1572_5605	39	5
SCE1572_1993	29	32
SCE1572_3633	35	6
SCE1572_1121	133	27
SCE1572_3531	50	4
SCE1572_1777	213	29
SCE1572_1754	37	11
SCE1572_854	169	230
SCE1572_6001	129	157
SCE1572_103	91	12
SCE1572_6348	0	0
SCE1572_4341	76	38
SCE1572_7308	0	0
SCE1572_4593	107	73
SCE1572_2443	1	0
SCE1572_11592	21	5
SCE1572_10298	78	34
SCE1572_8690	49	4
SCE1572_2866	552	67
SCE1572_5506	60	8
SCE1572_446	67	14
SCE1572_3504	72	58
SCE1572_767	38	5
SCE1572_11077	11	49
SCE1572_9066	246	28
SCE1572_4106	35	9
SCE1572_8733	138	29
SCE1572_6437	130	17
SCE1572_2522	61	47
SCE1572_5702	97	64
SCE1572_4399	42	3
SCE1572_2004	7	0
SCE1572_5982	16	2
SCE1572_2280	137	36
SCE1572_5937	72	6
SCE1572_907	32	5
SCE1572_1019	0	0
SCE1572_4462	0	0
SCE1572_2914	1949.98	241.72
SCE1572_1026	10	185
SCE1572_6747	0	0
SCE1572_3715	256	154
SCE1572_10463	519.27	805.96
SCE1572_10456	109	9
SCE1572_7170	122	84
SCE1572_10867	36	10

SCE1572_6781	36	161
SCE1572_8271	55	83
SCE1572_7746	466	216
SCE1572_7501	140	337
SCE1572_9785	23	4
SCE1572_10379	0	0
SCE1572_7156	59	48
SCE1572_10564	371	63
SCE1572_4873	304	334
SCE1572_2476	87	9
SCE1572_3851	40	200
SCE1572_1722	311	126
SCE1572_10270	94	272
SCE1572_1863	92	11
SCE1572_9554	77	75
SCE1572_4161	0	0
SCE1572_1982	0	0
SCE1572_6044	7	73
SCE1572_5967	87	8
SCE1572_10300	92	219
SCE1572_7582	224	350
SCE1572_522	39	20
SCE1572_7473	176	201
SCE1572_8194	82	7
SCE1572_6049	65	151
SCE1572_2032	118.59	28.63
SCE1572_1920	51	23
SCE1572_5324	157	157
SCE1572_2426	568	513
SCE1572_6490	65	11
SCE1572_1929	56	49
SCE1572_3483	201	115
SCE1572_8518	39	113
SCE1572_3738	16	6
SCE1572_3246	428	566
SCE1572_11563	58	13
SCE1572_4036	32	1
SCE1572_11241	139	57
SCE1572_9560	107	35
SCE1572_933	287	245
SCE1572_2196	125	812
SCE1572_3350	59	14
SCE1572_7245	79	49
SCE1572_2355	48	24
SCE1572_3647	40	82
SCE1572_9304	185	211
SCE1572_218	28	39
SCE1572_1484	155	33

SCE1572_1038	15	2
SCE1572_8793	7	0
SCE1572_5488	189	18
SCE1572_36	106	76
SCE1572_5827	111.16	36.34
SCE1572_5049	51	39
SCE1572_7939	20	28
SCE1572_2833	27	63
SCE1572_3374	198	3148
SCE1572_114	33	3
SCE1572_4240	74	20
SCE1572_2970	19.16	13
SCE1572_6536	329	277
SCE1572_4634	222	36
SCE1572_1638	16	4
SCE1572_7781	6	0
SCE1572_137	70	18
SCE1572_6304	21	22
SCE1572_3829	106	369
SCE1572_2629	100.27	22
SCE1572_2532	56	151
SCE1572_8479	110	309
SCE1572_3863	123	22
SCE1572_9466	149	97
SCE1572_913	16	11
SCE1572_8869	95	20
SCE1572_174	44	9
SCE1572_7822	189	486
SCE1572_10818	170	77
SCE1572_158	295	848
SCE1572_8343	10	1
SCE1572_6081	15	0
SCE1572_3007	29	12
SCE1572_7142	151.9	88.93
SCE1572_8850	31	26
SCE1572_1499	19	2
SCE1572_4970	35	4
SCE1572_3033	285	170
SCE1572_11487	13.25	0.75
SCE1572_3895	29	22
SCE1572_1297	54	232
SCE1572_8394	277	230
SCE1572_832	7	2
SCE1572_6684	79	95
SCE1572_2646	0	0
SCE1572_7935	115	26
SCE1572_2880	15	12
SCE1572_737	89	26

SCE1572_11026	16	6
SCE1572_8584	0	0
SCE1572_9851	27	5
SCE1572_300	42	13
SCE1572_1462	9	0
SCE1572_9135	30	6
SCE1572_3843	3	0
SCE1572_9223	455	39
SCE1572_7790	0	0
SCE1572_4905	62	501
SCE1572_235	3	2
SCE1572_6411	31	10
SCE1572_346	201	33
SCE1572_3217	150	40
SCE1572_4895	57	15
SCE1572_5062	131	148
SCE1572_11600	15	1
SCE1572_1706	137	95
SCE1572_1442	101	18
SCE1572_1395	140	3707.96
SCE1572_1762	447.76	33.22
SCE1572_10141	17	25
SCE1572_7863	42	2
SCE1572_9319	9	1
SCE1572_4398	53	21
SCE1572_5775	212	81
SCE1572_11182	60	8
SCE1572_3597	0	0
SCE1572_333	77	37
SCE1572_3240	2	0
SCE1572_7435	103	8
SCE1572_3733	0	0
SCE1572_2577	115	22
SCE1572_6127	181	300
SCE1572_394	234	161
SCE1572_11023	200	265
SCE1572_6499	50	23
SCE1572_11516	37	8
SCE1572_8327	220	60
SCE1572_5040	88	145
SCE1572_7568	271	67
SCE1572_499	67	17
SCE1572_662	3466	4029
SCE1572_1570	126	97
SCE1572_4042	18	6
SCE1572_4667	0	0
SCE1572_5851	0	0
SCE1572_1904	5	0

SCE1572_5389	532	688
SCE1572_1111	64	4
SCE1572_8090	83	7
SCE1572_1869	71	183
SCE1572_6325	42	75
SCE1572_10884	40	14
SCE1572_2956	23	6
SCE1572_481	125	14
SCE1572_9264	130	79
SCE1572_6309	66	36
SCE1572_415	77	82
SCE1572_6452	399.88	68
SCE1572_2108	63	128
SCE1572_9106	81	4
SCE1572_8175	6	0
SCE1572_2383	51	22
SCE1572_11103	26	21
SCE1572_1043	31	32
SCE1572_9634	206	109
SCE1572_10138	0	0
SCE1572_10471	103	65
SCE1572_3076	110	42
SCE1572_10026	159	366
SCE1572_7314	95	18
SCE1572_5623	98	138
SCE1572_2961	217	709
SCE1572_9505	33	11
SCE1572_1966	132	445
SCE1572_9229	1	0
SCE1572_4801	61	61
SCE1572_8879	66	9
SCE1572_8308	688	827
SCE1572_1144	68	26
SCE1572_7834	1	0
SCE1572_9325	0	0
SCE1572_3132	41	12
SCE1572_476	80	91
SCE1572_6066	23	2
SCE1572_5036	5	4
SCE1572_8367	107	15
SCE1572_8457	302	221
SCE1572_6832	51	9
SCE1572_4530	45	6
SCE1572_8350	15.55	0
SCE1572_11485	714	367
SCE1572_670	114	151
SCE1572_10991	81	40
SCE1572_5285	16	11

SCE1572_7685	25	6
SCE1572_5289	25	2
SCE1572_9226	78	19
SCE1572_2887	16	1
SCE1572_3146	28	4
SCE1572_3317	260	727
SCE1572_1466	136	14
SCE1572_8346	193	30
SCE1572_1278	54	13
SCE1572_928	143	56
SCE1572_8133	81	6
SCE1572_7010	96.72	33
SCE1572_2785	25	11
SCE1572_784	58	6
SCE1572_6738	32	30
SCE1572_10751	325	54
SCE1572_8429	116	59
SCE1572_6844	88	63
SCE1572_5382	59	9
SCE1572_9078	51	2
SCE1572_7185	39	6
SCE1572_5166	9	4
SCE1572_2630	174.21	21.22
SCE1572_6459	75	6
SCE1572_11262	63	62
SCE1572_724	186	23
SCE1572_11189	956	140
SCE1572_7093	30	11
SCE1572_11120	15	5
SCE1572_5728	85	44
SCE1572_7075	33	22
SCE1572_1205	35	3
SCE1572_8470	106	52
SCE1572_1662	58	82
SCE1572_11042	571	57
SCE1572_2558	94	20
SCE1572_2157	67	86
SCE1572_9046	943	137
SCE1572_6447	5	4
SCE1572_6174	116	64
SCE1572_7598	18	39
SCE1572_11258	14	16
SCE1572_3463	189	54
SCE1572_10058	261	735
SCE1572_10900	50	89
SCE1572_11197	75	32
SCE1572_4979	24	62
SCE1572_10368	13	2

SCE1572_2806	174.74	29.43
SCE1572_7869	222	166
SCE1572_5991	96	49
SCE1572_11046	184	37
SCE1572_2227	104	140
SCE1572_6255	108	54
SCE1572_10762	129	53
SCE1572_6248	22	18
SCE1572_1766	25	2
SCE1572_5698	47.19	57.89
SCE1572_694	13	11
SCE1572_6195	58	6
SCE1572_9258	31	9
SCE1572_1671	140	821
SCE1572_1239	39	6
SCE1572_5092	0	0
SCE1572_10963	48	16
SCE1572_10065	162	457
SCE1572_11064	115.64	59.95
SCE1572_1796	103	106
SCE1572_9126	16	6
SCE1572_3562	48	10
SCE1572_5365	46	33
SCE1572_4529	44	10
SCE1572_605	46	48
SCE1572_11141	246	37
SCE1572_7402	20	797
SCE1572_7767	0	0
SCE1572_2584	183	15
SCE1572_2078	160	44
SCE1572_7591	211	51
SCE1572_6023	266	134
SCE1572_3685	34	3
SCE1572_1559	33	5
SCE1572_9817	56	22
SCE1572_6707	0	0
SCE1572_1271	117	27
SCE1572_10484	221	38
SCE1572_9989	66	17
SCE1572_10752	271	108
SCE1572_10872	339	92
SCE1572_2637	91	29
SCE1572_1652	15	0
SCE1572_11118	345	1628
SCE1572_11411	122	24
SCE1572_3424	167	34
SCE1572_3824	90	91
SCE1572_4838	143	47

SCE1572_4728	566	102
SCE1572_1550	50	12
SCE1572_1308	802	4743
SCE1572_1055	309	53
SCE1572_4302	84	21
SCE1572_425	99	549
SCE1572_9861	32	9
SCE1572_554	27	26
SCE1572_8554	272	374
SCE1572_4070	247	306
SCE1572_11060	26	17
SCE1572_1049	7	1
SCE1572_3183	38	23
SCE1572_8659	374	442
SCE1572_6233	163	66
SCE1572_3355	398	55
SCE1572_2909	62	22
SCE1572_7327	579	112
SCE1572_1199	0	0
SCE1572_7951	85	177
SCE1572_11350	0	0
SCE1572_4775	0	0
SCE1572_2341	29	20
SCE1572_6960	203	32
SCE1572_10535	143	480
SCE1572_9173	11	2
SCE1572_7303	74	24
SCE1572_3290	30	4
SCE1572_2562	108	43
SCE1572_6812	41	4
SCE1572_7294	47	39
SCE1572_6156	32	5
SCE1572_10423	40	67
SCE1572_1798	45	83
SCE1572_867	55	43
SCE1572_9426	62	25
SCE1572_618	202	641
SCE1572_4790	50	39
SCE1572_10478	140	63
SCE1572_1538	45	17
SCE1572_7321	104	12
SCE1572_5318	91	15
SCE1572_4426	133	1222
SCE1572_9961	69	6
SCE1572_3399	43	14
SCE1572_2271	59	23
SCE1572_3322	1	0
SCE1572_8670	13	46

SCE1572_2240	98	72
SCE1572_9774	162	142
SCE1572_6094	245	23
SCE1572_1081	29	8
SCE1572_10555	157	13
SCE1572_6107	104	16
SCE1572_2084	75	128
SCE1572_11575	71	190
SCE1572_3778	94	32
SCE1572_10625	75	15
SCE1572_6937	20	9
SCE1572_10455	68	12
SCE1572_9644	24	42
SCE1572_3000	356	52
SCE1572_2608	94	23
SCE1572_7957	534	1955
SCE1572_8526	71	54
SCE1572_5398	93	18
SCE1572_9133	5	1
SCE1572_4520	108	114
SCE1572_10802	89	15
SCE1572_8383	104	25
SCE1572_2984	209	61
SCE1572_9913	0	0
SCE1572_7941	151	178
SCE1572_11333	169	91
SCE1572_5311	63	27
SCE1572_5915	301	158
SCE1572_2604	21	7
SCE1572_11523	7	4
SCE1572_7730	37	3
SCE1572_5304	54	29
SCE1572_5342	16	6
SCE1572_1301	121	27
SCE1572_5180	73	1112
SCE1572_10879	158	209
SCE1572_11359	62	17
SCE1572_1659	65	25
SCE1572_2143	70	35
SCE1572_9587	143	126
SCE1572_5128	362	663
SCE1572_10672	17	9
SCE1572_11172	29	7
SCE1572_7183	85	16
SCE1572_884	216	99
SCE1572_10854	87	36
SCE1572_10047	44	52
SCE1572_11214	322	51

SCE1572_492	0	0
SCE1572_5377	94	16
SCE1572_10758	116	34
SCE1572_7893	487	7931
SCE1572_2937	296	1266
SCE1572_8412	268	122
SCE1572_7674	174.22	19
SCE1572_7385	78	15
SCE1572_2347	2583	11839
SCE1572_5239	20	13
SCE1572_9085	33	11
SCE1572_5234	163	963
SCE1572_4208	86	97
SCE1572_2254	63	4
SCE1572_10983	63	45
SCE1572_6349	183	93
SCE1572_154	90	54
SCE1572_5751	406	174
SCE1572_251	114.99	4.68
SCE1572_9002	192	87
SCE1572_10974	2	1
SCE1572_9471	10	3
SCE1572_10276	799	185
SCE1572_8275	158	121
SCE1572_107	72	50
SCE1572_2037	44	116
SCE1572_2027	0	0
SCE1572_9580	92	19
SCE1572_722	40.92	5
SCE1572_790	0	0
SCE1572_10678	85	183
SCE1572_9628	199.96	37
SCE1572_6344	139	52
SCE1572_4279	57	30
SCE1572_6372	0	0
SCE1572_7358	57	49
SCE1572_5332	444	206
SCE1572_8119	125.38	127.51
SCE1572_3827	68	96
SCE1572_3017	126	63
SCE1572_5871	118	49
SCE1572_8940	164	231
SCE1572_3039	98	6
SCE1572_10237	158	32
SCE1572_6956	49	1
SCE1572_5857	0	0
SCE1572_2754	164	68
SCE1572_10904	50	317

SCE1572_11090	122	94
SCE1572_10466	185	54
SCE1572_5725	19.28	0
SCE1572_1181	16	0
SCE1572_1583	126	5
SCE1572_4974	146	918
SCE1572_10847	146	26
SCE1572_6622	74	23
SCE1572_9086	48	5
SCE1572_1509	169	19
SCE1572_7548	116	16
SCE1572_7427	359	92
SCE1572_5843	17	2
SCE1572_1156	0	0
SCE1572_3387	30	2
SCE1572_1721	8	0
SCE1572_8857	57	56
SCE1572_4982	42	3
SCE1572_6479	95	233
SCE1572_1439	58	9
SCE1572_5552	2	3
SCE1572_9144	31	5
SCE1572_819	64	16
SCE1572_2742	38	30
SCE1572_5765	118	153
SCE1572_8185	43	1
SCE1572_1519	381	93
SCE1572_5777	39	153
SCE1572_10680	0	0
SCE1572_3867	64	118
SCE1572_10169	105	405
SCE1572_1315	59	91
SCE1572_9968	67	9
SCE1572_3736	51	28
SCE1572_810	336	101
SCE1572_7909	52	42
SCE1572_2256	231	57
SCE1572_8214	261	90
SCE1572_3020	304	39
SCE1572_6906	0	0
SCE1572_6577	36	13
SCE1572_3489	30	4
SCE1572_6965	37	12
SCE1572_1831	0	0
SCE1572_8681	75	8
SCE1572_3756	445.41	51.28
SCE1572_5868	161	56
SCE1572_3928	274	120

SCE1572_4724	175	36
SCE1572_1444	60	31
SCE1572_7487	234	53
SCE1572_7528	116	44
SCE1572_6488	19	3
SCE1572_7581	2137	2430
SCE1572_11247	32	10
SCE1572_4786	1154	6602
SCE1572_297	94	23
SCE1572_6386	81	10
SCE1572_9944	1	0
SCE1572_3357	0	0
SCE1572_10508	71	140
SCE1572_2044	1	0
SCE1572_5255	2	0
SCE1572_4405	39	4
SCE1572_4497	78	89
SCE1572_6970	10	73
SCE1572_6083	206	21
SCE1572_8916	27	51
SCE1572_6801	63	21
SCE1572_1160	127	28
SCE1572_703	14	3
SCE1572_658	439	266
SCE1572_3891	140	77
SCE1572_9614	46	0
SCE1572_9589	472	452
SCE1572_5653	170	432
SCE1572_6562	59	21
SCE1572_10996	47	10
SCE1572_8164	9	4
SCE1572_7116	59	51
SCE1572_633	44	32
SCE1572_6992	131	19
SCE1572_5512	230	162
SCE1572_1502	108	175
SCE1572_145	76	26
SCE1572_433	377	121
SCE1572_7636	678	5880
SCE1572_1909	0	0
SCE1572_11261	83	15
SCE1572_6529	60	26
SCE1572_4675	93	28
SCE1572_11512	1167	1689
SCE1572_6191	30	35
SCE1572_5454	46	10
SCE1572_6379	54	0
SCE1572_8907	77	33

SCE1572_1482	146	26
SCE1572_9810	68	35
SCE1572_8771	0	0
SCE1572_9442	25	2
SCE1572_4746	208	44
SCE1572_4706	111	43
SCE1572_11442	61	28
SCE1572_9347	43	6
SCE1572_4991	12	4
SCE1572_3699	29	2
SCE1572_4956	122	85
SCE1572_7881	147	58
SCE1572_2392	232	261
SCE1572_8417	78	25
SCE1572_6319	65	25
SCE1572_8197	29	6
SCE1572_10418	38	22
SCE1572_10515	133	104
SCE1572_4360	156	118
SCE1572_8900	407	168
SCE1572_8949	24	19
SCE1572_9208	35	16
SCE1572_527	35	33
SCE1572_2191	325	1541
SCE1572_7236	95	120
SCE1572_366	123	174
SCE1572_7615	115	38
SCE1572_5493	0	0
SCE1572_9244	3	0
SCE1572_4756	83	18
SCE1572_3258	61	117
SCE1572_8621	136	202
SCE1572_1347	600	1043
SCE1572_6545	196	356
SCE1572_3973	52	72
SCE1572_874	258	52
SCE1572_3435	99	54
SCE1572_10544	12	1
SCE1572_4418	18	6
SCE1572_2618	0	0
SCE1572_732	137	30
SCE1572_5028	92	30
SCE1572_3336	118	608
SCE1572_5055	131	32
SCE1572_11503	119	20
SCE1572_6659	98	49
SCE1572_4969	60	8
SCE1572_8161	67	3

SCE1572_10952	0	0
SCE1572_4633	81	29
SCE1572_5749	72	22
SCE1572_5678	273	358
SCE1572_2652	659	138
SCE1572_7229	128	277
SCE1572_7105	125	24
SCE1572_6520	56	23
SCE1572_1768	226	31
SCE1572_6860	531	111
SCE1572_3239	56	16
SCE1572_7215	64	49
SCE1572_2622	29	1
SCE1572_3088	17	6
SCE1572_10855	158	45
SCE1572_8845	48	9
SCE1572_5306	711	1035
SCE1572_9360	84	321
SCE1572_278	12	2
SCE1572_10036	18	3
SCE1572_4164	99	16
SCE1572_3242	18	0
SCE1572_1413	16	5
SCE1572_8541	21	117
SCE1572_5886	286	56
SCE1572_1843	71	14
SCE1572_8862	49	5
SCE1572_6516	180	18
SCE1572_3162	174	228
SCE1572_11169	114	12
SCE1572_11019	37	44
SCE1572_9160	117	136
SCE1572_5205	99	42
SCE1572_3871	89	84
SCE1572_5020	80	25
SCE1572_4232	90	10
SCE1572_258	48	30
SCE1572_1062	295	15
SCE1572_10598	0	0
SCE1572_9707	132	18
SCE1572_7718	167	28
SCE1572_10089	60	63
SCE1572_10080	251	417
SCE1572_3706	87	29
SCE1572_5803	38	18
SCE1572_6893	219	40
SCE1572_6999	76	4
SCE1572_9679	210	292

SCE1572_3513	121	389
SCE1572_9856	58	14
SCE1572_583	46	68
SCE1572_2113	83	32
SCE1572_8355	116.84	19.83
SCE1572_1368	206	174
SCE1572_801	53	19
SCE1572_7222	87	696
SCE1572_7452	21	14
SCE1572_2659	134	42
SCE1572_3275	216	168
SCE1572_7534	2	0
SCE1572_404	128	57
SCE1572_10619	147	38
SCE1572_3921	108	50
SCE1572_4488	174.39	35
SCE1572_5095	183	258
SCE1572_8847	0	0
SCE1572_4835	134	45
SCE1572_2724	61	108
SCE1572_628	33	26
SCE1572_9309	0	0
SCE1572_6455	1	0
SCE1572_2575	124	26
SCE1572_7573	80	248
SCE1572_3003	24	31
SCE1572_5671	106	23
SCE1572_202	136	49
SCE1572_3455	68	9
SCE1572_11332	41	44
SCE1572_7804	418	151
SCE1572_10718	12	17
SCE1572_4686	87.77	184.74
SCE1572_4249	1	0
SCE1572_6869	42	12
SCE1572_10052	106	130
SCE1572_1709	161	51
SCE1572_969	69	82
SCE1572_5229	21	20
SCE1572_5143	80	5
SCE1572_1711	151	45
SCE1572_7843	237	418
SCE1572_10230	160.76	22.04
SCE1572_6294	24	167
SCE1572_3338	113	700
SCE1572_1012	65	4
SCE1572_4844	85	65
SCE1572_10282	22	33

SCE1572_6288	39	6
SCE1572_189	74	36
SCE1572_1058	5	1
SCE1572_1728	196	49
SCE1572_8474	93	37
SCE1572_2423	124	144
SCE1572_9406	13	13
SCE1572_2502	0	0
SCE1572_6413	134	34
SCE1572_5023	0	0
SCE1572_5220	94	111
SCE1572_4411	60	7
SCE1572_4015	187	124
SCE1572_1094	46	9
SCE1572_10263	86	21
SCE1572_5869	38	162
SCE1572_7228	117	628
SCE1572_9169	129.41	25.37
SCE1572_651	26	0
SCE1572_198	148	154
SCE1572_8376	6	0
SCE1572_5934	22	2
SCE1572_5030	666	1874
SCE1572_11284	50	31
SCE1572_11299	73	11
SCE1572_9896	13	0
SCE1572_5005	17	0
SCE1572_6878	357	32
SCE1572_10973	301	598
SCE1572_5136	89	35
SCE1572_5958	53	20
SCE1572_459	24	3
SCE1572_8758	256	484
SCE1572_7972	27	61
SCE1572_10646	126	25
SCE1572_9661	397	1140
SCE1572_5734	142	127
SCE1572_3619	194	30
SCE1572_10393	321	54
SCE1572_1211	33	11
SCE1572_86	0	0
SCE1572_7210	6	1
SCE1572_11560	427.69	96.63
SCE1572_3286	46	12
SCE1572_10040	156	92
SCE1572_3521	228	109
SCE1572_3611	207	88
SCE1572_11208	277	185

SCE1572_3545	0	0
SCE1572_3472	80	7
SCE1572_9902	60	28
SCE1572_7735	467	1241
SCE1572_8529	167	45
SCE1572_7764	5	1
SCE1572_5218	35	7
SCE1572_1547	32	19
SCE1572_3390	54	57
SCE1572_6109	60	4
SCE1572_3202	299	3032
SCE1572_7550	242	35
SCE1572_5119	278	250
SCE1572_11180	20	3
SCE1572_6852	0	0
SCE1572_9921	163	94
SCE1572_10245	60	7
SCE1572_10707	0	0
SCE1572_182	325	145
SCE1572_7084	441	98
SCE1572_9288	529	337
SCE1572_565	95	138
SCE1572_8684	33	23
SCE1572_5174	10	1
SCE1572_6598	82	26
SCE1572_9036	99	26
SCE1572_354	48	12
SCE1572_8789	85	88
SCE1572_4433	147	21
SCE1572_2202	74	274
SCE1572_2051	114	15
SCE1572_2519	177	26
SCE1572_8808	23	5
SCE1572_8126	140	25
SCE1572_11395	336	848
SCE1572_4291	35	23
SCE1572_11162	96	88
SCE1572_7847	29	11
SCE1572_621	144	37
SCE1572_1871	150	61
SCE1572_5664	34	30
SCE1572_3668	61	11
SCE1572_1247	125	15
SCE1572_3344	332	479
SCE1572_10570	217	155
SCE1572_778	160	90
SCE1572_6834	0	0
SCE1572_10181	90	50

SCE1572_3625	64	8
SCE1572_3676	286.57	186
SCE1572_3462	1	18
SCE1572_8015	14	73
SCE1572_5240	7	9
SCE1572_4519	72	187
SCE1572_52	3	0
SCE1572_4220	24	12
SCE1572_10248	117	20
SCE1572_2641	219	80
SCE1572_8560	45	13
SCE1572_3814	155	80
SCE1572_1870	13	1
SCE1572_10720	168	28
SCE1572_8318	25	5
SCE1572_4287	137	17
SCE1572_27	486	355
SCE1572_5573	117	27
SCE1572_271	490.82	1324.47
SCE1572_7575	156	154
SCE1572_3477	0	0
SCE1572_10788	128	37
SCE1572_7831	92	110
SCE1572_6019	23	7
SCE1572_8926	92	62.5
SCE1572_10949	30	3
SCE1572_6619	30	6
SCE1572_4002	450	2592
SCE1572_1375	237	98
SCE1572_1455	194	47
SCE1572_11589	31	20
SCE1572_3806	16	0
SCE1572_8656	142	661
SCE1572_11546	141.59	6.51
SCE1572_1548	352	71
SCE1572_6700	24.98	4.59
SCE1572_4268	348	42
SCE1572_2899	149	18
SCE1572_9243	145	49
SCE1572_6640	224	31
SCE1572_3939	17	9
SCE1572_6596	72	6
SCE1572_7541	24	17
SCE1572_826	157	20
SCE1572_3816	0	0
SCE1572_3748	388	284
SCE1572_5350	824	313
SCE1572_7442	41	7

SCE1572_6919	55	17
SCE1572_6028	25	74
SCE1572_7870	116	52
SCE1572_7692	332	229
SCE1572_10068	275	481
SCE1572_9887	62	32
SCE1572_4132	155	132
SCE1572_3136	78	13
SCE1572_5594	59	19
SCE1572_1393	60	20
SCE1572_11191	171	104
SCE1572_7697	134	75
SCE1572_9746	170	58
SCE1572_2483	26	6
SCE1572_9880	20	8
SCE1572_3058	153	27
SCE1572_5360	0	0
SCE1572_4853	28	5
SCE1572_4477	111	13
SCE1572_8241	64	99
SCE1572_5922	22	20
SCE1572_10321	42	54
SCE1572_7701	28	2
SCE1572_7504	53	11
SCE1572_5322	69	33
SCE1572_3839	113	20
SCE1572_8727	147	22
SCE1572_10878	69	7
SCE1572_9496	100	128
SCE1572_5410	0	0
SCE1572_771	127	59
SCE1572_9681	412	478
SCE1572_7838	79	9
SCE1572_3751	52	16
SCE1572_6463	0	0
SCE1572_6330	92	129
SCE1572_6320	18	7
SCE1572_11550	38	14
SCE1572_129	3	0
SCE1572_7099	23	1
SCE1572_5918	0	0
SCE1572_6070	14	12
SCE1572_1566	77	12
SCE1572_2510	151	66
SCE1572_5590	188	26
SCE1572_10261	417	162
SCE1572_2710	60	14
SCE1572_9562	4	0

SCE1572_7925	22	3
SCE1572_350	149	2206
SCE1572_6753	73	21
SCE1572_8567	228	91
SCE1572_4212	1	0
SCE1572_5584	96	16
SCE1572_83	43.84	19.66
SCE1572_3126	26	65
SCE1572_4546	39	5
SCE1572_9280	29	5
SCE1572_2144	57	80
SCE1572_7877	119	11
SCE1572_11112	45	16
SCE1572_5536	109	44
SCE1572_4629	301	301
SCE1572_4239	211	388
SCE1572_9675	172	171
SCE1572_4943	363	875
SCE1572_9639	0	0
SCE1572_9236	31	6
SCE1572_2690	144	37
SCE1572_9536	261	49
SCE1572_4765	53	15
SCE1572_1222	56	12
SCE1572_6185	154	34
SCE1572_1174	6	0
SCE1572_4948	58	14
SCE1572_2891	4	0
SCE1572_8890	551	78
SCE1572_8668	16	1
SCE1572_10820	112	85
SCE1572_2878	321	172
SCE1572_2056	71	4
SCE1572_7258	104	25
SCE1572_487	156	81
SCE1572_201	2	0
SCE1572_11034	345	2239
SCE1572_4620	34	27
SCE1572_392	174	9
SCE1572_6204	46	40
SCE1572_845	0	0
SCE1572_57	159	20
SCE1572_5473	26	17
SCE1572_441	456	81
SCE1572_7206	54	6
SCE1572_5821	84.33	44.51
SCE1572_5599	297.73	65
SCE1572_4280	34	9

SCE1572_4142	0	0
SCE1572_30	1206	1424
SCE1572_9216	210	144
SCE1572_5900	28	16
SCE1572_2316	51	13
SCE1572_8546	119	18
SCE1572_4035	85	28
SCE1572_4566	22	9
SCE1572_7285	255	98
SCE1572_4659	110	58
SCE1572_6695	29	10
SCE1572_7588	266	890
SCE1572_3693	181	29
SCE1572_10959	85	15
SCE1572_9959	94	50
SCE1572_9932	27	1
SCE1572_7641	365	1590
SCE1572_11517	63	19
SCE1572_9714	33	434
SCE1572_6942	117	23
SCE1572_763	33	7
SCE1572_9828	70	17
SCE1572_3221	53	55
SCE1572_11381	41	42
SCE1572_6166	201	281
SCE1572_10999	27	1
SCE1572_1266	734	3638
SCE1572_8247	43	10
SCE1572_7600	191	120
SCE1572_11321	57	18
SCE1572_4077	31	37
SCE1572_2870	24	5
SCE1572_8362	0	0
SCE1572_10940	39	10
SCE1572_951	78	46
SCE1572_10832	206	484
SCE1572_1389	42	200
SCE1572_2542	74	4
SCE1572_9514	123	407
SCE1572_3304	7	2
SCE1572_3912	15	18
SCE1572_1370	146	219
SCE1572_4295	57	9
SCE1572_10566	182	33
SCE1572_8421	68	34
SCE1572_1080	39	12
SCE1572_5339	35	10
SCE1572_4656	35	40

SCE1572_3040	64	12
SCE1572_10227	29	4
SCE1572_7147	88	102
SCE1572_6168	37	18
SCE1572_1674	240	282
SCE1572_8000	43	8
SCE1572_6925	14	4
SCE1572_11373	235	608
SCE1572_10654	87	232
SCE1572_11526	149	23
SCE1572_9522	5	1
SCE1572_3414	449	275
SCE1572_1896	0	0
SCE1572_4602	36	43
SCE1572_6798	114	52
SCE1572_4080	108	245
SCE1572_4046	0	0
SCE1572_5246	340	499
SCE1572_3212	25	17
SCE1572_4694	49	19
SCE1572_7983	304	157
SCE1572_6665	76	143
SCE1572_5568	46	16
SCE1572_3144	26	2
SCE1572_1125	232	35
SCE1572_8778	85	14
SCE1572_2334	0	0
SCE1572_518	0	0
SCE1572_10208	183	33
SCE1572_7369	14	2
SCE1572_10494	172	566
SCE1572_9621	106	20
SCE1572_8071	70	18
SCE1572_4448	20	4
SCE1572_6161	149	34
SCE1572_4937	186	186
SCE1572_8953	60.46	8
SCE1572_5974	0	0
SCE1572_5370	0	0
SCE1572_3855	91	22
SCE1572_3029	9	0
SCE1572_9805	67	19
SCE1572_10892	241	243
SCE1572_2766	138	49
SCE1572_11187	4	1
SCE1572_10505	97	18
SCE1572_1289	0	0
SCE1572_1035	130	43

SCE1572_6310	52	257
SCE1572_5179	7	47
SCE1572_2537	11	14
SCE1572_6910	441	65
SCE1572_3403	31	32
SCE1572_5182	24	334
SCE1572_9377	24	11
SCE1572_6223	52	12
SCE1572_6314	17	0
SCE1572_7551	124	42
SCE1572_5715	67	26
SCE1572_3608	226	1092
SCE1572_8766	92	13
SCE1572_5000	49	24
SCE1572_10593	1	0
SCE1572_8597	434	59
SCE1572_3179	89	6
SCE1572_3	415	480
SCE1572_1100	90	53
SCE1572_11340	57	9
SCE1572_8296	33	41
SCE1572_389	16	5
SCE1572_381	27	3
SCE1572_11253	807	4673
SCE1572_2597	2.21	5.91
SCE1572_9019	9	3
SCE1572_4089	87	101
SCE1572_3529	409	1678
SCE1572_4481	76	9
SCE1572_2321	76	167
SCE1572_9332	0	0
SCE1572_10122	249	37
SCE1572_6896	103	155
SCE1572_3372	67	41
SCE1572_3247	104	153
SCE1572_10434	531	216
SCE1572_10137	375	68
SCE1572_9031	243	70
SCE1572_7481	18	0
SCE1572_9653	325.08	47.73
SCE1572_7057	8	0
SCE1572_10190	211	254
SCE1572_11014	92	24
SCE1572_130	106	16
SCE1572_2927	21	2
SCE1572_215	95	32
SCE1572_7903	10	5
SCE1572_5295	42	4

SCE1572_8261	172	304
SCE1572_8648	96	6
SCE1572_6591	76.44	14
SCE1572_191	336	67
SCE1572_9062	154	36
SCE1572_10663	292	183
SCE1572_4467	48	6
SCE1572_4201	18	7
SCE1572_7106	18	3
SCE1572_11226	77	16
SCE1572_9212	98	52
SCE1572_483	76	19
SCE1572_3536	17	2
SCE1572_2471	74	9
SCE1572_2431	79	247
SCE1572_1981	0	0
SCE1572_3743	41	20
SCE1572_1737	38	6
SCE1572_8103	39	22
SCE1572_5541	47	76
SCE1572_10124	458	73
SCE1572_9576	28	3
SCE1572_4814	502	564
SCE1572_287	166	103
SCE1572_939	97	16
SCE1572_10096	258	268
SCE1572_5471	51	9
SCE1572_3832	78	100
SCE1572_3170	89	85
SCE1572_2094	16	13
SCE1572_7854	61	61
SCE1572_10582	9	4
SCE1572_9196	118	22
SCE1572_6709	98	82
SCE1572_4933	18	27
SCE1572_11276	59	13
SCE1572_8615	198	62
SCE1572_5393	295	58
SCE1572_5405	48	33
SCE1572_10589	9	1
SCE1572_2451	0	0
SCE1572_8320	12	8
SCE1572_1091	171.22	14
SCE1572_5467	111	18
SCE1572_7463	210	323
SCE1572_11039	98	33
SCE1572_7653	17	8
SCE1572_641	82	55

SCE1572_5148	51	2
SCE1572_10288	275	89
SCE1572_9654	39.29	5
SCE1572_10579	18	2
SCE1572_578	4	0
SCE1572_8086	9	0
SCE1572_711	63	122
SCE1572_6873	90	299
SCE1572_3209	95	38
SCE1572_3922	74	14
SCE1572_9978	81	5
SCE1572_7478	48	56
SCE1572_11264	33	48
SCE1572_5499	62	8
SCE1572_7404	15	109
SCE1572_6332	124	32
SCE1572_1334	17	0
SCE1572_3315	26	19
SCE1572_7994	25	0
SCE1572_6977	16	0
SCE1572_7278	22	3
SCE1572_11421	120	52
SCE1572_11450	109	78
SCE1572_9619	963	241
SCE1572_6744	25	2
SCE1572_7390	23	9
SCE1572_3313	62	8
SCE1572_11234	51	10
SCE1572_11398	181	422
SCE1572_3609	85	8
SCE1572_7167	0	0
SCE1572_5902	760	90
SCE1572_10184	17	33
SCE1572_6649	44	1
SCE1572_3427	174.15	60
SCE1572_8532	22	0
SCE1572_6125	40	48
SCE1572_8938	8	6
SCE1572_4767	327	241
SCE1572_1234	65	12
SCE1572_10344	2357	7504
SCE1572_10932	24	18
SCE1572_7134	43	8
SCE1572_543	55	15
SCE1572_8986	1	0
SCE1572_8489	169	82
SCE1572_997	35	19
SCE1572_9488	124	66

SCE1572_10993	0	0
SCE1572_9594	47	386
SCE1572_3883	32	8
SCE1572_9994	263	39
SCE1572_11081	123	187
SCE1572_769	36	4
SCE1572_594	10	1
SCE1572_7655	32	5
SCE1572_7399	31	16
SCE1572_127	43	10
SCE1572_1979	1807	2303
SCE1572_6129	762	811
SCE1572_265	210	45
SCE1572_6030	47	18
SCE1572_1946	183	50
SCE1572_1436	33	8
SCE1572_992	496	421
SCE1572_891	159	164
SCE1572_7262	73	31
SCE1572_10310	53.58	12.18
SCE1572_9761	9	7
SCE1572_6003	83	482
SCE1572_6242	49	15
SCE1572_7526	0	0
SCE1572_5928	147	66
SCE1572_11303	208	88
SCE1572_413	137	24
SCE1572_1641	53	7
SCE1572_2061	30	7
SCE1572_7240	113	77
SCE1572_9710	263	273
SCE1572_7050	0	0
SCE1572_2769	36	14
SCE1572_760	3842	691
SCE1572_311	116	43
SCE1572_10401	42	38
SCE1572_9201	130	22
SCE1572_11401	58	13
SCE1572_6779	26	8
SCE1572_7985	108	99
SCE1572_4812	84	33
SCE1572_4155	32	39
SCE1572_2358	126	76
SCE1572_8550	71	90
SCE1572_807	97	32
SCE1572_9738	58	5
SCE1572_8444	28	9
SCE1572_2745	78	72

SCE1572_4334	52	6
SCE1572_4191	162	74
SCE1572_5248	70	59
SCE1572_3195	27	0
SCE1572_10492	79	119
SCE1572_9528	299	234
SCE1572_10296	183	1395
SCE1572_1469	4	3
SCE1572_11108	48.61	21
SCE1572_10211	98	35
SCE1572_6176	180	115
SCE1572_8250	203	182
SCE1572_8777	54	17
SCE1572_3502	69	30
SCE1572_9683	68	150
SCE1572_2219	0	0
SCE1572_10483	69	19
SCE1572_5786	91	37
SCE1572_7447	1	0
SCE1572_10272	114	553
SCE1572_1596	45	3
SCE1572_9116	388	490
SCE1572_6500	36	30
SCE1572_3302	398	198
SCE1572_9358	94	109
SCE1572_7955	143	102
SCE1572_7171	13	0
SCE1572_1807	23	28
SCE1572_105	32	0
SCE1572_1756	4	0
SCE1572_11477	34.72	3
SCE1572_494	111	14
SCE1572_6346	46	24
SCE1572_7313	125	31
SCE1572_1293	61	14
SCE1572_2688	22	14
SCE1572_9911	52	15
SCE1572_5190	137	181
SCE1572_10507	144	86
SCE1572_5266	85	113
SCE1572_8701	72	38
SCE1572_11210	55	11
SCE1572_7269	361	829
SCE1572_4506	51	8
SCE1572_7009	264	37
SCE1572_9754	63	35
SCE1572_529	9	0
SCE1572_10361	30	435

SCE1572_10256	140	24
SCE1572_8571	11	2
SCE1572_2843	5	2
SCE1572_11203	44	33
SCE1572_677	176	42
SCE1572_9640	2071	2007.01
SCE1572_5508	5	0
SCE1572_8228	109	13
SCE1572_9462	59	48
SCE1572_3950	0	0
SCE1572_1201	59	10
SCE1572_11085	99	108
SCE1572_1773	0	0
SCE1572_9068	49	21
SCE1572_721	59.48	18
SCE1572_1811	84	25
SCE1572_5554	248	107
SCE1572_3666	61	2
SCE1572_7163	105	71
SCE1572_10533	65	61
SCE1572_4583	0	0
SCE1572_2681	227	16
SCE1572_10353	2034	1156
SCE1572_9061	117	25
SCE1572_2907	77	331
SCE1572_208	8	1
SCE1572_6667	196	81
SCE1572_6091	35	4
SCE1572_11278	10	0
SCE1572_909	294.04	60
SCE1572_9352	49	119
SCE1572_3711	37	0
SCE1572_2182	26	5
SCE1572_4111	42	5
SCE1572_9379	100	15
SCE1572_4591	15	6
SCE1572_7188	129	139
SCE1572_3725	692	5405
SCE1572_2282	50	50
SCE1572_6761	21	8
SCE1572_1732	91	22
SCE1572_7905	85	13
SCE1572_3717	343	195
SCE1572_2524	47	21
SCE1572_8294	50	18
SCE1572_4533	57	6
SCE1572_10665	120	227
SCE1572_14	85	37

SCE1572_5578	12	0
SCE1572_9847	110	23
SCE1572_10377	98	9
SCE1572_9556	289	257
SCE1572_1473	102	20
SCE1572_11281	199	21
SCE1572_361	2	0
SCE1572_6749	6	1
SCE1572_3443	37	3
SCE1572_6552	0	0
SCE1572_4369	189	133
SCE1572_5603	59	38
SCE1572_2066	488	173
SCE1572_4568	29	2
SCE1572_10094	24	0
SCE1572_900	165	38
SCE1572_4871	40	16
SCE1572_10177	50	66
SCE1572_7046	8	2
SCE1572_1854	0	0
SCE1572_8528	202	143
SCE1572_8280	44	5
SCE1572_5700	10	0
SCE1572_5199	88	35
SCE1572_4364	179	30
SCE1572_2949	7	0
SCE1572_11079	208	920
SCE1572_1182	0	0
SCE1572_7820	200	402
SCE1572_11286	209	184
SCE1572_5636	64	62
SCE1572_561	218	800
SCE1572_10396	24	9
SCE1572_1280	180	24
SCE1572_9521	0	0
SCE1572_11552	189	42
SCE1572_4920	26	2
SCE1572_6984	10	4
SCE1572_8267	175	87
SCE1572_7722	158	62
SCE1572_10926	0	0
SCE1572_10919	34	10
SCE1572_5662	7	7
SCE1572_556	20	3
SCE1572_11245	27	5
SCE1572_820	41	17
SCE1572_911	43	13
SCE1572_7259	0	0

SCE1572_5811	167	42
SCE1572_1902	391	49
SCE1572_1072	107	19
SCE1572_989	56	57
SCE1572_9543	137	24
SCE1572_3959	53	14
SCE1572_8238	132	403
SCE1572_2552	118	53
SCE1572_1529	62	21
SCE1572_10829	45	9
SCE1572_8221	69	8
SCE1572_3640	19	55
SCE1572_6613	80	23
SCE1572_11154	30	32
SCE1572_4916	166	656
SCE1572_280	103	22
SCE1572_4893	144	60
SCE1572_3086	71	9
SCE1572_10942	497	90
SCE1572_5113	11	5
SCE1572_9800	106	35
SCE1572_5769	712	2577
SCE1572_2675	43	112
SCE1572_2246	79	33
SCE1572_5313	27	5
SCE1572_1079	0	0
SCE1572_237	107	14
SCE1572_6783	16	26
SCE1572_3013	23	10
SCE1572_2925	399	86
SCE1572_6057	111	108
SCE1572_5387	122	73
SCE1572_9719	44	254
SCE1572_3905	372	840
SCE1572_1554	233	57
SCE1572_7750	34	2
SCE1572_10742	101	30
SCE1572_8964	62	51
SCE1572_5672	0	0
SCE1572_4792	15	1
SCE1572_8289	20	11
SCE1572_11357	92	20
SCE1572_10800	145	63
SCE1572_8860	26	3
SCE1572_8490	36	34
SCE1572_1325	21	2
SCE1572_5216	30	29
SCE1572_4308	0	0

SCE1572_2772	14	4
SCE1572_8720	12	5
SCE1572_3369	328	296
SCE1572_9137	10	5
SCE1572_5709	78	44
SCE1572_243	10	1
SCE1572_3226	88	14
SCE1572_11056	8	10
SCE1572_5548	142	24
SCE1572_2821	237	85
SCE1572_390	37	6
SCE1572_5538	234	130
SCE1572_5500	842	200
SCE1572_6469	80	8
SCE1572_2233	59	18
SCE1572_10157	99	64
SCE1572_616	90	139
SCE1572_8797	127	12
SCE1572_3270	60	171
SCE1572_7969	10	1
SCE1572_8257	92	107
SCE1572_8831	239	346
SCE1572_8665	57	9
SCE1572_669	117	30
SCE1572_5792	24	19
SCE1572_4820	92	46
SCE1572_9075	254	40
SCE1572_8341	20	7
SCE1572_4869	15	9
SCE1572_8729	21	12
SCE1572_2137	72	17
SCE1572_4544	88	30
SCE1572_2415	33	3
SCE1572_8699	190	38
SCE1572_5648	98	19
SCE1572_10813	19	11
SCE1572_9351	263	431
SCE1572_439	905	84
SCE1572_2912	1027.07	137.17
SCE1572_9411	190.42	422.82
SCE1572_3051	49	1
SCE1572_7699	30	11
SCE1572_8124	60	15
SCE1572_1919	108	34
SCE1572_2857	26	3
SCE1572_6772	10	0
SCE1572_5995	156	56
SCE1572_4181	59	28

SCE1572_8499	106	17
SCE1572_4550	119	18
SCE1572_11534	62	10
SCE1572_1824	73	46
SCE1572_4744	10	56
SCE1572_1829	692.18	1046.53
SCE1572_10078	323	314
SCE1572_2402	29	49
SCE1572_5879	54	7
SCE1572_5920	87	79
SCE1572_3092	28	0
SCE1572_9458	68	33
SCE1572_5625	4	2
SCE1572_5273	5	1
SCE1572_10523	44	2
SCE1572_9469	244	183
SCE1572_8740	71	19
SCE1572_7115	48	4
SCE1572_5846	108	90
SCE1572_6638	133	27
SCE1572_507	60	8
SCE1572_4570	140	24
SCE1572_8738	113	36
SCE1572_9124	85	25
SCE1572_9699	64	21
SCE1572_140	14	5
SCE1572_7733	80	161
SCE1572_8886	227.51	36
SCE1572_11436	194	79
SCE1572_7629	49	61
SCE1572_6187	84	42
SCE1572_6151	179	15
SCE1572_8888	18	3
SCE1572_6608	11	17
SCE1572_5352	348	330
SCE1572_2672	77	42
SCE1572_10107	117	291
SCE1572_2381	235	294
SCE1572_3850	42	47
SCE1572_9076	70	15
SCE1572_11040	163	51
SCE1572_2719	38	11
SCE1572_10385	48	7
SCE1572_9885	69	49
SCE1572_1778	63	15
SCE1572_864	334	232
SCE1572_6147	29	4
SCE1572_7729	70	72

SCE1572_4512	0	0
SCE1572_6564	0	0
SCE1572_6116	194	32
SCE1572_3043	0	0
SCE1572_7023	0	0
SCE1572_150	88	26
SCE1572_9312	172	109
SCE1572_9151	26	4
SCE1572_1613	338	34
SCE1572_6031	149	61
SCE1572_2668	15	10
SCE1572_3698	58	24
SCE1572_2125	157	36
SCE1572_2150	35	3
SCE1572_9301	24	49
SCE1572_4754	135	80
SCE1572_6506	1	0
SCE1572_10499	22	198
SCE1572_4577	58	11
SCE1572_4356	36	12
SCE1572_2704	187	275
SCE1572_9604	51	3
SCE1572_2155	123	29
SCE1572_2958	303	83
SCE1572_8378	71	45
SCE1572_5429	110	89
SCE1572_1626	77	86
SCE1572_2275	294	794
SCE1572_4121	380	427
SCE1572_2397	28	364
SCE1572_7065	232	94
SCE1572_893	39	33
SCE1572_4104	53	6
SCE1572_9819	78	10
SCE1572_1273	163	38
SCE1572_3453	149	27
SCE1572_11179	94	46
SCE1572_5380	60	7
SCE1572_1744	156	72
SCE1572_1758	484	61
SCE1572_2412	148	38
SCE1572_8135	81	10
SCE1572_60	232	80
SCE1572_9048	6	3
SCE1572_8925	0.5	0
SCE1572_6917	23	14
SCE1572_1783	58	57
SCE1572_8472	54	607

SCE1572_3343	227	222
SCE1572_1237	41	18
SCE1572_2425	866	680
SCE1572_1937	1	0
SCE1572_8467	47	5
SCE1572_1017	24	193
SCE1572_5168	58	68
SCE1572_5090	78	46
SCE1572_6021	450	246
SCE1572_7293	0	0
SCE1572_9772	27	29
SCE1572_4527	129	190
SCE1572_4862	11	0
SCE1572_10366	23	2
SCE1572_3971	205	69
SCE1572_7122	598	309
SCE1572_8650	74	12
SCE1572_2041	35	89
SCE1572_10961	47	47
SCE1572_3185	324	48
SCE1572_67	166	27
SCE1572_10252	0	0
SCE1572_10038	182	159
SCE1572_4428	18	13
SCE1572_9210	108	31
SCE1572_4770	48	60
SCE1572_8552	156	111
SCE1572_2632	146.94	26.57
SCE1572_10063	524	1662
SCE1572_2804	269	90
SCE1572_5108	226	90
SCE1572_10898	15	42
SCE1572_5420	112	98
SCE1572_3364	56	43
SCE1572_8442	18	8
SCE1572_10906	3	0
SCE1572_6514	38	4
SCE1572_3994	242	41
SCE1572_11201	106	102
SCE1572_6054	86	114
SCE1572_5039	9	10
SCE1572_2910	108	17
SCE1572_6449	578	72
SCE1572_11480	365	197
SCE1572_8130	82	17
SCE1572_10902	22	8
SCE1572_11066	81	31
SCE1572_4808	55	132

SCE1572_3687	181	18
SCE1572_8334	0	0
SCE1572_3793	177	182
SCE1572_7593	191	80
SCE1572_6258	6	3
SCE1572_3426	234	32
SCE1572_1535	27	22
SCE1572_11356	40	35
SCE1572_8676	107	49
SCE1572_3822	18	16
SCE1572_10642	253	433
SCE1572_9052	82.65	35
SCE1572_3232	88	109
SCE1572_1468	0	0
SCE1572_921	108	23
SCE1572_4643	99	33
SCE1572_2850	55	105
SCE1572_2986	0	0
SCE1572_10005	14	6
SCE1572_4829	38	69
SCE1572_9428	366	257
SCE1572_5799	59	20
SCE1572_1698	17	51
SCE1572_3765	126	62
SCE1572_3587	264	98
SCE1572_9251	245	50
SCE1572_4028	113	88
SCE1572_8158	76	8
SCE1572_10453	60	17
SCE1572_7796	468	126
SCE1572_2602	147.7	19.18
SCE1572_4836	51	66
SCE1572_256	14	1
SCE1572_4420	0	0
SCE1572_11143	61	30
SCE1572_9987	185	25
SCE1572_6814	99	54
SCE1572_3945	2065	1719
SCE1572_8206	86	66
SCE1572_2963	216	1115
SCE1572_7852	0	0
SCE1572_6158	39	14
SCE1572_1794	33	388
SCE1572_3577	67	57
SCE1572_2606	33	2
SCE1572_10793	22	6
SCE1572_3134	102	17
SCE1572_9397	35	29

SCE1572_1649	128.72	16.73
SCE1572_6373	82	22
SCE1572_1664	51	59
SCE1572_8714	237	160
SCE1572_7688	233	23
SCE1572_1053	85	73
SCE1572_7937	19	5
SCE1572_3841	160	33
SCE1572_9439	50	26
SCE1572_2082	44	20
SCE1572_10822	75	31
SCE1572_10537	0	0
SCE1572_11062	264	64
SCE1572_3984	60	77
SCE1572_6842	66	20
SCE1572_6559	94	58
SCE1572_2290	323	842
SCE1572_11310	110	15
SCE1572_3002	9	2
SCE1572_1883	0	0
SCE1572_5379	79	11
SCE1572_4777	142	232
SCE1572_7459	194	197
SCE1572_5980	1205	340
SCE1572_5763	0	0
SCE1572_11463	731	563
SCE1572_11573	0	0
SCE1572_325	19	2
SCE1572_7539	229	29
SCE1572_2445	57	9
SCE1572_3967	211	58
SCE1572_4559	22	27
SCE1572_1536	114	26
SCE1572_4636	84	7
SCE1572_7683	70	8
SCE1572_7144	48	43
SCE1572_9646	259	144
SCE1572_7783	0	0
SCE1572_5187	28	825
SCE1572_2353	7	9
SCE1572_4242	12	1
SCE1572_1984	0	0
SCE1572_10408	55	33
SCE1572_9822	28	8
SCE1572_10685	71	22
SCE1572_11135	100	49
SCE1572_6530	68	37
SCE1572_8196	43	10

SCE1572_2648	59	12
SCE1572_2378	31	164
SCE1572_1402	358	47
SCE1572_1708	15	0
SCE1572_2171	364	79
SCE1572_7247	0	0
SCE1572_1496	15	3
SCE1572_7252	133	17
SCE1572_1147	318	175
SCE1572_2953	11	0
SCE1572_2204	73	214
SCE1572_8657	6	34
SCE1572_4171	258	123
SCE1572_5486	295	30
SCE1572_11122	167	20
SCE1572_2189	27	31
SCE1572_2835	5	0
SCE1572_2210	105	234
SCE1572_4396	12	7
SCE1572_1491	0	0
SCE1572_1497	44.75	7.44
SCE1572_11489	0	0
SCE1572_681	32	6
SCE1572_3297	49	15
SCE1572_4139	40	6
SCE1572_5158	3	0
SCE1572_9829	9	0
SCE1572_4903	110	69
SCE1572_3215	592	804
SCE1572_302	46	6
SCE1572_8791	108	224
SCE1572_4326	49	13
SCE1572_4271	54	19
SCE1572_735	156	33
SCE1572_6882	0	0
SCE1572_1955	18	9
SCE1572_3009	13	8
SCE1572_4038	143	71
SCE1572_5070	54	34
SCE1572_7619	132	265
SCE1572_5016	265	1580
SCE1572_9981	252	61
SCE1572_1464	112	20
SCE1572_5290	176	30
SCE1572_4668	4	1
SCE1572_2589	150	106
SCE1572_1295	152	36
SCE1572_10199	296	122

SCE1572_663	0	0
SCE1572_3352	74	13
SCE1572_1764	55	7
SCE1572_2106	164	187
SCE1572_11315	1017	750
SCE1572_8867	88	19
SCE1572_10697	175	162
SCE1572_9838	0	0
SCE1572_787	431	757
SCE1572_1900	14	3
SCE1572_135	77	18
SCE1572_8516	106	7
SCE1572_11583	236	259
SCE1572_7865	80	20
SCE1572_1023	30	9
SCE1572_8097	67	17
SCE1572_11101	45	22
SCE1572_717	25	4
SCE1572_8392	0	0
SCE1572_7194	0	0
SCE1572_2780	36	2
SCE1572_10882	159	500
SCE1572_10324	70	45
SCE1572_5282	233	116
SCE1572_3645	280	236
SCE1572_1802	50	21
SCE1572_8877	1	0
SCE1572_2885	79	19
SCE1572_8306	72	2
SCE1572_6497	51	4
SCE1572_8173	37	4
SCE1572_4605	201	564
SCE1572_9266	134	99
SCE1572_1258	50	2
SCE1572_10336	36	12
SCE1572_3074	39	7
SCE1572_6306	33	8
SCE1572_3491	97	74
SCE1572_1517	1	0
SCE1572_3773	145	25
SCE1572_7948	131	88
SCE1572_440	1094	148
SCE1572_9897	41	14
SCE1572_10446	19	5
SCE1572_10772	36	14
SCE1572_8068	150.4	17.23
SCE1572_7643	385	200
SCE1572_5561	570	117

SCE1572_11006	0	0
SCE1572_7005	16	2
SCE1572_8544	58	19
SCE1572_1041	345	61
SCE1572_6676	71	19
SCE1572_4226	49	33
SCE1572_11561	131	91
SCE1572_3595	13.25	0.75
SCE1572_9292	76	60
SCE1572_7915	1	0
SCE1572_4661	23	5
SCE1572_3103	23	4
SCE1572_2360	39	105
SCE1572_9812	621	87
SCE1572_2735	65	21
SCE1572_3709	0	0
SCE1572_7784	32.02	64.61
SCE1572_7103	0	0
SCE1572_10087	0	0
SCE1572_11021	108	34
SCE1572_9503	580	804
SCE1572_3731	142	26
SCE1572_2327	394	88
SCE1572_7437	142	19
SCE1572_168	87	15
SCE1572_9867	89	75
SCE1572_6718	0	0
SCE1572_8838	0	0
SCE1572_6218	84	12
SCE1572_9323	93	61
SCE1572_8995	50	9
SCE1572_3897	63	41
SCE1572_6803	53	35
SCE1572_9394	35	3
SCE1572_8746	22	10
SCE1572_3062	22	2
SCE1572_230	105	19
SCE1572_10307	44	4
SCE1572_11418	209	79
SCE1572_4644	187	57
SCE1572_456	57	8
SCE1572_9569	0	0
SCE1572_8459	78	37
SCE1572_8365	53	10
SCE1572_4198	15	0
SCE1572_10020	108	19
SCE1572_9633	153	20
SCE1572_5580	76	32

SCE1572_5772	126	34
SCE1572_2488	2	0
SCE1572_10761	0	0
SCE1572_371	30	123
SCE1572_2300	56	121
SCE1572_8258	53	132
SCE1572_9448	142	40
SCE1572_4622	144	1410
SCE1572_6355	0	0
SCE1572_11495	165	13
SCE1572_937	19	6
SCE1572_11432	58	19
SCE1572_9012	61	22
SCE1572_2314	68	47
SCE1572_917	187	201
SCE1572_5479	91	18
SCE1572_4888	87	23
SCE1572_2591	90	22
SCE1572_3379	194	23
SCE1572_9513	43	16
SCE1572_384	0	0
SCE1572_10785	280	185
SCE1572_7800	151	122
SCE1572_10808	8	0
SCE1572_843	242	544
SCE1572_603	27	4
SCE1572_4960	94	69
SCE1572_1488	0	0
SCE1572_6395	0	0
SCE1572_9656	71	16
SCE1572_6000	94	129
SCE1572_8688	321	209
SCE1572_3370	13	84
SCE1572_11256	64	45
SCE1572_9716	18	69
SCE1572_414	63	19
SCE1572_6691	1	0
SCE1572_8628	77	89
SCE1572_8342	117.22	27
SCE1572_9709	173	116
SCE1572_1639	38	24
SCE1572_464	27	115
SCE1572_6209	44.01	3.02
SCE1572_7871	61	17
SCE1572_7765	27	10
SCE1572_5363	149	18
SCE1572_3073	25	4
SCE1572_55	794	104

SCE1572_7602	145	53
SCE1572_1427	2	0
SCE1572_5297	54	26
SCE1572_7922	82	20
SCE1572_6071	0	0
SCE1572_7480	62	38
SCE1572_3906	254	114
SCE1572_3487	131	14
SCE1572_260	121.36	13.05
SCE1572_10541	113	49
SCE1572_6202	247	70
SCE1572_5845	120	46
SCE1572_573	604	471
SCE1572_4478	36	18
SCE1572_8694	84	15
SCE1572_3873	43	15
SCE1572_10433	48	15
SCE1572_10972	65	114
SCE1572_4166	60	12
SCE1572_9271	33	22
SCE1572_1265	248	3921
SCE1572_10077	309	301
SCE1572_7523	276	51
SCE1572_9338	31	19
SCE1572_4282	26	1
SCE1572_2540	144	32
SCE1572_7904	27	4
SCE1572_8543	124	1058
SCE1572_6697	20	38
SCE1572_10313	86	17
SCE1572_9199	39	3
SCE1572_9670	132	193
SCE1572_7559	149	42
SCE1572_10994	57	34
SCE1572_6862	110	14
SCE1572_6976	145	19
SCE1572_10339	42	11
SCE1572_10725	60	11
SCE1572_2247	30	4
SCE1572_11279	28	48
SCE1572_5328	165	58
SCE1572_2	247	190
SCE1572_519	44	19
SCE1572_1306	957	16158
SCE1572_7465	193	133
SCE1572_468	49	65
SCE1572_1609	57	22
SCE1572_9821	124	22

SCE1572_5498	180	70
SCE1572_6235	58	25
SCE1572_2260	31	13
SCE1572_1004	55.24	22.78
SCE1572_10341	59	9
SCE1572_1645	339	258
SCE1572_10050	274	223
SCE1572_7411	69	21
SCE1572_2539	55	28
SCE1572_6203	58	121
SCE1572_4010	115	23
SCE1572_3249	104	62
SCE1572_8263	124	157
SCE1572_2188	130	98
SCE1572_10032	80	99
SCE1572_8102	34	71
SCE1572_118	193	370
SCE1572_7340	223	197
SCE1572_5461	60	6
SCE1572_8599	0	0
SCE1572_3172	569	134
SCE1572_624	1	0
SCE1572_4825	215	99
SCE1572_3475	45	46
SCE1572_10522	11	7
SCE1572_11397	2	0
SCE1572_4584	326	49
SCE1572_11138	5	0
SCE1572_11392	160	136
SCE1572_3206	181	63
SCE1572_9392	53	85
SCE1572_1963	86	14
SCE1572_10947	6	2
SCE1572_3482	86	9
SCE1572_1681	42	161
SCE1572_5780	67	40
SCE1572_11190	0	0
SCE1572_3801	266	72
SCE1572_8760	296	126
SCE1572_8159	5	0
SCE1572_7380	140	24
SCE1572_5032	226	937
SCE1572_10823	137	42
SCE1572_6825	0	0
SCE1572_9572	7	4
SCE1572_8610	120	28
SCE1572_3405	125	24
SCE1572_4358	125	18

SCE1572_5684	64	81
SCE1572_10774	209	330
SCE1572_8704	16	24
SCE1572_7976	47	5
SCE1572_10584	387	675
SCE1572_9865	115	45
SCE1572_10957	59	30
SCE1572_4495	96	28
SCE1572_227	29	6
SCE1572_5430	804	416
SCE1572_9630	63	9
SCE1572_4515	36	13
SCE1572_252	0	0
SCE1572_8174	10	1
SCE1572_6859	28	3
SCE1572_1943	83	20
SCE1572_220	85	77
SCE1572_2726	104	600
SCE1572_6163	75	11
SCE1572_3593	123	22
SCE1572_7003	231	24
SCE1572_10015	145	153
SCE1572_3982	91	96
SCE1572_11475	102.19	25.1
SCE1572_1288	329	136
SCE1572_21	65	15
SCE1572_1941	198	14
SCE1572_7013	11.45	0
SCE1572_2053	64	25
SCE1572_6690	44	32
SCE1572_3830	48	96
SCE1572_2530	0	0
SCE1572_2268	79	16
SCE1572_3037	63	11
SCE1572_11593	92	285
SCE1572_9956	61	15
SCE1572_3194	131	68
SCE1572_10183	114	191
SCE1572_8730	175	50
SCE1572_10876	171	64
SCE1572_11164	18	10
SCE1572_5374	203	83
SCE1572_9574	10	1
SCE1572_10010	32	9
SCE1572_3524	81	28
SCE1572_3307	261	2421
SCE1572_6078	2	0
SCE1572_4347	37	6

SCE1572_2512	65	591
SCE1572_972	185	208
SCE1572_9232	2	0
SCE1572_3125	38	18
SCE1572_8216	85	18
SCE1572_6010	97	438
SCE1572_7415	24	3
SCE1572_5530	309	67
SCE1572_3766	57	89
SCE1572_7079	14	68
SCE1572_8209	342	148
SCE1572_2389	17	12
SCE1572_5577	12	0
SCE1572_777	75	6
SCE1572_1733	208	38
SCE1572_7676	51	3
SCE1572_10756	101	35
SCE1572_9799	5	2
SCE1572_504	21	4
SCE1572_7307	25	2
SCE1572_7082	88	21
SCE1572_5123	62	8
SCE1572_1267	0	0
SCE1572_10049	150	55
SCE1572_958	41	20
SCE1572_6458	119	46
SCE1572_5518	109	17
SCE1572_8645	0	0
SCE1572_11151	48	14
SCE1572_4388	28	8
SCE1572_6092	83	4
SCE1572_1223	11	11
SCE1572_6553	0	0
SCE1572_10682	27	11
SCE1572_8229	21	5
SCE1572_5408	60	19
SCE1572_1913	0	0
SCE1572_8125	209	14
SCE1572_7878	65	13
SCE1572_6594	202	130
SCE1572_9252	4	1
SCE1572_2749	227	294
SCE1572_91	346	1459
SCE1572_1877	0	0
SCE1572_6268	123	28
SCE1572_6180	311	70
SCE1572_6816	435	58
SCE1572_9097	6	1

SCE1572_7253	61	9
SCE1572_5349	62	5
SCE1572_4650	119	128
SCE1572_2930	108	13
SCE1572_5639	26	5
SCE1572_10950	120	22
SCE1572_11018	0	0
SCE1572_3234	107	26
SCE1572_2475	162	57
SCE1572_9538	101	11
SCE1572_3782	60	58
SCE1572_8935	24	6
SCE1572_6317	0	0
SCE1572_6940	15	8
SCE1572_6017	370	528
SCE1572_7621	56	15
SCE1572_3418	99	34
SCE1572_6259	14	5
SCE1572_9375	0	0
SCE1572_6261	21	3
SCE1572_7243	130	88
SCE1572_1620	45	103
SCE1572_8809	122	8
SCE1572_2185	240	30
SCE1572_3692	52	21
SCE1572_10083	311	583
SCE1572_1676	81	77
SCE1572_10460	102	132
SCE1572_2939	22	28
SCE1572_9790	179	131
SCE1572_8013	92	106
SCE1572_714	23	1
SCE1572_6067	42	15
SCE1572_4003	51	57
SCE1572_9531	96	59
SCE1572_690	70	21
SCE1572_2639	103	20
SCE1572_5912	52	39
SCE1572_11580	50	1
SCE1572_8523	18	0
SCE1572_9794	69	32
SCE1572_6823	140	188
SCE1572_2058	3	0
SCE1572_9108	47	27
SCE1572_4435	27	18
SCE1572_3933	312	329
SCE1572_9616	1	0
SCE1572_1939	93.71	134.01

SCE1572_705	86	37
SCE1572_7734	7	0
SCE1572_5784	95	102
SCE1572_4436	118	36
SCE1572_4945	73	71
SCE1572_3995	66	27
SCE1572_11587	6	6
SCE1572_9926	76	314
SCE1572_5903	33	11
SCE1572_5271	13	3
SCE1572_9802	76	27
SCE1572_7705	0	0
SCE1572_1543	452	128
SCE1572_8349	83	3
SCE1572_10017	134	236
SCE1572_3918	27	32
SCE1572_1634	464	1033
SCE1572_2580	0	0
SCE1572_6482	224	77
SCE1572_8895	121	55
SCE1572_6982	10	0
SCE1572_73	102	176
SCE1572_11338	79	4
SCE1572_6750	50	16
SCE1572_1560	5	0
SCE1572_1546	282	56
SCE1572_2873	109	33
SCE1572_9756	16	4
SCE1572_3753	115	179
SCE1572_550	0	0
SCE1572_11365	10	2
SCE1572_9246	0	0
SCE1572_11268	212	104
SCE1572_851	1775	2226
SCE1572_3635	28	7
SCE1572_9	46	24
SCE1572_6505	75	26
SCE1572_4681	26	7
SCE1572_5572	595.59	127.72
SCE1572_1376	63.36	15.02
SCE1572_6791	7	3
SCE1572_11514	156	45
SCE1572_6955	121	13
SCE1572_1931	325	48
SCE1572_10042	1184	1554
SCE1572_1162	72	10
SCE1572_5732	153	203
SCE1572_10988	60	328

SCE1572_5953	177	47
SCE1572_7719	109	20
SCE1572_430	249	73
SCE1572_11036	12	51
SCE1572_10037	35	31
SCE1572_10631	24	0
SCE1572_1687	41	6
SCE1572_1714	73	25
SCE1572_5308	60	36
SCE1572_8874	338	33
SCE1572_5211	251	151
SCE1572_9383	2536	4183
SCE1572_1576	97	19
SCE1572_11557	177.37	43.99
SCE1572_8435	44	1
SCE1572_6888	75	9
SCE1572_11205	2	3
SCE1572_1344	59	14
SCE1572_3721	1	0
SCE1572_10449	73	143
SCE1572_368	322	150
SCE1572_9970	21	5
SCE1572_6990	1	0
SCE1572_1159	85	15
SCE1572_7974	85	34
SCE1572_5058	71	13
SCE1572_2346	43	324
SCE1572_10367	92	20
SCE1572_276	37	8
SCE1572_9320	67	37
SCE1572_7537	9	1
SCE1572_45	83	154
SCE1572_1840	54	46
SCE1572_1360	191	248
SCE1572_9698	68	11
SCE1572_8950	139	24
SCE1572_10700	0	0
SCE1572_2362	137	91
SCE1572_11438	175	46
SCE1572_5022	105	209
SCE1572_3331	42	175
SCE1572_4207	116	132
SCE1572_10602	152	31
SCE1572_183	591	600
SCE1572_160	232	53
SCE1572_11057	28	6
SCE1572_6374	129	15
SCE1572_6914	26	8

SCE1572_5562	884	187
SCE1572_7485	375	421
SCE1572_7450	8	17
SCE1572_6892	214.57	92
SCE1572_6522	51	36
SCE1572_6422	36	19
SCE1572_9611	43	3
SCE1572_4264	50	7
SCE1572_5167	0	0
SCE1572_6675	26	4
SCE1572_10135	38	13
SCE1572_1245	12	4
SCE1572_8829	0	0
SCE1572_7196	0.83	0
SCE1572_8323	53	26
SCE1572_7810	198	152
SCE1572_5160	66	20
SCE1572_3547	0	0
SCE1572_4921	11	6
SCE1572_9327	219	199
SCE1572_4692	104	42
SCE1572_4373	51	23
SCE1572_5611	7	2
SCE1572_4064	0	0
SCE1572_7806	80	30
SCE1572_4289	82	52
SCE1572_2292	85	43
SCE1572_9764	118	47
SCE1572_443	71	8
SCE1572_10656	210	167
SCE1572_3155	122	102
SCE1572_2382	26	4
SCE1572_4908	168	123
SCE1572_1213	0	0
SCE1572_6733	78	198
SCE1572_6427	11	4
SCE1572_5433	65	6
SCE1572_6997	19	4
SCE1572_5086	70	9
SCE1572_8419	4	0
SCE1572_5286	265	44
SCE1572_1320	45	45
SCE1572_812	0	0
SCE1572_7072	175	109
SCE1572_3256	46	10
SCE1572_4688	0	0
SCE1572_6297	14	42
SCE1572_11287	73	175

SCE1572_10411	30	13
SCE1572_10206	1065.38	196.87
SCE1572_5134	10	9
SCE1572_8605	96	54
SCE1572_6363	105	366
SCE1572_10269	20	5
SCE1572_3519	17	5
SCE1572_2654	36	6
SCE1572_429	173	116
SCE1572_2758	191	238
SCE1572_653	616	845
SCE1572_6600	217	83
SCE1572_9999	53	214
SCE1572_8006	2	0
SCE1572_7667	648.22	56.9
SCE1572_786	7	1
SCE1572_9967	87	24
SCE1572_403	63	46
SCE1572_6439	38	12
SCE1572_1974	0	0.24
SCE1572_8924	0	0
SCE1572_7387	108	81
SCE1572_10628	237	293
SCE1572_8908	17	4
SCE1572_8469	155	77
SCE1572_448	55	24
SCE1572_10488	559	3012
SCE1572_10108	69	85
SCE1572_4147	24	596
SCE1572_5939	121	13
SCE1572_10192	16	2
SCE1572_1489	71	8
SCE1572_10097	26	71
SCE1572_7571	132	211
SCE1572_943	225.53	28.4
SCE1572_9807	255	420
SCE1572_1926	472	1483
SCE1572_113	91	30
SCE1572_8109	82	20
SCE1572_3288	199	189
SCE1572_4228	63	15
SCE1572_6729	5	7
SCE1572_637	19	19
SCE1572_6713	49	35
SCE1572_5767	259	654
SCE1572_8959	0	0
SCE1572_2164	4	1
SCE1572_2795	36	21

SCE1572_955	0	0
SCE1572_11505	237	614
SCE1572_7290	30	6
SCE1572_6020	199	112
SCE1572_8273	29	21
SCE1572_3459	6402	763
SCE1572_932	0	0
SCE1572_3837	154	27
SCE1572_11297	281	163
SCE1572_3979	76	46
SCE1572_10090	199.17	165.77
SCE1572_10503	14	17
SCE1572_377	173.53	28
SCE1572_9898	50	5
SCE1572_3586	27	9
SCE1572_457	21	11
SCE1572_10126	2	0
SCE1572_8430	137	108
SCE1572_10044	383	644
SCE1572_4059	38	18
SCE1572_10668	0	0
SCE1572_9110	89	33
SCE1572_1096	60	47
SCE1572_7220	33	345
SCE1572_4094	29	7
SCE1572_8491	95	32
SCE1572_343	133	58
SCE1572_10616	54	34
SCE1572_7651	185	25
SCE1572_5945	82	25
SCE1572_7779	9	2
SCE1572_3016	1	0
SCE1572_9404	32	126
SCE1572_7227	135	1797
SCE1572_5540	31	29
SCE1572_4417	12	4
SCE1572_9700	131	61
SCE1572_5184	39	1151
SCE1572_1024	45	5
SCE1572_1349	10	62
SCE1572_5547	94	11
SCE1572_3094	50	44
SCE1572_11012	115	58
SCE1572_1046	59	17
SCE1572_6384	68	23
SCE1572_8697	1	0
SCE1572_3027	87	71
SCE1572_9845	128	10

SCE1572_3384	209	356
SCE1572_968	128	273
SCE1572_2035	0	0
SCE1572_4563	4	3
SCE1572_10852	184	48
SCE1572_8083	25	7
SCE1572_1131	29	3
SCE1572_7891	109	1707
SCE1572_524	38	20
SCE1572_4716	35	7
SCE1572_3503	54	37
SCE1572_11124	36	2
SCE1572_7906	219	40
SCE1572_8316	124	69
SCE1572_4073	159	715
SCE1572_4718	42	13
SCE1572_6850	13	4
SCE1572_1581	45	24
SCE1572_9346	280	142
SCE1572_8480	73	112
SCE1572_1015	118	18
SCE1572_10326	27	4
SCE1572_2752	93	76
SCE1572_5962	0	0
SCE1572_3758	359.09	75.81
SCE1572_8066	1092	167
SCE1572_6285	65	30
SCE1572_2456	698	240
SCE1572_1150	520	2541
SCE1572_3662	51	1
SCE1572_1185	89	8
SCE1572_740	18	2
SCE1572_3970	49	50
SCE1572_10440	25	1
SCE1572_7159	63	11
SCE1572_8246	38	39
SCE1572_706	11	1
SCE1572_3109	162	16
SCE1572_7123	7	1
SCE1572_5650	1	0
SCE1572_4647	0	0
SCE1572_10110	47.05	65
SCE1572_1507	294	45
SCE1572_750	81	11
SCE1572_10167	188	106
SCE1572_7564	72	10
SCE1572_5335	138	125
SCE1572_1433	0	0

SCE1572_8147	138	27
SCE1572_4995	49	41
SCE1572_3262	29	16
SCE1572_7282	80	23
SCE1572_8653	88	27
SCE1572_3610	0	0
SCE1572_7496	0	0
SCE1572_9946	15	0
SCE1572_2991	165	50
SCE1572_5226	58	59
SCE1572_5383	144	22
SCE1572_6686	5	2
SCE1572_4050	288	312
SCE1572_7446	12	0
SCE1572_4363	65	6
SCE1572_3658	54	6
SCE1572_9942	79	18
SCE1572_7324	0	0
SCE1572_152	59	26
SCE1572_7354	230.92	28
SCE1572_6807	35	10
SCE1572_8815	2	0
SCE1572_3429	95	8
SCE1572_5303	27	7
SCE1572_7187	309	86
SCE1572_3437	48	7
SCE1572_7273	1	0
SCE1572_5569	83	25
SCE1572_9904	34	11
SCE1572_858	596	8100
SCE1572_4985	5	0
SCE1572_2786	22	32
SCE1572_4722	115	111
SCE1572_6654	1036	1978
SCE1572_11145	95	51
SCE1572_8386	307.05	59.43
SCE1572_792	109	67
SCE1572_1408	37	2
SCE1572_10235	115.6	24.77
SCE1572_6625	51	24
SCE1572_5746	84	73
SCE1572_4134	305	43
SCE1572_8009	621	15021
SCE1572_2951	0	0
SCE1572_4702	348	71
SCE1572_1986	157.03	903
SCE1572_9084	70	67
SCE1572_5412	47	12

SCE1572_7403	181	2230
SCE1572_3250	148	131
SCE1572_1018	87	188
SCE1572_9494	647	300
SCE1572_8477	413	82
SCE1572_683	40	12
SCE1572_584	70	139
SCE1572_5444	63	6
SCE1572_5189	44	290
SCE1572_514	89	30
SCE1572_1319	69	11
SCE1572_5132	96	79
SCE1572_8640	81	170
SCE1572_5659	246	125
SCE1572_1418	53	31
SCE1572_2496	17	2
SCE1572_4704	100	11
SCE1572_1833	116	36
SCE1572_6337	87	55
SCE1572_9743	324	915
SCE1572_3026	43.03	23
SCE1572_6755	45	12
SCE1572_10679	481	1046
SCE1572_7993	60	8
SCE1572_5006	184	495
SCE1572_11457	715	333
SCE1572_1746	233	61
SCE1572_5712	27	2
SCE1572_6899	42	85
SCE1572_8962	40	12
SCE1572_8150	83	11
SCE1572_5320	63	69
SCE1572_10767	92	17
SCE1572_8062	0	0
SCE1572_6273	51	33
SCE1572_999	383	141
SCE1572_10862	124	322
SCE1572_749	144	83
SCE1572_1351	119	85
SCE1572_2846	18	2
SCE1572_8725	159	25
SCE1572_9331	375	1401
SCE1572_10987	47	113
SCE1572_11170	131	48
SCE1572_11556	1	0
SCE1572_3490	39	48
SCE1572_7135	51	17
SCE1572_9928	23	18

SCE1572_8120	52	6
SCE1572_10546	3	1
SCE1572_2013	143	17
SCE1572_5157	64	11
SCE1572_8780	68	39
SCE1572_11569	19	75
SCE1572_10981	218	379
SCE1572_7703	26	43
SCE1572_1859	32	2
SCE1572_1195	1	0
SCE1572_3925	204	88
SCE1572_2252	202	83
SCE1572_8033	0	0
SCE1572_7034	103	42
SCE1572_81	3	2
SCE1572_11535	159	74
SCE1572_10674	107	127
SCE1572_4788	159	70
SCE1572_9349	148	183
SCE1572_8114	68	12
SCE1572_4406	75	8
SCE1572_8856	36	62
SCE1572_4022	70	86
SCE1572_1176	128	41
SCE1572_11389	1487	1067
SCE1572_8915	21	198
SCE1572_5276	9	0
SCE1572_1906	93	281
SCE1572_835	31	4
SCE1572_3214	0	0
SCE1572_5491	151	41
SCE1572_6635	3	0
SCE1572_7125	135	45
SCE1572_10891	81	65
SCE1572_10719	170	34
SCE1572_3010	25	3
SCE1572_8224	7	2
SCE1572_6715	88	22
SCE1572_2550	47	11
SCE1572_733	101	16
SCE1572_9315	43	16
SCE1572_8744	205	45
SCE1572_5385	458	125
SCE1572_509	85	19
SCE1572_3456	129	14
SCE1572_1800	4	2
SCE1572_6576	60	8
SCE1572_7028	52	35

SCE1572_632	249	302
SCE1572_4638	21	2
SCE1572_4759	132	35
SCE1572_7682	100	7
SCE1572_8952	44	8
SCE1572_2567	355	298
SCE1572_2702	137	91
SCE1572_6540	8	0
SCE1572_4850	103	36
SCE1572_9255	46	12
SCE1572_4899	24	8
SCE1572_10777	491	223
SCE1572_2644	111	36
SCE1572_8390	1	0
SCE1572_3735	137	21
SCE1572_7059	102	66
SCE1572_2153	46	25
SCE1572_1115	16	8
SCE1572_3048	34	2
SCE1572_3643	0	0
SCE1572_3359	237	45
SCE1572_178	44	10
SCE1572_2823	40	6
SCE1572_5484	65.53	4.91
SCE1572_6085	75	31
SCE1572_9940	0	0
SCE1572_10112	34	0
SCE1572_5582	48	12
SCE1572_9523	99	87
SCE1572_3960	78	31
SCE1572_9581	66	43
SCE1572_2250	551.97	222
SCE1572_2043	35	184
SCE1572_5668	5	0
SCE1572_7826	200	93
SCE1572_4890	496	337
SCE1572_8711	64	16
SCE1572_4177	147.7	19.18
SCE1572_11518	115	24
SCE1572_8047	41	6
SCE1572_7770	96	47
SCE1572_11022	292	91
SCE1572_1446	40	6
SCE1572_8326	138	57
SCE1572_9139	0	0
SCE1572_728	65	79
SCE1572_5088	103	71
SCE1572_9365	152	83

SCE1572_2326	23	42
SCE1572_4097	16	3
SCE1572_8111	235	27
SCE1572_6064	54	7
SCE1572_8040	172	44
SCE1572_10573	24	14
SCE1572_10146	497	248
SCE1572_6532	98	16
SCE1572_1908	60	40
SCE1572_8375	56	116
SCE1572_5516	1	0
SCE1572_9300	166	142
SCE1572_10152	195	377
SCE1572_11572	92	161
SCE1572_101	3	0
SCE1572_674	198	166
SCE1572_4503	100	29
SCE1572_9583	144	36
SCE1572_8865	198	87
SCE1572_6494	25	10
SCE1572_11376	85	47
SCE1572_3004	5	0
SCE1572_454	62	11
SCE1572_1305	30	6
SCE1572_5214	119	22
SCE1572_5564	5	0
SCE1572_9065	156	23
SCE1572_6466	41	2
SCE1572_9551	71	16
SCE1572_10420	249	175
SCE1572_10695	26	6
SCE1572_2212	28	20
SCE1572_1882	381	75
SCE1572_4901	219	53
SCE1572_1033	79	15
SCE1572_10275	52	67
SCE1572_8153	81	6
SCE1572_5042	96	94
SCE1572_8372	79	38
SCE1572_3142	49	18
SCE1572_2628	168	24
SCE1572_4458	46	17
SCE1572_5435	84	16
SCE1572_10609	298	688
SCE1572_6443	2	3
SCE1572_4136	97	14
SCE1572_6513	0	0
SCE1572_1087	6	5

SCE1572_2836	13	15
SCE1572_9197	154	35
SCE1572_2952	7.99	1
SCE1572_4575	121	17
SCE1572_5159	48	23
SCE1572_1862	246	23
SCE1572_6702	208	21
SCE1572_4547	248	19
SCE1572_31	18	11
SCE1572_4272	101	79
SCE1572_4076	7	0
SCE1572_5076	173	298
SCE1572_10022	24.68	3.05
SCE1572_7642	49	10
SCE1572_1208	132	35
SCE1572_3899	209	35
SCE1572_2429	96	59
SCE1572_4752	8	12
SCE1572_5694	53	4
SCE1572_8514	73.19	0
SCE1572_1533	14	9
SCE1572_9649	49	4
SCE1572_4663	0	0
SCE1572_11423	44	8
SCE1572_5169	11	0
SCE1572_7830	280	85
SCE1572_4173	282	136
SCE1572_10498	57	34
SCE1572_11107	135	102
SCE1572_6308	96	16
SCE1572_5529	90	37
SCE1572_1490	20	2
SCE1572_8820	66.58	12
SCE1572_5876	162	590
SCE1572_1384	426	64
SCE1572_2306	142	180
SCE1572_3060	65	4
SCE1572_3707	44	24
SCE1572_8578	307.05	59.43
SCE1572_9777	25	194
SCE1572_8475	857	289
SCE1572_3445	488	131
SCE1572_8941	82	79
SCE1572_7368	623	2088
SCE1572_398	104	13
SCE1572_7049	29	4
SCE1572_93	183	38
SCE1572_10027	91.41	31

SCE1572_8922	92	62.5
SCE1572_6539	33	2
SCE1572_1126	0	0
SCE1572_7913	80	15
SCE1572_10880	267	540
SCE1572_9884	81	23
SCE1572_9038	30	21
SCE1572_7119	45	39
SCE1572_3229	245	57
SCE1572_8354	37	3
SCE1572_4087	32	6
SCE1572_10250	134	15
SCE1572_4244	256	1217
SCE1572_4394	81	12
SCE1572_5926	691	42
SCE1572_5067	286	307
SCE1572_8187	5	1
SCE1572_688	282	303
SCE1572_10796	0	0
SCE1572_3157	139	76
SCE1572_8451	67	239
SCE1572_6025	36	18
SCE1572_10699	91	942
SCE1572_4538	337	49
SCE1572_15	17	23
SCE1572_7431	62	5
SCE1572_9322	127	45
SCE1572_6155	90	37
SCE1572_6389	116	66
SCE1572_9422	120	152
SCE1572_9186	804	123
SCE1572_7661	92	7
SCE1572_5741	70	18
SCE1572_541	155	33
SCE1572_3420	200	347
SCE1572_7639	111	68
SCE1572_1813	20	4
SCE1572_1354	71	6
SCE1572_4328	46	8
SCE1572_9329	0	0
SCE1572_11254	124	113
SCE1572_8253	42	16
SCE1572_6677	125	80
SCE1572_11228	23	104
SCE1572_2088	128	36
SCE1572_9813	19	4
SCE1572_7097	63	19
SCE1572_4524	148	14

SCE1572_5101	92	25
SCE1572_3526	25	1215
SCE1572_9225	1	0
SCE1572_7594	14	8
SCE1572_10849	94	16
SCE1572_10428	88	17
SCE1572_8440	344	188
SCE1572_7744	66	905
SCE1572_5361	78	102
SCE1572_6376	45	1
SCE1572_5911	106	75
SCE1572_9745	26	8
SCE1572_9045	249	44
SCE1572_5254	61	6
SCE1572_357	186	86
SCE1572_1660	244	360
SCE1572_2765	134	75
SCE1572_8756	450	85
SCE1572_6849	87	5
SCE1572_1254	46	8
SCE1572_2789	36	15
SCE1572_3681	10	3
SCE1572_232	3	0
SCE1572_7052	13	0
SCE1572_6722	53	74
SCE1572_5056	441	142
SCE1572_4557	69	13
SCE1572_2348	47	7
SCE1572_6012	79	68
SCE1572_3808	114	117
SCE1572_9770	174	40
SCE1572_8137	59	11
SCE1572_3140	253	199
SCE1572_4735	555	10966
SCE1572_3342	680	701
SCE1572_10921	117	54
SCE1572_3186	87	110
SCE1572_6080	196	45
SCE1572_4526	66	14
SCE1572_11098	184	77
SCE1572_5904	27	13
SCE1572_4332	38	18
SCE1572_11272	44	12
SCE1572_8449	29	20
SCE1572_1326	1	1
SCE1572_1232	30	6
SCE1572_239	6	0
SCE1572_5657	53	38

SCE1572_411	296	67
SCE1572_3392	524	303
SCE1572_5959	196	71
SCE1572_11441	37	24
SCE1572_6802	416	781
SCE1572_5120	321	1341
SCE1572_7817	39	30
SCE1572_9418	271	1302
SCE1572_9854	451	65
SCE1572_8203	8	0
SCE1572_3468	30	2
SCE1572_8559	63	27
SCE1572_1164	70	47
SCE1572_6170	22	30
SCE1572_961	139	206
SCE1572_895	25	2
SCE1572_175	40	2
SCE1572_6680	44	18
SCE1572_4470	29	3
SCE1572_10645	139	82
SCE1572_9427	14	0
SCE1572_6661	157	88
SCE1572_65	56	165
SCE1572_2686	0	0
SCE1572_5161	640	2778
SCE1572_5855	62	14
SCE1572_7894	677	0
SCE1572_7953	105	24
SCE1572_11403	28	21
SCE1572_9147	39	100
SCE1572_10474	107	55
SCE1572_4446	0	0
SCE1572_3969	308	526
SCE1572_536	343	360
SCE1572_601	399	259
SCE1572_11185	68	13
SCE1572_1291	94	28
SCE1572_2988	25	8
SCE1572_10332	365	44
SCE1572_8078	0	0
SCE1572_2507	0	0
SCE1572_8303	17	30
SCE1572_1391	96	51
SCE1572_923	188	136
SCE1572_10749	151	588
SCE1572_1694	351	49
SCE1572_1654	173	39
SCE1572_6840	24	27

SCE1572_8587	0	0
SCE1572_675	74	4
SCE1572_5761	99	29
SCE1572_10351	142	42
SCE1572_4223	60	32
SCE1572_8409	0	0
SCE1572_4423	1	0
SCE1572_7180	26	4
SCE1572_417	18	4
SCE1572_7918	190	28
SCE1572_11215	212	41
SCE1572_11318	132	72
SCE1572_3584	4	0
SCE1572_10481	160	69
SCE1572_2388	0	0
SCE1572_7007	0	0
SCE1572_11067	35	20
SCE1572_9549	81	16
SCE1572_11559	210.63	45.01
SCE1572_8132	43	7
SCE1572_1667	23	13
SCE1572_4419	241	30
SCE1572_7044	1300	4043
SCE1572_423	56	93
SCE1572_8841	208	45
SCE1572_11177	63	52
SCE1572_2400	225	953
SCE1572_8425	25	69
SCE1572_1148	81	81
SCE1572_259	12	4
SCE1572_2520	155	31
SCE1572_5482	324	191
SCE1572_2783	52	42
SCE1572_10840	28	24
SCE1572_11115	178	77
SCE1572_809	460	432
SCE1572_7647	257	32
SCE1572_5969	133	20
SCE1572_472	38	89
SCE1572_2447	60	8
SCE1572_6153	0	0
SCE1572_2965	53	5
SCE1572_983	285	477
SCE1572_7346	0	0
SCE1572_4205	63	49
SCE1572_1421	160	22
SCE1572_5884	144	59
SCE1572_6571	13.25	1.56

SCE1572_11465	145	36
SCE1572_10155	62	20
SCE1572_7777	25	9
SCE1572_2969	184.81	8.81
SCE1572_5358	160	199
SCE1572_5805	40	214
SCE1572_9565	139	27
SCE1572_7795	41	21
SCE1572_4598	315	356
SCE1572_8170	84	7
SCE1572_3868	372	74
SCE1572_11348	38	20
SCE1572_9601	554	641
SCE1572_5754	51	43
SCE1572_3267	118	161
SCE1572_6382	156	32
SCE1572_3430	63	76
SCE1572_7457	19	1
SCE1572_1275	14	11
SCE1572_3567	68	7
SCE1572_3761	218	53
SCE1572_8232	46	29
SCE1572_5670	466	90
SCE1572_9678	49	10
SCE1572_7460	37	14
SCE1572_4870	13	49
SCE1572_11355	111	28
SCE1572_4510	243	100
SCE1572_599	50	13
SCE1572_11585	8	0
SCE1572_5933	95	16
SCE1572_5895	407	34
SCE1572_4020	39	40
SCE1572_7021	50.55	8.24
SCE1572_6036	7	0
SCE1572_3325	335	398
SCE1572_1241	33	17
SCE1572_3080	63	135
SCE1572_1009	77	19
SCE1572_2993	224	92
SCE1572_2922	90	14
SCE1572_9354	105	46
SCE1572_629	151	189
SCE1572_4365	211	22
SCE1572_6283	28	29
SCE1572_7532	13.02	3.43
SCE1572_4124	34	12
SCE1572_4794	1338	1238

SCE1572_5575	8	6
SCE1572_5649	517	280
SCE1572_6271	6	7
SCE1572_7174	0	0
SCE1572_7110	0	0
SCE1572_3535	65	7
SCE1572_8335	0	0
SCE1572_11595	1507	2268
SCE1572_10281	113	225
SCE1572_5840	25	1
SCE1572_163	296	64
SCE1572_2869	13	27
SCE1572_7712	41	21
SCE1572_6404	9	0
SCE1572_821	18	3
SCE1572_4386	51	8
SCE1572_6800	915	759
SCE1572_3900	178	98
SCE1572_7363	626	235
SCE1572_192	1	0
SCE1572_7756	25	4
SCE1572_2778	57	33
SCE1572_2434	149	47
SCE1572_2662	37	62
SCE1572_4053	128	220
SCE1572_622	0	0
SCE1572_2852	0	0
SCE1572_9130	31	6
SCE1572_6059	467	1651
SCE1572_1602	60	15
SCE1572_8024	8	3
SCE1572_5686	35	27
SCE1572_4832	0	0
SCE1572_2919	66	26
SCE1572_10578	0	0
SCE1572_382	16	2
SCE1572_718	26	7
SCE1572_4531	116	32
SCE1572_10909	129	58
SCE1572_8795	43	19
SCE1572_5630	213	300
SCE1572_11566	134	46
SCE1572_4742	27	32
SCE1572_3164	211	34
SCE1572_7041	68	6
SCE1572_8616	115	33
SCE1572_4867	0	0
SCE1572_9129	116	30

SCE1572_7963	207	343
SCE1572_4026	264	318
SCE1572_7506	338.88	43.87
SCE1572_6606	123	25
SCE1572_5618	85	12
SCE1572_2118	16	3
SCE1572_9150	44	117
SCE1572_2419	717	1719
SCE1572_3907	28	4
SCE1572_11094	168	35
SCE1572_2947	0	0
SCE1572_6872	78	52
SCE1572_4315	35	4
SCE1572_3997	186	290
SCE1572_11049	134	50
SCE1572_2669	153	31
SCE1572_7720	66	153
SCE1572_7999	7	1
SCE1572_5759	28	0
SCE1572_3113	291	31
SCE1572_11430	323	489
SCE1572_6145	53	7
SCE1572_10621	168	69
SCE1572_3064	117	17
SCE1572_6764	140	122
SCE1572_9159	45	6
SCE1572_9486	31	21
SCE1572_5442	61	13
SCE1572_2008	173	12
SCE1572_557	109	61
SCE1572_9456	0	0
SCE1572_6793	209	283
SCE1572_4262	105	22
SCE1572_6618	36	11
SCE1572_1256	145	49
SCE1572_8662	90	29
SCE1572_762	568	75
SCE1572_7237	435	1968
SCE1572_668	55	23
SCE1572_7162	92	25
SCE1572_8168	46	6
SCE1572_7192	27	1
SCE1572_1073	93	22
SCE1572_1588	24.39	4.8
SCE1572_9119	52	7
SCE1572_8095	17	1
SCE1572_4337	0	0
SCE1572_4305	211	54

SCE1572_10387	86	26
SCE1572_10705	32	3
SCE1572_9122	399	74
SCE1572_2943	3	0
SCE1572_3119	12	28
SCE1572_4445	135	177
SCE1572_4339	47	11
SCE1572_11008	21	19
SCE1572_1611	13	88
SCE1572_7632	1821	6824
SCE1572_11416	46	40
SCE1572_10734	41	10
SCE1572_6646	28	6
SCE1572_548	195	28
SCE1572_337	162	33
SCE1572_7634	238	898
SCE1572_11544	111	14
SCE1572_517	106	29
SCE1572_11266	692	1592
SCE1572_7518	647	1486
SCE1572_10371	47	19
SCE1572_1510	79	16
SCE1572_11248	64	14
SCE1572_7946	548	140
SCE1572_9918	129	26
SCE1572_8508	65	36
SCE1572_11322	27	11
SCE1572_592	42	46
SCE1572_2482	101	67
SCE1572_9831	22	6
SCE1572_9511	1	0
SCE1572_2897	13	0
SCE1572_4860	31	0
SCE1572_3775	33	14
SCE1572_1636	34	159
SCE1572_3274	63	18
SCE1572_8521	0	0
SCE1572_1805	15	6
SCE1572_9975	98	26
SCE1572_1618	70	33
SCE1572_7805	200	107
SCE1572_8981	0	0
SCE1572_6774	63	16
SCE1572_2676	32	154
SCE1572_3305	209	125
SCE1572_10105	25	10
SCE1572_7727	207	206
SCE1572_4565	132	126

SCE1572_841	161	114
SCE1572_11383	60	27
SCE1572_6426	36	4
SCE1572_2527	296	24
SCE1572_9090	28	2
SCE1572_6179	139	1214
SCE1572_11087	30	54
SCE1572_6244	48	5
SCE1572_5351	13	3
SCE1572_142	37.13	3
SCE1572_341	181	49
SCE1572_4823	42	30
SCE1572_348	57	190
SCE1572_315	627	104
SCE1572_6118	45	4
SCE1572_8493	16	3
SCE1572_7392	6.93	0
SCE1572_8286	31	36
SCE1572_6226	135	68
SCE1572_1521	43	6
SCE1572_7130	218	57
SCE1572_3878	232	78
SCE1572_10316	0	0
SCE1572_2127	9	1
SCE1572_5661	15	5
SCE1572_1456	24	5
SCE1572_2410	179	139
SCE1572_11345	111	129
SCE1572_4877	39	14
SCE1572_4213	405	621
SCE1572_7136	99	12
SCE1572_8988	4	0
SCE1572_205	139	29
SCE1572_9181	158	30
SCE1572_298	54	27
SCE1572_6033	617	400
SCE1572_882	263	118
SCE1572_8638	65	74
SCE1572_7990	0	0
SCE1572_9024	46	175
SCE1572_3605	743	169
SCE1572_2717	61	13
SCE1572_9697	26	16
SCE1572_125	94	16
SCE1572_10294	215	286
SCE1572_1924	198	177
SCE1572_6334	167	26
SCE1572_3881	24	3

SCE1572_6131	76	215
SCE1572_6113	39	35
SCE1572_4805	51	17
SCE1572_1575	52	21
SCE1572_43	18	5
SCE1572_3789	1216	4468
SCE1572_10966	49	9
SCE1572_10795	3	4
SCE1572_7698	75	19
SCE1572_199	102	73
SCE1572_7177	37	13
SCE1572_6433	15	4
SCE1572_11236	57	9
SCE1572_9237	72	12
SCE1572_6343	108	150
SCE1572_5677	65	40
SCE1572_1789	12	0
SCE1572_4761	0	0
SCE1572_8979	0	0
SCE1572_7019	433	118
SCE1572_2368	35	4
SCE1572_2370	58	58
SCE1572_7987	52	34
SCE1572_9020	0	0
SCE1572_312	86	18
SCE1572_3178	3	0
SCE1572_8881	148	30
SCE1572_8898	3	0
SCE1572_6789	228	46
SCE1572_5993	107	41
SCE1572_9470	85	221
SCE1572_7241	423	149
SCE1572_2130	14	1
SCE1572_5425	213	140
SCE1572_5877	45	31
SCE1572_3555	193	36
SCE1572_267	434	103
SCE1572_6048	358	329
SCE1572_3890	63	16
SCE1572_9684	332	444
SCE1572_9726	14	5
SCE1572_3791	133	46
SCE1572_1316	151	54
SCE1572_9290	228	385
SCE1572_2067	68	30
SCE1572_9111	49	3
SCE1572_9175	1153	727
SCE1572_1769	66	10

SCE1572_5609	75	8
SCE1572_695	6	9
SCE1572_609	0	0
SCE1572_3230	66	81
SCE1572_6477	23	5
SCE1572_7230	37	14
SCE1572_3395	20	2
SCE1572_8667	54	16
SCE1572_4517	105	22
SCE1572_9299	281	1236
SCE1572_3570	96	17
SCE1572_1449	30	14
SCE1572_2862	4	0
SCE1572_6930	18	0
SCE1572_3947	3631	3473
SCE1572_2696	43	12
SCE1572_7426	48	64
SCE1572_7477	34	54
SCE1572_5831	51	23
SCE1572_1552	94	30
SCE1572_8276	91	41
SCE1572_7264	99	19
SCE1572_6743	7	1
SCE1572_6005	75	66
SCE1572_10690	133	90
SCE1572_323	95	45
SCE1572_5125	107	9
SCE1572_2111	118	221
SCE1572_9054	142	31
SCE1572_4997	65	41
SCE1572_9874	71	13
SCE1572_8770	2	0
SCE1572_3886	77	22
SCE1572_5333	28	12
SCE1572_743	43	14
SCE1572_3719	66	443
SCE1572_106	141	15
SCE1572_3509	227	53
SCE1572_977	329	1764
SCE1572_6974	97	30
SCE1572_3826	342	92
SCE1572_7546	171	68
SCE1572_9781	16	0
SCE1572_1696	52	10
SCE1572_9203	54	26
SCE1572_2139	61	7
SCE1572_8297	803	678
SCE1572_3181	387	1105

SCE1572_8530	112	71
SCE1572_2902	35	7
SCE1572_8679	47	16
SCE1572_4102	2	0
SCE1572_10346	119	60
SCE1572_5260	95	550
SCE1572_3727	123	19
SCE1572_5247	100	19
SCE1572_5196	11	1
SCE1572_1998	120	17
SCE1572_6581	184	85
SCE1572_11329	159	163
SCE1572_1945	70	18
SCE1572_3415	68	222
SCE1572_2859	10	2
SCE1572_9218	26	9
SCE1572_6939	70	7
SCE1572_10888	11	0
SCE1572_1173	104	12
SCE1572_11455	232	64
SCE1572_10358	143	27
SCE1572_1647	170.15	120.42
SCE1572_9985	14	4
SCE1572_903	123	16
SCE1572_2128	236	34
SCE1572_2221	3	0
SCE1572_7407	0	0
SCE1572_10560	1	0
SCE1572_5115	74	58
SCE1572_7332	278	57
SCE1572_5951	26	6
SCE1572_1438	33	9
SCE1572_4911	240	231
SCE1572_1598	84.31	14
SCE1572_11312	0	0
SCE1572_4615	131	45
SCE1572_11428	43	9
SCE1572_9642	15	14
SCE1572_5706	83	115
SCE1572_9622	52	17
SCE1572_10175	257	208
SCE1572_7334	313	778
SCE1572_1889	244	895
SCE1572_10254	0	0
SCE1572_1133	19	10
SCE1572_3664	112	10
SCE1572_4150	96	47
SCE1572_1651	81	4

SCE1572_5208	8	0
SCE1572_1328	89	7
SCE1572_2895	35	21
SCE1572_2974	0	0
SCE1572_9702	51	11
SCE1572_2454	165	223
SCE1572_7382	0	0
SCE1572_4006	164	63
SCE1572_4235	37	6
SCE1572_9936	110	127
SCE1572_837	47	20
SCE1572_4652	103	48
SCE1572_1420	64	44
SCE1572_7080	68	139
SCE1572_8652	133	189
SCE1572_4188	139	208
SCE1572_7204	10	0
SCE1572_9373	83	11
SCE1572_849	1804	1782
SCE1572_1875	261	73
SCE1572_9284	0	0
SCE1572_5372	339	1890
SCE1572_5178	76	26
SCE1572_2073	56	4
SCE1572_8706	52	33
SCE1572_6592	52	58
SCE1572_7836	40	36
SCE1572_11591	160	58
SCE1572_8539	39	17
SCE1572_3835	165	28
SCE1572_6830	3	0
SCE1572_11030	50	43
SCE1572_3266	97	97
SCE1572_5948	155	40
SCE1572_148	18	4
SCE1572_23	39	51
SCE1572_5947	125	35
SCE1572_948	165.53	28.33
SCE1572_2338	50	84
SCE1572_4224	14	0
SCE1572_7888	98	45
SCE1572_1562	31	5
SCE1572_5532	54	11
SCE1572_10132	103	34
SCE1572_775	124	100
SCE1572_2070	40	3
SCE1572_9183	162	33
SCE1572_6949	89	17

SCE1572_5121	86	18
SCE1572_1874	20	7
SCE1572_8937	3175	11362
SCE1572_6818	16	3
SCE1572_10945	49	19
SCE1572_3281	38	24
SCE1572_5527	262	76
SCE1572_4618	185	59
SCE1572_3833	84	12
SCE1572_11166	54	62
SCE1572_1477	205	43
SCE1572_8127	305	61
SCE1572_8824	22.78	133.1
SCE1572_7061	0	0
SCE1572_2817	0	0
SCE1572_9804	0	0
SCE1572_871	350	288
SCE1572_6585	614	525
SCE1572_4607	24	14
SCE1572_9906	162	54
SCE1572_8011	85	103
SCE1572_7417	100	52
SCE1572_7349	89	18
SCE1572_9032	122	31
SCE1572_7874	36	23
SCE1572_6923	212	53
SCE1572_6015	75	9
SCE1572_7429	91	14
SCE1572_4005	20	14
SCE1572_1787	46	9
SCE1572_2514	8	0
SCE1572_5459	77	11
SCE1572_3630	111	81
SCE1572_8769	683	97
SCE1572_3935	52	16
SCE1572_5414	82	15
SCE1572_11016	3	0
SCE1572_7544	30	0
SCE1572_6671	74	12
SCE1572_7073	132	18
SCE1572_6329	87	30
SCE1572_64	104	72
SCE1572_5977	43	11
SCE1572_8807	22	1
SCE1572_6839	47	6.76
SCE1572_7760	245	97
SCE1572_4711	180	64
SCE1572_3348	27	7

SCE1572_1029	32	79
SCE1572_462	68	37
SCE1572_11038	0	0
SCE1572_10520	182	55
SCE1572_5154	31	9
SCE1572_6555	314	59
SCE1572_6123	224	142
SCE1572_5496	120	30
SCE1572_3450	65	11
SCE1572_4001	123	839
SCE1572_1269	107	38
SCE1572_4463	122	26
SCE1572_2265	108	90
SCE1572_1933	83	129
SCE1572_8218	199	48
SCE1572_1243	58	60
SCE1572_7233	56	15
SCE1572_5140	41	11
SCE1572_3120	39	9
SCE1572_8811	20	2
SCE1572_7929	78	83
SCE1572_8672	61	28
SCE1572_10059	1437	4009
SCE1572_2486	122	81
SCE1572_5570	1223.25	282.83
SCE1572_1716	0	0
SCE1572_7296	57	30
SCE1572_1286	907	6301
SCE1572_4143	51	12
SCE1572_10838	91	39
SCE1572_11467	212	235
SCE1572_3123	180	38
SCE1572_3810	57	14
SCE1572_1374	158	138
SCE1572_5194	7	3
SCE1572_11300	75	63
SCE1572_8122	29	4
SCE1572_3637	0	0
SCE1572_2420	1263	1893
SCE1572_8093	90	15
SCE1572_7376	107	38
SCE1572_5072	61	21
SCE1572_6105	31	0
SCE1572_9149	160	189
SCE1572_6795	238	168
SCE1572_11116	17	7
SCE1572_9809	28	20
SCE1572_1541	0	0

SCE1572_8590	115	167
SCE1572_4120	681	3058
SCE1572_4617	42	2
SCE1572_1565	107	44
SCE1572_9165	0	0
SCE1572_4976	520	2332
SCE1572_3465	0	0
SCE1572_11582	196	87
SCE1572_10816	51	7
SCE1572_2694	30	69
SCE1572_6704	78	102
SCE1572_2207	39	8
SCE1572_3780	11	12
SCE1572_7	176	285
SCE1572_5340	82	44
SCE1572_11554	112	7
SCE1572_71	61	15
SCE1572_7096	184	36
SCE1572_3671	24	13
SCE1572_8200	30	2
SCE1572_1544	102	17
SCE1572_970	79	42
SCE1572_6418	281	1469
SCE1572_1379	59	10
SCE1572_10013	43	6
SCE1572_7317	12	2
SCE1572_915	3	1
SCE1572_8692	0	0
SCE1572_5477	71	362
SCE1572_795	455	358
SCE1572_11497	16	0
SCE1572_1571	19	3
SCE1572_4127	0	0
SCE1572_9636	260	147
SCE1572_8368	55	4
SCE1572_4962	60	110
SCE1572_4857	64	17
SCE1572_11183	112	89
SCE1572_9518	102	87
SCE1572_11306	161	163
SCE1572_8265	92	59
SCE1572_4350	155	31
SCE1572_4031	1672	738
SCE1572_6200	143	95
SCE1572_4401	11	0
SCE1572_1229	172	74
SCE1572_2408	159	386
SCE1572_2593	0	0

SCE1572_6038	26	9
SCE1572_10076	225	246
SCE1572_10543	0	0
SCE1572_9672	105	119
SCE1572_575	1	0
SCE1572_8018	14	6
SCE1572_10896	5	0
SCE1572_5710	21	1
SCE1572_2223	95	161
SCE1572_10586	0	0
SCE1572_1362	391	741
SCE1572_8990	254	2081
SCE1572_10806	709	73
SCE1572_3669	0	0
SCE1572_1578	65	11
SCE1572_8503	69	5
SCE1572_9446	74	37
SCE1572_7700	79	10
SCE1572_570	174	217
SCE1572_9127	51	35
SCE1572_5203	60	158
SCE1572_788	332	169
SCE1572_10755	128	26
SCE1572_4690	101	65
SCE1572_5820	380.57	147.8
SCE1572_195	2	3
SCE1572_7850	250	160
SCE1572_5356	0	0
SCE1572_4298	112	36
SCE1572_7842	206	142
SCE1572_9792	18	2
SCE1572_4389	61	7
SCE1572_2190	56	71
SCE1572_9862	53	18
SCE1572_9955	123	20
SCE1572_4771	99	21
SCE1572_2841	15	4
SCE1572_4815	159	306
SCE1572_4105	45	7
SCE1572_648	254	162
SCE1572_2865	3	0
SCE1572_10221	351	52
SCE1572_8152	7	0
SCE1572_4625	43	8
SCE1572_11307	100	89
SCE1572_4678	5	0
SCE1572_3680	53.48	7
SCE1572_4846	67	63

SCE1572_7342	217	510
SCE1572_10731	62	40
SCE1572_10431	124	36
SCE1572_6886	23	8
SCE1572_339	31	14
SCE1572_351	53	59
SCE1572_2303	75	95
SCE1572_11009	145	59
SCE1572_2477	202	20
SCE1572_386	2	1
SCE1572_3916	175	76
SCE1572_10223	176	109
SCE1572_9273	134	69
SCE1572_10315	11	13
SCE1572_2197	557	212
SCE1572_7677	122	15
SCE1572_6353	84	24
SCE1572_5847	103	54
SCE1572_7645	89	30
SCE1572_396	0	0
SCE1572_1263	226	1492
SCE1572_8930	512	211
SCE1572_8718	95	54
SCE1572_646	0	0
SCE1572_116	91	65
SCE1572_4107	45	11
SCE1572_7978	263	86
SCE1572_5941	88	13
SCE1572_1961	183	23
SCE1572_1002	32	3
SCE1572_9336	17	14
SCE1572_3473	484	132
SCE1572_10099	72	52
SCE1572_9650	66	12
SCE1572_5825	101	40
SCE1572_9924	1008.79	1239
SCE1572_5598	119	145
SCE1572_5299	140	22
SCE1572_3541	0	0
SCE1572_10403	178	153
SCE1572_7289	188	23
SCE1572_6165	109	88
SCE1572_3066	25	5
SCE1572_8619	57	15
SCE1572_4157	7	2
SCE1572_1084	32	10
SCE1572_2081	248	47
SCE1572_4493	123	90

SCE1572_8762	216	47
SCE1572_10438	29	15
SCE1572_7493	127	20
SCE1572_2465	40	22
SCE1572_466	96	30
SCE1572_3197	30	8
SCE1572_6827	138	304
SCE1572_6934	197	190
SCE1572_7467	122	41
SCE1572_2244	111	31
SCE1572_7579	270	130
SCE1572_5545	36	13
SCE1572_10693	100	28
SCE1572_8314	327	151
SCE1572_2311	437	350
SCE1572_8612	97	11
SCE1572_11147	294	43
SCE1572_3204	87	49
SCE1572_6189	73	25
SCE1572_11522	72	12
SCE1572_626	43	19
SCE1572_7092	15	23
SCE1572_8149	38	8
SCE1572_157	227	566
SCE1572_6805	135	53
SCE1572_5015	320	3625
SCE1572_246	5	2
SCE1572_6876	15	14
SCE1572_6947	60	14
SCE1572_4648	46	10
SCE1572_3300	63	11
SCE1572_4322	165	26
SCE1572_7413	125.28	82.27
SCE1572_345	50	8
SCE1572_3174	99	68
SCE1572_1032	29.69	10.26
SCE1572_3024	152	31
SCE1572_4045	130	44
SCE1572_10955	704	802
SCE1572_10092	216	71
SCE1572_6989	16	7
SCE1572_7126	147	28
SCE1572_6902	162	12
SCE1572_11542	138	14
SCE1572_10244	8	0
SCE1572_1039	49	4
SCE1572_11361	89	51
SCE1572_5394	618	120

SCE1572_7001	254	100
SCE1572_1928	196	188
SCE1572_9659	270	156
SCE1572_10292	103	64
SCE1572_11372	113	183
SCE1572_10501	28	21
SCE1572_10469	48	71
SCE1572_7153	194	23
SCE1572_5337	381	70
SCE1572_4586	780	623
SCE1572_10442	128	17
SCE1572_1867	171	47
SCE1572_4092	78	13
SCE1572_3522	0	0
SCE1572_9718	184	257
SCE1572_8100	135	74
SCE1572_11051	136	442
SCE1572_5416	43	5
SCE1572_4826	628	339
SCE1572_1192	128	33
SCE1572_3500	286	109
SCE1572_10115	196.6	119
SCE1572_3036	94	9
SCE1572_3078	193	88
SCE1572_2318	27	0
SCE1572_9823	519	55
SCE1572_1406	347	236
SCE1572_7068	88	22
SCE1572_5314	0	0
SCE1572_10715	257	79
SCE1572_2090	7	0
SCE1572_11243	21	20
SCE1572_4940	260	1276
SCE1572_4118	50	2572
SCE1572_1558	147	24
SCE1572_5224	80	54
SCE1572_10858	1355	918
SCE1572_7747	93	52
SCE1572_11219	129	31
SCE1572_6498	225	344
SCE1572_10173	419	176
SCE1572_4019	83	42
SCE1572_8116	0	0
SCE1572_9140	60	11
SCE1572_2259	204	26
SCE1572_10910	221.43	20
SCE1572_10689	7	3
SCE1572_7190	94	48

SCE1572_9344	74	89
SCE1572_3439	53	17
SCE1572_10231	267	40
SCE1572_8189	63	20
SCE1572_10129	23	1
SCE1572_5468	496	87
SCE1572_8592	272	115
SCE1572_6688	368	49
SCE1572_2262	136	37
SCE1572_1587	22	0
SCE1572_8244	435	639
SCE1572_4709	129	9
SCE1572_3382	23	28
SCE1572_8088	185	19
SCE1572_8813	0	0
SCE1572_7356	279	119
SCE1572_8655	592	2512
SCE1572_7819	355	542
SCE1572_8227	62	93
SCE1572_7669	121	15
SCE1572_3496	195	112
SCE1572_3695	14	24
SCE1572_2549	0	0
SCE1572_8482	10	30
SCE1572_975	409	2722
SCE1572_7695	34	18
SCE1572_1331	89	25
SCE1572_3107	36	13
SCE1572_9343	235	848
SCE1572_5259	44	15
SCE1572_7280	39	6
SCE1572_4987	228	120
SCE1572_5451	296	54
SCE1572_2033	279.35	238.09
SCE1572_11425	305	178
SCE1572_7365	318	296
SCE1572_1748	383	142
SCE1572_8081	2	0
SCE1572_10527	63	25
SCE1572_8038	201	23
SCE1572_407	71	222
SCE1572_9948	153	18
SCE1572_4473	36	32
SCE1572_7898	75	79
SCE1572_1451	59	6
SCE1572_6098	194	83
SCE1572_6719	100	306
SCE1572_10233	17	2

SCE1572_3295	21	10
SCE1572_3787	111	73
SCE1572_586	166	56
SCE1572_8388	47	8
SCE1572_11126	95	31
SCE1572_2579	81	18
SCE1572_1505	43	5
SCE1572_11598	91	446
SCE1572_3401	15	3
SCE1572_856	0	0
SCE1572_11157	123.03	20.91
SCE1572_1363	102	221
SCE1572_4700	45	9
SCE1572_5008	221	295
SCE1572_4923	30	6
SCE1572_6864	1	0
SCE1572_4952	28	1
SCE1572_1629	5	1
SCE1572_8056	0	0
SCE1572_2998	79	29
SCE1572_1338	225	67
SCE1572_534	0	0
SCE1572_5084	183	1356
SCE1572_10864	26	34
SCE1572_4670	150	19
SCE1572_4408	43	5
SCE1572_7960	108	84
SCE1572_7920	293	60
SCE1572_6928	114	51
SCE1572_11482	77	119
SCE1572_5130	38	29
SCE1572_8999	249	492
SCE1572_6207	36.19	3.28
SCE1572_747	102	15
SCE1572_7318	69	24
SCE1572_5697	371	314
SCE1572_2626	48	22
SCE1572_8713	160	42
SCE1572_3408	165	108
SCE1572_6211	64	5
SCE1572_4720	156	25
SCE1572_5782	13	10
SCE1572_8210	70	45
SCE1572_8460	34	62
SCE1572_9741	162	346
SCE1572_1894	136	24
SCE1572_10738	293	59
SCE1572_9990	49	18

SCE1572_1915	485	94
SCE1572_7209	79	45
SCE1572_6968	82	178
SCE1572_5729	76	35
SCE1572_8852	156	32
SCE1572_3618	0	0
SCE1572_9891	56	1
SCE1572_1847	176	30
SCE1572_781	49	22
SCE1572_1724	104	206
SCE1572_5230	542	1248
SCE1572_4683	252	68
SCE1572_9081	69	4
SCE1572_5280	108	66
SCE1572_7658	0	0
SCE1572_6961	0	0
SCE1572_8854	50	93
SCE1572_5807	32	10
SCE1572_1152	17	7
SCE1572_10165	49	10
SCE1572_5520	42	26
SCE1572_11499	55	13
SCE1572_4959	0	0
SCE1572_2116	20	2
SCE1572_11538	63	396
SCE1572_8060	38	1
SCE1572_7558	57	1
SCE1572_5864	12	5
SCE1572_3367	209	1572
SCE1572_6339	29	35
SCE1572_8893	0	0
SCE1572_11490	0	10.33
SCE1572_11323	168	203
SCE1572_1167	9	17
SCE1572_5367	22	16
SCE1572_2151	99	8
SCE1572_9082	35	0
SCE1572_8945	237	496
SCE1572_10487	1469	19720
SCE1572_9834	110	20
SCE1572_8804	36	93
SCE1572_8902	102	23
SCE1572_1310	160	613
SCE1572_8191	374	65
SCE1572_8064	19	0
SCE1572_3333	272	239
SCE1572_8031	315.48	100.04
SCE1572_8256	31	24

SCE1572_708	76	53
SCE1572_10649	34	2
SCE1572_5833	209	181
SCE1572_4727	116	25
SCE1572_7808	521	175
SCE1572_10039	22	12
SCE1572_254	0	0
SCE1572_6367	44	87
SCE1572_7707	159	45
SCE1572_11059	205.3	19.5
SCE1572_2556	0	0
SCE1572_89	928	466
SCE1572_823	43	6
SCE1572_10365	110	134
SCE1572_1486	87	53
SCE1572_5720	42	9
SCE1572_274	0	0
SCE1572_8359	0	0
SCE1572_5250	248	510
SCE1572_9204	149	104
SCE1572_6460	134	25
SCE1572_7225	282	98
SCE1572_3279	91	62
SCE1572_3254	26	24
SCE1572_1215	63	2
SCE1572_6980	56	11
SCE1572_10061	1734	4584
SCE1572_4971	0	0
SCE1572_10259	21	3
SCE1572_8835	80	11
SCE1572_5955	0	0
SCE1572_1198	332	47
SCE1572_8437	1	6
SCE1572_4927	8	0
SCE1572_2096	30	39
SCE1572_6905	41	5
SCE1572_5882	75	28
SCE1572_6445	47	9
SCE1572_8555	11	56
SCE1572_9163	19	1
SCE1572_9120	19	7
SCE1572_10711	20	0
SCE1572_2277	263	881
SCE1572_5730	0	0
SCE1572_8432	19	2
SCE1572_1342	358	513
SCE1572_1108	122	14
SCE1572_7530	99	32

SCE1572_111	84	21
SCE1572_3770	0	0
SCE1572_5024	175	48
SCE1572_6420	391	217
SCE1572_11221	4	0
SCE1572_5053	157	48
SCE1572_965	15	0
SCE1572_10626	91	98
SCE1572_2393	113	275
SCE1572_5914	64	81
SCE1572_9533	118	52
SCE1572_9014	221	371
SCE1572_10970	424	477
SCE1572_10600	53	12
SCE1572_10085	272	320
SCE1572_3100	34	5
SCE1572_6821	70	9
SCE1572_7628	40	15
SCE1572_5037	219	655
SCE1572_7740	95	73
SCE1572_2737	59	34
SCE1572_6735	145	212
SCE1572_1425	110	55
SCE1572_4733	25	14
SCE1572_2700	13	2
SCE1572_1842	127.88	8
SCE1572_9618	37	16
SCE1572_5865	272	71
SCE1572_5756	86	13
SCE1572_162	88	21
SCE1572_10519	187	41
SCE1572_7513	31	4
SCE1572_432	132	249
SCE1572_10985	1496	1782
SCE1572_11399	100	68
SCE1572_8397	210	48
SCE1572_3236	296	920
SCE1572_569	75	49
SCE1572_6712	174	308
SCE1572_980	37	13
SCE1572_945	185.33	15.21
SCE1572_4483	91	59
SCE1572_5744	259	83
SCE1572_11289	0	0
SCE1572_9402	816	1602
SCE1572_6728	21	23
SCE1572_5674	18	3
SCE1572_503	16	2

SCE1572_1048	30	22
SCE1572_1604	85	23
SCE1572_10512	155	152
SCE1572_3977	113	32
SCE1572_10201	58	51
SCE1572_7535	1641	198
SCE1572_10684	24	3
SCE1572_6602	18	5
SCE1572_2793	68	3
SCE1572_4253	1	0
SCE1572_10871	713.92	113.51
SCE1572_1622	41	107
SCE1572_8321	180	55
SCE1572_185	69	6
SCE1572_650	1980.64	1425.18
SCE1572_9797	14	7
SCE1572_1637	31	11
SCE1572_6215	105	16
SCE1572_2615	93	39
SCE1572_7611	38	44
SCE1572_475	117	513
SCE1572_2656	254	58
SCE1572_9436	1325	1714
SCE1572_635	325	853
SCE1572_10267	130	90
SCE1572_1632	40	22
SCE1572_5284	51	51
SCE1572_4057	1	0
SCE1572_1470	222.29	80.99
SCE1572_2756	1	0
SCE1572_400	17	6
SCE1572_5013	85	76
SCE1572_6953	42	11
SCE1572_5556	1261.75	821.75
SCE1572_10859	534	635
SCE1572_1658	34	5
SCE1572_4932	524	894
SCE1572_9965	0	0
SCE1572_6542	102	68
SCE1572_359	191	47
SCE1572_7774	3	0
SCE1572_401	54	19
SCE1572_11549	73	18
SCE1572_8023	7	0
SCE1572_8404	22	2
SCE1572_3422	139	75
SCE1572_6475	276	659
SCE1572_10727	141	39

SCE1572_11507	41	7
SCE1572_953	43	18
SCE1572_5930	0	0
SCE1572_7214	54	73
SCE1572_6073	1	0
SCE1572_3803	138	20
SCE1572_2166	400	61
SCE1572_6391	0	0
SCE1572_1683	6	106
SCE1572_206	0	0
SCE1572_4345	35	16
SCE1572_11295	10	10
SCE1572_11150	96	57
SCE1572_1022	77	15
SCE1572_284	22	10
SCE1572_8043	0	0
SCE1572_1013	109	23
SCE1572_6360	73	112
SCE1572_1702	56	25
SCE1572_5589	143	47
SCE1572_3554	16	2
SCE1572_6361	250	58
SCE1572_9962	1	0
SCE1572_375	45	6
SCE1572_3360	100	34
SCE1572_1382	2	0
SCE1572_496	80	32
SCE1572_4906	29	16
SCE1572_10279	23	0
SCE1572_7742	141	100
SCE1572_1958	140	72
SCE1572_2484	84	64
SCE1572_5543	38	2
SCE1572_11096	143	34
SCE1572_3840	42	6
SCE1572_11175	205	187
SCE1572_9050	61	29
SCE1572_4536	80	142
SCE1572_2141	40	1
SCE1572_363	135	19
SCE1572_2979	52	8
SCE1572_6042	130	2064
SCE1572_139	27	27
SCE1572_10363	177	245
SCE1572_2086	123	69
SCE1572_7149	13	23
SCE1572_2884	73	43
SCE1572_7256	121	103

SCE1572_7012	0	0
SCE1572_10071	674	503
SCE1572_9429	25	7
SCE1572_6087	170	80
SCE1572_1454	65	15
SCE1572_5163	0	0
SCE1572_2811	65	2
SCE1572_6963	112	124
SCE1572_11528	45	4
SCE1572_2771	2	0
SCE1572_8139	30	38
SCE1572_8427	54	3
SCE1572_6847	81	2
SCE1572_18	237	337
SCE1572_9852	62	31
SCE1572_8582	52	15
SCE1572_3980	132	95
SCE1572_1717	41	53
SCE1572_713	142	77
SCE1572_4522	127	23
SCE1572_3892	86	66
SCE1572_1887	255	247
SCE1572_2458	119	163
SCE1572_3340	386	8414
SCE1572_2148	181.89	22
SCE1572_559	27	340
SCE1572_2023	139	60
SCE1572_4729	38	10
SCE1572_10328	159	19
SCE1572_11069	175	69
SCE1572_5035	63	19
SCE1572_8484	66	56
SCE1572_10964	134	31
SCE1572_3188	93	8
SCE1572_10046	343	1020
SCE1572_660	3122	3751
SCE1572_5654	263	1255
SCE1572_7637	146	861
SCE1572_10703	92	184
SCE1572_3683	80	7
SCE1572_2729	101	30
SCE1572_7670	8	1
SCE1572_6172	1	0
SCE1572_10213	9	0
SCE1572_5060	181	201
SCE1572_3517	115	18
SCE1572_2945	0	0
SCE1572_2730	73	22

SCE1572_10334	56	22
SCE1572_8557	79	16
SCE1572_2343	50	15
SCE1572_7160	37	8
SCE1572_2076	177	81
SCE1572_7855	67	48
SCE1572_4309	73	12
SCE1572_1238	36	8
SCE1572_7792	26	4
SCE1572_1398	401	79
SCE1572_7799	1	0
SCE1572_552	91	182
SCE1572_11352	9	0
SCE1572_8589	18	2
SCE1572_4555	187	36
SCE1572_8548	179	31
SCE1572_8291	206	35
SCE1572_6757	74	41
SCE1572_685	0	0
SCE1572_5480	444	89
SCE1572_7732	33	35
SCE1572_9613	8	0
SCE1572_8301	249	1497
SCE1572_171	22	16
SCE1572_1880	211	59
SCE1572_3629	15	2
SCE1572_6786	122	89
SCE1572_6227	31	4
SCE1572_4580	169	32
SCE1572_11419	0	0
SCE1572_2279	376	234
SCE1572_2386	583	320
SCE1572_9171	41	11
SCE1572_1656	13	23
SCE1572_10893	80	57
SCE1572_10557	90	16
SCE1572_4317	40.43	2.58
SCE1572_1303	37	73
SCE1572_925	123	96
SCE1572_5437	0	0
SCE1572_8974	56	15
SCE1572_6549	61	55
SCE1572_1493	2	0
SCE1572_234	63	17
SCE1572_6573	143	18
SCE1572_8782	106	237
SCE1572_7749	90	13
SCE1572_759	1872	523

SCE1572_7301	0	0
SCE1572_4425	62	127
SCE1572_3091	71	20
SCE1572_3569	7	1
SCE1572_2509	49	38
SCE1572_8045	220	30
SCE1572_1040	11	0
SCE1572_2867	0	0
SCE1572_7311	507	84
SCE1572_1412	34	55
SCE1572_8723	10	3
SCE1572_10277	34	13
SCE1572_613	82	24
SCE1572_8076	88.45	11.76
SCE1572_10633	151	381
SCE1572_3763	28	14
SCE1572_7561	17	5
SCE1572_5892	32	51
SCE1572_470	212	245
SCE1572_2449	28	3
SCE1572_11092	75	121
SCE1572_11217	52	28
SCE1572_1277	0	0
SCE1572_3432	113	151
SCE1572_8180	111	13
SCE1572_2967	2	0
SCE1572_10009	83	25
SCE1572_9257	34	2
SCE1572_10056	11	36
SCE1572_9388	129	81
SCE1572_4596	258	630
SCE1572_9424	91	22
SCE1572_7857	123	40
SCE1572_10178	114	152
SCE1572_9815	30.36	7
SCE1572_2440	103	35
SCE1572_6435	49	5
SCE1572_3582	27	5
SCE1572_1524	66	29
SCE1572_9381	99	76
SCE1572_5893	45	10
SCE1572_3866	141	684
SCE1572_2238	378	1086
SCE1572_10002	257	357
SCE1572_6324	109	153
SCE1572_10873	215	22
SCE1572_10781	4	0
SCE1572_8134	216	27

SCE1572_6157	73	18
SCE1572_5316	83	16
SCE1572_3320	209	122
SCE1572_11047	57	21
SCE1572_8178	1	0
SCE1572_11448	5	1
SCE1572_7680	53	15
SCE1572_3006	1	0
SCE1572_3728	226	24
SCE1572_6511	21	6
SCE1572_2838	205.3	19.5
SCE1572_930	117	394
SCE1572_2214	0	0
SCE1572_630	112	459
SCE1572_607	42	183
SCE1572_4392	116	18
SCE1572_1670	348	909
SCE1572_1760	0	0
SCE1572_7772	96	37
SCE1572_11335	0	0
SCE1572_672	69	83
SCE1572_2364	4	0
SCE1572_8352	18	3
SCE1572_5257	50	11
SCE1572_4202	93	46
SCE1572_9274	174	265
SCE1572_4757	62	14
SCE1572_2529	42	15
SCE1572_2799	73	12
SCE1572_7911	52	3
SCE1572_5853	21.39	5
SCE1572_7885	80	37
SCE1572_7140	0	0
SCE1572_3909	99	16
SCE1572_3622	271	24
SCE1572_3549	304	44
SCE1572_10842	3	2
SCE1572_10422	255	204
SCE1572_1704	7	0
SCE1572_2200	143	89
SCE1572_9269	254	120
SCE1572_7607	379	26
SCE1572_6278	85	81
SCE1572_11270	294	193
SCE1572_3641	79	19
SCE1572_1448	15	4
SCE1572_8222	65	21
SCE1572_6668	102	39

SCE1572_10391	139	23
SCE1572_3723	43	316
SCE1572_5401	24	0
SCE1572_2209	31	6
SCE1572_5051	98	24
SCE1572_2294	104	41
SCE1572_2739	102	63
SCE1572_9260	130	59
SCE1572_6238	89	12
SCE1572_2175	20	13
SCE1572_10144	71	15
SCE1572_1793	1	0
SCE1572_8299	44	4
SCE1572_5044	43	18
SCE1572_11579	134	33
SCE1572_3989	17	11
SCE1572_3448	172	67
SCE1572_2473	169	404
SCE1572_3614	57	14
SCE1572_4179	125	19
SCE1572_217	46	19
SCE1572_3012	42	5
SCE1572_7604	21	6
SCE1572_5446	72	11
SCE1572_6629	46	103
SCE1572_11570	18	182
SCE1572_3537	91	15
SCE1572_2564	23	5
SCE1572_7053	46	8
SCE1572_3293	53	15
SCE1572_6213	42	11
SCE1572_5011	39	36
SCE1572_3067	68	23
SCE1572_10740	42	10
SCE1572_9132	157	55
SCE1572_3161	20	20
SCE1572_2882	57	26
SCE1572_726	147	24
SCE1572_4371	78	14
SCE1572_7824	70	11
SCE1572_8143	9	0
SCE1572_2954	128	24
SCE1572_5103	192	547
SCE1572_9826	34	5
SCE1572_3579	76	107
SCE1572_9507	54	19
SCE1572_1085	161	27
SCE1572_7861	2	0

SCE1572_6231	61	14
SCE1572_11414	106	56
SCE1572_6492	50	21
SCE1572_1886	5	1
SCE1572_4664	190	13
SCE1572_9500	76	22
SCE1572_838	43	23
SCE1572_4803	95	38
SCE1572_5874	27	13
SCE1572_11531	35	7
SCE1572_8455	40	89
SCE1572_6926	27	2
SCE1572_9780	127	35
SCE1572_3260	120	78
SCE1572_3777	32	10
SCE1572_6220	57	5
SCE1572_1142	135	18
SCE1572_8822	37	4
SCE1572_7566	11	16
SCE1572_3604	491	139
SCE1572_10374	56	25
SCE1572_9914	1	0
SCE1572_4802	57	66
SCE1572_8785	41	137
SCE1572_4750	99	72
SCE1572_4672	69	16
SCE1572_3768	75	43
SCE1572_8251	46	14
SCE1572_2809	31.92	8.35
SCE1572_9608	90	31
SCE1572_11139	32	7
SCE1572_2017	215	34
SCE1572_7047	15	4
SCE1572_7038	131	45
SCE1572_4149	130	24
SCE1572_7596	221	225
SCE1572_10029	118	78
SCE1572_5074	223	34
SCE1572_2225	109	16
SCE1572_11002	11	4
SCE1572_9094	121	508
SCE1572_3159	920	1513
SCE1572_2634	272	46
SCE1572_11028	16	1
SCE1572_6136	293	96
SCE1572_4496	55	30
SCE1572_3130	90	19
SCE1572_8453	19	25

SCE1572_2427	881	482
SCE1572_8943	84	10
SCE1572_6301	164	106
SCE1572_4897	70	29
SCE1572_8863	48	7
SCE1572_3705	0	0
SCE1572_10023	36.35	11.64
SCE1572_7336	172	52
SCE1572_1113	4	5
SCE1572_2889	63	11
SCE1572_5818	131	18
SCE1572_7916	440	485
SCE1572_5727	102	157
SCE1572_2352	65	166
SCE1572_764	106	18
SCE1572_2372	187	214
SCE1572_9220	1442.33	262.39
SCE1572_8603	74	43
SCE1572_10779	92	13
SCE1572_2831	50	14
SCE1572_7868	146	206
SCE1572_4175	227	22
SCE1572_1591	173	92
SCE1572_10318	257	72
SCE1572_1416	211	56
SCE1572_3031	2	0
SCE1572_9916	112	33
SCE1572_7640	34	43
SCE1572_229	78	165
SCE1572_5600	280.79	72.78
SCE1572_8512	248.76	46
SCE1572_1206	14	1
SCE1572_8369	43	19
SCE1572_4246	80	24
SCE1572_10798	24	4
SCE1572_9317	525	1087
SCE1572_7389	1	0
SCE1572_7409	59	13
SCE1572_6745	55	96
SCE1572_4875	95	22
SCE1572_3319	107	60
SCE1572_865	32	30
SCE1572_4211	567	613
SCE1572_3632	139.83	41.23
SCE1572_9026	39	352
SCE1572_803	1	0
SCE1572_222	61	15
SCE1572_1836	10	10

SCE1572_313	87	8
SCE1572_3591	0	0
SCE1572_7031	63	16
SCE1572_9686	160	103
SCE1572_9177	214	24
SCE1572_5427	29	24
SCE1572_4749	82	19
SCE1572_7017	0	0
SCE1572_3530	80	29
SCE1572_11195	42	13
SCE1572_994	55	12
SCE1572_5607	293	199
SCE1572_8574	3	2
SCE1572_9624	193	75
SCE1572_4453	78	46
SCE1572_3702	253	60
SCE1572_11274	129	188
SCE1572_9889	35	26
SCE1572_1850	359	112
SCE1572_6557	99	8
SCE1572_2001	46	11
SCE1572_11105	18	12
SCE1572_1432	6	2
SCE1572_2121	5	0
SCE1572_697	140	45
SCE1572_6143	4	0
SCE1572_2065	61	24
SCE1572_6776	993	125
SCE1572_5970	3	0
SCE1572_2751	142	44
SCE1572_8576	88.36	9.58
SCE1572_6740	87	131
SCE1572_8716	106	90
SCE1572_11251	112	16
SCE1572_4275	90	43
SCE1572_10552	0	0
SCE1572_269	26	5
SCE1572_293	2	0
SCE1572_5558	1887.95	1049.22
SCE1572_2491	74	9
SCE1572_6291	19	8
SCE1572_11385	723	2446
SCE1572_10764	67	0
SCE1572_9238	25	7
SCE1572_9193	19	1
SCE1572_10936	0	0
SCE1572_11469	54	55
SCE1572_8617	68	5

SCE1572_9540	107	16
SCE1572_1968	17	11
SCE1572_3853	2	0
SCE1572_6246	74	7
SCE1572_9721	154	657
SCE1572_8165	0	0
SCE1572_6336	252	60
SCE1572_3572	0	0
SCE1572_1815	57	5
SCE1572_6007	82	69
SCE1572_4255	129	124
SCE1572_2981	45	41
SCE1572_2170	175	99
SCE1572_5696	209	62
SCE1572_1751	37	22
SCE1572_6198	145	12
SCE1572_7944	68	16
SCE1572_5924	38	12
SCE1572_10451	26	105
SCE1572_5522	52	19
SCE1572_4883	22	15
SCE1572_9188	157	46
SCE1572_10162	89	12
SCE1572_108	360	433
SCE1572_9728	77	41
SCE1572_11393	256	113
SCE1572_3129	29	8
SCE1572_3652	51	18
SCE1572_3190	13	0
SCE1572_7274	44	6
SCE1572_11370	79	10
SCE1572_5704	168	75
SCE1572_1988	396	56
SCE1572_4608	154	112
SCE1572_1075	105	36
SCE1572_10405	172	89
SCE1572_4731	56	7
SCE1572_3712	62	1859
SCE1572_10747	47	10
SCE1572_6932	82	514
SCE1572_9057	1	0
SCE1572_10886	426	45
SCE1572_310	21	5
SCE1572_757	176	17
SCE1572_11405	67	12
SCE1572_7090	0	0
SCE1572_9731	263	97
SCE1572_9585	64	22

SCE1572_8284	22	1
SCE1572_10150	49	117
SCE1572_7339	34	285
SCE1572_10291	19	12
SCE1572_7901	117	10
SCE1572_1171	85	19
SCE1572_2132	114	16
SCE1572_11238	40	8
SCE1572_10496	48	29
SCE1572_2048	17	14
SCE1572_905	99	13
SCE1572_8631	134	38
SCE1572_7424	27	27
SCE1572_11132	34	6
SCE1572_526	107	112
SCE1572_11075	61	223
SCE1572_8669	8	0
SCE1572_7989	282	206
SCE1572_10938	49	12
SCE1572_9286	119	86
SCE1572_798	213	92
SCE1572_10348	136	20
SCE1572_9787	64	10
SCE1572_9547	163	27
SCE1572_7350	32	2
SCE1572_9007	24	13
SCE1572_7471	0	0
SCE1572_9758	254	220
SCE1572_8772	141	122
SCE1572_11453	17	8
SCE1572_3397	276	83
SCE1572_6121	44	12
SCE1572_10302	73	6
SCE1572_10472	161	173
SCE1572_1059	43	8
SCE1572_1186	24	10
SCE1572_8319	263	174
SCE1572_2287	41	56
SCE1572_9113	36	58
SCE1572_10458	150	44
SCE1572_5613	174	23
SCE1572_2900	4	2
SCE1572_7933	0	0
SCE1572_2289	11	35
SCE1572_7330	150	28
SCE1572_7266	0	0
SCE1572_1135	3	0
SCE1572_2972	274	75

SCE1572_10562	437	79
SCE1572_2848	113	17
SCE1572_8564	315	224
SCE1572_985	141	53
SCE1572_8058	16	0
SCE1572_5245	42	9
SCE1572_4999	870	1939
SCE1572_10538	23	2
SCE1572_4304	57	9
SCE1572_8977	15	2
SCE1572_421	14	21
SCE1572_4115	144	66
SCE1572_1952	59	12
SCE1572_10717	42	10
SCE1572_8677	33	24
SCE1572_2002	71	66
SCE1572_480	46	15
SCE1572_1991	113	14
SCE1572_53	331	54
SCE1572_2160	79	14
SCE1572_3055	15	0
SCE1572_5262	0	0
SCE1572_1052	733	1543
SCE1572_7370	113	26
SCE1572_6484	64	11
SCE1572_8753	53	6
SCE1572_11089	54	43
SCE1572_9475	113	99
SCE1572_5081	1827	933
SCE1572_6583	61	23
SCE1572_8969	31	49
SCE1572_7158	67	30
SCE1572_4491	59	133
SCE1572_11343	38	78
SCE1572_10356	67	189
SCE1572_5222	458	134
SCE1572_1514	67	7
SCE1572_8982	53	13
SCE1572_6069	107	201
SCE1572_7323	3	0
SCE1572_4100	109	46
SCE1572_5965	48	19
SCE1572_9041	755	2328
SCE1572_3329	348	962
SCE1572_4500	36	4
SCE1572_6568	35	49
SCE1572_11446	369	213
SCE1572_9590	157	522

SCE1572_1972	104	160
SCE1572_4980	70	104
SCE1572_5643	0	0
SCE1572_4284	143	25
SCE1572_9468	111	106
SCE1572_6148	55	7
SCE1572_9306	109	88
SCE1572_8337	261.32	107.59
SCE1572_5632	41	38
SCE1572_9599	75	17
SCE1572_11353	1	0
SCE1572_7109	19	5
SCE1572_9570	737	104
SCE1572_3955	169	32
SCE1572_3798	74	43
SCE1572_2689	117	140
SCE1572_5344	51	10
SCE1572_3999	739	632
SCE1572_590	99	20
SCE1572_4501	97	179
SCE1572_3817	86	413
SCE1572_878	107	258
SCE1572_731	13	4
SCE1572_3940	608	499
SCE1572_2815	55	35
SCE1572_4126	65	10
SCE1572_8960	94	23
SCE1572_11282	111	108
SCE1572_4414	22	5
SCE1572_2231	16	46
SCE1572_165	49	7
SCE1572_3744	68	62
SCE1572_4195	74	64
SCE1572_307	0	0
SCE1572_7379	51	9
SCE1572_3327	225	842
SCE1572_10635	182	196
SCE1572_5796	51	21
SCE1572_3902	33	66
SCE1572_7754	76	82
SCE1572_5504	82	29
SCE1572_546	0	0
SCE1572_10082	84	262
SCE1572_7516	30	20
SCE1572_7231	99	74
SCE1572_3082	57	19
SCE1572_8537	67	28
SCE1572_8141	47	6

SCE1572_3560	207	22
SCE1572_3847	62	10
SCE1572_2740	17	5
SCE1572_7965	12	8
SCE1572_8234	0	0
SCE1572_6760	530	236
SCE1572_3166	152	40
SCE1572_6766	0	0
SCE1572_9459	0	0
SCE1572_34	126	194
SCE1572_7554	25	2
SCE1572_303	204	35
SCE1572_6874	479	2988
SCE1572_10915	0	0
SCE1572_6341	121	20
SCE1572_2105	278	597
SCE1572_2664	385	1487
SCE1572_9443	32	26
SCE1572_2920	124	13
SCE1572_2183	96	22
SCE1572_2854	0	0
SCE1572_6406	22	66
SCE1572_5666	26	7
SCE1572_10594	197	37
SCE1572_2917	26	5
SCE1572_10354	13	4
SCE1572_10143	65	110
SCE1572_1066	58	9
SCE1572_9553	69	12
SCE1572_4886	2	0
SCE1572_6352	74	30
SCE1572_9157	453	149
SCE1572_3310	43	6
SCE1572_9478	220	687
SCE1572_2776	0	0
SCE1572_2331	65	18
SCE1572_2714	82	45
SCE1572_2941	39	11
SCE1572_9484	326	587
SCE1572_3096	3	1
SCE1572_5987	60	19.84
SCE1572_10614	33	3
SCE1572_4465	262	160
SCE1572_193	147	89
SCE1572_2093	80	36
SCE1572_6253	53	128
SCE1572_9454	186	46
SCE1572_1820	26	15

SCE1572_1810	49	17
SCE1572_10923	509	17571
SCE1572_2498	0	0
SCE1572_8754	200	25
SCE1572_1803	13	4
SCE1572_7630	42	102
SCE1572_10548	109	16
SCE1572_489	71.1	15.07
SCE1572_10189	101	103
SCE1572_4865	44	44
SCE1572_7981	25	15
SCE1572_2674	75	170
SCE1572_3115	50	10
SCE1572_5906	308	749
SCE1572_11071	131	59
SCE1572_10102	53	82
SCE1572_5099	65	6
SCE1572_3962	123	38
SCE1572_7714	66	98
SCE1572_2871	0	0
SCE1572_8872	114	22
SCE1572_6663	27	7
SCE1572_11473	75	21
SCE1572_4831	264	202
SCE1572_8580	737	134
SCE1572_8506	26	4
SCE1572_4740	145	92
SCE1572_9775	0	0
SCE1572_4067	38	19
SCE1572_10732	202	43
SCE1572_7508	183.42	28.37
SCE1572_9665	24	40
SCE1572_10647	30	34
SCE1572_5069	153	55
SCE1572_2406	79	243
SCE1572_10074	63	0
SCE1572_2825	36	12
SCE1572_9558	0	0
SCE1572_6182	131	17
SCE1572_156	118	151
SCE1572_1825	23	6
SCE1572_7739	3	0
SCE1572_4260	324	128
SCE1572_5621	229.99	40
SCE1572_1774	50	6
SCE1572_2860	44	6
SCE1572_7088	89	14
SCE1572_6424	0	0

SCE1572_6175	104	72
SCE1572_6616	103	17
SCE1572_8736	28	9
SCE1572_2720	317	30
SCE1572_2055	34	19
SCE1572_6778	426	76
SCE1572_9517	365	252
SCE1572_1616	26	44
SCE1572_7100	20	22
SCE1572_449	29	10
SCE1572_4443	24	38
SCE1572_9766	75	78
SCE1572_6644	1	0
SCE1572_331	173	204
SCE1572_10569	326	234
SCE1572_4335	118	27
SCE1572_4796	129	28
SCE1572_10791	148	85
SCE1572_2679	41	450
SCE1572_4217	111	65
SCE1572_9525	12	14
SCE1572_9072	43	27
SCE1572_41	129	41
SCE1572_1128	86	31
SCE1572_1523	181	26
SCE1572_5212	76	64
SCE1572_9882	92	31
SCE1572_3480	172	81
SCE1572_9973	284	452
SCE1572_11074	6	0
SCE1572_6681	29	66
SCE1572_1122	593	52
SCE1572_5346	3	2
SCE1572_11532	0	0
SCE1572_4573	48	15
SCE1572_9154	137	201
SCE1572_2707	37	5
SCE1572_10551	63	10
SCE1572_8774	457	436
SCE1572_1851	58	15
SCE1572_8666	31	1
SCE1572_2665	79	248
SCE1572_5604	419	207
SCE1572_8623	126	166
SCE1572_8259	287	351
SCE1572_861	89	550
SCE1572_2416	201	100
SCE1572_8975	35	9

SCE1572_7268	328	279.52
SCE1572_1614	164	183
SCE1572_7725	130	51
SCE1572_2596	141.25	67.32
SCE1572_9387	364	316
SCE1572_7112	0	0
SCE1572_6111	29	33
SCE1572_167	65	8
SCE1572_2678	22	121
SCE1572_6643	623	265
SCE1572_6637	119.82	57
SCE1572_8288	42	51
SCE1572_2929	0	0
SCE1572_3951	111	63
SCE1572_9480	48	24
SCE1572_7250	403	67
SCE1572_4312	144	21
SCE1572_9416	117	1010
SCE1572_3621	16	1
SCE1572_2024	38	6
SCE1572_5793	274	197
SCE1572_6115	119	14
SCE1572_9350	708	1305
SCE1572_5673	43	22
SCE1572_2856	51	8
SCE1572_3499	33	19
SCE1572_2469	38	11
SCE1572_804	0	0
SCE1572_10588	18	2
SCE1572_4913	19	4
SCE1572_1784	14	2
SCE1572_9077	121	24
SCE1572_8235	18	11
SCE1572_10176	87	22
SCE1572_4817	166	623
SCE1572_10596	88	14
SCE1572_6146	188	47
SCE1572_1779	81	46
SCE1572_7045	156	325
SCE1572_3412	37	51
SCE1572_3708	0	0
SCE1572_11508	27	5
SCE1572_11472	18	0
SCE1572_5983	11	6
SCE1572_7511	97	20
SCE1572_8462	80	331
SCE1572_5626	19	3
SCE1572_1916	3	0

SCE1572_10664	74	224
SCE1572_3580	276	50
SCE1572_2405	84	283
SCE1572_10352	65	19
SCE1572_6056	705	915
SCE1572_1248	146	133
SCE1572_9995	136	29
SCE1572_564	62	576
SCE1572_797	377	255
SCE1572_7026	145	26
SCE1572_8144	43	4
SCE1572_3413	37	18
SCE1572_8743	3	0
SCE1572_2098	108	106
SCE1572_4455	854	366
SCE1572_9751	221	58
SCE1572_11358	114	29
SCE1572_9520	239.5	287.5
SCE1572_9879	54	43
SCE1572_2996	162	34
SCE1572_5082	136	109
SCE1572_9776	96	56
SCE1572_3796	41	3
SCE1572_6769	934	5830
SCE1572_2572	261	37
SCE1572_8883	69	13
SCE1572_4798	41	193
SCE1572_10929	47	14
SCE1572_179	67	21
SCE1572_10389	102	57
SCE1572_4016	78	27
SCE1572_6138	48	30
SCE1572_2731	245	97
SCE1572_2712	421	567
SCE1572_10072	558	642
SCE1572_10194	125	27
SCE1572_4329	46	21
SCE1572_6407	46	62
SCE1572_7776	111	237
SCE1572_9769	11	8
SCE1572_6501	44	10
SCE1572_10399	247	125
SCE1572_10992	5	4
SCE1572_9398	45	46
SCE1572_10415	40	34
SCE1572_5310	26	16
SCE1572_1335	29	5
SCE1572_4267	0	0

SCE1572_2142	97	11
SCE1572_6177	92	51
SCE1572_1014	67	20
SCE1572_9544	92	11
SCE1572_9324	3388	2190
SCE1572_4324	37	11
SCE1572_26	153	94
SCE1572_2978	13	1
SCE1572_9034	47	13
SCE1572_10100	94	22
SCE1572_11460	914	942
SCE1572_1322	85	10
SCE1572_10149	37	4
SCE1572_5118	50	62
SCE1572_10916	194	65
SCE1572_988	81	86
SCE1572_8428	62	20
SCE1572_3011	173	17
SCE1572_484	107	29
SCE1572_7809	0	0
SCE1572_7723	76	41
SCE1572_10305	78	22
SCE1572_6737	189	493
SCE1572_6717	133	162
SCE1572_544	42	17
SCE1572_6710	892	312
SCE1572_11584	104	23
SCE1572_9489	149	42
SCE1572_11244	41	39
SCE1572_10290	36	8
SCE1572_5615	101	10
SCE1572_10069	61	4
SCE1572_10708	27	7
SCE1572_6	98	55
SCE1572_6441	5	0
SCE1572_7982	108	28
SCE1572_9735	223	162
SCE1572_3511	101	70
SCE1572_9452	258	59
SCE1572_9305	47	35
SCE1572_2708	314	33
SCE1572_2915	1151	146
SCE1572_3956	180	26
SCE1572_766	17	2
SCE1572_1106	94	8
SCE1572_3949	233	506
SCE1572_1396	31	154
SCE1572_3068	83	80

SCE1572_4440	161	39
SCE1572_4413	8	0
SCE1572_9837	203	189
SCE1572_102	46	7
SCE1572_10925	0	0
SCE1572_2380	32	12
SCE1572_4904	50	289
SCE1572_4333	9	2
SCE1572_11140	59	20
SCE1572_6648	4	0
SCE1572_8330	131	341
SCE1572_8223	7	4
SCE1572_5191	255	379
SCE1572_6614	196	44
SCE1572_11443	115	32
SCE1572_1947	132	35
SCE1572_3059	14	1
SCE1572_3271	251	1131
SCE1572_1838	205	419
SCE1572_8965	95	69
SCE1572_691	46	6
SCE1572_10574	196	100
SCE1572_11233	145	159
SCE1572_3598	317	41
SCE1572_8510	43	8
SCE1572_4747	209	29
SCE1572_9693	117	937
SCE1572_4081	44	25
SCE1572_9482	15	12
SCE1572_1078	86	22
SCE1572_853	129	252
SCE1572_7931	155	54
SCE1572_7300	137	26
SCE1572_6196	29	5
SCE1572_3634	59	14
SCE1572_1994	242	37
SCE1572_7958	246	300
SCE1572_6350	80	41
SCE1572_9487	42	27
SCE1572_10210	165	13
SCE1572_10728	34	34
SCE1572_3321	365	275
SCE1572_3552	147	24
SCE1572_10375	95	10
SCE1572_6785	0	0
SCE1572_6347	46	21
SCE1572_6126	51	92
SCE1572_9117	201	48

SCE1572_1992	0	0
SCE1572_5265	0	0
SCE1572_8832	5	2
SCE1572_10721	1348	248
SCE1572_1452	0	0
SCE1572_9722	1813	7821
SCE1572_7961	14	0
SCE1572_10299	77	101
SCE1572_9069	80	183
SCE1572_7864	93	49
SCE1572_1776	76	16
SCE1572_3052	85	10
SCE1572_10792	150	98
SCE1572_8700	80	26
SCE1572_1025	34	354
SCE1572_7309	204	27
SCE1572_2283	84	18
SCE1572_2775	44	55
SCE1572_5505	1	2
SCE1572_7166	83	10
SCE1572_3093	142	48
SCE1572_680	14	12
SCE1572_5535	274	36
SCE1572_3846	47	5
SCE1572_4351	65	9
SCE1572_11084	16	21
SCE1572_1219	40	13
SCE1572_209	72	17
SCE1572_2003	223	42
SCE1572_219	24	36
SCE1572_2523	1	0
SCE1572_5317	3	0
SCE1572_8583	1688	287
SCE1572_8252	147	179
SCE1572_5936	79	23
SCE1572_128	4	0
SCE1572_7787	27	0
SCE1572_3486	346	72
SCE1572_1137	148.64	29
SCE1572_8272	21	13
SCE1572_9391	58	15
SCE1572_6400	130	197
SCE1572_4145	133	169.04
SCE1572_3398	80	8
SCE1572_9008	52	8
SCE1572_9888	179	43
SCE1572_7155	76	16
SCE1572_8527	12	0

SCE1572_7583	398	1053
SCE1572_6741	676	135
SCE1572_9788	165	60
SCE1572_7745	932	1866
SCE1572_7322	37	3
SCE1572_5966	37	9
SCE1572_4513	2	1
SCE1572_6135	103	78
SCE1572_8735	0	0
SCE1572_223	32	3
SCE1572_6921	467.98	164.53
SCE1572_11078	18	4
SCE1572_3316	155	66
SCE1572_1866	25	10
SCE1572_4730	162.85	40
SCE1572_8279	31	4
SCE1572_7474	117	105
SCE1572_9979	105	17
SCE1572_3714	94	75
SCE1572_908	13	3
SCE1572_1630	113	19
SCE1572_6032	21	15
SCE1572_11080	56	270
SCE1572_8535	131	34
SCE1572_8293	40	10
SCE1572_912	456	581
SCE1572_1313	1529	2663
SCE1572_998	81	61
SCE1572_8054	8	2
SCE1572_2492	179	43
SCE1572_10933	15	0
SCE1572_3046	26	2
SCE1572_11451	17	4
SCE1572_3223	9	1
SCE1572_4307	93	34
SCE1572_7788	0	0
SCE1572_4699	52	10
SCE1572_10343	44	104
SCE1572_6041	214	139
SCE1572_5665	36	8
SCE1572_897	80	16
SCE1572_9027	190	132
SCE1572_4286	0	0
SCE1572_10669	136	243
SCE1572_2546	120	27
SCE1572_2671	459	352
SCE1572_7345	11	1
SCE1572_10565	165	29

SCE1572_3559	152	76
SCE1572_8242	65	104
SCE1572_1504	158	92
SCE1572_4861	64	13
SCE1572_5771	1	0
SCE1572_314	52	9
SCE1572_2324	16	13
SCE1572_6762	0	0
SCE1572_890	48	39
SCE1572_10301	91	16
SCE1572_11388	383	283
SCE1572_6519	99	11
SCE1572_2069	199	36
SCE1572_2746	27	1
SCE1572_11196	130	53
SCE1572_1821	19	10
SCE1572_2114	517.97	195
SCE1572_9211	21	14
SCE1572_11349	0	0
SCE1572_2554	168	31
SCE1572_3858	49	59
SCE1572_9070	94	31
SCE1572_10187	17	3
SCE1572_5923	158	81
SCE1572_5708	154	55
SCE1572_9711	310	521
SCE1572_5421	40	89
SCE1572_597	154	199
SCE1572_10639	129	322
SCE1572_2583	10	1
SCE1572_6089	15	38
SCE1572_9597	232	4775
SCE1572_2134	0	0
SCE1572_7261	0	0
SCE1572_2060	14	7
SCE1572_5979	19	23
SCE1572_1770	55	11
SCE1572_9335	88	165
SCE1572_4256	120	302
SCE1572_4992	21	29
SCE1572_10969	252	212
SCE1572_9281	267	417
SCE1572_8675	62	20
SCE1572_2687	126	56
SCE1572_689	44	28
SCE1572_4192	118	132
SCE1572_9689	207	151
SCE1572_4611	37	10

SCE1572_7971	14	8
SCE1572_7479	179	62
SCE1572_1071	59	11
SCE1572_6228	217	34
SCE1572_9172	46	7
SCE1572_8447	11	0
SCE1572_7751	179	36
SCE1572_3231	71	107
SCE1572_319	141	21
SCE1572_4349	530	105
SCE1572_7623	73	42
SCE1572_11025	260	180
SCE1572_4122	37	15
SCE1572_9586	129	97
SCE1572_6002	40	93
SCE1572_1410	69	14
SCE1572_372	91	186
SCE1572_10142	123	105
SCE1572_10883	19	87
SCE1572_9053	1218.35	202
SCE1572_1112	10	5
SCE1572_6833	63	28
SCE1572_8752	60	9
SCE1572_4129	193	242
SCE1572_8594	79	55
SCE1572_2888	91	17
SCE1572_8728	110	21
SCE1572_8069	19.55	1
SCE1572_10323	56	47
SCE1572_9265	278	159
SCE1572_8305	56	15
SCE1572_10479	73	31
SCE1572_6326	12	3
SCE1572_3686	24	0
SCE1572_5835	1327	816
SCE1572_4669	85	7
SCE1572_4828	47	8
SCE1572_262	115	19
SCE1572_8142	14	1
SCE1572_5288	202	70
SCE1572_9477	0	0
SCE1572_2442	129	294
SCE1572_8581	1294.22	135.84
SCE1572_9227	49.97	24
SCE1572_734	87	17
SCE1572_8091	5	0
SCE1572_11486	94	70
SCE1572_4043	194	482

SCE1572_8393	172	62
SCE1572_2342	33	50
SCE1572_3732	55	22
SCE1572_3772	158	87
SCE1572_6517	16	33
SCE1572_3354	99	43
SCE1572_4528	0	0
SCE1572_11181	52	33
SCE1572_3077	214	40
SCE1572_8553	49	265
SCE1572_11102	71	56
SCE1572_7609	27	15
SCE1572_4553	26	1
SCE1572_5388	270	467
SCE1572_8283	38	3
SCE1572_661	0	0
SCE1572_8098	13	225
SCE1572_3301	47	18
SCE1572_1983	0	0
SCE1572_6855	17	16
SCE1572_7914	69	18
SCE1572_1145	19	2
SCE1572_3147	19	1
SCE1572_7329	22	1
SCE1572_6250	0	0
SCE1572_7315	17	4
SCE1572_7938	68	88
SCE1572_11420	202	83
SCE1572_9869	274	511
SCE1572_1089	121	34
SCE1572_7606	181	63
SCE1572_6451	276.76	57.74
SCE1572_4199	36	42
SCE1572_4800	71	695
SCE1572_9860	183	54
SCE1572_6247	42	11
SCE1572_3133	380	99
SCE1572_8456	83	374
SCE1572_11417	77	26
SCE1572_1204	0	0
SCE1572_1030	35	74
SCE1572_1146	171	48
SCE1572_9504	37	4
SCE1572_11484	35	0
SCE1572_8195	126	49
SCE1572_1921	0	0
SCE1572_1699	174	184
SCE1572_3245	94	29

SCE1572_3852	110	80
SCE1572_10844	8	2
SCE1572_4227	100	47
SCE1572_10073	141	247
SCE1572_4666	49	3
SCE1572_8839	12	0
SCE1572_8366	74	7
SCE1572_7833	188	116
SCE1572_7436	13	4
SCE1572_8213	176	97
SCE1572_9311	223	422
SCE1572_7567	42	8
SCE1572_1082	41	3
SCE1572_5537	126	36
SCE1572_6535	67	20
SCE1572_934	91	158
SCE1572_11564	12	4
SCE1572_11263	51	50
SCE1572_11020	27	8
SCE1572_2533	18	131
SCE1572_5489	0	0
SCE1572_4809	177	68
SCE1572_33	1365	3054
SCE1572_6300	101	155
SCE1572_9607	30	4
SCE1572_11524	19	4
SCE1572_10753	120	11
SCE1572_115	45	7
SCE1572_6102	33	4
SCE1572_6410	0	0
SCE1572_8792	66	33
SCE1572_610	70	47
SCE1572_1973	0	0
SCE1572_2828	0	0
SCE1572_136	83	11
SCE1572_3375	61	84
SCE1572_5690	45	29
SCE1572_2172	29	1
SCE1572_6082	45	3
SCE1572_5041	164	237
SCE1572_10895	99	79
SCE1572_5325	58	29
SCE1572_9645	42	237
SCE1572_7780	11	2
SCE1572_1037	1	0
SCE1572_1903	419	56
SCE1572_5816	59	12
SCE1572_7143	55	7

SCE1572_11435	162	52
SCE1572_2732	203	167
SCE1572_11223	15	4
SCE1572_4830	105	66
SCE1572_452	8	23
SCE1572_1556	66	44
SCE1572_159	344	257.48
SCE1572_4894	81	13
SCE1572_4109	52	9
SCE1572_5852	82	36
SCE1572_6473	358	296
SCE1572_3648	90	180
SCE1572_1719	180	98
SCE1572_2205	15	62
SCE1572_606	323	241
SCE1572_2179	31	39
SCE1572_4041	579	3115
SCE1572_2563	76	16
SCE1572_12	179	265
SCE1572_1252	148	27
SCE1572_1460	10	3
SCE1572_10773	26	13
SCE1572_2216	77	172
SCE1572_2645	4	0
SCE1572_147	73	28
SCE1572_3844	285	659
SCE1572_4030	405	390
SCE1572_1461	185	43
SCE1572_2588	235	38
SCE1572_7646	300	172
SCE1572_725	57	23
SCE1572_4884	22	21
SCE1572_7828	80	86
SCE1572_6491	4	0
SCE1572_4037	19	12
SCE1572_1414	59	9
SCE1572_4502	118	20
SCE1572_1298	75	13
SCE1572_1705	348	171
SCE1572_664	425	586
SCE1572_3423	3	0
SCE1572_3008	49	11
SCE1572_3034	147	154
SCE1572_3823	29	22
SCE1572_10890	70	22
SCE1572_1498	102.67	9.34
SCE1572_7798	38	21
SCE1572_2603	13.25	0.75

SCE1572_2181	59	16
SCE1572_2079	25	7
SCE1572_4839	176	156
SCE1572_10006	12	6
SCE1572_9960	81	17
SCE1572_6292	76	19
SCE1572_1231	159	273
SCE1572_617	220	404
SCE1572_6587	533	146
SCE1572_3757	338	45
SCE1572_1551	62	11
SCE1572_5046	0	0
SCE1572_6159	65	1
SCE1572_1801	32	114
SCE1572_4739	136	13
SCE1572_6496	91	15
SCE1572_5907	792	314
SCE1572_7900	67	20
SCE1572_3864	56	9
SCE1572_6811	196	57
SCE1572_10812	63	11
SCE1572_10554	98	15
SCE1572_1799	41	194
SCE1572_2241	145	41
SCE1572_1585	85	185
SCE1572_10534	61	116
SCE1572_8384	21	4
SCE1572_1270	145	216
SCE1572_2499	196	40
SCE1572_8329	124	34
SCE1572_1592	358	173
SCE1572_3563	453	109
SCE1572_9465	3	0
SCE1572_3991	867	3289
SCE1572_2879	267	211
SCE1572_3182	208	414
SCE1572_3568	301.97	32.84
SCE1572_553	45	31
SCE1572_1365	168	1757
SCE1572_4509	41	46
SCE1572_10424	70	11
SCE1572_5073	121	515
SCE1572_4429	93	74
SCE1572_9101	67	70
SCE1572_9859	140	55
SCE1572_6936	23	5
SCE1572_10057	279	97
SCE1572_9773	79	35

SCE1572_5078	110	305
SCE1572_10819	124	99
SCE1572_7599	1560	1606
SCE1572_2049	52	55
SCE1572_8939	33	171
SCE1572_3985	103	129
SCE1572_334	48	45
SCE1572_1884	74	76
SCE1572_10746	8	1
SCE1572_6106	37	5
SCE1572_5105	476	2405
SCE1572_11334	164	150
SCE1572_11412	122	179
SCE1572_7394	45	2
SCE1572_13	333	84
SCE1572_9423	25	14
SCE1572_10803	16	4
SCE1572_10429	47	7
SCE1572_1812	98	19
SCE1572_11576	28	23
SCE1572_2607	83	22
SCE1572_1525	312	641
SCE1572_8651	14	1
SCE1572_5999	66	251
SCE1572_11259	179	723
SCE1572_4917	23	3
SCE1572_10130	62	21
SCE1572_4485	40	72
SCE1572_1530	0	0
SCE1572_2805	113.36	48
SCE1572_428	67	71
SCE1572_8471	864	1023
SCE1572_870	26	28
SCE1572_4449	1	0
SCE1572_11041	334	168
SCE1572_3616	43	9
SCE1572_3464	480	95
SCE1572_5397	85	18
SCE1572_6024	325	130
SCE1572_3335	145	132
SCE1572_5644	26	1
SCE1572_11121	59	12
SCE1572_2195	104	44
SCE1572_4103	90	15
SCE1572_2973	1	0
SCE1572_785	103	53
SCE1572_5699	25.81	52.11
SCE1572_6767	11	2

SCE1572_4685	279	156
SCE1572_7768	490	197
SCE1572_1465	57	11
SCE1572_9093	21	1
SCE1572_2489	8	0
SCE1572_4170	0	0
SCE1572_5381	80	12
SCE1572_6448	411	80
SCE1572_9476	26	3
SCE1572_10901	40	26
SCE1572_4534	78	34
SCE1572_2834	761	3532
SCE1572_8658	80	194
SCE1572_8707	106	35
SCE1572_7018	31	4
SCE1572_5990	332	99
SCE1572_9156	217	231
SCE1572_2959	65	12
SCE1572_7094	0	0
SCE1572_11529	132	17
SCE1572_2226	89	13
SCE1572_5235	133	561
SCE1572_7076	134	63
SCE1572_2296	177	200
SCE1572_9566	0	0
SCE1572_5890	95	29
SCE1572_4942	22	52
SCE1572_4978	118	701
SCE1572_1441	127	20
SCE1572_10905	192	116
SCE1572_11109	93.15	44
SCE1572_10066	255	497
SCE1572_11275	155	324
SCE1572_7592	163	56
SCE1572_7821	55	129
SCE1572_1492	155.1	26.33
SCE1572_8871	202	65
SCE1572_8300	55	3
SCE1572_10641	70	34
SCE1572_1763	172.99	5.15
SCE1572_1797	43	14
SCE1572_2559	74	19
SCE1572_4138	105	15
SCE1572_4270	92	14
SCE1572_9125	26	9
SCE1572_11171	23	1
SCE1572_7791	29	2
SCE1572_927	49	8

SCE1572_10962	155	24
SCE1572_9003	186	321
SCE1572_9136	27	3
SCE1572_6706	612	887
SCE1572_292	8	27
SCE1572_10485	139	266
SCE1572_5093	56	276
SCE1572_1272	6	4
SCE1572_5100	32	23
SCE1572_9178	146	170
SCE1572_11063	90	28
SCE1572_305	60	13
SCE1572_2112	1	0
SCE1572_10394	0	0
SCE1572_10835	178	23
SCE1572_10624	100	127
SCE1572_9740	144	311
SCE1572_7372	87	48
SCE1572_1677	4	1
SCE1572_9363	40	42
SCE1572_8905	12	2
SCE1572_11129	73	35
SCE1572_5860	0	0
SCE1572_5283	60	14
SCE1572_4071	31	58
SCE1572_3936	0	0
SCE1572_7455	76	206
SCE1572_6725	52	36
SCE1572_1844	135	34
SCE1572_2505	26	7
SCE1572_6563	55	24
SCE1572_1296	0	0
SCE1572_652	773	1326
SCE1572_6730	1921	12296
SCE1572_7453	302	915
SCE1572_1672	30	13
SCE1572_5689	61	12
SCE1572_8439	82	28
SCE1572_1736	158	32
SCE1572_6290	0	0
SCE1572_79	88	21
SCE1572_3291	55	27
SCE1572_4084	58	7
SCE1572_5366	101	670
SCE1572_5844	39	13
SCE1572_6293	137	64
SCE1572_4200	74	89
SCE1572_4776	277	605

SCE1572_2158	0	0
SCE1572_6797	106	122
SCE1572_1960	201	17
SCE1572_10613	26	10
SCE1572_8382	25	2
SCE1572_5135	163	529
SCE1572_2011	57	1
SCE1572_10528	5	0
SCE1572_1934	71	54
SCE1572_11207	0	0
SCE1572_1743	67	16
SCE1572_5718	60	31
SCE1572_9134	74	15
SCE1572_1625	89	18
SCE1572_1381	70	23
SCE1572_2085	61	23
SCE1572_4014	0	0
SCE1572_4560	6	1
SCE1572_4649	134	22
SCE1572_3779	163	59
SCE1572_7107	409	107
SCE1572_11562	0	0
SCE1572_3755	694.59	78.72
SCE1572_5138	0	0
SCE1572_11283	59	34
SCE1572_10088	0	0
SCE1572_2807	4.64	0
SCE1572_10266	118	135
SCE1572_9441	101	354
SCE1572_8799	3	0
SCE1572_4843	97	48
SCE1572_3612	166	29
SCE1572_1710	31	20
SCE1572_3363	49	38
SCE1572_7352	66	10
SCE1572_1471	0	0
SCE1572_309	77	10
SCE1572_9103	159	83
SCE1572_5305	0	0
SCE1572_1350	65	30
SCE1572_8020	158	36
SCE1572_5984	121	47
SCE1572_3079	0	0
SCE1572_9249	104	14
SCE1572_6950	5	0
SCE1572_7522	80	12
SCE1572_701	105	439
SCE1572_6820	39	4

SCE1572_8389	78	12
SCE1572_11293	291	413
SCE1572_5739	159	55
SCE1572_2790	31	6
SCE1572_5209	7	2
SCE1572_7219	283	2056
SCE1572_8163	120	29
SCE1572_1300	138	15
SCE1572_491	965.44	379.25
SCE1572_5455	113	15
SCE1572_7184	0	0
SCE1572_5233	12	34
SCE1572_8913	55	9
SCE1572_4278	317	79
SCE1572_8414	117	32
SCE1572_3238	293	76
SCE1572_8464	80	83
SCE1572_8496	329	51
SCE1572_10606	94	31
SCE1572_5404	10	4
SCE1572_744	57	25
SCE1572_10856	268	425
SCE1572_964	150	343
SCE1572_10846	50	17
SCE1572_4640	316	78
SCE1572_947	158.67	8.79
SCE1572_6924	1	0
SCE1572_4090	46	10
SCE1572_8405	28	51
SCE1572_7549	228	28
SCE1572_6454	160	72
SCE1572_9207	90	31
SCE1572_8087	185	16
SCE1572_3098	0	0
SCE1572_9972	168	329
SCE1572_210	67	10
SCE1572_5524	62	17
SCE1572_5357	215	50
SCE1572_5795	75	24
SCE1572_10257	76	27
SCE1572_3514	132	360
SCE1572_5887	36	11
SCE1572_8946	422	722
SCE1572_11502	27	1
SCE1572_7205	203	107
SCE1572_9694	2626	6613
SCE1572_1141	31	7
SCE1572_2623	2	0

SCE1572_7419	17	2
SCE1572_6656	129	149
SCE1572_2270	769	1793
SCE1572_3151	46	219
SCE1572_7614	62	20
SCE1572_4679	152	19
SCE1572_5206	91	94
SCE1572_6611	22	3
SCE1572_6287	60	41
SCE1572_11290	1679	1089
SCE1572_196	8	0
SCE1572_6478	48	8
SCE1572_3070	35	13
SCE1572_9515	118	143
SCE1572_10283	55	103
SCE1572_11540	32	6
SCE1572_10033	227	693
SCE1572_5802	150	120
SCE1572_9492	194	44
SCE1572_7064	167	30
SCE1572_8345	0	0
SCE1572_582	36	8
SCE1572_1346	33	25
SCE1572_4569	92	32
SCE1572_655	1027	597
SCE1572_3021	12	1
SCE1572_5587	48	9
SCE1572_8858	163	57
SCE1572_4983	746	643
SCE1572_11173	41	20
SCE1572_6366	49	284
SCE1572_11004	117	82
SCE1572_6897	0	0
SCE1572_2818	91	26
SCE1572_9818	21	9
SCE1572_5567	93	127
SCE1572_11298	116	155
SCE1572_3929	160	113
SCE1572_831	0	0
SCE1572_772	60	16
SCE1572_5837	241	209
SCE1572_3974	15	25
SCE1572_8074	22	3
SCE1572_7618	230	183
SCE1572_6387	67	78
SCE1572_4377	125	34
SCE1572_7664	79	11
SCE1572_6885	58	51

SCE1572_5511	97	34
SCE1572_1891	35	2
SCE1572_4955	61	9
SCE1572_3104	47	9
SCE1572_10163	176	32
SCE1572_9407	124	71
SCE1572_2576	41	5
SCE1572_1057	103	18
SCE1572_3041	64	3
SCE1572_3894	33	6
SCE1572_9509	15	4
SCE1572_1729	21	38
SCE1572_10171	33	5
SCE1572_11043	270	93
SCE1572_1117	23	16
SCE1572_2373	35	5
SCE1572_188	51	264
SCE1572_6957	0	0
SCE1572_9168	52	13
SCE1572_7074	49	28
SCE1572_2983	25	28
SCE1572_6866	175	40
SCE1572_7234	125	51
SCE1572_6806	81	25
SCE1572_8920	233	146
SCE1572_8919	20	220
SCE1572_117	68	217
SCE1572_7702	165	23
SCE1572_1503	121	135
SCE1572_5774	57	2
SCE1572_1512	21	12
SCE1572_5065	87	120
SCE1572_3749	30	95
SCE1572_7921	1	0
SCE1572_11396	232	295
SCE1572_2257	209	70
SCE1572_4926	93	12
SCE1572_9141	29	4
SCE1572_6890	28	2
SCE1572_7139	0	0
SCE1572_10736	70	54
SCE1572_4587	385	376
SCE1572_473	51	165
SCE1572_9660	1297	1279
SCE1572_5186	186	2570
SCE1572_4194	1045	816
SCE1572_8894	206	64
SCE1572_8906	75	30

SCE1572_3388	29	1
SCE1572_6857	60	7
SCE1572_11148	35	25
SCE1572_10658	1943	691
SCE1572_9615	0	0
SCE1572_10048	133	242
SCE1572_4370	70	16
SCE1572_1483	257	41
SCE1572_8118	46.55	61.05
SCE1572_11491	68	20
SCE1572_5830	62	14
SCE1572_996	140	29
SCE1572_5897	24	5
SCE1572_702	42	9
SCE1572_2960	141	46
SCE1572_7942	158	38
SCE1572_4707	38	33
SCE1572_1967	16	8
SCE1572_4676	78	21
SCE1572_815	74	15
SCE1572_3168	231	51
SCE1572_4990	137	386
SCE1572_6378	58	7
SCE1572_11371	461	208
SCE1572_1594	91	60
SCE1572_9191	18	6
SCE1572_9059	18	6
SCE1572_10443	50	6
SCE1572_3127	16	17
SCE1572_7996	111.64	21
SCE1572_6471	38	10
SCE1572_3243	168	36
SCE1572_528	53	78
SCE1572_2255	28	3
SCE1572_5711	707	680
SCE1572_5870	40	29
SCE1572_3001	28	5
SCE1572_181	85	20
SCE1572_4973	943	3866
SCE1572_9432	102	32
SCE1572_2263	244	635
SCE1572_5494	51	5
SCE1572_738	63	23
SCE1572_10712	130	73
SCE1572_10975	308	1751
SCE1572_1753	30	40
SCE1572_4250	94	98
SCE1572_5439	148	20

SCE1572_4551	55	4
SCE1572_8107	56	9
SCE1572_7499	50	10
SCE1572_2026	143	47
SCE1572_3377	178	39
SCE1572_6978	27	1
SCE1572_1855	170	25
SCE1572_3220	105	80
SCE1572_3600	70	23
SCE1572_498	524	111
SCE1572_7276	84	5
SCE1572_6901	76	320
SCE1572_10	66	80
SCE1572_10204	1	0
SCE1572_3869	36	16
SCE1572_8184	1	0
SCE1572_434	156	60
SCE1572_5268	20	28
SCE1572_5027	96	321
SCE1572_35	33	60
SCE1572_4988	79.02	65
SCE1572_10524	74	13
SCE1572_6861	14	3
SCE1572_4630	376	45
SCE1572_6095	53	15
SCE1572_8115	39	2
SCE1572_8121	228	37
SCE1572_7359	15	4
SCE1572_5463	76	12
SCE1572_9340	1024	9881
SCE1572_10913	41	55
SCE1572_8039	168	25
SCE1572_3651	86	39
SCE1572_7556	0	0
SCE1572_3259	206	283
SCE1572_478	41	19
SCE1572_1403	1	0
SCE1572_9836	160	22
SCE1572_1179	46	9
SCE1572_5228	92	109
SCE1572_9089	145	24
SCE1572_6589	130	14
SCE1572_7533	0	0
SCE1572_3383	97	400
SCE1572_4498	76	57
SCE1572_10670	5	0
SCE1572_6100	218	19
SCE1572_5861	44	0

SCE1572_7527	125	17
SCE1572_1050	30	7
SCE1572_9842	0	0
SCE1572_8036	9	1
SCE1572_10675	335	115
SCE1572_10599	86	5
SCE1572_4113	75	28
SCE1572_7859	79	66
SCE1572_8602	103	26
SCE1572_8967	29	39
SCE1572_1357	174	202
SCE1572_3544	162	23
SCE1572_4402	21	39
SCE1572_1155	65.39	20
SCE1572_2031	59	6
SCE1572_5019	29	30
SCE1572_5449	129	35
SCE1572_8004	6	1
SCE1572_1692	7	0
SCE1572_5122	15	58
SCE1572_8358	175.24	31.96
SCE1572_7223	813	1310
SCE1572_3218	17	0
SCE1572_5223	488	345
SCE1572_8721	152	56
SCE1572_589	6	1
SCE1572_9849	167	71
SCE1572_936	34	1
SCE1572_8819	22	5
SCE1572_3626	85	11
SCE1572_3821	373	127
SCE1572_6371	8	2
SCE1572_7542	52	112
SCE1572_4382	171	15
SCE1572_97	27	6
SCE1572_6282	3	0
SCE1572_3198	15	68
SCE1572_8156	210	36
SCE1572_10159	4	2
SCE1572_2432	100	104
SCE1572_7672	2.8	0
SCE1572_9825	17.92	19.06
SCE1572_2479	14	20
SCE1572_4578	6	3
SCE1572_7152	33	7
SCE1572_10378	191	34
SCE1572_10430	180	59
SCE1572_9909	110	26

SCE1572_11367	40	7
SCE1572_1191	24	4
SCE1572_7552	25	0
SCE1572_6414	12	1
SCE1572_6544	560	642
SCE1572_9016	0	0
SCE1572_8260	215	364
SCE1572_4507	57	11
SCE1572_6240	396	48
SCE1572_2080	101	34
SCE1572_9806	218	135
SCE1572_4154	1	0
SCE1572_5144	0	0
SCE1572_577	0	0
SCE1572_288	38	64
SCE1572_2057	49	17
SCE1572_1605	203	33
SCE1572_5973	81	8
SCE1572_10826	0	0
SCE1572_7926	15	18
SCE1572_7883	70	6
SCE1572_10437	53	20
SCE1572_8956	14	0
SCE1572_7875	84	36
SCE1572_10012	93	124
SCE1572_2932	182	41
SCE1572_4964	37	37
SCE1572_6759	57	9
SCE1572_10590	149	27
SCE1572_10191	63	25
SCE1572_5542	159	30
SCE1572_7055	478	50
SCE1572_6392	79	60
SCE1572_9870	30	14
SCE1572_10136	19	11
SCE1572_5327	364	3507
SCE1572_482	229	58
SCE1572_9720	70	226
SCE1572_9262	26	12
SCE1572_8741	60	8
SCE1572_10726	28	3
SCE1572_9895	170	99
SCE1572_6782	6	26
SCE1572_9674	155	148
SCE1572_2691	336	257
SCE1572_10691	22	0
SCE1572_8501	14	1
SCE1572_7585	211	192

SCE1572_1457	18	3
SCE1572_6708	92	147
SCE1572_7853	93	124
SCE1572_10581	90	18
SCE1572_1097	132	48
SCE1572_3805	68	136
SCE1572_3044	40	1
SCE1572_4032	1668	162
SCE1572_5716	1318	1511
SCE1572_6186	2	0
SCE1572_6402	67	10
SCE1572_4628	2	0
SCE1572_5155	183	85
SCE1572_887	312	302
SCE1572_5533	393	67
SCE1572_10770	47	31
SCE1572_8765	177	32
SCE1572_5789	178	267
SCE1572_8547	68	7
SCE1572_2178	202	66
SCE1572_10545	35	9
SCE1572_4093	38	3
SCE1572_2797	213	34
SCE1572_2534	0	0
SCE1572_1918	62	20
SCE1572_7257	42	22
SCE1572_5513	23	13
SCE1572_2863	71	11
SCE1572_7494	44	29
SCE1572_10196	44	2
SCE1572_212	142	18
SCE1572_7129	114	53
SCE1572_2452	100	23
SCE1572_3740	168	28
SCE1572_2074	22	7
SCE1572_4627	87	7
SCE1572_9080	12	0
SCE1572_9555	64	252
SCE1572_7462	166	219
SCE1572_2335	104	441
SCE1572_502	22	2
SCE1572_2021	4	0
SCE1572_942	151	18
SCE1572_7024	123	30
SCE1572_10943	213	26
SCE1572_8787	71	124
SCE1572_8312	119	117
SCE1572_3964	175	45

SCE1572_5822	0	0
SCE1572_1217	3	0
SCE1572_5474	22	8
SCE1572_3368	539	777
SCE1572_4543	209	53
SCE1572_3089	32	106
SCE1572_8634	0	0
SCE1572_11341	67	12
SCE1572_249	0	0
SCE1572_4269	72	26
SCE1572_255	17	3
SCE1572_2894	223	40
SCE1572_5703	109	319
SCE1572_7286	112	203
SCE1572_6689	3	0
SCE1572_9296	115	119
SCE1572_4849	183	103
SCE1572_9215	20	7
SCE1572_123	6	0
SCE1572_6315	83	19
SCE1572_5292	39	233
SCE1572_8649	50	11
SCE1572_9463	16	0
SCE1572_2684	175	722
SCE1572_4601	24	10
SCE1572_9935	28	4
SCE1572_6630	25	352
SCE1572_10530	54	59
SCE1572_2636	199	30
SCE1572_8044	30	7
SCE1572_1378	88	17
SCE1572_8685	133	24
SCE1572_486	25	3
SCE1572_365	66	13
SCE1572_2302	309	378
SCE1572_121	19	8
SCE1572_4296	103	37
SCE1572_6971	194	16
SCE1572_3694	151	37
SCE1572_4725	235	100
SCE1572_6397	75	96
SCE1572_5241	35	18
SCE1572_10462	156	618
SCE1572_11380	12	43
SCE1572_9357	279	1054
SCE1572_9527	35	30
SCE1572_8625	126	61
SCE1572_5551	115	14

SCE1572_846	108	67
SCE1572_9993	254.78	39
SCE1572_90	246.87	222
SCE1572_2350	802	619
SCE1572_3225	67	17
SCE1572_3534	0	0
SCE1572_2545	39	3
SCE1572_410	16	227
SCE1572_9283	61	104
SCE1572_8748	42	5
SCE1572_2722	127	134
SCE1572_5560	163.05	41.78
SCE1572_133	329.13	244
SCE1572_10953	25	19
SCE1572_3674	207	731
SCE1572_7200	236	106
SCE1572_328	12	1
SCE1572_10226	60	7
SCE1572_2561	465	462
SCE1572_3145	3	1
SCE1572_9123	86	26
SCE1572_5681	107	23
SCE1572_6693	0	0
SCE1572_10637	10	2
SCE1572_8691	140	38
SCE1572_8927	8	2
SCE1572_9958	34	4
SCE1572_10833	0	0
SCE1572_1262	115	190
SCE1572_9669	47	71
SCE1572_6487	47	2
SCE1572_6995	224	20
SCE1572_69	150	26
SCE1572_3623	85	8
SCE1572_5392	136	45
SCE1572_9704	147	93
SCE1572_7443	120	36
SCE1572_6913	117	14
SCE1572_2264	38	267
SCE1572_5908	686	708
SCE1572_9749	74	45
SCE1572_8709	33	6
SCE1572_11324	114	667
SCE1572_4344	6	0
SCE1572_4460	17	3
SCE1572_5200	74	22
SCE1572_9011	34	18
SCE1572_388	40	17

SCE1572_5274	15	4
SCE1572_1817	159	60
SCE1572_5745	15	51
SCE1572_4712	575	195
SCE1572_6918	45	25
SCE1572_3283	223	32
SCE1572_6264	97	8
SCE1572_6525	36	12
SCE1572_8486	254	38
SCE1572_11462	180	129
SCE1572_7570	19	12
SCE1572_3874	152	73
SCE1572_8016	113	9
SCE1572_3149	63	23
SCE1572_4682	80	8
SCE1572_242	102	61
SCE1572_8810	72	11
SCE1572_7272	67	16
SCE1572_8240	29	9
SCE1572_8561	160	63
SCE1572_1914	144	35
SCE1572_2516	39	10
SCE1572_5258	3	0
SCE1572_8204	1	0
SCE1572_4438	101	21
SCE1572_5809	145	76
SCE1572_9378	0	0
SCE1572_4764	47	13
SCE1572_3913	183	195
SCE1572_5323	38	23
SCE1572_8400	78	65
SCE1572_2095	83	35
SCE1572_5652	91	432
SCE1572_2594	141	63
SCE1572_8568	13.01	2.37
SCE1572_1872	57	121
SCE1572_4160	48	21
SCE1572_62	18	0
SCE1572_7837	41	15
SCE1572_2926	15	2
SCE1572_6074	421	651
SCE1572_6508	1	0
SCE1572_1283	0	0
SCE1572_4167	31	8
SCE1572_4476	38	15
SCE1572_2711	157	192
SCE1572_6835	34	140
SCE1572_5628	41	5

SCE1572_6987	96	10
SCE1572_10093	233	281
SCE1572_1950	37	16
SCE1572_9931	47	80
SCE1572_70	311	70
SCE1572_8973	57	38
SCE1572_9579	251	40
SCE1572_892	0	0
SCE1572_9037	132	101
SCE1572_10413	1	0
SCE1572_612	55	145
SCE1572_10019	129	92
SCE1572_1281	231	24
SCE1572_5173	10	0
SCE1572_10262	85	23
SCE1572_2511	144	304
SCE1572_5595	0	0
SCE1572_10001	34	5
SCE1572_3831	50	26
SCE1572_9877	0	0
SCE1572_8805	30	4
SCE1572_6141	40	9
SCE1572_825	160	21
SCE1572_880	839	1353
SCE1572_1225	29	5
SCE1572_9901	1	0
SCE1572_8551	147	65
SCE1572_3786	757	469
SCE1572_4697	0	0
SCE1572_7890	590	10417
SCE1572_8891	234	41
SCE1572_5591	196	22
SCE1572_9240	0	0
SCE1572_10866	20	8
SCE1572_2877	78	56
SCE1572_4655	0	0
SCE1572_4186	53	16
SCE1572_8825	18	10
SCE1572_5526	66	25
SCE1572_11054	31	9
SCE1572_2167	90.1	10.62
SCE1572_7503	170	95
SCE1572_9629	182	26
SCE1572_8844	0	0
SCE1572_7384	613	201
SCE1572_3987	120	1568
SCE1572_11577	45	7
SCE1572_8317	33	83

SCE1572_10787	177	110
SCE1572_3263	607	2798
SCE1572_1008	180	41
SCE1572_875	338	109
SCE1572_5096	201	97
SCE1572_5943	98	14
SCE1572_710	82	31
SCE1572_2802	150	51
SCE1572_5378	184	18
SCE1572_2976	13.84	1
SCE1572_755	30	7
SCE1572_2236	297	119
SCE1572_7813	2	0
SCE1572_51	263	34
SCE1572_10242	123	13
SCE1572_1388	79	55
SCE1572_6108	209	27
SCE1572_3811	58	8
SCE1572_6359	36	6
SCE1572_1890	92	75
SCE1572_3053	43	7
SCE1572_11192	52	18
SCE1572_2761	146	351
SCE1572_9706	270	99
SCE1572_10247	0	0
SCE1572_3471	4	0
SCE1572_976	0	0
SCE1572_761	41	14
SCE1572_7364	182	145
SCE1572_10054	252	518
SCE1572_1221	132	10
SCE1572_6311	53	921
SCE1572_10215	1	0
SCE1572_959	90	74
SCE1572_1330	189	24
SCE1572_5145	59	29
SCE1572_9951	134	48
SCE1572_7085	96	23
SCE1572_2162	40	5
SCE1572_9798	10	0
SCE1572_11547	22.41	1.49
SCE1572_5635	868	261
SCE1572_3451	98	8
SCE1572_9499	337	1134
SCE1572_353	47	8
SCE1572_4594	135	49
SCE1572_568	17	50
SCE1572_11407	78	18

SCE1572_3137	237	186
SCE1572_855	429	1154
SCE1572_11031	37	66
SCE1572_8422	194	648
SCE1572_7761	16	2
SCE1572_9733	418	73
SCE1572_7696	275	67
SCE1572_3235	2704	907
SCE1572_4007	191	196
SCE1572_10745	42	13
SCE1572_7709	2607	16428
SCE1572_4292	53	12
SCE1572_5750	51	3
SCE1572_4854	27	8
SCE1572_981	269	40
SCE1572_1808	22	24
SCE1572_9022	70	49
SCE1572_379	75	142
SCE1572_3993	13	15
SCE1572_104	45	2
SCE1572_1780	54	44
SCE1572_11427	106	40
SCE1572_7302	121	14
SCE1572_9641	283	85
SCE1572_5423	257	46
SCE1572_3505	174	26
SCE1572_493	132.07	8
SCE1572_5997	63	52
SCE1572_6565	36	9
SCE1572_1450	17	19
SCE1572_7448	131	81
SCE1572_360	102	52
SCE1572_7164	58	8
SCE1572_9278	7	0
SCE1572_1755	39	70
SCE1572_6911	290	38
SCE1572_2904	21	1
SCE1572_5507	3	0
SCE1572_9457	128	18
SCE1572_10643	198	279
SCE1572_9461	108	68
SCE1572_3393	14	1
SCE1572_5994	197	83
SCE1572_9724	98	25
SCE1572_3303	52	32
SCE1572_9648	42	29
SCE1572_8230	52	41
SCE1572_7175	285	356

SCE1572_1597	259	51
SCE1572_1772	171	21
SCE1572_9230	80	9
SCE1572_2218	256	156
SCE1572_3856	293	62
SCE1572_131	10	10
SCE1572_2322	18	26
SCE1572_10255	133	29
SCE1572_7968	86	40
SCE1572_8636	6	4
SCE1572_4582	330	74
SCE1572_6194	106	79
SCE1572_9359	78	42
SCE1572_11153	257	993
SCE1572_5701	14	26
SCE1572_10506	0	0
SCE1572_3061	8	0
SCE1572_5502	19	3
SCE1572_7785	1	0
SCE1572_8732	0	0
SCE1572_6043	0	0
SCE1572_3710	1743	23984
SCE1572_6345	48	10
SCE1572_5267	125	178
SCE1572_9886	231	294
SCE1572_471	39	33
SCE1572_10559	8	3
SCE1572_7470	169	69
SCE1572_5319	133	21
SCE1572_322	59	14
SCE1572_2285	7	42
SCE1572_3656	35	20
SCE1572_5509	21	5
SCE1572_6039	32	46
SCE1572_3446	0	0
SCE1572_9213	231	23
SCE1572_8647	42	5
SCE1572_1183	73	20
SCE1572_549	0	0
SCE1572_5579	163	42
SCE1572_9783	105	60
SCE1572_11086	169	145
SCE1572_4567	112	40
SCE1572_521	16	4
SCE1572_9592	59	301
SCE1572_8281	45	25
SCE1572_4119	60	4942
SCE1572_48	52	6

SCE1572_2892	78	17
SCE1572_3724	39	311
SCE1572_1474	45	5
SCE1572_506	8	1
SCE1572_7475	396	773
SCE1572_1911	112	28
SCE1572_3323	0	0
SCE1572_11347	76	49
SCE1572_9730	125	94
SCE1572_9786	143	16
SCE1572_2948	43	10
SCE1572_4450	16	8
SCE1572_5165	277	797
SCE1572_4889	46	17
SCE1572_901	65	14
SCE1572_9353	129	168
SCE1572_7189	196	48
SCE1572_8641	140	26
SCE1572_1814	48	6
SCE1572_11280	100	294
SCE1572_6133	210	630
SCE1572_10760	11	96
SCE1572_7355	466	166
SCE1572_9941	31	15
SCE1572_9029	111	16
SCE1572_8331	137	249
SCE1572_3312	69	35
SCE1572_7168	259	276
SCE1572_10801	0	0
SCE1572_5901	144	36
SCE1572_11265	230	287
SCE1572_3314	138	52
SCE1572_6845	222	413
SCE1572_8800	37	1
SCE1572_5637	129	26
SCE1572_6046	373	322
SCE1572_3087	13	4
SCE1572_1642	19	7
SCE1572_2744	49	172
SCE1572_1090	57	9
SCE1572_3961	359	215
SCE1572_2570	20	0
SCE1572_5773	0	0
SCE1572_9200	0	0
SCE1572_7656	7	3
SCE1572_2844	3.5	0
SCE1572_6331	67	215
SCE1572_10931	34	10

SCE1572_3942	5898	3061
SCE1572_6051	55	59
SCE1572_10185	82	27
SCE1572_6058	27	40
SCE1572_9595	24	287
SCE1572_8739	124	16
SCE1572_3923	138	24
SCE1572_595	88	11
SCE1572_899	125	30
SCE1572_8987	72	60
SCE1572_4892	108	70
SCE1572_11327	29	23
SCE1572_1435	57	27
SCE1572_3601	26	10
SCE1572_5929	368	49
SCE1572_9060	308	67
SCE1572_11363	227	57
SCE1572_141	46.02	3
SCE1572_8533	152	33
SCE1572_126	51	11
SCE1572_6276	59	111
SCE1572_8026	1	0
SCE1572_7299	1002	249
SCE1572_4599	6	0
SCE1572_991	0	0
SCE1572_1139	96	27
SCE1572_10070	632	660
SCE1572_806	0	0
SCE1572_4994	106	23
SCE1572_8445	26	9
SCE1572_10567	135	37
SCE1572_8520	28	1
SCE1572_10369	389	67
SCE1572_10311	10	1
SCE1572_2005	112	327
SCE1572_4882	114	33
SCE1572_1555	0	0
SCE1572_317	62	21
SCE1572_445	128	22
SCE1572_979	0	0
SCE1572_7172	110	28
SCE1572_1996	110	11
SCE1572_6128	548	2368
SCE1572_7133	193	35
SCE1572_4811	92	91
SCE1572_9762	47	33
SCE1572_9982	99	25
SCE1572_6171	102	30

SCE1572_8673	51	5
SCE1572_3457	0	0
SCE1572_5921	0	0
SCE1572_9030	123	25
SCE1572_7051	55	3
SCE1572_2298	58	42
SCE1572_264	41	20
SCE1572_4590	4	0
SCE1572_10212	107	22
SCE1572_6004	76	100
SCE1572_753	58	27
SCE1572_7410	0	0
SCE1572_5249	140	114
SCE1572_9385	9	8
SCE1572_9115	151	160
SCE1572_7995	85	7
SCE1572_6124	20	22
SCE1572_11409	43	12
SCE1572_2768	6	1
SCE1572_7263	29	8
SCE1572_50	42	4
SCE1572_11533	69	12
SCE1572_1765	43	2
SCE1572_11082	70	50
SCE1572_1741	327	149
SCE1572_11488	147.7	19.18
SCE1572_4447	164	42
SCE1572_9371	37	18
SCE1572_5386	93	26
SCE1572_6626	60	34
SCE1572_11525	52	13
SCE1572_2136	51	3
SCE1572_6787	102	42
SCE1572_4190	121	84
SCE1572_6664	102	33
SCE1572_6784	312	82
SCE1572_6752	19	7
SCE1572_2705	13	6
SCE1572_6641	90	26
SCE1572_10376	116	10
SCE1572_4902	38	10
SCE1572_5791	121	59
SCE1572_3153	0	0
SCE1572_2163	34	8
SCE1572_10382	48	19
SCE1572_370	53	21
SCE1572_2667	413	76
SCE1572_2414	298	92

SCE1572_10814	46	23
SCE1572_3057	47	5
SCE1572_1897	6	2
SCE1572_9753	41	4
SCE1572_5089	17	11
SCE1572_1294	131	32
SCE1572_6144	0	0
SCE1572_10140	59	27
SCE1572_7114	108	28
SCE1572_1279	104	16
SCE1572_8887	17	0
SCE1572_1823	18	19
SCE1572_2123	88	53
SCE1572_3187	4	0
SCE1572_11415	45	10
SCE1572_10345	72	21
SCE1572_2913	773.95	95.11
SCE1572_8498	0	0
SCE1572_4505	1	0
SCE1572_6241	40	17
SCE1572_4745	84	85
SCE1572_5622	122	88
SCE1572_4859	124	15
SCE1572_1619	316	1801
SCE1572_9074	248	25
SCE1572_4182	30	8
SCE1572_6428	66	5
SCE1572_1200	86	11
SCE1572_2403	38	15
SCE1572_169	122	119
SCE1572_2147	134	20
SCE1572_2858	250	261
SCE1572_4319	110	7
SCE1572_6771	6	4
SCE1572_1600	83.99	36
SCE1572_4320	156	40
SCE1572_6639	21	3
SCE1572_4314	98.48	13.09
SCE1572_10287	1430	1003
SCE1572_8750	0	0
SCE1572_9152	127	68
SCE1572_10723	174	83
SCE1572_10154	177	501
SCE1572_4874	775	1034
SCE1572_7924	79	31
SCE1572_9638	42	74
SCE1572_8698	139	51
SCE1572_2124	303	39

SCE1572_10576	861	140
SCE1572_7635	2180	9329
SCE1572_3774	99	15
SCE1572_3716	339	50
SCE1572_6299	17	7
SCE1572_7686	4	0
SCE1572_1274	18	2
SCE1572_6252	239	142
SCE1572_6609	56	20
SCE1572_8885	1	0
SCE1572_9557	59	209
SCE1572_1612	31	22
SCE1572_4571	47	15
SCE1572_9830	0	0
SCE1572_3278	69	98
SCE1572_7391	144	25
SCE1572_1209	64	5
SCE1572_4821	110	7
SCE1572_8572	0	0
SCE1572_10330	0	0
SCE1572_5988	0	0
SCE1572_1259	10	1
SCE1572_10106	332	651
SCE1572_6438	1	0
SCE1572_10783	126	32
SCE1572_5617	172	30
SCE1572_11470	44	15
SCE1572_5981	32	22
SCE1572_863	105	133
SCE1572_531	99	302
SCE1572_5798	134	20
SCE1572_1124	98	14
SCE1572_4062	106	325
SCE1572_2718	199.98	30
SCE1572_5647	84	17
SCE1572_6612	18	4
SCE1572_1527	27	32
SCE1572_10927	191	283
SCE1572_9542	11	3
SCE1572_10666	117	152
SCE1572_6060	92	41
SCE1572_6409	10	0
SCE1572_1104	57	60
SCE1572_11551	2	0
SCE1572_7721	28	68
SCE1572_7035	172	22
SCE1572_1782	12	31
SCE1572_8268	1	0

SCE1572_2600	10	5
SCE1572_555	97	31
SCE1572_3953	126	71
SCE1572_6219	75	16
SCE1572_3345	715	2021
SCE1572_3497	246	521
SCE1572_3958	63	37
SCE1572_10381	99	26
SCE1572_3882	54	4
SCE1572_8984	40	1
SCE1572_10397	67	52
SCE1572_281	100	34
SCE1572_5312	206	47
SCE1572_7361	8	7
SCE1572_3675	0	0
SCE1572_9396	431	430
SCE1572_7738	332	140
SCE1572_562	171	410
SCE1572_6150	9	0
SCE1572_7984	209	249
SCE1572_8237	55	140
SCE1572_542	75	3
SCE1572_4936	34	25
SCE1572_5663	112	11
SCE1572_7736	39	43
SCE1572_4300	257	59
SCE1572_2611	66	16
SCE1572_6117	42	15
SCE1572_2682	74	21
SCE1572_3574	46	10
SCE1572_6739	1	0
SCE1572_391	0	0
SCE1572_8796	41	1
SCE1572_7199	59	111
SCE1572_9737	74	44
SCE1572_3351	111	52
SCE1572_3110	133	20
SCE1572_9145	39	40
SCE1572_3117	176	237
SCE1572_4361	28	3
SCE1572_9450	87	21
SCE1572_4265	57	6
SCE1572_5693	21	4
SCE1572_9495	94	70
SCE1572_9180	42	5
SCE1572_10743	35	16
SCE1572_2245	265.41	90
SCE1572_3425	37	9

SCE1572_6412	59	9
SCE1572_2924	320	61
SCE1572_4915	240	1161
SCE1572_9529	130	81
SCE1572_9691	84	199
SCE1572_1324	18	4
SCE1572_4025	175	113
SCE1572_2232	8	6
SCE1572_8014	83	21
SCE1572_9414	419	2759
SCE1572_4791	145	46
SCE1572_1212	29	10
SCE1572_1130	0	0
SCE1572_9997	423	366
SCE1572_11055	130	181
SCE1572_2436	21	45
SCE1572_10706	121	52
SCE1572_9771	22	7
SCE1572_3241	165	22
SCE1572_3565	0	0
SCE1572_6551	36	2
SCE1572_7797	95	33
SCE1572_2047	0	0
SCE1572_2638	215	49
SCE1572_4737	79	171
SCE1572_7326	80	64
SCE1572_11410	63	12
SCE1572_2962	1	0
SCE1572_7866	441	906
SCE1572_10452	81	114
SCE1572_3192	156	72
SCE1572_4075	48	57
SCE1572_4837	178	182
SCE1572_10618	131	53
SCE1572_10698	51	91
SCE1572_7161	39	9
SCE1572_4645	230	168
SCE1572_20	112	398
SCE1572_3596	147.7	19.18
SCE1572_2803	32	41
SCE1572_1042	0	0
SCE1572_4039	31	2
SCE1572_257	40	6
SCE1572_6813	323	55
SCE1572_9420	45	110
SCE1572_4325	51	10
SCE1572_4781	1218	660
SCE1572_9250	303	37

SCE1572_7950	188	119
SCE1572_6192	54	21
SCE1572_10920	25	25
SCE1572_1731	519	86
SCE1572_2107	159	134
SCE1572_2605	24	4
SCE1572_9055	84	11
SCE1572_9209	103	141
SCE1572_8759	241	331
SCE1572_9245	7	0
SCE1572_11065	2	4
SCE1572_5198	0	0
SCE1572_6931	6	0
SCE1572_4783	557	110.67
SCE1572_11061	3142.49	3711.59
SCE1572_9485	60	45
SCE1572_8830	185	262
SCE1572_8374	39	35
SCE1572_68	505	452
SCE1572_11142	0	4
SCE1572_6652	59	10
SCE1572_7077	0	0
SCE1572_2640	459	115
SCE1572_2725	119	54
SCE1572_3014	84	10
SCE1572_450	73	20
SCE1572_1795	144	232
SCE1572_4101	350	2589
SCE1572_8747	8	0
SCE1572_5301	15	8
SCE1572_1337	188	223
SCE1572_11123	213	44
SCE1572_2601	0	0
SCE1572_3983	27	12
SCE1572_3966	20	27
SCE1572_4421	0	0
SCE1572_5048	37	3
SCE1572_5111	933	1147
SCE1572_7320	41	5
SCE1572_7500	21	12
SCE1572_6916	40	17
SCE1572_9912	40	5
SCE1572_10536	81	164
SCE1572_4331	19	3
SCE1572_1539	110	57
SCE1572_8220	172	64
SCE1572_4487	91	59
SCE1572_11000	19	3

SCE1572_8702	71	125
SCE1572_9568	134	35
SCE1572_10051	273	175
SCE1572_10004	40	7
SCE1572_9977	221	43
SCE1572_336	76	20
SCE1572_11574	107	51
SCE1572_4822	0	0
SCE1572_4353	295	241
SCE1572_4919	67	7
SCE1572_6558	59	16
SCE1572_10821	0	0
SCE1572_2291	369	479
SCE1572_7956	63	116
SCE1572_9425	46	13
SCE1572_7896	2599	42290
SCE1572_2971	90.05	17
SCE1572_4511	111	27
SCE1572_426	32	113
SCE1572_6790	13	1
SCE1572_5428	20	16
SCE1572_7525	308	156
SCE1572_9049	184	49
SCE1572_512	11	0
SCE1572_3862	87	67
SCE1572_6090	44	396
SCE1572_9185	15	0
SCE1572_4637	130	41
SCE1572_7396	187	61
SCE1572_10907	356	1075
SCE1572_3257	51	10
SCE1572_929	105	87
SCE1572_5896	110	11
SCE1572_11331	44	34
SCE1572_10064	0	0
SCE1572_3484	36	10
SCE1572_8715	147	62
SCE1572_9419	73	344
SCE1572_5091	263	254
SCE1572_8637	119	49
SCE1572_6507	191	40
SCE1572_10407	43	26
SCE1572_9222	88	34
SCE1572_1467	478	344
SCE1572_2398	103	657
SCE1572_7251	0	0
SCE1572_5399	0	0
SCE1572_10400	74	252

SCE1572_4243	77	18
SCE1572_2424	349	260
SCE1572_294	0	0
SCE1572_8473	153	25
SCE1572_8162	2	3
SCE1572_4395	0	0
SCE1572_8868	146	25
SCE1572_2685	0	0
SCE1572_7221	12	42
SCE1572_1197	543	64
SCE1572_11198	63	23
SCE1572_2631	140	34
SCE1572_10139	0	0
SCE1572_9005	19	3
SCE1572_8391	141	24
SCE1572_8136	10	2
SCE1572_10769	9	3
SCE1572_4632	91	17
SCE1572_5107	153	97
SCE1572_10960	13	4
SCE1572_10903	49	8
SCE1572_4613	92	21
SCE1572_5029	91	149
SCE1572_10967	63	12
SCE1572_4385	376	70
SCE1572_7560	104	18
SCE1572_7401	13	450
SCE1572_8207	62	32
SCE1572_4532	0	0
SCE1572_3216	139	92
SCE1572_6303	1	0
SCE1572_2040	129	340
SCE1572_7043	49	317
SCE1572_6022	0	0
SCE1572_8344	39	4
SCE1572_1011	42	6
SCE1572_10174	0	0
SCE1572_1063	19	5
SCE1572_8443	10	2
SCE1572_9138	35	2
SCE1572_615	2	0
SCE1572_10350	11	3
SCE1572_3165	200	31
SCE1572_3794	33	8
SCE1572_5150	52	3
SCE1572_6257	42	10
SCE1572_9988	44	9
SCE1572_894	184	121

SCE1572_9474	217	1120
SCE1572_8585	26	7
SCE1572_10426	7	4
SCE1572_5071	197	134
SCE1572_2272	143	102
SCE1572_10750	25	14
SCE1572_8515	48.38	15.9
SCE1572_6029	180	241
SCE1572_5278	40	9
SCE1572_2156	6	7
SCE1572_2015	5	2
SCE1572_3849	66	2
SCE1572_4604	93	52
SCE1572_2987	181	225
SCE1572_2329	133	33
SCE1572_3356	19	2
SCE1572_10308	51	3
SCE1572_7715	32	1
SCE1572_7644	218	78
SCE1572_10325	104	61
SCE1572_7438	347	1317
SCE1572_2526	43	4
SCE1572_8878	45	8
SCE1572_8837	14	4
SCE1572_6249	9	1
SCE1572_9174	154	69
SCE1572_11119	426	688
SCE1572_2585	53	6
SCE1572_10735	13	6
SCE1572_3646	113	88
SCE1572_416	126	26
SCE1572_6683	100	231
SCE1572_9588	402	173
SCE1572_3135	14	9
SCE1572_9400	9	12
SCE1572_8328	0	0
SCE1572_3492	0	0
SCE1572_393	42	8
SCE1572_9850	0	0
SCE1572_1697	59	26
SCE1572_11007	282	218
SCE1572_6234	84	25
SCE1572_10360	145	77
SCE1572_3075	115	30
SCE1572_9632	311	340
SCE1572_8067	17	4
SCE1572_3639	17	12
SCE1572_11024	127	142

SCE1572_4518	248	152
SCE1572_9313	91	33
SCE1572_9105	76	55
SCE1572_5778	74	38
SCE1572_8545	112	35
SCE1572_2328	56	22
SCE1572_4660	74	498
SCE1572_2886	103	34
SCE1572_6515	22	2
SCE1572_716	315	58
SCE1572_11100	173	180
SCE1572_10532	0	0
SCE1572_10470	22	11
SCE1572_7912	281	14
SCE1572_10337	15	0
SCE1572_7947	13	0
SCE1572_2083	1	0
SCE1572_1110	1	0
SCE1572_8827	23.45	0
SCE1572_2194	20	5
SCE1572_10030	60	8
SCE1572_3730	0	0
SCE1572_8507	33	3
SCE1572_2461	43.67	0
SCE1572_6034	244	181
SCE1572_95	25	2
SCE1572_2784	119	12
SCE1572_8157	54	2
SCE1572_10079	54	4
SCE1572_9866	75	52
SCE1572_8146	52	21
SCE1572_10516	947	197
SCE1572_4218	41	72
SCE1572_9367	88	14
SCE1572_4778	31	43
SCE1572_1635	18	8
SCE1572_10025	41	28
SCE1572_5114	395	487
SCE1572_10477	26	33
SCE1572_455	59	19
SCE1572_420	28	9
SCE1572_8500	31	1
SCE1572_2734	643	501
SCE1572_1309	4762.36	14491.82
SCE1572_3739	2	0
SCE1572_10881	0	0
SCE1572_5343	135	52
SCE1572_10119	74	27

SCE1572_4197	116	47
SCE1572_6065	0	0
SCE1572_10447	208	110
SCE1572_919	64	44
SCE1572_7846	3	1
SCE1572_10622	317	99
SCE1572_8994	77	53
SCE1572_7248	16	2
SCE1572_7569	124	46
SCE1572_11422	80	33
SCE1572_7070	1	0
SCE1572_11586	223	217
SCE1572_910	665.17	166.09
SCE1572_1202	58	8
SCE1572_11136	143	81
SCE1572_7782	0	0
SCE1572_1236	288	133
SCE1572_9647	773	393
SCE1572_1985	562	10967
SCE1572_7882	69.57	46.42
SCE1572_7145	142	40
SCE1572_2384	89	68
SCE1572_236	3	0
SCE1572_2647	225	84
SCE1572_1819	111	25
SCE1572_2377	34	84
SCE1572_7936	52	9
SCE1572_2356	180	266
SCE1572_9303	182	438
SCE1572_5814	84	16
SCE1572_1463	96	58
SCE1572_10889	98	63
SCE1572_1495	0	0
SCE1572_1663	0	0
SCE1572_11029	3	0
SCE1572_1707	1776	174
SCE1572_9228	0	0
SCE1572_153	37	9
SCE1572_2861	0	0
SCE1572_723	57	8
SCE1572_2308	116	128
SCE1572_604	209	253
SCE1572_679	85	36
SCE1572_10686	81	163
SCE1572_1971	27	16
SCE1572_10373	154	78
SCE1572_4806	369	493
SCE1572_5487	110	32

SCE1572_1965	67	9
SCE1572_5539	192	190
SCE1572_10759	6	6
SCE1572_173	14	0
SCE1572_6537	53	15
SCE1572_10794	40	21
SCE1572_3764	206	44
SCE1572_6084	37	18
SCE1572_693	171	52
SCE1572_666	178	304
SCE1572_1978	58	257
SCE1572_3896	273	751
SCE1572_10251	1	0
SCE1572_5291	9	4
SCE1572_1394	79	118
SCE1572_4340	185	29
SCE1572_10897	45	40
SCE1572_682	191	19
SCE1572_7292	21	4
SCE1572_9259	70	18
SCE1572_8790	184	95
SCE1572_7469	125	57
SCE1572_6260	184	39
SCE1572_1956	14	2
SCE1572_2918	16	6
SCE1572_2174	81	10
SCE1572_6393	312	241
SCE1572_8176	248	27
SCE1572_3842	35	15
SCE1572_9839	0	0
SCE1572_9291	30	60
SCE1572_2569	14	6
SCE1572_5878	104	31
SCE1572_6621	43	12
SCE1572_1250	21	9
SCE1572_2481	472	1114
SCE1572_1443	205	36
SCE1572_9605	10	0
SCE1572_2851	21	2
SCE1572_4807	328	207
SCE1572_5850	129	28
SCE1572_11390	87	31
SCE1572_3588	86	8
SCE1572_11314	362	207
SCE1572_9561	354	53
SCE1572_10297	27	24
SCE1572_2444	14	7
SCE1572_5692	92	33

SCE1572_134	0	0
SCE1572_2911	109	21
SCE1572_6578	86	20
SCE1572_8029	90	69
SCE1572_10161	72	40
SCE1572_7405	70	37
SCE1572_9112	58	27
SCE1572_4674	38	10
SCE1572_7949	165	152
SCE1572_2574	17	7
SCE1572_8803	16	25
SCE1572_4714	56	10
SCE1572_1901	18	44
SCE1572_4723	47	20
SCE1572_6809	988	2496
SCE1572_10509	36	46
SCE1572_2391	75	892
SCE1572_2743	84	57
SCE1572_6907	70	12
SCE1572_5152	0	0
SCE1572_5867	81	12
SCE1572_1846	28	6
SCE1572_5743	29.09	93.21
SCE1572_1734	12	2
SCE1572_9620	8	2
SCE1572_8600	3	1
SCE1572_6169	0	0
SCE1572_828	32	3
SCE1572_8034	29	8
SCE1572_742	123	106
SCE1572_1727	243	81
SCE1572_11246	216	21
SCE1572_5776	110	105
SCE1572_8817	0	0
SCE1572_10285	16	15
SCE1572_8917	53	64
SCE1572_2543	130	25
SCE1572_8198	1	0
SCE1572_7195	143	20
SCE1572_10630	134	22
SCE1572_1356	149	354
SCE1572_1508	85	10
SCE1572_6883	171	58
SCE1572_7908	0	0
SCE1572_7578	31	75
SCE1572_3102	8	9
SCE1572_7803	1	0
SCE1572_9840	64	0

SCE1572_11005	151	71
SCE1572_9430	50	9
SCE1572_7844	224	2837
SCE1572_11045	30	5
SCE1572_1832	24	11
SCE1572_8052	70	3
SCE1572_7489	9	33
SCE1572_6724	95	51
SCE1572_6093	72	12
SCE1572_1386	0	0
SCE1572_2617	56	218
SCE1572_6804	70	13
SCE1572_8992	75	50
SCE1572_87	52	23
SCE1572_9864	17	4
SCE1572_2985	39	15
SCE1572_1161	45	4
SCE1572_10306	34	47
SCE1572_2006	59	103
SCE1572_3636	109	10
SCE1572_5221	28	15
SCE1572_9747	39	29
SCE1572_6651	3	4
SCE1572_6908	126	10
SCE1572_7666	7	4
SCE1572_10489	160	725
SCE1572_214	61	5
SCE1572_6837	54	46
SCE1572_4950	76	22
SCE1572_5054	19	7
SCE1572_5492	97	32
SCE1572_11260	270	125
SCE1572_7117	199	49
SCE1572_4705	25	10
SCE1572_7616	207	124
SCE1572_8172	48	8
SCE1572_1056	106.42	31.86
SCE1572_3252	665	195
SCE1572_7940	438	322
SCE1572_5188	77	853
SCE1572_1119	64	7
SCE1572_7815	3485	17984
SCE1572_7580	45	70
SCE1572_3920	183	24
SCE1572_9293	78	84
SCE1572_5832	54	39
SCE1572_1481	77	17
SCE1572_3558	10	0

SCE1572_3828	94	135
SCE1572_1419	3	0
SCE1572_419	78	63
SCE1572_1501	0	0
SCE1572_144	27	8
SCE1572_5131	11	3
SCE1572_6385	0	0
SCE1572_4172	292	118
SCE1572_10688	141	690
SCE1572_4452	26	5
SCE1572_8416	143	144
SCE1572_3628	33	9
SCE1572_3546	69	4
SCE1572_5752	499	487
SCE1572_5127	69	22
SCE1572_620	164	107
SCE1572_10809	3	3
SCE1572_5722	40	16
SCE1572_4957	3	1
SCE1572_2253	452	242
SCE1572_5957	31	17
SCE1572_538	67	59
SCE1572_4404	0	0
SCE1572_11568	317	890
SCE1572_8646	88	20
SCE1572_10264	159	31
SCE1572_5960	36	8
SCE1572_3436	43	41
SCE1572_9409	354	1529
SCE1572_10671	21	2
SCE1572_9235	36	3
SCE1572_8910	92	20
SCE1572_1180	24	15
SCE1572_1006	162	169
SCE1572_8278	61	18
SCE1572_10454	0	0
SCE1572_8689	75	28
SCE1572_8458	488	1600
SCE1572_2038	40	27
SCE1572_3222	90	92
SCE1572_1189	106	69
SCE1572_347	63	59
SCE1572_8846	30	20
SCE1572_10979	104	54
SCE1572_1401	69	23
SCE1572_10677	20	25
SCE1572_4966	12	8
SCE1572_3038	235	31

SCE1572_957	191	260
SCE1572_7710	30	36
SCE1572_272	0	0
SCE1572_4163	152	27
SCE1572_5331	81	70
SCE1572_1157	36	7
SCE1572_2781	7	4
SCE1572_920	1	0
SCE1572_6623	148	41
SCE1572_1314	275	1085
SCE1572_9289	18	12
SCE1572_4184	86	149
SCE1572_1720	34	40
SCE1572_5858	1	0
SCE1572_3386	1	0
SCE1572_1582	134	16
SCE1572_3888	165	19
SCE1572_9063	202	19
SCE1572_8540	118	29
SCE1572_3381	87	44
SCE1572_8614	133	83
SCE1572_10445	54	19
SCE1572_2994	1	0
SCE1572_3607	78	21
SCE1572_3653	6	0
SCE1572_9497	231	148
SCE1572_2494	76	19
SCE1572_3280	118	78
SCE1572_5348	520	304
SCE1572_8186	0	0
SCE1572_10651	218	231
SCE1572_2649	76	21
SCE1572_9087	157	24
SCE1572_4975	773	2406
SCE1572_10757	100	41
SCE1572_6868	24	3
SCE1572_1518	152	47
SCE1572_5017	88	39
SCE1572_818	6	1
SCE1572_791	150	284
SCE1572_6863	139	21
SCE1572_9922	0	0
SCE1572_7428	58	11
SCE1572_4034	278	109
SCE1572_10238	56	10
SCE1572_6815	165	30
SCE1572_5764	197	43
SCE1572_1690	18	4

SCE1572_2755	217	64
SCE1572_7694	120	66
SCE1572_10853	177	33
SCE1572_1712	92	18
SCE1572_2500	78	8
SCE1572_627	101	53
SCE1572_1093	32	18
SCE1572_10611	9	9
SCE1572_4248	105	122
SCE1572_7066	147	15
SCE1572_1044	14	9
SCE1572_2109	42	138
SCE1572_1679	134	174
SCE1572_8848	216	50
SCE1572_3688	277	39
SCE1572_203	87	35
SCE1572_2045	94	47
SCE1572_9969	25	17
SCE1572_4029	208	95
SCE1572_7212	1	0
SCE1572_5431	42	12
SCE1572_2059	180	17
SCE1572_2503	160	26
SCE1572_3784	40	753
SCE1572_6732	70	124
SCE1572_2309	32	46
SCE1572_657	991	343
SCE1572_5021	266	440
SCE1572_8215	14	1
SCE1572_190	75	20
SCE1572_7492	44	11
SCE1572_4131	22	12
SCE1572_7851	77	58
SCE1572_10848	65	16
SCE1572_4642	199	29
SCE1572_4834	0	0
SCE1572_9966	14	4
SCE1572_2169	55	17
SCE1572_10410	160	95
SCE1572_654	0	0
SCE1572_11296	88	37
SCE1572_9302	36	24
SCE1572_8085	0	0
SCE1572_6190	146	23
SCE1572_11209	54	24
SCE1572_5737	211	106
SCE1572_11285	68	659
SCE1572_3339	518	3247

SCE1572_4082	0	0
SCE1572_4785	147	71
SCE1572_1210	2	0
SCE1572_8377	0	0
SCE1572_7706	41	18
SCE1572_5872	501	201
SCE1572_3520	6	0
SCE1572_4410	0	0
SCE1572_9490	142	88
SCE1572_10860	392	272
SCE1572_340	54	7
SCE1572_6879	360	278
SCE1572_11213	8	3
SCE1572_4258	96	150
SCE1572_4845	69	36
SCE1572_8954	85	45
SCE1572_4687	31	8
SCE1572_6528	36	9
SCE1572_10392	20	8
SCE1572_1745	104	18
SCE1572_4099	40	24
SCE1572_197	76	137
SCE1572_1627	930	344
SCE1572_2875	49	29
SCE1572_9368	16	1
SCE1572_9294	89	326
SCE1572_1923	62	19
SCE1572_380	27	2
SCE1572_7520	19	10
SCE1572_11231	161.01	34
SCE1572_3337	643	4123
SCE1572_8876	556	72
SCE1572_7182	111	14
SCE1572_7246	80	30
SCE1572_885	314	34
SCE1572_5133	34	17
SCE1572_8059	0	0
SCE1572_6870	162	212
SCE1572_3163	433	97
SCE1572_3876	17	4
SCE1572_639	157	64
SCE1572_9447	0	0
SCE1572_5748	73	38
SCE1572_9472	226	74
SCE1572_7104	175	35
SCE1572_5464	100	18
SCE1572_8622	105	40
SCE1572_8517	65	13

SCE1572_11504	15	2
SCE1572_10604	83	30
SCE1572_4468	20	9
SCE1572_8003	1111.32	178.16
SCE1572_1572	275	70
SCE1572_949	369.5	155.96
SCE1572_8434	91	31
SCE1572_6546	386	106
SCE1572_7498	58	8
SCE1572_7218	87	57
SCE1572_7235	146	85
SCE1572_8407	12	11
SCE1572_10121	130	117
SCE1572_3919	51	7
SCE1572_510	102	42
SCE1572_5307	60	27
SCE1572_1103	365	132
SCE1572_9013	1	0
SCE1572_10273	153	141
SCE1572_3972	72	55
SCE1572_4635	46	9
SCE1572_2651	149	21
SCE1572_1936	325	116
SCE1572_250	24	12
SCE1572_10209	0	0
SCE1572_10514	60	31
SCE1572_5679	438	488
SCE1572_4755	78	28
SCE1572_2621	164	45
SCE1572_8106	212	16
SCE1572_4793	14	14
SCE1572_3184	3	0
SCE1572_1685	41	3
SCE1572_2192	0	0
SCE1572_8466	128	121
SCE1572_2513	85	114
SCE1572_1976	241	746
SCE1572_4241	50	15
SCE1572_1767	145	46
SCE1572_5406	8	3
SCE1572_3276	43	61
SCE1572_6605	58	261
SCE1572_8948	13	5
SCE1572_1669	14	43
SCE1572_1759	13	2
SCE1572_5687	0	0
SCE1572_3018	113	17
SCE1572_3512	40	16

SCE1572_5204	177	173
SCE1572_9161	23	20
SCE1572_4968	68	8
SCE1572_10035	11	2
SCE1572_296	37	36
SCE1572_4054	82	104
SCE1572_3454	75.75	23
SCE1572_1061	112	140
SCE1572_5885	74	15
SCE1572_6456	25	4
SCE1572_833	146	108
SCE1572_4078	58	13
SCE1572_7601	96	38
SCE1572_3269	51	118
SCE1572_2599	182	44
SCE1572_8901	44	25
SCE1572_817	0	0
SCE1572_7083	34	72
SCE1572_2199	165	39
SCE1572_9857	222	195
SCE1572_3701	42	11
SCE1572_6670	36	1
SCE1572_6658	353	116
SCE1572_10168	27	5
SCE1572_5804	51	35
SCE1572_7717	38	3
SCE1572_38	247	353
SCE1572_8169	98	10
SCE1572_3365	36	5
SCE1572_1367	92	81
SCE1572_279	0	0
SCE1572_4061	41	42
SCE1572_7510	2	0
SCE1572_4397	66	16
SCE1572_1348	477	898
SCE1572_4852	21	13
SCE1572_5272	114	19
SCE1572_11252	0	0
SCE1572_6027	18	7
SCE1572_1680	160	470
SCE1572_8123	4	0
SCE1572_1895	39	7
SCE1572_9617	62	17
SCE1572_7691	34	38
SCE1572_6076	0	0
SCE1572_2145	107	183
SCE1572_3285	90	43
SCE1572_4947	38	33

SCE1572_8660	10	4
SCE1572_10870	79	6
SCE1572_2773	879	167
SCE1572_4342	122	32
SCE1572_1653	44	0
SCE1572_5418	37	17
SCE1572_9043	31	18
SCE1572_5658	339	62
SCE1572_7704	13	6
SCE1572_1673	176	273
SCE1572_4592	70	69
SCE1572_8970	82	514
SCE1572_8861	1305	188
SCE1572_1429	43	39
SCE1572_5147	62	1
SCE1572_4009	164	32
SCE1572_1476	101	13
SCE1572_3800	0	0
SCE1572_6561	37	3
SCE1572_10320	16	1
SCE1572_6480	158	75
SCE1572_1860	140	89
SCE1572_3911	168	772
SCE1572_704	158	268
SCE1572_6224	87	135
SCE1572_9405	73	12
SCE1572_10067	980	1074
SCE1572_5102	136	222
SCE1572_644	173	137
SCE1572_9563	91	17
SCE1572_3441	62	75
SCE1572_5585	104	8
SCE1572_4407	12	2
SCE1572_5353	126	97
SCE1572_9099	860	4723
SCE1572_10918	19	42
SCE1572_873	12	0
SCE1572_3330	36	207
SCE1572_10877	176	38
SCE1572_834	58	67
SCE1572_1424	51	90
SCE1572_2366	226	64
SCE1572_4162	120	35
SCE1572_7374	11	5
SCE1572_7347	20	3
SCE1572_8566	63	82
SCE1572_200	49	18
SCE1572_10095	24	181

SCE1572_9680	692	900
SCE1572_5376	129	20
SCE1572_7098	110	33
SCE1572_10260	36	8
SCE1572_6966	67	21
SCE1572_3201	17	0
SCE1572_4589	25	6
SCE1572_7758	24	1
SCE1572_5944	134	27
SCE1572_11326	49	6
SCE1572_5031	563	1950
SCE1572_2228	231	127
SCE1572_8696	0	0
SCE1572_7015	17	7
SCE1572_6009	76	85
SCE1572_9577	3	1
SCE1572_5917	143	199
SCE1572_7441	142	9
SCE1572_8402	131	28
SCE1572_10156	43	44
SCE1572_2266	105	46
SCE1572_1392	36	4
SCE1572_2203	78	214
SCE1572_5593	91	15
SCE1572_11493	81	13
SCE1572_7386	126	22
SCE1572_4294	273	70
SCE1572_9195	75	18
SCE1572_4766	168	86
SCE1572_2518	247	97
SCE1572_3697	23	8
SCE1572_2050	93	25
SCE1572_1549	99	78
SCE1572_9662	228	532
SCE1572_10246	0	0
SCE1572_974	0	0
SCE1572_2234	95	23
SCE1572_7973	13	0
SCE1572_10465	111	22
SCE1572_9903	120	58
SCE1572_5219	3	1
SCE1572_6062	205	224
SCE1572_7305	155	25
SCE1572_6945	5	0
SCE1572_3931	767	1034
SCE1572_6985	58	2
SCE1572_4238	0	0
SCE1572_1568	230	70

SCE1572_1246	42	3
SCE1572_1839	5	1
SCE1572_9277	66	17
SCE1572_4206	259	296
SCE1572_3665	0	0
SCE1572_11161	147	36
SCE1572_436	97	32
SCE1572_5171	108	233
SCE1572_6461	110	36
SCE1572_566	181	55
SCE1572_8932	81	49
SCE1572_2698	58	7
SCE1572_1857	0	0
SCE1572_7679	108	167
SCE1572_5236	216	147
SCE1572_9535	0	0
SCE1572_4657	38	75
SCE1572_75	18	4
SCE1572_10868	164	17
SCE1572_2028	123	202
SCE1572_3815	1190	4171
SCE1572_9242	5	1
SCE1572_1158	33	5
SCE1572_2820	212	228
SCE1572_3461	62	8
SCE1572_1879	33	16
SCE1572_3813	0	0
SCE1572_3478	76	108
SCE1572_6799	153	18
SCE1572_3391	16	10
SCE1572_2767	30	18
SCE1572_7451	40	37
SCE1572_10217	50	135
SCE1572_6266	16	10
SCE1572_29	68	46
SCE1572_9708	131	120
SCE1572_2375	7	6
SCE1572_5935	142	19
SCE1572_11374	210	1087
SCE1572_1528	293	120
SCE1572_10869	179	18
SCE1572_4221	138	445
SCE1572_9361	0	0
SCE1572_10240	0	0
SCE1572_2658	687	217
SCE1572_4290	15.61	13
SCE1572_8488	358	70
SCE1572_6013	74	59

SCE1572_9376	2	0
SCE1572_2248	288.03	42
SCE1572_5910	102	110
SCE1572_2800	292	45
SCE1572_9713	27	203
SCE1572_244	54.71	10
SCE1572_11033	37	7
SCE1572_7763	0	0
SCE1572_11545	103	22
SCE1572_5094	165	348
SCE1572_4	167	268
SCE1572_3677	27	7
SCE1572_10789	82	86
SCE1572_5002	272	802
SCE1572_5338	18	2
SCE1572_8420	129	20
SCE1572_1332	28	3
SCE1572_7540	175	181
SCE1572_4695	11	4
SCE1572_3139	17	399
SCE1572_4288	33.87	27
SCE1572_6701	251	33
SCE1572_8411	52	38
SCE1572_2642	99	24
SCE1572_355	97	14
SCE1572_827	37	6
SCE1572_1665	226	177
SCE1572_1567	5	0
SCE1572_3737	56	6
SCE1572_7464	412	162
SCE1572_5470	301	22
SCE1572_11478	297	167
SCE1572_3248	7	1
SCE1572_4938	2	0
SCE1572_11188	1	0
SCE1572_5975	46	24
SCE1572_5472	193	33
SCE1572_9938	2	0
SCE1572_4480	42	14
SCE1572_2450	38	1
SCE1572_5787	27	67
SCE1572_11113	59	4
SCE1572_5043	28	50
SCE1572_5553	67	12
SCE1572_2614	47	22
SCE1572_9018	117	33
SCE1572_286	18	0
SCE1572_7649	158	31

SCE1572_5411	19	4
SCE1572_7484	54	23
SCE1572_77	49	9
SCE1572_3938	339	151
SCE1572_3904	0	0
SCE1572_579	22	96
SCE1572_10435	59	40
SCE1572_1036	67	8
SCE1572_7825	30	9
SCE1572_1099	83	143
SCE1572_4237	67	26
SCE1572_7366	285	1832
SCE1572_6590	6	5
SCE1572_2430	125	85
SCE1572_6633	1294	152
SCE1572_7587	325	1562
SCE1572_5899	42	9
SCE1572_6205	268	29
SCE1572_4375	147	79
SCE1572_1607	59	7
SCE1572_3649	20	5
SCE1572_3742	119	38
SCE1572_6313	102	17
SCE1572_11304	134	137
SCE1572_8776	70	5
SCE1572_10592	43	3
SCE1572_3404	30	17
SCE1572_4384	65	11
SCE1572_9933	197	60
SCE1572_9333	40	184
SCE1572_2934	167	241
SCE1572_4545	116	8
SCE1572_8596	12	1
SCE1572_11211	165	28
SCE1572_5515	458	79
SCE1572_6464	59	8
SCE1572_7902	38	4
SCE1572_3208	106	285
SCE1572_11277	43	42
SCE1572_9652	107	36
SCE1572_5294	92	14
SCE1572_7505	114	28
SCE1572_3528	54	3028
SCE1572_9676	5	3
SCE1572_1980	341	908
SCE1572_11369	38	2
SCE1572_4012	186	59
SCE1572_9876	8	1

SCE1572_11225	68	19
SCE1572_8295	106	113
SCE1572_8767	19	2
SCE1572_4540	278.1	53
SCE1572_4209	148	195
SCE1572_5210	6	1
SCE1572_1644	43	17
SCE1572_2536	140	57
SCE1572_5640	566	87
SCE1572_6431	278	61
SCE1572_5829	58	4
SCE1572_3444	588	84
SCE1572_10583	7	0
SCE1572_500	20	0
SCE1572_5602	51	72
SCE1572_9796	230	46
SCE1572_5714	28	19
SCE1572_6184	54	7
SCE1572_6357	41	9
SCE1572_10271	116	22
SCE1572_1713	35	22
SCE1572_4934	505	405
SCE1572_3171	127	245
SCE1572_8683	55	48
SCE1572_8262	103	32
SCE1572_10998	41	53
SCE1572_1878	19	10
SCE1572_4432	144	14
SCE1572_844	278	397
SCE1572_4621	97	193
SCE1572_8363	175.04	24
SCE1572_11511	113	44
SCE1572_8042	56	8
SCE1572_7547	114	23
SCE1572_1092	0	0
SCE1572_10941	95	9
SCE1572_8315	75	24
SCE1572_10547	40	10
SCE1572_4603	325	705
SCE1572_11320	41	2
SCE1572_2525	60	16
SCE1572_10571	481	448
SCE1572_11013	97	46
SCE1572_1361	154	245
SCE1572_5355	36	18
SCE1572_291	0	0
SCE1572_8049	140	6
SCE1572_3227	56	4

SCE1572_8072	56	10
SCE1572_768	170	84
SCE1572_2315	60	124
SCE1572_4049	74	229
SCE1572_4281	36	9
SCE1572_952	7	8
SCE1572_9603	25	2
SCE1572_10831	132	36
SCE1572_2906	312	30
SCE1572_28	40	30
SCE1572_5440	125	34
SCE1572_4431	17	3
SCE1572_9827	62	25
SCE1572_9217	119	94
SCE1572_634	203	293
SCE1572_4944	26	25
SCE1572_6167	53	7
SCE1572_11168	117	35
SCE1572_2814	116	269
SCE1572_8310	103	19
SCE1572_3176	107	57
SCE1572_4231	323	65
SCE1572_6527	0	0
SCE1572_9945	2878	2192
SCE1572_225	0	0
SCE1572_11382	95	249
SCE1572_367	55	50
SCE1572_488	75.57	24.38
SCE1572_770	67	59
SCE1572_7848	117	56
SCE1572_4169	195	227
SCE1572_10951	144	68
SCE1572_2439	25	20
SCE1572_2333	154	147
SCE1572_4141	43	10
SCE1572_572	14	6
SCE1572_3660	37	20
SCE1572_6574	169	35
SCE1572_10828	39	10
SCE1572_515	153	17
SCE1572_4768	61	14
SCE1572_8079	10	0
SCE1572_5243	5	0
SCE1572_7954	106	4
SCE1572_7832	48	126
SCE1572_7312	37	10
SCE1572_9143	532.64	156.41
SCE1572_7284	39	1

SCE1572_7150	54	17
SCE1572_6399	147	546
SCE1572_3211	7	0
SCE1572_10493	400	860
SCE1572_4156	161	77
SCE1572_6489	32	16
SCE1572_10228	21	0
SCE1572_6280	23	194
SCE1572_1260	222	36
SCE1572_5432	209	22
SCE1572_5824	24	12
SCE1572_8779	24	53
SCE1572_7146	63	16
SCE1572_11400	157	224
SCE1572_6993	70	12
SCE1572_10958	70	22
SCE1572_8607	271	406
SCE1572_3143	61	14
SCE1572_5252	99	395
SCE1572_58	125	48
SCE1572_1861	71	26
SCE1572_10475	124	81
SCE1572_11424	38	93
SCE1572_2881	82	25
SCE1572_2787	85	167
SCE1572_602	294	151
SCE1572_2955	1	0
SCE1572_2839	200	53
SCE1572_585	96	52
SCE1572_11199	13	0
SCE1572_2792	84	14
SCE1572_3776	186	78
SCE1572_9198	56	14
SCE1572_1987	9	2
SCE1572_8450	101	14
SCE1572_5012	26	49
SCE1572_10687	6	4
SCE1572_8304	58	17
SCE1572_1701	308	213
SCE1572_3769	71	252
SCE1572_8505	45	17
SCE1572_1494	120	27
SCE1572_1881	45	4
SCE1572_4958	46	3
SCE1572_6216	130	26
SCE1572_8710	24	9
SCE1572_10894	20	16
SCE1572_6547	457	223

SCE1572_2506	104	18
SCE1572_2016	199	38
SCE1572_5434	214	46
SCE1572_6465	10	3
SCE1572_10608	58	111
SCE1572_4665	20	2
SCE1572_8513	24.68	3.05
SCE1572_2305	47	82
SCE1572_4548	21	0
SCE1572_2883	65	182
SCE1572_10409	86	86
SCE1572_5624	0	0
SCE1572_2470	22	1
SCE1572_6909	38	0
SCE1572_4872	37	10
SCE1572_369	13	1
SCE1572_730	62	25
SCE1572_1276	98	20
SCE1572_8171	190	28
SCE1572_5875	5	2
SCE1572_1149	480	751
SCE1572_3358	23	4
SCE1572_5823	47	3
SCE1572_2428	130	110
SCE1572_9564	23	7
SCE1572_5077	164	299
SCE1572_1257	95	131
SCE1572_5441	165	40
SCE1572_6756	77	25
SCE1572_5321	81	33
SCE1572_8199	102	20
SCE1572_7648	243	54
SCE1572_7662	60	6
SCE1572_10193	73	10
SCE1572_8309	161	183
SCE1572_32	33	7
SCE1572_1194	150	85
SCE1572_10317	39	11
SCE1572_8724	116	28
SCE1572_1907	78	94
SCE1572_4174	12	5
SCE1572_2441	219	74
SCE1572_6154	70	4
SCE1572_10733	12	49
SCE1572_684	68	13
SCE1572_9039	52	28
SCE1572_10797	107	38
SCE1572_11413	145	31

SCE1572_9508	58	12
SCE1572_9890	224	42
SCE1572_9501	546	663
SCE1572_10790	479	715
SCE1572_4216	1182	925
SCE1572_7406	54	16
SCE1572_530	207	49
SCE1572_10776	525	312.99
SCE1572_2201	169	587
SCE1572_10062	392	491
SCE1572_1453	307.05	59.43
SCE1572_2736	37	30
SCE1572_4804	181	31
SCE1572_6225	63	17
SCE1572_7432	45	24
SCE1572_10491	380	488
SCE1572_11449	254	349
SCE1572_4273	148	41
SCE1572_5581	200	325
SCE1572_2553	157	29
SCE1572_100	194	50
SCE1572_1445	222	70
SCE1572_2251	175	60
SCE1572_7687	41	12
SCE1572_7148	114	210
SCE1572_2131	55	13
SCE1572_5485	917	169
SCE1572_9899	166	370
SCE1572_2104	219	838
SCE1572_8361	30	2
SCE1572_4909	4	0
SCE1572_10636	6	0
SCE1572_5707	31	18
SCE1572_7910	11	1
SCE1572_2916	92	10
SCE1572_7778	31	10
SCE1572_7249	441	61
SCE1572_9321	14	3
SCE1572_7124	495	318
SCE1572_2446	195	52
SCE1572_4023	299	1299
SCE1572_10364	25	37
SCE1572_4898	47	9
SCE1572_3428	24.85	14
SCE1572_3880	117	35
SCE1572_6086	1	0
SCE1572_6531	79	30
SCE1572_9268	87	55

SCE1572_11048	78	49
SCE1572_3729	6	1
SCE1572_9100	105	595
SCE1572_3207	91	439
SCE1572_2154	45	9
SCE1572_6617	39	2
SCE1572_2159	0	0
SCE1572_2568	45	14
SCE1572_614	35	11
SCE1572_8179	0	0
SCE1572_4013	123	129
SCE1572_5812	466	135
SCE1572_10280	102	385
SCE1572_715	0	0
SCE1572_11567	0	0
SCE1572_10696	63	31
SCE1572_665	39	17
SCE1572_9855	234	44
SCE1572_5007	1062	1049
SCE1572_8923	8	2
SCE1572_10476	72	71
SCE1572_3527	59	9549
SCE1572_9064	207	28
SCE1572_4851	410	99
SCE1572_2401	117	180
SCE1572_4721	189	210
SCE1572_2357	42	46
SCE1572_1792	25	24
SCE1572_9582	164	49
SCE1572_216	80	94
SCE1572_8325	180	170
SCE1572_10327	25	2
SCE1572_729	34	47
SCE1572_7325	349	811
SCE1572_1695	0	0
SCE1572_8070	15.01	3
SCE1572_11601	61	105
SCE1572_1074	1	0
SCE1572_4047	83	13
SCE1572_1132	39	4
SCE1572_6716	293	300
SCE1572_5856	14	1
SCE1572_10147	67	81
SCE1572_7563	275	54
SCE1572_4178	13.25	0.75
SCE1572_5563	556	251
SCE1572_4891	1	0
SCE1572_9364	231	92

SCE1572_2176	103	15
SCE1572_10572	39	25
SCE1572_10899	5	0
SCE1572_453	149	21
SCE1572_9131	9	4
SCE1572_6836	17	51
SCE1572_8084	0	0
SCE1572_8371	32	10
SCE1572_9682	222	241
SCE1572_11330	94	179
SCE1572_362	135	32
SCE1572_8077	36.62	3
SCE1572_7562	38	3
SCE1572_3860	52	42
SCE1572_3627	98	12
SCE1572_11429	155	46
SCE1572_5109	75	90
SCE1572_2521	52	7
SCE1572_720	4	1
SCE1572_537	159	152
SCE1572_10482	58	9
SCE1572_5104	87	105
SCE1572_2399	501	1381
SCE1572_8558	53	6
SCE1572_2365	281	258
SCE1572_8993	236	314
SCE1572_2029	137	26
SCE1572_5952	2	0
SCE1572_4471	153	47
SCE1572_6444	30	9
SCE1572_9455	38	47
SCE1572_2345	43	47
SCE1572_5407	0	0
SCE1572_1666	100	31
SCE1572_11402	8	2
SCE1572_4259	1	0
SCE1572_435	137	164
SCE1572_2653	112	28
SCE1572_1693	6	1
SCE1572_3720	177	64
SCE1572_6011	112	97
SCE1572_4148	33	93
SCE1572_4671	0	0
SCE1572_3585	56	8
SCE1572_5961	42	20
SCE1572_3460	106	49
SCE1572_11114	211	307
SCE1572_7919	209	22

SCE1572_11202	124	992
SCE1572_896	0	0
SCE1572_2964	135	1500
SCE1572_2764	5	0
SCE1572_4222	150	40
SCE1572_3576	47	7
SCE1572_37	30	19
SCE1572_1537	33	31
SCE1572_11558	243.31	48.37
SCE1572_6178	55	128
SCE1572_2413	56	108
SCE1572_1116	0	0
SCE1572_10539	200	52
SCE1572_3760	51.08	1
SCE1572_2485	75	80
SCE1572_10459	17	29
SCE1572_9732	283	35
SCE1572_8586	99	12
SCE1572_7610	115	154
SCE1572_9779	0	0
SCE1572_8639	90	128
SCE1572_6026	69	60
SCE1572_6560	0	0
SCE1572_1735	217	55
SCE1572_3968	204	42
SCE1572_9537	56	71
SCE1572_5359	52	231
SCE1572_3019	49	11
SCE1572_1655	0	0
SCE1572_1304	285	236
SCE1572_11255	322	256
SCE1572_5968	7	1
SCE1572_9254	95	16
SCE1572_5151	1	0
SCE1572_5753	0	0
SCE1572_10425	28	14
SCE1572_5894	32	2
SCE1572_7458	75	24
SCE1572_2091	11	0
SCE1572_3820	118	32
SCE1572_6570	52	19
SCE1572_2387	77	21
SCE1572_7595	156	351
SCE1572_2968	60	55
SCE1572_5483	127	11
SCE1572_9919	64	12
SCE1572_5362	46	35
SCE1572_1977	1182	11889

SCE1572_4327	27	5
SCE1572_16	113	97
SCE1572_10289	178	83
SCE1572_3564	251.03	36.16
SCE1572_11366	43	7
SCE1572_9382	0	0
SCE1572_4422	14	3
SCE1572_4204	14	103
SCE1572_3538	30	6
SCE1572_3470	93	16
SCE1572_11519	108	22
SCE1572_631	322	225
SCE1572_10172	87	39
SCE1572_11527	108	25
SCE1572_11537	154	218
SCE1572_8757	31	10
SCE1572_5631	30	8
SCE1572_7048	0	0
SCE1572_4523	14	0
SCE1572_66	123	84
SCE1572_2703	193	45
SCE1572_7743	253	504
SCE1572_5819	81	16
SCE1572_6375	214	17
SCE1572_3893	19	3
SCE1572_3210	17	19
SCE1572_3138	75	409
SCE1572_330	113	42
SCE1572_751	87	38
SCE1572_10219	184	67
SCE1572_8481	37	13
SCE1572_356	33	3
SCE1572_11229	7	3
SCE1572_2643	166	33
SCE1572_17	19	25
SCE1572_6848	81	2
SCE1572_3734	149	8
SCE1572_5066	415	317
SCE1572_7398	115	220
SCE1572_3141	402	63
SCE1572_2822	43	16
SCE1572_5919	86	81
SCE1572_2089	36	8
SCE1572_8441	117	101
SCE1572_8661	313	57
SCE1572_673	124	412
SCE1572_9051	72	12
SCE1572_3495	72	18

SCE1572_3981	83	32
SCE1572_2495	166	31
SCE1572_10667	36	8
SCE1572_6666	209	412
SCE1572_9541	41	7
SCE1572_1888	30	142
SCE1572_3287	24	0
SCE1572_10134	17	3
SCE1572_5162	0	0
SCE1572_779	181	82
SCE1572_2293	80	43
SCE1572_4525	52	10
SCE1572_5768	520	1511
SCE1572_2007	36	21
SCE1572_9808	1155	1413
SCE1572_5244	61	53
SCE1572_1387	293	70
SCE1572_5656	75	9
SCE1572_5038	194	798
SCE1572_10939	364	198
SCE1572_9417	539	3419
SCE1572_6053	192	403
SCE1572_3807	402	251
SCE1572_2759	118	257
SCE1572_2980	176	1064
SCE1572_3682	50	1
SCE1572_7089	73	27
SCE1572_4662	128	14
SCE1572_10402	474	410
SCE1572_8138	77	14
SCE1572_4736	175	590
SCE1572_5610	45	1
SCE1572_7507	82.36	11.5
SCE1572_9121	51	6
SCE1572_2340	0	0
SCE1572_11093	164	40
SCE1572_5217	254	35
SCE1572_170	27	4
SCE1572_10739	27	5
SCE1572_7945	10	0
SCE1572_11097	85	32
SCE1572_9148	157	346
SCE1572_231	1	0
SCE1572_8875	10.49	0
SCE1572_8333	155	107
SCE1572_4558	98	105
SCE1572_176	21	2
SCE1572_765	15	36

SCE1572_2670	167	18
SCE1572_10384	19	1
SCE1572_1938	11	6
SCE1572_6061	1085	1248
SCE1572_10704	44	76
SCE1572_8298	684	178
SCE1572_9182	47	10
SCE1572_719	87.98	41.39
SCE1572_3112	3	0
SCE1572_2876	86	49
SCE1572_4306	24	6
SCE1572_10103	32	29
SCE1572_3097	87	12
SCE1572_1868	44	89
SCE1572_9980	25	26
SCE1572_1516	342	402
SCE1572_11070	65	65
SCE1572_5848	111	29
SCE1572_9875	48	42
SCE1572_1648	67	9
SCE1572_10648	78	81
SCE1572_3944	79	85
SCE1572_2989	344	87
SCE1572_4338	71	10
SCE1572_6773	283	108
SCE1572_9107	18	23
SCE1572_904	57	24
SCE1572_9832	21	2
SCE1572_9412	96	140
SCE1572_8096	7	2
SCE1572_7757	32	8
SCE1572_2409	78	258
SCE1572_3952	37	10
SCE1572_9548	20	0
SCE1572_7755	51	27
SCE1572_7468	63	29
SCE1572_5758	17	11
SCE1572_3228	70	59
SCE1572_7118	230	48
SCE1572_2138	10	0
SCE1572_9739	0	0
SCE1572_3118	0	0
SCE1572_2942	96	38
SCE1572_4263	143	31
SCE1572_7288	163	124
SCE1572_7631	59	262
SCE1572_4066	144	160
SCE1572_6068	49	2

SCE1572_8889	72	24
SCE1572_7022	306.06	39.48
SCE1572_290	0	0
SCE1572_5195	137	44
SCE1572_6763	265	161
SCE1572_6647	32	2
SCE1572_1633	88	87
SCE1572_8167	0	0
SCE1572_1826	69	9
SCE1572_7986	102	25
SCE1572_1775	16	0
SCE1572_2903	0	0
SCE1572_5797	54	6
SCE1572_9637	122	709
SCE1572_8057	0	0
SCE1572_7102	180	88
SCE1572_4330	144	33
SCE1572_7633	724	488
SCE1572_6112	110	31
SCE1572_2126	127	20
SCE1572_9690	138	124
SCE1572_808	110	17
SCE1572_7517	225	179
SCE1572_3111	47	7
SCE1572_1127	32	6
SCE1572_2716	145	81
SCE1572_4858	61	15
SCE1572_9073	233	66
SCE1572_9623	225	48
SCE1572_2673	119	260
SCE1572_1511	121	375
SCE1572_3416	33	11
SCE1572_9974	69	23
SCE1572_9957	11	1
SCE1572_7728	0	0
SCE1572_1207	83	22
SCE1572_2249	78.54	24
SCE1572_6632	121	203
SCE1572_2528	2	1
SCE1572_9765	64	32
SCE1572_9042	53	95
SCE1572_9091	22	3
SCE1572_8160	30	4
SCE1572_9750	75	45
SCE1572_11001	20	2
SCE1572_4444	214	227
SCE1572_10104	32	36
SCE1572_2992	48	18

SCE1572_1617	33	13
SCE1572_9755	26	5
SCE1572_9524	53	25
SCE1572_11464	846	119
SCE1572_3879	52	60
SCE1572_984	95	99
SCE1572_6119	263	80
SCE1572_9598	329	2467
SCE1572_1235	180	23
SCE1572_3324	207	331
SCE1572_4753	102	122
SCE1572_1287	48	269
SCE1572_7531	55	116
SCE1572_9811	0	0
SCE1572_776	4	0
SCE1572_11588	55	23
SCE1572_7042	45	8
SCE1572_7371	31	5
SCE1572_3353	0	0
SCE1572_11476	8	13
SCE1572_7684	0	0
SCE1572_9445	89	24
SCE1572_11354	19	5
SCE1572_3620	17	2
SCE1572_4123	43	6
SCE1572_2798	60	6
SCE1572_8731	27	4
SCE1572_9814	68	150
SCE1572_6403	122	38
SCE1572_866	36	9
SCE1572_3005	10	0
SCE1572_4052	108	306
SCE1572_10295	72	141
SCE1572_4390	138	29
SCE1572_10568	454	468
SCE1572_4196	126	68
SCE1572_696	4	0
SCE1572_1240	383	47
SCE1572_3606	17	1
SCE1572_7029	214.47	57
SCE1572_1603	0	0
SCE1572_8336	307.05	59.43
SCE1572_6342	80	54
SCE1572_8880	92	12
SCE1572_10839	2	0
SCE1572_5676	29	5
SCE1572_1292	84	17
SCE1572_4833	199	883

SCE1572_560	488	3379
SCE1572_5050	8	0
SCE1572_5619	33	21
SCE1572_2779	285	417
SCE1572_5375	62	15
SCE1572_4868	102	28
SCE1572_2418	62	86
SCE1572_8025	0	0
SCE1572_1534	65	14
SCE1572_4313	0	0
SCE1572_1010	160	30
SCE1572_8961	17	4
SCE1572_7238	235	409
SCE1572_5057	265	470
SCE1572_2186	1	0
SCE1572_1788	3	1
SCE1572_8356	21	3
SCE1572_3081	24	48
SCE1572_6181	75	36
SCE1572_2847	67	30
SCE1572_2946	68	14
SCE1572_3200	177	355
SCE1572_2435	78	61
SCE1572_3996	4	1
SCE1572_6607	0	0
SCE1572_5932	232	99
SCE1572_204	108	27
SCE1572_5616	0	0
SCE1572_800	0	0
SCE1572_3469	302	47
SCE1572_320	75	10
SCE1572_3049	34	4
SCE1572_1700	198	132
SCE1572_1268	226	88
SCE1572_8794	53	10
SCE1572_109	75	35
SCE1572_5641	4	0
SCE1572_301	145	58
SCE1572_1640	66	16
SCE1572_4316	164	18
SCE1572_7962	224	916
SCE1572_2837	258	94
SCE1572_11316	1044	868
SCE1572_6217	4	3
SCE1572_4576	3	0
SCE1572_8231	142	43
SCE1572_10198	97	16
SCE1572_5695	68	8

SCE1572_7425	11	76
SCE1572_10922	789	35268
SCE1572_2715	235	224
SCE1572_2110	77	334
SCE1572_922	11	7
SCE1572_9483	238	256
SCE1572_10561	0	0
SCE1572_2741	14	3
SCE1572_11131	40	7
SCE1572_11200	110	165
SCE1572_7858	219	103
SCE1572_6554	8	0
SCE1572_9202	52	27
SCE1572_7265	58	20
SCE1572_6975	57	9
SCE1572_2840	142	43
SCE1572_6122	228	269
SCE1572_6045	657	1108
SCE1572_2680	34	4
SCE1572_3792	2	0
SCE1572_6580	30	2
SCE1572_326	79	35
SCE1572_8570	2	0
SCE1572_5126	32	23
SCE1572_3898	83	106
SCE1572_5950	70	7
SCE1572_5986	7	0
SCE1572_3180	103	94
SCE1572_4539	59.35	23.42
SCE1572_978	68	41
SCE1572_7934	31	1
SCE1572_8050	87	7
SCE1572_10887	81	50
SCE1572_11237	56	7
SCE1572_9395	26	10
SCE1572_3889	23	5
SCE1572_1553	39	10
SCE1572_3825	15	3
SCE1572_3713	255	187
SCE1572_8773	184	83
SCE1572_8048	103	14
SCE1572_1054	208	107
SCE1572_10359	52	22
SCE1572_3788	90	69
SCE1572_9986	40	12
SCE1572_3311	28	5
SCE1572_9663	68	188
SCE1572_3781	150	546

SCE1572_7304	129	14
SCE1572_3678	19	19
SCE1572_3056	36	5
SCE1572_2220	70	82
SCE1572_9784	64	4
SCE1572_10521	121	51
SCE1572_9355	89	113
SCE1572_10220	129	65
SCE1572_3594	25	2
SCE1572_1437	35	6
SCE1572_10253	0	0
SCE1572_8630	79	188
SCE1572_1997	8	1
SCE1572_5261	82	390
SCE1572_155	239	195
SCE1572_5242	31	3
SCE1572_5762	23	4
SCE1572_7393	52	2
SCE1572_2036	455	2106
SCE1572_10370	74	11
SCE1572_11088	43	26
SCE1572_5116	61	28
SCE1572_6130	19	52
SCE1572_9021	16	7
SCE1572_2042	126	206
SCE1572_4303	82	6
SCE1572_11249	32	59
SCE1572_3273	90	195
SCE1572_3063	151.18	122
SCE1572_6938	65	8
SCE1572_11267	428	1082
SCE1572_1953	52	30
SCE1572_1329	34	46
SCE1572_6538	75	18
SCE1572_5927	350	42
SCE1572_6152	141	53
SCE1572_4996	12	6
SCE1572_9677	64	13
SCE1572_4614	140	24
SCE1572_11339	32	9
SCE1572_3394	113	83
SCE1572_4393	47	11
SCE1572_2239	204	74
SCE1572_10182	140	165
SCE1572_9643	297	164
SCE1572_1599	0	0
SCE1572_4910	2	0
SCE1572_8745	3	0

SCE1572_508	106	9
SCE1572_6279	56	964
SCE1572_1726	97	24
SCE1572_11384	725	612
SCE1572_9910	60	16
SCE1572_1954	27	13
SCE1572_9298	134	114
SCE1572_3930	388	669
SCE1572_6035	141	93
SCE1572_3726	74	716
SCE1572_3865	20	3
SCE1572_5781	253	366
SCE1572_8522	355	285
SCE1572_299	108	31
SCE1572_9760	141	128
SCE1572_10028	28	2
SCE1572_11375	0	0
SCE1572_7991	101	166
SCE1572_7335	37	49
SCE1572_8971	188	218
SCE1572_4479	31	23
SCE1572_4887	40	6
SCE1572_8579	0	0
SCE1572_6746	31	21
SCE1572_4639	31	2
SCE1572_5608	171	29
SCE1572_9023	2458	2346
SCE1572_2229	39	328
SCE1572_11481	71	14
SCE1572_4245	57	83
SCE1572_1475	0	0
SCE1572_1925	0	0
SCE1572_1500	51	19
SCE1572_4597	0	0
SCE1572_3556	5	0
SCE1572_4880	320	347
SCE1572_3571	76	29
SCE1572_6843	42	16
SCE1572_1781	22	10
SCE1572_8287	90	116
SCE1572_593	186	139
SCE1572_399	17	4
SCE1572_6254	162	81
SCE1572_318	18	1
SCE1572_11596	58	262
SCE1572_9725	8	11
SCE1572_2274	45	4
SCE1572_1520	60	9

SCE1572_4355	29	12
SCE1572_3167	33	20
SCE1572_3872	159	164
SCE1572_6333	45	67
SCE1572_4366	100	37
SCE1572_6243	79	9
SCE1572_2064	43	16
SCE1572_8264	90	121
SCE1572_5424	82	35
SCE1572_678	85	51
SCE1572_338	123	33
SCE1572_9602	97	13
SCE1572_9187	52	38
SCE1572_7178	344	93
SCE1572_2320	105	589
SCE1572_1969	48	13
SCE1572_9098	38	5
SCE1572_7006	0	0
SCE1572_5501	111	36
SCE1572_1317	125	96
SCE1572_5061	42	80
SCE1572_7016	0	0
SCE1572_1610	262	84
SCE1572_4879	415	304
SCE1572_2661	72	40
SCE1572_6305	40	9
SCE1572_5669	51	7
SCE1572_8989	356	365
SCE1572_5992	93	62
SCE1572_6142	83	25
SCE1572_3298	18	6
SCE1572_5549	281	48
SCE1572_3189	6	0
SCE1572_10965	149	33
SCE1572_11106	85	45
SCE1572_6132	0	0
SCE1572_8531	65	34
SCE1572_143	6	6
SCE1572_349	52	75
SCE1572_5839	167	26
SCE1572_9685	131	117
SCE1572_3508	26	7
SCE1572_238	15	1
SCE1572_1065	143	15
SCE1572_5296	27	5
SCE1572_10153	35	69
SCE1572_8978	75	29
SCE1572_9593	123	920

SCE1572_8866	149	36
SCE1572_2728	48	16
SCE1572_2284	11	4
SCE1572_2149	72	49
SCE1572_1845	4	1
SCE1572_6476	226	1068
SCE1572_5329	217	26
SCE1572_7242	124	67
SCE1572_10930	121	40
SCE1572_6251	304	56
SCE1572_5940	75	13
SCE1572_4516	38	12
SCE1572_6160	22	10
SCE1572_7574	152	349
SCE1572_11391	107	30
SCE1572_7466	161	378
SCE1572_6236	91	14
SCE1572_8695	46	28
SCE1572_4494	57	17
SCE1572_266	57	8
SCE1572_9393	36	9
SCE1572_7545	171	54
SCE1572_3173	83	20
SCE1572_8687	67	15
SCE1572_1101	46	7
SCE1572_11137	58	169
SCE1572_8598	0	0
SCE1572_11017	67	75
SCE1572_9996	101	32
SCE1572_2695	25	57
SCE1572_4769	12	22
SCE1572_8897	394	321
SCE1572_1747	215	74
SCE1572_7829	49	81
SCE1572_8313	48	14
SCE1572_7360	121	92
SCE1572_7482	0	0
SCE1572_8101	276	1039
SCE1572_2319	69	191
SCE1572_10031	242	92
SCE1572_1003	145	27
SCE1572_10125	1	0
SCE1572_2422	13	3
SCE1572_5785	51	59
SCE1572_1601	11	0
SCE1572_3718	0	0
SCE1572_623	1328	4776
SCE1572_352	33	19

SCE1572_8217	270	36
SCE1572_5546	131	18
SCE1572_1031	59	134
SCE1572_2394	74	30
SCE1572_8148	112	24
SCE1572_5001	275	580
SCE1572_3783	12	3
SCE1572_5413	2	0
SCE1572_4787	410	1217
SCE1572_6991	108	11
SCE1572_10347	142	170
SCE1572_9573	82	5
SCE1572_10997	210	381
SCE1572_7381	24	7
SCE1572_285	109	12
SCE1572_2339	45	109
SCE1572_7412	71	104
SCE1572_1175	36	84
SCE1572_4658	72	61
SCE1572_6483	41	6
SCE1572_245	26.85	3
SCE1572_3205	189	215
SCE1572_11513	158	134
SCE1572_7430	197	30
SCE1572_8591	187	21
SCE1572_5517	146	26
SCE1572_4946	1	0
SCE1572_9219	101	8
SCE1572_6006	17	26
SCE1572_2173	7	2
SCE1572_226	40	8
SCE1572_467	828	730
SCE1572_342	199	498
SCE1572_1608	79	22
SCE1572_2464	193	22
SCE1572_10585	107	17
SCE1572_9824	36.61	5.2
SCE1572_9440	68	36
SCE1572_6875	142	24
SCE1572_7977	0	0
SCE1572_10775	246	49
SCE1572_5478	57	20
SCE1572_6858	105	25
SCE1572_1307	721	4537
SCE1572_3406	111	20
SCE1572_2538	42	27
SCE1572_3466	39	22
SCE1572_9701	15	0

SCE1572_3213	156	105
SCE1572_4623	53	135
SCE1572_5422	6	0
SCE1572_2515	10	6
SCE1572_3650	31	8
SCE1572_3690	2	0
SCE1572_85	147	133
SCE1572_6356	76	17
SCE1572_836	76	33
SCE1572_4824	142	117
SCE1572_2313	55	38
SCE1572_7899	128	82
SCE1572_11494	59	8
SCE1572_9954	317	81
SCE1572_9671	67	230
SCE1572_8104	109	33
SCE1572_2590	161	76
SCE1572_9267	8	0
SCE1572_574	69	107
SCE1572_872	115	44
SCE1572_1021	230	28
SCE1572_383	127	59
SCE1572_2890	141	22
SCE1572_4624	114	159
SCE1572_1574	80	14
SCE1572_11431	112	29
SCE1572_7004	29	3
SCE1572_10982	61	22
SCE1572_842	8	2
SCE1572_7659	99	26
SCE1572_918	356	167
SCE1572_7490	153	66
SCE1572_3152	366	132
SCE1572_6521	13	0
SCE1572_8248	32	16
SCE1572_9512	393	646
SCE1572_7708	33	5
SCE1572_4646	54	15
SCE1572_9502	68	57
SCE1572_2354	86	467
SCE1572_3617	218	26
SCE1572_1478	60	10
SCE1572_2097	15	189
SCE1572_4357	140	25
SCE1572_7589	88	309
SCE1572_4489	112	68
SCE1572_10166	0	0
SCE1572_3072	29	7

SCE1572_2211	148	101
SCE1572_10312	416	123
SCE1572_6915	87	14
SCE1572_7627	73	28
SCE1572_3870	149	48
SCE1572_8542	102	275
SCE1572_3488	37	11
SCE1572_7889	75	1621
SCE1572_10971	1	2
SCE1572_9270	0	0
SCE1572_2312	408	2786
SCE1572_7524	12	19
SCE1572_2187	80	18
SCE1572_10331	235	45
SCE1572_5779	101	33
SCE1572_6696	41	6
SCE1572_3696	84	68
SCE1572_9905	0	0
SCE1572_56	56	2
SCE1572_7283	63	14
SCE1572_5638	33	11
SCE1572_11305	3	0
SCE1572_10540	753	109
SCE1572_3378	134	22
SCE1572_3498	145	102
SCE1572_10782	73	11
SCE1572_2853	131	30
SCE1572_1621	118	383
SCE1572_8001	699.92	102.52
SCE1572_9015	0	0
SCE1572_3790	57	8
SCE1572_9939	47	6
SCE1572_7081	91	157
SCE1572_6933	8	12
SCE1572_1675	60	58
SCE1572_10338	269.47	558
SCE1572_7988	54	4
SCE1572_3349	48	14
SCE1572_7622	237	211
SCE1572_5172	56	41
SCE1572_6964	130	52
SCE1572_10417	1	0
SCE1572_3417	85	205
SCE1572_7071	25	11
SCE1572_11328	70	48
SCE1572_5976	82	21
SCE1572_5826	63	20
SCE1572_5571	186.16	35.44

SCE1572_9763	38	23
SCE1572_2541	0	0
SCE1572_4693	281	227
SCE1572_5300	124	21
SCE1572_6504	88	43
SCE1572_5497	158	132
SCE1572_9374	112	17
SCE1572_9287	637	1144
SCE1572_4949	28	7
SCE1572_10018	108	31
SCE1572_4760	76	15
SCE1572_1589	0	0
SCE1572_4434	171.91	47.98
SCE1572_5149	238	50
SCE1572_8012	1	0
SCE1572_8027	12	6
SCE1572_8094	71	9
SCE1572_4409	31	10
SCE1572_6018	2640	712
SCE1572_1910	53	9
SCE1572_1373	109	195
SCE1572_3481	12	2
SCE1572_11364	578	136
SCE1572_5270	20	1671
SCE1572_1244	0	0
SCE1572_879	193	437
SCE1572_3809	43	35
SCE1572_950	3.99	5.85
SCE1572_10243	12	0
SCE1572_8761	32	19
SCE1572_5033	203	1719
SCE1572_5946	75	16
SCE1572_667	31	15
SCE1572_2180	136	24
SCE1572_6751	383	162
SCE1572_3767	42	63
SCE1572_8201	388	51
SCE1572_74	43	34
SCE1572_1522	66	26
SCE1572_6794	31	20
SCE1572_8565	893	753
SCE1572_6983	257	53
SCE1572_4299	625	108
SCE1572_2936	40	6
SCE1572_9927	242	219
SCE1572_7835	49	94
SCE1572_9530	148	61
SCE1572_2052	0	0

SCE1572_2437	9	5
SCE1572_6831	193	130
SCE1572_7181	74	27
SCE1572_2581	30	26
SCE1572_5685	157	325
SCE1572_7291	5	0
SCE1572_9631	79	20
SCE1572_3124	16	2
SCE1572_11050	97	72
SCE1572_10617	79	12
SCE1572_6440	47	7
SCE1572_6419	93	41
SCE1572_2874	119	28
SCE1572_8674	27	47
SCE1572_11543	203	36
SCE1572_124	42	2
SCE1572_463	97	1481
SCE1572_2269	220	772
SCE1572_2014	107	9
SCE1572_11362	101	11
SCE1572_8089	41	8
SCE1572_10875	59	13
SCE1572_2905	55	215
SCE1572_1343	17	5
SCE1572_1942	0	0
SCE1572_3306	60	353
SCE1572_1228	64	47
SCE1572_1940	592	64
SCE1572_1682	465	1932
SCE1572_698	20	39
SCE1572_3802	51	18
SCE1572_9575	1	0
SCE1572_6582	24	30
SCE1572_1327	0	0
SCE1572_11011	0	0
SCE1572_4689	202.23	462.26
SCE1572_1876	215	129
SCE1572_4210	2	0
SCE1572_5576	40	7
SCE1572_2071	9	4
SCE1572_8403	218	64
SCE1572_4074	504	1659
SCE1572_8413	39	5
SCE1572_971	103	33
SCE1572_4779	0	0
SCE1572_5177	131	30
SCE1572_11035	27	118
SCE1572_2898	9	1

SCE1572_6077	2	4
SCE1572_6599	80	60
SCE1572_8208	137	45
SCE1572_10807	134	44
SCE1572_9801	61	10
SCE1572_10016	12	48
SCE1572_8705	0	0
SCE1572_6104	104	10
SCE1572_149	85.25	112
SCE1572_4348	83	10
SCE1572_4459	82	14
SCE1572_6653	12	0
SCE1572_22	164	132
SCE1572_3838	0	0
SCE1572_859	596	7198
SCE1572_4472	34	3
SCE1572_883	0	0
SCE1572_8703	35	35
SCE1572_852	565	856
SCE1572_10841	56	6
SCE1572_4651	109	62
SCE1572_10180	74	99
SCE1572_6267	21	9
SCE1572_9253	52	7
SCE1572_10558	43	7
SCE1572_9033	41	10
SCE1572_6099	147	89
SCE1572_270	52	17
SCE1572_4144	104	197
SCE1572_10722	116	16
SCE1572_4387	66	28
SCE1572_7440	17	5
SCE1572_4606	20	64
SCE1572_7298	197	19
SCE1572_2146	68.11	8
SCE1572_5409	1	0
SCE1572_10003	35	14
SCE1572_11163	64	181
SCE1572_8801	0	0
SCE1572_5724	0	0
SCE1572_8768	16	5
SCE1572_8717	8	1
SCE1572_5373	43	279
SCE1572_7416	178	387
SCE1572_6597	83	22
SCE1572_9793	76	24
SCE1572_8644	149	45
SCE1572_3691	647	56

SCE1572_7388	52	14
SCE1572_5004	20	27
SCE1572_5238	246	109
SCE1572_7254	39	1
SCE1572_3476	153	174
SCE1572_4108	75.55	16
SCE1572_10084	86	81
SCE1572_10610	8	1
SCE1572_2612	70	18
SCE1572_4900	151	21
SCE1572_6579	47	15
SCE1572_8909	80	16
SCE1572_4133	16	5
SCE1572_8468	105	95
SCE1572_11178	0	0
SCE1572_8008	231	2013
SCE1572_11440	70	20
SCE1572_10681	40	18
SCE1572_10109	41	53
SCE1572_4743	51	22
SCE1572_659	27	0
SCE1572_11288	203	693
SCE1572_8408	176	66
SCE1572_11317	312	169
SCE1572_10412	7	2
SCE1572_9435	477	967
SCE1572_458	82	19
SCE1572_8424	41	25
SCE1572_1975	153	233
SCE1572_3518	22	3
SCE1572_1964	41	5
SCE1572_402	57	7
SCE1572_505	14	2
SCE1572_1064	330	53
SCE1572_5014	127	311
SCE1572_2931	0	0
SCE1572_6421	61	4
SCE1572_11152	18	5
SCE1572_7357	33	17
SCE1572_7060	108	16
SCE1572_4051	194	423
SCE1572_6721	61	121
SCE1572_5139	34	7
SCE1572_9366	16	1
SCE1572_8998	235	588
SCE1572_3050	19	1
SCE1572_9162	139	2
SCE1572_4088	309	154

SCE1572_3638	52	9
SCE1572_112	20.47	0
SCE1572_944	186.94	24.87
SCE1572_8781	105	923
SCE1572_8255	297	174
SCE1572_10114	18.4	1
SCE1572_378	39	16
SCE1572_6474	31	39
SCE1572_1151	7	10
SCE1572_3373	28	5
SCE1572_707	140	449
SCE1572_10043	99	934
SCE1572_5806	23	26
SCE1572_8629	36	55
SCE1572_789	0	0
SCE1572_7654	25	7
SCE1572_11227	118	44
SCE1572_9610	27	6
SCE1572_3759	163.12	76.15
SCE1572_8431	67	13
SCE1572_6394	495	78
SCE1572_4813	94	274
SCE1572_956	0	0
SCE1572_9964	1007.99	153.74
SCE1572_3095	107	82
SCE1572_1045	64	33
SCE1572_9929	11	0
SCE1572_4715	91	99
SCE1572_4466	96	20
SCE1572_5790	50	56
SCE1572_10673	53	39
SCE1572_5555	710	345
SCE1572_4416	143	18
SCE1572_1095	38	106
SCE1572_1561	51	11
SCE1572_11536	66	55
SCE1572_931	414	3398
SCE1572_10824	139	110
SCE1572_10989	41	39
SCE1572_10632	0	0
SCE1572_8849	61	16
SCE1572_1369	114	90
SCE1572_8873	149	16
SCE1572_7841	52	12
SCE1572_6954	47	8
SCE1572_6734	64	64
SCE1572_277	161	39
SCE1572_6792	74	186

SCE1572_1930	186	140
SCE1572_6904	67	5
SCE1572_8436	293	90
SCE1572_5740	26	20
SCE1572_2501	25	27
SCE1572_6364	43	215
SCE1572_6853	247	105
SCE1572_6998	82	25
SCE1572_5197	6	0
SCE1572_2307	50	15
SCE1572_442	84	34
SCE1572_5755	321	433
SCE1572_1506	103	14
SCE1572_5989	0	1.16
SCE1572_5735	109	95
SCE1572_748	11	52
SCE1572_2480	7	1
SCE1572_4219	88	35
SCE1572_84	15	37
SCE1572_1353	75	20
SCE1572_9971	127	390
SCE1572_3015	81	12
SCE1572_1688	19	12
SCE1572_5883	53	28
SCE1572_46	181	152
SCE1572_8942	99	32
SCE1572_6298	81	72
SCE1572_10480	19	25
SCE1572_358	180	53
SCE1572_9328	453	244
SCE1572_11506	85	36
SCE1572_1422	67	27
SCE1572_7771	57	110
SCE1572_10041	11	0
SCE1572_3400	165	398
SCE1572_4564	63	24
SCE1572_7216	109	16
SCE1572_8620	41	89
SCE1572_7538	203	45
SCE1572_3101	10	25
SCE1572_7310	56	19
SCE1572_1841	89	270
SCE1572_8951	0	0
SCE1572_1109	0	0
SCE1572_4233	50	11
SCE1572_5651	0	0
SCE1572_6824	214	215
SCE1572_4772	79	8

SCE1572_10701	0	0
SCE1572_2548	66	10
SCE1572_8226	21	12
SCE1572_6678	552	899
SCE1572_9976	0	0
SCE1572_10309	135	2
SCE1572_10850	85	15
SCE1572_2619	73	15
SCE1572_10448	27	19
SCE1572_9314	264	131
SCE1572_10513	0	0
SCE1572_3255	18	51
SCE1572_447	53	17
SCE1572_5309	47	26
SCE1572_4922	132	11
SCE1572_10111	244	735
SCE1572_10948	244	102
SCE1572_10603	0	0
SCE1572_9947	13	6
SCE1572_8828	20.65	4
SCE1572_4063	224	142
SCE1572_7816	35	97
SCE1572_11439	266	54
SCE1572_5087	101	346
SCE1572_10390	169	35
SCE1572_4984	231	33
SCE1572_962	1241	255
SCE1572_6731	0	0
SCE1572_6274	118	284
SCE1572_9403	227	55
SCE1572_811	9	2
SCE1572_2950	1	0
SCE1572_7992	145	6
SCE1572_4011	255	299
SCE1572_6865	131	41
SCE1572_184	10	0
SCE1572_3282	10	2
SCE1572_902	36	4
SCE1572_4283	15	10
SCE1572_6898	326	341
SCE1572_3025	75	11
SCE1572_6173	41	76
SCE1572_10123	287	110
SCE1572_4703	90	11
SCE1572_8151	42	7
SCE1572_5490	89	16
SCE1572_8063	3	0
SCE1572_2627	190	31

SCE1572_7197	128	28
SCE1572_1417	143	29
SCE1572_1725	331	278
SCE1572_1628	120	88
SCE1572_1088	15	1
SCE1572_8112	86	11
SCE1572_4165	76	9
SCE1572_8032	0	0
SCE1572_4701	182	57
SCE1572_9146	0	0
SCE1572_7488	37	27
SCE1572_11458	148	106
SCE1572_8606	1	0
SCE1572_6672	0	0
SCE1572_2497	160	47
SCE1572_1834	11	4
SCE1572_5445	55	11
SCE1572_6338	39	45
SCE1572_4951	19	4
SCE1572_1853	164	21
SCE1572_8353	1425	3163
SCE1572_3978	71	22
SCE1572_6754	214	25
SCE1572_1385	58	20
SCE1572_8476	237	82
SCE1572_10644	187	1720
SCE1572_9231	35	2
SCE1572_8190	340	48
SCE1572_2845	48.68	0
SCE1572_11599	37	6
SCE1572_10978	452	632
SCE1572_1715	34	17
SCE1572_8492	395	309
SCE1572_10207	32	16
SCE1572_7058	30	4
SCE1572_10278	28	2
SCE1572_3458	96	9
SCE1572_9348	196	74
SCE1572_4112	128	16
SCE1572_8726	68	13
SCE1572_4380	220	34
SCE1572_1848	166	34
SCE1572_2087	0	0
SCE1572_3332	0	0
SCE1572_649	0	0
SCE1572_7681	52	16
SCE1572_10768	3	0
SCE1572_3366	40	290

SCE1572_642	225	200
SCE1572_495	0	0
SCE1572_8682	28	30
SCE1572_10830	54	12
SCE1572_7033	41	6
SCE1572_9004	41	5
SCE1572_6210	544.12	126.28
SCE1572_1318	16	2
SCE1572_10861	2085	3407
SCE1572_5279	303.95	98
SCE1572_1757	319	69
SCE1572_11216	66	25
SCE1572_1196	46	29
SCE1572_10504	61	30
SCE1572_5466	130	25
SCE1572_3924	54	162
SCE1572_8855	107	108
SCE1572_2551	75	18
SCE1572_5458	69	8
SCE1572_6620	41	11
SCE1572_8188	0	0
SCE1572_7975	37	2
SCE1572_7279	1	0
SCE1572_4931	95	25
SCE1572_1311	301	1343
SCE1572_11144	40	12
SCE1572_5963	102	22
SCE1572_6383	27	0
SCE1572_829	37	7
SCE1572_5384	34	15
SCE1572_1487	82	17
SCE1572_8840	210	36
SCE1572_523	77	51
SCE1572_3667	26	2
SCE1572_7557	30	5
SCE1572_1166	130	29
SCE1572_11125	21	8
SCE1572_1184	429	81
SCE1572_7067	50	1
SCE1572_5834	9	75
SCE1572_1163	28	19
SCE1572_4018	80	28
SCE1572_7445	118	26
SCE1572_1434	587	136
SCE1572_9655	27.29	1.49
SCE1572_7673	125.11	13.95
SCE1572_8245	79	51
SCE1572_4717	25	6

SCE1572_1580	55	10
SCE1572_5583	93	23
SCE1572_6541	31	9
SCE1572_9920	291	54
SCE1572_9846	27	2
SCE1572_1577	78	11
SCE1572_5334	315	407
SCE1572_6943	10	1
SCE1572_3108	63	14
SCE1572_9279	34	11
SCE1572_6286	12	0
SCE1572_8816	76	9
SCE1572_8082	0	0
SCE1572_3655	8	0
SCE1572_10716	44	4
SCE1572_11235	48	9
SCE1572_9715	20	5
SCE1572_11294	23	2
SCE1572_7203	0	0
SCE1572_8035	89.78	25
SCE1572_6584	54	57
SCE1572_8461	70	31
SCE1572_1336	54	74
SCE1572_793	265	147
SCE1572_7665	118	6
SCE1572_2794	3	0
SCE1572_6889	47	6
SCE1572_9744	80	142
SCE1572_3750	111	16
SCE1572_4096	10	0
SCE1572_2359	51	51
SCE1572_8788	0	0
SCE1572_6826	20	41
SCE1572_3548	60	13
SCE1572_11159	21	15
SCE1572_408	58	23
SCE1572_5227	2	0
SCE1572_11130	241	57
SCE1572_10467	246	215
SCE1572_3438	0	0
SCE1572_6817	69	4
SCE1572_7186	78	16
SCE1572_96	47	19
SCE1572_6495	4	0
SCE1572_8418	66	15
SCE1572_7341	287	323
SCE1572_7078	0	0
SCE1572_10851	58.02	49.41

SCE1572_7495	46	7
SCE1572_1364	781	1815
SCE1572_8373	313	266
SCE1572_1409	331	40
SCE1572_8385	257.75	46.02
SCE1572_3261	142	96
SCE1572_10236	1346	349
SCE1572_511	374	133
SCE1572_4374	14	0
SCE1572_5181	88	1195
SCE1572_151	16	3
SCE1572_9345	65	54
SCE1572_9339	194	641
SCE1572_3679	87	18
SCE1572_2753	14	2
SCE1572_409	54	12
SCE1572_8963	68	19
SCE1572_5866	33	13
SCE1572_9943	167	235
SCE1572_6922	19	67
SCE1572_7576	43	18
SCE1572_10653	911	3155
SCE1572_3540	310	45
SCE1572_6624	44	21
SCE1572_1407	31	12
SCE1572_7668	31.63	9.67
SCE1572_11058	246	110
SCE1572_11351	821	149
SCE1572_4301	242	31
SCE1572_11186	63	4
SCE1572_4581	27	5
SCE1572_7793	254	62
SCE1572_10556	0	0
SCE1572_4424	143	21
SCE1572_6548	0	0
SCE1572_11520	21	3
SCE1572_9256	286	157
SCE1572_2808	89.34	14.22
SCE1572_5481	6	1
SCE1572_1399	28	9
SCE1572_3976	72	40
SCE1572_11220	73	12
SCE1572_5675	43	25
SCE1572_8046	4	0
SCE1572_3532	106	16
SCE1572_497	87	5
SCE1572_5045	114	69
SCE1572_7862	133	276

SCE1572_10457	0	0
SCE1572_3988	40	21
SCE1572_4427	86	7
SCE1572_2966	322	69
SCE1572_1302	27	87
SCE1572_2224	66	11
SCE1572_3722	211	4036
SCE1572_8483	45	23
SCE1572_868	128	117
SCE1572_6323	66	35
SCE1572_2738	18	3
SCE1572_551	58	38
SCE1572_2777	160	25
SCE1572_10497	97	28
SCE1572_3054	6	0
SCE1572_9102	0	0
SCE1572_8755	64	6
SCE1572_418	1180	122
SCE1572_2763	261	44
SCE1572_2782	162	54
SCE1572_8075	94.94	49.52
SCE1572_8351	21	4
SCE1572_3440	310	51
SCE1572_5891	51	32
SCE1572_687	49	6
SCE1572_8834	487	124
SCE1572_8177	88	18
SCE1572_207	0	0
SCE1572_3032	25	11
SCE1572_10055	763	645
SCE1572_3818	192	254
SCE1572_5068	412	199
SCE1572_8131	32	8
SCE1572_2508	113	26
SCE1572_10008	59	14
SCE1572_374	192	33
SCE1572_1856	130	22
SCE1572_4912	18	9
SCE1572_9600	101	233
SCE1572_7917	483	340
SCE1572_10874	72	20
SCE1572_11091	72	44
SCE1572_186	104	28
SCE1572_9095	91	78
SCE1572_9559	37	21
SCE1572_6572	28.33	15.66
SCE1572_9421	228	720
SCE1572_1864	0	0

SCE1572_332	48	51
SCE1572_7794	8	6
SCE1572_1172	56	21
SCE1572_275	198	35
SCE1572_2385	173	113
SCE1572_3762	348	364
SCE1572_3689	31	20
SCE1572_1411	207	26
SCE1572_7456	113	25
SCE1572_8588	64	19
SCE1572_3566	83	13
SCE1572_8290	505	71
SCE1572_10780	25	12
SCE1572_7008	0	0
SCE1572_7731	16	3
SCE1572_324	214	74
SCE1572_2609	58	20
SCE1572_9401	26	21
SCE1572_8525	57	29
SCE1572_9917	93	18
SCE1572_9467	59	29
SCE1572_1069	109	10
SCE1572_3292	114	166
SCE1572_3169	175	161
SCE1572_5164	16	144
SCE1572_10170	124.6	70
SCE1572_4247	164	70
SCE1572_6962	47	33
SCE1572_6846	52	164
SCE1572_7255	403	1051
SCE1572_2472	42	511
SCE1572_9380	12	3
SCE1572_608	346	233
SCE1572_1253	26	5
SCE1572_10490	41	23
SCE1572_3421	58	31
SCE1572_7373	59	13
SCE1572_10214	128	66
SCE1572_2349	92	47
SCE1572_2276	254	580
SCE1572_2721	426	140
SCE1572_7020	26.37	2
SCE1572_19	197	392
SCE1572_7011	233	65
SCE1572_2701	233	45
SCE1572_7597	62	114
SCE1572_2824	84	26
SCE1572_5544	79	8

SCE1572_1255	348	108
SCE1572_8202	332	37
SCE1572_11095	58	10
SCE1572_6634	332	52
SCE1572_11539	50	5
SCE1572_7590	253	138
SCE1572_2982	0	0
SCE1572_3642	241	40
SCE1572_4673	81	41
SCE1572_8381	269	107
SCE1572_3684	37	2
SCE1572_11068	176	124
SCE1572_5215	214.85	217.58
SCE1572_9176	0	0
SCE1572_7660	125	15
SCE1572_2295	6	0
SCE1572_1951	77	11
SCE1572_10914	68	27
SCE1572_3771	108	59
SCE1572_4734	92	53
SCE1572_9248	91	51
SCE1572_9044	168	53
SCE1572_7753	24	6
SCE1572_2459	100	38
SCE1572_619	26	23
SCE1572_2208	12	3
SCE1572_6685	104	25
SCE1572_8140	63	146
SCE1572_2100	1	1
SCE1572_7943	43	74
SCE1572_3561	60	9
SCE1572_2812	109	49
SCE1572_2616	117	142
SCE1572_11378	58	197
SCE1572_7741	128	46
SCE1572_2788	39	11
SCE1572_10737	167	120
SCE1572_7952	587	717
SCE1572_8556	116	125
SCE1572_6446	12	2
SCE1572_7823	134	252
SCE1572_2620	176	45
SCE1572_3539	183	33
SCE1572_4863	70	30
SCE1572_758	18	7
SCE1572_2022	709	2900
SCE1572_1531	55	18
SCE1572_3493	261	45

SCE1572_9001	660	460
SCE1572_3160	75	26
SCE1572_6856	83	23
SCE1572_8396	40	71
SCE1572_7565	38	7
SCE1572_10607	51	40
SCE1572_4457	74	40
SCE1572_6302	352	2096
SCE1572_686	173	22
SCE1572_9609	154	245
SCE1572_6534	52	5
SCE1572_1067	99	12
SCE1572_8549	88	16
SCE1572_10335	157	159
SCE1572_6230	27	13
SCE1572_8511	78	27
SCE1572_3376	30	105
SCE1572_9056	275	47
SCE1572_6493	48	15
SCE1572_6929	84	9
SCE1572_7439	6	0
SCE1572_1885	35	63
SCE1572_1143	48	34
SCE1572_5873	223	66
SCE1572_10362	106	461
SCE1572_935	100.74	141.76
SCE1572_8154	67	6
SCE1572_5588	46	7
SCE1572_5557	192.25	184.25
SCE1572_10329	48	25
SCE1572_9506	133	21
SCE1572_1129	78	15
SCE1572_10473	181	500
SCE1572_9261	50	6
SCE1572_11027	58	16
SCE1572_10021	198.75	35.19
SCE1572_5766	711	1570
SCE1572_8864	35	0
SCE1572_8784	61	129
SCE1572_3341	526	480
SCE1572_8426	16	0
SCE1572_3308	20	1
SCE1572_5817	4	0
SCE1572_8452	28	99
SCE1572_11319	47	5
SCE1572_11590	29	9
SCE1572_638	59	34
SCE1572_676	73	11

SCE1572_924	135	228
SCE1572_477	19	7
SCE1572_5854	4	0
SCE1572_8399	163	83
SCE1572_9778	177	308
SCE1572_10799	267	99
SCE1572_6307	184	87
SCE1572_9318	956	611
SCE1572_8454	39	37
SCE1572_10319	134	90
SCE1572_11003	264	244
SCE1572_2102	52	74
SCE1572_9742	161	96
SCE1572_11176	17	45
SCE1572_9915	356	77
SCE1572_3158	223	52
SCE1572_1959	1	0
SCE1572_11222	129	23
SCE1572_1390	130	171
SCE1572_8976	20	2
SCE1572_9109	10	4
SCE1572_8307	1920	41
SCE1572_5075	6	1
SCE1572_5368	28	6
SCE1572_4535	43	8
SCE1572_1060	0	0
SCE1572_10024	93.52	26
SCE1572_7131	14	1
SCE1572_3131	153	33
SCE1572_9820	245	85
SCE1572_9221	908.67	268.61
SCE1572_2830	13	1
SCE1572_8737	104	20
SCE1572_397	32	10
SCE1572_8722	38	5
SCE1572_6430	107	46
SCE1572_10990	35	16
SCE1572_2957	1070	249
SCE1572_2633	140.06	4.43
SCE1572_2152	36	20
SCE1572_8609	191	46
SCE1572_2351	128	663
SCE1572_4214	0	0
SCE1572_10179	121	232
SCE1572_4310	249	32
SCE1572_2901	0	0
SCE1572_4780	247.26	172.24
SCE1572_2325	26	57

SCE1572_5726	363	1170
SCE1572_4085	50	7
SCE1572_10771	26	7
SCE1572_4758	503	115
SCE1572_5256	24	4
SCE1572_1299	150	30
SCE1572_5810	9783	5101
SCE1572_6758	74	17
SCE1572_4391	140	23
SCE1572_9224	134	12
SCE1572_5469	229	20
SCE1572_9835	33	24
SCE1572_8593	0	0
SCE1572_11483	270	156
SCE1572_1668	85	29
SCE1572_1459	698.67	109.32
SCE1572_3583	111	26
SCE1572_412	23	10
SCE1572_600	1	0
SCE1572_9438	36	29
SCE1572_2566	12	0
SCE1572_9883	40	20
SCE1572_6088	102	109
SCE1572_2140	40	8
SCE1572_10421	58	11
SCE1572_177	8	1
SCE1572_10303	323	80
SCE1572_8851	6	24
SCE1572_9275	243	121
SCE1572_7141	33	4
SCE1572_5063	9	3
SCE1572_1703	308	141
SCE1572_10427	4	1
SCE1572_4378	169	8
SCE1572_9816	30	19
SCE1572_5436	105	17
SCE1572_7434	14	25
SCE1572_8193	210	30
SCE1572_2565	343	399
SCE1572_2288	63	38
SCE1572_3407	321	347
SCE1572_451	22	17
SCE1572_10258	14	1
SCE1572_10908	117	87
SCE1572_6533	97	5
SCE1572_9584	111	49
SCE1572_11239	68	11
SCE1572_6703	64	17

SCE1572_11426	43	26
SCE1572_11433	221	182
SCE1572_7054	17	7
SCE1572_10145	502	335
SCE1572_6669	25	11
SCE1572_424	53	124
SCE1572_7603	93	12
SCE1572_5010	199	484
SCE1572_8712	65	11
SCE1572_1532	36	54
SCE1572_4541	483	61
SCE1572_11156	35	15
SCE1572_2923	144	29
SCE1572_8324	100	15
SCE1572_4907	75	52
SCE1572_1086	147	77
SCE1572_138	19	10
SCE1572_3447	39	26
SCE1572_11404	51	15
SCE1572_3467	81	17
SCE1572_2077	67	37
SCE1572_1034	175.58	25.14
SCE1572_9949	65	8
SCE1572_6714	592	601
SCE1572_10060	0	0
SCE1572_1650	24	6
SCE1572_3244	102	94
SCE1572_2213	115	106
SCE1572_1321	92	85
SCE1572_11242	274	58
SCE1572_727	31	3
SCE1572_1447	94	15
SCE1572_3859	231	152
SCE1572_7886	30	56
SCE1572_10741	31	19
SCE1572_5400	63	14
SCE1572_11565	23	20
SCE1572_6214	29	4
SCE1572_6468	104	14
SCE1572_10937	86	115
SCE1572_8931	34	6
SCE1572_5964	13	5
SCE1572_8632	21	34
SCE1572_3887	85	125
SCE1572_7678	26	129
SCE1572_2009	88	10
SCE1572_4732	22	7
SCE1572_9734	41	50

SCE1572_7472	89	63
SCE1572_3191	0	0
SCE1572_5760	45	10
SCE1572_558	66	200
SCE1572_645	5	7
SCE1572_11076	31	244
SCE1572_7111	115	46
SCE1572_8934	26	5
SCE1572_6047	8	50
SCE1572_7091	96	33
SCE1572_9453	70	58
SCE1572_2237	68	22
SCE1572_8524	174	426
SCE1572_10151	62	179
SCE1572_3396	247	96
SCE1572_9789	37	12
SCE1572_10660	12	0
SCE1572_906	59	0
SCE1572_7351	43	7
SCE1572_1804	1.99	0
SCE1572_7529	48	9
SCE1572_7789	0	0
SCE1572_10748	58	50
SCE1572_1989	0	1
SCE1572_1949	2	0
SCE1572_6237	50	9
SCE1572_9782	34	10
SCE1572_7306	3	0
SCE1572_2281	59	11
SCE1572_7964	18	7
SCE1572_7423	77	51
SCE1572_5667	5	0
SCE1572_10563	130	40
SCE1572_7275	89	14
SCE1572_11133	86	23
SCE1572_9114	2	0
SCE1572_7239	0	0
SCE1572_4027	305	338
SCE1572_5905	0	0
SCE1572_7267	313	42
SCE1572_2133	387	42
SCE1572_10810	78	71
SCE1572_4116	36	2
SCE1572_5080	451	1254
SCE1572_8448	232.9	33
SCE1572_7157	133	21
SCE1572_2129	32	9
SCE1572_794	256	150

SCE1572_4368	48	75
SCE1572_11311	254	49
SCE1572_11337	39	9
SCE1572_9843	46.55	61.05
SCE1572_2222	26	9
SCE1572_5124	74	3
SCE1572_1070	42	1
SCE1572_1187	39	1
SCE1572_545	76	7
SCE1572_2849	0	0
SCE1572_2230	252	353
SCE1572_1076	102	90
SCE1572_3932	1581	4145
SCE1572_7165	177	18
SCE1572_10357	75	295
SCE1572_1051	55	17
SCE1572_7137	53	5
SCE1572_3023	62	11
SCE1572_7337	179	300
SCE1572_11344	421	677
SCE1572_11394	56	87
SCE1572_11594	172	447
SCE1572_3525	208	1187
SCE1572_3917	250	64
SCE1572_7408	34	76
SCE1572_7932	75	22
SCE1572_3065	15	7
SCE1572_1835	45	17
SCE1572_5417	155	87
SCE1572_6263	57	22
SCE1572_2908	43	25
SCE1572_4998	37	32
SCE1572_5705	68	35
SCE1572_11269	102	53
SCE1572_10778	158	57
SCE1572_1114	138	236
SCE1572_1515	21	24
SCE1572_10885	93	21
SCE1572_6780	0	0
SCE1572_4885	81	17
SCE1572_329	57	51
SCE1572_7039	86	15
SCE1572_2000	9	0
SCE1572_9025	105	168
SCE1572_4176	198	36
SCE1572_3506	119	146
SCE1572_6256	45	8
SCE1572_5642	9	4

SCE1572_2374	122	431
SCE1572_5660	65.96	7
SCE1572_7032	205.46	41
SCE1572_4616	5	1
SCE1572_10293	0	0
SCE1572_8577	0	0
SCE1572_3910	122	97
SCE1572_1431	4	0
SCE1572_4795	47	53
SCE1572_316	182	40
SCE1572_5633	47	14
SCE1572_3703	60	11
SCE1572_4595	24	9
SCE1572_10587	71	9
SCE1572_10763	1	0
SCE1572_10935	43	15
SCE1572_4963	633	1023
SCE1572_6137	15	8
SCE1572_6748	41	10
SCE1572_5606	40	14
SCE1572_9687	289	262
SCE1572_2832	87	64
SCE1572_1818	17	9
SCE1572_4454	33	9
SCE1572_221	173	77
SCE1572_6037	4	0
SCE1572_11273	104	187
SCE1572_6277	92	44
SCE1572_3590	14	0
SCE1572_9591	343	1882
SCE1572_6556	68	9
SCE1572_9625	111	34
SCE1572_4274	43	92
SCE1572_3501	80	28
SCE1572_10414	53	30
SCE1572_5925	79	49
SCE1572_11104	15	42
SCE1572_3573	28	1
SCE1572_9635	143	60
SCE1572_9194	35	23
SCE1572_2062	92	26
SCE1572_1990	108	24
SCE1572_9006	160	94
SCE1572_4254	7	1
SCE1572_6660	73	29
SCE1572_6434	365	41
SCE1572_9096	34	38
SCE1572_11386	494	838

SCE1572_5521	252	116
SCE1572_2842	3.5	0
SCE1572_10730	32	3
SCE1572_2286	21	21
SCE1572_11466	128	37
SCE1572_1440	176	88
SCE1572_9727	62	15
SCE1572_5503	100	38
SCE1572_10188	102	74
SCE1572_3854	95	41
SCE1572_9729	130	60
SCE1572_888	23	1
SCE1572_8618	102	30
SCE1572_7014	12	1
SCE1572_7244	0	0
SCE1572_1233	141	20
SCE1572_9239	48	9
SCE1572_2490	131	22
SCE1572_61	102	88
SCE1572_6335	175	13
SCE1572_6765	140	18
SCE1572_9170	3	0
SCE1572_268	0	0
SCE1572_3927	98	65
SCE1572_993	34	29
SCE1572_8575	149.9	10.48
SCE1572_11250	10	0
SCE1572_2748	205	120
SCE1572_6245	42	4
SCE1572_10450	14	1
SCE1572_44	72	44
SCE1572_4878	30	6
SCE1572_1646	0	0
SCE1572_7509	0	0
SCE1572_6008	68	280
SCE1572_8285	57	45
SCE1572_5281	63	22
SCE1572_7812	181	661
SCE1572_10549	100.36	22.05
SCE1572_172	140	24
SCE1572_2944	100	17
SCE1572_2332	224.59	86
SCE1572_9460	99	14
SCE1572_194	0	0
SCE1572_4556	82	14
SCE1572_11456	33	7
SCE1572_869	0	0
SCE1572_1905	5	0

SCE1572_7101	691	823
SCE1572_11474	248.87	29.53
SCE1572_5193	50	105
SCE1572_5443	114	16
SCE1572_4741	89	20
SCE1572_10553	0	0
SCE1572_3114	228	31
SCE1572_4068	86	54
SCE1572_11072	113	106
SCE1572_9767	86	301
SCE1572_756	0	0
SCE1572_4464	238	32
SCE1572_7980	27	9
SCE1572_6512	111	16
SCE1572_9297	128	202
SCE1572_5574	175	50
SCE1572_6775	0	0
SCE1572_9184	53	12
SCE1572_3090	179	72
SCE1572_2940	44	37
SCE1572_1007	174	187
SCE1572_10101	32	7
SCE1572_9664	26	56
SCE1572_4521	28	4
SCE1572_9451	0	0
SCE1572_10349	0	0
SCE1572_6063	4	0
SCE1572_7711	86	93
SCE1572_6040	543	349
SCE1572_7295	220	105
SCE1572_5612	73	59
SCE1572_914	51	58
SCE1572_7338	85	308
SCE1572_9546	166	115
SCE1572_2361	179	131
SCE1572_10081	875	2216
SCE1572_6425	88	9
SCE1572_7087	146	26
SCE1572_3122	209	34
SCE1572_10629	451.27	730.92
SCE1572_6183	200	25
SCE1572_2407	185	309
SCE1572_9479	236	109
SCE1572_3946	660	288
SCE1572_2018	18	7
SCE1572_7193	143.17	73
SCE1572_8678	33	9
SCE1572_6295	92	224

SCE1572_4225	136	12
SCE1572_9757	43	14
SCE1572_2709	79	12
SCE1572_1134	22	8
SCE1572_5682	32	57
SCE1572_6996	44	12
SCE1572_4442	236	60
SCE1572_10956	41	56
SCE1572_3745	39	5
SCE1572_4336	267	21
SCE1572_3099	74	7
SCE1572_11336	8	2
SCE1572_1216	2	0
SCE1572_535	253	280
SCE1572_9158	108	24
SCE1572_166	22	24
SCE1572_3410	4	0
SCE1572_2161	545	83
SCE1572_3908	13	1
SCE1572_6777	15	5
SCE1572_6645	59	15
SCE1572_3965	40	14
SCE1572_4261	246	54
SCE1572_2344	169	398
SCE1572_6770	3	2
SCE1572_1828	81	12
SCE1572_5426	154	48
SCE1572_7726	16	164
SCE1572_598	30	7
SCE1572_3901	57	54
SCE1572_8478	7	0
SCE1572_10284	60	92
SCE1572_11240	42	17
SCE1572_4461	360	16
SCE1572_10638	12	0
SCE1572_3799	221	314
SCE1572_1786	102	80
SCE1572_10158	42	10
SCE1572_8338	321.52	92.96
SCE1572_8980	73	10
SCE1572_3954	87	25
SCE1572_6417	0	0
SCE1572_9079	0	0
SCE1572_3083	76	7
SCE1572_10595	197	22
SCE1572_3328	170	202
SCE1572_3434	352	273
SCE1572_9984	218	45

SCE1572_8968	143	39
SCE1572_39	220	77
SCE1572_2411	100	163
SCE1572_1999	25	5
SCE1572_3318	19	24
SCE1572_10817	104	32
SCE1572_9696	228	235
SCE1572_6524	331	140
SCE1572_6569	31	77
SCE1572_7689	0	0
SCE1572_6110	35	6
SCE1572_7027	206.07	118
SCE1572_2816	127	23
SCE1572_5263	0	0
SCE1572_2933	0	0
SCE1572_6272	77	53
SCE1572_7271	34	5
SCE1572_4318	113	9
SCE1572_8239	82	93
SCE1572_2660	0	0
SCE1572_4415	32	3
SCE1572_11571	36	751
SCE1572_308	65	62
SCE1572_3326	76	66
SCE1572_4125	110	19
SCE1572_7515	31	15
SCE1572_6405	57	31
SCE1572_802	244	142
SCE1572_9326	324	192
SCE1572_4981	43	66
SCE1572_7173	127	217
SCE1572_8233	42	30
SCE1572_10355	54	150
SCE1572_8626	70	19
SCE1572_822	2	0
SCE1572_2586	27.44	4
SCE1572_1120	183	29
SCE1572_10702	15	69
SCE1572_9415	1	0
SCE1572_6120	187	70
SCE1572_3047	45	10
SCE1572_1540	92	84
SCE1572_7232	139	23
SCE1572_3848	27	7
SCE1572_7998	76.72	15.17
SCE1572_8882	0	0
SCE1572_4751	100	168
SCE1572_6050	0	0

SCE1572_10386	405	78
SCE1572_9410	458	3304
SCE1572_3578	11	15
SCE1572_9550	128	36
SCE1572_6199	2	0
SCE1572_4504	213	48
SCE1572_7179	60	15
SCE1572_7040	12	0
SCE1572_4185	270	192
SCE1572_2868	77	29
SCE1572_304	533	63
SCE1572_11445	158	94
SCE1572_4021	53	32
SCE1572_4574	107	27
SCE1572_2367	0	0
SCE1572_1750	69	27
SCE1572_986	488	756
SCE1572_7378	14	5
SCE1572_1790	43	5
SCE1572_2796	22	9
SCE1572_2184	340	46
SCE1572_2119	76	65
SCE1572_6351	209	26
SCE1572_8494	17	0
SCE1572_8812	61	10
SCE1572_898	34	9
SCE1572_11468	70	86
SCE1572_6079	0	0
SCE1572_8680	34	12
SCE1572_3347	577	129
SCE1572_6055	321	195
SCE1572_5153	89	11
SCE1572_119	156	176
SCE1572_8896	70	143
SCE1572_1242	287	129
SCE1572_3419	216	112
SCE1572_9666	3	9
SCE1572_2487	175	284
SCE1572_1264	0	0
SCE1572_5098	189	337
SCE1572_2474	113	321
SCE1572_3592	17	6
SCE1572_1830	155	65
SCE1572_5597	226	343
SCE1572_7095	230	35
SCE1572_10249	0	0
SCE1572_4482	366	367
SCE1572_161	92	11

SCE1572_4585	55	30
SCE1572_63	122	45
SCE1572_2872	73	22
SCE1572_2379	39	71
SCE1572_6262	66	17
SCE1572_5456	26	4
SCE1572_8360	11	1
SCE1572_7880	28	12
SCE1572_6935	94	39
SCE1572_6679	64	22
SCE1572_3121	107	75
SCE1572_4352	0	0
SCE1572_1623	0	0
SCE1572_8671	7	1
SCE1572_10766	75	1544
SCE1572_4866	33	126
SCE1572_1285	1458	12485
SCE1572_9791	0	0
SCE1572_24	90	49
SCE1572_9539	66	6
SCE1572_3941	2432	1390
SCE1572_6796	53	42
SCE1572_9330	355	465
SCE1572_1657	43	15
SCE1572_3963	190	24
SCE1572_7879	167	60
SCE1572_5175	59	13
SCE1572_8423	272	74
SCE1572_4437	0	0
SCE1572_2421	55	37
SCE1572_6988	41	3
SCE1572_11010	81	167
SCE1572_5170	295	1526
SCE1572_8563	56	145
SCE1572_3747	0	0
SCE1572_3998	131	99
SCE1572_4977	215	1491
SCE1572_6959	1	0
SCE1572_1684	94	51
SCE1572_7333	0	0
SCE1572_3804	117	20
SCE1572_9627	614.79	96
SCE1572_7297	49	10
SCE1572_3672	19.52	5
SCE1572_1107	162	15
SCE1572_11117	44	12
SCE1572_11541	24	1
SCE1572_10815	49	15

SCE1572_11555	105	19
SCE1572_7690	46.55	61.05
SCE1572_6318	38	10
SCE1572_6941	68	12
SCE1572_8	179	78
SCE1572_9759	110	18
SCE1572_7657	78	22
SCE1572_6851	8	2
SCE1572_7002	0	0
SCE1572_9166	60	7
SCE1572_2975	111.19	9.19
SCE1572_4128	22	10
SCE1572_2261	96	128
SCE1572_4234	78	10
SCE1572_461	11	2
SCE1572_9873	24	8
SCE1572_9372	96	25
SCE1572_72	149	84
SCE1572_228	10	0
SCE1572_82	0	0
SCE1572_5341	273	127
SCE1572_10486	48	36
SCE1572_6682	44	119
SCE1572_9571	49	22
SCE1572_9040	63	168
SCE1572_11052	171	820
SCE1572_11360	277	521
SCE1572_5202	48	31
SCE1572_6880	1	0
SCE1572_4610	40	15
SCE1572_7675	823.86	88.05
SCE1572_1661	111	137
SCE1572_7892	685	13084
SCE1572_9047	2	0
SCE1572_5277	0	0
SCE1572_5998	59	48
SCE1572_8266	456	143
SCE1572_938	14	6
SCE1572_7383	171	26
SCE1572_2457	1	0
SCE1572_2054	186	111
SCE1572_571	0	0
SCE1572_4856	100	12
SCE1572_591	232	146
SCE1572_4653	160	31
SCE1572_3661	61	5
SCE1572_10229	9	3
SCE1572_6595	0	0

SCE1572_10091	290	692
SCE1572_4346	0	0
SCE1572_2896	28	3
SCE1572_692	12	6
SCE1572_881	158	96
SCE1572_4474	0	0
SCE1572_7801	202	137
SCE1572_6981	187	7
SCE1572_2582	169	38
SCE1572_5949	350	79
SCE1572_6269	15	18
SCE1572_11015	5	2
SCE1572_5129	0	0
SCE1572_1584	52	104
SCE1572_427	54	258
SCE1572_857	48	431
SCE1572_3371	84	19
SCE1572_6197	3	1
SCE1572_640	20	14
SCE1572_9128	32	76
SCE1572_1193	135	98
SCE1572_5713	127	28
SCE1572_7201	82	29
SCE1572_11346	118	204
SCE1572_11037	110	218
SCE1572_7343	126	60
SCE1572_8128	14	0
SCE1572_1226	107	14
SCE1572_513	105	22
SCE1572_5913	19	4
SCE1572_1944	131	18
SCE1572_2663	33	9
SCE1572_4762	190	1267
SCE1572_3875	82	69
SCE1572_9233	139	254
SCE1572_3797	198	118
SCE1572_9285	52	26
SCE1572_11165	58	4
SCE1572_9532	73	472
SCE1572_8340	0	0
SCE1572_9803	44	29
SCE1572_8719	68	16
SCE1572_7418	0	0
SCE1572_4619	0	0
SCE1572_3836	7	0
SCE1572_1873	21	4
SCE1572_7970	47	30
SCE1572_3265	0	0

SCE1572_7069	79	125
SCE1572_8463	775	1419
SCE1572_7553	22	5
SCE1572_11110	117.44	141.12
SCE1572_3915	15	0
SCE1572_5528	238	51
SCE1572_6920	18	0
SCE1572_9578	79	12
SCE1572_8019	0	0.02
SCE1572_8538	85	30
SCE1572_10597	45	5
SCE1572_3834	1	0
SCE1572_261	70	9
SCE1572_877	227	191
SCE1572_7620	62	24
SCE1572_8823	42	11
SCE1572_10432	254	66
SCE1572_3485	115	36
SCE1572_9925	279.21	367
SCE1572_736	72	22
SCE1572_7536	274	30
SCE1572_9893	173	40
SCE1572_3474	77	14
SCE1572_4680	2	0
SCE1572_5298	43	17
SCE1572_940	276	32
SCE1572_8983	3	0
SCE1572_6162	100	57
SCE1572_11509	216	62
SCE1572_9247	100	9
SCE1572_8387	80	7
SCE1572_10404	118	40
SCE1572_7127	1	0
SCE1572_7652	252	42
SCE1572_1001	0	0
SCE1572_3203	62	307
SCE1572_2243	224.59	53
SCE1572_10340	190	25
SCE1572_4698	5	1
SCE1572_92	267	247
SCE1572_547	71	31
SCE1572_8219	18	8
SCE1572_4004	0	0
SCE1572_4774	75	0
SCE1572_5326	0	0
SCE1572_6895	0	0
SCE1572_6208	0	0
SCE1572_6946	47	11

SCE1572_406	1	0
SCE1572_6188	32	9
SCE1572_5364	5	0
SCE1572_3196	183	215
SCE1572_3934	0	0
SCE1572_8611	98	16
SCE1572_6810	625	715
SCE1572_8806	0	0
SCE1572_4158	78	17
SCE1572_625	0	0
SCE1572_9337	546	680
SCE1572_9717	200	998
SCE1572_848	1913	574
SCE1572_9658	69	60
SCE1572_6828	79	26
SCE1572_7207	15	19
SCE1572_5783	66	52
SCE1572_7362	10	1
SCE1572_6948	327	112
SCE1572_6328	58	43
SCE1572_5971	121	96
SCE1572_11515	11	5
SCE1572_9998	53	5
SCE1572_7827	102	124
SCE1572_11498	0	0
SCE1572_8627	49	13
SCE1572_6396	47	66
SCE1572_1816	32	4
SCE1572_1689	30	17
SCE1572_540	199	53
SCE1572_4847	141	99
SCE1572_54	65	14
SCE1572_2120	166	114
SCE1572_1480	141	19
SCE1572_3175	211	161
SCE1572_6472	32	2
SCE1572_5395	0	0
SCE1572_7930	307	59
SCE1572_6485	27	15
SCE1572_2317	30	8
SCE1572_11521	58	9
SCE1572_6877	471	472
SCE1572_2337	21	5
SCE1572_9657	68	28
SCE1572_99	14	3
SCE1572_10461	213	400
SCE1572_2610	42	30
SCE1572_2693	61	11

SCE1572_5047	48	26
SCE1572_465	57	56
SCE1572_1806	36	10
SCE1572_1545	285	88
SCE1572_10098	47	79
SCE1572_783	190	32
SCE1572_9872	70	138
SCE1572_1542	12	13
SCE1572_6838	90	20
SCE1572_283	87	29
SCE1572_4492	62	21
SCE1572_850	2315	3252
SCE1572_3599	19	14
SCE1572_9433	82	45
SCE1572_1837	211	165
SCE1572_10439	287	438
SCE1572_10075	1439	1171
SCE1572_10995	167	47
SCE1572_5183	41	327
SCE1572_10468	6	1
SCE1572_10502	133	43
SCE1572_1563	10	0
SCE1572_8311	41	3
SCE1572_3752	201	162
SCE1572_4381	180	34
SCE1572_164	54	14
SCE1572_2810	3	0
SCE1572_4276	216	50
SCE1572_5476	195	2076
SCE1572_10133	35	11
SCE1572_11496	113	13
SCE1572_3154	18	8
SCE1572_10954	138	17
SCE1572_11204	156	144
SCE1572_6699	69	14
SCE1572_6692	3	0
SCE1572_2592	0	0
SCE1572_11437	53	34
SCE1572_4044	35	38
SCE1572_4691	64	45
SCE1572_5415	479	56
SCE1572_4748	270	55
SCE1572_7421	12	2
SCE1572_5627	52	3
SCE1572_6284	57	47
SCE1572_6442	191	45
SCE1572_916	0	0
SCE1572_240	27.64	0

SCE1572_11184	34	3
SCE1572_6705	1854	2921
SCE1572_4810	0	0
SCE1572_2750	95	59
SCE1572_5828	56	13
SCE1572_7923	11	1
SCE1572_7154	29	8
SCE1572_9510	10	0
SCE1572_7887	192	397
SCE1572_9937	8	0
SCE1572_4961	34	60
SCE1572_8370	65	9
SCE1572_2727	58	12
SCE1572_8092	16	1
SCE1572_8693	111	10
SCE1572_2531	15	0
SCE1572_746	166	101
SCE1572_2099	39	10
SCE1572_5565	193	60
SCE1572_10542	137	38
SCE1572_9272	55	22
SCE1572_7316	0	0
SCE1572_8991	7	6
SCE1572_9863	604	97
SCE1572_5231	183	290
SCE1572_840	208	216
SCE1572_11308	73	7
SCE1572_2198	0	0
SCE1572_10222	36	25
SCE1572_4626	324	239
SCE1572_11257	49	33
SCE1572_10224	736	204
SCE1572_6698	42	18
SCE1572_580	30	28
SCE1572_6523	1	0
SCE1572_5519	0	0
SCE1572_8347	128	56
SCE1572_10511	0	0
SCE1572_444	25	3
SCE1572_5371	286	156
SCE1572_7543	55	97
SCE1572_7928	245	80
SCE1572_6072	46	8
SCE1572_6289	17	16
SCE1572_7872	252	331
SCE1572_7766	63	10
SCE1572_7584	15	0
SCE1572_2304	168	201

SCE1572_10314	31	2
SCE1572_6450	380	123
SCE1572_9907	92	62
SCE1572_385	1	0
SCE1572_10765	88	304
SCE1572_647	0	0
SCE1572_8929	20	30
SCE1572_6354	13	2
SCE1572_5287	25	66
SCE1572_6201	168	32
SCE1572_9991	140	17
SCE1572_395	98	58
SCE1572_9673	57	78
SCE1572_774	173	172
SCE1572_10195	22	3
SCE1572_796	312	253
SCE1572_1428	63	8
SCE1572_1400	19	8
SCE1572_2573	70	8
SCE1572_5836	15	6
SCE1572_11301	28	32
SCE1572_2655	114	34
SCE1572_4710	34	8
SCE1572_479	87	11
SCE1572_5447	93	14
SCE1572_6103	21	4
SCE1572_3603	521	134
SCE1572_4110	59	6
SCE1572_813	68	13
SCE1572_8604	59	51
SCE1572_8055	17	6
SCE1572_5747	8	1
SCE1572_1893	4	17
SCE1572_2990	4	0
SCE1572_3542	0	0
SCE1572_5938	18	2
SCE1572_1912	17	0
SCE1572_10333	0	0
SCE1572_967	0	0
SCE1572_11447	367	146
SCE1572_8007	109	948
SCE1572_780	14	2
SCE1572_10863	71	344
SCE1572_6841	386	205
SCE1572_9493	0	0
SCE1572_10754	145	25
SCE1572_6212	253	42
SCE1572_1405	50	14

SCE1572_5390	0	0
SCE1572_7572	78.33	75
SCE1572_8821	14	14
SCE1572_1339	88	181
SCE1572_4924	48	32
SCE1572_7839	58	33
SCE1572_533	11	16
SCE1572_6340	63	22
SCE1572_1485	190	67
SCE1572_6969	66	246
SCE1572_4180	8	7
SCE1572_6543	139	71
SCE1572_7713	27	5
SCE1572_1020	50.31	73.74
SCE1572_1352	131	78
SCE1572_6221	64	14
SCE1572_4928	2	0
SCE1572_11194	55	115
SCE1572_8274	41	29
SCE1572_7612	315	265
SCE1572_8192	49	10
SCE1572_2938	149	117
SCE1572_1723	7	2
SCE1572_10164	38	5
SCE1572_10441	13	16
SCE1572_10980	26	19
SCE1572_3704	39	76
SCE1572_2165	161	37
SCE1572_2117	191	133
SCE1572_4941	54	35
SCE1572_8030	355.68	124.41
SCE1572_6903	173	17
SCE1572_4896	7	2
SCE1572_7270	366	122
SCE1572_7226	96	146
SCE1572_6720	6094	9354
SCE1572_9067	58	16
SCE1572_8853	4	1
SCE1572_3409	57	14
SCE1572_4072	265	296
SCE1572_1230	360	184
SCE1572_9833	58	8
SCE1572_4537	54.65	9.58
SCE1572_5721	166	71
SCE1572_11218	131.99	41
SCE1572_4782	95	32
SCE1572_10205	130.69	13.29
SCE1572_5110	79	297

SCE1572_4953	303	77
SCE1572_4499	72	23
SCE1572_5453	89	12
SCE1572_1383	0	0
SCE1572_10128	1	0
SCE1572_3035	41	7
SCE1572_3385	111	48
SCE1572_2578	105	8
SCE1572_8254	77	1595
SCE1572_10620	87	29
SCE1572_4986	34	36
SCE1572_6819	52	3
SCE1572_7748	25	1
SCE1572_1165	205	95
SCE1572_6381	15	36
SCE1572_3296	0	0
SCE1572_5450	91	36
SCE1572_7191	278	268
SCE1572_2557	388	77
SCE1572_3615	111	15
SCE1572_10857	273.61	72
SCE1572_4117	22	1332
SCE1572_6867	1083	429
SCE1572_8380	28	12
SCE1572_1761	88	10
SCE1572_10234	6.45	0
SCE1572_10232	65	6
SCE1572_1579	24	11
SCE1572_5315	22	14
SCE1572_4151	64	239
SCE1572_3657	29	13
SCE1572_10615	139	35
SCE1572_1341	100	259
SCE1572_8243	220	211
SCE1572_7414	30	2
SCE1572_10911	3	0
SCE1572_5225	132	164
SCE1572_2396	24	26
SCE1572_4400	39	8
SCE1572_8842	83	50
SCE1572_10419	98	20
SCE1572_7959	11	8
SCE1572_1153	87.56	110.88
SCE1572_5336	39	10
SCE1572_4719	129	17
SCE1572_6586	253	24
SCE1572_7897	11	10
SCE1572_11158	86.55	34.21

SCE1572_3884	40	6
SCE1572_8080	16	1
SCE1572_3106	50	38
SCE1572_8814	68.31	14
SCE1572_5302	112	12
SCE1572_8654	1210	2779
SCE1572_10843	8	0
SCE1572_7605	68	16
SCE1572_1593	47	36
SCE1572_11127	71	20
SCE1572_6369	117	47
SCE1572_741	38	25
SCE1572_1749	52	17
SCE1572_11146	156	137
SCE1572_6097	44	31
SCE1572_7486	43	14
SCE1572_10655	38	122
SCE1572_4816	1	0
SCE1572_2034	391	285
SCE1572_1214	25	6
SCE1572_10804	67	10
SCE1572_4252	54	8
SCE1572_11597	159	780
SCE1572_6887	21	2
SCE1572_7811	27	3
SCE1572_1359	270	351
SCE1572_10714	79	9
SCE1572_5251	237	412
SCE1572_7395	30	2
SCE1572_587	106	20
SCE1572_6808	181	294
SCE1572_2363	18	4
SCE1572_9844	11	10
SCE1572_10526	15	3
SCE1572_9342	106	591
SCE1572_4146	32	5
SCE1572_7281	25	48
SCE1572_4372	307	48
SCE1572_814	37	6
SCE1572_5025	85	25
SCE1572_1083	31	2
SCE1572_6457	130	28
SCE1572_3233	95	26
SCE1572_671	65	90
SCE1572_4379	95	15
SCE1572_10683	205	190
SCE1572_1027	117	1222
SCE1572_1631	22	2

SCE1572_8958	121	91
SCE1572_7873	78	113
SCE1572_233	9	9
SCE1572_10045	401	1907
SCE1572_344	312	39
SCE1572_5842	166	19
SCE1572_5402	67	18
SCE1572_10627	143	190
SCE1572_1	243	620
SCE1572_5460	49	1
SCE1572_5738	249	109
SCE1572_5815	45	19
SCE1572_6362	190	58
SCE1572_247	193.36	19.56
SCE1572_2757	61	16
SCE1572_8113	56	7
SCE1572_6567	79	58
SCE1572_3516	53	90
SCE1572_10268	242	69
SCE1572_4095	99	34
SCE1572_6711	82	60
SCE1572_7319	37	20
SCE1572_8996	124	73
SCE1572_10202	160.93	25.85
SCE1572_4229	118	34
SCE1572_10113	42	38
SCE1572_3289	136	33
SCE1572_5800	180	209
SCE1572_2827	23	2
SCE1572_10116	87	70
SCE1572_8211	276	148
SCE1572_6952	65	7
SCE1572_1962	129	20
SCE1572_9390	94	72
SCE1572_960	58	44
SCE1572_10946	391	74
SCE1572_2999	51	9
SCE1572_1927	86	67
SCE1572_9035	54	11
SCE1572_10724	0	0
SCE1572_4058	30	8
SCE1572_6655	44	9
SCE1572_438	0	0
SCE1572_5849	46	4
SCE1572_6601	537	258
SCE1572_8322	38	58
SCE1572_10662	176	56
SCE1572_10127	10	2

SCE1572_3361	7	0
SCE1572_5931	65	15
SCE1572_7120	236	53
SCE1572_8914	40	6
SCE1572_7213	84	5
SCE1572_5808	146	25
SCE1572_1738	36	0
SCE1572_1178	101	16
SCE1572_5531	46	0
SCE1572_3085	0	0
SCE1572_4189	166	167
SCE1572_1290	53	16
SCE1572_6503	16	0
SCE1572_7966	48	9
SCE1572_7867	13	42
SCE1572_10986	1784	13931
SCE1572_8302	181	213
SCE1572_712	7	1
SCE1572_1371	190	72
SCE1572_10014	180	29
SCE1572_8836	0	0
SCE1572_4135	63	9
SCE1572_9308	1618	4088
SCE1572_7860	43	27
SCE1572_11530	85	21
SCE1572_1168	493	92
SCE1572_3268	227	1279
SCE1572_5142	237	34
SCE1572_954	4	1
SCE1572_6321	19.12	8
SCE1572_9892	0	0
SCE1572_8065	199	19
SCE1572_8022	17	8
SCE1572_1397	56	45
SCE1572_6727	147	133
SCE1572_946	200	28.41
SCE1572_2467	107	33
SCE1572_1047	95	13
SCE1572_4000	58	51
SCE1572_7773	72	45
SCE1572_2072	114	22
SCE1572_6390	96	101
SCE1572_1177	91	39
SCE1572_8664	48	4
SCE1572_5863	153	36
SCE1572_10634	195	192
SCE1572_3028	0	0
SCE1572_1678	52	57

SCE1572_376	80	12
SCE1572_4086	203	43
SCE1572_4789	111	400
SCE1572_9083	29	1
SCE1572_8181	13	1
SCE1572_94	126	38
SCE1572_8595	0	0
SCE1572_4562	100	54
SCE1572_6368	71	173
SCE1572_7514	0	0
SCE1572_709	0	0
SCE1572_10710	42.28	8
SCE1572_3553	4	0
SCE1572_253	54	9
SCE1572_8944	4	0
SCE1572_6674	22	16
SCE1572_6736	127	272
SCE1572_2448	37	8
SCE1572_11291	57	20
SCE1572_5059	98	56
SCE1572_3156	41	17
SCE1572_1016	0	0
SCE1572_10518	437	113
SCE1572_7907	286	706
SCE1572_1586	82.08	58.94
SCE1572_10977	104	68
SCE1572_6973	1	0
SCE1572_9853	185	30
SCE1572_1932	289	34
SCE1572_9952	24	8
SCE1572_5881	135	50
SCE1572_5954	62	18
SCE1572_110	343	468
SCE1572_6687	10	7
SCE1572_8903	148	13
SCE1572_5683	301	390
SCE1572_8061	3	0
SCE1572_2297	138	210
SCE1572_9316	65	59
SCE1572_7818	29	15
SCE1572_9386	22	12
SCE1572_4554	23	2
SCE1572_2012	91	17
SCE1572_3431	167	182
SCE1572_8225	138	30
SCE1572_9164	128	16
SCE1572_3644	110	23
SCE1572_966	174	136

SCE1572_9205	41	20
SCE1572_2462	87	56
SCE1572_5733	269	935
SCE1572_636	432	526
SCE1572_10837	87	55
SCE1572_8783	129	314
SCE1572_5757	182	82
SCE1572_5085	0	0
SCE1572_9612	4	0
SCE1572_5009	230	148
SCE1572_5052	119.36	27.99
SCE1572_6416	44	4
SCE1572_2625	0	0
SCE1572_5034	86	12
SCE1572_7625	307	220
SCE1572_3253	42	13
SCE1572_8099	93	322
SCE1572_4840	89	275
SCE1572_431	168	42
SCE1572_4065	101	22
SCE1572_10086	346	626
SCE1572_8010	76	172
SCE1572_7807	487	159
SCE1572_4797	142	205
SCE1572_10784	406	172
SCE1572_3402	46	72
SCE1572_10601	48	7
SCE1572_6016	188	234
SCE1572_6822	165	817
SCE1572_656	1004	898
SCE1572_8398	170	43
SCE1572_7062	0	0
SCE1572_9868	81	72

TableS3 Transcripts detected under different pH conditions (edgeR)

Locus tag	logFoldChange	logConc	PValue	FDR
SCE1572_3527	-8.60991438	-11.1416	6.37E-21	6.70E-17
SCE1572_4119	-7.635412187	-11.6046	5.55E-18	2.92E-14
SCE1572_5270	-7.65599141	-13.1793	2.74E-17	9.62E-14
SCE1572_3528	-7.080685475	-12.034	2.68E-16	7.05E-13
SCE1572_4117	-7.191370235	-13.2741	4.49E-16	9.19E-13
SCE1572_8972	-6.902167314	-11.4858	5.67E-16	9.19E-13
SCE1572_4118	-6.956242225	-12.2072	6.11E-16	9.19E-13
SCE1572_10922	-6.753614141	-8.32851	7.99E-16	1.05E-12
SCE1572_3526	-6.874307896	-13.2482	3.11E-15	3.63E-12
SCE1572_10923	-6.380810321	-9.14727	1.06E-14	1.12E-11
SCE1572_7894	38.08986581	-30.9711	1.97E-14	1.88E-11
SCE1572_7402	-6.587931306	-13.7133	3.93E-14	3.44E-11
SCE1572_3712	-6.177538232	-12.2862	1.03E-13	8.32E-11
SCE1572_1395	-5.998550436	-11.2006	1.93E-13	1.36E-10
SCE1572_5184	-6.154693386	-12.9665	1.94E-13	1.36E-10
SCE1572_8009	-5.86766727	-9.11692	3.30E-13	1.99E-10
SCE1572_7401	-6.38476496	-14.4364	3.32E-13	1.99E-10
SCE1572_5187	-6.152318874	-13.4457	3.40E-13	1.99E-10
SCE1572_3340	-5.717542553	-9.87797	9.41E-13	5.21E-10
SCE1572_9597	-5.63472751	-10.6539	1.82E-12	9.20E-10
SCE1572_4147	-5.905629507	-13.7914	1.91E-12	9.20E-10
SCE1572_7889	-5.705273172	-12.2477	1.92E-12	9.20E-10
SCE1572_4152	-5.812304919	-13.2531	2.09E-12	9.56E-10
SCE1572_4735	-5.575829284	-9.42494	2.33E-12	1.02E-09
SCE1572_1985	-5.55787848	-9.41583	2.66E-12	1.12E-09
SCE1572_10766	-5.635061834	-12.2829	3.08E-12	1.21E-09
SCE1572_7892	-5.526979353	-9.14575	3.18E-12	1.21E-09
SCE1572_8254	-5.643977655	-12.2404	3.23E-12	1.21E-09
SCE1572_1948	-5.671526655	-13.034	3.92E-12	1.37E-09
SCE1572_3722	-5.529034757	-10.8436	3.92E-12	1.37E-09
SCE1572_11571	-5.654167583	-13.3322	5.97E-12	2.03E-09
SCE1572_7890	-5.413504577	-9.41788	6.90E-12	2.27E-09
SCE1572_3139	-5.824205582	-14.3296	9.21E-12	2.94E-09
SCE1572_1306	-5.349009388	-8.75232	1.01E-11	3.12E-09
SCE1572_187	-5.474574112	-12.6377	1.13E-11	3.30E-09
SCE1572_9596	-5.666380792	-13.7956	1.13E-11	3.30E-09
SCE1572_7896	-5.295711508	-7.3376	1.34E-11	3.82E-09
SCE1572_5185	-5.351055506	-11.3049	1.39E-11	3.84E-09
SCE1572_7893	-5.296932593	-9.75296	1.50E-11	4.04E-09
SCE1572_3784	-5.506001447	-13.2543	1.59E-11	4.17E-09
SCE1572_6311	-5.390560379	-12.906	2.18E-11	5.59E-09
SCE1572_3374	-5.262286692	-11.0687	2.24E-11	5.59E-09
SCE1572_6279	-5.376957901	-12.8334	2.29E-11	5.59E-09
SCE1572_1265	-5.254233103	-10.7479	2.34E-11	5.59E-09
SCE1572_6042	-5.260282929	-11.6767	2.86E-11	6.68E-09
SCE1572_7891	-5.240486505	-11.9408	3.38E-11	7.72E-09

SCE1572_463	-5.20386657	-12.1274	4.41E-11	9.86E-09
SCE1572_350	-5.159472042	-11.5303	5.09E-11	1.11E-08
SCE1572_6014	-5.39358207	-13.7744	5.69E-11	1.22E-08
SCE1572_5180	-5.200540001	-12.5391	5.85E-11	1.23E-08
SCE1572_3710	-5.053851301	-8.03492	6.64E-11	1.37E-08
SCE1572_858	-5.035961159	-9.59205	8.35E-11	1.67E-08
SCE1572_10487	-5.018176738	-8.29949	8.41E-11	1.67E-08
SCE1572_5186	-5.05981732	-11.2601	9.13E-11	1.78E-08
SCE1572_1284	-5.022413248	-10.5796	9.84E-11	1.88E-08
SCE1572_7227	-5.005982584	-11.7494	1.46E-10	2.73E-08
SCE1572_7744	-5.04880335	-12.7604	1.56E-10	2.87E-08
SCE1572_5181	-5.034786771	-12.3524	1.59E-10	2.89E-08
SCE1572_3987	-4.979242736	-11.9327	1.76E-10	3.15E-08
SCE1572_7844	-4.934219	-11.0547	1.90E-10	3.32E-08
SCE1572_10361	-5.129404482	-13.8576	2.33E-10	4.01E-08
SCE1572_8098	-5.38476496	-14.9364	2.41E-10	4.08E-08
SCE1572_859	-4.865635354	-9.67721	2.54E-10	4.14E-08
SCE1572_7403	-4.894405595	-11.3822	2.55E-10	4.14E-08
SCE1572_1026	-5.480876853	-15.2668	2.56E-10	4.14E-08
SCE1572_7367	-4.858407581	-10.1996	2.78E-10	4.44E-08
SCE1572_6630	-5.086998916	-14.1418	4.57E-10	7.15E-08
SCE1572_5182	-5.070165279	-14.2092	4.62E-10	7.15E-08
SCE1572_5015	-4.773260672	-10.6206	5.03E-10	7.67E-08
SCE1572_9714	-4.9885806	-13.7905	5.13E-10	7.70E-08
SCE1572_2391	-4.843504696	-12.6786	5.58E-10	8.26E-08
SCE1572_2964	-4.745354675	-11.8797	7.89E-10	1.15E-07
SCE1572_2397	-4.971863205	-14.0359	8.06E-10	1.16E-07
SCE1572_2472	-4.876285545	-13.4987	8.30E-10	1.18E-07
SCE1572_410	-5.097971974	-14.7802	9.56E-10	1.34E-07
SCE1572_5476	-4.683683902	-11.38	9.76E-10	1.34E-07
SCE1572_559	-4.925926921	-14.1113	9.81E-10	1.34E-07
SCE1572_5188	-4.741038878	-12.6919	1.19E-09	1.61E-07
SCE1572_1365	-4.65800454	-11.6079	1.24E-09	1.65E-07
SCE1572_1977	-4.60174892	-8.82131	1.32E-09	1.73E-07
SCE1572_3202	-4.613475851	-10.7985	1.40E-09	1.80E-07
SCE1572_8472	-4.762088691	-13.1933	1.40E-09	1.80E-07
SCE1572_1027	-4.656087337	-12.1308	1.43E-09	1.81E-07
SCE1572_1794	-4.82694221	-13.8713	1.46E-09	1.83E-07
SCE1572_9595	-4.851367913	-14.3186	1.88E-09	2.33E-07
SCE1572_9340	-4.541864828	-9.05827	1.95E-09	2.38E-07
SCE1572_10699	-4.643212096	-12.4998	1.98E-09	2.39E-07
SCE1572_2679	-4.727652674	-13.6078	2.00E-09	2.39E-07
SCE1572_5327	-4.539650562	-10.5516	2.18E-09	2.58E-07
SCE1572_7476	36.2567094	-31.8877	2.49E-09	2.91E-07
SCE1572_4622	-4.562977933	-11.8778	2.53E-09	2.92E-07
SCE1572_10924	-4.491386971	-9.39002	2.73E-09	3.13E-07
SCE1572_3307	-4.484904854	-11.0588	3.35E-09	3.79E-07
SCE1572_10644	-4.472721877	-11.5459	3.85E-09	4.31E-07

SCE1572_4800	-4.562545535	-12.8982	3.91E-09	4.33E-07
SCE1572_3726	-4.545785899	-12.8469	4.25E-09	4.63E-07
SCE1572_10043	-4.509345607	-12.4452	4.32E-09	4.63E-07
SCE1572_7220	-4.657481919	-13.9561	4.32E-09	4.63E-07
SCE1572_11285	-4.548095301	-12.9677	4.39E-09	4.67E-07
SCE1572_4426	-4.471169621	-12.0383	4.74E-09	4.98E-07
SCE1572_1683	-5.414381441	-16.037	4.78E-09	4.98E-07
SCE1572_1025	-4.651566196	-13.916	4.88E-09	5.03E-07
SCE1572_2097	-4.926775316	-14.9589	5.13E-09	5.24E-07
SCE1572_7636	-4.387882464	-9.73012	5.33E-09	5.39E-07
SCE1572_1285	-4.369556684	-8.63465	5.58E-09	5.59E-07
SCE1572_8008	-4.394805903	-11.28	6.23E-09	6.18E-07
SCE1572_8395	-4.483311782	-13.0875	6.38E-09	6.27E-07
SCE1572_6179	-4.398035121	-12.0112	6.80E-09	6.58E-07
SCE1572_564	-4.487152178	-13.1314	6.82E-09	6.58E-07
SCE1572_11073	-5.714366983	-16.472	7.55E-09	7.22E-07
SCE1572_8919	-4.730855106	-14.6418	7.71E-09	7.30E-07
SCE1572_2512	-4.456069994	-13.0788	8.02E-09	7.50E-07
SCE1572_8781	-4.407364807	-12.4113	8.09E-09	7.50E-07
SCE1572_8007	-4.391982411	-12.365	8.13E-09	7.50E-07
SCE1572_9416	-4.381198345	-12.2682	8.23E-09	7.53E-07
SCE1572_5033	-4.353441399	-11.4871	8.43E-09	7.65E-07
SCE1572_931	-4.308406667	-10.4815	9.26E-09	8.33E-07
SCE1572_8543	-4.364351089	-12.1928	9.44E-09	8.41E-07
SCE1572_8990	-4.30580005	-11.1876	1.02E-08	9.02E-07
SCE1572_6090	-4.441348488	-13.6491	1.14E-08	1.00E-06
SCE1572_857	-4.438005045	-13.5252	1.20E-08	1.04E-06
SCE1572_10986	-4.236534788	-8.41003	1.29E-08	1.11E-06
SCE1572_9026	-4.445452887	-13.8211	1.38E-08	1.17E-06
SCE1572_3725	-4.236874162	-9.77614	1.38E-08	1.17E-06
SCE1572_9693	-4.272964005	-12.3224	1.63E-08	1.37E-06
SCE1572_11202	-4.271423487	-12.2393	1.69E-08	1.41E-06
SCE1572_7726	-4.628975492	-15.0147	1.86E-08	1.54E-06
SCE1572_8915	-4.508462684	-14.6827	1.99E-08	1.63E-06
SCE1572_7222	-4.271423487	-12.7505	2.01E-08	1.64E-06
SCE1572_10296	-4.201773055	-11.7126	2.24E-08	1.80E-06
SCE1572_9598	-4.178021714	-10.8782	2.24E-08	1.80E-06
SCE1572_3367	-4.182449857	-11.5306	2.31E-08	1.84E-06
SCE1572_7586	-4.317435885	-13.4156	2.34E-08	1.84E-06
SCE1572_8307	4.277915104	-12.5613	2.35E-08	1.84E-06
SCE1572_4905	-4.28589397	-13.2321	2.40E-08	1.87E-06
SCE1572_11570	-4.609293126	-14.8546	2.45E-08	1.88E-06
SCE1572_4101	-4.158391625	-10.7988	2.45E-08	1.88E-06
SCE1572_9594	-4.309291673	-13.62	2.50E-08	1.91E-06
SCE1572_2229	-4.343573273	-13.872	2.59E-08	1.96E-06
SCE1572_5084	-4.160865112	-11.7331	2.90E-08	2.18E-06
SCE1572_9593	-4.174399033	-12.2995	2.99E-08	2.22E-06
SCE1572_9410	-4.12221767	-10.4289	3.01E-08	2.22E-06

SCE1572_7219	-4.132389793	-11.1183	3.01E-08	2.22E-06
SCE1572_10499	-4.441348488	-14.6491	3.32E-08	2.43E-06
SCE1572_7339	-4.338778755	-14.0723	3.46E-08	2.51E-06
SCE1572_5183	-4.267018308	-13.8381	3.64E-08	2.62E-06
SCE1572_1286	-4.067829841	-9.47033	3.94E-08	2.82E-06
SCE1572_3724	-4.266792038	-13.9104	4.15E-08	2.93E-06
SCE1572_5106	-4.308081958	-14.0877	4.15E-08	2.93E-06
SCE1572_560	-4.063066785	-10.3669	4.27E-08	3.00E-06
SCE1572_4977	-4.06529518	-11.5484	4.81E-08	3.35E-06
SCE1572_2312	-4.042977688	-10.6353	5.03E-08	3.48E-06
SCE1572_5164	-4.441348488	-15.1085	5.91E-08	4.06E-06
SCE1572_975	-4.005917806	-10.6503	6.19E-08	4.23E-06
SCE1572_6280	-4.347774373	-14.6318	6.42E-08	4.36E-06
SCE1572_1017	-4.278918024	-14.6048	6.81E-08	4.55E-06
SCE1572_11076	-4.247964514	-14.251	6.82E-08	4.55E-06
SCE1572_4001	-4.041435982	-12.366	6.83E-08	4.55E-06
SCE1572_9414	-3.990546795	-10.6231	6.92E-08	4.55E-06
SCE1572_2346	-4.185008735	-13.8104	6.93E-08	4.55E-06
SCE1572_295	-5.015584583	-16.4995	6.99E-08	4.57E-06
SCE1572_4762	-4.008768688	-11.755	7.09E-08	4.60E-06
SCE1572_1263	-3.994276345	-11.5119	7.32E-08	4.72E-06
SCE1572_6730	-3.949683133	-8.44672	7.73E-08	4.96E-06
SCE1572_11034	-3.969609748	-10.9139	8.01E-08	5.11E-06
SCE1572_3337	-3.952227306	-10.0244	8.28E-08	5.25E-06
SCE1572_3723	-4.148939481	-13.8285	8.73E-08	5.50E-06
SCE1572_7709	-3.927117851	-8.01747	8.85E-08	5.52E-06
SCE1572_7366	-3.955809166	-11.1965	8.87E-08	5.52E-06
SCE1572_9417	-3.936640732	-10.2867	9.26E-08	5.73E-06
SCE1572_1307	-3.925090982	-9.8728	9.64E-08	5.93E-06
SCE1572_5366	-4.00122929	-12.6704	9.85E-08	6.03E-06
SCE1572_9777	-4.227480139	-14.5716	1.01E-07	6.11E-06
SCE1572_6769	-3.913424915	-9.50521	1.02E-07	6.11E-06
SCE1572_2196	-3.97097512	-12.3779	1.02E-07	6.11E-06
SCE1572_3339	-3.919506869	-10.3526	1.02E-07	6.11E-06
SCE1572_9713	-4.181871902	-14.4834	1.04E-07	6.20E-06
SCE1572_3366	-4.129404482	-13.9426	1.05E-07	6.20E-06
SCE1572_3719	-4.018192256	-13.2757	1.07E-07	6.27E-06
SCE1572_6874	-3.912506074	-10.4691	1.08E-07	6.30E-06
SCE1572_4660	-4.021972053	-13.1088	1.12E-07	6.52E-06
SCE1572_5770	-3.893525124	-9.37541	1.14E-07	6.58E-06
SCE1572_10095	-4.186306873	-14.6511	1.18E-07	6.76E-06
SCE1572_1526	-3.88753753	-9.44145	1.19E-07	6.77E-06
SCE1572_2264	-4.084191905	-14.0392	1.19E-07	6.77E-06
SCE1572_2398	-3.94467252	-12.6704	1.33E-07	7.50E-06
SCE1572_4974	-3.923949272	-12.1774	1.33E-07	7.50E-06
SCE1572_3986	-3.901204492	-11.6921	1.41E-07	7.87E-06
SCE1572_9532	-3.964241978	-13.1573	1.51E-07	8.41E-06
SCE1572_1308	-3.835549214	-9.76397	1.65E-07	9.14E-06

SCE1572_8301	-3.859280061	-11.4396	1.66E-07	9.16E-06
SCE1572_6302	-3.84541487	-10.9471	1.68E-07	9.18E-06
SCE1572_2980	-3.867274304	-11.9361	1.72E-07	9.39E-06
SCE1572_5016	-3.847283781	-11.3557	1.74E-07	9.43E-06
SCE1572_3338	-3.902455636	-12.5578	1.75E-07	9.44E-06
SCE1572_5189	-3.991900958	-13.8738	1.85E-07	9.88E-06
SCE1572_7043	-3.965052673	-13.732	1.85E-07	9.88E-06
SCE1572_861	-3.898977864	-12.904	1.89E-07	9.97E-06
SCE1572_6932	-3.919496032	-13.0119	1.90E-07	9.97E-06
SCE1572_8970	-3.919496032	-13.0119	1.90E-07	9.97E-06
SCE1572_11538	-3.923500184	-13.3902	1.94E-07	1.01E-05
SCE1572_11253	-3.805131946	-9.77021	1.98E-07	1.03E-05
SCE1572_4786	-3.787683402	-9.26293	2.18E-07	1.13E-05
SCE1572_5234	-3.834087321	-12.0634	2.22E-07	1.15E-05
SCE1572_4002	-3.797492299	-10.6167	2.23E-07	1.15E-05
SCE1572_10760	-4.396954369	-15.6713	2.26E-07	1.15E-05
SCE1572_7637	-3.831468356	-12.2236	2.35E-07	1.19E-05
SCE1572_10904	-3.935906327	-13.7174	2.36E-07	1.20E-05
SCE1572_6294	-4.070165279	-14.7092	2.39E-07	1.20E-05
SCE1572_4978	-3.842051072	-12.5255	2.41E-07	1.20E-05
SCE1572_11313	-3.814271114	-12.0297	2.44E-07	1.22E-05
SCE1572_1671	-3.823378882	-12.2882	2.47E-07	1.23E-05
SCE1572_1619	-3.782225205	-11.1343	2.52E-07	1.24E-05
SCE1572_5373	-3.969280044	-13.9183	2.58E-07	1.27E-05
SCE1572_10975	-3.778600315	-11.1731	2.60E-07	1.27E-05
SCE1572_11324	-3.820076424	-12.5863	2.67E-07	1.30E-05
SCE1572_9637	-3.810327967	-12.4933	2.71E-07	1.31E-05
SCE1572_3525	-3.784087989	-11.7367	2.80E-07	1.35E-05
SCE1572_1986	-3.795109268	-12.1367	2.83E-07	1.36E-05
SCE1572_3268	-3.765675549	-11.6198	3.02E-07	1.44E-05
SCE1572_5372	-3.750452543	-11.0488	3.04E-07	1.45E-05
SCE1572_9099	-3.728718458	-9.71665	3.13E-07	1.48E-05
SCE1572_5260	-3.804855687	-12.8569	3.29E-07	1.55E-05
SCE1572_2726	-3.799802459	-12.7289	3.31E-07	1.55E-05
SCE1572_6003	-3.809273392	-13.0495	3.34E-07	1.56E-05
SCE1572_9591	-3.727409634	-11.0434	3.55E-07	1.65E-05
SCE1572_6044	-4.653893124	-16.1949	3.85E-07	1.78E-05
SCE1572_10488	-3.701225069	-10.3519	3.88E-07	1.79E-05
SCE1572_4041	-3.699020397	-10.3023	3.93E-07	1.81E-05
SCE1572_9100	-3.773923828	-12.728	4.03E-07	1.84E-05
SCE1572_10976	35.38729351	-32.3224	4.03E-07	1.84E-05
SCE1572_9342	-3.750517353	-12.726	4.26E-07	1.93E-05
SCE1572_3306	-3.828057265	-13.5083	4.32E-07	1.95E-05
SCE1572_2533	-4.134921487	-15.0918	4.36E-07	1.95E-05
SCE1572_977	-3.694114559	-11.1202	4.38E-07	1.95E-05
SCE1572_2320	-3.759301793	-12.7353	4.38E-07	1.95E-05
SCE1572_6366	-3.806460762	-13.8113	4.78E-07	2.12E-05
SCE1572_425	-3.742729206	-12.8285	4.96E-07	2.19E-05

SCE1572_7815	-3.638906867	-7.7428	5.10E-07	2.24E-05
SCE1572_5292	-3.850207413	-14.1187	5.31E-07	2.32E-05
SCE1572_4904	-3.80249298	-13.7841	5.32E-07	2.32E-05
SCE1572_4204	-4.150569092	-15.4466	5.42E-07	2.36E-05
SCE1572_8918	-3.888094848	-14.4783	5.50E-07	2.38E-05
SCE1572_6418	-3.657615847	-11.366	5.58E-07	2.41E-05
SCE1572_7228	-3.695679516	-12.611	5.63E-07	2.42E-05
SCE1572_9719	-3.800676555	-13.9694	5.84E-07	2.50E-05
SCE1572_2354	-3.712437472	-13.0467	5.88E-07	2.50E-05
SCE1572_6312	-3.793755	-13.7599	5.89E-07	2.50E-05
SCE1572_10322	-3.900780107	-14.3788	5.96E-07	2.52E-05
SCE1572_5170	-3.64239159	-11.3034	6.07E-07	2.55E-05
SCE1572_3138	-3.718561829	-13.2411	6.22E-07	2.61E-05
SCE1572_11374	-3.643314194	-11.7933	6.41E-07	2.68E-05
SCE1572_7404	-4.132717216	-15.356	6.51E-07	2.70E-05
SCE1572_2963	-3.63936398	-11.7546	6.56E-07	2.71E-05
SCE1572_9474	-3.639155272	-11.7481	6.69E-07	2.76E-05
SCE1572_5105	-3.608426902	-10.6302	6.79E-07	2.79E-05
SCE1572_3219	-3.668166358	-12.5409	6.81E-07	2.79E-05
SCE1572_2351	-3.644288547	-12.5071	7.28E-07	2.97E-05
SCE1572_1266	-3.580717062	-10.0192	7.69E-07	3.12E-05
SCE1572_1287	-3.757923349	-13.8653	7.73E-07	3.13E-05
SCE1572_3330	-3.794985443	-14.2618	7.87E-07	3.17E-05
SCE1572_9645	-3.767849313	-14.053	7.98E-07	3.20E-05
SCE1572_5834	-4.330317176	-15.9941	8.03E-07	3.21E-05
SCE1572_10988	-3.722084896	-13.5613	8.15E-07	3.25E-05
SCE1572_3336	-3.636707951	-12.6282	8.28E-07	3.29E-05
SCE1572_8553	-3.706562192	-13.8612	8.75E-07	3.46E-05
SCE1572_1150	-3.560236334	-10.5267	9.12E-07	3.58E-05
SCE1572_9717	-3.590463303	-11.8901	9.13E-07	3.58E-05
SCE1572_4940	-3.566468288	-11.5236	9.54E-07	3.73E-05
SCE1572_6822	-3.579293541	-12.1732	9.99E-07	3.88E-05
SCE1572_6970	-4.139319951	-15.9376	1.00E-06	3.88E-05
SCE1572_2677	-4.030415388	-15.6136	1.06E-06	4.08E-05
SCE1572_1611	-4.030415388	-15.6136	1.06E-06	4.08E-05
SCE1572_11597	-3.565870846	-12.2334	1.09E-06	4.17E-05
SCE1572_4915	-3.545685148	-11.6495	1.09E-06	4.17E-05
SCE1572_7587	-3.536306317	-11.2168	1.11E-06	4.21E-05
SCE1572_3608	-3.544001666	-11.737	1.12E-06	4.24E-05
SCE1572_9418	-3.535788179	-11.4792	1.13E-06	4.29E-05
SCE1572_10045	-3.521054189	-10.9212	1.18E-06	4.43E-05
SCE1572_10688	-3.562324686	-12.4085	1.21E-06	4.54E-05
SCE1572_5654	-3.525976147	-11.5273	1.22E-06	4.55E-05
SCE1572_2191	-3.516778726	-11.2265	1.24E-06	4.62E-05
SCE1572_5477	-3.621522255	-13.3687	1.26E-06	4.70E-05
SCE1572_11118	-3.50985592	-11.1438	1.28E-06	4.76E-05
SCE1572_4244	-3.52053694	-11.5689	1.29E-06	4.78E-05
SCE1572_11052	-3.533051072	-12.1448	1.31E-06	4.80E-05

SCE1572_3866	-3.54972465	-12.4148	1.31E-06	4.80E-05
SCE1572_11598	-3.564528747	-13.0391	1.34E-06	4.87E-05
SCE1572_10272	-3.549669143	-12.7215	1.34E-06	4.87E-05
SCE1572_6476	-3.511940456	-11.7531	1.35E-06	4.88E-05
SCE1572_2834	-3.485940471	-10.0145	1.36E-06	4.92E-05
SCE1572_9592	-3.622400114	-13.6354	1.39E-06	5.00E-05
SCE1572_2347	-3.467851165	-8.26044	1.42E-06	5.08E-05
SCE1572_188	-3.643392264	-13.8351	1.43E-06	5.12E-05
SCE1572_5805	-3.690962379	-14.1618	1.45E-06	5.16E-05
SCE1572_2036	-3.481990473	-10.7585	1.46E-06	5.20E-05
SCE1572_3263	-3.476051028	-10.3456	1.47E-06	5.22E-05
SCE1572_3207	-3.541705976	-13.0506	1.51E-06	5.34E-05
SCE1572_3370	-3.963301192	-15.6471	1.54E-06	5.43E-05
SCE1572_2043	-3.665702426	-14.3671	1.56E-06	5.48E-05
SCE1572_7425	-4.059919382	-15.8398	1.62E-06	5.65E-05
SCE1572_3817	-3.535156704	-13.1354	1.68E-06	5.84E-05
SCE1572_8824	-3.818094406	-14.9105	1.68E-06	5.84E-05
SCE1572_3203	-3.579322022	-13.5853	1.72E-06	5.94E-05
SCE1572_5652	-3.518516349	-13.0621	1.73E-06	5.98E-05
SCE1572_8656	-3.490182829	-12.4344	1.74E-06	5.98E-05
SCE1572_7237	-3.449066402	-10.8398	1.78E-06	6.10E-05
SCE1572_4120	-3.43828519	-10.1985	1.80E-06	6.12E-05
SCE1572_6310	-3.576608318	-13.8405	1.80E-06	6.12E-05
SCE1572_3911	-3.471563102	-12.2011	1.88E-06	6.39E-05
SCE1572_4976	-3.436407747	-10.5886	1.89E-06	6.39E-05
SCE1572_5093	-3.572593022	-13.7355	1.92E-06	6.47E-05
SCE1572_10863	-3.547941122	-13.4055	1.95E-06	6.53E-05
SCE1572_9162	4.847517586	-16.634	1.95E-06	6.53E-05
SCE1572_3271	-3.443263147	-11.636	1.96E-06	6.55E-05
SCE1572_6365	-3.48232027	-12.8332	2.01E-06	6.70E-05
SCE1572_5261	-3.521201796	-13.211	2.05E-06	6.79E-05
SCE1572_8939	-3.644881883	-14.4624	2.07E-06	6.84E-05
SCE1572_2378	-3.674779181	-14.5376	2.09E-06	6.90E-05
SCE1572_1311	-3.4290474	-11.3811	2.14E-06	7.04E-05
SCE1572_10489	-3.451332577	-12.2816	2.18E-06	7.14E-05
SCE1572_4073	-3.440339963	-12.2961	2.27E-06	7.42E-05
SCE1572_2678	-3.730855106	-15.0043	2.28E-06	7.43E-05
SCE1572_11080	-3.540884162	-13.7514	2.29E-06	7.43E-05
SCE1572_9419	-3.507863683	-13.3854	2.30E-06	7.43E-05
SCE1572_6364	-3.593351582	-14.1063	2.36E-06	7.63E-05
SCE1572_9722	-3.380397646	-8.81484	2.38E-06	7.64E-05
SCE1572_10500	-3.541821281	-13.7254	2.41E-06	7.72E-05
SCE1572_5161	-3.389326276	-10.3126	2.42E-06	7.73E-05
SCE1572_4833	-3.421068494	-11.982	2.42E-06	7.73E-05
SCE1572_9299	-3.408460195	-11.4905	2.43E-06	7.73E-05
SCE1572_11079	-3.41647382	-11.9205	2.49E-06	7.88E-05
SCE1572_8519	-3.78599666	-15.0439	2.50E-06	7.89E-05
SCE1572_7641	-3.394481883	-11.1202	2.51E-06	7.92E-05

SCE1572_7635	-3.368817795	-8.55468	2.53E-06	7.93E-05
SCE1572_9526	-3.44393165	-12.653	2.55E-06	7.93E-05
SCE1572_10309	4.80539211	-16.6551	2.55E-06	7.93E-05
SCE1572_5731	-4.129404482	-16.2645	2.55E-06	7.93E-05
SCE1572_6128	-3.38284477	-10.5397	2.57E-06	7.95E-05
SCE1572_3851	-3.593351582	-14.2106	2.57E-06	7.95E-05
SCE1572_4023	-3.390607528	-11.4099	2.60E-06	8.02E-05
SCE1572_6010	-3.446297704	-13.0061	2.62E-06	8.06E-05
SCE1572_9409	-3.382190628	-11.1705	2.72E-06	8.35E-05
SCE1572_427	-3.52776324	-13.8104	2.74E-06	8.37E-05
SCE1572_8456	-3.443278516	-13.2325	2.87E-06	8.75E-05
SCE1572_8655	-3.35659087	-10.4415	2.95E-06	8.96E-05
SCE1572_2937	-3.368031811	-11.4357	2.96E-06	8.96E-05
SCE1572_7260	-3.370201642	-11.6554	3.01E-06	9.09E-05
SCE1572_475	-3.403873783	-12.7569	3.11E-06	9.39E-05
SCE1572_8541	-3.749470784	-15.0621	3.13E-06	9.40E-05
SCE1572_1799	-3.513784325	-14.2148	3.28E-06	9.83E-05
SCE1572_91	-3.347559427	-11.2208	3.36E-06	0.0001
SCE1572_1389	-3.522962254	-14.1754	3.37E-06	0.0001
SCE1572_3151	-3.522648591	-14.0443	3.38E-06	0.0001
SCE1572_4798	-3.50632852	-14.2185	3.47E-06	0.000102
SCE1572_1396	-3.584013717	-14.583	3.47E-06	0.000102
SCE1572_10362	-3.392125973	-12.9052	3.52E-06	0.000103
SCE1572_2400	-3.3539747	-11.8384	3.52E-06	0.000103
SCE1572_1682	-3.32621596	-10.805	3.58E-06	0.000105
SCE1572_11596	-3.446865493	-13.7478	3.59E-06	0.000105
SCE1572_7613	-3.473057348	-13.9488	3.62E-06	0.000105
SCE1572_9721	-3.364386507	-12.3802	3.62E-06	0.000105
SCE1572_5120	-3.334087522	-11.3357	3.69E-06	0.000107
SCE1572_6605	-3.441348488	-13.7505	3.74E-06	0.000108
SCE1572_4973	-3.306935449	-9.79462	3.80E-06	0.000109
SCE1572_5736	-3.415564463	-13.5129	3.81E-06	0.000109
SCE1572_1349	-3.903691703	-16.0554	3.81E-06	0.000109
SCE1572_2022	-3.303618855	-10.2077	3.93E-06	0.000112
SCE1572_2110	-3.388341239	-13.3682	4.01E-06	0.000114
SCE1572_3529	-3.307993455	-10.9992	4.04E-06	0.000115
SCE1572_5032	-3.323149762	-11.8474	4.05E-06	0.000115
SCE1572_5073	-3.360988872	-12.7298	4.11E-06	0.000116
SCE1572_1978	-3.419067041	-13.7617	4.23E-06	0.000119
SCE1572_5359	-3.42273281	-13.9174	4.34E-06	0.000122
SCE1572_2907	-3.375324353	-13.3748	4.35E-06	0.000122
SCE1572_7631	-3.422203439	-13.7355	4.38E-06	0.000123
SCE1572_5235	-3.347998012	-12.5999	4.39E-06	0.000123
SCE1572_2335	-3.355618614	-12.951	4.52E-06	0.000126
SCE1572_7962	-3.303272353	-11.8702	4.61E-06	0.000128
SCE1572_9094	-3.341244937	-12.7397	4.61E-06	0.000128
SCE1572_5038	-3.311755581	-12.0734	4.66E-06	0.000129
SCE1572_2684	-3.316067402	-12.22	4.76E-06	0.000131

SCE1572_1888	-3.514280011	-14.6652	4.82E-06	0.000132
SCE1572_2676	-3.538210028	-14.5601	4.86E-06	0.000133
SCE1572_7678	-3.582211024	-14.8377	4.96E-06	0.000136
SCE1572_1636	-3.496843601	-14.4933	5.13E-06	0.00014
SCE1572_9333	-3.473057348	-14.2707	5.15E-06	0.00014
SCE1572_4774	34.91567248	-32.5582	5.15E-06	0.00014
SCE1572_701	-3.335255099	-12.9473	5.26E-06	0.000142
SCE1572_11259	-3.285459547	-12.2027	5.39E-06	0.000145
SCE1572_2770	-3.45905049	-14.4298	5.56E-06	0.000149
SCE1572_3383	-3.315366835	-13.0716	5.56E-06	0.000149
SCE1572_630	-3.306418908	-12.8686	5.69E-06	0.000152
SCE1572_6901	-3.345424068	-13.4086	5.73E-06	0.000153
SCE1572_469	-3.271423487	-12.3359	5.83E-06	0.000155
SCE1572_9341	-3.285124398	-12.6532	5.89E-06	0.000156
SCE1572_8462	-3.320182799	-13.3472	5.89E-06	0.000156
SCE1572_8514	34.88042843	-32.5758	6.00E-06	0.000158
SCE1572_1314	-3.251615006	-11.6001	6.01E-06	0.000158
SCE1572_5888	-3.836208106	-15.8261	6.05E-06	0.000159
SCE1572_6781	-3.432415364	-14.4431	6.33E-06	0.000166
SCE1572_10462	-3.257484296	-12.4151	6.48E-06	0.000169
SCE1572_1297	-3.37451698	-13.8871	6.53E-06	0.00017
SCE1572_4916	-3.25393606	-12.3272	6.77E-06	0.000176
SCE1572_9926	-3.318116723	-13.4222	6.77E-06	0.000176
SCE1572_2664	-3.220897784	-11.13	6.81E-06	0.000176
SCE1572_607	-3.394805903	-14.2395	6.93E-06	0.000179
SCE1572_11153	-3.221448845	-11.7129	7.09E-06	0.000183
SCE1572_2801	-3.251103503	-12.5438	7.13E-06	0.000183
SCE1572_3991	-3.194968597	-9.97183	7.25E-06	0.000186
SCE1572_1372	-3.332535001	-13.9625	7.28E-06	0.000186
SCE1572_6008	-3.313243663	-13.5851	7.59E-06	0.000194
SCE1572_7632	-3.177310211	-8.91003	7.66E-06	0.000195
SCE1572_10749	-3.232691093	-12.4745	7.69E-06	0.000195
SCE1572_7438	-3.195671265	-11.2926	7.82E-06	0.000198
SCE1572_5252	-3.26777571	-13.066	7.90E-06	0.0002
SCE1572_2104	-3.207442861	-11.9507	8.10E-06	0.000204
SCE1572_5869	-3.363345976	-14.3996	8.25E-06	0.000208
SCE1572_9357	-3.188961327	-11.6106	8.33E-06	0.000209
SCE1572_1310	-3.209238656	-12.4027	8.75E-06	0.000219
SCE1572_7634	-3.187177358	-11.8408	8.82E-06	0.00022
SCE1572_9331	-3.172917942	-11.192	8.82E-06	0.00022
SCE1572_6047	-3.915279677	-16.3716	8.88E-06	0.000221
SCE1572_8101	-3.183878969	-11.6287	8.95E-06	0.000222
SCE1572_3789	-3.148909444	-9.50682	9.29E-06	0.00023
SCE1572_8015	-3.653893124	-15.6949	9.52E-06	0.000235
SCE1572_10357	-3.247175941	-13.4768	9.56E-06	0.000236
SCE1572_3327	-3.175318719	-11.9278	9.73E-06	0.000239
SCE1572_3331	-3.330317176	-14.2717	9.84E-06	0.000241
SCE1572_7957	-3.143680448	-10.6967	1.02E-05	0.000248

SCE1572_11035	-3.399179034	-14.8747	1.02E-05	0.000248
SCE1572_10169	-3.218956067	-13.0055	1.02E-05	0.000248
SCE1572_9999	-3.284970019	-13.9588	1.02E-05	0.000248
SCE1572_4817	-3.179472409	-12.3644	1.03E-05	0.00025
SCE1572_9555	-3.248703411	-13.7048	1.07E-05	0.000258
SCE1572_5769	-3.127166878	-10.2899	1.07E-05	0.000259
SCE1572_623	-3.117971177	-9.39518	1.10E-05	0.000265
SCE1572_8937	-3.110811798	-8.14125	1.11E-05	0.000266
SCE1572_1889	-3.146430022	-11.8252	1.11E-05	0.000267
SCE1572_561	-3.147095352	-11.9875	1.12E-05	0.000267
SCE1572_10764	34.75294298	-32.6396	1.14E-05	0.000272
SCE1572_9360	-3.205535551	-13.3341	1.17E-05	0.000279
SCE1572_6399	-3.164508283	-12.5473	1.19E-05	0.000283
SCE1572_7812	-3.140084061	-12.2593	1.20E-05	0.000284
SCE1572_2905	-3.238256623	-13.9287	1.20E-05	0.000284
SCE1572_10280	-3.187712781	-13.0629	1.22E-05	0.000288
SCE1572_8786	-3.315817606	-14.3494	1.23E-05	0.000289
SCE1572_11228	-3.448301249	-15.0815	1.26E-05	0.000296
SCE1572_9343	-3.122826995	-11.8913	1.27E-05	0.000297
SCE1572_10883	-3.466439469	-15.3481	1.27E-05	0.000297
SCE1572_464	-3.362026036	-14.8933	1.27E-05	0.000297
SCE1572_5876	-3.136144628	-12.4213	1.29E-05	0.0003
SCE1572_3781	-3.135361937	-12.5327	1.29E-05	0.0003
SCE1572_5999	-3.198572922	-13.6855	1.30E-05	0.000302
SCE1572_6059	-3.093269152	-10.9153	1.34E-05	0.000308
SCE1572_2617	-3.23225289	-13.9057	1.34E-05	0.000308
SCE1572_5179	-4.018657417	-16.5125	1.35E-05	0.00031
SCE1572_3815	-3.080855226	-9.57203	1.36E-05	0.000312
SCE1572_5110	-3.18196186	-13.4344	1.43E-05	0.000327
SCE1572_6835	-3.313243663	-14.5851	1.46E-05	0.000334
SCE1572_3264	-3.159876834	-13.3569	1.50E-05	0.000342
SCE1572_579	-3.396954369	-15.1713	1.51E-05	0.000342
SCE1572_371	-3.307047397	-14.7688	1.51E-05	0.000342
SCE1572_10653	-3.063540533	-9.96612	1.51E-05	0.000342
SCE1572_1763	3.798548913	-15.7939	1.52E-05	0.000344
SCE1572_6969	-3.169543873	-13.7	1.54E-05	0.000347
SCE1572_8236	-3.182372299	-13.5883	1.54E-05	0.000348
SCE1572_9294	-3.14441821	-13.2813	1.55E-05	0.000348
SCE1572_3674	-3.091664126	-12.0899	1.56E-05	0.000349
SCE1572_5031	-3.063690784	-10.6604	1.56E-05	0.000349
SCE1572_4789	-3.12086381	-12.9744	1.57E-05	0.000351
SCE1572_9840	34.68685379	-32.6726	1.59E-05	0.000354
SCE1572_10074	34.66413371	-32.684	1.59E-05	0.000354
SCE1572_4151	-3.172290295	-13.7431	1.63E-05	0.000361
SCE1572_2206	-3.112725741	-13.018	1.64E-05	0.000364
SCE1572_2269	-3.082520811	-12.0066	1.67E-05	0.000369
SCE1572_5733	-3.068783679	-11.7233	1.68E-05	0.000371
SCE1572_7079	-3.551531406	-15.7461	1.70E-05	0.000374

SCE1572_5777	-3.243409111	-14.4221	1.73E-05	0.000381
SCE1572_2202	-3.160002204	-13.5397	1.75E-05	0.000383
SCE1572_2374	-3.092230209	-12.8523	1.77E-05	0.000386
SCE1572_20	-3.100693186	-12.9715	1.81E-05	0.000396
SCE1572_11385	-3.029780339	-10.3165	1.85E-05	0.000403
SCE1572_4744	-3.756850314	-16.1288	1.87E-05	0.000405
SCE1572_8555	-3.61934679	-16.0601	1.87E-05	0.000405
SCE1572_7338	-3.128819092	-13.3554	1.87E-05	0.000405
SCE1572_1358	-3.024450543	-10.4485	1.91E-05	0.000413
SCE1572_2201	-3.067760744	-12.3944	1.92E-05	0.000414
SCE1572_1681	-3.210022942	-14.3319	1.93E-05	0.000415
SCE1572_11075	-3.141586049	-13.8277	1.98E-05	0.000424
SCE1572_7368	-3.016241131	-10.538	2.03E-05	0.000434
SCE1572_9024	-3.199072643	-14.2061	2.06E-05	0.000441
SCE1572_9499	-3.022023631	-11.4216	2.11E-05	0.00045
SCE1572_11288	-3.042799112	-12.1425	2.12E-05	0.000451
SCE1572_9441	-3.080817554	-13.1306	2.13E-05	0.000452
SCE1572_3829	-3.070980039	-13.0658	2.16E-05	0.000457
SCE1572_4588	-3.103546314	-13.733	2.21E-05	0.000467
SCE1572_2277	-3.015502707	-11.7825	2.24E-05	0.000473
SCE1572_9767	-3.078778409	-13.3635	2.25E-05	0.000475
SCE1572_7588	-3.013802577	-11.767	2.27E-05	0.000477
SCE1572_8451	-3.106201105	-13.71	2.30E-05	0.000483
SCE1572_6363	-3.072877808	-13.0785	2.31E-05	0.000484
SCE1572_7589	-3.083454896	-13.328	2.36E-05	0.000493
SCE1572_4074	-2.990241735	-10.8568	2.37E-05	0.000494
SCE1572_4866	-3.204309291	-14.6827	2.39E-05	0.000497
SCE1572_7814	-2.986559716	-10.6412	2.39E-05	0.000497
SCE1572_4736	-3.024783519	-12.3656	2.45E-05	0.000508
SCE1572_8458	-2.984542339	-10.9062	2.46E-05	0.00051
SCE1572_3769	-3.098956291	-13.63	2.48E-05	0.000512
SCE1572_9404	-3.248703411	-14.7048	2.49E-05	0.000513
SCE1572_8099	-3.063181554	-13.2585	2.50E-05	0.000514
SCE1572_8422	-3.011360648	-12.2236	2.50E-05	0.000514
SCE1572_10451	-3.285229287	-14.9861	2.55E-05	0.000523
SCE1572_5087	-3.047840232	-13.1471	2.57E-05	0.000527
SCE1572_10535	-3.018442746	-12.6601	2.60E-05	0.000531
SCE1572_1966	-3.024690894	-12.7725	2.62E-05	0.000534
SCE1572_930	-3.023110587	-12.9473	2.67E-05	0.000543
SCE1572_10702	-3.473057348	-15.6858	2.67E-05	0.000543
SCE1572_906	34.56949684	-32.7313	2.69E-05	0.000545
SCE1572_10765	-3.059919382	-13.3398	2.70E-05	0.000545
SCE1572_9590	-3.004708735	-12.5322	2.73E-05	0.00055
SCE1572_9339	-2.995691191	-12.2314	2.73E-05	0.00055
SCE1572_9557	-3.09613957	-13.8985	2.76E-05	0.000555
SCE1572_10344	-2.942131556	-8.6554	2.85E-05	0.000571
SCE1572_2961	-2.979514072	-12.0779	2.95E-05	0.000591
SCE1572_10494	-2.989816975	-12.408	2.96E-05	0.000591

SCE1572_5726	-2.959890564	-11.3454	2.96E-05	0.000591
SCE1572_673	-3.003727704	-12.8731	3.04E-05	0.000606
SCE1572_9514	-2.997793966	-12.8878	3.05E-05	0.000607
SCE1572_3198	-3.451995733	-15.6963	3.09E-05	0.000614
SCE1572_9671	-3.050824348	-13.7377	3.12E-05	0.000617
SCE1572_4461	3.220429609	-14.4476	3.14E-05	0.000621
SCE1572_10063	-2.936705152	-10.8274	3.17E-05	0.000626
SCE1572_2405	-3.023764307	-13.425	3.21E-05	0.000632
SCE1572_748	-3.512431587	-16.1136	3.24E-05	0.000637
SCE1572_5027	-3.012890473	-13.2378	3.28E-05	0.000643
SCE1572_2285	-3.856385988	-16.5937	3.28E-05	0.000643
SCE1572_5135	-2.969819245	-12.4956	3.29E-05	0.000643
SCE1572_11051	-2.971863205	-12.7558	3.30E-05	0.000645
SCE1572_10400	-3.039250045	-13.6001	3.38E-05	0.000658
SCE1572_5192	-3.021445234	-13.7741	3.44E-05	0.000669
SCE1572_1926	-2.92308332	-10.985	3.49E-05	0.000677
SCE1572_6873	-3.003572065	-13.3356	3.50E-05	0.000679
SCE1572_7044	-2.908338067	-9.53074	3.53E-05	0.000682
SCE1572_4975	-2.90951981	-10.2801	3.56E-05	0.000686
SCE1572_4967	-3.058019849	-13.8933	3.57E-05	0.000687
SCE1572_11378	-3.035494311	-13.9535	3.57E-05	0.000687
SCE1572_1621	-2.96998102	-12.9616	3.67E-05	0.000705
SCE1572_618	-2.937392551	-12.2023	3.68E-05	0.000706
SCE1572_4221	-2.960560556	-12.7404	3.70E-05	0.000708
SCE1572_2409	-2.997248524	-13.5452	3.72E-05	0.000711
SCE1572_2744	-3.082978398	-14.173	3.74E-05	0.000713
SCE1572_707	-2.952712105	-12.7236	3.76E-05	0.000715
SCE1572_9421	-2.930386569	-12.0311	3.79E-05	0.00072
SCE1572_10286	-2.917253118	-11.8317	3.86E-05	0.000732
SCE1572_9041	-2.895965996	-10.3209	3.87E-05	0.000732
SCE1572_7671	4.013978732	-16.4658	3.92E-05	0.00074
SCE1572_3236	-2.907460172	-11.666	3.95E-05	0.000744
SCE1572_3513	-2.956186595	-12.9322	3.96E-05	0.000746
SCE1572_6981	3.468116051	-15.5164	4.01E-05	0.000753
SCE1572_1309	-2.876913691	-7.67328	4.04E-05	0.000757
SCE1572_10882	-2.924324816	-12.5542	4.11E-05	0.00077
SCE1572_11569	-3.252314664	-15.4551	4.15E-05	0.000775
SCE1572_9478	-2.914230062	-12.0907	4.20E-05	0.000784
SCE1572_1976	-2.901565971	-11.9655	4.29E-05	0.000798
SCE1572_2633	3.711170997	-16.0549	4.34E-05	0.000806
SCE1572_6379	34.44174129	-32.7952	4.69E-05	0.00087
SCE1572_348	-3.008389081	-13.9921	4.75E-05	0.000879
SCE1572_3415	-2.978376512	-13.7525	4.80E-05	0.000886
SCE1572_7453	-2.870646681	-11.6555	4.84E-05	0.000892
SCE1572_10033	-2.881586542	-12.0619	4.86E-05	0.000895
SCE1572_10907	-2.865811001	-11.4206	4.88E-05	0.000898
SCE1572_9720	-2.962319433	-13.7188	4.92E-05	0.000904
SCE1572_11292	-2.869744371	-12.1461	5.15E-05	0.000944

SCE1572_3376	-3.078778409	-14.8829	5.20E-05	0.000951
SCE1572_8785	-3.011903565	-14.4657	5.22E-05	0.000952
SCE1572_6331	-2.953527146	-13.7863	5.23E-05	0.000954
SCE1572_11077	-3.426701713	-16.1564	5.25E-05	0.000955
SCE1572_1801	-3.104313501	-14.777	5.27E-05	0.000958
SCE1572_1511	-2.903307035	-12.9587	5.29E-05	0.000959
SCE1572_10111	-2.862286589	-11.9673	5.30E-05	0.00096
SCE1572_4486	-2.999343942	-14.2446	5.49E-05	0.000993
SCE1572_4787	-2.84105684	-11.2292	5.51E-05	0.000994
SCE1572_8657	-3.773923828	-16.8573	5.57E-05	0.001003
SCE1572_10046	-2.843712158	-11.4853	5.58E-05	0.001004
SCE1572_9971	-2.890069114	-12.8955	5.59E-05	0.001004
SCE1572_6133	-2.856385988	-12.1868	5.60E-05	0.001004
SCE1572_8238	-2.881665396	-12.844	5.67E-05	0.001014
SCE1572_11593	-2.90267964	-13.3543	5.69E-05	0.001014
SCE1572_8452	-3.093425185	-14.9751	5.69E-05	0.001014
SCE1572_117	-2.945511878	-13.769	5.75E-05	0.001022
SCE1572_426	-3.091602449	-14.7834	5.75E-05	0.001022
SCE1572_10082	-2.912529066	-13.4806	5.78E-05	0.001023
SCE1572_5037	-2.851987524	-12.1284	5.79E-05	0.001023
SCE1572_3462	-5.441348488	-18.6085	5.79E-05	0.001023
SCE1572_4062	-2.88779894	-13.1574	5.80E-05	0.001023
SCE1572_1345	-2.868509547	-12.5912	5.88E-05	0.001032
SCE1572_2665	-2.921839049	-13.5645	5.89E-05	0.001032
SCE1572_7573	-2.903691703	-13.5554	5.89E-05	0.001032
SCE1572_4840	-2.898977864	-13.404	5.89E-05	0.001032
SCE1572_6719	-2.88495514	-13.2429	5.98E-05	0.001047
SCE1572_2431	-2.91600997	-13.5674	6.00E-05	0.001049
SCE1572_2205	-3.318729202	-15.7629	6.12E-05	0.001067
SCE1572_5002	-2.831419072	-11.826	6.13E-05	0.001067
SCE1572_5768	-2.810343619	-10.9017	6.38E-05	0.001109
SCE1572_473	-2.965320359	-14.1741	6.40E-05	0.001111
SCE1572_2109	-2.987630521	-14.4431	6.48E-05	0.001122
SCE1572_4841	-2.832138442	-12.1318	6.53E-05	0.00113
SCE1572_407	-2.916092234	-13.7214	6.57E-05	0.001135
SCE1572_6846	-2.928535774	-14.1645	6.60E-05	0.001136
SCE1572_531	-2.880471606	-13.2596	6.60E-05	0.001136
SCE1572_8410	-2.836328335	-12.5102	6.75E-05	0.00116
SCE1572_1841	-2.872505653	-13.4172	6.81E-05	0.001168
SCE1572_2406	-2.892455242	-13.5792	6.92E-05	0.001185
SCE1572_4049	-2.90117391	-13.6692	6.98E-05	0.001193
SCE1572_9661	-2.793246399	-11.2996	7.02E-05	0.001199
SCE1572_2238	-2.793989451	-11.37	7.09E-05	0.001207
SCE1572_10984	-2.796207992	-11.4433	7.09E-05	0.001207
SCE1572_1680	-2.826012339	-12.5943	7.17E-05	0.001218
SCE1572_2683	-2.839412909	-13.0766	7.21E-05	0.001223
SCE1572_1906	-2.866690996	-13.3567	7.25E-05	0.001228
SCE1572_5165	-2.796117235	-11.8174	7.32E-05	0.001238

SCE1572_158	-2.794772797	-11.7272	7.43E-05	0.001253
SCE1572_3181	-2.785064385	-11.3405	7.44E-05	0.001253
SCE1572_5909	-2.888518609	-13.6755	7.44E-05	0.001253
SCE1572_3345	-2.770477662	-10.4622	7.60E-05	0.001276
SCE1572_10655	-2.954233311	-14.6042	7.64E-05	0.001281
SCE1572_9716	-3.210022942	-15.5543	7.65E-05	0.001281
SCE1572_558	-2.870885557	-13.8494	7.67E-05	0.001282
SCE1572_2404	-2.894656005	-13.9277	7.76E-05	0.001295
SCE1572_11036	-3.358886328	-16.0648	7.84E-05	0.001308
SCE1572_5030	-2.763950358	-10.5678	7.99E-05	0.00133
SCE1572_10059	-2.751605843	-9.46455	8.05E-05	0.001335
SCE1572_11280	-2.827239642	-13.2717	8.05E-05	0.001335
SCE1572_9743	-2.769201417	-11.6047	8.19E-05	0.001357
SCE1572_10664	-2.869325043	-13.6851	8.30E-05	0.001372
SCE1572_5703	-2.820651776	-13.1507	8.34E-05	0.001377
SCE1572_2005	-2.81721539	-13.1132	8.39E-05	0.001383
SCE1572_4813	-2.814866718	-13.3672	8.66E-05	0.001425
SCE1572_11568	-2.760745983	-11.6405	8.71E-05	0.001432
SCE1572_5103	-2.781858009	-12.3533	8.81E-05	0.001444
SCE1572_10058	-2.76511793	-11.9187	8.81E-05	0.001444
SCE1572_5080	-2.746761497	-11.1388	8.94E-05	0.001462
SCE1572_3089	-2.999343942	-14.8295	8.99E-05	0.001468
SCE1572_3317	-2.754867228	-11.9294	9.20E-05	0.0015
SCE1572_10270	-2.804297477	-13.3725	9.30E-05	0.001512
SCE1572_10154	-2.77248473	-12.4754	9.30E-05	0.001512
SCE1572_7912	3.055647911	-14.7226	9.36E-05	0.001519
SCE1572_4605	-2.759923148	-12.2982	9.37E-05	0.001519
SCE1572_2399	-2.734254298	-10.9934	9.39E-05	0.00152
SCE1572_6922	-3.089585164	-15.5365	9.46E-05	0.001528
SCE1572_10065	-2.767623839	-12.6055	9.51E-05	0.001535
SCE1572_3329	-2.738373075	-11.5171	9.68E-05	0.00156
SCE1572_5122	-3.222513887	-15.8111	9.91E-05	0.001593
SCE1572_4990	-2.765848441	-12.8482	0.000101	0.001624
SCE1572_2204	-2.823065915	-13.7278	0.000102	0.00164
SCE1572_4052	-2.773923828	-13.1874	0.000103	0.001647
SCE1572_2845	34.29211105	-32.87	0.000103	0.001653
SCE1572_10733	-3.30117083	-16.0937	0.000104	0.001664
SCE1572_4870	-3.185693613	-16.0359	0.000104	0.001664
SCE1572_3896	-2.731335444	-11.8708	0.000106	0.00168
SCE1572_2474	-2.777674012	-13.1202	0.000106	0.001685
SCE1572_10473	-2.737361885	-12.4607	0.000107	0.001693
SCE1572_1302	-2.959479481	-15.0946	0.000108	0.001703
SCE1572_3924	-2.856385988	-14.1461	0.000108	0.001703
SCE1572_1062	3.026257062	-14.6378	0.00011	0.00174
SCE1572_271	-2.703572711	-11.0384	0.000112	0.001768
SCE1572_6854	4.068426516	-17.0236	0.000113	0.001772
SCE1572_6847	4.068426516	-17.0236	0.000113	0.001772
SCE1572_6848	4.068426516	-17.0236	0.000113	0.001772

SCE1572_3749	-2.9343885	-14.9551	0.000113	0.001779
SCE1572_65	-2.830390779	-14.1067	0.000116	0.00181
SCE1572_855	-2.699017158	-11.2349	0.000116	0.00181
SCE1572_8479	-2.761526801	-13.1671	0.000117	0.001819
SCE1572_11137	-2.814321928	-14.0641	0.000117	0.001825
SCE1572_2275	-2.704746339	-11.7772	0.000117	0.001825
SCE1572_10061	-2.673926633	-9.23235	0.000121	0.001877
SCE1572_9498	-2.697155587	-12.0033	0.000125	0.001946
SCE1572_5743	-2.951384933	-14.9911	0.000126	0.001946
SCE1572_10016	-3.271423487	-16.1085	0.000127	0.001958
SCE1572_1980	-2.684344045	-11.5734	0.000127	0.001958
SCE1572_7716	34.24144264	-32.8953	0.000127	0.001958
SCE1572_9614	34.21041575	-32.9108	0.000127	0.001958
SCE1572_5531	34.21041575	-32.9108	0.000127	0.001958
SCE1572_7735	-2.681432147	-11.1212	0.000128	0.001959
SCE1572_10151	-2.801042954	-13.9745	0.000128	0.001959
SCE1572_5078	-2.742729206	-13.1765	0.000128	0.001962
SCE1572_3932	-2.661958221	-9.3716	0.000129	0.00197
SCE1572_6131	-2.771688823	-13.6954	0.000131	0.001992
SCE1572_5006	-2.699146246	-12.4561	0.000131	0.001992
SCE1572_7583	-2.675088588	-11.355	0.000132	0.002013
SCE1572_3514	-2.718882464	-12.9254	0.000134	0.002041
SCE1572_10356	-2.767576721	-13.8793	0.000138	0.002092
SCE1572_11163	-2.771269374	-13.9436	0.00014	0.002115
SCE1572_5926	2.768800991	-13.2811	0.000144	0.002179
SCE1572_635	-2.66352951	-11.6531	0.000144	0.002179
SCE1572_7255	-2.654334412	-11.3474	0.000146	0.002205
SCE1572_1670	-2.656616475	-11.5579	0.000149	0.002246
SCE1572_3270	-2.782385406	-14.0311	0.000149	0.002247
SCE1572_2319	-2.740327858	-13.8505	0.00015	0.002247
SCE1572_2290	-2.653709555	-11.667	0.000153	0.0023
SCE1572_2883	-2.756850314	-13.9284	0.000156	0.002336
SCE1572_3208	-2.698321142	-13.2521	0.000157	0.002342
SCE1572_2203	-2.727488255	-13.6801	0.000157	0.002346
SCE1572_2278	-2.732754639	-13.8543	0.000157	0.002346
SCE1572_2969	3.119333506	-15.359	0.000157	0.002346
SCE1572_7992	3.323523102	-15.8111	0.000158	0.00235
SCE1572_10640	-2.630001183	-10.8268	0.000161	0.00239
SCE1572_2263	-2.651298931	-12.0728	0.000162	0.002405
SCE1572_7802	-2.723550305	-13.7975	0.000162	0.002405
SCE1572_1583	3.383928342	-16.0439	0.000164	0.002428
SCE1572_3199	-2.662892515	-12.6579	0.000165	0.002434
SCE1572_8542	-2.702285953	-13.3056	0.000167	0.002462
SCE1572_11373	-2.642834054	-12.1313	0.000168	0.002469
SCE1572_9663	-2.738549497	-13.8725	0.00017	0.002495
SCE1572_9308	-2.608607076	-9.3649	0.00017	0.002498
SCE1572_7954	3.456496968	-16.3295	0.000171	0.002506
SCE1572_8670	-3.094545725	-16.0815	0.000171	0.002506

SCE1572_6737	-2.654624899	-12.4396	0.000171	0.002506
SCE1572_11505	-2.644775084	-12.1181	0.000172	0.00251
SCE1572_10081	-2.612026446	-10.25	0.000172	0.002516
SCE1572_9694	-2.603861472	-8.66862	0.000173	0.002519
SCE1572_2040	-2.669587168	-12.9832	0.000173	0.002523
SCE1572_6809	-2.608458474	-10.0766	0.000175	0.002545
SCE1572_8518	-2.806200231	-14.6407	0.000177	0.002565
SCE1572_7455	-2.709996501	-13.7263	0.00018	0.002602
SCE1572_6159	4.750944326	-17.6823	0.000181	0.002613
SCE1572_11594	-2.649289754	-12.5783	0.000181	0.002614
SCE1572_11267	-2.609441284	-11.283	0.000183	0.002639
SCE1572_8330	-2.651628415	-12.97	0.000184	0.002645
SCE1572_3433	-2.778383476	-14.6921	0.000184	0.002647
SCE1572_1	-2.622735389	-12.093	0.000184	0.002651
SCE1572_10654	-2.686460986	-13.543	0.000188	0.002703
SCE1572_7822	-2.633993566	-12.45	0.000191	0.002732
SCE1572_11395	-2.607026519	-11.6334	0.000192	0.002747
SCE1572_10355	-2.745354675	-14.2016	0.000193	0.002756
SCE1572_5861	34.14628541	-32.9429	0.000195	0.002781
SCE1572_1653	34.14628541	-32.9429	0.000195	0.002781
SCE1572_2461	34.13542442	-32.9483	0.000195	0.002781
SCE1572_9362	-2.623583783	-12.608	0.000197	0.002804
SCE1572_11546	3.171494536	-15.7694	0.000198	0.002816
SCE1572_11382	-2.661569811	-13.4286	0.0002	0.002829
SCE1572_10862	-2.648144055	-13.0509	0.0002	0.002829
SCE1572_4378	3.129455949	-15.493	0.0002	0.002829
SCE1572_4842	-2.917786532	-15.3467	0.0002	0.002829
SCE1572_11160	-2.780437135	-14.6536	0.000201	0.002829
SCE1572_5767	-2.607762025	-12.0085	0.000203	0.002858
SCE1572_5653	-2.616920053	-12.6113	0.000204	0.002867
SCE1572_1095	-2.751416428	-14.7056	0.00021	0.002947
SCE1572_1341	-2.644375585	-13.3632	0.00021	0.00295
SCE1572_11575	-2.691531976	-13.8337	0.000213	0.002978
SCE1572_8049	3.272897029	-15.8364	0.000214	0.00299
SCE1572_4606	-2.949495392	-15.5325	0.000214	0.00299
SCE1572_7334	-2.585030985	-11.7467	0.000214	0.00299
SCE1572_8998	-2.594578885	-12.1554	0.000215	0.00299
SCE1572_2532	-2.702473304	-14.1706	0.000218	0.003036
SCE1572_342	-2.594800799	-12.3952	0.000224	0.003109
SCE1572_10633	-2.606665935	-12.7875	0.000224	0.003113
SCE1572_157	-2.589533243	-12.2079	0.000225	0.003115
SCE1572_860	-2.666561429	-13.8061	0.000228	0.003161
SCE1572_10217	-2.704382894	-14.3332	0.000229	0.003165
SCE1572_7907	-2.575076524	-11.8818	0.000232	0.003201
SCE1572_9470	-2.64993511	-13.5948	0.000238	0.003274
SCE1572_9040	-2.686460986	-14.0087	0.00024	0.003307
SCE1572_4043	-2.584399981	-12.4371	0.000241	0.003307
SCE1572_4519	-2.648392945	-13.8351	0.000241	0.003309

SCE1572_392	3.001595007	-15.3871	0.000242	0.003312
SCE1572_6028	-2.837020663	-15.2668	0.000242	0.003312
SCE1572_6682	-2.706809632	-14.5164	0.000243	0.003325
SCE1572_4256	-2.602937631	-13.1208	0.000245	0.003347
SCE1572_4148	-2.766188179	-14.9017	0.000245	0.003347
SCE1572_6406	-2.856385988	-15.4416	0.000246	0.003351
SCE1572_5147	4.682772823	-17.7164	0.000248	0.003372
SCE1572_612	-2.669972864	-14.2129	0.000249	0.003383
SCE1572_5906	-2.553458855	-11.7857	0.000254	0.003451
SCE1572_5745	-3.036958233	-15.9038	0.000255	0.003456
SCE1572_4596	-2.55940425	-12.0383	0.000255	0.003458
SCE1572_8306	3.898501514	-17.1085	0.000256	0.003459
SCE1572_11380	-3.112725741	-16.1879	0.000259	0.003493
SCE1572_10639	-2.59111311	-13.0224	0.000259	0.003493
SCE1572_9668	-2.582756467	-12.6803	0.00026	0.003498
SCE1572_2419	-2.532948008	-10.5769	0.00026	0.003498
SCE1572_10107	-2.58593411	-13.1659	0.000268	0.003593
SCE1572_4943	-2.540736956	-11.555	0.000272	0.003646
SCE1572_6733	-2.615377888	-13.7361	0.000273	0.003652
SCE1572_8524	-2.563189611	-12.6047	0.000273	0.003652
SCE1572_5203	-2.66831364	-14.0882	0.000273	0.003653
SCE1572_9150	-2.682356588	-14.5286	0.000274	0.003653
SCE1572_5010	-2.553662104	-12.4157	0.000276	0.003685
SCE1572_1869	-2.637376206	-13.8608	0.000281	0.003736
SCE1572_2408	-2.550997569	-12.7408	0.00029	0.003851
SCE1572_2360	-2.700266786	-14.6937	0.000296	0.003931
SCE1572_2037	-2.669972864	-14.5348	0.000296	0.003931
SCE1572_8563	-2.643977655	-14.1999	0.000299	0.003965
SCE1572_10091	-2.526142625	-11.8862	0.000302	0.00399
SCE1572_289	2.615658627	-13.2905	0.000302	0.00399
SCE1572_6475	-2.527033685	-11.9572	0.000303	0.003995
SCE1572_2481	-2.510313955	-11.1914	0.000305	0.00401
SCE1572_9303	-2.538415906	-12.5522	0.000305	0.00401
SCE1572_7816	-2.742053312	-14.8289	0.000305	0.00401
SCE1572_251	3.347432577	-16.1576	0.000306	0.00401
SCE1572_3400	-2.541725893	-12.692	0.000306	0.00401
SCE1572_5014	-2.56350957	-13.0588	0.000306	0.004012
SCE1572_2352	-2.624095105	-13.9948	0.000307	0.004014
SCE1572_7832	-2.66374091	-14.4124	0.000307	0.004017
SCE1572_8783	-2.554816981	-13.0406	0.000309	0.004034
SCE1572_8237	-2.61934679	-14.2382	0.000313	0.004078
SCE1572_562	-2.533051072	-12.6448	0.000318	0.004141
SCE1572_2270	-2.492743472	-10.496	0.00032	0.004156
SCE1572_7221	-3.078778409	-16.2048	0.00032	0.004156
SCE1572_7501	-2.538745251	-12.9305	0.000323	0.004195
SCE1572_6479	-2.565754023	-13.4765	0.000325	0.004207
SCE1572_6792	-2.601128933	-13.8192	0.000329	0.004247
SCE1572_1364	-2.487998582	-10.476	0.000329	0.004247

SCE1572_2761	-2.536926148	-12.8709	0.000329	0.004247
SCE1572_2473	-2.528755534	-12.6639	0.000331	0.004265
SCE1572_1622	-2.655338469	-14.644	0.000332	0.004276
SCE1572_2393	-2.554532333	-13.2318	0.000336	0.004314
SCE1572_9768	-2.593351582	-14.0325	0.000338	0.00434
SCE1572_7519	-2.672521795	-14.7075	0.000338	0.00434
SCE1572_4623	-2.62031863	-14.2911	0.000339	0.004347
SCE1572_6782	-3.386900704	-17.0508	0.000343	0.004395
SCE1572_6295	-2.555216453	-13.528	0.000344	0.004402
SCE1572_2760	-2.518281149	-12.9203	0.000349	0.004457
SCE1572_8654	-2.470982274	-9.85292	0.00035	0.004461
SCE1572_6274	-2.538527557	-13.1773	0.00035	0.004461
SCE1572_11266	-2.47341988	-10.6579	0.000353	0.004492
SCE1572_7325	-2.487898365	-11.6382	0.000356	0.004521
SCE1572_1356	-2.519860517	-12.8501	0.000357	0.004522
SCE1572_7342	-2.504225691	-12.3155	0.000357	0.004522
SCE1572_7518	-2.471019986	-10.7561	0.000358	0.004531
SCE1572_878	-2.541183756	-13.3171	0.000359	0.004539
SCE1572_5230	-2.474676665	-11.0097	0.00036	0.004548
SCE1572_10832	-2.503786197	-12.3908	0.000362	0.004565
SCE1572_9662	-2.493815908	-12.2494	0.000368	0.004634
SCE1572_3844	-2.480740033	-11.934	0.000368	0.004635
SCE1572_2344	-2.507168671	-12.6747	0.000371	0.004668
SCE1572_11398	-2.492676789	-12.583	0.000373	0.004678
SCE1572_3640	-2.804855687	-15.6788	0.000374	0.004684
SCE1572_7269	-2.470796752	-11.5979	0.000377	0.004724
SCE1572_2675	-2.652513654	-14.5767	0.000378	0.004724
SCE1572_10609	-2.478519721	-11.8708	0.000379	0.004736
SCE1572_7466	-2.502749033	-12.7469	0.000388	0.004846
SCE1572_3417	-2.54151265	-13.6491	0.000401	0.005001
SCE1572_8258	-2.587897152	-14.3073	0.000403	0.005016
SCE1572_8425	-2.736091754	-15.3173	0.000404	0.005023
SCE1572_7558	4.561466527	-17.777	0.000406	0.005035
SCE1572_2011	4.561466527	-17.777	0.000406	0.005035
SCE1572_10300	-2.522648591	-13.5443	0.000407	0.005042
SCE1572_8804	-2.640657297	-14.8389	0.000408	0.005044
SCE1572_2276	-2.46264789	-12.1092	0.00041	0.005066
SCE1572_6368	-2.556304595	-13.9013	0.000414	0.005116
SCE1572_8658	-2.549408234	-13.7326	0.000418	0.005156
SCE1572_33	-2.433222598	-9.6979	0.000422	0.005195
SCE1572_1698	-2.856385988	-15.8135	0.000423	0.005197
SCE1572_6836	-2.856385988	-15.8135	0.000423	0.005197
SCE1572_3905	-2.446510194	-11.5668	0.000424	0.005197
SCE1572_9147	-2.629877458	-14.7289	0.000424	0.005197
SCE1572_10026	-2.47424037	-12.7792	0.000425	0.005199
SCE1572_4689	-2.464130986	-12.4373	0.00043	0.005264
SCE1572_7489	-3.145892605	-16.5863	0.000436	0.005331
SCE1572_4999	-2.427648984	-10.3505	0.00044	0.005369

SCE1572_8353	-2.421755124	-9.64158	0.00044	0.005369
SCE1572_11287	-2.53281004	-13.873	0.000441	0.00537
SCE1572_9667	-2.515730685	-13.679	0.000446	0.005412
SCE1572_9691	-2.515730685	-13.679	0.000446	0.005412
SCE1572_8630	-2.522231591	-13.7643	0.000456	0.005531
SCE1572_7574	-2.4705792	-12.846	0.000456	0.005531
SCE1572_3849	3.772970632	-17.1713	0.000459	0.005547
SCE1572_2811	3.750944326	-17.1823	0.000459	0.005547
SCE1572_947	2.902598919	-15.4706	0.000461	0.005568
SCE1572_568	-2.827816836	-15.8278	0.000462	0.005571
SCE1572_879	-2.450455919	-12.5115	0.000462	0.005571
SCE1572_1634	-2.426067031	-11.2582	0.000468	0.005624
SCE1572_1669	-2.89033332	-16.0767	0.000468	0.005624
SCE1572_8349	3.518653444	-16.7135	0.00047	0.005627
SCE1572_10056	-2.98191687	-16.3788	0.00047	0.005627
SCE1572_2814	-2.484904854	-13.2288	0.00047	0.005627
SCE1572_10097	-2.720730888	-15.2684	0.000472	0.005648
SCE1572_6683	-2.479316339	-13.4457	0.000476	0.005694
SCE1572_843	-2.440023091	-12.1903	0.000477	0.0057
SCE1572_5766	-2.414266581	-10.6484	0.000479	0.00571
SCE1572_964	-2.464669563	-12.868	0.000479	0.00571
SCE1572_9891	4.535931435	-17.7898	0.000481	0.005725
SCE1572_3734	2.947745033	-15.5839	0.000482	0.005726
SCE1572_4808	-2.534457893	-14.2806	0.000485	0.005756
SCE1572_2231	-2.794985443	-15.9317	0.000488	0.005775
SCE1572_6909	33.9347813	-33.0487	0.000488	0.005775
SCE1572_3711	33.89630716	-33.0679	0.000488	0.005775
SCE1572_7607	2.594190833	-14.0602	0.000489	0.005775
SCE1572_2442	-2.459868576	-13.088	0.000495	0.005843
SCE1572_6253	-2.543503032	-14.3295	0.000497	0.005864
SCE1572_9600	-2.477398149	-13.4323	0.000498	0.005868
SCE1572_7595	-2.441348488	-12.8231	0.000504	0.005929
SCE1572_5287	-2.671961417	-15.3494	0.000527	0.006195
SCE1572_2289	-2.941274885	-16.3991	0.000531	0.006231
SCE1572_9420	-2.560930104	-14.5569	0.000532	0.006235
SCE1572_8904	3.705856436	-17.2048	0.000534	0.006244
SCE1572_1215	3.705856436	-17.2048	0.000534	0.006244
SCE1572_8317	-2.602068799	-14.9838	0.000536	0.00626
SCE1572_10987	-2.537013598	-14.5061	0.000537	0.006263
SCE1572_9899	-2.427765516	-12.7403	0.000547	0.006379
SCE1572_10679	-2.39219754	-11.2232	0.000552	0.006427
SCE1572_9411	-2.422282093	-12.545	0.000553	0.006429
SCE1572_4776	-2.398472653	-12.0162	0.000559	0.006501
SCE1572_6130	-2.723935692	-15.7193	0.000566	0.006567
SCE1572_9352	-2.551531406	-14.4387	0.000566	0.006567
SCE1572_7630	-2.551531406	-14.6611	0.000569	0.006589
SCE1572_4080	-2.453173924	-13.3477	0.000569	0.006589
SCE1572_7867	-2.963301192	-16.1471	0.000576	0.006644

SCE1572_6297	-2.856385988	-16.0937	0.000576	0.006644
SCE1572_2041	-2.617873901	-14.891	0.000577	0.006644
SCE1572_7821	-2.501291029	-14.2972	0.000577	0.006644
SCE1572_2339	-2.547754715	-14.5635	0.000577	0.006644
SCE1572_4603	-2.388607026	-11.7906	0.000578	0.006647
SCE1572_7617	-2.401058767	-12.2583	0.000582	0.006685
SCE1572_5003	-2.593351582	-14.7846	0.000586	0.006724
SCE1572_9148	-2.411430966	-12.8289	0.000591	0.006769
SCE1572_6049	-2.487460413	-14.0631	0.000596	0.006816
SCE1572_10493	-2.375760147	-11.4975	0.000596	0.006816
SCE1572_6178	-2.490063774	-14.3028	0.000597	0.006821
SCE1572_8782	-2.432246281	-13.3852	0.000599	0.006836
SCE1572_7759	-2.448012219	-13.5202	0.000603	0.006869
SCE1572_3949	-2.390230917	-12.2699	0.000608	0.006922
SCE1572_424	-2.497699343	-14.3524	0.000616	0.007007
SCE1572_4051	-2.396024498	-12.5313	0.000617	0.007008
SCE1572_1738	33.85677879	-33.0877	0.000622	0.007057
SCE1572_6388	-2.504622663	-14.244	0.000623	0.007063
SCE1572_10343	-2.512431587	-14.6136	0.000624	0.007063
SCE1572_2674	-2.451995733	-13.8744	0.000625	0.007063
SCE1572_9069	-2.46519523	-13.7747	0.00063	0.007121
SCE1572_6827	-2.410826544	-13.0153	0.000631	0.007123
SCE1572_7720	-2.48441721	-14.0426	0.000634	0.00714
SCE1572_2377	-2.576278069	-14.9536	0.000635	0.00714
SCE1572_10150	-2.527078363	-14.451	0.000635	0.00714
SCE1572_2105	-2.374069536	-12.0232	0.000637	0.007154
SCE1572_2210	-2.427542689	-13.4012	0.000647	0.007263
SCE1572_847	-2.348583008	-10.3395	0.000649	0.007272
SCE1572_8140	-2.483968122	-14.1099	0.00065	0.007286
SCE1572_7416	-2.391879812	-12.6575	0.000654	0.00732
SCE1572_8010	-2.449760728	-13.8564	0.00066	0.007372
SCE1572_6569	-2.584013717	-15.083	0.000663	0.007407
SCE1572_1052	-2.345276445	-10.6389	0.000669	0.007464
SCE1572_7665	3.026257062	-15.9597	0.000672	0.007477
SCE1572_3255	-2.773923828	-15.7723	0.000672	0.007477
SCE1572_11424	-2.562654785	-14.7999	0.000676	0.007513
SCE1572_1762	2.481178187	-13.7632	0.00068	0.007551
SCE1572_7997	3.382482477	-16.7389	0.000681	0.007554
SCE1572_8851	-3.271423487	-17.1085	0.000682	0.007554
SCE1572_5787	-2.582625175	-15.283	0.000696	0.007704
SCE1572_7229	-2.385165653	-13.1366	0.0007	0.007745
SCE1572_3497	-2.354048544	-12.2097	0.000703	0.007763
SCE1572_2673	-2.398973537	-13.2349	0.000712	0.007851
SCE1572_2759	-2.394404987	-13.2494	0.000712	0.007851
SCE1572_9741	-2.366201712	-12.8062	0.00072	0.007928
SCE1572_9740	-2.382269256	-12.9681	0.000721	0.007937
SCE1572_5001	-2.348044769	-12.0519	0.000725	0.007967
SCE1572_3666	3.659313851	-17.2281	0.000727	0.007974

SCE1572_292	-3.026310989	-16.816	0.000727	0.007974
SCE1572_1031	-2.454869628	-14.2191	0.000737	0.008078
SCE1572_3269	-2.481641194	-14.416	0.00074	0.008097
SCE1572_8125	2.628580723	-14.9361	0.000744	0.008132
SCE1572_1029	-2.575204235	-15.0416	0.000745	0.008132
SCE1572_2216	-2.430901701	-13.847	0.000751	0.008198
SCE1572_4491	-2.444062873	-14.2245	0.000762	0.008308
SCE1572_9317	-2.3213861	-11.1323	0.000767	0.008349
SCE1572_968	-2.364180628	-13.1471	0.000772	0.008399
SCE1572_6736	-2.370201642	-13.1554	0.000785	0.008529
SCE1572_5094	-2.348044769	-12.7889	0.000787	0.008542
SCE1572_7578	-2.546045867	-15.102	0.000789	0.008557
SCE1572_2321	-2.407200266	-13.8777	0.000793	0.008582
SCE1572_8945	-2.336876549	-12.272	0.000796	0.008582
SCE1572_7776	-2.36575087	-13.3519	0.000797	0.008582
SCE1572_9082	33.81613681	-33.108	0.000797	0.008582
SCE1572_11484	33.81613681	-33.108	0.000797	0.008582
SCE1572_8864	33.81613681	-33.108	0.000797	0.008582
SCE1572_10112	33.77431663	-33.1289	0.000797	0.008582
SCE1572_1363	-2.386900704	-13.4633	0.000804	0.008644
SCE1572_10338	-2.321563942	-12.0944	0.000811	0.008713
SCE1572_5171	-2.38072213	-13.384	0.000814	0.008728
SCE1572_3760	4.403263133	-17.8561	0.000815	0.008728
SCE1572_3662	4.401001855	-17.8573	0.000815	0.008728
SCE1572_3273	-2.386900704	-13.6439	0.000817	0.008741
SCE1572_6053	-2.341097015	-12.5737	0.000819	0.008753
SCE1572_7866	-2.310155882	-11.3895	0.000823	0.00877
SCE1572_1585	-2.393414011	-13.7231	0.000823	0.00877
SCE1572_3654	-2.447273322	-14.3507	0.000823	0.00877
SCE1572_1175	-2.493815908	-14.9124	0.000826	0.008788
SCE1572_2299	-2.343467282	-12.7824	0.000827	0.008798
SCE1572_1018	-2.383068843	-13.6947	0.00084	0.008928
SCE1572_1620	-2.466070918	-14.6043	0.000843	0.008944
SCE1572_7721	-2.551531406	-15.2461	0.000845	0.008959
SCE1572_2511	-2.349425999	-12.9846	0.000847	0.008967
SCE1572_6968	-2.389604913	-13.7768	0.000848	0.008971
SCE1572_3126	-2.593351582	-15.3321	0.000849	0.008972
SCE1572_9683	-2.412779336	-14.0353	0.00085	0.008972
SCE1572_9814	-2.412779336	-14.0353	0.00085	0.008972
SCE1572_4979	-2.640657297	-15.4239	0.000854	0.009
SCE1572_6002	-2.488654203	-14.7629	0.000855	0.009003
SCE1572_1525	-2.310201815	-11.8887	0.000858	0.009029
SCE1572_7045	-2.330317176	-12.8786	0.000859	0.00903
SCE1572_10054	-2.310951851	-12.1964	0.000869	0.009124
SCE1572_4737	-2.385495254	-13.8327	0.000876	0.009181
SCE1572_542	3.372432703	-16.7866	0.00088	0.009208
SCE1572_5124	3.353067378	-16.7963	0.00088	0.009208
SCE1572_6092	3.103615944	-16.506	0.000883	0.009226

SCE1572_11275	-2.335149085	-12.8855	0.000883	0.009226
SCE1572_2681	2.555125	-14.7802	0.00089	0.00929
SCE1572_9435	-2.290950111	-11.2859	0.000891	0.00929
SCE1572_5250	-2.311580614	-12.2192	0.000895	0.009316
SCE1572_7887	-2.319456183	-12.5845	0.000905	0.009415
SCE1572_10678	-2.377732389	-13.7309	0.000906	0.009415
SCE1572_5470	2.502764571	-14.347	0.000907	0.009415
SCE1572_5685	-2.321098646	-12.874	0.000918	0.009523
SCE1572_9167	4.120893936	-17.9973	0.000921	0.009538
SCE1572_1070	4.120893936	-17.9973	0.000921	0.009538
SCE1572_7745	-2.272970613	-10.3285	0.000935	0.00967
SCE1572_472	-2.499229405	-14.8317	0.000948	0.009796
SCE1572_1838	-2.302749821	-12.4983	0.00095	0.009806
SCE1572_11176	-2.675813742	-15.9038	0.000964	0.009942
SCE1572_6533	3.00656126	-16.2326	0.000975	0.009998
SCE1572_10898	-2.756850314	-16.0439	0.000975	0.009998
SCE1572_11104	-2.756850314	-16.0439	0.000975	0.009998
SCE1572_7862	-2.324665508	-13.1116	0.000975	0.009998
SCE1572_248	2.529476413	-14.793	0.000976	0.009998
SCE1572_11580	4.372432703	-17.8716	0.000977	0.009998
SCE1572_3682	4.372432703	-17.8716	0.000977	0.009998
SCE1572_7067	4.372432703	-17.8716	0.000977	0.009998
SCE1572_10880	-2.287543152	-12.1247	0.000978	0.010001
SCE1572_7441	2.708398631	-15.5337	0.000984	0.01005
SCE1572_9128	-2.519351	-15.0695	0.000987	0.010072
SCE1572_9106	3.068426516	-16.5236	0.000995	0.010128
SCE1572_1651	3.068426516	-16.5236	0.000995	0.010128
SCE1572_6985	3.586557508	-17.2645	0.001	0.010158
SCE1572_5774	3.561466527	-17.277	0.001	0.010158
SCE1572_1513	3.333438571	-16.8061	0.001002	0.010174
SCE1572_229	-2.352343482	-13.8676	0.001003	0.010176
SCE1572_6629	-2.434362058	-14.5885	0.001035	0.010483
SCE1572_2833	-2.493815908	-15.3274	0.001041	0.010536
SCE1572_8183	2.938029879	-16.0038	0.001043	0.010541
SCE1572_8640	-2.34096442	-13.8189	0.001043	0.010541
SCE1572_2300	-2.382931802	-14.3304	0.001053	0.010626
SCE1572_4686	-2.345119948	-13.701	0.00106	0.010679
SCE1572_1941	2.550578211	-14.9751	0.00106	0.010679
SCE1572_8791	-2.323890907	-13.4124	0.001067	0.010737
SCE1572_9402	-2.244656577	-10.5344	0.001072	0.010781
SCE1572_493	2.773735495	-15.6709	0.001075	0.010802
SCE1572_2694	-2.473057348	-15.1858	0.001077	0.010809
SCE1572_7820	-2.278618988	-12.546	0.001078	0.01081
SCE1572_3926	-2.290282514	-12.9361	0.001081	0.010829
SCE1572_10973	-2.261805484	-11.9647	0.001091	0.010922
SCE1572_8145	-2.607026519	-15.6334	0.001095	0.010937
SCE1572_8784	-2.351913405	-14.2225	0.001096	0.010937
SCE1572_7743	-2.265709836	-12.2134	0.001096	0.010937

SCE1572_3080	-2.370959161	-14.1664	0.00111	0.011068
SCE1572_3200	-2.275493151	-12.7238	0.001111	0.011068
SCE1572_11338	3.032357261	-16.5416	0.001122	0.011164
SCE1572_4203	-2.296298156	-13.3356	0.001125	0.011184
SCE1572_7951	-2.329638101	-13.755	0.001128	0.011208
SCE1572_4664	2.597992403	-15.0583	0.001134	0.011249
SCE1572_8999	-2.25393606	-12.2422	0.001135	0.011252
SCE1572_8796	4.086128518	-18.0147	0.001141	0.011269
SCE1572_2141	4.050504608	-18.0325	0.001141	0.011269
SCE1572_3044	4.050504608	-18.0325	0.001141	0.011269
SCE1572_8984	4.050504608	-18.0325	0.001141	0.011269
SCE1572_3182	-2.264470726	-12.4965	0.001143	0.011275
SCE1572_11010	-2.315277777	-13.8317	0.001157	0.011408
SCE1572_19	-2.264081512	-12.5751	0.00116	0.011426
SCE1572_4193	-2.225440101	-10.1997	0.001168	0.01149
SCE1572_10106	-2.242897789	-11.8327	0.001174	0.011521
SCE1572_6956	4.343286357	-17.8861	0.001176	0.011521
SCE1572_3051	4.343286357	-17.8861	0.001176	0.011521
SCE1572_5460	4.343286357	-17.8861	0.001176	0.011521
SCE1572_56	3.535931435	-17.2898	0.001176	0.011521
SCE1572_7475	-2.236391471	-11.5816	0.001183	0.011578
SCE1572_7531	-2.348044769	-14.3738	0.001189	0.011619
SCE1572_7619	-2.276877917	-13.1464	0.00119	0.011624
SCE1572_8455	-2.425228823	-14.7947	0.001195	0.011638
SCE1572_5421	-2.425228823	-14.7947	0.001195	0.011638
SCE1572_8620	-2.389604913	-14.7768	0.001195	0.011638
SCE1572_1339	-2.311837755	-13.7138	0.001223	0.011898
SCE1572_11194	-2.335553824	-14.3801	0.001234	0.011996
SCE1572_6666	-2.250564882	-12.4966	0.001239	0.012034
SCE1572_2008	2.57824224	-15.1837	0.001244	0.012058
SCE1572_1872	-2.35739671	-14.3176	0.001245	0.012058
SCE1572_6203	-2.332305729	-14.3051	0.001245	0.012058
SCE1572_7542	-2.378338691	-14.4396	0.001252	0.012114
SCE1572_10066	-2.234172092	-12.2178	0.001257	0.012151
SCE1572_372	-2.302787658	-13.67	0.001259	0.012158
SCE1572_5233	-2.773923828	-16.3573	0.001267	0.012217
SCE1572_5269	-2.233119599	-12.3734	0.001267	0.012217
SCE1572_9356	-2.248703411	-12.8829	0.001283	0.012358
SCE1572_4942	-2.512431587	-15.6136	0.001287	0.012385
SCE1572_6654	-2.20444191	-10.2102	0.001289	0.012388
SCE1572_7408	-2.431888159	-15.0258	0.00129	0.012388
SCE1572_6296	-2.302450383	-14.0695	0.001296	0.012435
SCE1572_8052	3.272897029	-16.8364	0.001306	0.012526
SCE1572_9972	-2.241049838	-12.8164	0.001316	0.012607
SCE1572_10536	-2.289125489	-13.8448	0.001319	0.012625
SCE1572_105	33.68685379	-33.1726	0.001334	0.012736
SCE1572_4860	33.6410501	-33.1955	0.001334	0.012736
SCE1572_6105	33.6410501	-33.1955	0.001334	0.012736

SCE1572_4621	-2.263967682	-13.5973	0.00134	0.01278
SCE1572_10281	-2.265025716	-13.3765	0.001344	0.012797
SCE1572_4547	2.43484531	-14.5924	0.001346	0.012797
SCE1572_5193	-2.341812815	-14.5144	0.001346	0.012797
SCE1572_10703	-2.271423487	-13.6699	0.00135	0.012831
SCE1572_5264	-2.271423487	-13.4386	0.001353	0.012833
SCE1572_1842	2.727223352	-15.6942	0.001353	0.012833
SCE1572_8106	2.456496968	-14.8295	0.001355	0.012838
SCE1572_8896	-2.302011807	-14.0489	0.001359	0.012867
SCE1572_4972	-2.412779336	-15.0353	0.001365	0.012908
SCE1572_6681	-2.457836611	-15.2423	0.001388	0.013094
SCE1572_8884	3.509936226	-17.3028	0.001388	0.013094
SCE1572_8157	3.483464015	-17.316	0.001388	0.013094
SCE1572_10686	-2.280301638	-13.8492	0.001391	0.013107
SCE1572_11037	-2.258248098	-13.4187	0.001395	0.013136
SCE1572_3648	-2.271423487	-13.7016	0.001398	0.013151
SCE1572_799	-2.225327072	-12.9037	0.001403	0.013192
SCE1572_5022	-2.264537101	-13.4827	0.001407	0.013204
SCE1572_3691	2.258843493	-13.121	0.001407	0.013204
SCE1572_8375	-2.32204956	-14.3608	0.00141	0.013219
SCE1572_2695	-2.460457311	-15.4551	0.001415	0.013252
SCE1572_7284	4.013978732	-18.0508	0.00142	0.013252
SCE1572_7254	4.013978732	-18.0508	0.00142	0.013252
SCE1572_1187	4.013978732	-18.0508	0.00142	0.013252
SCE1572_1724	-2.257484296	-13.5	0.001421	0.013252
SCE1572_7080	-2.302901719	-14.0903	0.001421	0.013252
SCE1572_853	-2.237476155	-13.1992	0.001428	0.013304
SCE1572_10152	-2.222513887	-12.6106	0.001429	0.013304
SCE1572_6999	2.976504026	-16.5695	0.001433	0.013327
SCE1572_4336	2.396955022	-14.467	0.001435	0.013336
SCE1572_2315	-2.318729202	-14.2629	0.00144	0.013364
SCE1572_11554	2.728576513	-15.8861	0.001446	0.013412
SCE1572_5895	2.309998656	-13.8153	0.001448	0.013419
SCE1572_552	-2.271423487	-13.6857	0.001449	0.013419
SCE1572_474	-2.223805467	-12.9505	0.001454	0.013447
SCE1572_4274	-2.368720688	-14.7186	0.001457	0.013464
SCE1572_1362	-2.193728422	-11.6213	0.001459	0.013469
SCE1572_10652	-2.502749033	-15.5543	0.001462	0.013481
SCE1572_118	-2.21034791	-12.6316	0.001463	0.013481
SCE1572_4815	-2.215928374	-12.9084	0.001464	0.013481
SCE1572_4425	-2.305911863	-14.222	0.001465	0.013481
SCE1572_1348	-2.184149666	-11.3393	0.001482	0.01363
SCE1572_4763	-2.282650742	-14.1879	0.00149	0.013684
SCE1572_3224	3.231076853	-16.8573	0.001495	0.013701
SCE1572_2793	3.231076853	-16.8573	0.001495	0.013701
SCE1572_8482	-2.856385988	-16.5791	0.001495	0.013701
SCE1572_7733	-2.28041227	-13.8671	0.001506	0.013788
SCE1572_6802	-2.180162507	-11.5387	0.001532	0.014016

SCE1572_11360	-2.182820883	-12.124	0.00156	0.014256
SCE1572_8758	-2.190286724	-12.2341	0.001561	0.014257
SCE1572_4319	2.702581304	-15.8991	0.001578	0.014383
SCE1572_4821	2.702581304	-15.8991	0.001578	0.014383
SCE1572_10083	-2.17800479	-11.9594	0.001583	0.014415
SCE1572_2108	-2.294143564	-14.2048	0.001592	0.014485
SCE1572_3171	-2.219376739	-13.2308	0.001599	0.014534
SCE1572_9311	-2.191622776	-12.4325	0.001602	0.014546
SCE1572_1122	2.240025089	-13.2373	0.001611	0.01462
SCE1572_9223	2.272897029	-13.6359	0.001622	0.014666
SCE1572_2527	2.353067378	-14.2963	0.001622	0.014666
SCE1572_2542	2.938029879	-16.5888	0.001623	0.014666
SCE1572_675	2.938029879	-16.5888	0.001623	0.014666
SCE1572_7667	2.238559489	-13.1081	0.001623	0.014666
SCE1572_584	-2.261081543	-14.0694	0.001631	0.014728
SCE1572_2787	-2.245736843	-13.7969	0.001644	0.014831
SCE1572_1901	-2.560930104	-15.8788	0.001649	0.014866
SCE1572_10461	-2.180570057	-12.5042	0.001653	0.014884
SCE1572_9869	-2.170570885	-12.1459	0.001656	0.014896
SCE1572_3805	-2.271423487	-14.106	0.001661	0.014923
SCE1572_3510	-2.349425999	-14.9846	0.001661	0.014923
SCE1572_9350	-2.153652028	-10.7848	0.001666	0.014939
SCE1572_10179	-2.210541245	-13.3051	0.001666	0.014939
SCE1572_9872	-2.250664927	-14.0746	0.001682	0.015071
SCE1572_8575	2.566866274	-15.3848	0.001685	0.015083
SCE1572_10485	-2.20776485	-13.1064	0.001692	0.015129
SCE1572_10295	-2.241049838	-14.0388	0.001703	0.015221
SCE1572_7398	-2.20729315	-13.3801	0.00171	0.015264
SCE1572_8809	2.659313851	-15.7281	0.001711	0.015264
SCE1572_6721	-2.259549387	-14.2687	0.001713	0.015271
SCE1572_8161	3.209703203	-16.868	0.001715	0.015271
SCE1572_6376	4.220429609	-17.9476	0.001718	0.015278
SCE1572_5610	4.220429609	-17.9476	0.001718	0.015278
SCE1572_8463	-2.144029861	-10.6591	0.00173	0.015371
SCE1572_7544	33.59374439	-33.2192	0.001744	0.015477
SCE1572_7972	-2.447273322	-15.3507	0.00175	0.015521
SCE1572_10508	-2.250959384	-14.054	0.001756	0.01555
SCE1572_1868	-2.287725299	-14.7259	0.001756	0.01555
SCE1572_791	-2.192351916	-13.0042	0.001765	0.015609
SCE1572_6845	-2.167005592	-12.4513	0.001767	0.015615
SCE1572_4709	2.569878767	-15.6029	0.00177	0.015615
SCE1572_8060	3.976504026	-18.0695	0.001774	0.015615
SCE1572_2450	3.976504026	-18.0695	0.001774	0.015615
SCE1572_8800	3.938029879	-18.0888	0.001774	0.015615
SCE1572_1893	-3.358886328	-17.6498	0.001774	0.015615
SCE1572_10449	-2.241470265	-14.0186	0.001776	0.015616
SCE1572_10665	-2.191081379	-13.3268	0.001782	0.015655
SCE1572_7823	-2.18261422	-13.1718	0.001791	0.015725

SCE1572_432	-2.1870313	-13.1913	0.001811	0.015884
SCE1572_7083	-2.353885647	-15.0648	0.001818	0.015938
SCE1572_5128	-2.14444266	-11.7571	0.00183	0.015997
SCE1572_6849	2.849591914	-16.3111	0.001831	0.015997
SCE1572_497	2.849591914	-16.3111	0.001831	0.015997
SCE1572_6089	-2.612460405	-16.1161	0.001831	0.015997
SCE1572_9389	-2.208229661	-13.5108	0.001846	0.016113
SCE1572_6335	2.479347907	-15.1177	0.001858	0.016202
SCE1572_10000	-2.220439558	-14.0491	0.001865	0.016256
SCE1572_6902	2.483464015	-15.2311	0.001887	0.016434
SCE1572_9310	-2.144311569	-12.297	0.001892	0.01646
SCE1572_1615	-2.144384298	-12.2291	0.001902	0.016536
SCE1572_4144	-2.193035588	-13.5322	0.001913	0.016613
SCE1572_8102	-2.333707765	-15.0749	0.001928	0.016728
SCE1572_4239	-2.150237141	-12.533	0.001937	0.016797
SCE1572_2111	-2.176682997	-13.3582	0.001946	0.016818
SCE1572_7393	3.429016231	-17.3433	0.001948	0.016818
SCE1572_9078	3.401001855	-17.3573	0.001948	0.016818
SCE1572_5148	3.401001855	-17.3573	0.001948	0.016818
SCE1572_452	-2.794985443	-16.9317	0.001948	0.016818
SCE1572_617	-2.148275256	-12.4737	0.001955	0.016868
SCE1572_4935	-2.171887814	-13.3364	0.001958	0.016877
SCE1572_2325	-2.403873783	-15.4268	0.001961	0.016877
SCE1572_6375	2.382580658	-14.779	0.001961	0.016877
SCE1572_3647	-2.307047397	-14.8537	0.001964	0.016887
SCE1572_1030	-2.351593836	-15.0241	0.001965	0.016888
SCE1572_6069	-2.181008192	-13.4972	0.00198	0.017
SCE1572_9287	-2.116145262	-10.956	0.001982	0.017008
SCE1572_6899	-2.288497	-14.7926	0.001995	0.017096
SCE1572_11490	-33.32704563	-33.3525	0.001996	0.017096
SCE1572_10086	-2.126814106	-11.8312	0.002005	0.01716
SCE1572_11330	-2.200650413	-13.6743	0.002008	0.017161
SCE1572_1584	-2.271423487	-14.493	0.002009	0.017161
SCE1572_3830	-2.271423487	-14.6085	0.002017	0.017216
SCE1572_439	2.158033072	-12.5864	0.002027	0.017295
SCE1572_10599	2.832913173	-16.3194	0.002042	0.017387
SCE1572_84	-2.573986257	-16.1353	0.002042	0.017387
SCE1572_8516	2.649142045	-15.9259	0.00206	0.01753
SCE1572_379	-2.192351916	-14.0042	0.002079	0.017678
SCE1572_9484	-2.119912026	-11.9205	0.002082	0.017686
SCE1572_10997	-2.130825157	-12.5495	0.002086	0.017686
SCE1572_6649	4.188008132	-17.9638	0.002088	0.017686
SCE1572_8435	4.188008132	-17.9638	0.002088	0.017686
SCE1572_8185	4.154841268	-17.9804	0.002088	0.017686
SCE1572_2056	2.878323632	-16.6186	0.002091	0.017694
SCE1572_6545	-2.132447074	-12.6483	0.002102	0.017769
SCE1572_7148	-2.152778991	-13.4199	0.002111	0.017832
SCE1572_3258	-2.211050869	-14.2929	0.002119	0.017887

SCE1572_6367	-2.254935364	-14.7423	0.002125	0.017919
SCE1572_4819	-2.158212877	-13.5222	0.002143	0.01806
SCE1572_4609	2.36221286	-14.7892	0.002151	0.018118
SCE1572_6114	2.515172875	-15.4783	0.002153	0.018119
SCE1572_10608	-2.207858358	-14.3673	0.002157	0.018125
SCE1572_711	-2.224880901	-14.2395	0.002158	0.018125
SCE1572_10120	-2.108915957	-12.0899	0.002185	0.018338
SCE1572_5077	-2.137873157	-12.9027	0.002186	0.018338
SCE1572_2177	2.758323856	-16.0937	0.002193	0.018346
SCE1572_3039	2.758323856	-16.0937	0.002193	0.018346
SCE1572_10918	-2.415813396	-15.8734	0.002193	0.018346
SCE1572_1404	2.449053984	-15.3738	0.002195	0.018348
SCE1572_9335	-2.178314083	-13.7806	0.002212	0.018477
SCE1572_2413	-2.218956067	-14.4124	0.00222	0.018529
SCE1572_8779	-2.414381441	-15.537	0.002224	0.01855
SCE1572_6670	3.898501514	-18.1085	0.002228	0.018569
SCE1572_8331	-2.133393336	-13.1645	0.002253	0.018761
SCE1572_11343	-2.308898192	-14.9268	0.002262	0.01882
SCE1572_3041	3.143614012	-16.901	0.002266	0.018836
SCE1572_9664	-2.378338691	-15.4396	0.002269	0.018836
SCE1572_9233	-2.141167101	-13.1397	0.002269	0.018836
SCE1572_10534	-2.198667145	-14.2991	0.002277	0.01887
SCE1572_7276	2.798965841	-16.3364	0.002278	0.01887
SCE1572_7213	2.798965841	-16.3364	0.002278	0.01887
SCE1572_6665	-2.18336731	-13.9896	0.002287	0.018927
SCE1572_76	33.49420871	-33.2689	0.002295	0.018931
SCE1572_3092	33.49420871	-33.2689	0.002295	0.018931
SCE1572_5759	33.49420871	-33.2689	0.002295	0.018931
SCE1572_240	33.4755395	-33.2783	0.002295	0.018931
SCE1572_1258	3.372432703	-17.3716	0.002316	0.019094
SCE1572_7771	-2.219893186	-14.3864	0.002326	0.019155
SCE1572_1313	-2.071887509	-9.71488	0.002333	0.019198
SCE1572_787	-2.084028918	-11.5356	0.002348	0.01931
SCE1572_10048	-2.135004289	-13.2064	0.002355	0.019351
SCE1572_4545	2.586557508	-15.7645	0.002359	0.019351
SCE1572_3992	-2.391717721	-15.6098	0.002359	0.019351
SCE1572_6154	2.85785953	-16.6288	0.002379	0.019486
SCE1572_1782	-2.640657297	-16.4239	0.002379	0.019486
SCE1572_4032	2.092630084	-11.6716	0.002387	0.019537
SCE1572_745	-2.088242389	-11.7576	0.00239	0.019544
SCE1572_6878	2.208356777	-13.9536	0.002393	0.019549
SCE1572_5057	-2.098091884	-12.2303	0.002394	0.019549
SCE1572_3902	-2.271423487	-15.1491	0.002412	0.019685
SCE1572_8648	2.728576513	-16.1085	0.002415	0.019688
SCE1572_7286	-2.129404482	-13.4571	0.002422	0.019734
SCE1572_6971	2.328489355	-14.8935	0.002434	0.019813
SCE1572_5098	-2.105785844	-12.7141	0.002436	0.019816
SCE1572_4501	-2.155326422	-13.6516	0.002448	0.019901

SCE1572_1347	-2.069128239	-11.0658	0.002458	0.019964
SCE1572_45	-2.163170596	-13.8726	0.002482	0.020146
SCE1572_10584	-2.073977423	-11.696	0.002495	0.020229
SCE1572_9291	-2.271423487	-15.2866	0.00251	0.02033
SCE1572_7843	-2.09003937	-12.3954	0.002513	0.02033
SCE1572_3177	-2.104669942	-13.1271	0.002513	0.02033
SCE1572_6276	-2.183196304	-14.355	0.002524	0.020387
SCE1572_7199	-2.183196304	-14.355	0.002524	0.020387
SCE1572_1707	2.080048883	-11.5748	0.002531	0.020432
SCE1572_10068	-2.078028762	-12.1869	0.002545	0.020487
SCE1572_9573	2.764200423	-16.3537	0.002546	0.020487
SCE1572_765	-2.534457893	-16.1551	0.002546	0.020487
SCE1572_6381	-2.534457893	-16.1551	0.002546	0.020487
SCE1572_6712	-2.095266532	-12.8386	0.002549	0.020497
SCE1572_8534	-2.105686283	-13.2651	0.002597	0.020869
SCE1572_8261	-2.093086246	-12.8564	0.002615	0.020994
SCE1572_10669	-2.108773149	-13.1874	0.002625	0.021061
SCE1572_1303	-2.25179468	-14.9938	0.002637	0.021137
SCE1572_11273	-2.117878229	-13.5698	0.002654	0.021246
SCE1572_3867	-2.154066536	-14.2522	0.002655	0.021246
SCE1572_3704	-2.233948782	-14.9268	0.002656	0.021246
SCE1572_7238	-2.070863573	-12.4173	0.002663	0.021281
SCE1572_652	-2.049963943	-10.7099	0.002673	0.021328
SCE1572_1373	-2.110569476	-13.5057	0.002674	0.021328
SCE1572_945	2.335580889	-14.963	0.002675	0.021328
SCE1572_2850	-2.204309291	-14.4457	0.002697	0.021488
SCE1572_3930	-2.057373046	-11.7006	0.002707	0.021515
SCE1572_9081	2.83710097	-16.6392	0.00271	0.021515
SCE1572_3546	2.83710097	-16.6392	0.00271	0.021515
SCE1572_6817	2.83710097	-16.6392	0.00271	0.021515
SCE1572_576	-2.070125097	-12.5459	0.00271	0.021515
SCE1572_10981	-2.0692932	-12.5264	0.002716	0.021545
SCE1572_7784	-2.284207654	-15.1862	0.002733	0.021663
SCE1572_10017	-2.087977346	-13.2191	0.002755	0.021824
SCE1572_7835	-2.211302495	-14.6088	0.002762	0.021832
SCE1572_7202	3.343286357	-17.3861	0.002763	0.021832
SCE1572_6068	3.343286357	-17.3861	0.002763	0.021832
SCE1572_5388	-2.06188663	-12.2214	0.002771	0.021853
SCE1572_11495	2.394459009	-15.1601	0.002772	0.021853
SCE1572_10210	2.394459009	-15.1601	0.002772	0.021853
SCE1572_5469	2.245852206	-14.6129	0.002774	0.021853
SCE1572_5251	-2.069180765	-12.4059	0.002778	0.021872
SCE1572_1091	2.340929012	-15.08	0.002788	0.021937
SCE1572_4932	-2.042131507	-11.2747	0.002802	0.022026
SCE1572_4657	-2.252314664	-14.9551	0.002804	0.022026
SCE1572_936	3.816039354	-18.1498	0.002812	0.022066
SCE1572_5788	-2.049031066	-11.8535	0.002813	0.022066
SCE1572_2584	2.337385756	-14.9822	0.002818	0.022089

SCE1572_5061	-2.201034159	-14.8364	0.002832	0.022177
SCE1572_8946	-2.046179325	-11.585	0.002836	0.022197
SCE1572_9778	-2.070604478	-12.8263	0.002847	0.022252
SCE1572_9978	2.746498421	-16.3626	0.002848	0.022252
SCE1572_1221	2.451042537	-15.5103	0.002856	0.022296
SCE1572_5725	32.95588694	-33.5381	0.002864	0.022296
SCE1572_4079	32.9347813	-33.5487	0.002864	0.022296
SCE1572_8624	32.9347813	-33.5487	0.002864	0.022296
SCE1572_8064	32.9347813	-33.5487	0.002864	0.022296
SCE1572_3420	-2.06635915	-12.6522	0.002881	0.022415
SCE1572_8854	-2.166726108	-14.602	0.002886	0.022433
SCE1572_3969	-2.043555936	-12.0406	0.002904	0.02256
SCE1572_10073	-2.080239366	-13.1495	0.002918	0.022652
SCE1572_10555	2.322757544	-15.196	0.002925	0.022688
SCE1572_11386	-2.033862689	-11.3639	0.002938	0.022764
SCE1572_10118	-2.085411501	-13.3323	0.002942	0.022764
SCE1572_3622	2.225763054	-14.3599	0.002943	0.022764
SCE1572_7597	-2.150117191	-14.2999	0.002943	0.022764
SCE1572_7956	-2.152124559	-14.2759	0.002949	0.022791
SCE1572_7682	2.565077781	-15.9679	0.002969	0.02293
SCE1572_197	-2.121528057	-14.0205	0.002985	0.023038
SCE1572_6045	-2.025416093	-10.9567	0.00299	0.02306
SCE1572_10642	-2.046653127	-12.3229	0.002993	0.023061
SCE1572_1960	2.292165363	-14.8242	0.002996	0.023066
SCE1572_5076	-2.05596378	-12.8666	0.003001	0.023091
SCE1572_9003	-2.058694163	-12.7607	0.003013	0.023163
SCE1572_10283	-2.176564301	-14.4596	0.003036	0.023293
SCE1572_3195	33.44174129	-33.2952	0.003043	0.023293
SCE1572_2318	33.44174129	-33.2952	0.003043	0.023293
SCE1572_7787	33.44174129	-33.2952	0.003043	0.023293
SCE1572_659	33.44174129	-33.2952	0.003043	0.023293
SCE1572_6383	33.44174129	-33.2952	0.003043	0.023293
SCE1572_10153	-2.250664927	-15.0746	0.003116	0.023832
SCE1572_1231	-2.051297673	-12.9907	0.003126	0.023892
SCE1572_6100	2.248833324	-14.6854	0.003151	0.02407
SCE1572_6995	2.21400334	-14.6288	0.003154	0.02407
SCE1572_4962	-2.145892605	-14.3494	0.003163	0.024124
SCE1572_5143	2.728576513	-16.3716	0.003189	0.024305
SCE1572_8260	-2.031025278	-12.5655	0.003207	0.024412
SCE1572_10044	-2.021139783	-11.7374	0.003208	0.024412
SCE1572_4053	-2.052783201	-13.3028	0.003234	0.024593
SCE1572_3384	-2.039797786	-12.6019	0.003245	0.024664
SCE1572_11572	-2.078778409	-13.7662	0.003255	0.024717
SCE1572_666	-2.04361757	-12.8317	0.003259	0.024733
SCE1572_2436	-2.370959161	-15.7514	0.003273	0.024817
SCE1572_10910	2.197355304	-14.6372	0.003292	0.024935
SCE1572_9383	-1.99340674	-9.02416	0.003293	0.024935
SCE1572_9711	-2.020438644	-12.0429	0.003296	0.024935

SCE1572_8087	2.259957973	-14.9278	0.003301	0.024935
SCE1572_6151	2.305501695	-14.9981	0.003303	0.024935
SCE1572_11346	-2.06120578	-13.416	0.003304	0.024935
SCE1572_6487	3.283165365	-17.4162	0.003305	0.024935
SCE1572_9290	-2.027248108	-12.4827	0.003344	0.025213
SCE1572_4536	-2.099242512	-13.9577	0.003358	0.025283
SCE1572_9744	-2.099242512	-13.9577	0.003358	0.025283
SCE1572_6732	-2.09633678	-14.1517	0.003382	0.025444
SCE1572_9014	-2.018796304	-12.5319	0.003388	0.025468
SCE1572_1114	-2.04554208	-13.1979	0.003401	0.02555
SCE1572_4184	-2.064327253	-13.8708	0.003442	0.025839
SCE1572_3159	-1.989129708	-10.4891	0.003448	0.02587
SCE1572_983	-2.014450834	-12.1672	0.003452	0.025881
SCE1572_1798	-2.154609822	-14.76	0.003463	0.025941
SCE1572_704	-2.033731929	-13.0086	0.003476	0.026019
SCE1572_10861	-1.979878051	-9.31343	0.003506	0.026231
SCE1572_1012	2.750944326	-16.6823	0.003535	0.026402
SCE1572_3060	2.750944326	-16.6823	0.003535	0.026402
SCE1572_2724	-2.095573652	-14.3507	0.003537	0.026402
SCE1572_9845	2.406648418	-15.5325	0.003548	0.026464
SCE1572_7173	-2.044290033	-13.3184	0.003551	0.02647
SCE1572_5699	-2.2850536	-15.4968	0.003556	0.026487
SCE1572_73	-2.058429764	-13.6276	0.00356	0.026499
SCE1572_2720	2.130024947	-14.0859	0.003578	0.026599
SCE1572_9042	-2.113358641	-14.5446	0.003581	0.026599
SCE1572_7598	-2.386900704	-15.9658	0.003583	0.026599
SCE1572_7337	-2.0164264	-12.8372	0.003584	0.026599
SCE1572_8702	-2.087460652	-14.1357	0.003603	0.026719
SCE1572_934	-2.067409595	-13.7877	0.003605	0.026719
SCE1572_2145	-2.045656339	-13.5649	0.003623	0.026834
SCE1572_11042	2.053033434	-13.1984	0.003635	0.026904
SCE1572_5021	-1.997500765	-12.2752	0.00364	0.026921
SCE1572_924	-2.027497904	-13.2386	0.003657	0.027028
SCE1572_9512	-1.988428339	-11.7166	0.003659	0.027028
SCE1572_2407	-2.011505054	-12.7921	0.003664	0.027047
SCE1572_10080	-2.003783507	-12.3558	0.003668	0.027052
SCE1572_7449	-1.97719446	-11.2087	0.003728	0.027481
SCE1572_418	2.00241032	-12.1258	0.003737	0.027523
SCE1572_7963	-2.000001296	-12.6357	0.00374	0.027531
SCE1572_6126	-2.122560101	-14.5955	0.003751	0.027588
SCE1572_6678	-1.975076336	-11.2331	0.003756	0.027594
SCE1572_6198	2.323523102	-15.3111	0.003757	0.027594
SCE1572_10900	-2.103300728	-14.6337	0.003766	0.027644
SCE1572_9807	-1.991315568	-12.3392	0.003769	0.027645
SCE1572_7081	-2.058249596	-13.7923	0.003795	0.027803
SCE1572_4922	2.313539014	-15.4416	0.003796	0.027803
SCE1572_6908	2.383928342	-15.5439	0.003817	0.027919
SCE1572_9516	-2.218956067	-15.4124	0.003817	0.027919

SCE1572_8787	-2.075872678	-14.1415	0.003844	0.028095
SCE1572_9351	-1.984048557	-12.2983	0.003857	0.028153
SCE1572_8016	2.378830474	-15.6984	0.003862	0.028153
SCE1572_4318	2.378830474	-15.6984	0.003862	0.028153
SCE1572_9301	-2.30117083	-15.5937	0.003862	0.028153
SCE1572_2379	-2.135768388	-14.9759	0.003901	0.028418
SCE1572_10972	-2.081945688	-14.2659	0.003905	0.028427
SCE1572_10183	-2.015962301	-13.4883	0.003915	0.028474
SCE1572_10698	-2.106792785	-14.6034	0.003919	0.028484
SCE1572_2906	2.107088136	-14.0973	0.003922	0.028489
SCE1572_880	-1.960842611	-10.6363	0.003932	0.028539
SCE1572_7223	-1.959663041	-10.6823	0.003935	0.028543
SCE1572_6826	-2.307047397	-15.8537	0.003938	0.028543
SCE1572_6632	-2.017896167	-13.4014	0.003942	0.028557
SCE1572_4963	-1.963952227	-11.0412	0.00396	0.028641
SCE1572_3179	2.619347443	-16.1631	0.003964	0.028641
SCE1572_7394	3.220429609	-17.4476	0.003965	0.028641
SCE1572_104	3.220429609	-17.4476	0.003965	0.028641
SCE1572_2223	-2.032484757	-13.7431	0.003968	0.028645
SCE1572_1862	2.147529062	-14.4605	0.003979	0.028686
SCE1572_6094	2.141652496	-14.4634	0.003979	0.028686
SCE1572_6127	-2.00039629	-12.8292	0.003982	0.028689
SCE1572_4303	2.501166017	-16.2222	0.003989	0.028718
SCE1572_8581	1.980679121	-11.9817	0.003993	0.02873
SCE1572_10629	-1.967146125	-11.5278	0.00401	0.028831
SCE1572_4751	-2.01988472	-13.6754	0.004025	0.028916
SCE1572_11119	-1.962978622	-11.613	0.004046	0.028956
SCE1572_8686	2.728576513	-16.6935	0.004046	0.028956
SCE1572_1111	2.728576513	-16.6935	0.004046	0.028956
SCE1572_9784	2.728576513	-16.6935	0.004046	0.028956
SCE1572_3119	-2.493815908	-16.4973	0.004046	0.028956
SCE1572_941	2.976504026	-16.9846	0.004047	0.028956
SCE1572_2084	-2.042604797	-14.0791	0.004063	0.028977
SCE1572_6173	-2.161798996	-14.8907	0.004064	0.028977
SCE1572_651	33.38729351	-33.3224	0.004066	0.028977
SCE1572_10694	33.33070998	-33.3507	0.004066	0.028977
SCE1572_7994	33.33070998	-33.3507	0.004066	0.028977
SCE1572_7552	33.33070998	-33.3507	0.004066	0.028977
SCE1572_7886	-2.171887814	-15.3364	0.004073	0.029002
SCE1572_1099	-2.056255392	-13.926	0.004075	0.029002
SCE1572_2006	-2.075280965	-14.4089	0.004137	0.029366
SCE1572_10806	2.008393771	-12.8638	0.004157	0.029366
SCE1572_596	-1.947863516	-10.7591	0.004166	0.029366
SCE1572_2460	32.85677879	-33.5877	0.004166	0.029366
SCE1572_5586	32.85677879	-33.5877	0.004166	0.029366
SCE1572_10709	32.85677879	-33.5877	0.004166	0.029366
SCE1572_3242	32.85677879	-33.5877	0.004166	0.029366
SCE1572_7481	32.85677879	-33.5877	0.004166	0.029366

SCE1572_8523	32.85677879	-33.5877	0.004166	0.029366
SCE1572_6930	32.85677879	-33.5877	0.004166	0.029366
SCE1572_11472	32.85677879	-33.5877	0.004166	0.029366
SCE1572_62	32.85677879	-33.5877	0.004166	0.029366
SCE1572_286	32.85677879	-33.5877	0.004166	0.029366
SCE1572_6920	32.85677879	-33.5877	0.004166	0.029366
SCE1572_11344	-1.956759088	-11.6331	0.00418	0.029447
SCE1572_3081	-2.271423487	-15.6085	0.004189	0.02947
SCE1572_7281	-2.212529798	-15.5791	0.004189	0.02947
SCE1572_8874	2.08506183	-13.9709	0.004192	0.029472
SCE1572_9736	2.442822031	-15.8364	0.004233	0.029719
SCE1572_2578	2.442822031	-15.8364	0.004233	0.029719
SCE1572_8903	2.23759016	-15.2385	0.004236	0.029719
SCE1572_11601	-2.054931667	-14.371	0.004272	0.029952
SCE1572_35	-2.133919963	-15.2178	0.004278	0.029966
SCE1572_1424	-2.090851241	-14.6113	0.004279	0.029966
SCE1572_6325	-2.107924755	-14.8829	0.004284	0.029979
SCE1572_9434	-1.959479481	-12.0096	0.0043	0.030069
SCE1572_4071	-2.175208172	-15.2874	0.004323	0.030192
SCE1572_4382	2.239538432	-15.0311	0.004323	0.030192
SCE1572_1262	-1.995789044	-13.4858	0.004336	0.030262
SCE1572_5581	-1.971863205	-12.6994	0.004358	0.030396
SCE1572_7543	-2.089976616	-14.5029	0.004364	0.030404
SCE1572_1755	-2.115304285	-14.9861	0.004365	0.030404
SCE1572_2305	-2.07438664	-14.7374	0.004387	0.030535
SCE1572_6586	2.126607587	-14.4095	0.004405	0.030641
SCE1572_2028	-1.987120464	-13.3931	0.004427	0.030745
SCE1572_6705	-1.927244601	-9.50915	0.004427	0.030745
SCE1572_7	-1.966809977	-12.8864	0.004429	0.030745
SCE1572_9283	-2.041125868	-14.3779	0.004438	0.030787
SCE1572_8133	2.483464015	-16.2311	0.004447	0.030815
SCE1572_8153	2.483464015	-16.2311	0.004447	0.030815
SCE1572_1380	1.986667869	-12.9984	0.004458	0.030867
SCE1572_2487	-1.969959495	-12.893	0.004491	0.03108
SCE1572_6808	-1.971249945	-12.8437	0.0045	0.03112
SCE1572_8460	-2.138156956	-15.1727	0.00451	0.03117
SCE1572_2975	2.325394885	-15.695	0.004545	0.031303
SCE1572_4036	3.728576513	-18.1935	0.004551	0.031303
SCE1572_7715	3.728576513	-18.1935	0.004551	0.031303
SCE1572_9795	3.682772823	-18.2164	0.004551	0.031303
SCE1572_8666	3.682772823	-18.2164	0.004551	0.031303
SCE1572_8500	3.682772823	-18.2164	0.004551	0.031303
SCE1572_7934	3.682772823	-18.2164	0.004551	0.031303
SCE1572_2042	-1.980644091	-13.3616	0.004565	0.031367
SCE1572_4485	-2.119420394	-14.9476	0.004566	0.031367
SCE1572_1822	2.143614012	-14.816	0.004604	0.031606
SCE1572_4777	-1.979657363	-13.1896	0.004608	0.031619
SCE1572_5585	2.429016231	-15.8433	0.004614	0.031636

SCE1572_4829	-2.13202043	-15.0153	0.004619	0.031649
SCE1572_2254	2.705856436	-16.7048	0.00464	0.031738
SCE1572_11186	2.705856436	-16.7048	0.00464	0.031738
SCE1572_3324	-1.948623936	-12.6614	0.004641	0.031738
SCE1572_9973	-1.94185533	-12.2085	0.004652	0.031793
SCE1572_1613	2.041993108	-13.9493	0.004664	0.031857
SCE1572_5102	-1.978376512	-13.2525	0.004685	0.031977
SCE1572_5483	2.257829581	-15.4694	0.004695	0.032023
SCE1572_3101	-2.593351582	-16.7106	0.004701	0.032045
SCE1572_4175	2.095693382	-14.5505	0.004738	0.032241
SCE1572_6745	-2.075026274	-14.5103	0.004738	0.032241
SCE1572_7991	-1.988251436	-13.6769	0.004739	0.032241
SCE1572_9638	-2.08855943	-14.8926	0.004762	0.032361
SCE1572_2411	-1.976295452	-13.6972	0.004767	0.032361
SCE1572_10523	3.188008132	-17.4638	0.004771	0.032361
SCE1572_10196	3.188008132	-17.4638	0.004771	0.032361
SCE1572_7661	2.444783547	-16.028	0.004772	0.032361
SCE1572_8396	-2.099242512	-14.9577	0.004823	0.032688
SCE1572_10143	-2.030415388	-14.2916	0.004837	0.032757
SCE1572_10856	-1.936653328	-12.2948	0.004847	0.032787
SCE1572_5012	-2.185693613	-15.5359	0.004847	0.032787
SCE1572_611	-2.310951851	-16.0038	0.004863	0.032872
SCE1572_11249	-2.154066536	-15.2522	0.004878	0.03295
SCE1572_7675	1.954580802	-12.6202	0.004895	0.033049
SCE1572_1149	-1.917201989	-11.4637	0.004911	0.03312
SCE1572_4	-1.953808385	-12.9686	0.004914	0.03312
SCE1572_1795	-1.959479481	-13.1795	0.004915	0.03312
SCE1572_10456	2.326835836	-15.7244	0.004935	0.033231
SCE1572_9063	2.138860482	-14.7404	0.004947	0.03329
SCE1572_10704	-2.059919382	-14.8398	0.004955	0.033322
SCE1572_11031	-2.106364241	-15.0666	0.00496	0.033334
SCE1572_10870	2.44739476	-16.2491	0.004963	0.033336
SCE1572_5040	-1.991900958	-13.8738	0.004997	0.033539
SCE1572_7435	2.41507704	-15.8502	0.005032	0.033755
SCE1572_11087	-2.119420394	-15.3626	0.005044	0.033812
SCE1572_3516	-2.035356129	-14.5836	0.005052	0.033842
SCE1572_11372	-1.966944363	-13.5255	0.005061	0.033866
SCE1572_7055	1.985587131	-13.4211	0.005062	0.033866
SCE1572_5488	2.120893936	-14.8274	0.005073	0.033901
SCE1572_9931	-2.03876273	-14.7552	0.005073	0.033901
SCE1572_10463	-1.905646848	-11.356	0.005091	0.034
SCE1572_1361	-1.941274885	-13.0918	0.005109	0.034078
SCE1572_9609	-1.941274885	-13.0918	0.005109	0.034078
SCE1572_10011	-1.902688035	-11.1418	0.00512	0.034125
SCE1572_6912	2.301466181	-15.5851	0.005139	0.034186
SCE1572_8916	-2.188961327	-15.4798	0.005139	0.034186
SCE1572_8405	-2.136493907	-15.4536	0.005139	0.034186
SCE1572_5231	-1.935632739	-12.8457	0.005142	0.034186

SCE1572_6720	-1.889617416	-7.81122	0.00515	0.034218
SCE1572_5029	-1.982797367	-13.83	0.005192	0.034471
SCE1572_986	-1.902928574	-11.447	0.005195	0.034471
SCE1572_10774	-1.930386569	-12.6566	0.005203	0.034489
SCE1572_8608	-1.916838505	-12.1613	0.005204	0.034489
SCE1572_7571	-1.948128556	-13.3107	0.005213	0.034531
SCE1572_10834	2.062000247	-14.3829	0.005256	0.034789
SCE1572_698	-2.234897611	-15.8898	0.00526	0.034793
SCE1572_1502	-1.967747097	-13.5904	0.005277	0.034861
SCE1572_11548	-1.954450274	-13.5838	0.005277	0.034861
SCE1572_9281	-1.914631129	-12.3112	0.005292	0.034941
SCE1572_6074	-1.900260797	-11.6614	0.005323	0.035119
SCE1572_10069	2.659313851	-16.7281	0.005329	0.035119
SCE1572_6421	2.659313851	-16.7281	0.005329	0.035119
SCE1572_7582	-1.915279677	-12.5642	0.005338	0.035158
SCE1572_8002	2.124764111	-14.71	0.005349	0.035163
SCE1572_11059	2.124764111	-14.71	0.005349	0.035163
SCE1572_2838	2.124764111	-14.71	0.005349	0.035163
SCE1572_1885	-2.119420394	-15.1402	0.005356	0.035179
SCE1572_5125	2.300118498	-15.7378	0.005361	0.035179
SCE1572_2014	2.300118498	-15.7378	0.005361	0.035179
SCE1572_9823	1.966807528	-13.293	0.005416	0.035513
SCE1572_8065	2.11727362	-14.7512	0.005426	0.035533
SCE1572_2338	-2.01988472	-14.6754	0.005426	0.035533
SCE1572_11246	2.091146592	-14.6199	0.005438	0.035575
SCE1572_7688	2.069200702	-14.4996	0.005447	0.035575
SCE1572_8798	2.167688147	-15.2735	0.005458	0.035575
SCE1572_4961	-2.090851241	-15.1963	0.005458	0.035575
SCE1572_8300	2.924973726	-17.0103	0.005472	0.035575
SCE1572_7051	2.924973726	-17.0103	0.005472	0.035575
SCE1572_8427	2.898501514	-17.0236	0.005472	0.035575
SCE1572_2046	33.27181629	-33.3801	0.005479	0.035575
SCE1572_10094	33.27181629	-33.3801	0.005479	0.035575
SCE1572_10631	33.27181629	-33.3801	0.005479	0.035575
SCE1572_5401	33.27181629	-33.3801	0.005479	0.035575
SCE1572_3686	33.27181629	-33.3801	0.005479	0.035575
SCE1572_3287	33.27181629	-33.3801	0.005479	0.035575
SCE1572_8827	33.23836981	-33.3969	0.005479	0.035575
SCE1572_9219	2.386787996	-15.8644	0.005492	0.035633
SCE1572_5486	2.026257062	-14.1378	0.005525	0.035826
SCE1572_10886	1.971433037	-13.5802	0.005561	0.036039
SCE1572_4236	-1.95655015	-13.6785	0.005566	0.036051
SCE1572_4225	2.231076853	-15.3573	0.00557	0.036055
SCE1572_4218	-2.083796484	-14.9298	0.005592	0.036151
SCE1572_423	-2.003227376	-14.5202	0.005595	0.036151
SCE1572_10098	-2.020615384	-14.7643	0.005596	0.036151
SCE1572_6708	-1.947533876	-13.8319	0.005623	0.036305
SCE1572_9074	2.038916634	-14.3945	0.005661	0.03653

SCE1572_9213	2.056763598	-14.5058	0.005669	0.036556
SCE1572_4213	-1.888094848	-11.7234	0.005679	0.03659
SCE1572_7298	2.102700819	-14.7585	0.005684	0.03659
SCE1572_3997	-1.912173766	-12.834	0.005684	0.03659
SCE1572_2059	2.132966768	-14.9038	0.00569	0.036606
SCE1572_8856	-2.055694796	-15.1314	0.005698	0.03663
SCE1572_2092	2.556395538	-16.4577	0.005716	0.036727
SCE1572_1673	-1.904749009	-12.9174	0.005724	0.036757
SCE1572_9025	-1.949495392	-13.6402	0.005741	0.036839
SCE1572_1765	3.154841268	-17.4804	0.00576	0.036848
SCE1572_7863	3.120893936	-17.4973	0.00576	0.036848
SCE1572_4617	3.120893936	-17.4973	0.00576	0.036848
SCE1572_124	3.120893936	-17.4973	0.00576	0.036848
SCE1572_2301	-2.711996078	-17.1658	0.00576	0.036848
SCE1572_11092	-1.961468034	-14.1196	0.005768	0.036878
SCE1572_7943	-2.054612098	-14.8756	0.005792	0.037014
SCE1572_1328	2.396955022	-16.0519	0.005802	0.037052
SCE1572_1940	1.938029879	-13.0888	0.005811	0.03709
SCE1572_508	2.286571966	-15.7446	0.005828	0.037175
SCE1572_364	3.635467109	-18.24	0.005837	0.037209
SCE1572_2477	2.064859901	-14.7034	0.005856	0.037304
SCE1572_11595	-1.86116471	-9.84115	0.005908	0.037616
SCE1572_9297	-1.92963497	-13.3644	0.005923	0.037687
SCE1572_7463	-1.892568324	-12.6687	0.005944	0.037788
SCE1572_7819	-1.881897314	-11.9166	0.005948	0.037788
SCE1572_9224	2.209703203	-15.368	0.005956	0.037788
SCE1572_5682	-2.104313501	-15.277	0.005956	0.037788
SCE1572_852	-1.870783416	-11.2517	0.005957	0.037788
SCE1572_1829	-1.867817994	-10.9603	0.005969	0.037842
SCE1572_7901	2.277013138	-15.5973	0.005981	0.037851
SCE1572_10376	2.264629413	-15.6035	0.005981	0.037851
SCE1572_5043	-2.107924755	-15.4679	0.005981	0.037851
SCE1572_5514	2.372432703	-15.8716	0.005997	0.037885
SCE1572_2151	2.357933133	-15.8788	0.005997	0.037885
SCE1572_6557	2.357933133	-15.8788	0.005997	0.037885
SCE1572_1107	2.16153592	-15.0701	0.006023	0.038026
SCE1572_10439	-1.88130362	-12.2236	0.006046	0.03815
SCE1572_2921	2.012608	-14.2966	0.006065	0.038242
SCE1572_2420	-1.855243259	-10.0989	0.006103	0.038271
SCE1572_6109	2.635467109	-16.74	0.006131	0.038271
SCE1572_11113	2.611219562	-16.7522	0.006131	0.038271
SCE1572_5005	32.77431663	-33.6289	0.006149	0.038271
SCE1572_6314	32.77431663	-33.6289	0.006149	0.038271
SCE1572_1334	32.77431663	-33.6289	0.006149	0.038271
SCE1572_3218	32.77431663	-33.6289	0.006149	0.038271
SCE1572_8887	32.77431663	-33.6289	0.006149	0.038271
SCE1572_3201	32.77431663	-33.6289	0.006149	0.038271
SCE1572_8494	32.77431663	-33.6289	0.006149	0.038271

SCE1572_1912	32.77431663	-33.6289	0.006149	0.038271
SCE1572_3479	32.68685379	-33.6726	0.006149	0.038271
SCE1572_1181	32.68685379	-33.6726	0.006149	0.038271
SCE1572_3806	32.68685379	-33.6726	0.006149	0.038271
SCE1572_6977	32.68685379	-33.6726	0.006149	0.038271
SCE1572_9781	32.68685379	-33.6726	0.006149	0.038271
SCE1572_11497	32.68685379	-33.6726	0.006149	0.038271
SCE1572_8058	32.68685379	-33.6726	0.006149	0.038271
SCE1572_9463	32.68685379	-33.6726	0.006149	0.038271
SCE1572_1775	32.68685379	-33.6726	0.006149	0.038271
SCE1572_8426	32.68685379	-33.6726	0.006149	0.038271
SCE1572_6503	32.68685379	-33.6726	0.006149	0.038271
SCE1572_6498	-1.883907051	-12.5735	0.006163	0.038335
SCE1572_5378	2.082213468	-14.8467	0.006177	0.038396
SCE1572_6517	-2.315817606	-16.1713	0.006202	0.038507
SCE1572_10184	-2.228354765	-16.1276	0.006202	0.038507
SCE1572_3753	-1.909749213	-13.5288	0.00624	0.038713
SCE1572_11430	-1.869723787	-12.0589	0.006242	0.038713
SCE1572_4627	2.364165087	-16.0683	0.006406	0.039655
SCE1572_8050	2.364165087	-16.0683	0.006406	0.039655
SCE1572_6697	-2.197422906	-15.9086	0.006406	0.039655
SCE1572_2075	2.535931435	-16.4679	0.006447	0.039868
SCE1572_8776	2.535931435	-16.4679	0.006447	0.039868
SCE1572_7069	-1.933427024	-14.0587	0.006507	0.040193
SCE1572_9413	-1.952144971	-14.2657	0.006508	0.040193
SCE1572_34	-1.894056406	-13.4049	0.006513	0.040199
SCE1572_10790	-1.849341073	-11.5006	0.006518	0.040207
SCE1572_3451	2.343286357	-15.8861	0.006554	0.040382
SCE1572_2326	-2.140178954	-15.7355	0.006554	0.040382
SCE1572_9274	-1.878328541	-12.9471	0.006564	0.04042
SCE1572_5597	-1.873309291	-12.5724	0.006568	0.04042
SCE1572_2401	-1.892911864	-13.5124	0.006598	0.040581
SCE1572_10873	2.017337744	-14.5897	0.006623	0.040712
SCE1572_757	2.10054529	-14.92	0.006633	0.04075
SCE1572_2708	1.978803142	-14.024	0.006644	0.040794
SCE1572_4524	2.130674957	-15.1851	0.006689	0.041048
SCE1572_8242	-1.949495392	-14.3321	0.006717	0.041194
SCE1572_4258	-1.915279677	-13.7866	0.006722	0.041203
SCE1572_10423	-2.015584583	-14.9995	0.006742	0.041298
SCE1572_6702	2.036698808	-14.6471	0.006749	0.041303
SCE1572_10753	2.17603549	-15.5103	0.00675	0.041303
SCE1572_3113	1.959255545	-14.124	0.006807	0.041626
SCE1572_3687	2.058497399	-14.8586	0.006824	0.041657
SCE1572_6516	2.050504608	-14.8626	0.006824	0.041657
SCE1572_5541	-1.964762149	-14.7922	0.006824	0.041657
SCE1572_682	2.058077828	-14.7808	0.006857	0.041809
SCE1572_7829	-1.996563646	-14.7162	0.006857	0.041809
SCE1572_9392	-1.952893969	-14.6248	0.006885	0.041959

SCE1572_8607	-1.854610363	-12.3197	0.006904	0.04205
SCE1572_7679	-1.900240277	-13.6242	0.006942	0.042217
SCE1572_6459	2.372432703	-16.2866	0.006944	0.042217
SCE1572_777	2.372432703	-16.2866	0.006944	0.042217
SCE1572_2297	-1.877144548	-13.2821	0.006949	0.042222
SCE1572_6466	3.086128518	-17.5147	0.006975	0.042332
SCE1572_11320	3.086128518	-17.5147	0.006975	0.042332
SCE1572_1975	-1.878221789	-13.1327	0.006981	0.042346
SCE1572_1972	-1.892911864	-13.6823	0.007011	0.042501
SCE1572_6083	2.022759617	-14.6541	0.007051	0.042687
SCE1572_11081	-1.875803442	-13.4488	0.007052	0.042687
SCE1572_6224	-1.905295588	-13.9336	0.007056	0.042687
SCE1572_5829	2.586557508	-16.7645	0.007066	0.042687
SCE1572_11165	2.586557508	-16.7645	0.007066	0.042687
SCE1572_4739	2.115599636	-15.2995	0.007074	0.042687
SCE1572_4427	2.347486346	-16.0767	0.007078	0.042687
SCE1572_4669	2.330612527	-16.0851	0.007078	0.042687
SCE1572_7995	2.330612527	-16.0851	0.007078	0.042687
SCE1572_2133	1.932448846	-13.6992	0.007099	0.042788
SCE1572_2662	-2.016166432	-15.1117	0.007108	0.04282
SCE1572_5191	-1.843124088	-12.4133	0.007132	0.042937
SCE1572_6264	2.328489355	-15.8935	0.007167	0.043123
SCE1572_10869	2.042467289	-14.8666	0.007175	0.043144
SCE1572_6300	-1.88933641	-13.7263	0.007178	0.043144
SCE1572_4365	1.990244083	-14.6032	0.007212	0.043322
SCE1572_2310	-1.868664318	-13.1095	0.007219	0.043339
SCE1572_616	-1.898511463	-13.8881	0.007262	0.043563
SCE1572_5727	-1.893618894	-13.71	0.007269	0.043563
SCE1572_7877	2.163962658	-15.5164	0.007269	0.043563
SCE1572_8244	-1.826224017	-11.6512	0.007277	0.043565
SCE1572_8503	2.515172875	-16.4783	0.007282	0.043565
SCE1572_8617	2.494111259	-16.4888	0.007282	0.043565
SCE1572_3755	1.869936028	-12.8242	0.007328	0.043812
SCE1572_5789	-1.856385988	-12.9253	0.007332	0.043812
SCE1572_288	-2.023495974	-15.0695	0.007335	0.043812
SCE1572_3109	2.068426516	-15.0236	0.007346	0.043852
SCE1572_3011	2.075741899	-14.9324	0.007355	0.043852
SCE1572_6903	2.075741899	-14.9324	0.007355	0.043852
SCE1572_885	1.935734421	-14.0024	0.007366	0.043893
SCE1572_1370	-1.856385988	-13.2112	0.007407	0.044053
SCE1572_6400	-1.871107493	-13.3713	0.00742	0.044053
SCE1572_5972	33.21041575	-33.4108	0.007447	0.044053
SCE1572_10279	33.21041575	-33.4108	0.007447	0.044053
SCE1572_8532	33.14628541	-33.4429	0.007447	0.044053
SCE1572_1587	33.14628541	-33.4429	0.007447	0.044053
SCE1572_10691	33.14628541	-33.4429	0.007447	0.044053
SCE1572_7911	2.84405373	-17.0508	0.007459	0.044053
SCE1572_5150	2.84405373	-17.0508	0.007459	0.044053

SCE1572_5627	2.84405373	-17.0508	0.007459	0.044053
SCE1572_6819	2.84405373	-17.0508	0.007459	0.044053
SCE1572_9604	2.816039354	-17.0648	0.007459	0.044053
SCE1572_7676	2.816039354	-17.0648	0.007459	0.044053
SCE1572_5750	2.816039354	-17.0648	0.007459	0.044053
SCE1572_2136	2.816039354	-17.0648	0.007459	0.044053
SCE1572_10308	2.816039354	-17.0648	0.007459	0.044053
SCE1572_4402	-2.164508283	-15.8546	0.007473	0.044063
SCE1572_1933	-1.907611311	-14.0004	0.007476	0.044063
SCE1572_1660	-1.832539246	-12.4822	0.007477	0.044063
SCE1572_5246	-1.824928556	-12.0073	0.007478	0.044063
SCE1572_8282	-1.831232511	-12.3368	0.007494	0.044065
SCE1572_5306	-1.81313279	-10.9489	0.007495	0.044065
SCE1572_7003	1.995363054	-14.4751	0.007515	0.044065
SCE1572_1281	1.995363054	-14.4751	0.007515	0.044065
SCE1572_321	3.586557508	-18.2645	0.007531	0.044065
SCE1572_2622	3.586557508	-18.2645	0.007531	0.044065
SCE1572_2172	3.586557508	-18.2645	0.007531	0.044065
SCE1572_3388	3.586557508	-18.2645	0.007531	0.044065
SCE1572_9083	3.586557508	-18.2645	0.007531	0.044065
SCE1572_4952	3.535931435	-18.2898	0.007531	0.044065
SCE1572_8520	3.535931435	-18.2898	0.007531	0.044065
SCE1572_3573	3.535931435	-18.2898	0.007531	0.044065
SCE1572_3664	2.21400334	-15.6288	0.007537	0.044065
SCE1572_11279	-2.049031066	-15.4973	0.007537	0.044065
SCE1572_7165	2.026257062	-14.8747	0.007545	0.044091
SCE1572_11512	-1.804788254	-10.2382	0.007552	0.044102
SCE1572_4432	2.091146592	-15.2048	0.007556	0.044102
SCE1572_3568	1.929455709	-14.0557	0.007592	0.044291
SCE1572_1270	-1.846401899	-13.2261	0.007608	0.044358
SCE1572_10927	-1.838652902	-12.8324	0.007634	0.044485
SCE1572_12	-1.837456259	-12.9267	0.007658	0.044601
SCE1572_6706	-1.806825939	-11.1684	0.007698	0.044807
SCE1572_247	2.033887616	-14.751	0.007714	0.044876
SCE1572_3459	1.797344228	-9.58357	0.007763	0.045135
SCE1572_8155	2.353067378	-16.2963	0.007783	0.045179
SCE1572_10302	2.333438571	-16.3061	0.007783	0.045179
SCE1572_4060	-2.271423487	-16.1935	0.007783	0.045179
SCE1572_8797	2.132298699	-15.4067	0.007816	0.045309
SCE1572_2356	-1.834852826	-12.9199	0.007822	0.045309
SCE1572_6896	-1.861047365	-13.7122	0.007826	0.045309
SCE1572_4636	2.313539014	-16.0937	0.007828	0.045309
SCE1572_8170	2.313539014	-16.0937	0.007828	0.045309
SCE1572_4188	-1.852922132	-13.2838	0.007831	0.045309
SCE1572_3429	2.298432121	-15.9086	0.007842	0.045321
SCE1572_10692	-2.105413536	-15.7529	0.007842	0.045321
SCE1572_7226	-1.876285545	-13.8061	0.007858	0.045341
SCE1572_5432	1.976504026	-14.6101	0.007859	0.045341

SCE1572_7919	1.976504026	-14.6101	0.007859	0.045341
SCE1572_6205	1.936684708	-14.2315	0.007884	0.045464
SCE1572_2101	-1.917786532	-14.2618	0.007907	0.045547
SCE1572_2779	-1.820508951	-12.2641	0.007907	0.045547
SCE1572_8241	-1.900780107	-14.3788	0.007956	0.045804
SCE1572_11482	-1.89945471	-14.1127	0.00798	0.045916
SCE1572_862	-1.838683986	-13.0523	0.007998	0.045995
SCE1572_3368	-1.799052813	-11.3555	0.008011	0.046027
SCE1572_6633	1.818270901	-11.9007	0.008012	0.046027
SCE1572_11200	-1.856385988	-13.6196	0.008065	0.046302
SCE1572_2395	-1.881476969	-14.1405	0.008124	0.046617
SCE1572_8693	2.201064284	-15.6353	0.008149	0.046686
SCE1572_8480	-1.88895385	-14.1949	0.008156	0.046686
SCE1572_6360	-1.88895385	-14.1949	0.008156	0.046686
SCE1572_2073	2.535931435	-16.7898	0.008158	0.046686
SCE1572_3227	2.535931435	-16.7898	0.008158	0.046686
SCE1572_9247	2.202507701	-15.7866	0.008188	0.046794
SCE1572_2704	-1.827816836	-12.8684	0.008188	0.046794
SCE1572_574	-1.904366017	-14.2685	0.00819	0.046794
SCE1572_3560	1.962631852	-14.617	0.008205	0.046827
SCE1572_11394	-1.907012061	-14.5683	0.008205	0.046827
SCE1572_1729	-2.127033578	-15.8734	0.008222	0.046896
SCE1572_6904	2.472737609	-16.4995	0.008234	0.046941
SCE1572_961	-1.838982942	-13.2908	0.008277	0.047161
SCE1572_8088	2.01203046	-14.8038	0.008297	0.04724
SCE1572_8621	-1.842172129	-13.3207	0.0083	0.04724
SCE1572_9113	-1.959479481	-15.1795	0.008314	0.047292
SCE1572_3344	-1.800265901	-12.054	0.008332	0.047371
SCE1572_10239	2.001595007	-14.8871	0.00835	0.047449
SCE1572_8176	1.927885321	-14.3389	0.008358	0.047449
SCE1572_3766	-1.914266904	-14.5392	0.008359	0.047449
SCE1572_6740	-1.861902993	-13.9553	0.008384	0.047562
SCE1572_6434	1.882777162	-13.7588	0.008429	0.047795
SCE1572_2402	-2.028152336	-15.4571	0.00844	0.047812
SCE1572_11114	-1.812419144	-12.7019	0.008446	0.047812
SCE1572_4527	-1.83005184	-13.4029	0.008446	0.047812
SCE1572_9154	-1.824443095	-13.3189	0.008459	0.04782
SCE1572_5781	-1.804129751	-12.4441	0.008462	0.04782
SCE1572_6735	-1.819434852	-13.2396	0.00847	0.04782
SCE1572_175	3.050504608	-17.5325	0.008475	0.04782
SCE1572_8115	3.013978732	-17.5508	0.008475	0.04782
SCE1572_6617	3.013978732	-17.5508	0.008475	0.04782
SCE1572_8831	-1.805184907	-12.5257	0.008489	0.047873
SCE1572_1315	-1.896575078	-14.4983	0.008509	0.047961
SCE1572_8202	1.894162579	-13.9012	0.008542	0.048123
SCE1572_1106	2.283165365	-15.9162	0.008588	0.048327
SCE1572_3188	2.267735324	-15.9239	0.008588	0.048327
SCE1572_10102	-1.901055037	-14.6508	0.008634	0.048564

SCE1572_8569	2.296261022	-16.1023	0.008664	0.048655
SCE1572_8090	2.296261022	-16.1023	0.008664	0.048655
SCE1572_8194	2.278773596	-16.111	0.008664	0.048655
SCE1572_7536	1.919718	-14.191	0.008674	0.048683
SCE1572_6017	-1.784436146	-11.9056	0.008709	0.048852
SCE1572_1342	-1.790422725	-11.9502	0.008723	0.048889
SCE1572_5937	2.313539014	-16.316	0.008734	0.048889
SCE1572_6596	2.313539014	-16.316	0.008734	0.048889
SCE1572_2039	-2.225619797	-16.2164	0.008734	0.048889
SCE1572_2869	-2.325871271	-16.4658	0.008776	0.049096
SCE1572_11281	1.972883711	-14.679	0.008794	0.049151
SCE1572_3728	1.963792975	-14.4909	0.008795	0.049151
SCE1572_3636	2.174832743	-15.6484	0.008816	0.04919
SCE1572_1069	2.174832743	-15.6484	0.008816	0.04919
SCE1572_8674	-2.071124837	-15.5388	0.008816	0.04919
SCE1572_634	-1.800844424	-12.7634	0.008886	0.049556
SCE1572_10492	-1.862460502	-14.0942	0.008899	0.049564
SCE1572_3756	1.847242295	-13.4538	0.0089	0.049564
SCE1572_850	-1.761738551	-9.27153	0.008902	0.049564
SCE1572_10377	2.173361356	-15.8012	0.008928	0.049633
SCE1572_9644	-2.078778409	-15.7048	0.008928	0.049633
SCE1572_9670	-1.819486405	-13.3751	0.008928	0.049633
SCE1572_4368	-1.915279677	-14.7866	0.008966	0.049812
SCE1572_11274	-1.814785083	-13.4106	0.00897	0.049812
SCE1572_844	-1.785477611	-12.3175	0.009014	0.050033
SCE1572_5902	1.806579025	-12.6626	0.009019	0.050034
SCE1572_7535	1.779579416	-11.5386	0.009033	0.050082
SCE1572_38	-1.786580629	-12.4875	0.009059	0.050179
SCE1572_11412	-1.824501927	-13.4862	0.00906	0.050179
SCE1572_3247	-1.828371612	-13.7146	0.009168	0.050539
SCE1572_3861	-1.765382789	-11.2716	0.009172	0.050539
SCE1572_11293	-1.776546116	-12.2561	0.009183	0.050539
SCE1572_1394	-1.850285788	-14.1003	0.009186	0.050539
SCE1572_1412	-1.965320359	-15.2591	0.009222	0.050539
SCE1572_8350	32.64569646	-33.6932	0.009224	0.050539
SCE1572_6081	32.59374439	-33.7192	0.009224	0.050539
SCE1572_1652	32.59374439	-33.7192	0.009224	0.050539
SCE1572_1708	32.59374439	-33.7192	0.009224	0.050539
SCE1572_4198	32.59374439	-33.7192	0.009224	0.050539
SCE1572_9946	32.59374439	-33.7192	0.009224	0.050539
SCE1572_965	32.59374439	-33.7192	0.009224	0.050539
SCE1572_3055	32.59374439	-33.7192	0.009224	0.050539
SCE1572_10933	32.59374439	-33.7192	0.009224	0.050539
SCE1572_9185	32.59374439	-33.7192	0.009224	0.050539
SCE1572_10337	32.59374439	-33.7192	0.009224	0.050539
SCE1572_9701	32.59374439	-33.7192	0.009224	0.050539
SCE1572_3915	32.59374439	-33.7192	0.009224	0.050539
SCE1572_2531	32.59374439	-33.7192	0.009224	0.050539

SCE1572_7584	32.59374439	-33.7192	0.009224	0.050539
SCE1572_5041	-1.802614731	-13.0703	0.009226	0.050539
SCE1572_3231	-1.863143354	-14.2479	0.009232	0.050545
SCE1572_1498	2.187024843	-15.7408	0.009245	0.050567
SCE1572_1616	-2.030415388	-15.6136	0.009245	0.050567
SCE1572_8108	1.945807229	-14.7629	0.009304	0.050861
SCE1572_9900	-1.766078154	-11.7216	0.009309	0.050862
SCE1572_5484	2.466937115	-16.5286	0.009323	0.050862
SCE1572_995	2.451042537	-16.5103	0.009323	0.050862
SCE1572_6428	2.451042537	-16.5103	0.009323	0.050862
SCE1572_2934	-1.800608531	-13.0452	0.009332	0.050882
SCE1572_5415	1.825103437	-13.3379	0.009365	0.051038
SCE1572_10284	-1.888094848	-14.4783	0.009385	0.051119
SCE1572_1739	2.509936226	-16.8028	0.009436	0.051238
SCE1572_8368	2.509936226	-16.8028	0.009436	0.051238
SCE1572_4551	2.509936226	-16.8028	0.009436	0.051238
SCE1572_3882	2.483464015	-16.816	0.009436	0.051238
SCE1572_10079	2.483464015	-16.816	0.009436	0.051238
SCE1572_7988	2.483464015	-16.816	0.009436	0.051238
SCE1572_6398	-1.910833772	-14.6733	0.009464	0.051338
SCE1572_3799	-1.778141677	-12.6522	0.009464	0.051338
SCE1572_8590	-1.809637729	-13.5789	0.009502	0.051515
SCE1572_8826	1.995654262	-15.1113	0.009515	0.051563
SCE1572_5	-1.781961147	-12.9544	0.009542	0.05168
SCE1572_18	-1.779285019	-12.5508	0.009554	0.051721
SCE1572_7580	-1.908853408	-14.8829	0.009559	0.051721
SCE1572_7353	1.89357727	-14.2041	0.009576	0.051783
SCE1572_304	1.809288312	-13.1759	0.009675	0.052276
SCE1572_11542	2.029746048	-15.2355	0.009677	0.052276
SCE1572_4797	-1.801156467	-13.2789	0.009727	0.052508
SCE1572_11400	-1.78415766	-13.1425	0.00973	0.052508
SCE1572_3402	-1.917786532	-14.8467	0.009737	0.052509
SCE1572_3458	2.143614012	-15.816	0.009741	0.052509
SCE1572_10978	-1.755025273	-11.6315	0.009754	0.052509
SCE1572_9932	3.483464015	-18.316	0.009775	0.052509
SCE1572_10999	3.483464015	-18.316	0.009775	0.052509
SCE1572_7192	3.483464015	-18.316	0.009775	0.052509
SCE1572_2746	3.483464015	-18.316	0.009775	0.052509
SCE1572_11502	3.483464015	-18.316	0.009775	0.052509
SCE1572_6978	3.483464015	-18.316	0.009775	0.052509
SCE1572_1197	1.813384901	-13.1511	0.009784	0.052514
SCE1572_4980	-1.842580188	-14.2786	0.009786	0.052514
SCE1572_1354	2.293361132	-16.3261	0.009812	0.05258
SCE1572_7883	2.272897029	-16.3364	0.009812	0.05258
SCE1572_7693	-1.758065727	-12.045	0.009813	0.05258
SCE1572_5663	2.076499816	-15.5601	0.009819	0.052584
SCE1572_8830	-1.773465028	-12.9111	0.009873	0.052849
SCE1572_565	-1.810092335	-13.8543	0.009882	0.05286

SCE1572_1065	1.981557254	-15.1601	0.009887	0.05286
SCE1572_3887	-1.827816836	-14.0059	0.00989	0.05286
SCE1572_8227	-1.856385988	-14.4468	0.009909	0.05293
SCE1572_3292	-1.813572904	-13.5895	0.009941	0.053073
SCE1572_9412	-1.815744003	-13.8364	0.009969	0.0532
SCE1572_5630	-1.765532557	-12.7117	0.009981	0.053237
SCE1572_9503	-1.742566088	-11.278	0.009996	0.05329
SCE1572_5701	-2.164508283	-16.4396	0.010042	0.053504
SCE1572_10868	1.998665676	-14.971	0.010081	0.053669
SCE1572_2230	-1.757667937	-12.4731	0.010087	0.053669
SCE1572_7066	2.021358262	-15.1402	0.010088	0.053669
SCE1572_1068	33.07917121	-33.4765	0.010215	0.054236
SCE1572_10228	33.07917121	-33.4765	0.010215	0.054236
SCE1572_4548	33.07917121	-33.4765	0.010215	0.054236
SCE1572_112	33.04229299	-33.4949	0.010215	0.054236
SCE1572_9669	-1.866581755	-14.8413	0.010253	0.054316
SCE1572_9111	2.758323856	-17.0937	0.010256	0.054316
SCE1572_4666	2.758323856	-17.0937	0.010256	0.054316
SCE1572_11309	2.728576513	-17.1085	0.010256	0.054316
SCE1572_4612	2.728576513	-17.1085	0.010256	0.054316
SCE1572_11537	-1.772821271	-13.176	0.010302	0.054523
SCE1572_6742	2.236371153	-15.9396	0.010319	0.054523
SCE1572_5543	2.976504026	-17.5695	0.010331	0.054523
SCE1572_11369	2.976504026	-17.5695	0.010331	0.054523
SCE1572_1408	2.938029879	-17.5888	0.010331	0.054523
SCE1572_7975	2.938029879	-17.5888	0.010331	0.054523
SCE1572_3684	2.938029879	-17.5888	0.010331	0.054523
SCE1572_7762	-1.864798228	-14.5639	0.010345	0.054568
SCE1572_6636	1.856681339	-14.0002	0.010376	0.054704
SCE1572_2914	1.740627088	-11.2703	0.010403	0.054819
SCE1572_10643	-1.766188179	-12.8167	0.010408	0.054819
SCE1572_5095	-1.766950904	-12.93	0.010414	0.054821
SCE1572_106	1.96123727	-15.1703	0.010505	0.055275
SCE1572_7477	-1.938848148	-15.2723	0.010552	0.055494
SCE1572_5156	2.406648418	-16.5325	0.01057	0.055506
SCE1572_6211	2.406648418	-16.5325	0.01057	0.055506
SCE1572_1209	2.406648418	-16.5325	0.01057	0.055506
SCE1572_7868	-1.768099455	-13.2553	0.010583	0.055543
SCE1572_1996	2.050504608	-15.5731	0.010598	0.055568
SCE1572_5896	2.050504608	-15.5731	0.010598	0.055568
SCE1572_10941	2.12850712	-15.8236	0.010635	0.055639
SCE1572_9692	-1.985119302	-15.6928	0.010635	0.055639
SCE1572_1939	-1.787489233	-13.8853	0.010638	0.055639
SCE1572_3472	2.243149686	-16.1288	0.010643	0.055639
SCE1572_3683	2.243149686	-16.1288	0.010643	0.055639
SCE1572_8387	2.243149686	-16.1288	0.010643	0.055639
SCE1572_10595	1.891196714	-14.6527	0.010666	0.055728
SCE1572_7714	-1.841739212	-14.3639	0.010678	0.055757

SCE1572_7873	-1.806200231	-14.1407	0.010682	0.055757
SCE1572_664	-1.73486131	-11.7305	0.01069	0.055772
SCE1572_5083	-1.797969302	-14.0379	0.010729	0.055922
SCE1572_8652	-1.778383476	-13.3847	0.010729	0.055922
SCE1572_366	-1.771852478	-13.5008	0.010738	0.055938
SCE1572_9943	-1.764236141	-13.0634	0.010756	0.056005
SCE1572_653	-1.727444478	-11.1987	0.010761	0.056005
SCE1572_10205	2.026312258	-15.3124	0.010768	0.056014
SCE1572_2670	1.942355804	-14.9167	0.010799	0.056148
SCE1572_9679	-1.747002528	-12.7415	0.010825	0.056255
SCE1572_8940	-1.765620524	-13.0888	0.010833	0.056269
SCE1572_8969	-1.931937021	-15.409	0.010846	0.056305
SCE1572_5267	-1.781372633	-13.4727	0.010864	0.056374
SCE1572_10002	-1.745579202	-12.4508	0.010881	0.056434
SCE1572_7461	-1.716154503	-10.3757	0.010895	0.05648
SCE1572_6166	-1.754798116	-12.8007	0.010916	0.05656
SCE1572_7703	-1.997248524	-15.6301	0.010926	0.056566
SCE1572_49	2.456496968	-16.8295	0.010934	0.056566
SCE1572_5694	2.456496968	-16.8295	0.010934	0.056566
SCE1572_1466	2.008684432	-15.2461	0.010972	0.056739
SCE1572_7412	-1.822116086	-14.2684	0.01101	0.056878
SCE1572_2866	1.771011779	-13.1062	0.01101	0.056878
SCE1572_9961	2.252138469	-16.3467	0.011035	0.056895
SCE1572_185	2.252138469	-16.3467	0.011035	0.056895
SCE1572_7041	2.231076853	-16.3573	0.011035	0.056895
SCE1572_8303	-2.090851241	-16.1963	0.011035	0.056895
SCE1572_4981	-1.889552852	-14.9582	0.011054	0.056965
SCE1572_5892	-1.943848829	-15.3573	0.011077	0.057049
SCE1572_9718	-1.75348608	-12.9289	0.011081	0.057049
SCE1572_2913	1.753147938	-12.6097	0.011093	0.057081
SCE1572_5591	1.883854738	-14.6564	0.011148	0.057285
SCE1572_8271	-1.865103205	-14.6153	0.011148	0.057285
SCE1572_8554	-1.730855106	-12.3763	0.011149	0.057285
SCE1572_6723	-1.780437135	-13.7385	0.011186	0.05744
SCE1572_3042	2.107088136	-15.6823	0.011195	0.05744
SCE1572_6104	2.107088136	-15.6823	0.011195	0.05744
SCE1572_2438	-1.867033232	-14.9038	0.011219	0.057535
SCE1572_940	1.83710097	-14.1392	0.011227	0.057548
SCE1572_2920	1.982333105	-15.3662	0.011294	0.057862
SCE1572_3215	-1.713021813	-11.2632	0.011302	0.057876
SCE1572_1185	2.204309944	-15.9556	0.011323	0.057934
SCE1572_3828	-1.793650232	-13.8778	0.011325	0.057934
SCE1572_8759	-1.729221558	-12.5517	0.011383	0.058206
SCE1572_528	-1.828905251	-14.6868	0.011393	0.058229
SCE1572_9066	1.863736096	-14.3186	0.011408	0.058275
SCE1572_5917	-1.748176771	-13.2952	0.011487	0.058649
SCE1572_110	-1.71972344	-12.0473	0.011493	0.058649
SCE1572_8629	-1.882858199	-15.2178	0.011498	0.058649

SCE1572_8873	1.947745033	-15.0839	0.01152	0.058733
SCE1572_4630	1.791312268	-13.6703	0.011529	0.058733
SCE1572_11449	-1.729822026	-12.4756	0.011531	0.058733
SCE1572_10363	-1.740455876	-12.9914	0.011542	0.05876
SCE1572_3036	2.113240363	-15.8312	0.011618	0.059077
SCE1572_9665	-2.008389081	-15.74	0.011618	0.059077
SCE1572_8111	1.850205957	-14.3778	0.011621	0.059077
SCE1572_6232	1.885080999	-14.5303	0.011638	0.059104
SCE1572_9177	1.885080999	-14.5303	0.011638	0.059104
SCE1572_7674	1.92541544	-14.8471	0.011671	0.059234
SCE1572_6324	-1.760627005	-13.6807	0.011674	0.059234
SCE1572_3931	-1.70236119	-10.8949	0.011723	0.059453
SCE1572_8639	-1.779570391	-13.9476	0.011737	0.059494
SCE1572_5623	-1.7652381	-13.8319	0.011787	0.059719
SCE1572_6333	-1.845659581	-14.9145	0.01184	0.059955
SCE1572_1932	1.816039354	-14.0623	0.011845	0.059955
SCE1572_1300	1.930210374	-15.1858	0.01187	0.060051
SCE1572_2784	2.038431776	-15.4536	0.011892	0.060133
SCE1572_1005	1.805211177	-14.0321	0.011926	0.060241
SCE1572_935	-1.76423736	-13.7926	0.011928	0.060241
SCE1572_3259	-1.729581203	-12.7779	0.01193	0.060241
SCE1572_11055	-1.748901561	-13.4324	0.01198	0.060462
SCE1572_4316	1.916203516	-14.9298	0.011988	0.060474
SCE1572_5349	2.360844728	-16.5554	0.012	0.060477
SCE1572_7431	2.360844728	-16.5554	0.012	0.060477
SCE1572_2323	-1.800117768	-14.4984	0.01201	0.060497
SCE1572_5823	2.698202864	-17.1237	0.012065	0.060748
SCE1572_10242	1.9706513	-15.372	0.012093	0.060858
SCE1572_10469	-1.836208106	-14.8261	0.012129	0.06101
SCE1572_6330	-1.759088786	-13.9261	0.012155	0.061111
SCE1572_2464	1.861601932	-14.6675	0.012186	0.061225
SCE1572_2712	-1.700951989	-11.761	0.012189	0.061225
SCE1572_3767	-1.856385988	-15.0087	0.012201	0.061255
SCE1572_9963	1.745308288	-12.9706	0.012232	0.061384
SCE1572_10452	-1.764463498	-14.1071	0.012354	0.061964
SCE1572_6991	2.024032396	-15.5863	0.012365	0.061982
SCE1572_6776	1.718442136	-12.2328	0.012371	0.061982
SCE1572_6240	1.772970632	-13.5863	0.012375	0.061982
SCE1572_3150	-1.827816836	-14.7429	0.012392	0.062035
SCE1572_2915	1.707424072	-12.0142	0.012407	0.062041
SCE1572_4874	-1.687391457	-10.8875	0.01242	0.062041
SCE1572_8154	2.209703203	-16.368	0.012425	0.062041
SCE1572_10459	-2.041941641	-16.2208	0.012425	0.062041
SCE1572_5207	-1.771852478	-14.0857	0.012434	0.062041
SCE1572_5967	2.171520009	-15.972	0.012434	0.062041
SCE1572_313	2.171520009	-15.972	0.012434	0.062041
SCE1572_5755	-1.703217215	-12.1512	0.012476	0.062221
SCE1572_4624	-1.751416428	-13.6206	0.012489	0.062254

SCE1572_1409	1.777335825	-13.8472	0.0125	0.062281
SCE1572_3478	-1.778383476	-14.1921	0.012516	0.062331
SCE1572_957	-1.716362472	-12.8936	0.012589	0.062667
SCE1572_10332	1.780897548	-13.7079	0.012612	0.062672
SCE1572_8322	-1.881476969	-15.1405	0.012623	0.062672
SCE1572_8591	1.88315355	-14.7239	0.012631	0.062672
SCE1572_3284	2.898501514	-17.6085	0.012638	0.062672
SCE1572_2780	2.898501514	-17.6085	0.012638	0.062672
SCE1572_11124	2.898501514	-17.6085	0.012638	0.062672
SCE1572_6551	2.898501514	-17.6085	0.012638	0.062672
SCE1572_4116	2.898501514	-17.6085	0.012638	0.062672
SCE1572_6449	1.733577194	-13.0211	0.012645	0.062676
SCE1572_120	2.401001855	-16.8573	0.012692	0.062851
SCE1572_6497	2.401001855	-16.8573	0.012692	0.062851
SCE1572_5927	1.787470202	-13.7717	0.012747	0.063006
SCE1572_4282	3.429016231	-18.3433	0.012767	0.063006
SCE1572_4553	3.429016231	-18.3433	0.012767	0.063006
SCE1572_5644	3.429016231	-18.3433	0.012767	0.063006
SCE1572_10440	3.372432703	-18.3716	0.012767	0.063006
SCE1572_5840	3.372432703	-18.3716	0.012767	0.063006
SCE1572_7748	3.372432703	-18.3716	0.012767	0.063006
SCE1572_2342	-1.870885557	-15.3494	0.012771	0.063006
SCE1572_4245	-1.813572904	-14.5895	0.012851	0.063368
SCE1572_854	-1.716034102	-13.0703	0.01286	0.063368
SCE1572_6343	-1.745354675	-13.7016	0.012863	0.063368
SCE1572_3579	-1.76496296	-14.1988	0.012947	0.063669
SCE1572_6955	1.947000032	-15.3838	0.012953	0.063669
SCE1572_5939	1.947000032	-15.3838	0.012953	0.063669
SCE1572_260	1.945747792	-15.3789	0.012953	0.063669
SCE1572_8038	1.856066248	-14.6062	0.012954	0.063669
SCE1572_6793	-1.708722598	-12.7675	0.012968	0.063706
SCE1572_9684	-1.690799922	-12.1088	0.013043	0.064044
SCE1572_4806	-1.689390317	-11.957	0.01305	0.064052
SCE1572_4268	1.779202586	-13.7759	0.01307	0.06412
SCE1572_545	2.169149104	-16.1658	0.013121	0.064285
SCE1572_3083	2.169149104	-16.1658	0.013121	0.064285
SCE1572_1532	-1.856385988	-15.2311	0.013122	0.064285
SCE1572_1240	1.755188243	-13.6256	0.01313	0.064291
SCE1572_5615	2.064859901	-15.7034	0.013163	0.064422
SCE1572_2454	-1.706001173	-13.1099	0.013173	0.064422
SCE1572_9923	-1.71639827	-13.1949	0.013175	0.064422
SCE1572_3117	-1.700735117	-13.0194	0.013205	0.06453
SCE1572_7434	-2.107924755	-16.4679	0.013209	0.06453
SCE1572_6754	1.82618731	-14.5008	0.013239	0.064647
SCE1572_3487	1.954644592	-15.2731	0.01328	0.064787
SCE1572_6589	1.943589404	-15.2786	0.01328	0.064787
SCE1572_8957	-1.724474312	-13.7805	0.013293	0.064791
SCE1572_9209	-1.724474312	-13.7805	0.013293	0.064791

SCE1572_583	-1.835324372	-14.888	0.013319	0.064862
SCE1572_11474	1.803715583	-14.2718	0.01332	0.064862
SCE1572_1257	-1.73499088	-13.8918	0.013363	0.065038
SCE1572_10042	-1.66374091	-10.2879	0.013419	0.065285
SCE1572_3267	-1.719697316	-13.5867	0.013481	0.065552
SCE1572_3827	-1.768923147	-14.3573	0.013493	0.06556
SCE1572_5008	-1.688092072	-12.6973	0.013495	0.06556
SCE1572_2458	-1.725333878	-13.5717	0.013527	0.065686
SCE1572_4317	2.698559746	-17.3411	0.013538	0.06571
SCE1572_1509	1.881528436	-14.8691	0.013548	0.065729
SCE1572_3278	-1.777608874	-14.3319	0.013571	0.065811
SCE1572_3246	-1.674614743	-11.7504	0.013606	0.06595
SCE1572_214	2.337385756	-16.5672	0.013641	0.066047
SCE1572_3661	2.337385756	-16.5672	0.013641	0.066047
SCE1572_1020	-1.823025714	-14.7649	0.013654	0.066047
SCE1572_3588	2.154841268	-15.9804	0.013664	0.066047
SCE1572_3609	2.137967449	-15.9888	0.013664	0.066047
SCE1572_3623	2.137967449	-15.9888	0.013664	0.066047
SCE1572_7979	1.751909017	-13.6583	0.013699	0.066184
SCE1572_5620	1.789518369	-14.305	0.01372	0.066234
SCE1572_11167	-1.886133331	-15.3861	0.013722	0.066234
SCE1572_5065	-1.735370587	-14.0186	0.013854	0.06684
SCE1572_8993	-1.683401187	-12.6049	0.013895	0.067004
SCE1572_6029	-1.692459727	-12.9911	0.01392	0.067004
SCE1572_9539	2.188008132	-16.3788	0.014007	0.067004
SCE1572_5433	2.165981825	-16.3898	0.014007	0.067004
SCE1572_4363	2.165981825	-16.3898	0.014007	0.067004
SCE1572_5099	2.165981825	-16.3898	0.014007	0.067004
SCE1572_10232	2.165981825	-16.3898	0.014007	0.067004
SCE1572_6823	-1.696729322	-13.3516	0.014051	0.067004
SCE1572_752	32.49420871	-33.7689	0.014079	0.067004
SCE1572_9427	32.49420871	-33.7689	0.014079	0.067004
SCE1572_4224	32.49420871	-33.7689	0.014079	0.067004
SCE1572_7961	32.49420871	-33.7689	0.014079	0.067004
SCE1572_8956	32.49420871	-33.7689	0.014079	0.067004
SCE1572_173	32.49420871	-33.7689	0.014079	0.067004
SCE1572_4523	32.49420871	-33.7689	0.014079	0.067004
SCE1572_4374	32.49420871	-33.7689	0.014079	0.067004
SCE1572_3590	32.49420871	-33.7689	0.014079	0.067004
SCE1572_8128	32.49420871	-33.7689	0.014079	0.067004
SCE1572_9858	32.38729351	-33.8224	0.014079	0.067004
SCE1572_4257	32.38729351	-33.8224	0.014079	0.067004
SCE1572_9896	32.38729351	-33.8224	0.014079	0.067004
SCE1572_7171	32.38729351	-33.8224	0.014079	0.067004
SCE1572_7052	32.38729351	-33.8224	0.014079	0.067004
SCE1572_2897	32.38729351	-33.8224	0.014079	0.067004
SCE1572_3190	32.38729351	-33.8224	0.014079	0.067004
SCE1572_7947	32.38729351	-33.8224	0.014079	0.067004

SCE1572_7973	32.38729351	-33.8224	0.014079	0.067004
SCE1572_11199	32.38729351	-33.8224	0.014079	0.067004
SCE1572_6521	32.38729351	-33.8224	0.014079	0.067004
SCE1572_1154	32.37055024	-33.8308	0.014079	0.067004
SCE1572_7967	33.00878188	-33.5117	0.014146	0.067142
SCE1572_10711	33.00878188	-33.5117	0.014146	0.067142
SCE1572_2570	33.00878188	-33.5117	0.014146	0.067142
SCE1572_500	33.00878188	-33.5117	0.014146	0.067142
SCE1572_9548	33.00878188	-33.5117	0.014146	0.067142
SCE1572_9666	-2.856385988	-18.316	0.014146	0.067142
SCE1572_3456	1.932448846	-15.2842	0.014162	0.067158
SCE1572_7304	1.932448846	-15.2842	0.014162	0.067158
SCE1572_1902	1.724891466	-13.5806	0.014186	0.067239
SCE1572_141	2.667803091	-17.1389	0.014225	0.067306
SCE1572_4958	2.667175968	-17.1392	0.014225	0.067306
SCE1572_1596	2.635467109	-17.1551	0.014225	0.067306
SCE1572_6082	2.635467109	-17.1551	0.014225	0.067306
SCE1572_1758	1.716702413	-13.2687	0.014283	0.067485
SCE1572_4541	1.713718554	-13.2702	0.014283	0.067485
SCE1572_10464	-1.94280074	-15.6029	0.014285	0.067485
SCE1572_10250	1.887775108	-15.207	0.014289	0.067485
SCE1572_10294	-1.683101974	-12.7395	0.014296	0.067491
SCE1572_349	-1.799802459	-14.7289	0.014425	0.068068
SCE1572_9250	1.762297131	-13.9672	0.01448	0.068267
SCE1572_5567	-1.720949363	-13.9296	0.01448	0.068267
SCE1572_9680	-1.650576451	-11.0693	0.014511	0.068381
SCE1572_9786	1.88844785	-15.1136	0.014518	0.068383
SCE1572_6000	-1.728061891	-13.9106	0.014527	0.068396
SCE1572_9436	-1.642798237	-10.136	0.014549	0.068437
SCE1572_5452	1.787470202	-14.4092	0.014549	0.068437
SCE1572_9925	-1.665852937	-12.3711	0.01457	0.068498
SCE1572_7872	-1.66483097	-12.5195	0.014575	0.068498
SCE1572_9330	-1.660835179	-12.0271	0.01461	0.068622
SCE1572_5706	-1.741874107	-14.0832	0.014618	0.068622
SCE1572_6056	-1.647571973	-11.0439	0.014621	0.068622
SCE1572_11023	-1.677415847	-12.8466	0.014653	0.068743
SCE1572_515	1.898501514	-15.0211	0.014719	0.069023
SCE1572_4572	1.976504026	-15.4846	0.014749	0.069094
SCE1572_11169	1.976504026	-15.4846	0.014749	0.069094
SCE1572_3531	2.372432703	-16.8716	0.014761	0.069094
SCE1572_4769	-2.145892605	-16.6713	0.014761	0.069094
SCE1572_5678	-1.662482123	-12.4052	0.014802	0.069258
SCE1572_4772	2.032357261	-16.0416	0.014821	0.069285
SCE1572_5544	2.032357261	-16.0416	0.014821	0.069285
SCE1572_10627	-1.681407759	-13.3286	0.014947	0.069839
SCE1572_3818	-1.675145673	-12.9067	0.014963	0.069884
SCE1572_5842	1.855688431	-14.882	0.01498	0.069932
SCE1572_7610	-1.692719977	-13.6374	0.015036	0.070163

SCE1572_11053	-1.647769598	-12.0175	0.015097	0.070416
SCE1572_4662	1.921221591	-15.2898	0.015107	0.070434
SCE1572_2102	-1.780437135	-14.7385	0.01513	0.070479
SCE1572_6722	-1.752956398	-14.7248	0.01513	0.070479
SCE1572_2227	-1.700266786	-13.7786	0.015168	0.070621
SCE1572_3695	-2.049031066	-16.4973	0.015186	0.070673
SCE1572_4443	-1.9343885	-15.777	0.015207	0.070741
SCE1572_5389	-1.642405806	-11.4527	0.01526	0.070958
SCE1572_2291	-1.647828327	-11.9778	0.015284	0.071037
SCE1572_7462	-1.671171115	-13.1186	0.015306	0.071078
SCE1572_4209	-1.669300435	-13.2851	0.015306	0.071078
SCE1572_2144	-1.760461568	-14.6161	0.015329	0.07115
SCE1572_3379	1.804927399	-14.6318	0.015388	0.071334
SCE1572_7153	1.804927399	-14.6318	0.015388	0.071334
SCE1572_1662	-1.770994497	-14.5857	0.015388	0.071334
SCE1572_5732	-1.679371562	-13.2321	0.015397	0.071342
SCE1572_5190	-1.673237291	-13.3945	0.015433	0.071477
SCE1572_3375	-1.733003572	-14.532	0.015445	0.071503
SCE1572_10879	-1.675001871	-13.1879	0.015461	0.071506
SCE1572_2418	-1.743491931	-14.5033	0.0155	0.071506
SCE1572_8169	2.021358262	-15.7252	0.01551	0.071506
SCE1572_1891	2.85785953	-17.6288	0.015514	0.071506
SCE1572_9138	2.85785953	-17.6288	0.015514	0.071506
SCE1572_9231	2.85785953	-17.6288	0.015514	0.071506
SCE1572_7750	2.816039354	-17.6498	0.015514	0.071506
SCE1572_3048	2.816039354	-17.6498	0.015514	0.071506
SCE1572_10649	2.816039354	-17.6498	0.015514	0.071506
SCE1572_9257	2.816039354	-17.6498	0.015514	0.071506
SCE1572_1791	2.313539014	-16.5791	0.015528	0.071539
SCE1572_1359	-1.64993511	-12.4274	0.015551	0.071586
SCE1572_8068	1.854386473	-15.0237	0.015552	0.071586
SCE1572_671	-1.74090877	-14.4364	0.015602	0.071783
SCE1572_5683	-1.645134124	-12.273	0.015619	0.071825
SCE1572_6396	-1.761228755	-14.894	0.015624	0.071825
SCE1572_3617	1.79632112	-14.4592	0.015649	0.071858
SCE1572_5329	1.789688027	-14.4625	0.015649	0.071858
SCE1572_3162	-1.661370005	-13.0556	0.015652	0.071858
SCE1572_8876	1.677592584	-13.0491	0.015686	0.071985
SCE1572_1979	-1.621341392	-9.69915	0.015704	0.072016
SCE1572_8264	-1.698433628	-13.9881	0.015707	0.072016
SCE1572_3432	-1.689649264	-13.6642	0.015731	0.072096
SCE1572_1282	-1.888094848	-15.4783	0.015754	0.072116
SCE1572_3888	1.846971214	-14.8864	0.015756	0.072116
SCE1572_55	1.661131992	-12.5268	0.015767	0.072116
SCE1572_6870	-1.659493939	-13.1596	0.015767	0.072116
SCE1572_1606	1.772970632	-14.3639	0.01578	0.072116
SCE1572_11298	-1.689566897	-13.6264	0.015782	0.072116
SCE1572_8751	1.672941814	-12.9734	0.015784	0.072116

SCE1572_2030	2.143614012	-16.401	0.015808	0.072164
SCE1572_8755	2.143614012	-16.401	0.015808	0.072164
SCE1572_5602	-1.768923147	-14.7723	0.015816	0.07217
SCE1572_5334	-1.641100453	-12.2094	0.015848	0.072281
SCE1572_5302	1.950968934	-15.4973	0.015859	0.072281
SCE1572_344	1.728576513	-13.9081	0.015861	0.072281
SCE1572_2749	-1.644547345	-12.6804	0.016119	0.073424
SCE1572_10178	-1.686460986	-13.6531	0.016131	0.07345
SCE1572_9732	1.743951739	-14.0565	0.01615	0.073504
SCE1572_10491	-1.632305216	-11.9432	0.016163	0.073529
SCE1572_6246	2.130674957	-16.1851	0.016233	0.073722
SCE1572_8366	2.130674957	-16.1851	0.016233	0.073722
SCE1572_3099	2.130674957	-16.1851	0.016233	0.073722
SCE1572_11308	2.11104615	-16.1949	0.016233	0.073722
SCE1572_8623	-1.669182995	-13.5173	0.016267	0.073846
SCE1572_1360	-1.648190969	-12.9277	0.016293	0.073931
SCE1572_4445	-1.66221344	-13.4213	0.016368	0.074174
SCE1572_7354	1.772470911	-14.3642	0.016368	0.074174
SCE1572_1021	1.766711642	-14.3671	0.016368	0.074174
SCE1572_2500	2.013978732	-16.0508	0.016397	0.07424
SCE1572_8632	-1.966568906	-15.9536	0.016397	0.07424
SCE1572_2513	-1.694922565	-14.0723	0.016601	0.075133
SCE1572_2912	1.633074131	-12.1414	0.016634	0.075252
SCE1572_6467	2.01808313	-15.8788	0.016656	0.075252
SCE1572_6425	2.01808313	-15.8788	0.016656	0.075252
SCE1572_2476	2.001595007	-15.8871	0.016656	0.075252
SCE1572_1390	-1.666908189	-13.4734	0.016664	0.075254
SCE1572_10012	-1.686460986	-13.9468	0.016701	0.075357
SCE1572_7853	-1.686460986	-13.9468	0.016701	0.075357
SCE1572_2148	1.776067312	-14.7103	0.016725	0.07542
SCE1572_912	-1.620927826	-11.6858	0.01673	0.07542
SCE1572_2261	-1.686460986	-13.901	0.016736	0.07542
SCE1572_7758	3.313539014	-18.401	0.016783	0.075565
SCE1572_11541	3.313539014	-18.401	0.016783	0.075565
SCE1572_6209	2.59378743	-17.1663	0.01681	0.075656
SCE1572_6987	1.991610919	-15.74	0.01685	0.075803
SCE1572_670	-1.676938212	-13.6578	0.016909	0.076037
SCE1572_9538	1.927356377	-15.6347	0.016929	0.076062
SCE1572_11362	1.927356377	-15.6347	0.016929	0.076062
SCE1572_7549	1.754111605	-14.3734	0.016979	0.076255
SCE1572_9353	-1.652513654	-13.4917	0.017006	0.076343
SCE1572_1679	-1.648277792	-13.439	0.017034	0.076435
SCE1572_9334	-1.695563193	-14.1776	0.017068	0.076553
SCE1572_7647	1.734201062	-14.1907	0.017083	0.07659
SCE1572_597	-1.641261567	-13.2418	0.017136	0.076792
SCE1572_8690	2.343286357	-16.8861	0.017199	0.076825
SCE1572_9649	2.343286357	-16.8861	0.017199	0.076825
SCE1572_1039	2.343286357	-16.8861	0.017199	0.076825

SCE1572_699	2.313539014	-16.901	0.017199	0.076825
SCE1572_7115	2.313539014	-16.901	0.017199	0.076825
SCE1572_8664	2.313539014	-16.901	0.017199	0.076825
SCE1572_851	-1.598058055	-9.73657	0.017199	0.076825
SCE1572_7673	1.893436594	-15.3089	0.017209	0.076825
SCE1572_481	1.887005876	-15.3069	0.017209	0.076825
SCE1572_6374	1.832913173	-15.2344	0.017248	0.07697
SCE1572_4403	-1.796514532	-15.0734	0.017317	0.077242
SCE1572_11271	-1.656340998	-13.5822	0.01734	0.077309
SCE1572_3276	-1.77589607	-15.015	0.017346	0.077309
SCE1572_9948	1.816039354	-14.9798	0.017398	0.077473
SCE1572_6799	1.816039354	-14.9798	0.017398	0.077473
SCE1572_11122	1.790352711	-14.8407	0.017461	0.077719
SCE1572_1336	-1.725989351	-14.7113	0.017495	0.077838
SCE1572_9673	-1.723935692	-14.6343	0.017524	0.077935
SCE1572_1643	1.636458311	-12.6396	0.017578	0.078145
SCE1572_10943	1.762846415	-14.4759	0.017591	0.078166
SCE1572_4419	1.734575254	-14.2836	0.017636	0.078336
SCE1572_5266	-1.682211513	-14.0787	0.017669	0.078401
SCE1572_9116	-1.608148584	-11.9252	0.01767	0.078401
SCE1572_10937	-1.690648783	-14.0576	0.017673	0.078401
SCE1572_9738	2.264629413	-16.6035	0.0177	0.078451
SCE1572_3974	-2.008389081	-16.4181	0.0177	0.078451
SCE1572_7506	1.678044396	-13.7636	0.017719	0.078503
SCE1572_762	1.649504942	-13.0042	0.017775	0.078717
SCE1572_440	1.61452017	-12.0411	0.017798	0.078787
SCE1572_5444	2.120893936	-16.4124	0.017862	0.079036
SCE1572_9886	-1.61934679	-12.6678	0.018003	0.079626
SCE1572_8201	1.656064013	-13.5573	0.01813	0.080156
SCE1572_5765	-1.64616828	-13.6235	0.018146	0.080162
SCE1572_8158	1.976504026	-16.0695	0.018154	0.080162
SCE1572_10282	-1.856385988	-15.9416	0.018154	0.080162
SCE1572_3020	1.691101808	-13.9268	0.018194	0.080305
SCE1572_5973	2.068426516	-16.0236	0.018211	0.08033
SCE1572_11396	-1.618013636	-12.6622	0.018219	0.08033
SCE1572_6054	-1.678048747	-14.0639	0.018226	0.08033
SCE1572_149	-1.66515048	-14.083	0.01823	0.08033
SCE1572_3482	1.984916266	-15.8954	0.018255	0.080354
SCE1572_10364	-1.837020663	-15.7668	0.018255	0.080354
SCE1572_2185	1.728576513	-14.2866	0.018261	0.080354
SCE1572_10180	-1.691326742	-14.2741	0.018266	0.080354
SCE1572_943	1.717933032	-14.371	0.018277	0.080368
SCE1572_8134	1.728576513	-14.4386	0.018289	0.080384
SCE1572_10375	1.976504026	-15.7476	0.018315	0.080397
SCE1572_3515	-1.87408799	-15.6373	0.018315	0.080397
SCE1572_7289	1.759603409	-14.6544	0.018323	0.080397
SCE1572_2995	1.752960672	-14.7219	0.018335	0.080397
SCE1572_4704	1.913001084	-15.6418	0.018335	0.080397

SCE1572_2630	1.765907391	-14.7675	0.01834	0.080397
SCE1572_3973	-1.74090877	-14.7583	0.018346	0.080397
SCE1572_285	1.911798337	-15.5169	0.018361	0.080397
SCE1572_942	1.797056251	-14.9893	0.018365	0.080397
SCE1572_11496	1.848315757	-15.4332	0.018368	0.080397
SCE1572_7845	-1.596857525	-11.8478	0.018425	0.080612
SCE1572_2235	-1.627251989	-13.3557	0.018452	0.080699
SCE1572_10666	-1.648986281	-13.6343	0.018571	0.081183
SCE1572_3598	1.679363538	-13.8605	0.018669	0.08158
SCE1572_2124	1.686348278	-13.9292	0.018704	0.081698
SCE1572_10062	-1.596292857	-11.9163	0.018723	0.081747
SCE1572_9337	-1.588057282	-11.4424	0.018757	0.081861
SCE1572_4301	1.69324344	-14.257	0.018892	0.082313
SCE1572_7036	-1.677062288	-14.2239	0.018892	0.082313
SCE1572_5361	-1.65844661	-14.2146	0.018892	0.082313
SCE1572_6704	-1.65844661	-14.2146	0.018892	0.082313
SCE1572_6004	-1.667352163	-14.2476	0.01891	0.082356
SCE1572_7539	1.709799306	-14.3449	0.018947	0.082484
SCE1572_1402	1.657803439	-13.6743	0.019021	0.082773
SCE1572_8754	1.728576513	-14.5496	0.019074	0.082934
SCE1572_6183	1.728576513	-14.5496	0.019074	0.082934
SCE1572_2606	2.772970632	-17.6713	0.019112	0.083034
SCE1572_6539	2.772970632	-17.6713	0.019112	0.083034
SCE1572_724	1.744173368	-14.6621	0.019148	0.083153
SCE1572_10460	-1.643392264	-13.8351	0.019171	0.083218
SCE1572_6816	1.635467109	-13.3821	0.01918	0.083224
SCE1572_7022	1.683197125	-13.9131	0.019209	0.083315
SCE1572_7214	-1.706360544	-14.7211	0.01923	0.083374
SCE1572_10328	1.793531955	-14.9131	0.019323	0.08374
SCE1572_2381	-1.594578885	-12.6554	0.019343	0.083793
SCE1572_10110	-1.737668482	-14.9042	0.019356	0.083815
SCE1572_2899	1.777820032	-14.9989	0.019391	0.083874
SCE1572_5363	1.777820032	-14.9989	0.019391	0.083874
SCE1572_4129	-1.597829687	-12.9378	0.019394	0.083874
SCE1572_7175	-1.592338809	-12.3782	0.019449	0.084062
SCE1572_156	-1.627185177	-13.633	0.019453	0.084062
SCE1572_9924	-1.567973795	-10.5668	0.019507	0.084261
SCE1572_10299	-1.662848429	-14.231	0.019552	0.08442
SCE1572_5111	-1.569339892	-10.6788	0.019588	0.084507
SCE1572_1903	1.632028025	-13.4344	0.019613	0.084507
SCE1572_11265	-1.590840363	-12.6883	0.019613	0.084507
SCE1572_7450	-2.358886328	-17.1498	0.019615	0.084507
SCE1572_1167	-2.188961327	-17.0648	0.019615	0.084507
SCE1572_1108	1.851958928	-15.3244	0.019628	0.084507
SCE1572_7302	1.840084828	-15.3304	0.019628	0.084507
SCE1572_5475	-1.78599666	-15.3069	0.019673	0.084666
SCE1572_11264	-1.811991868	-15.3788	0.01972	0.084832
SCE1572_2463	1.838755146	-15.5085	0.019768	0.084934

SCE1572_6039	-1.794985443	-15.4317	0.019768	0.084934
SCE1572_2309	-1.794985443	-15.4317	0.019768	0.084934
SCE1572_6351	1.735495927	-14.4896	0.019794	0.085013
SCE1572_6911	1.66055809	-13.9796	0.01983	0.08513
SCE1572_6688	1.637428625	-13.6244	0.019859	0.085161
SCE1572_5929	1.637428625	-13.6244	0.019859	0.085161
SCE1572_6519	1.898501514	-15.6491	0.019868	0.085161
SCE1572_9422	-1.612460405	-13.6161	0.019885	0.085161
SCE1572_9611	2.569878767	-17.1879	0.01991	0.085161
SCE1572_10592	2.569878767	-17.1879	0.01991	0.085161
SCE1572_4399	2.535931435	-17.2048	0.01991	0.085161
SCE1572_4982	2.535931435	-17.2048	0.01991	0.085161
SCE1572_1246	2.535931435	-17.2048	0.01991	0.085161
SCE1572_6058	-1.83846408	-15.6551	0.019919	0.085164
SCE1572_8287	-1.637551386	-14.0186	0.019927	0.085164
SCE1572_3963	1.713469621	-14.6161	0.019965	0.08528
SCE1572_8576	1.933872371	-15.8308	0.01997	0.08528
SCE1572_5114	-1.573492606	-11.9167	0.019978	0.08528
SCE1572_9808	-1.562292101	-10.3744	0.020019	0.085359
SCE1572_6929	1.950968934	-15.9124	0.020021	0.085359
SCE1572_219	-1.856385988	-15.816	0.020021	0.085359
SCE1572_51	1.680032661	-14.1303	0.020033	0.085377
SCE1572_6469	2.050504608	-16.0325	0.02007	0.085431
SCE1572_3398	2.050504608	-16.0325	0.02007	0.085431
SCE1572_4682	2.050504608	-16.0325	0.02007	0.085431
SCE1572_4145	-1.617361914	-13.4652	0.020085	0.085461
SCE1572_8681	1.957395203	-16.0791	0.020116	0.085489
SCE1572_4406	1.957395203	-16.0791	0.020116	0.085489
SCE1572_5609	1.957395203	-16.0791	0.020116	0.085489
SCE1572_4310	1.688578445	-14.2135	0.020151	0.085496
SCE1572_6939	2.050504608	-16.2252	0.020157	0.085496
SCE1572_5950	2.050504608	-16.2252	0.020157	0.085496
SCE1572_7149	-2.094545725	-16.5815	0.020166	0.085496
SCE1572_1656	-2.094545725	-16.5815	0.020166	0.085496
SCE1572_1772	1.754111605	-14.7884	0.020167	0.085496
SCE1572_6220	2.239538432	-16.6161	0.020203	0.085526
SCE1572_1815	2.239538432	-16.6161	0.020203	0.085526
SCE1572_6518	2.21400334	-16.6288	0.020203	0.085526
SCE1572_5254	2.07435135	-16.4356	0.020206	0.085526
SCE1572_9307	-1.607026519	-13.5484	0.020224	0.085566
SCE1572_8263	-1.611847926	-13.5691	0.020254	0.085657
SCE1572_5801	-1.622497928	-13.9032	0.020327	0.085911
SCE1572_2306	-1.613529464	-13.3727	0.02033	0.085911
SCE1572_4070	-1.580444098	-12.5906	0.020401	0.086177
SCE1572_570	-1.590031224	-13.0912	0.02053	0.086684
SCE1572_2013	1.800985008	-15.0698	0.02055	0.086735
SCE1572_629	-1.595261172	-13.2932	0.020577	0.0868
SCE1572_2758	-1.588812422	-12.9574	0.020582	0.0868

SCE1572_1411	1.721623752	-14.4965	0.020593	0.086814
SCE1572_7952	-1.560036103	-11.352	0.020612	0.086858
SCE1572_6016	-1.587199355	-12.981	0.020629	0.086895
SCE1572_2657	1.605001828	-13.1892	0.020687	0.087106
SCE1572_4514	-1.574434369	-12.5141	0.020703	0.087113
SCE1572_1582	1.794665703	-15.1604	0.020706	0.087113
SCE1572_4055	-1.633993566	-14.1199	0.020727	0.087167
SCE1572_1330	1.705856436	-14.6199	0.020848	0.087641
SCE1572_1247	1.787470202	-15.2572	0.020879	0.087667
SCE1572_7660	1.787470202	-15.2572	0.020879	0.087667
SCE1572_4859	1.775882228	-15.2629	0.020879	0.087667
SCE1572_1961	1.720714395	-14.6739	0.020919	0.087799
SCE1572_3832	-1.629877458	-14.2289	0.020948	0.087886
SCE1572_3412	-1.734395463	-15.2525	0.020973	0.087958
SCE1572_10628	-1.577437093	-12.6517	0.021002	0.088044
SCE1572_3757	1.637602853	-13.7471	0.021032	0.088134
SCE1572_6568	-1.756850314	-15.3215	0.021073	0.088272
SCE1572_5358	-1.586120013	-13.2142	0.02114	0.088504
SCE1572_5535	1.656683594	-14.0595	0.021162	0.088504
SCE1572_4477	1.822552661	-15.4461	0.021179	0.088504
SCE1572_8180	1.822552661	-15.4461	0.021179	0.088504
SCE1572_10306	-1.738549497	-15.3725	0.021179	0.088504
SCE1572_729	-1.738549497	-15.3725	0.021179	0.088504
SCE1572_636	-1.555454974	-11.7966	0.021284	0.088904
SCE1572_5550	1.871534467	-15.537	0.021292	0.088904
SCE1572_3533	1.687934528	-14.4589	0.02135	0.089111
SCE1572_9496	-1.627567297	-13.8716	0.02138	0.089156
SCE1572_2987	-1.585358791	-13.0367	0.021381	0.089156
SCE1572_2302	-1.562202883	-12.2766	0.021386	0.089156
SCE1572_10177	-1.671961417	-14.8494	0.021424	0.08928
SCE1572_6701	1.655725948	-14.1855	0.021471	0.08944
SCE1572_9501	-1.551531406	-11.4607	0.021505	0.089544
SCE1572_7426	-1.686460986	-14.901	0.021525	0.089549
SCE1572_8612	1.869057736	-15.6638	0.021539	0.089549
SCE1572_2142	1.869057736	-15.6638	0.021539	0.089549
SCE1572_9028	-1.618074011	-14.1803	0.02154	0.089549
SCE1572_7236	-1.608458474	-13.9551	0.021614	0.089821
SCE1572_6407	-1.702057841	-14.9546	0.021631	0.089856
SCE1572_660	-1.536228213	-8.95283	0.021647	0.089886
SCE1572_2916	1.930210374	-15.7707	0.021674	0.089941
SCE1572_8259	-1.561843781	-12.3834	0.021679	0.089941
SCE1572_1995	1.657023252	-14.2751	0.021686	0.089941
SCE1572_863	-1.612460405	-13.8087	0.021781	0.090014
SCE1572_7267	1.626277937	-13.8523	0.021842	0.090014
SCE1572_9349	-1.57766996	-13.3309	0.021842	0.090014
SCE1572_8597	1.607484696	-13.3714	0.021887	0.090014
SCE1572_4954	32.27181629	-33.8801	0.0219	0.090014
SCE1572_7650	32.27181629	-33.8801	0.0219	0.090014

SCE1572_5578	32.27181629	-33.8801	0.0219	0.090014
SCE1572_5577	32.27181629	-33.8801	0.0219	0.090014
SCE1572_7446	32.27181629	-33.8801	0.0219	0.090014
SCE1572_1789	32.27181629	-33.8801	0.0219	0.090014
SCE1572_8527	32.27181629	-33.8801	0.0219	0.090014
SCE1572_8839	32.27181629	-33.8801	0.0219	0.090014
SCE1572_9080	32.27181629	-33.8801	0.0219	0.090014
SCE1572_873	32.27181629	-33.8801	0.0219	0.090014
SCE1572_10243	32.27181629	-33.8801	0.0219	0.090014
SCE1572_6653	32.27181629	-33.8801	0.0219	0.090014
SCE1572_6286	32.27181629	-33.8801	0.0219	0.090014
SCE1572_2566	32.27181629	-33.8801	0.0219	0.090014
SCE1572_10660	32.27181629	-33.8801	0.0219	0.090014
SCE1572_10638	32.27181629	-33.8801	0.0219	0.090014
SCE1572_7040	32.27181629	-33.8801	0.0219	0.090014
SCE1572_7013	32.20412948	-33.914	0.0219	0.090014
SCE1572_8437	-3.856385988	-19.401	0.0219	0.090014
SCE1572_3186	-1.609839705	-14.0813	0.021954	0.090166
SCE1572_10624	-1.616251984	-13.8772	0.021968	0.090166
SCE1572_2010	1.916203516	-15.9298	0.021972	0.090166
SCE1572_3663	1.783858948	-15.1658	0.021989	0.090166
SCE1572_2287	-1.721226404	-15.111	0.021989	0.090166
SCE1572_10956	-1.721226404	-15.111	0.021989	0.090166
SCE1572_686	1.703773122	-14.7465	0.022078	0.090463
SCE1572_7035	1.695409649	-14.7506	0.022078	0.090463
SCE1572_1061	-1.593351582	-13.7252	0.022095	0.090498
SCE1572_6108	1.681048143	-14.4624	0.022199	0.090537
SCE1572_7938	-1.643392264	-14.42	0.022199	0.090537
SCE1572_6481	3.252138469	-18.4317	0.022208	0.090537
SCE1572_7099	3.252138469	-18.4317	0.022208	0.090537
SCE1572_714	3.252138469	-18.4317	0.022208	0.090537
SCE1572_10129	3.252138469	-18.4317	0.022208	0.090537
SCE1572_11171	3.252138469	-18.4317	0.022208	0.090537
SCE1572_888	3.252138469	-18.4317	0.022208	0.090537
SCE1572_8807	3.188008132	-18.4638	0.022208	0.090537
SCE1572_8284	3.188008132	-18.4638	0.022208	0.090537
SCE1572_7329	3.188008132	-18.4638	0.022208	0.090537
SCE1572_2470	3.188008132	-18.4638	0.022208	0.090537
SCE1572_2259	1.700562137	-14.5071	0.022298	0.090807
SCE1572_2157	-1.631599051	-14.4473	0.022298	0.090807
SCE1572_1188	1.918401072	-16.0986	0.022309	0.090807
SCE1572_2786	-1.811991868	-15.9638	0.022309	0.090807
SCE1572_8308	-1.536902252	-11.1345	0.02232	0.090818
SCE1572_5225	-1.584581372	-13.4925	0.022343	0.090877
SCE1572_273	-1.543122754	-11.8607	0.022352	0.090878
SCE1572_6115	1.816039354	-15.3424	0.022418	0.091111
SCE1572_57	1.719531373	-14.8761	0.022434	0.091141
SCE1572_3038	1.650897149	-14.2781	0.022451	0.091175

SCE1572_8217	1.635467109	-14.0701	0.022481	0.091236
SCE1572_10878	2.029746048	-16.2355	0.022492	0.091236
SCE1572_8929	-1.856385988	-16.0791	0.022492	0.091236
SCE1572_8566	-1.651695568	-14.5261	0.022509	0.09127
SCE1572_468	-1.679081456	-14.8749	0.022566	0.091465
SCE1572_632	-1.549826294	-12.5943	0.022598	0.091559
SCE1572_7958	-1.557727672	-12.6078	0.022631	0.091658
SCE1572_11464	1.558272603	-12.3838	0.022728	0.092013
SCE1572_8228	1.79632112	-15.4592	0.022756	0.092087
SCE1572_3898	-1.62430451	-14.142	0.022835	0.092087
SCE1572_10203	-1.662633614	-14.9572	0.022854	0.092087
SCE1572_8118	-1.662633614	-14.9572	0.022854	0.092087
SCE1572_9843	-1.662633614	-14.9572	0.022854	0.092087
SCE1572_7690	-1.662633614	-14.9572	0.022854	0.092087
SCE1572_1235	1.696867653	-14.6858	0.022867	0.092087
SCE1572_9812	1.564082475	-12.8328	0.022875	0.092087
SCE1572_5461	2.050504608	-16.4476	0.022886	0.092087
SCE1572_7662	2.050504608	-16.4476	0.022886	0.092087
SCE1572_2798	2.050504608	-16.4476	0.022886	0.092087
SCE1572_1451	2.026257062	-16.4597	0.022886	0.092087
SCE1572_2031	2.026257062	-16.4597	0.022886	0.092087
SCE1572_4110	2.026257062	-16.4597	0.022886	0.092087
SCE1572_6783	-1.971863205	-16.3433	0.022886	0.092087
SCE1572_6006	-1.884400364	-16.2995	0.022886	0.092087
SCE1572_10985	-1.523810649	-10.0204	0.022904	0.092122
SCE1572_4054	-1.614311201	-14.1645	0.022932	0.092176
SCE1572_7321	1.84405373	-15.5508	0.022943	0.092176
SCE1572_1173	1.84405373	-15.5508	0.022943	0.092176
SCE1572_9991	1.770396689	-15.0851	0.023012	0.092416
SCE1572_2184	1.614405493	-13.727	0.023061	0.092579
SCE1572_4778	-1.743491931	-15.5033	0.023145	0.092879
SCE1572_9355	-1.615869019	-14.0455	0.023173	0.092958
SCE1572_2711	-1.561765239	-13.2537	0.023195	0.093012
SCE1572_9655	2.923564672	-18.0207	0.023239	0.093151
SCE1572_1153	-1.61207879	-14.071	0.023271	0.093245
SCE1572_108	-1.537793605	-12.0685	0.02331	0.093362
SCE1572_6397	-1.627567297	-14.2866	0.02336	0.093502
SCE1572_5419	-1.770994497	-15.5857	0.023362	0.093502
SCE1572_7249	1.582474021	-13.3358	0.023439	0.093528
SCE1572_5684	-1.61127349	-14.5236	0.023469	0.093528
SCE1572_5914	-1.61127349	-14.5236	0.023469	0.093528
SCE1572_1858	2.252138469	-16.9317	0.023483	0.093528
SCE1572_5849	2.252138469	-16.9317	0.023483	0.093528
SCE1572_2829	2.220429609	-16.9476	0.023483	0.093528
SCE1572_6118	2.220429609	-16.9476	0.023483	0.093528
SCE1572_11528	2.220429609	-16.9476	0.023483	0.093528
SCE1572_1161	2.220429609	-16.9476	0.023483	0.093528
SCE1572_1881	2.220429609	-16.9476	0.023483	0.093528

SCE1572_2274	2.220429609	-16.9476	0.023483	0.093528
SCE1572_4026	-1.539912323	-12.5149	0.023533	0.093528
SCE1572_7101	-1.523630207	-11.1348	0.023553	0.093528
SCE1572_4232	1.898501514	-15.7866	0.023597	0.093528
SCE1572_218	-1.749470784	-15.6471	0.023597	0.093528
SCE1572_826	1.701269167	-14.8852	0.023601	0.093528
SCE1572_1859	2.728576513	-17.6935	0.02363	0.093528
SCE1572_7350	2.728576513	-17.6935	0.02363	0.093528
SCE1572_5894	2.728576513	-17.6935	0.02363	0.093528
SCE1572_6647	2.728576513	-17.6935	0.02363	0.093528
SCE1572_6472	2.728576513	-17.6935	0.02363	0.093528
SCE1572_4039	2.682772823	-17.7164	0.02363	0.093528
SCE1572_4639	2.682772823	-17.7164	0.02363	0.093528
SCE1572_10314	2.682772823	-17.7164	0.02363	0.093528
SCE1572_1083	2.682772823	-17.7164	0.02363	0.093528
SCE1572_3796	2.501166017	-17.2222	0.023635	0.093528
SCE1572_1082	2.501166017	-17.2222	0.023635	0.093528
SCE1572_1685	2.501166017	-17.2222	0.023635	0.093528
SCE1572_6988	2.501166017	-17.2222	0.023635	0.093528
SCE1572_8311	2.501166017	-17.2222	0.023635	0.093528
SCE1572_3985	-1.596150215	-13.8446	0.023648	0.093544
SCE1572_3914	1.752423255	-15.2747	0.023752	0.093794
SCE1572_7677	1.752423255	-15.2747	0.023752	0.093794
SCE1572_7669	1.740549155	-15.2806	0.023752	0.093794
SCE1572_11061	-1.511552686	-8.95573	0.023752	0.093794
SCE1572_3920	1.659313851	-14.6432	0.023758	0.093794
SCE1572_9570	1.553657604	-12.5805	0.023769	0.093794
SCE1572_4679	1.728576513	-14.9456	0.023774	0.093794
SCE1572_10093	-1.541663663	-12.6942	0.023812	0.093909
SCE1572_604	-1.54705793	-12.8483	0.023913	0.094273
SCE1572_6913	1.79158631	-15.3546	0.02397	0.094463
SCE1572_8471	-1.515126415	-10.8167	0.023993	0.094516
SCE1572_10859	-1.521340337	-11.5078	0.024077	0.09481
SCE1572_30	-1.511142726	-10.3376	0.024115	0.094925
SCE1572_8290	1.558968971	-13.1285	0.024145	0.094938
SCE1572_6573	1.718522848	-15.0286	0.024152	0.094938
SCE1572_4416	1.718522848	-15.0286	0.024152	0.094938
SCE1572_3452	1.679188486	-14.5177	0.024154	0.094938
SCE1572_10052	-1.565870846	-13.8183	0.024182	0.095013
SCE1572_6275	-1.534959787	-12.6578	0.02421	0.095051
SCE1572_9520	-1.534959787	-12.6578	0.02421	0.095051
SCE1572_10954	1.749638128	-15.0955	0.02436	0.095604
SCE1572_8861	1.523821753	-11.7413	0.024389	0.09568
SCE1572_1661	-1.575039704	-13.7473	0.024399	0.09568
SCE1572_11323	-1.544441981	-13.1647	0.024409	0.09568
SCE1572_1312	-1.517647499	-11.7695	0.024416	0.09568
SCE1572_2930	1.783024297	-15.4658	0.024459	0.095777
SCE1572_1329	-1.707522602	-15.388	0.024459	0.095777

SCE1572_8252	-1.555566919	-13.3517	0.024487	0.095823
SCE1572_6001	-1.554816981	-13.5406	0.024494	0.095823
SCE1572_3506	-1.566430283	-13.6512	0.024498	0.095823
SCE1572_10190	-1.539008985	-12.8386	0.024517	0.095864
SCE1572_3355	1.58384142	-13.4845	0.024575	0.096055
SCE1572_8550	-1.613529464	-14.3727	0.024643	0.096283
SCE1572_10913	-1.695231196	-15.124	0.024818	0.096912
SCE1572_3984	-1.631319432	-14.6067	0.024822	0.096912
SCE1572_4836	-1.643392264	-14.8351	0.024833	0.096919
SCE1572_8769	1.544405439	-12.6857	0.024851	0.096953
SCE1572_8890	1.549082803	-12.9978	0.024961	0.097341
SCE1572_7309	1.646114353	-14.4798	0.024969	0.097341
SCE1572_10835	1.680747988	-14.6938	0.025012	0.097471
SCE1572_3325	-1.520030822	-12.1812	0.025037	0.097531
SCE1572_2303	-1.612460405	-14.2942	0.025084	0.097679
SCE1572_3567	2.008684432	-16.2461	0.025121	0.097681
SCE1572_8547	2.008684432	-16.2461	0.025121	0.097681
SCE1572_1514	1.987310781	-16.2568	0.025121	0.097681
SCE1572_4919	1.987310781	-16.2568	0.025121	0.097681
SCE1572_6717	-1.555991054	-13.4959	0.025279	0.098256
SCE1572_23	-1.65844661	-15.2146	0.025345	0.098471
SCE1572_5547	1.823733746	-15.6865	0.025353	0.098471
SCE1572_2190	-1.613815684	-14.7149	0.025423	0.098671
SCE1572_9537	-1.613815684	-14.7149	0.025423	0.098671
SCE1572_4464	1.623394276	-14.2461	0.025483	0.098822
SCE1572_4933	-1.856385988	-16.2311	0.025517	0.098822
SCE1572_10340	1.654575932	-14.5866	0.025521	0.098822
SCE1572_8659	-1.512431587	-12.0261	0.025523	0.098822
SCE1572_212	1.708398631	-15.0337	0.02553	0.098822
SCE1572_8601	-1.654752127	-14.9783	0.02553	0.098822
SCE1572_10053	-1.654752127	-14.9783	0.02553	0.098822
SCE1572_3232	-1.580176193	-14.0797	0.025537	0.098822
SCE1572_1694	1.569193889	-13.6585	0.025558	0.098866
SCE1572_5551	1.766711642	-15.3671	0.025639	0.099099
SCE1572_7335	-1.676679966	-15.2814	0.025639	0.099099
SCE1572_1853	1.693811095	-14.8186	0.025646	0.099099
SCE1572_5598	-1.556514814	-13.6561	0.025672	0.099161
SCE1572_2304	-1.530157755	-13.1718	0.025682	0.099166
SCE1572_10365	-1.556152964	-13.7698	0.025771	0.099471
SCE1572_9515	-1.548651774	-13.6722	0.025789	0.099484
SCE1572_4287	1.739145755	-15.1007	0.025793	0.099484
SCE1572_3114	1.607270217	-14.2999	0.025818	0.099543
SCE1572_662	-1.488573638	-8.82586	0.025855	0.099651
SCE1572_10275	-1.637072959	-14.8102	0.025883	0.099722
SCE1572_1280	1.635467109	-14.6551	0.025938	0.099781
SCE1572_784	2.001595007	-16.472	0.025952	0.099781
SCE1572_6195	2.001595007	-16.472	0.025952	0.099781
SCE1572_8803	-1.915279677	-16.3716	0.025952	0.099781

SCE1572_10141	-1.827816836	-16.3278	0.025952	0.099781
SCE1572_7984	-1.524066287	-12.8598	0.025956	0.099781
SCE1572_11529	1.685507791	-15.1276	0.026075	0.100204
SCE1572_1516	-1.504622663	-12.159	0.026185	0.100591
SCE1572_4112	1.728576513	-15.1935	0.026378	0.101158
SCE1572_9164	1.728576513	-15.1935	0.026378	0.101158
SCE1572_10321	-1.633993566	-15.1199	0.026378	0.101158
SCE1572_4670	1.70946769	-14.9551	0.026402	0.101158
SCE1572_9046	1.511658391	-12.2039	0.02642	0.101158
SCE1572_6958	2.16153592	-16.6551	0.026429	0.101158
SCE1572_2965	2.134568873	-16.6686	0.026429	0.101158
SCE1572_7137	2.134568873	-16.6686	0.026429	0.101158
SCE1572_9998	2.134568873	-16.6686	0.026429	0.101158
SCE1572_7092	-1.888094848	-16.4783	0.026429	0.101158
SCE1572_3444	1.535931435	-12.8975	0.026465	0.10126
SCE1572_9230	1.880579606	-15.9476	0.026515	0.101286
SCE1572_7838	1.86243226	-15.9566	0.026515	0.101286
SCE1572_6243	1.86243226	-15.9566	0.026515	0.101286
SCE1572_10714	1.86243226	-15.9566	0.026515	0.101286
SCE1572_10085	-1.505888741	-12.4888	0.02652	0.101286
SCE1572_1988	1.550578211	-13.4751	0.026575	0.101458
SCE1572_1218	1.646962747	-14.5904	0.026619	0.101589
SCE1572_8182	1.816039354	-15.5648	0.026673	0.101722
SCE1572_1337	-1.517734535	-13.0157	0.026673	0.101722
SCE1572_435	-1.530943409	-13.4657	0.026691	0.101754
SCE1572_6122	-1.509995835	-12.7413	0.026824	0.102224
SCE1572_3845	1.676109093	-14.8274	0.026923	0.102564
SCE1572_6765	1.687934528	-15.0439	0.026992	0.102787
SCE1572_2616	-1.550805887	-13.6834	0.027045	0.102955
SCE1572_7827	-1.553194455	-13.8802	0.027187	0.103455
SCE1572_4011	-1.501071724	-12.5843	0.027229	0.103578
SCE1572_10032	-1.578852012	-14.2178	0.027271	0.103695
SCE1572_3328	-1.520244034	-13.1597	0.027281	0.103695
SCE1572_10540	1.516898243	-12.5311	0.027289	0.103695
SCE1572_10347	-1.531067304	-13.4139	0.027307	0.103724
SCE1572_5613	1.647958053	-14.7102	0.027374	0.103926
SCE1572_1777	1.605305138	-14.3971	0.02738	0.103926
SCE1572_443	1.878323632	-16.1186	0.027504	0.104032
SCE1572_1489	1.878323632	-16.1186	0.027504	0.104032
SCE1572_7683	1.85785953	-16.1288	0.027504	0.104032
SCE1572_2573	1.85785953	-16.1288	0.027504	0.104032
SCE1572_9633	1.664036261	-14.9038	0.02751	0.104032
SCE1572_10307	2.188008132	-16.9638	0.027518	0.104032
SCE1572_8299	2.188008132	-16.9638	0.027518	0.104032
SCE1572_10716	2.188008132	-16.9638	0.027518	0.104032
SCE1572_6416	2.188008132	-16.9638	0.027518	0.104032
SCE1572_1863	1.79270685	-15.702	0.027526	0.104032
SCE1572_9544	1.79270685	-15.702	0.027526	0.104032

SCE1572_161	1.79270685	-15.702	0.027526	0.104032
SCE1572_1674	-1.504084244	-12.6703	0.027565	0.104142
SCE1572_6182	1.674536673	-15.133	0.027667	0.104489
SCE1572_9681	-1.485789768	-11.8998	0.027679	0.104495
SCE1572_11110	-1.53642208	-13.6852	0.027698	0.104504
SCE1572_1768	1.594559165	-14.3063	0.027701	0.104504
SCE1572_2565	-1.489603657	-12.1623	0.027742	0.104621
SCE1572_9601	-1.481861868	-11.4745	0.027756	0.104635
SCE1572_944	1.638673399	-14.6021	0.027768	0.104643
SCE1572_11189	1.500160304	-12.1784	0.027797	0.104681
SCE1572_2602	1.673571714	-14.9595	0.027832	0.104681
SCE1572_4177	1.673571714	-14.9595	0.027832	0.104681
SCE1572_11488	1.673571714	-14.9595	0.027832	0.104681
SCE1572_3596	1.673571714	-14.9595	0.027832	0.104681
SCE1572_5534	1.535931435	-13.4318	0.027855	0.104681
SCE1572_4022	-1.568405225	-14.4157	0.027857	0.104681
SCE1572_11468	-1.568405225	-14.4157	0.027857	0.104681
SCE1572_3241	1.635467109	-14.7806	0.027952	0.104978
SCE1572_6061	-1.473346379	-10.509	0.027956	0.104978
SCE1572_2009	1.866080037	-15.8028	0.028016	0.10509
SCE1572_1761	1.866080037	-15.8028	0.028016	0.10509
SCE1572_9626	1.796780089	-15.7837	0.028016	0.10509
SCE1572_9149	-1.511737816	-13.2514	0.02806	0.105156
SCE1572_5614	1.96561571	-16.2676	0.028084	0.105156
SCE1572_5660	1.964741085	-16.2681	0.028084	0.105156
SCE1572_3535	1.943589404	-16.2786	0.028084	0.105156
SCE1572_6952	1.943589404	-16.2786	0.028084	0.105156
SCE1572_2070	2.465542107	-17.24	0.028123	0.105192
SCE1572_9199	2.429016231	-17.2583	0.028123	0.105192
SCE1572_2545	2.429016231	-17.2583	0.028123	0.105192
SCE1572_3	-1.481346556	-11.8916	0.028179	0.105363
SCE1572_8190	1.553004948	-13.6963	0.028204	0.105421
SCE1572_3426	1.598941233	-14.2583	0.028216	0.105425
SCE1572_3196	-1.503916498	-13.0615	0.028238	0.105473
SCE1572_5104	-1.542725509	-14.1149	0.028253	0.105489
SCE1572_825	1.658187185	-14.8364	0.028268	0.10551
SCE1572_398	1.728576513	-15.493	0.02829	0.105526
SCE1572_7941	-1.508752179	-13.3364	0.028293	0.105526
SCE1572_8302	-1.50628722	-13.0762	0.028347	0.105658
SCE1572_5716	-1.468576777	-10.2308	0.028348	0.105658
SCE1572_8001	1.499861162	-12.6281	0.028415	0.105868
SCE1572_10072	-1.473731662	-11.4682	0.028428	0.105881
SCE1572_331	-1.509220601	-13.14	0.028453	0.105935
SCE1572_8011	-1.548533078	-14.1455	0.028474	0.105975
SCE1572_7831	-1.529221245	-14.041	0.028492	0.106005
SCE1572_1198	1.549027093	-13.7287	0.028536	0.106132
SCE1572_9854	1.523192323	-13.2738	0.028566	0.106206
SCE1572_9064	1.614708548	-14.4431	0.028686	0.106561

SCE1572_6640	1.581735125	-14.3127	0.028697	0.106561
SCE1572_1907	-1.54061012	-14.2735	0.028697	0.106561
SCE1572_10182	-1.508462684	-13.4457	0.028702	0.106561
SCE1572_4856	1.787470202	-15.5791	0.028778	0.106804
SCE1572_4134	1.554977191	-13.854	0.028899	0.107219
SCE1572_5217	1.587978183	-14.1345	0.028933	0.107289
SCE1572_8045	1.603045631	-14.3494	0.028955	0.107289
SCE1572_10108	-1.572289966	-14.4345	0.028963	0.107289
SCE1572_9987	1.616101784	-14.6059	0.028969	0.107289
SCE1572_7651	1.616101784	-14.6059	0.028969	0.107289
SCE1572_2689	-1.530341784	-13.6937	0.029055	0.107566
SCE1572_7581	-1.456792893	-9.53943	0.029207	0.108091
SCE1572_6544	-1.468569957	-11.4656	0.029265	0.108138
SCE1572_3387	2.635467109	-17.74	0.029323	0.108138
SCE1572_3468	2.635467109	-17.74	0.029323	0.108138
SCE1572_8200	2.635467109	-17.74	0.029323	0.108138
SCE1572_8361	2.635467109	-17.74	0.029323	0.108138
SCE1572_6580	2.635467109	-17.74	0.029323	0.108138
SCE1572_7414	2.635467109	-17.74	0.029323	0.108138
SCE1572_7395	2.635467109	-17.74	0.029323	0.108138
SCE1572_5118	-1.581763608	-14.8945	0.029345	0.108138
SCE1572_889	1.741400553	-15.3797	0.029361	0.108138
SCE1572_1991	1.741400553	-15.3797	0.029361	0.108138
SCE1572_1649	1.672303394	-15.1573	0.029363	0.108138
SCE1572_6437	1.663481485	-15.1386	0.029363	0.108138
SCE1572_4719	1.652340927	-15.1441	0.029363	0.108138
SCE1572_6810	-1.465510539	-11.3087	0.0294	0.108236
SCE1572_4533	1.976504026	-16.4846	0.029464	0.10828
SCE1572_4265	1.976504026	-16.4846	0.029464	0.10828
SCE1572_1430	1.950968934	-16.4973	0.029464	0.10828
SCE1572_3737	1.950968934	-16.4973	0.029464	0.10828
SCE1572_10841	1.950968934	-16.4973	0.029464	0.10828
SCE1572_4753	-1.529735483	-13.8919	0.029507	0.108402
SCE1572_3230	-1.566879371	-14.5014	0.029562	0.108512
SCE1572_10147	-1.545184299	-14.4905	0.029562	0.108512
SCE1572_5710	3.120893936	-18.4973	0.029588	0.108512
SCE1572_9093	3.120893936	-18.4973	0.029588	0.108512
SCE1572_2904	3.120893936	-18.4973	0.029588	0.108512
SCE1572_10955	-1.459450295	-11.14	0.029618	0.108584
SCE1572_10674	-1.518641187	-13.8284	0.029668	0.108728
SCE1572_5862	1.568796069	-14.3192	0.029732	0.108923
SCE1572_4770	-1.593351582	-14.9476	0.029754	0.108965
SCE1572_8739	1.682772823	-15.2164	0.029821	0.109059
SCE1572_1717	-1.641791937	-15.1508	0.029821	0.109059
SCE1572_10998	-1.641791937	-15.1508	0.029821	0.109059
SCE1572_10109	-1.641791937	-15.1508	0.029821	0.109059
SCE1572_10509	-1.625060442	-15.3467	0.029878	0.109125
SCE1572_5590	1.582725646	-14.566	0.029886	0.109125

SCE1572_1357	-1.486691474	-13.1429	0.029901	0.109125
SCE1572_2167	1.813319853	-15.7424	0.029901	0.109125
SCE1572_4703	1.760997991	-15.7178	0.029901	0.109125
SCE1572_8967	-1.698844711	-15.6218	0.029901	0.109125
SCE1572_4169	-1.490641661	-12.9765	0.02998	0.109377
SCE1572_7009	1.563517267	-14.0666	0.030027	0.10951
SCE1572_8971	-1.48501896	-13.0321	0.030152	0.109928
SCE1572_3688	1.55691646	-13.9939	0.030184	0.110004
SCE1572_5228	-1.516045856	-14.0476	0.030278	0.110308
SCE1572_6534	2.107088136	-16.6823	0.030291	0.11032
SCE1572_5800	-1.486929523	-13.0939	0.030339	0.110457
SCE1572_9178	-1.490989864	-13.3939	0.030437	0.110766
SCE1572_816	1.588398855	-14.5087	0.030445	0.110766
SCE1572_6684	-1.537498347	-14.2567	0.030502	0.110933
SCE1572_8221	1.83710097	-16.1392	0.030577	0.111089
SCE1572_5458	1.83710097	-16.1392	0.030577	0.111089
SCE1572_2146	1.81837124	-16.1486	0.030577	0.111089
SCE1572_4747	1.57795465	-14.4108	0.030637	0.11127
SCE1572_7252	1.696396107	-15.1221	0.030663	0.111328
SCE1572_470	-1.480140971	-12.8612	0.030803	0.111797
SCE1572_6976	1.66055809	-14.9796	0.030946	0.112238
SCE1572_7629	-1.587450981	-14.9208	0.030946	0.112238
SCE1572_8448	1.547749223	-14.2395	0.030958	0.112241
SCE1572_8363	1.595154845	-14.6752	0.030971	0.112251
SCE1572_7136	1.772970632	-15.5863	0.031061	0.112424
SCE1572_8586	1.772970632	-15.5863	0.031061	0.112424
SCE1572_1067	1.772970632	-15.5863	0.031061	0.112424
SCE1572_3627	1.758323856	-15.5937	0.031061	0.112424
SCE1572_728	-1.552836422	-14.5304	0.031157	0.112732
SCE1572_10442	1.641113672	-15.1498	0.031171	0.112744
SCE1572_421	-1.856385988	-16.5937	0.031233	0.112928
SCE1572_4206	-1.464068565	-12.5804	0.031274	0.113036
SCE1572_1119	1.921221591	-16.2898	0.031424	0.113349
SCE1572_7939	-1.756850314	-16.1288	0.031424	0.113349
SCE1572_5268	-1.756850314	-16.1288	0.031424	0.113349
SCE1572_494	1.715637457	-15.3926	0.031436	0.113349
SCE1572_11544	1.715637457	-15.3926	0.031436	0.113349
SCE1572_7182	1.715637457	-15.3926	0.031436	0.113349
SCE1572_6862	1.702581304	-15.3991	0.031436	0.113349
SCE1572_9344	-1.537703552	-14.3509	0.031461	0.113363
SCE1572_4200	-1.537703552	-14.3509	0.031461	0.113363
SCE1572_4538	1.51047145	-13.6878	0.031528	0.113565
SCE1572_903	1.671091018	-15.2222	0.03172	0.114219
SCE1572_1753	-1.686460986	-15.5791	0.031743	0.114261
SCE1572_6910	1.490843545	-13.29	0.031819	0.114495
SCE1572_3549	1.517072408	-13.8398	0.031851	0.114572
SCE1572_2572	1.547029144	-14.0748	0.031886	0.114659
SCE1572_1142	1.635467109	-15.0701	0.031943	0.11482

SCE1572_3441	-1.546045867	-14.602	0.031953	0.11482
SCE1572_10	-1.548957463	-14.5103	0.032077	0.11513
SCE1572_4481	1.806579025	-15.9846	0.032083	0.11513
SCE1572_4165	1.806579025	-15.9846	0.032083	0.11513
SCE1572_8816	1.806579025	-15.9846	0.032083	0.11513
SCE1572_5439	1.616101784	-14.9278	0.032121	0.115227
SCE1572_10230	1.595288866	-14.7981	0.032266	0.115508
SCE1572_1421	1.591072989	-14.8028	0.032266	0.115508
SCE1572_9836	1.591072989	-14.8028	0.032266	0.115508
SCE1572_5109	-1.534457893	-14.3332	0.032276	0.115508
SCE1572_8144	2.154841268	-16.9804	0.032309	0.115508
SCE1572_2526	2.154841268	-16.9804	0.032309	0.115508
SCE1572_5295	2.120893936	-16.9973	0.032309	0.115508
SCE1572_50	2.120893936	-16.9973	0.032309	0.115508
SCE1572_6245	2.120893936	-16.9973	0.032309	0.115508
SCE1572_7524	-1.9343885	-16.777	0.032309	0.115508
SCE1572_4814	-1.439431286	-11.6379	0.03235	0.115614
SCE1572_9304	-1.461141215	-13.0672	0.032384	0.115669
SCE1572_2322	-1.801938204	-16.2583	0.032387	0.115669
SCE1572_5153	1.744878325	-15.7259	0.032496	0.116021
SCE1572_10970	-1.441348488	-11.8806	0.032511	0.116034
SCE1572_5220	-1.511250502	-14.019	0.032551	0.116098
SCE1572_2423	-1.487152178	-13.6314	0.032559	0.116098
SCE1572_4598	-1.4479489	-12.306	0.032563	0.116098
SCE1572_672	-1.537938462	-14.4517	0.032573	0.116098
SCE1572_3000	1.503870226	-13.6054	0.032597	0.116104
SCE1572_6656	-1.479364752	-13.5783	0.032597	0.116104
SCE1572_10805	1.650574001	-14.9846	0.032641	0.116223
SCE1572_1476	1.686348278	-15.5142	0.032771	0.116607
SCE1572_7640	-1.610225401	-15.4366	0.032771	0.116607
SCE1572_3430	-1.542071077	-14.5809	0.032922	0.117095
SCE1572_1366	-1.459413204	-12.9996	0.032931	0.117095
SCE1572_2051	1.654575932	-15.3236	0.032962	0.117167
SCE1572_3540	1.512847822	-13.8095	0.032997	0.117251
SCE1572_2372	-1.465996014	-13.0493	0.03308	0.117504
SCE1572_4984	1.535931435	-14.2454	0.033122	0.117614
SCE1572_7550	1.518156733	-14.1694	0.033155	0.117694
SCE1572_8257	-1.489328517	-14.061	0.033185	0.117759
SCE1572_4121	-1.439660138	-12.0395	0.033246	0.117935
SCE1572_8917	-1.543503032	-14.8295	0.033277	0.118006
SCE1572_1322	1.816039354	-15.8278	0.033334	0.118011
SCE1572_3052	1.816039354	-15.8278	0.033334	0.118011
SCE1572_1508	1.816039354	-15.8278	0.033334	0.118011
SCE1572_8943	1.798965841	-15.8364	0.033334	0.118011
SCE1572_3014	1.798965841	-15.8364	0.033334	0.118011
SCE1572_3205	-1.457373912	-13.0383	0.033425	0.118291
SCE1572_7341	-1.441906915	-12.4433	0.033482	0.118322
SCE1572_8704	-1.856385988	-16.401	0.03349	0.118322

SCE1572_7485	-1.438353125	-12.0593	0.033505	0.118322
SCE1572_3564	1.523969718	-14.1195	0.033522	0.118322
SCE1572_8283	2.391541526	-17.277	0.03354	0.118322
SCE1572_4093	2.391541526	-17.277	0.03354	0.118322
SCE1572_7717	2.391541526	-17.277	0.03354	0.118322
SCE1572_7562	2.391541526	-17.277	0.03354	0.118322
SCE1572_142	2.358127422	-17.2938	0.03354	0.118322
SCE1572_2248	1.506334365	-13.9123	0.033551	0.118322
SCE1572_9653	1.496403071	-13.7328	0.033568	0.118322
SCE1572_9160	-1.488521609	-13.7146	0.033568	0.118322
SCE1572_11492	-1.459283502	-13.3612	0.033589	0.11833
SCE1572_9964	1.441493361	-12.0727	0.033593	0.11833
SCE1572_7473	-1.46304356	-13.1382	0.033779	0.118946
SCE1572_11345	-1.488234876	-13.7907	0.033848	0.119146
SCE1572_6423	1.525323335	-14.0097	0.033864	0.119163
SCE1572_4968	1.816039354	-16.1498	0.03402	0.119552
SCE1572_5695	1.816039354	-16.1498	0.03402	0.119552
SCE1572_1036	1.794665703	-16.1604	0.03402	0.119552
SCE1572_4992	-1.737087059	-16.0683	0.03402	0.119552
SCE1572_8066	1.437629361	-11.9553	0.034086	0.119746
SCE1572_5679	-1.427373765	-11.8407	0.034127	0.119851
SCE1572_9936	-1.47874846	-13.8085	0.034142	0.119862
SCE1572_4248	-1.487915307	-13.871	0.03416	0.119884
SCE1572_2106	-1.460765942	-13.2413	0.03419	0.11995
SCE1572_4089	-1.486691474	-14.1429	0.034241	0.120067
SCE1572_10864	-1.65844661	-15.7995	0.034271	0.120067
SCE1572_7437	1.630396119	-14.9947	0.034436	0.120067
SCE1572_5935	1.630396119	-14.9947	0.034436	0.120067
SCE1572_2775	-1.593351582	-15.0731	0.034452	0.120067
SCE1572_8309	-1.456206447	-13.2702	0.034613	0.120067
SCE1572_5142	1.529856921	-14.2054	0.034625	0.120067
SCE1572_2128	1.523756721	-14.2084	0.034625	0.120067
SCE1572_216	-1.504084244	-14.2552	0.034669	0.120067
SCE1572_11147	1.501984103	-13.8805	0.034764	0.120067
SCE1572_5494	2.07907376	-16.6963	0.034765	0.120067
SCE1572_8673	2.07907376	-16.6963	0.034765	0.120067
SCE1572_9898	2.050504608	-16.7106	0.034765	0.120067
SCE1572_11539	2.050504608	-16.7106	0.034765	0.120067
SCE1572_946	1.544105776	-14.4574	0.034767	0.120067
SCE1572_4862	32.14628541	-33.9429	0.034783	0.120067
SCE1572_2953	32.14628541	-33.9429	0.034783	0.120067
SCE1572_5169	32.14628541	-33.9429	0.034783	0.120067
SCE1572_10888	32.14628541	-33.9429	0.034783	0.120067
SCE1572_4401	32.14628541	-33.9429	0.034783	0.120067
SCE1572_1040	32.14628541	-33.9429	0.034783	0.120067
SCE1572_8447	32.14628541	-33.9429	0.034783	0.120067
SCE1572_512	32.14628541	-33.9429	0.034783	0.120067
SCE1572_2091	32.14628541	-33.9429	0.034783	0.120067

SCE1572_1601	32.14628541	-33.9429	0.034783	0.120067
SCE1572_9929	32.14628541	-33.9429	0.034783	0.120067
SCE1572_10041	32.14628541	-33.9429	0.034783	0.120067
SCE1572_8875	32.07779656	-33.9772	0.034783	0.120067
SCE1572_6486	32.00878188	-34.0117	0.034783	0.120067
SCE1572_11278	32.00878188	-34.0117	0.034783	0.120067
SCE1572_5700	32.00878188	-34.0117	0.034783	0.120067
SCE1572_6772	32.00878188	-34.0117	0.034783	0.120067
SCE1572_6982	32.00878188	-34.0117	0.034783	0.120067
SCE1572_7204	32.00878188	-34.0117	0.034783	0.120067
SCE1572_5173	32.00878188	-34.0117	0.034783	0.120067
SCE1572_9798	32.00878188	-34.0117	0.034783	0.120067
SCE1572_6409	32.00878188	-34.0117	0.034783	0.120067
SCE1572_9605	32.00878188	-34.0117	0.034783	0.120067
SCE1572_3558	32.00878188	-34.0117	0.034783	0.120067
SCE1572_8079	32.00878188	-34.0117	0.034783	0.120067
SCE1572_7945	32.00878188	-34.0117	0.034783	0.120067
SCE1572_2138	32.00878188	-34.0117	0.034783	0.120067
SCE1572_3005	32.00878188	-34.0117	0.034783	0.120067
SCE1572_184	32.00878188	-34.0117	0.034783	0.120067
SCE1572_4096	32.00878188	-34.0117	0.034783	0.120067
SCE1572_11250	32.00878188	-34.0117	0.034783	0.120067
SCE1572_228	32.00878188	-34.0117	0.034783	0.120067
SCE1572_1563	32.00878188	-34.0117	0.034783	0.120067
SCE1572_9510	32.00878188	-34.0117	0.034783	0.120067
SCE1572_10221	1.483464015	-13.6156	0.034848	0.120128
SCE1572_3716	1.489861786	-13.669	0.03489	0.120128
SCE1572_2296	-1.447674127	-13.1378	0.034909	0.120128
SCE1572_3685	2.231076853	-17.3573	0.034945	0.120128
SCE1572_4472	2.231076853	-17.3573	0.034945	0.120128
SCE1572_11184	2.231076853	-17.3573	0.034945	0.120128
SCE1572_2369	2.188008132	-17.3788	0.034945	0.120128
SCE1572_114	2.188008132	-17.3788	0.034945	0.120128
SCE1572_2415	2.188008132	-17.3788	0.034945	0.120128
SCE1572_10614	2.188008132	-17.3788	0.034945	0.120128
SCE1572_8507	2.188008132	-17.3788	0.034945	0.120128
SCE1572_356	2.188008132	-17.3788	0.034945	0.120128
SCE1572_9186	1.437113699	-12.3967	0.034949	0.120128
SCE1572_972	-1.440481745	-13.0776	0.03504	0.120366
SCE1572_2392	-1.441348488	-12.7505	0.035041	0.120366
SCE1572_5698	-1.566256472	-14.9857	0.035082	0.120467
SCE1572_3476	-1.45697914	-13.3433	0.035099	0.120486
SCE1572_4549	1.606897956	-15.1669	0.035155	0.120547
SCE1572_7527	1.606897956	-15.1669	0.035155	0.120547
SCE1572_8041	1.898501514	-16.3012	0.035193	0.120547
SCE1572_3966	-1.704382894	-16.1551	0.035193	0.120547
SCE1572_5004	-1.704382894	-16.1551	0.035193	0.120547
SCE1572_5457	1.64186488	-15.33	0.035227	0.120547

SCE1572_5455	1.64186488	-15.33	0.035227	0.120547
SCE1572_3490	-1.570983769	-15.2583	0.035227	0.120547
SCE1572_10266	-1.465596035	-13.7138	0.035235	0.120547
SCE1572_969	-1.520451035	-14.4605	0.035245	0.120547
SCE1572_2220	-1.499692475	-14.4501	0.035245	0.120547
SCE1572_4072	-1.431028303	-12.5638	0.035254	0.120547
SCE1572_6888	1.787470202	-15.9941	0.035325	0.120587
SCE1572_6015	1.787470202	-15.9941	0.035325	0.120587
SCE1572_5656	1.787470202	-15.9941	0.035325	0.120587
SCE1572_9629	1.535931435	-14.5894	0.035333	0.120587
SCE1572_595	1.728576513	-15.7341	0.035335	0.120587
SCE1572_479	1.71208839	-15.7423	0.035335	0.120587
SCE1572_9561	1.468261608	-13.5957	0.03535	0.120602
SCE1572_9358	-1.48501896	-14.0321	0.035592	0.121388
SCE1572_9707	1.603045631	-15.0863	0.035778	0.121921
SCE1572_7073	1.603045631	-15.0863	0.035778	0.121921
SCE1572_1931	1.48790992	-13.7289	0.035783	0.121921
SCE1572_3283	1.529476413	-14.293	0.03589	0.122243
SCE1572_8288	-1.551531406	-15.1611	0.035919	0.122303
SCE1572_2161	1.443649501	-12.9609	0.035932	0.122309
SCE1572_7916	-1.411904711	-11.8419	0.035948	0.122324
SCE1572_8193	1.535931435	-14.3829	0.036009	0.122461
SCE1572_7702	1.571336771	-14.7485	0.036012	0.122461
SCE1572_10067	-1.403563826	-10.6908	0.036034	0.122496
SCE1572_9651	1.662688577	-15.4191	0.036071	0.122504
SCE1572_237	1.662688577	-15.4191	0.036071	0.122504
SCE1572_1226	1.662688577	-15.4191	0.036071	0.122504
SCE1572_7147	-1.48441721	-14.1276	0.036096	0.122548
SCE1572_2803	-1.628975492	-15.5147	0.036232	0.12297
SCE1572_2479	-1.78599666	-16.6288	0.036253	0.123002
SCE1572_6387	-1.490736515	-14.5177	0.036287	0.123078
SCE1572_1480	1.620200352	-14.9997	0.036337	0.123209
SCE1572_1384	1.463286133	-13.3261	0.036368	0.123235
SCE1572_7166	1.781687849	-15.845	0.03639	0.123235
SCE1572_4348	1.781687849	-15.845	0.03639	0.123235
SCE1572_4207	-1.457836611	-13.7423	0.036431	0.123235
SCE1572_1740	2.586557508	-17.7645	0.036521	0.123235
SCE1572_3699	2.586557508	-17.7645	0.036521	0.123235
SCE1572_4568	2.586557508	-17.7645	0.036521	0.123235
SCE1572_7791	2.586557508	-17.7645	0.036521	0.123235
SCE1572_2193	2.535931435	-17.7898	0.036521	0.123235
SCE1572_7328	2.535931435	-17.7898	0.036521	0.123235
SCE1572_7701	2.535931435	-17.7898	0.036521	0.123235
SCE1572_9090	2.535931435	-17.7898	0.036521	0.123235
SCE1572_6890	2.535931435	-17.7898	0.036521	0.123235
SCE1572_10028	2.535931435	-17.7898	0.036521	0.123235
SCE1572_10278	2.535931435	-17.7898	0.036521	0.123235
SCE1572_4027	-1.419637491	-12.3667	0.036538	0.123254

SCE1572_119	-1.445452887	-13.3211	0.03662	0.123493
SCE1572_3185	1.483464015	-13.7311	0.036653	0.12356
SCE1572_6797	-1.47424037	-13.8642	0.036664	0.12356
SCE1572_1523	1.527982682	-14.5933	0.036854	0.124121
SCE1572_3956	1.519989891	-14.5973	0.036854	0.124121
SCE1572_535	-1.417712929	-12.6374	0.036881	0.124174
SCE1572_2628	1.535931435	-14.7048	0.037063	0.124744
SCE1572_4584	1.462594823	-13.7118	0.0372	0.125166
SCE1572_2096	-1.64993511	-15.5973	0.037275	0.125298
SCE1572_2179	-1.602629396	-15.5737	0.037275	0.125298
SCE1572_6474	-1.602629396	-15.5737	0.037275	0.125298
SCE1572_5640	1.43029126	-12.8997	0.037348	0.125505
SCE1572_5062	-1.447453851	-13.5721	0.037453	0.125786
SCE1572_1034	1.532648478	-14.6395	0.037456	0.125786
SCE1572_7195	1.566519755	-14.9526	0.037568	0.126122
SCE1572_981	1.478110781	-13.9968	0.0376	0.126189
SCE1572_3615	1.616101784	-15.3428	0.03766	0.126269
SCE1572_11310	1.603045631	-15.3494	0.03766	0.126269
SCE1572_9455	-1.578084825	-15.2922	0.03766	0.126269
SCE1572_4873	-1.407200266	-12.3777	0.037764	0.126577
SCE1572_4721	-1.42342658	-13.0552	0.037821	0.126728
SCE1572_11467	-1.420019979	-12.8913	0.03787	0.126758
SCE1572_5818	1.592074513	-15.0918	0.037878	0.126758
SCE1572_5546	1.592074513	-15.0918	0.037878	0.126758
SCE1572_1944	1.592074513	-15.0918	0.037878	0.126758
SCE1572_6812	2.086128518	-17.0147	0.038006	0.126935
SCE1572_9753	2.086128518	-17.0147	0.038006	0.126935
SCE1572_4900	1.574663829	-14.8781	0.038011	0.126935
SCE1572_905	1.657493415	-15.5286	0.038021	0.126935
SCE1572_4314	1.639942177	-15.5274	0.038021	0.126935
SCE1572_1614	-1.429571321	-13.2569	0.038021	0.126935
SCE1572_9672	-1.451995733	-13.889	0.038055	0.126935
SCE1572_7206	1.898501514	-16.5236	0.038112	0.126935
SCE1572_3658	1.898501514	-16.5236	0.038112	0.126935
SCE1572_5797	1.898501514	-16.5236	0.038112	0.126935
SCE1572_4104	1.871534467	-16.537	0.038112	0.126935
SCE1572_8753	1.871534467	-16.537	0.038112	0.126935
SCE1572_2585	1.871534467	-16.537	0.038112	0.126935
SCE1572_8558	1.871534467	-16.537	0.038112	0.126935
SCE1572_15	-1.707522602	-16.388	0.038112	0.126935
SCE1572_8628	-1.480370377	-14.3222	0.038193	0.127164
SCE1572_8013	-1.475781986	-14.0677	0.03824	0.127282
SCE1572_9632	-1.400043653	-12.3484	0.038284	0.127387
SCE1572_3858	-1.539356692	-14.9448	0.038351	0.127568
SCE1572_5060	-1.422629291	-13.118	0.038417	0.127662
SCE1572_1123	1.695409649	-15.7506	0.038439	0.127662
SCE1572_1200	1.695409649	-15.7506	0.038439	0.127662
SCE1572_8112	1.695409649	-15.7506	0.038439	0.127662

SCE1572_11377	-1.622895858	-15.6598	0.038439	0.127662
SCE1572_4642	1.507220138	-14.4462	0.038463	0.127701
SCE1572_10231	1.46734435	-14.0022	0.038694	0.128427
SCE1572_4192	-1.433174557	-13.73	0.03884	0.12887
SCE1572_11583	-1.405588725	-12.7438	0.038872	0.128935
SCE1572_2471	1.768104877	-16.0038	0.03892	0.129014
SCE1572_2491	1.768104877	-16.0038	0.03892	0.129014
SCE1572_1503	-1.429375847	-13.6956	0.039014	0.129285
SCE1572_4343	1.528551905	-14.4861	0.039065	0.129373
SCE1572_548	1.528551905	-14.4861	0.039065	0.129373
SCE1572_9994	1.482093283	-14.0313	0.039152	0.129508
SCE1572_3885	1.698202864	-15.6237	0.039155	0.129508
SCE1572_4926	1.682772823	-15.6314	0.039155	0.129508
SCE1572_7603	1.682772823	-15.6314	0.039155	0.129508
SCE1572_2166	1.441695365	-13.4062	0.03919	0.129559
SCE1572_4075	-1.519351	-14.9846	0.039195	0.129559
SCE1572_4211	-1.383961826	-11.49	0.039298	0.129857
SCE1572_11024	-1.43248592	-13.6243	0.03934	0.129955
SCE1572_2139	1.851958928	-16.3244	0.03945	0.130239
SCE1572_4389	1.851958928	-16.3244	0.03945	0.130239
SCE1572_3544	1.54486456	-14.7618	0.039505	0.130378
SCE1572_4575	1.559976909	-15.1903	0.039688	0.130543
SCE1572_3754	3.050504608	-18.5325	0.039696	0.130543
SCE1572_3308	3.050504608	-18.5325	0.039696	0.130543
SCE1572_8069	3.017673215	-18.5489	0.039696	0.130543
SCE1572_3494	2.976504026	-18.5695	0.039696	0.130543
SCE1572_7457	2.976504026	-18.5695	0.039696	0.130543
SCE1572_9163	2.976504026	-18.5695	0.039696	0.130543
SCE1572_9193	2.976504026	-18.5695	0.039696	0.130543
SCE1572_3147	2.976504026	-18.5695	0.039696	0.130543
SCE1572_10384	2.976504026	-18.5695	0.039696	0.130543
SCE1572_3050	2.976504026	-18.5695	0.039696	0.130543
SCE1572_4066	-1.42342658	-13.4476	0.039704	0.130543
SCE1572_6386	1.746498421	-15.8626	0.039747	0.130562
SCE1572_8135	1.746498421	-15.8626	0.039747	0.130562
SCE1572_2174	1.746498421	-15.8626	0.039747	0.130562
SCE1572_10076	-1.400156801	-12.8153	0.039832	0.130802
SCE1572_8862	2.021358262	-16.7252	0.039955	0.130913
SCE1572_6435	2.021358262	-16.7252	0.039955	0.130913
SCE1572_9086	1.991610919	-16.74	0.039955	0.130913
SCE1572_6244	1.991610919	-16.74	0.039955	0.130913
SCE1572_2651	1.555427611	-14.8877	0.039957	0.130913
SCE1572_453	1.555427611	-14.8877	0.039957	0.130913
SCE1572_4880	-1.388287245	-12.3131	0.039964	0.130913
SCE1572_9627	1.407563901	-12.769	0.040074	0.130913
SCE1572_7730	2.353067378	-17.2963	0.040094	0.130913
SCE1572_3443	2.353067378	-17.2963	0.040094	0.130913
SCE1572_8354	2.353067378	-17.2963	0.040094	0.130913

SCE1572_7322	2.353067378	-17.2963	0.040094	0.130913
SCE1572_5048	2.353067378	-17.2963	0.040094	0.130913
SCE1572_6561	2.353067378	-17.2963	0.040094	0.130913
SCE1572_8077	2.338173898	-17.3037	0.040094	0.130913
SCE1572_9235	2.313539014	-17.316	0.040094	0.130913
SCE1572_6207	2.192399901	-17.2479	0.040094	0.130913
SCE1572_6062	-1.39929831	-12.9501	0.040099	0.130913
SCE1572_2519	1.495742345	-14.6095	0.040111	0.130913
SCE1572_1459	1.404630342	-12.583	0.040122	0.130913
SCE1572_8486	1.469333686	-14.0752	0.040128	0.130913
SCE1572_80	-1.422431275	-13.4381	0.040169	0.131007
SCE1572_8076	1.639550596	-15.6822	0.04042	0.131743
SCE1572_8139	-1.612460405	-15.6161	0.04042	0.131743
SCE1572_6170	-1.718882464	-16.0103	0.040452	0.131808
SCE1572_8239	-1.453030294	-14.2451	0.040641	0.132366
SCE1572_10122	1.479125079	-14.1088	0.040649	0.132366
SCE1572_9734	-1.557727672	-15.1928	0.040716	0.132545
SCE1572_3407	-1.383785853	-12.3109	0.040935	0.133216
SCE1572_9602	1.628049637	-15.5433	0.040976	0.133262
SCE1572_8296	-1.584581372	-15.4925	0.040976	0.133262
SCE1572_4050	-1.386900704	-12.4658	0.040987	0.133262
SCE1572_10477	-1.615377888	-15.8211	0.041225	0.133995
SCE1572_476	-1.457290032	-14.2786	0.041253	0.134043
SCE1572_422	-1.466679778	-14.4873	0.041318	0.134214
SCE1572_9682	-1.389896957	-12.8398	0.041374	0.13435
SCE1572_5168	-1.500905333	-14.7208	0.041386	0.13435
SCE1572_7478	-1.493815908	-14.9973	0.041442	0.134415
SCE1572_9249	1.621661309	-15.4396	0.041444	0.134415
SCE1572_6468	1.621661309	-15.4396	0.041444	0.134415
SCE1572_6963	-1.418264875	-13.8127	0.041557	0.134739
SCE1572_1125	1.457274491	-14.1999	0.041605	0.134853
SCE1572_1233	1.54619977	-14.9627	0.041743	0.135259
SCE1572_5369	1.67853583	-15.7591	0.041837	0.135408
SCE1572_3626	1.67853583	-15.7591	0.041837	0.135408
SCE1572_9055	1.661462317	-15.7676	0.041837	0.135408
SCE1572_6125	-1.534457893	-15.24	0.041842	0.135408
SCE1572_8638	-1.45850904	-14.5776	0.041853	0.135408
SCE1572_4497	-1.461754699	-14.3129	0.041897	0.135508
SCE1572_3575	1.545712456	-14.8926	0.042011	0.135834
SCE1572_3449	1.548004267	-15.1963	0.042185	0.136159
SCE1572_1998	1.548004267	-15.1963	0.042185	0.136159
SCE1572_6129	-1.361334404	-11.0749	0.042189	0.136159
SCE1572_9483	-1.376605724	-12.7461	0.042214	0.136159
SCE1572_167	1.750944326	-16.1823	0.042215	0.136159
SCE1572_6938	1.750944326	-16.1823	0.042215	0.136159
SCE1572_9949	1.750944326	-16.1823	0.042215	0.136159
SCE1572_3625	1.728576513	-16.1935	0.042215	0.136159
SCE1572_5067	-1.373646996	-12.4825	0.042238	0.136191

SCE1572_4802	-1.482927592	-14.7548	0.042251	0.136192
SCE1572_2308	-1.413442492	-13.7645	0.042264	0.136193
SCE1572_8880	1.667175968	-15.6392	0.042332	0.13637
SCE1572_3431	-1.395513835	-13.2477	0.042387	0.136506
SCE1572_9457	1.558651512	-15.1085	0.042482	0.13677
SCE1572_10871	1.38151953	-12.5403	0.04256	0.136834
SCE1572_8496	1.418094945	-13.6763	0.042564	0.136834
SCE1572_10705	2.143614012	-17.401	0.042606	0.136834
SCE1572_1002	2.143614012	-17.401	0.042606	0.136834
SCE1572_223	2.143614012	-17.401	0.042606	0.136834
SCE1572_828	2.143614012	-17.401	0.042606	0.136834
SCE1572_10730	2.143614012	-17.401	0.042606	0.136834
SCE1572_4415	2.143614012	-17.401	0.042606	0.136834
SCE1572_688	-1.375046118	-12.5021	0.042643	0.136875
SCE1572_1507	1.436395761	-13.8477	0.042645	0.136875
SCE1572_11233	-1.404397352	-13.4471	0.042658	0.136877
SCE1572_363	1.557464597	-15.0311	0.042743	0.137107
SCE1572_8003	1.369605439	-11.8959	0.042895	0.137511
SCE1572_3084	1.728576513	-16.0236	0.042908	0.137511
SCE1572_3003	-1.640657297	-15.9239	0.042908	0.137511
SCE1572_4208	-1.445071575	-14.1804	0.042988	0.137641
SCE1572_7095	1.444783547	-14.2061	0.042988	0.137641
SCE1572_490	-1.432213179	-14.1796	0.042988	0.137641
SCE1572_8367	1.563152904	-15.3693	0.043079	0.137891
SCE1572_8502	1.453469275	-14.1611	0.043107	0.137939
SCE1572_6394	1.394459009	-13.0751	0.043202	0.138199
SCE1572_11141	1.461637653	-14.1175	0.043219	0.138212
SCE1572_6009	-1.43288691	-14.3648	0.043292	0.138403
SCE1572_78	1.517897512	-14.7753	0.043362	0.138476
SCE1572_834	-1.479531682	-14.7315	0.043362	0.138476
SCE1572_7548	1.586557508	-15.2645	0.043367	0.138476
SCE1572_10722	1.586557508	-15.2645	0.043367	0.138476
SCE1572_4334	1.84405373	-16.5508	0.043422	0.138487
SCE1572_8120	1.84405373	-16.5508	0.043422	0.138487
SCE1572_48	1.84405373	-16.5508	0.043422	0.138487
SCE1572_7700	1.710429166	-15.8806	0.043437	0.138487
SCE1572_11370	1.710429166	-15.8806	0.043437	0.138487
SCE1572_10047	-1.512431587	-15.1136	0.043614	0.13901
SCE1572_3505	1.471080291	-14.6218	0.043676	0.139167
SCE1572_10635	-1.378338691	-13.1322	0.043745	0.139344
SCE1572_2800	1.426547975	-13.8526	0.043763	0.139359
SCE1572_9071	-1.352343482	-11.3676	0.043873	0.139667
SCE1572_7918	1.491077199	-14.5049	0.043936	0.139781
SCE1572_8171	1.491077199	-14.5049	0.043936	0.139781
SCE1572_3803	1.515172875	-14.9783	0.044013	0.1399
SCE1572_1664	-1.481641194	-14.916	0.044013	0.1399
SCE1572_5785	-1.481641194	-14.916	0.044013	0.1399
SCE1572_2820	-1.376393047	-12.9131	0.044144	0.140227

SCE1572_4433	1.535931435	-14.8975	0.044177	0.140227
SCE1572_11166	-1.470732295	-14.8389	0.044177	0.140227
SCE1572_1047	1.597992403	-15.5583	0.044178	0.140227
SCE1572_4411	1.828112186	-16.3364	0.044263	0.140227
SCE1572_10245	1.828112186	-16.3364	0.044263	0.140227
SCE1572_5380	1.828112186	-16.3364	0.044263	0.140227
SCE1572_6857	1.828112186	-16.3364	0.044263	0.140227
SCE1572_10226	1.828112186	-16.3364	0.044263	0.140227
SCE1572_9166	1.828112186	-16.3364	0.044263	0.140227
SCE1572_1607	1.80386464	-16.3485	0.044263	0.140227
SCE1572_8048	1.607722118	-15.4466	0.044445	0.140678
SCE1572_1506	1.607722118	-15.4466	0.044445	0.140678
SCE1572_11019	-1.52140174	-15.359	0.044445	0.140678
SCE1572_3141	1.402348281	-13.3793	0.044537	0.140927
SCE1572_1855	1.494111259	-14.6669	0.044566	0.140942
SCE1572_7168	-1.363139656	-12.6308	0.044569	0.140942
SCE1572_11017	-1.434152987	-14.546	0.04467	0.141182
SCE1572_2390	-1.356431512	-12.2164	0.044671	0.141182
SCE1572_1007	-1.375374451	-13.1986	0.044752	0.141307
SCE1572_4855	2.013978732	-17.0508	0.044792	0.141307
SCE1572_4405	2.013978732	-17.0508	0.044792	0.141307
SCE1572_6820	2.013978732	-17.0508	0.044792	0.141307
SCE1572_8344	2.013978732	-17.0508	0.044792	0.141307
SCE1572_1132	2.013978732	-17.0508	0.044792	0.141307
SCE1572_917	-1.375580718	-13.0945	0.045243	0.142638
SCE1572_3879	-1.477874365	-14.8898	0.045253	0.142638
SCE1572_320	1.635467109	-15.9181	0.045254	0.142638
SCE1572_9993	1.436282516	-14.0542	0.04533	0.142833
SCE1572_10472	-1.375134837	-13.3107	0.045438	0.143132
SCE1572_3469	1.412392401	-13.797	0.045547	0.143336
SCE1572_8150	1.644184326	-15.7763	0.045557	0.143336
SCE1572_9373	1.644184326	-15.7763	0.045557	0.143336
SCE1572_136	1.644184326	-15.7763	0.045557	0.143336
SCE1572_4168	2.483464015	-17.816	0.045652	0.143387
SCE1572_5712	2.483464015	-17.816	0.045652	0.143387
SCE1572_6926	2.483464015	-17.816	0.045652	0.143387
SCE1572_380	2.483464015	-17.816	0.045652	0.143387
SCE1572_9846	2.483464015	-17.816	0.045652	0.143387
SCE1572_7188	-1.379137304	-13.6284	0.045655	0.143387
SCE1572_10865	-1.340787081	-11.2708	0.045686	0.143438
SCE1572_6101	1.483464015	-14.5087	0.0457	0.143438
SCE1572_8497	1.376453221	-12.9433	0.045713	0.143438
SCE1572_9156	-1.361621296	-12.8868	0.045738	0.143475
SCE1572_103	1.651408652	-15.6471	0.045785	0.143521
SCE1572_4155	-1.556825706	-15.5508	0.045785	0.143521
SCE1572_1626	-1.430901701	-14.347	0.045793	0.143521
SCE1572_8467	1.96123727	-16.7552	0.045981	0.143893
SCE1572_7976	1.96123727	-16.7552	0.045981	0.143893

SCE1572_3846	1.96123727	-16.7552	0.045981	0.143893
SCE1572_3057	1.96123727	-16.7552	0.045981	0.143893
SCE1572_11319	1.96123727	-16.7552	0.045981	0.143893
SCE1572_11085	-1.396954369	-14.0014	0.046014	0.143955
SCE1572_5465	1.549606372	-15.3761	0.046096	0.144098
SCE1572_6338	-1.477874365	-15.3049	0.046096	0.144098
SCE1572_9863	1.36706841	-12.7743	0.046101	0.144098
SCE1572_2132	1.561466527	-15.277	0.046203	0.144332
SCE1572_5443	1.561466527	-15.277	0.046203	0.144332
SCE1572_3933	-1.347965042	-12.3698	0.046221	0.144345
SCE1572_3913	-1.363053963	-13.132	0.046322	0.144617
SCE1572_914	-1.45697914	-14.9283	0.046415	0.144866
SCE1572_8039	1.477037746	-14.6754	0.04656	0.145273
SCE1572_2718	1.46539783	-14.4182	0.046632	0.145331
SCE1572_2636	1.458310538	-14.4217	0.046632	0.145331
SCE1572_1852	-1.434922219	-14.3829	0.046632	0.145331
SCE1572_10070	-1.333964953	-11.3584	0.046634	0.145331
SCE1572_5074	1.442013572	-14.2493	0.046662	0.145376
SCE1572_6634	1.403176226	-13.6557	0.046705	0.145467
SCE1572_4444	-1.356504988	-12.9095	0.047019	0.146312
SCE1572_536	-1.341211817	-12.2365	0.047078	0.146312
SCE1572_1428	1.705856436	-16.2048	0.047081	0.146312
SCE1572_5499	1.682772823	-16.2164	0.047081	0.146312
SCE1572_3313	1.682772823	-16.2164	0.047081	0.146312
SCE1572_5123	1.682772823	-16.2164	0.047081	0.146312
SCE1572_3461	1.682772823	-16.2164	0.047081	0.146312
SCE1572_10124	1.377955742	-13.179	0.047088	0.146312
SCE1572_10576	1.349162923	-12.2539	0.04729	0.146737
SCE1572_10651	-1.354988204	-12.8835	0.047334	0.146737
SCE1572_3086	1.708398631	-16.0337	0.047336	0.146737
SCE1572_8094	1.708398631	-16.0337	0.047336	0.146737
SCE1572_10587	1.708398631	-16.0337	0.047336	0.146737
SCE1572_5086	1.687934528	-16.0439	0.047336	0.146737
SCE1572_6821	1.687934528	-16.0439	0.047336	0.146737
SCE1572_261	1.687934528	-16.0439	0.047336	0.146737
SCE1572_1994	1.437986385	-14.1293	0.047405	0.146907
SCE1572_9819	1.692050637	-15.8898	0.047494	0.147096
SCE1572_4234	1.692050637	-15.8898	0.047494	0.147096
SCE1572_7383	1.44598931	-14.6343	0.047582	0.14724
SCE1572_4077	-1.526680542	-15.6117	0.04759	0.14724
SCE1572_9065	1.490416776	-14.789	0.047623	0.14724
SCE1572_1720	-1.505888741	-15.4888	0.047648	0.14724
SCE1572_351	-1.426146082	-14.8882	0.04766	0.14724
SCE1572_8450	1.579433074	-15.4607	0.047679	0.14724
SCE1572_4602	-1.52776324	-15.3954	0.047679	0.14724
SCE1572_8269	1.524435796	-15.1256	0.047688	0.14724
SCE1572_4358	1.524435796	-15.1256	0.047688	0.14724
SCE1572_3762	-1.336274631	-12.2181	0.047691	0.14724

SCE1572_3064	1.511478391	-15.2146	0.047694	0.14724
SCE1572_6898	-1.336323262	-12.3123	0.047763	0.14741
SCE1572_11214	1.387068049	-13.6918	0.047805	0.147496
SCE1572_4372	1.405708858	-13.77	0.047858	0.147572
SCE1572_4650	-1.376605724	-13.7461	0.047858	0.147572
SCE1572_8556	-1.379226777	-13.7816	0.047886	0.147616
SCE1572_1699	-1.352041947	-13.2102	0.047914	0.14766
SCE1572_1205	2.272897029	-17.3364	0.048037	0.147864
SCE1572_2150	2.272897029	-17.3364	0.048037	0.147864
SCE1572_9394	2.272897029	-17.3364	0.048037	0.147864
SCE1572_11477	2.261309055	-17.3422	0.048037	0.147864
SCE1572_4715	-1.392985467	-14.1249	0.048164	0.148212
SCE1572_7430	1.443737737	-14.429	0.048403	0.148905
SCE1572_6788	1.440451126	-14.4796	0.048459	0.149034
SCE1572_532	1.404686902	-13.8319	0.048513	0.149155
SCE1572_9115	-1.354946843	-13.4133	0.048575	0.149303
SCE1572_5910	-1.380357859	-13.9666	0.048714	0.149686
SCE1572_7599	-1.313349351	-10.0652	0.048746	0.14974
SCE1572_4312	1.506184092	-14.9124	0.048874	0.150045
SCE1572_4424	1.496130427	-14.9174	0.048874	0.150045
SCE1572_10348	1.494111259	-14.9888	0.048958	0.150225
SCE1572_6628	-1.379483233	-14.1476	0.048961	0.150225
SCE1572_11251	1.535931435	-15.2898	0.049237	0.151029
SCE1572_5908	-1.316964271	-11.2487	0.049304	0.151187
SCE1572_11547	2.639334926	-18.1628	0.049336	0.151187
SCE1572_4138	1.535931435	-15.3829	0.049338	0.151187
SCE1572_11460	-1.314956382	-10.8357	0.049357	0.151187
SCE1572_4558	-1.370959161	-14.029	0.049528	0.151187
SCE1572_9121	1.816039354	-16.5648	0.04953	0.151187
SCE1572_10265	1.787470202	-16.5791	0.04953	0.151187
SCE1572_3309	1.787470202	-16.5791	0.04953	0.151187
SCE1572_1774	1.787470202	-16.5791	0.04953	0.151187
SCE1572_10443	1.787470202	-16.5791	0.04953	0.151187
SCE1572_9261	1.787470202	-16.5791	0.04953	0.151187
SCE1572_6238	1.619347443	-15.6631	0.04954	0.151187
SCE1572_10162	1.619347443	-15.6631	0.04954	0.151187
SCE1572_5453	1.619347443	-15.6631	0.04954	0.151187
SCE1572_3342	-1.315303185	-11.2622	0.049553	0.151187
SCE1572_2858	-1.333545199	-12.6966	0.04958	0.151187
SCE1572_5784	-1.373993221	-14.0723	0.049613	0.151187
SCE1572_750	1.608994897	-15.7938	0.049632	0.151187
SCE1572_6339	-1.542725509	-15.6999	0.049632	0.151187
SCE1572_3148	1.596472977	-15.9376	0.049705	0.151187
SCE1572_10193	1.596472977	-15.9376	0.049705	0.151187
SCE1572_8980	1.596472977	-15.9376	0.049705	0.151187
SCE1572_4084	1.779202586	-16.3608	0.049706	0.151187
SCE1572_6378	1.779202586	-16.3608	0.049706	0.151187
SCE1572_2698	1.779202586	-16.3608	0.049706	0.151187

SCE1572_8453	-1.667352163	-16.2476	0.049706	0.151187
SCE1572_17	-1.667352163	-16.2476	0.049706	0.151187
SCE1572_10480	-1.667352163	-16.2476	0.049706	0.151187
SCE1572_10677	-1.593351582	-16.2106	0.049706	0.151187
SCE1572_4380	1.422473385	-14.2591	0.0499	0.151734
SCE1572_8727	1.468817239	-14.8639	0.050056	0.152165
SCE1572_9053	1.321077956	-11.739	0.050103	0.152264
SCE1572_9075	1.395333105	-14.0382	0.050215	0.152558
SCE1572_6992	1.514072001	-15.0528	0.050366	0.152937
SCE1572_10096	-1.326285422	-12.6548	0.050368	0.152937
SCE1572_1064	1.366978273	-13.6464	0.050539	0.153412
SCE1572_10568	-1.315239719	-11.845	0.050896	0.154451
SCE1572_9087	1.438234761	-14.7537	0.051015	0.154767
SCE1572_3165	1.418236392	-14.3945	0.051115	0.155024
SCE1572_10015	-1.34890224	-13.4748	0.051164	0.155128
SCE1572_10626	-1.378338691	-14.1322	0.051294	0.15548
SCE1572_8766	1.551698751	-15.5815	0.051409	0.155736
SCE1572_10779	1.551698751	-15.5815	0.051409	0.155736
SCE1572_9710	-1.325261639	-12.6277	0.051444	0.155799
SCE1572_7508	1.421289415	-14.5208	0.051459	0.155799
SCE1572_6088	-1.36718247	-13.9732	0.051558	0.156052
SCE1572_2147	1.472737609	-14.9995	0.051649	0.156178
SCE1572_5798	1.472737609	-14.9995	0.051649	0.156178
SCE1572_4003	-1.431888159	-14.9408	0.051649	0.156178
SCE1572_7710	-1.534457893	-15.6551	0.051673	0.156178
SCE1572_8286	-1.487152178	-15.6314	0.051673	0.156178
SCE1572_2722	-1.348827991	-13.6661	0.051817	0.156567
SCE1572_309	1.673434959	-15.8991	0.051956	0.156927
SCE1572_3944	-1.377033675	-14.3369	0.051966	0.156927
SCE1572_840	-1.325871271	-12.9658	0.052023	0.157055
SCE1572_5242	2.097810323	-17.4239	0.052094	0.157133
SCE1572_727	2.097810323	-17.4239	0.052094	0.157133
SCE1572_10949	2.050504608	-17.4476	0.052094	0.157133
SCE1572_8989	-1.30744271	-12.1997	0.052146	0.157211
SCE1572_3619	1.42159876	-14.4401	0.052165	0.157211
SCE1572_7754	-1.381047978	-14.3907	0.052165	0.157211
SCE1572_1826	1.667175968	-16.0543	0.052253	0.157387
SCE1572_6582	-1.593351582	-15.9476	0.052253	0.157387
SCE1572_7711	-1.384317543	-14.2108	0.052387	0.157745
SCE1572_9029	1.522992379	-15.2963	0.052484	0.157857
SCE1572_3750	1.522992379	-15.2963	0.052484	0.157857
SCE1572_6512	1.522992379	-15.2963	0.052484	0.157857
SCE1572_3850	-1.433694916	-15.22	0.052484	0.157857
SCE1572_8953	1.646485587	-16.2345	0.05255	0.158011
SCE1572_4013	-1.340136237	-13.7166	0.052638	0.158231
SCE1572_3034	-1.338537683	-13.4603	0.052683	0.158321
SCE1572_9107	-1.625060442	-16.3467	0.052818	0.158502
SCE1572_5942	1.508186445	-15.3968	0.052823	0.158502

SCE1572_2176	1.508186445	-15.3968	0.052823	0.158502
SCE1572_7611	-1.482927592	-15.3398	0.052823	0.158502
SCE1572_3857	-1.396954369	-14.5863	0.052859	0.158502
SCE1572_6514	1.976504026	-17.0695	0.052888	0.158502
SCE1572_7902	1.976504026	-17.0695	0.052888	0.158502
SCE1572_6608	-1.89945471	-16.92	0.052888	0.158502
SCE1572_10718	-1.773923828	-16.8573	0.052888	0.158502
SCE1572_6592	-1.428964764	-14.9143	0.052894	0.158502
SCE1572_1024	1.898501514	-16.7866	0.052986	0.15864
SCE1572_1474	1.898501514	-16.7866	0.052986	0.15864
SCE1572_10597	1.898501514	-16.7866	0.052986	0.15864
SCE1572_11100	-1.328648356	-13.2302	0.05311	0.158968
SCE1572_5320	-1.40266802	-14.6506	0.053131	0.158984
SCE1572_9579	1.378191972	-14.0468	0.05324	0.159266
SCE1572_9696	-1.315050419	-12.8388	0.053268	0.159304
SCE1572_6714	-1.293191302	-11.4732	0.05336	0.159534
SCE1572_6228	1.402664904	-14.269	0.053378	0.159542
SCE1572_8295	-1.363681995	-13.9194	0.053498	0.159856
SCE1572_5495	1.477514749	-15.1491	0.053577	0.160046
SCE1572_3097	1.586557508	-15.6795	0.053623	0.160077
SCE1572_10114	2.930210374	-18.5927	0.053633	0.160077
SCE1572_318	2.898501514	-18.6085	0.053633	0.160077
SCE1572_8886	1.38843766	-14.1936	0.053903	0.160834
SCE1572_183	-1.293227857	-11.4756	0.053918	0.160834
SCE1572_6716	-1.305485323	-12.4817	0.053932	0.160834
SCE1572_1006	-1.33245292	-13.3231	0.054046	0.161103
SCE1572_8835	1.591072989	-15.8028	0.054096	0.161103
SCE1572_319	1.475810443	-14.9276	0.054105	0.161103
SCE1572_10530	-1.399179034	-14.8747	0.054105	0.161103
SCE1572_4520	-1.349425999	-13.8996	0.054126	0.161103
SCE1572_315	1.320458428	-12.6971	0.054141	0.161103
SCE1572_6334	1.411841087	-14.6514	0.054151	0.161103
SCE1572_5839	1.411841087	-14.6514	0.054151	0.161103
SCE1572_8346	1.414142955	-14.4438	0.054161	0.161103
SCE1572_7540	-1.320058262	-13.218	0.054428	0.161853
SCE1572_4340	1.401976978	-14.4988	0.054463	0.161875
SCE1572_5969	1.461930854	-15.0049	0.054497	0.161875
SCE1572_3110	1.461930854	-15.0049	0.054497	0.161875
SCE1572_5790	-1.434922219	-14.9679	0.054497	0.161875
SCE1572_1483	1.376649058	-14.0119	0.054578	0.162069
SCE1572_8051	1.57657342	-15.9476	0.054625	0.162072
SCE1572_1162	1.57657342	-15.9476	0.054625	0.162072
SCE1572_7564	1.57657342	-15.9476	0.054625	0.162072
SCE1572_7828	-1.375760147	-14.3194	0.054713	0.162242
SCE1572_988	-1.357838239	-14.3104	0.054713	0.162242
SCE1572_3793	-1.311612577	-13.2058	0.05482	0.162513
SCE1572_9460	1.550578211	-15.4751	0.054921	0.162569
SCE1572_4552	1.535931435	-15.4825	0.054921	0.162569

SCE1572_5943	1.535931435	-15.4825	0.054921	0.162569
SCE1572_1124	1.535931435	-15.4825	0.054921	0.162569
SCE1572_4199	-1.493815908	-15.4124	0.054921	0.162569
SCE1572_4795	-1.44475509	-15.0522	0.054931	0.162569
SCE1572_526	-1.337311423	-13.9191	0.054981	0.162664
SCE1572_1731	1.321902487	-12.9706	0.054995	0.162664
SCE1572_6390	-1.344672469	-14.0719	0.055087	0.162893
SCE1572_53	1.344376418	-13.6307	0.055127	0.162963
SCE1572_2017	1.389306521	-14.2757	0.055212	0.163043
SCE1572_2939	-1.61934679	-16.0601	0.055216	0.163043
SCE1572_1807	-1.555216453	-16.028	0.055216	0.163043
SCE1572_3382	-1.555216453	-16.028	0.055216	0.163043
SCE1572_5383	1.439069896	-14.8788	0.055282	0.163192
SCE1572_198	-1.328756662	-13.4554	0.055315	0.163243
SCE1572_7749	1.519989891	-15.5973	0.055487	0.163676
SCE1572_4007	-1.308704503	-13.0974	0.055492	0.163676
SCE1572_8583	1.284768775	-11.2505	0.055521	0.163713
SCE1572_7311	1.322101027	-13.0044	0.055599	0.163898
SCE1572_402	1.754111605	-16.3734	0.055867	0.164467
SCE1572_4731	1.728576513	-16.3861	0.055867	0.164467
SCE1572_11237	1.728576513	-16.3861	0.055867	0.164467
SCE1572_8113	1.728576513	-16.3861	0.055867	0.164467
SCE1572_9293	-1.378338691	-14.3546	0.05587	0.164467
SCE1572_2225	1.496760838	-15.3094	0.055959	0.164544
SCE1572_10548	1.496760838	-15.3094	0.055959	0.164544
SCE1572_7216	1.496760838	-15.3094	0.055959	0.164544
SCE1572_10962	1.419738418	-14.7629	0.055959	0.164544
SCE1572_984	-1.330924499	-14.0939	0.05608	0.164855
SCE1572_7507	1.568886482	-15.7498	0.05613	0.164956
SCE1572_891	-1.316092536	-13.3583	0.05626	0.165099
SCE1572_3872	-1.316092536	-13.3583	0.05626	0.165099
SCE1572_3982	-1.348591348	-14.1471	0.056402	0.165099
SCE1572_8912	31.85677879	-34.0877	0.056522	0.165099
SCE1572_9088	31.85677879	-34.0877	0.056522	0.165099
SCE1572_1462	31.85677879	-34.0877	0.056522	0.165099
SCE1572_3029	31.85677879	-34.0877	0.056522	0.165099
SCE1572_8086	31.85677879	-34.0877	0.056522	0.165099
SCE1572_529	31.85677879	-34.0877	0.056522	0.165099
SCE1572_9829	31.85677879	-34.0877	0.056522	0.165099
SCE1572_5276	31.85677879	-34.0877	0.056522	0.165099
SCE1572_6404	31.85677879	-34.0877	0.056522	0.165099
SCE1572_10213	31.85677879	-34.0877	0.056522	0.165099
SCE1572_11352	31.85677879	-34.0877	0.056522	0.165099
SCE1572_8143	31.85677879	-34.0877	0.056522	0.165099
SCE1572_6150	31.85677879	-34.0877	0.056522	0.165099
SCE1572_2000	31.85677879	-34.0877	0.056522	0.165099
SCE1572_9296	-1.320751199	-13.8233	0.056546	0.165099
SCE1572_4322	1.394459009	-14.6601	0.056549	0.165099

SCE1572_11326	1.758323856	-16.5937	0.056561	0.165099
SCE1572_687	1.758323856	-16.5937	0.056561	0.165099
SCE1572_11084	-1.66374091	-16.4973	0.056561	0.165099
SCE1572_747	1.494111259	-15.4038	0.056571	0.165099
SCE1572_563	-1.412286023	-15.3377	0.056571	0.165099
SCE1572_7736	-1.412286023	-15.3377	0.056571	0.165099
SCE1572_6960	1.39391243	-14.3608	0.056699	0.165425
SCE1572_3918	-1.516535985	-15.816	0.056867	0.165732
SCE1572_4742	-1.516535985	-15.816	0.056867	0.165732
SCE1572_1527	-1.516535985	-15.816	0.056867	0.165732
SCE1572_11301	-1.464068565	-15.7898	0.056867	0.165732
SCE1572_6863	1.455200163	-14.9379	0.056941	0.165901
SCE1572_415	-1.362188951	-14.3813	0.057097	0.165905
SCE1572_4837	-1.303484696	-13.2017	0.057099	0.165905
SCE1572_2797	1.375823292	-14.2824	0.057114	0.165905
SCE1572_4656	-1.464068565	-15.4679	0.057268	0.165905
SCE1572_7020	2.449402179	-17.8331	0.057273	0.165905
SCE1572_3144	2.429016231	-17.8433	0.057273	0.165905
SCE1572_4920	2.429016231	-17.8433	0.057273	0.165905
SCE1572_3046	2.429016231	-17.8433	0.057273	0.165905
SCE1572_3667	2.429016231	-17.8433	0.057273	0.165905
SCE1572_5289	2.372432703	-17.8716	0.057273	0.165905
SCE1572_1766	2.372432703	-17.8716	0.057273	0.165905
SCE1572_9442	2.372432703	-17.8716	0.057273	0.165905
SCE1572_6744	2.372432703	-17.8716	0.057273	0.165905
SCE1572_7307	2.372432703	-17.8716	0.057273	0.165905
SCE1572_895	2.372432703	-17.8716	0.057273	0.165905
SCE1572_7554	2.372432703	-17.8716	0.057273	0.165905
SCE1572_8382	2.372432703	-17.8716	0.057273	0.165905
SCE1572_95	2.372432703	-17.8716	0.057273	0.165905
SCE1572_9603	2.372432703	-17.8716	0.057273	0.165905
SCE1572_10327	2.372432703	-17.8716	0.057273	0.165905
SCE1572_3594	2.372432703	-17.8716	0.057273	0.165905
SCE1572_2848	1.461292634	-15.2397	0.05744	0.16623
SCE1572_3018	1.461292634	-15.2397	0.05744	0.16623
SCE1572_9374	1.448468594	-15.2461	0.05744	0.16623
SCE1572_6877	-1.274483287	-11.8124	0.057449	0.16623
SCE1572_7250	1.317123351	-13.3331	0.057525	0.166307
SCE1572_3557	1.433833247	-14.8173	0.057537	0.166307
SCE1572_11526	1.424183077	-14.8221	0.057537	0.166307
SCE1572_428	-1.355081416	-14.5856	0.057539	0.166307
SCE1572_3440	1.332275576	-13.7192	0.057668	0.1665
SCE1572_3812	-1.856385988	-17.0791	0.057683	0.1665
SCE1572_3455	1.646114353	-16.0648	0.057716	0.1665
SCE1572_6556	1.646114353	-16.0648	0.057716	0.1665
SCE1572_9968	1.624740702	-16.0755	0.057716	0.1665
SCE1572_1965	1.624740702	-16.0755	0.057716	0.1665
SCE1572_1648	1.624740702	-16.0755	0.057716	0.1665

SCE1572_2588	1.357165946	-14.1313	0.057833	0.166791
SCE1572_7929	-1.3610607	-14.3633	0.057938	0.167047
SCE1572_7459	-1.293562464	-13.0825	0.057985	0.167138
SCE1572_7596	-1.297302119	-12.8926	0.058018	0.167188
SCE1572_2777	1.406648418	-14.7106	0.058053	0.167234
SCE1572_5034	1.569878767	-15.6879	0.058066	0.167234
SCE1572_3727	1.423163505	-15.0983	0.058151	0.167433
SCE1572_4048	-1.316161802	-13.6824	0.058201	0.167532
SCE1572_2485	-1.364532891	-14.4181	0.0584	0.168037
SCE1572_11329	-1.307268686	-13.3627	0.058409	0.168037
SCE1572_10892	-1.283346654	-12.7746	0.058526	0.168273
SCE1572_4482	-1.275359902	-12.1758	0.058669	0.168273
SCE1572_5506	1.635467109	-16.24	0.058699	0.168273
SCE1572_11182	1.635467109	-16.24	0.058699	0.168273
SCE1572_4969	1.635467109	-16.24	0.058699	0.168273
SCE1572_507	1.635467109	-16.24	0.058699	0.168273
SCE1572_7993	1.635467109	-16.24	0.058699	0.168273
SCE1572_6576	1.635467109	-16.24	0.058699	0.168273
SCE1572_2447	1.635467109	-16.24	0.058699	0.168273
SCE1572_8741	1.635467109	-16.24	0.058699	0.168273
SCE1572_10030	1.635467109	-16.24	0.058699	0.168273
SCE1572_6464	1.611219562	-16.2522	0.058699	0.168273
SCE1572_11494	1.611219562	-16.2522	0.058699	0.168273
SCE1572_1678	-1.403873783	-14.9268	0.058743	0.168353
SCE1572_1120	1.386295356	-14.5066	0.0589	0.168759
SCE1572_4136	1.521134433	-15.4899	0.058972	0.168781
SCE1572_355	1.521134433	-15.4899	0.058972	0.168781
SCE1572_5379	1.572925642	-15.8119	0.058988	0.168781
SCE1572_7664	1.572925642	-15.8119	0.058988	0.168781
SCE1572_1577	1.554547113	-15.8211	0.058988	0.168781
SCE1572_387	-1.357580131	-14.628	0.05906	0.168941
SCE1572_5215	-1.28963966	-12.9372	0.059201	0.169299
SCE1572_4634	1.353067378	-14.2113	0.059378	0.169712
SCE1572_1260	1.353067378	-14.2113	0.059378	0.169712
SCE1572_9189	1.364165087	-14.5683	0.059446	0.169858
SCE1572_849	-1.253721485	-9.88536	0.059482	0.169915
SCE1572_10789	-1.340136237	-14.3016	0.059567	0.170108
SCE1572_9396	-1.268072278	-11.9436	0.059585	0.170108
SCE1572_4250	-1.331544479	-14.1088	0.059644	0.170108
SCE1572_7060	1.483464015	-15.316	0.059679	0.170108
SCE1572_9540	1.470043499	-15.3228	0.059679	0.170108
SCE1572_4509	-1.437433438	-15.2529	0.059679	0.170108
SCE1572_592	-1.40266802	-15.2355	0.059679	0.170108
SCE1572_2357	-1.40266802	-15.2355	0.059679	0.170108
SCE1572_4629	-1.271423487	-12.4599	0.05977	0.170322
SCE1572_10335	-1.289685693	-13.3897	0.059793	0.170341
SCE1572_2226	1.503870226	-15.6054	0.059909	0.170514
SCE1572_7691	-1.431888159	-15.5258	0.059909	0.170514

SCE1572_9096	-1.431888159	-15.5258	0.059909	0.170514
SCE1572_3948	-1.271423487	-12.5336	0.059924	0.170514
SCE1572_3735	1.434291173	-14.9483	0.059934	0.170514
SCE1572_9101	-1.334617314	-14.5958	0.060041	0.170771
SCE1572_4338	1.556395538	-15.9577	0.060065	0.170792
SCE1572_5007	-1.253654399	-10.6498	0.060136	0.170949
SCE1572_8546	1.453469275	-15.1611	0.060244	0.171117
SCE1572_4550	1.453469275	-15.1611	0.060244	0.171117
SCE1572_1015	1.441294561	-15.1672	0.060244	0.171117
SCE1572_10751	1.317984919	-13.6439	0.060264	0.171127
SCE1572_2624	1.318437813	-13.7838	0.060287	0.171148
SCE1572_9660	-1.251261272	-10.3626	0.060319	0.171191
SCE1572_2561	-1.262085622	-11.8371	0.060501	0.171661
SCE1572_2432	-1.328007015	-14.0213	0.060547	0.171719
SCE1572_10912	-1.29146124	-13.5236	0.060563	0.171719
SCE1572_6824	-1.27814935	-12.9487	0.060576	0.171719
SCE1572_9379	1.465542107	-15.4181	0.060603	0.171719
SCE1572_8324	1.465542107	-15.4181	0.060603	0.171719
SCE1572_7898	-1.346385545	-14.4272	0.060649	0.171779
SCE1572_8725	1.397603278	-14.7151	0.060694	0.171779
SCE1572_5376	1.417875673	-15.0269	0.060706	0.171779
SCE1572_8420	1.417875673	-15.0269	0.060706	0.171779
SCE1572_1962	1.417875673	-15.0269	0.060706	0.171779
SCE1572_11304	-1.30336638	-13.6114	0.060762	0.171893
SCE1572_6191	-1.493815908	-15.6754	0.060997	0.172456
SCE1572_11306	-1.289234763	-13.3537	0.061064	0.172456
SCE1572_3808	-1.308898192	-13.8419	0.061081	0.172456
SCE1572_2890	1.408696247	-14.894	0.061089	0.172456
SCE1572_5299	1.398427911	-14.8991	0.061089	0.172456
SCE1572_6051	-1.372706823	-14.8615	0.061089	0.172456
SCE1572_3164	1.36221286	-14.2892	0.061128	0.172456
SCE1572_8789	-1.32146417	-14.2591	0.061128	0.172456
SCE1572_8280	1.866080037	-16.8028	0.061138	0.172456
SCE1572_7207	-1.612460405	-16.6161	0.061138	0.172456
SCE1572_2345	-1.399747584	-15.2031	0.061142	0.172456
SCE1572_3693	1.370441405	-14.5146	0.061261	0.172701
SCE1572_10014	1.362448614	-14.5186	0.061261	0.172701
SCE1572_4559	-1.566879371	-16.0863	0.061334	0.172858
SCE1572_2928	1.411386337	-15.1042	0.061571	0.17348
SCE1572_1605	1.349518311	-14.3386	0.061701	0.1738
SCE1572_3785	-1.307047397	-13.7688	0.061784	0.173987
SCE1572_6212	1.319252665	-14.0058	0.061833	0.174023
SCE1572_1796	-1.312843414	-13.9863	0.061833	0.174023
SCE1572_8416	-1.281477152	-13.5286	0.061846	0.174023
SCE1572_8119	-1.295726647	-13.7112	0.061904	0.17414
SCE1572_377	1.360262879	-14.5703	0.061929	0.174162
SCE1572_5286	1.318993444	-13.9388	0.061972	0.174238
SCE1572_5206	-1.318217699	-14.1623	0.062051	0.174412

SCE1572_4189	-1.280088348	-13.3141	0.062292	0.175044
SCE1572_10189	-1.299712531	-14.0211	0.062336	0.175099
SCE1572_3022	1.938029879	-17.0888	0.062562	0.175099
SCE1572_8173	1.938029879	-17.0888	0.062562	0.175099
SCE1572_8822	1.938029879	-17.0888	0.062562	0.175099
SCE1572_10149	1.938029879	-17.0888	0.062562	0.175099
SCE1572_9504	1.938029879	-17.0888	0.062562	0.175099
SCE1572_769	1.898501514	-17.1085	0.062562	0.175099
SCE1572_6426	1.898501514	-17.1085	0.062562	0.175099
SCE1572_4500	1.898501514	-17.1085	0.062562	0.175099
SCE1572_1392	1.898501514	-17.1085	0.062562	0.175099
SCE1572_902	1.898501514	-17.1085	0.062562	0.175099
SCE1572_7566	-1.811991868	-16.9638	0.062562	0.175099
SCE1572_533	-1.811991868	-16.9638	0.062562	0.175099
SCE1572_839	-1.686460986	-16.901	0.062562	0.175099
SCE1572_8205	-1.686460986	-16.901	0.062562	0.175099
SCE1572_8121	1.352013161	-14.1723	0.062756	0.175581
SCE1572_1764	1.702581304	-16.3991	0.062845	0.175581
SCE1572_6148	1.702581304	-16.3991	0.062845	0.175581
SCE1572_7143	1.702581304	-16.3991	0.062845	0.175581
SCE1572_3318	-1.608458474	-16.277	0.062845	0.175581
SCE1572_4179	1.446433284	-15.0866	0.062851	0.175581
SCE1572_5618	1.553004948	-15.6963	0.062901	0.175581
SCE1572_6218	1.535931435	-15.7048	0.062901	0.175581
SCE1572_3833	1.535931435	-15.7048	0.062901	0.175581
SCE1572_10104	-1.441348488	-15.6085	0.062901	0.175581
SCE1572_4937	-1.271423487	-13.1543	0.062931	0.175619
SCE1572_8464	-1.324534824	-14.345	0.063127	0.176119
SCE1572_3122	1.348472804	-14.2961	0.063247	0.176406
SCE1572_6190	1.394839116	-14.8368	0.063295	0.176495
SCE1572_5324	-1.271423487	-13.3989	0.063334	0.176555
SCE1572_3222	-1.303132347	-14.1858	0.063353	0.176562
SCE1572_5601	1.32613905	-14.1468	0.063448	0.176781
SCE1572_7198	-1.254145496	-12.3098	0.063508	0.176902
SCE1572_3642	1.319537754	-14.0761	0.063534	0.176925
SCE1572_7652	1.313539014	-14.0087	0.063611	0.177094
SCE1572_130	1.456496968	-15.3295	0.063661	0.17714
SCE1572_3532	1.456496968	-15.3295	0.063661	0.17714
SCE1572_1899	1.603045631	-16.0863	0.063789	0.177397
SCE1572_8879	1.603045631	-16.0863	0.063789	0.177397
SCE1572_2627	1.344235811	-14.4315	0.063826	0.177397
SCE1572_1131	2.001595007	-17.472	0.063872	0.177397
SCE1572_4470	2.001595007	-17.472	0.063872	0.177397
SCE1572_7004	2.001595007	-17.472	0.063872	0.177397
SCE1572_11476	-1.971863205	-17.3433	0.063872	0.177397
SCE1572_346	1.335234085	-14.3458	0.063916	0.177473
SCE1572_5533	1.280872825	-13.3513	0.064199	0.178211
SCE1572_10089	-1.341812815	-14.7514	0.064349	0.178402

SCE1572_5459	1.535931435	-15.8304	0.064352	0.178402
SCE1572_1951	1.535931435	-15.8304	0.064352	0.178402
SCE1572_2977	1.534994316	-15.8308	0.064352	0.178402
SCE1572_6111	-1.457836611	-15.7423	0.064352	0.178402
SCE1572_10202	1.366773621	-14.6823	0.064443	0.178526
SCE1572_2503	1.35006489	-14.6823	0.064443	0.178526
SCE1572_10393	1.300118498	-13.6528	0.064448	0.178526
SCE1572_2713	-1.332824032	-14.5543	0.064521	0.178636
SCE1572_7729	-1.312065472	-14.5439	0.064521	0.178636
SCE1572_10648	-1.325871271	-14.3809	0.064641	0.178693
SCE1572_4467	1.728576513	-16.6085	0.064661	0.178693
SCE1572_1814	1.728576513	-16.6085	0.064661	0.178693
SCE1572_8504	1.698202864	-16.6237	0.064661	0.178693
SCE1572_8141	1.698202864	-16.6237	0.064661	0.178693
SCE1572_9939	1.698202864	-16.6237	0.064661	0.178693
SCE1572_6889	1.698202864	-16.6237	0.064661	0.178693
SCE1572_5752	-1.236305444	-11.7482	0.064689	0.178702
SCE1572_5941	1.487568413	-15.6136	0.064707	0.178702
SCE1572_7727	-1.264437057	-13.0035	0.064715	0.178702
SCE1572_4299	1.26140139	-12.6722	0.064808	0.178911
SCE1572_8855	-1.284844003	-13.9453	0.064841	0.178956
SCE1572_6813	1.282607154	-13.6351	0.064935	0.179086
SCE1572_3774	1.451042537	-15.4254	0.06494	0.179086
SCE1572_10554	1.436395761	-15.4327	0.06494	0.179086
SCE1572_6452	1.284536988	-13.328	0.064993	0.179157
SCE1572_9675	-1.263011247	-13.2714	0.064999	0.179157
SCE1572_9640	-1.226143738	-9.70002	0.065043	0.17923
SCE1572_808	1.422473385	-15.2591	0.065096	0.179236
SCE1572_1227	1.409297996	-15.2657	0.065096	0.179236
SCE1572_5518	1.409297996	-15.2657	0.065096	0.179236
SCE1572_332	-1.358886328	-15.0648	0.065204	0.179487
SCE1572_1104	-1.345424068	-14.8236	0.065333	0.179795
SCE1572_10564	1.286571966	-13.4372	0.065397	0.179854
SCE1572_9853	1.353067378	-14.4744	0.065429	0.179854
SCE1572_9766	-1.328007015	-14.4364	0.065429	0.179854
SCE1572_2634	1.292477398	-13.888	0.065437	0.179854
SCE1572_2763	1.297050891	-13.9498	0.06544	0.179854
SCE1572_643	-1.249728416	-12.6382	0.065559	0.180008
SCE1572_6657	-1.236234059	-12.0141	0.065576	0.180008
SCE1572_3811	1.586557508	-16.2645	0.065616	0.180008
SCE1572_7164	1.586557508	-16.2645	0.065616	0.180008
SCE1572_1202	1.586557508	-16.2645	0.065616	0.180008
SCE1572_7498	1.586557508	-16.2645	0.065616	0.180008
SCE1572_9833	1.586557508	-16.2645	0.065616	0.180008
SCE1572_4587	-1.237297703	-12.1218	0.065962	0.180909
SCE1572_1691	-1.262263488	-13.3943	0.066231	0.1816
SCE1572_10647	-1.451995733	-15.6963	0.066313	0.181766
SCE1572_67	1.348728442	-14.6285	0.066326	0.181766

SCE1572_4720	1.370122542	-14.7289	0.066364	0.181822
SCE1572_5539	-1.256316595	-13.1161	0.066404	0.181869
SCE1572_3390	-1.349425999	-14.8996	0.066433	0.181869
SCE1572_6584	-1.349425999	-14.8996	0.066433	0.181869
SCE1572_774	-1.263060014	-13.263	0.066529	0.182086
SCE1572_4469	1.278773596	-13.4411	0.066681	0.182454
SCE1572_4745	-1.288497	-14.2926	0.066976	0.183212
SCE1572_1884	-1.309897635	-14.4648	0.067138	0.183609
SCE1572_10077	-1.233580136	-12.4409	0.067273	0.18393
SCE1572_3343	-1.239290866	-12.883	0.067427	0.184302
SCE1572_3824	-1.287365031	-14.1937	0.06761	0.184755
SCE1572_7493	1.395333105	-15.0382	0.067668	0.184769
SCE1572_1441	1.395333105	-15.0382	0.067668	0.184769
SCE1572_2126	1.395333105	-15.0382	0.067668	0.184769
SCE1572_7856	1.320593771	-14.31	0.067719	0.184861
SCE1572_4392	1.416632507	-15.1795	0.067795	0.184898
SCE1572_3517	1.404141562	-15.1858	0.067795	0.184898
SCE1572_5515	1.263999553	-13.122	0.067799	0.184898
SCE1572_10078	-1.230653881	-12.3785	0.067827	0.184898
SCE1572_6116	1.328489355	-14.3935	0.067838	0.184898
SCE1572_10369	1.266113667	-13.3586	0.067838	0.184898
SCE1572_6215	1.442822031	-15.3364	0.067926	0.184994
SCE1572_6107	1.429016231	-15.3433	0.067926	0.184994
SCE1572_1279	1.429016231	-15.3433	0.067926	0.184994
SCE1572_1055	1.272119086	-13.6938	0.067975	0.18508
SCE1572_10634	-1.249055674	-13.0973	0.068132	0.185458
SCE1572_2189	-1.470732295	-15.8389	0.068226	0.185619
SCE1572_2692	-1.418264875	-15.8127	0.068226	0.185619
SCE1572_3613	-1.317227177	-14.7164	0.068435	0.18612
SCE1572_3899	1.306652628	-14.2752	0.068446	0.18612
SCE1572_2049	-1.352343482	-14.9526	0.068833	0.187029
SCE1572_3221	-1.324862746	-14.9388	0.068833	0.187029
SCE1572_5711	-1.215248018	-11.256	0.06885	0.187029
SCE1572_3947	-1.207238968	-8.89943	0.068851	0.187029
SCE1572_2910	1.396001174	-15.2723	0.069322	0.188261
SCE1572_7305	1.360844728	-14.7335	0.069406	0.188439
SCE1572_5204	-1.238446165	-13.2424	0.069623	0.188978
SCE1572_1887	-1.225437282	-12.7221	0.069659	0.189029
SCE1572_7575	-1.252807809	-13.4174	0.069736	0.189145
SCE1572_887	-1.224426008	-12.4316	0.069738	0.189145
SCE1572_9023	-1.204141585	-9.46386	0.069759	0.189153
SCE1572_5756	1.45440155	-15.6301	0.069913	0.189496
SCE1572_5319	1.391541526	-14.9697	0.069957	0.189496
SCE1572_9506	1.391541526	-14.9697	0.069957	0.189496
SCE1572_7157	1.391541526	-14.9697	0.069957	0.189496
SCE1572_9733	1.246111086	-13.2449	0.069981	0.18951
SCE1572_9056	1.27727547	-13.8646	0.070057	0.189572
SCE1572_10720	1.313539014	-14.5937	0.070058	0.189572

SCE1572_3740	1.313539014	-14.5937	0.070058	0.189572
SCE1572_6163	1.497963585	-15.8494	0.070235	0.189893
SCE1572_3025	1.497963585	-15.8494	0.070235	0.189893
SCE1572_1993	-1.413442492	-15.7645	0.070235	0.189893
SCE1572_4030	-1.216975703	-12.0589	0.070266	0.189893
SCE1572_760	1.203676416	-10.0234	0.070453	0.189893
SCE1572_4351	1.581019325	-16.0973	0.070543	0.189893
SCE1572_8370	1.581019325	-16.0973	0.070543	0.189893
SCE1572_5468	1.239829327	-12.9949	0.070553	0.189893
SCE1572_11586	-1.23207482	-12.9123	0.070612	0.189893
SCE1572_5416	1.832913173	-16.8194	0.070633	0.189893
SCE1572_1505	1.832913173	-16.8194	0.070633	0.189893
SCE1572_4408	1.832913173	-16.8194	0.070633	0.189893
SCE1572_1790	1.832913173	-16.8194	0.070633	0.189893
SCE1572_4111	1.798965841	-16.8364	0.070633	0.189893
SCE1572_3012	1.798965841	-16.8364	0.070633	0.189893
SCE1572_8748	1.798965841	-16.8364	0.070633	0.189893
SCE1572_8647	1.798965841	-16.8364	0.070633	0.189893
SCE1572_9180	1.798965841	-16.8364	0.070633	0.189893
SCE1572_3602	-1.334159242	-15.1703	0.070659	0.189893
SCE1572_1558	1.343286357	-14.8012	0.070674	0.189893
SCE1572_726	1.343286357	-14.8012	0.070674	0.189893
SCE1572_3552	1.343286357	-14.8012	0.070674	0.189893
SCE1572_10512	-1.243226595	-13.4315	0.070694	0.189893
SCE1572_5879	1.676109093	-16.4124	0.070754	0.189893
SCE1572_6064	1.676109093	-16.4124	0.070754	0.189893
SCE1572_340	1.676109093	-16.4124	0.070754	0.189893
SCE1572_6184	1.676109093	-16.4124	0.070754	0.189893
SCE1572_3680	1.662149151	-16.4193	0.070754	0.189893
SCE1572_1641	1.649142045	-16.4259	0.070754	0.189893
SCE1572_6145	1.649142045	-16.4259	0.070754	0.189893
SCE1572_6167	1.649142045	-16.4259	0.070754	0.189893
SCE1572_5979	-1.54705793	-16.3077	0.070754	0.189893
SCE1572_11500	-1.271423487	-14.0936	0.070928	0.190263
SCE1572_3266	-1.271423487	-14.0936	0.070928	0.190263
SCE1572_6358	1.345960491	-14.9254	0.071042	0.190472
SCE1572_989	-1.296958579	-14.8734	0.071042	0.190472
SCE1572_5549	1.278040332	-13.8338	0.071113	0.190614
SCE1572_9589	-1.2089594	-11.8421	0.071543	0.191717
SCE1572_4044	-1.390067984	-15.5049	0.071569	0.191738
SCE1572_7191	-1.218571605	-12.601	0.071626	0.191841
SCE1572_8191	1.25310316	-13.4089	0.071887	0.19249
SCE1572_605	-1.332824032	-15.1392	0.07194	0.192585
SCE1572_5091	-1.221189185	-12.6797	0.071978	0.192636
SCE1572_9547	1.322417165	-14.6417	0.07215	0.193031
SCE1572_6604	-1.257068194	-14.0425	0.072162	0.193031
SCE1572_11332	-1.373303101	-15.285	0.072494	0.19387
SCE1572_8774	-1.203557457	-11.8914	0.072592	0.194044

SCE1572_8473	1.342108166	-14.7429	0.072596	0.194044
SCE1572_6415	1.494111259	-15.9888	0.07274	0.194116
SCE1572_5634	1.494111259	-15.9888	0.07274	0.194116
SCE1572_210	1.472737609	-15.9995	0.07274	0.194116
SCE1572_6402	1.472737609	-15.9995	0.07274	0.194116
SCE1572_10804	1.472737609	-15.9995	0.07274	0.194116
SCE1572_3994	1.289887746	-14.0553	0.07276	0.194116
SCE1572_8312	-1.246970443	-13.8109	0.072917	0.194116
SCE1572_8357	2.816039354	-18.6498	0.072973	0.194116
SCE1572_8095	2.816039354	-18.6498	0.072973	0.194116
SCE1572_3606	2.816039354	-18.6498	0.072973	0.194116
SCE1572_2887	2.728576513	-18.6935	0.072973	0.194116
SCE1572_8668	2.728576513	-18.6935	0.072973	0.194116
SCE1572_11028	2.728576513	-18.6935	0.072973	0.194116
SCE1572_3621	2.728576513	-18.6935	0.072973	0.194116
SCE1572_9368	2.728576513	-18.6935	0.072973	0.194116
SCE1572_10320	2.728576513	-18.6935	0.072973	0.194116
SCE1572_9366	2.728576513	-18.6935	0.072973	0.194116
SCE1572_8092	2.728576513	-18.6935	0.072973	0.194116
SCE1572_8080	2.728576513	-18.6935	0.072973	0.194116
SCE1572_7718	1.304925883	-14.598	0.073012	0.194168
SCE1572_1243	-1.320333088	-14.8111	0.073102	0.194309
SCE1572_5447	1.460380402	-15.5202	0.073138	0.194309
SCE1572_4270	1.444783547	-15.528	0.073138	0.194309
SCE1572_5294	1.444783547	-15.528	0.073138	0.194309
SCE1572_8291	1.285794023	-14.2856	0.073224	0.194439
SCE1572_10409	-1.271423487	-14.2672	0.073224	0.194439
SCE1572_1203	1.321531804	-14.4901	0.073359	0.194568
SCE1572_456	1.561466527	-16.277	0.073402	0.194568
SCE1572_723	1.561466527	-16.277	0.073402	0.194568
SCE1572_266	1.561466527	-16.277	0.073402	0.194568
SCE1572_3790	1.561466527	-16.277	0.073402	0.194568
SCE1572_8042	1.535931435	-16.2898	0.073402	0.194568
SCE1572_3585	1.535931435	-16.2898	0.073402	0.194568
SCE1572_4911	-1.216281933	-12.8142	0.073593	0.195024
SCE1572_10585	1.382580658	-15.279	0.073839	0.195526
SCE1572_8499	1.369034126	-15.2858	0.073839	0.195526
SCE1572_4394	1.483464015	-15.7311	0.073894	0.195526
SCE1572_5867	1.483464015	-15.7311	0.073894	0.195526
SCE1572_3015	1.483464015	-15.7311	0.073894	0.195526
SCE1572_1828	1.483464015	-15.7311	0.073894	0.195526
SCE1572_6934	-1.219227276	-13.0975	0.073981	0.195613
SCE1572_302	1.667175968	-16.6392	0.074001	0.195613
SCE1572_8168	1.667175968	-16.6392	0.074001	0.195613
SCE1572_691	1.667175968	-16.6392	0.074001	0.195613
SCE1572_9089	1.323523102	-14.8111	0.07408	0.195674
SCE1572_3493	1.264629413	-13.9336	0.074102	0.195674
SCE1572_4970	1.85785953	-17.1288	0.074136	0.195674

SCE1572_6091	1.85785953	-17.1288	0.074136	0.195674
SCE1572_4315	1.85785953	-17.1288	0.074136	0.195674
SCE1572_2368	1.85785953	-17.1288	0.074136	0.195674
SCE1572_4379	1.391541526	-15.4551	0.074627	0.196921
SCE1572_4649	1.335234085	-14.9307	0.074729	0.196995
SCE1572_10630	1.335234085	-14.9307	0.074729	0.196995
SCE1572_3378	1.335234085	-14.9307	0.074729	0.196995
SCE1572_1426	-1.197820397	-12.1529	0.07473	0.196995
SCE1572_8682	-1.370959161	-15.8364	0.074784	0.197088
SCE1572_2098	-1.244456439	-13.9521	0.075038	0.197707
SCE1572_6972	1.292165363	-14.3242	0.075064	0.197728
SCE1572_8243	-1.211162962	-12.9423	0.07509	0.197746
SCE1572_9955	1.349162923	-15.0613	0.075479	0.198719
SCE1572_5352	-1.194802205	-12.2889	0.075504	0.198737
SCE1572_7905	1.437527731	-15.6386	0.075563	0.198793
SCE1572_4986	-1.353885647	-15.5648	0.075563	0.198793
SCE1572_783	1.298432121	-14.4086	0.075611	0.19887
SCE1572_303	1.271718838	-14.2926	0.075744	0.199146
SCE1572_169	-1.235503913	-13.7807	0.075754	0.199146
SCE1572_5806	-1.448301249	-16.0815	0.075828	0.199289
SCE1572_9363	-1.341812815	-15.3364	0.076042	0.199798
SCE1572_4383	1.287543805	-14.6067	0.076097	0.199798
SCE1572_3835	1.287543805	-14.6067	0.076097	0.199798
SCE1572_11211	1.287543805	-14.6067	0.076097	0.199798
SCE1572_4364	1.305501695	-14.4981	0.076222	0.200076
SCE1572_5472	1.276639431	-14.3751	0.076324	0.200243
SCE1572_1148	-1.271423487	-14.3536	0.076324	0.200243
SCE1572_1633	-1.254935364	-14.2423	0.076408	0.200414
SCE1572_10484	1.268551559	-14.1756	0.076478	0.200549
SCE1572_5557	-1.210104508	-13.1373	0.076601	0.200723
SCE1572_441	1.221616524	-13.1071	0.07662	0.200723
SCE1572_10370	1.47859826	-15.859	0.076689	0.200723
SCE1572_11299	1.458969453	-15.8689	0.076689	0.200723
SCE1572_10782	1.458969453	-15.8689	0.076689	0.200723
SCE1572_676	1.458969453	-15.8689	0.076689	0.200723
SCE1572_10744	1.334297574	-14.8671	0.076717	0.200723
SCE1572_4391	1.334297574	-14.8671	0.076717	0.200723
SCE1572_5226	-1.296085541	-14.8232	0.076717	0.200723
SCE1572_9220	1.187193156	-11.4286	0.076817	0.20091
SCE1572_11070	-1.271423487	-14.6711	0.076826	0.20091
SCE1572_8156	1.272897029	-14.2514	0.077056	0.201411
SCE1572_8840	1.272897029	-14.2514	0.077056	0.201411
SCE1572_5042	-1.241049838	-14.1237	0.077086	0.201439
SCE1572_733	1.386787996	-15.3644	0.077387	0.202124
SCE1572_1178	1.386787996	-15.3644	0.077387	0.202124
SCE1572_1928	-1.211302495	-13.1088	0.077516	0.202396
SCE1572_262	1.32613905	-15.1468	0.077529	0.202396
SCE1572_2544	1.231076853	-13.5353	0.077591	0.202493

SCE1572_11469	-1.297895698	-14.9254	0.077612	0.202493
SCE1572_4255	-1.214392542	-13.7108	0.077624	0.202493
SCE1572_5608	1.288448033	-14.5556	0.077696	0.202592
SCE1572_4728	1.200809414	-12.7849	0.077701	0.202592
SCE1572_2715	-1.202261463	-12.8516	0.077725	0.202606
SCE1572_3288	-1.197041291	-13.0941	0.077814	0.202787
SCE1572_6778	1.21535862	-13.2022	0.07785	0.20283
SCE1572_809	-1.180820938	-11.8933	0.078	0.203127
SCE1572_9630	1.535931435	-16.1199	0.078056	0.203127
SCE1572_4135	1.535931435	-16.1199	0.078056	0.203127
SCE1572_6840	-1.441348488	-16.0236	0.078056	0.203127
SCE1572_6057	-1.231895123	-13.9188	0.078178	0.203127
SCE1572_11282	-1.231895123	-13.9188	0.078178	0.203127
SCE1572_10503	-1.551531406	-16.7461	0.078188	0.203127
SCE1572_5621	1.252075742	-14.1098	0.078261	0.203127
SCE1572_4811	-1.255656171	-14.1778	0.078307	0.203127
SCE1572_1041	1.228291727	-13.5129	0.078373	0.203127
SCE1572_2806	1.298426961	-14.5293	0.07846	0.203127
SCE1572_2733	-1.361621296	-15.6942	0.078469	0.203127
SCE1572_1085	1.304605889	-14.6506	0.078515	0.203127
SCE1572_9576	1.950968934	-17.4973	0.078524	0.203127
SCE1572_6859	1.950968934	-17.4973	0.078524	0.203127
SCE1572_2449	1.950968934	-17.4973	0.078524	0.203127
SCE1572_2255	1.950968934	-17.4973	0.078524	0.203127
SCE1572_10726	1.950968934	-17.4973	0.078524	0.203127
SCE1572_4361	1.950968934	-17.4973	0.078524	0.203127
SCE1572_1332	1.950968934	-17.4973	0.078524	0.203127
SCE1572_2896	1.950968934	-17.4973	0.078524	0.203127
SCE1572_11452	1.898501514	-17.5236	0.078524	0.203127
SCE1572_381	1.898501514	-17.5236	0.078524	0.203127
SCE1572_9136	1.898501514	-17.5236	0.078524	0.203127
SCE1572_7811	1.898501514	-17.5236	0.078524	0.203127
SCE1572_245	1.890464195	-17.5276	0.078524	0.203127
SCE1572_4752	-1.856385988	-17.401	0.078524	0.203127
SCE1572_6933	-1.856385988	-17.401	0.078524	0.203127
SCE1572_9400	-1.686460986	-17.316	0.078524	0.203127
SCE1572_7063	1.236060261	-13.7673	0.078566	0.203148
SCE1572_7429	1.429016231	-15.5359	0.078625	0.203148
SCE1572_6236	1.429016231	-15.5359	0.078625	0.203148
SCE1572_2922	1.413074687	-15.5439	0.078625	0.203148
SCE1572_3970	-1.300569833	-15.0642	0.078655	0.203148
SCE1572_9979	1.355359189	-15.2926	0.078667	0.203148
SCE1572_5436	1.355359189	-15.2926	0.078667	0.203148
SCE1572_374	1.269144894	-14.3788	0.079097	0.204208
SCE1572_2333	-1.204309291	-13.4603	0.079132	0.204248
SCE1572_4722	-1.220349302	-13.8735	0.079187	0.20434
SCE1572_11351	1.190646404	-12.2433	0.079229	0.204397
SCE1572_4592	-1.250664927	-14.5746	0.07932	0.204583

SCE1572_8580	1.188008132	-12.3977	0.07946	0.204894
SCE1572_9674	-1.204752447	-13.4507	0.079504	0.204956
SCE1572_9119	1.621661309	-16.4396	0.079722	0.205368
SCE1572_2521	1.621661309	-16.4396	0.079722	0.205368
SCE1572_9253	1.621661309	-16.4396	0.079722	0.205368
SCE1572_3475	-1.303132347	-15.1858	0.079888	0.205694
SCE1572_9398	-1.303132347	-15.1858	0.079888	0.205694
SCE1572_4804	1.27422609	-14.4665	0.079926	0.205741
SCE1572_1447	1.376274769	-15.4627	0.080032	0.205876
SCE1572_1769	1.451042537	-16.0103	0.080111	0.205876
SCE1572_7352	1.451042537	-16.0103	0.080111	0.205876
SCE1572_2548	1.451042537	-16.0103	0.080111	0.205876
SCE1572_870	-1.378338691	-15.9396	0.080111	0.205876
SCE1572_5381	1.465542107	-15.74	0.080134	0.205876
SCE1572_7522	1.465542107	-15.74	0.080134	0.205876
SCE1572_376	1.465542107	-15.74	0.080134	0.205876
SCE1572_6884	1.285524637	-14.7146	0.080298	0.206246
SCE1572_4565	-1.204309291	-13.6827	0.080334	0.206286
SCE1572_10391	1.32395563	-14.8722	0.08052	0.206665
SCE1572_7232	1.32395563	-14.8722	0.08052	0.206665
SCE1572_537	-1.206468045	-13.4131	0.080587	0.206784
SCE1572_10721	1.170984984	-11.5181	0.080606	0.206784
SCE1572_5467	1.353067378	-15.2113	0.081052	0.207878
SCE1572_10571	-1.168885325	-11.8349	0.081338	0.208561
SCE1572_5548	1.293361132	-14.8261	0.08142	0.208581
SCE1572_6875	1.293361132	-14.8261	0.08142	0.208581
SCE1572_4794	-1.159356686	-10.3637	0.081496	0.208581
SCE1572_9045	1.229146826	-13.9838	0.081499	0.208581
SCE1572_10942	1.193825458	-12.969	0.081665	0.208581
SCE1572_4047	1.403176226	-15.6557	0.081697	0.208581
SCE1572_3566	1.403176226	-15.6557	0.081697	0.208581
SCE1572_1138	1.764200423	-16.8537	0.081703	0.208581
SCE1572_6905	1.764200423	-16.8537	0.081703	0.208581
SCE1572_2576	1.764200423	-16.8537	0.081703	0.208581
SCE1572_5628	1.764200423	-16.8537	0.081703	0.208581
SCE1572_7320	1.764200423	-16.8537	0.081703	0.208581
SCE1572_1964	1.764200423	-16.8537	0.081703	0.208581
SCE1572_9004	1.764200423	-16.8537	0.081703	0.208581
SCE1572_722	1.761382658	-16.8552	0.081703	0.208581
SCE1572_3912	-1.534457893	-16.6551	0.081703	0.208581
SCE1572_7926	-1.534457893	-16.6551	0.081703	0.208581
SCE1572_6269	-1.534457893	-16.6551	0.081703	0.208581
SCE1572_10495	-1.203251984	-13.7052	0.081779	0.208724
SCE1572_8325	-1.188961327	-13.2429	0.081951	0.209111
SCE1572_485	1.313539014	-15.1531	0.082164	0.209518
SCE1572_5272	1.313539014	-15.1531	0.082164	0.209518
SCE1572_1515	-1.464068565	-16.2048	0.08217	0.209518
SCE1572_4801	-1.271423487	-14.7628	0.082288	0.209615

SCE1572_7854	-1.271423487	-14.7628	0.082288	0.209615
SCE1572_4056	-1.247964514	-14.751	0.082288	0.209615
SCE1572_5290	1.281117536	-14.5103	0.082308	0.209615
SCE1572_1847	1.281117536	-14.5103	0.082308	0.209615
SCE1572_2490	1.302567896	-14.9471	0.08272	0.210615
SCE1572_8385	1.214216052	-13.9265	0.082972	0.211176
SCE1572_8548	1.25819598	-14.4745	0.083001	0.211176
SCE1572_9554	-1.233455637	-14.4457	0.083001	0.211176
SCE1572_10562	1.196285234	-13.1559	0.08325	0.21176
SCE1572_7365	-1.167993897	-12.4323	0.083305	0.211846
SCE1572_948	1.275266715	-14.5959	0.083434	0.212124
SCE1572_695	-1.856385988	-17.816	0.083497	0.212233
SCE1572_5446	1.439069896	-15.8788	0.083771	0.212716
SCE1572_8810	1.439069896	-15.8788	0.083771	0.212716
SCE1572_5485	1.168475	-12.0727	0.083795	0.212716
SCE1572_10206	1.164629428	-11.8544	0.083809	0.212716
SCE1572_5403	1.327614199	-15.3065	0.083829	0.212716
SCE1572_6616	1.327614199	-15.3065	0.083829	0.212716
SCE1572_10156	-1.304590351	-15.2506	0.083829	0.212716
SCE1572_6734	-1.271423487	-14.6935	0.083935	0.212936
SCE1572_8891	1.241389228	-14.0795	0.084155	0.213345
SCE1572_10476	-1.251245605	-14.5337	0.084157	0.213345
SCE1572_4945	-1.231346048	-14.5237	0.084157	0.213345
SCE1572_6341	1.325511655	-15.0731	0.084245	0.213412
SCE1572_5284	-1.271423487	-15.0211	0.084245	0.213412
SCE1572_2359	-1.271423487	-15.0211	0.084245	0.213412
SCE1572_5776	-1.204309291	-13.9457	0.084265	0.213412
SCE1572_2597	-2.690535248	-18.8399	0.084472	0.213817
SCE1572_9058	1.313539014	-14.8774	0.084523	0.213817
SCE1572_2370	-1.271423487	-14.8355	0.084523	0.213817
SCE1572_7088	1.396955022	-15.5519	0.084547	0.213817
SCE1572_7275	1.396955022	-15.5519	0.084547	0.213817
SCE1572_2808	1.379961303	-15.5379	0.084547	0.213817
SCE1572_1492	1.286995773	-14.6956	0.084717	0.213939
SCE1572_9468	-1.204928075	-13.9323	0.08474	0.213939
SCE1572_4530	1.635467109	-16.6551	0.084778	0.213939
SCE1572_9159	1.635467109	-16.6551	0.084778	0.213939
SCE1572_375	1.635467109	-16.6551	0.084778	0.213939
SCE1572_7274	1.603045631	-16.6713	0.084778	0.213939
SCE1572_2860	1.603045631	-16.6713	0.084778	0.213939
SCE1572_1450	-1.431888159	-16.5258	0.084778	0.213939
SCE1572_3210	-1.431888159	-16.5258	0.084778	0.213939
SCE1572_8941	-1.217652231	-14.3628	0.084966	0.21432
SCE1572_1320	-1.271423487	-15.2016	0.08497	0.21432
SCE1572_1443	1.238131611	-14.2688	0.085047	0.214464
SCE1572_4215	-1.145892605	-10.7119	0.085085	0.214506
SCE1572_5423	1.210639106	-13.9289	0.085205	0.214741
SCE1572_11157	1.285321695	-15.029	0.085219	0.214741

SCE1572_8391	1.283165365	-14.8312	0.085373	0.214753
SCE1572_10256	1.272897029	-14.8364	0.085373	0.214753
SCE1572_4570	1.272897029	-14.8364	0.085373	0.214753
SCE1572_7380	1.272897029	-14.8364	0.085373	0.214753
SCE1572_4614	1.272897029	-14.8364	0.085373	0.214753
SCE1572_172	1.272897029	-14.8364	0.085373	0.214753
SCE1572_11154	-1.364532891	-15.74	0.085407	0.214753
SCE1572_1043	-1.317227177	-15.7164	0.085407	0.214753
SCE1572_3403	-1.317227177	-15.7164	0.085407	0.214753
SCE1572_11201	-1.215928374	-13.9933	0.085651	0.215315
SCE1572_8246	-1.308898192	-15.4268	0.08585	0.215764
SCE1572_9759	1.340011225	-15.2178	0.086056	0.21623
SCE1572_9837	-1.168329994	-13.0797	0.086185	0.216502
SCE1572_92	-1.159094787	-12.689	0.086243	0.216596
SCE1572_5330	1.512847822	-16.1314	0.086418	0.216827
SCE1572_3030	1.489388849	-16.1432	0.086418	0.216827
SCE1572_7253	1.489388849	-16.1432	0.086418	0.216827
SCE1572_4955	1.489388849	-16.1432	0.086418	0.216827
SCE1572_275	1.228650116	-14.3142	0.086774	0.217669
SCE1572_11003	-1.157766705	-12.7059	0.086864	0.217728
SCE1572_10617	1.44739476	-15.7491	0.086932	0.217728
SCE1572_2709	1.44739476	-15.7491	0.086932	0.217728
SCE1572_9578	1.44739476	-15.7491	0.086932	0.217728
SCE1572_8389	1.429016231	-15.7583	0.086932	0.217728
SCE1572_10137	1.191860457	-13.3744	0.086937	0.217728
SCE1572_8868	1.274544882	-14.7766	0.086984	0.217728
SCE1572_5808	1.274544882	-14.7766	0.086984	0.217728
SCE1572_11262	-1.248339874	-14.7278	0.086984	0.217728
SCE1572_9201	1.291512707	-14.9526	0.087048	0.217786
SCE1572_1856	1.291512707	-14.9526	0.087048	0.217786
SCE1572_1630	1.300827962	-15.1594	0.087092	0.217843
SCE1572_8466	-1.190286724	-13.7341	0.0873	0.218313
SCE1572_6839	1.526142118	-16.5377	0.087489	0.218734
SCE1572_69	1.256955485	-14.7289	0.087746	0.219296
SCE1572_10683	-1.161798996	-13.0688	0.087756	0.219296
SCE1572_8857	-1.245888395	-14.8734	0.087908	0.21944
SCE1572_465	-1.245888395	-14.8734	0.087908	0.21944
SCE1572_9958	1.816039354	-17.1498	0.088001	0.21944
SCE1572_3049	1.816039354	-17.1498	0.088001	0.21944
SCE1572_2680	1.816039354	-17.1498	0.088001	0.21944
SCE1572_7208	1.772970632	-17.1713	0.088001	0.21944
SCE1572_6102	1.772970632	-17.1713	0.088001	0.21944
SCE1572_7141	1.772970632	-17.1713	0.088001	0.21944
SCE1572_3578	-1.718882464	-17.0103	0.088001	0.21944
SCE1572_6048	-1.149551484	-12.2706	0.088201	0.219592
SCE1572_4164	1.357933133	-15.3788	0.088244	0.219592
SCE1572_3909	1.357933133	-15.3788	0.088244	0.219592
SCE1572_6532	1.343286357	-15.3861	0.088244	0.219592

SCE1572_8611	1.343286357	-15.3861	0.088244	0.219592
SCE1572_11381	-1.306188905	-15.3186	0.088244	0.219592
SCE1572_4061	-1.306188905	-15.3186	0.088244	0.219592
SCE1572_2983	-1.434922219	-15.9679	0.088271	0.219592
SCE1572_4126	1.429016231	-16.0213	0.088271	0.219592
SCE1572_9787	1.406648418	-16.0325	0.088271	0.219592
SCE1572_4894	1.367986798	-15.6733	0.088356	0.219648
SCE1572_11493	1.367986798	-15.6733	0.088356	0.219648
SCE1572_7732	-1.356312385	-15.6067	0.088356	0.219648
SCE1572_3068	-1.218312151	-14.345	0.088658	0.220346
SCE1572_3341	-1.139395093	-11.7206	0.088722	0.220432
SCE1572_1783	-1.246332506	-14.8481	0.088734	0.220432
SCE1572_5658	1.179521666	-13.5138	0.088833	0.220624
SCE1572_2365	-1.148224422	-12.6207	0.088933	0.220821
SCE1572_11503	1.301466181	-15.0851	0.089023	0.220835
SCE1572_4502	1.289291467	-15.0912	0.089023	0.220835
SCE1572_2282	-1.271423487	-15.0496	0.089023	0.220835
SCE1572_11263	-1.242854335	-15.0353	0.089023	0.220835
SCE1572_6278	-1.201882554	-14.3189	0.08923	0.221297
SCE1572_3134	1.313539014	-15.3135	0.089347	0.22143
SCE1572_1544	1.313539014	-15.3135	0.089347	0.22143
SCE1572_6313	1.313539014	-15.3135	0.089347	0.22143
SCE1572_8950	1.262555085	-14.8415	0.089529	0.221829
SCE1572_11072	-1.179164979	-13.9194	0.089863	0.22206
SCE1572_9084	-1.208229661	-14.5958	0.089879	0.22206
SCE1572_7086	1.593646933	-16.4536	0.089896	0.22206
SCE1572_10816	1.593646933	-16.4536	0.089896	0.22206
SCE1572_3919	1.593646933	-16.4536	0.089896	0.22206
SCE1572_5669	1.593646933	-16.4536	0.089896	0.22206
SCE1572_2466	1.565077781	-16.4679	0.089896	0.22206
SCE1572_122	1.565077781	-16.4679	0.089896	0.22206
SCE1572_1917	1.565077781	-16.4679	0.089896	0.22206
SCE1572_4264	1.565077781	-16.4679	0.089896	0.22206
SCE1572_4085	1.565077781	-16.4679	0.089896	0.22206
SCE1572_7100	-1.408927011	-16.3028	0.089896	0.22206
SCE1572_6124	-1.408927011	-16.3028	0.089896	0.22206
SCE1572_9772	-1.37451698	-15.8871	0.089974	0.222121
SCE1572_6283	-1.32204956	-15.8608	0.089974	0.222121
SCE1572_10697	-1.160062378	-13.298	0.089985	0.222121
SCE1572_7572	-1.208749124	-14.4333	0.090135	0.222392
SCE1572_3943	-1.124496078	-9.95558	0.090137	0.222392
SCE1572_10021	1.226293954	-14.3075	0.090328	0.222764
SCE1572_2667	1.170646971	-13.2245	0.09033	0.222764
SCE1572_11543	1.223987429	-14.2759	0.090855	0.223402
SCE1572_3170	-1.205080992	-14.2509	0.090855	0.223402
SCE1572_3226	1.38065321	-15.5601	0.090939	0.223402
SCE1572_10596	1.38065321	-15.5601	0.090939	0.223402
SCE1572_9367	1.38065321	-15.5601	0.090939	0.223402

SCE1572_6915	1.364165087	-15.5683	0.090939	0.223402
SCE1572_4964	-1.271423487	-15.484	0.090939	0.223402
SCE1572_2894	1.207548318	-14.1321	0.090956	0.223402
SCE1572_3773	1.264629413	-14.7816	0.091029	0.223402
SCE1572_10754	1.264629413	-14.7816	0.091029	0.223402
SCE1572_5337	1.172940683	-13.342	0.091033	0.223402
SCE1572_6066	2.252138469	-17.9317	0.091098	0.223402
SCE1572_10366	2.252138469	-17.9317	0.091098	0.223402
SCE1572_10538	2.252138469	-17.9317	0.091098	0.223402
SCE1572_11294	2.252138469	-17.9317	0.091098	0.223402
SCE1572_2827	2.252138469	-17.9317	0.091098	0.223402
SCE1572_4554	2.252138469	-17.9317	0.091098	0.223402
SCE1572_5934	2.188008132	-17.9638	0.091098	0.223402
SCE1572_3062	2.188008132	-17.9638	0.091098	0.223402
SCE1572_8404	2.188008132	-17.9638	0.091098	0.223402
SCE1572_502	2.188008132	-17.9638	0.091098	0.223402
SCE1572_6515	2.188008132	-17.9638	0.091098	0.223402
SCE1572_1631	2.188008132	-17.9638	0.091098	0.223402
SCE1572_1151	-1.78599666	-17.6288	0.091098	0.223402
SCE1572_3612	1.245634949	-14.577	0.091186	0.223513
SCE1572_10565	1.236917732	-14.5813	0.091186	0.223513
SCE1572_10186	-1.199273701	-14.372	0.091293	0.223723
SCE1572_6222	1.313539014	-15.2311	0.091386	0.223898
SCE1572_2863	1.418892014	-15.8889	0.091543	0.224074
SCE1572_7824	1.398427911	-15.8991	0.091543	0.224074
SCE1572_10424	1.398427911	-15.8991	0.091543	0.224074
SCE1572_5019	-1.320333088	-15.8111	0.091543	0.224074
SCE1572_10441	-1.570983769	-16.8433	0.091583	0.22412
SCE1572_445	1.269144894	-14.9638	0.091614	0.224145
SCE1572_4252	1.483464015	-16.316	0.092047	0.225053
SCE1572_6728	-1.40266802	-16.2355	0.092047	0.225053
SCE1572_5318	1.329480558	-15.4861	0.092114	0.225053
SCE1572_3537	1.329480558	-15.4861	0.092114	0.225053
SCE1572_6496	1.329480558	-15.4861	0.092114	0.225053
SCE1572_5593	1.329480558	-15.4861	0.092114	0.225053
SCE1572_8661	1.185705346	-13.632	0.092205	0.225222
SCE1572_10961	-1.271423487	-15.1389	0.09233	0.225476
SCE1572_10378	1.2185425	-14.361	0.092385	0.225559
SCE1572_5617	1.247950672	-14.5269	0.09241	0.225567
SCE1572_5451	1.183142376	-13.7113	0.092579	0.225927
SCE1572_3580	1.19324478	-13.8173	0.092633	0.226007
SCE1572_2426	-1.124491383	-11.6172	0.092722	0.226098
SCE1572_8650	1.353067378	-15.7963	0.092993	0.226098
SCE1572_6671	1.353067378	-15.7963	0.092993	0.226098
SCE1572_4309	1.333438571	-15.8061	0.092993	0.226098
SCE1572_2799	1.333438571	-15.8061	0.092993	0.226098
SCE1572_9405	1.333438571	-15.8061	0.092993	0.226098
SCE1572_11220	1.333438571	-15.8061	0.092993	0.226098

SCE1572_3837	1.240475551	-14.6827	0.093082	0.226098
SCE1572_213	1.281830154	-14.8933	0.093167	0.226098
SCE1572_3169	-1.151129253	-13.3024	0.093205	0.226098
SCE1572_4020	-1.307949363	-15.3898	0.09322	0.226098
SCE1572_9145	-1.307949363	-15.3898	0.09322	0.226098
SCE1572_11175	-1.138837847	-13.0803	0.093228	0.226098
SCE1572_1168	1.150458393	-12.959	0.093306	0.226098
SCE1572_11204	-1.15594627	-13.4658	0.093375	0.226098
SCE1572_9687	-1.129920806	-12.5893	0.09379	0.226098
SCE1572_3923	1.252138469	-14.8467	0.093896	0.226098
SCE1572_413	1.241646095	-14.852	0.093896	0.226098
SCE1572_9543	1.241646095	-14.852	0.093896	0.226098
SCE1572_3782	-1.222513887	-14.8111	0.093896	0.226098
SCE1572_1675	-1.222513887	-14.8111	0.093896	0.226098
SCE1572_3871	-1.188007479	-14.2595	0.093913	0.226098
SCE1572_8355	1.287354194	-15.1045	0.094086	0.226098
SCE1572_10248	1.277013138	-15.0973	0.094086	0.226098
SCE1572_3804	1.277013138	-15.0973	0.094086	0.226098
SCE1572_1721	31.68685379	-34.1726	0.094203	0.226098
SCE1572_7057	31.68685379	-34.1726	0.094203	0.226098
SCE1572_10808	31.68685379	-34.1726	0.094203	0.226098
SCE1572_6540	31.68685379	-34.1726	0.094203	0.226098
SCE1572_8203	31.68685379	-34.1726	0.094203	0.226098
SCE1572_11585	31.68685379	-34.1726	0.094203	0.226098
SCE1572_5208	31.68685379	-34.1726	0.094203	0.226098
SCE1572_2514	31.68685379	-34.1726	0.094203	0.226098
SCE1572_10244	31.68685379	-34.1726	0.094203	0.226098
SCE1572_4927	31.68685379	-34.1726	0.094203	0.226098
SCE1572_9613	31.68685379	-34.1726	0.094203	0.226098
SCE1572_8669	31.68685379	-34.1726	0.094203	0.226098
SCE1572_4413	31.68685379	-34.1726	0.094203	0.226098
SCE1572_2489	31.68685379	-34.1726	0.094203	0.226098
SCE1572_196	31.68685379	-34.1726	0.094203	0.226098
SCE1572_3061	31.68685379	-34.1726	0.094203	0.226098
SCE1572_8747	31.68685379	-34.1726	0.094203	0.226098
SCE1572_5050	31.68685379	-34.1726	0.094203	0.226098
SCE1572_6554	31.68685379	-34.1726	0.094203	0.226098
SCE1572_9267	31.68685379	-34.1726	0.094203	0.226098
SCE1572_3655	31.68685379	-34.1726	0.094203	0.226098
SCE1572_9937	31.68685379	-34.1726	0.094203	0.226098
SCE1572_10843	31.68685379	-34.1726	0.094203	0.226098
SCE1572_460	31.49420871	-34.2689	0.094203	0.226098
SCE1572_5629	31.49420871	-34.2689	0.094203	0.226098
SCE1572_1333	31.49420871	-34.2689	0.094203	0.226098
SCE1572_2004	31.49420871	-34.2689	0.094203	0.226098
SCE1572_8793	31.49420871	-34.2689	0.094203	0.226098
SCE1572_2949	31.49420871	-34.2689	0.094203	0.226098
SCE1572_7734	31.49420871	-34.2689	0.094203	0.226098

SCE1572_4076	31.49420871	-34.2689	0.094203	0.226098
SCE1572_6036	31.49420871	-34.2689	0.094203	0.226098
SCE1572_8152	31.49420871	-34.2689	0.094203	0.226098
SCE1572_2090	31.49420871	-34.2689	0.094203	0.226098
SCE1572_8023	31.49420871	-34.2689	0.094203	0.226098
SCE1572_1704	31.49420871	-34.2689	0.094203	0.226098
SCE1572_1692	31.49420871	-34.2689	0.094203	0.226098
SCE1572_9278	31.49420871	-34.2689	0.094203	0.226098
SCE1572_9245	31.49420871	-34.2689	0.094203	0.226098
SCE1572_10583	31.49420871	-34.2689	0.094203	0.226098
SCE1572_3211	31.49420871	-34.2689	0.094203	0.226098
SCE1572_5986	31.49420871	-34.2689	0.094203	0.226098
SCE1572_8478	31.49420871	-34.2689	0.094203	0.226098
SCE1572_3836	31.49420871	-34.2689	0.094203	0.226098
SCE1572_3361	31.49420871	-34.2689	0.094203	0.226098
SCE1572_7392	31.47970914	-34.2762	0.094203	0.226098
SCE1572_9122	1.159368084	-13.2686	0.094218	0.226098
SCE1572_939	1.328489355	-15.3935	0.094262	0.226101
SCE1572_10198	1.328489355	-15.3935	0.094262	0.226101
SCE1572_1566	1.410400553	-15.7676	0.094337	0.226229
SCE1572_716	1.169803536	-13.6149	0.094534	0.22633
SCE1572_3369	-1.123324848	-12.41	0.094589	0.22633
SCE1572_2025	-1.168853753	-14.0723	0.094615	0.22633
SCE1572_7132	1.728576513	-16.8716	0.094618	0.22633
SCE1572_2162	1.728576513	-16.8716	0.094618	0.22633
SCE1572_9912	1.728576513	-16.8716	0.094618	0.22633
SCE1572_9654	1.702738681	-16.8845	0.094618	0.22633
SCE1572_5605	1.692050637	-16.8898	0.094618	0.22633
SCE1572_4546	1.692050637	-16.8898	0.094618	0.22633
SCE1572_8413	1.692050637	-16.8898	0.094618	0.22633
SCE1572_3745	1.692050637	-16.8898	0.094618	0.22633
SCE1572_1676	-1.198360025	-14.3902	0.09464	0.22633
SCE1572_10273	-1.153586997	-13.495	0.094659	0.22633
SCE1572_6281	-1.127015832	-12.5391	0.094865	0.22677
SCE1572_2773	1.124591576	-12.1118	0.095002	0.227047
SCE1572_7949	-1.153028786	-13.3864	0.095032	0.227067
SCE1572_9327	-1.133261048	-12.9878	0.095138	0.227268
SCE1572_2213	-1.153853891	-13.9068	0.095169	0.22729
SCE1572_5691	1.284969861	-15.3278	0.095247	0.227331
SCE1572_2944	1.284969861	-15.3278	0.095247	0.227331
SCE1572_7391	1.254645325	-14.7866	0.095272	0.227331
SCE1572_4284	1.24459166	-14.7916	0.095272	0.227331
SCE1572_656	-1.110451568	-10.8024	0.095457	0.227688
SCE1572_7585	-1.135286799	-13.0405	0.095498	0.227688
SCE1572_5079	-1.271423487	-15.606	0.095587	0.227688
SCE1572_10728	-1.271423487	-15.606	0.095587	0.227688
SCE1572_6251	1.169149104	-13.6658	0.095678	0.227688
SCE1572_5300	1.290455401	-15.0202	0.095685	0.227688

SCE1572_8752	1.465542107	-16.1551	0.095725	0.227688
SCE1572_1520	1.465542107	-16.1551	0.095725	0.227688
SCE1572_3561	1.465542107	-16.1551	0.095725	0.227688
SCE1572_5382	1.441294561	-16.1672	0.095725	0.227688
SCE1572_10341	1.441294561	-16.1672	0.095725	0.227688
SCE1572_1414	1.441294561	-16.1672	0.095725	0.227688
SCE1572_6412	1.441294561	-16.1672	0.095725	0.227688
SCE1572_2396	-1.386900704	-16.0508	0.095725	0.227688
SCE1572_5636	-1.225619797	-14.7164	0.095921	0.227846
SCE1572_10847	1.217961354	-14.7484	0.095921	0.227846
SCE1572_1482	1.217961354	-14.7484	0.095921	0.227846
SCE1572_5517	1.217961354	-14.7484	0.095921	0.227846
SCE1572_7087	1.217961354	-14.7484	0.095921	0.227846
SCE1572_308	-1.203251984	-14.7052	0.095921	0.227846
SCE1572_4385	1.153882348	-13.3516	0.096056	0.228115
SCE1572_7133	1.191750533	-14.3326	0.096145	0.228171
SCE1572_5919	-1.185008735	-14.3104	0.096145	0.228171
SCE1572_10084	-1.185008735	-14.3104	0.096145	0.228171
SCE1572_4865	-1.271423487	-15.2341	0.096179	0.228198
SCE1572_146	1.198061796	-14.1733	0.0962	0.228198
SCE1572_1970	1.246424818	-14.9751	0.096432	0.228645
SCE1572_7386	1.246424818	-14.9751	0.096432	0.228645
SCE1572_68	-1.111462872	-11.7933	0.096528	0.228823
SCE1572_11334	-1.142690173	-13.4003	0.096559	0.228843
SCE1572_6893	1.181435478	-14.1451	0.096623	0.228891
SCE1572_7906	1.181435478	-14.1451	0.096623	0.228891
SCE1572_9927	-1.127347309	-12.8467	0.096659	0.228918
SCE1572_8860	1.84405373	-17.5508	0.096786	0.228918
SCE1572_2857	1.84405373	-17.5508	0.096786	0.228918
SCE1572_9476	1.84405373	-17.5508	0.096786	0.228918
SCE1572_3010	1.787470202	-17.5791	0.096786	0.228918
SCE1572_486	1.787470202	-17.5791	0.096786	0.228918
SCE1572_444	1.787470202	-17.5791	0.096786	0.228918
SCE1572_764	1.286571966	-15.2446	0.097062	0.229327
SCE1572_2069	1.195276132	-14.2902	0.097079	0.229327
SCE1572_3058	1.231076853	-14.6874	0.097149	0.229327
SCE1572_1248	-1.136881364	-13.5709	0.097163	0.229327
SCE1572_9347	1.569878767	-16.6879	0.097221	0.229327
SCE1572_1521	1.569878767	-16.6879	0.097221	0.229327
SCE1572_10013	1.569878767	-16.6879	0.097221	0.229327
SCE1572_823	1.569878767	-16.6879	0.097221	0.229327
SCE1572_3310	1.569878767	-16.6879	0.097221	0.229327
SCE1572_4123	1.569878767	-16.6879	0.097221	0.229327
SCE1572_3840	1.535931435	-16.7048	0.097221	0.229327
SCE1572_1011	1.535931435	-16.7048	0.097221	0.229327
SCE1572_10551	1.383928342	-16.0439	0.097309	0.229381
SCE1572_7766	1.383928342	-16.0439	0.097309	0.229381
SCE1572_2501	-1.382454799	-15.9941	0.097309	0.229381

SCE1572_8562	-1.117949006	-12.4171	0.097354	0.22939
SCE1572_8512	1.163625269	-13.9524	0.097357	0.22939
SCE1572_7523	1.164675628	-13.803	0.097613	0.229942
SCE1572_10372	1.347486346	-15.5767	0.09784	0.230373
SCE1572_1963	1.347486346	-15.5767	0.09784	0.230373
SCE1572_4125	1.262008713	-15.1788	0.097899	0.23041
SCE1572_11459	-1.240396591	-15.1544	0.097899	0.23041
SCE1572_5119	-1.118266699	-12.6511	0.098021	0.230646
SCE1572_10208	1.199882232	-14.4134	0.098115	0.230712
SCE1572_3539	1.199882232	-14.4134	0.098115	0.230712
SCE1572_10566	1.191977034	-14.4174	0.098115	0.230712
SCE1572_90	-1.118231635	-12.8225	0.098417	0.231369
SCE1572_1894	1.231076853	-14.8573	0.098488	0.231433
SCE1572_2180	1.231076853	-14.8573	0.098488	0.231433
SCE1572_8093	1.313539014	-15.4941	0.098858	0.232148
SCE1572_4103	1.313539014	-15.4941	0.098858	0.232148
SCE1572_10802	1.297419348	-15.5022	0.098858	0.232148
SCE1572_9473	-1.158948758	-13.8428	0.099067	0.232587
SCE1572_9920	1.158564354	-13.7236	0.099157	0.232746
SCE1572_8756	1.132966768	-13.0819	0.099443	0.233364
SCE1572_11254	-1.137406139	-13.8063	0.099702	0.23392
SCE1572_1184	1.133560348	-13.1511	0.09981	0.234122
SCE1572_10395	2.635467109	-18.74	0.099976	0.234249
SCE1572_11600	2.635467109	-18.74	0.099976	0.234249
SCE1572_4792	2.635467109	-18.74	0.099976	0.234249
SCE1572_238	2.635467109	-18.74	0.099976	0.234249
SCE1572_1088	2.635467109	-18.74	0.099976	0.234249
SCE1572_1319	1.377669351	-15.9095	0.100074	0.234428
SCE1572_7026	1.208045885	-14.7533	0.100304	0.234862
SCE1572_10533	-1.179793012	-14.7169	0.100304	0.234862
SCE1572_3244	-1.153586997	-14.08	0.100347	0.23488
SCE1572_4176	1.188008132	-14.2938	0.100357	0.23488
SCE1572_9760	-1.131872135	-13.6237	0.10056	0.235304
SCE1572_868	-1.141788207	-13.7583	0.100606	0.235359
SCE1572_5584	1.313539014	-15.401	0.100711	0.235396
SCE1572_6308	1.313539014	-15.401	0.100711	0.235396
SCE1572_5933	1.298432121	-15.4086	0.100711	0.235396
SCE1572_9254	1.298432121	-15.4086	0.100711	0.235396
SCE1572_9708	-1.144891081	-13.7233	0.101179	0.236376
SCE1572_9983	1.196182063	-14.4597	0.101281	0.236376
SCE1572_8765	1.196182063	-14.4597	0.101281	0.236376
SCE1572_9237	1.313539014	-15.816	0.101288	0.236376
SCE1572_11522	1.313539014	-15.816	0.101288	0.236376
SCE1572_6093	1.313539014	-15.816	0.101288	0.236376
SCE1572_9051	1.313539014	-15.816	0.101288	0.236376
SCE1572_3335	-1.135908516	-13.5813	0.101342	0.236449
SCE1572_4163	1.221616524	-14.6921	0.101402	0.236538
SCE1572_165	1.535931435	-16.4825	0.101443	0.236581

SCE1572_1733	1.181088718	-14.2193	0.101555	0.23672
SCE1572_11277	-1.237476155	-15.2842	0.101557	0.23672
SCE1572_85	-1.127033578	-13.566	0.101593	0.23672
SCE1572_6727	-1.127033578	-13.566	0.101593	0.23672
SCE1572_467	-1.089689183	-11.0909	0.10164	0.236777
SCE1572_2635	-1.161798996	-14.3907	0.101724	0.236922
SCE1572_7985	-1.145892605	-14.0014	0.101748	0.236923
SCE1572_11162	-1.145892605	-14.1713	0.101933	0.237258
SCE1572_8250	-1.11388221	-13.1069	0.101937	0.237258
SCE1572_3901	-1.193420975	-14.8996	0.102141	0.237682
SCE1572_5335	-1.128683315	-13.6563	0.10226	0.237905
SCE1572_9556	-1.102122354	-12.6032	0.102315	0.237981
SCE1572_4140	-1.238256623	-15.2506	0.102354	0.238019
SCE1572_5463	1.391541526	-15.777	0.102406	0.23807
SCE1572_2002	-1.166070487	-14.5964	0.102421	0.23807
SCE1572_10946	1.130147945	-13.2832	0.102606	0.238448
SCE1572_7560	1.25909123	-15.2583	0.103108	0.239454
SCE1572_1745	1.25909123	-15.2583	0.103108	0.239454
SCE1572_2506	1.25909123	-15.2583	0.103108	0.239454
SCE1572_2979	1.429016231	-16.3433	0.103178	0.23951
SCE1572_9008	1.429016231	-16.3433	0.103178	0.23951
SCE1572_3136	1.313539014	-15.7006	0.10344	0.240014
SCE1572_4092	1.313539014	-15.7006	0.10344	0.240014
SCE1572_6555	1.140554212	-13.6049	0.103501	0.240102
SCE1572_7064	1.20539021	-14.5482	0.103818	0.240737
SCE1572_10020	1.235536502	-15.1921	0.103821	0.240737
SCE1572_7622	-1.103779427	-12.8886	0.104024	0.241156
SCE1572_9628	1.162690769	-14.267	0.104121	0.241327
SCE1572_9536	1.141772665	-13.8722	0.104182	0.241416
SCE1572_642	-1.101498486	-12.9647	0.104249	0.241519
SCE1572_2697	1.21400334	-14.8069	0.104391	0.241599
SCE1572_8126	1.21400334	-14.8069	0.104391	0.241599
SCE1572_4357	1.21400334	-14.8069	0.104391	0.241599
SCE1572_347	-1.176786613	-14.7635	0.104391	0.241599
SCE1572_8703	-1.271423487	-15.5642	0.104399	0.241599
SCE1572_1321	-1.157252467	-14.227	0.104481	0.241735
SCE1572_9690	-1.117095341	-13.6621	0.104558	0.241808
SCE1572_1924	-1.109672417	-13.145	0.104613	0.241808
SCE1572_1816	1.728576513	-17.1935	0.104627	0.241808
SCE1572_2537	-1.61934679	-17.0601	0.104627	0.241808
SCE1572_9525	-1.493815908	-16.9973	0.104627	0.241808
SCE1572_1808	-1.396954369	-16.1713	0.104716	0.241906
SCE1572_166	-1.396954369	-16.1713	0.104716	0.241906
SCE1572_10163	1.188008132	-14.4638	0.105079	0.242693
SCE1572_11379	-1.212529798	-15.0791	0.105137	0.242774
SCE1572_8778	1.330612527	-15.5851	0.10529	0.242913
SCE1572_1598	1.318853445	-15.591	0.10529	0.242913
SCE1572_2792	1.313539014	-15.5937	0.10529	0.242913

SCE1572_296	-1.231895123	-15.5038	0.10529	0.242913
SCE1572_2632	1.19593356	-14.7281	0.105383	0.243075
SCE1572_9104	1.141652496	-13.9634	0.105506	0.243304
SCE1572_10121	-1.119420394	-13.7471	0.105746	0.243804
SCE1572_4846	-1.18261422	-14.6718	0.10585	0.243992
SCE1572_1439	1.416632507	-16.1795	0.106088	0.244293
SCE1572_1066	1.416632507	-16.1795	0.106088	0.244293
SCE1572_10482	1.416632507	-16.1795	0.106088	0.244293
SCE1572_11521	1.416632507	-16.1795	0.106088	0.244293
SCE1572_5448	1.281117536	-15.5103	0.10612	0.244293
SCE1572_168	1.264629413	-15.5186	0.10612	0.244293
SCE1572_5920	-1.132260739	-14.3201	0.10658	0.245297
SCE1572_9890	1.143614012	-14.0937	0.106741	0.24556
SCE1572_3180	-1.139511812	-14.0729	0.106741	0.24556
SCE1572_9821	1.223341205	-14.9867	0.106878	0.245715
SCE1572_3863	1.2116594	-14.9925	0.106878	0.245715
SCE1572_3593	1.2116594	-14.9925	0.106878	0.245715
SCE1572_7327	1.098641129	-12.7011	0.106971	0.245874
SCE1572_8468	-1.127033578	-14.0514	0.107031	0.245959
SCE1572_5394	1.093148945	-12.6043	0.107091	0.246042
SCE1572_11534	1.360844728	-16.0554	0.107321	0.246355
SCE1572_3847	1.360844728	-16.0554	0.107321	0.246355
SCE1572_9801	1.337385756	-16.0672	0.107321	0.246355
SCE1572_8812	1.337385756	-16.0672	0.107321	0.246355
SCE1572_2373	1.535931435	-16.9679	0.107412	0.246512
SCE1572_9855	1.139509614	-14.0286	0.10759	0.246725
SCE1572_7348	1.283165365	-15.4162	0.107623	0.246725
SCE1572_5377	1.283165365	-15.4162	0.107623	0.246725
SCE1572_10849	1.283165365	-15.4162	0.107623	0.246725
SCE1572_125	1.283165365	-15.4162	0.107623	0.246725
SCE1572_5841	1.22738237	-14.9205	0.107908	0.247217
SCE1572_11222	1.216241812	-14.9261	0.107908	0.247217
SCE1572_1540	-1.140178954	-14.2355	0.107908	0.247217
SCE1572_6815	1.188008132	-14.5569	0.10794	0.247238
SCE1572_3333	-1.084827454	-12.6993	0.108102	0.247555
SCE1572_4540	1.120115993	-13.7698	0.108262	0.247796
SCE1572_5670	1.100909561	-13.0155	0.108301	0.247796
SCE1572_3436	-1.202710737	-15.3016	0.108304	0.247796
SCE1572_2554	1.166697625	-14.5202	0.108315	0.247796
SCE1572_1785	-1.079360865	-12.4275	0.108325	0.247796
SCE1572_8685	1.198896448	-14.8734	0.108388	0.247832
SCE1572_973	-1.171887814	-14.8364	0.108388	0.247832
SCE1572_4631	1.136661252	-14.204	0.108516	0.248069
SCE1572_2924	1.11976727	-13.5672	0.108552	0.248069
SCE1572_7340	-1.092575407	-12.982	0.108563	0.248069
SCE1572_7037	-1.15594627	-14.4658	0.108677	0.248222
SCE1572_2745	-1.15594627	-14.4658	0.108677	0.248222
SCE1572_10386	1.104952392	-13.2199	0.108941	0.248772

SCE1572_8358	1.183569319	-14.4678	0.109026	0.248912
SCE1572_1915	1.095828598	-12.9553	0.109068	0.248951
SCE1572_3077	1.148115404	-14.1618	0.109091	0.248951
SCE1572_9698	1.356607736	-15.92	0.109441	0.249643
SCE1572_11239	1.356607736	-15.92	0.109441	0.249643
SCE1572_1057	1.245152039	-15.2653	0.109548	0.249821
SCE1572_5985	1.654575932	-16.9086	0.109695	0.249821
SCE1572_6432	1.654575932	-16.9086	0.109695	0.249821
SCE1572_767	1.654575932	-16.9086	0.109695	0.249821
SCE1572_9098	1.654575932	-16.9086	0.109695	0.249821
SCE1572_8722	1.654575932	-16.9086	0.109695	0.249821
SCE1572_10164	1.654575932	-16.9086	0.109695	0.249821
SCE1572_3731	1.177883914	-14.7684	0.109709	0.249821
SCE1572_2003	1.13715899	-14.0969	0.109896	0.250147
SCE1572_5734	-1.110361054	-13.6243	0.1099	0.250147
SCE1572_9857	-1.084337934	-12.9926	0.109974	0.250232
SCE1572_2258	1.164675628	-14.388	0.110009	0.250232
SCE1572_220	-1.128819092	-14.3554	0.110009	0.250232
SCE1572_1072	1.222115986	-15.1988	0.110117	0.250369
SCE1572_334	-1.178314083	-15.1551	0.110117	0.250369
SCE1572_7626	1.293361132	-15.8261	0.11036	0.250704
SCE1572_9179	1.293361132	-15.8261	0.11036	0.250704
SCE1572_6907	1.272897029	-15.8364	0.11036	0.250704
SCE1572_6993	1.272897029	-15.8364	0.11036	0.250704
SCE1572_3453	1.192857531	-14.7065	0.110502	0.250867
SCE1572_10590	1.192857531	-14.7065	0.110502	0.250867
SCE1572_1252	1.183142376	-14.7113	0.110502	0.250867
SCE1572_11286	-1.087626311	-13.078	0.110582	0.250992
SCE1572_3555	1.151108549	-14.3123	0.110901	0.251663
SCE1572_9685	-1.108365205	-13.7416	0.111036	0.251914
SCE1572_3839	1.22682738	-15.1224	0.111163	0.25208
SCE1572_1464	1.21400334	-15.1288	0.111163	0.25208
SCE1572_10594	1.141174967	-14.2777	0.111181	0.25208
SCE1572_4839	-1.097394087	-13.3211	0.111358	0.252427
SCE1572_2553	1.165216267	-14.6172	0.11158	0.252632
SCE1572_1687	1.501166017	-16.7222	0.111592	0.252632
SCE1572_8047	1.501166017	-16.7222	0.111592	0.252632
SCE1572_6483	1.501166017	-16.7222	0.111592	0.252632
SCE1572_6696	1.501166017	-16.7222	0.111592	0.252632
SCE1572_7033	1.501166017	-16.7222	0.111592	0.252632
SCE1572_2081	1.128183972	-13.9391	0.111764	0.252967
SCE1572_1010	1.143614012	-14.5791	0.111913	0.253248
SCE1572_7474	-1.115304285	-13.9012	0.111973	0.25333
SCE1572_10402	-1.062160338	-11.9094	0.112014	0.253368
SCE1572_6694	1.21890214	-15.056	0.112103	0.253516
SCE1572_9587	-1.088832074	-13.6249	0.112299	0.253905
SCE1572_1170	1.199882232	-14.9984	0.112538	0.25439
SCE1572_5232	-1.229603311	-15.5851	0.112679	0.254653

SCE1572_6448	1.089643002	-13.191	0.112926	0.255149
SCE1572_3250	-1.095393123	-13.5721	0.112947	0.255149
SCE1572_4983	-1.057066594	-11.2576	0.113103	0.255414
SCE1572_9387	-1.067409595	-12.2877	0.113113	0.255414
SCE1572_10467	-1.077101831	-12.8481	0.113187	0.255426
SCE1572_9825	-1.360400969	-16.4855	0.113191	0.255426
SCE1572_1823	-1.349425999	-16.4846	0.113191	0.255426
SCE1572_10853	1.151787944	-14.4375	0.113407	0.255858
SCE1572_9997	-1.062609472	-12.0734	0.113513	0.256044
SCE1572_2968	-1.145892605	-14.8494	0.113722	0.25646
SCE1572_7039	1.247950672	-15.5269	0.113941	0.256899
SCE1572_3359	1.125466666	-14.0032	0.114178	0.257378
SCE1572_6046	-1.059308545	-12.2565	0.114398	0.257669
SCE1572_10385	1.506184092	-16.4973	0.114552	0.257669
SCE1572_3437	1.506184092	-16.4973	0.114552	0.257669
SCE1572_8863	1.506184092	-16.4973	0.114552	0.257669
SCE1572_10601	1.506184092	-16.4973	0.114552	0.257669
SCE1572_2348	1.475810443	-16.5125	0.114552	0.257669
SCE1572_10429	1.475810443	-16.5125	0.114552	0.257669
SCE1572_3576	1.475810443	-16.5125	0.114552	0.257669
SCE1572_3111	1.475810443	-16.5125	0.114552	0.257669
SCE1572_6440	1.475810443	-16.5125	0.114552	0.257669
SCE1572_8641	1.157419812	-14.7786	0.114752	0.258008
SCE1572_9316	-1.131698723	-14.741	0.114752	0.258008
SCE1572_3470	1.267735324	-15.4239	0.11503	0.258214
SCE1572_11244	-1.199273701	-15.372	0.11503	0.258214
SCE1572_10989	-1.199273701	-15.372	0.11503	0.258214
SCE1572_7930	1.108028309	-13.6211	0.11504	0.258214
SCE1572_8386	1.09778683	-13.6158	0.11504	0.258214
SCE1572_8578	1.09778683	-13.6158	0.11504	0.258214
SCE1572_1453	1.09778683	-13.6158	0.11504	0.258214
SCE1572_8336	1.09778683	-13.6158	0.11504	0.258214
SCE1572_3744	-1.138156956	-14.6727	0.115368	0.258604
SCE1572_3624	2.120893936	-17.9973	0.11546	0.258604
SCE1572_2927	2.120893936	-17.9973	0.11546	0.258604
SCE1572_1325	2.120893936	-17.9973	0.11546	0.258604
SCE1572_4638	2.120893936	-17.9973	0.11546	0.258604
SCE1572_2851	2.120893936	-17.9973	0.11546	0.258604
SCE1572_10671	2.120893936	-17.9973	0.11546	0.258604
SCE1572_176	2.120893936	-17.9973	0.11546	0.258604
SCE1572_9832	2.120893936	-17.9973	0.11546	0.258604
SCE1572_6887	2.120893936	-17.9973	0.11546	0.258604
SCE1572_3570	1.226076172	-15.3573	0.11552	0.258684
SCE1572_7330	1.150040281	-14.6754	0.115657	0.258809
SCE1572_4506	1.401001855	-16.3573	0.115723	0.258809
SCE1572_4386	1.401001855	-16.3573	0.115723	0.258809
SCE1572_2856	1.401001855	-16.3573	0.115723	0.258809
SCE1572_345	1.372432703	-16.3716	0.115723	0.258809

SCE1572_6304	-1.338537683	-16.2676	0.115723	0.258809
SCE1572_2455	-1.101498486	-13.8012	0.115892	0.259131
SCE1572_11146	-1.084053351	-13.5018	0.115962	0.259232
SCE1572_1442	1.216862994	-15.2794	0.116408	0.260063
SCE1572_10444	-1.068930623	-12.8175	0.116417	0.260063
SCE1572_3999	-1.045773681	-11.2769	0.116427	0.260063
SCE1572_9774	-1.081320604	-13.4487	0.116438	0.260063
SCE1572_5542	1.134568873	-14.5836	0.116457	0.260063
SCE1572_9758	-1.064098514	-12.8085	0.116491	0.260083
SCE1572_230	1.194894517	-15.2124	0.116812	0.260689
SCE1572_11555	1.194894517	-15.2124	0.116812	0.260689
SCE1572_2495	1.149419634	-14.5289	0.116941	0.260923
SCE1572_5525	1.085511058	-13.6821	0.117011	0.261022
SCE1572_10810	-1.135768388	-14.4759	0.11717	0.261323
SCE1572_10331	1.113240363	-14.0093	0.117338	0.261403
SCE1572_5837	-1.065893283	-12.8834	0.11739	0.261403
SCE1572_3406	1.201064284	-15.1353	0.11755	0.261403
SCE1572_3069	-1.153586997	-15.08	0.11755	0.261403
SCE1572_6585	-1.045502254	-11.5444	0.117594	0.261403
SCE1572_4295	1.391541526	-16.1921	0.117629	0.261403
SCE1572_11340	1.391541526	-16.1921	0.117629	0.261403
SCE1572_8665	1.391541526	-16.1921	0.117629	0.261403
SCE1572_2445	1.391541526	-16.1921	0.117629	0.261403
SCE1572_11236	1.391541526	-16.1921	0.117629	0.261403
SCE1572_4304	1.391541526	-16.1921	0.117629	0.261403
SCE1572_6759	1.391541526	-16.1921	0.117629	0.261403
SCE1572_1090	1.391541526	-16.1921	0.117629	0.261403
SCE1572_6975	1.391541526	-16.1921	0.117629	0.261403
SCE1572_4914	1.366006434	-16.2048	0.117629	0.261403
SCE1572_3864	1.366006434	-16.2048	0.117629	0.261403
SCE1572_8107	1.366006434	-16.2048	0.117629	0.261403
SCE1572_10038	-1.076511802	-13.2831	0.117778	0.261624
SCE1572_1882	1.07340501	-13.2923	0.117778	0.261624
SCE1572_4349	1.064179545	-12.8114	0.118009	0.262026
SCE1572_5013	-1.109960064	-14.3648	0.118009	0.262026
SCE1572_11311	1.102551356	-13.8918	0.11821	0.262416
SCE1572_1374	-1.076167196	-13.4873	0.118382	0.262644
SCE1572_6593	-1.328007015	-16.0213	0.118412	0.262644
SCE1572_9980	-1.328007015	-16.0213	0.118412	0.262644
SCE1572_1478	1.313539014	-16.0791	0.118412	0.262644
SCE1572_10950	1.17603549	-15.0103	0.11851	0.262806
SCE1572_4522	1.193699244	-14.9374	0.11907	0.263851
SCE1572_2816	1.193699244	-14.9374	0.11907	0.263851
SCE1572_2016	1.11727362	-14.2512	0.119073	0.263851
SCE1572_139	-1.271423487	-15.9386	0.119107	0.263851
SCE1572_7424	-1.271423487	-15.9386	0.119107	0.263851
SCE1572_5794	-1.063910242	-13.0899	0.119166	0.26387
SCE1572_5833	-1.063910242	-13.0899	0.119166	0.26387

SCE1572_8488	1.083109273	-13.3869	0.119229	0.263955
SCE1572_4149	1.165981825	-14.8898	0.11933	0.264122
SCE1572_10022	1.74503776	-17.5764	0.119582	0.264237
SCE1572_8513	1.74503776	-17.5764	0.119582	0.264237
SCE1572_9725	-1.730855106	-17.4638	0.119582	0.264237
SCE1572_459	1.728576513	-17.6085	0.119582	0.264237
SCE1572_7415	1.728576513	-17.6085	0.119582	0.264237
SCE1572_3881	1.728576513	-17.6085	0.119582	0.264237
SCE1572_10684	1.728576513	-17.6085	0.119582	0.264237
SCE1572_6868	1.728576513	-17.6085	0.119582	0.264237
SCE1572_11307	-1.103300728	-14.1337	0.11968	0.264397
SCE1572_2224	1.313539014	-15.9416	0.119726	0.264443
SCE1572_1076	-1.090851241	-14.1113	0.119821	0.264597
SCE1572_4783	1.059989646	-12.7377	0.119963	0.264855
SCE1572_3464	1.0656115	-12.9551	0.120144	0.265199
SCE1572_9553	1.252138469	-15.8467	0.120281	0.265391
SCE1572_11533	1.252138469	-15.8467	0.120281	0.265391
SCE1572_1398	1.072254191	-13.2179	0.120317	0.265415
SCE1572_6741	1.052640352	-12.4546	0.12043	0.265553
SCE1572_1003	1.153598101	-14.7261	0.120456	0.265553
SCE1572_4456	-1.114697983	-14.6844	0.120456	0.265553
SCE1572_2701	1.100909561	-14.0155	0.120588	0.265789
SCE1572_9475	-1.080601145	-13.9687	0.120704	0.26599
SCE1572_9181	1.125466666	-14.5882	0.121194	0.266988
SCE1572_1298	1.256955485	-15.7289	0.121234	0.266988
SCE1572_5940	1.256955485	-15.7289	0.121234	0.266988
SCE1572_4311	1.131932207	-14.5376	0.121519	0.267561
SCE1572_933	-1.043154499	-12.6427	0.121838	0.268206
SCE1572_4459	1.278773596	-15.611	0.12202	0.268495
SCE1572_4556	1.278773596	-15.611	0.12202	0.268495
SCE1572_5886	1.081092928	-13.7099	0.122225	0.268835
SCE1572_4381	1.132966768	-14.4038	0.122233	0.268835
SCE1572_10959	1.231076853	-15.5353	0.122363	0.268835
SCE1572_1159	1.231076853	-15.5353	0.122363	0.268835
SCE1572_10850	1.231076853	-15.5353	0.122363	0.268835
SCE1572_8694	1.21400334	-15.5439	0.122363	0.268835
SCE1572_6764	-1.072877808	-13.6635	0.122379	0.268835
SCE1572_8772	-1.062609472	-13.6583	0.122379	0.268835
SCE1572_5571	1.12166912	-14.3496	0.122484	0.268968
SCE1572_8477	1.06102248	-13.1697	0.12249	0.268968
SCE1572_992	-1.0349036	-11.8576	0.122602	0.269157
SCE1572_8454	-1.195474634	-15.4461	0.122678	0.26919
SCE1572_7451	-1.158948758	-15.4278	0.122678	0.26919
SCE1572_8297	-1.027308773	-11.1663	0.122694	0.26919
SCE1572_10055	-1.029039591	-11.2391	0.122876	0.269533
SCE1572_8565	-1.025413177	-11.014	0.122918	0.269569
SCE1572_10301	1.236371153	-15.4396	0.122969	0.269624
SCE1572_2033	-1.040856694	-12.6828	0.12302	0.26968

SCE1572_11384	-1.026974145	-11.3139	0.123077	0.269749
SCE1572_9523	-1.085010363	-14.1573	0.123243	0.270056
SCE1572_2981	-1.137122395	-15.2688	0.123485	0.270531
SCE1572_88	-1.069115312	-14.0532	0.123692	0.270923
SCE1572_5464	1.202507701	-15.2866	0.123716	0.270923
SCE1572_10403	-1.053077899	-13.3269	0.123823	0.271101
SCE1572_8310	1.167149527	-15.2263	0.123931	0.271281
SCE1572_7268	-1.040679186	-12.4513	0.12397	0.271309
SCE1572_9834	1.188008132	-15.1418	0.124318	0.271738
SCE1572_7719	1.174832743	-15.1484	0.124318	0.271738
SCE1572_6139	-1.078778409	-13.8829	0.124358	0.271738
SCE1572_835	1.682772823	-17.2164	0.124578	0.271738
SCE1572_7513	1.682772823	-17.2164	0.124578	0.271738
SCE1572_6227	1.682772823	-17.2164	0.124578	0.271738
SCE1572_8279	1.682772823	-17.2164	0.124578	0.271738
SCE1572_7018	1.682772823	-17.2164	0.124578	0.271738
SCE1572_3290	1.635467109	-17.24	0.124578	0.271738
SCE1572_3489	1.635467109	-17.24	0.124578	0.271738
SCE1572_2247	1.635467109	-17.24	0.124578	0.271738
SCE1572_9607	1.635467109	-17.24	0.124578	0.271738
SCE1572_8805	1.635467109	-17.24	0.124578	0.271738
SCE1572_8160	1.635467109	-17.24	0.124578	0.271738
SCE1572_7058	1.635467109	-17.24	0.124578	0.271738
SCE1572_5697	-1.030768859	-12.2785	0.124579	0.271738
SCE1572_5214	1.163962658	-15.0164	0.124812	0.272126
SCE1572_367	-1.133919963	-14.9809	0.124812	0.272126
SCE1572_7563	1.076976819	-13.7644	0.124834	0.272126
SCE1572_8373	-1.036687076	-12.5208	0.124964	0.272351
SCE1572_5393	1.075166662	-13.6622	0.125138	0.272674
SCE1572_2954	1.143614012	-14.901	0.125225	0.272807
SCE1572_6549	-1.122045863	-14.8374	0.125347	0.273018
SCE1572_6460	1.150809514	-14.8385	0.125463	0.273214
SCE1572_7300	1.126168878	-14.7943	0.125574	0.273341
SCE1572_2029	1.126168878	-14.7943	0.125574	0.273341
SCE1572_9300	-1.046131175	-13.4311	0.125749	0.273647
SCE1572_10358	1.133560348	-14.7361	0.125777	0.273647
SCE1572_11086	-1.050453141	-13.4031	0.125818	0.273647
SCE1572_7126	1.120893936	-14.69	0.125872	0.273647
SCE1572_3868	1.058281958	-13.3192	0.12607	0.273647
SCE1572_3100	1.494111259	-16.9888	0.12613	0.273647
SCE1572_1658	1.494111259	-16.9888	0.12613	0.273647
SCE1572_9826	1.494111259	-16.9888	0.12613	0.273647
SCE1572_10001	1.494111259	-16.9888	0.12613	0.273647
SCE1572_7271	1.494111259	-16.9888	0.12613	0.273647
SCE1572_2836	-1.477874365	-16.8898	0.12613	0.273647
SCE1572_3993	-1.477874365	-16.8898	0.12613	0.273647
SCE1572_1559	1.451042537	-17.0103	0.12613	0.273647
SCE1572_10171	1.451042537	-17.0103	0.12613	0.273647

SCE1572_1158	1.451042537	-17.0103	0.12613	0.273647
SCE1572_7708	1.451042537	-17.0103	0.12613	0.273647
SCE1572_2557	1.061702814	-13.2601	0.126211	0.273766
SCE1572_4580	1.129455949	-14.493	0.126424	0.274032
SCE1572_3955	1.129455949	-14.493	0.126424	0.274032
SCE1572_6201	1.120893936	-14.4973	0.126424	0.274032
SCE1572_2819	1.110447148	-14.4582	0.12649	0.274032
SCE1572_6298	-1.101498486	-14.4386	0.12649	0.274032
SCE1572_9868	-1.101498486	-14.4386	0.12649	0.274032
SCE1572_5480	1.047258948	-13.0584	0.126636	0.274292
SCE1572_4555	1.105545971	-14.3351	0.126784	0.274554
SCE1572_1367	-1.087711534	-14.2618	0.126886	0.274719
SCE1572_11215	1.098944963	-14.1508	0.127153	0.274995
SCE1572_9977	1.090214318	-14.0864	0.127268	0.274995
SCE1572_11406	1.616101784	-16.9278	0.127301	0.274995
SCE1572_6509	1.616101784	-16.9278	0.127301	0.274995
SCE1572_2707	1.616101784	-16.9278	0.127301	0.274995
SCE1572_6106	1.616101784	-16.9278	0.127301	0.274995
SCE1572_3365	1.57657342	-16.9476	0.127301	0.274995
SCE1572_3056	1.57657342	-16.9476	0.127301	0.274995
SCE1572_9824	1.544230758	-16.9071	0.127301	0.274995
SCE1572_10388	-1.464068565	-16.7898	0.127301	0.274995
SCE1572_11258	-1.464068565	-16.7898	0.127301	0.274995
SCE1572_5735	-1.073094771	-14.0245	0.127339	0.27502
SCE1572_5420	-1.078778409	-13.9825	0.127405	0.275107
SCE1572_1597	1.072959459	-13.8489	0.127636	0.275549
SCE1572_4277	1.071721094	-13.767	0.127757	0.275752
SCE1572_191	1.054804745	-13.4643	0.128119	0.275752
SCE1572_3670	1.476321946	-16.7347	0.128197	0.275752
SCE1572_4139	1.465542107	-16.74	0.128197	0.275752
SCE1572_2823	1.465542107	-16.74	0.128197	0.275752
SCE1572_1446	1.465542107	-16.74	0.128197	0.275752
SCE1572_257	1.465542107	-16.74	0.128197	0.275752
SCE1572_4887	1.465542107	-16.74	0.128197	0.275752
SCE1572_2936	1.465542107	-16.74	0.128197	0.275752
SCE1572_3884	1.465542107	-16.74	0.128197	0.275752
SCE1572_8914	1.465542107	-16.74	0.128197	0.275752
SCE1572_9878	1.429016231	-16.7583	0.128197	0.275752
SCE1572_7185	1.429016231	-16.7583	0.128197	0.275752
SCE1572_1239	1.429016231	-16.7583	0.128197	0.275752
SCE1572_6288	1.429016231	-16.7583	0.128197	0.275752
SCE1572_7280	1.429016231	-16.7583	0.128197	0.275752
SCE1572_3127	-1.358886328	-16.6498	0.128197	0.275752
SCE1572_3945	-1.00685125	-9.81385	0.128202	0.275752
SCE1572_7612	-1.022064018	-12.519	0.128826	0.276999
SCE1572_6536	-1.023221879	-12.4556	0.128857	0.276999
SCE1572_1725	-1.019677153	-12.4487	0.128861	0.276999
SCE1572_6429	1.444783547	-16.528	0.129437	0.27784

SCE1572_102	1.444783547	-16.528	0.129437	0.27784
SCE1572_9172	1.444783547	-16.528	0.129437	0.27784
SCE1572_1101	1.444783547	-16.528	0.129437	0.27784
SCE1572_7495	1.444783547	-16.528	0.129437	0.27784
SCE1572_5588	1.444783547	-16.528	0.129437	0.27784
SCE1572_3161	-1.271423487	-16.3716	0.129437	0.27784
SCE1572_11316	-1.005068723	-10.7988	0.129728	0.278408
SCE1572_927	1.343286357	-16.3861	0.129867	0.278463
SCE1572_10903	1.343286357	-16.3861	0.129867	0.278463
SCE1572_2286	-1.271423487	-16.3012	0.129867	0.278463
SCE1572_2567	-1.018916793	-12.3481	0.130071	0.278463
SCE1572_3645	-1.024783519	-12.6875	0.130197	0.278463
SCE1572_4008	1.340011225	-16.2178	0.130482	0.278463
SCE1572_8913	1.340011225	-16.2178	0.130482	0.278463
SCE1572_962	1.011510476	-11.5577	0.130533	0.278463
SCE1572_11392	-1.036958233	-13.4888	0.130595	0.278463
SCE1572_4132	-1.039693201	-13.5332	0.13062	0.278463
SCE1572_4925	-1.044012991	-13.6373	0.130682	0.278463
SCE1572_1201	1.289291467	-16.0912	0.130701	0.278463
SCE1572_1379	1.289291467	-16.0912	0.130701	0.278463
SCE1572_6652	1.289291467	-16.0912	0.130701	0.278463
SCE1572_5117	-1.271423487	-16.0496	0.130701	0.278463
SCE1572_1809	-1.271423487	-16.0496	0.130701	0.278463
SCE1572_10920	-1.271423487	-16.0496	0.130701	0.278463
SCE1572_11095	1.264629413	-16.1035	0.130701	0.278463
SCE1572_4944	-1.214839959	-16.0213	0.130701	0.278463
SCE1572_1546	1.060772943	-13.72	0.13072	0.278463
SCE1572_742	-1.056829436	-13.8583	0.130815	0.278463
SCE1572_8441	-1.05927025	-13.9292	0.130862	0.278463
SCE1572_554	-1.216975703	-15.9658	0.130877	0.278463
SCE1572_6011	-1.063981407	-13.9899	0.130899	0.278463
SCE1572_16	-1.051157367	-13.9834	0.130899	0.278463
SCE1572_61	-1.058429764	-14.1276	0.130992	0.278463
SCE1572_5680	-1.005553515	-11.3487	0.130993	0.278463
SCE1572_6490	1.291512707	-15.9526	0.131019	0.278463
SCE1572_3450	1.291512707	-15.9526	0.131019	0.278463
SCE1572_1578	1.291512707	-15.9526	0.131019	0.278463
SCE1572_4384	1.291512707	-15.9526	0.131019	0.278463
SCE1572_8712	1.291512707	-15.9526	0.131019	0.278463
SCE1572_5157	1.269144894	-15.9638	0.131019	0.278463
SCE1572_6484	1.269144894	-15.9638	0.131019	0.278463
SCE1572_10455	1.231076853	-15.8573	0.131133	0.278463
SCE1572_8114	1.231076853	-15.8573	0.131133	0.278463
SCE1572_6941	1.231076853	-15.8573	0.131133	0.278463
SCE1572_5216	-1.222513887	-15.8111	0.131133	0.278463
SCE1572_11405	1.209703203	-15.868	0.131133	0.278463
SCE1572_11341	1.209703203	-15.868	0.131133	0.278463
SCE1572_5553	1.209703203	-15.868	0.131133	0.278463

SCE1572_5540	-1.175208172	-15.7874	0.131133	0.278463
SCE1572_6738	-1.178314083	-15.74	0.131224	0.278463
SCE1572_865	-1.178314083	-15.74	0.131224	0.278463
SCE1572_4653	1.096308297	-14.5554	0.131242	0.278463
SCE1572_6005	-1.086998916	-14.5569	0.131259	0.278463
SCE1572_3326	-1.067890093	-14.5473	0.131259	0.278463
SCE1572_1251	1.118523031	-14.6405	0.131292	0.278463
SCE1572_3352	1.23759016	-15.7385	0.131297	0.278463
SCE1572_10524	1.23759016	-15.7385	0.131297	0.278463
SCE1572_527	-1.186534589	-15.6067	0.131354	0.278463
SCE1572_8663	1.243149686	-15.6288	0.131399	0.278463
SCE1572_1574	1.243149686	-15.6288	0.131399	0.278463
SCE1572_8490	-1.188961327	-15.5648	0.131399	0.278463
SCE1572_1169	1.132298699	-14.9067	0.131422	0.278463
SCE1572_11261	1.196725349	-15.5525	0.131434	0.278463
SCE1572_329	-1.110958815	-14.9408	0.13145	0.278463
SCE1572_9196	1.151787944	-15.0225	0.131462	0.278463
SCE1572_10557	1.220429609	-15.4476	0.131478	0.278463
SCE1572_5490	1.204309944	-15.4556	0.131478	0.278463
SCE1572_5632	-1.161798996	-15.3907	0.131478	0.278463
SCE1572_7996	1.138969309	-15.096	0.131483	0.278463
SCE1572_739	1.180272483	-15.3802	0.13149	0.278463
SCE1572_10767	1.164675628	-15.388	0.13149	0.278463
SCE1572_1429	-1.130560951	-15.3377	0.13149	0.278463
SCE1572_7144	-1.112725741	-15.1879	0.131501	0.278463
SCE1572_6800	-1.001751629	-10.9907	0.1316	0.278616
SCE1572_1368	-1.027866456	-13.1288	0.132401	0.280256
SCE1572_9295	-1.034662775	-13.2805	0.132689	0.28081
SCE1572_1548	1.038261012	-13.3889	0.132898	0.281195
SCE1572_11513	-1.033731929	-13.5086	0.1332	0.281778
SCE1572_8127	1.050504608	-13.6018	0.133373	0.282087
SCE1572_10969	-1.022064018	-12.8409	0.133562	0.28243
SCE1572_877	-1.022303828	-12.9915	0.133985	0.283267
SCE1572_10775	1.056381174	-13.9149	0.134208	0.283625
SCE1572_7256	-1.039060777	-13.8908	0.134208	0.283625
SCE1572_11558	1.059187686	-13.9321	0.134262	0.283682
SCE1572_9796	1.050504608	-14.009	0.134492	0.28411
SCE1572_7096	1.082213468	-14.3467	0.135592	0.286376
SCE1572_4231	1.041599055	-13.5146	0.135793	0.286743
SCE1572_5746	-1.068930623	-14.4024	0.135872	0.286796
SCE1572_1053	-1.05185711	-14.3939	0.135872	0.286796
SCE1572_6012	-1.05510558	-14.4979	0.136289	0.287617
SCE1572_10264	1.087263158	-14.5599	0.136401	0.287797
SCE1572_4195	-1.061970121	-14.5888	0.136638	0.288239
SCE1572_674	-1.017106298	-13.1913	0.136768	0.288456
SCE1572_9298	-1.038224311	-13.744	0.136876	0.288626
SCE1572_2428	-1.030415388	-13.7916	0.137123	0.288646
SCE1572_8073	1.113240363	-14.7463	0.137164	0.288646

SCE1572_538	-1.087977346	-14.7191	0.137164	0.288646
SCE1572_770	-1.087977346	-14.7191	0.137164	0.288646
SCE1572_11454	-1.001479136	-12.6337	0.137421	0.288646
SCE1572_46	-1.019505113	-13.3196	0.13743	0.288646
SCE1572_10142	-1.043154499	-13.8651	0.137478	0.288646
SCE1572_5561	1.013029902	-12.6809	0.137635	0.288646
SCE1572_4702	1.021772889	-13.3971	0.137773	0.288646
SCE1572_11487	2.871534467	-19.037	0.137874	0.288646
SCE1572_3595	2.871534467	-19.037	0.137874	0.288646
SCE1572_2603	2.871534467	-19.037	0.137874	0.288646
SCE1572_4178	2.871534467	-19.037	0.137874	0.288646
SCE1572_2997	2.535931435	-18.7898	0.137874	0.288646
SCE1572_256	2.535931435	-18.7898	0.137874	0.288646
SCE1572_2130	2.535931435	-18.7898	0.137874	0.288646
SCE1572_1206	2.535931435	-18.7898	0.137874	0.288646
SCE1572_3059	2.535931435	-18.7898	0.137874	0.288646
SCE1572_8142	2.535931435	-18.7898	0.137874	0.288646
SCE1572_8651	2.535931435	-18.7898	0.137874	0.288646
SCE1572_8501	2.535931435	-18.7898	0.137874	0.288646
SCE1572_3393	2.535931435	-18.7898	0.137874	0.288646
SCE1572_8215	2.535931435	-18.7898	0.137874	0.288646
SCE1572_5856	2.535931435	-18.7898	0.137874	0.288646
SCE1572_7131	2.535931435	-18.7898	0.137874	0.288646
SCE1572_10258	2.535931435	-18.7898	0.137874	0.288646
SCE1572_10450	2.535931435	-18.7898	0.137874	0.288646
SCE1572_2976	2.519348551	-18.7981	0.137874	0.288646
SCE1572_9848	2.429016231	-18.8433	0.137874	0.288646
SCE1572_1870	2.429016231	-18.8433	0.137874	0.288646
SCE1572_2978	2.429016231	-18.8433	0.137874	0.288646
SCE1572_6790	2.429016231	-18.8433	0.137874	0.288646
SCE1572_369	2.429016231	-18.8433	0.137874	0.288646
SCE1572_2830	2.429016231	-18.8433	0.137874	0.288646
SCE1572_3908	2.429016231	-18.8433	0.137874	0.288646
SCE1572_8181	2.429016231	-18.8433	0.137874	0.288646
SCE1572_7105	1.109398297	-14.9181	0.137938	0.288665
SCE1572_3405	1.109398297	-14.9181	0.137938	0.288665
SCE1572_9950	1.135751894	-14.9663	0.138111	0.28897
SCE1572_4929	-1.082389663	-14.9551	0.138292	0.28929
SCE1572_6473	-0.997061075	-12.3469	0.138433	0.289396
SCE1572_2107	-1.024629722	-13.504	0.138471	0.289396
SCE1572_2115	1.127125889	-15.0348	0.13848	0.289396
SCE1572_10271	1.127125889	-15.0348	0.13848	0.289396
SCE1572_2577	1.114634945	-15.041	0.13848	0.289396
SCE1572_8728	1.117618804	-15.1067	0.13888	0.290118
SCE1572_2911	1.104443415	-15.1132	0.13888	0.290118
SCE1572_10516	0.993745309	-11.9389	0.138963	0.290232
SCE1572_9518	-1.041941641	-14.1358	0.139034	0.290276
SCE1572_6642	1.148115404	-15.1618	0.139094	0.290276

SCE1572_9621	1.134568873	-15.1686	0.139094	0.290276
SCE1572_587	1.134568873	-15.1686	0.139094	0.290276
SCE1572_1304	-0.999248427	-12.6748	0.139176	0.29039
SCE1572_10738	1.040690318	-13.6548	0.139298	0.290586
SCE1572_8394	-1.003171372	-12.7139	0.139447	0.29084
SCE1572_5247	1.124505189	-15.2476	0.13955	0.290997
SCE1572_10505	1.158564354	-15.3086	0.139794	0.291448
SCE1572_3940	-0.986391979	-11.5881	0.139841	0.291489
SCE1572_8985	-1.101498486	-15.2866	0.140049	0.291781
SCE1572_10897	-1.101498486	-15.2866	0.140049	0.291781
SCE1572_9095	-1.049031066	-14.2969	0.140065	0.291781
SCE1572_2888	1.148908312	-15.3959	0.140317	0.292075
SCE1572_4632	1.148908312	-15.3959	0.140317	0.292075
SCE1572_9563	1.148908312	-15.3959	0.140317	0.292075
SCE1572_2012	1.148908312	-15.3959	0.140317	0.292075
SCE1572_874	1.03936405	-13.8377	0.140543	0.292487
SCE1572_8549	1.188008132	-15.4638	0.140598	0.292543
SCE1572_5011	-1.15594627	-15.4658	0.140892	0.29304
SCE1572_7836	-1.119420394	-15.4476	0.140892	0.29304
SCE1572_10090	-1.00660607	-13.188	0.141083	0.29338
SCE1572_5331	-1.060856501	-14.4589	0.141144	0.293448
SCE1572_871	-0.990137377	-12.3829	0.141181	0.293451
SCE1572_5414	1.179237922	-15.5613	0.141201	0.293451
SCE1572_5497	-1.012036858	-13.5194	0.141316	0.293632
SCE1572_4009	1.086128518	-14.5147	0.141346	0.293635
SCE1572_10075	-0.974097971	-10.3513	0.141447	0.293787
SCE1572_2160	1.225002339	-15.6379	0.141526	0.293836
SCE1572_4371	1.20662381	-15.6471	0.141526	0.293836
SCE1572_7649	1.078160951	-14.5645	0.141771	0.294286
SCE1572_6789	1.037904571	-14.0153	0.141986	0.294635
SCE1572_2119	-1.045863787	-14.5583	0.141995	0.294635
SCE1572_909	1.021554534	-13.6401	0.14217	0.294939
SCE1572_1537	-1.181225678	-15.6942	0.142227	0.294943
SCE1572_1771	1.069613431	-14.6161	0.142228	0.294943
SCE1572_1137	1.086274114	-14.6567	0.142469	0.295385
SCE1572_8716	-1.035356129	-14.0836	0.142675	0.295754
SCE1572_6026	-1.069789626	-14.6858	0.14298	0.29614
SCE1572_9658	-1.069789626	-14.6858	0.14298	0.29614
SCE1572_8820	1.200630978	-15.8725	0.143002	0.29614
SCE1572_9650	1.188008132	-15.8788	0.143002	0.29614
SCE1572_580	-1.171887814	-15.8364	0.143002	0.29614
SCE1572_7032	1.053738248	-14.1734	0.143244	0.296537
SCE1572_9565	1.092630084	-14.7566	0.143251	0.296537
SCE1572_4748	1.024032396	-13.7644	0.14331	0.296537
SCE1572_3037	1.246424818	-15.9751	0.143419	0.296537
SCE1572_7159	1.246424818	-15.9751	0.143419	0.296537
SCE1572_3300	1.246424818	-15.9751	0.143419	0.296537
SCE1572_2889	1.246424818	-15.9751	0.143419	0.296537

SCE1572_10812	1.246424818	-15.9751	0.143419	0.296537
SCE1572_11439	1.028971446	-13.7884	0.143467	0.296578
SCE1572_11155	-1.019360199	-13.7861	0.143628	0.296852
SCE1572_2543	1.107088136	-14.8604	0.144133	0.297721
SCE1572_5466	1.107088136	-14.8604	0.144133	0.297721
SCE1572_9169	1.079330193	-14.8531	0.144133	0.297721
SCE1572_8699	1.050504608	-14.2846	0.14431	0.297978
SCE1572_1118	0.997676919	-13.0832	0.144314	0.297978
SCE1572_4367	1.008126006	-13.3058	0.144365	0.298024
SCE1572_2652	0.984186711	-12.4572	0.14446	0.298161
SCE1572_3155	-1.013111491	-13.8919	0.144677	0.298505
SCE1572_11046	1.042685103	-14.327	0.144784	0.298505
SCE1572_6470	1.313539014	-16.2311	0.144797	0.298505
SCE1572_1865	1.313539014	-16.2311	0.144797	0.298505
SCE1572_253	1.313539014	-16.2311	0.144797	0.298505
SCE1572_1910	1.286571966	-16.2446	0.144797	0.298505
SCE1572_4353	-0.979741679	-12.6348	0.145002	0.298853
SCE1572_10201	-1.085867834	-14.9283	0.145136	0.298853
SCE1572_6885	-1.085867834	-14.9283	0.145136	0.298853
SCE1572_4000	-1.085867834	-14.9283	0.145136	0.298853
SCE1572_7116	-1.06120578	-14.916	0.145136	0.298853
SCE1572_8604	-1.06120578	-14.916	0.145136	0.298853
SCE1572_7920	1.016442772	-13.6427	0.145207	0.29894
SCE1572_7021	1.345571363	-16.3423	0.145296	0.299065
SCE1572_2732	-0.989791862	-13.169	0.145348	0.299113
SCE1572_7104	1.050504608	-14.4032	0.145556	0.299398
SCE1572_4488	1.045467	-14.4058	0.145556	0.299398
SCE1572_8897	-0.975801155	-12.2192	0.145675	0.299398
SCE1572_6478	1.313539014	-16.401	0.145812	0.299398
SCE1572_8172	1.313539014	-16.401	0.145812	0.299398
SCE1572_8021	1.283165365	-16.4162	0.145812	0.299398
SCE1572_9705	1.283165365	-16.4162	0.145812	0.299398
SCE1572_8388	1.283165365	-16.4162	0.145812	0.299398
SCE1572_4828	1.283165365	-16.4162	0.145812	0.299398
SCE1572_353	1.283165365	-16.4162	0.145812	0.299398
SCE1572_6954	1.283165365	-16.4162	0.145812	0.299398
SCE1572_8872	1.102034908	-15.0473	0.145885	0.299398
SCE1572_2072	1.102034908	-15.0473	0.145885	0.299398
SCE1572_8683	-1.075026274	-15.0103	0.145885	0.299398
SCE1572_11433	-0.991315568	-13.0456	0.146067	0.299451
SCE1572_7332	1.014627572	-13.7176	0.146075	0.299451
SCE1572_4439	1.413074687	-16.5439	0.146339	0.299451
SCE1572_4105	1.413074687	-16.5439	0.146339	0.299451
SCE1572_115	1.413074687	-16.5439	0.146339	0.299451
SCE1572_11577	1.413074687	-16.5439	0.146339	0.299451
SCE1572_6526	1.38065321	-16.5601	0.146339	0.299451
SCE1572_47	1.38065321	-16.5601	0.146339	0.299451
SCE1572_1497	1.317089669	-16.5039	0.146339	0.299451

SCE1572_637	-1.271423487	-16.4456	0.146339	0.299451
SCE1572_3678	-1.271423487	-16.4456	0.146339	0.299451
SCE1572_2643	1.059221825	-14.4838	0.146409	0.299451
SCE1572_6860	0.986728697	-12.77	0.146472	0.299451
SCE1572_1415	2.050504608	-18.0325	0.146765	0.299451
SCE1572_1490	2.050504608	-18.0325	0.146765	0.299451
SCE1572_3395	2.050504608	-18.0325	0.146765	0.299451
SCE1572_8811	2.050504608	-18.0325	0.146765	0.299451
SCE1572_2116	2.050504608	-18.0325	0.146765	0.299451
SCE1572_4665	2.050504608	-18.0325	0.146765	0.299451
SCE1572_11001	2.050504608	-18.0325	0.146765	0.299451
SCE1572_8976	2.050504608	-18.0325	0.146765	0.299451
SCE1572_7663	1.976504026	-18.0695	0.146765	0.299451
SCE1572_1499	1.976504026	-18.0695	0.146765	0.299451
SCE1572_325	1.976504026	-18.0695	0.146765	0.299451
SCE1572_8432	1.976504026	-18.0695	0.146765	0.299451
SCE1572_1145	1.976504026	-18.0695	0.146765	0.299451
SCE1572_3356	1.976504026	-18.0695	0.146765	0.299451
SCE1572_8767	1.976504026	-18.0695	0.146765	0.299451
SCE1572_5240	-1.633993566	-17.7048	0.146765	0.299451
SCE1572_2353	-1.633993566	-17.7048	0.146765	0.299451
SCE1572_3094	-1.086998916	-15.1418	0.147151	0.300181
SCE1572_1737	1.391541526	-16.777	0.147385	0.300543
SCE1572_2024	1.391541526	-16.777	0.147385	0.300543
SCE1572_5562	0.969584612	-12.0261	0.147628	0.30091
SCE1572_5212	-1.023495974	-14.5695	0.147699	0.30091
SCE1572_1299	1.050504608	-14.6256	0.148053	0.30091
SCE1572_8117	1.667175968	-17.6392	0.148066	0.30091
SCE1572_4917	1.667175968	-17.6392	0.148066	0.30091
SCE1572_7925	1.603045631	-17.6713	0.148066	0.30091
SCE1572_7278	1.603045631	-17.6713	0.148066	0.30091
SCE1572_6611	1.603045631	-17.6713	0.148066	0.30091
SCE1572_8074	1.603045631	-17.6713	0.148066	0.30091
SCE1572_9091	1.603045631	-17.6713	0.148066	0.30091
SCE1572_3518	1.603045631	-17.6713	0.148066	0.30091
SCE1572_10195	1.603045631	-17.6713	0.148066	0.30091
SCE1572_11056	-1.593351582	-17.5325	0.148066	0.30091
SCE1572_5039	-1.42342658	-17.4476	0.148066	0.30091
SCE1572_7264	1.11000562	-15.2548	0.148109	0.30091
SCE1572_211	1.095358844	-15.2622	0.148109	0.30091
SCE1572_5648	1.095358844	-15.2622	0.148109	0.30091
SCE1572_1812	1.095358844	-15.2622	0.148109	0.30091
SCE1572_9875	-1.078778409	-15.2048	0.148109	0.30091
SCE1572_907	1.406648418	-17.0325	0.14827	0.300946
SCE1572_6156	1.406648418	-17.0325	0.14827	0.300946
SCE1572_7655	1.406648418	-17.0325	0.14827	0.300946
SCE1572_4146	1.406648418	-17.0325	0.14827	0.300946
SCE1572_9142	1.378421865	-17.0466	0.14827	0.300946

SCE1572_2855	1.586683447	-17.2826	0.14853	0.300951
SCE1572_10227	1.586557508	-17.2645	0.14853	0.300951
SCE1572_6147	1.586557508	-17.2645	0.14853	0.300951
SCE1572_7049	1.586557508	-17.2645	0.14853	0.300951
SCE1572_8122	1.586557508	-17.2645	0.14853	0.300951
SCE1572_9141	1.586557508	-17.2645	0.14853	0.300951
SCE1572_6214	1.586557508	-17.2645	0.14853	0.300951
SCE1572_10315	-1.512431587	-17.1136	0.14853	0.300951
SCE1572_1542	-1.386900704	-17.0508	0.14853	0.300951
SCE1572_501	1.143614012	-15.316	0.148628	0.301034
SCE1572_7314	1.12850712	-15.3236	0.148628	0.301034
SCE1572_155	-0.977886993	-12.9394	0.148755	0.301234
SCE1572_7119	-1.06497261	-15.3049	0.149176	0.302028
SCE1572_10050	-0.974291304	-12.744	0.149603	0.302804
SCE1572_8147	1.082213468	-14.7618	0.149617	0.302804
SCE1572_2971	1.133768043	-15.4034	0.149757	0.302853
SCE1572_10401	-1.127033578	-15.3734	0.149757	0.302853
SCE1572_10113	-1.127033578	-15.3734	0.149757	0.302853
SCE1572_6949	1.116847103	-15.4119	0.149757	0.302853
SCE1572_7751	1.042467289	-14.3666	0.150005	0.30324
SCE1572_9867	-1.024508747	-14.3412	0.150005	0.30324
SCE1572_796	-0.969014843	-12.5593	0.150154	0.303481
SCE1572_5846	-1.008389081	-14.0701	0.150781	0.304608
SCE1572_2265	-1.008389081	-14.0701	0.150781	0.304608
SCE1572_6983	1.006280608	-13.8267	0.150798	0.304608
SCE1572_6113	-1.115304285	-15.4861	0.151025	0.304891
SCE1572_8374	-1.115304285	-15.4861	0.151025	0.304891
SCE1572_11356	-1.078778409	-15.4679	0.151025	0.304891
SCE1572_10719	1.050504608	-14.4451	0.151089	0.304961
SCE1572_5244	-1.068606604	-14.8642	0.15147	0.305671
SCE1572_7913	1.143614012	-15.5791	0.15172	0.306118
SCE1572_9251	1.021358262	-13.9032	0.151874	0.306257
SCE1572_6007	-1.022395939	-14.4605	0.151877	0.306257
SCE1572_3875	-1.022395939	-14.4605	0.151877	0.306257
SCE1572_11411	1.07435135	-14.9356	0.151987	0.306422
SCE1572_4824	-0.992041087	-13.6834	0.152215	0.306821
SCE1572_5462	1.188008132	-15.6564	0.152461	0.307083
SCE1572_8138	1.188008132	-15.6564	0.152461	0.307083
SCE1572_3474	1.188008132	-15.6564	0.152461	0.307083
SCE1572_6591	1.177477464	-15.6617	0.152461	0.307083
SCE1572_10748	-1.057298682	-14.9426	0.15253	0.307163
SCE1572_4693	-0.963545654	-12.713	0.152563	0.307171
SCE1572_7859	-1.012036858	-14.5194	0.152724	0.307398
SCE1572_2499	1.021358262	-14.2252	0.152764	0.307398
SCE1572_6709	-1.014265648	-14.2074	0.152764	0.307398
SCE1572_8509	1.003743236	-13.7486	0.152998	0.307811
SCE1572_3486	0.993279739	-13.3912	0.153097	0.307838
SCE1572_1929	-1.078778409	-14.9825	0.153099	0.307838

SCE1572_7358	-1.053243317	-14.9697	0.153099	0.307838
SCE1572_2520	1.050504608	-14.5783	0.153173	0.307868
SCE1572_4350	1.050504608	-14.5783	0.153173	0.307868
SCE1572_4934	-0.953062007	-11.8725	0.153342	0.30815
SCE1572_498	0.967583648	-12.7796	0.153545	0.308498
SCE1572_4586	-0.947181526	-11.2483	0.153845	0.309041
SCE1572_5058	1.177883914	-15.7684	0.154099	0.309306
SCE1572_9874	1.177883914	-15.7684	0.154099	0.309306
SCE1572_6804	1.157419812	-15.7786	0.154099	0.309306
SCE1572_5664	-1.090851241	-15.6963	0.154099	0.309306
SCE1572_8881	1.031139283	-14.6353	0.154124	0.309306
SCE1572_1840	-1.040097941	-15.0543	0.154327	0.309596
SCE1572_6837	-1.040097941	-15.0543	0.154327	0.309596
SCE1572_4658	-1.032235823	-14.6432	0.154628	0.310117
SCE1572_4475	-0.939332063	-8.75101	0.154645	0.310117
SCE1572_10175	-0.966238656	-12.8405	0.154775	0.310316
SCE1572_10103	-1.129404482	-15.7645	0.155007	0.310722
SCE1572_11123	1.003854515	-14.0964	0.155108	0.310867
SCE1572_8737	1.107088136	-15.1823	0.155689	0.311907
SCE1572_2639	1.093148945	-15.1893	0.155689	0.311907
SCE1572_8522	-0.954566382	-12.3802	0.155717	0.311907
SCE1572_929	-1.000121465	-14.1149	0.155871	0.312143
SCE1572_1234	1.165981825	-15.8898	0.155983	0.312143
SCE1572_2959	1.165981825	-15.8898	0.155983	0.312143
SCE1572_3040	1.143614012	-15.901	0.155983	0.312143
SCE1572_9919	1.143614012	-15.901	0.155983	0.312143
SCE1572_2311	-0.951145129	-12.0821	0.156131	0.31238
SCE1572_3968	1.008684432	-14.1611	0.156269	0.31249
SCE1572_3577	-1.038224311	-14.744	0.156275	0.31249
SCE1572_9502	-1.01685066	-14.7333	0.156275	0.31249
SCE1572_8484	-1.03438429	-14.7676	0.156872	0.313592
SCE1572_454	1.223341205	-15.9867	0.157034	0.313592
SCE1572_1551	1.223341205	-15.9867	0.157034	0.313592
SCE1572_3023	1.223341205	-15.9867	0.157034	0.313592
SCE1572_8613	1.199882232	-15.9984	0.157034	0.313592
SCE1572_3668	1.199882232	-15.9984	0.157034	0.313592
SCE1572_2693	1.199882232	-15.9984	0.157034	0.313592
SCE1572_7118	0.989104063	-13.9783	0.157126	0.313715
SCE1572_1576	1.080561842	-15.2696	0.157209	0.313822
SCE1572_10650	-0.93780211	-10.8631	0.15742	0.314152
SCE1572_6216	1.050504608	-14.8321	0.157497	0.314152
SCE1572_9918	1.03936405	-14.8377	0.157497	0.314152
SCE1572_5637	1.03936405	-14.8377	0.157497	0.314152
SCE1572_337	1.024032396	-14.5014	0.157554	0.314152
SCE1572_9183	1.024032396	-14.5014	0.157554	0.314152
SCE1572_6204	-1.069789626	-15.2707	0.158037	0.314996
SCE1572_11416	-1.069789626	-15.2707	0.158037	0.314996
SCE1572_10646	1.062000247	-14.8829	0.158152	0.315142

SCE1572_10200	-1.160392175	-15.9941	0.15817	0.315142
SCE1572_5132	-0.990241735	-14.2491	0.158457	0.315654
SCE1572_9984	1.004907741	-14.0635	0.158704	0.316086
SCE1572_6451	0.989568946	-13.7115	0.159011	0.316638
SCE1572_7176	-0.98191687	-14.0569	0.159127	0.316754
SCE1572_2669	1.031768045	-14.5877	0.159223	0.316754
SCE1572_4537	1.240700448	-16.1774	0.159401	0.316754
SCE1572_4864	1.21400334	-16.1288	0.159401	0.316754
SCE1572_4714	1.21400334	-16.1288	0.159401	0.316754
SCE1572_10238	1.21400334	-16.1288	0.159401	0.316754
SCE1572_8072	1.21400334	-16.1288	0.159401	0.316754
SCE1572_1792	-1.212529798	-16.0791	0.159401	0.316754
SCE1572_1580	1.188008132	-16.1418	0.159401	0.316754
SCE1572_244	1.180381069	-16.1457	0.159401	0.316754
SCE1572_3254	-1.15594627	-16.0508	0.159401	0.316754
SCE1572_9077	1.06247725	-14.9416	0.159556	0.317002
SCE1572_3758	0.97246192	-13.3272	0.159784	0.317228
SCE1572_3752	-0.960221799	-13.198	0.159799	0.317228
SCE1572_11300	-1.01988472	-14.5904	0.159824	0.317228
SCE1572_5572	0.949911586	-12.586	0.159841	0.317228
SCE1572_10428	1.10054529	-15.42	0.159851	0.317228
SCE1572_1378	1.10054529	-15.42	0.159851	0.317228
SCE1572_2019	1.002938925	-14.3469	0.15995	0.317365
SCE1572_5149	0.979538086	-13.9242	0.160042	0.317487
SCE1572_6942	1.075379276	-14.9965	0.160311	0.317902
SCE1572_10851	-1.039676247	-14.9509	0.160311	0.317902
SCE1572_959	-0.989023756	-14.3428	0.160482	0.318181
SCE1572_314	1.25909123	-16.2583	0.160738	0.318201
SCE1572_4109	1.25909123	-16.2583	0.160738	0.318201
SCE1572_7936	1.25909123	-16.2583	0.160738	0.318201
SCE1572_3638	1.25909123	-16.2583	0.160738	0.318201
SCE1572_7183	1.137967449	-15.4888	0.160846	0.318201
SCE1572_4091	1.120893936	-15.4973	0.160846	0.318201
SCE1572_5435	1.120893936	-15.4973	0.160846	0.318201
SCE1572_5814	1.120893936	-15.4973	0.160846	0.318201
SCE1572_1000	-0.936759404	-11.8608	0.160859	0.318201
SCE1572_2923	1.040520519	-14.6795	0.161103	0.318201
SCE1572_1417	1.030466855	-14.6846	0.161103	0.318201
SCE1572_10234	31.37615295	-34.328	0.161491	0.318201
SCE1572_10531	31.27181629	-34.3801	0.161491	0.318201
SCE1572_8573	31.27181629	-34.3801	0.161491	0.318201
SCE1572_7781	31.27181629	-34.3801	0.161491	0.318201
SCE1572_8175	31.27181629	-34.3801	0.161491	0.318201
SCE1572_8376	31.27181629	-34.3801	0.161491	0.318201
SCE1572_1174	31.27181629	-34.3801	0.161491	0.318201
SCE1572_239	31.27181629	-34.3801	0.161491	0.318201
SCE1572_11074	31.27181629	-34.3801	0.161491	0.318201
SCE1572_123	31.27181629	-34.3801	0.161491	0.318201

SCE1572_4344	31.27181629	-34.3801	0.161491	0.318201
SCE1572_4599	31.27181629	-34.3801	0.161491	0.318201
SCE1572_6931	31.27181629	-34.3801	0.161491	0.318201
SCE1572_3653	31.27181629	-34.3801	0.161491	0.318201
SCE1572_3520	31.27181629	-34.3801	0.161491	0.318201
SCE1572_10636	31.27181629	-34.3801	0.161491	0.318201
SCE1572_3189	31.27181629	-34.3801	0.161491	0.318201
SCE1572_5422	31.27181629	-34.3801	0.161491	0.318201
SCE1572_5197	31.27181629	-34.3801	0.161491	0.318201
SCE1572_3054	31.27181629	-34.3801	0.161491	0.318201
SCE1572_2295	31.27181629	-34.3801	0.161491	0.318201
SCE1572_7439	31.27181629	-34.3801	0.161491	0.318201
SCE1572_3027	-0.978227111	-14.3971	0.161605	0.318367
SCE1572_5248	-1.024783519	-14.6875	0.161784	0.3186
SCE1572_3228	-1.024783519	-14.6875	0.161784	0.3186
SCE1572_10465	1.063560761	-15.0666	0.161941	0.31885
SCE1572_4884	-1.204309291	-16.2676	0.162194	0.319234
SCE1572_3424	1.024817964	-14.4579	0.162197	0.319234
SCE1572_4321	0.969980903	-13.4435	0.162414	0.319601
SCE1572_10422	-0.949495392	-12.8601	0.162839	0.320377
SCE1572_5249	-0.975030484	-13.7124	0.162911	0.320459
SCE1572_1033	1.125466666	-15.5882	0.163047	0.320547
SCE1572_7385	1.107088136	-15.5973	0.163047	0.320547
SCE1572_1129	1.107088136	-15.5973	0.163047	0.320547
SCE1572_5944	1.039778201	-14.783	0.163236	0.32086
SCE1572_11255	-0.940506609	-12.528	0.16337	0.321062
SCE1572_775	-0.961083366	-13.8945	0.163682	0.321279
SCE1572_3856	0.969137057	-13.619	0.163725	0.321279
SCE1572_7800	-0.963756085	-13.6089	0.163725	0.321279
SCE1572_3063	-0.962037341	-13.6081	0.163725	0.321279
SCE1572_4328	1.252138469	-16.4317	0.163786	0.321279
SCE1572_1254	1.252138469	-16.4317	0.163786	0.321279
SCE1572_7053	1.252138469	-16.4317	0.163786	0.321279
SCE1572_6072	1.252138469	-16.4317	0.163786	0.321279
SCE1572_5229	-1.201034159	-16.3364	0.163786	0.321279
SCE1572_11243	-1.201034159	-16.3364	0.163786	0.321279
SCE1572_11536	-1.008389081	-14.7806	0.164013	0.321665
SCE1572_22	-0.958265602	-13.4925	0.164082	0.32168
SCE1572_10237	1.032357261	-14.5416	0.164112	0.32168
SCE1572_1736	1.032357261	-14.5416	0.164112	0.32168
SCE1572_4323	1.169149104	-15.6658	0.164267	0.321743
SCE1572_6436	1.150040281	-15.6754	0.164267	0.321743
SCE1572_4473	-1.101498486	-15.6085	0.164267	0.321743
SCE1572_4998	-1.061970121	-15.5888	0.164267	0.321743
SCE1572_1473	1.07907376	-15.1963	0.164737	0.322252
SCE1572_8199	1.07907376	-15.1963	0.164737	0.322252
SCE1572_11030	-1.053832052	-15.1584	0.164737	0.322252
SCE1572_5754	-1.0252629	-15.1441	0.164737	0.322252

SCE1572_2752	-0.980192189	-14.2999	0.164742	0.322252
SCE1572_8400	-1.008389081	-14.5396	0.164802	0.322252
SCE1572_4988	-0.989645359	-14.5302	0.164802	0.322252
SCE1572_10755	1.028136795	-14.8433	0.164826	0.322252
SCE1572_9345	-1.003943176	-14.8049	0.164826	0.322252
SCE1572_2425	-0.922591208	-11.1097	0.164833	0.322252
SCE1572_2791	-0.945195255	-12.8793	0.165354	0.323048
SCE1572_10602	1.022307716	-14.5924	0.165519	0.323048
SCE1572_3024	1.022307716	-14.5924	0.165519	0.323048
SCE1572_2893	1.347486346	-16.5767	0.165529	0.323048
SCE1572_8132	1.347486346	-16.5767	0.165529	0.323048
SCE1572_3053	1.347486346	-16.5767	0.165529	0.323048
SCE1572_11366	1.347486346	-16.5767	0.165529	0.323048
SCE1572_10558	1.347486346	-16.5767	0.165529	0.323048
SCE1572_7351	1.347486346	-16.5767	0.165529	0.323048
SCE1572_7517	-0.941458073	-13.0447	0.165552	0.323048
SCE1572_10037	-1.09633678	-15.6517	0.165578	0.323048
SCE1572_4724	1.009862623	-14.3829	0.165999	0.323809
SCE1572_4216	-0.917718722	-10.6633	0.166079	0.323904
SCE1572_11242	0.968627601	-13.7155	0.166119	0.323923
SCE1572_795	-0.925516529	-12.0367	0.16644	0.324488
SCE1572_7618	-0.941633274	-13.0129	0.166773	0.325078
SCE1572_4557	1.136661252	-15.789	0.166992	0.325384
SCE1572_8883	1.136661252	-15.789	0.166992	0.325384
SCE1572_2879	-0.931826744	-12.8026	0.167257	0.325838
SCE1572_6574	1.000172932	-14.4284	0.167349	0.325838
SCE1572_10390	1.000172932	-14.4284	0.167349	0.325838
SCE1572_11094	0.991610919	-14.4327	0.167349	0.325838
SCE1572_5802	-0.949495392	-13.6256	0.16755	0.32617
SCE1572_4194	-0.914561602	-10.8426	0.167847	0.326644
SCE1572_8987	-1.008389081	-14.6551	0.167856	0.326644
SCE1572_5082	-0.952144971	-13.7657	0.16791	0.326682
SCE1572_1922	1.097810323	-15.3389	0.168059	0.326682
SCE1572_5398	1.097810323	-15.3389	0.168059	0.326682
SCE1572_9917	1.097810323	-15.3389	0.168059	0.326682
SCE1572_1712	1.082213468	-15.3467	0.168059	0.326682
SCE1572_1848	1.016153103	-14.4622	0.168062	0.326682
SCE1572_10656	-0.940882262	-13.1445	0.168176	0.326844
SCE1572_5429	-0.965797204	-14.0649	0.168281	0.326987
SCE1572_2925	0.942556694	-13.1602	0.168491	0.327292
SCE1572_4010	1.050504608	-15.009	0.1685	0.327292
SCE1572_9688	-0.919611112	-12.0913	0.169138	0.328471
SCE1572_2966	0.950968934	-13.4738	0.16924	0.328608
SCE1572_93	0.996348838	-14.3117	0.169528	0.328644
SCE1572_390	1.353067378	-16.7963	0.169557	0.328644
SCE1572_4347	1.353067378	-16.7963	0.169557	0.328644
SCE1572_4235	1.353067378	-16.7963	0.169557	0.328644
SCE1572_827	1.353067378	-16.7963	0.169557	0.328644

SCE1572_11599	1.353067378	-16.7963	0.169557	0.328644
SCE1572_814	1.353067378	-16.7963	0.169557	0.328644
SCE1572_2654	1.313539014	-16.816	0.169557	0.328644
SCE1572_6359	1.313539014	-16.816	0.169557	0.328644
SCE1572_3841	1.006110488	-14.5103	0.169571	0.328644
SCE1572_11410	1.120893936	-15.9124	0.170178	0.329698
SCE1572_10967	1.120893936	-15.9124	0.170178	0.329698
SCE1572_1890	-0.976680221	-14.3173	0.17023	0.329738
SCE1572_2734	-0.911415353	-11.5448	0.170328	0.329829
SCE1572_6382	1.013978732	-14.5508	0.170371	0.329829
SCE1572_8852	1.013978732	-14.5508	0.170371	0.329829
SCE1572_1121	1.028971446	-14.7884	0.170516	0.329933
SCE1572_6638	1.028971446	-14.7884	0.170516	0.329933
SCE1572_573	-0.912601997	-11.6345	0.170559	0.329933
SCE1572_9915	0.937523403	-13.3222	0.170615	0.329933
SCE1572_734	1.084057168	-15.4283	0.170644	0.329933
SCE1572_10313	1.067378426	-15.4366	0.170644	0.329933
SCE1572_8536	1.059451321	-15.4372	0.170644	0.329933
SCE1572_3140	-0.925054533	-12.8837	0.171725	0.331961
SCE1572_7364	-0.943537937	-13.3496	0.17177	0.331988
SCE1572_6229	1.17603549	-16.0103	0.171981	0.332152
SCE1572_10725	1.17603549	-16.0103	0.171981	0.332152
SCE1572_9140	1.17603549	-16.0103	0.171981	0.332152
SCE1572_3703	1.17603549	-16.0103	0.171981	0.332152
SCE1572_5316	1.103615944	-15.506	0.172071	0.332205
SCE1572_10819	-0.946583797	-13.9017	0.172072	0.332205
SCE1572_416	1.005416718	-14.8546	0.172504	0.332978
SCE1572_8492	-0.917177672	-12.2449	0.172683	0.333261
SCE1572_6507	0.984077246	-14.2438	0.17281	0.333446
SCE1572_6165	-0.962670781	-14.0797	0.173058	0.333803
SCE1572_9306	-0.962670781	-14.0797	0.173058	0.333803
SCE1572_10116	-0.957763008	-14.4074	0.173302	0.334212
SCE1572_9528	-0.917786532	-12.6463	0.173544	0.334418
SCE1572_6606	1.027234829	-14.9003	0.173572	0.334418
SCE1572_9030	1.027234829	-14.9003	0.173572	0.334418
SCE1572_11177	-0.994583282	-14.8546	0.173572	0.334418
SCE1572_5997	-0.994583282	-14.8546	0.173572	0.334418
SCE1572_10415	-1.036958233	-15.4888	0.1736	0.334418
SCE1572_4746	0.969584612	-14.1136	0.173729	0.334606
SCE1572_4641	0.981557254	-14.6601	0.173915	0.334902
SCE1572_10297	-1.101498486	-16.0236	0.173951	0.33491
SCE1572_9000	-0.934827342	-13.4272	0.174286	0.335207
SCE1572_644	-0.934827342	-13.4272	0.174286	0.335207
SCE1572_6260	0.96673625	-14.289	0.174304	0.335207
SCE1572_1477	0.981791858	-14.1406	0.174421	0.335207
SCE1572_5838	1.360844728	-17.0554	0.174456	0.335207
SCE1572_9144	1.360844728	-17.0554	0.174456	0.335207
SCE1572_1562	1.360844728	-17.0554	0.174456	0.335207

SCE1572_7371	1.360844728	-17.0554	0.174456	0.335207
SCE1572_4017	1.313539014	-17.0791	0.174456	0.335207
SCE1572_11045	1.313539014	-17.0791	0.174456	0.335207
SCE1572_7557	1.313539014	-17.0791	0.174456	0.335207
SCE1572_9623	0.957395203	-13.9941	0.174508	0.335248
SCE1572_7433	-1.013111491	-14.8919	0.174694	0.335481
SCE1572_8964	-0.989652519	-14.8802	0.174694	0.335481
SCE1572_3137	-0.921839049	-12.9795	0.174833	0.335686
SCE1572_996	0.999878535	-14.6999	0.174898	0.33569
SCE1572_5612	-0.964241978	-14.6573	0.174898	0.33569
SCE1572_1022	1.088472458	-15.6067	0.175242	0.336104
SCE1572_4707	-1.067890093	-15.5473	0.175242	0.336104
SCE1572_4947	-1.067890093	-15.5473	0.175242	0.336104
SCE1572_7998	1.066958148	-15.6011	0.175242	0.336104
SCE1572_1757	0.93746467	-13.4805	0.175658	0.336842
SCE1572_4995	-1.014265648	-15.2074	0.175703	0.336866
SCE1572_5528	0.950968934	-13.9099	0.175796	0.336982
SCE1572_9453	-1.000121465	-14.6999	0.175927	0.337123
SCE1572_1837	-0.916646513	-13.1498	0.175933	0.337123
SCE1572_8365	1.134568873	-16.1686	0.17611	0.337215
SCE1572_8794	1.134568873	-16.1686	0.17611	0.337215
SCE1572_2273	-1.094545725	-16.0815	0.17611	0.337215
SCE1572_619	-1.094545725	-16.0815	0.17611	0.337215
SCE1572_163	0.938029879	-13.5888	0.176198	0.337323
SCE1572_10827	1.130674957	-15.6851	0.177011	0.338127
SCE1572_3922	1.130674957	-15.6851	0.177011	0.338127
SCE1572_2350	-0.89776066	-11.2329	0.177075	0.338127
SCE1572_3910	-0.940598992	-13.9282	0.177083	0.338127
SCE1572_7547	1.037904571	-15.0153	0.177117	0.338127
SCE1572_7156	-0.973742938	-14.9597	0.177117	0.338127
SCE1572_9462	-0.973742938	-14.9597	0.177117	0.338127
SCE1572_5998	-0.973742938	-14.9597	0.177117	0.338127
SCE1572_3146	1.535931435	-17.2898	0.177284	0.338127
SCE1572_3907	1.535931435	-17.2898	0.177284	0.338127
SCE1572_9935	1.535931435	-17.2898	0.177284	0.338127
SCE1572_4521	1.535931435	-17.2898	0.177284	0.338127
SCE1572_2586	1.506785089	-17.3044	0.177284	0.338127
SCE1572_7775	1.483464015	-17.316	0.177284	0.338127
SCE1572_603	1.483464015	-17.316	0.177284	0.338127
SCE1572_7904	1.483464015	-17.316	0.177284	0.338127
SCE1572_10326	1.483464015	-17.316	0.177284	0.338127
SCE1572_170	1.483464015	-17.316	0.177284	0.338127
SCE1572_8731	1.483464015	-17.316	0.177284	0.338127
SCE1572_3780	-1.396954369	-17.1713	0.177284	0.338127
SCE1572_1665	-0.918850075	-13.0496	0.177293	0.338127
SCE1572_966	-0.915942832	-13.4283	0.177476	0.338414
SCE1572_2171	0.932590405	-13.2877	0.177653	0.33869
SCE1572_5971	-0.93752275	-13.9416	0.177754	0.338821

SCE1572_335	1.007112012	-14.7993	0.178128	0.339473
SCE1572_10895	-0.945847615	-14.2269	0.17823	0.339543
SCE1572_10323	-1.018657417	-15.0125	0.178429	0.339543
SCE1572_6284	-0.993122325	-14.9997	0.178429	0.339543
SCE1572_6832	1.231076853	-16.2723	0.178488	0.339543
SCE1572_5471	1.231076853	-16.2723	0.178488	0.339543
SCE1572_7379	1.231076853	-16.2723	0.178488	0.339543
SCE1572_2999	1.231076853	-16.2723	0.178488	0.339543
SCE1572_8995	1.202507701	-16.2866	0.178488	0.339543
SCE1572_9430	1.202507701	-16.2866	0.178488	0.339543
SCE1572_6237	1.202507701	-16.2866	0.178488	0.339543
SCE1572_11463	-0.894687003	-11.3681	0.178523	0.33955
SCE1572_10824	-0.933842128	-13.7433	0.178579	0.339594
SCE1572_3764	0.955645421	-14.1205	0.178732	0.339762
SCE1572_5479	1.066446152	-15.3546	0.178732	0.339762
SCE1572_3434	-0.904749009	-12.4174	0.178813	0.339815
SCE1572_2938	-0.922619686	-13.6487	0.178915	0.339815
SCE1572_1843	1.07096871	-15.7149	0.178922	0.339815
SCE1572_11481	1.07096871	-15.7149	0.178922	0.339815
SCE1572_9527	-1.049031066	-15.6754	0.178922	0.339815
SCE1572_4086	0.967647675	-14.1477	0.179513	0.340816
SCE1572_10732	0.960523241	-14.1513	0.179513	0.340816
SCE1572_11519	1.024032396	-15.0863	0.179814	0.341325
SCE1572_2940	-1.021445234	-15.359	0.1804	0.342348
SCE1572_2996	0.980963675	-14.4798	0.18045	0.342348
SCE1572_3696	-0.966568906	-14.4536	0.18045	0.342348
SCE1572_5530	0.93395035	-13.5247	0.180539	0.342392
SCE1572_9060	0.929273863	-13.5271	0.180539	0.342392
SCE1572_6393	-0.898910604	-12.5943	0.180623	0.34249
SCE1572_6879	-0.898511463	-12.3881	0.180797	0.342572
SCE1572_3123	0.972502096	-14.3236	0.180839	0.342572
SCE1572_3714	-0.945653326	-14.3018	0.180839	0.342572
SCE1572_11185	1.115599636	-15.7995	0.180992	0.342572
SCE1572_684	1.115599636	-15.7995	0.180992	0.342572
SCE1572_8726	1.115599636	-15.7995	0.180992	0.342572
SCE1572_813	1.115599636	-15.7995	0.180992	0.342572
SCE1572_4441	1.094225985	-15.8102	0.180992	0.342572
SCE1572_8431	1.094225985	-15.8102	0.180992	0.342572
SCE1572_993	-1.041941641	-15.7208	0.180992	0.342572
SCE1572_1780	-0.975967604	-15.0863	0.18128	0.343055
SCE1572_5434	0.946481543	-14.061	0.18141	0.343241
SCE1572_4429	-0.941718042	-14.3192	0.181772	0.343863
SCE1572_6951	1.033431094	-15.4536	0.182184	0.344379
SCE1572_5647	1.033431094	-15.4536	0.182184	0.344379
SCE1572_1292	1.033431094	-15.4536	0.182184	0.344379
SCE1572_11559	0.954969977	-14.0881	0.182209	0.344379
SCE1572_265	0.950968934	-14.0904	0.182209	0.344379
SCE1572_205	0.989536591	-14.705	0.182243	0.344382

SCE1572_735	0.969584612	-14.5286	0.182526	0.344856
SCE1572_11007	-0.900056459	-12.7396	0.182677	0.345079
SCE1572_10934	1.008684432	-15.1611	0.182833	0.345311
SCE1572_9945	-0.878604693	-9.39905	0.183052	0.345662
SCE1572_9217	-0.931194575	-13.9688	0.183136	0.345759
SCE1572_8430	-0.928278906	-13.767	0.183293	0.345993
SCE1572_7326	-0.949495392	-14.5325	0.183629	0.346335
SCE1572_6261	1.535931435	-17.7048	0.183672	0.346335
SCE1572_10259	1.535931435	-17.7048	0.183672	0.346335
SCE1572_6639	1.535931435	-17.7048	0.183672	0.346335
SCE1572_8356	1.535931435	-17.7048	0.183672	0.346335
SCE1572_11520	1.535931435	-17.7048	0.183672	0.346335
SCE1572_8878	1.220429609	-16.4476	0.184043	0.34664
SCE1572_7042	1.220429609	-16.4476	0.184043	0.34664
SCE1572_6256	1.220429609	-16.4476	0.184043	0.34664
SCE1572_8952	1.188008132	-16.4638	0.184043	0.34664
SCE1572_11423	1.188008132	-16.4638	0.184043	0.34664
SCE1572_11008	-1.127033578	-16.3734	0.184043	0.34664
SCE1572_9549	1.068426516	-15.5236	0.184097	0.34664
SCE1572_5819	1.068426516	-15.5236	0.184097	0.34664
SCE1572_2671	-0.888504762	-12.0426	0.184481	0.347115
SCE1572_590	1.036005038	-15.2178	0.184481	0.347115
SCE1572_1276	1.021358262	-15.2252	0.184481	0.347115
SCE1572_10419	1.021358262	-15.2252	0.184481	0.347115
SCE1572_419	-0.963301192	-14.5621	0.184778	0.347611
SCE1572_3275	-0.908853408	-13.1199	0.184957	0.347887
SCE1572_10515	-0.91658077	-13.8156	0.185413	0.348682
SCE1572_1369	-0.930386569	-14.0311	0.185571	0.348916
SCE1572_5689	1.07435135	-15.9356	0.185698	0.348967
SCE1572_5889	-1.06497261	-15.8898	0.185698	0.348967
SCE1572_2581	-1.06497261	-15.8898	0.185698	0.348967
SCE1572_5599	0.924069488	-13.5734	0.185841	0.349174
SCE1572_893	-1.030415388	-15.5286	0.186153	0.349636
SCE1572_471	-1.030415388	-15.5286	0.186153	0.349636
SCE1572_11183	-0.939801996	-14.0519	0.186433	0.350098
SCE1572_2	-0.892911864	-12.9344	0.18686	0.350274
SCE1572_11065	-2.271423487	-19.1935	0.186957	0.350274
SCE1572_6077	-2.271423487	-19.1935	0.186957	0.350274
SCE1572_11231	0.972120155	-14.4843	0.18699	0.350274
SCE1572_4376	1.898501514	-18.1085	0.187026	0.350274
SCE1572_10579	1.898501514	-18.1085	0.187026	0.350274
SCE1572_740	1.898501514	-18.1085	0.187026	0.350274
SCE1572_2846	1.898501514	-18.1085	0.187026	0.350274
SCE1572_9792	1.898501514	-18.1085	0.187026	0.350274
SCE1572_8589	1.898501514	-18.1085	0.187026	0.350274
SCE1572_10588	1.898501514	-18.1085	0.187026	0.350274
SCE1572_1274	1.898501514	-18.1085	0.187026	0.350274
SCE1572_5338	1.898501514	-18.1085	0.187026	0.350274

SCE1572_5938	1.898501514	-18.1085	0.187026	0.350274
SCE1572_952	-1.464068565	-17.7898	0.187026	0.350274
SCE1572_8151	1.313539014	-16.5937	0.187314	0.350438
SCE1572_1377	1.278773596	-16.611	0.187314	0.350438
SCE1572_7442	1.278773596	-16.611	0.187314	0.350438
SCE1572_11507	1.278773596	-16.611	0.187314	0.350438
SCE1572_9541	1.278773596	-16.611	0.187314	0.350438
SCE1572_3035	1.278773596	-16.611	0.187314	0.350438
SCE1572_1786	-0.92092624	-14.1963	0.187562	0.35084
SCE1572_9847	0.98637427	-15.041	0.187792	0.351083
SCE1572_3644	0.98637427	-15.041	0.187792	0.351083
SCE1572_1013	0.973198882	-15.0476	0.187792	0.351083
SCE1572_3347	0.889776766	-12.6017	0.18787	0.351167
SCE1572_9580	1.004210956	-15.3077	0.188103	0.351344
SCE1572_7294	-1.002236854	-15.2735	0.188103	0.351344
SCE1572_890	-0.971863205	-15.2583	0.188103	0.351344
SCE1572_4760	1.069613431	-15.6161	0.18837	0.351344
SCE1572_10625	1.050504608	-15.6256	0.18837	0.351344
SCE1572_8137	1.151787944	-16.0225	0.188387	0.351344
SCE1572_1071	1.151787944	-16.0225	0.188387	0.351344
SCE1572_2281	1.151787944	-16.0225	0.188387	0.351344
SCE1572_4577	1.127125889	-16.0348	0.188387	0.351344
SCE1572_7428	1.127125889	-16.0348	0.188387	0.351344
SCE1572_10421	1.127125889	-16.0348	0.188387	0.351344
SCE1572_5066	-0.882794991	-12.1908	0.188387	0.351344
SCE1572_2410	-0.906548783	-13.3921	0.188399	0.351344
SCE1572_2691	-0.884730613	-12.4945	0.188506	0.351471
SCE1572_6013	-0.944613171	-14.6474	0.188534	0.351471
SCE1572_2575	0.982333105	-14.8662	0.188974	0.352229
SCE1572_7084	0.898501514	-12.9938	0.189039	0.352288
SCE1572_4831	-0.88524085	-12.8422	0.189342	0.352669
SCE1572_1484	0.960306799	-14.5332	0.189378	0.352669
SCE1572_541	0.960306799	-14.5332	0.189378	0.352669
SCE1572_10891	-0.953941297	-14.5124	0.189378	0.352669
SCE1572_10212	1.010611881	-15.093	0.189492	0.35282
SCE1572_1549	-0.927469086	-14.2361	0.189594	0.352946
SCE1572_8762	0.928875163	-14.0388	0.189721	0.35312
SCE1572_8733	0.979119975	-14.7102	0.189901	0.353195
SCE1572_4390	0.979119975	-14.7102	0.189901	0.353195
SCE1572_3504	-0.959479481	-14.6795	0.189901	0.353195
SCE1572_6729	-1.756850314	-18.1288	0.189984	0.353195
SCE1572_645	-1.756850314	-18.1288	0.189984	0.353195
SCE1572_8495	0.950968934	-14.4124	0.190076	0.353195
SCE1572_3243	0.950968934	-14.4124	0.190076	0.353195
SCE1572_10581	1.050504608	-15.3626	0.190097	0.353195
SCE1572_7349	1.034384942	-15.3707	0.190097	0.353195
SCE1572_1625	1.034384942	-15.3707	0.190097	0.353195
SCE1572_6096	0.894000122	-12.8693	0.19018	0.353287

SCE1572_10544	2.313539014	-18.901	0.191276	0.354822
SCE1572_4242	2.313539014	-18.901	0.191276	0.354822
SCE1572_3021	2.313539014	-18.901	0.191276	0.354822
SCE1572_6414	2.313539014	-18.901	0.191276	0.354822
SCE1572_328	2.313539014	-18.901	0.191276	0.354822
SCE1572_8596	2.313539014	-18.901	0.191276	0.354822
SCE1572_7014	2.313539014	-18.901	0.191276	0.354822
SCE1572_950	-1.823471365	-18.4211	0.191276	0.354822
SCE1572_4028	-0.910676143	-14.0537	0.191596	0.35529
SCE1572_3646	-0.910676143	-14.0537	0.191596	0.35529
SCE1572_7454	-0.915279677	-14.2866	0.191771	0.355553
SCE1572_3163	0.886886885	-13.0144	0.192543	0.35692
SCE1572_8841	0.937163135	-14.0973	0.192578	0.356922
SCE1572_8402	0.954644592	-14.7731	0.192833	0.357332
SCE1572_10877	0.940080618	-14.3398	0.19292	0.357431
SCE1572_4438	0.994470573	-15.1682	0.193208	0.357686
SCE1572_7909	-0.963301192	-15.1471	0.193208	0.357686
SCE1572_2783	-0.963301192	-15.1471	0.193208	0.357686
SCE1572_3860	-0.963301192	-15.1471	0.193208	0.357686
SCE1572_1645	-0.877509279	-12.4853	0.193282	0.357686
SCE1572_11413	0.954289293	-14.6264	0.193353	0.357686
SCE1572_10517	1.029746048	-15.7355	0.193364	0.357686
SCE1572_1410	1.029746048	-15.7355	0.193364	0.357686
SCE1572_6699	1.029746048	-15.7355	0.193364	0.357686
SCE1572_6431	0.916780248	-13.6686	0.193854	0.358484
SCE1572_11518	0.989104063	-14.9783	0.193898	0.358484
SCE1572_11134	0.976504026	-14.9846	0.193898	0.358484
SCE1572_7810	-0.88999438	-13.2548	0.194112	0.358818
SCE1572_4261	0.916203516	-13.8448	0.194293	0.359088
SCE1572_10519	0.917918968	-14.2413	0.194396	0.359216
SCE1572_3809	-0.974441749	-15.4157	0.194519	0.359281
SCE1572_6408	1.107088136	-16.1823	0.194613	0.359281
SCE1572_1696	1.107088136	-16.1823	0.194613	0.359281
SCE1572_4699	1.107088136	-16.1823	0.194613	0.359281
SCE1572_4525	1.107088136	-16.1823	0.194613	0.359281
SCE1572_7960	-0.908853408	-14.1199	0.194636	0.359281
SCE1572_9292	-0.930386569	-14.6161	0.194796	0.359514
SCE1572_925	-0.913871482	-13.9298	0.19502	0.359863
SCE1572_3633	1.272897029	-16.8364	0.195166	0.35988
SCE1572_1437	1.272897029	-16.8364	0.195166	0.35988
SCE1572_6110	1.272897029	-16.8364	0.195166	0.35988
SCE1572_10285	-1.178314083	-16.74	0.195166	0.35988
SCE1572_7511	1.00656126	-15.2326	0.195242	0.359957
SCE1572_3175	-0.881241176	-13.1675	0.195482	0.360337
SCE1572_365	1.072530914	-15.8211	0.196191	0.36158
SCE1572_3131	0.941570236	-14.5426	0.19649	0.362068
SCE1572_3321	-0.862958642	-12.386	0.196662	0.362295
SCE1572_2327	0.891196714	-13.1527	0.196682	0.362295

SCE1572_897	1.050504608	-15.5325	0.19698	0.362716
SCE1572_8909	1.050504608	-15.5325	0.19698	0.362716
SCE1572_3605	0.864915477	-12.2244	0.197058	0.362797
SCE1572_921	0.959902059	-15.0543	0.197655	0.363831
SCE1572_11387	-0.861603697	-12.3748	0.197705	0.363861
SCE1572_4463	0.958874132	-14.8779	0.1978	0.363972
SCE1572_9995	0.958058359	-14.7208	0.197887	0.364067
SCE1572_8275	-0.886505976	-13.5822	0.197929	0.36408
SCE1572_3377	0.918907725	-14.3129	0.197981	0.364096
SCE1572_132	-0.850917775	-10.2726	0.198006	0.364096
SCE1572_10966	1.173361356	-16.3012	0.198245	0.364407
SCE1572_77	1.173361356	-16.3012	0.198245	0.364407
SCE1572_3168	0.907900212	-13.9314	0.198957	0.365653
SCE1572_4272	-0.916992752	-14.2125	0.19918	0.365998
SCE1572_7288	-0.876891643	-13.542	0.199458	0.366446
SCE1572_11102	-0.92903129	-14.7149	0.199543	0.366537
SCE1572_4262	0.983390412	-15.1067	0.199701	0.366571
SCE1572_4517	0.983390412	-15.1067	0.199701	0.366571
SCE1572_513	0.983390412	-15.1067	0.199701	0.366571
SCE1572_11	-0.949495392	-15.0731	0.199701	0.366571
SCE1572_9037	-0.88524085	-13.8422	0.199849	0.36667
SCE1572_4793	-1.271423487	-16.8861	0.199929	0.36667
SCE1572_8821	-1.271423487	-16.8861	0.199929	0.36667
SCE1572_9642	-1.171887814	-16.8364	0.199929	0.36667
SCE1572_6876	-1.171887814	-16.8364	0.199929	0.36667
SCE1572_9930	-0.89876459	-13.9373	0.19997	0.366682
SCE1572_11560	0.874598922	-13.0261	0.200515	0.367617
SCE1572_11090	-0.895275001	-13.9508	0.201014	0.368467
SCE1572_10071	-0.849233296	-11.508	0.201049	0.368467
SCE1572_6457	0.943589404	-14.7786	0.20128	0.368827
SCE1572_4582	0.885445362	-13.4056	0.201373	0.368929
SCE1572_5764	0.924363578	-14.1693	0.201441	0.368929
SCE1572_3095	-0.887508505	-14.144	0.201441	0.368929
SCE1572_10235	0.951052137	-14.9517	0.201563	0.369087
SCE1572_316	0.914443058	-14.2786	0.201694	0.369264
SCE1572_11089	-0.94280074	-15.1029	0.201872	0.369461
SCE1572_9879	-0.94280074	-15.1029	0.201872	0.369461
SCE1572_8177	1.01808313	-15.3788	0.202197	0.369989
SCE1572_5922	-1.133919963	-16.3028	0.202302	0.369989
SCE1572_9161	-1.069789626	-16.2707	0.202302	0.369989
SCE1572_11565	-1.069789626	-16.2707	0.202302	0.369989
SCE1572_9134	1.031139283	-15.6353	0.202499	0.370157
SCE1572_815	1.031139283	-15.6353	0.202499	0.370157
SCE1572_5885	1.031139283	-15.6353	0.202499	0.370157
SCE1572_4166	1.050504608	-15.9476	0.202662	0.37039
SCE1572_1570	-0.894056406	-13.9049	0.20288	0.370661
SCE1572_5353	-0.894056406	-13.9049	0.20288	0.370661
SCE1572_8744	0.916203516	-14.1078	0.203546	0.371789

SCE1572_8148	0.950968934	-14.9973	0.203602	0.371789
SCE1572_7648	0.898501514	-13.8536	0.203604	0.371789
SCE1572_8533	0.932109907	-14.5473	0.203874	0.372218
SCE1572_1317	-0.890601703	-13.9181	0.203949	0.372289
SCE1572_4771	0.96561571	-15.1827	0.20418	0.372648
SCE1572_8225	0.930210374	-14.6858	0.204404	0.372861
SCE1572_5115	-0.919951117	-14.6598	0.204404	0.372861
SCE1572_732	0.919718	-14.691	0.204404	0.372861
SCE1572_5783	-0.927469086	-14.8211	0.205024	0.373928
SCE1572_159	-0.853475296	-12.4762	0.205252	0.374121
SCE1572_606	-0.848922469	-12.5693	0.205366	0.374121
SCE1572_9406	-1.271423487	-16.993	0.205414	0.374121
SCE1572_9280	1.264629413	-17.1035	0.205414	0.374121
SCE1572_1335	1.264629413	-17.1035	0.205414	0.374121
SCE1572_6196	1.264629413	-17.1035	0.205414	0.374121
SCE1572_1225	1.264629413	-17.1035	0.205414	0.374121
SCE1572_5676	1.264629413	-17.1035	0.205414	0.374121
SCE1572_5717	-0.858199558	-12.8503	0.205573	0.374279
SCE1572_6500	-1.008389081	-15.6551	0.205609	0.374279
SCE1572_2736	-0.968860717	-15.6353	0.205609	0.374279
SCE1572_4758	0.857501052	-12.7835	0.205643	0.374279
SCE1572_5949	0.876006877	-13.316	0.205795	0.374491
SCE1572_6180	0.880064266	-13.4885	0.206054	0.374831
SCE1572_70	0.880064266	-13.4885	0.206054	0.374831
SCE1572_1930	-0.861547693	-13.3593	0.206132	0.374908
SCE1572_5146	1.102034908	-16.0473	0.206388	0.375028
SCE1572_1465	1.102034908	-16.0473	0.206388	0.375028
SCE1572_4507	1.102034908	-16.0473	0.206388	0.375028
SCE1572_10840	-1.049031066	-15.9973	0.206388	0.375028
SCE1572_5427	-0.998404993	-15.972	0.206388	0.375028
SCE1572_5570	0.841288735	-11.4933	0.206412	0.375028
SCE1572_1927	-0.911247923	-14.4473	0.206468	0.375065
SCE1572_4466	0.991610919	-15.24	0.206639	0.375146
SCE1572_8869	0.976504026	-15.2476	0.206639	0.375146
SCE1572_8000	1.154841268	-16.4804	0.206864	0.375146
SCE1572_7134	1.154841268	-16.4804	0.206864	0.375146
SCE1572_4625	1.154841268	-16.4804	0.206864	0.375146
SCE1572_1059	1.154841268	-16.4804	0.206864	0.375146
SCE1572_8510	1.154841268	-16.4804	0.206864	0.375146
SCE1572_4535	1.154841268	-16.4804	0.206864	0.375146
SCE1572_10710	1.130479985	-16.4925	0.206864	0.375146
SCE1572_4360	-0.868664318	-13.6095	0.206869	0.375146
SCE1572_926	0.947000032	-14.8838	0.207044	0.375399
SCE1572_2435	-0.916758606	-14.5854	0.207221	0.375656
SCE1572_11107	-0.867033232	-13.8189	0.207345	0.375752
SCE1572_7360	-0.876122206	-13.9723	0.207346	0.375752
SCE1572_8130	0.998665676	-15.471	0.207703	0.376076
SCE1572_1487	0.998665676	-15.471	0.207703	0.376076

SCE1572_9960	0.980963675	-15.4798	0.207703	0.376076
SCE1572_3467	0.980963675	-15.4798	0.207703	0.376076
SCE1572_4885	0.980963675	-15.4798	0.207703	0.376076
SCE1572_2424	-0.846708074	-12.4588	0.207816	0.376173
SCE1572_9469	-0.856385988	-12.9703	0.207851	0.376173
SCE1572_9381	-0.88999438	-14.2548	0.208035	0.376173
SCE1572_1397	-0.955921661	-15.0439	0.208043	0.376173
SCE1572_5681	0.946481543	-15.061	0.208043	0.376173
SCE1572_5671	0.932935011	-15.0677	0.208043	0.376173
SCE1572_4994	0.932935011	-15.0677	0.208043	0.376173
SCE1572_8285	-0.930386569	-15.0311	0.208043	0.376173
SCE1572_3220	-0.879106064	-14.1754	0.208685	0.377206
SCE1572_11315	-0.832066309	-10.9231	0.208876	0.377206
SCE1572_7869	-0.852047052	-13.1088	0.208931	0.377206
SCE1572_8814	1.015246478	-15.7428	0.208992	0.377206
SCE1572_5523	1.008684432	-15.7461	0.208992	0.377206
SCE1572_6140	1.008684432	-15.7461	0.208992	0.377206
SCE1572_2946	1.008684432	-15.7461	0.208992	0.377206
SCE1572_9397	-1.000121465	-15.6999	0.208992	0.377206
SCE1572_446	0.987310781	-15.7568	0.208992	0.377206
SCE1572_4263	0.934251539	-14.6365	0.209009	0.377206
SCE1572_9054	0.924127322	-14.6415	0.209009	0.377206
SCE1572_9061	0.955085043	-14.9364	0.209174	0.377438
SCE1572_7538	0.902059334	-14.1149	0.209265	0.377538
SCE1572_4504	0.878323632	-14.0337	0.209786	0.378413
SCE1572_11552	0.898501514	-14.2162	0.210081	0.378725
SCE1572_4796	0.932448846	-14.7842	0.210103	0.378725
SCE1572_5850	0.932448846	-14.7842	0.210103	0.378725
SCE1572_7197	0.921221591	-14.7898	0.210103	0.378725
SCE1572_10276	0.839246745	-12.1068	0.21031	0.378991
SCE1572_411	0.871940688	-13.5557	0.210439	0.378991
SCE1572_8029	-0.888094848	-14.3933	0.210439	0.378991
SCE1572_3788	-0.888094848	-14.3933	0.210439	0.378991
SCE1572_280	0.955645421	-15.1205	0.210466	0.378991
SCE1572_11545	0.955645421	-15.1205	0.210466	0.378991
SCE1572_7120	0.883299108	-13.8882	0.210683	0.379162
SCE1572_2188	-0.863765518	-13.8749	0.210683	0.379162
SCE1572_2268	1.032357261	-15.5416	0.210778	0.379162
SCE1572_1510	1.032357261	-15.5416	0.210778	0.379162
SCE1572_8557	1.032357261	-15.5416	0.210778	0.379162
SCE1572_7186	1.013978732	-15.5508	0.210778	0.379162
SCE1572_7740	-0.891392438	-14.3136	0.211018	0.379529
SCE1572_133	-0.839647102	-12.5469	0.211742	0.380333
SCE1572_1752	1.429016231	-17.3433	0.21179	0.380333
SCE1572_9151	1.429016231	-17.3433	0.21179	0.380333
SCE1572_6675	1.429016231	-17.3433	0.21179	0.380333
SCE1572_2382	1.429016231	-17.3433	0.21179	0.380333
SCE1572_7180	1.429016231	-17.3433	0.21179	0.380333

SCE1572_7792	1.429016231	-17.3433	0.21179	0.380333
SCE1572_8506	1.429016231	-17.3433	0.21179	0.380333
SCE1572_5456	1.429016231	-17.3433	0.21179	0.380333
SCE1572_6614	0.883854738	-14.1564	0.211978	0.380439
SCE1572_11367	1.243149686	-16.6288	0.21203	0.380439
SCE1572_10004	1.243149686	-16.6288	0.21203	0.380439
SCE1572_11131	1.243149686	-16.6288	0.21203	0.380439
SCE1572_5576	1.243149686	-16.6288	0.21203	0.380439
SCE1572_3130	0.972502096	-15.3236	0.212069	0.380444
SCE1572_10194	0.939473295	-14.8332	0.212197	0.380608
SCE1572_9390	-0.886759637	-14.3312	0.212594	0.381162
SCE1572_7878	1.050504608	-15.8321	0.212687	0.381162
SCE1572_8517	1.050504608	-15.8321	0.212687	0.381162
SCE1572_4861	1.028136795	-15.8433	0.212687	0.381162
SCE1572_9677	1.028136795	-15.8433	0.212687	0.381162
SCE1572_8958	-0.86035489	-13.9802	0.212743	0.381197
SCE1572_2932	0.878819149	-14.2608	0.213024	0.381538
SCE1572_6796	-0.935820455	-15.1334	0.213042	0.381538
SCE1572_5112	-0.908853408	-15.1199	0.213042	0.381538
SCE1572_11388	-0.834881148	-12.3306	0.213256	0.381812
SCE1572_593	-0.851205749	-13.3644	0.213267	0.381812
SCE1572_2957	0.831969662	-11.6818	0.213751	0.382571
SCE1572_1096	-0.919121743	-14.9627	0.213798	0.382571
SCE1572_8206	-0.889552852	-14.4582	0.213837	0.382571
SCE1572_3892	-0.889552852	-14.4582	0.213837	0.382571
SCE1572_7940	-0.827553306	-12.1406	0.214405	0.383522
SCE1572_3998	-0.867357106	-13.8621	0.214561	0.383736
SCE1572_5213	0.925877953	-14.7369	0.214893	0.383845
SCE1572_10255	0.925877953	-14.7369	0.214893	0.383845
SCE1572_9452	0.857160719	-13.7466	0.214988	0.383845
SCE1572_3679	1.001595007	-15.3871	0.215077	0.383845
SCE1572_312	0.984916266	-15.3954	0.215077	0.383845
SCE1572_5121	0.984916266	-15.3954	0.215077	0.383845
SCE1572_11234	1.07907376	-16.1963	0.215096	0.383845
SCE1572_5344	1.07907376	-16.1963	0.215096	0.383845
SCE1572_4325	1.07907376	-16.1963	0.215096	0.383845
SCE1572_3257	1.07907376	-16.1963	0.215096	0.383845
SCE1572_3115	1.050504608	-16.2106	0.215096	0.383845
SCE1572_7499	1.050504608	-16.2106	0.215096	0.383845
SCE1572_4508	-1.030415388	-16.1136	0.215096	0.383845
SCE1572_2540	0.898501514	-14.6085	0.2153	0.384078
SCE1572_6392	-0.874533334	-14.5882	0.2153	0.384078
SCE1572_4626	-0.832440292	-12.5731	0.215341	0.384086
SCE1572_1434	0.838330365	-12.5511	0.215417	0.384157
SCE1572_6185	0.907900212	-14.5164	0.215643	0.384429
SCE1572_4844	-0.884400364	-14.4776	0.215643	0.384429
SCE1572_8230	-0.928535774	-15.1645	0.215784	0.384561
SCE1572_7487	0.871020778	-13.8943	0.21579	0.384561

SCE1572_3491	-0.88096401	-14.2888	0.216187	0.385202
SCE1572_4767	-0.831165998	-12.5605	0.216441	0.38559
SCE1572_8850	-1.017666895	-15.8662	0.216737	0.385988
SCE1572_9443	-0.971863205	-15.8433	0.216737	0.385988
SCE1572_6210	0.835875205	-12.6594	0.21681	0.386052
SCE1572_5396	1.011510476	-15.6451	0.217703	0.387324
SCE1572_2619	1.011510476	-15.6451	0.217703	0.387324
SCE1572_2384	-0.883152897	-14.4119	0.217706	0.387324
SCE1572_1654	0.877802522	-14.3335	0.217858	0.387324
SCE1572_6855	-1.183960646	-16.6498	0.21793	0.387324
SCE1572_6289	-1.183960646	-16.6498	0.21793	0.387324
SCE1572_9552	1.130674957	-16.6851	0.21793	0.387324
SCE1572_11033	1.130674957	-16.6851	0.21793	0.387324
SCE1572_829	1.130674957	-16.6851	0.21793	0.387324
SCE1572_3822	-1.101498486	-16.6085	0.21793	0.387324
SCE1572_1157	1.091146592	-16.7048	0.21793	0.387324
SCE1572_2558	0.96123727	-15.2552	0.218709	0.388579
SCE1572_10607	-0.92092624	-15.1963	0.218709	0.388579
SCE1572_6034	-0.840532037	-12.9782	0.218976	0.388987
SCE1572_4727	0.942701318	-14.9426	0.219176	0.389063
SCE1572_4205	-0.908853408	-14.8975	0.219176	0.389063
SCE1572_3748	-0.821257764	-12.3187	0.219177	0.389063
SCE1572_11295	-1.271423487	-17.3716	0.219277	0.389063
SCE1572_1836	-1.271423487	-17.3716	0.219277	0.389063
SCE1572_131	-1.271423487	-17.3716	0.219277	0.389063
SCE1572_1160	0.909906278	-14.7955	0.219315	0.389063
SCE1572_5713	0.909906278	-14.7955	0.219315	0.389063
SCE1572_10965	0.903350914	-14.5617	0.219493	0.389313
SCE1572_3862	-0.894569182	-14.439	0.21955	0.389348
SCE1572_27	-0.818286198	-11.9952	0.219606	0.389376
SCE1572_10820	-0.873459501	-14.0851	0.21967	0.389376
SCE1572_9730	-0.860228054	-13.9333	0.219695	0.389376
SCE1572_4300	0.851558013	-13.7494	0.219713	0.389376
SCE1572_4879	-0.822383474	-12.221	0.220052	0.389497
SCE1572_10216	1.143614012	-16.316	0.220226	0.389497
SCE1572_8845	1.143614012	-16.316	0.220226	0.389497
SCE1572_5587	1.143614012	-16.316	0.220226	0.389497
SCE1572_11235	1.143614012	-16.316	0.220226	0.389497
SCE1572_7529	1.143614012	-16.316	0.220226	0.389497
SCE1572_9239	1.143614012	-16.316	0.220226	0.389497
SCE1572_7966	1.143614012	-16.316	0.220226	0.389497
SCE1572_520	1.113240363	-16.3312	0.220226	0.389497
SCE1572_6445	1.113240363	-16.3312	0.220226	0.389497
SCE1572_3104	1.113240363	-16.3312	0.220226	0.389497
SCE1572_4898	1.113240363	-16.3312	0.220226	0.389497
SCE1572_2656	0.859280205	-13.7702	0.220922	0.390493
SCE1572_9586	-0.860109074	-13.8879	0.221022	0.390493
SCE1572_160	0.858637054	-13.9005	0.221022	0.390493

SCE1572_8397	0.85785953	-14.0439	0.221133	0.390493
SCE1572_2648	1.026257062	-15.9597	0.221198	0.390493
SCE1572_1952	1.026257062	-15.9597	0.221198	0.390493
SCE1572_5816	1.026257062	-15.9597	0.221198	0.390493
SCE1572_11121	1.026257062	-15.9597	0.221198	0.390493
SCE1572_4490	1.001595007	-15.972	0.221198	0.390493
SCE1572_9508	1.001595007	-15.972	0.221198	0.390493
SCE1572_2727	1.001595007	-15.972	0.221198	0.390493
SCE1572_4726	0.893914245	-14.4815	0.221471	0.39091
SCE1572_3990	-0.819406532	-12.4452	0.221647	0.391129
SCE1572_10397	-0.905774015	-14.8102	0.221699	0.391129
SCE1572_74	-0.932621574	-15.4366	0.221791	0.391129
SCE1572_4713	0.941570236	-15.1276	0.221818	0.391129
SCE1572_5963	0.941570236	-15.1276	0.221818	0.391129
SCE1572_867	-0.916328528	-15.0897	0.221818	0.391129
SCE1572_6135	-0.870325179	-14.2075	0.222922	0.393009
SCE1572_8457	-0.820921307	-12.6803	0.223193	0.393421
SCE1572_2484	-0.879106064	-14.4973	0.223474	0.393851
SCE1572_2034	-0.815216799	-12.3106	0.223752	0.394275
SCE1572_10845	0.904018519	-14.7478	0.223941	0.394449
SCE1572_3972	-0.882858199	-14.7178	0.223941	0.394449
SCE1572_2638	0.862059518	-14.012	0.223964	0.394449
SCE1572_9492	0.869057736	-14.1638	0.224397	0.39508
SCE1572_7387	-0.856385988	-14.1461	0.224397	0.39508
SCE1572_11132	1.231076853	-16.8573	0.224728	0.395398
SCE1572_8931	1.231076853	-16.8573	0.224728	0.395398
SCE1572_3894	1.188008132	-16.8788	0.224728	0.395398
SCE1572_8709	1.188008132	-16.8788	0.224728	0.395398
SCE1572_8867	0.940080618	-15.3398	0.225187	0.396139
SCE1572_3713	-0.82396451	-12.9229	0.225248	0.39618
SCE1572_11226	0.995363054	-15.5601	0.225552	0.396649
SCE1572_4526	0.96561571	-15.7676	0.225898	0.39719
SCE1572_6980	1.076499816	-16.0601	0.226132	0.39727
SCE1572_2555	1.050504608	-16.0731	0.226132	0.39727
SCE1572_11210	1.050504608	-16.0731	0.226132	0.39727
SCE1572_1770	1.050504608	-16.0731	0.226132	0.39727
SCE1572_5445	1.050504608	-16.0731	0.226132	0.39727
SCE1572_10736	-0.897027972	-14.7514	0.226375	0.397468
SCE1572_8526	-0.87656387	-14.7412	0.226375	0.397468
SCE1572_1934	-0.87656387	-14.7412	0.226375	0.397468
SCE1572_8364	0.862877605	-14.2688	0.226434	0.397468
SCE1572_1008	0.862877605	-14.2688	0.226434	0.397468
SCE1572_7989	-0.818372662	-12.7805	0.226646	0.397774
SCE1572_2582	0.881528436	-14.3691	0.226824	0.398019
SCE1572_7445	0.910779844	-14.9019	0.226861	0.398019
SCE1572_650	-0.796600949	-9.97915	0.227413	0.39886
SCE1572_1569	-0.905295588	-15.0186	0.227416	0.39886
SCE1572_3172	0.814772165	-12.5843	0.227945	0.399029

SCE1572_11180	1.465542107	-17.74	0.228158	0.399029
SCE1572_556	1.465542107	-17.74	0.228158	0.399029
SCE1572_7347	1.465542107	-17.74	0.228158	0.399029
SCE1572_3865	1.465542107	-17.74	0.228158	0.399029
SCE1572_3102	-1.441348488	-17.6085	0.228158	0.399029
SCE1572_3294	1.391541526	-17.777	0.228158	0.399029
SCE1572_6488	1.391541526	-17.777	0.228158	0.399029
SCE1572_1571	1.391541526	-17.777	0.228158	0.399029
SCE1572_5626	1.391541526	-17.777	0.228158	0.399029
SCE1572_5502	1.391541526	-17.777	0.228158	0.399029
SCE1572_4331	1.391541526	-17.777	0.228158	0.399029
SCE1572_11000	1.391541526	-17.777	0.228158	0.399029
SCE1572_9005	1.391541526	-17.777	0.228158	0.399029
SCE1572_3893	1.391541526	-17.777	0.228158	0.399029
SCE1572_10611	-1.271423487	-17.5236	0.228158	0.399029
SCE1572_233	-1.271423487	-17.5236	0.228158	0.399029
SCE1572_7231	-0.851520233	-14.2741	0.228288	0.39915
SCE1572_4498	-0.856385988	-14.6531	0.228303	0.39915
SCE1572_11364	0.816039354	-12.5623	0.228382	0.399222
SCE1572_5312	0.860488188	-14.0729	0.228647	0.399618
SCE1572_8216	0.968042448	-15.4038	0.228785	0.399691
SCE1572_5397	0.968042448	-15.4038	0.228785	0.399691
SCE1572_7138	-0.856385988	-14.3774	0.228802	0.399691
SCE1572_9197	0.866080037	-14.4955	0.229379	0.400633
SCE1572_8415	0.917610337	-14.9551	0.22966	0.401057
SCE1572_6599	-0.856385988	-14.5791	0.230032	0.401639
SCE1572_11389	-0.792579016	-10.3947	0.230358	0.402142
SCE1572_11584	0.905454275	-15.0815	0.230503	0.402203
SCE1572_5500	0.802396746	-12.0127	0.230526	0.402203
SCE1572_4989	-0.98191687	-15.7938	0.230584	0.402203
SCE1572_4288	-0.944374907	-15.7751	0.230584	0.402203
SCE1572_4620	-0.938848148	-15.7723	0.230584	0.402203
SCE1572_11357	0.930210374	-15.2707	0.23149	0.403448
SCE1572_10367	0.930210374	-15.2707	0.23149	0.403448
SCE1572_8910	0.930210374	-15.2707	0.23149	0.403448
SCE1572_4790	-0.912969516	-15.2289	0.23149	0.403448
SCE1572_5049	-0.884400364	-15.2146	0.23149	0.403448
SCE1572_1535	-0.975967604	-16.0863	0.23162	0.403608
SCE1572_833	-0.83648643	-13.7211	0.231735	0.403742
SCE1572_4645	-0.818250859	-13.0746	0.23217	0.404433
SCE1572_1467	-0.796821434	-12.0299	0.23224	0.404488
SCE1572_7567	1.120893936	-16.4973	0.232558	0.404757
SCE1572_393	1.120893936	-16.4973	0.232558	0.404757
SCE1572_8089	1.086128518	-16.5147	0.232558	0.404757
SCE1572_2522	-0.895275001	-14.9508	0.232621	0.404757
SCE1572_9790	-0.821030711	-13.4349	0.232642	0.404757
SCE1572_2361	-0.821030711	-13.4349	0.232642	0.404757
SCE1572_9689	-0.816341269	-13.2275	0.232683	0.404757

SCE1572_6194	-0.847283781	-14.1776	0.232703	0.404757
SCE1572_9266	-0.834690917	-13.8458	0.23281	0.404877
SCE1572_10908	-0.844002263	-14.0368	0.233097	0.405309
SCE1572_1166	0.892963331	-14.7533	0.233372	0.40572
SCE1572_9735	-0.81037359	-13.1231	0.233437	0.405767
SCE1572_3229	0.832324438	-13.8087	0.233693	0.406072
SCE1572_4065	0.927356377	-15.1347	0.233786	0.406072
SCE1572_2629	0.916891112	-15.1399	0.233786	0.406072
SCE1572_10549	0.914910328	-15.1376	0.233786	0.406072
SCE1572_6080	0.851433261	-14.1402	0.233806	0.406072
SCE1572_10144	0.971433037	-15.6652	0.234057	0.406165
SCE1572_489	0.966746656	-15.6608	0.234057	0.406165
SCE1572_2742	-0.930386569	-15.6161	0.234057	0.406165
SCE1572_8848	0.839607825	-13.9941	0.234091	0.406165
SCE1572_4276	0.839607825	-13.9941	0.234091	0.406165
SCE1572_5357	0.832913173	-13.9975	0.234091	0.406165
SCE1572_11486	-0.846117652	-14.3516	0.234293	0.406381
SCE1572_9495	-0.846117652	-14.3516	0.234293	0.406381
SCE1572_7851	-0.862617941	-14.6311	0.234924	0.407409
SCE1572_3158	0.829036695	-13.9428	0.235019	0.407506
SCE1572_2509	-0.904641156	-15.2622	0.23517	0.407634
SCE1572_3363	-0.904641156	-15.2622	0.23517	0.407634
SCE1572_6866	0.85785953	-14.3069	0.23547	0.408087
SCE1572_1990	0.898501514	-15.0236	0.235759	0.408453
SCE1572_9158	0.898501514	-15.0236	0.235759	0.408453
SCE1572_6361	0.836379802	-13.7816	0.235974	0.408757
SCE1572_998	-0.862310822	-14.5582	0.236316	0.409283
SCE1572_4908	-0.82162057	-13.5261	0.23676	0.409983
SCE1572_2892	0.926515891	-15.5071	0.236841	0.409988
SCE1572_4158	0.926515891	-15.5071	0.236841	0.409988
SCE1572_10569	-0.793060052	-12.5839	0.237131	0.410424
SCE1572_7215	-0.886133331	-14.8861	0.237473	0.410766
SCE1572_3509	0.827204546	-13.9163	0.237493	0.410766
SCE1572_11050	-0.841435646	-14.3086	0.237566	0.410766
SCE1572_2613	1.021358262	-16.2252	0.237758	0.410766
SCE1572_7642	1.021358262	-16.2252	0.237758	0.410766
SCE1572_9678	1.021358262	-16.2252	0.237758	0.410766
SCE1572_10165	1.021358262	-16.2252	0.237758	0.410766
SCE1572_7297	1.021358262	-16.2252	0.237758	0.410766
SCE1572_8192	1.021358262	-16.2252	0.237758	0.410766
SCE1572_11103	-0.963301192	-16.1471	0.237758	0.410766
SCE1572_9401	-0.963301192	-16.1471	0.237758	0.410766
SCE1572_1193	-0.809317734	-13.8477	0.238268	0.410929
SCE1572_3394	-0.826283956	-14.0959	0.238467	0.410929
SCE1572_6881	0.812640778	-13.4226	0.238535	0.410929
SCE1572_2165	0.850040025	-14.4233	0.238708	0.410929
SCE1572_7577	1.816039354	-18.1498	0.23879	0.410929
SCE1572_7555	1.816039354	-18.1498	0.23879	0.410929

SCE1572_1827	1.816039354	-18.1498	0.23879	0.410929
SCE1572_5843	1.816039354	-18.1498	0.23879	0.410929
SCE1572_3536	1.816039354	-18.1498	0.23879	0.410929
SCE1572_2496	1.816039354	-18.1498	0.23879	0.410929
SCE1572_10233	1.816039354	-18.1498	0.23879	0.410929
SCE1572_766	1.816039354	-18.1498	0.23879	0.410929
SCE1572_7419	1.816039354	-18.1498	0.23879	0.410929
SCE1572_3620	1.816039354	-18.1498	0.23879	0.410929
SCE1572_5982	1.728576513	-18.1935	0.23879	0.410929
SCE1572_7005	1.728576513	-18.1935	0.23879	0.410929
SCE1572_10192	1.728576513	-18.1935	0.23879	0.410929
SCE1572_382	1.728576513	-18.1935	0.23879	0.410929
SCE1572_503	1.728576513	-18.1935	0.23879	0.410929
SCE1572_3554	1.728576513	-18.1935	0.23879	0.410929
SCE1572_7761	1.728576513	-18.1935	0.23879	0.410929
SCE1572_7248	1.728576513	-18.1935	0.23879	0.410929
SCE1572_3124	1.728576513	-18.1935	0.23879	0.410929
SCE1572_1318	1.728576513	-18.1935	0.23879	0.410929
SCE1572_9438	-0.959479481	-15.6795	0.238889	0.411031
SCE1572_6268	0.863736096	-14.8186	0.238976	0.411115
SCE1572_960	-0.872874111	-15.0348	0.239089	0.411241
SCE1572_267	0.803627218	-12.9695	0.239395	0.4117
SCE1572_5719	1.20662381	-16.6471	0.240055	0.41243
SCE1572_9010	1.20662381	-16.6471	0.240055	0.41243
SCE1572_9723	1.20662381	-16.6471	0.240055	0.41243
SCE1572_3074	1.20662381	-16.6471	0.240055	0.41243
SCE1572_1895	1.20662381	-16.6471	0.240055	0.41243
SCE1572_7565	1.169149104	-16.6658	0.240055	0.41243
SCE1572_6786	-0.81641958	-13.9903	0.240113	0.412463
SCE1572_2853	0.855108919	-14.7233	0.240305	0.412657
SCE1572_5177	0.855108919	-14.7233	0.240305	0.412657
SCE1572_7456	0.904899286	-14.9615	0.240647	0.413155
SCE1572_7625	-0.790688355	-12.6718	0.240724	0.413155
SCE1572_8020	0.86243226	-14.4566	0.241055	0.413155
SCE1572_5793	-0.795443224	-12.8334	0.241183	0.413155
SCE1572_8818	0.976504026	-15.5695	0.241368	0.413155
SCE1572_1776	0.976504026	-15.5695	0.241368	0.413155
SCE1572_7155	0.976504026	-15.5695	0.241368	0.413155
SCE1572_2563	0.976504026	-15.5695	0.241368	0.413155
SCE1572_4108	0.967936363	-15.5738	0.241368	0.413155
SCE1572_10836	0.957395203	-15.5791	0.241368	0.413155
SCE1572_6219	0.957395203	-15.5791	0.241368	0.413155
SCE1572_5946	0.957395203	-15.5791	0.241368	0.413155
SCE1572_1628	-0.82396451	-14.0103	0.241919	0.413155
SCE1572_923	-0.804297477	-13.3725	0.241934	0.413155
SCE1572_8053	1.21400334	-17.1288	0.241983	0.413155
SCE1572_4853	1.21400334	-17.1288	0.241983	0.413155
SCE1572_3001	1.21400334	-17.1288	0.241983	0.413155

SCE1572_3311	1.21400334	-17.1288	0.241983	0.413155
SCE1572_3373	1.21400334	-17.1288	0.241983	0.413155
SCE1572_7030	1.16153592	-17.1551	0.241983	0.413155
SCE1572_9851	1.16153592	-17.1551	0.241983	0.413155
SCE1572_11245	1.16153592	-17.1551	0.241983	0.413155
SCE1572_5313	1.16153592	-17.1551	0.241983	0.413155
SCE1572_3582	1.16153592	-17.1551	0.241983	0.413155
SCE1572_11508	1.16153592	-17.1551	0.241983	0.413155
SCE1572_10168	1.16153592	-17.1551	0.241983	0.413155
SCE1572_4327	1.16153592	-17.1551	0.241983	0.413155
SCE1572_10739	1.16153592	-17.1551	0.241983	0.413155
SCE1572_5296	1.16153592	-17.1551	0.241983	0.413155
SCE1572_4581	1.16153592	-17.1551	0.241983	0.413155
SCE1572_7713	1.16153592	-17.1551	0.241983	0.413155
SCE1572_1301	0.892552248	-14.8566	0.242006	0.413155
SCE1572_2546	0.880579606	-14.8626	0.242006	0.413155
SCE1572_1494	0.880579606	-14.8626	0.242006	0.413155
SCE1572_900	0.846971214	-14.3864	0.242009	0.413155
SCE1572_4960	-0.825359092	-14.3619	0.242009	0.413155
SCE1572_3852	-0.811991868	-14.1418	0.242359	0.413686
SCE1572_3364	-0.89033332	-15.0767	0.24263	0.414013
SCE1572_8902	0.877439899	-15.0955	0.24263	0.414013
SCE1572_9770	0.849591914	-14.3111	0.242879	0.414304
SCE1572_9893	0.841276646	-14.3152	0.242879	0.414304
SCE1572_2283	0.950968934	-15.4124	0.24337	0.41494
SCE1572_4756	0.933690943	-15.421	0.24337	0.41494
SCE1572_5362	-0.877144548	-15.3671	0.24337	0.41494
SCE1572_901	0.943589404	-15.7786	0.244179	0.415982
SCE1572_1534	0.943589404	-15.7786	0.244179	0.415982
SCE1572_54	0.943589404	-15.7786	0.244179	0.415982
SCE1572_11248	0.921221591	-15.7898	0.244179	0.415982
SCE1572_6221	0.921221591	-15.7898	0.244179	0.415982
SCE1572_2468	-0.837526961	-14.6437	0.24426	0.416054
SCE1572_4608	-0.811991868	-13.6564	0.244409	0.416125
SCE1572_11130	0.808575835	-13.8206	0.244419	0.416125
SCE1572_4493	-0.820762078	-13.9763	0.244421	0.416125
SCE1572_1094	1.082213468	-16.3467	0.244669	0.416278
SCE1572_1787	1.082213468	-16.3467	0.244669	0.416278
SCE1572_1179	1.082213468	-16.3467	0.244669	0.416278
SCE1572_4635	1.082213468	-16.3467	0.244669	0.416278
SCE1572_4330	0.854107395	-14.5863	0.245206	0.417056
SCE1572_6711	-0.820762078	-14.5613	0.245206	0.417056
SCE1572_11462	-0.790797646	-13.4419	0.245248	0.417061
SCE1572_4185	-0.779570391	-12.8626	0.246635	0.419352
SCE1572_8564	-0.779570391	-12.6402	0.24703	0.419955
SCE1572_2703	0.829180454	-14.1513	0.247139	0.420001
SCE1572_3630	-0.816857624	-14.1264	0.247139	0.420001
SCE1572_10737	-0.79460979	-13.5482	0.247177	0.420001

SCE1572_9485	-0.856385988	-14.9941	0.247574	0.420607
SCE1572_5688	1.024032396	-16.0863	0.247778	0.420614
SCE1572_5532	1.024032396	-16.0863	0.247778	0.420614
SCE1572_9035	1.024032396	-16.0863	0.247778	0.420614
SCE1572_11576	-0.987630521	-16.028	0.247778	0.420614
SCE1572_11327	-0.937004448	-16.0027	0.247778	0.420614
SCE1572_11331	-0.89945471	-15.42	0.247954	0.420789
SCE1572_10570	-0.78599666	-13.1747	0.247961	0.420789
SCE1572_7935	0.873626846	-14.9205	0.248583	0.421538
SCE1572_1536	0.861026809	-14.9268	0.248583	0.421538
SCE1572_9214	0.817702403	-13.9765	0.248702	0.421538
SCE1572_5218	1.050504608	-16.7252	0.248713	0.421538
SCE1572_4716	1.050504608	-16.7252	0.248713	0.421538
SCE1572_2902	1.050504608	-16.7252	0.248713	0.421538
SCE1572_11531	1.050504608	-16.7252	0.248713	0.421538
SCE1572_6161	0.860282192	-14.5402	0.248722	0.421538
SCE1572_1387	0.79405035	-13.5315	0.248962	0.421876
SCE1572_2243	0.81180594	-13.924	0.249151	0.422129
SCE1572_3563	0.783759428	-12.8977	0.249374	0.422315
SCE1572_3116	0.85327226	-14.4612	0.249382	0.422315
SCE1572_2125	0.85327226	-14.4612	0.249382	0.422315
SCE1572_5223	-0.771138701	-12.0129	0.249918	0.423011
SCE1572_306	0.982333105	-15.8662	0.249994	0.423011
SCE1572_5442	0.958874132	-15.8779	0.249994	0.423011
SCE1572_2717	0.958874132	-15.8779	0.249994	0.423011
SCE1572_628	-0.927469086	-15.8211	0.249994	0.423011
SCE1572_10287	-0.759729946	-10.4675	0.250195	0.423242
SCE1572_9286	-0.802870478	-14.0329	0.250211	0.423242
SCE1572_7917	-0.764935044	-12.0309	0.25033	0.423376
SCE1572_2240	-0.826638644	-14.3012	0.25052	0.423492
SCE1572_6265	-0.811991868	-14.2938	0.25052	0.423492
SCE1572_4750	-0.811991868	-14.2938	0.25052	0.423492
SCE1572_6567	-0.825623734	-14.6126	0.250907	0.424078
SCE1572_4939	-0.787389895	-13.242	0.251183	0.424477
SCE1572_6571	1.814950938	-18.5088	0.251568	0.42505
SCE1572_11510	0.950968934	-15.6754	0.251644	0.42505
SCE1572_9076	0.950968934	-15.6754	0.251644	0.42505
SCE1572_2832	-0.828479991	-14.472	0.252053	0.425672
SCE1572_1819	0.87913619	-14.9744	0.25216	0.425785
SCE1572_3702	0.804679492	-13.7485	0.252352	0.425973
SCE1572_6336	0.798965841	-13.7514	0.252352	0.425973
SCE1572_5622	-0.800117768	-13.9984	0.252625	0.426365
SCE1572_9190	0.907900212	-15.5164	0.252913	0.426436
SCE1572_1481	0.907900212	-15.5164	0.252913	0.426436
SCE1572_717	1.372432703	-17.3716	0.253153	0.426436
SCE1572_3073	1.372432703	-17.3716	0.253153	0.426436
SCE1572_7756	1.372432703	-17.3716	0.253153	0.426436
SCE1572_6456	1.372432703	-17.3716	0.253153	0.426436

SCE1572_10798	1.313539014	-17.401	0.253153	0.426436
SCE1572_1191	1.313539014	-17.401	0.253153	0.426436
SCE1572_2605	1.313539014	-17.401	0.253153	0.426436
SCE1572_5256	1.313539014	-17.401	0.253153	0.426436
SCE1572_1223	-1.271423487	-17.2341	0.253153	0.426436
SCE1572_8407	-1.145892605	-17.1713	0.253153	0.426436
SCE1572_10188	-0.808451511	-14.2525	0.253383	0.426755
SCE1572_1461	0.833693219	-14.2147	0.25375	0.427304
SCE1572_509	0.890039936	-15.3648	0.253916	0.42731
SCE1572_1171	0.890039936	-15.3648	0.253916	0.42731
SCE1572_1593	-0.886759637	-15.3312	0.253916	0.42731
SCE1572_10486	-0.856385988	-15.316	0.253916	0.42731
SCE1572_10964	0.840469393	-14.6833	0.253967	0.427327
SCE1572_530	0.807353626	-14.0394	0.254682	0.428332
SCE1572_10885	0.875417901	-15.2278	0.254727	0.428332
SCE1572_4613	0.859821046	-15.2355	0.254727	0.428332
SCE1572_5912	-0.856385988	-15.2006	0.254727	0.428332
SCE1572_2452	0.848870747	-15.1098	0.255394	0.429385
SCE1572_3819	0.818213725	-14.3633	0.255543	0.429429
SCE1572_8965	-0.810092335	-14.3543	0.255543	0.429429
SCE1572_2199	0.809496508	-14.3676	0.255543	0.429429
SCE1572_9428	-0.761348198	-12.4328	0.255688	0.429604
SCE1572_11527	0.839607825	-14.9941	0.255952	0.429979
SCE1572_11458	-0.789890576	-13.7248	0.256023	0.43003
SCE1572_9105	-0.804855687	-14.6788	0.25718	0.431905
SCE1572_3980	-0.796884976	-13.8864	0.257349	0.432119
SCE1572_9062	0.825438052	-14.4751	0.257995	0.433129
SCE1572_8552	-0.780437135	-13.6536	0.258032	0.433129
SCE1572_1273	0.829377154	-14.3952	0.258209	0.433355
SCE1572_4851	0.778699992	-13.0391	0.258258	0.433368
SCE1572_9001	-0.750591324	-11.5876	0.258385	0.433513
SCE1572_6979	1.143614012	-16.901	0.25882	0.433897
SCE1572_681	1.143614012	-16.901	0.25882	0.433897
SCE1572_4087	1.143614012	-16.901	0.25882	0.433897
SCE1572_11540	1.143614012	-16.901	0.25882	0.433897
SCE1572_1127	1.143614012	-16.901	0.25882	0.433897
SCE1572_2579	0.898501514	-15.4386	0.258884	0.433928
SCE1572_8528	-0.773083341	-13.2844	0.258921	0.433928
SCE1572_9117	0.794665703	-14.0755	0.259002	0.433995
SCE1572_6838	0.898501514	-15.2866	0.259338	0.434349
SCE1572_4741	0.882381849	-15.2947	0.259338	0.434349
SCE1572_3106	-0.875494811	-15.2476	0.259338	0.434349
SCE1572_7742	-0.775728324	-13.8018	0.259515	0.434577
SCE1572_9155	-0.78599666	-13.6694	0.259743	0.43489
SCE1572_7953	0.85785953	-15.0439	0.259979	0.435215
SCE1572_7370	0.848315757	-14.9332	0.26021	0.435463
SCE1572_2508	0.848315757	-14.9332	0.26021	0.435463
SCE1572_8610	0.828112186	-14.8364	0.2604	0.435712

SCE1572_6795	-0.768923147	-13.0499	0.260522	0.435847
SCE1572_199	-0.788822704	-14.2624	0.261123	0.436783
SCE1572_6442	0.814152245	-14.1588	0.261229	0.43689
SCE1572_7955	-0.783977492	-13.7773	0.261458	0.436892
SCE1572_2272	-0.783977492	-13.7773	0.261458	0.436892
SCE1572_11238	1.050504608	-16.5325	0.261464	0.436892
SCE1572_226	1.050504608	-16.5325	0.261464	0.436892
SCE1572_2140	1.050504608	-16.5325	0.261464	0.436892
SCE1572_9981	0.775119099	-13.7395	0.261479	0.436892
SCE1572_10420	-0.762632667	-12.9879	0.262816	0.438708
SCE1572_3562	0.991610919	-16.24	0.262816	0.438708
SCE1572_6345	0.991610919	-16.24	0.262816	0.438708
SCE1572_10996	0.96123727	-16.2552	0.262816	0.438708
SCE1572_10747	0.96123727	-16.2552	0.262816	0.438708
SCE1572_9182	0.96123727	-16.2552	0.262816	0.438708
SCE1572_5512	-0.765783439	-13.1008	0.262883	0.43871
SCE1572_1519	0.763064889	-13.1371	0.262901	0.43871
SCE1572_4369	-0.764463498	-13.3847	0.263071	0.438924
SCE1572_1647	-0.772691358	-13.5322	0.263177	0.439031
SCE1572_841	-0.773396623	-13.6116	0.263236	0.439061
SCE1572_631	-0.7542878	-12.6211	0.263492	0.4391
SCE1572_1222	0.950968934	-15.9973	0.263542	0.4391
SCE1572_994	0.924973726	-16.0103	0.263542	0.4391
SCE1572_1351	-0.78599666	-14.0414	0.263575	0.4391
SCE1572_11450	-0.788641381	-14.1667	0.26367	0.4391
SCE1572_9888	0.786127536	-14.2384	0.26372	0.4391
SCE1572_2492	0.786127536	-14.2384	0.26372	0.4391
SCE1572_7283	0.898501514	-15.8012	0.263939	0.4391
SCE1572_3108	0.898501514	-15.8012	0.263939	0.4391
SCE1572_5400	0.898501514	-15.8012	0.263939	0.4391
SCE1572_5686	-0.897027972	-15.7514	0.263939	0.4391
SCE1572_1586	-0.793639817	-14.5734	0.263998	0.4391
SCE1572_7817	-0.892911864	-15.5973	0.26415	0.4391
SCE1572_9656	0.878323632	-15.6186	0.26415	0.4391
SCE1572_9551	0.878323632	-15.6186	0.26415	0.4391
SCE1572_28	-0.856385988	-15.5791	0.26415	0.4391
SCE1572_4335	0.85633206	-14.8747	0.26417	0.4391
SCE1572_8342	0.846763943	-14.8795	0.26417	0.4391
SCE1572_1271	0.84405373	-14.8809	0.26417	0.4391
SCE1572_5573	0.84405373	-14.8809	0.26417	0.4391
SCE1572_437	0.866080037	-14.9809	0.26422	0.4391
SCE1572_403	-0.81770552	-14.9431	0.26422	0.4391
SCE1572_11203	-0.856385988	-15.4416	0.264252	0.4391
SCE1572_6328	-0.839707247	-15.0514	0.264262	0.4391
SCE1572_2589	-0.770525251	-13.7151	0.265151	0.440508
SCE1572_11068	-0.766188179	-13.4867	0.266295	0.441492
SCE1572_677	0.795690709	-14.2676	0.266358	0.441492
SCE1572_706	2.188008132	-18.9638	0.266667	0.441492

SCE1572_5196	2.188008132	-18.9638	0.266667	0.441492
SCE1572_7345	2.188008132	-18.9638	0.266667	0.441492
SCE1572_7910	2.188008132	-18.9638	0.266667	0.441492
SCE1572_9957	2.188008132	-18.9638	0.266667	0.441492
SCE1572_8360	2.188008132	-18.9638	0.266667	0.441492
SCE1572_7923	2.188008132	-18.9638	0.266667	0.441492
SCE1572_8343	2.050504608	-19.0325	0.266667	0.441492
SCE1572_5174	2.050504608	-19.0325	0.266667	0.441492
SCE1572_594	2.050504608	-19.0325	0.266667	0.441492
SCE1572_243	2.050504608	-19.0325	0.266667	0.441492
SCE1572_7969	2.050504608	-19.0325	0.266667	0.441492
SCE1572_8174	2.050504608	-19.0325	0.266667	0.441492
SCE1572_9574	2.050504608	-19.0325	0.266667	0.441492
SCE1572_2583	2.050504608	-19.0325	0.266667	0.441492
SCE1572_10311	2.050504608	-19.0325	0.266667	0.441492
SCE1572_1259	2.050504608	-19.0325	0.266667	0.441492
SCE1572_6943	2.050504608	-19.0325	0.266667	0.441492
SCE1572_7362	2.050504608	-19.0325	0.266667	0.441492
SCE1572_1113	-1.593351582	-18.5325	0.266667	0.441492
SCE1572_5863	0.816039354	-14.4798	0.266904	0.441816
SCE1572_9403	0.773765287	-13.8895	0.267534	0.442788
SCE1572_10860	-0.744176484	-12.3424	0.267576	0.442788
SCE1572_10019	-0.783758188	-13.9261	0.267715	0.442949
SCE1572_2376	-0.750820232	-12.8558	0.267898	0.443182
SCE1572_3929	-0.769674355	-13.6224	0.268427	0.443988
SCE1572_11062	0.772970632	-13.6713	0.268609	0.444218
SCE1572_36	-0.791430546	-14.2056	0.268824	0.444504
SCE1572_8292	0.84405373	-15.2434	0.269208	0.445069
SCE1572_864	-0.74570019	-12.5726	0.269404	0.445323
SCE1572_3371	0.872966422	-15.3734	0.269623	0.445475
SCE1572_6315	0.855688431	-15.382	0.269623	0.445475
SCE1572_9305	-0.846117652	-15.3516	0.269623	0.445475
SCE1572_6844	-0.789271792	-14.4751	0.27006	0.446005
SCE1572_6356	0.889041185	-15.5258	0.270071	0.446005
SCE1572_9599	0.869932362	-15.5353	0.270071	0.446005
SCE1572_9517	-0.736950757	-12.449	0.270389	0.44646
SCE1572_11096	0.800985008	-14.5698	0.27044	0.446474
SCE1572_5130	-0.881476969	-15.6405	0.270547	0.446581
SCE1572_4780	-0.749814842	-13.0044	0.270836	0.446916
SCE1572_362	0.80539211	-14.6551	0.270856	0.446916
SCE1572_7299	0.737241374	-11.7292	0.270877	0.446916
SCE1572_3498	-0.763939739	-13.7673	0.27101	0.446964
SCE1572_305	0.93502739	-15.8898	0.271034	0.446964
SCE1572_3548	0.93502739	-15.8898	0.271034	0.446964
SCE1572_4847	-0.761228755	-13.809	0.271292	0.447291
SCE1572_7504	0.997065349	-16.0998	0.271493	0.447291
SCE1572_3895	-0.872874111	-16.0348	0.271493	0.447291
SCE1572_3823	-0.872874111	-16.0348	0.271493	0.447291

SCE1572_5052	0.820912562	-14.8405	0.271817	0.447291
SCE1572_2874	0.816039354	-14.8424	0.271817	0.447291
SCE1572_10829	1.050504608	-16.3626	0.271828	0.447291
SCE1572_4700	1.050504608	-16.3626	0.271828	0.447291
SCE1572_2154	1.050504608	-16.3626	0.271828	0.447291
SCE1572_174	1.01808313	-16.3788	0.271828	0.447291
SCE1572_9988	1.01808313	-16.3788	0.271828	0.447291
SCE1572_6655	1.01808313	-16.3788	0.271828	0.447291
SCE1572_8949	-0.9343885	-16.277	0.271828	0.447291
SCE1572_5792	-0.9343885	-16.277	0.271828	0.447291
SCE1572_1228	-0.826012339	-14.9162	0.272377	0.448054
SCE1572_3583	0.822552661	-14.9461	0.272377	0.448054
SCE1572_9204	-0.752694685	-13.7337	0.272741	0.448583
SCE1572_116	-0.78599666	-14.4284	0.272846	0.448686
SCE1572_8218	0.780238633	-14.0827	0.273009	0.448884
SCE1572_8609	0.782443385	-14.143	0.27343	0.449505
SCE1572_2183	0.854107395	-15.1713	0.273699	0.449808
SCE1572_4875	0.839000503	-15.1788	0.273699	0.449808
SCE1572_7692	-0.735587844	-12.5864	0.273861	0.44997
SCE1572_2599	0.776939535	-14.2099	0.273884	0.44997
SCE1572_4901	0.775443118	-13.9421	0.274202	0.450353
SCE1572_3761	0.768840383	-13.9454	0.274202	0.450353
SCE1572_7793	0.763064889	-13.722	0.274414	0.45063
SCE1572_8646	0.866080037	-15.3028	0.274487	0.45068
SCE1572_7591	0.77725036	-13.9967	0.274631	0.450846
SCE1572_1701	-0.739346567	-12.6927	0.274763	0.450993
SCE1572_11025	-0.74090877	-12.9364	0.274879	0.451112
SCE1572_2155	0.813110023	-14.7932	0.275231	0.451522
SCE1572_10099	-0.801938204	-14.7583	0.275231	0.451522
SCE1572_7696	0.765775131	-13.6088	0.275258	0.451522
SCE1572_9953	0.751416489	-13.1275	0.275344	0.451582
SCE1572_2187	0.880579606	-15.4476	0.27538	0.451582
SCE1572_1295	0.806579025	-14.4846	0.276118	0.452721
SCE1572_9619	0.727079165	-11.7813	0.276267	0.452895
SCE1572_4040	0.918401072	-15.5986	0.276398	0.453039
SCE1572_5911	-0.772321723	-14.2151	0.27669	0.453376
SCE1572_3121	-0.758775191	-14.2083	0.27669	0.453376
SCE1572_7224	-0.74570019	-13.5726	0.276749	0.453403
SCE1572_10893	-0.782385406	-14.6161	0.277576	0.454656
SCE1572_910	0.730334402	-12.3168	0.277601	0.454656
SCE1572_2298	-0.805759915	-15.0683	0.277818	0.454941
SCE1572_2117	-0.749277095	-13.3771	0.277912	0.455024
SCE1572_9546	-0.741874107	-13.5832	0.278643	0.45615
SCE1572_394	-0.731975646	-13.0928	0.27885	0.456167
SCE1572_8256	-0.902189677	-15.9239	0.278896	0.456167
SCE1572_3128	-0.856385988	-15.901	0.278896	0.456167
SCE1572_10673	-0.828905251	-15.1868	0.278913	0.456167
SCE1572_2215	-0.801938204	-15.1733	0.278913	0.456167

SCE1572_5832	-0.801938204	-15.1733	0.278913	0.456167
SCE1572_4640	0.746955042	-13.3989	0.279583	0.457116
SCE1572_9027	-0.745961998	-13.3864	0.279583	0.457116
SCE1572_1554	0.759872643	-13.845	0.279624	0.457116
SCE1572_3071	0.754478376	-13.572	0.280078	0.457787
SCE1572_11004	-0.758610772	-14.0795	0.280291	0.457992
SCE1572_7189	0.758323856	-14.0937	0.280291	0.457992
SCE1572_7842	-0.734670079	-13.2754	0.281137	0.459264
SCE1572_4440	0.774091172	-14.3853	0.2812	0.459264
SCE1572_277	0.774091172	-14.3853	0.2812	0.459264
SCE1572_2877	-0.79337619	-14.6471	0.281411	0.459538
SCE1572_6858	0.798965841	-15.0144	0.281607	0.459787
SCE1572_8333	-0.736766068	-13.6847	0.281683	0.459839
SCE1572_6248	-0.98191687	-16.3788	0.282111	0.459994
SCE1572_9928	-0.917786532	-16.3467	0.282111	0.459994
SCE1572_4190	-0.744877673	-14.0379	0.282246	0.459994
SCE1572_8775	0.787470202	-14.7572	0.282505	0.459994
SCE1572_899	0.787470202	-14.7572	0.282505	0.459994
SCE1572_10858	-0.709696694	-10.5703	0.282831	0.459994
SCE1572_5172	-0.82162057	-15.111	0.282957	0.459994
SCE1572_1455	0.773900503	-14.1162	0.283027	0.459994
SCE1572_9192	0.779202586	-14.5389	0.283112	0.459994
SCE1572_4370	0.85785953	-15.6288	0.283299	0.459994
SCE1572_4672	0.83710097	-15.6392	0.283299	0.459994
SCE1572_5441	0.772970632	-14.3494	0.283546	0.459994
SCE1572_2640	0.725436805	-12.8496	0.283613	0.459994
SCE1572_11578	1.313539014	-17.816	0.283653	0.459994
SCE1572_10036	1.313539014	-17.816	0.283653	0.459994
SCE1572_7106	1.313539014	-17.816	0.283653	0.459994
SCE1572_8888	1.313539014	-17.816	0.283653	0.459994
SCE1572_821	1.313539014	-17.816	0.283653	0.459994
SCE1572_5674	1.313539014	-17.816	0.283653	0.459994
SCE1572_8352	1.313539014	-17.816	0.283653	0.459994
SCE1572_1457	1.313539014	-17.816	0.283653	0.459994
SCE1572_2738	1.313539014	-17.816	0.283653	0.459994
SCE1572_9385	-1.101498486	-17.6085	0.283653	0.459994
SCE1572_9885	-0.777608874	-14.8319	0.283729	0.459994
SCE1572_10303	0.742038773	-13.3648	0.283732	0.459994
SCE1572_1686	1.008684432	-16.7461	0.283853	0.459994
SCE1572_9871	1.008684432	-16.7461	0.283853	0.459994
SCE1572_5139	1.008684432	-16.7461	0.283853	0.459994
SCE1572_763	0.96561571	-16.7676	0.283853	0.459994
SCE1572_7152	0.96561571	-16.7676	0.283853	0.459994
SCE1572_32	0.96561571	-16.7676	0.283853	0.459994
SCE1572_11456	0.96561571	-16.7676	0.283853	0.459994
SCE1572_2120	-0.72927407	-13.5895	0.283984	0.459994
SCE1572_8411	-0.818911282	-15.2193	0.284504	0.459994
SCE1572_9187	-0.818911282	-15.2193	0.284504	0.459994

SCE1572_7942	0.784429748	-14.4176	0.284547	0.459994
SCE1572_11561	-0.745795126	-13.9229	0.285092	0.459994
SCE1572_9909	0.809496508	-14.9526	0.285106	0.459994
SCE1572_2182	1.107088136	-17.1823	0.285115	0.459994
SCE1572_5639	1.107088136	-17.1823	0.285115	0.459994
SCE1572_269	1.107088136	-17.1823	0.285115	0.459994
SCE1572_2917	1.107088136	-17.1823	0.285115	0.459994
SCE1572_4452	1.107088136	-17.1823	0.285115	0.459994
SCE1572_9755	1.107088136	-17.1823	0.285115	0.459994
SCE1572_1253	1.107088136	-17.1823	0.285115	0.459994
SCE1572_8934	1.107088136	-17.1823	0.285115	0.459994
SCE1572_694	-1.030415388	-17.1136	0.285115	0.459994
SCE1572_5855	0.875417901	-15.8127	0.285285	0.459994
SCE1572_4757	0.875417901	-15.8127	0.285285	0.459994
SCE1572_5830	0.875417901	-15.8127	0.285285	0.459994
SCE1572_1293	0.851958928	-15.8244	0.285285	0.459994
SCE1572_6231	0.851958928	-15.8244	0.285285	0.459994
SCE1572_4768	0.851958928	-15.8244	0.285285	0.459994
SCE1572_3143	0.851958928	-15.8244	0.285285	0.459994
SCE1572_9889	-0.842580188	-15.7786	0.285285	0.459994
SCE1572_7465	-0.734248885	-13.3696	0.285457	0.459994
SCE1572_4890	-0.713831958	-12.0181	0.28558	0.459994
SCE1572_7472	-0.77296998	-14.467	0.285645	0.459994
SCE1572_11501	31.00878188	-34.5117	0.285714	0.459994
SCE1572_9606	31.00878188	-34.5117	0.285714	0.459994
SCE1572_3045	31.00878188	-34.5117	0.285714	0.459994
SCE1572_4818	31.00878188	-34.5117	0.285714	0.459994
SCE1572_1904	31.00878188	-34.5117	0.285714	0.459994
SCE1572_5508	31.00878188	-34.5117	0.285714	0.459994
SCE1572_2835	31.00878188	-34.5117	0.285714	0.459994
SCE1572_11138	31.00878188	-34.5117	0.285714	0.459994
SCE1572_8159	31.00878188	-34.5117	0.285714	0.459994
SCE1572_1560	31.00878188	-34.5117	0.285714	0.459994
SCE1572_4985	31.00878188	-34.5117	0.285714	0.459994
SCE1572_5668	31.00878188	-34.5117	0.285714	0.459994
SCE1572_3004	31.00878188	-34.5117	0.285714	0.459994
SCE1572_5564	31.00878188	-34.5117	0.285714	0.459994
SCE1572_10896	31.00878188	-34.5117	0.285714	0.459994
SCE1572_4678	31.00878188	-34.5117	0.285714	0.459994
SCE1572_2121	31.00878188	-34.5117	0.285714	0.459994
SCE1572_6441	31.00878188	-34.5117	0.285714	0.459994
SCE1572_8091	31.00878188	-34.5117	0.285714	0.459994
SCE1572_10528	31.00878188	-34.5117	0.285714	0.459994
SCE1572_6950	31.00878188	-34.5117	0.285714	0.459994
SCE1572_10670	31.00878188	-34.5117	0.285714	0.459994
SCE1572_6945	31.00878188	-34.5117	0.285714	0.459994
SCE1572_1567	31.00878188	-34.5117	0.285714	0.459994
SCE1572_5243	31.00878188	-34.5117	0.285714	0.459994

SCE1572_10899	31.00878188	-34.5117	0.285714	0.459994
SCE1572_2764	31.00878188	-34.5117	0.285714	0.459994
SCE1572_3556	31.00878188	-34.5117	0.285714	0.459994
SCE1572_7291	31.00878188	-34.5117	0.285714	0.459994
SCE1572_5667	31.00878188	-34.5117	0.285714	0.459994
SCE1572_1905	31.00878188	-34.5117	0.285714	0.459994
SCE1572_5364	31.00878188	-34.5117	0.285714	0.459994
SCE1572_2256	0.74743554	-13.8512	0.28596	0.46032
SCE1572_7568	0.744636364	-13.6194	0.286011	0.460331
SCE1572_4749	0.838201004	-15.3907	0.286292	0.460642
SCE1572_458	0.838201004	-15.3907	0.286292	0.460642
SCE1572_4956	-0.750077086	-14.0234	0.286807	0.461401
SCE1572_4278	0.733134795	-13.3874	0.28722	0.461994
SCE1572_11111	0.751296589	-13.7048	0.287448	0.46229
SCE1572_10267	-0.74090877	-13.9364	0.287646	0.462405
SCE1572_10830	0.898501514	-16.0236	0.287651	0.462405
SCE1572_409	0.898501514	-16.0236	0.287651	0.462405
SCE1572_1406	-0.715274684	-12.5328	0.288053	0.462981
SCE1572_7855	-0.790296797	-14.868	0.288345	0.463316
SCE1572_6758	0.850567037	-15.545	0.288382	0.463316
SCE1572_1706	-0.743247012	-13.8595	0.288394	0.463316
SCE1572_10100	0.823733746	-15.1865	0.288451	0.463337
SCE1572_5607	-0.713291253	-12.7778	0.288813	0.463847
SCE1572_9216	-0.727102971	-13.2514	0.2893	0.464558
SCE1572_2493	0.733314432	-13.4432	0.289705	0.465137
SCE1572_797	-0.707356211	-12.4171	0.290058	0.465599
SCE1572_10983	-0.78599666	-14.9589	0.290081	0.465599
SCE1572_2439	-0.949495392	-16.2106	0.290499	0.465721
SCE1572_5454	0.930210374	-16.2707	0.290499	0.465721
SCE1572_3256	0.930210374	-16.2707	0.290499	0.465721
SCE1572_4648	0.930210374	-16.2707	0.290499	0.465721
SCE1572_4090	0.930210374	-16.2707	0.290499	0.465721
SCE1572_3574	0.930210374	-16.2707	0.290499	0.465721
SCE1572_5740	-0.892911864	-16.1823	0.290499	0.465721
SCE1572_6578	0.832913173	-15.3194	0.290511	0.465721
SCE1572_7633	-0.702314938	-11.4782	0.290717	0.465848
SCE1572_2631	0.770396689	-14.5851	0.290777	0.465848
SCE1572_4827	0.760054744	-14.5903	0.290777	0.465848
SCE1572_8687	0.887775108	-15.707	0.290856	0.465848
SCE1572_9464	0.866080037	-15.7178	0.290856	0.465848
SCE1572_8418	0.866080037	-15.7178	0.290856	0.465848
SCE1572_5071	-0.715460858	-13.3494	0.291268	0.46643
SCE1572_11082	-0.78599666	-14.8069	0.291353	0.46643
SCE1572_2032	0.778959298	-14.8288	0.291353	0.46643
SCE1572_3694	0.757527887	-14.4696	0.291686	0.466892
SCE1572_5055	0.761999514	-14.6768	0.292372	0.467706
SCE1572_1294	0.761999514	-14.6768	0.292372	0.467706
SCE1572_8594	-0.749002452	-14.6509	0.292372	0.467706

SCE1572_1388	-0.749002452	-14.6509	0.292372	0.467706
SCE1572_11093	0.764200423	-14.3537	0.292453	0.467764
SCE1572_9942	0.86243226	-15.4566	0.292915	0.46829
SCE1572_11407	0.84405373	-15.4658	0.292915	0.46829
SCE1572_5365	-0.79225565	-15.4095	0.292915	0.46829
SCE1572_1914	0.769218497	-14.5439	0.293207	0.468685
SCE1572_5237	-0.720554185	-13.1234	0.293546	0.468873
SCE1572_11276	0.910779844	-15.9019	0.293828	0.468873
SCE1572_7409	0.910779844	-15.9019	0.293828	0.468873
SCE1572_10875	0.910779844	-15.9019	0.293828	0.468873
SCE1572_7373	0.910779844	-15.9019	0.293828	0.468873
SCE1572_5175	0.910779844	-15.9019	0.293828	0.468873
SCE1572_1220	0.88611779	-15.9143	0.293828	0.468873
SCE1572_11563	0.88611779	-15.9143	0.293828	0.468873
SCE1572_11401	0.88611779	-15.9143	0.293828	0.468873
SCE1572_4078	0.88611779	-15.9143	0.293828	0.468873
SCE1572_1346	-0.870885557	-15.8494	0.293828	0.468873
SCE1572_2207	1.013978732	-16.5508	0.293949	0.468873
SCE1572_4400	1.013978732	-16.5508	0.293949	0.468873
SCE1572_8149	0.976504026	-16.5695	0.293949	0.468873
SCE1572_7884	0.764200423	-14.7688	0.294153	0.469017
SCE1572_7024	0.764200423	-14.7688	0.294153	0.469017
SCE1572_7170	-0.733003572	-14.032	0.294173	0.469017
SCE1572_3289	0.771645235	-14.6276	0.294891	0.470091
SCE1572_8383	0.785160041	-15.0213	0.295371	0.470643
SCE1572_7258	0.785160041	-15.0213	0.295371	0.470643
SCE1572_6685	0.785160041	-15.0213	0.295371	0.470643
SCE1572_8866	0.777820032	-14.4989	0.295496	0.470771
SCE1572_5236	-0.71620833	-13.2162	0.295633	0.470917
SCE1572_8724	0.779202586	-14.8608	0.296154	0.471675
SCE1572_13	0.715637457	-13.3076	0.296199	0.471675
SCE1572_11219	0.785607458	-14.7108	0.296766	0.472481
SCE1572_9454	0.744173368	-14.1621	0.296795	0.472481
SCE1572_6713	-0.78599666	-15.3215	0.296983	0.472598
SCE1572_7117	0.750491289	-14.0678	0.297112	0.472598
SCE1572_608	-0.700981404	-12.5441	0.297243	0.472598
SCE1572_9702	0.941570236	-16.1276	0.297457	0.472598
SCE1572_126	0.941570236	-16.1276	0.297457	0.472598
SCE1572_1561	0.941570236	-16.1276	0.297457	0.472598
SCE1572_5257	0.913001084	-16.1418	0.297457	0.472598
SCE1572_8649	0.913001084	-16.1418	0.297457	0.472598
SCE1572_4233	0.913001084	-16.1418	0.297457	0.472598
SCE1572_7085	0.789977058	-15.1392	0.29782	0.472598
SCE1572_824	0.719531373	-13.3761	0.297873	0.472598
SCE1572_9236	1.097810323	-16.9239	0.298081	0.472598
SCE1572_9130	1.097810323	-16.9239	0.298081	0.472598
SCE1572_2209	1.097810323	-16.9239	0.298081	0.472598
SCE1572_2790	1.097810323	-16.9239	0.298081	0.472598

SCE1572_3746	1.050504608	-16.9476	0.298081	0.472598
SCE1572_9135	1.050504608	-16.9476	0.298081	0.472598
SCE1572_6619	1.050504608	-16.9476	0.298081	0.472598
SCE1572_7290	1.050504608	-16.9476	0.298081	0.472598
SCE1572_1305	1.050504608	-16.9476	0.298081	0.472598
SCE1572_1232	1.050504608	-16.9476	0.298081	0.472598
SCE1572_4923	1.050504608	-16.9476	0.298081	0.472598
SCE1572_3538	1.050504608	-16.9476	0.298081	0.472598
SCE1572_4878	1.050504608	-16.9476	0.298081	0.472598
SCE1572_4290	-1.007464573	-16.8611	0.298081	0.472598
SCE1572_2094	-0.971863205	-16.8433	0.298081	0.472598
SCE1572_2324	-0.971863205	-16.8433	0.298081	0.472598
SCE1572_11557	0.740091022	-14.2286	0.298272	0.472829
SCE1572_359	0.751416489	-14.1275	0.298512	0.473077
SCE1572_8834	0.702158165	-12.7525	0.298518	0.473077
SCE1572_8163	0.777486113	-14.8111	0.298867	0.473559
SCE1572_7488	-0.816857624	-15.7113	0.299162	0.473956
SCE1572_8714	-0.704608333	-13.0882	0.299322	0.474138
SCE1572_5088	-0.734670079	-14.2754	0.299879	0.474947
SCE1572_633	-0.811991868	-15.4638	0.300098	0.475152
SCE1572_6690	-0.811991868	-15.4638	0.300098	0.475152
SCE1572_7082	0.795690709	-15.2676	0.300655	0.475818
SCE1572_162	0.795690709	-15.2676	0.300655	0.475818
SCE1572_79	0.795690709	-15.2676	0.300655	0.475818
SCE1572_10160	-0.688709927	-11.6607	0.300703	0.475822
SCE1572_2989	0.711897772	-13.2589	0.300853	0.475988
SCE1572_4730	0.754048328	-14.3588	0.301634	0.47703
SCE1572_3174	-0.729529708	-14.3351	0.301634	0.47703
SCE1572_4593	-0.71978106	-14.2278	0.301674	0.47703
SCE1572_7946	0.697325579	-12.5798	0.301698	0.47703
SCE1572_6923	0.728576513	-13.9656	0.301738	0.47703
SCE1572_11428	0.984916266	-16.3954	0.301975	0.477262
SCE1572_3616	0.984916266	-16.3954	0.301975	0.477262
SCE1572_9785	1.252138469	-17.4317	0.302645	0.477817
SCE1572_3103	1.252138469	-17.4317	0.302645	0.477817
SCE1572_3358	1.252138469	-17.4317	0.302645	0.477817
SCE1572_5762	1.252138469	-17.4317	0.302645	0.477817
SCE1572_7897	-1.133919963	-17.3028	0.302645	0.477817
SCE1572_9844	-1.133919963	-17.3028	0.302645	0.477817
SCE1572_9263	-1.008389081	-17.24	0.302645	0.477817
SCE1572_7801	-0.711244087	-13.3154	0.3027	0.477832
SCE1572_2211	-0.720181604	-13.7597	0.30303	0.478281
SCE1572_3511	-0.742495021	-14.2997	0.303468	0.478901
SCE1572_10145	-0.687897218	-12.0137	0.303593	0.478996
SCE1572_9747	-0.844002263	-15.6218	0.303817	0.478996
SCE1572_7944	0.816039354	-15.6498	0.303817	0.478996
SCE1572_8719	0.816039354	-15.6498	0.303817	0.478996
SCE1572_7605	0.816039354	-15.6498	0.303817	0.478996

SCE1572_5870	-0.807476387	-15.6035	0.303817	0.478996
SCE1572_9022	-0.756850314	-14.8215	0.303847	0.478996
SCE1572_3581	0.802577094	-15.4086	0.303974	0.47907
SCE1572_1073	0.808303705	-15.1942	0.303985	0.47907
SCE1572_1728	0.728576513	-14.0788	0.30483	0.480083
SCE1572_7240	-0.718031065	-14.15	0.30501	0.480083
SCE1572_64	-0.74090877	-14.2583	0.30521	0.480083
SCE1572_6175	-0.74090877	-14.2583	0.30521	0.480083
SCE1572_1038	1.635467109	-18.24	0.305221	0.480083
SCE1572_3629	1.635467109	-18.24	0.305221	0.480083
SCE1572_8977	1.635467109	-18.24	0.305221	0.480083
SCE1572_2926	1.635467109	-18.24	0.305221	0.480083
SCE1572_11504	1.635467109	-18.24	0.305221	0.480083
SCE1572_6271	-1.493815908	-17.9973	0.305221	0.480083
SCE1572_2156	-1.493815908	-17.9973	0.305221	0.480083
SCE1572_11083	-1.271423487	-17.8861	0.305221	0.480083
SCE1572_5662	-1.271423487	-17.8861	0.305221	0.480083
SCE1572_11161	0.758323856	-14.5087	0.30568	0.480733
SCE1572_4130	-0.705519322	-13.4219	0.305822	0.480823
SCE1572_11363	0.722234986	-13.8638	0.305829	0.480823
SCE1572_11579	0.750271584	-14.6382	0.305958	0.480955
SCE1572_66	-0.721226404	-14.0261	0.306292	0.481408
SCE1572_3261	-0.706638868	-13.8261	0.307089	0.482588
SCE1572_181	0.816039354	-15.3278	0.307454	0.483018
SCE1572_6522	-0.768923147	-15.2723	0.307454	0.483018
SCE1572_2137	0.811038673	-15.5648	0.307913	0.483536
SCE1572_209	0.811038673	-15.5648	0.307913	0.483536
SCE1572_4953	0.704963956	-13.4385	0.307922	0.483536
SCE1572_2710	0.828112186	-15.8364	0.308333	0.483964
SCE1572_6947	0.828112186	-15.8364	0.308333	0.483964
SCE1572_5283	0.828112186	-15.8364	0.308333	0.483964
SCE1572_708	-0.751416428	-14.7056	0.30853	0.484058
SCE1572_8994	-0.732557401	-14.6961	0.30853	0.484058
SCE1572_6272	-0.732557401	-14.6961	0.30853	0.484058
SCE1572_10236	0.675955981	-11.2727	0.308641	0.484159
SCE1572_11208	-0.689062782	-12.8709	0.308754	0.484264
SCE1572_7243	-0.708487293	-13.9526	0.309035	0.484611
SCE1572_3213	-0.700266786	-13.6937	0.309105	0.484611
SCE1572_7505	0.754111605	-14.8734	0.309113	0.484611
SCE1572_3603	0.687626885	-12.6479	0.30953	0.485192
SCE1572_6831	-0.701334263	-13.3861	0.309743	0.485454
SCE1572_7986	0.757145665	-15.0353	0.309795	0.485462
SCE1572_10690	-0.707994148	-13.9199	0.310322	0.486217
SCE1572_5657	-0.791430546	-15.2056	0.310597	0.486531
SCE1572_9954	0.69706554	-13.3694	0.310616	0.486531
SCE1572_1572	0.702581304	-13.5772	0.310809	0.486705
SCE1572_2430	-0.715030139	-14.0059	0.310819	0.486705
SCE1572_7128	0.708947706	-13.4938	0.31136	0.48748

SCE1572_107	-0.745354675	-14.7866	0.311421	0.487502
SCE1572_135	0.825438052	-15.4751	0.311546	0.487552
SCE1572_4243	0.825438052	-15.4751	0.311546	0.487552
SCE1572_9709	-0.694776255	-13.5472	0.31178	0.487734
SCE1572_429	-0.694776255	-13.5472	0.31178	0.487734
SCE1572_3279	-0.717825157	-14.4625	0.311801	0.487734
SCE1572_3234	0.769603781	-14.9725	0.312343	0.488365
SCE1572_11256	-0.763276583	-14.9476	0.312343	0.488365
SCE1572_4691	-0.763276583	-14.9476	0.312343	0.488365
SCE1572_21	0.84405373	-15.7289	0.312663	0.488646
SCE1572_1454	0.84405373	-15.7289	0.312663	0.488646
SCE1572_5931	0.84405373	-15.7289	0.312663	0.488646
SCE1572_2234	0.774870165	-15.1468	0.313448	0.489475
SCE1572_8843	0.759603409	-15.1544	0.313448	0.489475
SCE1572_2608	0.759603409	-15.1544	0.313448	0.489475
SCE1572_297	0.759603409	-15.1544	0.313448	0.489475
SCE1572_8960	0.759603409	-15.1544	0.313448	0.489475
SCE1572_1700	-0.686460986	-13.3566	0.313532	0.489475
SCE1572_9866	-0.743044515	-14.7289	0.313566	0.489475
SCE1572_4907	-0.743044515	-14.7289	0.313566	0.489475
SCE1572_482	0.709799306	-13.8449	0.313889	0.489538
SCE1572_10600	0.871534467	-16.037	0.313932	0.489538
SCE1572_4292	0.871534467	-16.037	0.313932	0.489538
SCE1572_9184	0.871534467	-16.037	0.313932	0.489538
SCE1572_6223	0.84405373	-16.0508	0.313932	0.489538
SCE1572_7841	0.84405373	-16.0508	0.313932	0.489538
SCE1572_1048	-0.82396451	-16.0103	0.313932	0.489538
SCE1572_8398	0.711702694	-14.2757	0.314245	0.489881
SCE1572_7163	-0.706925089	-14.2615	0.314245	0.489881
SCE1572_1735	0.708768032	-13.922	0.314559	0.490288
SCE1572_1105	0.781043933	-14.9124	0.314692	0.490288
SCE1572_4511	0.768104877	-14.9188	0.314692	0.490288
SCE1572_2314	-0.738549497	-14.8725	0.314692	0.490288
SCE1572_6146	0.728576513	-14.1389	0.314826	0.490423
SCE1572_6070	-1.049031066	-16.9973	0.315121	0.490487
SCE1572_680	-1.049031066	-16.9973	0.315121	0.490487
SCE1572_2880	-0.949495392	-16.9476	0.315121	0.490487
SCE1572_9482	-0.949495392	-16.9476	0.315121	0.490487
SCE1572_4738	-0.89945471	-16.42	0.315146	0.490487
SCE1572_451	-0.89945471	-16.42	0.315146	0.490487
SCE1572_10518	0.67988702	-12.8977	0.315371	0.490763
SCE1572_4600	-0.74090877	-14.6733	0.315564	0.490992
SCE1572_2642	0.772970632	-15.0863	0.316047	0.49167
SCE1572_11465	0.738560602	-14.5186	0.316206	0.491845
SCE1572_8274	-0.771852478	-15.5857	0.316443	0.492082
SCE1572_8298	0.670695597	-12.2467	0.316452	0.492082
SCE1572_4543	0.70801519	-13.9758	0.316504	0.49209
SCE1572_4294	0.692050637	-13.5825	0.316597	0.492161

SCE1572_11399	-0.715030139	-14.3278	0.316697	0.492245
SCE1572_2329	0.739464829	-14.6437	0.317431	0.493313
SCE1572_7427	0.692854591	-13.1878	0.317496	0.493341
SCE1572_9450	0.779202586	-15.2759	0.317704	0.493517
SCE1572_10263	0.762523845	-15.2842	0.317704	0.493517
SCE1572_8926	-0.713645816	-14.4488	0.317845	0.493591
SCE1572_8922	-0.713645816	-14.4488	0.317845	0.493591
SCE1572_431	0.728576513	-14.3012	0.318158	0.493931
SCE1572_5811	0.719963383	-14.3055	0.318158	0.493931
SCE1572_8677	-0.811991868	-15.8788	0.318503	0.494322
SCE1572_9835	-0.811991868	-15.8788	0.318503	0.494322
SCE1572_5496	0.728576513	-14.7866	0.318877	0.494776
SCE1572_4442	0.704328967	-13.7987	0.31889	0.494776
SCE1572_8319	-0.675447994	-12.9526	0.319009	0.494887
SCE1572_5293	-0.766188179	-15.4867	0.319771	0.495923
SCE1572_6097	-0.766188179	-15.4867	0.319771	0.495923
SCE1572_10785	-0.673521931	-12.8632	0.320127	0.496402
SCE1572_3855	0.776939535	-15.2099	0.320336	0.496507
SCE1572_1732	0.776939535	-15.2099	0.320336	0.496507
SCE1572_9424	0.776939535	-15.2099	0.320336	0.496507
SCE1572_7313	0.740164487	-14.7335	0.32075	0.496593
SCE1572_7948	-0.697432104	-13.9471	0.320849	0.496593
SCE1572_7521	0.898501514	-16.2866	0.321053	0.496593
SCE1572_10383	0.898501514	-16.2866	0.321053	0.496593
SCE1572_5893	0.898501514	-16.2866	0.321053	0.496593
SCE1572_11415	0.898501514	-16.2866	0.321053	0.496593
SCE1572_5760	0.898501514	-16.2866	0.321053	0.496593
SCE1572_3047	0.898501514	-16.2866	0.321053	0.496593
SCE1572_10953	-0.875494811	-16.2476	0.321053	0.496593
SCE1572_4529	0.866080037	-16.3028	0.321053	0.496593
SCE1572_178	0.866080037	-16.3028	0.321053	0.496593
SCE1572_6501	0.866080037	-16.3028	0.321053	0.496593
SCE1572_3315	-0.818911282	-16.2193	0.321053	0.496593
SCE1572_10980	-0.818911282	-16.2193	0.321053	0.496593
SCE1572_3917	0.694360798	-13.7106	0.321735	0.497575
SCE1572_10319	-0.697187393	-13.9145	0.321905	0.497765
SCE1572_10729	-0.801938204	-15.7583	0.32241	0.498426
SCE1572_2885	0.784429748	-15.4176	0.322724	0.498426
SCE1572_3641	0.784429748	-15.4176	0.322724	0.498426
SCE1572_1624	0.766051218	-15.4268	0.322724	0.498426
SCE1572_9226	0.766051218	-15.4268	0.322724	0.498426
SCE1572_1323	-0.761228755	-15.394	0.322724	0.498426
SCE1572_9762	-0.761228755	-15.394	0.322724	0.498426
SCE1572_6962	-0.761228755	-15.394	0.322724	0.498426
SCE1572_1098	0.686582424	-13.7837	0.322759	0.498426
SCE1572_1849	0.702581304	-13.8991	0.322919	0.4986
SCE1572_3859	-0.667601959	-13.1437	0.323236	0.499016
SCE1572_5556	-0.652768857	-10.7016	0.323672	0.499609

SCE1572_4015	-0.678725338	-13.4429	0.323866	0.499609
SCE1572_9336	-0.991315568	-16.7461	0.323905	0.499609
SCE1572_2048	-0.991315568	-16.7461	0.323905	0.499609
SCE1572_11139	0.921221591	-16.7898	0.323905	0.499609
SCE1572_10101	0.921221591	-16.7898	0.323905	0.499609
SCE1572_1844	0.717929269	-14.6113	0.324125	0.499802
SCE1572_6413	0.707202862	-14.6167	0.324125	0.499802
SCE1572_8540	0.753238567	-14.8232	0.324489	0.500215
SCE1572_2149	-0.71620833	-14.8012	0.324489	0.500215
SCE1572_5134	-1.119420394	-17.4476	0.324544	0.500228
SCE1572_3133	0.669075501	-13.0939	0.324966	0.500804
SCE1572_1943	0.781687849	-15.345	0.325362	0.500931
SCE1572_3173	0.781687849	-15.345	0.325362	0.500931
SCE1572_9127	-0.728281162	-15.2926	0.325362	0.500931
SCE1572_5804	-0.728281162	-15.2926	0.325362	0.500931
SCE1572_10615	0.718234569	-14.5694	0.325652	0.500931
SCE1572_1743	0.794665703	-15.6604	0.325787	0.500931
SCE1572_7272	0.794665703	-15.6604	0.325787	0.500931
SCE1572_2611	0.772970632	-15.6713	0.325787	0.500931
SCE1572_4397	0.772970632	-15.6713	0.325787	0.500931
SCE1572_1640	0.772970632	-15.6713	0.325787	0.500931
SCE1572_11193	0.883854738	-16.1564	0.325858	0.500931
SCE1572_3008	0.883854738	-16.1564	0.325858	0.500931
SCE1572_3019	0.883854738	-16.1564	0.325858	0.500931
SCE1572_11403	-0.856385988	-16.0937	0.325858	0.500931
SCE1572_10501	-0.856385988	-16.0937	0.325858	0.500931
SCE1572_1564	-0.805759915	-16.0683	0.325858	0.500931
SCE1572_8273	-0.805759915	-16.0683	0.325858	0.500931
SCE1572_6768	-0.715030139	-14.7429	0.326217	0.50141
SCE1572_11290	-0.646825211	-10.2924	0.326679	0.502048
SCE1572_1420	-0.730855106	-14.9638	0.3269	0.502239
SCE1572_919	-0.730855106	-14.9638	0.3269	0.502239
SCE1572_9583	0.728576513	-14.5236	0.327085	0.502378
SCE1572_5901	0.728576513	-14.5236	0.327085	0.502378
SCE1572_11270	-0.664208179	-12.7974	0.327665	0.503195
SCE1572_3503	-0.725989351	-15.2113	0.327732	0.503224
SCE1572_9907	-0.702057841	-14.4546	0.328433	0.504153
SCE1572_5992	-0.686460986	-14.4468	0.328433	0.504153
SCE1572_2267	-0.728896253	-14.8987	0.328733	0.504249
SCE1572_29	-0.707522602	-14.888	0.328733	0.504249
SCE1572_4823	-0.78599666	-15.5439	0.328735	0.504249
SCE1572_8233	-0.78599666	-15.5439	0.328735	0.504249
SCE1572_2610	-0.78599666	-15.5439	0.328735	0.504249
SCE1572_5064	-0.672786049	-13.6799	0.329013	0.504579
SCE1572_9324	-0.641920482	-9.28202	0.329046	0.504579
SCE1572_641	-0.695231196	-14.624	0.329322	0.504929
SCE1572_4696	0.700562137	-14.4221	0.329702	0.505259
SCE1572_10310	0.865756961	-16.0185	0.329842	0.505259

SCE1572_4479	-0.840789133	-15.9546	0.329842	0.505259
SCE1572_5100	-0.794985443	-15.9317	0.329842	0.505259
SCE1572_5126	-0.794985443	-15.9317	0.329842	0.505259
SCE1572_5583	0.744173368	-15.1621	0.329874	0.505259
SCE1572_7502	-0.723935692	-15.1343	0.329874	0.505259
SCE1572_2362	-0.681186044	-13.8906	0.330292	0.505292
SCE1572_2702	-0.681186044	-13.8906	0.330292	0.505292
SCE1572_7107	0.663066559	-12.9848	0.330337	0.505292
SCE1572_5428	-0.949495392	-16.5325	0.330406	0.505292
SCE1572_10894	-0.949495392	-16.5325	0.330406	0.505292
SCE1572_10241	0.938029879	-16.5888	0.330406	0.505292
SCE1572_11516	0.938029879	-16.5888	0.330406	0.505292
SCE1572_276	0.938029879	-16.5888	0.330406	0.505292
SCE1572_7160	0.938029879	-16.5888	0.330406	0.505292
SCE1572_2448	0.938029879	-16.5888	0.330406	0.505292
SCE1572_11328	-0.727102971	-14.8364	0.330424	0.505292
SCE1572_2743	-0.711996078	-14.5809	0.330723	0.505676
SCE1572_6927	-0.675056223	-14.003	0.331082	0.506151
SCE1572_6035	-0.671030946	-13.8541	0.33113	0.506151
SCE1572_2371	-0.670519442	-13.4861	0.331203	0.506165
SCE1572_9195	0.787470202	-15.4941	0.331331	0.506165
SCE1572_6538	0.787470202	-15.4941	0.331331	0.506165
SCE1572_2551	0.787470202	-15.4941	0.331331	0.506165
SCE1572_5425	-0.665996884	-13.2615	0.331769	0.506411
SCE1572_5051	0.758323856	-15.0937	0.331819	0.506411
SCE1572_1726	0.743526854	-15.1011	0.331819	0.506411
SCE1572_6603	-0.722084896	-15.0613	0.331819	0.506411
SCE1572_6287	-0.722084896	-15.0613	0.331819	0.506411
SCE1572_601	-0.647986839	-12.365	0.331825	0.506411
SCE1572_3238	0.675405854	-13.4721	0.33183	0.506411
SCE1572_6200	-0.681407759	-13.8286	0.331936	0.506499
SCE1572_6542	-0.686460986	-14.3135	0.332032	0.506572
SCE1572_6033	-0.646152998	-11.737	0.332176	0.506718
SCE1572_10432	0.67286708	-13.6769	0.332609	0.507305
SCE1572_8688	-0.652353132	-12.6766	0.332707	0.507322
SCE1572_4305	0.694788199	-13.9555	0.332826	0.507322
SCE1572_5600	0.676454113	-13.5341	0.333152	0.507322
SCE1572_4936	-0.827816836	-15.8278	0.333198	0.507322
SCE1572_3350	0.80386464	-15.8485	0.333198	0.507322
SCE1572_1344	0.80386464	-15.8485	0.333198	0.507322
SCE1572_3634	0.80386464	-15.8485	0.333198	0.507322
SCE1572_322	0.80386464	-15.8485	0.333198	0.507322
SCE1572_10008	0.80386464	-15.8485	0.333198	0.507322
SCE1572_11148	-0.78599666	-15.8069	0.333198	0.507322
SCE1572_9856	0.779202586	-15.8608	0.333198	0.507322
SCE1572_4948	0.779202586	-15.8608	0.333198	0.507322
SCE1572_11475	0.754071269	-15.0311	0.333593	0.507839
SCE1572_1143	-0.773923828	-15.3573	0.333635	0.507839

SCE1572_100	0.684633165	-14.0716	0.333862	0.508038
SCE1572_2486	-0.680536152	-14.0582	0.333862	0.508038
SCE1572_8040	0.695409649	-14.2506	0.334119	0.508356
SCE1572_894	-0.666724768	-13.4723	0.334361	0.508651
SCE1572_3216	-0.67604437	-13.8722	0.334427	0.508678
SCE1572_5224	-0.704382894	-14.6551	0.334789	0.50908
SCE1572_6342	-0.704382894	-14.6551	0.334789	0.50908
SCE1572_2620	0.696155035	-14.2178	0.335083	0.509339
SCE1572_8833	0.950968934	-16.4124	0.335401	0.509339
SCE1572_5720	0.950968934	-16.4124	0.335401	0.509339
SCE1572_5245	0.950968934	-16.4124	0.335401	0.509339
SCE1572_702	0.950968934	-16.4124	0.335401	0.509339
SCE1572_5899	0.950968934	-16.4124	0.335401	0.509339
SCE1572_6357	0.916203516	-16.4298	0.335401	0.509339
SCE1572_10932	-0.856385988	-16.316	0.335401	0.509339
SCE1572_11174	0.671992985	-13.4364	0.335498	0.509339
SCE1572_11530	0.745650026	-15.2926	0.335692	0.509339
SCE1572_6725	-0.74090877	-15.2583	0.335692	0.509339
SCE1572_6700	1.172783931	-17.2729	0.335879	0.509339
SCE1572_1588	1.07375996	-17.2579	0.335879	0.509339
SCE1572_8318	1.050504608	-17.2106	0.335879	0.509339
SCE1572_3066	1.050504608	-17.2106	0.335879	0.509339
SCE1572_1999	1.050504608	-17.2106	0.335879	0.509339
SCE1572_2870	0.991610919	-17.24	0.335879	0.509339
SCE1572_1456	0.991610919	-17.24	0.335879	0.509339
SCE1572_5897	0.991610919	-17.24	0.335879	0.509339
SCE1572_1376	0.805264036	-15.7463	0.336064	0.509399
SCE1572_1778	0.798965841	-15.7514	0.336064	0.509399
SCE1572_4228	0.798965841	-15.7514	0.336064	0.509399
SCE1572_1911	0.728576513	-14.8861	0.336711	0.510085
SCE1572_2653	0.728576513	-14.8861	0.336711	0.510085
SCE1572_11355	0.715637457	-14.8926	0.336711	0.510085
SCE1572_7882	-0.687704689	-14.865	0.336711	0.510085
SCE1572_4447	0.693811095	-14.3186	0.337519	0.51105
SCE1572_2591	0.760997991	-15.2178	0.337541	0.51105
SCE1572_8485	0.744878325	-15.2259	0.337541	0.51105
SCE1572_2313	-0.737991287	-15.1788	0.337541	0.51105
SCE1572_9449	0.718522848	-14.5286	0.338327	0.512165
SCE1572_10539	0.671992985	-14.0213	0.339255	0.513496
SCE1572_3676	-0.647838522	-12.8425	0.339526	0.513832
SCE1572_620	-0.655338469	-13.644	0.339713	0.514042
SCE1572_10792	-0.657314641	-13.7717	0.340455	0.515077
SCE1572_10783	0.705856436	-14.7048	0.340545	0.515077
SCE1572_11434	0.694360798	-14.7106	0.340545	0.515077
SCE1572_9982	0.714076943	-15.0569	0.340729	0.515283
SCE1572_10612	-0.67386095	-14.1468	0.340934	0.515517
SCE1572_6040	-0.633698325	-11.9275	0.340982	0.515517
SCE1572_4320	0.692050637	-14.3898	0.341254	0.515825

SCE1572_5865	0.666292235	-13.5749	0.341333	0.515825
SCE1572_2385	-0.656974222	-13.5661	0.341333	0.515825
SCE1572_7124	-0.633021727	-12.0614	0.341443	0.515917
SCE1572_10029	-0.674182657	-14.1095	0.341685	0.516127
SCE1572_3280	-0.674182657	-14.1095	0.341685	0.516127
SCE1572_6041	-0.648897573	-13.2633	0.34173	0.516127
SCE1572_7130	0.663870824	-13.893	0.341972	0.51642
SCE1572_8602	0.714637322	-15	0.342113	0.516495
SCE1572_2482	-0.679301195	-14.3313	0.34212	0.516495
SCE1572_6784	0.656426727	-13.372	0.342442	0.516907
SCE1572_5347	0.816039354	-16.0648	0.342553	0.516917
SCE1572_4143	0.816039354	-16.0648	0.342553	0.516917
SCE1572_1009	0.74743554	-15.4361	0.342597	0.516917
SCE1572_7413	-0.664705718	-14.0278	0.343084	0.517565
SCE1572_8197	1.001595007	-16.972	0.343208	0.517565
SCE1572_227	1.001595007	-16.972	0.343208	0.517565
SCE1572_3408	-0.659988775	-13.6329	0.343223	0.517565
SCE1572_204	0.728576513	-14.9386	0.34338	0.517728
SCE1572_1746	0.66202532	-13.796	0.343733	0.518187
SCE1572_7922	0.764200423	-15.3537	0.344281	0.518854
SCE1572_8109	0.764200423	-15.3537	0.344281	0.518854
SCE1572_3964	0.687934528	-14.222	0.34445	0.518854
SCE1572_3787	-0.66683218	-14.2014	0.34445	0.518854
SCE1572_621	0.689048149	-14.5038	0.344529	0.518854
SCE1572_2690	0.689048149	-14.5038	0.344529	0.518854
SCE1572_1594	-0.670519442	-14.4861	0.344529	0.518854
SCE1572_9318	-0.625585249	-11.1155	0.344571	0.518854
SCE1572_10857	0.654628661	-13.5605	0.344664	0.518919
SCE1572_5828	0.835491717	-15.9396	0.345195	0.519349
SCE1572_1102	0.809496508	-15.9526	0.345195	0.519349
SCE1572_11232	0.809496508	-15.9526	0.345195	0.519349
SCE1572_11499	0.809496508	-15.9526	0.345195	0.519349
SCE1572_2131	0.809496508	-15.9526	0.345195	0.519349
SCE1572_2412	0.690102365	-14.4648	0.345374	0.519542
SCE1572_9234	-0.631466955	-12.0248	0.345509	0.519672
SCE1572_669	0.692050637	-14.8049	0.345619	0.519763
SCE1572_10852	0.667175968	-14.1392	0.345813	0.519979
SCE1572_5702	-0.671510645	-14.3935	0.346168	0.520439
SCE1572_9032	0.70511754	-14.751	0.346613	0.520885
SCE1572_523	-0.677062288	-14.7239	0.346613	0.520885
SCE1572_7423	-0.677062288	-14.7239	0.346613	0.520885
SCE1572_10475	-0.65707718	-14.0465	0.347047	0.521464
SCE1572_8973	-0.686460986	-15.1531	0.347134	0.521519
SCE1572_5560	0.693006557	-14.3265	0.347623	0.522104
SCE1572_5579	0.684987244	-14.323	0.347623	0.522104
SCE1572_6018	0.619165296	-10.2725	0.348034	0.522647
SCE1572_5859	0.728576513	-15.1085	0.348353	0.522888
SCE1572_10435	-0.710708533	-15.0912	0.348353	0.522888

SCE1572_1908	-0.686460986	-15.0791	0.348353	0.522888
SCE1572_8535	0.674536673	-14.633	0.348393	0.522888
SCE1572_10848	0.750944326	-15.6823	0.349299	0.524172
SCE1572_9751	0.658498077	-13.8705	0.350144	0.525365
SCE1572_1556	-0.686460986	-14.9416	0.350467	0.525775
SCE1572_9323	-0.663002014	-14.4585	0.350643	0.525964
SCE1572_4025	-0.640391338	-13.5578	0.350895	0.526083
SCE1572_7397	0.770396689	-15.5851	0.350922	0.526083
SCE1572_9828	0.770396689	-15.5851	0.350922	0.526083
SCE1572_9648	-0.737087059	-15.5683	0.350922	0.526083
SCE1572_7172	0.702581304	-14.8991	0.35139	0.526683
SCE1572_7624	-0.638205818	-13.7622	0.351422	0.526683
SCE1572_9288	-0.620904356	-11.9716	0.351796	0.527054
SCE1572_5948	0.682772823	-14.3945	0.35192	0.527054
SCE1572_3927	-0.679081456	-14.3749	0.35192	0.527054
SCE1572_3389	0.806579025	-16.4846	0.351957	0.527054
SCE1572_10051	-0.629877458	-12.9215	0.352049	0.527054
SCE1572_11358	0.703485532	-14.8481	0.352237	0.527054
SCE1572_3411	-0.733766701	-15.4705	0.352333	0.527054
SCE1572_5877	-0.733766701	-15.4705	0.352333	0.527054
SCE1572_553	-0.733766701	-15.4705	0.352333	0.527054
SCE1572_10187	1.231076853	-17.8573	0.352672	0.527054
SCE1572_255	1.231076853	-17.8573	0.352672	0.527054
SCE1572_4460	1.231076853	-17.8573	0.352672	0.527054
SCE1572_4431	1.231076853	-17.8573	0.352672	0.527054
SCE1572_10134	1.231076853	-17.8573	0.352672	0.527054
SCE1572_4097	1.143614012	-17.901	0.352672	0.527054
SCE1572_2118	1.143614012	-17.901	0.352672	0.527054
SCE1572_8493	1.143614012	-17.901	0.352672	0.527054
SCE1572_6818	1.143614012	-17.901	0.352672	0.527054
SCE1572_151	1.143614012	-17.901	0.352672	0.527054
SCE1572_7731	1.143614012	-17.901	0.352672	0.527054
SCE1572_6301	-0.641791937	-13.6508	0.352913	0.527339
SCE1572_8992	-0.686460986	-14.7572	0.353019	0.527422
SCE1572_5405	-0.730855106	-15.3788	0.353571	0.528022
SCE1572_4226	-0.701107762	-15.3639	0.353571	0.528022
SCE1572_11257	-0.701107762	-15.3639	0.353571	0.528022
SCE1572_6872	-0.686460986	-14.7006	0.353741	0.528126
SCE1572_6332	0.682772823	-14.7164	0.353741	0.528126
SCE1572_1192	0.684182394	-14.6713	0.354411	0.528857
SCE1572_4302	0.728576513	-15.3012	0.354665	0.528857
SCE1572_111	0.728576513	-15.3012	0.354665	0.528857
SCE1572_8014	0.711298522	-15.3098	0.354665	0.528857
SCE1572_7028	-0.700266786	-15.2786	0.354665	0.528857
SCE1572_8247	0.832913173	-16.3194	0.354733	0.528857
SCE1572_127	0.832913173	-16.3194	0.354733	0.528857
SCE1572_8196	0.832913173	-16.3194	0.354733	0.528857
SCE1572_2948	0.832913173	-16.3194	0.354733	0.528857

SCE1572_4141	0.832913173	-16.3194	0.354733	0.528857
SCE1572_9466	-0.652167809	-13.7839	0.35488	0.529001
SCE1572_3260	-0.64993511	-14.0973	0.355403	0.529705
SCE1572_6715	0.728576513	-15.2341	0.355639	0.529831
SCE1572_7068	0.728576513	-15.2341	0.355639	0.529831
SCE1572_2421	-0.699517139	-15.1981	0.355639	0.529831
SCE1572_5009	-0.635386802	-13.166	0.355833	0.529971
SCE1572_9497	-0.629127811	-13.1629	0.355833	0.529971
SCE1572_7796	0.621661309	-12.7697	0.355962	0.530087
SCE1572_3445	0.625890849	-12.7114	0.356572	0.530664
SCE1572_10619	0.680321344	-14.4697	0.356667	0.530664
SCE1572_2550	0.823733746	-16.1865	0.356891	0.530664
SCE1572_4339	0.823733746	-16.1865	0.356891	0.530664
SCE1572_4393	0.823733746	-16.1865	0.356891	0.530664
SCE1572_6946	0.823733746	-16.1865	0.356891	0.530664
SCE1572_2212	-0.78599666	-16.1288	0.356891	0.530664
SCE1572_9809	-0.78599666	-16.1288	0.356891	0.530664
SCE1572_2341	-0.735370587	-16.1035	0.356891	0.530664
SCE1572_8449	-0.735370587	-16.1035	0.356891	0.530664
SCE1572_7724	-0.642405806	-13.9527	0.356904	0.530664
SCE1572_9724	0.699430167	-15.0642	0.357297	0.531173
SCE1572_1189	-0.652027489	-14.2753	0.358416	0.532761
SCE1572_1612	-0.776658795	-15.9867	0.358608	0.532892
SCE1572_5981	-0.730855106	-15.9638	0.358608	0.532892
SCE1572_4574	0.715155997	-14.9453	0.358656	0.532892
SCE1572_11438	0.656225669	-14.2061	0.35915	0.533469
SCE1572_6594	-0.635579817	-13.3532	0.359221	0.533469
SCE1572_1164	-0.696729322	-14.8516	0.359247	0.533469
SCE1572_610	-0.696729322	-14.8516	0.359247	0.533469
SCE1572_7850	-0.627567297	-13.0496	0.359358	0.533559
SCE1572_9686	-0.635995919	-13.6893	0.359464	0.533633
SCE1572_648	-0.622588803	-13.0292	0.35951	0.533633
SCE1572_9175	-0.606058243	-10.855	0.359587	0.533672
SCE1572_9129	0.679666912	-14.8111	0.359789	0.533897
SCE1572_3810	0.754111605	-15.8734	0.360004	0.533989
SCE1572_3614	0.754111605	-15.8734	0.360004	0.533989
SCE1572_3409	0.754111605	-15.8734	0.360004	0.533989
SCE1572_6176	-0.625060442	-13.5248	0.360742	0.535008
SCE1572_5375	0.775882228	-15.7629	0.361157	0.535096
SCE1572_9727	0.775882228	-15.7629	0.361157	0.535096
SCE1572_9956	0.752423255	-15.7747	0.361157	0.535096
SCE1572_71	0.752423255	-15.7747	0.361157	0.535096
SCE1572_222	0.752423255	-15.7747	0.361157	0.535096
SCE1572_4858	0.752423255	-15.7747	0.361157	0.535096
SCE1572_6627	-0.723935692	-15.7193	0.361157	0.535096
SCE1572_9568	0.665382686	-14.5958	0.36157	0.535218
SCE1572_504	1.120893936	-17.4973	0.361697	0.535218
SCE1572_8384	1.120893936	-17.4973	0.361697	0.535218

SCE1572_5693	1.120893936	-17.4973	0.361697	0.535218
SCE1572_7292	1.120893936	-17.4973	0.361697	0.535218
SCE1572_8351	1.120893936	-17.4973	0.361697	0.535218
SCE1572_1873	1.120893936	-17.4973	0.361697	0.535218
SCE1572_6103	1.120893936	-17.4973	0.361697	0.535218
SCE1572_8828	1.096646389	-17.5095	0.361697	0.535218
SCE1572_10025	-0.721226404	-15.611	0.362123	0.535773
SCE1572_10157	-0.642066867	-14.3788	0.362906	0.536836
SCE1572_8708	-0.718882464	-15.5103	0.362944	0.536836
SCE1572_7059	-0.643392264	-14.3351	0.36319	0.537124
SCE1572_10977	-0.65844661	-14.2995	0.363456	0.537361
SCE1572_483	0.728576513	-15.4456	0.363648	0.537361
SCE1572_6292	0.728576513	-15.4456	0.363648	0.537361
SCE1572_2494	0.728576513	-15.4456	0.363648	0.537361
SCE1572_7698	0.70946769	-15.4551	0.363648	0.537361
SCE1572_9118	0.652849187	-14.2719	0.363707	0.537361
SCE1572_4892	-0.645819002	-14.2514	0.363707	0.537361
SCE1572_7865	0.728576513	-15.3716	0.364258	0.5381
SCE1572_10304	-0.607342269	-12.274	0.364607	0.53854
SCE1572_2446	0.635467109	-14.0396	0.364957	0.53898
SCE1572_2839	0.644512248	-14.0076	0.36513	0.53916
SCE1572_1573	0.680747988	-15.1938	0.365261	0.539278
SCE1572_7029	0.640318547	-13.9047	0.365604	0.539708
SCE1572_1275	-0.923500184	-17.0601	0.366588	0.541073
SCE1572_7982	0.676109093	-14.9124	0.366683	0.541073
SCE1572_7114	0.676109093	-14.9124	0.366683	0.541073
SCE1572_569	-0.657314641	-14.7717	0.367202	0.541764
SCE1572_4342	0.659313851	-14.7281	0.367428	0.542021
SCE1572_7218	-0.661370005	-14.5556	0.368002	0.542715
SCE1572_2280	0.656683594	-14.5595	0.368002	0.542715
SCE1572_7178	0.615682457	-13.2108	0.368142	0.542726
SCE1572_4483	-0.646271896	-14.4983	0.368164	0.542726
SCE1572_4487	-0.646271896	-14.4983	0.368164	0.542726
SCE1572_6967	0.658187185	-14.3364	0.368708	0.5433
SCE1572_8713	0.658187185	-14.3364	0.368708	0.5433
SCE1572_5141	0.649142045	-14.3409	0.368708	0.5433
SCE1572_6677	-0.627567297	-14.0496	0.36938	0.543576
SCE1572_4807	-0.60735844	-12.668	0.369406	0.543576
SCE1572_7899	-0.628975492	-14.0147	0.369455	0.543576
SCE1572_1141	0.875417901	-16.8127	0.369464	0.543576
SCE1572_9815	0.845321477	-16.8278	0.369464	0.543576
SCE1572_2061	0.828112186	-16.8364	0.369464	0.543576
SCE1572_1050	0.828112186	-16.8364	0.369464	0.543576
SCE1572_8044	0.828112186	-16.8364	0.369464	0.543576
SCE1572_755	0.828112186	-16.8364	0.369464	0.543576
SCE1572_598	0.828112186	-16.8364	0.369464	0.543576
SCE1572_4428	-0.801938204	-16.7583	0.369464	0.543576
SCE1572_1416	0.64232078	-13.9293	0.36966	0.543789

SCE1572_4683	0.618393595	-13.6611	0.370127	0.544346
SCE1572_7377	-0.613343057	-13.5963	0.370208	0.544346
SCE1572_9312	-0.613343057	-13.5963	0.370208	0.544346
SCE1572_3801	0.613933947	-13.5809	0.370247	0.544346
SCE1572_3826	0.622867072	-13.2228	0.370708	0.544759
SCE1572_6123	-0.613815684	-13.2149	0.370708	0.544759
SCE1572_4063	-0.613815684	-13.2149	0.370708	0.544759
SCE1572_1423	0.605046595	-12.7218	0.371165	0.544759
SCE1572_1238	0.898501514	-16.6085	0.371253	0.544759
SCE1572_10290	0.898501514	-16.6085	0.371253	0.544759
SCE1572_5665	0.898501514	-16.6085	0.371253	0.544759
SCE1572_582	0.898501514	-16.6085	0.371253	0.544759
SCE1572_5960	0.898501514	-16.6085	0.371253	0.544759
SCE1572_10260	0.898501514	-16.6085	0.371253	0.544759
SCE1572_2089	0.898501514	-16.6085	0.371253	0.544759
SCE1572_10667	0.898501514	-16.6085	0.371253	0.544759
SCE1572_6032	-0.78599666	-16.5439	0.371253	0.544759
SCE1572_11159	-0.78599666	-16.5439	0.371253	0.544759
SCE1572_6141	0.880579606	-16.4476	0.372408	0.546103
SCE1572_5278	0.880579606	-16.4476	0.372408	0.546103
SCE1572_6305	0.880579606	-16.4476	0.372408	0.546103
SCE1572_7541	-0.773923828	-16.3573	0.372408	0.546103
SCE1572_2935	1.898501514	-19.1085	0.372947	0.546103
SCE1572_9319	1.898501514	-19.1085	0.372947	0.546103
SCE1572_10589	1.898501514	-19.1085	0.372947	0.546103
SCE1572_7537	1.898501514	-19.1085	0.372947	0.546103
SCE1572_2127	1.898501514	-19.1085	0.372947	0.546103
SCE1572_3223	1.898501514	-19.1085	0.372947	0.546103
SCE1572_8036	1.898501514	-19.1085	0.372947	0.546103
SCE1572_6249	1.898501514	-19.1085	0.372947	0.546103
SCE1572_2898	1.898501514	-19.1085	0.372947	0.546103
SCE1572_10795	-1.686460986	-18.901	0.372947	0.546103
SCE1572_6651	-1.686460986	-18.901	0.372947	0.546103
SCE1572_10448	-0.764463498	-16.1921	0.373174	0.546359
SCE1572_1550	0.787470202	-16.0791	0.37369	0.546566
SCE1572_6615	0.758323856	-16.0937	0.37369	0.546566
SCE1572_10938	0.758323856	-16.0937	0.37369	0.546566
SCE1572_9038	-0.756850314	-16.0439	0.37369	0.546566
SCE1572_540	0.637280679	-14.0112	0.373903	0.546566
SCE1572_9049	0.637428625	-14.1244	0.37399	0.546566
SCE1572_1278	0.783024297	-15.9658	0.374041	0.546566
SCE1572_8684	-0.750591324	-15.9095	0.374041	0.546566
SCE1572_3166	0.654575932	-14.4086	0.37422	0.546566
SCE1572_10222	-0.745354675	-15.7866	0.37428	0.546566
SCE1572_819	0.728576513	-15.6935	0.374441	0.546566
SCE1572_8711	0.728576513	-15.6935	0.374441	0.546566
SCE1572_6774	0.705856436	-15.7048	0.374441	0.546566
SCE1572_3890	0.705856436	-15.7048	0.374441	0.546566

SCE1572_7031	0.705856436	-15.7048	0.374441	0.546566
SCE1572_7146	0.705856436	-15.7048	0.374441	0.546566
SCE1572_2038	-0.704382894	-15.6551	0.374441	0.546566
SCE1572_3075	0.667175968	-14.8173	0.374515	0.546566
SCE1572_11549	0.74847607	-15.5136	0.374612	0.546566
SCE1572_6157	0.74847607	-15.5136	0.374612	0.546566
SCE1572_3495	0.728576513	-15.5236	0.374612	0.546566
SCE1572_9372	0.669682824	-15.0791	0.374637	0.546566
SCE1572_551	-0.661370005	-15.1405	0.374655	0.546566
SCE1572_5976	0.693811095	-15.3186	0.374665	0.546566
SCE1572_10176	0.71208839	-15.2423	0.374666	0.546566
SCE1572_1078	0.695409649	-15.2506	0.374666	0.546566
SCE1572_3473	0.603045631	-12.7119	0.374877	0.546797
SCE1572_7019	0.604156678	-12.8731	0.37518	0.547163
SCE1572_10872	0.61015602	-13.2291	0.375949	0.548209
SCE1572_6587	0.596743677	-12.5696	0.376334	0.548694
SCE1572_5385	0.601996016	-12.791	0.376987	0.54957
SCE1572_3807	-0.59191535	-12.3822	0.377349	0.550022
SCE1572_5107	-0.613948487	-13.7648	0.377523	0.550199
SCE1572_9704	-0.610909953	-13.8241	0.377724	0.550416
SCE1572_1860	-0.617873901	-13.891	0.377941	0.550656
SCE1572_591	-0.603267051	-13.1696	0.378392	0.551236
SCE1572_5953	0.641593211	-14.1824	0.379009	0.552058
SCE1572_10663	-0.597298766	-12.8407	0.379208	0.552272
SCE1572_8529	0.620427709	-14.2557	0.37934	0.552388
SCE1572_7950	-0.611652399	-13.4688	0.379736	0.552888
SCE1572_2462	-0.635834913	-14.5683	0.380799	0.554359
SCE1572_4740	-0.615076353	-13.8418	0.381822	0.555771
SCE1572_3786	-0.58071811	-11.4747	0.382205	0.5561
SCE1572_11431	0.67795044	-14.8608	0.382259	0.5561
SCE1572_5978	0.665011384	-14.8673	0.382259	0.5561
SCE1572_5848	0.665011384	-14.8673	0.382259	0.5561
SCE1572_6546	0.593113096	-13.0333	0.382494	0.556364
SCE1572_9581	-0.653294122	-14.9582	0.382988	0.557005
SCE1572_3897	-0.651695568	-15.0261	0.383391	0.557516
SCE1572_11014	0.667175968	-15.1392	0.383825	0.55807
SCE1572_2279	-0.587199355	-12.481	0.384134	0.558441
SCE1572_3903	-0.59111311	-13.0224	0.384777	0.559174
SCE1572_6803	-0.672786049	-15.2649	0.384798	0.559174
SCE1572_4941	-0.645819002	-15.2514	0.384798	0.559174
SCE1572_9631	0.710429166	-15.3806	0.385344	0.55989
SCE1572_4690	-0.635579817	-14.3532	0.385497	0.560036
SCE1572_7600	-0.600885255	-13.4513	0.385865	0.560131
SCE1572_40	0.635467109	-14.4181	0.38587	0.560131
SCE1572_3217	0.635467109	-14.4181	0.38587	0.560131
SCE1572_4222	0.635467109	-14.4181	0.38587	0.560131
SCE1572_10889	-0.633993566	-14.3975	0.38587	0.560131
SCE1572_2559	0.690102365	-15.4648	0.385936	0.560131

SCE1572_10770	-0.671030946	-15.4391	0.385936	0.560131
SCE1572_8920	-0.597061901	-13.1665	0.386081	0.560214
SCE1572_2972	0.597789905	-13.5301	0.386536	0.560214
SCE1572_137	0.687934528	-15.5439	0.386579	0.560214
SCE1572_8071	0.687934528	-15.5439	0.386579	0.560214
SCE1572_5741	0.687934528	-15.5439	0.386579	0.560214
SCE1572_1945	0.687934528	-15.5439	0.386579	0.560214
SCE1572_9259	0.687934528	-15.5439	0.386579	0.560214
SCE1572_2612	0.687934528	-15.5439	0.386579	0.560214
SCE1572_7494	-0.669972864	-15.5348	0.386579	0.560214
SCE1572_9803	-0.669972864	-15.5348	0.386579	0.560214
SCE1572_7914	0.667175968	-15.5543	0.386579	0.560214
SCE1572_8265	-0.63050458	-14.4904	0.386681	0.560286
SCE1572_4759	0.643687615	-14.6067	0.387594	0.561377
SCE1572_1208	0.643687615	-14.6067	0.387594	0.561377
SCE1572_1947	0.643687615	-14.6067	0.387594	0.561377
SCE1572_8124	0.728576513	-15.7866	0.388042	0.561793
SCE1572_1602	0.728576513	-15.7866	0.388042	0.561793
SCE1572_7179	0.728576513	-15.7866	0.388042	0.561793
SCE1572_8245	-0.640068081	-14.7054	0.38863	0.562509
SCE1572_5835	-0.569896174	-10.6703	0.388643	0.562509
SCE1572_10224	0.579713127	-12.0955	0.388786	0.562637
SCE1572_9198	0.728576513	-15.8861	0.388873	0.562687
SCE1572_11347	-0.638205818	-14.7622	0.389201	0.562806
SCE1572_8530	-0.613815684	-14.2149	0.389575	0.562806
SCE1572_759	0.568274096	-10.743	0.389984	0.562806
SCE1572_8423	0.606585989	-13.545	0.390002	0.562806
SCE1572_10661	-0.59858823	-14.2485	0.390029	0.562806
SCE1572_4903	-0.59858823	-14.2485	0.390029	0.562806
SCE1572_7369	1.535931435	-18.2898	0.390133	0.562806
SCE1572_1784	1.535931435	-18.2898	0.390133	0.562806
SCE1572_1956	1.535931435	-18.2898	0.390133	0.562806
SCE1572_505	1.535931435	-18.2898	0.390133	0.562806
SCE1572_2753	1.535931435	-18.2898	0.390133	0.562806
SCE1572_780	1.535931435	-18.2898	0.390133	0.562806
SCE1572_10368	1.429016231	-18.3433	0.390133	0.562806
SCE1572_2700	1.429016231	-18.3433	0.390133	0.562806
SCE1572_1759	1.429016231	-18.3433	0.390133	0.562806
SCE1572_6354	1.429016231	-18.3433	0.390133	0.562806
SCE1572_11587	-1.271423487	-18.1085	0.390133	0.562806
SCE1572_10759	-1.271423487	-18.1085	0.390133	0.562806
SCE1572_143	-1.271423487	-18.1085	0.390133	0.562806
SCE1572_8568	1.185238511	-18.2203	0.390133	0.562806
SCE1572_2375	-1.049031066	-17.9973	0.390133	0.562806
SCE1572_8991	-1.049031066	-17.9973	0.390133	0.562806
SCE1572_7072	-0.5883967	-13.5838	0.390613	0.563422
SCE1572_2001	0.79270685	-16.202	0.390757	0.563552
SCE1572_10471	-0.607290773	-14.3391	0.391012	0.563842

SCE1572_287	-0.582884583	-13.6627	0.391614	0.564339
SCE1572_10740	0.798965841	-16.3364	0.391806	0.564339
SCE1572_10717	0.798965841	-16.3364	0.391806	0.564339
SCE1572_6257	0.798965841	-16.3364	0.391806	0.564339
SCE1572_10158	0.798965841	-16.3364	0.391806	0.564339
SCE1572_10734	0.764200423	-16.3537	0.391806	0.564339
SCE1572_9033	0.764200423	-16.3537	0.391806	0.564339
SCE1572_6748	0.764200423	-16.3537	0.391806	0.564339
SCE1572_9036	0.657493415	-15.0286	0.391947	0.564339
SCE1572_10381	0.657493415	-15.0286	0.391947	0.564339
SCE1572_7659	0.657493415	-15.0286	0.391947	0.564339
SCE1572_3550	0.654575932	-15.0866	0.392776	0.565099
SCE1572_4931	0.654575932	-15.0866	0.392776	0.565099
SCE1572_5367	-0.811991868	-16.4638	0.392904	0.565099
SCE1572_171	-0.811991868	-16.4638	0.392904	0.565099
SCE1572_6674	-0.811991868	-16.4638	0.392904	0.565099
SCE1572_5966	0.768104877	-16.5038	0.392904	0.565099
SCE1572_3425	0.768104877	-16.5038	0.392904	0.565099
SCE1572_153	0.768104877	-16.5038	0.392904	0.565099
SCE1572_4654	-0.607568661	-14.083	0.392996	0.565153
SCE1572_341	0.613712556	-14.1362	0.393501	0.565802
SCE1572_8327	0.603045631	-13.8494	0.393981	0.56636
SCE1572_3599	-0.830850896	-16.6658	0.393996	0.56636
SCE1572_2958	0.596711065	-13.3844	0.394192	0.566564
SCE1572_11057	0.950968934	-16.9973	0.394949	0.567187
SCE1572_6646	0.950968934	-16.9973	0.394949	0.567187
SCE1572_1846	0.950968934	-16.9973	0.394949	0.567187
SCE1572_5368	0.950968934	-16.9973	0.394949	0.567187
SCE1572_97	0.898501514	-17.0236	0.394949	0.567187
SCE1572_9610	0.898501514	-17.0236	0.394949	0.567187
SCE1572_8808	0.930210374	-17.2707	0.395441	0.567351
SCE1572_4661	0.930210374	-17.2707	0.395441	0.567351
SCE1572_6477	0.930210374	-17.2707	0.395441	0.567351
SCE1572_2564	0.930210374	-17.2707	0.395441	0.567351
SCE1572_6936	0.930210374	-17.2707	0.395441	0.567351
SCE1572_3889	0.930210374	-17.2707	0.395441	0.567351
SCE1572_5782	-0.892911864	-17.1823	0.395441	0.567351
SCE1572_4289	-0.614311201	-14.6645	0.395509	0.567371
SCE1572_7987	-0.65844661	-15.2995	0.395739	0.567545
SCE1572_1077	0.608994897	-14.2938	0.395792	0.567545
SCE1572_9461	-0.603998826	-14.2723	0.395792	0.567545
SCE1572_4830	-0.601572089	-14.3142	0.396445	0.568403
SCE1572_10342	-0.686460986	-15.401	0.396923	0.568934
SCE1572_4924	-0.686460986	-15.401	0.396923	0.568934
SCE1572_8947	-0.593351582	-14.0325	0.397004	0.568973
SCE1572_1718	-0.613212004	-14.3644	0.397134	0.569081
SCE1572_1946	0.600420161	-14.1137	0.398208	0.570542
SCE1572_9806	-0.580054759	-13.271	0.398342	0.570657

SCE1572_499	0.707202862	-15.6167	0.399684	0.572112
SCE1572_8097	0.707202862	-15.6167	0.399684	0.572112
SCE1572_3225	0.707202862	-15.6167	0.399684	0.572112
SCE1572_9989	0.685507791	-15.6276	0.399684	0.572112
SCE1572_9277	0.685507791	-15.6276	0.399684	0.572112
SCE1572_6262	0.685507791	-15.6276	0.399684	0.572112
SCE1572_3414	-0.564139661	-12.2366	0.400283	0.572892
SCE1572_11455	0.586557508	-13.7645	0.400599	0.573265
SCE1572_8614	-0.591180483	-13.9783	0.40078	0.573447
SCE1572_10223	-0.580176193	-13.5797	0.400939	0.573596
SCE1572_2621	0.594275421	-14.2688	0.401002	0.573608
SCE1572_3447	-0.686460986	-15.7006	0.401305	0.573729
SCE1572_8849	0.659313851	-15.7281	0.401305	0.573729
SCE1572_2757	0.659313851	-15.7281	0.401305	0.573729
SCE1572_10901	-0.64993511	-15.6823	0.401305	0.573729
SCE1572_9388	-0.600046235	-14.018	0.40144	0.573766
SCE1572_9529	-0.588905677	-14.0124	0.40144	0.573766
SCE1572_10731	-0.639155272	-15.0554	0.401512	0.573792
SCE1572_5772	0.618393595	-14.6611	0.40226	0.574626
SCE1572_4377	0.606897956	-14.6669	0.40226	0.574626
SCE1572_5440	0.606897956	-14.6669	0.40226	0.574626
SCE1572_2706	0.569878767	-12.8659	0.402621	0.575064
SCE1572_5603	-0.636707951	-15.1282	0.402827	0.575251
SCE1572_4518	-0.56515469	-13.0924	0.402862	0.575251
SCE1572_2200	-0.587285581	-13.8757	0.40299	0.575356
SCE1572_9302	-0.686460986	-15.816	0.403125	0.575471
SCE1572_5491	0.609429248	-14.3955	0.403527	0.575966
SCE1572_10787	-0.58517765	-13.569	0.404213	0.576784
SCE1572_4888	0.647958053	-15.2102	0.404264	0.576784
SCE1572_11133	0.631279312	-15.2186	0.404264	0.576784
SCE1572_5024	0.594825124	-14.1754	0.404412	0.576917
SCE1572_5980	0.554003008	-11.3714	0.404479	0.576934
SCE1572_8982	0.756057249	-15.9793	0.405182	0.577545
SCE1572_9168	0.728576513	-15.993	0.405182	0.577545
SCE1572_11525	0.728576513	-15.993	0.405182	0.577545
SCE1572_7075	-0.686460986	-15.9416	0.405182	0.577545
SCE1572_9271	-0.686460986	-15.9416	0.405182	0.577545
SCE1572_7187	0.573774786	-13.3446	0.40553	0.577962
SCE1572_7833	-0.574815631	-13.4872	0.405726	0.578164
SCE1572_2262	0.606585989	-14.545	0.406536	0.579239
SCE1572_4187	-0.562281575	-12.8632	0.407015	0.579578
SCE1572_10324	-0.633993566	-14.8829	0.407034	0.579578
SCE1572_8210	-0.633993566	-14.8829	0.407034	0.579578
SCE1572_8378	-0.613529464	-14.8727	0.407034	0.579578
SCE1572_846	-0.582625175	-14.283	0.407071	0.579578
SCE1572_4993	0.728576513	-16.1085	0.407523	0.579578
SCE1572_354	0.728576513	-16.1085	0.407523	0.579578
SCE1572_5582	0.728576513	-16.1085	0.407523	0.579578

SCE1572_5412	0.698202864	-16.1237	0.407523	0.579578
SCE1572_7516	-0.686460986	-16.0791	0.407523	0.579578
SCE1572_8846	-0.686460986	-16.0791	0.407523	0.579578
SCE1572_6337	-0.609839705	-14.5813	0.407672	0.579578
SCE1572_9268	-0.609839705	-14.5813	0.407672	0.579578
SCE1572_10837	-0.609839705	-14.5813	0.407672	0.579578
SCE1572_10831	0.603045631	-14.5863	0.407672	0.579578
SCE1572_3967	0.591694707	-13.9039	0.40771	0.579578
SCE1572_9490	-0.581107986	-13.8889	0.40771	0.579578
SCE1572_10117	-0.631599051	-14.9473	0.408475	0.580586
SCE1572_4465	-0.55992858	-13.0158	0.408534	0.580592
SCE1572_10591	0.578829393	-13.6186	0.408714	0.580769
SCE1572_26	-0.568624496	-13.7875	0.409346	0.581548
SCE1572_9092	0.57657342	-13.6402	0.409377	0.581548
SCE1572_2218	-0.556825706	-13.0508	0.409428	0.581548
SCE1572_6055	-0.552324314	-12.7266	0.409798	0.581996
SCE1572_9975	0.642846639	-15.0359	0.410039	0.582259
SCE1572_8249	-0.584581372	-13.9925	0.410148	0.582261
SCE1572_338	0.626696899	-14.7	0.410185	0.582261
SCE1572_4435	-0.686460986	-16.2311	0.410207	0.582261
SCE1572_658	-0.548628793	-12.2768	0.411318	0.58376
SCE1572_8968	0.603045631	-14.4709	0.411382	0.583772
SCE1572_3820	0.611219562	-14.7522	0.41158	0.583816
SCE1572_11048	-0.600731112	-14.7434	0.41158	0.583816
SCE1572_8414	0.598941233	-14.7583	0.41158	0.583816
SCE1572_10218	-0.564064355	-13.3398	0.411647	0.583833
SCE1572_2050	0.623879134	-15.102	0.411745	0.583893
SCE1572_10318	0.564276061	-13.6057	0.412037	0.584229
SCE1572_6763	-0.552491816	-13.0031	0.412986	0.585496
SCE1572_3120	0.84405373	-16.4658	0.413309	0.585716
SCE1572_7161	0.84405373	-16.4658	0.413309	0.585716
SCE1572_11337	0.84405373	-16.4658	0.413309	0.585716
SCE1572_241	0.553742711	-12.6724	0.414036	0.586668
SCE1572_10292	-0.58492296	-14.3502	0.414588	0.58737
SCE1572_4965	0.57824224	-14.1837	0.41505	0.587946
SCE1572_8270	0.610520751	-14.6232	0.415387	0.588053
SCE1572_5449	0.610520751	-14.6232	0.415387	0.588053
SCE1572_4068	-0.600046235	-14.6029	0.415387	0.588053
SCE1572_7448	-0.577850488	-14.0069	0.415406	0.588053
SCE1572_11224	0.655955841	-15.2704	0.415664	0.588053
SCE1572_2607	0.644184326	-15.2763	0.415664	0.588053
SCE1572_1207	0.644184326	-15.2763	0.415664	0.588053
SCE1572_6597	0.644184326	-15.2763	0.415664	0.588053
SCE1572_2815	-0.61934679	-15.2382	0.415664	0.588053
SCE1572_876	0.560260764	-13.3853	0.415719	0.588053
SCE1572_3604	0.549214655	-12.6642	0.415792	0.588053
SCE1572_6623	0.580477874	-14.41	0.415853	0.588053
SCE1572_4273	0.580477874	-14.41	0.415853	0.588053

SCE1572_186	0.621661309	-14.9396	0.416456	0.588828
SCE1572_6645	0.704328967	-15.7987	0.416821	0.588931
SCE1572_1851	0.679666912	-15.8111	0.416821	0.588931
SCE1572_9391	0.679666912	-15.8111	0.416821	0.588931
SCE1572_741	-0.667352163	-15.7476	0.416821	0.588931
SCE1572_8329	0.595309982	-14.6727	0.416911	0.588931
SCE1572_2163	0.816039354	-16.6498	0.416921	0.588931
SCE1572_4710	0.816039354	-16.6498	0.416921	0.588931
SCE1572_1543	0.548755475	-12.7834	0.417485	0.589648
SCE1572_6273	-0.643392264	-15.3351	0.41793	0.590038
SCE1572_8706	-0.615377888	-15.3211	0.41793	0.590038
SCE1572_11181	-0.615377888	-15.3211	0.41793	0.590038
SCE1572_7483	0.551117839	-13.0273	0.418481	0.59058
SCE1572_1875	0.566657951	-13.5846	0.41859	0.59058
SCE1572_8691	0.609932016	-14.5049	0.418623	0.59058
SCE1572_10529	-0.574815631	-14.4872	0.418623	0.59058
SCE1572_6559	-0.574815631	-14.4872	0.418623	0.59058
SCE1572_2078	0.591072989	-14.3028	0.418651	0.59058
SCE1572_164	0.676109093	-15.9124	0.419933	0.592151
SCE1572_4291	-0.665702426	-15.8671	0.419933	0.592151
SCE1572_9194	-0.665702426	-15.8671	0.419933	0.592151
SCE1572_10567	0.595938744	-14.5504	0.420144	0.59237
SCE1572_881	-0.55260524	-13.7491	0.420444	0.592635
SCE1572_5202	-0.640657297	-15.4239	0.420445	0.592635
SCE1572_1880	0.567032652	-13.8916	0.420631	0.592818
SCE1572_1867	0.591840176	-14.2073	0.421104	0.593239
SCE1572_11172	0.779202586	-16.8608	0.421156	0.593239
SCE1572_4094	0.779202586	-16.8608	0.421156	0.593239
SCE1572_3072	0.779202586	-16.8608	0.421156	0.593239
SCE1572_2739	-0.576278069	-14.3686	0.421407	0.593513
SCE1572_1824	-0.605160884	-14.8368	0.422202	0.594554
SCE1572_10274	-0.559768981	-14.2291	0.422429	0.594793
SCE1572_9445	0.619347443	-15.1631	0.422712	0.595113
SCE1572_5690	-0.637551386	-15.5186	0.423254	0.595636
SCE1572_8232	-0.605842526	-15.5027	0.423254	0.595636
SCE1572_1196	-0.605842526	-15.5027	0.423254	0.595636
SCE1572_9486	-0.709544599	-16.0202	0.42351	0.595837
SCE1572_6746	-0.709544599	-16.0202	0.42351	0.595837
SCE1572_792	-0.569328353	-14.2764	0.423827	0.596203
SCE1572_7011	0.570394845	-13.7502	0.424065	0.596458
SCE1572_2750	-0.584210928	-14.4672	0.42451	0.596765
SCE1572_10727	0.582725646	-14.481	0.42451	0.596765
SCE1572_9531	-0.569104036	-14.4597	0.42451	0.596765
SCE1572_2429	-0.569104036	-14.4597	0.42451	0.596765
SCE1572_3715	-0.538210028	-13.0601	0.424825	0.597128
SCE1572_1604	0.614405493	-15.227	0.42521	0.59751
SCE1572_10262	0.614405493	-15.227	0.42521	0.59751
SCE1572_10905	-0.544441981	-13.472	0.425416	0.597719

SCE1572_4434	0.569724919	-14.1886	0.425951	0.59839
SCE1572_9487	-0.633993566	-15.6199	0.42641	0.598716
SCE1572_2538	-0.633993566	-15.6199	0.42641	0.598716
SCE1572_9072	-0.600046235	-15.6029	0.42641	0.598716
SCE1572_7860	-0.600046235	-15.6029	0.42641	0.598716
SCE1572_4531	0.586557508	-14.7645	0.427377	0.599993
SCE1572_10115	-0.547121739	-13.4365	0.427503	0.599995
SCE1572_4107	0.760997991	-16.2178	0.427663	0.599995
SCE1572_7492	0.728576513	-16.2341	0.427663	0.599995
SCE1572_5714	-0.711996078	-16.1658	0.427663	0.599995
SCE1572_1163	-0.711996078	-16.1658	0.427663	0.599995
SCE1572_11191	-0.55401069	-13.6343	0.427935	0.600103
SCE1572_11437	-0.630965874	-15.2858	0.427969	0.600103
SCE1572_5070	-0.603998826	-15.2723	0.427969	0.600103
SCE1572_10616	-0.603998826	-15.2723	0.427969	0.600103
SCE1572_193	-0.547484573	-13.8558	0.428493	0.600613
SCE1572_6099	-0.547484573	-13.8558	0.428493	0.600613
SCE1572_5211	-0.538284672	-13.0885	0.428504	0.600613
SCE1572_7639	-0.564470462	-14.2525	0.428925	0.601123
SCE1572_3105	-0.555991054	-13.9959	0.429503	0.601853
SCE1572_3961	-0.531776303	-12.5755	0.429595	0.601903
SCE1572_9780	0.587978183	-14.6345	0.429976	0.602125
SCE1572_772	0.635467109	-15.74	0.429983	0.602125
SCE1572_2525	0.635467109	-15.74	0.429983	0.602125
SCE1572_9910	0.635467109	-15.74	0.429983	0.602125
SCE1572_5280	-0.560930104	-14.2938	0.430538	0.602566
SCE1572_5552	-1.856385988	-19.401	0.430642	0.602566
SCE1572_11205	-1.856385988	-19.401	0.430642	0.602566
SCE1572_6443	-1.856385988	-19.401	0.430642	0.602566
SCE1572_195	-1.856385988	-19.401	0.430642	0.602566
SCE1572_8162	-1.856385988	-19.401	0.430642	0.602566
SCE1572_9264	-0.552836422	-14.0304	0.430879	0.602789
SCE1572_336	0.654575932	-15.4086	0.43103	0.602789
SCE1572_190	0.635467109	-15.4181	0.43103	0.602789
SCE1572_1353	0.635467109	-15.4181	0.43103	0.602789
SCE1572_5101	0.608282279	-15.1098	0.431572	0.603386
SCE1572_7162	0.608282279	-15.1098	0.431572	0.603386
SCE1572_4448	1.050504608	-17.5325	0.431875	0.603493
SCE1572_1813	1.050504608	-17.5325	0.431875	0.603493
SCE1572_484	0.612062504	-14.8938	0.431878	0.603493
SCE1572_7773	-0.593351582	-14.8626	0.431878	0.603493
SCE1572_6202	0.547660728	-13.6547	0.432366	0.603702
SCE1572_2103	-0.547754715	-13.5635	0.432367	0.603702
SCE1572_8293	0.728576513	-16.3716	0.432544	0.603702
SCE1572_10547	0.728576513	-16.3716	0.432544	0.603702
SCE1572_4159	-0.715030139	-16.3278	0.432544	0.603702
SCE1572_3212	-0.715030139	-16.3278	0.432544	0.603702
SCE1572_9969	-0.715030139	-16.3278	0.432544	0.603702

SCE1572_11060	-0.65844661	-16.2995	0.432544	0.603702
SCE1572_5473	-0.65844661	-16.2995	0.432544	0.603702
SCE1572_10776	-0.525222628	-12.0304	0.43263	0.603742
SCE1572_746	-0.554595538	-13.6769	0.43351	0.604889
SCE1572_2366	0.548755475	-13.7834	0.433581	0.604909
SCE1572_7245	-0.582352583	-14.7342	0.434301	0.605636
SCE1572_5925	-0.582352583	-14.7342	0.434301	0.605636
SCE1572_4868	0.593646933	-14.9536	0.434415	0.605636
SCE1572_8740	0.630396119	-15.4947	0.434448	0.605636
SCE1572_6676	0.630396119	-15.4947	0.434448	0.605636
SCE1572_7970	-0.623725231	-15.4627	0.434448	0.605636
SCE1572_1876	-0.534457893	-13.3138	0.435055	0.606402
SCE1572_7707	0.549606372	-14.2911	0.43569	0.607206
SCE1572_3033	-0.525996314	-12.9114	0.435754	0.607216
SCE1572_9073	0.548368538	-13.7392	0.435932	0.607383
SCE1572_2088	0.558651512	-14.6085	0.43632	0.607763
SCE1572_9550	0.558651512	-14.6085	0.43632	0.607763
SCE1572_3305	-0.52984864	-13.3569	0.436992	0.608618
SCE1572_1146	0.561466527	-14.1921	0.437118	0.608712
SCE1572_4997	-0.606607679	-15.0035	0.437178	0.608716
SCE1572_6373	0.626696899	-15.285	0.437711	0.608747
SCE1572_1463	-0.544441981	-14.472	0.438028	0.608747
SCE1572_7361	-1.078778409	-17.7898	0.438083	0.608747
SCE1572_4180	-1.078778409	-17.7898	0.438083	0.608747
SCE1572_8070	1.051466084	-17.9471	0.438083	0.608747
SCE1572_1496	1.050504608	-17.9476	0.438083	0.608747
SCE1572_3401	1.050504608	-17.9476	0.438083	0.608747
SCE1572_3825	1.050504608	-17.9476	0.438083	0.608747
SCE1572_10526	1.050504608	-17.9476	0.438083	0.608747
SCE1572_5402	0.624740702	-15.5755	0.438288	0.608747
SCE1572_689	-0.61934679	-15.5601	0.438288	0.608747
SCE1572_6565	0.728576513	-16.5236	0.438358	0.608747
SCE1572_6528	0.728576513	-16.5236	0.438358	0.608747
SCE1572_4281	0.728576513	-16.5236	0.438358	0.608747
SCE1572_866	0.728576513	-16.5236	0.438358	0.608747
SCE1572_9393	0.728576513	-16.5236	0.438358	0.608747
SCE1572_4883	-0.718882464	-16.5103	0.438358	0.608747
SCE1572_4106	0.687934528	-16.5439	0.438358	0.608747
SCE1572_8975	0.687934528	-16.5439	0.438358	0.608747
SCE1572_10545	0.687934528	-16.5439	0.438358	0.608747
SCE1572_2316	0.700562137	-16.0071	0.438759	0.609223
SCE1572_5045	-0.54705793	-14.2228	0.43889	0.609324
SCE1572_8401	0.539420421	-13.9054	0.438993	0.609387
SCE1572_4876	0.565077781	-14.3829	0.439492	0.61
SCE1572_2330	0.542564527	-13.8323	0.439946	0.61055
SCE1572_7616	-0.53213284	-13.3696	0.440791	0.611641
SCE1572_10887	-0.575429674	-14.7016	0.441081	0.611963
SCE1572_4676	0.621661309	-15.3546	0.441283	0.612001

SCE1572_3607	0.621661309	-15.3546	0.441283	0.612001
SCE1572_2387	0.603045631	-15.3639	0.441283	0.612001
SCE1572_2241	0.550933598	-14.4248	0.441569	0.612317
SCE1572_2054	-0.526680542	-13.5267	0.441753	0.612492
SCE1572_9173	1.188008132	-18.4638	0.442579	0.612506
SCE1572_8571	1.188008132	-18.4638	0.442579	0.612506
SCE1572_7780	1.188008132	-18.4638	0.442579	0.612506
SCE1572_6767	1.188008132	-18.4638	0.442579	0.612506
SCE1572_10035	1.188008132	-18.4638	0.442579	0.612506
SCE1572_461	1.188008132	-18.4638	0.442579	0.612506
SCE1572_8446	1.050504608	-18.5325	0.442579	0.612506
SCE1572_11365	1.050504608	-18.5325	0.442579	0.612506
SCE1572_2859	1.050504608	-18.5325	0.442579	0.612506
SCE1572_10637	1.050504608	-18.5325	0.442579	0.612506
SCE1572_8136	1.050504608	-18.5325	0.442579	0.612506
SCE1572_8443	1.050504608	-18.5325	0.442579	0.612506
SCE1572_3282	1.050504608	-18.5325	0.442579	0.612506
SCE1572_10127	1.050504608	-18.5325	0.442579	0.612506
SCE1572_9326	-0.516535985	-12.7311	0.442662	0.61254
SCE1572_2358	-0.542071077	-14.0809	0.44285	0.61272
SCE1572_11443	0.574066564	-14.7707	0.443737	0.613816
SCE1572_10995	0.557691954	-14.2243	0.443759	0.613816
SCE1572_3797	-0.524709916	-13.4375	0.444154	0.614227
SCE1572_3286	0.667175968	-16.1392	0.444231	0.614227
SCE1572_9255	0.667175968	-16.1392	0.444231	0.614227
SCE1572_9742	-0.52546911	-13.7355	0.444366	0.614332
SCE1572_10268	0.538915293	-13.6798	0.444994	0.615103
SCE1572_5947	0.565077781	-14.646	0.44504	0.615103
SCE1572_2970	-0.711837549	-16.7133	0.445397	0.615505
SCE1572_3463	0.535931435	-14.0349	0.445448	0.615505
SCE1572_4489	-0.551531406	-14.2461	0.445753	0.615795
SCE1572_9206	0.532757187	-13.769	0.445834	0.615795
SCE1572_11445	-0.522231591	-13.7643	0.445834	0.615795
SCE1572_7113	0.578681082	-14.5205	0.446133	0.616046
SCE1572_10542	0.578681082	-14.5205	0.446133	0.616046
SCE1572_5812	0.515947061	-12.723	0.446234	0.616105
SCE1572_6856	0.580053988	-15.2442	0.447167	0.617312
SCE1572_59	0.654575932	-15.8236	0.447595	0.617416
SCE1572_4895	0.654575932	-15.8236	0.447595	0.617416
SCE1572_8619	0.654575932	-15.8236	0.447595	0.617416
SCE1572_11441	-0.646932622	-15.7963	0.447595	0.617416
SCE1572_1639	-0.608458474	-15.777	0.447595	0.617416
SCE1572_3281	-0.608458474	-15.777	0.447595	0.617416
SCE1572_1089	0.559976909	-14.6903	0.447721	0.61751
SCE1572_10855	0.540504165	-14.2957	0.447861	0.617621
SCE1572_9031	0.524106	-13.6664	0.448354	0.618221
SCE1572_10507	-0.52776324	-13.8954	0.44845	0.618272
SCE1572_3233	0.597992403	-15.0583	0.448598	0.618314

SCE1572_6164	0.582725646	-15.066	0.448598	0.618314
SCE1572_5655	0.556395538	-14.4577	0.449426	0.619212
SCE1572_9448	0.556395538	-14.4577	0.449426	0.619212
SCE1572_7145	0.556395538	-14.4577	0.449426	0.619212
SCE1572_517	0.598515972	-14.9005	0.449714	0.619284
SCE1572_2666	-0.560930104	-14.8788	0.449714	0.619284
SCE1572_11091	-0.560930104	-14.8788	0.449714	0.619284
SCE1572_44	-0.560930104	-14.8788	0.449714	0.619284
SCE1572_8730	0.535931435	-14.146	0.45117	0.620972
SCE1572_5574	0.535931435	-14.146	0.45117	0.620972
SCE1572_5387	-0.530510708	-14.1332	0.45117	0.620972
SCE1572_6252	-0.520303799	-13.1682	0.451258	0.620972
SCE1572_1486	-0.556400446	-14.6081	0.451337	0.620972
SCE1572_2123	-0.539912323	-14.5998	0.451337	0.620972
SCE1572_5482	-0.509002312	-12.7348	0.451353	0.620972
SCE1572_8035	0.573042514	-15.1274	0.452285	0.622092
SCE1572_1331	0.560453754	-15.1337	0.452285	0.622092
SCE1572_7559	0.555427611	-14.3877	0.452459	0.622168
SCE1572_9489	0.555427611	-14.3877	0.452459	0.622168
SCE1572_10693	0.565077781	-14.9679	0.453092	0.622931
SCE1572_3959	0.649142045	-15.9259	0.453309	0.622931
SCE1572_7388	0.621661309	-15.9396	0.453309	0.622931
SCE1572_1713	-0.601572089	-15.8991	0.453309	0.622931
SCE1572_8338	0.518803022	-13.2598	0.45331	0.622931
SCE1572_2244	0.568796069	-14.8192	0.453721	0.623415
SCE1572_2483	0.84405373	-17.0508	0.454082	0.623503
SCE1572_10695	0.84405373	-17.0508	0.454082	0.623503
SCE1572_5951	0.84405373	-17.0508	0.454082	0.623503
SCE1572_913	-0.730855106	-16.9638	0.454082	0.623503
SCE1572_5285	-0.730855106	-16.9638	0.454082	0.623503
SCE1572_678	-0.534457893	-14.6526	0.454224	0.623616
SCE1572_4857	0.641113672	-15.6498	0.454799	0.624081
SCE1572_6703	0.641113672	-15.6498	0.454799	0.624081
SCE1572_234	0.618393595	-15.6611	0.454799	0.624081
SCE1572_6225	0.618393595	-15.6611	0.454799	0.624081
SCE1572_5348	-0.496983187	-12.0583	0.455125	0.624446
SCE1572_4240	0.616101784	-15.4278	0.455728	0.625111
SCE1572_11284	-0.581763608	-15.3945	0.455728	0.625111
SCE1572_1352	-0.523402704	-14.0341	0.455902	0.625267
SCE1572_3252	0.49845673	-12.2012	0.456619	0.62617
SCE1572_5677	-0.570983769	-15.0213	0.456779	0.626225
SCE1572_1139	0.558651512	-15.0236	0.456779	0.626225
SCE1572_1479	0.563152904	-14.8693	0.457093	0.626411
SCE1572_11391	0.563152904	-14.8693	0.457093	0.626411
SCE1572_9749	-0.553823218	-14.8428	0.457093	0.626411
SCE1572_794	-0.500242178	-13.0791	0.457249	0.626489
SCE1572_9018	0.554547113	-14.7361	0.457328	0.626489
SCE1572_8932	-0.546283328	-14.7162	0.457328	0.626489

SCE1572_11509	0.529267705	-13.8389	0.458134	0.627511
SCE1572_1117	-0.747861531	-16.4317	0.45842	0.627821
SCE1572_10040	-0.509583224	-13.789	0.459959	0.629382
SCE1572_11589	-0.639155272	-16.0554	0.459963	0.629382
SCE1572_1710	-0.639155272	-16.0554	0.459963	0.629382
SCE1572_6794	-0.639155272	-16.0554	0.459963	0.629382
SCE1572_3689	-0.639155272	-16.0554	0.459963	0.629382
SCE1572_9143	0.496404535	-12.5204	0.459972	0.629382
SCE1572_11514	0.522125635	-14.3049	0.460345	0.629382
SCE1572_242	-0.529735483	-14.3919	0.460424	0.629382
SCE1572_8631	0.54673819	-14.5365	0.460507	0.629382
SCE1572_10572	-0.629877458	-15.7289	0.460565	0.629382
SCE1572_2181	0.611219562	-15.7522	0.460565	0.629382
SCE1572_6558	0.611219562	-15.7522	0.460565	0.629382
SCE1572_11027	0.586557508	-15.7645	0.460565	0.629382
SCE1572_9067	0.586557508	-15.7645	0.460565	0.629382
SCE1572_3977	0.548755475	-14.7834	0.460677	0.629382
SCE1572_10436	-0.54061012	-14.7735	0.460677	0.629382
SCE1572_655	-0.488790142	-11.0806	0.46078	0.629382
SCE1572_8626	0.609932016	-15.5049	0.460787	0.629382
SCE1572_10483	0.589173456	-15.5153	0.460787	0.629382
SCE1572_7480	-0.56515469	-15.0924	0.460817	0.629382
SCE1572_2288	-0.542071077	-15.0809	0.460817	0.629382
SCE1572_8255	-0.500047862	-12.8649	0.461311	0.629975
SCE1572_10410	-0.519351	-13.7476	0.461688	0.630408
SCE1572_5222	0.501691111	-12.7408	0.461973	0.630716
SCE1572_5018	-0.493144419	-12.8205	0.46312	0.6322
SCE1572_8842	-0.540240245	-14.684	0.463906	0.63319
SCE1572_9750	-0.534457893	-14.8332	0.464298	0.633585
SCE1572_5527	0.514072001	-13.5528	0.464393	0.633585
SCE1572_3372	-0.562886301	-14.9817	0.464747	0.633585
SCE1572_978	-0.54151265	-14.971	0.464747	0.633585
SCE1572_4414	0.866080037	-17.3028	0.465039	0.633585
SCE1572_8819	0.866080037	-17.3028	0.465039	0.633585
SCE1572_7553	0.866080037	-17.3028	0.465039	0.633585
SCE1572_5853	0.825512995	-17.3231	0.465039	0.633585
SCE1572_11592	0.798965841	-17.3364	0.465039	0.633585
SCE1572_8229	0.798965841	-17.3364	0.465039	0.633585
SCE1572_9970	0.798965841	-17.3364	0.465039	0.633585
SCE1572_310	0.798965841	-17.3364	0.465039	0.633585
SCE1572_5509	0.798965841	-17.3364	0.465039	0.633585
SCE1572_2337	0.798965841	-17.3364	0.465039	0.633585
SCE1572_9967	0.586557508	-15.1795	0.465259	0.633721
SCE1572_5308	-0.534457893	-15.1551	0.465259	0.633721
SCE1572_5891	-0.598998145	-15.3573	0.465843	0.634188
SCE1572_2649	0.584186604	-15.3734	0.465843	0.634188
SCE1572_3303	-0.570983769	-15.3433	0.465843	0.634188
SCE1572_11473	0.565077781	-15.3829	0.465843	0.634188

SCE1572_11425	-0.494491486	-12.8293	0.466006	0.634327
SCE1572_9221	0.486819946	-11.745	0.466164	0.634392
SCE1572_10645	-0.510034419	-13.9552	0.466174	0.634392
SCE1572_11150	-0.519351	-14.4846	0.466269	0.63444
SCE1572_11212	0.581019325	-15.5973	0.466499	0.634588
SCE1572_2331	0.581019325	-15.5973	0.466499	0.634588
SCE1572_5537	0.535931435	-14.6199	0.46683	0.634956
SCE1572_7874	-0.625060442	-15.8467	0.467198	0.635114
SCE1572_7142	-0.499043708	-13.8327	0.467486	0.635114
SCE1572_5977	0.695409649	-16.2506	0.467814	0.635114
SCE1572_3507	0.772970632	-16.6713	0.467852	0.635114
SCE1572_1436	0.772970632	-16.6713	0.467852	0.635114
SCE1572_6306	0.772970632	-16.6713	0.467852	0.635114
SCE1572_3794	0.772970632	-16.6713	0.467852	0.635114
SCE1572_4005	-0.756850314	-16.6288	0.467852	0.635114
SCE1572_640	-0.756850314	-16.6288	0.467852	0.635114
SCE1572_3883	0.728576513	-16.6935	0.467852	0.635114
SCE1572_7757	0.728576513	-16.6935	0.467852	0.635114
SCE1572_8131	0.728576513	-16.6935	0.467852	0.635114
SCE1572_7452	-0.686460986	-16.5937	0.467852	0.635114
SCE1572_9211	-0.686460986	-16.5937	0.467852	0.635114
SCE1572_2809	0.663189062	-16.6644	0.467852	0.635114
SCE1572_4754	-0.516535985	-13.9941	0.468424	0.635808
SCE1572_10034	0.509936226	-13.8028	0.469075	0.636499
SCE1572_10024	0.57533982	-15.0697	0.469114	0.636499
SCE1572_5386	0.567295606	-15.0737	0.469114	0.636499
SCE1572_2984	0.505198307	-13.8744	0.469589	0.637061
SCE1572_8966	-0.504084244	-13.7552	0.470579	0.63824
SCE1572_9006	-0.504084244	-13.7552	0.470579	0.63824
SCE1572_3320	-0.494801693	-13.3744	0.470709	0.638334
SCE1572_3249	-0.525180079	-14.3662	0.471039	0.638699
SCE1572_6112	0.555739916	-14.8257	0.471336	0.638918
SCE1572_3979	-0.54705793	-14.8077	0.471336	0.638918
SCE1572_258	-0.593351582	-15.4476	0.471443	0.638918
SCE1572_6138	-0.593351582	-15.4476	0.471443	0.638918
SCE1572_2748	-0.498833983	-13.4003	0.472304	0.640002
SCE1572_3392	-0.481174469	-12.0552	0.472527	0.640221
SCE1572_1504	-0.491204695	-13.7798	0.472635	0.640285
SCE1572_430	0.498753886	-13.6186	0.472743	0.640349
SCE1572_11446	-0.478656101	-12.5624	0.474349	0.642441
SCE1572_599	0.671992985	-16.0213	0.474911	0.642872
SCE1572_4326	0.642846639	-16.0359	0.474911	0.642872
SCE1572_8627	0.642846639	-16.0359	0.474911	0.642872
SCE1572_5619	-0.61934679	-15.9751	0.474911	0.642872
SCE1572_9737	-0.52140174	-14.859	0.475519	0.643613
SCE1572_802	-0.490433269	-13.1532	0.476199	0.644417
SCE1572_6811	0.510396343	-13.9697	0.47649	0.644417
SCE1572_9659	-0.480010109	-13.0124	0.4768	0.644417

SCE1572_11483	-0.480010109	-13.0124	0.4768	0.644417
SCE1572_11297	-0.485725321	-12.9519	0.477062	0.644417
SCE1572_10940	0.692050637	-16.3898	0.477216	0.644417
SCE1572_2516	0.692050637	-16.3898	0.477216	0.644417
SCE1572_10828	0.692050637	-16.3898	0.477216	0.644417
SCE1572_1553	0.692050637	-16.3898	0.477216	0.644417
SCE1572_2099	0.692050637	-16.3898	0.477216	0.644417
SCE1572_5336	0.692050637	-16.3898	0.477216	0.644417
SCE1572_4729	0.654575932	-16.4086	0.477216	0.644417
SCE1572_6471	0.654575932	-16.4086	0.477216	0.644417
SCE1572_4902	0.654575932	-16.4086	0.477216	0.644417
SCE1572_4674	0.654575932	-16.4086	0.477216	0.644417
SCE1572_6318	0.654575932	-16.4086	0.477216	0.644417
SCE1572_9697	-0.570983769	-16.3433	0.477216	0.644417
SCE1572_5310	-0.570983769	-16.3433	0.477216	0.644417
SCE1572_395	-0.514694638	-14.4571	0.47747	0.644454
SCE1572_8005	-0.500047862	-14.4498	0.47747	0.644454
SCE1572_10466	0.505070471	-14.0504	0.477489	0.644454
SCE1572_7420	0.497250967	-14.0543	0.477489	0.644454
SCE1572_8695	-0.555216453	-15.528	0.477755	0.644731
SCE1572_10312	0.486501726	-12.872	0.478011	0.644993
SCE1572_4217	-0.499375434	-14.2851	0.478105	0.645036
SCE1572_8403	0.496760838	-13.8094	0.478356	0.645293
SCE1572_154	-0.534457893	-14.5701	0.478927	0.645503
SCE1572_10788	0.519123147	-14.5888	0.478927	0.645503
SCE1572_11466	0.519123147	-14.5888	0.478927	0.645503
SCE1572_9761	-0.908853408	-17.7048	0.479207	0.645503
SCE1572_6687	-0.756850314	-17.6288	0.479207	0.645503
SCE1572_2389	-0.768923147	-16.8573	0.479616	0.645503
SCE1572_3639	-0.768923147	-16.8573	0.479616	0.645503
SCE1572_3635	0.728576513	-16.8861	0.479616	0.645503
SCE1572_4949	0.728576513	-16.8861	0.479616	0.645503
SCE1572_7737	-0.686460986	-16.816	0.479616	0.645503
SCE1572_11105	-0.686460986	-16.816	0.479616	0.645503
SCE1572_9289	-0.686460986	-16.816	0.479616	0.645503
SCE1572_5303	0.676109093	-16.9124	0.479616	0.645503
SCE1572_3348	0.676109093	-16.9124	0.479616	0.645503
SCE1572_6663	0.676109093	-16.9124	0.479616	0.645503
SCE1572_10708	0.676109093	-16.9124	0.479616	0.645503
SCE1572_3677	0.676109093	-16.9124	0.479616	0.645503
SCE1572_3848	0.676109093	-16.9124	0.479616	0.645503
SCE1572_4503	0.514451708	-14.9426	0.480071	0.645923
SCE1572_3941	-0.464365141	-9.84909	0.480092	0.645923
SCE1572_848	0.465290745	-10.6602	0.480112	0.645923
SCE1572_7621	0.629040839	-15.8364	0.480463	0.645981
SCE1572_7233	0.629040839	-15.8364	0.480463	0.645981
SCE1572_8974	0.629040839	-15.8364	0.480463	0.645981
SCE1572_8305	0.629040839	-15.8364	0.480463	0.645981

SCE1572_543	0.603045631	-15.8494	0.480463	0.645981
SCE1572_4229	0.523756721	-14.7084	0.480626	0.646117
SCE1572_1183	0.596472977	-15.4376	0.481726	0.647349
SCE1572_450	0.596472977	-15.4376	0.481726	0.647349
SCE1572_10874	0.57657342	-15.4476	0.481726	0.647349
SCE1572_9895	-0.491389171	-13.6741	0.481978	0.647604
SCE1572_10431	0.512847822	-14.6314	0.482809	0.648638
SCE1572_8996	-0.507051736	-14.1215	0.483017	0.648834
SCE1572_6377	0.635467109	-16.1551	0.483994	0.649317
SCE1572_6755	0.635467109	-16.1551	0.483994	0.649317
SCE1572_37	-0.612460405	-16.1161	0.483994	0.649317
SCE1572_9816	-0.612460405	-16.1161	0.483994	0.649317
SCE1572_3631	0.603045631	-16.1713	0.483994	0.649317
SCE1572_10007	0.603045631	-16.1713	0.483994	0.649317
SCE1572_11272	0.603045631	-16.1713	0.483994	0.649317
SCE1572_6121	0.603045631	-16.1713	0.483994	0.649317
SCE1572_6996	0.603045631	-16.1713	0.483994	0.649317
SCE1572_11117	0.603045631	-16.1713	0.483994	0.649317
SCE1572_11359	0.595309982	-15.6727	0.484925	0.649961
SCE1572_5524	0.595309982	-15.6727	0.484925	0.649961
SCE1572_9617	0.595309982	-15.6727	0.484925	0.649961
SCE1572_5520	-0.579545782	-15.6471	0.484925	0.649961
SCE1572_10407	-0.54559845	-15.6301	0.484925	0.649961
SCE1572_11088	-0.54559845	-15.6301	0.484925	0.649961
SCE1572_11426	-0.54559845	-15.6301	0.484925	0.649961
SCE1572_5780	-0.527262391	-14.9995	0.48504	0.649961
SCE1572_1608	0.572925642	-15.3119	0.485092	0.649961
SCE1572_10408	-0.534457893	-15.2806	0.485092	0.649961
SCE1572_7235	-0.490989864	-13.8939	0.485562	0.650509
SCE1572_5990	0.474259324	-13.1913	0.487759	0.653263
SCE1572_2497	0.495915756	-14.2552	0.487769	0.653263
SCE1572_2755	0.490127745	-13.8127	0.487804	0.653263
SCE1572_10353	-0.456245206	-10.111	0.488171	0.653607
SCE1572_749	-0.476537917	-13.921	0.488185	0.653607
SCE1572_5635	0.462221749	-11.7988	0.488416	0.653779
SCE1572_11225	0.568111841	-15.5258	0.48859	0.653779
SCE1572_8963	0.568111841	-15.5258	0.48859	0.653779
SCE1572_9860	0.489388849	-14.0582	0.488629	0.653779
SCE1572_4280	0.646114353	-16.5648	0.488687	0.653779
SCE1572_898	0.646114353	-16.5648	0.488687	0.653779
SCE1572_5873	0.485082294	-13.7708	0.488834	0.653893
SCE1572_9210	0.529267705	-14.8389	0.489574	0.654632
SCE1572_299	0.529267705	-14.8389	0.489574	0.654632
SCE1572_10018	0.529267705	-14.8389	0.489574	0.654632
SCE1572_3496	-0.471448095	-13.4861	0.489789	0.654837
SCE1572_7646	-0.468869551	-12.8659	0.489975	0.655003
SCE1572_2062	0.551698751	-15.0815	0.490487	0.65552
SCE1572_2818	0.535931435	-15.0894	0.490487	0.65552

SCE1572_8616	0.529672445	-14.7485	0.491483	0.656684
SCE1572_4882	0.517072408	-14.7548	0.491483	0.656684
SCE1572_2682	0.545712456	-15.3926	0.491653	0.656828
SCE1572_886	-0.4510882	-9.34668	0.492137	0.657391
SCE1572_10497	0.521134433	-14.9899	0.492683	0.658037
SCE1572_11314	-0.465064557	-12.5968	0.493019	0.658396
SCE1572_4083	-0.570983769	-15.7583	0.49314	0.658396
SCE1572_4099	-0.534457893	-15.74	0.49314	0.658396
SCE1572_7027	-0.467075856	-13.4087	0.494046	0.659295
SCE1572_3717	-0.456689035	-12.6788	0.494173	0.659295
SCE1572_9884	0.54486456	-15.2618	0.494252	0.659295
SCE1572_6613	0.526942652	-15.2707	0.494252	0.659295
SCE1572_7282	0.526942652	-15.2707	0.494252	0.659295
SCE1572_10994	-0.525996314	-15.2333	0.494252	0.659295
SCE1572_10498	-0.525996314	-15.2333	0.494252	0.659295
SCE1572_9265	-0.46536537	-12.9776	0.494637	0.65961
SCE1572_2668	-0.686460986	-17.0791	0.494712	0.65961
SCE1572_4283	-0.686460986	-17.0791	0.494712	0.65961
SCE1572_1100	-0.507490845	-14.5836	0.494757	0.65961
SCE1572_10458	0.497963585	-14.3494	0.494801	0.65961
SCE1572_9921	-0.477284184	-13.7418	0.495611	0.660605
SCE1572_358	0.492509155	-14.0836	0.495854	0.660845
SCE1572_9188	0.499635306	-14.2844	0.496003	0.660862
SCE1572_8802	0.458614387	-12.4448	0.496287	0.660862
SCE1572_5487	0.509936226	-14.8028	0.496348	0.660862
SCE1572_951	-0.509583224	-14.789	0.496348	0.660862
SCE1572_5090	-0.509583224	-14.789	0.496348	0.660862
SCE1572_4453	-0.509583224	-14.789	0.496348	0.660862
SCE1572_6370	0.558651512	-15.6085	0.496369	0.660862
SCE1572_3274	0.535931435	-15.6199	0.496369	0.660862
SCE1572_3954	0.527663819	-15.1501	0.496484	0.660932
SCE1572_5696	0.481739335	-13.8627	0.496597	0.660998
SCE1572_3346	0.456323577	-12.535	0.497159	0.661662
SCE1572_3632	0.490484065	-14.4469	0.497463	0.661819
SCE1572_10758	0.499094667	-14.7208	0.497893	0.661819
SCE1572_278	1.313539014	-18.401	0.497906	0.661819
SCE1572_7317	1.313539014	-18.401	0.497906	0.661819
SCE1572_8905	1.313539014	-18.401	0.497906	0.661819
SCE1572_1734	1.313539014	-18.401	0.497906	0.661819
SCE1572_4407	1.313539014	-18.401	0.497906	0.661819
SCE1572_3481	1.313539014	-18.401	0.497906	0.661819
SCE1572_6446	1.313539014	-18.401	0.497906	0.661819
SCE1572_7421	1.313539014	-18.401	0.497906	0.661819
SCE1572_10325	-0.501721106	-14.3779	0.498646	0.66272
SCE1572_10496	-0.544441981	-15.472	0.499021	0.66305
SCE1572_2875	-0.514694638	-15.4571	0.499021	0.66305
SCE1572_10791	-0.471361058	-13.8841	0.499418	0.663493
SCE1572_5761	0.499952138	-14.9498	0.500122	0.664345

SCE1572_3483	-0.465861847	-13.4452	0.500954	0.665224
SCE1572_3397	0.462061538	-13.4517	0.500954	0.665224
SCE1572_810	0.462682453	-13.1682	0.500973	0.665224
SCE1572_4021	-0.543503032	-15.3295	0.501238	0.665492
SCE1572_6052	-0.444721359	-11.4504	0.501508	0.665766
SCE1572_9776	-0.493815908	-14.4973	0.501691	0.665925
SCE1572_10825	-0.464068565	-13.2898	0.503432	0.668152
SCE1572_1261	-0.476742395	-14.6814	0.504157	0.66903
SCE1572_525	-0.526680542	-15.1117	0.504734	0.669626
SCE1572_3958	-0.503596929	-15.1001	0.504734	0.669626
SCE1572_1338	0.476268514	-13.7536	0.504921	0.66979
SCE1572_2020	-0.557727672	-15.6928	0.505261	0.670072
SCE1572_8424	-0.557727672	-15.6928	0.505261	0.670072
SCE1572_10351	0.48600621	-14.4225	0.507914	0.673505
SCE1572_1180	-0.593351582	-16.4476	0.508053	0.673604
SCE1572_3091	0.556395538	-15.4577	0.50907	0.674782
SCE1572_11127	0.556395538	-15.4577	0.50907	0.674782
SCE1572_11429	0.481138962	-14.2936	0.509345	0.675062
SCE1572_4162	0.506184092	-14.6754	0.510281	0.676164
SCE1572_9636	-0.448728019	-13.0825	0.510436	0.676164
SCE1572_10305	0.554547113	-15.3211	0.510498	0.676164
SCE1572_7657	0.554547113	-15.3211	0.510498	0.676164
SCE1572_3886	0.535931435	-15.3304	0.510498	0.676164
SCE1572_10786	0.661462317	-16.2676	0.511408	0.676518
SCE1572_10339	0.661462317	-16.2676	0.511408	0.676518
SCE1572_6953	0.661462317	-16.2676	0.511408	0.676518
SCE1572_6213	0.661462317	-16.2676	0.511408	0.676518
SCE1572_6247	0.661462317	-16.2676	0.511408	0.676518
SCE1572_3701	0.661462317	-16.2676	0.511408	0.676518
SCE1572_8823	0.661462317	-16.2676	0.511408	0.676518
SCE1572_5140	0.626696899	-16.285	0.511408	0.676518
SCE1572_9171	0.626696899	-16.285	0.511408	0.676518
SCE1572_6620	0.626696899	-16.285	0.511408	0.676518
SCE1572_9641	0.46384382	-13.4165	0.511683	0.676741
SCE1572_6353	0.535931435	-15.2048	0.511706	0.676741
SCE1572_6641	0.519989891	-15.0973	0.51274	0.677938
SCE1572_737	0.503870226	-15.1054	0.51274	0.677938
SCE1572_9783	-0.464068565	-14.3829	0.512992	0.678187
SCE1572_1194	-0.451995733	-13.8744	0.513345	0.678207
SCE1572_1711	0.475128156	-14.3284	0.51353	0.678207
SCE1572_4495	0.506184092	-14.9973	0.513635	0.678207
SCE1572_1969	0.613099296	-16.0508	0.513776	0.678207
SCE1572_3167	-0.548957463	-16.0103	0.513776	0.678207
SCE1572_3523	-0.507863683	-14.8854	0.514417	0.678207
SCE1572_2884	-0.507863683	-14.8854	0.514417	0.678207
SCE1572_6171	0.494111259	-14.9038	0.514417	0.678207
SCE1572_8618	0.494111259	-14.9038	0.514417	0.678207
SCE1572_8603	-0.488234876	-14.8756	0.514417	0.678207

SCE1572_3798	-0.488234876	-14.8756	0.514417	0.678207
SCE1572_6997	0.976504026	-17.5695	0.514813	0.678207
SCE1572_9813	0.976504026	-17.5695	0.514813	0.678207
SCE1572_4913	0.976504026	-17.5695	0.514813	0.678207
SCE1572_11524	0.976504026	-17.5695	0.514813	0.678207
SCE1572_5411	0.976504026	-17.5695	0.514813	0.678207
SCE1572_4951	0.976504026	-17.5695	0.514813	0.678207
SCE1572_5913	0.976504026	-17.5695	0.514813	0.678207
SCE1572_417	0.898501514	-17.6085	0.514813	0.678207
SCE1572_148	0.898501514	-17.6085	0.514813	0.678207
SCE1572_11078	0.898501514	-17.6085	0.514813	0.678207
SCE1572_6612	0.898501514	-17.6085	0.514813	0.678207
SCE1572_1324	0.898501514	-17.6085	0.514813	0.678207
SCE1572_1690	0.898501514	-17.6085	0.514813	0.678207
SCE1572_75	0.898501514	-17.6085	0.514813	0.678207
SCE1572_2363	0.898501514	-17.6085	0.514813	0.678207
SCE1572_9769	-0.811991868	-17.4638	0.514813	0.678207
SCE1572_7959	-0.811991868	-17.4638	0.514813	0.678207
SCE1572_5956	0.57657342	-15.8626	0.515523	0.678717
SCE1572_4646	0.57657342	-15.8626	0.515523	0.678717
SCE1572_3183	-0.54705793	-15.8077	0.515523	0.678717
SCE1572_5323	-0.54705793	-15.8077	0.515523	0.678717
SCE1572_9763	-0.54705793	-15.8077	0.515523	0.678717
SCE1572_2655	0.474003686	-14.7333	0.515719	0.678891
SCE1572_7830	0.448468594	-13.4242	0.51669	0.679625
SCE1572_4734	-0.475781986	-14.5677	0.516759	0.679625
SCE1572_5996	0.469333686	-14.5752	0.516759	0.679625
SCE1572_4293	0.469333686	-14.5752	0.516759	0.679625
SCE1572_7871	0.571851009	-15.6844	0.516859	0.679625
SCE1572_10453	0.548004267	-15.6963	0.516859	0.679625
SCE1572_10917	-0.489014922	-15.6584	0.516859	0.679625
SCE1572_5675	-0.489014922	-15.6584	0.516859	0.679625
SCE1572_2908	-0.489014922	-15.6584	0.516859	0.679625
SCE1572_11478	-0.440808659	-12.8945	0.517693	0.680581
SCE1572_10510	0.54673819	-15.5365	0.517909	0.680581
SCE1572_9805	0.54673819	-15.5365	0.517909	0.680581
SCE1572_3795	0.525043119	-15.5473	0.517909	0.680581
SCE1572_4114	-0.524189557	-15.5125	0.517909	0.680581
SCE1572_2170	-0.449568995	-13.6532	0.518093	0.680737
SCE1572_3951	-0.454287544	-14.3076	0.518312	0.68094
SCE1572_6389	-0.457836611	-14.2423	0.518622	0.681263
SCE1572_6753	0.526083649	-15.4024	0.518754	0.681265
SCE1572_10345	0.506184092	-15.4124	0.518754	0.681265
SCE1572_9582	0.471418673	-14.2074	0.518908	0.681383
SCE1572_9407	-0.466974296	-14.1415	0.519173	0.681527
SCE1572_5936	0.508795305	-15.2798	0.519446	0.681527
SCE1572_2778	-0.482927592	-15.2548	0.519446	0.681527
SCE1572_5558	-0.423920143	-10.2346	0.519664	0.681527

SCE1572_9124	0.494111259	-15.1669	0.520023	0.681527
SCE1572_5025	0.494111259	-15.1669	0.520023	0.681527
SCE1572_9437	-0.444002942	-13.6074	0.521279	0.681527
SCE1572_11408	0.787470202	-17.0791	0.521385	0.681527
SCE1572_7685	0.787470202	-17.0791	0.521385	0.681527
SCE1572_4589	0.787470202	-17.0791	0.521385	0.681527
SCE1572_4717	0.787470202	-17.0791	0.521385	0.681527
SCE1572_1214	0.787470202	-17.0791	0.521385	0.681527
SCE1572_5345	0.728576513	-17.1085	0.521385	0.681527
SCE1572_8935	0.728576513	-17.1085	0.521385	0.681527
SCE1572_4306	0.728576513	-17.1085	0.521385	0.681527
SCE1572_7753	0.728576513	-17.1085	0.521385	0.681527
SCE1572_1650	0.728576513	-17.1085	0.521385	0.681527
SCE1572_6020	-0.442153789	-13.4715	0.521718	0.681527
SCE1572_6327	1.728576513	-19.1935	0.521739	0.681527
SCE1572_208	1.728576513	-19.1935	0.521739	0.681527
SCE1572_7670	1.728576513	-19.1935	0.521739	0.681527
SCE1572_10746	1.728576513	-19.1935	0.521739	0.681527
SCE1572_506	1.728576513	-19.1935	0.521739	0.681527
SCE1572_9876	1.728576513	-19.1935	0.521739	0.681527
SCE1572_1997	1.728576513	-19.1935	0.521739	0.681527
SCE1572_8717	1.728576513	-19.1935	0.521739	0.681527
SCE1572_10610	1.728576513	-19.1935	0.521739	0.681527
SCE1572_177	1.728576513	-19.1935	0.521739	0.681527
SCE1572_5747	1.728576513	-19.1935	0.521739	0.681527
SCE1572_2952	1.726772016	-19.1944	0.521739	0.681527
SCE1572_1049	1.535931435	-19.2898	0.521739	0.681527
SCE1572_786	1.535931435	-19.2898	0.521739	0.681527
SCE1572_7123	1.535931435	-19.2898	0.521739	0.681527
SCE1572_7999	1.535931435	-19.2898	0.521739	0.681527
SCE1572_6743	1.535931435	-19.2898	0.521739	0.681527
SCE1572_3569	1.535931435	-19.2898	0.521739	0.681527
SCE1572_3248	1.535931435	-19.2898	0.521739	0.681527
SCE1572_5968	1.535931435	-19.2898	0.521739	0.681527
SCE1572_2480	1.535931435	-19.2898	0.521739	0.681527
SCE1572_4254	1.535931435	-19.2898	0.521739	0.681527
SCE1572_8671	1.535931435	-19.2898	0.521739	0.681527
SCE1572_712	1.535931435	-19.2898	0.521739	0.681527
SCE1572_11361	-0.468115398	-14.6194	0.522119	0.681939
SCE1572_5059	-0.464068565	-14.4825	0.522531	0.682392
SCE1572_4685	-0.432704394	-12.9887	0.522925	0.682821
SCE1572_7336	0.45440155	-14.1301	0.523373	0.683321
SCE1572_5417	-0.438242578	-13.834	0.523886	0.683907
SCE1572_6891	0.682772823	-16.7164	0.524474	0.68391
SCE1572_4167	0.682772823	-16.7164	0.524474	0.68391
SCE1572_4687	0.682772823	-16.7164	0.524474	0.68391
SCE1572_3650	0.682772823	-16.7164	0.524474	0.68391
SCE1572_1730	-0.64993511	-16.6823	0.524474	0.68391

SCE1572_5239	-0.64993511	-16.6823	0.524474	0.68391
SCE1572_2175	-0.64993511	-16.6823	0.524474	0.68391
SCE1572_8272	-0.579545782	-16.6471	0.524474	0.68391
SCE1572_4852	-0.579545782	-16.6471	0.524474	0.68391
SCE1572_4611	0.616101784	-16.4278	0.526087	0.685673
SCE1572_7312	0.616101784	-16.4278	0.526087	0.685673
SCE1572_4872	0.616101784	-16.4278	0.526087	0.685673
SCE1572_3952	0.616101784	-16.4278	0.526087	0.685673
SCE1572_2696	0.569878767	-16.1879	0.526983	0.686073
SCE1572_11409	0.569878767	-16.1879	0.526983	0.686073
SCE1572_6621	0.569878767	-16.1879	0.526983	0.686073
SCE1572_2767	-0.534457893	-16.1551	0.526983	0.686073
SCE1572_10167	-0.44475509	-13.5522	0.527127	0.686073
SCE1572_4849	-0.442224176	-13.5924	0.52715	0.686073
SCE1572_1568	0.444783547	-13.7061	0.527224	0.686073
SCE1572_2831	0.565077781	-15.9679	0.527497	0.686073
SCE1572_1405	0.565077781	-15.9679	0.527497	0.686073
SCE1572_5354	-0.534457893	-15.9327	0.527497	0.686073
SCE1572_2895	-0.534457893	-15.9327	0.527497	0.686073
SCE1572_4651	-0.457435473	-14.3323	0.527664	0.686073
SCE1572_8644	0.455891937	-14.338	0.527664	0.686073
SCE1572_8231	0.452058878	-14.4055	0.527707	0.686073
SCE1572_2840	0.452058878	-14.4055	0.527707	0.686073
SCE1572_6162	-0.460457311	-14.4551	0.527752	0.686073
SCE1572_3239	0.535931435	-15.7898	0.527794	0.686073
SCE1572_8718	-0.456455381	-14.5311	0.527797	0.686073
SCE1572_11168	0.469658216	-14.6937	0.527889	0.686073
SCE1572_3880	0.469658216	-14.6937	0.527889	0.686073
SCE1572_2876	-0.459868576	-14.673	0.527889	0.686073
SCE1572_9916	0.491537316	-14.7676	0.527935	0.686073
SCE1572_5954	0.512847822	-15.6314	0.527962	0.686073
SCE1572_6420	-0.421949922	-12.5072	0.527985	0.686073
SCE1572_11491	0.494111259	-15.4888	0.528051	0.686073
SCE1572_1418	-0.497699343	-15.3524	0.52809	0.686073
SCE1572_8436	0.431480271	-13.3502	0.529472	0.687784
SCE1572_6119	0.445567407	-13.5131	0.529857	0.688199
SCE1572_11136	-0.451402153	-13.9436	0.53109	0.689715
SCE1572_4644	0.442580959	-14.0036	0.531241	0.689827
SCE1572_10520	0.45501144	-14.0489	0.531402	0.689865
SCE1572_566	0.447062687	-14.0529	0.531402	0.689865
SCE1572_10712	-0.438880233	-14.0874	0.531572	0.689924
SCE1572_5538	-0.42342658	-13.2471	0.531579	0.689924
SCE1572_10715	0.430420314	-13.5388	0.532731	0.691334
SCE1572_9646	-0.424540201	-13.1001	0.533176	0.691826
SCE1572_94	0.457928923	-14.5809	0.533441	0.692085
SCE1572_7443	0.465542107	-14.6551	0.533764	0.692419
SCE1572_72	-0.444572389	-13.8877	0.534475	0.693202
SCE1572_1056	0.46852765	-14.8298	0.5345	0.693202

SCE1572_2716	-0.4313644	-13.9336	0.534726	0.693405
SCE1572_10742	0.4798974	-14.9109	0.53492	0.693405
SCE1572_2729	0.4798974	-14.9109	0.53492	0.693405
SCE1572_7623	-0.473916351	-14.9024	0.53492	0.693405
SCE1572_793	-0.421247282	-13.0687	0.535083	0.693531
SCE1572_1291	0.475810443	-15.0125	0.535383	0.693835
SCE1572_150	0.487568413	-15.1136	0.535895	0.694242
SCE1572_693	0.44598931	-14.1343	0.535896	0.694242
SCE1572_3953	-0.443890683	-14.13	0.535896	0.694242
SCE1572_2991	0.451042537	-14.1884	0.536238	0.6946
SCE1572_613	0.501166017	-15.2222	0.536462	0.69472
SCE1572_6626	-0.451995733	-15.1963	0.536462	0.69472
SCE1572_7503	-0.431888159	-13.7039	0.536717	0.694902
SCE1572_2414	0.424183077	-13.3221	0.536735	0.694902
SCE1572_9500	0.517072408	-15.3398	0.537095	0.695196
SCE1572_4950	0.517072408	-15.3398	0.537095	0.695196
SCE1572_1545	0.423963003	-13.3864	0.537187	0.695208
SCE1572_778	-0.441348488	-13.7866	0.537303	0.695208
SCE1572_2595	0.432375243	-13.7972	0.537303	0.695208
SCE1572_11440	0.535931435	-15.4679	0.537803	0.695771
SCE1572_11553	0.465542107	-14.4327	0.537882	0.695787
SCE1572_9933	0.443737737	-13.929	0.538311	0.696256
SCE1572_6362	0.440451126	-13.9796	0.538688	0.696658
SCE1572_11302	-0.460185131	-14.5599	0.538917	0.696869
SCE1572_7786	-0.529299621	-15.7369	0.539497	0.697533
SCE1572_2086	-0.437433438	-14.168	0.540457	0.698688
SCE1572_1147	-0.409751644	-12.8114	0.54058	0.698761
SCE1572_7209	-0.459495835	-14.7957	0.540855	0.698896
SCE1572_6661	-0.436234357	-13.8165	0.540956	0.698896
SCE1572_4471	0.431375504	-14.2875	0.54154	0.698896
SCE1572_3768	-0.468869551	-14.8659	0.541636	0.698896
SCE1572_9338	-0.56515469	-16.0924	0.54165	0.698896
SCE1572_10741	-0.56515469	-16.0924	0.54165	0.698896
SCE1572_1547	-0.519351	-16.0695	0.54165	0.698896
SCE1572_8761	-0.519351	-16.0695	0.54165	0.698896
SCE1572_5916	0.427195795	-13.9134	0.541844	0.698896
SCE1572_2386	-0.405999509	-11.9388	0.542632	0.698896
SCE1572_2941	0.554547113	-16.3211	0.542911	0.698896
SCE1572_10317	0.554547113	-16.3211	0.542911	0.698896
SCE1572_2788	0.554547113	-16.3211	0.542911	0.698896
SCE1572_2719	0.517072408	-16.3398	0.542911	0.698896
SCE1572_2469	0.517072408	-16.3398	0.542911	0.698896
SCE1572_1971	-0.516535985	-16.316	0.542911	0.698896
SCE1572_703	0.950968934	-17.9973	0.542974	0.698896
SCE1572_1900	0.950968934	-17.9973	0.542974	0.698896
SCE1572_6861	0.950968934	-17.9973	0.542974	0.698896
SCE1572_9321	0.950968934	-17.9973	0.542974	0.698896
SCE1572_4422	0.950968934	-17.9973	0.542974	0.698896

SCE1572_2741	0.950968934	-17.9973	0.542974	0.698896
SCE1572_99	0.950968934	-17.9973	0.542974	0.698896
SCE1572_4677	-0.856385988	-17.901	0.542974	0.698896
SCE1572_8938	-0.856385988	-17.901	0.542974	0.698896
SCE1572_5575	-0.856385988	-17.901	0.542974	0.698896
SCE1572_2232	-0.856385988	-17.901	0.542974	0.698896
SCE1572_7794	-0.856385988	-17.901	0.542974	0.698896
SCE1572_5271	0.84405373	-18.0508	0.542974	0.698896
SCE1572_5351	0.84405373	-18.0508	0.542974	0.698896
SCE1572_908	0.84405373	-18.0508	0.542974	0.698896
SCE1572_2422	0.84405373	-18.0508	0.542974	0.698896
SCE1572_8321	0.439069896	-14.0569	0.543393	0.698896
SCE1572_7697	-0.434152987	-14.046	0.543393	0.698896
SCE1572_2765	-0.434152987	-14.046	0.543393	0.698896
SCE1572_1255	0.416632507	-13.0946	0.543642	0.698896
SCE1572_7287	-0.61934679	-16.5601	0.544247	0.698896
SCE1572_2688	-0.61934679	-16.5601	0.544247	0.698896
SCE1572_5315	-0.61934679	-16.5601	0.544247	0.698896
SCE1572_1438	0.603045631	-16.5863	0.544247	0.698896
SCE1572_3628	0.603045631	-16.5863	0.544247	0.698896
SCE1572_4454	0.603045631	-16.5863	0.544247	0.698896
SCE1572_8678	0.603045631	-16.5863	0.544247	0.698896
SCE1572_2663	0.603045631	-16.5863	0.544247	0.698896
SCE1572_8635	0.558651512	-16.6085	0.544247	0.698896
SCE1572_9861	0.558651512	-16.6085	0.544247	0.698896
SCE1572_10010	0.558651512	-16.6085	0.544247	0.698896
SCE1572_11339	0.558651512	-16.6085	0.544247	0.698896
SCE1572_2129	0.558651512	-16.6085	0.544247	0.698896
SCE1572_6188	0.558651512	-16.6085	0.544247	0.698896
SCE1572_1811	0.477037746	-15.1754	0.544556	0.698896
SCE1572_6234	0.477037746	-15.1754	0.544556	0.698896
SCE1572_5569	0.459759755	-15.184	0.544556	0.698896
SCE1572_10009	0.459759755	-15.184	0.544556	0.698896
SCE1572_6142	0.459759755	-15.184	0.544556	0.698896
SCE1572_10170	-0.439546246	-14.1483	0.544605	0.698896
SCE1572_2427	-0.401314614	-11.3455	0.54469	0.698896
SCE1572_9248	-0.436054189	-14.6034	0.545115	0.698896
SCE1572_9643	-0.414656371	-12.9076	0.545311	0.698896
SCE1572_3989	-0.643392264	-16.92	0.545468	0.698896
SCE1572_1968	-0.643392264	-16.92	0.545468	0.698896
SCE1572_5089	-0.643392264	-16.92	0.545468	0.698896
SCE1572_5758	-0.643392264	-16.92	0.545468	0.698896
SCE1572_4681	0.621661309	-16.9396	0.545468	0.698896
SCE1572_718	0.621661309	-16.9396	0.545468	0.698896
SCE1572_5666	0.621661309	-16.9396	0.545468	0.698896
SCE1572_2505	0.621661309	-16.9396	0.545468	0.698896
SCE1572_8585	0.621661309	-16.9396	0.545468	0.698896
SCE1572_3508	0.621661309	-16.9396	0.545468	0.698896

SCE1572_10771	0.621661309	-16.9396	0.545468	0.698896
SCE1572_31	-0.560930104	-16.8788	0.545468	0.698896
SCE1572_8235	-0.560930104	-16.8788	0.545468	0.698896
SCE1572_10269	0.728576513	-17.3716	0.545915	0.699042
SCE1572_2194	0.728576513	-17.3716	0.545915	0.699042
SCE1572_3649	0.728576513	-17.3716	0.545915	0.699042
SCE1572_9715	0.728576513	-17.3716	0.545915	0.699042
SCE1572_3672	0.693529566	-17.3891	0.545915	0.699042
SCE1572_7098	0.465542107	-14.7806	0.547051	0.700326
SCE1572_1779	-0.45513544	-14.7618	0.547051	0.700326
SCE1572_1953	-0.477874365	-15.3898	0.547186	0.700414
SCE1572_6894	0.421801561	-13.8025	0.547495	0.700725
SCE1572_5878	0.474819921	-14.8662	0.548173	0.701506
SCE1572_2228	-0.408359132	-13.2733	0.548388	0.701697
SCE1572_10389	-0.431888159	-14.4408	0.548527	0.701789
SCE1572_10352	0.503016813	-15.5583	0.548796	0.701943
SCE1572_9108	-0.471722138	-15.5388	0.548796	0.701943
SCE1572_6021	-0.400156801	-12.3153	0.548847	0.701943
SCE1572_1850	0.409061625	-13.0459	0.549241	0.702361
SCE1572_8605	-0.441348488	-14.5236	0.549518	0.702629
SCE1572_9256	-0.406172899	-12.9662	0.549959	0.703108
SCE1572_5053	0.438234761	-14.2537	0.550376	0.703556
SCE1572_1046	0.523756721	-15.7084	0.550667	0.703757
SCE1572_8304	0.499094667	-15.7208	0.550667	0.703757
SCE1572_3772	-0.410586235	-13.8201	0.551404	0.704613
SCE1572_1472	0.421395003	-13.6573	0.551735	0.704759
SCE1572_3900	-0.4103999	-13.6483	0.551735	0.704759
SCE1572_3204	-0.443189835	-14.6647	0.551786	0.704759
SCE1572_4436	0.441294561	-14.6672	0.551786	0.704759
SCE1572_3319	-0.436847096	-14.3693	0.552341	0.705195
SCE1572_8440	-0.399747584	-12.7031	0.552563	0.705195
SCE1572_4765	0.549606372	-15.8761	0.552865	0.705195
SCE1572_7680	0.549606372	-15.8761	0.552865	0.705195
SCE1572_3293	0.549606372	-15.8761	0.552865	0.705195
SCE1572_6095	0.549606372	-15.8761	0.552865	0.705195
SCE1572_6537	0.549606372	-15.8761	0.552865	0.705195
SCE1572_9911	0.522125635	-15.8898	0.552865	0.705195
SCE1572_8582	0.522125635	-15.8898	0.552865	0.705195
SCE1572_1751	-0.52140174	-15.859	0.552865	0.705195
SCE1572_10418	-0.482927592	-15.8398	0.552865	0.705195
SCE1572_9625	0.435529538	-14.7525	0.553092	0.705399
SCE1572_7839	-0.457836611	-15.2423	0.554174	0.706693
SCE1572_5709	-0.445452887	-14.8211	0.554538	0.707016
SCE1572_10622	0.407558923	-13.2246	0.554562	0.707016
SCE1572_6	-0.438073356	-14.4955	0.554649	0.707042
SCE1572_4354	0.582725646	-16.066	0.55548	0.707758
SCE1572_9034	0.582725646	-16.066	0.55548	0.707758
SCE1572_4764	0.582725646	-16.066	0.55548	0.707758

SCE1572_9425	0.551698751	-16.0815	0.55548	0.707758
SCE1572_8266	0.40159519	-12.6971	0.555611	0.707838
SCE1572_1518	0.421915175	-14.2922	0.555781	0.70797
SCE1572_11022	0.410606432	-13.3447	0.556938	0.709318
SCE1572_557	-0.4339765	-14.344	0.556974	0.709318
SCE1572_8316	-0.425751633	-14.1621	0.55773	0.710194
SCE1572_8780	-0.469362865	-15.0071	0.557945	0.710297
SCE1572_4675	0.460380402	-15.0202	0.557945	0.710297
SCE1572_8213	-0.411904711	-13.6638	0.558305	0.710328
SCE1572_4781	-0.387447283	-10.8852	0.55834	0.710328
SCE1572_8859	0.472737609	-15.4995	0.558578	0.710328
SCE1572_1014	0.472737609	-15.4995	0.558578	0.710328
SCE1572_6535	0.472737609	-15.4995	0.558578	0.710328
SCE1572_7900	0.472737609	-15.4995	0.558578	0.710328
SCE1572_7074	-0.464068565	-15.4825	0.558578	0.710328
SCE1572_6842	0.451042537	-15.5103	0.558578	0.710328
SCE1572_5160	0.451042537	-15.5103	0.558578	0.710328
SCE1572_8894	0.41507704	-13.8502	0.558781	0.710501
SCE1572_754	-0.416621403	-14.7115	0.558985	0.710587
SCE1572_11376	-0.416621403	-14.7115	0.558985	0.710587
SCE1572_10387	0.45440155	-15.1301	0.559974	0.711673
SCE1572_9123	0.45440155	-15.1301	0.559974	0.711673
SCE1572_6174	-0.413442492	-14.2645	0.560159	0.711823
SCE1572_8278	0.489388849	-15.6432	0.561338	0.713062
SCE1572_4081	-0.455848058	-15.6418	0.561338	0.713062
SCE1572_8901	-0.455848058	-15.6418	0.561338	0.713062
SCE1572_5371	-0.396954369	-12.9709	0.56188	0.713651
SCE1572_10939	-0.392985467	-12.6249	0.562107	0.713651
SCE1572_2774	0.483464015	-15.2311	0.562277	0.713651
SCE1572_2737	-0.476243279	-15.2084	0.562277	0.713651
SCE1572_11283	-0.476243279	-15.2084	0.562277	0.713651
SCE1572_638	-0.476243279	-15.2084	0.562277	0.713651
SCE1572_4246	0.465542107	-15.24	0.562277	0.713651
SCE1572_2658	0.391191569	-12.1006	0.56302	0.714508
SCE1572_8667	0.483464015	-15.816	0.564607	0.716222
SCE1572_7406	0.483464015	-15.816	0.564607	0.716222
SCE1572_5649	-0.386686034	-12.1218	0.56471	0.716222
SCE1572_5890	0.440451126	-14.9796	0.564864	0.716222
SCE1572_3245	0.425184369	-14.9872	0.564864	0.716222
SCE1572_7932	0.497963585	-15.3494	0.564915	0.716222
SCE1572_4848	0.47859826	-15.359	0.564915	0.716222
SCE1572_5200	0.47859826	-15.359	0.564915	0.716222
SCE1572_7590	-0.396954369	-13.1477	0.566495	0.718138
SCE1572_3157	-0.400409928	-14.0101	0.566723	0.718234
SCE1572_2873	0.452366718	-14.7872	0.566775	0.718234
SCE1572_8104	0.452366718	-14.7872	0.566775	0.718234
SCE1572_10291	-0.608458474	-16.777	0.567307	0.718491
SCE1572_4037	-0.608458474	-16.777	0.567307	0.718491

SCE1572_7570	-0.608458474	-16.777	0.567307	0.718491
SCE1572_1688	-0.608458474	-16.777	0.567307	0.718491
SCE1572_8653	0.433120629	-15.0863	0.56732	0.718491
SCE1572_2751	0.418892014	-14.3889	0.567621	0.718786
SCE1572_4826	-0.381944201	-11.8436	0.568041	0.71917
SCE1572_3962	0.423163505	-14.5983	0.568243	0.71917
SCE1572_6177	-0.420286873	-14.5955	0.568243	0.71917
SCE1572_6403	0.411386337	-14.6042	0.568243	0.71917
SCE1572_11151	0.506184092	-15.9973	0.568539	0.71917
SCE1572_8313	0.506184092	-15.9973	0.568539	0.71917
SCE1572_3349	0.506184092	-15.9973	0.568539	0.71917
SCE1572_11067	-0.464068565	-15.9679	0.568539	0.71917
SCE1572_3656	-0.464068565	-15.9679	0.568539	0.71917
SCE1572_10823	0.434291173	-14.4483	0.569406	0.720093
SCE1572_9534	0.423721931	-14.4536	0.569406	0.720093
SCE1572_5945	0.442272328	-15.1928	0.570106	0.720719
SCE1572_2881	0.442272328	-15.1928	0.570106	0.720719
SCE1572_6998	0.442272328	-15.1928	0.570106	0.720719
SCE1572_3891	-0.408927011	-13.9955	0.570308	0.720824
SCE1572_1833	0.416632507	-14.6795	0.570342	0.720824
SCE1572_9328	-0.378793585	-12.3164	0.570396	0.720824
SCE1572_8615	0.403736823	-13.9017	0.570951	0.721439
SCE1572_10563	0.429016231	-14.5213	0.571351	0.721685
SCE1572_3435	-0.396954369	-14.5014	0.571351	0.721685
SCE1572_6814	-0.396954369	-14.5014	0.571351	0.721685
SCE1572_10161	-0.42342658	-14.9476	0.571582	0.721717
SCE1572_2661	-0.42342658	-14.9476	0.571582	0.721717
SCE1572_3976	-0.42342658	-14.9476	0.571582	0.721717
SCE1572_8100	-0.404061256	-14.0504	0.571789	0.721891
SCE1572_5565	0.414142955	-13.9438	0.572349	0.722512
SCE1572_7694	-0.408927011	-14.2178	0.572821	0.72281
SCE1572_11480	-0.381722653	-12.6266	0.57311	0.72281
SCE1572_3454	0.44818854	-15.3101	0.573297	0.72281
SCE1572_1533	-0.633993566	-17.2048	0.573302	0.72281
SCE1572_3135	-0.633993566	-17.2048	0.573302	0.72281
SCE1572_1044	-0.633993566	-17.2048	0.573302	0.72281
SCE1572_4869	-0.534457893	-17.1551	0.573302	0.72281
SCE1572_6869	0.535931435	-16.2048	0.573359	0.72281
SCE1572_5431	0.535931435	-16.2048	0.573359	0.72281
SCE1572_3132	0.501166017	-16.2222	0.573359	0.72281
SCE1572_7687	0.501166017	-16.2222	0.573359	0.72281
SCE1572_7747	-0.432704394	-14.5737	0.573479	0.72281
SCE1572_4604	-0.432704394	-14.5737	0.573479	0.72281
SCE1572_11317	-0.386900704	-12.8503	0.573672	0.722967
SCE1572_9365	-0.398535405	-13.882	0.574237	0.723592
SCE1572_10817	0.429016231	-14.8433	0.575233	0.72476
SCE1572_1445	0.393709362	-13.7316	0.576214	0.72588
SCE1572_11218	0.415309328	-14.4926	0.57626	0.72588

SCE1572_1719	-0.394280235	-13.6402	0.576412	0.725985
SCE1572_9748	0.465542107	-15.4327	0.576985	0.726446
SCE1572_690	0.465542107	-15.4327	0.576985	0.726446
SCE1572_7411	0.444783547	-15.4431	0.576985	0.726446
SCE1572_11318	-0.396954369	-14.0863	0.57712	0.726529
SCE1572_9754	-0.42342658	-15.1402	0.577675	0.727141
SCE1572_6974	0.42159876	-14.9401	0.578107	0.72751
SCE1572_6757	-0.419522126	-14.91	0.578107	0.72751
SCE1572_5426	0.410400553	-14.2676	0.578852	0.728354
SCE1572_4701	0.403481139	-14.0231	0.579003	0.728354
SCE1572_10867	0.57657342	-16.4476	0.579401	0.728354
SCE1572_3484	0.57657342	-16.4476	0.579401	0.728354
SCE1572_1806	0.57657342	-16.4476	0.579401	0.728354
SCE1572_2650	0.535931435	-16.4679	0.579401	0.728354
SCE1572_5339	0.535931435	-16.4679	0.579401	0.728354
SCE1572_6807	0.535931435	-16.4679	0.579401	0.728354
SCE1572_7981	-0.534457893	-16.4181	0.579401	0.728354
SCE1572_8211	-0.372352396	-13.0345	0.580211	0.729286
SCE1572_8031	0.385548098	-13.2206	0.580301	0.729312
SCE1572_2795	-0.493815908	-15.9124	0.580886	0.729781
SCE1572_2007	-0.493815908	-15.9124	0.580886	0.729781
SCE1572_10181	-0.42342658	-14.6256	0.581159	0.729781
SCE1572_8588	0.480649	-15.5695	0.581299	0.729781
SCE1572_8277	0.457928923	-15.5809	0.581299	0.729781
SCE1572_11517	0.457928923	-15.5809	0.581299	0.729781
SCE1572_414	0.457928923	-15.5809	0.581299	0.729781
SCE1572_7097	0.457928923	-15.5809	0.581299	0.729781
SCE1572_5035	0.457928923	-15.5809	0.581299	0.729781
SCE1572_2878	-0.371258755	-12.8171	0.581698	0.730195
SCE1572_2253	-0.370107762	-12.324	0.582187	0.730721
SCE1572_3771	-0.399179034	-14.3747	0.583268	0.731991
SCE1572_11040	0.404879325	-14.1829	0.584812	0.733737
SCE1572_1957	0.437527731	-15.1386	0.584996	0.733737
SCE1572_2434	0.393156182	-14.3066	0.58525	0.733737
SCE1572_8212	-0.949495392	-18.5325	0.585507	0.733737
SCE1572_5036	-0.949495392	-18.5325	0.585507	0.733737
SCE1572_6447	-0.949495392	-18.5325	0.585507	0.733737
SCE1572_10992	-0.949495392	-18.5325	0.585507	0.733737
SCE1572_3002	0.898501514	-18.6085	0.585507	0.733737
SCE1572_7779	0.898501514	-18.6085	0.585507	0.733737
SCE1572_4333	0.898501514	-18.6085	0.585507	0.733737
SCE1572_1987	0.898501514	-18.6085	0.585507	0.733737
SCE1572_811	0.898501514	-18.6085	0.585507	0.733737
SCE1572_8406	0.38792121	-13.8865	0.585567	0.733737
SCE1572_10414	-0.450393628	-15.3761	0.585652	0.733757
SCE1572_713	-0.388462908	-13.9852	0.585991	0.733762
SCE1572_4494	0.474003686	-15.7333	0.586413	0.733762
SCE1572_7545	0.391541526	-14.1071	0.586478	0.733762

SCE1572_6206	0.408487418	-14.2388	0.587042	0.733762
SCE1572_2467	0.42564938	-14.8006	0.587117	0.733762
SCE1572_433	0.368133989	-12.9548	0.587131	0.733762
SCE1572_1140	-0.487152178	-16.1314	0.587167	0.733762
SCE1572_9043	-0.487152178	-16.1314	0.587167	0.733762
SCE1572_5707	-0.487152178	-16.1314	0.587167	0.733762
SCE1572_6134	-0.441348488	-16.1085	0.587167	0.733762
SCE1572_1093	-0.441348488	-16.1085	0.587167	0.733762
SCE1572_9370	0.635467109	-16.74	0.587191	0.733762
SCE1572_3197	0.635467109	-16.74	0.587191	0.733762
SCE1572_4182	0.635467109	-16.74	0.587191	0.733762
SCE1572_5631	0.635467109	-16.74	0.587191	0.733762
SCE1572_2317	0.635467109	-16.74	0.587191	0.733762
SCE1572_4058	0.635467109	-16.74	0.587191	0.733762
SCE1572_1081	0.586557508	-16.7645	0.587191	0.733762
SCE1572_3129	0.586557508	-16.7645	0.587191	0.733762
SCE1572_7263	0.586557508	-16.7645	0.587191	0.733762
SCE1572_8034	0.586557508	-16.7645	0.587191	0.733762
SCE1572_7154	0.586557508	-16.7645	0.587191	0.733762
SCE1572_2249	0.438969705	-15.2533	0.589173	0.736133
SCE1572_7805	-0.369034284	-13.5008	0.589302	0.736133
SCE1572_4012	0.385092275	-13.9826	0.589403	0.736133
SCE1572_7372	-0.413442492	-14.6795	0.589438	0.736133
SCE1572_3485	0.404141562	-14.6858	0.589438	0.736133
SCE1572_1666	0.418236392	-14.8945	0.590607	0.737376
SCE1572_2672	-0.396954369	-14.8639	0.590607	0.737376
SCE1572_1155	0.437646536	-15.517	0.590644	0.737376
SCE1572_1684	-0.389259977	-14.58	0.591473	0.738285
SCE1572_3422	-0.381301105	-14.0196	0.591513	0.738285
SCE1572_5744	0.370345369	-13.4976	0.591758	0.738504
SCE1572_8508	-0.418980675	-15.0973	0.592079	0.738641
SCE1572_8870	0.416632507	-15.0946	0.592079	0.738641
SCE1572_6309	-0.396954369	-15.0863	0.592079	0.738641
SCE1572_11404	0.494111259	-15.9038	0.592573	0.739135
SCE1572_2714	-0.405724579	-14.7688	0.592826	0.739135
SCE1572_9433	-0.405724579	-14.7688	0.592826	0.739135
SCE1572_1919	0.396001174	-14.7723	0.592826	0.739135
SCE1572_11021	0.396001174	-14.7723	0.592826	0.739135
SCE1572_6865	0.40444751	-14.498	0.593272	0.739428
SCE1572_4637	0.393392321	-14.5035	0.593272	0.739428
SCE1572_4562	-0.382454799	-14.4941	0.593272	0.739428
SCE1572_5056	0.363464239	-12.7263	0.59351	0.739637
SCE1572_2730	0.458969453	-15.3689	0.593987	0.739881
SCE1572_2872	0.458969453	-15.3689	0.593987	0.739881
SCE1572_5749	0.439069896	-15.3788	0.593987	0.739881
SCE1572_736	0.439069896	-15.3788	0.593987	0.739881
SCE1572_6380	0.47859826	-16.359	0.595081	0.740893
SCE1572_1754	0.47859826	-16.359	0.595081	0.740893

SCE1572_4324	0.47859826	-16.359	0.595081	0.740893
SCE1572_3488	0.47859826	-16.359	0.595081	0.740893
SCE1572_6841	-0.358446549	-12.5575	0.595369	0.741164
SCE1572_4375	-0.37553189	-13.9418	0.59607	0.74185
SCE1572_9044	0.392973481	-14.1334	0.596182	0.74185
SCE1572_4791	0.384923647	-14.3418	0.596309	0.74185
SCE1572_1767	0.384923647	-14.3418	0.596309	0.74185
SCE1572_721	0.452984254	-15.6614	0.596551	0.74185
SCE1572_2233	0.441294561	-15.6672	0.596551	0.74185
SCE1572_1610	0.369682092	-13.4806	0.596589	0.74185
SCE1572_8262	0.41507704	-14.8502	0.596596	0.74185
SCE1572_9432	0.401001855	-14.8573	0.596596	0.74185
SCE1572_6806	0.424570326	-15.2016	0.596696	0.74185
SCE1572_5020	0.406648418	-15.2106	0.596696	0.74185
SCE1572_11333	-0.378338691	-13.7392	0.596895	0.742009
SCE1572_10662	0.38065321	-14.0601	0.597321	0.742351
SCE1572_98	0.667175968	-17.1392	0.597593	0.742351
SCE1572_2956	0.667175968	-17.1392	0.597593	0.742351
SCE1572_1825	0.667175968	-17.1392	0.597593	0.742351
SCE1572_3391	-0.593351582	-17.0325	0.597593	0.742351
SCE1572_6266	-0.593351582	-17.0325	0.597593	0.742351
SCE1572_6450	0.355917616	-12.9373	0.598299	0.74314
SCE1572_1709	0.387068049	-14.1918	0.598775	0.743555
SCE1572_7242	-0.383316367	-14.1833	0.598775	0.743555
SCE1572_9320	-0.414787662	-15.0557	0.598935	0.743579
SCE1572_2077	-0.414787662	-15.0557	0.598935	0.743579
SCE1572_24	-0.394280235	-14.6402	0.599945	0.744724
SCE1572_8560	0.519989891	-16.0973	0.600141	0.744724
SCE1572_3499	-0.474956881	-16.0473	0.600141	0.744724
SCE1572_352	-0.474956881	-16.0473	0.600141	0.744724
SCE1572_875	0.361271624	-13.109	0.600584	0.745187
SCE1572_466	0.406648418	-14.9476	0.600817	0.745299
SCE1572_4457	-0.383898216	-14.9278	0.600817	0.745299
SCE1572_585	-0.386900704	-14.5508	0.601315	0.745829
SCE1572_4618	0.377314924	-13.9865	0.601711	0.746232
SCE1572_6622	0.414467923	-15.327	0.602008	0.746425
SCE1572_6405	-0.392729783	-15.2999	0.602008	0.746425
SCE1572_2293	-0.375760147	-14.8194	0.602421	0.74674
SCE1572_1591	-0.360357215	-13.7144	0.602513	0.74674
SCE1572_2659	0.402348281	-14.4643	0.602528	0.74674
SCE1572_4196	-0.381606405	-14.1611	0.602546	0.74674
SCE1572_4186	0.456496968	-15.8295	0.603651	0.747492
SCE1572_1290	0.456496968	-15.8295	0.603651	0.747492
SCE1572_8103	-0.445452887	-15.8211	0.603651	0.747492
SCE1572_3466	-0.445452887	-15.8211	0.603651	0.747492
SCE1572_3751	0.429016231	-15.8433	0.603651	0.747492
SCE1572_3935	0.429016231	-15.8433	0.603651	0.747492
SCE1572_7681	0.429016231	-15.8433	0.603651	0.747492

SCE1572_8545	0.406648418	-14.7252	0.603804	0.747594
SCE1572_5279	0.361556877	-13.2623	0.603976	0.747719
SCE1572_3742	0.375466763	-14.6221	0.605009	0.748819
SCE1572_8438	0.512847822	-16.6314	0.605363	0.748819
SCE1572_9384	0.512847822	-16.6314	0.605363	0.748819
SCE1572_9258	0.512847822	-16.6314	0.605363	0.748819
SCE1572_5154	0.512847822	-16.6314	0.605363	0.748819
SCE1572_11054	0.512847822	-16.6314	0.605363	0.748819
SCE1572_6541	0.512847822	-16.6314	0.605363	0.748819
SCE1572_11063	0.413074687	-15.0439	0.605575	0.748993
SCE1572_6918	-0.42342658	-15.6256	0.606222	0.74953
SCE1572_8281	-0.42342658	-15.6256	0.606222	0.74953
SCE1572_2153	-0.391717721	-15.6098	0.606222	0.74953
SCE1572_8871	0.364420183	-13.8532	0.606331	0.749576
SCE1572_9634	-0.353107285	-13.4661	0.607785	0.751284
SCE1572_9444	0.398427911	-14.8142	0.608087	0.75157
SCE1572_7807	0.34347152	-12.5732	0.608439	0.751917
SCE1572_8738	0.378830474	-14.6984	0.609084	0.752625
SCE1572_5900	-0.464068565	-16.2898	0.609666	0.753079
SCE1572_3262	-0.413442492	-16.2645	0.609666	0.753079
SCE1572_4906	-0.413442492	-16.2645	0.609666	0.753079
SCE1572_41	0.382251764	-14.5091	0.610715	0.754197
SCE1572_1176	0.371024508	-14.5147	0.610715	0.754197
SCE1572_2824	0.420454218	-15.1471	0.610979	0.754435
SCE1572_2637	0.378390158	-15.0106	0.612007	0.754577
SCE1572_11581	-0.369603881	-14.9947	0.612007	0.754577
SCE1572_7205	-0.347554556	-13.4901	0.612083	0.754577
SCE1572_8908	0.816039354	-17.6498	0.612094	0.754577
SCE1572_11451	0.816039354	-17.6498	0.612094	0.754577
SCE1572_7315	0.816039354	-17.6498	0.612094	0.754577
SCE1572_8067	0.816039354	-17.6498	0.612094	0.754577
SCE1572_9864	0.816039354	-17.6498	0.612094	0.754577
SCE1572_3876	0.816039354	-17.6498	0.612094	0.754577
SCE1572_8961	0.816039354	-17.6498	0.612094	0.754577
SCE1572_399	0.816039354	-17.6498	0.612094	0.754577
SCE1572_10522	-0.61934679	-17.5601	0.612094	0.754577
SCE1572_922	-0.61934679	-17.5601	0.612094	0.754577
SCE1572_5810	-0.331926646	-7.90718	0.612263	0.754577
SCE1572_10929	0.475810443	-16.0125	0.61235	0.754577
SCE1572_5633	0.475810443	-16.0125	0.61235	0.754577
SCE1572_8251	0.444783547	-16.028	0.61235	0.754577
SCE1572_10447	-0.352343482	-13.4526	0.612386	0.754577
SCE1572_1355	-0.473057348	-15.7707	0.614163	0.75659
SCE1572_10490	-0.437433438	-15.7529	0.614163	0.75659
SCE1572_11145	-0.373993221	-14.5723	0.614778	0.75717
SCE1572_6724	-0.373993221	-14.5723	0.614778	0.75717
SCE1572_10288	0.35613089	-13.404	0.61487	0.757195
SCE1572_224	0.43484531	-15.5924	0.615458	0.757654

SCE1572_1923	0.43484531	-15.5924	0.615458	0.757654
SCE1572_5309	-0.417274354	-15.566	0.615458	0.757654
SCE1572_11435	0.367986798	-14.1733	0.616703	0.759096
SCE1572_7384	0.337268085	-12.2381	0.617144	0.75955
SCE1572_10360	-0.358300938	-13.9701	0.617459	0.75985
SCE1572_9996	0.386787996	-14.8644	0.618637	0.761121
SCE1572_9359	-0.378338691	-14.8546	0.618637	0.761121
SCE1572_8954	-0.353885647	-14.7429	0.618968	0.7612
SCE1572_11106	-0.353885647	-14.7429	0.618968	0.7612
SCE1572_8083	0.565077781	-16.9679	0.619281	0.7612
SCE1572_9429	0.565077781	-16.9679	0.619281	0.7612
SCE1572_9238	0.565077781	-16.9679	0.619281	0.7612
SCE1572_2079	0.565077781	-16.9679	0.619281	0.7612
SCE1572_7654	0.565077781	-16.9679	0.619281	0.7612
SCE1572_7381	0.506184092	-16.9973	0.619281	0.7612
SCE1572_6136	0.338370867	-13.3036	0.619375	0.761228
SCE1572_3942	-0.32520071	-8.64059	0.619789	0.761647
SCE1572_697	0.366006434	-14.3829	0.619883	0.761674
SCE1572_9152	-0.370201642	-14.1554	0.620316	0.762117
SCE1572_5915	-0.341584558	-12.9248	0.621668	0.763689
SCE1572_6917	-0.555216453	-16.528	0.622031	0.763868
SCE1572_10573	-0.493815908	-16.4973	0.622031	0.763868
SCE1572_4607	-0.493815908	-16.4973	0.622031	0.763868
SCE1572_683	0.465542107	-16.24	0.623263	0.764558
SCE1572_8962	0.465542107	-16.24	0.623263	0.764558
SCE1572_9986	0.465542107	-16.24	0.623263	0.764558
SCE1572_11144	0.465542107	-16.24	0.623263	0.764558
SCE1572_3404	-0.451995733	-16.1963	0.623263	0.764558
SCE1572_1689	-0.451995733	-16.1963	0.623263	0.764558
SCE1572_4987	-0.345424068	-13.3236	0.623409	0.764558
SCE1572_9472	0.33930211	-13.6787	0.623594	0.764558
SCE1572_7396	0.344733635	-13.9547	0.623751	0.764558
SCE1572_2152	-0.42342658	-15.9476	0.623857	0.764558
SCE1572_3870	0.362782533	-14.2914	0.623951	0.764558
SCE1572_10502	0.357594194	-14.4527	0.624045	0.764558
SCE1572_9212	-0.357153361	-14.5359	0.624094	0.764558
SCE1572_6919	0.422473385	-15.7591	0.624149	0.764558
SCE1572_2169	0.422473385	-15.7591	0.624149	0.764558
SCE1572_1051	0.422473385	-15.7591	0.624149	0.764558
SCE1572_838	-0.368720688	-15.7186	0.624149	0.764558
SCE1572_8189	0.383928342	-15.5439	0.624286	0.764558
SCE1572_5826	0.383928342	-15.5439	0.624286	0.764558
SCE1572_1552	0.376274769	-14.9627	0.624287	0.764558
SCE1572_10685	0.418892014	-15.3889	0.624339	0.764558
SCE1572_3978	0.418892014	-15.3889	0.624339	0.764558
SCE1572_4496	-0.396954369	-15.3494	0.624339	0.764558
SCE1572_4585	-0.396954369	-15.3494	0.624339	0.764558
SCE1572_9157	0.332775233	-12.6722	0.624441	0.764595

SCE1572_2721	0.334003116	-12.7615	0.627355	0.768074
SCE1572_9488	-0.361621296	-14.1942	0.627557	0.768232
SCE1572_11414	-0.350857955	-14.4259	0.628296	0.76899
SCE1572_7928	0.343286357	-13.5642	0.628323	0.76899
SCE1572_5430	-0.320811514	-11.5177	0.628801	0.769485
SCE1572_9959	-0.360690825	-14.5943	0.62892	0.769541
SCE1572_4298	0.366006434	-14.7048	0.629284	0.769719
SCE1572_2644	0.353067378	-14.7113	0.629284	0.769719
SCE1572_5501	0.353067378	-14.7113	0.629284	0.769719
SCE1572_5809	-0.33944191	-13.9796	0.630132	0.770666
SCE1572_7978	0.341230748	-13.4609	0.630212	0.770674
SCE1572_6530	-0.393414011	-15.045	0.63067	0.771055
SCE1572_462	-0.393414011	-15.045	0.63067	0.771055
SCE1572_9545	-0.35813512	-14.33	0.631974	0.77256
SCE1572_9103	-0.333579963	-13.8495	0.632449	0.772878
SCE1572_5195	0.367176977	-14.4148	0.632455	0.772878
SCE1572_3176	-0.362846515	-14.4063	0.632455	0.772878
SCE1572_11149	-1.008389081	-18.24	0.63315	0.773458
SCE1572_1087	-1.008389081	-18.24	0.63315	0.773458
SCE1572_6590	-1.008389081	-18.24	0.63315	0.773458
SCE1572_1086	-0.338537683	-13.9603	0.633228	0.773464
SCE1572_6658	0.334119891	-13.0327	0.633364	0.773527
SCE1572_5742	0.328422693	-13.0633	0.633601	0.773527
SCE1572_11471	0.416632507	-15.6795	0.633648	0.773527
SCE1572_3707	-0.396954369	-15.6713	0.633648	0.773527
SCE1572_11321	0.391541526	-15.6921	0.633648	0.773527
SCE1572_7122	-0.318884841	-11.9458	0.634158	0.773847
SCE1572_7643	-0.326565041	-12.5772	0.634237	0.773847
SCE1572_8997	0.465542107	-15.9181	0.634719	0.773847
SCE1572_2343	0.465542107	-15.9181	0.634719	0.773847
SCE1572_4241	0.465542107	-15.9181	0.634719	0.773847
SCE1572_2307	0.465542107	-15.9181	0.634719	0.773847
SCE1572_9559	-0.454287544	-15.8926	0.634719	0.773847
SCE1572_6242	0.436395761	-15.9327	0.634719	0.773847
SCE1572_3297	0.436395761	-15.9327	0.634719	0.773847
SCE1572_10815	0.436395761	-15.9327	0.634719	0.773847
SCE1572_7798	-0.415813396	-15.8734	0.634719	0.773847
SCE1572_5340	-0.373303101	-14.785	0.634991	0.774089
SCE1572_2178	0.342393876	-13.8422	0.635274	0.774344
SCE1572_7983	-0.318116723	-12.9222	0.635763	0.774675
SCE1572_7723	-0.381047978	-14.8907	0.635841	0.774675
SCE1572_555	0.374293045	-14.9164	0.635841	0.774675
SCE1572_9728	-0.362188951	-14.8813	0.635841	0.774675
SCE1572_8662	0.362448614	-15.0186	0.636814	0.77573
SCE1572_5847	-0.339810462	-14.4728	0.637284	0.77573
SCE1572_10919	0.494111259	-16.4888	0.63737	0.77573
SCE1572_10931	0.494111259	-16.4888	0.63737	0.77573
SCE1572_9782	0.494111259	-16.4888	0.63737	0.77573

SCE1572_3428	-0.443604462	-16.4722	0.63737	0.77573
SCE1572_4733	-0.434922219	-16.4679	0.63737	0.77573
SCE1572_9423	-0.434922219	-16.4679	0.63737	0.77573
SCE1572_10750	-0.434922219	-16.4679	0.63737	0.77573
SCE1572_6598	0.385688799	-15.1645	0.637939	0.776049
SCE1572_1391	-0.358886328	-14.5648	0.638129	0.776049
SCE1572_7857	0.349162923	-14.5613	0.638129	0.776049
SCE1572_8633	-0.343935987	-14.5573	0.638129	0.776049
SCE1572_487	-0.325871271	-13.8809	0.638643	0.776049
SCE1572_10813	-0.482927592	-16.8398	0.638759	0.776049
SCE1572_10136	-0.482927592	-16.8398	0.638759	0.776049
SCE1572_9408	0.364613198	-14.666	0.639076	0.776049
SCE1572_11485	-0.311279476	-11.6938	0.639116	0.776049
SCE1572_8320	-0.686460986	-17.401	0.63922	0.776049
SCE1572_7965	-0.686460986	-17.401	0.63922	0.776049
SCE1572_4966	-0.686460986	-17.401	0.63922	0.776049
SCE1572_7937	0.654575932	-17.4086	0.63922	0.776049
SCE1572_10446	0.654575932	-17.4086	0.63922	0.776049
SCE1572_7109	0.654575932	-17.4086	0.63922	0.776049
SCE1572_1063	0.654575932	-17.4086	0.63922	0.776049
SCE1572_11354	0.654575932	-17.4086	0.63922	0.776049
SCE1572_43	0.57657342	-17.4476	0.63922	0.776049
SCE1572_6602	0.57657342	-17.4476	0.63922	0.776049
SCE1572_11152	0.57657342	-17.4476	0.63922	0.776049
SCE1572_3009	-0.570983769	-17.3433	0.63922	0.776049
SCE1572_3421	-0.367638802	-15.2874	0.639255	0.776049
SCE1572_10430	0.33778656	-14.0062	0.639955	0.776809
SCE1572_5821	-0.349507033	-14.7565	0.640146	0.776951
SCE1572_4659	-0.348044769	-14.3738	0.640511	0.777135
SCE1572_11044	-0.406353067	-15.4536	0.640815	0.777135
SCE1572_3736	-0.406353067	-15.4536	0.640815	0.777135
SCE1572_179	0.402348281	-15.4643	0.640815	0.777135
SCE1572_6966	0.402348281	-15.4643	0.640815	0.777135
SCE1572_11217	-0.378338691	-15.4396	0.640815	0.777135
SCE1572_9039	-0.378338691	-15.4396	0.640815	0.777135
SCE1572_3419	-0.323890907	-13.4124	0.640912	0.777163
SCE1572_7530	0.357933133	-14.8788	0.641364	0.777532
SCE1572_8942	0.357933133	-14.8788	0.641364	0.777532
SCE1572_8701	-0.349425999	-14.9846	0.642763	0.779065
SCE1572_10405	-0.320892163	-13.7425	0.642776	0.779065
SCE1572_7217	-0.330802034	-14.269	0.643347	0.779667
SCE1572_3235	0.304497209	-10.0806	0.643771	0.780091
SCE1572_6323	-0.356312385	-15.1067	0.644386	0.780688
SCE1572_2960	0.344565909	-14.3619	0.644413	0.780688
SCE1572_3741	0.367477821	-14.7448	0.645163	0.781507
SCE1572_10092	0.333716896	-13.7412	0.645353	0.781647
SCE1572_9793	0.391541526	-15.277	0.646292	0.782514
SCE1572_4223	-0.364532891	-15.24	0.646292	0.782514

SCE1572_488	0.36069201	-15.2698	0.646292	0.782514
SCE1572_970	-0.359960162	-14.8454	0.646728	0.782932
SCE1572_5081	-0.301895839	-10.343	0.646786	0.782932
SCE1572_7417	-0.328007015	-14.5213	0.646908	0.782989
SCE1572_7270	0.313539014	-12.9703	0.647538	0.783661
SCE1572_7752	0.487568413	-16.1136	0.647858	0.783868
SCE1572_911	0.45440155	-16.1301	0.647858	0.783868
SCE1572_4838	0.333858998	-14.3363	0.648233	0.784052
SCE1572_5589	0.333858998	-14.3363	0.648233	0.784052
SCE1572_2026	0.333858998	-14.3363	0.648233	0.784052
SCE1572_7864	-0.34697452	-14.6166	0.648382	0.784142
SCE1572_2237	0.356607736	-15.42	0.648562	0.784269
SCE1572_6461	0.340011225	-14.7178	0.650046	0.785911
SCE1572_1727	0.313539014	-13.5612	0.650069	0.785911
SCE1572_627	-0.341132459	-14.5004	0.651145	0.786774
SCE1572_11269	-0.3269186	-14.4933	0.651145	0.786774
SCE1572_5408	0.387539595	-15.6161	0.651311	0.786774
SCE1572_1820	-0.477874365	-16.3898	0.651531	0.786774
SCE1572_6618	0.439069896	-16.3788	0.651531	0.786774
SCE1572_5887	0.439069896	-16.3788	0.651531	0.786774
SCE1572_7609	-0.42342658	-16.3626	0.651531	0.786774
SCE1572_6485	-0.42342658	-16.3626	0.651531	0.786774
SCE1572_614	0.398427911	-16.3991	0.651531	0.786774
SCE1572_10133	0.398427911	-16.3991	0.651531	0.786774
SCE1572_8790	-0.317717139	-13.6468	0.651876	0.78695
SCE1572_971	0.370682921	-14.828	0.651939	0.78695
SCE1572_6664	0.356607736	-14.8351	0.651939	0.78695
SCE1572_11064	-0.323614632	-14.3139	0.651976	0.78695
SCE1572_602	-0.310155882	-12.9745	0.652648	0.78767
SCE1572_8075	-0.332418752	-14.594	0.652863	0.78784
SCE1572_8417	0.370122542	-15.2289	0.653067	0.787906
SCE1572_6756	0.351506864	-15.2382	0.653067	0.787906
SCE1572_8208	0.3347555	-14.3985	0.653542	0.78825
SCE1572_5392	0.324186258	-14.4038	0.653542	0.78825
SCE1572_5923	-0.307492742	-13.8717	0.653578	0.78825
SCE1572_11126	0.344235811	-14.9315	0.654113	0.788625
SCE1572_9070	0.328969054	-14.9391	0.654113	0.788625
SCE1572_10606	0.328969054	-14.9391	0.654113	0.788625
SCE1572_2465	-0.408927011	-15.8028	0.654708	0.789161
SCE1572_1632	-0.408927011	-15.8028	0.654708	0.789161
SCE1572_5827	0.341583928	-14.7035	0.654801	0.789182
SCE1572_8475	0.296802225	-11.7345	0.655049	0.789392
SCE1572_3194	-0.325463327	-14.133	0.655916	0.789508
SCE1572_10890	0.398427911	-15.3991	0.655991	0.789508
SCE1572_10958	0.398427911	-15.3991	0.655991	0.789508
SCE1572_5127	0.377669351	-15.4095	0.655991	0.789508
SCE1572_5304	-0.37451698	-15.3871	0.655991	0.789508
SCE1572_2336	-0.348044769	-15.3738	0.655991	0.789508

SCE1572_7808	0.302504963	-12.4553	0.656323	0.789508
SCE1572_9822	0.535931435	-16.7898	0.656347	0.789508
SCE1572_4388	0.535931435	-16.7898	0.656347	0.789508
SCE1572_11020	0.483464015	-16.816	0.656347	0.789508
SCE1572_4854	0.483464015	-16.816	0.656347	0.789508
SCE1572_144	0.483464015	-16.816	0.656347	0.789508
SCE1572_8729	-0.464068565	-16.7048	0.656347	0.789508
SCE1572_1512	-0.464068565	-16.7048	0.656347	0.789508
SCE1572_7500	-0.464068565	-16.7048	0.656347	0.789508
SCE1572_8226	-0.464068565	-16.7048	0.656347	0.789508
SCE1572_9440	-0.353885647	-15.0648	0.656635	0.789605
SCE1572_4845	-0.332824032	-15.0543	0.656635	0.789605
SCE1572_1230	-0.303132347	-12.6858	0.656653	0.789605
SCE1572_9647	-0.295484385	-11.5871	0.657051	0.789994
SCE1572_10930	0.325511655	-14.5731	0.657235	0.790124
SCE1572_1054	-0.312450755	-13.4725	0.657766	0.790673
SCE1572_963	0.293361132	-11.8667	0.658957	0.791713
SCE1572_2055	-0.431888159	-16.0258	0.659012	0.791713
SCE1572_2568	0.413074687	-16.0439	0.659012	0.791713
SCE1572_997	-0.390067984	-16.0049	0.659012	0.791713
SCE1572_10979	-0.325871271	-14.4658	0.65933	0.791713
SCE1572_1035	0.324679571	-14.4692	0.65933	0.791713
SCE1572_5047	-0.386900704	-15.5508	0.659535	0.791713
SCE1572_10155	0.360844728	-15.5554	0.659535	0.791713
SCE1572_8428	0.360844728	-15.5554	0.659535	0.791713
SCE1572_8675	0.360844728	-15.5554	0.659535	0.791713
SCE1572_7614	0.360844728	-15.5554	0.659535	0.791713
SCE1572_1918	0.360844728	-15.5554	0.659535	0.791713
SCE1572_1958	-0.312065472	-14.0439	0.660624	0.792929
SCE1572_2804	0.308185779	-13.4118	0.660773	0.793018
SCE1572_788	-0.297263492	-12.8055	0.661184	0.793421
SCE1572_9273	-0.313858753	-14.1062	0.662318	0.794691
SCE1572_7405	-0.351593836	-15.0241	0.662457	0.794768
SCE1572_2257	0.306652628	-13.7752	0.662767	0.795048
SCE1572_4499	0.374939558	-15.3467	0.663121	0.795156
SCE1572_4279	-0.345424068	-15.3236	0.663121	0.795156
SCE1572_7296	-0.345424068	-15.3236	0.663121	0.795156
SCE1572_3814	-0.317227177	-13.8945	0.663159	0.795156
SCE1572_7169	-0.308704503	-13.5974	0.663396	0.795349
SCE1572_10406	-0.311612577	-13.7058	0.663794	0.795553
SCE1572_5176	0.396001174	-15.7723	0.663922	0.795553
SCE1572_8777	0.396001174	-15.7723	0.663922	0.795553
SCE1572_7150	0.396001174	-15.7723	0.663922	0.795553
SCE1572_7615	0.32613905	-14.6468	0.663944	0.795553
SCE1572_10214	-0.315817606	-14.1713	0.664171	0.795734
SCE1572_6572	-0.416177098	-16.2969	0.66464	0.796017
SCE1572_10023	0.371440819	-16.331	0.66464	0.796017
SCE1572_807	0.328489355	-14.8935	0.664785	0.796017

SCE1572_5492	0.328489355	-14.8935	0.664785	0.796017
SCE1572_436	0.328489355	-14.8935	0.664785	0.796017
SCE1572_10916	0.306121542	-13.8823	0.665858	0.797157
SCE1572_8700	0.35006489	-15.1823	0.665889	0.797157
SCE1572_9560	0.340760482	-14.7581	0.666715	0.798055
SCE1572_10807	0.335234085	-14.4307	0.667152	0.798487
SCE1572_5659	-0.294693266	-13.2393	0.668516	0.800028
SCE1572_4712	0.288664345	-12.3061	0.668769	0.800241
SCE1572_3878	0.301155289	-13.6218	0.66927	0.800748
SCE1572_360	-0.299437863	-14.5071	0.669732	0.80121
SCE1572_10574	-0.300569833	-13.5642	0.66996	0.801392
SCE1572_6326	0.728576513	-18.1085	0.670342	0.801485
SCE1572_3783	0.728576513	-18.1085	0.670342	0.801485
SCE1572_9380	0.728576513	-18.1085	0.670342	0.801485
SCE1572_2208	0.728576513	-18.1085	0.670342	0.801485
SCE1572_9906	0.313539014	-14.1461	0.670598	0.801608
SCE1572_2782	0.313539014	-14.1461	0.670598	0.801608
SCE1572_7125	0.313539014	-14.4092	0.670933	0.801917
SCE1572_6543	-0.302229534	-14.0591	0.6715	0.802464
SCE1572_4766	-0.305370819	-13.7842	0.671854	0.802464
SCE1572_8531	-0.336518515	-15.1386	0.67196	0.802464
SCE1572_10185	0.331241015	-15.1373	0.67196	0.802464
SCE1572_5592	0.323523102	-14.3111	0.671981	0.802464
SCE1572_7525	-0.290039165	-12.9174	0.672219	0.802464
SCE1572_1023	0.465542107	-16.6551	0.672306	0.802464
SCE1572_7825	0.465542107	-16.6551	0.672306	0.802464
SCE1572_6444	0.465542107	-16.6551	0.672306	0.802464
SCE1572_2826	0.416632507	-16.6795	0.672306	0.802464
SCE1572_8240	0.416632507	-16.6795	0.672306	0.802464
SCE1572_11590	0.416632507	-16.6795	0.672306	0.802464
SCE1572_3026	-0.367714508	-15.7181	0.672668	0.802714
SCE1572_1531	0.340011225	-15.7178	0.672668	0.802714
SCE1572_5028	0.345247873	-14.9783	0.673545	0.803384
SCE1572_5748	-0.329526442	-14.9746	0.673545	0.803384
SCE1572_113	0.329480558	-14.9861	0.673545	0.803384
SCE1572_5778	-0.309897635	-14.9648	0.673545	0.803384
SCE1572_9346	-0.29188759	-13.054	0.673611	0.803384
SCE1572_785	-0.312843414	-14.4863	0.673704	0.803403
SCE1572_1539	-0.322953788	-14.3864	0.674632	0.804281
SCE1572_5779	0.342393876	-14.8422	0.674836	0.804281
SCE1572_2735	0.358626903	-15.4861	0.674898	0.804281
SCE1572_8222	0.358626903	-15.4861	0.674898	0.804281
SCE1572_7755	-0.353885647	-15.4798	0.674898	0.804281
SCE1572_9202	-0.325871271	-15.4658	0.674898	0.804281
SCE1572_1190	-0.28791161	-12.7423	0.675096	0.804425
SCE1572_89	-0.277628637	-11.3324	0.675174	0.804427
SCE1572_8399	-0.297734764	-13.8316	0.676245	0.8051
SCE1572_5795	0.372432703	-15.2866	0.676549	0.8051

SCE1572_8315	0.372432703	-15.2866	0.676549	0.8051
SCE1572_7458	0.372432703	-15.2866	0.676549	0.8051
SCE1572_7303	0.353067378	-15.2963	0.676549	0.8051
SCE1572_1444	-0.318729202	-15.2629	0.676549	0.8051
SCE1572_5718	-0.318729202	-15.2629	0.676549	0.8051
SCE1572_10514	-0.318729202	-15.2629	0.676549	0.8051
SCE1572_3362	-0.330317176	-14.5791	0.676811	0.8051
SCE1572_1080	0.429016231	-16.2583	0.676811	0.8051
SCE1572_6439	0.391541526	-16.277	0.676811	0.8051
SCE1572_5924	0.391541526	-16.277	0.676811	0.8051
SCE1572_4516	0.391541526	-16.277	0.676811	0.8051
SCE1572_773	-0.364532891	-16.24	0.676811	0.8051
SCE1572_2441	0.293910207	-13.7014	0.677187	0.805456
SCE1572_10399	-0.28884054	-13.2364	0.678329	0.806723
SCE1572_2245	0.288802335	-13.4215	0.678968	0.807222
SCE1572_10433	0.406648418	-15.9476	0.679362	0.807222
SCE1572_4573	0.406648418	-15.9476	0.679362	0.807222
SCE1572_6493	0.406648418	-15.9476	0.679362	0.807222
SCE1572_3660	-0.383898216	-15.9278	0.679362	0.807222
SCE1572_7319	-0.383898216	-15.9278	0.679362	0.807222
SCE1572_524	-0.345424068	-15.9086	0.679362	0.807222
SCE1572_11348	-0.345424068	-15.9086	0.679362	0.807222
SCE1572_10132	0.327614199	-14.8065	0.679644	0.807375
SCE1572_2886	0.327614199	-14.8065	0.679644	0.807375
SCE1572_586	0.296261022	-14.1023	0.679803	0.807472
SCE1572_7193	-0.299662636	-14.0178	0.68021	0.807865
SCE1572_1581	-0.364532891	-15.6551	0.680978	0.808229
SCE1572_7432	-0.364532891	-15.6551	0.680978	0.808229
SCE1572_4484	-0.332824032	-15.6392	0.680978	0.808229
SCE1572_6346	-0.332824032	-15.6392	0.680978	0.808229
SCE1572_9	-0.332824032	-15.6392	0.680978	0.808229
SCE1572_5975	-0.332824032	-15.6392	0.680978	0.808229
SCE1572_11041	-0.280036617	-12.8055	0.681869	0.809196
SCE1572_6023	-0.282230242	-13.1328	0.68199	0.809248
SCE1572_5883	-0.350857955	-15.4259	0.682082	0.809266
SCE1572_7375	0.603045631	-17.1713	0.683327	0.810285
SCE1572_10793	0.603045631	-17.1713	0.683327	0.810285
SCE1572_9831	0.603045631	-17.1713	0.683327	0.810285
SCE1572_6511	0.535931435	-17.2048	0.683327	0.810285
SCE1572_7604	0.535931435	-17.2048	0.683327	0.810285
SCE1572_4035	0.330612527	-15.0851	0.683479	0.810374
SCE1572_3971	0.299532156	-13.7995	0.683632	0.810465
SCE1572_6349	-0.29488246	-13.6661	0.683957	0.810758
SCE1572_2349	-0.302450383	-14.6544	0.684618	0.811451
SCE1572_7467	0.301761846	-14.5493	0.684869	0.811656
SCE1572_10474	-0.311316214	-14.4321	0.685079	0.811814
SCE1572_8429	-0.296085541	-14.3232	0.685259	0.811936
SCE1572_3821	0.282923646	-12.9276	0.685367	0.811973

SCE1572_10373	-0.290039165	-13.9174	0.685864	0.81247
SCE1572_6948	0.274368416	-13.1132	0.686584	0.81309
SCE1572_1667	-0.448301249	-16.5815	0.686852	0.81309
SCE1572_5513	-0.448301249	-16.5815	0.686852	0.81309
SCE1572_7668	0.438282274	-16.5651	0.686852	0.81309
SCE1572_3671	-0.386900704	-16.5508	0.686852	0.81309
SCE1572_9007	-0.386900704	-16.5508	0.686852	0.81309
SCE1572_5957	-0.404690018	-16.1727	0.688138	0.813951
SCE1572_1219	0.35006489	-16.1823	0.688138	0.813951
SCE1572_6883	0.288448033	-14.0556	0.688521	0.813951
SCE1572_9746	0.279986454	-14.0598	0.688521	0.813951
SCE1572_3093	0.293361132	-14.3261	0.688653	0.813951
SCE1572_6750	0.372432703	-15.8716	0.688667	0.813951
SCE1572_4835	0.302812607	-14.4145	0.688701	0.813951
SCE1572_7190	-0.301797136	-14.6237	0.688805	0.813951
SCE1572_9800	0.327213951	-14.7649	0.688856	0.813951
SCE1572_8707	0.327213951	-14.7649	0.688856	0.813951
SCE1572_5728	-0.32146417	-14.7591	0.688856	0.813951
SCE1572_5594	0.363292049	-15.6282	0.688885	0.813951
SCE1572_455	0.363292049	-15.6282	0.688885	0.813951
SCE1572_3706	0.313539014	-15.043	0.688947	0.813951
SCE1572_283	0.313539014	-15.043	0.688947	0.813951
SCE1572_10620	0.313539014	-15.043	0.688947	0.813951
SCE1572_9887	-0.317227177	-15.2164	0.688972	0.813951
SCE1572_8207	-0.317227177	-15.2164	0.688972	0.813951
SCE1572_2456	0.268769144	-12.0165	0.689911	0.81497
SCE1572_9243	0.293775759	-14.2962	0.691976	0.817047
SCE1572_1256	0.293775759	-14.2962	0.691976	0.817047
SCE1572_10346	-0.283496319	-14.2926	0.691976	0.817047
SCE1572_10146	-0.268517756	-12.2378	0.691981	0.817047
SCE1572_9652	0.300118498	-14.7378	0.693271	0.818479
SCE1572_6350	-0.307047397	-14.8537	0.693717	0.818913
SCE1572_10675	0.271103747	-13.0767	0.693961	0.819109
SCE1572_6828	0.331917543	-15.1914	0.694839	0.819962
SCE1572_1045	-0.315817606	-15.1713	0.694839	0.819962
SCE1572_1440	-0.271423487	-13.7341	0.695242	0.820256
SCE1572_1381	0.334297574	-15.3671	0.695557	0.820256
SCE1572_3600	0.334297574	-15.3671	0.695557	0.820256
SCE1572_1898	-0.32204956	-15.3608	0.695557	0.820256
SCE1572_1401	0.313539014	-15.3774	0.695557	0.820256
SCE1572_9974	0.313539014	-15.3774	0.695557	0.820256
SCE1572_10329	-0.330317176	-15.5791	0.696416	0.820993
SCE1572_5987	0.325126988	-15.5849	0.696416	0.820993
SCE1572_1393	0.313539014	-15.5791	0.696416	0.820993
SCE1572_657	0.259252994	-11.5061	0.696755	0.821057
SCE1572_2251	0.272897029	-14.0144	0.696788	0.821057
SCE1572_5991	-0.30117083	-14.5937	0.69686	0.821057
SCE1572_6550	0.289291467	-14.5912	0.69686	0.821057

SCE1572_10404	0.289291467	-14.5912	0.69686	0.821057
SCE1572_10220	-0.282564045	-14.1767	0.697815	0.82209
SCE1572_9275	-0.265474221	-13.2717	0.698266	0.822311
SCE1572_6254	-0.271423487	-13.8536	0.698368	0.822311
SCE1572_4643	0.313539014	-14.8566	0.698385	0.822311
SCE1572_4673	-0.289125489	-14.8448	0.698385	0.822311
SCE1572_300	0.420454218	-16.1471	0.698707	0.822311
SCE1572_4718	0.420454218	-16.1471	0.698707	0.822311
SCE1572_11195	0.420454218	-16.1471	0.698707	0.822311
SCE1572_10745	0.420454218	-16.1471	0.698707	0.822311
SCE1572_3253	0.420454218	-16.1471	0.698707	0.822311
SCE1572_3302	-0.264155487	-12.5605	0.699356	0.822635
SCE1572_5737	-0.278244753	-13.469	0.699791	0.822635
SCE1572_4088	-0.266747	-12.9244	0.700009	0.822635
SCE1572_10577	0.406648418	-16.5325	0.700163	0.822635
SCE1572_11247	0.406648418	-16.5325	0.700163	0.822635
SCE1572_1084	0.406648418	-16.5325	0.700163	0.822635
SCE1572_5807	0.406648418	-16.5325	0.700163	0.822635
SCE1572_3777	0.406648418	-16.5325	0.700163	0.822635
SCE1572_8371	0.406648418	-16.5325	0.700163	0.822635
SCE1572_397	0.406648418	-16.5325	0.700163	0.822635
SCE1572_2068	0.535931435	-17.7898	0.700311	0.822635
SCE1572_6925	0.535931435	-17.7898	0.700311	0.822635
SCE1572_2772	0.535931435	-17.7898	0.700311	0.822635
SCE1572_9985	0.535931435	-17.7898	0.700311	0.822635
SCE1572_8837	0.535931435	-17.7898	0.700311	0.822635
SCE1572_9966	0.535931435	-17.7898	0.700311	0.822635
SCE1572_2515	-0.534457893	-17.74	0.700311	0.822635
SCE1572_6019	0.444783547	-17.028	0.701513	0.823586
SCE1572_9564	0.444783547	-17.028	0.701513	0.823586
SCE1572_5137	-0.42342658	-16.9476	0.701513	0.823586
SCE1572_11164	-0.42342658	-16.9476	0.701513	0.823586
SCE1572_8825	-0.42342658	-16.9476	0.701513	0.823586
SCE1572_11012	-0.283914431	-14.3418	0.702412	0.82455
SCE1572_2080	0.299325154	-14.8207	0.702916	0.824957
SCE1572_4019	-0.288701478	-14.8098	0.702916	0.824957
SCE1572_4045	0.291512707	-14.4526	0.703396	0.825337
SCE1572_6255	-0.271423487	-14.4386	0.703396	0.825337
SCE1572_9439	-0.328007015	-15.5213	0.703597	0.825389
SCE1572_3831	-0.328007015	-15.5213	0.703597	0.825389
SCE1572_9882	0.297942159	-14.9546	0.704296	0.825932
SCE1572_10027	0.288660298	-14.9593	0.704296	0.825932
SCE1572_8434	0.282174843	-14.9625	0.704296	0.825932
SCE1572_2802	0.284969861	-14.2429	0.704603	0.826163
SCE1572_8267	-0.263155871	-13.7464	0.70465	0.826163
SCE1572_5282	-0.265218338	-13.3324	0.705758	0.826826
SCE1572_3439	0.369034126	-15.7858	0.705766	0.826826
SCE1572_447	0.369034126	-15.7858	0.705766	0.826826

SCE1572_9622	0.34155339	-15.7995	0.705766	0.826826
SCE1572_270	0.34155339	-15.7995	0.705766	0.826826
SCE1572_1749	0.34155339	-15.7995	0.705766	0.826826
SCE1572_7257	-0.338537683	-15.7676	0.705766	0.826826
SCE1572_11043	0.266233299	-13.3855	0.70637	0.827441
SCE1572_2416	-0.264227986	-13.546	0.706577	0.827592
SCE1572_8214	0.264629413	-13.4336	0.707024	0.828023
SCE1572_3937	0.282174843	-13.9625	0.707166	0.828098
SCE1572_3017	-0.271423487	-14.2162	0.707604	0.828518
SCE1572_7695	-0.353885647	-16.0648	0.708588	0.828932
SCE1572_9011	-0.353885647	-16.0648	0.708588	0.828932
SCE1572_3399	0.347486346	-16.0767	0.708588	0.828932
SCE1572_743	0.347486346	-16.0767	0.708588	0.828932
SCE1572_1797	0.347486346	-16.0767	0.708588	0.828932
SCE1572_9757	0.347486346	-16.0767	0.708588	0.828932
SCE1572_7486	0.347486346	-16.0767	0.708588	0.828932
SCE1572_5241	-0.312065472	-16.0439	0.708588	0.828932
SCE1572_6226	-0.282070731	-14.1113	0.709297	0.829577
SCE1572_7592	0.269949745	-14.1154	0.709297	0.829577
SCE1572_3559	-0.271423487	-13.9456	0.709653	0.829854
SCE1572_25	-0.271423487	-14.6389	0.709771	0.829854
SCE1572_2045	-0.271423487	-14.6389	0.709771	0.829854
SCE1572_6482	0.269144894	-13.6564	0.710165	0.830222
SCE1572_9699	0.33625909	-15.4973	0.710452	0.830373
SCE1572_6801	0.313539014	-15.5087	0.710452	0.830373
SCE1572_3427	0.26587258	-14.018	0.710784	0.830669
SCE1572_1747	0.267315997	-13.7147	0.711114	0.830704
SCE1572_324	0.260590134	-13.718	0.711114	0.830704
SCE1572_9810	-0.313243663	-15.0851	0.711208	0.830704
SCE1572_5705	-0.313243663	-15.0851	0.711208	0.830704
SCE1572_3312	-0.292182047	-15.0746	0.711208	0.830704
SCE1572_9314	-0.260452369	-13.1546	0.711544	0.830964
SCE1572_2292	-0.288297306	-14.7757	0.711589	0.830964
SCE1572_7551	0.290455401	-14.5202	0.711807	0.831127
SCE1572_9820	0.255823516	-13.5205	0.712207	0.831502
SCE1572_9904	0.356607736	-16.42	0.71239	0.83153
SCE1572_9279	0.356607736	-16.42	0.71239	0.83153
SCE1572_5604	-0.254104011	-12.4913	0.712778	0.83189
SCE1572_7211	0.288664345	-14.628	0.713627	0.83254
SCE1572_215	0.298432121	-14.9086	0.71373	0.83254
SCE1572_8491	0.298432121	-14.9086	0.71373	0.83254
SCE1572_4782	0.298432121	-14.9086	0.71373	0.83254
SCE1572_3778	0.283165365	-14.9162	0.71373	0.83254
SCE1572_8476	0.259767757	-13.5703	0.715045	0.833981
SCE1572_6710	0.244074194	-11.6503	0.715701	0.834517
SCE1572_3381	-0.28791161	-14.7423	0.715742	0.834517
SCE1572_1075	0.272897029	-14.7514	0.715742	0.834517
SCE1572_1668	0.279986454	-15.0598	0.716288	0.834971

SCE1572_10057	0.252784983	-13.3315	0.71629	0.834971
SCE1572_1705	-0.246332506	-12.7631	0.71672	0.835322
SCE1572_7840	-0.271423487	-14.3606	0.716751	0.835322
SCE1572_6422	-0.349425999	-15.9846	0.717848	0.836508
SCE1572_7225	0.253418021	-13.3164	0.719453	0.838286
SCE1572_60	0.264629413	-13.6035	0.719977	0.838804
SCE1572_6659	-0.271423487	-14.5788	0.721047	0.83984
SCE1572_8483	-0.303132347	-15.6858	0.721106	0.83984
SCE1572_10814	-0.271423487	-15.6699	0.721106	0.83984
SCE1572_5288	0.257504979	-13.7997	0.722139	0.840725
SCE1572_768	-0.254349974	-13.7926	0.722139	0.840725
SCE1572_4038	-0.26129927	-14.0387	0.722501	0.840725
SCE1572_11039	0.298892238	-14.8639	0.722591	0.840725
SCE1572_3206	0.251142477	-13.9549	0.723161	0.840725
SCE1572_7606	0.251142477	-13.9549	0.723161	0.840725
SCE1572_11342	-0.323890907	-15.4124	0.723265	0.840725
SCE1572_2539	-0.297418696	-15.3991	0.723265	0.840725
SCE1572_5221	-0.370959161	-16.3364	0.723661	0.840725
SCE1572_10659	-0.255481943	-14.6937	0.723706	0.840725
SCE1572_10676	0.266541534	-14.2241	0.72376	0.840725
SCE1572_5868	0.252138469	-14.1244	0.724423	0.840725
SCE1572_7849	0.235632257	-11.8158	0.724795	0.840725
SCE1572_11417	0.294923336	-15.2099	0.724796	0.840725
SCE1572_9765	-0.271423487	-15.1935	0.724796	0.840725
SCE1572_1638	0.728576513	-17.6935	0.725047	0.840725
SCE1572_9756	0.728576513	-17.6935	0.725047	0.840725
SCE1572_10803	0.728576513	-17.6935	0.725047	0.840725
SCE1572_521	0.728576513	-17.6935	0.725047	0.840725
SCE1572_4430	0.635467109	-17.74	0.725047	0.840725
SCE1572_6433	0.635467109	-17.74	0.725047	0.840725
SCE1572_2841	0.635467109	-17.74	0.725047	0.840725
SCE1572_1448	0.635467109	-17.74	0.725047	0.840725
SCE1572_7047	0.635467109	-17.74	0.725047	0.840725
SCE1572_11223	0.635467109	-17.74	0.725047	0.840725
SCE1572_9509	0.635467109	-17.74	0.725047	0.840725
SCE1572_7359	0.635467109	-17.74	0.725047	0.840725
SCE1572_5274	0.635467109	-17.74	0.725047	0.840725
SCE1572_4615	0.270146418	-14.4309	0.725376	0.840725
SCE1572_7038	0.270146418	-14.4309	0.725376	0.840725
SCE1572_514	0.297419348	-15.0022	0.725938	0.840725
SCE1572_7645	0.297419348	-15.0022	0.725938	0.840725
SCE1572_9399	0.281117536	-15.0103	0.725938	0.840725
SCE1572_4544	0.281117536	-15.0103	0.725938	0.840725
SCE1572_9446	-0.271423487	-14.984	0.725938	0.840725
SCE1572_4571	0.376274769	-15.9627	0.726541	0.840725
SCE1572_6579	0.376274769	-15.9627	0.726541	0.840725
SCE1572_480	0.345247873	-15.9783	0.726541	0.840725
SCE1572_282	1.313539014	-19.401	0.726708	0.840725

SCE1572_2122	1.313539014	-19.401	0.726708	0.840725
SCE1572_7210	1.313539014	-19.401	0.726708	0.840725
SCE1572_6749	1.313539014	-19.401	0.726708	0.840725
SCE1572_9097	1.313539014	-19.401	0.726708	0.840725
SCE1572_4560	1.313539014	-19.401	0.726708	0.840725
SCE1572_8004	1.313539014	-19.401	0.726708	0.840725
SCE1572_589	1.313539014	-19.401	0.726708	0.840725
SCE1572_2768	1.313539014	-19.401	0.726708	0.840725
SCE1572_818	1.313539014	-19.401	0.726708	0.840725
SCE1572_5210	1.313539014	-19.401	0.726708	0.840725
SCE1572_3729	1.313539014	-19.401	0.726708	0.840725
SCE1572_1693	1.313539014	-19.401	0.726708	0.840725
SCE1572_5481	1.313539014	-19.401	0.726708	0.840725
SCE1572_5075	1.313539014	-19.401	0.726708	0.840725
SCE1572_10468	1.313539014	-19.401	0.726708	0.840725
SCE1572_10809	-1.271423487	-19.1085	0.726708	0.840725
SCE1572_1469	-0.856385988	-18.901	0.726708	0.840725
SCE1572_4563	-0.856385988	-18.901	0.726708	0.840725
SCE1572_6217	-0.856385988	-18.901	0.726708	0.840725
SCE1572_3360	0.284969861	-14.8278	0.726822	0.840725
SCE1572_281	0.284969861	-14.8278	0.726822	0.840725
SCE1572_3418	0.270470292	-14.8351	0.726822	0.840725
SCE1572_4095	0.270470292	-14.8351	0.726822	0.840725
SCE1572_4820	-0.271423487	-14.6699	0.727525	0.841446
SCE1572_8892	-0.262085622	-13.922	0.727827	0.84161
SCE1572_8469	-0.262085622	-13.922	0.727827	0.84161
SCE1572_8544	0.338629995	-15.6405	0.728199	0.841751
SCE1572_11432	0.338629995	-15.6405	0.728199	0.841751
SCE1572_3082	0.313539014	-15.6531	0.728199	0.841751
SCE1572_8030	0.244053913	-12.9768	0.728269	0.841751
SCE1572_6547	-0.236273032	-12.375	0.728508	0.841919
SCE1572_1425	-0.271423487	-14.4121	0.728574	0.841919
SCE1572_6384	0.292477398	-15.388	0.729261	0.842527
SCE1572_3067	0.292477398	-15.388	0.729261	0.842527
SCE1572_11268	-0.243942751	-13.4793	0.729357	0.842545
SCE1572_8792	-0.271423487	-15.1491	0.729993	0.843188
SCE1572_9608	0.266233299	-14.9705	0.730525	0.843617
SCE1572_9207	0.266233299	-14.9705	0.730525	0.843617
SCE1572_5555	-0.230200824	-11.7424	0.730843	0.843891
SCE1572_6187	-0.271423487	-14.8012	0.730928	0.843897
SCE1572_6921	0.236672722	-12.5772	0.731368	0.844312
SCE1572_11566	0.271103747	-14.3987	0.731701	0.844511
SCE1572_3791	0.260296992	-14.4041	0.731701	0.844511
SCE1572_7931	0.249813416	-14.178	0.732016	0.844526
SCE1572_6779	0.429016231	-16.8433	0.732196	0.844526
SCE1572_6914	0.429016231	-16.8433	0.732196	0.844526
SCE1572_9745	0.429016231	-16.8433	0.732196	0.844526
SCE1572_8289	-0.408927011	-16.8028	0.732196	0.844526

SCE1572_457	-0.338537683	-16.7676	0.732196	0.844526
SCE1572_39	0.243149686	-13.6694	0.732559	0.844853
SCE1572_6965	0.353067378	-16.2963	0.734082	0.846283
SCE1572_9789	0.353067378	-16.2963	0.734082	0.846283
SCE1572_7479	0.25819598	-13.9745	0.734579	0.846283
SCE1572_567	-0.249397181	-14.1601	0.734662	0.846283
SCE1572_8515	0.333960517	-15.8998	0.734719	0.846283
SCE1572_10963	0.313539014	-15.901	0.734719	0.846283
SCE1572_2728	0.313539014	-15.901	0.734719	0.846283
SCE1572_522	-0.307949363	-15.8898	0.734719	0.846283
SCE1572_2440	0.285794023	-14.7856	0.734915	0.846283
SCE1572_5799	0.289291467	-15.5912	0.734943	0.846283
SCE1572_11140	0.289291467	-15.5912	0.734943	0.846283
SCE1572_4341	-0.271423487	-14.9456	0.734964	0.846283
SCE1572_2723	-0.296514468	-15.3481	0.735006	0.846283
SCE1572_8525	-0.296514468	-15.3481	0.735006	0.846283
SCE1572_5325	-0.271423487	-15.3355	0.735006	0.846283
SCE1572_7102	-0.239002009	-13.7178	0.736428	0.847827
SCE1572_7241	0.233921846	-12.7216	0.737128	0.84854
SCE1572_5739	0.260099755	-14.1464	0.737256	0.848595
SCE1572_8470	-0.243942751	-14.4793	0.738042	0.849407
SCE1572_4843	-0.256473146	-14.6011	0.738386	0.849709
SCE1572_6504	-0.238256623	-14.7506	0.738787	0.850077
SCE1572_10658	0.220104798	-10.5152	0.739101	0.850346
SCE1572_8439	0.278773596	-15.111	0.739827	0.850995
SCE1572_8421	-0.271423487	-15.106	0.739827	0.850995
SCE1572_3765	-0.248339874	-14.2278	0.740206	0.851338
SCE1572_8889	0.313539014	-15.316	0.740515	0.851507
SCE1572_10957	-0.295671033	-15.2987	0.740515	0.851507
SCE1572_8348	0.232920553	-13.4178	0.74077	0.851708
SCE1572_1592	-0.222235937	-12.7343	0.74121	0.85212
SCE1572_10623	-0.221384185	-12.6921	0.741979	0.852911
SCE1572_10794	-0.341812815	-15.8364	0.742425	0.853051
SCE1572_3988	-0.341812815	-15.8364	0.742425	0.853051
SCE1572_7795	-0.306188905	-15.8186	0.742425	0.853051
SCE1572_7712	-0.306188905	-15.8186	0.742425	0.853051
SCE1572_2066	0.224685623	-12.5108	0.742755	0.853337
SCE1572_7399	-0.317227177	-16.2164	0.743746	0.853823
SCE1572_5844	0.313539014	-16.2006	0.743746	0.853823
SCE1572_6673	0.276064308	-16.2193	0.743746	0.853823
SCE1572_10135	0.276064308	-16.2193	0.743746	0.853823
SCE1572_6489	-0.271423487	-16.1935	0.743746	0.853823
SCE1572_8248	-0.271423487	-16.1935	0.743746	0.853823
SCE1572_10207	-0.271423487	-16.1935	0.743746	0.853823
SCE1572_9153	0.235836657	-13.4641	0.74428	0.854192
SCE1572_2143	-0.271423487	-15.0642	0.744483	0.854192
SCE1572_2063	0.245634949	-15.077	0.744483	0.854192
SCE1572_5068	-0.22154758	-12.5319	0.744662	0.854192

SCE1572_8858	0.244414653	-14.1027	0.744749	0.854192
SCE1572_11135	-0.242277141	-14.5642	0.745165	0.854192
SCE1572_10039	-0.396954369	-16.6713	0.745262	0.854192
SCE1572_4131	-0.396954369	-16.6713	0.745262	0.854192
SCE1572_9386	-0.396954369	-16.6713	0.745262	0.854192
SCE1572_8444	0.366006434	-16.7048	0.745262	0.854192
SCE1572_8736	0.366006434	-16.7048	0.745262	0.854192
SCE1572_420	0.366006434	-16.7048	0.745262	0.854192
SCE1572_1399	0.366006434	-16.7048	0.745262	0.854192
SCE1572_1714	0.274544882	-15.2766	0.745803	0.854192
SCE1572_6189	0.274544882	-15.2766	0.745803	0.854192
SCE1572_4183	0.494111259	-17.4888	0.745855	0.854192
SCE1572_3519	0.494111259	-17.4888	0.745855	0.854192
SCE1572_7561	0.494111259	-17.4888	0.745855	0.854192
SCE1572_2740	0.494111259	-17.4888	0.745855	0.854192
SCE1572_1343	0.494111259	-17.4888	0.745855	0.854192
SCE1572_7440	0.494111259	-17.4888	0.745855	0.854192
SCE1572_4396	-0.493815908	-17.4973	0.745855	0.854192
SCE1572_5328	0.236917732	-14.0813	0.747154	0.855128
SCE1572_7010	0.279924733	-14.8734	0.747454	0.855128
SCE1572_7091	0.269144894	-14.8788	0.747454	0.855128
SCE1572_10991	-0.253501579	-14.8626	0.747454	0.855128
SCE1572_7876	0.290455401	-15.5202	0.747485	0.855128
SCE1572_1529	0.290455401	-15.5202	0.747485	0.855128
SCE1572_10130	0.290455401	-15.5202	0.747485	0.855128
SCE1572_317	0.290455401	-15.5202	0.747485	0.855128
SCE1572_4492	0.290455401	-15.5202	0.747485	0.855128
SCE1572_9285	-0.271423487	-15.493	0.747485	0.855128
SCE1572_6601	-0.213872464	-12.1535	0.747628	0.855198
SCE1572_5096	-0.220284638	-13.568	0.748643	0.856266
SCE1572_5791	-0.235203299	-14.2927	0.749065	0.856655
SCE1572_7355	0.217723226	-12.5739	0.749522	0.857008
SCE1572_4173	-0.219334976	-13.08	0.749633	0.857008
SCE1572_7135	0.313539014	-15.8135	0.749699	0.857008
SCE1572_4805	0.313539014	-15.8135	0.749699	0.857008
SCE1572_1485	0.232342931	-13.8755	0.74985	0.857088
SCE1572_511	0.220188537	-12.8924	0.749942	0.857099
SCE1572_9852	-0.271423487	-15.2393	0.750881	0.857987
SCE1572_10696	-0.248339874	-15.2278	0.750881	0.857987
SCE1572_5511	0.241026514	-14.8498	0.751363	0.858444
SCE1572_1241	-0.314492209	-16.1276	0.752733	0.859658
SCE1572_7357	-0.314492209	-16.1276	0.752733	0.859658
SCE1572_9132	0.241837548	-14.1555	0.753019	0.859658
SCE1572_4202	-0.255826632	-14.6621	0.753221	0.859658
SCE1572_11179	-0.240396591	-14.6544	0.753221	0.859658
SCE1572_6329	0.264629413	-15.0186	0.75332	0.859658
SCE1572_189	-0.231895123	-15.0038	0.75332	0.859658
SCE1572_9585	0.269144894	-15.4638	0.753325	0.859658

SCE1572_6679	0.269144894	-15.4638	0.753325	0.859658
SCE1572_5281	0.246424818	-15.4751	0.753325	0.859658
SCE1572_6340	0.246424818	-15.4751	0.753325	0.859658
SCE1572_42	0.233049096	-14.515	0.754708	0.861143
SCE1572_145	0.276064308	-15.2193	0.755763	0.86216
SCE1572_5178	0.276064308	-15.2193	0.755763	0.86216
SCE1572_1936	0.214891426	-13.0924	0.756245	0.862432
SCE1572_4785	-0.221498262	-14.0188	0.756344	0.862432
SCE1572_7638	-0.304590351	-15.7506	0.756575	0.862432
SCE1572_5673	-0.304590351	-15.7506	0.756575	0.862432
SCE1572_9862	0.286571966	-15.7446	0.756575	0.862432
SCE1572_2813	-0.271423487	-15.7341	0.756575	0.862432
SCE1572_11192	0.25909123	-15.7583	0.756575	0.862432
SCE1572_8625	-0.224880901	-14.2395	0.756942	0.862469
SCE1572_7579	-0.216975703	-13.1439	0.757006	0.862469
SCE1572_6994	-0.271423487	-16.6085	0.7571	0.862469
SCE1572_4220	-0.271423487	-16.6085	0.7571	0.862469
SCE1572_250	-0.271423487	-16.6085	0.7571	0.862469
SCE1572_5824	-0.271423487	-16.6085	0.7571	0.862469
SCE1572_10756	0.257504979	-14.7997	0.758834	0.864242
SCE1572_298	-0.271423487	-15.4386	0.758903	0.864242
SCE1572_3291	-0.244951276	-15.4254	0.758903	0.864242
SCE1572_8489	-0.228096055	-13.8143	0.759739	0.865055
SCE1572_7770	-0.241049838	-14.6237	0.759863	0.865055
SCE1572_4150	-0.241049838	-14.6237	0.759863	0.865055
SCE1572_2475	0.235536502	-14.1071	0.760126	0.86526
SCE1572_4016	0.25909123	-15.1733	0.76046	0.865453
SCE1572_8511	0.25909123	-15.1733	0.76046	0.865453
SCE1572_4480	0.313539014	-16.0937	0.761112	0.866101
SCE1572_2766	0.222391126	-14.3319	0.761364	0.866294
SCE1572_4850	0.245152039	-14.7653	0.762406	0.867386
SCE1572_9322	0.225408103	-14.4532	0.763571	0.868494
SCE1572_7594	-0.464068565	-17.2898	0.763627	0.868494
SCE1572_7971	-0.464068565	-17.2898	0.763627	0.868494
SCE1572_9903	-0.222513887	-14.3111	0.763994	0.868817
SCE1572_2821	0.207928826	-13.5444	0.76422	0.86898
SCE1572_4569	0.252138469	-14.9317	0.765497	0.870244
SCE1572_4930	-0.233948782	-14.9268	0.765497	0.870244
SCE1572_7295	-0.204309291	-13.4457	0.767341	0.871004
SCE1572_805	0.212178299	-13.1315	0.767518	0.871004
SCE1572_5201	0.360844728	-16.5554	0.767877	0.871004
SCE1572_6411	0.360844728	-16.5554	0.767877	0.871004
SCE1572_7778	0.360844728	-16.5554	0.767877	0.871004
SCE1572_8757	0.360844728	-16.5554	0.767877	0.871004
SCE1572_4409	0.360844728	-16.5554	0.767877	0.871004
SCE1572_3152	0.199882232	-12.9134	0.768617	0.871004
SCE1572_7644	0.211358619	-13.6667	0.768855	0.871004
SCE1572_4478	-0.271423487	-16.0236	0.768941	0.871004

SCE1572_6025	-0.271423487	-16.0236	0.768941	0.871004
SCE1572_5355	-0.271423487	-16.0236	0.768941	0.871004
SCE1572_4711	0.220429609	-13.9476	0.768956	0.871004
SCE1572_7879	0.20539021	-14.0482	0.768996	0.871004
SCE1572_7310	0.288003922	-15.6658	0.76926	0.871004
SCE1572_3524	0.261071594	-15.1199	0.769335	0.871004
SCE1572_4018	0.243149686	-15.1288	0.769335	0.871004
SCE1572_3501	0.243149686	-15.1288	0.769335	0.871004
SCE1572_4098	0.728576513	-18.6935	0.769726	0.871004
SCE1572_7046	0.728576513	-18.6935	0.769726	0.871004
SCE1572_8054	0.728576513	-18.6935	0.769726	0.871004
SCE1572_10844	0.728576513	-18.6935	0.769726	0.871004
SCE1572_6371	0.728576513	-18.6935	0.769726	0.871004
SCE1572_8927	0.728576513	-18.6935	0.769726	0.871004
SCE1572_9620	0.728576513	-18.6935	0.769726	0.871004
SCE1572_8923	0.728576513	-18.6935	0.769726	0.871004
SCE1572_11402	0.728576513	-18.6935	0.769726	0.871004
SCE1572_842	0.728576513	-18.6935	0.769726	0.871004
SCE1572_11336	0.728576513	-18.6935	0.769726	0.871004
SCE1572_6851	0.728576513	-18.6935	0.769726	0.871004
SCE1572_5898	0.535931435	-18.7898	0.769726	0.871004
SCE1572_832	0.535931435	-18.7898	0.769726	0.871004
SCE1572_3304	0.535931435	-18.7898	0.769726	0.871004
SCE1572_5611	0.535931435	-18.7898	0.769726	0.871004
SCE1572_8224	0.535931435	-18.7898	0.769726	0.871004
SCE1572_4157	0.535931435	-18.7898	0.769726	0.871004
SCE1572_5209	0.535931435	-18.7898	0.769726	0.871004
SCE1572_8096	0.535931435	-18.7898	0.769726	0.871004
SCE1572_2173	0.535931435	-18.7898	0.769726	0.871004
SCE1572_10412	0.535931435	-18.7898	0.769726	0.871004
SCE1572_1723	0.535931435	-18.7898	0.769726	0.871004
SCE1572_4896	0.535931435	-18.7898	0.769726	0.871004
SCE1572_9676	-0.534457893	-18.74	0.769726	0.871004
SCE1572_4171	-0.202710737	-13.2166	0.772399	0.873788
SCE1572_1269	0.222115986	-14.6988	0.772519	0.873788
SCE1572_7376	0.222115986	-14.6988	0.772519	0.873788
SCE1572_10797	0.222115986	-14.6988	0.772519	0.873788
SCE1572_7797	0.254038002	-14.8864	0.772975	0.874209
SCE1572_10641	-0.229603311	-15.0851	0.773533	0.874559
SCE1572_5504	0.228147522	-15.0857	0.773533	0.874559
SCE1572_7201	0.228147522	-15.0857	0.773533	0.874559
SCE1572_5145	-0.246761433	-15.3232	0.774231	0.87516
SCE1572_9467	-0.246761433	-15.3232	0.774231	0.87516
SCE1572_4156	-0.20729315	-13.8946	0.774791	0.87532
SCE1572_2355	-0.271423487	-15.6085	0.775122	0.87532
SCE1572_9480	-0.271423487	-15.6085	0.775122	0.87532
SCE1572_7265	0.264629413	-15.6035	0.775122	0.87532
SCE1572_1385	0.264629413	-15.6035	0.775122	0.87532

SCE1572_2609	0.264629413	-15.6035	0.775122	0.87532
SCE1572_5000	-0.241676144	-15.5937	0.775122	0.87532
SCE1572_5478	0.239538432	-15.6161	0.775122	0.87532
SCE1572_11291	0.239538432	-15.6161	0.775122	0.87532
SCE1572_1103	0.195935047	-12.9154	0.775651	0.875781
SCE1572_3521	-0.206717798	-13.393	0.775696	0.875781
SCE1572_5026	0.313539014	-15.9941	0.776274	0.876245
SCE1572_4067	-0.271423487	-15.9456	0.776274	0.876245
SCE1572_7804	0.197530906	-12.7206	0.776485	0.876389
SCE1572_2242	0.220429609	-14.3626	0.777016	0.8768
SCE1572_7848	-0.20841369	-14.3546	0.777016	0.8768
SCE1572_9505	0.313539014	-16.4416	0.77773	0.877041
SCE1572_9085	0.313539014	-16.4416	0.77773	0.877041
SCE1572_1211	0.313539014	-16.4416	0.77773	0.877041
SCE1572_5903	0.313539014	-16.4416	0.77773	0.877041
SCE1572_3416	0.313539014	-16.4416	0.77773	0.877041
SCE1572_5638	0.313539014	-16.4416	0.77773	0.877041
SCE1572_10723	-0.203519423	-13.7845	0.777912	0.877153
SCE1572_5391	0.203775447	-13.4481	0.778833	0.878006
SCE1572_11143	-0.247576745	-15.2747	0.778919	0.878006
SCE1572_10504	-0.247576745	-15.2747	0.778919	0.878006
SCE1572_3874	-0.213320532	-13.9746	0.779213	0.878243
SCE1572_202	0.20132951	-14.3424	0.779525	0.878501
SCE1572_1316	0.21209375	-14.1968	0.780915	0.879973
SCE1572_8538	0.231076853	-15.0353	0.78149	0.880527
SCE1572_4567	0.21400334	-14.6288	0.781815	0.880799
SCE1572_3673	-0.204752447	-13.9507	0.783054	0.882026
SCE1572_8679	0.283165365	-15.9162	0.783156	0.882026
SCE1572_5568	0.252138469	-15.9317	0.783156	0.882026
SCE1572_4024	0.183860467	-11.396	0.783642	0.882478
SCE1572_11390	0.217323698	-14.9949	0.785265	0.883987
SCE1572_6181	-0.212529798	-14.9941	0.785265	0.883987
SCE1572_5708	0.21400334	-14.1694	0.785318	0.883987
SCE1572_5069	0.204604642	-14.1741	0.785318	0.883987
SCE1572_5072	0.266996428	-15.532	0.785998	0.884658
SCE1572_9269	-0.189629396	-13.2457	0.786381	0.884995
SCE1572_982	0.216862994	-14.7794	0.786598	0.885144
SCE1572_3763	-0.271423487	-16.3861	0.786774	0.885153
SCE1572_10425	-0.271423487	-16.3861	0.786774	0.885153
SCE1572_9481	0.19324478	-13.3173	0.787175	0.885508
SCE1572_5995	0.20662381	-14.1471	0.787454	0.885728
SCE1572_11206	-0.190503492	-13.4526	0.787665	0.885764
SCE1572_6505	0.256955485	-15.2289	0.787739	0.885764
SCE1572_3160	0.256955485	-15.2289	0.787739	0.885764
SCE1572_6307	-0.190805027	-13.7102	0.787831	0.885773
SCE1572_2590	-0.188434122	-13.9041	0.788574	0.886514
SCE1572_333	-0.214090312	-14.9554	0.788912	0.886798
SCE1572_9883	-0.271423487	-15.8716	0.789628	0.887034

SCE1572_3743	-0.235799577	-15.8537	0.789628	0.887034
SCE1572_958	-0.235799577	-15.8537	0.789628	0.887034
SCE1572_11173	-0.235799577	-15.8537	0.789628	0.887034
SCE1572_264	-0.235799577	-15.8537	0.789628	0.887034
SCE1572_9205	-0.235799577	-15.8537	0.789628	0.887034
SCE1572_8323	-0.243942751	-15.4793	0.791053	0.888446
SCE1572_2909	0.223341205	-15.4867	0.791053	0.888446
SCE1572_7343	-0.201034159	-14.2514	0.791279	0.888489
SCE1572_10778	0.199467247	-14.1252	0.791603	0.888489
SCE1572_8393	0.200644957	-14.0033	0.791873	0.888489
SCE1572_9802	0.221616524	-15.1921	0.791892	0.888489
SCE1572_10257	0.221616524	-15.1921	0.791892	0.888489
SCE1572_2074	0.38065321	-17.0601	0.792443	0.888489
SCE1572_9771	0.38065321	-17.0601	0.792443	0.888489
SCE1572_4732	0.38065321	-17.0601	0.792443	0.888489
SCE1572_10672	-0.353885647	-17.0648	0.792443	0.888489
SCE1572_3939	-0.353885647	-17.0648	0.792443	0.888489
SCE1572_1818	-0.353885647	-17.0648	0.792443	0.888489
SCE1572_10550	0.313539014	-17.0937	0.792443	0.888489
SCE1572_2604	0.313539014	-17.0937	0.792443	0.888489
SCE1572_3659	-0.271423487	-17.0236	0.792443	0.888489
SCE1572_1955	-0.271423487	-17.0236	0.792443	0.888489
SCE1572_4912	-0.271423487	-17.0236	0.792443	0.888489
SCE1572_4275	-0.205835145	-14.7344	0.792819	0.888721
SCE1572_3285	-0.205835145	-14.7344	0.792819	0.888721
SCE1572_4799	-0.183960646	-13.3424	0.792969	0.888795
SCE1572_11591	0.192523613	-14.1035	0.793618	0.889427
SCE1572_2837	0.185214917	-13.4106	0.794126	0.889902
SCE1572_10148	-0.320333088	-16.3111	0.795106	0.89022
SCE1572_10438	-0.320333088	-16.3111	0.795106	0.89022
SCE1572_7818	-0.320333088	-16.3111	0.795106	0.89022
SCE1572_4356	0.313539014	-16.316	0.795106	0.89022
SCE1572_10336	0.313539014	-16.316	0.795106	0.89022
SCE1572_2825	0.313539014	-16.316	0.795106	0.89022
SCE1572_6525	0.313539014	-16.316	0.795106	0.89022
SCE1572_2239	0.191548489	-13.7525	0.795481	0.89022
SCE1572_2596	-0.202279701	-14.086	0.795594	0.89022
SCE1572_9951	0.209703203	-14.368	0.795689	0.89022
SCE1572_10846	0.284969861	-15.8278	0.795724	0.89022
SCE1572_1810	0.255823516	-15.8424	0.795724	0.89022
SCE1572_2057	0.255823516	-15.8424	0.795724	0.89022
SCE1572_6277	-0.20729315	-14.702	0.795797	0.89022
SCE1572_8372	-0.215570252	-14.9176	0.795849	0.89022
SCE1572_5692	0.20774435	-14.9095	0.795849	0.89022
SCE1572_9313	0.191977034	-14.9174	0.795849	0.89022
SCE1572_918	-0.179394349	-12.7638	0.796823	0.891215
SCE1572_3720	0.196182063	-13.9597	0.797345	0.891704
SCE1572_3779	0.194661618	-14.0778	0.797534	0.89182

SCE1572_7858	-0.183136955	-13.4628	0.797977	0.892164
SCE1572_4594	0.190682266	-14.3477	0.798011	0.892164
SCE1572_9276	0.19324478	-13.7323	0.798569	0.892568
SCE1572_6087	-0.183960646	-13.8278	0.79882	0.892568
SCE1572_10951	-0.188961327	-14.0648	0.799437	0.892568
SCE1572_9788	0.188008132	-14.0569	0.799437	0.892568
SCE1572_9458	-0.228354765	-15.1276	0.799731	0.892568
SCE1572_5322	-0.20729315	-15.117	0.799731	0.892568
SCE1572_1470	0.18520387	-13.6255	0.799767	0.892568
SCE1572_1272	-0.686460986	-18.401	0.7999	0.892568
SCE1572_8636	-0.686460986	-18.401	0.7999	0.892568
SCE1572_6771	-0.686460986	-18.401	0.7999	0.892568
SCE1572_10687	-0.686460986	-18.401	0.7999	0.892568
SCE1572_2560	-0.464068565	-18.2898	0.7999	0.892568
SCE1572_11523	-0.464068565	-18.2898	0.7999	0.892568
SCE1572_9572	-0.464068565	-18.2898	0.7999	0.892568
SCE1572_8223	-0.464068565	-18.2898	0.7999	0.892568
SCE1572_10426	-0.464068565	-18.2898	0.7999	0.892568
SCE1572_7666	-0.464068565	-18.2898	0.7999	0.892568
SCE1572_2781	-0.464068565	-18.2898	0.7999	0.892568
SCE1572_6637	-0.199588573	-14.3247	0.800281	0.892899
SCE1572_11574	-0.202381843	-14.4865	0.800831	0.89333
SCE1572_6480	-0.196461429	-13.9272	0.800837	0.89333
SCE1572_10968	0.231076853	-15.7723	0.801477	0.8936
SCE1572_3652	0.231076853	-15.7723	0.801477	0.8936
SCE1572_3802	0.231076853	-15.7723	0.801477	0.8936
SCE1572_6624	-0.204309291	-15.7676	0.801477	0.8936
SCE1572_323	-0.193420975	-14.6626	0.801505	0.8936
SCE1572_7076	-0.18261422	-14.1718	0.801853	0.893701
SCE1572_11457	-0.169002422	-11.7629	0.801967	0.893701
SCE1572_999	0.170225742	-12.8331	0.801986	0.893701
SCE1572_5959	0.193539238	-13.8113	0.80202	0.893701
SCE1572_3480	-0.185008735	-13.8104	0.80202	0.893701
SCE1572_7177	0.23759016	-16.2385	0.802807	0.894294
SCE1572_980	0.23759016	-16.2385	0.802807	0.894294
SCE1572_8481	0.23759016	-16.2385	0.802807	0.894294
SCE1572_4633	0.210445521	-15.0946	0.803434	0.894803
SCE1572_3530	0.192523613	-15.1035	0.803434	0.894803
SCE1572_9706	0.17603549	-13.3404	0.803691	0.894995
SCE1572_10219	0.186049279	-13.8987	0.804212	0.895013
SCE1572_11013	-0.195072601	-14.6318	0.804241	0.895013
SCE1572_1133	-0.345424068	-16.9086	0.804303	0.895013
SCE1572_1821	-0.345424068	-16.9086	0.804303	0.895013
SCE1572_7520	-0.345424068	-16.9086	0.804303	0.895013
SCE1572_1878	-0.345424068	-16.9086	0.804303	0.895013
SCE1572_138	-0.345424068	-16.9086	0.804303	0.895013
SCE1572_8698	0.175092244	-14.2978	0.804673	0.89533
SCE1572_3351	-0.177447339	-14.4461	0.805675	0.89635

SCE1572_368	-0.169325299	-12.9136	0.80598	0.896594
SCE1572_10289	-0.170729487	-13.7681	0.806635	0.897215
SCE1572_11005	-0.182765867	-13.9994	0.806708	0.897215
SCE1572_11101	-0.239002009	-15.7178	0.806915	0.897255
SCE1572_5097	-0.20729315	-15.702	0.806915	0.897255
SCE1572_1627	0.163398664	-11.5498	0.807683	0.898013
SCE1572_7741	0.205014557	-14.4317	0.80801	0.898283
SCE1572_9494	-0.162620276	-11.9103	0.808149	0.898342
SCE1572_10211	0.21400334	-14.8215	0.808448	0.898579
SCE1572_7318	0.252138469	-15.3467	0.809098	0.899207
SCE1572_4227	-0.182156149	-14.5943	0.809496	0.899554
SCE1572_9703	-0.227029368	-16.1713	0.809946	0.899864
SCE1572_1879	-0.227029368	-16.1713	0.809946	0.899864
SCE1572_2641	0.181435478	-13.6451	0.810049	0.899883
SCE1572_11129	-0.210881945	-15.0339	0.810446	0.90023
SCE1572_4271	0.235536502	-15.6921	0.812063	0.901645
SCE1572_401	0.235536502	-15.6921	0.812063	0.901645
SCE1572_9507	0.235536502	-15.6921	0.812063	0.901645
SCE1572_10445	0.235536502	-15.6921	0.812063	0.901645
SCE1572_5332	-0.163508148	-12.453	0.812808	0.902377
SCE1572_9050	-0.198667145	-15.2991	0.813134	0.902643
SCE1572_1128	0.200644957	-15.0033	0.813769	0.903253
SCE1572_1097	0.188008132	-14.3788	0.814693	0.903699
SCE1572_2504	0.372432703	-16.8716	0.814851	0.903699
SCE1572_2988	0.372432703	-16.8716	0.814851	0.903699
SCE1572_8933	0.313539014	-16.901	0.814851	0.903699
SCE1572_4899	0.313539014	-16.901	0.814851	0.903699
SCE1572_9873	0.313539014	-16.901	0.814851	0.903699
SCE1572_9952	0.313539014	-16.901	0.814851	0.903699
SCE1572_9700	-0.168737823	-14.2114	0.814858	0.903699
SCE1572_11535	-0.167993897	-13.9323	0.815094	0.903865
SCE1572_8314	-0.156681401	-12.8977	0.815621	0.904354
SCE1572_7746	-0.162124845	-12.384	0.815759	0.904412
SCE1572_9897	0.278773596	-16.111	0.816583	0.904562
SCE1572_761	0.278773596	-16.111	0.816583	0.904562
SCE1572_5133	-0.271423487	-16.106	0.816583	0.904562
SCE1572_1715	-0.271423487	-16.106	0.816583	0.904562
SCE1572_8379	0.243149686	-16.1288	0.816583	0.904562
SCE1572_10884	0.243149686	-16.1288	0.816583	0.904562
SCE1572_5606	0.243149686	-16.1288	0.816583	0.904562
SCE1572_3965	0.243149686	-16.1288	0.816583	0.904562
SCE1572_4296	0.205623675	-14.7455	0.816928	0.904677
SCE1572_6609	0.21400334	-15.6288	0.816944	0.904677
SCE1572_5159	-0.210022942	-15.6392	0.816944	0.904677
SCE1572_1748	0.160029975	-12.828	0.817158	0.904818
SCE1572_1236	-0.156780921	-13.0809	0.817357	0.904916
SCE1572_5341	-0.167351033	-13.1528	0.817419	0.904916
SCE1572_10049	0.17603549	-14.1884	0.818643	0.906176

SCE1572_1165	-0.161798996	-13.5688	0.819079	0.906563
SCE1572_3759	-0.172405748	-13.8932	0.819787	0.907194
SCE1572_9731	0.16758266	-13.3741	0.819821	0.907194
SCE1572_8459	-0.195474634	-14.9461	0.820079	0.907384
SCE1572_8721	0.169149104	-14.1658	0.820478	0.90773
SCE1572_3587	0.158260788	-13.3639	0.820891	0.908091
SCE1572_3921	-0.160392175	-14.4941	0.821483	0.908651
SCE1572_9227	-0.213395675	-15.5795	0.821578	0.90866
SCE1572_11109	-0.189371242	-14.693	0.822178	0.909193
SCE1572_6293	-0.173391404	-14.1445	0.822277	0.909193
SCE1572_9542	0.603045631	-18.1713	0.822492	0.909193
SCE1572_10350	0.603045631	-18.1713	0.822492	0.909193
SCE1572_5131	0.603045631	-18.1713	0.822492	0.909193
SCE1572_6067	0.21400334	-16.0439	0.82277	0.909213
SCE1572_2529	0.21400334	-16.0439	0.82277	0.909213
SCE1572_6117	0.21400334	-16.0439	0.82277	0.909213
SCE1572_10799	0.159915825	-13.3485	0.822992	0.909363
SCE1572_63	0.167460754	-14.4822	0.823703	0.909468
SCE1572_11260	-0.160392175	-13.1722	0.824147	0.909468
SCE1572_8643	-0.203251984	-15.2052	0.824282	0.909468
SCE1572_10119	0.183142376	-15.2113	0.824282	0.909468
SCE1572_7181	0.183142376	-15.2113	0.824282	0.909468
SCE1572_3586	0.313539014	-16.7311	0.824298	0.909468
SCE1572_7980	0.313539014	-16.7311	0.824298	0.909468
SCE1572_3380	-0.271423487	-16.7341	0.824298	0.909468
SCE1572_10470	-0.271423487	-16.7341	0.824298	0.909468
SCE1572_9218	0.25909123	-16.7583	0.824298	0.909468
SCE1572_6038	0.25909123	-16.7583	0.824298	0.909468
SCE1572_9125	0.25909123	-16.7583	0.824298	0.909468
SCE1572_8445	0.25909123	-16.7583	0.824298	0.909468
SCE1572_2222	0.25909123	-16.7583	0.824298	0.909468
SCE1572_5155	-0.165114585	-13.7309	0.824994	0.910046
SCE1572_6581	-0.157252467	-13.727	0.824994	0.910046
SCE1572_6562	0.21890214	-15.556	0.825983	0.911041
SCE1572_4825	-0.152587258	-13.5047	0.826167	0.911149
SCE1572_1744	-0.15594627	-13.9658	0.826502	0.911327
SCE1572_3192	-0.15594627	-13.9658	0.826502	0.911327
SCE1572_7826	-0.166726108	-13.602	0.826841	0.911605
SCE1572_357	-0.158529431	-13.7108	0.82761	0.912041
SCE1572_5259	0.281117536	-16.0103	0.828551	0.912041
SCE1572_11470	0.281117536	-16.0103	0.828551	0.912041
SCE1572_3873	0.247950672	-16.0269	0.828551	0.912041
SCE1572_10935	0.247950672	-16.0269	0.828551	0.912041
SCE1572_1657	0.247950672	-16.0269	0.828551	0.912041
SCE1572_6168	-0.231895123	-16.0038	0.828551	0.912041
SCE1572_3413	-0.231895123	-16.0038	0.828551	0.912041
SCE1572_9371	-0.231895123	-16.0038	0.828551	0.912041
SCE1572_6084	-0.231895123	-16.0038	0.828551	0.912041

SCE1572_5803	-0.193420975	-15.9846	0.828551	0.912041
SCE1572_3125	-0.193420975	-15.9846	0.828551	0.912041
SCE1572_4059	-0.193420975	-15.9846	0.828551	0.912041
SCE1572_4332	-0.193420975	-15.9846	0.828551	0.912041
SCE1572_771	-0.16538185	-14.2578	0.828623	0.912041
SCE1572_383	-0.16538185	-14.2578	0.828623	0.912041
SCE1572_4307	0.180272483	-14.8802	0.828783	0.912121
SCE1572_7129	-0.166453927	-14.4131	0.830044	0.913271
SCE1572_9012	0.199882232	-15.4984	0.830176	0.913271
SCE1572_10982	0.199882232	-15.4984	0.830176	0.913271
SCE1572_9524	-0.187359222	-15.5076	0.830176	0.913271
SCE1572_7602	0.180565148	-14.2396	0.830445	0.913472
SCE1572_9479	-0.156964762	-13.3681	0.830773	0.913611
SCE1572_5521	-0.152124559	-13.2759	0.830929	0.913611
SCE1572_4755	0.20662381	-15.1471	0.831006	0.913611
SCE1572_2747	-0.183960646	-15.1498	0.831006	0.913611
SCE1572_9794	-0.16289903	-15.1392	0.831006	0.913611
SCE1572_7363	0.142078413	-12.1102	0.833527	0.916142
SCE1572_11112	0.220429609	-15.9476	0.833964	0.916142
SCE1572_11170	0.177037014	-14.3843	0.834026	0.916142
SCE1572_10921	-0.15594627	-14.3809	0.834026	0.916142
SCE1572_330	0.156438053	-14.5872	0.83408	0.916142
SCE1572_7968	-0.167086827	-14.8194	0.834135	0.916142
SCE1572_5297	-0.216975703	-15.4658	0.834171	0.916142
SCE1572_9203	-0.216975703	-15.4658	0.834171	0.916142
SCE1572_1618	-0.186534589	-15.1067	0.834179	0.916142
SCE1572_2998	0.174376266	-15.1126	0.834179	0.916142
SCE1572_1703	-0.144188299	-12.9903	0.835982	0.918027
SCE1572_4652	-0.169885461	-14.5578	0.836273	0.91825
SCE1572_738	0.18229448	-15.4431	0.837983	0.919745
SCE1572_3149	0.18229448	-15.4431	0.837983	0.919745
SCE1572_11198	0.18229448	-15.4431	0.837983	0.919745
SCE1572_884	-0.145892605	-13.5014	0.838131	0.919745
SCE1572_311	0.160292753	-14.5514	0.838408	0.919745
SCE1572_2453	0.160687526	-14.0002	0.838473	0.919745
SCE1572_719	-0.183530134	-14.7783	0.839163	0.919745
SCE1572_5881	0.16153592	-14.3332	0.839662	0.919745
SCE1572_7532	0.65302548	-17.9531	0.840166	0.919745
SCE1572_6850	0.429016231	-17.8433	0.840166	0.919745
SCE1572_731	0.429016231	-17.8433	0.840166	0.919745
SCE1572_10354	0.429016231	-17.8433	0.840166	0.919745
SCE1572_1803	0.429016231	-17.8433	0.840166	0.919745
SCE1572_7436	0.429016231	-17.8433	0.840166	0.919745
SCE1572_3087	0.429016231	-17.8433	0.840166	0.919745
SCE1572_10960	0.429016231	-17.8433	0.840166	0.919745
SCE1572_2437	-0.42342658	-17.9476	0.840166	0.919745
SCE1572_4991	0.313539014	-17.901	0.840166	0.919745
SCE1572_1245	0.313539014	-17.901	0.840166	0.919745

SCE1572_4417	0.313539014	-17.901	0.840166	0.919745
SCE1572_259	0.313539014	-17.901	0.840166	0.919745
SCE1572_11564	0.313539014	-17.901	0.840166	0.919745
SCE1572_7903	-0.271423487	-17.8716	0.840166	0.919745
SCE1572_9137	-0.271423487	-17.8716	0.840166	0.919745
SCE1572_1112	-0.271423487	-17.8716	0.840166	0.919745
SCE1572_2600	-0.271423487	-17.8716	0.840166	0.919745
SCE1572_10604	0.196725349	-15.0525	0.840181	0.919745
SCE1572_3460	-0.158212877	-14.5222	0.840489	0.919745
SCE1572_6695	0.264629413	-16.6035	0.84052	0.919745
SCE1572_449	0.264629413	-16.6035	0.84052	0.919745
SCE1572_1212	0.264629413	-16.6035	0.84052	0.919745
SCE1572_1032	0.261522875	-16.5681	0.84052	0.919745
SCE1572_10780	-0.212529798	-16.5791	0.84052	0.919745
SCE1572_2647	0.150040281	-13.5904	0.840716	0.919864
SCE1572_8220	0.154841268	-13.9804	0.841366	0.920474
SCE1572_1435	-0.193420975	-15.3996	0.841624	0.920474
SCE1572_753	-0.168329994	-15.3871	0.841624	0.920474
SCE1572_9112	-0.168329994	-15.3871	0.841624	0.920474
SCE1572_10380	0.163962658	-14.5164	0.842517	0.921356
SCE1572_2114	0.137970931	-12.3815	0.842738	0.921501
SCE1572_109	-0.171887814	-15.0144	0.843022	0.921716
SCE1572_5961	-0.201034159	-15.8364	0.843823	0.922496
SCE1572_4029	-0.140839377	-13.5583	0.843954	0.922543
SCE1572_1741	-0.137445182	-12.9073	0.844609	0.923163
SCE1572_3078	-0.138398068	-13.6675	0.844771	0.923245
SCE1572_6344	0.147077868	-14.2838	0.844929	0.923321
SCE1572_4181	-0.19613536	-15.3485	0.845104	0.923416
SCE1572_2076	-0.14366794	-13.7898	0.845726	0.923999
SCE1572_4191	-0.14102685	-13.9188	0.846854	0.925117
SCE1572_10773	-0.271423487	-16.493	0.84754	0.925117
SCE1572_8911	0.223341205	-16.4867	0.84754	0.925117
SCE1572_1637	0.223341205	-16.4867	0.84754	0.925117
SCE1572_2433	-0.216975703	-16.4658	0.84754	0.925117
SCE1572_5904	-0.216975703	-16.4658	0.84754	0.925117
SCE1572_5874	-0.216975703	-16.4658	0.84754	0.925117
SCE1572_1954	-0.216975703	-16.4658	0.84754	0.925117
SCE1572_6230	-0.216975703	-16.4658	0.84754	0.925117
SCE1572_7569	0.159210867	-14.4546	0.848305	0.925493
SCE1572_8294	0.202507701	-15.7866	0.848324	0.925493
SCE1572_3142	0.173361356	-15.8012	0.848324	0.925493
SCE1572_9990	0.173361356	-15.8012	0.848324	0.925493
SCE1572_200	0.173361356	-15.8012	0.848324	0.925493
SCE1572_10777	-0.132744173	-12.3232	0.848755	0.925837
SCE1572_8412	-0.136071634	-13.1951	0.848816	0.925837
SCE1572_9729	-0.15594627	-14.2289	0.849863	0.926883
SCE1572_6798	-0.138973191	-14.4268	0.850141	0.92709
SCE1572_9110	0.159915825	-14.9334	0.850972	0.927803

SCE1572_7885	-0.158948758	-14.9278	0.850972	0.927803
SCE1572_1595	-0.152124559	-15.2759	0.851626	0.928324
SCE1572_7468	-0.152124559	-15.2759	0.851626	0.928324
SCE1572_7888	-0.148566739	-14.6402	0.852577	0.929264
SCE1572_2478	-0.136071634	-14.1951	0.852982	0.929562
SCE1572_5350	0.125058193	-11.7052	0.853079	0.929562
SCE1572_6120	0.146187956	-13.8554	0.853204	0.929562
SCE1572_10818	-0.128819092	-13.8554	0.853204	0.929562
SCE1572_405	-0.136493907	-13.4536	0.853859	0.93018
SCE1572_10416	-0.396954369	-17.6713	0.854437	0.930424
SCE1572_4921	-0.396954369	-17.6713	0.854437	0.930424
SCE1572_5983	-0.396954369	-17.6713	0.854437	0.930424
SCE1572_1938	-0.396954369	-17.6713	0.854437	0.930424
SCE1572_4269	0.198061796	-15.2583	0.854685	0.930501
SCE1572_1861	0.177883914	-15.2684	0.854685	0.930501
SCE1572_2725	-0.131493226	-14.3686	0.855393	0.931177
SCE1572_11418	0.132154897	-13.6879	0.855993	0.931637
SCE1572_9002	-0.129404482	-13.6795	0.855993	0.931637
SCE1572_10876	0.146429028	-13.9846	0.856418	0.931707
SCE1572_801	0.208569454	-15.7056	0.856588	0.931707
SCE1572_5522	0.181088718	-15.7193	0.856588	0.931707
SCE1572_6570	0.181088718	-15.7193	0.856588	0.931707
SCE1572_2614	-0.176266254	-15.6865	0.856588	0.931707
SCE1572_5563	-0.124025968	-12.1482	0.856589	0.931707
SCE1572_7089	0.16351357	-15.2211	0.85762	0.932539
SCE1572_6285	-0.15594627	-15.2289	0.85762	0.932539
SCE1572_1350	-0.15594627	-15.2289	0.85762	0.932539
SCE1572_11049	0.150809514	-14.3385	0.858696	0.933612
SCE1572_2197	0.122189576	-12.2688	0.859504	0.934387
SCE1572_4124	0.231076853	-16.3573	0.859853	0.934387
SCE1572_8680	0.231076853	-16.3573	0.859853	0.934387
SCE1572_1449	-0.171887814	-16.3364	0.859853	0.934387
SCE1572_9870	-0.171887814	-16.3364	0.859853	0.934387
SCE1572_9348	0.133832991	-13.7814	0.860164	0.934572
SCE1572_8408	0.143614012	-13.9416	0.860201	0.934572
SCE1572_3651	-0.130560951	-14.8377	0.8604	0.934692
SCE1572_11371	-0.123240265	-12.419	0.860802	0.935033
SCE1572_779	-0.129129605	-13.7648	0.861261	0.935434
SCE1572_7469	-0.138529217	-14.2942	0.861851	0.935978
SCE1572_8339	0.158564354	-14.8086	0.862574	0.936667
SCE1572_4115	-0.145892605	-14.0863	0.862951	0.936883
SCE1572_1817	0.134568873	-14.0836	0.862951	0.936883
SCE1572_3314	0.136661252	-14.289	0.863376	0.937181
SCE1572_8773	-0.122900962	-13.7442	0.863404	0.937181
SCE1572_10031	0.123877794	-13.4723	0.863697	0.937402
SCE1572_11227	0.151787944	-14.5225	0.86393	0.937435
SCE1572_8676	-0.144666345	-14.5154	0.86393	0.937435
SCE1572_6499	-0.151129253	-15.6098	0.863995	0.937435

SCE1572_9941	-0.224117772	-16.2629	0.865284	0.937963
SCE1572_667	-0.224117772	-16.2629	0.865284	0.937963
SCE1572_7515	-0.224117772	-16.2629	0.865284	0.937963
SCE1572_6577	0.198061796	-16.2583	0.865284	0.937963
SCE1572_4515	0.198061796	-16.2583	0.865284	0.937963
SCE1572_5545	0.198061796	-16.2583	0.865284	0.937963
SCE1572_3107	0.198061796	-16.2583	0.865284	0.937963
SCE1572_2417	0.157419812	-16.2786	0.865284	0.937963
SCE1572_3070	0.157419812	-16.2786	0.865284	0.937963
SCE1572_2868	0.137382059	-15.1311	0.865756	0.938129
SCE1572_1413	0.406648418	-17.5325	0.866239	0.938129
SCE1572_389	0.406648418	-17.5325	0.866239	0.938129
SCE1572_8768	0.406648418	-17.5325	0.866239	0.938129
SCE1572_4133	0.406648418	-17.5325	0.866239	0.938129
SCE1572_11120	0.313539014	-17.5791	0.866239	0.938129
SCE1572_5661	0.313539014	-17.5791	0.866239	0.938129
SCE1572_6777	0.313539014	-17.5791	0.866239	0.938129
SCE1572_985	0.140207411	-14.2598	0.866329	0.938129
SCE1572_6152	0.140207411	-14.2598	0.866329	0.938129
SCE1572_4366	0.162979337	-14.7668	0.866725	0.938365
SCE1572_8276	-0.121180851	-14.7608	0.866725	0.938365
SCE1572_11230	-0.118033853	-12.8314	0.867006	0.938573
SCE1572_6625	-0.183960646	-15.5648	0.867417	0.938824
SCE1572_11047	0.169149104	-15.5809	0.867417	0.938824
SCE1572_9260	-0.131698723	-14.241	0.867758	0.939
SCE1572_11071	-0.120643535	-14.2355	0.867758	0.939
SCE1572_3906	-0.115628814	-13.2827	0.8696	0.940896
SCE1572_1371	0.12850712	-13.8236	0.870444	0.94168
SCE1572_3700	0.139771946	-14.4333	0.870578	0.94168
SCE1572_7806	0.143614012	-15.0791	0.870682	0.94168
SCE1572_7246	0.143614012	-15.0791	0.870682	0.94168
SCE1572_7200	-0.116700892	-13.3882	0.871144	0.942083
SCE1572_710	0.131932207	-15.0376	0.873012	0.944005
SCE1572_6773	0.118347254	-13.2437	0.873196	0.944107
SCE1572_10657	0.143614012	-14.401	0.873664	0.944516
SCE1572_882	-0.115147547	-13.2327	0.873892	0.944665
SCE1572_6600	0.115088314	-13.6252	0.874102	0.944796
SCE1572_11427	0.134568873	-14.6686	0.874319	0.944814
SCE1572_8622	0.120893936	-14.6754	0.874319	0.944814
SCE1572_3500	0.120263525	-13.2295	0.87458	0.944814
SCE1572_6158	0.20662381	-16.1471	0.87496	0.944814
SCE1572_4877	0.20662381	-16.1471	0.87496	0.944814
SCE1572_9208	-0.14214047	-16.1288	0.87496	0.944814
SCE1572_4345	-0.14214047	-16.1288	0.87496	0.944814
SCE1572_10743	-0.14214047	-16.1288	0.87496	0.944814
SCE1572_10990	-0.14214047	-16.1288	0.87496	0.944814
SCE1572_6454	-0.119420394	-13.9476	0.87506	0.944814
SCE1572_5729	-0.152778991	-15.0049	0.87526	0.944814

SCE1572_10123	0.112123726	-13.2204	0.875261	0.944814
SCE1572_5757	-0.121180851	-13.7608	0.875513	0.944814
SCE1572_1242	-0.117743816	-13.1054	0.875532	0.944814
SCE1572_11058	-0.110268695	-13.3316	0.875675	0.944814
SCE1572_10478	-0.119420394	-14.1402	0.875731	0.944814
SCE1572_9797	-0.271423487	-17.3861	0.876186	0.944814
SCE1572_2060	-0.271423487	-17.3861	0.876186	0.944814
SCE1572_2444	-0.271423487	-17.3861	0.876186	0.944814
SCE1572_3088	0.231076853	-17.3573	0.876186	0.944814
SCE1572_400	0.231076853	-17.3573	0.876186	0.944814
SCE1572_3592	0.231076853	-17.3573	0.876186	0.944814
SCE1572_8055	0.231076853	-17.3573	0.876186	0.944814
SCE1572_11097	0.137967449	-14.9888	0.877429	0.946058
SCE1572_6760	-0.104217987	-12.2272	0.877734	0.94629
SCE1572_10398	-0.133919963	-14.3434	0.878028	0.946413
SCE1572_8895	-0.133919963	-14.3434	0.878028	0.946413
SCE1572_2332	0.11346164	-13.5748	0.878432	0.946751
SCE1572_3938	-0.104686763	-12.8717	0.87863	0.946868
SCE1572_6871	0.179237922	-16.0613	0.87929	0.947168
SCE1572_7837	0.179237922	-16.0613	0.87929	0.947168
SCE1572_7628	0.143614012	-16.0791	0.87929	0.947168
SCE1572_4610	0.143614012	-16.0791	0.87929	0.947168
SCE1572_3076	0.117618804	-14.6067	0.879471	0.947168
SCE1572_2093	-0.119420394	-14.9476	0.879524	0.947168
SCE1572_4564	0.120893936	-15.4124	0.879539	0.947168
SCE1572_182	-0.107036669	-12.9314	0.881326	0.948995
SCE1572_7285	0.108220106	-13.389	0.881446	0.949027
SCE1572_10140	-0.14366794	-15.3747	0.88223	0.949774
SCE1572_221	-0.1035818	-13.8428	0.883312	0.950113
SCE1572_404	-0.104313501	-14.277	0.883398	0.950113
SCE1572_6662	0.116646965	-13.6596	0.883706	0.950113
SCE1572_5775	0.116646965	-13.6596	0.883706	0.950113
SCE1572_10909	-0.118177227	-14.2589	0.884668	0.950113
SCE1572_937	0.391541526	-17.277	0.884696	0.950113
SCE1572_6940	-0.364532891	-17.24	0.884696	0.950113
SCE1572_5301	-0.364532891	-17.24	0.884696	0.950113
SCE1572_6137	-0.364532891	-17.24	0.884696	0.950113
SCE1572_4042	0.313539014	-17.316	0.884696	0.950113
SCE1572_4418	0.313539014	-17.316	0.884696	0.950113
SCE1572_9191	0.313539014	-17.316	0.884696	0.950113
SCE1572_9059	0.313539014	-17.316	0.884696	0.950113
SCE1572_3298	0.313539014	-17.316	0.884696	0.950113
SCE1572_3975	-0.271423487	-17.1935	0.884696	0.950113
SCE1572_4230	-0.271423487	-17.1935	0.884696	0.950113
SCE1572_1115	-0.271423487	-17.1935	0.884696	0.950113
SCE1572_1967	-0.271423487	-17.1935	0.884696	0.950113
SCE1572_4450	-0.271423487	-17.1935	0.884696	0.950113
SCE1572_9902	-0.171887814	-15.3364	0.884803	0.950113

SCE1572_11442	-0.148041072	-15.3244	0.884803	0.950113
SCE1572_8672	-0.148041072	-15.3244	0.884803	0.950113
SCE1572_5116	-0.148041072	-15.3244	0.884803	0.950113
SCE1572_5526	0.129114443	-15.3494	0.884803	0.950113
SCE1572_11216	0.129114443	-15.3494	0.884803	0.950113
SCE1572_4006	0.108848594	-14.0261	0.88498	0.950206
SCE1572_7528	0.127125889	-14.5348	0.885717	0.9509
SCE1572_9174	-0.113161403	-14.0058	0.88604	0.951151
SCE1572_830	-0.15594627	-15.9658	0.8871	0.95179
SCE1572_9513	0.154841268	-15.9804	0.8871	0.95179
SCE1572_9315	0.154841268	-15.9804	0.8871	0.95179
SCE1572_2822	0.154841268	-15.9804	0.8871	0.95179
SCE1572_2064	0.154841268	-15.9804	0.8871	0.95179
SCE1572_2812	-0.117949006	-14.502	0.887179	0.95179
SCE1572_10172	-0.11388221	-14.8293	0.88901	0.953657
SCE1572_4031	-0.091541361	-10.5761	0.890569	0.955206
SCE1572_478	-0.161798996	-15.8907	0.890635	0.955206
SCE1572_6928	-0.110958815	-14.4408	0.891349	0.955873
SCE1572_8341	0.243149686	-17.1288	0.89207	0.956355
SCE1572_1874	0.243149686	-17.1288	0.89207	0.956355
SCE1572_9215	0.243149686	-17.1288	0.89207	0.956355
SCE1572_4809	0.108719222	-13.916	0.89295	0.9572
SCE1572_2594	-0.109152058	-14.1351	0.893912	0.957316
SCE1572_4773	0.195702523	-15.8725	0.893954	0.957316
SCE1572_987	0.195702523	-15.8725	0.893954	0.957316
SCE1572_4458	0.164675628	-15.888	0.893954	0.957316
SCE1572_4889	0.164675628	-15.888	0.893954	0.957316
SCE1572_7870	-0.11388221	-14.4143	0.893961	0.957316
SCE1572_2459	0.124505189	-14.7476	0.894017	0.957316
SCE1572_5503	0.124505189	-14.7476	0.894017	0.957316
SCE1572_6060	-0.105413536	-14.7529	0.894017	0.957316
SCE1572_7158	-0.112224892	-15.207	0.894059	0.957316
SCE1572_2847	-0.112224892	-15.207	0.894059	0.957316
SCE1572_5704	-0.107924755	-13.8829	0.894774	0.957983
SCE1572_2552	-0.116700892	-14.3882	0.895221	0.958365
SCE1572_5238	-0.097093306	-13.3381	0.895664	0.958704
SCE1572_434	0.107088136	-14.0973	0.895996	0.958704
SCE1572_5882	0.150040281	-15.1754	0.896145	0.958704
SCE1572_4113	0.150040281	-15.1754	0.896145	0.958704
SCE1572_8689	0.150040281	-15.1754	0.896145	0.958704
SCE1572_2547	-0.117095341	-15.1621	0.896145	0.958704
SCE1572_11582	-0.099657139	-13.6647	0.896176	0.958704
SCE1572_2992	0.143614012	-15.816	0.897076	0.959315
SCE1572_2082	-0.133919963	-15.8028	0.897076	0.959315
SCE1572_83	-0.11443901	-15.8178	0.897076	0.959315
SCE1572_6668	0.115599636	-14.7146	0.897112	0.959315
SCE1572_5928	-0.116145262	-14.0715	0.898	0.960167
SCE1572_3571	0.118523031	-15.1405	0.89815	0.96023

SCE1572_5820	0.093092083	-12.8037	0.898511	0.960518
SCE1572_11393	-0.091602449	-13.2834	0.899808	0.961807
SCE1572_3396	0.091981244	-13.4268	0.900458	0.962338
SCE1572_10261	0.092630084	-12.6716	0.900488	0.962338
SCE1572_5343	0.104952392	-14.3049	0.901097	0.962794
SCE1572_2687	-0.101498486	-14.3012	0.901097	0.962794
SCE1572_5993	0.112491495	-14.644	0.901428	0.96305
SCE1572_6531	0.125466666	-15.0882	0.901936	0.963494
SCE1572_1500	0.153074341	-15.7333	0.902795	0.96402
SCE1572_4329	-0.140178954	-15.7355	0.902795	0.96402
SCE1572_6347	-0.140178954	-15.7355	0.902795	0.96402
SCE1572_2524	-0.109152058	-15.72	0.902795	0.96402
SCE1572_11066	0.114230205	-15.0465	0.903725	0.964916
SCE1572_6886	0.252138469	-16.9317	0.904233	0.965164
SCE1572_3697	0.252138469	-16.9317	0.904233	0.965164
SCE1572_3295	-0.201034159	-16.8364	0.904233	0.965164
SCE1572_4788	-0.087823549	-13.9724	0.904445	0.965292
SCE1572_2626	-0.145892605	-15.6713	0.905421	0.965844
SCE1572_5958	0.134568873	-15.6686	0.905421	0.965844
SCE1572_10437	0.134568873	-15.6686	0.905421	0.965844
SCE1572_781	-0.116145262	-15.6564	0.905421	0.965844
SCE1572_9571	-0.116145262	-15.6564	0.905421	0.965844
SCE1572_3448	0.088752077	-13.9473	0.90614	0.966512
SCE1572_11511	0.089323857	-14.5537	0.906651	0.966959
SCE1572_872	0.114634945	-14.541	0.907871	0.968163
SCE1572_10713	0.202507701	-16.7866	0.909315	0.969015
SCE1572_7777	0.202507701	-16.7866	0.909315	0.969015
SCE1572_8746	-0.133919963	-16.8028	0.909315	0.969015
SCE1572_284	-0.133919963	-16.8028	0.909315	0.969015
SCE1572_1781	-0.133919963	-16.8028	0.909315	0.969015
SCE1572_6160	-0.133919963	-16.8028	0.909315	0.969015
SCE1572_4128	-0.133919963	-16.8028	0.909315	0.969015
SCE1572_8865	-0.085010363	-13.6573	0.909432	0.969041
SCE1572_5498	0.091146592	-13.8829	0.910125	0.969146
SCE1572_9584	-0.091717465	-14.4889	0.910218	0.969146
SCE1572_373	0.143614012	-15.5937	0.910267	0.969146
SCE1572_1172	0.143614012	-15.5937	0.910267	0.969146
SCE1572_1920	-0.122560101	-15.5955	0.910267	0.969146
SCE1572_4373	-0.122560101	-15.5955	0.910267	0.969146
SCE1572_5831	-0.122560101	-15.5955	0.910267	0.969146
SCE1572_87	-0.094545725	-15.5815	0.910267	0.969146
SCE1572_9282	-0.074794162	-10.4676	0.910622	0.969333
SCE1572_491	0.076613934	-11.4525	0.910628	0.969333
SCE1572_5845	0.111905152	-14.4783	0.911348	0.969903
SCE1572_6458	0.09983232	-14.4843	0.911348	0.969903
SCE1572_4034	0.079333261	-13.2499	0.911544	0.970014
SCE1572_9222	0.10054529	-14.92	0.911773	0.970159
SCE1572_5984	0.092850899	-14.4568	0.912449	0.970549

SCE1572_11099	0.102034908	-15.5473	0.912508	0.970549
SCE1572_1609	0.102034908	-15.5473	0.912508	0.970549
SCE1572_6263	0.102034908	-15.5473	0.912508	0.970549
SCE1572_10580	-0.145892605	-16.6713	0.913871	0.971606
SCE1572_9377	-0.145892605	-16.6713	0.913871	0.971606
SCE1572_1832	-0.145892605	-16.6713	0.913871	0.971606
SCE1572_1579	-0.145892605	-16.6713	0.913871	0.971606
SCE1572_8551	-0.094118955	-14.0825	0.914522	0.972016
SCE1572_58	0.109398297	-14.4181	0.914571	0.972016
SCE1572_6149	0.122855452	-14.8671	0.914627	0.972016
SCE1572_8749	-0.109960064	-14.8648	0.914627	0.972016
SCE1572_3928	-0.080282	-13.191	0.914863	0.972133
SCE1572_3193	-0.076776056	-12.0193	0.914921	0.972133
SCE1572_7722	0.078160951	-14.0645	0.915343	0.972482
SCE1572_8195	0.091146592	-14.3975	0.915594	0.972482
SCE1572_9533	-0.089220156	-14.4019	0.915594	0.972482
SCE1572_1268	0.089323857	-13.5537	0.91562	0.972482
SCE1572_9624	0.09221486	-13.7829	0.915843	0.97262
SCE1572_2510	-0.077412867	-14.0521	0.916148	0.97284
SCE1572_3946	-0.075026274	-11.9254	0.916234	0.97284
SCE1572_6583	0.135751894	-15.4663	0.916668	0.972907
SCE1572_2085	0.135751894	-15.4663	0.916668	0.972907
SCE1572_1702	-0.107924755	-15.4679	0.916668	0.972907
SCE1572_10374	-0.107924755	-15.4679	0.916668	0.972907
SCE1572_5738	-0.07960588	-13.3294	0.916943	0.973102
SCE1572_5017	-0.097394087	-14.8211	0.917302	0.973384
SCE1572_7725	0.078518984	-14.3461	0.918522	0.97437
SCE1572_7772	0.104085648	-14.7963	0.918577	0.97437
SCE1572_2882	-0.138973191	-15.4268	0.918602	0.97437
SCE1572_7620	0.097810323	-15.4239	0.918602	0.97437
SCE1572_7464	0.075227037	-12.6803	0.919869	0.975616
SCE1572_6419	-0.089816681	-14.7451	0.921013	0.976682
SCE1572_8347	-0.078778409	-14.2898	0.921253	0.976682
SCE1572_6805	0.077471655	-14.2911	0.921253	0.976682
SCE1572_7093	0.17603549	-16.5103	0.92171	0.976682
SCE1572_7699	0.17603549	-16.5103	0.92171	0.976682
SCE1572_9262	-0.15594627	-16.5508	0.92171	0.976682
SCE1572_7847	0.127125889	-16.5348	0.92171	0.976682
SCE1572_4266	-0.101498486	-16.5236	0.92171	0.976682
SCE1572_3983	-0.101498486	-16.5236	0.92171	0.976682
SCE1572_11383	-0.119420394	-15.3626	0.922212	0.97682
SCE1572_5307	-0.119420394	-15.3626	0.922212	0.97682
SCE1572_1659	0.107088136	-15.3604	0.922212	0.97682
SCE1572_6319	0.107088136	-15.3604	0.922212	0.97682
SCE1572_2518	0.077030902	-13.4193	0.922988	0.977544
SCE1572_11069	0.071263168	-13.9136	0.923432	0.977915
SCE1572_5510	-0.101498486	-15.3012	0.923899	0.978213
SCE1572_6833	-0.101498486	-15.3012	0.923899	0.978213

SCE1572_4260	0.068426516	-13.0236	0.92421	0.978444
SCE1572_7001	0.07340501	-13.3772	0.924863	0.979037
SCE1572_928	0.081092928	-14.2099	0.925419	0.979527
SCE1572_1144	0.115599636	-15.2995	0.925514	0.979529
SCE1572_6867	0.064560203	-11.2807	0.92572	0.979648
SCE1572_6075	0.077098818	-13.593	0.925867	0.979705
SCE1572_5907	0.063312384	-11.7315	0.926677	0.980463
SCE1572_1524	-0.085010363	-15.2423	0.927062	0.980772
SCE1572_7881	0.070267863	-14.1647	0.927714	0.981363
SCE1572_2380	0.143614012	-16.401	0.928218	0.981797
SCE1572_8	-0.073009929	-13.8089	0.928407	0.981899
SCE1572_4706	0.096727625	-14.5831	0.9285	0.981899
SCE1572_10800	-0.068794321	-14.1149	0.929872	0.98296
SCE1572_147	0.11104615	-15.1949	0.929971	0.98296
SCE1572_7627	0.11104615	-15.1949	0.929971	0.98296
SCE1572_2067	-0.090851241	-15.1963	0.929971	0.98296
SCE1572_3502	-0.069789626	-15.1858	0.929971	0.98296
SCE1572_339	-0.124582099	-16.3127	0.931075	0.983814
SCE1572_10277	0.115599636	-16.2995	0.931075	0.983814
SCE1572_8209	-0.063024338	-12.8798	0.931117	0.983814
SCE1572_9865	0.082213468	-14.5248	0.931291	0.983814
SCE1572_8978	0.099414208	-15.1501	0.931341	0.983814
SCE1572_8461	-0.09633678	-15.1517	0.931341	0.983814
SCE1572_3385	-0.061970121	-14.5038	0.932173	0.984595
SCE1572_1590	0.087657606	-14.4904	0.933033	0.985279
SCE1572_6502	0.087657606	-14.4904	0.933033	0.985279
SCE1572_8561	0.073224684	-14.0439	0.933195	0.985279
SCE1572_7490	-0.058429764	-14.0426	0.933195	0.985279
SCE1572_7760	0.06530161	-13.4252	0.933758	0.985675
SCE1572_2731	0.06530161	-13.4252	0.933758	0.985675
SCE1572_7924	0.078160951	-15.0645	0.935144	0.987039
SCE1572_11550	0.169149104	-16.1658	0.93615	0.987803
SCE1572_7460	0.130674957	-16.1851	0.93615	0.987803
SCE1572_7230	0.130674957	-16.1851	0.93615	0.987803
SCE1572_3925	-0.058429764	-13.6276	0.936642	0.988223
SCE1572_11421	-0.06497261	-14.3898	0.937026	0.988529
SCE1572_2113	0.103615944	-15.006	0.937453	0.988583
SCE1572_3981	0.103615944	-15.006	0.937453	0.988583
SCE1572_10298	-0.073484109	-15.0071	0.937453	0.988583
SCE1572_4534	-0.073484109	-15.0071	0.937453	0.988583
SCE1572_2985	0.107088136	-16.0973	0.938413	0.989241
SCE1572_3869	-0.101498486	-16.1085	0.938413	0.989241
SCE1572_9773	-0.096925756	-14.977	0.938547	0.989241
SCE1572_326	-0.096925756	-14.977	0.938547	0.989241
SCE1572_4812	0.076499816	-14.9751	0.938547	0.989241
SCE1572_798	-0.060275823	-13.5644	0.939262	0.989895
SCE1572_8381	0.058571889	-13.287	0.939446	0.989991
SCE1572_11447	0.058388207	-12.8388	0.940437	0.990833

SCE1572_5418	-0.149432963	-16.045	0.94052	0.990833
SCE1572_5852	-0.083796484	-14.9298	0.940623	0.990833
SCE1572_11158	0.067692228	-14.9276	0.940623	0.990833
SCE1572_8567	0.053671887	-13.5231	0.940891	0.991017
SCE1572_9588	-0.055000024	-12.6506	0.941089	0.991126
SCE1572_3916	-0.068139889	-13.8439	0.941511	0.991246
SCE1572_5136	0.075026927	-14.891	0.941609	0.991246
SCE1572_5199	0.058725115	-14.8991	0.941609	0.991246
SCE1572_4219	0.058725115	-14.8991	0.941609	0.991246
SCE1572_9364	0.056763598	-13.5058	0.941674	0.991246
SCE1572_990	0.066017607	-13.8153	0.942466	0.991702
SCE1572_11115	-0.062476597	-13.8222	0.942466	0.991702
SCE1572_8253	0.120893936	-15.9973	0.942485	0.991702
SCE1572_6843	0.120893936	-15.9973	0.942485	0.991702
SCE1572_9859	0.076499816	-14.2382	0.943128	0.99218
SCE1572_343	-0.074122047	-14.2369	0.943128	0.99218
SCE1572_751	-0.076407505	-14.8481	0.943487	0.992359
SCE1572_5450	0.066446152	-14.8546	0.943487	0.992359
SCE1572_4455	-0.049031066	-11.5666	0.943857	0.99265
SCE1572_10681	-0.119420394	-15.9476	0.944323	0.992842
SCE1572_1237	-0.083796484	-15.9298	0.944323	0.992842
SCE1572_7706	-0.083796484	-15.9298	0.944323	0.992842
SCE1572_2571	-0.069789626	-14.1858	0.944907	0.993257
SCE1572_2023	-0.05937301	-14.1806	0.944907	0.993257
SCE1572_1538	0.132966768	-15.9038	0.946047	0.993801
SCE1572_8505	0.132966768	-15.9038	0.946047	0.993801
SCE1572_1835	0.132966768	-15.9038	0.946047	0.993801
SCE1572_581	-0.069789626	-14.7707	0.946086	0.993801
SCE1572_3299	-0.069789626	-14.7707	0.946086	0.993801
SCE1572_7601	0.0656115	-14.777	0.946086	0.993801
SCE1572_2942	0.0656115	-14.777	0.946086	0.993801
SCE1572_5554	-0.058694163	-13.3457	0.947573	0.994989
SCE1572_6030	0.113240363	-15.8312	0.947666	0.994989
SCE1572_3301	0.113240363	-15.8312	0.947666	0.994989
SCE1572_519	-0.059919382	-15.8398	0.947666	0.994989
SCE1572_3854	-0.059119883	-14.7298	0.94769	0.994989
SCE1572_5751	-0.049031066	-12.6393	0.947916	0.995127
SCE1572_4285	0.064859901	-14.7034	0.948457	0.995496
SCE1572_5825	0.064859901	-14.7034	0.948457	0.995496
SCE1572_6024	0.050504608	-13.0102	0.949795	0.996671
SCE1572_2294	0.071464226	-14.6645	0.949924	0.996671
SCE1572_3354	-0.068331622	-14.6657	0.949924	0.996671
SCE1572_10752	0.055838052	-13.275	0.949955	0.996671
SCE1572_4694	0.095358844	-15.7622	0.950626	0.997176
SCE1572_10945	0.095358844	-15.7622	0.950626	0.997176
SCE1572_5872	0.046191615	-12.3836	0.950776	0.997233
SCE1572_6892	-0.049680873	-13.5591	0.951561	0.997803
SCE1572_10481	-0.058019849	-13.9783	0.951925	0.997803

SCE1572_2562	0.05719926	-14.6029	0.951975	0.997803
SCE1572_1340	-0.078778409	-15.7048	0.951983	0.997803
SCE1572_4160	-0.078778409	-15.7048	0.951983	0.997803
SCE1572_11108	-0.060559681	-15.6957	0.951983	0.997803
SCE1572_9068	-0.049031066	-15.69	0.951983	0.997803
SCE1572_5108	0.056902379	-13.5375	0.952218	0.99795
SCE1572_9354	-0.080739925	-14.5746	0.952622	0.998175
SCE1572_2266	-0.080739925	-14.5746	0.952622	0.998175
SCE1572_7546	0.058966187	-13.9408	0.953203	0.998684
SCE1572_2236	0.04807787	-13.1389	0.954361	0.999798
SCE1572_6192	0.091146592	-15.6199	0.954483	0.999826
SCE1572_5932	-0.042799112	-13.4498	0.954966	1
SCE1572_1229	-0.054612098	-13.8756	0.955187	1
SCE1572_7768	0.043162632	-12.4141	0.955339	1
SCE1572_2250	0.042606694	-12.2421	0.955348	1
SCE1572_8760	-0.039250045	-13.1001	0.955513	1
SCE1572_10541	-0.065954369	-14.476	0.95561	1
SCE1572_9764	0.056630711	-14.4749	0.95561	1
SCE1572_9817	0.076499816	-15.5601	0.955638	1
SCE1572_10334	0.076499816	-15.5601	0.955638	1
SCE1572_2328	0.076499816	-15.5601	0.955638	1
SCE1572_11209	-0.101498486	-15.5236	0.956735	1
SCE1572_448	-0.075026274	-15.5103	0.956735	1
SCE1572_6563	-0.075026274	-15.5103	0.956735	1
SCE1572_6643	-0.038183683	-12.027	0.957198	1
SCE1572_2271	0.087657606	-15.4904	0.957778	1
SCE1572_4539	0.070083467	-15.4731	0.957778	1
SCE1572_10706	-0.052999968	-14.3838	0.958244	1
SCE1572_744	-0.082389663	-15.4551	0.958772	1
SCE1572_2065	0.07435135	-15.4356	0.958772	1
SCE1572_6235	-0.057298682	-15.4426	0.958772	1
SCE1572_6964	0.050504608	-14.3321	0.959679	1
SCE1572_4918	-0.049031066	-14.3274	0.959679	1
SCE1572_8742	-0.089220156	-15.4019	0.95972	1
SCE1572_152	-0.089220156	-15.4019	0.95972	1
SCE1572_1697	-0.089220156	-15.4019	0.95972	1
SCE1572_6098	-0.046550076	-13.706	0.959943	1
SCE1572_2053	0.084720323	-15.3716	0.960626	1
SCE1572_10527	0.062000247	-15.3829	0.960626	1
SCE1572_10191	0.062000247	-15.3829	0.960626	1
SCE1572_2919	0.072530914	-15.3211	0.961491	1
SCE1572_1522	0.072530914	-15.3211	0.961491	1
SCE1572_5311	-0.049031066	-15.3274	0.961491	1
SCE1572_8559	-0.049031066	-15.3274	0.961491	1
SCE1572_3611	-0.037368148	-13.617	0.962233	1
SCE1572_5715	0.094225985	-15.3102	0.962319	1
SCE1572_4237	0.094225985	-15.3102	0.962319	1
SCE1572_7356	-0.042119939	-13.184	0.962411	1

SCE1572_4172	0.035758022	-13.1573	0.963003	1
SCE1572_1750	0.082213468	-15.2618	0.963112	1
SCE1572_10914	0.061151852	-15.2723	0.963112	1
SCE1572_4387	-0.03438429	-15.2676	0.963112	1
SCE1572_301	0.050504608	-14.1745	0.963447	1
SCE1572_6660	0.060420077	-15.1696	0.965301	1
SCE1572_4863	-0.049031066	-15.1754	0.965301	1
SCE1572_6751	-0.030072908	-12.733	0.965319	1
SCE1572_10525	-0.075872678	-15.1415	0.965974	1
SCE1572_547	-0.075872678	-15.1415	0.965974	1
SCE1572_4725	-0.03876273	-13.4333	0.966538	1
SCE1572_3316	-0.039693201	-14.0332	0.966885	1
SCE1572_3960	0.059782421	-15.0737	0.967244	1
SCE1572_11197	-0.042604797	-15.0791	0.967244	1
SCE1572_10784	-0.032352325	-12.6477	0.967253	1
SCE1572_836	-0.067890093	-15.0473	0.967844	1
SCE1572_6524	-0.030019097	-12.9435	0.968037	1
SCE1572_6853	-0.037301773	-13.3622	0.968151	1
SCE1572_4247	-0.043154499	-13.9501	0.968654	1
SCE1572_5721	-0.046131175	-13.9311	0.96893	1
SCE1572_9849	-0.037466314	-13.9268	0.96893	1
SCE1572_11325	-0.033983263	-13.3066	0.969263	1
SCE1572_7974	0.050504608	-14.9451	0.970039	1
SCE1572_3090	0.042467289	-13.8666	0.970243	1
SCE1572_1600	-0.049202825	-14.9125	0.970542	1
SCE1572_7875	-0.049031066	-14.9124	0.970542	1
SCE1572_203	0.042236992	-14.9074	0.970542	1
SCE1572_8592	-0.029450697	-13.227	0.970952	1
SCE1572_14	-0.071485917	-14.8841	0.971028	1
SCE1572_1224	-0.054612098	-14.8756	0.971028	1
SCE1572_705	-0.054612098	-14.8756	0.971028	1
SCE1572_8474	0.058281958	-14.8192	0.972393	1
SCE1572_3209	0.050504608	-14.7846	0.97282	1
SCE1572_4803	0.050504608	-14.7846	0.97282	1
SCE1572_7151	-0.049031066	-14.7969	0.97282	1
SCE1572_10838	-0.049031066	-14.7969	0.97282	1
SCE1572_1177	-0.049031066	-14.7969	0.97282	1
SCE1572_1722	0.03206736	-13.0645	0.973958	1
SCE1572_10434	0.026257062	-12.2898	0.974391	1
SCE1572_11303	-0.030415388	-13.6136	0.974907	1
SCE1572_4692	0.036698808	-14.6471	0.975128	1
SCE1572_7738	-0.025667073	-12.9413	0.976079	1
SCE1572_5536	0.037329219	-14.5797	0.976141	1
SCE1572_6462	-0.029222585	-13.5332	0.97626	1
SCE1572_6430	-0.053518457	-14.561	0.97646	1
SCE1572_7065	0.031968656	-13.4872	0.976883	1
SCE1572_7111	0.050504608	-14.509	0.977369	1
SCE1572_6369	0.044352381	-14.481	0.977656	1

SCE1572_4197	0.031968656	-14.4872	0.977656	1
SCE1572_2805	-0.031618135	-14.4886	0.977656	1
SCE1572_949	-0.027025183	-12.7863	0.978423	1
SCE1572_9881	-0.032636627	-14.4302	0.978477	1
SCE1572_7400	0.03407906	-14.3128	0.979948	1
SCE1572_10618	0.03407906	-14.3128	0.979948	1
SCE1572_11196	0.023023871	-14.3183	0.979948	1
SCE1572_1871	0.026657866	-14.1137	0.982535	1
SCE1572_6233	0.032910548	-13.9969	0.98382	1
SCE1572_7470	0.020931492	-13.9388	0.984394	1
SCE1572_10928	-0.027497904	-13.8236	0.985547	1
SCE1572_11098	-0.014648072	-13.7983	0.98578	1
SCE1572_11436	0.024708607	-13.7416	0.986332	1
SCE1572_5994	-0.024411099	-13.6949	0.986744	1
SCE1572_5374	0.018872999	-13.6733	0.98694	1
SCE1572_10173	-0.020048672	-12.6084	0.987254	1
SCE1572_8332	-0.024330625	-13.5621	0.987842	1
SCE1572_6588	0.013415289	-12.429	0.988722	1
SCE1572_10948	-0.013111491	-13.3919	0.989186	1
SCE1572_1528	0.016442772	-13.1427	0.990868	1
SCE1572_8930	0.007477324	-12.3329	0.994724	1
SCE1572_7491	-0.078778409	-16.2898	1	1
SCE1572_5965	0.0656115	-15.777	1	1
SCE1572_10382	0.0656115	-15.777	1	1
SCE1572_837	-0.03876273	-15.7552	1	1
SCE1572_4723	-0.03876273	-15.7552	1	1
SCE1572_3775	-0.03438429	-16.2676	1	1
SCE1572_5580	-0.023495974	-15.0695	1	1
SCE1572_5596	0.013553472	-13.6081	1	1
SCE1572_10621	0.012369479	-13.9431	1	1
SCE1572_4684	0.009312921	-12.9763	1	1
SCE1572_10902	0.188008132	-16.9638	1	1
SCE1572_5474	0.188008132	-16.9638	1	1
SCE1572_1134	0.188008132	-16.9638	1	1
SCE1572_6761	0.120893936	-16.9973	1	1
SCE1572_11125	0.120893936	-16.9973	1	1
SCE1572_2535	-0.119420394	-16.9476	1	1
SCE1572_6937	-0.119420394	-16.9476	1	1
SCE1572_4468	-0.119420394	-16.9476	1	1
SCE1572_3657	-0.11388221	-16.4143	1	1
SCE1572_1407	0.097810323	-16.4239	1	1
SCE1572_3013	-0.069789626	-16.7707	1	1
SCE1572_412	-0.069789626	-16.7707	1	1
SCE1572_8734	-0.06497261	-16.3898	1	1
SCE1572_10411	-0.06497261	-16.3898	1	1
SCE1572_1672	-0.06497261	-16.3898	1	1
SCE1572_9818	-0.049031066	-16.9124	1	1
SCE1572_1250	-0.049031066	-16.9124	1	1

SCE1572_6267	-0.049031066	-16.9124	1	1
SCE1572_2615	-0.017666895	-14.7812	1	1
SCE1572_7593	-0.015922754	-13.7438	1	1
SCE1572_2993	0.012369479	-13.528	1	1
SCE1572_1565	0.010611881	-14.593	1	1
SCE1572_782	-0.008389081	-16.74	1	1
SCE1572_1186	-0.008389081	-16.74	1	1
SCE1572_4601	-0.008389081	-16.74	1	1
SCE1572_6935	-0.002236854	-14.7735	1	1
SCE1572_10006	-0.271423487	-17.6085	1	1
SCE1572_4996	-0.271423487	-17.6085	1	1
SCE1572_8027	-0.271423487	-17.6085	1	1
SCE1572_692	-0.271423487	-17.6085	1	1
SCE1572_140	0.21400334	-17.6288	1	1
SCE1572_6259	0.21400334	-17.6288	1	1
SCE1572_9726	0.21400334	-17.6288	1	1
SCE1572_7378	0.21400334	-17.6288	1	1
SCE1572_2705	-0.15594627	-17.5508	1	1
SCE1572_10735	-0.15594627	-17.5508	1	1
SCE1572_7704	-0.15594627	-17.5508	1	1
SCE1572_9947	-0.15594627	-17.5508	1	1
SCE1572_1644	0.067378426	-15.9366	1	1
SCE1572_5298	0.067378426	-15.9366	1	1
SCE1572_496	0.050504608	-15.0325	1	1
SCE1572_10131	-0.049031066	-15.9124	1	1
SCE1572_8907	-0.049031066	-15.0379	1	1
SCE1572_6698	-0.049031066	-15.9124	1	1
SCE1572_5424	-0.043154499	-14.9501	1	1
SCE1572_1422	0.039778201	-15.283	1	1
SCE1572_442	0.033431094	-14.9536	1	1
SCE1572_9052	-0.03176359	-14.9444	1	1
SCE1572_2095	-0.025667073	-14.9413	1	1
SCE1572_5321	0.024032396	-15.0014	1	1
SCE1572_8337	0.008850066	-13.304	1	1
SCE1572_8900	0.005144074	-12.6629	1	1
SCE1572_7927	-0.004319417	-14.1773	1	1
SCE1572_7765	0.16153592	-16.6551	1	1
SCE1572_10613	0.107088136	-16.6823	1	1
SCE1572_3601	0.107088136	-16.6823	1	1
SCE1572_9395	0.107088136	-16.6823	1	1
SCE1572_2785	-0.086998916	-16.6418	1	1
SCE1572_7071	-0.086998916	-16.6418	1	1
SCE1572_3032	-0.086998916	-16.6418	1	1
SCE1572_6669	-0.086998916	-16.6418	1	1
SCE1572_3732	0.050504608	-15.5731	1	1
SCE1572_9272	0.050504608	-15.5731	1	1
SCE1572_5205	-0.03438429	-14.6827	1	1
SCE1572_10521	-0.024985592	-14.3978	1	1

SCE1572_7034	0.022759617	-14.6541	1	1
SCE1572_4510	0.009532827	-13.4092	1	1
SCE1572_6787	0.008684432	-14.6611	1	1
SCE1572_510	0.008684432	-14.6611	1	1
SCE1572_2587	1.050504608	-19.5325	1	1
SCE1572_8487	1.050504608	-19.5325	1	1
SCE1572_4881	1.050504608	-19.5325	1	1
SCE1572_9133	1.050504608	-19.5325	1	1
SCE1572_1058	1.050504608	-19.5325	1	1
SCE1572_7764	1.050504608	-19.5325	1	1
SCE1572_9522	1.050504608	-19.5325	1	1
SCE1572_5273	1.050504608	-19.5325	1	1
SCE1572_8187	1.050504608	-19.5325	1	1
SCE1572_1629	1.050504608	-19.5325	1	1
SCE1572_11448	1.050504608	-19.5325	1	1
SCE1572_1886	1.050504608	-19.5325	1	1
SCE1572_1839	1.050504608	-19.5325	1	1
SCE1572_9242	1.050504608	-19.5325	1	1
SCE1572_4616	1.050504608	-19.5325	1	1
SCE1572_4698	1.050504608	-19.5325	1	1
SCE1572_5645	0.728576513	-19.6935	1	1
SCE1572_11187	0.728576513	-19.6935	1	1
SCE1572_4668	0.728576513	-19.6935	1	1
SCE1572_9252	0.728576513	-19.6935	1	1
SCE1572_2164	0.728576513	-19.6935	1	1
SCE1572_1677	0.728576513	-19.6935	1	1
SCE1572_720	0.728576513	-19.6935	1	1
SCE1572_3996	0.728576513	-19.6935	1	1
SCE1572_1845	0.728576513	-19.6935	1	1
SCE1572_10427	0.728576513	-19.6935	1	1
SCE1572_8853	0.728576513	-19.6935	1	1
SCE1572_954	0.728576513	-19.6935	1	1
SCE1572_235	-0.686460986	-19.401	1	1
SCE1572_81	-0.686460986	-19.401	1	1
SCE1572_10842	-0.686460986	-19.401	1	1
SCE1572_8574	-0.686460986	-19.401	1	1
SCE1572_5346	-0.686460986	-19.401	1	1
SCE1572_6770	-0.686460986	-19.401	1	1
SCE1572_9471	0.465542107	-18.24	1	1
SCE1572_3681	0.465542107	-18.24	1	1
SCE1572_8723	0.465542107	-18.24	1	1
SCE1572_1460	0.465542107	-18.24	1	1
SCE1572_6465	0.465542107	-18.24	1	1
SCE1572_10947	0.313539014	-18.901	1	1
SCE1572_1432	0.313539014	-18.901	1	1
SCE1572_1897	0.313539014	-18.901	1	1
SCE1572_3551	0.313539014	-18.316	1	1
SCE1572_9019	0.313539014	-18.316	1	1

SCE1572_10769	0.313539014	-18.316	1	1
SCE1572_5063	0.313539014	-18.316	1	1
SCE1572_10229	0.313539014	-18.316	1	1
SCE1572_6900	-0.271423487	-18.6085	1	1
SCE1572_5625	-0.271423487	-19.1935	1	1
SCE1572_9048	-0.271423487	-18.6085	1	1
SCE1572_6258	-0.271423487	-18.6085	1	1
SCE1572_1800	-0.271423487	-19.1935	1	1
SCE1572_2900	-0.271423487	-19.1935	1	1
SCE1572_10159	-0.271423487	-19.1935	1	1
SCE1572_4578	-0.271423487	-18.6085	1	1
SCE1572_6427	0.188008132	-17.9638	1	1
SCE1572_11002	0.188008132	-17.9638	1	1
SCE1572_4695	0.188008132	-17.9638	1	1
SCE1572_2284	0.188008132	-17.9638	1	1
SCE1572_1834	0.188008132	-17.9638	1	1
SCE1572_6610	-0.183960646	-17.1498	1	1
SCE1572_7653	-0.183960646	-17.1498	1	1
SCE1572_11453	-0.183960646	-17.1498	1	1
SCE1572_8022	-0.183960646	-17.1498	1	1
SCE1572_3065	-0.171887814	-17.3364	1	1
SCE1572_9120	0.169149104	-17.1658	1	1
SCE1572_1642	0.169149104	-17.1658	1	1
SCE1572_6752	0.169149104	-17.1658	1	1
SCE1572_5054	0.169149104	-17.1658	1	1
SCE1572_477	0.169149104	-17.1658	1	1
SCE1572_7769	0.143614012	-16.816	1	1
SCE1572_1249	0.143614012	-18.401	1	1
SCE1572_3738	0.143614012	-17.401	1	1
SCE1572_11026	0.143614012	-17.401	1	1
SCE1572_9126	0.143614012	-17.401	1	1
SCE1572_5342	0.143614012	-17.401	1	1
SCE1572_10396	0.143614012	-16.816	1	1
SCE1572_8024	0.143614012	-18.401	1	1
SCE1572_10559	0.143614012	-18.401	1	1
SCE1572_2918	0.143614012	-17.401	1	1
SCE1572_11213	0.143614012	-18.401	1	1
SCE1572_5406	0.143614012	-18.401	1	1
SCE1572_8710	0.143614012	-16.816	1	1
SCE1572_4595	0.143614012	-16.816	1	1
SCE1572_4251	-0.133919963	-17.8028	1	1
SCE1572_5113	-0.133919963	-17.8028	1	1
SCE1572_7374	-0.133919963	-17.8028	1	1
SCE1572_11515	-0.133919963	-17.8028	1	1
SCE1572_6575	0.107088136	-17.6823	1	1
SCE1572_8948	0.107088136	-17.6823	1	1
SCE1572_5964	0.107088136	-17.6823	1	1
SCE1572_5166	-0.101498486	-18.1085	1	1

SCE1572_8164	-0.101498486	-18.1085	1	1
SCE1572_10582	-0.101498486	-18.1085	1	1
SCE1572_8442	-0.101498486	-17.1085	1	1
SCE1572_1635	-0.101498486	-17.1085	1	1
SCE1572_5291	-0.101498486	-18.1085	1	1
SCE1572_9131	-0.101498486	-18.1085	1	1
SCE1572_2071	-0.101498486	-18.1085	1	1
SCE1572_5642	-0.101498486	-18.1085	1	1
SCE1572_8219	-0.101498486	-17.1085	1	1
SCE1572_3154	-0.101498486	-17.1085	1	1
SCE1572_8795	-0.093086246	-15.8564	1	1
SCE1572_626	-0.093086246	-15.8564	1	1
SCE1572_8369	-0.093086246	-15.8564	1	1
SCE1572_6320	0.091146592	-17.2048	1	1
SCE1572_4201	0.091146592	-17.2048	1	1
SCE1572_2769	0.091146592	-16.2048	1	1
SCE1572_10772	0.091146592	-16.2048	1	1
SCE1572_6027	0.091146592	-17.2048	1	1
SCE1572_758	0.091146592	-17.2048	1	1
SCE1572_7964	0.091146592	-17.2048	1	1
SCE1572_2018	0.091146592	-17.2048	1	1
SCE1572_5384	-0.090851241	-16.1963	1	1
SCE1572_7390	0.082213468	-16.8467	1	1
SCE1572_588	-0.078778409	-17.2898	1	1
SCE1572_7056	-0.078778409	-17.2898	1	1
SCE1572_6989	-0.078778409	-17.2898	1	1
SCE1572_9021	-0.078778409	-17.2898	1	1
SCE1572_8539	-0.073484109	-16.0071	1	1
SCE1572_665	-0.073484109	-16.0071	1	1
SCE1572_1617	0.072530914	-16.3211	1	1
SCE1572_5866	0.072530914	-16.3211	1	1
SCE1572_4476	0.069613431	-16.1161	1	1
SCE1572_2403	0.069613431	-16.1161	1	1
SCE1572_6529	-0.06497261	-15.3898	1	1
SCE1572_4398	0.064179545	-15.6334	1	1
SCE1572_370	0.064179545	-15.6334	1	1
SCE1572_408	0.062995552	-15.5027	1	1
SCE1572_9618	-0.061970121	-16.0888	1	1
SCE1572_1950	-0.061970121	-16.0888	1	1
SCE1572_2383	-0.058429764	-15.6276	1	1
SCE1572_4743	-0.058429764	-15.6276	1	1
SCE1572_10003	0.050504608	-16.2252	1	1
SCE1572_8433	0.050504608	-19.0325	1	1
SCE1572_11444	0.050504608	-16.7106	1	1
SCE1572_9880	0.050504608	-17.0325	1	1
SCE1572_2843	0.050504608	-19.0325	1	1
SCE1572_4591	0.050504608	-17.4476	1	1
SCE1572_4871	0.050504608	-16.0325	1	1

SCE1572_6984	0.050504608	-18.0325	1	1
SCE1572_9799	0.050504608	-19.0325	1	1
SCE1572_6686	0.050504608	-19.0325	1	1
SCE1572_6494	0.050504608	-16.7106	1	1
SCE1572_1805	0.050504608	-17.4476	1	1
SCE1572_10105	0.050504608	-16.7106	1	1
SCE1572_246	0.050504608	-19.0325	1	1
SCE1572_8832	0.050504608	-19.0325	1	1
SCE1572_1866	0.050504608	-16.7106	1	1
SCE1572_5404	0.050504608	-18.0325	1	1
SCE1572_8906	0.050504608	-15.1256	1	1
SCE1572_10866	0.050504608	-17.0325	1	1
SCE1572_2015	0.050504608	-19.0325	1	1
SCE1572_4705	0.050504608	-16.7106	1	1
SCE1572_5722	0.050504608	-16.0325	1	1
SCE1572_10392	0.050504608	-17.0325	1	1
SCE1572_3512	0.050504608	-16.0325	1	1
SCE1572_8660	0.050504608	-18.0325	1	1
SCE1572_5875	0.050504608	-19.0325	1	1
SCE1572_9109	0.050504608	-18.0325	1	1
SCE1572_11015	0.050504608	-19.0325	1	1
SCE1572_5836	0.050504608	-17.4476	1	1
SCE1572_4359	-0.049031066	-17.4973	1	1
SCE1572_10005	-0.049031066	-17.4973	1	1
SCE1572_6791	-0.049031066	-18.4973	1	1
SCE1572_5333	-0.049031066	-16.4973	1	1
SCE1572_5194	-0.049031066	-18.4973	1	1
SCE1572_8018	-0.049031066	-17.4973	1	1
SCE1572_10689	-0.049031066	-18.4973	1	1
SCE1572_7656	-0.049031066	-18.4973	1	1
SCE1572_3842	-0.049031066	-16.1754	1	1
SCE1572_2569	-0.049031066	-17.4973	1	1
SCE1572_572	-0.049031066	-17.4973	1	1
SCE1572_11229	-0.049031066	-18.4973	1	1
SCE1572_11156	-0.049031066	-16.1754	1	1
SCE1572_7880	-0.049031066	-16.4973	1	1
SCE1572_938	-0.049031066	-17.4973	1	1
SCE1572_8380	-0.049031066	-16.4973	1	1
SCE1572_6322	-0.041125868	-15.3779	1	1
SCE1572_7484	-0.040097941	-15.5543	1	1
SCE1572_9426	0.038916634	-15.3945	1	1
SCE1572_9827	0.038916634	-15.3945	1	1
SCE1572_730	0.038916634	-15.3945	1	1
SCE1572_725	0.037904571	-15.5153	1	1
SCE1572_388	-0.036958233	-15.9888	1	1
SCE1572_6241	-0.036958233	-15.9888	1	1
SCE1572_6916	-0.036958233	-15.9888	1	1
SCE1572_9431	0.036698808	-15.6471	1	1

SCE1572_3692	0.036698808	-15.6471	1	1
SCE1572_1575	0.036698808	-15.6471	1	1
SCE1572_8146	0.036698808	-15.6471	1	1
SCE1572_7262	-0.035795239	-15.1215	1	1
SCE1572_10479	-0.035795239	-15.1215	1	1
SCE1572_10371	0.035237851	-15.7922	1	1
SCE1572_96	0.035237851	-15.7922	1	1
SCE1572_544	0.033431094	-15.9536	1	1
SCE1572_11240	0.033431094	-15.9536	1	1
SCE1572_1892	-0.032636627	-15.4302	1	1
SCE1572_679	-0.031957552	-14.9038	1	1
SCE1572_11506	-0.031957552	-14.9038	1	1
SCE1572_6352	0.031139283	-15.1353	1	1
SCE1572_4122	0.031139283	-16.1353	1	1
SCE1572_2394	0.031139283	-15.1353	1	1
SCE1572_10944	-0.030415388	-15.6136	1	1
SCE1572_2195	-0.030415388	-14.6136	1	1
SCE1572_10359	-0.030415388	-15.6136	1	1
SCE1572_6270	0.028136795	-16.3433	1	1
SCE1572_5815	-0.027497904	-15.8236	1	1
SCE1572_5786	0.026917788	-14.8349	1	1
SCE1572_4100	-0.026801118	-14.5476	1	1
SCE1572_8715	-0.025947453	-14.1166	1	1
SCE1572_11009	0.025842554	-14.1622	1	1
SCE1572_2536	0.024969516	-14.2124	1	1
SCE1572_10682	0.024032396	-16.5863	1	1
SCE1572_11322	0.024032396	-16.5863	1	1
SCE1572_6291	-0.023495974	-17.0695	1	1
SCE1572_121	-0.023495974	-17.0695	1	1
SCE1572_904	-0.023495974	-15.4846	1	1
SCE1572_1400	-0.023495974	-17.0695	1	1
SCE1572_639	0.023197262	-14.0462	1	1
SCE1572_7234	0.021935456	-14.3744	1	1
SCE1572_11296	-0.021445234	-14.859	1	1
SCE1572_1802	-0.01988472	-15.6754	1	1
SCE1572_6492	-0.01988472	-15.6754	1	1
SCE1572_9908	0.018969034	-13.8388	1	1
SCE1572_9635	-0.018442746	-14.1601	1	1
SCE1572_3995	0.01808313	-15.2938	1	1
SCE1572_4566	0.01808313	-16.8788	1	1
SCE1572_6680	0.01808313	-15.8788	1	1
SCE1572_2796	0.01808313	-16.8788	1	1
SCE1572_2260	-0.017666895	-16.3662	1	1
SCE1572_3776	-0.017666895	-13.7812	1	1
SCE1572_1830	-0.017666895	-14.0442	1	1
SCE1572_6031	0.017007696	-14.1185	1	1
SCE1572_5884	0.015858465	-14.1672	1	1
SCE1572_953	-0.015083734	-15.8954	1	1

SCE1572_5044	-0.015083734	-15.8954	1	1
SCE1572_7576	-0.015083734	-15.8954	1	1
SCE1572_10757	0.014880698	-14.6928	1	1
SCE1572_11241	0.014627572	-14.2176	1	1
SCE1572_6321	-0.014412869	-17.065	1	1
SCE1572_378	0.013978732	-16.0508	1	1
SCE1572_668	-0.01362573	-15.541	1	1
SCE1572_11588	-0.01362573	-15.541	1	1
SCE1572_8537	-0.012689219	-15.2568	1	1
SCE1572_6520	0.012369479	-15.528	1	1
SCE1572_2246	-0.012036858	-15.0194	1	1
SCE1572_10762	0.011883314	-14.3239	1	1
SCE1572_2252	0.011748564	-13.6769	1	1
SCE1572_11420	0.011748564	-13.6769	1	1
SCE1572_5529	0.010976244	-14.8428	1	1
SCE1572_6155	0.010976244	-14.8428	1	1
SCE1572_6566	0.008684432	-15.2461	1	1
SCE1572_11116	0.008684432	-17.2461	1	1
SCE1572_1152	0.008684432	-17.2461	1	1
SCE1572_5796	0.008684432	-15.6611	1	1
SCE1572_6299	0.008684432	-17.2461	1	1
SCE1572_2574	0.008684432	-17.2461	1	1
SCE1572_7015	0.008684432	-17.2461	1	1
SCE1572_7054	0.008684432	-17.2461	1	1
SCE1572_9657	0.008684432	-15.2461	1	1
SCE1572_8637	0.008684432	-14.4387	1	1
SCE1572_6726	-0.008389081	-16.1551	1	1
SCE1572_4893	-0.008389081	-14.1551	1	1
SCE1572_8720	-0.008389081	-17.74	1	1
SCE1572_2789	-0.008389081	-16.1551	1	1
SCE1572_5864	-0.008389081	-17.74	1	1
SCE1572_4174	-0.008389081	-17.74	1	1
SCE1572_6650	-0.008389081	-15.4181	1	1
SCE1572_10199	0.007292541	-13.1234	1	1
SCE1572_9530	0.007292541	-14.1234	1	1
SCE1572_1004	0.006522085	-15.5448	1	1
SCE1572_11422	0.006110488	-15.0103	1	1
SCE1572_1742	-0.005143422	-13.8509	1	1
SCE1572_8326	0.004210956	-14.2228	1	1
SCE1572_217	0.004210956	-15.8077	1	1
SCE1572_5871	-0.003490282	-14.4448	1	1
SCE1572_6667	0.003436354	-13.7162	1	1
SCE1572_10822	0.003198893	-15.102	1	1
SCE1572_6085	0.003198893	-15.102	1	1
SCE1572_1288	0.003057445	-12.9688	1	1
SCE1572_1375	0.002609918	-13.4418	1	1
SCE1572_5813	0.002499235	-14.2752	1	1
SCE1572_3007	0.001595007	-16.472	1	1

SCE1572_10854	0.001595007	-14.8871	1	1
SCE1572_3698	0.001595007	-15.472	1	1
SCE1572_4355	0.001595007	-16.472	1	1
SCE1572_2754	-0.001334324	-13.971	1	1
SCE1572_820	-0.001334324	-15.971	1	1
SCE1572_3156	-0.001334324	-15.971	1	1
SCE1572_3442	0.000656058	-14.3295	1	1
SCE1572_4897	-0.000121465	-15.1999	1	1

TableS7 Homologous genes in So0157-2 and So ce56

Sorangium cellulosum So0157-2	*Sorangium cellulosum* So ce56	E-value （So0157-2 vs So ce56）	E-value （So ce56 vs So0157-2）
SCE1572_1	sce_1	0.00E+00	0.00E+00
SCE1572_10	sce_10	9.00E-171	3.00E-166
SCE1572_100	sce_87	0.00E+00	0.00E+00
SCE1572_10000	sce_8822	2.00E-55	2.00E-55
SCE1572_10002	sce_8823	0.00E+00	0.00E+00
SCE1572_10003	sce_8824	1.00E-148	1.00E-149
SCE1572_10006	sce_8830	6.00E-49	6.00E-49
SCE1572_10007	sce_8831	4.00E-139	2.00E-121
SCE1572_10008	sce_8832	4.00E-77	3.00E-91
SCE1572_10009	sce_8833	8.00E-122	3.00E-120
SCE1572_10010	sce_8834	8.00E-30	2.00E-23
SCE1572_10011	sce_8835	9.00E-151	9.00E-151
SCE1572_10012	sce_8836	2.00E-144	4.00E-136
SCE1572_10013	sce_8837	2.00E-80	2.00E-79
SCE1572_10014	sce_8838	0.00E+00	0.00E+00
SCE1572_10015	sce_8839	0.00E+00	0.00E+00
SCE1572_10015	sce_10220	0.00E+00	0.00E+00
SCE1572_10016	sce_8840	3.00E-07	1.00E-11
SCE1572_10017	sce_8841	1.00E-136	6.00E-145
SCE1572_10018	sce_9498	2.00E-172	2.00E-172
SCE1572_10019	sce_9499	0.00E+00	0.00E+00
SCE1572_1002	sce_757	1.00E-79	7.00E-80
SCE1572_10020	sce_9500	1.00E-111	1.00E-127
SCE1572_10024	sce_10364	2.00E-148	2.00E-128
SCE1572_10026	sce_8842	2.00E-48	6.00E-54
SCE1572_10027	sce_8843	6.00E-60	1.00E-68
SCE1572_10028	sce_8844	4.00E-107	5.00E-115
SCE1572_10029	sce_8845	0.00E+00	0.00E+00
SCE1572_1003	sce_758	7.00E-175	7.00E-172
SCE1572_10030	sce_8846	2.00E-107	4.00E-111
SCE1572_10033	sce_8847	0.00E+00	0.00E+00
SCE1572_10034	sce_8851	6.00E-172	3.00E-177
SCE1572_10036	sce_8852	4.00E-54	3.00E-46
SCE1572_10037	sce_8853	4.00E-89	2.00E-103
SCE1572_10038	sce_8854	5.00E-176	3.00E-167
SCE1572_1004	sce_5158	0.00E+00	0.00E+00
SCE1572_1004	sce_1116	0.00E+00	0.00E+00
SCE1572_1004	sce_7318	0.00E+00	0.00E+00
SCE1572_10040	sce_8856	0.00E+00	0.00E+00
SCE1572_10042	sce_8858	0.00E+00	0.00E+00
SCE1572_10043	sce_8859	5.00E-147	5.00E-147
SCE1572_10044	sce_8860	3.00E-36	3.00E-36
SCE1572_10045	sce_8861	3.00E-40	3.00E-39
SCE1572_10046	sce_8862	0.00E+00	0.00E+00
SCE1572_10047	sce_8863	2.00E-43	2.00E-43
SCE1572_10048	sce_8864	0.00E+00	0.00E+00
SCE1572_10049	sce_8865	2.00E-179	7.00E-175
SCE1572_1005	sce_1115	0.00E+00	0.00E+00
SCE1572_1005	sce_5159	0.00E+00	0.00E+00
SCE1572_10050	sce_8866	0.00E+00	0.00E+00
SCE1572_10051	sce_8868	0.00E+00	0.00E+00
SCE1572_10052	sce_8869	0.00E+00	0.00E+00
SCE1572_10053	sce_8870	1.00E-165	1.00E-169
SCE1572_10054	sce_8871	0.00E+00	0.00E+00
SCE1572_10055	sce_8872	0.00E+00	0.00E+00
SCE1572_10056	sce_8873	3.00E-47	3.00E-47
SCE1572_10057	sce_8874	1.00E-161	3.00E-149
SCE1572_10058	sce_8875	8.00E-65	5.00E-65
SCE1572_10059	sce_8876	0.00E+00	0.00E+00
SCE1572_1006	sce_759	2.00E-114	6.00E-126
SCE1572_10061	sce_8878	1.00E-118	5.00E-109
SCE1572_10062	sce_8879	2.00E-49	1.00E-49
SCE1572_10063	sce_8880	3.00E-66	3.00E-66
SCE1572_10064	sce_8881	2.00E-09	2.00E-09
SCE1572_10065	sce_8882	3.00E-42	3.00E-42
SCE1572_10066	sce_8883	9.00E-113	9.00E-113
SCE1572_10067	sce_8884	0.00E+00	0.00E+00
SCE1572_10068	sce_8885	4.00E-55	3.00E-65
SCE1572_10069	sce_8886	6.00E-24	1.00E-21
SCE1572_1007	sce_760	0.00E+00	0.00E+00
SCE1572_10070	sce_8887	5.00E-83	7.00E-91
SCE1572_10071	sce_8888	1.00E-63	1.00E-63
SCE1572_10072	sce_8889	1.00E-96	3.00E-102
SCE1572_10073	sce_8890	7.00E-60	7.00E-60

SCE1572_10074	sce_8891	4.00E-30	4.00E-30
SCE1572_10075	sce_8892	3.00E-104	5.00E-105
SCE1572_10076	sce_8893	3.00E-52	3.00E-52
SCE1572_10077	sce_8894	2.00E-64	2.00E-64
SCE1572_10078	sce_8895	7.00E-43	9.00E-43
SCE1572_10079	sce_8896	2.00E-22	2.00E-22
SCE1572_1008	sce_761	0.00E+00	0.00E+00
SCE1572_10080	sce_8897	2.00E-75	2.00E-75
SCE1572_10081	sce_8898	5.00E-132	5.00E-132
SCE1572_10082	sce_8899	2.00E-49	2.00E-49
SCE1572_10083	sce_8901	3.00E-65	2.00E-58
SCE1572_10084	sce_8902	2.00E-143	2.00E-143
SCE1572_10085	sce_8903	0.00E+00	0.00E+00
SCE1572_10086	sce_8904	4.00E-39	2.00E-40
SCE1572_10089	sce_8905	6.00E-95	2.00E-97
SCE1572_1009	sce_763	0.00E+00	0.00E+00
SCE1572_10090	sce_8907	3.00E-180	1.00E-180
SCE1572_10091	sce_8909	0.00E+00	0.00E+00
SCE1572_10092	sce_8910	0.00E+00	0.00E+00
SCE1572_10093	sce_8911	8.00E-21	8.00E-21
SCE1572_10095	sce_8913	3.00E-63	8.00E-65
SCE1572_10096	sce_8914	0.00E+00	0.00E+00
SCE1572_10097	sce_8915	3.00E-59	2.00E-54
SCE1572_10098	sce_8916	4.00E-142	3.00E-130
SCE1572_10099	sce_8917	3.00E-117	5.00E-117
SCE1572_10100	sce_8918	2.00E-114	4.00E-105
SCE1572_10101	sce_8919	2.00E-125	9.00E-139
SCE1572_10102	sce_8920	6.00E-128	5.00E-128
SCE1572_10103	sce_8921	3.00E-85	6.00E-87
SCE1572_10104	sce_8922	2.00E-31	1.00E-47
SCE1572_10105	sce_8923	8.00E-36	2.00E-48
SCE1572_10106	sce_8924	3.00E-137	4.00E-137
SCE1572_10107	sce_8925	4.00E-41	4.00E-41
SCE1572_10108	sce_8926	0.00E+00	0.00E+00
SCE1572_10109	sce_8927	5.00E-60	1.00E-53
SCE1572_1011	sce_9659	3.00E-45	7.00E-46
SCE1572_10111	sce_8928	0.00E+00	0.00E+00
SCE1572_10115	sce_8930	0.00E+00	0.00E+00
SCE1572_10117	sce_8934	1.00E-95	1.00E-93
SCE1572_10118	sce_8935	2.00E-110	4.00E-113
SCE1572_10119	sce_8937	3.00E-144	7.00E-144
SCE1572_10120	sce_8938	0.00E+00	2.00E-177
SCE1572_10121	sce_8939	2.00E-76	1.00E-81
SCE1572_10122	sce_8940	0.00E+00	0.00E+00
SCE1572_10123	sce_8941	2.00E-48	1.00E-48
SCE1572_1013	sce_768	0.00E+00	0.00E+00
SCE1572_10130	sce_8942	4.00E-116	2.00E-118
SCE1572_1014	sce_769	3.00E-76	3.00E-76
SCE1572_10141	sce_8944	2.00E-119	2.00E-119
SCE1572_10142	sce_8945	0.00E+00	0.00E+00
SCE1572_10143	sce_8946	4.00E-121	3.00E-120
SCE1572_10144	sce_8947	2.00E-149	3.00E-159
SCE1572_10145	sce_8948	0.00E+00	0.00E+00
SCE1572_10146	sce_8949	0.00E+00	0.00E+00
SCE1572_10147	sce_8950	2.00E-153	4.00E-164
SCE1572_10148	sce_8951	1.00E-47	1.00E-47
SCE1572_10149	sce_8952	3.00E-33	1.00E-55
SCE1572_1015	sce_3750	0.00E+00	0.00E+00
SCE1572_1015	sce_5229	0.00E+00	0.00E+00
SCE1572_1015	sce_6712	0.00E+00	0.00E+00
SCE1572_1015	sce_5226	0.00E+00	0.00E+00
SCE1572_1015	sce_9954	0.00E+00	0.00E+00
SCE1572_10150	sce_8953	3.00E-98	4.00E-106
SCE1572_10151	sce_8954	8.00E-64	9.00E-66
SCE1572_10152	sce_8955	1.00E-51	1.00E-51
SCE1572_10153	sce_8956	3.00E-65	7.00E-79
SCE1572_10154	sce_8957	4.00E-123	4.00E-133
SCE1572_10155	sce_8958	4.00E-154	1.00E-170
SCE1572_10156	sce_8959	9.00E-24	1.00E-24
SCE1572_10157	sce_8960	0.00E+00	0.00E+00
SCE1572_10159	sce_8961	1.00E-13	3.00E-11
SCE1572_10160	sce_8962	0.00E+00	0.00E+00
SCE1572_10161	sce_8963	2.00E-176	2.00E-169
SCE1572_10165	sce_8964	2.00E-149	8.00E-159
SCE1572_10167	sce_8966	0.00E+00	0.00E+00
SCE1572_10168	sce_8967	6.00E-68	1.00E-67

SCE1572_10169	sce_8968	1.00E-55	5.00E-48
SCE1572_1017	sce_772	4.00E-45	2.00E-45
SCE1572_10170	sce_8969	2.00E-143	1.00E-134
SCE1572_10172	sce_8970	8.00E-134	8.00E-134
SCE1572_10173	sce_8972	5.00E-55	2.00E-50
SCE1572_10175	sce_8974	7.00E-33	6.00E-31
SCE1572_10176	sce_8975	2.00E-37	4.00E-29
SCE1572_10177	sce_8976	1.00E-53	4.00E-63
SCE1572_10178	sce_8977	8.00E-113	3.00E-113
SCE1572_10179	sce_8978	2.00E-126	3.00E-123
SCE1572_10180	sce_8979	8.00E-146	3.00E-145
SCE1572_10181	sce_8980	7.00E-89	2.00E-74
SCE1572_10182	sce_8981	0.00E+00	0.00E+00
SCE1572_10183	sce_9845	9.00E-94	4.00E-102
SCE1572_10184	sce_9844	3.00E-84	4.00E-83
SCE1572_10186	sce_8992	2.00E-147	4.00E-151
SCE1572_10187	sce_8993	3.00E-62	3.00E-52
SCE1572_10188	sce_8994	9.00E-130	2.00E-123
SCE1572_10189	sce_8995	0.00E+00	0.00E+00
SCE1572_10190	sce_8996	0.00E+00	0.00E+00
SCE1572_10191	sce_8997	6.00E-45	6.00E-45
SCE1572_10192	sce_8998	6.00E-46	6.00E-46
SCE1572_10193	sce_8999	4.00E-147	3.00E-161
SCE1572_10194	sce_9000	0.00E+00	0.00E+00
SCE1572_10195	sce_9001	7.00E-63	7.00E-63
SCE1572_10198	sce_9003	1.00E-33	2.00E-17
SCE1572_10199	sce_9004	0.00E+00	0.00E+00
SCE1572_102	sce_88	2.00E-78	5.00E-70
SCE1572_10200	sce_9005	1.00E-73	1.00E-73
SCE1572_10201	sce_9006	3.00E-112	1.00E-125
SCE1572_10206	sce_8592	0.00E+00	0.00E+00
SCE1572_10206	sce_3937	0.00E+00	0.00E+00
SCE1572_10208	sce_9676	0.00E+00	0.00E+00
SCE1572_10210	sce_9675	9.00E-127	9.00E-127
SCE1572_10212	sce_9674	3.00E-129	2.00E-119
SCE1572_10213	sce_9673	4.00E-20	1.00E-17
SCE1572_10214	sce_9672	1.00E-143	9.00E-153
SCE1572_10216	sce_9670	4.00E-26	4.00E-25
SCE1572_10217	sce_9669	0.00E+00	0.00E+00
SCE1572_10218	sce_9668	0.00E+00	0.00E+00
SCE1572_10219	sce_9667	0.00E+00	0.00E+00
SCE1572_1022	sce_6293	8.00E-39	3.00E-40
SCE1572_10220	sce_9666	0.00E+00	0.00E+00
SCE1572_10221	sce_2358	0.00E+00	0.00E+00
SCE1572_10222	sce_9656	9.00E-118	7.00E-126
SCE1572_10223	sce_9655	0.00E+00	0.00E+00
SCE1572_10231	sce_7445	6.00E-98	4.00E-84
SCE1572_10231	sce_4534	6.00E-98	4.00E-84
SCE1572_10235	sce_7404	0.00E+00	0.00E+00
SCE1572_10239	sce_6868	8.00E-132	6.00E-129
SCE1572_10242	sce_1432	1.00E-89	8.00E-88
SCE1572_10245	sce_6866	2.00E-86	2.00E-86
SCE1572_10248	sce_7083	2.00E-55	9.00E-56
SCE1572_10250	sce_5192	6.00E-122	8.00E-117
SCE1572_10256	sce_1659	8.00E-172	6.00E-161
SCE1572_10257	sce_8036	0.00E+00	2.00E-180
SCE1572_10257	sce_6864	0.00E+00	5.00E-178
SCE1572_10259	sce_9277	3.00E-14	1.00E-08
SCE1572_10260	sce_9652	5.00E-76	9.00E-77
SCE1572_10261	sce_9651	0.00E+00	0.00E+00
SCE1572_10262	sce_9650	0.00E+00	0.00E+00
SCE1572_10265	sce_9648	6.00E-85	1.00E-84
SCE1572_10266	sce_9647	4.00E-110	2.00E-114
SCE1572_10267	sce_9646	0.00E+00	0.00E+00
SCE1572_10268	sce_9645	0.00E+00	0.00E+00
SCE1572_10269	sce_9644	9.00E-127	2.00E-125
SCE1572_10270	sce_2619	3.00E-34	2.00E-37
SCE1572_10271	sce_9643	4.00E-147	2.00E-154
SCE1572_10272	sce_9642	0.00E+00	0.00E+00
SCE1572_10273	sce_9641	9.00E-100	5.00E-96
SCE1572_10274	sce_9639	1.00E-174	1.00E-175
SCE1572_10275	sce_9638	3.00E-136	3.00E-136
SCE1572_10276	sce_9637	4.00E-167	3.00E-167
SCE1572_10277	sce_9636	6.00E-177	0.00E+00
SCE1572_10278	sce_9635	1.00E-158	3.00E-150
SCE1572_10279	sce_9634	3.00E-84	5.00E-83

SCE1572_10280	sce_9633	0.00E+00	0.00E+00
SCE1572_10281	sce_9631	1.00E-68	3.00E-69
SCE1572_10282	sce_9630	2.00E-38	4.00E-38
SCE1572_10283	sce_9629	9.00E-141	3.00E-133
SCE1572_10284	sce_9628	1.00E-106	5.00E-108
SCE1572_10285	sce_9627	8.00E-65	1.00E-82
SCE1572_10286	sce_9626	1.00E-109	9.00E-110
SCE1572_10287	sce_9625	0.00E+00	0.00E+00
SCE1572_10287	sce_5292	0.00E+00	0.00E+00
SCE1572_10287	sce_5639	0.00E+00	0.00E+00
SCE1572_10287	sce_6281	0.00E+00	0.00E+00
SCE1572_10288	sce_9624	0.00E+00	0.00E+00
SCE1572_10289	sce_9623	4.00E-58	2.00E-58
SCE1572_10290	sce_9622	5.00E-75	2.00E-62
SCE1572_10291	sce_9621	7.00E-63	7.00E-63
SCE1572_10292	sce_9620	4.00E-54	4.00E-54
SCE1572_10294	sce_9619	0.00E+00	0.00E+00
SCE1572_10295	sce_9617	2.00E-134	1.00E-142
SCE1572_10296	sce_9616	1.00E-63	1.00E-56
SCE1572_10297	sce_9615	1.00E-140	1.00E-140
SCE1572_10298	sce_9614	5.00E-138	4.00E-132
SCE1572_10299	sce_9613	9.00E-142	1.00E-143
SCE1572_103	sce_89	2.00E-120	2.00E-122
SCE1572_1030	sce_6285	7.00E-57	1.00E-65
SCE1572_10300	sce_9612	7.00E-77	4.00E-64
SCE1572_10301	sce_9611	0.00E+00	0.00E+00
SCE1572_10301	sce_9956	0.00E+00	0.00E+00
SCE1572_10302	sce_9610	2.00E-37	1.00E-51
SCE1572_10303	sce_9609	0.00E+00	0.00E+00
SCE1572_10304	sce_9607	0.00E+00	0.00E+00
SCE1572_10304	sce_4238	0.00E+00	0.00E+00
SCE1572_10305	sce_9606	1.00E-139	8.00E-145
SCE1572_10306	sce_9605	2.00E-57	4.00E-57
SCE1572_10307	sce_9604	2.00E-46	2.00E-55
SCE1572_10308	sce_9603	3.00E-88	6.00E-88
SCE1572_10309	sce_9602	3.00E-61	1.00E-62
SCE1572_10311	sce_9601	5.00E-70	9.00E-90
SCE1572_10312	sce_9600	0.00E+00	0.00E+00
SCE1572_10313	sce_9007	9.00E-69	6.00E-62
SCE1572_10314	sce_9008	7.00E-32	2.00E-39
SCE1572_10315	sce_9009	3.00E-26	3.00E-26
SCE1572_10317	sce_9010	3.00E-127	3.00E-133
SCE1572_10318	sce_9011	0.00E+00	0.00E+00
SCE1572_10319	sce_9012	4.00E-149	3.00E-160
SCE1572_10320	sce_9013	2.00E-55	4.00E-57
SCE1572_10321	sce_9015	2.00E-63	6.00E-62
SCE1572_10322	sce_9016	5.00E-18	3.00E-16
SCE1572_10323	sce_9017	3.00E-121	6.00E-138
SCE1572_10325	sce_9018	0.00E+00	0.00E+00
SCE1572_10326	sce_9019	6.00E-32	1.00E-26
SCE1572_10329	sce_9020	1.00E-98	1.00E-98
SCE1572_1033	sce_9382	9.00E-53	7.00E-49
SCE1572_10331	sce_9022	0.00E+00	0.00E+00
SCE1572_10332	sce_9023	0.00E+00	0.00E+00
SCE1572_10335	sce_9025	6.00E-49	5.00E-37
SCE1572_10336	sce_9027	7.00E-124	2.00E-127
SCE1572_10338	sce_1108	0.00E+00	0.00E+00
SCE1572_10338	sce_9028	0.00E+00	0.00E+00
SCE1572_10339	sce_9029	5.00E-68	4.00E-68
SCE1572_1034	sce_778	0.00E+00	0.00E+00
SCE1572_10340	sce_9030	0.00E+00	0.00E+00
SCE1572_10341	sce_9031	6.00E-90	1.00E-75
SCE1572_10342	sce_9032	0.00E+00	2.00E-154
SCE1572_10343	sce_9033	2.00E-148	2.00E-139
SCE1572_10344	sce_9034	1.00E-167	2.00E-169
SCE1572_10345	sce_9035	1.00E-134	1.00E-133
SCE1572_10346	sce_9037	0.00E+00	0.00E+00
SCE1572_10347	sce_9038	0.00E+00	0.00E+00
SCE1572_1035	sce_10117	0.00E+00	0.00E+00
SCE1572_10353	sce_8066	1.00E-13	7.00E-24
SCE1572_10354	sce_9218	3.00E-25	3.00E-25
SCE1572_1036	sce_7392	9.00E-19	4.00E-18
SCE1572_10367	sce_4494	0.00E+00	0.00E+00
SCE1572_10368	sce_9055	1.00E-45	1.00E-49
SCE1572_10369	sce_9056	0.00E+00	0.00E+00
SCE1572_10370	sce_9057	0.00E+00	0.00E+00

SCE1572_10371	sce_9058	3.00E-134	4.00E-155
SCE1572_10372	sce_9059	1.00E-82	2.00E-85
SCE1572_10373	sce_9061	0.00E+00	0.00E+00
SCE1572_10374	sce_9062	2.00E-50	2.00E-51
SCE1572_10375	sce_9065	4.00E-103	1.00E-103
SCE1572_10376	sce_9066	5.00E-159	6.00E-159
SCE1572_10378	sce_9067	0.00E+00	0.00E+00
SCE1572_1038	sce_6088	1.00E-12	3.00E-16
SCE1572_10380	sce_9069	0.00E+00	0.00E+00
SCE1572_10381	sce_9070	0.00E+00	0.00E+00
SCE1572_10382	sce_9071	4.00E-114	2.00E-99
SCE1572_10383	sce_9072	4.00E-132	3.00E-121
SCE1572_10384	sce_9073	2.00E-69	5.00E-81
SCE1572_10385	sce_9074	2.00E-131	9.00E-140
SCE1572_10386	sce_9075	0.00E+00	0.00E+00
SCE1572_10391	sce_9046	1.00E-146	7.00E-145
SCE1572_10393	sce_3027	0.00E+00	0.00E+00
SCE1572_10394	sce_9047	9.00E-09	9.00E-09
SCE1572_10397	sce_9216	2.00E-131	3.00E-141
SCE1572_10398	sce_9215	0.00E+00	0.00E+00
SCE1572_10399	sce_9214	0.00E+00	0.00E+00
SCE1572_104	sce_90	3.00E-41	4.00E-71
SCE1572_1040	sce_775	2.00E-51	3.00E-52
SCE1572_10400	sce_9209	6.00E-172	6.00E-172
SCE1572_10402	sce_2173	2.00E-13	1.00E-11
SCE1572_10435	sce_9076	6.00E-107	1.00E-101
SCE1572_10436	sce_9077	5.00E-139	2.00E-144
SCE1572_10437	sce_9078	2.00E-121	3.00E-119
SCE1572_10438	sce_9079	5.00E-48	2.00E-55
SCE1572_10439	sce_9080	7.00E-124	7.00E-124
SCE1572_1044	sce_780	1.00E-54	1.00E-54
SCE1572_10441	sce_9082	1.00E-44	7.00E-29
SCE1572_10442	sce_9083	0.00E+00	0.00E+00
SCE1572_10443	sce_9084	2.00E-92	4.00E-110
SCE1572_10444	sce_9085	0.00E+00	0.00E+00
SCE1572_10446	sce_9086	5.00E-68	7.00E-80
SCE1572_10447	sce_9087	0.00E+00	0.00E+00
SCE1572_10448	sce_9088	5.00E-89	5.00E-89
SCE1572_10449	sce_9089	0.00E+00	0.00E+00
SCE1572_1045	sce_781	7.00E-66	1.00E-66
SCE1572_10451	sce_9093	8.00E-35	1.00E-34
SCE1572_10452	sce_9094	2.00E-121	4.00E-112
SCE1572_10453	sce_9095	3.00E-175	8.00E-178
SCE1572_10455	sce_9096	8.00E-150	4.00E-162
SCE1572_10456	sce_9097	3.00E-59	1.00E-67
SCE1572_10459	sce_9098	7.00E-65	5.00E-68
SCE1572_1046	sce_782	0.00E+00	0.00E+00
SCE1572_10460	sce_9099	5.00E-148	4.00E-153
SCE1572_10461	sce_9100	0.00E+00	0.00E+00
SCE1572_10462	sce_9101	0.00E+00	0.00E+00
SCE1572_10463	sce_9102	0.00E+00	0.00E+00
SCE1572_10464	sce_9103	5.00E-96	1.00E-80
SCE1572_10465	sce_9104	1.00E-58	4.00E-67
SCE1572_10466	sce_9105	0.00E+00	0.00E+00
SCE1572_10469	sce_9115	4.00E-90	2.00E-93
SCE1572_1047	sce_784	1.00E-125	8.00E-129
SCE1572_10471	sce_9116	0.00E+00	0.00E+00
SCE1572_10472	sce_9117	0.00E+00	0.00E+00
SCE1572_10473	sce_9118	3.00E-174	0.00E+00
SCE1572_10474	sce_9119	0.00E+00	0.00E+00
SCE1572_10475	sce_9120	0.00E+00	0.00E+00
SCE1572_10476	sce_9121	2.00E-124	2.00E-124
SCE1572_10477	sce_9122	1.00E-50	1.00E-50
SCE1572_10478	sce_9123	2.00E-82	7.00E-82
SCE1572_10479	sce_9124	2.00E-68	6.00E-69
SCE1572_1048	sce_785	4.00E-97	7.00E-102
SCE1572_10480	sce_9125	2.00E-41	1.00E-35
SCE1572_10481	sce_9126	9.00E-144	4.00E-134
SCE1572_10482	sce_9127	9.00E-69	1.00E-63
SCE1572_10483	sce_9128	3.00E-99	8.00E-89
SCE1572_10484	sce_9129	2.00E-163	2.00E-165
SCE1572_10485	sce_9130	0.00E+00	0.00E+00
SCE1572_10486	sce_9131	0.00E+00	5.00E-163
SCE1572_10487	sce_9133	0.00E+00	0.00E+00
SCE1572_10488	sce_9135	0.00E+00	0.00E+00
SCE1572_10489	sce_9136	0.00E+00	0.00E+00

SCE1572_1049	sce_786	4.00E-40	4.00E-40
SCE1572_10490	sce_9137	2.00E-118	3.00E-117
SCE1572_10491	sce_9138	1.00E-167	6.00E-160
SCE1572_10492	sce_9140	1.00E-65	9.00E-69
SCE1572_10493	sce_9141	0.00E+00	0.00E+00
SCE1572_10494	sce_9142	0.00E+00	0.00E+00
SCE1572_10495	sce_9143	0.00E+00	0.00E+00
SCE1572_10496	sce_9144	2.00E-58	2.00E-58
SCE1572_10497	sce_9145	2.00E-134	2.00E-134
SCE1572_10498	sce_9146	3.00E-179	4.00E-174
SCE1572_10499	sce_9147	3.00E-69	3.00E-69
SCE1572_105	sce_91	2.00E-21	2.00E-21
SCE1572_10500	sce_9148	6.00E-94	5.00E-86
SCE1572_10501	sce_9149	1.00E-45	1.00E-45
SCE1572_10502	sce_9150	0.00E+00	0.00E+00
SCE1572_10503	sce_9151	4.00E-75	2.00E-71
SCE1572_10504	sce_9152	2.00E-102	2.00E-102
SCE1572_10507	sce_9156	3.00E-84	3.00E-84
SCE1572_10508	sce_9157	2.00E-73	2.00E-73
SCE1572_10509	sce_9159	3.00E-60	5.00E-68
SCE1572_1051	sce_3616	6.00E-131	1.00E-137
SCE1572_10510	sce_9160	1.00E-142	4.00E-164
SCE1572_10516	sce_2347	1.00E-13	4.00E-13
SCE1572_10518	sce_2335	5.00E-10	1.00E-12
SCE1572_10519	sce_2334	5.00E-21	1.00E-20
SCE1572_1052	sce_8827	0.00E+00	0.00E+00
SCE1572_10520	sce_2333	2.00E-30	2.00E-38
SCE1572_10521	sce_2332	5.00E-28	1.00E-27
SCE1572_10522	sce_2331	6.00E-14	2.00E-11
SCE1572_10524	sce_2329	6.00E-27	4.00E-27
SCE1572_10526	sce_2327	1.00E-10	1.00E-10
SCE1572_10527	sce_2326	1.00E-18	1.00E-18
SCE1572_10529	sce_2324	6.00E-65	4.00E-65
SCE1572_1053	sce_8826	2.00E-109	8.00E-118
SCE1572_10534	sce_2320	8.00E-52	9.00E-52
SCE1572_1054	sce_8825	2.00E-177	2.00E-177
SCE1572_1055	sce_4687	0.00E+00	0.00E+00
SCE1572_1056	sce_778	0.00E+00	0.00E+00
SCE1572_10564	sce_9181	2.00E-122	3.00E-124
SCE1572_10565	sce_9180	3.00E-11	2.00E-15
SCE1572_10566	sce_9179	7.00E-52	7.00E-52
SCE1572_10567	sce_9178	5.00E-51	5.00E-51
SCE1572_1057	sce_792	0.00E+00	0.00E+00
SCE1572_1058	sce_793	7.00E-49	3.00E-37
SCE1572_10585	sce_3805	7.00E-47	7.00E-47
SCE1572_10586	sce_3806	6.00E-16	6.00E-16
SCE1572_1059	sce_4898	3.00E-70	3.00E-70
SCE1572_10591	sce_9173	6.00E-08	4.00E-08
SCE1572_10594	sce_7746	0.00E+00	0.00E+00
SCE1572_10595	sce_7745	0.00E+00	0.00E+00
SCE1572_10595	sce_142	0.00E+00	0.00E+00
SCE1572_10595	sce_3777	0.00E+00	0.00E+00
SCE1572_10596	sce_7744	2.00E-107	6.00E-112
SCE1572_10597	sce_7743	2.00E-38	3.00E-38
SCE1572_10600	sce_7488	3.00E-15	5.00E-11
SCE1572_10604	sce_9219	0.00E+00	2.00E-180
SCE1572_10607	sce_9220	1.00E-96	1.00E-96
SCE1572_10608	sce_9221	1.00E-131	8.00E-129
SCE1572_10609	sce_9222	0.00E+00	0.00E+00
SCE1572_10609	sce_1814	0.00E+00	0.00E+00
SCE1572_10609	sce_3148	0.00E+00	0.00E+00
SCE1572_1061	sce_794	2.00E-151	3.00E-150
SCE1572_10610	sce_9042	2.00E-28	2.00E-28
SCE1572_10615	sce_9224	1.00E-164	6.00E-162
SCE1572_10616	sce_9225	2.00E-85	1.00E-83
SCE1572_10620	sce_9228	0.00E+00	0.00E+00
SCE1572_10621	sce_9229	0.00E+00	0.00E+00
SCE1572_10622	sce_9230	0.00E+00	0.00E+00
SCE1572_10622	sce_9231	0.00E+00	0.00E+00
SCE1572_10622	sce_389	0.00E+00	0.00E+00
SCE1572_10623	sce_9231	0.00E+00	0.00E+00
SCE1572_10623	sce_9237	0.00E+00	0.00E+00
SCE1572_10623	sce_9230	0.00E+00	0.00E+00
SCE1572_10624	sce_9232	5.00E-136	5.00E-138
SCE1572_10625	sce_9233	2.00E-34	2.00E-34
SCE1572_10626	sce_9234	0.00E+00	0.00E+00

SCE1572_10627	sce_9235	0.00E+00	0.00E+00
SCE1572_10628	sce_9236	0.00E+00	0.00E+00
SCE1572_10628	sce_389	0.00E+00	0.00E+00
SCE1572_10629	sce_9237	0.00E+00	0.00E+00
SCE1572_10629	sce_9231	0.00E+00	0.00E+00
SCE1572_10629	sce_2666	0.00E+00	0.00E+00
SCE1572_10629	sce_389	0.00E+00	0.00E+00
SCE1572_10629	sce_7637	0.00E+00	0.00E+00
SCE1572_10633	sce_9240	0.00E+00	0.00E+00
SCE1572_10634	sce_9238	0.00E+00	0.00E+00
SCE1572_10634	sce_4347	0.00E+00	0.00E+00
SCE1572_10635	sce_9239	5.00E-162	5.00E-180
SCE1572_10637	sce_9241	9.00E-06	1.00E-12
SCE1572_10638	sce_9242	5.00E-15	3.00E-13
SCE1572_10639	sce_9244	0.00E+00	0.00E+00
SCE1572_10640	sce_9245	0.00E+00	0.00E+00
SCE1572_10641	sce_9246	8.00E-150	1.00E-161
SCE1572_10642	sce_9247	0.00E+00	0.00E+00
SCE1572_10643	sce_9248	5.00E-157	6.00E-157
SCE1572_10644	sce_9249	2.00E-173	1.00E-173
SCE1572_10645	sce_9250	7.00E-147	9.00E-146
SCE1572_10646	sce_9251	2.00E-119	1.00E-131
SCE1572_10647	sce_9252	2.00E-91	2.00E-91
SCE1572_10648	sce_9253	1.00E-160	1.00E-158
SCE1572_1065	sce_9451	2.00E-117	2.00E-117
SCE1572_10650	sce_9254	3.00E-57	3.00E-57
SCE1572_10651	sce_9255	0.00E+00	0.00E+00
SCE1572_10652	sce_9256	9.00E-97	1.00E-104
SCE1572_10653	sce_9257	0.00E+00	0.00E+00
SCE1572_10654	sce_9258	0.00E+00	5.00E-175
SCE1572_10655	sce_9259	1.00E-34	1.00E-34
SCE1572_10656	sce_9260	0.00E+00	0.00E+00
SCE1572_10657	sce_9261	4.00E-166	5.00E-166
SCE1572_10658	sce_9263	8.00E-41	9.00E-37
SCE1572_10659	sce_9264	0.00E+00	0.00E+00
SCE1572_1066	sce_9453	2.00E-120	2.00E-119
SCE1572_10660	sce_9265	7.00E-61	2.00E-61
SCE1572_10661	sce_9266	0.00E+00	0.00E+00
SCE1572_10662	sce_9267	0.00E+00	0.00E+00
SCE1572_10663	sce_9271	0.00E+00	0.00E+00
SCE1572_10664	sce_9272	6.00E-123	6.00E-123
SCE1572_10665	sce_9273	9.00E-132	1.00E-131
SCE1572_10666	sce_9274	8.00E-160	4.00E-160
SCE1572_10667	sce_9275	4.00E-121	2.00E-92
SCE1572_10669	sce_9279	2.00E-149	2.00E-149
SCE1572_1067	sce_9454	0.00E+00	0.00E+00
SCE1572_10671	sce_9283	7.00E-35	1.00E-55
SCE1572_10672	sce_9284	1.00E-109	2.00E-99
SCE1572_10673	sce_9285	0.00E+00	0.00E+00
SCE1572_10674	sce_9286	4.00E-115	1.00E-115
SCE1572_10675	sce_9287	0.00E+00	0.00E+00
SCE1572_10676	sce_9288	1.00E-143	2.00E-149
SCE1572_10677	sce_9289	1.00E-59	1.00E-64
SCE1572_10678	sce_9290	1.00E-137	3.00E-137
SCE1572_10679	sce_9291	0.00E+00	0.00E+00
SCE1572_10681	sce_9292	3.00E-110	1.00E-110
SCE1572_10682	sce_9293	1.00E-102	9.00E-91
SCE1572_10683	sce_9294	5.00E-149	2.00E-146
SCE1572_10685	sce_9295	0.00E+00	0.00E+00
SCE1572_10686	sce_9296	0.00E+00	0.00E+00
SCE1572_10687	sce_9297	3.00E-40	3.00E-40
SCE1572_10688	sce_9298	9.00E-116	9.00E-116
SCE1572_10692	sce_9299	7.00E-94	3.00E-96
SCE1572_10693	sce_9300	0.00E+00	0.00E+00
SCE1572_10695	sce_9306	7.00E-89	7.00E-89
SCE1572_10696	sce_9307	2.00E-136	5.00E-61
SCE1572_10697	sce_9308	0.00E+00	0.00E+00
SCE1572_10698	sce_9309	1.00E-92	5.00E-78
SCE1572_10699	sce_9310	7.00E-130	2.00E-118
SCE1572_107	sce_95	2.00E-107	2.00E-107
SCE1572_10702	sce_9311	4.00E-33	4.00E-36
SCE1572_10703	sce_9312	1.00E-140	2.00E-144
SCE1572_10704	sce_9313	1.00E-80	1.00E-71
SCE1572_10710	sce_9505	1.00E-83	4.00E-76
SCE1572_10711	sce_9314	7.00E-90	8.00E-77
SCE1572_10712	sce_9315	0.00E+00	0.00E+00

SCE1572_10713	sce_9316	1.00E-86	1.00E-86
SCE1572_10717	sce_9370	3.00E-120	4.00E-118
SCE1572_10718	sce_9378	3.00E-31	2.00E-42
SCE1572_10719	sce_9379	0.00E+00	0.00E+00
SCE1572_1072	sce_795	0.00E+00	0.00E+00
SCE1572_10725	sce_9383	5.00E-143	8.00E-149
SCE1572_10728	sce_9384	5.00E-100	5.00E-100
SCE1572_10729	sce_9385	8.00E-111	3.00E-113
SCE1572_10730	sce_9387	7.00E-102	3.00E-100
SCE1572_10731	sce_9388	1.00E-31	8.00E-22
SCE1572_10732	sce_9390	0.00E+00	0.00E+00
SCE1572_10733	sce_9391	1.00E-39	8.00E-38
SCE1572_10735	sce_9393	3.00E-83	7.00E-80
SCE1572_10736	sce_9394	0.00E+00	0.00E+00
SCE1572_10737	sce_9395	0.00E+00	0.00E+00
SCE1572_10738	sce_9396	0.00E+00	0.00E+00
SCE1572_10745	sce_10010	1.00E-91	1.00E-91
SCE1572_10747	sce_9992	6.00E-139	4.00E-156
SCE1572_10748	sce_9993	5.00E-92	1.00E-124
SCE1572_10749	sce_10027	3.00E-158	3.00E-158
SCE1572_10750	sce_10004	1.00E-65	1.00E-65
SCE1572_10751	sce_827	0.00E+00	0.00E+00
SCE1572_10752	sce_5142	1.00E-113	3.00E-114
SCE1572_10753	sce_6048	0.00E+00	0.00E+00
SCE1572_10754	sce_1347	1.00E-74	2.00E-83
SCE1572_10755	sce_10007	0.00E+00	0.00E+00
SCE1572_10756	sce_9959	0.00E+00	0.00E+00
SCE1572_10757	sce_9958	0.00E+00	0.00E+00
SCE1572_10758	sce_10009	7.00E-47	2.00E-49
SCE1572_10762	sce_10003	0.00E+00	0.00E+00
SCE1572_10765	sce_10018	0.00E+00	0.00E+00
SCE1572_10767	sce_10013	3.00E-171	2.00E-159
SCE1572_10768	sce_8396	8.00E-28	7.00E-28
SCE1572_10769	sce_8395	1.00E-33	1.00E-33
SCE1572_1077	sce_5210	1.00E-105	5.00E-82
SCE1572_10772	sce_10029	7.00E-83	1.00E-75
SCE1572_10773	sce_10030	9.00E-81	5.00E-81
SCE1572_10774	sce_10031	2.00E-93	1.00E-93
SCE1572_10776	sce_9407	0.00E+00	0.00E+00
SCE1572_10776	sce_3765	0.00E+00	0.00E+00
SCE1572_10776	sce_8451	0.00E+00	0.00E+00
SCE1572_10776	sce_4817	0.00E+00	0.00E+00
SCE1572_10777	sce_9040	4.00E-162	5.00E-160
SCE1572_10778	sce_2922	0.00E+00	0.00E+00
SCE1572_1078	sce_5665	9.00E-177	9.00E-177
SCE1572_10782	sce_9409	4.00E-132	8.00E-155
SCE1572_10783	sce_9410	0.00E+00	0.00E+00
SCE1572_10784	sce_9411	0.00E+00	0.00E+00
SCE1572_10785	sce_9412	0.00E+00	0.00E+00
SCE1572_10787	sce_9413	7.00E-176	7.00E-174
SCE1572_10789	sce_9414	7.00E-73	4.00E-73
SCE1572_10790	sce_9415	5.00E-110	2.00E-110
SCE1572_10791	sce_9416	9.00E-169	2.00E-159
SCE1572_10792	sce_9417	0.00E+00	0.00E+00
SCE1572_10793	sce_9418	4.00E-97	7.00E-105
SCE1572_10794	sce_9419	9.00E-61	5.00E-57
SCE1572_10795	sce_9420	1.00E-12	1.00E-12
SCE1572_10797	sce_9422	3.00E-149	3.00E-158
SCE1572_10798	sce_9424	1.00E-56	1.00E-56
SCE1572_108	sce_96	0.00E+00	0.00E+00
SCE1572_1080	sce_796	6.00E-70	2.00E-64
SCE1572_10802	sce_9425	0.00E+00	0.00E+00
SCE1572_10805	sce_4489	8.00E-140	3.00E-148
SCE1572_10807	sce_2093	0.00E+00	0.00E+00
SCE1572_10808	sce_9441	3.00E-41	2.00E-37
SCE1572_1081	sce_798	4.00E-44	4.00E-48
SCE1572_10810	sce_9443	2.00E-55	1.00E-55
SCE1572_10812	sce_9444	9.00E-49	3.00E-51
SCE1572_10813	sce_9446	2.00E-22	4.00E-22
SCE1572_10814	sce_9447	4.00E-174	7.00E-174
SCE1572_10815	sce_9448	3.00E-158	6.00E-169
SCE1572_10816	sce_9449	5.00E-84	7.00E-90
SCE1572_10817	sce_2995	0.00E+00	0.00E+00
SCE1572_10818	sce_2996	0.00E+00	0.00E+00
SCE1572_10819	sce_2998	0.00E+00	0.00E+00
SCE1572_1082	sce_799	3.00E-95	1.00E-107

SCE1572_10820	sce_9459	8.00E-84	8.00E-84
SCE1572_10822	sce_9460	2.00E-113	2.00E-124
SCE1572_10823	sce_6965	1.00E-139	1.00E-132
SCE1572_10824	sce_8729	3.00E-45	3.00E-45
SCE1572_10825	sce_8728	5.00E-52	3.00E-63
SCE1572_10827	sce_3440	1.00E-155	4.00E-148
SCE1572_10828	sce_3441	2.00E-58	2.00E-58
SCE1572_1083	sce_800	1.00E-54	2.00E-54
SCE1572_10830	sce_3442	0.00E+00	0.00E+00
SCE1572_10831	sce_2163	1.00E-160	3.00E-156
SCE1572_10834	sce_1601	0.00E+00	0.00E+00
SCE1572_10834	sce_6742	0.00E+00	0.00E+00
SCE1572_10838	sce_9473	1.00E-174	0.00E+00
SCE1572_1084	sce_801	2.00E-133	3.00E-112
SCE1572_10840	sce_5249	2.00E-23	2.00E-23
SCE1572_10841	sce_9475	3.00E-113	6.00E-120
SCE1572_10842	sce_9476	2.00E-26	4.00E-27
SCE1572_10846	sce_1385	0.00E+00	0.00E+00
SCE1572_10847	sce_1384	0.00E+00	0.00E+00
SCE1572_10848	sce_1383	5.00E-175	0.00E+00
SCE1572_10849	sce_1382	1.00E-153	5.00E-159
SCE1572_10850	sce_1381	5.00E-161	2.00E-166
SCE1572_10852	sce_2474	6.00E-179	6.00E-179
SCE1572_10853	sce_2473	1.00E-123	1.00E-110
SCE1572_10854	sce_2472	6.00E-144	2.00E-161
SCE1572_10855	sce_2471	0.00E+00	0.00E+00
SCE1572_10856	sce_2470	2.00E-155	8.00E-163
SCE1572_10857	sce_5146	1.00E-154	3.00E-155
SCE1572_10858	sce_9486	0.00E+00	0.00E+00
SCE1572_10859	sce_9487	0.00E+00	0.00E+00
SCE1572_1086	sce_802	0.00E+00	0.00E+00
SCE1572_10860	sce_9488	6.00E-57	6.00E-57
SCE1572_10861	sce_9489	0.00E+00	0.00E+00
SCE1572_10862	sce_9491	0.00E+00	0.00E+00
SCE1572_10863	sce_9492	5.00E-130	4.00E-120
SCE1572_10865	sce_3077	1.00E-68	2.00E-68
SCE1572_10867	sce_9494	6.00E-69	6.00E-74
SCE1572_10868	sce_9495	0.00E+00	0.00E+00
SCE1572_10869	sce_9496	0.00E+00	0.00E+00
SCE1572_10871	sce_3937	0.00E+00	0.00E+00
SCE1572_10871	sce_8592	0.00E+00	0.00E+00
SCE1572_10879	sce_9509	2.00E-170	1.00E-172
SCE1572_10880	sce_9510	0.00E+00	0.00E+00
SCE1572_10882	sce_9511	1.00E-141	8.00E-145
SCE1572_10883	sce_9512	3.00E-23	1.00E-23
SCE1572_10884	sce_9513	6.00E-103	3.00E-114
SCE1572_10885	sce_9514	0.00E+00	0.00E+00
SCE1572_10886	sce_9515	2.00E-158	3.00E-142
SCE1572_10887	sce_9517	5.00E-134	2.00E-128
SCE1572_10889	sce_9521	0.00E+00	0.00E+00
SCE1572_10890	sce_9522	1.00E-71	6.00E-90
SCE1572_10892	sce_9524	7.00E-154	7.00E-172
SCE1572_10893	sce_9525	9.00E-173	1.00E-180
SCE1572_10894	sce_9529	2.00E-52	4.00E-44
SCE1572_10897	sce_9530	2.00E-99	5.00E-99
SCE1572_10898	sce_9531	1.00E-74	3.00E-79
SCE1572_109	sce_97	2.00E-121	7.00E-130
SCE1572_10901	sce_9534	2.00E-168	9.00E-159
SCE1572_10904	sce_9537	5.00E-81	7.00E-84
SCE1572_10905	sce_9539	4.00E-96	4.00E-96
SCE1572_10906	sce_9540	1.00E-20	9.00E-25
SCE1572_10907	sce_9541	0.00E+00	0.00E+00
SCE1572_10908	sce_9542	4.00E-111	1.00E-111
SCE1572_10912	sce_9548	0.00E+00	0.00E+00
SCE1572_10913	sce_9550	3.00E-30	3.00E-30
SCE1572_10914	sce_8717	5.00E-161	4.00E-168
SCE1572_10916	sce_9551	0.00E+00	0.00E+00
SCE1572_10917	sce_9552	0.00E+00	0.00E+00
SCE1572_10919	sce_9553	1.00E-108	5.00E-107
SCE1572_10920	sce_9554	4.00E-99	2.00E-92
SCE1572_10921	sce_8693	6.00E-51	7.00E-48
SCE1572_10922	sce_9555	6.00E-140	8.00E-154
SCE1572_10923	sce_9556	3.00E-125	5.00E-128
SCE1572_10924	sce_9557	0.00E+00	0.00E+00
SCE1572_10924	sce_10204	0.00E+00	0.00E+00
SCE1572_10924	sce_5835	0.00E+00	0.00E+00

SCE1572_10924	sce_388	0.00E+00	0.00E+00
SCE1572_10924	sce_7640	0.00E+00	0.00E+00
SCE1572_10924	sce_4513	0.00E+00	0.00E+00
SCE1572_10924	sce_6535	0.00E+00	0.00E+00
SCE1572_10924	sce_3003	0.00E+00	0.00E+00
SCE1572_10924	sce_9920	0.00E+00	0.00E+00
SCE1572_10924	sce_3085	0.00E+00	0.00E+00
SCE1572_10924	sce_7661	0.00E+00	0.00E+00
SCE1572_10924	sce_7109	0.00E+00	0.00E+00
SCE1572_10924	sce_3917	0.00E+00	0.00E+00
SCE1572_10924	sce_6107	0.00E+00	0.00E+00
SCE1572_10924	sce_6095	0.00E+00	0.00E+00
SCE1572_10924	sce_6093	0.00E+00	0.00E+00
SCE1572_10924	sce_6092	0.00E+00	0.00E+00
SCE1572_10924	sce_6094	0.00E+00	0.00E+00
SCE1572_10924	sce_8618	0.00E+00	0.00E+00
SCE1572_10924	sce_2065	0.00E+00	0.00E+00
SCE1572_10927	sce_9558	0.00E+00	0.00E+00
SCE1572_10928	sce_9559	4.00E-164	0.00E+00
SCE1572_10929	sce_2568	2.00E-16	5.00E-12
SCE1572_10940	sce_8783	4.00E-52	2.00E-52
SCE1572_10941	sce_6032	1.00E-14	3.00E-22
SCE1572_10947	sce_9423	7.00E-38	2.00E-45
SCE1572_10949	sce_9560	3.00E-49	3.00E-49
SCE1572_1095	sce_5295	2.00E-64	2.00E-60
SCE1572_10951	sce_9561	0.00E+00	0.00E+00
SCE1572_10955	sce_9564	0.00E+00	0.00E+00
SCE1572_10956	sce_9569	2.00E-131	4.00E-130
SCE1572_10957	sce_9570	1.00E-67	9.00E-64
SCE1572_10958	sce_9572	7.00E-153	3.00E-159
SCE1572_10959	sce_9573	0.00E+00	0.00E+00
SCE1572_10960	sce_9574	1.00E-53	6.00E-57
SCE1572_10961	sce_9575	2.00E-51	2.00E-54
SCE1572_10964	sce_9678	0.00E+00	0.00E+00
SCE1572_10965	sce_9679	0.00E+00	0.00E+00
SCE1572_10966	sce_9680	6.00E-63	1.00E-61
SCE1572_10967	sce_9681	8.00E-91	2.00E-91
SCE1572_10968	sce_9682	4.00E-97	1.00E-97
SCE1572_10969	sce_9683	0.00E+00	0.00E+00
SCE1572_10970	sce_9684	0.00E+00	0.00E+00
SCE1572_10971	sce_9685	3.00E-18	8.00E-11
SCE1572_10972	sce_9686	7.00E-155	3.00E-148
SCE1572_10973	sce_9687	0.00E+00	0.00E+00
SCE1572_10973	sce_9694	0.00E+00	0.00E+00
SCE1572_10975	sce_9696	2.00E-41	2.00E-41
SCE1572_10976	sce_9697	2.00E-10	4.00E-15
SCE1572_10977	sce_9698	2.00E-63	2.00E-62
SCE1572_10978	sce_9699	4.00E-80	3.00E-80
SCE1572_10979	sce_9701	0.00E+00	0.00E+00
SCE1572_1098	sce_3750	0.00E+00	0.00E+00
SCE1572_1098	sce_5229	0.00E+00	0.00E+00
SCE1572_1098	sce_6712	0.00E+00	0.00E+00
SCE1572_1098	sce_5226	0.00E+00	0.00E+00
SCE1572_10980	sce_9702	1.00E-110	9.00E-107
SCE1572_10981	sce_9703	0.00E+00	0.00E+00
SCE1572_10982	sce_9704	1.00E-130	1.00E-130
SCE1572_10983	sce_9705	0.00E+00	0.00E+00
SCE1572_10984	sce_9706	0.00E+00	0.00E+00
SCE1572_10985	sce_9707	3.00E-38	3.00E-38
SCE1572_10986	sce_9708	1.00E-175	1.00E-163
SCE1572_10987	sce_9709	2.00E-110	4.00E-113
SCE1572_10988	sce_9710	4.00E-69	1.00E-61
SCE1572_10989	sce_9711	4.00E-165	5.00E-153
SCE1572_10990	sce_9712	4.00E-45	4.00E-45
SCE1572_10991	sce_9713	3.00E-124	6.00E-136
SCE1572_10992	sce_9714	3.00E-34	1.00E-21
SCE1572_10994	sce_9715	8.00E-97	9.00E-109
SCE1572_10995	sce_9716	0.00E+00	0.00E+00
SCE1572_10997	sce_9718	0.00E+00	0.00E+00
SCE1572_10998	sce_9719	6.00E-88	3.00E-105
SCE1572_10999	sce_9721	1.00E-22	1.00E-22
SCE1572_11	sce_11	4.00E-148	6.00E-152
SCE1572_110	sce_98	0.00E+00	0.00E+00
SCE1572_11000	sce_9722	3.00E-30	4.00E-30
SCE1572_11001	sce_9723	2.00E-36	1.00E-27
SCE1572_11002	sce_9724	1.00E-20	2.00E-13

SCE1572_11003	sce_9725	8.00E-158	2.00E-154
SCE1572_11004	sce_9726	0.00E+00	0.00E+00
SCE1572_11005	sce_9727	0.00E+00	0.00E+00
SCE1572_11007	sce_9729	0.00E+00	0.00E+00
SCE1572_11009	sce_9730	0.00E+00	0.00E+00
SCE1572_11010	sce_9731	7.00E-142	3.00E-139
SCE1572_11019	sce_9732	1.00E-40	7.00E-30
SCE1572_11020	sce_9733	8.00E-77	4.00E-68
SCE1572_11021	sce_9734	5.00E-118	2.00E-138
SCE1572_11022	sce_9735	0.00E+00	0.00E+00
SCE1572_11022	sce_3075	0.00E+00	0.00E+00
SCE1572_11023	sce_9737	0.00E+00	0.00E+00
SCE1572_11024	sce_9738	2.00E-66	2.00E-66
SCE1572_11025	sce_9739	0.00E+00	0.00E+00
SCE1572_11027	sce_9741	1.00E-54	2.00E-46
SCE1572_11028	sce_9742	4.00E-27	2.00E-19
SCE1572_11029	sce_9743	1.00E-23	7.00E-23
SCE1572_11030	sce_9744	2.00E-72	7.00E-66
SCE1572_11033	sce_9745	6.00E-08	7.00E-07
SCE1572_11034	sce_9746	0.00E+00	0.00E+00
SCE1572_11035	sce_9747	7.00E-80	5.00E-71
SCE1572_11036	sce_9748	7.00E-42	6.00E-42
SCE1572_11037	sce_9749	0.00E+00	0.00E+00
SCE1572_11038	sce_9750	2.00E-15	2.00E-15
SCE1572_11039	sce_9751	0.00E+00	0.00E+00
SCE1572_11040	sce_9752	0.00E+00	0.00E+00
SCE1572_11041	sce_9754	8.00E-26	6.00E-27
SCE1572_11042	sce_9755	5.00E-101	6.00E-101
SCE1572_11043	sce_9757	0.00E+00	0.00E+00
SCE1572_11043	sce_3883	0.00E+00	0.00E+00
SCE1572_11043	sce_5226	0.00E+00	0.00E+00
SCE1572_11043	sce_5403	0.00E+00	0.00E+00
SCE1572_11043	sce_5751	0.00E+00	0.00E+00
SCE1572_11043	sce_9665	0.00E+00	0.00E+00
SCE1572_11043	sce_7154	0.00E+00	0.00E+00
SCE1572_11043	sce_7969	0.00E+00	0.00E+00
SCE1572_11043	sce_9954	0.00E+00	0.00E+00
SCE1572_11043	sce_5746	0.00E+00	0.00E+00
SCE1572_11043	sce_6712	0.00E+00	0.00E+00
SCE1572_11043	sce_2053	0.00E+00	0.00E+00
SCE1572_11043	sce_3750	0.00E+00	0.00E+00
SCE1572_11043	sce_5229	0.00E+00	0.00E+00
SCE1572_11044	sce_7609	7.00E-81	2.00E-75
SCE1572_11046	sce_9758	0.00E+00	0.00E+00
SCE1572_11046	sce_5980	0.00E+00	0.00E+00
SCE1572_11047	sce_9760	6.00E-86	3.00E-85
SCE1572_11048	sce_9761	2.00E-177	0.00E+00
SCE1572_11049	sce_9762	0.00E+00	0.00E+00
SCE1572_11050	sce_9763	2.00E-93	3.00E-87
SCE1572_11051	sce_9765	1.00E-93	1.00E-108
SCE1572_11052	sce_9766	1.00E-150	5.00E-146
SCE1572_11053	sce_9767	0.00E+00	0.00E+00
SCE1572_11054	sce_9771	7.00E-117	2.00E-124
SCE1572_11055	sce_9772	0.00E+00	0.00E+00
SCE1572_11056	sce_9773	6.00E-50	9.00E-50
SCE1572_11062	sce_4581	0.00E+00	0.00E+00
SCE1572_11062	sce_9106	0.00E+00	0.00E+00
SCE1572_11064	sce_9779	0.00E+00	0.00E+00
SCE1572_11066	sce_9780	0.00E+00	0.00E+00
SCE1572_11068	sce_9813	0.00E+00	0.00E+00
SCE1572_11069	sce_9814	0.00E+00	0.00E+00
SCE1572_11070	sce_9815	8.00E-98	1.00E-101
SCE1572_11072	sce_9822	4.00E-122	5.00E-131
SCE1572_11073	sce_9823	4.00E-35	2.00E-28
SCE1572_11074	sce_9824	1.00E-08	2.00E-12
SCE1572_11075	sce_9825	7.00E-42	1.00E-54
SCE1572_11076	sce_9826	2.00E-54	8.00E-74
SCE1572_11077	sce_9827	1.00E-79	1.00E-79
SCE1572_11078	sce_7074	1.00E-09	8.00E-09
SCE1572_11079	sce_9829	2.00E-56	1.00E-45
SCE1572_11080	sce_9830	4.00E-92	1.00E-93
SCE1572_11081	sce_9831	3.00E-167	3.00E-174
SCE1572_11082	sce_9832	6.00E-112	4.00E-119
SCE1572_11083	sce_9833	6.00E-37	3.00E-45
SCE1572_11090	sce_9835	0.00E+00	0.00E+00
SCE1572_11091	sce_9836	8.00E-120	6.00E-122

SCE1572_11093	sce_9837	0.00E+00	0.00E+00
SCE1572_11094	sce_9838	0.00E+00	0.00E+00
SCE1572_11096	sce_9839	2.00E-151	8.00E-157
SCE1572_11097	sce_9840	0.00E+00	0.00E+00
SCE1572_11098	sce_9841	0.00E+00	0.00E+00
SCE1572_11099	sce_9842	3.00E-119	6.00E-123
SCE1572_111	sce_99	5.00E-139	1.00E-149
SCE1572_11102	sce_9843	3.00E-112	2.00E-117
SCE1572_11103	sce_6096	7.00E-52	2.00E-53
SCE1572_11104	sce_6097	1.00E-64	1.00E-71
SCE1572_11105	sce_6098	2.00E-15	1.00E-26
SCE1572_11106	sce_6099	4.00E-79	8.00E-88
SCE1572_11111	sce_311	4.00E-130	2.00E-122
SCE1572_11112	sce_1119	4.00E-54	1.00E-51
SCE1572_11113	sce_9847	0.00E+00	0.00E+00
SCE1572_11114	sce_7062	9.00E-71	2.00E-71
SCE1572_11117	sce_6854	2.00E-54	2.00E-55
SCE1572_11118	sce_8356	0.00E+00	0.00E+00
SCE1572_11119	sce_8357	0.00E+00	0.00E+00
SCE1572_11119	sce_225	0.00E+00	0.00E+00
SCE1572_11119	sce_6235	0.00E+00	0.00E+00
SCE1572_11119	sce_2161	0.00E+00	0.00E+00
SCE1572_11119	sce_5276	0.00E+00	0.00E+00
SCE1572_11120	sce_6777	1.00E-41	2.00E-43
SCE1572_11121	sce_6778	6.00E-85	6.00E-85
SCE1572_11123	sce_1320	0.00E+00	0.00E+00
SCE1572_11125	sce_3466	2.00E-61	1.00E-71
SCE1572_11126	sce_9853	0.00E+00	0.00E+00
SCE1572_11130	sce_3722	0.00E+00	0.00E+00
SCE1572_11131	sce_3725	4.00E-109	1.00E-112
SCE1572_11132	sce_3724	0.00E+00	0.00E+00
SCE1572_11133	sce_3723	3.00E-148	1.00E-152
SCE1572_11134	sce_8378	2.00E-142	3.00E-140
SCE1572_11137	sce_8625	3.00E-59	1.00E-57
SCE1572_1114	sce_7128	2.00E-27	1.00E-27
SCE1572_11145	sce_9872	6.00E-101	6.00E-101
SCE1572_11151	sce_9873	1.00E-110	2.00E-114
SCE1572_11152	sce_9874	1.00E-23	9.00E-16
SCE1572_11153	sce_8393	1.00E-72	6.00E-63
SCE1572_11156	sce_7817	6.00E-22	1.00E-19
SCE1572_11159	sce_9876	9.00E-38	1.00E-30
SCE1572_11160	sce_9877	6.00E-99	6.00E-90
SCE1572_11161	sce_9887	0.00E+00	0.00E+00
SCE1572_11162	sce_9888	6.00E-178	0.00E+00
SCE1572_11163	sce_9890	3.00E-166	4.00E-166
SCE1572_11165	sce_9891	2.00E-87	1.00E-96
SCE1572_11166	sce_9892	2.00E-116	7.00E-112
SCE1572_11167	sce_8358	1.00E-26	1.00E-19
SCE1572_11170	sce_10020	3.00E-150	6.00E-163
SCE1572_11171	sce_10021	2.00E-44	2.00E-44
SCE1572_11172	sce_9911	8.00E-107	2.00E-100
SCE1572_11173	sce_9912	9.00E-111	7.00E-111
SCE1572_11174	sce_9913	0.00E+00	0.00E+00
SCE1572_11175	sce_9914	0.00E+00	0.00E+00
SCE1572_11177	sce_9915	8.00E-104	3.00E-103
SCE1572_11179	sce_9922	6.00E-169	3.00E-169
SCE1572_11180	sce_9924	2.00E-79	6.00E-81
SCE1572_11181	sce_9925	4.00E-107	4.00E-107
SCE1572_11182	sce_9926	5.00E-63	5.00E-78
SCE1572_11183	sce_9927	8.00E-131	8.00E-131
SCE1572_11189	sce_2238	0.00E+00	0.00E+00
SCE1572_11191	sce_9930	0.00E+00	0.00E+00
SCE1572_11192	sce_9931	3.00E-156	9.00E-150
SCE1572_11193	sce_9932	2.00E-84	3.00E-84
SCE1572_11194	sce_9933	2.00E-102	3.00E-92
SCE1572_11195	sce_9948	2.00E-79	5.00E-83
SCE1572_11196	sce_9947	4.00E-152	2.00E-180
SCE1572_11198	sce_9949	0.00E+00	0.00E+00
SCE1572_11199	sce_9950	1.00E-66	2.00E-58
SCE1572_11200	sce_9951	0.00E+00	7.00E-175
SCE1572_11201	sce_9952	7.00E-59	9.00E-68
SCE1572_11202	sce_9953	2.00E-152	3.00E-147
SCE1572_11206	sce_9954	0.00E+00	0.00E+00
SCE1572_11206	sce_5226	0.00E+00	0.00E+00
SCE1572_11206	sce_7154	0.00E+00	0.00E+00
SCE1572_11206	sce_5403	0.00E+00	0.00E+00

SCE1572_11206	sce_1803	0.00E+00	0.00E+00
SCE1572_11206	sce_3750	0.00E+00	0.00E+00
SCE1572_11206	sce_6712	0.00E+00	0.00E+00
SCE1572_11206	sce_7969	0.00E+00	0.00E+00
SCE1572_11206	sce_5751	0.00E+00	0.00E+00
SCE1572_11206	sce_9665	0.00E+00	0.00E+00
SCE1572_11206	sce_5746	0.00E+00	0.00E+00
SCE1572_11206	sce_2053	0.00E+00	0.00E+00
SCE1572_11206	sce_5229	0.00E+00	0.00E+00
SCE1572_11206	sce_9757	0.00E+00	0.00E+00
SCE1572_11206	sce_4073	0.00E+00	0.00E+00
SCE1572_11206	sce_3491	0.00E+00	0.00E+00
SCE1572_11206	sce_7465	0.00E+00	0.00E+00
SCE1572_11206	sce_7657	0.00E+00	0.00E+00
SCE1572_11208	sce_9956	0.00E+00	0.00E+00
SCE1572_11209	sce_9957	3.00E-118	9.00E-147
SCE1572_11210	sce_9963	5.00E-51	5.00E-51
SCE1572_11211	sce_9964	2.00E-144	2.00E-141
SCE1572_11215	sce_3793	0.00E+00	0.00E+00
SCE1572_11215	sce_7069	0.00E+00	0.00E+00
SCE1572_11215	sce_5085	0.00E+00	0.00E+00
SCE1572_11215	sce_5086	0.00E+00	0.00E+00
SCE1572_11215	sce_6701	0.00E+00	0.00E+00
SCE1572_11217	sce_9972	3.00E-142	7.00E-142
SCE1572_11218	sce_9973	0.00E+00	0.00E+00
SCE1572_11218	sce_9974	0.00E+00	0.00E+00
SCE1572_11225	sce_2399	3.00E-161	1.00E-161
SCE1572_11226	sce_2398	4.00E-121	1.00E-122
SCE1572_11227	sce_2397	3.00E-156	2.00E-156
SCE1572_11228	sce_2396	1.00E-81	8.00E-82
SCE1572_11230	sce_4485	3.00E-120	4.00E-120
SCE1572_11231	sce_9974	0.00E+00	0.00E+00
SCE1572_11231	sce_9973	0.00E+00	0.00E+00
SCE1572_11231	sce_8694	0.00E+00	0.00E+00
SCE1572_11231	sce_2645	0.00E+00	0.00E+00
SCE1572_11232	sce_9975	2.00E-131	5.00E-134
SCE1572_11233	sce_9976	3.00E-65	2.00E-62
SCE1572_11235	sce_9977	9.00E-148	1.00E-151
SCE1572_11236	sce_9978	0.00E+00	0.00E+00
SCE1572_11237	sce_9979	6.00E-141	1.00E-134
SCE1572_11238	sce_9980	4.00E-171	2.00E-163
SCE1572_11239	sce_9981	3.00E-164	4.00E-168
SCE1572_11240	sce_3744	4.00E-65	1.00E-65
SCE1572_11242	sce_1013	0.00E+00	0.00E+00
SCE1572_11243	sce_4521	1.00E-71	1.00E-78
SCE1572_11244	sce_10033	9.00E-100	7.00E-100
SCE1572_11246	sce_5205	9.00E-137	7.00E-131
SCE1572_11249	sce_10035	4.00E-61	1.00E-70
SCE1572_11253	sce_10036	0.00E+00	0.00E+00
SCE1572_11253	sce_5546	0.00E+00	0.00E+00
SCE1572_11254	sce_10037	0.00E+00	0.00E+00
SCE1572_11255	sce_10038	0.00E+00	0.00E+00
SCE1572_11256	sce_10039	3.00E-96	2.00E-106
SCE1572_11257	sce_10040	1.00E-58	6.00E-58
SCE1572_11258	sce_10041	3.00E-62	3.00E-62
SCE1572_11259	sce_10042	0.00E+00	0.00E+00
SCE1572_11260	sce_10043	6.00E-171	6.00E-171
SCE1572_11261	sce_10044	4.00E-37	4.00E-37
SCE1572_11262	sce_10045	2.00E-163	0.00E+00
SCE1572_11263	sce_10046	3.00E-148	2.00E-153
SCE1572_11264	sce_10047	7.00E-67	7.00E-67
SCE1572_11265	sce_10048	0.00E+00	0.00E+00
SCE1572_11266	sce_10049	3.00E-173	3.00E-173
SCE1572_11267	sce_10050	6.00E-91	6.00E-91
SCE1572_11268	sce_10051	0.00E+00	0.00E+00
SCE1572_11269	sce_10052	4.00E-108	7.00E-112
SCE1572_11270	sce_10053	0.00E+00	0.00E+00
SCE1572_11271	sce_10054	0.00E+00	0.00E+00
SCE1572_11272	sce_10055	6.00E-119	2.00E-133
SCE1572_11273	sce_10056	2.00E-157	2.00E-149
SCE1572_11274	sce_10057	2.00E-148	2.00E-148
SCE1572_11275	sce_10058	2.00E-149	2.00E-157
SCE1572_11276	sce_10060	1.00E-63	1.00E-63
SCE1572_11277	sce_10061	0.00E+00	0.00E+00
SCE1572_11279	sce_10062	4.00E-73	4.00E-73
SCE1572_1128	sce_808	0.00E+00	0.00E+00

SCE1572_11280	sce_10063	0.00E+00	0.00E+00
SCE1572_11281	sce_10064	0.00E+00	0.00E+00
SCE1572_11282	sce_10065	3.00E-156	1.00E-160
SCE1572_11283	sce_10067	2.00E-88	9.00E-82
SCE1572_11284	sce_10068	5.00E-131	4.00E-133
SCE1572_11285	sce_10069	8.00E-109	8.00E-109
SCE1572_11286	sce_1373	1.00E-106	7.00E-119
SCE1572_11287	sce_10070	4.00E-71	5.00E-67
SCE1572_11288	sce_10071	0.00E+00	0.00E+00
SCE1572_11290	sce_10072	0.00E+00	0.00E+00
SCE1572_11291	sce_10074	7.00E-35	7.00E-35
SCE1572_11292	sce_10075	4.00E-164	0.00E+00
SCE1572_11293	sce_10076	2.00E-173	1.00E-169
SCE1572_11294	sce_10077	2.00E-32	3.00E-36
SCE1572_11295	sce_10078	1.00E-56	3.00E-47
SCE1572_11296	sce_10079	0.00E+00	0.00E+00
SCE1572_11297	sce_10080	0.00E+00	1.00E-150
SCE1572_11298	sce_10081	2.00E-130	1.00E-130
SCE1572_11299	sce_10082	1.00E-135	1.00E-135
SCE1572_113	sce_101	1.00E-164	9.00E-166
SCE1572_11300	sce_10083	0.00E+00	0.00E+00
SCE1572_11301	sce_10084	2.00E-40	3.00E-41
SCE1572_11302	sce_10085	0.00E+00	0.00E+00
SCE1572_11303	sce_10086	0.00E+00	0.00E+00
SCE1572_11304	sce_10087	0.00E+00	0.00E+00
SCE1572_11306	sce_10088	0.00E+00	0.00E+00
SCE1572_11307	sce_10089	3.00E-139	9.00E-139
SCE1572_11308	sce_10090	0.00E+00	2.00E-163
SCE1572_11309	sce_10091	0.00E+00	4.00E-161
SCE1572_11313	sce_10092	6.00E-95	7.00E-95
SCE1572_11315	sce_10095	0.00E+00	0.00E+00
SCE1572_11316	sce_10096	0.00E+00	0.00E+00
SCE1572_11317	sce_10097	0.00E+00	0.00E+00
SCE1572_11318	sce_10098	0.00E+00	0.00E+00
SCE1572_1132	sce_809	8.00E-69	1.00E-69
SCE1572_11320	sce_10100	2.00E-75	3.00E-84
SCE1572_11321	sce_4678	6.00E-137	4.00E-144
SCE1572_11322	sce_10101	2.00E-128	2.00E-119
SCE1572_11323	sce_10102	0.00E+00	0.00E+00
SCE1572_11324	sce_10103	1.00E-87	4.00E-86
SCE1572_11325	sce_10105	3.00E-166	3.00E-170
SCE1572_11326	sce_10106	1.00E-122	1.00E-127
SCE1572_11327	sce_10107	1.00E-151	7.00E-134
SCE1572_1133	sce_810	2.00E-10	3.00E-08
SCE1572_11330	sce_10110	9.00E-69	1.00E-70
SCE1572_11331	sce_10111	1.00E-103	1.00E-103
SCE1572_11332	sce_10112	1.00E-86	2.00E-86
SCE1572_11333	sce_10113	0.00E+00	0.00E+00
SCE1572_11334	sce_10114	3.00E-40	6.00E-34
SCE1572_11336	sce_10135	8.00E-07	5.00E-07
SCE1572_1134	sce_8742	2.00E-25	2.00E-25
SCE1572_11340	sce_10140	5.00E-135	2.00E-142
SCE1572_11342	sce_10141	5.00E-163	7.00E-163
SCE1572_11343	sce_10142	2.00E-83	1.00E-91
SCE1572_11344	sce_10143	0.00E+00	0.00E+00
SCE1572_11345	sce_10144	2.00E-57	5.00E-57
SCE1572_11346	sce_10145	0.00E+00	0.00E+00
SCE1572_11347	sce_10146	6.00E-142	4.00E-142
SCE1572_11348	sce_10147	9.00E-25	3.00E-25
SCE1572_11351	sce_3937	0.00E+00	0.00E+00
SCE1572_11351	sce_8592	0.00E+00	0.00E+00
SCE1572_11354	sce_10188	4.00E-105	3.00E-118
SCE1572_11355	sce_8736	5.00E-30	9.00E-34
SCE1572_11356	sce_10189	1.00E-72	3.00E-78
SCE1572_11360	sce_10195	1.00E-178	7.00E-165
SCE1572_11361	sce_10196	0.00E+00	0.00E+00
SCE1572_11362	sce_10202	2.00E-178	5.00E-176
SCE1572_11363	sce_10203	0.00E+00	0.00E+00
SCE1572_11363	sce_2733	0.00E+00	0.00E+00
SCE1572_11364	sce_10204	0.00E+00	0.00E+00
SCE1572_11364	sce_5835	0.00E+00	0.00E+00
SCE1572_11364	sce_4513	0.00E+00	0.00E+00
SCE1572_11364	sce_388	0.00E+00	0.00E+00
SCE1572_11364	sce_7640	0.00E+00	0.00E+00
SCE1572_11364	sce_7661	0.00E+00	0.00E+00
SCE1572_11364	sce_3085	0.00E+00	0.00E+00

SCE1572_11364	sce_9557	0.00E+00	0.00E+00
SCE1572_11364	sce_7109	0.00E+00	0.00E+00
SCE1572_11364	sce_6535	0.00E+00	0.00E+00
SCE1572_11364	sce_9920	0.00E+00	0.00E+00
SCE1572_11364	sce_3003	0.00E+00	0.00E+00
SCE1572_11364	sce_6092	0.00E+00	0.00E+00
SCE1572_11364	sce_6093	0.00E+00	0.00E+00
SCE1572_11364	sce_6095	0.00E+00	0.00E+00
SCE1572_11364	sce_2065	0.00E+00	0.00E+00
SCE1572_11364	sce_8618	0.00E+00	0.00E+00
SCE1572_11364	sce_3917	0.00E+00	0.00E+00
SCE1572_1137	sce_9398	1.00E-109	1.00E-134
SCE1572_11370	sce_10205	2.00E-121	7.00E-135
SCE1572_11371	sce_10206	1.00E-118	9.00E-129
SCE1572_11372	sce_10209	1.00E-156	2.00E-159
SCE1572_11373	sce_10210	2.00E-139	2.00E-139
SCE1572_11374	sce_10211	0.00E+00	0.00E+00
SCE1572_11376	sce_10212	2.00E-152	3.00E-152
SCE1572_11377	sce_10213	4.00E-111	4.00E-111
SCE1572_11378	sce_10214	0.00E+00	0.00E+00
SCE1572_11379	sce_10215	8.00E-145	3.00E-147
SCE1572_11380	sce_10216	2.00E-74	1.00E-74
SCE1572_11381	sce_10217	8.00E-138	2.00E-157
SCE1572_11382	sce_10218	0.00E+00	0.00E+00
SCE1572_11384	sce_10219	0.00E+00	0.00E+00
SCE1572_11385	sce_10220	0.00E+00	0.00E+00
SCE1572_11385	sce_8839	0.00E+00	0.00E+00
SCE1572_11386	sce_10221	0.00E+00	0.00E+00
SCE1572_11387	sce_10223	2.00E-146	2.00E-146
SCE1572_11389	sce_10228	0.00E+00	0.00E+00
SCE1572_11389	sce_2553	0.00E+00	0.00E+00
SCE1572_11390	sce_10229	3.00E-122	3.00E-131
SCE1572_11391	sce_10230	8.00E-133	6.00E-133
SCE1572_11392	sce_10231	0.00E+00	0.00E+00
SCE1572_11393	sce_10233	0.00E+00	0.00E+00
SCE1572_11394	sce_10235	2.00E-53	2.00E-53
SCE1572_11395	sce_10237	0.00E+00	0.00E+00
SCE1572_11396	sce_10238	5.00E-54	2.00E-55
SCE1572_11398	sce_10239	0.00E+00	0.00E+00
SCE1572_11399	sce_10240	0.00E+00	0.00E+00
SCE1572_1140	sce_814	8.00E-24	1.00E-27
SCE1572_11400	sce_10241	0.00E+00	0.00E+00
SCE1572_11402	sce_10242	5.00E-47	4.00E-51
SCE1572_11404	sce_10243	2.00E-115	1.00E-108
SCE1572_11410	sce_10267	1.00E-132	2.00E-142
SCE1572_11412	sce_10269	2.00E-55	2.00E-49
SCE1572_11413	sce_10270	0.00E+00	0.00E+00
SCE1572_11414	sce_10271	0.00E+00	0.00E+00
SCE1572_11415	sce_10272	1.00E-119	1.00E-119
SCE1572_11416	sce_10274	2.00E-107	3.00E-92
SCE1572_1142	sce_815	0.00E+00	0.00E+00
SCE1572_11420	sce_10275	0.00E+00	0.00E+00
SCE1572_11421	sce_10276	0.00E+00	0.00E+00
SCE1572_11422	sce_10277	3.00E-144	3.00E-156
SCE1572_11424	sce_10283	5.00E-106	1.00E-96
SCE1572_11429	sce_3038	2.00E-105	2.00E-97
SCE1572_1143	sce_816	3.00E-38	2.00E-33
SCE1572_11430	sce_10284	0.00E+00	0.00E+00
SCE1572_11431	sce_10285	0.00E+00	0.00E+00
SCE1572_11432	sce_10288	0.00E+00	2.00E-176
SCE1572_11433	sce_10289	0.00E+00	0.00E+00
SCE1572_11435	sce_10290	0.00E+00	0.00E+00
SCE1572_11436	sce_10291	5.00E-179	7.00E-175
SCE1572_11437	sce_10292	1.00E-66	9.00E-73
SCE1572_11438	sce_10293	0.00E+00	0.00E+00
SCE1572_11439	sce_10294	0.00E+00	0.00E+00
SCE1572_11440	sce_10295	3.00E-113	2.00E-113
SCE1572_11441	sce_10296	4.00E-78	5.00E-80
SCE1572_11442	sce_10297	8.00E-80	4.00E-85
SCE1572_11443	sce_10298	4.00E-48	5.00E-48
SCE1572_11444	sce_10299	2.00E-57	7.00E-58
SCE1572_11445	sce_10300	0.00E+00	0.00E+00
SCE1572_11446	sce_10301	0.00E+00	0.00E+00
SCE1572_11447	sce_10302	0.00E+00	0.00E+00
SCE1572_11449	sce_10304	2.00E-146	1.00E-152
SCE1572_11450	sce_10305	0.00E+00	0.00E+00

SCE1572_11451	sce_10306	1.00E-85	1.00E-85
SCE1572_11452	sce_10307	6.00E-64	5.00E-64
SCE1572_11453	sce_10308	3.00E-57	3.00E-57
SCE1572_11457	sce_5222	0.00E+00	0.00E+00
SCE1572_11457	sce_5216	0.00E+00	0.00E+00
SCE1572_11458	sce_3037	5.00E-40	1.00E-35
SCE1572_11459	sce_5221	6.00E-25	2.00E-27
SCE1572_1146	sce_817	1.00E-72	7.00E-65
SCE1572_11460	sce_5222	0.00E+00	0.00E+00
SCE1572_11460	sce_5216	0.00E+00	0.00E+00
SCE1572_11460	sce_3035	0.00E+00	0.00E+00
SCE1572_11462	sce_1332	1.00E-14	1.00E-16
SCE1572_11468	sce_10309	7.00E-77	5.00E-70
SCE1572_11469	sce_10310	1.00E-94	2.00E-97
SCE1572_1147	sce_3880	0.00E+00	0.00E+00
SCE1572_11470	sce_10311	2.00E-103	1.00E-112
SCE1572_11471	sce_10312	1.00E-153	6.00E-147
SCE1572_11474	sce_9503	0.00E+00	0.00E+00
SCE1572_11474	sce_10365	0.00E+00	0.00E+00
SCE1572_11474	sce_9908	0.00E+00	0.00E+00
SCE1572_11478	sce_10313	6.00E-132	6.00E-131
SCE1572_1148	sce_3879	0.00E+00	0.00E+00
SCE1572_11480	sce_10315	0.00E+00	0.00E+00
SCE1572_11481	sce_10316	3.00E-129	1.00E-132
SCE1572_11482	sce_10317	4.00E-144	3.00E-144
SCE1572_11483	sce_10318	8.00E-103	8.00E-103
SCE1572_11484	sce_10319	2.00E-143	3.00E-146
SCE1572_11485	sce_10321	0.00E+00	0.00E+00
SCE1572_11486	sce_10322	3.00E-74	1.00E-75
SCE1572_11491	sce_10323	1.00E-180	6.00E-179
SCE1572_11492	sce_10324	0.00E+00	0.00E+00
SCE1572_11493	sce_10326	0.00E+00	0.00E+00
SCE1572_11499	sce_10332	4.00E-57	5.00E-54
SCE1572_115	sce_103	3.00E-71	2.00E-81
SCE1572_11500	sce_10333	7.00E-149	1.00E-140
SCE1572_11502	sce_10334	2.00E-42	3.00E-49
SCE1572_11503	sce_10335	7.00E-115	7.00E-103
SCE1572_11505	sce_10337	2.00E-82	1.00E-81
SCE1572_11506	sce_10338	2.00E-111	2.00E-114
SCE1572_11507	sce_10339	4.00E-133	4.00E-133
SCE1572_11508	sce_10340	1.00E-92	8.00E-92
SCE1572_11509	sce_10341	0.00E+00	0.00E+00
SCE1572_11510	sce_10342	3.00E-40	2.00E-45
SCE1572_11511	sce_10344	0.00E+00	0.00E+00
SCE1572_11512	sce_10345	6.00E-141	3.00E-144
SCE1572_11513	sce_10346	0.00E+00	0.00E+00
SCE1572_11514	sce_10347	0.00E+00	0.00E+00
SCE1572_11515	sce_10348	1.00E-88	9.00E-92
SCE1572_11516	sce_10349	5.00E-117	2.00E-139
SCE1572_11517	sce_10350	0.00E+00	0.00E+00
SCE1572_11518	sce_10351	8.00E-116	7.00E-116
SCE1572_11519	sce_10352	2.00E-124	8.00E-127
SCE1572_11520	sce_10353	8.00E-29	1.00E-36
SCE1572_11523	sce_7057	1.00E-15	6.00E-17
SCE1572_11524	sce_7056	1.00E-24	1.00E-20
SCE1572_11525	sce_7055	6.00E-95	2.00E-97
SCE1572_11526	sce_10265	6.00E-21	1.00E-21
SCE1572_11529	sce_10371	0.00E+00	0.00E+00
SCE1572_11530	sce_10372	3.00E-23	4.00E-23
SCE1572_11531	sce_10373	1.00E-70	1.00E-81
SCE1572_11533	sce_10380	1.00E-133	1.00E-122
SCE1572_11534	sce_10381	1.00E-119	4.00E-127
SCE1572_11535	sce_10384	0.00E+00	0.00E+00
SCE1572_11536	sce_10385	0.00E+00	0.00E+00
SCE1572_11537	sce_10386	5.00E-129	1.00E-121
SCE1572_11538	sce_10387	6.00E-92	2.00E-91
SCE1572_11539	sce_10388	5.00E-95	3.00E-99
SCE1572_11540	sce_10389	2.00E-91	5.00E-85
SCE1572_11541	sce_10390	2.00E-76	1.00E-68
SCE1572_11542	sce_10391	3.00E-125	2.00E-121
SCE1572_11543	sce_10392	6.00E-175	0.00E+00
SCE1572_11544	sce_10393	9.00E-131	1.00E-126
SCE1572_11545	sce_10394	0.00E+00	0.00E+00
SCE1572_11546	sce_10396	1.00E-130	6.00E-124
SCE1572_11548	sce_10397	3.00E-99	3.00E-99
SCE1572_11549	sce_10398	1.00E-156	2.00E-158

SCE1572_11553	sce_10402	0.00E+00	0.00E+00
SCE1572_11554	sce_10403	3.00E-114	1.00E-117
SCE1572_11555	sce_6028	2.00E-173	0.00E+00
SCE1572_11564	sce_10405	2.00E-72	8.00E-69
SCE1572_11565	sce_8641	2.00E-06	2.00E-12
SCE1572_11569	sce_167	2.00E-79	3.00E-78
SCE1572_11571	sce_7925	5.00E-89	4.00E-101
SCE1572_11574	sce_9347	0.00E+00	0.00E+00
SCE1572_11575	sce_10410	3.00E-163	3.00E-163
SCE1572_11576	sce_8743	1.00E-77	1.00E-77
SCE1572_11577	sce_10411	2.00E-153	2.00E-145
SCE1572_11578	sce_10415	7.00E-62	3.00E-62
SCE1572_11579	sce_10416	4.00E-147	9.00E-133
SCE1572_11580	sce_10417	4.00E-64	2.00E-69
SCE1572_11581	sce_10418	8.00E-165	5.00E-158
SCE1572_11582	sce_10419	0.00E+00	0.00E+00
SCE1572_11583	sce_10447	0.00E+00	0.00E+00
SCE1572_11584	sce_10448	1.00E-149	3.00E-150
SCE1572_11586	sce_10450	0.00E+00	0.00E+00
SCE1572_11588	sce_10452	2.00E-150	2.00E-148
SCE1572_11589	sce_10454	3.00E-98	1.00E-106
SCE1572_11590	sce_10455	1.00E-153	1.00E-153
SCE1572_11593	sce_10456	1.00E-97	1.00E-97
SCE1572_11594	sce_10457	3.00E-133	7.00E-140
SCE1572_11595	sce_10458	0.00E+00	0.00E+00
SCE1572_11596	sce_10459	7.00E-83	7.00E-83
SCE1572_11597	sce_10460	1.00E-102	4.00E-104
SCE1572_11598	sce_10461	9.00E-76	1.00E-74
SCE1572_11599	sce_10462	4.00E-119	2.00E-134
SCE1572_116	sce_104	2.00E-145	1.00E-144
SCE1572_11600	sce_10463	1.00E-90	1.00E-90
SCE1572_11601	sce_10464	1.00E-111	4.00E-96
SCE1572_1164	sce_854	6.00E-32	2.00E-26
SCE1572_1165	sce_856	0.00E+00	0.00E+00
SCE1572_1166	sce_857	0.00E+00	0.00E+00
SCE1572_1168	sce_4696	0.00E+00	0.00E+00
SCE1572_1169	sce_858	0.00E+00	0.00E+00
SCE1572_117	sce_105	3.00E-135	4.00E-126
SCE1572_1175	sce_5251	3.00E-84	2.00E-84
SCE1572_1176	sce_2435	1.00E-86	4.00E-87
SCE1572_1177	sce_860	9.00E-128	1.00E-127
SCE1572_1178	sce_2432	9.00E-117	5.00E-104
SCE1572_1179	sce_2431	3.00E-132	6.00E-128
SCE1572_118	sce_106	6.00E-80	6.00E-80
SCE1572_1180	sce_2430	3.00E-88	2.00E-85
SCE1572_1181	sce_5268	1.00E-57	1.00E-55
SCE1572_1184	sce_5086	0.00E+00	0.00E+00
SCE1572_1184	sce_5085	0.00E+00	0.00E+00
SCE1572_1184	sce_6701	0.00E+00	0.00E+00
SCE1572_1184	sce_3793	0.00E+00	0.00E+00
SCE1572_1185	sce_2391	6.00E-104	3.00E-100
SCE1572_1186	sce_862	2.00E-121	2.00E-121
SCE1572_1187	sce_863	2.00E-35	9.00E-37
SCE1572_1188	sce_9528	3.00E-59	2.00E-63
SCE1572_1189	sce_202	1.00E-150	1.00E-151
SCE1572_119	sce_107	6.00E-150	6.00E-150
SCE1572_1190	sce_1118	0.00E+00	0.00E+00
SCE1572_1192	sce_866	0.00E+00	0.00E+00
SCE1572_1193	sce_867	0.00E+00	0.00E+00
SCE1572_1197	sce_3917	0.00E+00	0.00E+00
SCE1572_1197	sce_6092	0.00E+00	0.00E+00
SCE1572_1197	sce_6107	0.00E+00	0.00E+00
SCE1572_1197	sce_6093	0.00E+00	0.00E+00
SCE1572_1197	sce_6094	0.00E+00	0.00E+00
SCE1572_1197	sce_6095	0.00E+00	0.00E+00
SCE1572_1197	sce_7661	0.00E+00	0.00E+00
SCE1572_1197	sce_388	0.00E+00	0.00E+00
SCE1572_1197	sce_9920	0.00E+00	0.00E+00
SCE1572_1197	sce_6535	0.00E+00	0.00E+00
SCE1572_1197	sce_3003	0.00E+00	0.00E+00
SCE1572_1197	sce_2065	0.00E+00	0.00E+00
SCE1572_1197	sce_10204	0.00E+00	0.00E+00
SCE1572_1197	sce_8602	0.00E+00	0.00E+00
SCE1572_1197	sce_7640	0.00E+00	0.00E+00
SCE1572_1197	sce_9557	0.00E+00	0.00E+00
SCE1572_1197	sce_4513	0.00E+00	0.00E+00

SCE1572_1197	sce_5835	0.00E+00	0.00E+00
SCE1572_1198	sce_868	0.00E+00	0.00E+00
SCE1572_12	sce_12	3.00E-103	9.00E-99
SCE1572_120	sce_108	3.00E-133	5.00E-138
SCE1572_1200	sce_837	4.00E-104	2.00E-99
SCE1572_1202	sce_869	1.00E-75	1.00E-78
SCE1572_1203	sce_870	6.00E-89	5.00E-69
SCE1572_1207	sce_871	0.00E+00	0.00E+00
SCE1572_1208	sce_872	0.00E+00	0.00E+00
SCE1572_1209	sce_873	2.00E-82	6.00E-77
SCE1572_121	sce_109	1.00E-105	5.00E-113
SCE1572_1211	sce_874	3.00E-56	2.00E-49
SCE1572_1212	sce_875	5.00E-51	3.00E-59
SCE1572_1216	sce_2569	5.00E-23	9.00E-22
SCE1572_1219	sce_879	6.00E-97	8.00E-95
SCE1572_122	sce_110	2.00E-167	3.00E-167
SCE1572_1222	sce_881	3.00E-34	3.00E-45
SCE1572_1230	sce_4062	0.00E+00	0.00E+00
SCE1572_1230	sce_9373	0.00E+00	0.00E+00
SCE1572_1231	sce_885	0.00E+00	0.00E+00
SCE1572_1233	sce_895	0.00E+00	0.00E+00
SCE1572_1234	sce_896	0.00E+00	0.00E+00
SCE1572_1236	sce_898	0.00E+00	0.00E+00
SCE1572_1242	sce_900	0.00E+00	0.00E+00
SCE1572_1243	sce_9368	1.00E-08	2.00E-15
SCE1572_1245	sce_5041	3.00E-29	3.00E-33
SCE1572_1248	sce_901	4.00E-76	2.00E-76
SCE1572_125	sce_125	7.00E-49	5.00E-50
SCE1572_1250	sce_1317	7.00E-17	8.00E-17
SCE1572_1251	sce_1318	1.00E-22	1.00E-28
SCE1572_1252	sce_902	0.00E+00	0.00E+00
SCE1572_1253	sce_903	2.00E-87	2.00E-87
SCE1572_1254	sce_904	2.00E-60	2.00E-60
SCE1572_1255	sce_7640	0.00E+00	0.00E+00
SCE1572_1255	sce_5835	0.00E+00	0.00E+00
SCE1572_1255	sce_388	0.00E+00	0.00E+00
SCE1572_1255	sce_10204	0.00E+00	0.00E+00
SCE1572_1255	sce_4513	0.00E+00	0.00E+00
SCE1572_1257	sce_846	0.00E+00	0.00E+00
SCE1572_1258	sce_1687	3.00E-90	3.00E-90
SCE1572_1259	sce_1688	7.00E-43	7.00E-43
SCE1572_126	sce_127	2.00E-153	4.00E-175
SCE1572_1260	sce_1689	0.00E+00	0.00E+00
SCE1572_1261	sce_1690	9.00E-159	3.00E-148
SCE1572_1262	sce_10368	5.00E-122	3.00E-106
SCE1572_1263	sce_909	0.00E+00	0.00E+00
SCE1572_1265	sce_910	5.00E-46	4.00E-46
SCE1572_1266	sce_911	7.00E-19	7.00E-19
SCE1572_1268	sce_916	4.00E-176	0.00E+00
SCE1572_1269	sce_917	4.00E-145	5.00E-150
SCE1572_127	sce_128	4.00E-80	4.00E-96
SCE1572_1270	sce_1557	2.00E-153	2.00E-162
SCE1572_1271	sce_1556	7.00E-170	3.00E-162
SCE1572_1272	sce_1553	4.00E-15	2.00E-06
SCE1572_1273	sce_1552	0.00E+00	0.00E+00
SCE1572_1274	sce_1551	8.00E-69	6.00E-62
SCE1572_1275	sce_1550	6.00E-67	6.00E-67
SCE1572_1276	sce_1549	0.00E+00	0.00E+00
SCE1572_1280	sce_1493	2.00E-147	2.00E-146
SCE1572_1281	sce_919	1.00E-178	0.00E+00
SCE1572_1284	sce_920	0.00E+00	0.00E+00
SCE1572_1285	sce_921	0.00E+00	0.00E+00
SCE1572_1286	sce_922	0.00E+00	0.00E+00
SCE1572_1287	sce_923	1.00E-74	5.00E-72
SCE1572_1288	sce_924	2.00E-154	1.00E-150
SCE1572_1290	sce_925	4.00E-131	6.00E-134
SCE1572_1291	sce_926	1.00E-97	2.00E-90
SCE1572_1292	sce_927	0.00E+00	0.00E+00
SCE1572_1293	sce_928	1.00E-165	5.00E-172
SCE1572_1294	sce_929	3.00E-126	7.00E-154
SCE1572_1295	sce_930	5.00E-142	3.00E-136
SCE1572_1297	sce_931	2.00E-64	1.00E-70
SCE1572_1298	sce_932	4.00E-27	4.00E-27
SCE1572_1299	sce_933	9.00E-142	4.00E-132
SCE1572_13	sce_13	0.00E+00	0.00E+00
SCE1572_1300	sce_934	7.00E-155	2.00E-155

SCE1572_1301	sce_936	0.00E+00	0.00E+00
SCE1572_1302	sce_937	3.00E-81	4.00E-91
SCE1572_1303	sce_938	3.00E-98	4.00E-101
SCE1572_1304	sce_939	0.00E+00	0.00E+00
SCE1572_1305	sce_940	2.00E-31	2.00E-30
SCE1572_1306	sce_942	5.00E-72	5.00E-74
SCE1572_1307	sce_943	6.00E-80	6.00E-80
SCE1572_1308	sce_944	0.00E+00	0.00E+00
SCE1572_1309	sce_945	0.00E+00	0.00E+00
SCE1572_1309	sce_457	0.00E+00	0.00E+00
SCE1572_131	sce_129	4.00E-67	6.00E-62
SCE1572_1310	sce_946	1.00E-54	1.00E-54
SCE1572_1311	sce_947	2.00E-107	3.00E-99
SCE1572_1312	sce_948	8.00E-51	8.00E-51
SCE1572_1313	sce_949	1.00E-152	1.00E-152
SCE1572_1314	sce_950	1.00E-52	1.00E-44
SCE1572_1315	sce_951	2.00E-97	7.00E-113
SCE1572_1316	sce_952	0.00E+00	0.00E+00
SCE1572_1318	sce_953	1.00E-72	2.00E-71
SCE1572_1319	sce_954	4.00E-152	2.00E-136
SCE1572_132	sce_130	0.00E+00	0.00E+00
SCE1572_1320	sce_956	0.00E+00	0.00E+00
SCE1572_1321	sce_957	4.00E-159	2.00E-150
SCE1572_1323	sce_959	0.00E+00	8.00E-179
SCE1572_1324	sce_960	1.00E-76	3.00E-76
SCE1572_1329	sce_8257	9.00E-14	6.00E-18
SCE1572_133	sce_131	0.00E+00	0.00E+00
SCE1572_1337	sce_961	0.00E+00	0.00E+00
SCE1572_1338	sce_962	0.00E+00	0.00E+00
SCE1572_1339	sce_963	0.00E+00	0.00E+00
SCE1572_1341	sce_965	2.00E-138	2.00E-138
SCE1572_1342	sce_967	7.00E-164	7.00E-164
SCE1572_1343	sce_968	8.00E-65	1.00E-56
SCE1572_1344	sce_969	0.00E+00	7.00E-179
SCE1572_1345	sce_970	3.00E-180	0.00E+00
SCE1572_1346	sce_971	1.00E-83	5.00E-79
SCE1572_1347	sce_972	0.00E+00	0.00E+00
SCE1572_1348	sce_973	9.00E-166	8.00E-159
SCE1572_135	sce_9966	4.00E-120	3.00E-120
SCE1572_1350	sce_974	1.00E-64	4.00E-65
SCE1572_1351	sce_975	2.00E-166	2.00E-166
SCE1572_1352	sce_976	0.00E+00	0.00E+00
SCE1572_1353	sce_978	3.00E-113	2.00E-109
SCE1572_1354	sce_979	3.00E-146	6.00E-165
SCE1572_1355	sce_980	6.00E-156	8.00E-162
SCE1572_1356	sce_981	6.00E-102	2.00E-99
SCE1572_1357	sce_982	0.00E+00	0.00E+00
SCE1572_1358	sce_984	0.00E+00	0.00E+00
SCE1572_1359	sce_985	0.00E+00	0.00E+00
SCE1572_136	sce_9965	6.00E-152	1.00E-150
SCE1572_1360	sce_986	0.00E+00	0.00E+00
SCE1572_1361	sce_987	0.00E+00	0.00E+00
SCE1572_1362	sce_988	0.00E+00	0.00E+00
SCE1572_1363	sce_989	9.00E-43	9.00E-43
SCE1572_1364	sce_990	4.00E-97	4.00E-97
SCE1572_1365	sce_991	1.00E-122	3.00E-129
SCE1572_1366	sce_992	0.00E+00	0.00E+00
SCE1572_1367	sce_993	1.00E-151	9.00E-159
SCE1572_1368	sce_994	0.00E+00	0.00E+00
SCE1572_1369	sce_995	0.00E+00	0.00E+00
SCE1572_137	sce_133	0.00E+00	0.00E+00
SCE1572_1370	sce_996	0.00E+00	0.00E+00
SCE1572_1371	sce_998	0.00E+00	0.00E+00
SCE1572_1372	sce_999	1.00E-62	1.00E-62
SCE1572_1374	sce_1000	0.00E+00	0.00E+00
SCE1572_1375	sce_1002	0.00E+00	0.00E+00
SCE1572_1377	sce_5241	7.00E-63	1.00E-77
SCE1572_1379	sce_1428	1.00E-37	6.00E-38
SCE1572_1380	sce_4696	0.00E+00	0.00E+00
SCE1572_1381	sce_6188	0.00E+00	0.00E+00
SCE1572_1384	sce_8656	0.00E+00	0.00E+00
SCE1572_1385	sce_6414	4.00E-133	5.00E-132
SCE1572_1387	sce_9995	0.00E+00	0.00E+00
SCE1572_1389	sce_1692	2.00E-26	2.00E-26
SCE1572_1390	sce_4602	2.00E-163	1.00E-160
SCE1572_1392	sce_1004	8.00E-130	1.00E-132

SCE1572_1393	sce_1005	3.00E-168	2.00E-178
SCE1572_1394	sce_6012	4.00E-39	2.00E-40
SCE1572_1395	sce_1006	2.00E-179	8.00E-172
SCE1572_1396	sce_1007	2.00E-93	7.00E-107
SCE1572_1397	sce_1333	2.00E-38	8.00E-42
SCE1572_1398	sce_1331	4.00E-53	1.00E-49
SCE1572_1399	sce_1330	6.00E-40	2.00E-41
SCE1572_14	sce_14	0.00E+00	0.00E+00
SCE1572_1400	sce_1341	1.00E-54	1.00E-65
SCE1572_1401	sce_1340	1.00E-135	7.00E-134
SCE1572_1405	sce_1339	1.00E-158	2.00E-128
SCE1572_1406	sce_1338	0.00E+00	0.00E+00
SCE1572_1407	sce_1337	1.00E-157	3.00E-157
SCE1572_1408	sce_1336	1.00E-80	4.00E-85
SCE1572_1409	sce_1335	0.00E+00	0.00E+00
SCE1572_1410	sce_1334	1.00E-111	9.00E-121
SCE1572_1411	sce_1008	0.00E+00	0.00E+00
SCE1572_1412	sce_1009	6.00E-140	1.00E-136
SCE1572_1413	sce_1010	3.00E-47	2.00E-48
SCE1572_1414	sce_1011	9.00E-30	5.00E-36
SCE1572_1416	sce_5962	0.00E+00	0.00E+00
SCE1572_1417	sce_4679	2.00E-39	3.00E-39
SCE1572_1418	sce_4680	2.00E-37	2.00E-37
SCE1572_1423	sce_1015	0.00E+00	0.00E+00
SCE1572_1424	sce_3601	1.00E-119	5.00E-113
SCE1572_1425	sce_3600	1.00E-150	8.00E-154
SCE1572_1426	sce_1017	1.00E-128	1.00E-128
SCE1572_1428	sce_1018	1.00E-158	2.00E-151
SCE1572_1429	sce_1019	2.00E-122	2.00E-122
SCE1572_1430	sce_1022	5.00E-122	2.00E-129
SCE1572_1431	sce_1023	2.00E-31	2.00E-31
SCE1572_1432	sce_1024	4.00E-30	1.00E-67
SCE1572_1434	sce_8817	0.00E+00	0.00E+00
SCE1572_1434	sce_10193	0.00E+00	0.00E+00
SCE1572_1435	sce_4560	2.00E-25	5.00E-29
SCE1572_1437	sce_6320	6.00E-68	6.00E-68
SCE1572_1438	sce_6321	1.00E-143	7.00E-148
SCE1572_1439	sce_6322	2.00E-161	4.00E-152
SCE1572_144	sce_1298	3.00E-11	3.00E-11
SCE1572_1440	sce_6323	0.00E+00	0.00E+00
SCE1572_1441	sce_6330	0.00E+00	0.00E+00
SCE1572_1442	sce_6326	2.00E-108	4.00E-106
SCE1572_1443	sce_1084	0.00E+00	0.00E+00
SCE1572_1445	sce_2350	0.00E+00	0.00E+00
SCE1572_1447	sce_6187	0.00E+00	0.00E+00
SCE1572_1448	sce_2658	7.00E-43	5.00E-49
SCE1572_1449	sce_2659	4.00E-48	4.00E-48
SCE1572_145	sce_6050	5.00E-46	6.00E-53
SCE1572_1450	sce_9885	3.00E-157	3.00E-157
SCE1572_1451	sce_9884	4.00E-149	5.00E-144
SCE1572_1453	sce_1676	0.00E+00	0.00E+00
SCE1572_1455	sce_7521	0.00E+00	0.00E+00
SCE1572_1459	sce_8592	0.00E+00	0.00E+00
SCE1572_1459	sce_3937	0.00E+00	0.00E+00
SCE1572_146	sce_6051	0.00E+00	0.00E+00
SCE1572_1463	sce_9794	3.00E-123	3.00E-127
SCE1572_1464	sce_9795	6.00E-115	1.00E-131
SCE1572_1465	sce_7514	6.00E-79	2.00E-82
SCE1572_147	sce_6052	0.00E+00	0.00E+00
SCE1572_149	sce_1469	4.00E-70	5.00E-62
SCE1572_1492	sce_7318	0.00E+00	0.00E+00
SCE1572_1492	sce_5158	0.00E+00	0.00E+00
SCE1572_1492	sce_1116	0.00E+00	0.00E+00
SCE1572_1494	sce_2498	0.00E+00	0.00E+00
SCE1572_1496	sce_8802	1.00E-60	3.00E-52
SCE1572_1497	sce_9879	1.00E-69	3.00E-68
SCE1572_15	sce_15	4.00E-25	1.00E-19
SCE1572_150	sce_138	8.00E-85	2.00E-97
SCE1572_1504	sce_10169	0.00E+00	0.00E+00
SCE1572_1511	sce_7061	5.00E-83	6.00E-73
SCE1572_1512	sce_9866	2.00E-141	6.00E-137
SCE1572_1514	sce_1037	2.00E-100	3.00E-121
SCE1572_1515	sce_1039	2.00E-16	1.00E-16
SCE1572_1516	sce_1040	0.00E+00	0.00E+00
SCE1572_1518	sce_1041	0.00E+00	0.00E+00
SCE1572_1521	sce_1044	5.00E-63	3.00E-58

SCE1572_1523	sce_1046	3.00E-106	3.00E-99
SCE1572_1525	sce_1047	0.00E+00	0.00E+00
SCE1572_1526	sce_1048	0.00E+00	0.00E+00
SCE1572_1527	sce_1049	3.00E-20	2.00E-20
SCE1572_1528	sce_1051	0.00E+00	0.00E+00
SCE1572_1529	sce_1052	0.00E+00	0.00E+00
SCE1572_1532	sce_1053	2.00E-59	4.00E-61
SCE1572_1533	sce_1054	1.00E-75	7.00E-92
SCE1572_1534	sce_1056	1.00E-119	3.00E-115
SCE1572_1535	sce_1057	4.00E-79	8.00E-93
SCE1572_1538	sce_1058	1.00E-152	7.00E-153
SCE1572_1539	sce_1059	5.00E-166	6.00E-163
SCE1572_1540	sce_1060	0.00E+00	0.00E+00
SCE1572_1543	sce_1061	7.00E-13	7.00E-18
SCE1572_1544	sce_1063	5.00E-79	5.00E-79
SCE1572_1545	sce_1065	0.00E+00	0.00E+00
SCE1572_1546	sce_1066	0.00E+00	0.00E+00
SCE1572_1547	sce_1071	2.00E-84	1.00E-84
SCE1572_1548	sce_1074	0.00E+00	0.00E+00
SCE1572_1549	sce_1075	4.00E-142	3.00E-140
SCE1572_155	sce_140	0.00E+00	0.00E+00
SCE1572_1550	sce_1076	6.00E-45	4.00E-46
SCE1572_1551	sce_1078	3.00E-163	7.00E-140
SCE1572_1552	sce_9982	9.00E-78	1.00E-97
SCE1572_1553	sce_1080	4.00E-52	3.00E-70
SCE1572_1554	sce_1081	0.00E+00	0.00E+00
SCE1572_1556	sce_4904	3.00E-52	5.00E-53
SCE1572_1558	sce_1087	0.00E+00	0.00E+00
SCE1572_1559	sce_1088	2.00E-58	2.00E-60
SCE1572_156	sce_141	4.00E-133	4.00E-133
SCE1572_1560	sce_1090	4.00E-112	1.00E-92
SCE1572_1561	sce_1091	2.00E-124	9.00E-126
SCE1572_1562	sce_1092	4.00E-49	2.00E-49
SCE1572_1563	sce_1094	4.00E-27	4.00E-27
SCE1572_1564	sce_1095	6.00E-71	1.00E-70
SCE1572_1567	sce_1096	3.00E-18	1.00E-16
SCE1572_1568	sce_1097	0.00E+00	0.00E+00
SCE1572_1569	sce_1098	6.00E-132	1.00E-132
SCE1572_157	sce_5982	0.00E+00	0.00E+00
SCE1572_1571	sce_1122	7.00E-26	2.00E-32
SCE1572_1572	sce_1123	0.00E+00	0.00E+00
SCE1572_1573	sce_1124	4.00E-176	2.00E-178
SCE1572_1574	sce_1125	1.00E-99	3.00E-99
SCE1572_1575	sce_1126	2.00E-150	4.00E-150
SCE1572_1576	sce_1127	2.00E-180	9.00E-160
SCE1572_1577	sce_1128	2.00E-152	5.00E-155
SCE1572_158	sce_5981	7.00E-84	1.00E-104
SCE1572_1581	sce_1132	2.00E-111	1.00E-110
SCE1572_1582	sce_1133	2.00E-121	2.00E-125
SCE1572_1584	sce_1604	8.00E-95	8.00E-95
SCE1572_1585	sce_7900	1.00E-95	1.00E-95
SCE1572_159	sce_5980	0.00E+00	0.00E+00
SCE1572_16	sce_16	6.00E-160	3.00E-162
SCE1572_1600	sce_8426	0.00E+00	0.00E+00
SCE1572_1604	sce_1140	1.00E-37	8.00E-46
SCE1572_1605	sce_1143	0.00E+00	0.00E+00
SCE1572_1606	sce_1144	0.00E+00	0.00E+00
SCE1572_1607	sce_1145	2.00E-108	7.00E-109
SCE1572_1608	sce_1146	0.00E+00	0.00E+00
SCE1572_1609	sce_1148	0.00E+00	0.00E+00
SCE1572_161	sce_8630	1.00E-133	1.00E-135
SCE1572_1610	sce_1149	0.00E+00	0.00E+00
SCE1572_1612	sce_1151	8.00E-99	3.00E-88
SCE1572_1614	sce_1152	0.00E+00	0.00E+00
SCE1572_1615	sce_1153	0.00E+00	0.00E+00
SCE1572_1616	sce_1154	1.00E-142	4.00E-128
SCE1572_1617	sce_1155	8.00E-137	1.00E-136
SCE1572_1618	sce_1156	0.00E+00	0.00E+00
SCE1572_1619	sce_1157	6.00E-105	6.00E-96
SCE1572_162	sce_8629	0.00E+00	0.00E+00
SCE1572_1620	sce_1158	1.00E-60	1.00E-60
SCE1572_1621	sce_1159	2.00E-60	3.00E-70
SCE1572_1625	sce_8406	5.00E-30	3.00E-31
SCE1572_1626	sce_1164	7.00E-83	1.00E-77
SCE1572_1627	sce_7633	0.00E+00	0.00E+00
SCE1572_1628	sce_1165	4.00E-145	7.00E-147

SCE1572_1629	sce_1166	1.00E-51	1.00E-43
SCE1572_1633	sce_1167	0.00E+00	0.00E+00
SCE1572_1634	sce_1168	0.00E+00	0.00E+00
SCE1572_1636	sce_1170	2.00E-99	2.00E-99
SCE1572_1640	sce_9109	5.00E-28	7.00E-26
SCE1572_1643	sce_8347	0.00E+00	0.00E+00
SCE1572_1645	sce_4581	0.00E+00	0.00E+00
SCE1572_1645	sce_9106	0.00E+00	0.00E+00
SCE1572_1649	sce_6171	0.00E+00	0.00E+00
SCE1572_165	sce_147	8.00E-131	3.00E-154
SCE1572_1651	sce_5536	9.00E-51	4.00E-51
SCE1572_1658	sce_1173	4.00E-80	1.00E-80
SCE1572_1659	sce_1174	7.00E-153	4.00E-137
SCE1572_1660	sce_1175	7.00E-168	3.00E-166
SCE1572_1661	sce_1176	1.00E-177	1.00E-177
SCE1572_1663	sce_1177	1.00E-09	1.00E-08
SCE1572_1664	sce_1178	1.00E-167	1.00E-167
SCE1572_1665	sce_1180	1.00E-165	3.00E-169
SCE1572_1666	sce_1181	0.00E+00	0.00E+00
SCE1572_1667	sce_1183	3.00E-99	1.00E-92
SCE1572_1668	sce_1184	5.00E-175	2.00E-180
SCE1572_1669	sce_1185	9.00E-43	1.00E-51
SCE1572_1670	sce_1186	0.00E+00	0.00E+00
SCE1572_1671	sce_1187	0.00E+00	0.00E+00
SCE1572_1672	sce_1188	4.00E-78	5.00E-88
SCE1572_1673	sce_1189	0.00E+00	0.00E+00
SCE1572_1674	sce_1190	4.00E-161	2.00E-148
SCE1572_1675	sce_1191	3.00E-89	2.00E-85
SCE1572_1677	sce_1192	4.00E-18	1.00E-24
SCE1572_1678	sce_1193	0.00E+00	0.00E+00
SCE1572_1679	sce_1194	0.00E+00	0.00E+00
SCE1572_168	sce_148	6.00E-105	1.00E-107
SCE1572_1680	sce_1195	3.00E-153	3.00E-153
SCE1572_1681	sce_1196	1.00E-62	8.00E-72
SCE1572_1682	sce_1197	1.00E-116	1.00E-108
SCE1572_1683	sce_1198	3.00E-43	3.00E-43
SCE1572_1684	sce_1199	3.00E-144	3.00E-144
SCE1572_1685	sce_1200	9.00E-87	8.00E-100
SCE1572_1686	sce_1201	8.00E-24	1.00E-20
SCE1572_1687	sce_1202	9.00E-79	5.00E-72
SCE1572_1688	sce_1203	1.00E-69	3.00E-69
SCE1572_169	sce_149	1.00E-138	6.00E-133
SCE1572_1690	sce_1204	3.00E-56	4.00E-50
SCE1572_1691	sce_1205	8.00E-71	9.00E-71
SCE1572_1692	sce_1206	6.00E-14	2.00E-21
SCE1572_1693	sce_1207	1.00E-32	4.00E-10
SCE1572_1696	sce_1209	9.00E-116	1.00E-107
SCE1572_1697	sce_1210	2.00E-17	2.00E-26
SCE1572_1698	sce_1211	8.00E-33	8.00E-42
SCE1572_1699	sce_1212	0.00E+00	0.00E+00
SCE1572_17	sce_17	1.00E-49	6.00E-51
SCE1572_170	sce_151	9.00E-71	2.00E-71
SCE1572_1700	sce_1213	8.00E-159	4.00E-154
SCE1572_1701	sce_1214	0.00E+00	0.00E+00
SCE1572_1702	sce_1215	3.00E-105	8.00E-112
SCE1572_1703	sce_1216	0.00E+00	0.00E+00
SCE1572_1705	sce_1218	0.00E+00	0.00E+00
SCE1572_1706	sce_1219	0.00E+00	0.00E+00
SCE1572_1707	sce_1221	3.00E-169	6.00E-171
SCE1572_1708	sce_1222	2.00E-25	2.00E-25
SCE1572_1709	sce_1223	0.00E+00	0.00E+00
SCE1572_171	sce_152	7.00E-39	7.00E-39
SCE1572_1710	sce_1224	4.00E-43	2.00E-40
SCE1572_1711	sce_1225	2.00E-162	5.00E-169
SCE1572_1712	sce_1226	6.00E-149	3.00E-159
SCE1572_1713	sce_1227	2.00E-84	5.00E-85
SCE1572_1717	sce_1230	3.00E-154	5.00E-161
SCE1572_1718	sce_1231	0.00E+00	0.00E+00
SCE1572_1719	sce_1232	0.00E+00	0.00E+00
SCE1572_1720	sce_1233	3.00E-111	2.00E-103
SCE1572_1722	sce_1235	0.00E+00	0.00E+00
SCE1572_1724	sce_1236	5.00E-135	8.00E-135
SCE1572_1725	sce_1237	0.00E+00	0.00E+00
SCE1572_1727	sce_1238	0.00E+00	0.00E+00
SCE1572_1728	sce_1240	0.00E+00	6.00E-129
SCE1572_173	sce_153	2.00E-24	2.00E-35

SCE1572_1731	sce_9222	0.00E+00	0.00E+00
SCE1572_1731	sce_1814	0.00E+00	0.00E+00
SCE1572_1739	sce_1242	3.00E-116	1.00E-104
SCE1572_174	sce_154	2.00E-98	4.00E-88
SCE1572_1740	sce_1243	1.00E-105	3.00E-121
SCE1572_175	sce_155	8.00E-118	1.00E-134
SCE1572_176	sce_156	8.00E-32	3.00E-32
SCE1572_1761	sce_1670	1.00E-107	1.00E-107
SCE1572_1762	sce_1116	0.00E+00	0.00E+00
SCE1572_1762	sce_5158	0.00E+00	0.00E+00
SCE1572_1762	sce_7318	0.00E+00	0.00E+00
SCE1572_1763	sce_1115	0.00E+00	0.00E+00
SCE1572_1763	sce_5159	0.00E+00	0.00E+00
SCE1572_1768	sce_5098	0.00E+00	0.00E+00
SCE1572_177	sce_7124	8.00E-38	4.00E-37
SCE1572_1771	sce_1085	5.00E-179	7.00E-180
SCE1572_1772	sce_1086	0.00E+00	0.00E+00
SCE1572_1774	sce_5484	1.00E-74	4.00E-76
SCE1572_1775	sce_5485	2.00E-09	4.00E-17
SCE1572_1776	sce_5486	0.00E+00	0.00E+00
SCE1572_1776	sce_1479	0.00E+00	0.00E+00
SCE1572_1777	sce_5487	0.00E+00	0.00E+00
SCE1572_1777	sce_1478	0.00E+00	0.00E+00
SCE1572_1778	sce_8627	1.00E-99	4.00E-104
SCE1572_1780	sce_1565	1.00E-124	1.00E-124
SCE1572_1781	sce_1564	1.00E-142	5.00E-152
SCE1572_1782	sce_7064	5.00E-53	5.00E-53
SCE1572_1783	sce_7065	2.00E-70	2.00E-70
SCE1572_1785	sce_7067	0.00E+00	0.00E+00
SCE1572_179	sce_159	0.00E+00	0.00E+00
SCE1572_18	sce_18	0.00E+00	0.00E+00
SCE1572_1806	sce_1266	2.00E-87	2.00E-89
SCE1572_1807	sce_1267	1.00E-78	9.00E-49
SCE1572_1808	sce_1268	2.00E-111	3.00E-105
SCE1572_1809	sce_1269	6.00E-138	5.00E-137
SCE1572_181	sce_1372	0.00E+00	0.00E+00
SCE1572_1810	sce_5174	7.00E-92	5.00E-97
SCE1572_1811	sce_5175	2.00E-176	2.00E-171
SCE1572_1812	sce_1270	1.00E-130	5.00E-146
SCE1572_1813	sce_1271	2.00E-84	5.00E-75
SCE1572_1814	sce_1272	2.00E-147	3.00E-157
SCE1572_1815	sce_1273	3.00E-143	4.00E-180
SCE1572_1816	sce_1275	3.00E-80	2.00E-79
SCE1572_1818	sce_9112	4.00E-22	4.00E-22
SCE1572_1820	sce_1284	1.00E-61	2.00E-66
SCE1572_1824	sce_2457	0.00E+00	0.00E+00
SCE1572_1825	sce_2458	7.00E-101	1.00E-96
SCE1572_1829	sce_6076	0.00E+00	0.00E+00
SCE1572_183	sce_9533	0.00E+00	0.00E+00
SCE1572_1830	sce_1292	0.00E+00	0.00E+00
SCE1572_1832	sce_1293	3.00E-30	3.00E-36
SCE1572_1833	sce_1294	0.00E+00	0.00E+00
SCE1572_1834	sce_1295	1.00E-39	1.00E-42
SCE1572_1835	sce_1296	9.00E-71	6.00E-78
SCE1572_1836	sce_1297	3.00E-25	4.00E-25
SCE1572_1837	sce_1299	0.00E+00	0.00E+00
SCE1572_1838	sce_1300	0.00E+00	0.00E+00
SCE1572_1840	sce_1302	1.00E-141	3.00E-152
SCE1572_1841	sce_1303	6.00E-45	7.00E-50
SCE1572_1842	sce_1304	0.00E+00	0.00E+00
SCE1572_1843	sce_1305	5.00E-93	5.00E-93
SCE1572_1844	sce_1306	1.00E-177	0.00E+00
SCE1572_1846	sce_1308	2.00E-50	1.00E-46
SCE1572_1849	sce_1492	0.00E+00	0.00E+00
SCE1572_185	sce_7864	1.00E-85	1.00E-96
SCE1572_1850	sce_1491	0.00E+00	0.00E+00
SCE1572_1851	sce_1490	6.00E-47	4.00E-47
SCE1572_1852	sce_1488	2.00E-136	7.00E-136
SCE1572_1853	sce_1487	2.00E-98	5.00E-99
SCE1572_1855	sce_1309	0.00E+00	0.00E+00
SCE1572_1856	sce_1310	0.00E+00	0.00E+00
SCE1572_1858	sce_1312	5.00E-51	5.00E-51
SCE1572_1859	sce_1313	2.00E-35	1.00E-47
SCE1572_186	sce_7863	6.00E-126	5.00E-136
SCE1572_1860	sce_1315	0.00E+00	0.00E+00
SCE1572_1862	sce_3679	2.00E-179	0.00E+00

SCE1572_1866	sce_6298	7.00E-12	3.00E-08
SCE1572_1869	sce_1342	1.00E-157	3.00E-162
SCE1572_1870	sce_1344	1.00E-62	1.00E-87
SCE1572_1871	sce_3671	1.00E-93	3.00E-93
SCE1572_1872	sce_1345	0.00E+00	0.00E+00
SCE1572_1874	sce_1390	3.00E-87	2.00E-83
SCE1572_1875	sce_1391	0.00E+00	0.00E+00
SCE1572_1876	sce_1392	0.00E+00	0.00E+00
SCE1572_1876	sce_5578	0.00E+00	0.00E+00
SCE1572_1878	sce_1394	6.00E-15	4.00E-19
SCE1572_1879	sce_1395	9.00E-66	1.00E-66
SCE1572_188	sce_171	1.00E-117	9.00E-127
SCE1572_1880	sce_1396	0.00E+00	0.00E+00
SCE1572_1881	sce_1397	4.00E-71	8.00E-67
SCE1572_1882	sce_1398	0.00E+00	0.00E+00
SCE1572_1882	sce_7661	0.00E+00	0.00E+00
SCE1572_1884	sce_1399	3.00E-113	2.00E-102
SCE1572_1885	sce_1401	3.00E-128	5.00E-157
SCE1572_1887	sce_1402	0.00E+00	0.00E+00
SCE1572_1889	sce_1404	0.00E+00	0.00E+00
SCE1572_189	sce_172	5.00E-57	1.00E-53
SCE1572_1891	sce_1405	3.00E-06	6.00E-06
SCE1572_1892	sce_1406	4.00E-155	2.00E-159
SCE1572_1893	sce_1407	3.00E-70	4.00E-71
SCE1572_1894	sce_1408	0.00E+00	3.00E-180
SCE1572_1895	sce_1409	7.00E-67	5.00E-98
SCE1572_1898	sce_1410	0.00E+00	0.00E+00
SCE1572_1899	sce_1411	1.00E-148	1.00E-166
SCE1572_19	sce_19	0.00E+00	0.00E+00
SCE1572_190	sce_3728	5.00E-157	3.00E-167
SCE1572_1902	sce_1414	0.00E+00	0.00E+00
SCE1572_1903	sce_1415	0.00E+00	0.00E+00
SCE1572_1905	sce_1416	3.00E-34	9.00E-30
SCE1572_1906	sce_1417	0.00E+00	0.00E+00
SCE1572_1907	sce_1418	0.00E+00	0.00E+00
SCE1572_1908	sce_1419	6.00E-106	4.00E-106
SCE1572_1914	sce_1421	7.00E-128	2.00E-136
SCE1572_1915	sce_1422	0.00E+00	0.00E+00
SCE1572_1917	sce_1436	5.00E-94	6.00E-97
SCE1572_1918	sce_1437	2.00E-107	5.00E-100
SCE1572_1919	sce_1438	0.00E+00	0.00E+00
SCE1572_1920	sce_1439	9.00E-134	2.00E-143
SCE1572_1922	sce_1440	5.00E-132	7.00E-150
SCE1572_1923	sce_1441	0.00E+00	1.00E-174
SCE1572_1924	sce_1442	0.00E+00	0.00E+00
SCE1572_1925	sce_1443	9.00E-16	1.00E-15
SCE1572_1926	sce_1444	0.00E+00	0.00E+00
SCE1572_1926	sce_2656	0.00E+00	0.00E+00
SCE1572_1927	sce_1445	0.00E+00	0.00E+00
SCE1572_1928	sce_1446	9.00E-119	7.00E-125
SCE1572_1929	sce_1447	8.00E-140	0.00E+00
SCE1572_1930	sce_1448	0.00E+00	0.00E+00
SCE1572_1931	sce_2577	0.00E+00	0.00E+00
SCE1572_1932	sce_1449	0.00E+00	0.00E+00
SCE1572_1934	sce_1450	1.00E-67	1.00E-67
SCE1572_1946	sce_5198	3.00E-26	2.00E-23
SCE1572_1948	sce_1316	3.00E-79	7.00E-72
SCE1572_1950	sce_848	1.00E-89	9.00E-93
SCE1572_1951	sce_849	2.00E-57	7.00E-65
SCE1572_1952	sce_850	1.00E-60	4.00E-59
SCE1572_1953	sce_1455	5.00E-86	5.00E-86
SCE1572_1954	sce_6015	2.00E-90	2.00E-90
SCE1572_1955	sce_1456	9.00E-78	9.00E-78
SCE1572_1956	sce_1457	2.00E-81	1.00E-116
SCE1572_1957	sce_6060	2.00E-44	5.00E-38
SCE1572_1958	sce_2565	1.00E-115	8.00E-114
SCE1572_1960	sce_1462	0.00E+00	0.00E+00
SCE1572_1961	sce_1463	0.00E+00	0.00E+00
SCE1572_1962	sce_1464	0.00E+00	0.00E+00
SCE1572_1963	sce_1465	3.00E-112	4.00E-111
SCE1572_1964	sce_1466	5.00E-103	4.00E-103
SCE1572_1965	sce_1467	1.00E-132	1.00E-132
SCE1572_1966	sce_1468	1.00E-177	0.00E+00
SCE1572_1967	sce_1470	4.00E-55	2.00E-55
SCE1572_1969	sce_1471	4.00E-67	3.00E-71
SCE1572_1971	sce_1472	1.00E-83	4.00E-76

SCE1572_1972	sce_1473	0.00E+00	0.00E+00
SCE1572_1975	sce_1474	0.00E+00	0.00E+00
SCE1572_1976	sce_1475	0.00E+00	0.00E+00
SCE1572_199	sce_173	0.00E+00	0.00E+00
SCE1572_1993	sce_1476	1.00E-117	4.00E-108
SCE1572_1994	sce_1477	0.00E+00	0.00E+00
SCE1572_1995	sce_1478	0.00E+00	0.00E+00
SCE1572_1995	sce_5487	0.00E+00	0.00E+00
SCE1572_1996	sce_1479	0.00E+00	0.00E+00
SCE1572_1996	sce_5486	0.00E+00	0.00E+00
SCE1572_1997	sce_1480	2.00E-14	8.00E-11
SCE1572_1998	sce_1481	1.00E-78	1.00E-80
SCE1572_1999	sce_1482	2.00E-57	6.00E-63
SCE1572_2	sce_2	0.00E+00	0.00E+00
SCE1572_20	sce_20	2.00E-158	0.00E+00
SCE1572_200	sce_6011	2.00E-59	8.00E-62
SCE1572_2000	sce_1483	2.00E-28	7.00E-29
SCE1572_2001	sce_1484	1.00E-92	8.00E-96
SCE1572_2002	sce_3672	5.00E-62	3.00E-61
SCE1572_2003	sce_1486	9.00E-65	1.00E-55
SCE1572_2005	sce_8025	2.00E-105	8.00E-98
SCE1572_2009	sce_1587	3.00E-16	1.00E-15
SCE1572_2014	sce_6082	1.00E-104	1.00E-85
SCE1572_2016	sce_1496	0.00E+00	0.00E+00
SCE1572_2017	sce_1497	0.00E+00	0.00E+00
SCE1572_2018	sce_1498	1.00E-74	3.00E-81
SCE1572_2019	sce_1499	0.00E+00	0.00E+00
SCE1572_2020	sce_1500	9.00E-88	1.00E-90
SCE1572_2022	sce_3705	0.00E+00	0.00E+00
SCE1572_2023	sce_5152	2.00E-133	7.00E-143
SCE1572_2024	sce_1510	0.00E+00	5.00E-176
SCE1572_2025	sce_1511	4.00E-168	4.00E-172
SCE1572_2026	sce_9276	9.00E-77	3.00E-75
SCE1572_2028	sce_1517	3.00E-75	3.00E-75
SCE1572_2029	sce_1512	2.00E-156	2.00E-156
SCE1572_203	sce_1460	6.00E-107	2.00E-108
SCE1572_2030	sce_8774	2.00E-14	3.00E-21
SCE1572_2031	sce_1513	4.00E-74	4.00E-74
SCE1572_2032	sce_1515	4.00E-54	7.00E-51
SCE1572_2033	sce_1676	0.00E+00	0.00E+00
SCE1572_2036	sce_1518	5.00E-49	7.00E-50
SCE1572_2038	sce_1519	8.00E-88	6.00E-91
SCE1572_2039	sce_1520	2.00E-35	2.00E-44
SCE1572_2040	sce_1521	5.00E-114	3.00E-114
SCE1572_2041	sce_1679	1.00E-70	6.00E-79
SCE1572_2042	sce_1522	3.00E-151	3.00E-137
SCE1572_2043	sce_1523	6.00E-103	1.00E-110
SCE1572_2045	sce_1527	0.00E+00	3.00E-174
SCE1572_2046	sce_1529	6.00E-131	9.00E-118
SCE1572_2048	sce_1530	1.00E-46	3.00E-55
SCE1572_205	sce_6005	0.00E+00	0.00E+00
SCE1572_2054	sce_1546	2.00E-127	1.00E-132
SCE1572_2055	sce_1567	7.00E-90	1.00E-75
SCE1572_2059	sce_1569	1.00E-138	3.00E-134
SCE1572_2064	sce_3848	2.00E-14	1.00E-14
SCE1572_2065	sce_1576	4.00E-173	2.00E-174
SCE1572_2066	sce_1577	0.00E+00	0.00E+00
SCE1572_2067	sce_1578	2.00E-156	8.00E-153
SCE1572_2069	sce_1579	0.00E+00	1.00E-141
SCE1572_2071	sce_1583	4.00E-25	3.00E-14
SCE1572_2072	sce_1588	0.00E+00	0.00E+00
SCE1572_2074	sce_1589	1.00E-71	1.00E-51
SCE1572_2075	sce_1590	1.00E-33	1.00E-31
SCE1572_2076	sce_1591	0.00E+00	0.00E+00
SCE1572_2077	sce_10225	3.00E-117	1.00E-127
SCE1572_2078	sce_7460	3.00E-128	3.00E-140
SCE1572_2080	sce_1592	7.00E-170	2.00E-177
SCE1572_2081	sce_1593	0.00E+00	0.00E+00
SCE1572_2082	sce_1594	1.00E-69	2.00E-66
SCE1572_2086	sce_1596	0.00E+00	0.00E+00
SCE1572_2089	sce_5892	7.00E-50	4.00E-45
SCE1572_2092	sce_7399	4.00E-55	9.00E-43
SCE1572_2095	sce_1609	4.00E-132	4.00E-133
SCE1572_2096	sce_1610	2.00E-53	5.00E-61
SCE1572_2097	sce_1611	1.00E-62	1.00E-62
SCE1572_2098	sce_1612	1.00E-113	7.00E-113

SCE1572_2099	sce_1613	3.00E-140	1.00E-144
SCE1572_21	sce_22	2.00E-28	1.00E-31
SCE1572_2100	sce_1614	2.00E-18	1.00E-29
SCE1572_2101	sce_1617	4.00E-70	2.00E-65
SCE1572_2102	sce_1618	1.00E-137	2.00E-137
SCE1572_2103	sce_1619	0.00E+00	0.00E+00
SCE1572_2104	sce_1620	1.00E-170	3.00E-177
SCE1572_2105	sce_1621	2.00E-133	3.00E-133
SCE1572_2106	sce_1622	8.00E-137	1.00E-142
SCE1572_2107	sce_1623	0.00E+00	0.00E+00
SCE1572_2108	sce_1624	1.00E-122	1.00E-147
SCE1572_2109	sce_1625	2.00E-47	3.00E-47
SCE1572_2110	sce_1626	4.00E-163	9.00E-163
SCE1572_2111	sce_1627	0.00E+00	0.00E+00
SCE1572_2114	sce_1629	2.00E-131	6.00E-138
SCE1572_2115	sce_1630	0.00E+00	0.00E+00
SCE1572_2117	sce_1631	0.00E+00	0.00E+00
SCE1572_2119	sce_1632	2.00E-87	6.00E-73
SCE1572_2120	sce_1633	0.00E+00	0.00E+00
SCE1572_2121	sce_1634	3.00E-34	3.00E-34
SCE1572_2123	sce_1635	2.00E-164	5.00E-160
SCE1572_2125	sce_1645	0.00E+00	0.00E+00
SCE1572_2126	sce_1646	6.00E-19	1.00E-25
SCE1572_2128	sce_1647	7.00E-155	2.00E-149
SCE1572_2129	sce_1648	1.00E-74	1.00E-84
SCE1572_2130	sce_1649	1.00E-36	1.00E-35
SCE1572_2131	sce_1650	8.00E-13	7.00E-14
SCE1572_2132	sce_1652	4.00E-110	5.00E-117
SCE1572_2136	sce_1655	6.00E-93	8.00E-93
SCE1572_2137	sce_1656	1.00E-169	5.00E-147
SCE1572_2139	sce_7075	4.00E-99	5.00E-131
SCE1572_214	sce_2177	6.00E-49	2.00E-55
SCE1572_2142	sce_1658	0.00E+00	0.00E+00
SCE1572_2143	sce_1666	0.00E+00	0.00E+00
SCE1572_2144	sce_1667	0.00E+00	0.00E+00
SCE1572_2145	sce_1668	0.00E+00	0.00E+00
SCE1572_2148	sce_841	0.00E+00	0.00E+00
SCE1572_215	sce_6003	3.00E-179	3.00E-179
SCE1572_2151	sce_1719	1.00E-127	7.00E-131
SCE1572_2152	sce_1720	3.00E-105	4.00E-105
SCE1572_2153	sce_1721	3.00E-95	3.00E-95
SCE1572_2154	sce_1722	1.00E-100	3.00E-101
SCE1572_2161	sce_389	0.00E+00	0.00E+00
SCE1572_2161	sce_2666	0.00E+00	0.00E+00
SCE1572_2161	sce_7637	0.00E+00	0.00E+00
SCE1572_2161	sce_9237	0.00E+00	0.00E+00
SCE1572_2163	sce_175	3.00E-136	3.00E-145
SCE1572_2170	sce_5759	0.00E+00	0.00E+00
SCE1572_2171	sce_5758	0.00E+00	0.00E+00
SCE1572_2174	sce_764	6.00E-167	2.00E-166
SCE1572_2176	sce_1727	2.00E-66	2.00E-85
SCE1572_2177	sce_1728	6.00E-133	4.00E-128
SCE1572_2178	sce_10158	3.00E-40	3.00E-52
SCE1572_2181	sce_1323	8.00E-105	2.00E-104
SCE1572_2184	sce_6334	3.00E-22	2.00E-33
SCE1572_2185	sce_9438	0.00E+00	0.00E+00
SCE1572_2187	sce_7422	0.00E+00	0.00E+00
SCE1572_219	sce_5881	7.00E-64	7.00E-64
SCE1572_2190	sce_2564	8.00E-80	8.00E-80
SCE1572_2191	sce_7916	0.00E+00	0.00E+00
SCE1572_2193	sce_3807	1.00E-78	3.00E-78
SCE1572_2195	sce_176	3.00E-69	7.00E-77
SCE1572_2196	sce_9450	6.00E-155	1.00E-149
SCE1572_2197	sce_3917	0.00E+00	0.00E+00
SCE1572_2197	sce_6094	0.00E+00	0.00E+00
SCE1572_2197	sce_6092	0.00E+00	0.00E+00
SCE1572_2197	sce_6107	0.00E+00	0.00E+00
SCE1572_2197	sce_6093	0.00E+00	0.00E+00
SCE1572_2197	sce_6095	0.00E+00	0.00E+00
SCE1572_2197	sce_7661	0.00E+00	0.00E+00
SCE1572_2197	sce_388	0.00E+00	0.00E+00
SCE1572_2197	sce_6535	0.00E+00	0.00E+00
SCE1572_2197	sce_9920	0.00E+00	0.00E+00
SCE1572_2197	sce_8602	0.00E+00	0.00E+00
SCE1572_2197	sce_10204	0.00E+00	0.00E+00
SCE1572_2197	sce_4513	0.00E+00	0.00E+00

SCE1572_2197	sce_3003	0.00E+00	0.00E+00
SCE1572_2197	sce_7640	0.00E+00	0.00E+00
SCE1572_2197	sce_5835	0.00E+00	0.00E+00
SCE1572_2197	sce_9557	0.00E+00	0.00E+00
SCE1572_2197	sce_3085	0.00E+00	0.00E+00
SCE1572_2199	sce_1746	0.00E+00	0.00E+00
SCE1572_22	sce_23	0.00E+00	0.00E+00
SCE1572_2200	sce_1747	3.00E-175	3.00E-175
SCE1572_2201	sce_1748	0.00E+00	0.00E+00
SCE1572_2202	sce_1749	7.00E-74	6.00E-82
SCE1572_2203	sce_1750	4.00E-149	2.00E-151
SCE1572_2204	sce_1751	3.00E-137	3.00E-129
SCE1572_2205	sce_1752	3.00E-36	3.00E-36
SCE1572_2206	sce_1753	2.00E-148	2.00E-141
SCE1572_2207	sce_1754	2.00E-58	1.00E-56
SCE1572_2209	sce_1755	1.00E-115	2.00E-121
SCE1572_2210	sce_1586	6.00E-24	5.00E-29
SCE1572_2212	sce_1756	3.00E-85	2.00E-53
SCE1572_2215	sce_1759	2.00E-141	5.00E-165
SCE1572_2216	sce_1760	6.00E-115	9.00E-123
SCE1572_2218	sce_1762	0.00E+00	0.00E+00
SCE1572_222	sce_10163	2.00E-124	2.00E-124
SCE1572_2222	sce_1765	3.00E-36	2.00E-29
SCE1572_2223	sce_1766	3.00E-119	8.00E-121
SCE1572_2226	sce_1775	4.00E-76	2.00E-64
SCE1572_2227	sce_1103	1.00E-118	1.00E-115
SCE1572_2228	sce_1776	0.00E+00	0.00E+00
SCE1572_2229	sce_1777	2.00E-64	7.00E-65
SCE1572_223	sce_9852	6.00E-109	2.00E-117
SCE1572_2230	sce_1778	0.00E+00	0.00E+00
SCE1572_2231	sce_1779	4.00E-95	4.00E-95
SCE1572_2232	sce_1780	2.00E-65	2.00E-65
SCE1572_2233	sce_1781	0.00E+00	0.00E+00
SCE1572_2234	sce_1782	2.00E-150	5.00E-133
SCE1572_2235	sce_1783	0.00E+00	4.00E-178
SCE1572_2236	sce_1784	0.00E+00	0.00E+00
SCE1572_2237	sce_1785	8.00E-56	9.00E-56
SCE1572_2238	sce_1786	3.00E-163	2.00E-172
SCE1572_2239	sce_1787	3.00E-125	7.00E-136
SCE1572_224	sce_9851	3.00E-115	1.00E-123
SCE1572_2242	sce_1794	2.00E-104	5.00E-114
SCE1572_2246	sce_1793	8.00E-33	5.00E-32
SCE1572_2249	sce_1790	3.00E-62	1.00E-67
SCE1572_2251	sce_1796	2.00E-35	7.00E-32
SCE1572_2252	sce_1797	3.00E-163	2.00E-162
SCE1572_2253	sce_1798	5.00E-156	2.00E-165
SCE1572_2254	sce_1799	2.00E-122	2.00E-150
SCE1572_2255	sce_1800	2.00E-80	2.00E-80
SCE1572_2256	sce_1801	0.00E+00	0.00E+00
SCE1572_2258	sce_1803	0.00E+00	0.00E+00
SCE1572_2258	sce_5403	0.00E+00	0.00E+00
SCE1572_2258	sce_5226	0.00E+00	0.00E+00
SCE1572_2258	sce_7154	0.00E+00	0.00E+00
SCE1572_2258	sce_7969	0.00E+00	0.00E+00
SCE1572_2258	sce_4073	0.00E+00	0.00E+00
SCE1572_2258	sce_9665	0.00E+00	0.00E+00
SCE1572_2258	sce_5751	0.00E+00	0.00E+00
SCE1572_2258	sce_2053	0.00E+00	0.00E+00
SCE1572_2258	sce_9954	0.00E+00	0.00E+00
SCE1572_2258	sce_9757	0.00E+00	0.00E+00
SCE1572_2258	sce_5746	0.00E+00	0.00E+00
SCE1572_2258	sce_3491	0.00E+00	0.00E+00
SCE1572_2259	sce_1804	3.00E-106	2.00E-98
SCE1572_226	sce_2494	1.00E-54	9.00E-50
SCE1572_2261	sce_1805	0.00E+00	0.00E+00
SCE1572_2262	sce_1806	0.00E+00	0.00E+00
SCE1572_2263	sce_1807	1.00E-141	8.00E-133
SCE1572_2264	sce_1808	7.00E-71	8.00E-64
SCE1572_2265	sce_1809	4.00E-91	5.00E-84
SCE1572_2266	sce_1810	2.00E-172	0.00E+00
SCE1572_2267	sce_1811	5.00E-125	2.00E-134
SCE1572_2268	sce_1812	0.00E+00	0.00E+00
SCE1572_2269	sce_1813	1.00E-151	3.00E-163
SCE1572_2270	sce_1814	0.00E+00	0.00E+00
SCE1572_2270	sce_9222	0.00E+00	0.00E+00
SCE1572_2270	sce_3148	0.00E+00	0.00E+00

SCE1572_2271	sce_1815	1.00E-163	4.00E-166
SCE1572_2272	sce_1817	0.00E+00	0.00E+00
SCE1572_2273	sce_1818	3.00E-45	5.00E-60
SCE1572_2274	sce_1819	8.00E-31	4.00E-39
SCE1572_2275	sce_1820	0.00E+00	0.00E+00
SCE1572_2276	sce_1821	0.00E+00	0.00E+00
SCE1572_2277	sce_1822	0.00E+00	0.00E+00
SCE1572_2278	sce_1823	4.00E-127	1.00E-134
SCE1572_2279	sce_1824	0.00E+00	0.00E+00
SCE1572_2280	sce_1826	0.00E+00	0.00E+00
SCE1572_2281	sce_1827	1.00E-100	5.00E-91
SCE1572_2282	sce_1828	1.00E-104	1.00E-108
SCE1572_2283	sce_1829	0.00E+00	0.00E+00
SCE1572_2284	sce_1831	4.00E-49	5.00E-49
SCE1572_2285	sce_1832	2.00E-38	2.00E-38
SCE1572_2286	sce_1833	4.00E-66	1.00E-65
SCE1572_2287	sce_1834	2.00E-68	2.00E-68
SCE1572_2288	sce_1835	6.00E-136	1.00E-126
SCE1572_2289	sce_1836	7.00E-27	7.00E-27
SCE1572_2290	sce_1837	3.00E-72	4.00E-82
SCE1572_2291	sce_1838	0.00E+00	0.00E+00
SCE1572_2292	sce_1840	2.00E-65	2.00E-65
SCE1572_2293	sce_1841	0.00E+00	2.00E-178
SCE1572_2294	sce_1842	0.00E+00	0.00E+00
SCE1572_2296	sce_1843	9.00E-162	2.00E-164
SCE1572_2297	sce_1844	3.00E-75	3.00E-48
SCE1572_2298	sce_1845	6.00E-71	5.00E-71
SCE1572_2299	sce_1846	2.00E-85	1.00E-85
SCE1572_23	sce_24	2.00E-130	2.00E-125
SCE1572_230	sce_3089	2.00E-21	1.00E-21
SCE1572_2300	sce_1847	9.00E-128	2.00E-122
SCE1572_2301	sce_1848	3.00E-32	3.00E-33
SCE1572_2302	sce_1849	0.00E+00	0.00E+00
SCE1572_2303	sce_1850	0.00E+00	0.00E+00
SCE1572_2304	sce_1851	5.00E-169	2.00E-170
SCE1572_2305	sce_1852	3.00E-168	2.00E-164
SCE1572_2306	sce_1853	5.00E-157	5.00E-162
SCE1572_2307	sce_1854	3.00E-118	1.00E-124
SCE1572_2308	sce_1855	0.00E+00	0.00E+00
SCE1572_2309	sce_1856	1.00E-121	1.00E-125
SCE1572_2310	sce_1857	0.00E+00	0.00E+00
SCE1572_2311	sce_1858	0.00E+00	0.00E+00
SCE1572_2312	sce_1859	5.00E-173	3.00E-173
SCE1572_2313	sce_1860	6.00E-138	5.00E-140
SCE1572_2314	sce_1861	6.00E-93	7.00E-89
SCE1572_2315	sce_1862	1.00E-15	1.00E-15
SCE1572_2316	sce_1863	6.00E-170	0.00E+00
SCE1572_2317	sce_1864	1.00E-63	6.00E-63
SCE1572_2318	sce_1865	2.00E-40	2.00E-40
SCE1572_2319	sce_1866	6.00E-101	9.00E-113
SCE1572_2320	sce_1867	2.00E-109	4.00E-129
SCE1572_2321	sce_1868	8.00E-61	7.00E-61
SCE1572_2322	sce_1869	6.00E-109	3.00E-119
SCE1572_2323	sce_1870	0.00E+00	0.00E+00
SCE1572_2324	sce_1871	1.00E-56	2.00E-56
SCE1572_2325	sce_1872	6.00E-138	1.00E-151
SCE1572_2326	sce_1873	1.00E-96	2.00E-103
SCE1572_2327	sce_1875	0.00E+00	0.00E+00
SCE1572_2328	sce_1877	3.00E-146	3.00E-151
SCE1572_2329	sce_1878	0.00E+00	0.00E+00
SCE1572_2330	sce_1879	0.00E+00	0.00E+00
SCE1572_2331	sce_10208	1.00E-41	6.00E-41
SCE1572_2332	sce_10207	5.00E-100	3.00E-104
SCE1572_2333	sce_1883	0.00E+00	0.00E+00
SCE1572_2335	sce_1885	1.00E-112	3.00E-128
SCE1572_2336	sce_1886	2.00E-158	0.00E+00
SCE1572_2337	sce_1887	7.00E-59	3.00E-45
SCE1572_2338	sce_1888	3.00E-120	5.00E-139
SCE1572_2339	sce_1889	3.00E-88	1.00E-90
SCE1572_234	sce_5301	4.00E-44	3.00E-45
SCE1572_2341	sce_1890	7.00E-69	5.00E-76
SCE1572_2342	sce_1891	2.00E-34	2.00E-34
SCE1572_2343	sce_1893	1.00E-72	1.00E-72
SCE1572_2344	sce_1894	0.00E+00	0.00E+00
SCE1572_2345	sce_1895	1.00E-80	2.00E-80
SCE1572_2346	sce_1896	7.00E-69	6.00E-69

SCE1572_2347	sce_1897	9.00E-98	9.00E-98
SCE1572_2348	sce_1898	2.00E-125	6.00E-125
SCE1572_2349	sce_1899	1.00E-144	2.00E-142
SCE1572_235	sce_5302	4.00E-38	4.00E-38
SCE1572_2351	sce_1902	0.00E+00	0.00E+00
SCE1572_2352	sce_1903	1.00E-75	1.00E-78
SCE1572_2353	sce_1904	1.00E-37	3.00E-51
SCE1572_2354	sce_1905	2.00E-109	2.00E-109
SCE1572_2355	sce_1906	1.00E-128	2.00E-118
SCE1572_2356	sce_1907	0.00E+00	0.00E+00
SCE1572_2357	sce_1908	4.00E-91	4.00E-91
SCE1572_2358	sce_1909	6.00E-125	2.00E-125
SCE1572_2359	sce_1910	4.00E-121	2.00E-133
SCE1572_2361	sce_1912	0.00E+00	0.00E+00
SCE1572_2362	sce_1913	0.00E+00	0.00E+00
SCE1572_2363	sce_1914	7.00E-52	3.00E-60
SCE1572_2364	sce_1915	5.00E-16	1.00E-21
SCE1572_2365	sce_1916	2.00E-110	6.00E-94
SCE1572_2366	sce_1917	0.00E+00	0.00E+00
SCE1572_2368	sce_1918	1.00E-94	1.00E-98
SCE1572_2369	sce_1919	6.00E-27	1.00E-27
SCE1572_237	sce_5613	2.00E-23	1.00E-23
SCE1572_2370	sce_1920	2.00E-159	0.00E+00
SCE1572_2371	sce_1921	0.00E+00	0.00E+00
SCE1572_2372	sce_1922	0.00E+00	0.00E+00
SCE1572_2373	sce_1923	2.00E-41	5.00E-37
SCE1572_2374	sce_1925	2.00E-54	2.00E-54
SCE1572_2375	sce_1926	2.00E-45	2.00E-45
SCE1572_2376	sce_1927	0.00E+00	0.00E+00
SCE1572_2377	sce_1928	2.00E-32	6.00E-58
SCE1572_2378	sce_1929	2.00E-51	2.00E-51
SCE1572_2379	sce_1930	1.00E-91	3.00E-91
SCE1572_238	sce_5612	2.00E-12	2.00E-12
SCE1572_2380	sce_1931	1.00E-101	5.00E-88
SCE1572_2381	sce_1932	0.00E+00	0.00E+00
SCE1572_2382	sce_1933	2.00E-133	3.00E-136
SCE1572_2383	sce_1935	1.00E-143	1.00E-138
SCE1572_2384	sce_1937	8.00E-93	1.00E-93
SCE1572_2385	sce_1938	0.00E+00	0.00E+00
SCE1572_2386	sce_1939	0.00E+00	0.00E+00
SCE1572_2387	sce_1940	0.00E+00	0.00E+00
SCE1572_2389	sce_1941	9.00E-35	3.00E-31
SCE1572_2390	sce_1942	0.00E+00	0.00E+00
SCE1572_2392	sce_1945	0.00E+00	0.00E+00
SCE1572_2393	sce_1946	1.00E-155	3.00E-154
SCE1572_2394	sce_1947	0.00E+00	0.00E+00
SCE1572_2395	sce_1948	0.00E+00	0.00E+00
SCE1572_2396	sce_1950	4.00E-96	5.00E-119
SCE1572_2397	sce_1951	1.00E-24	1.00E-24
SCE1572_2398	sce_1952	2.00E-54	2.00E-49
SCE1572_2399	sce_1953	8.00E-78	8.00E-78
SCE1572_24	sce_25	0.00E+00	0.00E+00
SCE1572_2400	sce_1954	0.00E+00	0.00E+00
SCE1572_2401	sce_1955	2.00E-80	2.00E-79
SCE1572_2402	sce_1956	5.00E-70	5.00E-70
SCE1572_2403	sce_1957	2.00E-79	4.00E-78
SCE1572_2404	sce_1958	7.00E-94	2.00E-101
SCE1572_2405	sce_1959	0.00E+00	0.00E+00
SCE1572_2406	sce_1960	2.00E-150	4.00E-142
SCE1572_2407	sce_1961	4.00E-165	1.00E-160
SCE1572_2408	sce_1962	3.00E-108	1.00E-107
SCE1572_2409	sce_1963	3.00E-68	3.00E-68
SCE1572_241	sce_10193	0.00E+00	0.00E+00
SCE1572_241	sce_3499	0.00E+00	0.00E+00
SCE1572_241	sce_8817	0.00E+00	0.00E+00
SCE1572_2410	sce_1964	9.00E-174	9.00E-174
SCE1572_2411	sce_1965	3.00E-113	1.00E-118
SCE1572_2412	sce_1966	0.00E+00	0.00E+00
SCE1572_2413	sce_1967	3.00E-84	3.00E-84
SCE1572_2414	sce_1968	0.00E+00	0.00E+00
SCE1572_2415	sce_1969	2.00E-21	1.00E-24
SCE1572_2416	sce_1970	7.00E-17	2.00E-14
SCE1572_2418	sce_1971	2.00E-41	2.00E-39
SCE1572_2419	sce_1973	0.00E+00	0.00E+00
SCE1572_2420	sce_1974	0.00E+00	0.00E+00
SCE1572_2421	sce_1975	2.00E-113	4.00E-118

SCE1572_2423	sce_1976	2.00E-43	1.00E-43
SCE1572_2424	sce_1977	1.00E-159	2.00E-159
SCE1572_2425	sce_1978	0.00E+00	0.00E+00
SCE1572_2426	sce_1979	3.00E-146	3.00E-146
SCE1572_2427	sce_1980	0.00E+00	0.00E+00
SCE1572_2428	sce_1981	2.00E-69	3.00E-69
SCE1572_2429	sce_1982	3.00E-76	3.00E-76
SCE1572_2430	sce_1983	0.00E+00	0.00E+00
SCE1572_2431	sce_1985	7.00E-79	4.00E-67
SCE1572_2432	sce_1986	6.00E-169	7.00E-171
SCE1572_2433	sce_1987	4.00E-102	1.00E-104
SCE1572_2434	sce_1988	0.00E+00	0.00E+00
SCE1572_2435	sce_1989	7.00E-156	1.00E-153
SCE1572_2436	sce_1990	3.00E-88	6.00E-93
SCE1572_2437	sce_1991	2.00E-42	8.00E-45
SCE1572_2438	sce_1992	1.00E-136	1.00E-135
SCE1572_2439	sce_1993	3.00E-115	2.00E-127
SCE1572_244	sce_8398	5.00E-73	3.00E-82
SCE1572_2440	sce_1994	0.00E+00	0.00E+00
SCE1572_2441	sce_1995	0.00E+00	0.00E+00
SCE1572_2442	sce_1997	4.00E-81	2.00E-75
SCE1572_2443	sce_8759	3.00E-18	3.00E-17
SCE1572_2444	sce_1996	3.00E-112	7.00E-100
SCE1572_2446	sce_1998	0.00E+00	0.00E+00
SCE1572_245	sce_513	7.00E-52	3.00E-62
SCE1572_2453	sce_1999	0.00E+00	0.00E+00
SCE1572_2454	sce_2000	8.00E-148	2.00E-162
SCE1572_2455	sce_2001	5.00E-177	2.00E-180
SCE1572_2456	sce_2002	1.00E-60	2.00E-62
SCE1572_2458	sce_2003	4.00E-165	3.00E-169
SCE1572_2459	sce_2004	2.00E-100	2.00E-102
SCE1572_2464	sce_2005	0.00E+00	0.00E+00
SCE1572_2466	sce_2006	2.00E-162	4.00E-159
SCE1572_2467	sce_2007	0.00E+00	0.00E+00
SCE1572_2468	sce_2010	3.00E-148	3.00E-148
SCE1572_2469	sce_2011	9.00E-133	3.00E-112
SCE1572_2471	sce_2012	0.00E+00	0.00E+00
SCE1572_2472	sce_2013	4.00E-39	1.00E-52
SCE1572_2473	sce_2014	5.00E-75	1.00E-79
SCE1572_2474	sce_2015	1.00E-179	0.00E+00
SCE1572_2475	sce_2016	0.00E+00	0.00E+00
SCE1572_2476	sce_2017	1.00E-81	6.00E-89
SCE1572_2477	sce_2018	1.00E-137	1.00E-147
SCE1572_2478	sce_2019	7.00E-129	4.00E-118
SCE1572_2479	sce_2020	4.00E-69	4.00E-69
SCE1572_2481	sce_7593	3.00E-115	8.00E-109
SCE1572_2482	sce_2042	0.00E+00	0.00E+00
SCE1572_2486	sce_2056	1.00E-116	2.00E-132
SCE1572_2487	sce_2058	8.00E-129	3.00E-137
SCE1572_2489	sce_2059	3.00E-33	1.00E-37
SCE1572_2490	sce_2066	0.00E+00	0.00E+00
SCE1572_2494	sce_2073	4.00E-180	0.00E+00
SCE1572_2495	sce_2074	6.00E-113	5.00E-113
SCE1572_2497	sce_2075	0.00E+00	0.00E+00
SCE1572_2499	sce_2419	0.00E+00	0.00E+00
SCE1572_25	sce_27	5.00E-83	2.00E-69
SCE1572_2500	sce_2418	2.00E-134	9.00E-119
SCE1572_2503	sce_7548	2.00E-157	6.00E-161
SCE1572_2504	sce_3033	2.00E-69	2.00E-69
SCE1572_2505	sce_3034	3.00E-113	1.00E-113
SCE1572_251	sce_7318	0.00E+00	0.00E+00
SCE1572_251	sce_1116	0.00E+00	0.00E+00
SCE1572_251	sce_5158	0.00E+00	0.00E+00
SCE1572_2510	sce_2355	4.00E-13	4.00E-10
SCE1572_2511	sce_2094	4.00E-135	5.00E-139
SCE1572_2517	sce_8744	1.00E-10	1.00E-10
SCE1572_2519	sce_2437	0.00E+00	0.00E+00
SCE1572_2520	sce_2097	1.00E-150	2.00E-160
SCE1572_2521	sce_2098	9.00E-89	1.00E-98
SCE1572_2522	sce_2099	2.00E-93	2.00E-113
SCE1572_2524	sce_2100	9.00E-57	1.00E-79
SCE1572_2525	sce_2101	0.00E+00	0.00E+00
SCE1572_2526	sce_2102	2.00E-138	6.00E-117
SCE1572_2527	sce_3701	9.00E-99	9.00E-100
SCE1572_253	sce_5453	5.00E-44	7.00E-40
SCE1572_2537	sce_2104	2.00E-73	4.00E-67

SCE1572_2538	sce_2118	0.00E+00	1.00E-179
SCE1572_2539	sce_2119	0.00E+00	0.00E+00
SCE1572_2543	sce_2126	8.00E-52	2.00E-61
SCE1572_2544	sce_5086	0.00E+00	0.00E+00
SCE1572_2544	sce_5085	0.00E+00	0.00E+00
SCE1572_2544	sce_6701	0.00E+00	0.00E+00
SCE1572_2547	sce_2128	0.00E+00	0.00E+00
SCE1572_255	sce_8414	2.00E-22	2.00E-26
SCE1572_2551	sce_2130	0.00E+00	0.00E+00
SCE1572_2553	sce_2131	0.00E+00	0.00E+00
SCE1572_2554	sce_2132	0.00E+00	0.00E+00
SCE1572_2555	sce_2133	3.00E-93	9.00E-101
SCE1572_2557	sce_8817	0.00E+00	0.00E+00
SCE1572_256	sce_8408	6.00E-15	3.00E-16
SCE1572_2560	sce_2134	2.00E-30	7.00E-40
SCE1572_2561	sce_2135	1.00E-147	8.00E-137
SCE1572_2562	sce_2136	0.00E+00	0.00E+00
SCE1572_2563	sce_2137	4.00E-122	1.00E-122
SCE1572_2564	sce_2138	9.00E-40	7.00E-35
SCE1572_2567	sce_2139	0.00E+00	0.00E+00
SCE1572_2568	sce_2140	5.00E-22	7.00E-31
SCE1572_2570	sce_2143	2.00E-23	2.00E-23
SCE1572_2573	sce_2144	8.00E-99	2.00E-104
SCE1572_2574	sce_2145	2.00E-74	8.00E-64
SCE1572_2575	sce_2146	0.00E+00	0.00E+00
SCE1572_2577	sce_2148	2.00E-159	8.00E-153
SCE1572_2578	sce_2156	0.00E+00	0.00E+00
SCE1572_258	sce_8407	6.00E-78	2.00E-75
SCE1572_2581	sce_2162	2.00E-58	3.00E-59
SCE1572_2586	sce_6226	0.00E+00	0.00E+00
SCE1572_2587	sce_6227	7.00E-29	1.00E-28
SCE1572_2588	sce_3750	0.00E+00	0.00E+00
SCE1572_2588	sce_6712	0.00E+00	0.00E+00
SCE1572_2588	sce_9954	0.00E+00	0.00E+00
SCE1572_2588	sce_5226	0.00E+00	0.00E+00
SCE1572_2588	sce_5751	0.00E+00	0.00E+00
SCE1572_2589	sce_6703	0.00E+00	0.00E+00
SCE1572_259	sce_8408	1.00E-15	3.00E-16
SCE1572_2594	sce_2174	0.00E+00	0.00E+00
SCE1572_26	sce_28	9.00E-157	0.00E+00
SCE1572_260	sce_6878	2.00E-87	8.00E-93
SCE1572_2604	sce_2186	1.00E-80	4.00E-74
SCE1572_2605	sce_2187	2.00E-137	1.00E-124
SCE1572_2606	sce_2188	1.00E-43	2.00E-42
SCE1572_2608	sce_2189	0.00E+00	0.00E+00
SCE1572_2609	sce_2190	7.00E-84	7.00E-84
SCE1572_2610	sce_2192	1.00E-124	1.00E-116
SCE1572_2612	sce_2195	0.00E+00	4.00E-125
SCE1572_2613	sce_2196	8.00E-63	3.00E-54
SCE1572_2614	sce_2199	1.00E-66	3.00E-55
SCE1572_2615	sce_2200	0.00E+00	0.00E+00
SCE1572_2616	sce_7835	1.00E-65	3.00E-49
SCE1572_2617	sce_2202	2.00E-38	1.00E-38
SCE1572_2619	sce_2204	0.00E+00	0.00E+00
SCE1572_262	sce_4682	2.00E-111	8.00E-96
SCE1572_2620	sce_3715	0.00E+00	0.00E+00
SCE1572_2621	sce_3716	0.00E+00	0.00E+00
SCE1572_2624	sce_5143	0.00E+00	0.00E+00
SCE1572_2626	sce_8582	2.00E-79	2.00E-83
SCE1572_2627	sce_5133	0.00E+00	0.00E+00
SCE1572_2628	sce_5134	0.00E+00	0.00E+00
SCE1572_2629	sce_5147	0.00E+00	0.00E+00
SCE1572_2629	sce_4662	0.00E+00	0.00E+00
SCE1572_2630	sce_4663	0.00E+00	0.00E+00
SCE1572_2630	sce_5148	0.00E+00	0.00E+00
SCE1572_2631	sce_5156	0.00E+00	0.00E+00
SCE1572_2632	sce_1664	0.00E+00	0.00E+00
SCE1572_2632	sce_5137	0.00E+00	0.00E+00
SCE1572_2633	sce_5137	0.00E+00	0.00E+00
SCE1572_2633	sce_1664	0.00E+00	0.00E+00
SCE1572_2634	sce_5138	0.00E+00	0.00E+00
SCE1572_2634	sce_1665	0.00E+00	0.00E+00
SCE1572_2635	sce_3717	1.00E-165	2.00E-162
SCE1572_2636	sce_3502	3.00E-97	1.00E-102
SCE1572_2637	sce_3712	0.00E+00	0.00E+00
SCE1572_2638	sce_3711	0.00E+00	0.00E+00

SCE1572_2639	sce_3710	1.00E-124	1.00E-124
SCE1572_264	sce_1106	2.00E-39	1.00E-38
SCE1572_2640	sce_3709	2.00E-160	7.00E-155
SCE1572_2641	sce_3708	0.00E+00	0.00E+00
SCE1572_2642	sce_3707	4.00E-121	1.00E-135
SCE1572_2643	sce_3706	6.00E-129	2.00E-133
SCE1572_2644	sce_6530	0.00E+00	1.00E-174
SCE1572_2647	sce_6531	0.00E+00	0.00E+00
SCE1572_2647	sce_9918	0.00E+00	0.00E+00
SCE1572_2647	sce_894	0.00E+00	0.00E+00
SCE1572_265	sce_5651	1.00E-51	5.00E-55
SCE1572_2652	sce_9920	0.00E+00	0.00E+00
SCE1572_2652	sce_6535	0.00E+00	0.00E+00
SCE1572_2652	sce_3003	0.00E+00	0.00E+00
SCE1572_2652	sce_5835	0.00E+00	0.00E+00
SCE1572_2652	sce_10204	0.00E+00	0.00E+00
SCE1572_2652	sce_9557	0.00E+00	0.00E+00
SCE1572_2652	sce_7640	0.00E+00	0.00E+00
SCE1572_2652	sce_388	0.00E+00	0.00E+00
SCE1572_2652	sce_2065	0.00E+00	0.00E+00
SCE1572_2652	sce_4513	0.00E+00	0.00E+00
SCE1572_2652	sce_6092	0.00E+00	0.00E+00
SCE1572_2652	sce_7661	0.00E+00	0.00E+00
SCE1572_2652	sce_6107	0.00E+00	0.00E+00
SCE1572_2652	sce_8618	0.00E+00	0.00E+00
SCE1572_2652	sce_3085	0.00E+00	0.00E+00
SCE1572_2652	sce_6095	0.00E+00	0.00E+00
SCE1572_2652	sce_3917	0.00E+00	0.00E+00
SCE1572_2653	sce_2205	6.00E-110	5.00E-128
SCE1572_2654	sce_2206	3.00E-78	2.00E-84
SCE1572_2655	sce_2207	0.00E+00	0.00E+00
SCE1572_2656	sce_2208	0.00E+00	0.00E+00
SCE1572_2656	sce_3055	0.00E+00	6.00E-166
SCE1572_2657	sce_2209	5.00E-129	4.00E-137
SCE1572_2658	sce_2210	0.00E+00	0.00E+00
SCE1572_2659	sce_2212	0.00E+00	0.00E+00
SCE1572_2661	sce_2213	7.00E-105	1.00E-88
SCE1572_2662	sce_2214	6.00E-45	2.00E-44
SCE1572_2663	sce_2215	4.00E-30	3.00E-39
SCE1572_2664	sce_2216	4.00E-124	2.00E-124
SCE1572_2665	sce_2217	6.00E-107	6.00E-107
SCE1572_2669	sce_2218	0.00E+00	0.00E+00
SCE1572_2670	sce_2219	0.00E+00	0.00E+00
SCE1572_2671	sce_2220	4.00E-136	8.00E-132
SCE1572_2672	sce_2221	3.00E-96	4.00E-113
SCE1572_2673	sce_2222	0.00E+00	0.00E+00
SCE1572_2674	sce_2223	0.00E+00	0.00E+00
SCE1572_2675	sce_2224	2.00E-162	1.00E-156
SCE1572_2676	sce_2225	4.00E-112	2.00E-112
SCE1572_2677	sce_2226	1.00E-70	1.00E-72
SCE1572_2678	sce_2227	3.00E-41	6.00E-51
SCE1572_2679	sce_2228	1.00E-112	4.00E-102
SCE1572_2688	sce_2229	3.00E-54	4.00E-51
SCE1572_2689	sce_2230	4.00E-99	6.00E-99
SCE1572_269	sce_9355	7.00E-28	6.00E-28
SCE1572_2691	sce_2231	0.00E+00	0.00E+00
SCE1572_2692	sce_2232	2.00E-73	2.00E-73
SCE1572_2693	sce_2233	1.00E-140	0.00E+00
SCE1572_2694	sce_2234	1.00E-112	9.00E-107
SCE1572_2695	sce_2235	9.00E-76	4.00E-66
SCE1572_27	sce_29	8.00E-40	1.00E-39
SCE1572_2700	sce_1425	1.00E-20	4.00E-23
SCE1572_2701	sce_6712	0.00E+00	0.00E+00
SCE1572_2701	sce_3750	0.00E+00	0.00E+00
SCE1572_2701	sce_9954	0.00E+00	0.00E+00
SCE1572_2701	sce_5229	0.00E+00	0.00E+00
SCE1572_2701	sce_5226	0.00E+00	0.00E+00
SCE1572_2701	sce_9665	0.00E+00	0.00E+00
SCE1572_2701	sce_5403	0.00E+00	0.00E+00
SCE1572_2701	sce_5751	0.00E+00	0.00E+00
SCE1572_2701	sce_7154	0.00E+00	0.00E+00
SCE1572_2702	sce_2240	0.00E+00	0.00E+00
SCE1572_2703	sce_2241	4.00E-67	4.00E-67
SCE1572_2704	sce_2242	0.00E+00	0.00E+00
SCE1572_2705	sce_2243	5.00E-64	1.00E-56
SCE1572_2706	sce_2244	0.00E+00	0.00E+00

SCE1572_2706	sce_2245	0.00E+00	0.00E+00
SCE1572_2707	sce_2246	4.00E-67	9.00E-66
SCE1572_2708	sce_2247	8.00E-148	3.00E-152
SCE1572_2709	sce_2248	2.00E-76	6.00E-87
SCE1572_271	sce_6076	0.00E+00	0.00E+00
SCE1572_2710	sce_2249	6.00E-75	8.00E-75
SCE1572_2711	sce_2250	1.00E-147	5.00E-151
SCE1572_2712	sce_2251	9.00E-65	3.00E-69
SCE1572_2713	sce_2252	2.00E-138	7.00E-133
SCE1572_2714	sce_2253	1.00E-94	2.00E-94
SCE1572_2715	sce_2254	0.00E+00	0.00E+00
SCE1572_2716	sce_2255	2.00E-126	6.00E-132
SCE1572_2718	sce_2256	0.00E+00	0.00E+00
SCE1572_2719	sce_2257	1.00E-53	1.00E-53
SCE1572_2720	sce_2258	0.00E+00	0.00E+00
SCE1572_2721	sce_2259	1.00E-82	4.00E-70
SCE1572_2722	sce_2260	0.00E+00	0.00E+00
SCE1572_2723	sce_2261	8.00E-67	1.00E-69
SCE1572_2724	sce_2262	4.00E-102	4.00E-122
SCE1572_2725	sce_2263	2.00E-160	1.00E-160
SCE1572_2726	sce_2264	3.00E-111	3.00E-111
SCE1572_2727	sce_2265	4.00E-114	2.00E-119
SCE1572_2728	sce_2266	3.00E-172	3.00E-176
SCE1572_2729	sce_2267	2.00E-149	2.00E-149
SCE1572_273	sce_9375	0.00E+00	0.00E+00
SCE1572_2730	sce_2268	2.00E-165	3.00E-177
SCE1572_2731	sce_2269	0.00E+00	0.00E+00
SCE1572_2732	sce_2270	8.00E-105	5.00E-106
SCE1572_2733	sce_2272	2.00E-92	3.00E-89
SCE1572_2734	sce_2273	2.00E-46	3.00E-46
SCE1572_2735	sce_2274	3.00E-107	2.00E-132
SCE1572_2736	sce_2275	1.00E-139	3.00E-134
SCE1572_2737	sce_2276	0.00E+00	0.00E+00
SCE1572_2738	sce_2277	2.00E-48	2.00E-43
SCE1572_2739	sce_2278	4.00E-139	2.00E-141
SCE1572_2740	sce_2279	2.00E-59	5.00E-67
SCE1572_2741	sce_2280	3.00E-21	1.00E-24
SCE1572_2742	sce_2281	2.00E-88	3.00E-88
SCE1572_2743	sce_2282	4.00E-163	4.00E-175
SCE1572_2744	sce_2283	4.00E-46	2.00E-52
SCE1572_2745	sce_2284	2.00E-116	6.00E-137
SCE1572_2746	sce_2285	8.00E-54	2.00E-62
SCE1572_2747	sce_2286	7.00E-72	6.00E-71
SCE1572_2748	sce_2287	0.00E+00	0.00E+00
SCE1572_2749	sce_2288	3.00E-153	7.00E-175
SCE1572_275	sce_8423	4.00E-111	4.00E-111
SCE1572_2750	sce_2289	1.00E-166	2.00E-166
SCE1572_2751	sce_2290	7.00E-144	5.00E-147
SCE1572_2752	sce_2291	1.00E-161	8.00E-157
SCE1572_2754	sce_2292	9.00E-81	4.00E-79
SCE1572_2758	sce_2304	0.00E+00	0.00E+00
SCE1572_2759	sce_2305	0.00E+00	0.00E+00
SCE1572_2760	sce_2306	3.00E-78	8.00E-70
SCE1572_2761	sce_2307	4.00E-103	1.00E-99
SCE1572_2763	sce_2308	0.00E+00	0.00E+00
SCE1572_2765	sce_2310	7.00E-51	4.00E-76
SCE1572_2766	sce_2311	3.00E-116	3.00E-116
SCE1572_2767	sce_2312	4.00E-56	7.00E-69
SCE1572_2768	sce_2313	2.00E-54	5.00E-55
SCE1572_2769	sce_2314	1.00E-38	7.00E-61
SCE1572_2770	sce_4153	3.00E-21	3.00E-19
SCE1572_2771	sce_7299	2.00E-25	1.00E-25
SCE1572_2774	sce_7302	6.00E-125	1.00E-119
SCE1572_2777	sce_10401	0.00E+00	0.00E+00
SCE1572_2781	sce_2367	2.00E-57	3.00E-54
SCE1572_2782	sce_2368	0.00E+00	0.00E+00
SCE1572_2783	sce_2369	2.00E-80	8.00E-83
SCE1572_2784	sce_5173	6.00E-157	3.00E-169
SCE1572_2787	sce_2372	8.00E-28	2.00E-29
SCE1572_2788	sce_2375	5.00E-48	5.00E-48
SCE1572_2789	sce_2376	7.00E-52	2.00E-49
SCE1572_2790	sce_2381	6.00E-75	3.00E-75
SCE1572_2792	sce_2439	0.00E+00	0.00E+00
SCE1572_2795	sce_2451	4.00E-48	4.00E-48
SCE1572_2796	sce_2449	2.00E-50	3.00E-46
SCE1572_2797	sce_1364	0.00E+00	0.00E+00

SCE1572_2798	sce_1365	1.00E-71	2.00E-69
SCE1572_2799	sce_2462	5.00E-109	3.00E-111
SCE1572_28	sce_30	9.00E-74	5.00E-90
SCE1572_2800	sce_1367	0.00E+00	0.00E+00
SCE1572_2803	sce_6975	3.00E-82	2.00E-91
SCE1572_2804	sce_2463	0.00E+00	0.00E+00
SCE1572_2805	sce_2465	0.00E+00	0.00E+00
SCE1572_2806	sce_4688	4.00E-138	2.00E-130
SCE1572_281	sce_6002	0.00E+00	0.00E+00
SCE1572_2813	sce_10023	2.00E-86	2.00E-86
SCE1572_2814	sce_7618	0.00E+00	0.00E+00
SCE1572_2815	sce_4673	7.00E-128	2.00E-132
SCE1572_2820	sce_7859	9.00E-172	0.00E+00
SCE1572_2821	sce_7860	1.00E-177	2.00E-176
SCE1572_2824	sce_6417	0.00E+00	0.00E+00
SCE1572_2825	sce_1113	6.00E-147	8.00E-137
SCE1572_2826	sce_1112	3.00E-103	5.00E-98
SCE1572_2827	sce_1111	6.00E-39	8.00E-47
SCE1572_2829	sce_1110	1.00E-23	1.00E-23
SCE1572_283	sce_1702	9.00E-83	4.00E-80
SCE1572_2835	sce_521	2.00E-14	2.00E-14
SCE1572_2836	sce_520	3.00E-09	4.00E-09
SCE1572_284	sce_9986	5.00E-93	2.00E-98
SCE1572_2846	sce_514	6.00E-29	4.00E-35
SCE1572_2848	sce_10371	0.00E+00	0.00E+00
SCE1572_2850	sce_10151	1.00E-101	1.00E-101
SCE1572_2858	sce_10022	2.00E-126	6.00E-118
SCE1572_2872	sce_2416	3.00E-14	3.00E-31
SCE1572_2873	sce_1277	1.00E-68	5.00E-68
SCE1572_2875	sce_2477	6.00E-133	1.00E-129
SCE1572_2876	sce_2478	0.00E+00	0.00E+00
SCE1572_2877	sce_2483	2.00E-32	1.00E-30
SCE1572_2878	sce_2484	0.00E+00	0.00E+00
SCE1572_288	sce_6683	2.00E-53	3.00E-57
SCE1572_2883	sce_2505	6.00E-61	5.00E-61
SCE1572_2884	sce_2506	2.00E-139	2.00E-139
SCE1572_2885	sce_2507	3.00E-166	2.00E-174
SCE1572_2886	sce_2508	0.00E+00	0.00E+00
SCE1572_2887	sce_2510	1.00E-14	7.00E-25
SCE1572_2888	sce_2511	9.00E-166	2.00E-166
SCE1572_2895	sce_2512	7.00E-128	1.00E-118
SCE1572_2897	sce_2548	3.00E-91	4.00E-83
SCE1572_2899	sce_2549	6.00E-172	3.00E-159
SCE1572_29	sce_31	2.00E-106	2.00E-106
SCE1572_2912	sce_7637	0.00E+00	0.00E+00
SCE1572_2912	sce_389	0.00E+00	0.00E+00
SCE1572_2912	sce_2666	0.00E+00	0.00E+00
SCE1572_2912	sce_9231	0.00E+00	0.00E+00
SCE1572_2912	sce_9237	0.00E+00	0.00E+00
SCE1572_2913	sce_7637	0.00E+00	0.00E+00
SCE1572_2913	sce_9231	0.00E+00	0.00E+00
SCE1572_2913	sce_389	0.00E+00	0.00E+00
SCE1572_2913	sce_9230	0.00E+00	0.00E+00
SCE1572_2913	sce_2666	0.00E+00	0.00E+00
SCE1572_2913	sce_9237	0.00E+00	0.00E+00
SCE1572_2914	sce_7637	0.00E+00	0.00E+00
SCE1572_2914	sce_389	0.00E+00	0.00E+00
SCE1572_2914	sce_2666	0.00E+00	0.00E+00
SCE1572_2914	sce_9230	0.00E+00	0.00E+00
SCE1572_2914	sce_9237	0.00E+00	0.00E+00
SCE1572_2914	sce_9231	0.00E+00	0.00E+00
SCE1572_2915	sce_4641	0.00E+00	0.00E+00
SCE1572_2916	sce_6296	4.00E-128	7.00E-128
SCE1572_2917	sce_2550	5.00E-132	1.00E-109
SCE1572_2926	sce_6392	2.00E-36	2.00E-29
SCE1572_2932	sce_2553	0.00E+00	0.00E+00
SCE1572_2934	sce_2554	3.00E-129	6.00E-148
SCE1572_2935	sce_2555	2.00E-14	6.00E-10
SCE1572_2936	sce_2556	1.00E-35	6.00E-36
SCE1572_2950	sce_7501	6.00E-08	8.00E-08
SCE1572_2954	sce_3808	1.00E-69	1.00E-70
SCE1572_2957	sce_2577	0.00E+00	0.00E+00
SCE1572_2960	sce_5463	3.00E-141	4.00E-143
SCE1572_2961	sce_2578	0.00E+00	0.00E+00
SCE1572_2963	sce_2579	0.00E+00	0.00E+00
SCE1572_297	sce_8687	3.00E-118	4.00E-117

SCE1572_2979	sce_2583	7.00E-60	1.00E-66
SCE1572_298	sce_8686	1.00E-142	3.00E-132
SCE1572_2980	sce_2584	2.00E-101	2.00E-99
SCE1572_2983	sce_2585	3.00E-106	8.00E-109
SCE1572_2984	sce_2586	6.00E-50	7.00E-50
SCE1572_2985	sce_2587	5.00E-60	6.00E-60
SCE1572_2987	sce_2588	0.00E+00	0.00E+00
SCE1572_299	sce_9374	2.00E-110	2.00E-106
SCE1572_2991	sce_2589	0.00E+00	0.00E+00
SCE1572_2991	sce_5830	0.00E+00	0.00E+00
SCE1572_2991	sce_5824	0.00E+00	0.00E+00
SCE1572_2991	sce_5827	0.00E+00	0.00E+00
SCE1572_2991	sce_2940	0.00E+00	0.00E+00
SCE1572_2992	sce_2590	2.00E-52	2.00E-52
SCE1572_2993	sce_2591	3.00E-93	1.00E-93
SCE1572_2995	sce_2593	0.00E+00	5.00E-180
SCE1572_2996	sce_2596	0.00E+00	0.00E+00
SCE1572_2998	sce_2597	1.00E-70	1.00E-70
SCE1572_3	sce_3	0.00E+00	0.00E+00
SCE1572_30	sce_32	0.00E+00	0.00E+00
SCE1572_3002	sce_6068	2.00E-68	2.00E-60
SCE1572_3003	sce_9937	2.00E-73	1.00E-70
SCE1572_3005	sce_9938	7.00E-26	3.00E-25
SCE1572_3007	sce_2598	4.00E-55	6.00E-68
SCE1572_3008	sce_2600	2.00E-63	2.00E-63
SCE1572_3009	sce_6056	5.00E-40	4.00E-40
SCE1572_3010	sce_2938	2.00E-31	4.00E-23
SCE1572_3011	sce_2937	0.00E+00	0.00E+00
SCE1572_3014	sce_2467	3.00E-147	5.00E-149
SCE1572_3015	sce_2476	6.00E-105	1.00E-117
SCE1572_3020	sce_7521	0.00E+00	0.00E+00
SCE1572_3023	sce_6869	1.00E-96	9.00E-95
SCE1572_3024	sce_6870	9.00E-99	1.00E-114
SCE1572_3032	sce_2601	1.00E-112	1.00E-112
SCE1572_3033	sce_2602	0.00E+00	0.00E+00
SCE1572_3034	sce_2603	4.00E-110	5.00E-114
SCE1572_3035	sce_2604	4.00E-60	3.00E-71
SCE1572_3036	sce_2606	4.00E-157	5.00E-173
SCE1572_3037	sce_2607	2.00E-105	1.00E-129
SCE1572_3061	sce_7454	7.00E-30	7.00E-13
SCE1572_3062	sce_7455	3.00E-62	2.00E-53
SCE1572_3063	sce_5710	0.00E+00	0.00E+00
SCE1572_3064	sce_2609	3.00E-43	3.00E-43
SCE1572_3065	sce_2610	9.00E-42	7.00E-47
SCE1572_3066	sce_2611	7.00E-67	6.00E-68
SCE1572_3067	sce_2612	2.00E-110	7.00E-104
SCE1572_3068	sce_2614	2.00E-120	1.00E-97
SCE1572_3069	sce_7327	4.00E-13	2.00E-10
SCE1572_3070	sce_7328	7.00E-51	8.00E-51
SCE1572_3071	sce_7329	0.00E+00	0.00E+00
SCE1572_3073	sce_7330	2.00E-06	7.00E-12
SCE1572_3074	sce_7331	3.00E-65	4.00E-65
SCE1572_3077	sce_9583	0.00E+00	0.00E+00
SCE1572_308	sce_2028	2.00E-142	8.00E-133
SCE1572_3080	sce_157	7.00E-86	7.00E-86
SCE1572_3082	sce_1504	1.00E-55	2.00E-51
SCE1572_3084	sce_7092	7.00E-97	2.00E-87
SCE1572_309	sce_7865	2.00E-135	2.00E-132
SCE1572_3093	sce_2615	8.00E-65	8.00E-65
SCE1572_3099	sce_9462	5.00E-56	4.00E-73
SCE1572_31	sce_33	1.00E-72	4.00E-72
SCE1572_310	sce_7866	1.00E-91	5.00E-85
SCE1572_3101	sce_2618	2.00E-47	3.00E-59
SCE1572_3103	sce_2621	1.00E-72	1.00E-70
SCE1572_3105	sce_2481	2.00E-53	1.00E-43
SCE1572_3107	sce_2623	4.00E-147	4.00E-131
SCE1572_3108	sce_2624	1.00E-143	1.00E-143
SCE1572_3109	sce_2625	0.00E+00	0.00E+00
SCE1572_311	sce_9786	1.00E-58	5.00E-53
SCE1572_3110	sce_2626	0.00E+00	0.00E+00
SCE1572_3111	sce_2627	4.00E-118	6.00E-120
SCE1572_3113	sce_1386	0.00E+00	0.00E+00
SCE1572_3114	sce_1387	0.00E+00	0.00E+00
SCE1572_3115	sce_2628	2.00E-121	2.00E-121
SCE1572_3116	sce_2629	8.00E-112	4.00E-109
SCE1572_3117	sce_2630	0.00E+00	0.00E+00

SCE1572_3118	sce_2631	4.00E-08	1.00E-08
SCE1572_3119	sce_2632	1.00E-54	5.00E-49
SCE1572_312	sce_9787	2.00E-26	1.00E-25
SCE1572_3120	sce_2633	1.00E-28	2.00E-26
SCE1572_3124	sce_2634	2.00E-49	3.00E-43
SCE1572_3125	sce_2636	4.00E-70	4.00E-70
SCE1572_3126	sce_2637	7.00E-57	2.00E-58
SCE1572_3127	sce_6335	2.00E-21	1.00E-24
SCE1572_3131	sce_6339	0.00E+00	0.00E+00
SCE1572_3133	sce_6341	0.00E+00	0.00E+00
SCE1572_3137	sce_3750	0.00E+00	0.00E+00
SCE1572_3137	sce_7657	0.00E+00	0.00E+00
SCE1572_3137	sce_6712	0.00E+00	0.00E+00
SCE1572_3137	sce_9954	0.00E+00	0.00E+00
SCE1572_3137	sce_3491	0.00E+00	0.00E+00
SCE1572_3137	sce_5226	0.00E+00	0.00E+00
SCE1572_3137	sce_9665	0.00E+00	0.00E+00
SCE1572_3137	sce_5751	0.00E+00	0.00E+00
SCE1572_3137	sce_7154	0.00E+00	0.00E+00
SCE1572_3137	sce_5403	0.00E+00	0.00E+00
SCE1572_3137	sce_5746	0.00E+00	0.00E+00
SCE1572_3138	sce_2638	4.00E-74	5.00E-74
SCE1572_314	sce_2561	2.00E-92	5.00E-93
SCE1572_3140	sce_1537	0.00E+00	0.00E+00
SCE1572_3141	sce_7640	0.00E+00	0.00E+00
SCE1572_3141	sce_388	0.00E+00	0.00E+00
SCE1572_3141	sce_5835	0.00E+00	0.00E+00
SCE1572_3141	sce_10204	0.00E+00	0.00E+00
SCE1572_3141	sce_3085	0.00E+00	0.00E+00
SCE1572_3141	sce_4513	0.00E+00	0.00E+00
SCE1572_3141	sce_7109	0.00E+00	0.00E+00
SCE1572_3141	sce_9557	0.00E+00	0.00E+00
SCE1572_3141	sce_3003	0.00E+00	0.00E+00
SCE1572_3141	sce_9920	0.00E+00	0.00E+00
SCE1572_3141	sce_7661	0.00E+00	0.00E+00
SCE1572_3141	sce_3917	0.00E+00	0.00E+00
SCE1572_3141	sce_6107	0.00E+00	0.00E+00
SCE1572_3141	sce_6093	0.00E+00	0.00E+00
SCE1572_3141	sce_6095	0.00E+00	0.00E+00
SCE1572_3141	sce_6094	0.00E+00	0.00E+00
SCE1572_3141	sce_2065	0.00E+00	0.00E+00
SCE1572_3141	sce_6535	0.00E+00	0.00E+00
SCE1572_3141	sce_4466	0.00E+00	0.00E+00
SCE1572_3141	sce_6092	0.00E+00	0.00E+00
SCE1572_3149	sce_5504	4.00E-173	0.00E+00
SCE1572_315	sce_616	0.00E+00	0.00E+00
SCE1572_3151	sce_2640	4.00E-77	1.00E-55
SCE1572_3152	sce_2641	0.00E+00	0.00E+00
SCE1572_3152	sce_5502	0.00E+00	0.00E+00
SCE1572_3154	sce_2642	1.00E-39	4.00E-15
SCE1572_3155	sce_2643	2.00E-89	5.00E-83
SCE1572_3156	sce_2644	5.00E-73	2.00E-72
SCE1572_3157	sce_2645	0.00E+00	0.00E+00
SCE1572_3157	sce_9974	0.00E+00	0.00E+00
SCE1572_3157	sce_9973	0.00E+00	0.00E+00
SCE1572_3158	sce_5229	0.00E+00	0.00E+00
SCE1572_3158	sce_6712	0.00E+00	0.00E+00
SCE1572_3158	sce_3750	0.00E+00	0.00E+00
SCE1572_3158	sce_9954	0.00E+00	0.00E+00
SCE1572_3158	sce_5403	0.00E+00	0.00E+00
SCE1572_3158	sce_5226	0.00E+00	0.00E+00
SCE1572_3158	sce_774	0.00E+00	0.00E+00
SCE1572_3158	sce_9665	0.00E+00	0.00E+00
SCE1572_3159	sce_2654	3.00E-176	3.00E-173
SCE1572_316	sce_619	0.00E+00	0.00E+00
SCE1572_3160	sce_10130	4.00E-08	5.00E-08
SCE1572_3161	sce_10131	5.00E-27	5.00E-27
SCE1572_3162	sce_2655	0.00E+00	0.00E+00
SCE1572_3163	sce_2656	0.00E+00	0.00E+00
SCE1572_3163	sce_1444	0.00E+00	0.00E+00
SCE1572_3164	sce_9942	0.00E+00	0.00E+00
SCE1572_3165	sce_9943	0.00E+00	0.00E+00
SCE1572_3166	sce_9944	0.00E+00	0.00E+00
SCE1572_3169	sce_5119	4.00E-116	1.00E-110
SCE1572_317	sce_620	5.00E-105	1.00E-101
SCE1572_3172	sce_389	0.00E+00	0.00E+00

SCE1572_3172	sce_7637	0.00E+00	0.00E+00
SCE1572_3172	sce_9237	0.00E+00	0.00E+00
SCE1572_3172	sce_2666	0.00E+00	0.00E+00
SCE1572_3172	sce_9230	0.00E+00	0.00E+00
SCE1572_3172	sce_9231	0.00E+00	0.00E+00
SCE1572_3173	sce_2667	4.00E-79	2.00E-87
SCE1572_3174	sce_2668	2.00E-93	9.00E-101
SCE1572_3181	sce_2671	0.00E+00	0.00E+00
SCE1572_3182	sce_2672	0.00E+00	0.00E+00
SCE1572_3183	sce_2673	4.00E-123	4.00E-122
SCE1572_3188	sce_2679	0.00E+00	0.00E+00
SCE1572_3189	sce_2680	9.00E-21	3.00E-26
SCE1572_3190	sce_2681	1.00E-69	4.00E-72
SCE1572_3191	sce_2683	7.00E-20	7.00E-20
SCE1572_3192	sce_2684	6.00E-66	9.00E-66
SCE1572_3193	sce_2685	0.00E+00	0.00E+00
SCE1572_3194	sce_2686	0.00E+00	0.00E+00
SCE1572_3196	sce_2688	0.00E+00	0.00E+00
SCE1572_3197	sce_2689	4.00E-163	4.00E-175
SCE1572_3198	sce_2690	6.00E-57	8.00E-59
SCE1572_3199	sce_2692	6.00E-150	2.00E-149
SCE1572_32	sce_34	2.00E-86	3.00E-73
SCE1572_3200	sce_2694	3.00E-66	5.00E-71
SCE1572_3201	sce_2695	2.00E-25	2.00E-25
SCE1572_3202	sce_2696	0.00E+00	0.00E+00
SCE1572_3203	sce_2697	6.00E-123	1.00E-124
SCE1572_3204	sce_2698	1.00E-67	2.00E-70
SCE1572_3205	sce_2699	0.00E+00	0.00E+00
SCE1572_3206	sce_2700	0.00E+00	0.00E+00
SCE1572_3207	sce_2701	3.00E-167	1.00E-167
SCE1572_3208	sce_2702	7.00E-114	7.00E-114
SCE1572_3209	sce_2703	2.00E-110	8.00E-125
SCE1572_3210	sce_2704	9.00E-97	2.00E-103
SCE1572_3211	sce_2705	6.00E-19	2.00E-07
SCE1572_3212	sce_2706	1.00E-47	5.00E-44
SCE1572_3213	sce_2707	3.00E-125	4.00E-119
SCE1572_3215	sce_2708	0.00E+00	0.00E+00
SCE1572_3216	sce_2709	0.00E+00	4.00E-172
SCE1572_3217	sce_2710	0.00E+00	0.00E+00
SCE1572_3218	sce_2711	2.00E-13	5.00E-28
SCE1572_3219	sce_2712	2.00E-60	4.00E-62
SCE1572_322	sce_4699	3.00E-61	1.00E-68
SCE1572_3220	sce_2713	2.00E-42	2.00E-42
SCE1572_3221	sce_2714	9.00E-63	9.00E-58
SCE1572_3222	sce_2715	8.00E-122	7.00E-127
SCE1572_3223	sce_2716	4.00E-34	2.00E-38
SCE1572_3225	sce_2717	0.00E+00	0.00E+00
SCE1572_3226	sce_2718	1.00E-125	1.00E-136
SCE1572_3227	sce_2719	5.00E-113	1.00E-116
SCE1572_3228	sce_2720	3.00E-87	3.00E-87
SCE1572_3229	sce_2721	2.00E-42	2.00E-42
SCE1572_3230	sce_2722	6.00E-119	5.00E-119
SCE1572_3231	sce_2723	3.00E-151	3.00E-154
SCE1572_3232	sce_2724	3.00E-174	0.00E+00
SCE1572_3233	sce_2726	0.00E+00	0.00E+00
SCE1572_3234	sce_2727	2.00E-105	8.00E-99
SCE1572_3235	sce_2728	0.00E+00	0.00E+00
SCE1572_3236	sce_2732	0.00E+00	0.00E+00
SCE1572_3238	sce_2733	0.00E+00	0.00E+00
SCE1572_3238	sce_10203	0.00E+00	0.00E+00
SCE1572_3239	sce_2734	2.00E-166	1.00E-161
SCE1572_3241	sce_2735	0.00E+00	0.00E+00
SCE1572_3243	sce_2737	0.00E+00	0.00E+00
SCE1572_3244	sce_2738	6.00E-36	2.00E-35
SCE1572_3245	sce_2740	2.00E-175	2.00E-174
SCE1572_3246	sce_2741	0.00E+00	0.00E+00
SCE1572_3247	sce_2742	5.00E-82	1.00E-91
SCE1572_3248	sce_2744	5.00E-64	3.00E-64
SCE1572_3249	sce_2745	4.00E-167	8.00E-174
SCE1572_3250	sce_2746	2.00E-55	2.00E-54
SCE1572_3252	sce_2748	0.00E+00	0.00E+00
SCE1572_3253	sce_2749	1.00E-100	2.00E-103
SCE1572_3254	sce_2750	3.00E-153	7.00E-166
SCE1572_3255	sce_2751	1.00E-100	4.00E-100
SCE1572_3256	sce_2752	3.00E-82	4.00E-93
SCE1572_3257	sce_2753	5.00E-106	2.00E-111

SCE1572_3259	sce_2754	0.00E+00	0.00E+00
SCE1572_3260	sce_2755	7.00E-11	6.00E-06
SCE1572_3261	sce_2756	0.00E+00	0.00E+00
SCE1572_3262	sce_2757	1.00E-120	7.00E-129
SCE1572_3263	sce_2758	7.00E-167	7.00E-167
SCE1572_3264	sce_2759	0.00E+00	0.00E+00
SCE1572_3266	sce_2760	1.00E-160	2.00E-159
SCE1572_3267	sce_2761	0.00E+00	0.00E+00
SCE1572_3268	sce_2762	3.00E-89	1.00E-89
SCE1572_3269	sce_2764	5.00E-129	8.00E-137
SCE1572_3270	sce_2765	0.00E+00	0.00E+00
SCE1572_3271	sce_2766	0.00E+00	0.00E+00
SCE1572_3273	sce_2767	2.00E-118	1.00E-118
SCE1572_3274	sce_2768	9.00E-176	3.00E-172
SCE1572_3275	sce_2769	9.00E-58	9.00E-58
SCE1572_3276	sce_2770	5.00E-99	4.00E-111
SCE1572_3278	sce_2773	0.00E+00	3.00E-180
SCE1572_3279	sce_2774	6.00E-144	1.00E-137
SCE1572_3280	sce_2775	0.00E+00	0.00E+00
SCE1572_3281	sce_2776	7.00E-136	1.00E-130
SCE1572_3283	sce_2777	0.00E+00	0.00E+00
SCE1572_3284	sce_2784	2.00E-07	2.00E-08
SCE1572_3285	sce_2782	5.00E-166	5.00E-166
SCE1572_3286	sce_2778	8.00E-99	2.00E-100
SCE1572_3288	sce_2785	0.00E+00	0.00E+00
SCE1572_3292	sce_2786	0.00E+00	0.00E+00
SCE1572_3293	sce_2787	6.00E-83	2.00E-82
SCE1572_3294	sce_2788	2.00E-83	3.00E-74
SCE1572_3297	sce_2789	0.00E+00	0.00E+00
SCE1572_3298	sce_2790	6.00E-55	3.00E-67
SCE1572_3299	sce_2791	5.00E-166	1.00E-174
SCE1572_33	sce_35	0.00E+00	0.00E+00
SCE1572_330	sce_4328	0.00E+00	6.00E-168
SCE1572_3300	sce_2792	6.00E-101	8.00E-108
SCE1572_3301	sce_2793	7.00E-146	4.00E-134
SCE1572_3302	sce_2794	0.00E+00	0.00E+00
SCE1572_3306	sce_2797	0.00E+00	0.00E+00
SCE1572_3307	sce_2798	2.00E-180	0.00E+00
SCE1572_3308	sce_2799	7.00E-55	1.00E-41
SCE1572_3309	sce_2800	4.00E-90	1.00E-92
SCE1572_331	sce_4326	1.00E-159	3.00E-170
SCE1572_3310	sce_2801	1.00E-108	3.00E-108
SCE1572_3311	sce_2802	1.00E-58	4.00E-60
SCE1572_3312	sce_2804	1.00E-61	5.00E-70
SCE1572_3313	sce_2805	6.00E-74	1.00E-86
SCE1572_3314	sce_2807	0.00E+00	0.00E+00
SCE1572_3315	sce_2808	1.00E-37	2.00E-32
SCE1572_3316	sce_2811	0.00E+00	0.00E+00
SCE1572_3317	sce_2812	0.00E+00	0.00E+00
SCE1572_3318	sce_2813	2.00E-104	2.00E-105
SCE1572_3319	sce_2814	5.00E-134	2.00E-143
SCE1572_332	sce_180	1.00E-78	2.00E-76
SCE1572_3320	sce_2815	0.00E+00	0.00E+00
SCE1572_3320	sce_3653	0.00E+00	0.00E+00
SCE1572_3321	sce_2816	0.00E+00	0.00E+00
SCE1572_3322	sce_2817	3.00E-11	3.00E-11
SCE1572_3324	sce_2819	7.00E-149	5.00E-153
SCE1572_3325	sce_2820	0.00E+00	0.00E+00
SCE1572_3326	sce_2821	3.00E-73	5.00E-77
SCE1572_3327	sce_2822	2.00E-61	2.00E-61
SCE1572_3328	sce_2823	3.00E-69	2.00E-69
SCE1572_3329	sce_2824	0.00E+00	0.00E+00
SCE1572_333	sce_181	3.00E-135	2.00E-127
SCE1572_3330	sce_2826	7.00E-43	3.00E-50
SCE1572_3331	sce_2827	9.00E-45	1.00E-46
SCE1572_3332	sce_2828	1.00E-21	1.00E-21
SCE1572_3333	sce_2829	0.00E+00	0.00E+00
SCE1572_3335	sce_2831	0.00E+00	0.00E+00
SCE1572_3336	sce_2832	0.00E+00	0.00E+00
SCE1572_3337	sce_2833	4.00E-153	1.00E-164
SCE1572_3338	sce_2834	7.00E-40	7.00E-40
SCE1572_3339	sce_2835	2.00E-171	2.00E-171
SCE1572_334	sce_183	7.00E-111	1.00E-122
SCE1572_3340	sce_2836	0.00E+00	0.00E+00
SCE1572_3341	sce_2837	2.00E-145	1.00E-144
SCE1572_3342	sce_2838	0.00E+00	0.00E+00

SCE1572_3343	sce_2839	1.00E-132	1.00E-132
SCE1572_3344	sce_2840	0.00E+00	0.00E+00
SCE1572_3345	sce_2841	0.00E+00	0.00E+00
SCE1572_335	sce_184	3.00E-166	3.00E-160
SCE1572_3352	sce_2846	0.00E+00	0.00E+00
SCE1572_3358	sce_2848	4.00E-50	2.00E-63
SCE1572_3359	sce_2849	0.00E+00	0.00E+00
SCE1572_336	sce_185	1.00E-78	7.00E-76
SCE1572_3360	sce_2850	0.00E+00	0.00E+00
SCE1572_3363	sce_2851	1.00E-76	1.00E-76
SCE1572_3364	sce_2853	5.00E-73	3.00E-73
SCE1572_3365	sce_2854	1.00E-91	2.00E-85
SCE1572_3366	sce_2855	3.00E-123	9.00E-113
SCE1572_3367	sce_2856	0.00E+00	0.00E+00
SCE1572_3368	sce_2857	0.00E+00	0.00E+00
SCE1572_3369	sce_2859	2.00E-123	1.00E-130
SCE1572_337	sce_187	0.00E+00	0.00E+00
SCE1572_3370	sce_2860	3.00E-52	3.00E-52
SCE1572_3371	sce_2861	0.00E+00	0.00E+00
SCE1572_3372	sce_2862	0.00E+00	0.00E+00
SCE1572_3373	sce_2863	1.00E-21	2.00E-20
SCE1572_3374	sce_2864	2.00E-128	7.00E-124
SCE1572_3375	sce_2865	2.00E-180	6.00E-179
SCE1572_3376	sce_2866	2.00E-93	7.00E-93
SCE1572_3377	sce_7568	4.00E-43	8.00E-48
SCE1572_3378	sce_7569	2.00E-22	2.00E-22
SCE1572_3379	sce_7570	2.00E-38	9.00E-42
SCE1572_3380	sce_2867	3.00E-94	3.00E-94
SCE1572_3381	sce_2868	3.00E-133	4.00E-133
SCE1572_3382	sce_2869	1.00E-136	4.00E-136
SCE1572_3383	sce_2870	5.00E-76	4.00E-73
SCE1572_3384	sce_2871	0.00E+00	0.00E+00
SCE1572_3385	sce_2872	4.00E-180	0.00E+00
SCE1572_3387	sce_2873	3.00E-42	8.00E-36
SCE1572_3388	sce_2874	3.00E-95	1.00E-84
SCE1572_3389	sce_2875	4.00E-49	3.00E-33
SCE1572_339	sce_7549	2.00E-108	6.00E-107
SCE1572_3390	sce_2876	4.00E-61	5.00E-61
SCE1572_3391	sce_2877	2.00E-67	4.00E-75
SCE1572_3392	sce_2878	0.00E+00	0.00E+00
SCE1572_3393	sce_2879	1.00E-39	1.00E-39
SCE1572_3394	sce_2880	8.00E-71	1.00E-71
SCE1572_3395	sce_2881	6.00E-15	4.00E-21
SCE1572_3396	sce_2882	9.00E-63	2.00E-54
SCE1572_3397	sce_2884	0.00E+00	0.00E+00
SCE1572_3398	sce_2885	4.00E-19	5.00E-19
SCE1572_34	sce_36	3.00E-145	4.00E-145
SCE1572_340	sce_7550	1.00E-97	2.00E-96
SCE1572_3400	sce_2887	0.00E+00	0.00E+00
SCE1572_3402	sce_2889	9.00E-116	2.00E-111
SCE1572_3403	sce_2890	5.00E-87	8.00E-92
SCE1572_3404	sce_2891	3.00E-118	3.00E-118
SCE1572_3405	sce_2892	0.00E+00	0.00E+00
SCE1572_3406	sce_2893	2.00E-172	2.00E-172
SCE1572_3407	sce_2894	0.00E+00	0.00E+00
SCE1572_3408	sce_2895	4.00E-144	4.00E-144
SCE1572_3409	sce_2896	1.00E-68	2.00E-97
SCE1572_341	sce_188	0.00E+00	0.00E+00
SCE1572_3410	sce_2897	2.00E-13	6.00E-16
SCE1572_3411	sce_2898	2.00E-82	7.00E-84
SCE1572_3412	sce_2899	7.00E-100	3.00E-101
SCE1572_3414	sce_2900	0.00E+00	0.00E+00
SCE1572_3415	sce_2901	5.00E-61	1.00E-63
SCE1572_3416	sce_2903	2.00E-59	2.00E-69
SCE1572_3417	sce_2904	2.00E-99	3.00E-107
SCE1572_3418	sce_2905	0.00E+00	2.00E-180
SCE1572_3419	sce_2906	0.00E+00	0.00E+00
SCE1572_342	sce_189	5.00E-120	5.00E-120
SCE1572_3420	sce_2907	0.00E+00	0.00E+00
SCE1572_3421	sce_2908	0.00E+00	0.00E+00
SCE1572_3422	sce_2909	0.00E+00	0.00E+00
SCE1572_3423	sce_2910	4.00E-09	6.00E-09
SCE1572_3424	sce_2911	3.00E-119	3.00E-119
SCE1572_3425	sce_2912	2.00E-20	1.00E-21
SCE1572_3427	sce_2913	0.00E+00	0.00E+00
SCE1572_3429	sce_2914	0.00E+00	0.00E+00

SCE1572_343	sce_190	0.00E+00	0.00E+00
SCE1572_3430	sce_2915	9.00E-61	4.00E-61
SCE1572_3431	sce_2916	1.00E-31	9.00E-30
SCE1572_3432	sce_2917	6.00E-87	3.00E-81
SCE1572_3433	sce_2918	1.00E-101	8.00E-130
SCE1572_3434	sce_2919	0.00E+00	0.00E+00
SCE1572_3435	sce_2920	8.00E-112	1.00E-111
SCE1572_3436	sce_2925	3.00E-107	7.00E-106
SCE1572_3437	sce_2931	2.00E-117	3.00E-117
SCE1572_3439	sce_2932	1.00E-163	1.00E-163
SCE1572_344	sce_2552	6.00E-128	2.00E-116
SCE1572_3441	sce_2933	3.00E-99	3.00E-93
SCE1572_3442	sce_2934	0.00E+00	0.00E+00
SCE1572_3443	sce_2935	2.00E-57	3.00E-69
SCE1572_3444	sce_5835	0.00E+00	0.00E+00
SCE1572_3444	sce_7640	0.00E+00	0.00E+00
SCE1572_3444	sce_388	0.00E+00	0.00E+00
SCE1572_3444	sce_3085	0.00E+00	0.00E+00
SCE1572_3444	sce_10204	0.00E+00	0.00E+00
SCE1572_3444	sce_4513	0.00E+00	0.00E+00
SCE1572_3444	sce_9557	0.00E+00	0.00E+00
SCE1572_3444	sce_7109	0.00E+00	0.00E+00
SCE1572_3444	sce_9920	0.00E+00	0.00E+00
SCE1572_3444	sce_6535	0.00E+00	0.00E+00
SCE1572_3444	sce_3003	0.00E+00	0.00E+00
SCE1572_3444	sce_7661	0.00E+00	0.00E+00
SCE1572_3444	sce_3917	0.00E+00	0.00E+00
SCE1572_3444	sce_6093	0.00E+00	0.00E+00
SCE1572_3444	sce_6107	0.00E+00	0.00E+00
SCE1572_3444	sce_6094	0.00E+00	0.00E+00
SCE1572_3444	sce_6092	0.00E+00	0.00E+00
SCE1572_3444	sce_8618	0.00E+00	0.00E+00
SCE1572_3444	sce_7643	0.00E+00	0.00E+00
SCE1572_3445	sce_225	0.00E+00	0.00E+00
SCE1572_3445	sce_2161	0.00E+00	0.00E+00
SCE1572_3445	sce_6235	0.00E+00	0.00E+00
SCE1572_3445	sce_8357	0.00E+00	0.00E+00
SCE1572_3445	sce_5276	0.00E+00	0.00E+00
SCE1572_3447	sce_2939	4.00E-53	5.00E-53
SCE1572_3448	sce_2940	0.00E+00	0.00E+00
SCE1572_3448	sce_5830	0.00E+00	0.00E+00
SCE1572_3448	sce_5827	0.00E+00	0.00E+00
SCE1572_3448	sce_5824	0.00E+00	0.00E+00
SCE1572_3448	sce_2589	0.00E+00	0.00E+00
SCE1572_3449	sce_2941	0.00E+00	0.00E+00
SCE1572_345	sce_2359	3.00E-85	3.00E-90
SCE1572_3450	sce_2942	9.00E-172	6.00E-178
SCE1572_3451	sce_2943	0.00E+00	0.00E+00
SCE1572_3452	sce_2944	0.00E+00	0.00E+00
SCE1572_3453	sce_2945	0.00E+00	0.00E+00
SCE1572_3455	sce_2946	0.00E+00	0.00E+00
SCE1572_3456	sce_2947	7.00E-179	2.00E-178
SCE1572_3458	sce_2948	8.00E-82	1.00E-81
SCE1572_3459	sce_2952	2.00E-34	2.00E-38
SCE1572_346	sce_3609	0.00E+00	0.00E+00
SCE1572_3460	sce_2953	1.00E-35	5.00E-36
SCE1572_3461	sce_2954	3.00E-70	8.00E-71
SCE1572_3462	sce_2955	3.00E-21	5.00E-15
SCE1572_3463	sce_2956	0.00E+00	0.00E+00
SCE1572_3464	sce_2957	0.00E+00	0.00E+00
SCE1572_3467	sce_2958	7.00E-91	7.00E-97
SCE1572_3468	sce_2960	4.00E-56	7.00E-68
SCE1572_3469	sce_2962	0.00E+00	0.00E+00
SCE1572_347	sce_9865	9.00E-115	1.00E-114
SCE1572_3470	sce_2963	0.00E+00	0.00E+00
SCE1572_3472	sce_2964	1.00E-99	2.00E-96
SCE1572_3473	sce_2965	4.00E-69	5.00E-62
SCE1572_3474	sce_2966	0.00E+00	0.00E+00
SCE1572_3475	sce_2967	8.00E-68	8.00E-104
SCE1572_3476	sce_2969	0.00E+00	0.00E+00
SCE1572_3480	sce_2972	0.00E+00	0.00E+00
SCE1572_3481	sce_2973	7.00E-22	1.00E-26
SCE1572_3483	sce_2974	7.00E-146	8.00E-155
SCE1572_3484	sce_2975	2.00E-69	1.00E-68
SCE1572_3490	sce_1329	7.00E-83	5.00E-96
SCE1572_3493	sce_2976	0.00E+00	0.00E+00

SCE1572_3494	sce_2977	2.00E-48	8.00E-49
SCE1572_3496	sce_2978	0.00E+00	0.00E+00
SCE1572_3497	sce_2980	2.00E-61	2.00E-61
SCE1572_3498	sce_2981	0.00E+00	0.00E+00
SCE1572_3499	sce_2982	3.00E-65	3.00E-65
SCE1572_35	sce_37	5.00E-127	2.00E-124
SCE1572_350	sce_9998	6.00E-159	0.00E+00
SCE1572_3500	sce_2983	0.00E+00	0.00E+00
SCE1572_3501	sce_2984	0.00E+00	0.00E+00
SCE1572_3502	sce_2985	7.00E-119	5.00E-127
SCE1572_3503	sce_2986	5.00E-135	2.00E-138
SCE1572_3504	sce_2987	1.00E-145	1.00E-153
SCE1572_3505	sce_2988	6.00E-144	6.00E-144
SCE1572_3506	sce_2989	1.00E-107	2.00E-107
SCE1572_3507	sce_2990	1.00E-52	4.00E-50
SCE1572_3508	sce_2991	8.00E-61	3.00E-68
SCE1572_3509	sce_2992	0.00E+00	0.00E+00
SCE1572_3510	sce_2993	2.00E-62	4.00E-72
SCE1572_3511	sce_2994	2.00E-125	7.00E-114
SCE1572_3513	sce_7498	0.00E+00	0.00E+00
SCE1572_3514	sce_7500	8.00E-136	6.00E-128
SCE1572_3516	sce_7492	9.00E-143	4.00E-145
SCE1572_3517	sce_8421	1.00E-46	1.00E-46
SCE1572_352	sce_193	6.00E-75	1.00E-69
SCE1572_3521	sce_1043	1.00E-139	6.00E-131
SCE1572_3523	sce_3138	2.00E-51	4.00E-60
SCE1572_3525	sce_7491	0.00E+00	0.00E+00
SCE1572_3528	sce_2106	7.00E-57	3.00E-52
SCE1572_3529	sce_7067	0.00E+00	0.00E+00
SCE1572_353	sce_194	2.00E-75	6.00E-78
SCE1572_3533	sce_7120	0.00E+00	0.00E+00
SCE1572_3534	sce_8653	1.00E-20	1.00E-20
SCE1572_354	sce_195	1.00E-142	4.00E-151
SCE1572_3549	sce_5143	0.00E+00	0.00E+00
SCE1572_355	sce_196	7.00E-149	1.00E-140
SCE1572_3550	sce_1370	0.00E+00	0.00E+00
SCE1572_3552	sce_6024	2.00E-46	6.00E-49
SCE1572_3553	sce_6183	7.00E-21	7.00E-28
SCE1572_3554	sce_10012	1.00E-108	1.00E-104
SCE1572_3555	sce_5751	0.00E+00	0.00E+00
SCE1572_3555	sce_5746	0.00E+00	0.00E+00
SCE1572_3555	sce_9665	0.00E+00	0.00E+00
SCE1572_3555	sce_5226	0.00E+00	0.00E+00
SCE1572_3555	sce_5403	0.00E+00	0.00E+00
SCE1572_3555	sce_7969	0.00E+00	0.00E+00
SCE1572_3555	sce_4073	0.00E+00	0.00E+00
SCE1572_3555	sce_7154	0.00E+00	0.00E+00
SCE1572_3555	sce_2053	0.00E+00	0.00E+00
SCE1572_3555	sce_9757	0.00E+00	0.00E+00
SCE1572_3555	sce_9954	0.00E+00	0.00E+00
SCE1572_3555	sce_6712	0.00E+00	0.00E+00
SCE1572_3555	sce_3750	0.00E+00	0.00E+00
SCE1572_3555	sce_1803	0.00E+00	0.00E+00
SCE1572_3564	sce_5229	0.00E+00	0.00E+00
SCE1572_3564	sce_6712	0.00E+00	0.00E+00
SCE1572_3564	sce_3750	0.00E+00	0.00E+00
SCE1572_3564	sce_9954	0.00E+00	0.00E+00
SCE1572_3564	sce_5403	0.00E+00	0.00E+00
SCE1572_3564	sce_774	0.00E+00	0.00E+00
SCE1572_3564	sce_9665	0.00E+00	0.00E+00
SCE1572_3568	sce_5229	0.00E+00	0.00E+00
SCE1572_3568	sce_6712	0.00E+00	0.00E+00
SCE1572_3568	sce_3750	0.00E+00	0.00E+00
SCE1572_3568	sce_9954	0.00E+00	0.00E+00
SCE1572_3568	sce_5403	0.00E+00	0.00E+00
SCE1572_3568	sce_9665	0.00E+00	0.00E+00
SCE1572_3568	sce_774	0.00E+00	0.00E+00
SCE1572_3568	sce_5226	0.00E+00	0.00E+00
SCE1572_357	sce_198	0.00E+00	0.00E+00
SCE1572_3576	sce_6709	3.00E-40	2.00E-40
SCE1572_3577	sce_170	6.00E-105	1.00E-104
SCE1572_3579	sce_6216	4.00E-49	3.00E-49
SCE1572_358	sce_199	0.00E+00	0.00E+00
SCE1572_3581	sce_4603	4.00E-90	1.00E-81
SCE1572_3585	sce_9857	2.00E-86	2.00E-86
SCE1572_359	sce_200	0.00E+00	0.00E+00

SCE1572_3592	sce_7649	4.00E-63	8.00E-57
SCE1572_3593	sce_7648	0.00E+00	0.00E+00
SCE1572_3594	sce_7651	1.00E-117	4.00E-132
SCE1572_3599	sce_1701	3.00E-21	3.00E-21
SCE1572_36	sce_38	0.00E+00	0.00E+00
SCE1572_360	sce_201	2.00E-73	2.00E-73
SCE1572_3604	sce_1597	0.00E+00	0.00E+00
SCE1572_3605	sce_1598	0.00E+00	0.00E+00
SCE1572_3605	sce_1599	0.00E+00	0.00E+00
SCE1572_3607	sce_4487	0.00E+00	0.00E+00
SCE1572_3608	sce_3024	1.00E-148	9.00E-153
SCE1572_3609	sce_3025	0.00E+00	0.00E+00
SCE1572_3614	sce_6533	1.00E-22	4.00E-33
SCE1572_3619	sce_2155	0.00E+00	0.00E+00
SCE1572_3620	sce_2154	3.00E-60	1.00E-46
SCE1572_3621	sce_2153	6.00E-46	9.00E-46
SCE1572_3622	sce_2152	0.00E+00	0.00E+00
SCE1572_3623	sce_2151	2.00E-63	4.00E-63
SCE1572_3624	sce_2150	2.00E-30	2.00E-30
SCE1572_3625	sce_2149	5.00E-95	5.00E-95
SCE1572_3626	sce_6850	3.00E-158	1.00E-139
SCE1572_3627	sce_6851	3.00E-94	8.00E-108
SCE1572_3628	sce_1042	2.00E-63	2.00E-63
SCE1572_3629	sce_7856	1.00E-45	6.00E-44
SCE1572_3630	sce_6065	0.00E+00	0.00E+00
SCE1572_3631	sce_6047	3.00E-159	7.00E-166
SCE1572_3632	sce_6048	0.00E+00	0.00E+00
SCE1572_3633	sce_6046	7.00E-76	1.00E-72
SCE1572_3634	sce_2425	2.00E-140	2.00E-131
SCE1572_3639	sce_6070	1.00E-56	2.00E-56
SCE1572_3641	sce_6018	2.00E-161	4.00E-167
SCE1572_3646	sce_4691	2.00E-114	1.00E-105
SCE1572_3647	sce_4692	4.00E-102	8.00E-101
SCE1572_3648	sce_4693	4.00E-153	5.00E-154
SCE1572_3650	sce_2436	1.00E-76	8.00E-77
SCE1572_3654	sce_3043	7.00E-75	1.00E-74
SCE1572_3656	sce_5240	7.00E-16	1.00E-17
SCE1572_3658	sce_5238	2.00E-66	4.00E-101
SCE1572_367	sce_203	1.00E-93	9.00E-104
SCE1572_3670	sce_2337	7.00E-117	3.00E-118
SCE1572_3675	sce_8689	1.00E-09	1.00E-08
SCE1572_3677	sce_7357	9.00E-70	2.00E-58
SCE1572_3678	sce_7356	2.00E-71	2.00E-71
SCE1572_368	sce_204	0.00E+00	0.00E+00
SCE1572_3680	sce_6700	1.00E-20	9.00E-20
SCE1572_3688	sce_1325	0.00E+00	0.00E+00
SCE1572_3689	sce_3046	2.00E-115	7.00E-126
SCE1572_3691	sce_3794	0.00E+00	0.00E+00
SCE1572_3696	sce_3047	1.00E-06	6.00E-10
SCE1572_37	sce_39	5.00E-97	2.00E-90
SCE1572_3700	sce_3049	0.00E+00	0.00E+00
SCE1572_3701	sce_3051	6.00E-78	6.00E-78
SCE1572_3702	sce_3052	0.00E+00	0.00E+00
SCE1572_3703	sce_3053	2.00E-142	4.00E-150
SCE1572_3704	sce_10152	2.00E-27	1.00E-27
SCE1572_3707	sce_7655	4.00E-114	6.00E-113
SCE1572_3710	sce_8390	8.00E-40	8.00E-40
SCE1572_3711	sce_8388	7.00E-23	7.00E-23
SCE1572_3712	sce_3061	2.00E-137	3.00E-138
SCE1572_3717	sce_7923	2.00E-14	8.00E-16
SCE1572_3719	sce_3062	9.00E-125	1.00E-125
SCE1572_3720	sce_3065	0.00E+00	0.00E+00
SCE1572_3722	sce_3066	0.00E+00	0.00E+00
SCE1572_3725	sce_7838	3.00E-124	2.00E-121
SCE1572_373	sce_205	9.00E-126	2.00E-134
SCE1572_3731	sce_3071	0.00E+00	0.00E+00
SCE1572_3732	sce_3072	0.00E+00	0.00E+00
SCE1572_3734	sce_5891	3.00E-93	5.00E-101
SCE1572_3738	sce_3078	7.00E-62	2.00E-98
SCE1572_374	sce_206	0.00E+00	0.00E+00
SCE1572_3745	sce_3083	4.00E-57	2.00E-129
SCE1572_3746	sce_3084	6.00E-84	6.00E-83
SCE1572_3748	sce_3085	0.00E+00	0.00E+00
SCE1572_3748	sce_7640	0.00E+00	0.00E+00
SCE1572_3748	sce_388	0.00E+00	0.00E+00
SCE1572_3748	sce_5835	0.00E+00	0.00E+00

SCE1572_3748	sce_10204	0.00E+00	0.00E+00
SCE1572_3748	sce_3003	0.00E+00	0.00E+00
SCE1572_3748	sce_9557	0.00E+00	0.00E+00
SCE1572_3748	sce_7109	0.00E+00	0.00E+00
SCE1572_3748	sce_7661	0.00E+00	0.00E+00
SCE1572_3748	sce_4513	0.00E+00	0.00E+00
SCE1572_3748	sce_9920	0.00E+00	0.00E+00
SCE1572_3748	sce_3917	0.00E+00	0.00E+00
SCE1572_3748	sce_6535	0.00E+00	0.00E+00
SCE1572_3748	sce_6093	0.00E+00	0.00E+00
SCE1572_3749	sce_3086	3.00E-48	2.00E-55
SCE1572_3750	sce_3087	4.00E-88	2.00E-90
SCE1572_3752	sce_9196	6.00E-49	6.00E-49
SCE1572_3755	sce_4641	0.00E+00	0.00E+00
SCE1572_3758	sce_8592	0.00E+00	0.00E+00
SCE1572_3758	sce_3937	0.00E+00	0.00E+00
SCE1572_3759	sce_8592	0.00E+00	0.00E+00
SCE1572_3759	sce_3937	0.00E+00	0.00E+00
SCE1572_3761	sce_3092	0.00E+00	0.00E+00
SCE1572_3762	sce_3093	0.00E+00	0.00E+00
SCE1572_3763	sce_3100	2.00E-51	1.00E-53
SCE1572_3764	sce_3101	2.00E-145	7.00E-149
SCE1572_3765	sce_3102	0.00E+00	0.00E+00
SCE1572_3766	sce_3103	5.00E-176	4.00E-164
SCE1572_3767	sce_3104	2.00E-126	1.00E-128
SCE1572_3768	sce_3105	6.00E-161	7.00E-163
SCE1572_3769	sce_3106	5.00E-50	3.00E-57
SCE1572_3771	sce_3107	5.00E-149	4.00E-159
SCE1572_3772	sce_3108	1.00E-159	1.00E-159
SCE1572_3773	sce_3109	0.00E+00	0.00E+00
SCE1572_3774	sce_3110	0.00E+00	0.00E+00
SCE1572_3775	sce_3111	2.00E-71	5.00E-72
SCE1572_3776	sce_3112	0.00E+00	0.00E+00
SCE1572_3777	sce_3113	3.00E-140	2.00E-154
SCE1572_3778	sce_3114	2.00E-173	9.00E-174
SCE1572_3779	sce_3115	0.00E+00	0.00E+00
SCE1572_3780	sce_3116	5.00E-41	5.00E-41
SCE1572_3781	sce_3117	2.00E-157	8.00E-149
SCE1572_3782	sce_3118	9.00E-156	9.00E-148
SCE1572_3784	sce_3119	8.00E-84	7.00E-74
SCE1572_3785	sce_3120	1.00E-48	1.00E-48
SCE1572_3786	sce_3121	0.00E+00	0.00E+00
SCE1572_3786	sce_6618	0.00E+00	0.00E+00
SCE1572_3787	sce_3122	1.00E-97	3.00E-91
SCE1572_3788	sce_3123	2.00E-143	2.00E-143
SCE1572_3789	sce_3124	9.00E-152	9.00E-152
SCE1572_379	sce_211	8.00E-173	3.00E-162
SCE1572_3790	sce_3126	0.00E+00	0.00E+00
SCE1572_3791	sce_3127	0.00E+00	0.00E+00
SCE1572_3793	sce_3129	0.00E+00	0.00E+00
SCE1572_3794	sce_3130	6.00E-53	4.00E-44
SCE1572_3795	sce_3131	1.00E-146	4.00E-155
SCE1572_3796	sce_3132	0.00E+00	0.00E+00
SCE1572_3797	sce_3133	0.00E+00	0.00E+00
SCE1572_3798	sce_3134	0.00E+00	0.00E+00
SCE1572_3799	sce_3135	0.00E+00	0.00E+00
SCE1572_38	sce_40	1.00E-179	0.00E+00
SCE1572_3801	sce_5620	1.00E-53	1.00E-53
SCE1572_3803	sce_3137	0.00E+00	0.00E+00
SCE1572_3805	sce_3139	1.00E-97	9.00E-75
SCE1572_3807	sce_5178	0.00E+00	0.00E+00
SCE1572_3808	sce_3140	3.00E-178	0.00E+00
SCE1572_3809	sce_3141	4.00E-56	2.00E-70
SCE1572_381	sce_6106	1.00E-16	2.00E-17
SCE1572_3815	sce_2107	1.00E-125	1.00E-123
SCE1572_3817	sce_4644	9.00E-87	1.00E-86
SCE1572_3818	sce_3145	0.00E+00	0.00E+00
SCE1572_382	sce_212	1.00E-47	8.00E-46
SCE1572_3820	sce_3147	7.00E-159	5.00E-141
SCE1572_3821	sce_3148	0.00E+00	0.00E+00
SCE1572_3821	sce_1814	0.00E+00	0.00E+00
SCE1572_3821	sce_9222	0.00E+00	0.00E+00
SCE1572_3822	sce_3149	3.00E-15	2.00E-12
SCE1572_3823	sce_3151	9.00E-61	8.00E-67
SCE1572_3824	sce_3152	1.00E-169	7.00E-158
SCE1572_3826	sce_3153	0.00E+00	0.00E+00

SCE1572_3827	sce_3154	6.00E-19	9.00E-19
SCE1572_3828	sce_3155	3.00E-117	3.00E-117
SCE1572_3829	sce_3156	0.00E+00	4.00E-142
SCE1572_383	sce_7076	1.00E-144	2.00E-140
SCE1572_3830	sce_3157	2.00E-86	9.00E-87
SCE1572_3831	sce_3158	4.00E-101	1.00E-105
SCE1572_3832	sce_3159	2.00E-145	1.00E-150
SCE1572_3833	sce_3161	5.00E-126	2.00E-116
SCE1572_3835	sce_7642	2.00E-169	0.00E+00
SCE1572_3839	sce_3162	8.00E-152	8.00E-152
SCE1572_3840	sce_3163	2.00E-88	2.00E-88
SCE1572_3841	sce_3164	9.00E-177	0.00E+00
SCE1572_3842	sce_3165	2.00E-98	2.00E-98
SCE1572_3846	sce_2595	7.00E-20	8.00E-20
SCE1572_3848	sce_3166	9.00E-82	4.00E-82
SCE1572_3849	sce_3167	3.00E-82	1.00E-87
SCE1572_3850	sce_3168	6.00E-112	6.00E-112
SCE1572_3851	sce_3169	2.00E-109	2.00E-109
SCE1572_3852	sce_3170	3.00E-145	1.00E-145
SCE1572_3854	sce_3171	2.00E-132	2.00E-120
SCE1572_3855	sce_3172	2.00E-78	2.00E-85
SCE1572_3856	sce_3180	2.00E-83	4.00E-141
SCE1572_3857	sce_3181	0.00E+00	0.00E+00
SCE1572_3858	sce_3182	2.00E-109	5.00E-129
SCE1572_3859	sce_3183	0.00E+00	0.00E+00
SCE1572_3860	sce_3184	1.00E-102	3.00E-106
SCE1572_3861	sce_3185	0.00E+00	0.00E+00
SCE1572_3861	sce_5602	0.00E+00	0.00E+00
SCE1572_3861	sce_5033	0.00E+00	0.00E+00
SCE1572_3863	sce_3186	0.00E+00	0.00E+00
SCE1572_3864	sce_3187	0.00E+00	0.00E+00
SCE1572_3866	sce_3188	3.00E-86	2.00E-88
SCE1572_3867	sce_3189	2.00E-69	4.00E-65
SCE1572_3868	sce_3190	3.00E-153	2.00E-160
SCE1572_3869	sce_3191	2.00E-78	4.00E-76
SCE1572_387	sce_213	9.00E-20	3.00E-24
SCE1572_3870	sce_3192	1.00E-56	4.00E-66
SCE1572_3871	sce_3194	2.00E-104	6.00E-107
SCE1572_3872	sce_3195	0.00E+00	0.00E+00
SCE1572_3873	sce_3196	4.00E-151	7.00E-162
SCE1572_3874	sce_3197	0.00E+00	0.00E+00
SCE1572_3875	sce_3198	8.00E-89	7.00E-95
SCE1572_3876	sce_3199	6.00E-44	6.00E-44
SCE1572_3877	sce_3200	4.00E-11	2.00E-12
SCE1572_3878	sce_3201	0.00E+00	0.00E+00
SCE1572_3879	sce_3202	2.00E-174	2.00E-174
SCE1572_388	sce_214	8.00E-52	2.00E-56
SCE1572_3880	sce_3204	0.00E+00	0.00E+00
SCE1572_3881	sce_3205	2.00E-95	9.00E-94
SCE1572_3882	sce_3206	5.00E-119	1.00E-142
SCE1572_3883	sce_3207	2.00E-62	2.00E-69
SCE1572_3884	sce_3208	2.00E-86	2.00E-86
SCE1572_3885	sce_3209	2.00E-76	2.00E-76
SCE1572_3886	sce_3210	6.00E-101	1.00E-98
SCE1572_3887	sce_3211	0.00E+00	0.00E+00
SCE1572_3888	sce_3212	0.00E+00	0.00E+00
SCE1572_3889	sce_3213	4.00E-101	2.00E-105
SCE1572_389	sce_5187	8.00E-20	2.00E-19
SCE1572_3890	sce_3215	0.00E+00	0.00E+00
SCE1572_3891	sce_3216	0.00E+00	0.00E+00
SCE1572_3892	sce_3221	2.00E-133	5.00E-139
SCE1572_3893	sce_3222	3.00E-65	2.00E-60
SCE1572_3894	sce_3223	3.00E-86	2.00E-87
SCE1572_3895	sce_3224	2.00E-82	3.00E-81
SCE1572_3896	sce_3225	7.00E-135	3.00E-152
SCE1572_3897	sce_3226	1.00E-131	5.00E-144
SCE1572_3898	sce_3227	0.00E+00	0.00E+00
SCE1572_3899	sce_3228	0.00E+00	7.00E-178
SCE1572_39	sce_41	0.00E+00	0.00E+00
SCE1572_39	sce_370	0.00E+00	0.00E+00
SCE1572_390	sce_5188	9.00E-53	1.00E-56
SCE1572_3900	sce_3229	5.00E-102	3.00E-102
SCE1572_3901	sce_3230	8.00E-69	6.00E-78
SCE1572_3903	sce_3232	0.00E+00	0.00E+00
SCE1572_3905	sce_3234	0.00E+00	0.00E+00
SCE1572_3906	sce_3235	0.00E+00	0.00E+00

SCE1572_3907	sce_3236	4.00E-118	1.00E-131
SCE1572_3908	sce_3238	1.00E-13	1.00E-22
SCE1572_3909	sce_3239	0.00E+00	0.00E+00
SCE1572_3910	sce_3240	4.00E-113	9.00E-111
SCE1572_3911	sce_3241	7.00E-86	7.00E-86
SCE1572_3912	sce_3242	9.00E-29	1.00E-14
SCE1572_3913	sce_3243	0.00E+00	0.00E+00
SCE1572_3914	sce_3245	7.00E-129	1.00E-136
SCE1572_3915	sce_3246	2.00E-103	3.00E-80
SCE1572_3916	sce_3247	1.00E-136	7.00E-148
SCE1572_3917	sce_3249	2.00E-127	2.00E-134
SCE1572_3918	sce_3250	2.00E-85	2.00E-85
SCE1572_3919	sce_3251	1.00E-68	5.00E-86
SCE1572_392	sce_215	1.00E-51	3.00E-57
SCE1572_3920	sce_3252	2.00E-119	9.00E-119
SCE1572_3921	sce_3254	1.00E-114	9.00E-98
SCE1572_3922	sce_3255	5.00E-90	6.00E-96
SCE1572_3923	sce_3256	0.00E+00	0.00E+00
SCE1572_3924	sce_3257	3.00E-84	1.00E-84
SCE1572_3925	sce_3258	9.00E-150	3.00E-159
SCE1572_3926	sce_3259	3.00E-142	6.00E-155
SCE1572_3927	sce_3260	3.00E-42	3.00E-42
SCE1572_3928	sce_3261	0.00E+00	0.00E+00
SCE1572_3929	sce_3262	0.00E+00	0.00E+00
SCE1572_393	sce_2460	1.00E-22	7.00E-20
SCE1572_3930	sce_3263	3.00E-115	7.00E-123
SCE1572_3931	sce_3264	3.00E-141	3.00E-141
SCE1572_3932	sce_3265	1.00E-140	1.00E-140
SCE1572_3933	sce_3267	9.00E-164	6.00E-167
SCE1572_3935	sce_3268	0.00E+00	0.00E+00
SCE1572_3937	sce_3270	7.00E-103	2.00E-94
SCE1572_3938	sce_3271	4.00E-128	5.00E-131
SCE1572_3939	sce_3272	2.00E-42	5.00E-38
SCE1572_394	sce_217	0.00E+00	0.00E+00
SCE1572_3940	sce_3273	6.00E-99	7.00E-99
SCE1572_3941	sce_3274	0.00E+00	0.00E+00
SCE1572_3942	sce_3275	0.00E+00	0.00E+00
SCE1572_3943	sce_3276	0.00E+00	0.00E+00
SCE1572_3944	sce_3277	3.00E-27	3.00E-22
SCE1572_3945	sce_3278	0.00E+00	0.00E+00
SCE1572_3946	sce_3279	3.00E-174	2.00E-166
SCE1572_3947	sce_3281	0.00E+00	0.00E+00
SCE1572_3947	sce_3280	0.00E+00	0.00E+00
SCE1572_3948	sce_3282	0.00E+00	0.00E+00
SCE1572_3949	sce_3283	0.00E+00	0.00E+00
SCE1572_395	sce_218	4.00E-149	1.00E-132
SCE1572_3951	sce_3285	0.00E+00	0.00E+00
SCE1572_3952	sce_3286	2.00E-101	5.00E-102
SCE1572_3953	sce_3287	2.00E-96	2.00E-93
SCE1572_3958	sce_8665	2.00E-07	3.00E-14
SCE1572_3959	sce_3289	0.00E+00	0.00E+00
SCE1572_3960	sce_2531	4.00E-25	9.00E-24
SCE1572_3961	sce_3291	2.00E-176	5.00E-177
SCE1572_3962	sce_3292	0.00E+00	0.00E+00
SCE1572_3963	sce_3293	0.00E+00	0.00E+00
SCE1572_3964	sce_3294	0.00E+00	0.00E+00
SCE1572_3965	sce_3295	2.00E-68	1.00E-64
SCE1572_3967	sce_10164	3.00E-101	1.00E-101
SCE1572_3968	sce_3296	0.00E+00	0.00E+00
SCE1572_3969	sce_3297	0.00E+00	0.00E+00
SCE1572_3970	sce_3299	8.00E-118	8.00E-118
SCE1572_3971	sce_3300	0.00E+00	0.00E+00
SCE1572_3972	sce_3301	2.00E-38	2.00E-38
SCE1572_3973	sce_3302	1.00E-171	7.00E-159
SCE1572_3974	sce_3303	2.00E-73	7.00E-74
SCE1572_3975	sce_3304	6.00E-62	1.00E-73
SCE1572_3976	sce_3305	2.00E-131	2.00E-131
SCE1572_3977	sce_3306	4.00E-137	2.00E-137
SCE1572_3978	sce_3307	1.00E-138	5.00E-132
SCE1572_3979	sce_3308	2.00E-145	8.00E-152
SCE1572_398	sce_219	0.00E+00	0.00E+00
SCE1572_3980	sce_3309	0.00E+00	0.00E+00
SCE1572_3981	sce_3310	0.00E+00	0.00E+00
SCE1572_3982	sce_3311	5.00E-118	6.00E-118
SCE1572_3983	sce_3314	4.00E-84	4.00E-91
SCE1572_3984	sce_3315	1.00E-124	2.00E-122

SCE1572_3985	sce_3316	0.00E+00	0.00E+00
SCE1572_3986	sce_3317	7.00E-140	2.00E-136
SCE1572_3987	sce_3318	6.00E-72	5.00E-72
SCE1572_3988	sce_3319	5.00E-67	1.00E-71
SCE1572_3989	sce_3320	2.00E-111	7.00E-103
SCE1572_3990	sce_3321	0.00E+00	0.00E+00
SCE1572_3991	sce_3322	0.00E+00	0.00E+00
SCE1572_3992	sce_3323	4.00E-07	3.00E-06
SCE1572_3993	sce_3324	1.00E-22	3.00E-18
SCE1572_3997	sce_3327	0.00E+00	0.00E+00
SCE1572_3998	sce_3328	0.00E+00	0.00E+00
SCE1572_3999	sce_3329	0.00E+00	0.00E+00
SCE1572_4	sce_4	1.00E-76	1.00E-83
SCE1572_400	sce_8410	2.00E-45	5.00E-35
SCE1572_4000	sce_3330	2.00E-144	2.00E-136
SCE1572_4001	sce_3331	0.00E+00	0.00E+00
SCE1572_4002	sce_3332	0.00E+00	0.00E+00
SCE1572_4003	sce_3333	1.00E-77	1.00E-77
SCE1572_4005	sce_3335	2.00E-56	4.00E-56
SCE1572_4006	sce_3336	0.00E+00	0.00E+00
SCE1572_4007	sce_3337	0.00E+00	0.00E+00
SCE1572_4008	sce_3338	5.00E-49	5.00E-60
SCE1572_4009	sce_3339	0.00E+00	0.00E+00
SCE1572_4010	sce_3340	0.00E+00	0.00E+00
SCE1572_4011	sce_3341	8.00E-113	7.00E-111
SCE1572_4012	sce_3342	0.00E+00	0.00E+00
SCE1572_4013	sce_3343	6.00E-172	1.00E-155
SCE1572_4015	sce_3344	3.00E-51	5.00E-53
SCE1572_4017	sce_3345	9.00E-116	1.00E-113
SCE1572_4018	sce_3346	0.00E+00	0.00E+00
SCE1572_4019	sce_3347	2.00E-123	5.00E-111
SCE1572_4020	sce_3348	7.00E-95	1.00E-86
SCE1572_4021	sce_3349	2.00E-112	2.00E-112
SCE1572_4022	sce_3350	0.00E+00	0.00E+00
SCE1572_4023	sce_3351	5.00E-78	2.00E-80
SCE1572_4024	sce_3352	0.00E+00	0.00E+00
SCE1572_4025	sce_3353	2.00E-150	6.00E-138
SCE1572_4026	sce_3354	0.00E+00	0.00E+00
SCE1572_4027	sce_3355	0.00E+00	0.00E+00
SCE1572_4028	sce_3356	2.00E-108	2.00E-108
SCE1572_4029	sce_3357	1.00E-92	1.00E-92
SCE1572_4030	sce_3358	4.00E-116	5.00E-120
SCE1572_4031	sce_3360	1.00E-115	1.00E-115
SCE1572_4032	sce_3361	1.00E-97	1.00E-97
SCE1572_4034	sce_3362	2.00E-99	2.00E-99
SCE1572_4035	sce_3363	6.00E-165	5.00E-165
SCE1572_4036	sce_3364	8.00E-107	1.00E-119
SCE1572_4037	sce_3365	2.00E-65	3.00E-59
SCE1572_4038	sce_3366	0.00E+00	0.00E+00
SCE1572_404	sce_224	9.00E-155	9.00E-155
SCE1572_4041	sce_3368	0.00E+00	0.00E+00
SCE1572_4043	sce_3369	0.00E+00	0.00E+00
SCE1572_4044	sce_3370	4.00E-104	4.00E-96
SCE1572_4045	sce_3372	0.00E+00	0.00E+00
SCE1572_4047	sce_3374	9.00E-164	7.00E-158
SCE1572_4048	sce_5533	1.00E-66	7.00E-59
SCE1572_4049	sce_3375	9.00E-143	3.00E-143
SCE1572_405	sce_225	0.00E+00	0.00E+00
SCE1572_405	sce_2161	0.00E+00	0.00E+00
SCE1572_405	sce_6235	0.00E+00	0.00E+00
SCE1572_405	sce_8357	0.00E+00	0.00E+00
SCE1572_405	sce_5276	0.00E+00	0.00E+00
SCE1572_4050	sce_3376	0.00E+00	0.00E+00
SCE1572_4050	sce_5364	0.00E+00	0.00E+00
SCE1572_4050	sce_4711	0.00E+00	0.00E+00
SCE1572_4051	sce_3377	6.00E-95	6.00E-95
SCE1572_4052	sce_3378	9.00E-150	1.00E-157
SCE1572_4053	sce_3379	1.00E-173	5.00E-166
SCE1572_4054	sce_3380	4.00E-126	4.00E-126
SCE1572_4055	sce_3381	0.00E+00	0.00E+00
SCE1572_4056	sce_3382	1.00E-89	2.00E-107
SCE1572_4058	sce_3383	1.00E-99	1.00E-75
SCE1572_4059	sce_3384	2.00E-174	0.00E+00
SCE1572_4060	sce_3385	9.00E-75	9.00E-75
SCE1572_4061	sce_3386	1.00E-132	5.00E-137
SCE1572_4062	sce_3387	2.00E-124	1.00E-124

SCE1572_4063	sce_3388	2.00E-119	2.00E-133
SCE1572_4065	sce_3390	0.00E+00	0.00E+00
SCE1572_4066	sce_3391	0.00E+00	0.00E+00
SCE1572_4067	sce_3392	2.00E-66	1.00E-65
SCE1572_4068	sce_3393	0.00E+00	0.00E+00
SCE1572_407	sce_226	4.00E-82	4.00E-82
SCE1572_4070	sce_3394	1.00E-79	2.00E-87
SCE1572_4071	sce_3396	2.00E-55	4.00E-50
SCE1572_4072	sce_3397	0.00E+00	0.00E+00
SCE1572_4073	sce_3398	0.00E+00	0.00E+00
SCE1572_4074	sce_3399	0.00E+00	0.00E+00
SCE1572_4075	sce_3401	4.00E-60	4.00E-47
SCE1572_4077	sce_3403	1.00E-70	1.00E-70
SCE1572_4078	sce_3404	2.00E-114	2.00E-122
SCE1572_408	sce_227	0.00E+00	0.00E+00
SCE1572_4080	sce_3406	0.00E+00	0.00E+00
SCE1572_4084	sce_3407	1.00E-94	1.00E-105
SCE1572_4085	sce_3410	1.00E-47	3.00E-48
SCE1572_4086	sce_3412	0.00E+00	0.00E+00
SCE1572_4089	sce_3415	2.00E-175	1.00E-170
SCE1572_409	sce_5625	2.00E-116	1.00E-121
SCE1572_4090	sce_3416	1.00E-126	4.00E-135
SCE1572_4091	sce_3417	2.00E-147	1.00E-151
SCE1572_4092	sce_3418	2.00E-92	2.00E-92
SCE1572_4093	sce_3419	2.00E-125	1.00E-138
SCE1572_4095	sce_3420	0.00E+00	0.00E+00
SCE1572_4098	sce_3422	9.00E-53	2.00E-52
SCE1572_41	sce_42	4.00E-62	1.00E-57
SCE1572_410	sce_228	2.00E-39	1.00E-48
SCE1572_4100	sce_3438	0.00E+00	0.00E+00
SCE1572_4101	sce_3439	0.00E+00	0.00E+00
SCE1572_4105	sce_3459	6.00E-135	6.00E-142
SCE1572_411	sce_229	0.00E+00	0.00E+00
SCE1572_411	sce_805	0.00E+00	0.00E+00
SCE1572_4110	sce_3461	1.00E-140	2.00E-141
SCE1572_4111	sce_3462	1.00E-102	1.00E-102
SCE1572_4112	sce_3463	2.00E-82	2.00E-95
SCE1572_4113	sce_3464	5.00E-122	6.00E-128
SCE1572_4115	sce_3465	4.00E-168	3.00E-157
SCE1572_4116	sce_7384	1.00E-67	1.00E-70
SCE1572_4118	sce_3468	2.00E-121	3.00E-124
SCE1572_4119	sce_3469	4.00E-103	4.00E-103
SCE1572_412	sce_230	8.00E-75	2.00E-83
SCE1572_4122	sce_3467	6.00E-74	2.00E-74
SCE1572_4125	sce_3471	1.00E-128	2.00E-180
SCE1572_4128	sce_3472	4.00E-65	5.00E-65
SCE1572_4129	sce_3473	0.00E+00	0.00E+00
SCE1572_413	sce_231	0.00E+00	0.00E+00
SCE1572_4130	sce_3474	6.00E-138	6.00E-138
SCE1572_4131	sce_3475	3.00E-21	5.00E-19
SCE1572_4132	sce_3476	0.00E+00	0.00E+00
SCE1572_4133	sce_3477	6.00E-73	2.00E-83
SCE1572_4138	sce_3483	2.00E-129	8.00E-139
SCE1572_4139	sce_3484	2.00E-105	7.00E-114
SCE1572_414	sce_232	6.00E-40	1.00E-46
SCE1572_4140	sce_3485	1.00E-178	1.00E-174
SCE1572_4141	sce_3486	7.00E-153	1.00E-139
SCE1572_4147	sce_7614	1.00E-82	1.00E-76
SCE1572_4148	sce_7615	2.00E-39	2.00E-39
SCE1572_4151	sce_3080	1.00E-173	2.00E-153
SCE1572_4152	sce_8359	1.00E-80	1.00E-73
SCE1572_4155	sce_2035	1.00E-104	6.00E-101
SCE1572_4156	sce_3492	0.00E+00	0.00E+00
SCE1572_4158	sce_2442	8.00E-66	2.00E-63
SCE1572_4166	sce_9855	2.00E-20	7.00E-18
SCE1572_4171	sce_3495	0.00E+00	0.00E+00
SCE1572_4172	sce_3496	0.00E+00	0.00E+00
SCE1572_4173	sce_3497	0.00E+00	0.00E+00
SCE1572_4174	sce_3498	3.00E-48	3.00E-54
SCE1572_4179	sce_3503	2.00E-44	9.00E-65
SCE1572_418	sce_9373	0.00E+00	0.00E+00
SCE1572_418	sce_4062	0.00E+00	0.00E+00
SCE1572_4180	sce_3504	5.00E-55	1.00E-51
SCE1572_4181	sce_3505	0.00E+00	0.00E+00
SCE1572_4184	sce_3506	4.00E-78	1.00E-78
SCE1572_4185	sce_3507	0.00E+00	0.00E+00

SCE1572_4186	sce_3508	6.00E-57	9.00E-57
SCE1572_4187	sce_3509	0.00E+00	0.00E+00
SCE1572_4188	sce_3510	0.00E+00	0.00E+00
SCE1572_4189	sce_3511	0.00E+00	0.00E+00
SCE1572_419	sce_233	0.00E+00	0.00E+00
SCE1572_4190	sce_3512	0.00E+00	0.00E+00
SCE1572_4191	sce_3513	0.00E+00	0.00E+00
SCE1572_4192	sce_3514	0.00E+00	0.00E+00
SCE1572_4193	sce_3515	2.00E-131	3.00E-131
SCE1572_4194	sce_3516	9.00E-146	5.00E-139
SCE1572_4195	sce_3517	2.00E-120	4.00E-123
SCE1572_4199	sce_3518	6.00E-138	9.00E-126
SCE1572_420	sce_234	5.00E-101	7.00E-91
SCE1572_4200	sce_479	3.00E-85	9.00E-107
SCE1572_4201	sce_3519	1.00E-58	3.00E-49
SCE1572_4202	sce_3520	0.00E+00	0.00E+00
SCE1572_4202	sce_10425	0.00E+00	0.00E+00
SCE1572_4202	sce_7361	0.00E+00	0.00E+00
SCE1572_4203	sce_3521	2.00E-156	2.00E-156
SCE1572_4204	sce_3522	7.00E-37	6.00E-27
SCE1572_4205	sce_3523	3.00E-120	5.00E-114
SCE1572_4206	sce_3524	0.00E+00	0.00E+00
SCE1572_4207	sce_3525	4.00E-140	2.00E-139
SCE1572_4208	sce_3526	8.00E-136	9.00E-125
SCE1572_4209	sce_3527	2.00E-137	3.00E-136
SCE1572_421	sce_235	2.00E-72	6.00E-78
SCE1572_4210	sce_3528	5.00E-22	2.00E-15
SCE1572_4211	sce_3529	0.00E+00	0.00E+00
SCE1572_4213	sce_3531	2.00E-124	2.00E-124
SCE1572_4215	sce_3533	0.00E+00	0.00E+00
SCE1572_4216	sce_3534	0.00E+00	0.00E+00
SCE1572_4217	sce_3535	0.00E+00	0.00E+00
SCE1572_4219	sce_3536	1.00E-148	2.00E-152
SCE1572_422	sce_236	0.00E+00	0.00E+00
SCE1572_4220	sce_3537	1.00E-46	4.00E-50
SCE1572_4221	sce_3538	2.00E-108	2.00E-108
SCE1572_4222	sce_10449	2.00E-06	4.00E-08
SCE1572_4223	sce_3539	3.00E-62	3.00E-64
SCE1572_4225	sce_3540	0.00E+00	0.00E+00
SCE1572_4227	sce_3542	0.00E+00	0.00E+00
SCE1572_4228	sce_1020	2.00E-122	3.00E-126
SCE1572_4229	sce_118	1.00E-165	2.00E-148
SCE1572_423	sce_237	3.00E-71	6.00E-68
SCE1572_4232	sce_116	5.00E-110	2.00E-67
SCE1572_4233	sce_115	3.00E-50	3.00E-30
SCE1572_4234	sce_114	2.00E-26	2.00E-24
SCE1572_4235	sce_113	1.00E-29	1.00E-29
SCE1572_4236	sce_3543	4.00E-127	2.00E-130
SCE1572_4237	sce_3544	2.00E-173	3.00E-173
SCE1572_4239	sce_3545	6.00E-131	8.00E-123
SCE1572_424	sce_238	1.00E-137	1.00E-140
SCE1572_4240	sce_1034	2.00E-107	8.00E-113
SCE1572_4244	sce_3546	0.00E+00	0.00E+00
SCE1572_4245	sce_3547	4.00E-97	2.00E-82
SCE1572_4246	sce_3548	1.00E-38	4.00E-41
SCE1572_4247	sce_3549	1.00E-175	2.00E-175
SCE1572_4248	sce_3550	0.00E+00	0.00E+00
SCE1572_425	sce_7595	3.00E-110	3.00E-110
SCE1572_4250	sce_3551	0.00E+00	0.00E+00
SCE1572_4251	sce_3553	7.00E-53	6.00E-43
SCE1572_4252	sce_3554	2.00E-62	5.00E-67
SCE1572_4255	sce_3556	1.00E-32	2.00E-32
SCE1572_4256	sce_3557	7.00E-120	2.00E-119
SCE1572_4258	sce_3559	2.00E-114	5.00E-117
SCE1572_426	sce_7596	1.00E-59	4.00E-61
SCE1572_4260	sce_9165	0.00E+00	0.00E+00
SCE1572_4268	sce_4615	7.00E-28	6.00E-29
SCE1572_427	sce_7597	2.00E-105	2.00E-104
SCE1572_4273	sce_4652	1.00E-08	8.00E-08
SCE1572_428	sce_821	2.00E-64	2.00E-78
SCE1572_4282	sce_3583	1.00E-53	4.00E-53
SCE1572_4284	sce_4504	4.00E-114	4.00E-114
SCE1572_4288	sce_3587	1.00E-143	2.00E-150
SCE1572_4289	sce_2038	1.00E-99	2.00E-99
SCE1572_429	sce_9917	0.00E+00	0.00E+00
SCE1572_4292	sce_3630	7.00E-142	8.00E-142

SCE1572_4293	sce_3631	0.00E+00	0.00E+00
SCE1572_4294	sce_3632	0.00E+00	0.00E+00
SCE1572_4295	sce_3633	2.00E-107	2.00E-113
SCE1572_4296	sce_3634	0.00E+00	0.00E+00
SCE1572_4299	sce_3635	0.00E+00	0.00E+00
SCE1572_430	sce_9918	0.00E+00	0.00E+00
SCE1572_430	sce_894	0.00E+00	0.00E+00
SCE1572_430	sce_6531	0.00E+00	0.00E+00
SCE1572_4300	sce_3636	0.00E+00	0.00E+00
SCE1572_4301	sce_3637	0.00E+00	0.00E+00
SCE1572_4303	sce_1742	3.00E-32	1.00E-31
SCE1572_4304	sce_6369	4.00E-60	2.00E-65
SCE1572_4305	sce_6370	1.00E-104	2.00E-102
SCE1572_4306	sce_6371	9.00E-63	4.00E-65
SCE1572_4307	sce_6372	9.00E-110	2.00E-118
SCE1572_431	sce_893	0.00E+00	0.00E+00
SCE1572_4311	sce_3642	0.00E+00	0.00E+00
SCE1572_4312	sce_3643	0.00E+00	0.00E+00
SCE1572_4314	sce_5479	0.00E+00	0.00E+00
SCE1572_4315	sce_5478	1.00E-54	4.00E-62
SCE1572_4316	sce_5477	0.00E+00	2.00E-177
SCE1572_4317	sce_5476	2.00E-168	7.00E-178
SCE1572_4318	sce_5475	0.00E+00	0.00E+00
SCE1572_4318	sce_6689	0.00E+00	0.00E+00
SCE1572_4319	sce_5474	8.00E-180	0.00E+00
SCE1572_432	sce_247	0.00E+00	0.00E+00
SCE1572_432	sce_2052	0.00E+00	0.00E+00
SCE1572_4325	sce_9304	4.00E-56	1.00E-61
SCE1572_4326	sce_1249	4.00E-48	2.00E-63
SCE1572_4327	sce_3613	2.00E-62	3.00E-60
SCE1572_4328	sce_3644	5.00E-158	1.00E-147
SCE1572_4329	sce_3645	2.00E-139	1.00E-139
SCE1572_433	sce_248	0.00E+00	0.00E+00
SCE1572_433	sce_3612	0.00E+00	0.00E+00
SCE1572_433	sce_886	0.00E+00	0.00E+00
SCE1572_433	sce_5797	0.00E+00	0.00E+00
SCE1572_4331	sce_1730	7.00E-126	6.00E-128
SCE1572_4332	sce_1731	5.00E-135	2.00E-141
SCE1572_4334	sce_1451	6.00E-101	9.00E-95
SCE1572_4336	sce_9941	3.00E-179	0.00E+00
SCE1572_4338	sce_3614	0.00E+00	0.00E+00
SCE1572_4339	sce_3615	3.00E-82	4.00E-90
SCE1572_4340	sce_3617	0.00E+00	0.00E+00
SCE1572_4341	sce_3618	2.00E-155	4.00E-169
SCE1572_4342	sce_3619	1.00E-155	0.00E+00
SCE1572_4343	sce_3620	6.00E-51	2.00E-51
SCE1572_4345	sce_6956	4.00E-50	9.00E-56
SCE1572_4347	sce_3647	1.00E-45	2.00E-45
SCE1572_4348	sce_3648	1.00E-131	8.00E-131
SCE1572_435	sce_9784	5.00E-124	2.00E-117
SCE1572_4350	sce_8789	0.00E+00	0.00E+00
SCE1572_4351	sce_8790	1.00E-116	2.00E-116
SCE1572_4353	sce_10400	1.00E-123	2.00E-123
SCE1572_4357	sce_4503	5.00E-180	2.00E-178
SCE1572_4358	sce_4502	1.00E-151	3.00E-152
SCE1572_4359	sce_3650	4.00E-72	6.00E-83
SCE1572_436	sce_9783	7.00E-107	2.00E-119
SCE1572_4360	sce_3651	0.00E+00	0.00E+00
SCE1572_4366	sce_3652	6.00E-85	5.00E-85
SCE1572_4367	sce_3653	0.00E+00	0.00E+00
SCE1572_4367	sce_2815	0.00E+00	0.00E+00
SCE1572_4368	sce_3654	2.00E-124	2.00E-124
SCE1572_4369	sce_3656	0.00E+00	0.00E+00
SCE1572_437	sce_251	0.00E+00	0.00E+00
SCE1572_4372	sce_1774	5.00E-138	1.00E-119
SCE1572_4375	sce_7552	1.00E-71	3.00E-141
SCE1572_4378	sce_7961	5.00E-28	3.00E-28
SCE1572_4379	sce_2664	3.00E-104	3.00E-113
SCE1572_4380	sce_9237	0.00E+00	0.00E+00
SCE1572_4380	sce_2666	0.00E+00	0.00E+00
SCE1572_4381	sce_9236	0.00E+00	0.00E+00
SCE1572_4381	sce_389	0.00E+00	0.00E+00
SCE1572_4387	sce_3657	0.00E+00	0.00E+00
SCE1572_4388	sce_5785	2.00E-67	1.00E-74
SCE1572_4390	sce_8231	3.00E-145	9.00E-150
SCE1572_4391	sce_3655	0.00E+00	0.00E+00

SCE1572_4392	sce_7959	2.00E-175	0.00E+00
SCE1572_4393	sce_5977	2.00E-32	1.00E-52
SCE1572_4394	sce_5975	3.00E-102	1.00E-112
SCE1572_4397	sce_3659	0.00E+00	0.00E+00
SCE1572_4398	sce_3660	0.00E+00	0.00E+00
SCE1572_44	sce_43	3.00E-97	1.00E-104
SCE1572_4400	sce_3662	2.00E-126	3.00E-113
SCE1572_4401	sce_3663	2.00E-56	1.00E-57
SCE1572_4424	sce_3665	0.00E+00	0.00E+00
SCE1572_4424	sce_549	0.00E+00	0.00E+00
SCE1572_4426	sce_7566	5.00E-112	5.00E-112
SCE1572_4427	sce_7565	3.00E-156	2.00E-159
SCE1572_4429	sce_3666	3.00E-152	5.00E-153
SCE1572_4430	sce_3667	2.00E-147	7.00E-115
SCE1572_4431	sce_3668	1.00E-87	3.00E-79
SCE1572_4434	sce_7318	0.00E+00	0.00E+00
SCE1572_4434	sce_1116	0.00E+00	0.00E+00
SCE1572_4434	sce_5158	0.00E+00	0.00E+00
SCE1572_4435	sce_1685	2.00E-19	5.00E-19
SCE1572_4438	sce_5548	5.00E-09	3.00E-09
SCE1572_4439	sce_5547	2.00E-17	4.00E-18
SCE1572_444	sce_257	9.00E-64	1.00E-63
SCE1572_4440	sce_3676	0.00E+00	0.00E+00
SCE1572_4441	sce_3678	0.00E+00	0.00E+00
SCE1572_4442	sce_3679	0.00E+00	0.00E+00
SCE1572_4443	sce_6978	2.00E-31	2.00E-34
SCE1572_4444	sce_3689	0.00E+00	0.00E+00
SCE1572_4445	sce_3690	0.00E+00	0.00E+00
SCE1572_4447	sce_3691	0.00E+00	0.00E+00
SCE1572_4448	sce_3692	2.00E-61	1.00E-58
SCE1572_4449	sce_4170	4.00E-16	5.00E-18
SCE1572_445	sce_258	4.00E-148	4.00E-144
SCE1572_4450	sce_7444	3.00E-64	3.00E-64
SCE1572_4450	sce_4542	3.00E-64	3.00E-64
SCE1572_4452	sce_9472	1.00E-49	5.00E-50
SCE1572_4453	sce_3693	9.00E-113	9.00E-110
SCE1572_4454	sce_3694	5.00E-84	2.00E-94
SCE1572_4455	sce_3696	6.00E-147	2.00E-140
SCE1572_4456	sce_3699	8.00E-105	5.00E-98
SCE1572_4458	sce_3700	3.00E-169	2.00E-167
SCE1572_4459	sce_3702	1.00E-142	1.00E-142
SCE1572_446	sce_259	0.00E+00	0.00E+00
SCE1572_4460	sce_7662	2.00E-12	1.00E-11
SCE1572_4461	sce_7663	2.00E-54	3.00E-48
SCE1572_4463	sce_7475	0.00E+00	0.00E+00
SCE1572_4466	sce_3703	3.00E-171	2.00E-171
SCE1572_4469	sce_7563	0.00E+00	0.00E+00
SCE1572_447	sce_260	3.00E-136	3.00E-136
SCE1572_4470	sce_3713	4.00E-63	1.00E-63
SCE1572_4471	sce_3714	3.00E-172	0.00E+00
SCE1572_4475	sce_5993	0.00E+00	0.00E+00
SCE1572_4476	sce_1358	7.00E-39	6.00E-55
SCE1572_4478	sce_6103	2.00E-49	4.00E-48
SCE1572_448	sce_261	0.00E+00	0.00E+00
SCE1572_4480	sce_6105	3.00E-34	2.00E-24
SCE1572_4486	sce_7885	9.00E-75	6.00E-75
SCE1572_4487	sce_3726	5.00E-113	1.00E-121
SCE1572_4489	sce_3719	2.00E-124	6.00E-140
SCE1572_449	sce_262	9.00E-108	2.00E-107
SCE1572_4490	sce_7400	7.00E-114	7.00E-108
SCE1572_4491	sce_6061	8.00E-68	4.00E-74
SCE1572_4493	sce_2657	3.00E-149	6.00E-139
SCE1572_4494	sce_7608	1.00E-07	3.00E-06
SCE1572_4495	sce_3745	0.00E+00	0.00E+00
SCE1572_4496	sce_3746	5.00E-84	8.00E-89
SCE1572_4497	sce_3747	2.00E-143	2.00E-145
SCE1572_4498	sce_3748	5.00E-153	0.00E+00
SCE1572_4499	sce_3749	5.00E-99	5.00E-99
SCE1572_45	sce_44	6.00E-154	6.00E-156
SCE1572_450	sce_263	1.00E-179	2.00E-169
SCE1572_4500	sce_3763	7.00E-111	1.00E-109
SCE1572_4501	sce_3764	3.00E-56	1.00E-119
SCE1572_4504	sce_3765	0.00E+00	0.00E+00
SCE1572_4504	sce_9407	0.00E+00	0.00E+00
SCE1572_4504	sce_8451	0.00E+00	0.00E+00
SCE1572_4504	sce_4817	0.00E+00	0.00E+00

SCE1572_4507	sce_3766	4.00E-141	1.00E-139
SCE1572_4508	sce_3767	2.00E-27	1.00E-25
SCE1572_4509	sce_3769	5.00E-78	4.00E-78
SCE1572_451	sce_267	6.00E-97	2.00E-88
SCE1572_4510	sce_3770	2.00E-107	6.00E-117
SCE1572_4514	sce_3771	9.00E-138	9.00E-138
SCE1572_4515	sce_3772	1.00E-41	1.00E-43
SCE1572_4516	sce_3773	1.00E-92	3.00E-89
SCE1572_4517	sce_3774	0.00E+00	5.00E-167
SCE1572_4519	sce_3778	2.00E-162	2.00E-162
SCE1572_452	sce_268	1.00E-53	3.00E-53
SCE1572_4520	sce_8632	1.00E-112	1.00E-112
SCE1572_4521	sce_3779	2.00E-123	8.00E-110
SCE1572_4523	sce_3782	4.00E-63	1.00E-63
SCE1572_4524	sce_3783	3.00E-142	7.00E-142
SCE1572_4525	sce_3784	6.00E-107	1.00E-95
SCE1572_4526	sce_3785	1.00E-87	6.00E-95
SCE1572_4527	sce_3787	3.00E-122	2.00E-125
SCE1572_4529	sce_3788	3.00E-126	2.00E-111
SCE1572_453	sce_269	0.00E+00	0.00E+00
SCE1572_4530	sce_3789	2.00E-135	6.00E-152
SCE1572_4533	sce_3790	3.00E-175	4.00E-175
SCE1572_4534	sce_3791	4.00E-144	3.00E-160
SCE1572_4535	sce_3792	1.00E-45	2.00E-42
SCE1572_4536	sce_9770	2.00E-150	5.00E-143
SCE1572_4538	sce_3794	0.00E+00	0.00E+00
SCE1572_4539	sce_3796	6.00E-99	8.00E-98
SCE1572_4540	sce_3793	0.00E+00	0.00E+00
SCE1572_4540	sce_7069	0.00E+00	0.00E+00
SCE1572_4540	sce_5086	0.00E+00	0.00E+00
SCE1572_4541	sce_3794	0.00E+00	0.00E+00
SCE1572_4543	sce_3797	0.00E+00	0.00E+00
SCE1572_4544	sce_3798	0.00E+00	0.00E+00
SCE1572_4552	sce_1380	0.00E+00	0.00E+00
SCE1572_4553	sce_1379	3.00E-88	5.00E-89
SCE1572_4557	sce_3799	3.00E-155	4.00E-155
SCE1572_4558	sce_3801	8.00E-163	9.00E-152
SCE1572_4559	sce_3802	1.00E-79	6.00E-75
SCE1572_4563	sce_3813	8.00E-38	8.00E-38
SCE1572_4564	sce_3814	2.00E-16	3.00E-17
SCE1572_4565	sce_3816	0.00E+00	0.00E+00
SCE1572_4566	sce_3818	3.00E-78	2.00E-83
SCE1572_4567	sce_3819	9.00E-139	9.00E-139
SCE1572_4568	sce_3820	2.00E-10	3.00E-11
SCE1572_458	sce_270	0.00E+00	0.00E+00
SCE1572_4580	sce_3097	1.00E-155	2.00E-154
SCE1572_4582	sce_3821	0.00E+00	0.00E+00
SCE1572_4583	sce_3822	7.00E-08	1.00E-07
SCE1572_4584	sce_3823	1.00E-142	1.00E-142
SCE1572_4585	sce_3824	3.00E-132	4.00E-126
SCE1572_4586	sce_3825	0.00E+00	0.00E+00
SCE1572_4587	sce_3826	0.00E+00	0.00E+00
SCE1572_4588	sce_3827	9.00E-60	2.00E-58
SCE1572_4589	sce_3828	8.00E-56	5.00E-54
SCE1572_459	sce_271	9.00E-112	3.00E-111
SCE1572_4591	sce_3829	3.00E-18	1.00E-18
SCE1572_4592	sce_3830	0.00E+00	0.00E+00
SCE1572_4593	sce_3831	5.00E-93	4.00E-93
SCE1572_4594	sce_3832	2.00E-150	2.00E-144
SCE1572_4596	sce_3833	0.00E+00	0.00E+00
SCE1572_4598	sce_3835	0.00E+00	0.00E+00
SCE1572_46	sce_45	8.00E-155	3.00E-146
SCE1572_460	sce_272	3.00E-08	5.00E-14
SCE1572_4600	sce_3837	2.00E-180	0.00E+00
SCE1572_4602	sce_3838	2.00E-75	1.00E-68
SCE1572_4603	sce_3839	0.00E+00	0.00E+00
SCE1572_4604	sce_3840	1.00E-103	4.00E-105
SCE1572_4605	sce_3841	5.00E-137	3.00E-130
SCE1572_4606	sce_3842	1.00E-69	7.00E-59
SCE1572_4607	sce_3843	2.00E-92	5.00E-92
SCE1572_4608	sce_3844	7.00E-124	1.00E-134
SCE1572_4609	sce_3845	3.00E-14	1.00E-14
SCE1572_461	sce_273	3.00E-25	1.00E-21
SCE1572_4610	sce_3846	2.00E-140	2.00E-136
SCE1572_4611	sce_3847	5.00E-140	1.00E-136
SCE1572_4618	sce_3850	0.00E+00	0.00E+00

SCE1572_462	sce_274	2.00E-101	3.00E-108
SCE1572_4623	sce_3852	3.00E-64	2.00E-72
SCE1572_4624	sce_3853	3.00E-75	3.00E-75
SCE1572_4625	sce_3855	3.00E-64	1.00E-66
SCE1572_4626	sce_3856	6.00E-98	9.00E-92
SCE1572_4627	sce_3857	3.00E-40	2.00E-40
SCE1572_4629	sce_3859	0.00E+00	0.00E+00
SCE1572_4629	sce_3861	0.00E+00	0.00E+00
SCE1572_463	sce_277	1.00E-127	2.00E-125
SCE1572_4630	sce_6341	0.00E+00	0.00E+00
SCE1572_4633	sce_3860	0.00E+00	0.00E+00
SCE1572_4634	sce_3861	0.00E+00	0.00E+00
SCE1572_4634	sce_3859	0.00E+00	0.00E+00
SCE1572_4637	sce_3862	0.00E+00	0.00E+00
SCE1572_4638	sce_3863	1.00E-135	1.00E-132
SCE1572_4639	sce_3864	9.00E-72	2.00E-96
SCE1572_4640	sce_3865	2.00E-106	1.00E-104
SCE1572_4644	sce_10174	3.00E-105	1.00E-105
SCE1572_4645	sce_3868	0.00E+00	0.00E+00
SCE1572_4646	sce_3869	3.00E-123	4.00E-139
SCE1572_4648	sce_3870	3.00E-52	2.00E-49
SCE1572_4650	sce_3872	3.00E-172	1.00E-164
SCE1572_4651	sce_3873	2.00E-165	3.00E-168
SCE1572_4652	sce_3874	0.00E+00	0.00E+00
SCE1572_4653	sce_3875	0.00E+00	0.00E+00
SCE1572_4654	sce_3876	1.00E-89	7.00E-84
SCE1572_4657	sce_3877	7.00E-51	2.00E-59
SCE1572_4658	sce_3878	2.00E-147	2.00E-147
SCE1572_4659	sce_4570	2.00E-62	2.00E-69
SCE1572_466	sce_278	2.00E-161	4.00E-151
SCE1572_4660	sce_3881	6.00E-67	4.00E-67
SCE1572_4661	sce_3882	1.00E-97	5.00E-78
SCE1572_4662	sce_3883	0.00E+00	0.00E+00
SCE1572_4662	sce_9757	0.00E+00	0.00E+00
SCE1572_4662	sce_5226	0.00E+00	0.00E+00
SCE1572_4662	sce_5403	0.00E+00	0.00E+00
SCE1572_4662	sce_7154	0.00E+00	0.00E+00
SCE1572_4664	sce_3885	4.00E-156	6.00E-145
SCE1572_4665	sce_3886	6.00E-61	1.00E-56
SCE1572_4666	sce_3887	4.00E-34	9.00E-35
SCE1572_467	sce_279	0.00E+00	0.00E+00
SCE1572_4670	sce_7871	9.00E-124	5.00E-124
SCE1572_4673	sce_3906	0.00E+00	0.00E+00
SCE1572_4674	sce_3907	6.00E-49	6.00E-49
SCE1572_4675	sce_3910	1.00E-137	6.00E-130
SCE1572_468	sce_280	5.00E-127	2.00E-132
SCE1572_4684	sce_1283	0.00E+00	0.00E+00
SCE1572_4685	sce_1282	0.00E+00	0.00E+00
SCE1572_4689	sce_8439	3.00E-163	5.00E-154
SCE1572_469	sce_281	6.00E-118	6.00E-118
SCE1572_4693	sce_1662	0.00E+00	0.00E+00
SCE1572_4696	sce_7939	6.00E-137	8.00E-137
SCE1572_4697	sce_5765	1.00E-19	3.00E-25
SCE1572_4699	sce_3926	4.00E-112	2.00E-95
SCE1572_47	sce_46	1.00E-145	2.00E-149
SCE1572_470	sce_282	7.00E-137	1.00E-140
SCE1572_4700	sce_3927	2.00E-89	1.00E-84
SCE1572_4701	sce_5100	5.00E-16	8.00E-21
SCE1572_4703	sce_3929	3.00E-88	1.00E-98
SCE1572_4704	sce_3930	8.00E-86	9.00E-86
SCE1572_4705	sce_3931	4.00E-155	5.00E-145
SCE1572_4706	sce_3932	2.00E-133	3.00E-133
SCE1572_4707	sce_3933	2.00E-112	1.00E-112
SCE1572_4709	sce_9357	0.00E+00	0.00E+00
SCE1572_4710	sce_3935	3.00E-79	5.00E-82
SCE1572_4711	sce_8816	0.00E+00	0.00E+00
SCE1572_4712	sce_8817	0.00E+00	0.00E+00
SCE1572_4712	sce_10193	0.00E+00	0.00E+00
SCE1572_4712	sce_3499	0.00E+00	0.00E+00
SCE1572_4713	sce_3939	0.00E+00	0.00E+00
SCE1572_4714	sce_3940	3.00E-84	4.00E-93
SCE1572_4716	sce_3941	1.00E-59	7.00E-48
SCE1572_4717	sce_3942	1.00E-80	2.00E-86
SCE1572_4718	sce_3943	3.00E-78	5.00E-91
SCE1572_4719	sce_3945	0.00E+00	0.00E+00
SCE1572_472	sce_283	2.00E-55	6.00E-62

SCE1572_4720	sce_3946	5.00E-129	6.00E-129
SCE1572_4721	sce_3947	4.00E-159	2.00E-162
SCE1572_4722	sce_3948	1.00E-160	2.00E-169
SCE1572_4723	sce_3949	3.00E-44	3.00E-44
SCE1572_4724	sce_3950	8.00E-37	1.00E-44
SCE1572_4725	sce_3951	0.00E+00	0.00E+00
SCE1572_4726	sce_3952	0.00E+00	0.00E+00
SCE1572_4727	sce_6342	1.00E-120	4.00E-122
SCE1572_4728	sce_6341	0.00E+00	0.00E+00
SCE1572_4729	sce_6340	6.00E-35	7.00E-28
SCE1572_473	sce_284	3.00E-118	3.00E-118
SCE1572_4730	sce_6339	0.00E+00	0.00E+00
SCE1572_4731	sce_6338	2.00E-146	8.00E-160
SCE1572_4732	sce_6337	3.00E-84	2.00E-63
SCE1572_4733	sce_6336	1.00E-59	1.00E-59
SCE1572_4734	sce_3954	8.00E-170	2.00E-173
SCE1572_4735	sce_3956	0.00E+00	0.00E+00
SCE1572_4736	sce_3957	2.00E-139	9.00E-138
SCE1572_4738	sce_3958	7.00E-51	7.00E-57
SCE1572_474	sce_285	2.00E-54	2.00E-54
SCE1572_4740	sce_3961	0.00E+00	0.00E+00
SCE1572_4741	sce_3962	6.00E-161	4.00E-175
SCE1572_4742	sce_3963	5.00E-74	4.00E-74
SCE1572_4743	sce_3965	4.00E-119	4.00E-109
SCE1572_4744	sce_3966	7.00E-42	2.00E-42
SCE1572_4745	sce_3967	0.00E+00	0.00E+00
SCE1572_4746	sce_3968	0.00E+00	0.00E+00
SCE1572_4747	sce_3969	1.00E-62	2.00E-63
SCE1572_475	sce_286	0.00E+00	0.00E+00
SCE1572_4750	sce_3971	1.00E-177	1.00E-177
SCE1572_4751	sce_3972	2.00E-178	9.00E-159
SCE1572_4752	sce_3974	2.00E-35	2.00E-35
SCE1572_4754	sce_7978	2.00E-119	2.00E-119
SCE1572_4755	sce_10127	2.00E-83	3.00E-91
SCE1572_4758	sce_3976	0.00E+00	0.00E+00
SCE1572_476	sce_287	3.00E-90	3.00E-90
SCE1572_4762	sce_3978	5.00E-154	4.00E-162
SCE1572_4763	sce_3979	3.00E-110	1.00E-111
SCE1572_4764	sce_3980	2.00E-89	8.00E-90
SCE1572_4765	sce_3981	5.00E-156	2.00E-146
SCE1572_4766	sce_3982	0.00E+00	0.00E+00
SCE1572_4767	sce_3983	8.00E-150	3.00E-140
SCE1572_477	sce_288	2.00E-91	3.00E-86
SCE1572_4771	sce_3987	4.00E-177	0.00E+00
SCE1572_4772	sce_3988	6.00E-166	2.00E-167
SCE1572_4773	sce_7233	3.00E-122	2.00E-125
SCE1572_4774	sce_3989	4.00E-25	1.00E-24
SCE1572_4776	sce_3991	0.00E+00	0.00E+00
SCE1572_4777	sce_3992	0.00E+00	0.00E+00
SCE1572_478	sce_289	5.00E-154	7.00E-145
SCE1572_4780	sce_3993	0.00E+00	0.00E+00
SCE1572_4781	sce_3995	2.00E-120	2.00E-112
SCE1572_4782	sce_3997	6.00E-27	1.00E-21
SCE1572_4783	sce_299	2.00E-121	7.00E-127
SCE1572_4785	sce_3999	0.00E+00	0.00E+00
SCE1572_4786	sce_4000	0.00E+00	0.00E+00
SCE1572_4787	sce_4001	9.00E-108	3.00E-110
SCE1572_4788	sce_4002	5.00E-135	2.00E-136
SCE1572_4789	sce_4004	0.00E+00	0.00E+00
SCE1572_479	sce_290	0.00E+00	0.00E+00
SCE1572_4790	sce_4005	4.00E-123	4.00E-121
SCE1572_4791	sce_4006	9.00E-96	1.00E-99
SCE1572_4793	sce_4007	6.00E-72	7.00E-72
SCE1572_4794	sce_4008	0.00E+00	0.00E+00
SCE1572_4795	sce_4010	7.00E-83	3.00E-73
SCE1572_4796	sce_4011	3.00E-70	2.00E-61
SCE1572_4797	sce_4012	5.00E-101	5.00E-113
SCE1572_4798	sce_4013	1.00E-45	1.00E-45
SCE1572_4799	sce_4014	0.00E+00	0.00E+00
SCE1572_48	sce_47	3.00E-119	1.00E-94
SCE1572_480	sce_291	9.00E-126	9.00E-126
SCE1572_4800	sce_4015	1.00E-90	1.00E-90
SCE1572_4801	sce_4016	3.00E-176	2.00E-173
SCE1572_4802	sce_4017	0.00E+00	0.00E+00
SCE1572_4803	sce_4018	0.00E+00	0.00E+00
SCE1572_4807	sce_4021	0.00E+00	0.00E+00

SCE1572_4808	sce_4022	8.00E-132	6.00E-138
SCE1572_4809	sce_4023	0.00E+00	0.00E+00
SCE1572_481	sce_292	6.00E-147	1.00E-144
SCE1572_4811	sce_4025	8.00E-106	1.00E-100
SCE1572_4812	sce_4026	3.00E-61	5.00E-66
SCE1572_4813	sce_4027	0.00E+00	0.00E+00
SCE1572_4814	sce_4028	4.00E-147	4.00E-138
SCE1572_4815	sce_4029	0.00E+00	0.00E+00
SCE1572_4817	sce_4031	2.00E-83	2.00E-83
SCE1572_4818	sce_4032	4.00E-40	1.00E-37
SCE1572_4819	sce_4033	4.00E-122	5.00E-132
SCE1572_482	sce_293	0.00E+00	0.00E+00
SCE1572_4820	sce_4034	0.00E+00	0.00E+00
SCE1572_4821	sce_4035	0.00E+00	0.00E+00
SCE1572_4823	sce_4036	4.00E-99	9.00E-101
SCE1572_4824	sce_4037	0.00E+00	0.00E+00
SCE1572_4825	sce_4038	4.00E-133	6.00E-133
SCE1572_4826	sce_4039	0.00E+00	0.00E+00
SCE1572_4827	sce_4040	8.00E-86	5.00E-73
SCE1572_4828	sce_4041	1.00E-33	1.00E-33
SCE1572_4829	sce_4042	7.00E-136	8.00E-155
SCE1572_483	sce_294	0.00E+00	0.00E+00
SCE1572_4830	sce_4043	8.00E-132	5.00E-143
SCE1572_4831	sce_4045	4.00E-169	1.00E-169
SCE1572_4833	sce_4046	2.00E-171	2.00E-171
SCE1572_4835	sce_4047	9.00E-68	2.00E-67
SCE1572_4836	sce_4048	2.00E-85	1.00E-94
SCE1572_4837	sce_4049	1.00E-128	5.00E-128
SCE1572_4838	sce_4050	5.00E-73	5.00E-73
SCE1572_4839	sce_4051	0.00E+00	0.00E+00
SCE1572_484	sce_295	0.00E+00	0.00E+00
SCE1572_4840	sce_4052	3.00E-76	2.00E-80
SCE1572_4841	sce_4053	0.00E+00	0.00E+00
SCE1572_4842	sce_4054	1.00E-50	3.00E-50
SCE1572_4843	sce_4055	3.00E-178	2.00E-173
SCE1572_4844	sce_4056	7.00E-109	1.00E-108
SCE1572_4845	sce_4057	2.00E-121	9.00E-135
SCE1572_4846	sce_4058	0.00E+00	0.00E+00
SCE1572_4847	sce_4059	6.00E-157	4.00E-163
SCE1572_4848	sce_4060	0.00E+00	1.00E-175
SCE1572_4849	sce_3628	3.00E-16	3.00E-18
SCE1572_485	sce_296	4.00E-113	3.00E-113
SCE1572_4851	sce_4062	0.00E+00	0.00E+00
SCE1572_4851	sce_9373	0.00E+00	0.00E+00
SCE1572_4853	sce_4063	2.00E-38	4.00E-39
SCE1572_4854	sce_4066	2.00E-95	3.00E-107
SCE1572_4855	sce_4067	3.00E-52	3.00E-59
SCE1572_4856	sce_4069	2.00E-125	1.00E-139
SCE1572_4857	sce_6129	7.00E-43	6.00E-35
SCE1572_4859	sce_4071	5.00E-129	5.00E-149
SCE1572_486	sce_297	4.00E-39	5.00E-39
SCE1572_4861	sce_4073	0.00E+00	0.00E+00
SCE1572_4861	sce_5403	0.00E+00	0.00E+00
SCE1572_4861	sce_7154	0.00E+00	0.00E+00
SCE1572_4862	sce_4075	4.00E-65	8.00E-53
SCE1572_4863	sce_4077	7.00E-123	4.00E-124
SCE1572_4864	sce_4078	1.00E-105	2.00E-120
SCE1572_4866	sce_4080	1.00E-115	1.00E-115
SCE1572_4868	sce_4081	0.00E+00	0.00E+00
SCE1572_4869	sce_4082	7.00E-45	3.00E-57
SCE1572_487	sce_298	4.00E-118	5.00E-110
SCE1572_4870	sce_4083	3.00E-51	9.00E-60
SCE1572_4871	sce_4084	4.00E-96	5.00E-96
SCE1572_4872	sce_4086	0.00E+00	0.00E+00
SCE1572_4873	sce_4087	4.00E-104	1.00E-103
SCE1572_4874	sce_4088	9.00E-93	9.00E-93
SCE1572_4875	sce_4089	2.00E-143	2.00E-143
SCE1572_4876	sce_4090	0.00E+00	0.00E+00
SCE1572_4877	sce_4091	4.00E-105	2.00E-107
SCE1572_4879	sce_4092	0.00E+00	0.00E+00
SCE1572_4880	sce_4094	7.00E-177	0.00E+00
SCE1572_4882	sce_4095	0.00E+00	0.00E+00
SCE1572_4883	sce_4096	9.00E-58	1.00E-57
SCE1572_4884	sce_4097	2.00E-110	3.00E-115
SCE1572_4889	sce_4102	9.00E-123	8.00E-159
SCE1572_4890	sce_914	1.00E-141	1.00E-141

SCE1572_49	sce_48	3.00E-92	3.00E-92
SCE1572_4901	sce_4861	0.00E+00	0.00E+00
SCE1572_4901	sce_4106	0.00E+00	0.00E+00
SCE1572_4902	sce_4107	2.00E-132	3.00E-140
SCE1572_4903	sce_4108	2.00E-147	7.00E-141
SCE1572_4906	sce_4116	2.00E-73	2.00E-73
SCE1572_4907	sce_4117	1.00E-86	3.00E-71
SCE1572_4908	sce_4118	2.00E-171	7.00E-173
SCE1572_4911	sce_4121	0.00E+00	0.00E+00
SCE1572_4912	sce_4122	2.00E-41	2.00E-41
SCE1572_4913	sce_4123	2.00E-43	2.00E-43
SCE1572_4914	sce_4124	9.00E-90	1.00E-79
SCE1572_4916	sce_4127	0.00E+00	0.00E+00
SCE1572_4917	sce_4128	2.00E-47	6.00E-72
SCE1572_4918	sce_4129	0.00E+00	0.00E+00
SCE1572_4919	sce_4130	2.00E-60	2.00E-51
SCE1572_4920	sce_4131	1.00E-45	3.00E-53
SCE1572_4921	sce_4132	4.00E-72	5.00E-66
SCE1572_4922	sce_4134	5.00E-80	4.00E-96
SCE1572_4923	sce_4135	5.00E-176	5.00E-176
SCE1572_4924	sce_4136	5.00E-103	2.00E-102
SCE1572_4925	sce_4137	0.00E+00	0.00E+00
SCE1572_4926	sce_4138	7.00E-113	2.00E-135
SCE1572_4929	sce_4140	1.00E-112	5.00E-113
SCE1572_493	sce_5846	1.00E-92	2.00E-68
SCE1572_4930	sce_4141	5.00E-143	1.00E-140
SCE1572_4931	sce_4142	5.00E-128	7.00E-121
SCE1572_4932	sce_4143	1.00E-167	7.00E-170
SCE1572_4933	sce_4144	3.00E-30	3.00E-30
SCE1572_4934	sce_4145	1.00E-76	8.00E-74
SCE1572_4935	sce_4146	8.00E-115	3.00E-113
SCE1572_4936	sce_4147	2.00E-131	2.00E-121
SCE1572_4937	sce_4149	8.00E-154	3.00E-142
SCE1572_4939	sce_4152	0.00E+00	0.00E+00
SCE1572_494	sce_5847	3.00E-38	7.00E-42
SCE1572_4940	sce_4154	1.00E-120	1.00E-120
SCE1572_4941	sce_4155	7.00E-133	2.00E-120
SCE1572_4942	sce_4156	2.00E-77	1.00E-75
SCE1572_4943	sce_4157	0.00E+00	0.00E+00
SCE1572_4944	sce_4158	8.00E-110	1.00E-88
SCE1572_4945	sce_4159	0.00E+00	0.00E+00
SCE1572_4947	sce_4161	8.00E-123	2.00E-112
SCE1572_4949	sce_4162	2.00E-71	2.00E-72
SCE1572_4950	sce_4899	8.00E-39	3.00E-34
SCE1572_4951	sce_4163	3.00E-88	3.00E-88
SCE1572_4952	sce_4164	9.00E-15	3.00E-11
SCE1572_4953	sce_1234	4.00E-23	6.00E-23
SCE1572_4955	sce_4165	4.00E-137	1.00E-142
SCE1572_4956	sce_4166	0.00E+00	0.00E+00
SCE1572_4958	sce_4167	2.00E-76	9.00E-70
SCE1572_496	sce_5848	1.00E-145	8.00E-146
SCE1572_4960	sce_4168	0.00E+00	5.00E-135
SCE1572_4961	sce_4169	2.00E-36	2.00E-37
SCE1572_4962	sce_4173	9.00E-39	9.00E-35
SCE1572_4963	sce_4174	0.00E+00	0.00E+00
SCE1572_4964	sce_4175	4.00E-74	2.00E-76
SCE1572_4965	sce_3793	0.00E+00	0.00E+00
SCE1572_4965	sce_7069	0.00E+00	0.00E+00
SCE1572_4965	sce_5086	0.00E+00	0.00E+00
SCE1572_4965	sce_6701	0.00E+00	0.00E+00
SCE1572_4965	sce_5085	0.00E+00	0.00E+00
SCE1572_4967	sce_4176	6.00E-180	0.00E+00
SCE1572_4968	sce_4177	0.00E+00	0.00E+00
SCE1572_4969	sce_4178	9.00E-94	1.00E-98
SCE1572_497	sce_2515	3.00E-42	3.00E-49
SCE1572_4972	sce_4181	8.00E-41	2.00E-46
SCE1572_4973	sce_4182	0.00E+00	0.00E+00
SCE1572_4974	sce_4183	2.00E-93	7.00E-94
SCE1572_4975	sce_4184	0.00E+00	0.00E+00
SCE1572_4976	sce_4185	0.00E+00	0.00E+00
SCE1572_4977	sce_4186	0.00E+00	0.00E+00
SCE1572_4978	sce_4187	2.00E-162	7.00E-167
SCE1572_498	sce_8618	0.00E+00	0.00E+00
SCE1572_498	sce_2065	0.00E+00	0.00E+00
SCE1572_498	sce_10204	0.00E+00	0.00E+00
SCE1572_498	sce_388	0.00E+00	0.00E+00

SCE1572_498	sce_7640	0.00E+00	0.00E+00
SCE1572_498	sce_4513	0.00E+00	0.00E+00
SCE1572_498	sce_3003	0.00E+00	0.00E+00
SCE1572_498	sce_7109	0.00E+00	0.00E+00
SCE1572_498	sce_7661	0.00E+00	0.00E+00
SCE1572_4984	sce_4189	0.00E+00	0.00E+00
SCE1572_4986	sce_4190	6.00E-160	5.00E-150
SCE1572_4987	sce_4191	0.00E+00	0.00E+00
SCE1572_4988	sce_4192	2.00E-62	5.00E-69
SCE1572_4989	sce_4193	4.00E-80	5.00E-86
SCE1572_4990	sce_4194	2.00E-113	2.00E-113
SCE1572_4991	sce_4195	7.00E-31	3.00E-26
SCE1572_4992	sce_4196	6.00E-14	4.00E-23
SCE1572_4993	sce_4197	4.00E-96	1.00E-104
SCE1572_4994	sce_4198	2.00E-172	7.00E-163
SCE1572_4995	sce_4199	2.00E-110	7.00E-117
SCE1572_4996	sce_4200	5.00E-72	6.00E-67
SCE1572_4998	sce_4201	2.00E-111	6.00E-126
SCE1572_4999	sce_4202	0.00E+00	0.00E+00
SCE1572_5	sce_5	2.00E-174	2.00E-177
SCE1572_50	sce_49	1.00E-122	2.00E-128
SCE1572_5001	sce_4203	2.00E-150	3.00E-157
SCE1572_5002	sce_4204	1.00E-77	5.00E-77
SCE1572_5003	sce_4205	3.00E-65	8.00E-67
SCE1572_5004	sce_4206	2.00E-44	4.00E-46
SCE1572_5005	sce_4207	2.00E-26	7.00E-20
SCE1572_5006	sce_4208	0.00E+00	0.00E+00
SCE1572_5007	sce_4209	1.00E-121	2.00E-121
SCE1572_5008	sce_4210	2.00E-83	3.00E-90
SCE1572_5009	sce_4211	1.00E-61	1.00E-61
SCE1572_5010	sce_4212	0.00E+00	0.00E+00
SCE1572_5011	sce_4213	4.00E-118	3.00E-119
SCE1572_5012	sce_4214	4.00E-114	5.00E-114
SCE1572_5013	sce_4215	1.00E-140	2.00E-146
SCE1572_5014	sce_4216	2.00E-171	2.00E-171
SCE1572_5015	sce_4217	7.00E-86	2.00E-91
SCE1572_5016	sce_4218	0.00E+00	0.00E+00
SCE1572_5017	sce_4219	2.00E-85	1.00E-79
SCE1572_5018	sce_4220	0.00E+00	0.00E+00
SCE1572_5019	sce_4221	3.00E-108	4.00E-107
SCE1572_5020	sce_4222	0.00E+00	0.00E+00
SCE1572_5021	sce_4223	0.00E+00	0.00E+00
SCE1572_5022	sce_4225	9.00E-157	9.00E-157
SCE1572_5024	sce_4226	0.00E+00	0.00E+00
SCE1572_5025	sce_4227	9.00E-168	9.00E-179
SCE1572_5026	sce_4228	1.00E-82	3.00E-86
SCE1572_5027	sce_4229	0.00E+00	0.00E+00
SCE1572_5028	sce_4230	5.00E-112	4.00E-134
SCE1572_5029	sce_4231	3.00E-114	2.00E-114
SCE1572_5030	sce_4232	0.00E+00	0.00E+00
SCE1572_5031	sce_4233	2.00E-65	1.00E-67
SCE1572_5032	sce_4234	0.00E+00	0.00E+00
SCE1572_5033	sce_4235	7.00E-112	2.00E-119
SCE1572_5034	sce_4236	1.00E-146	2.00E-146
SCE1572_5035	sce_4237	1.00E-125	2.00E-132
SCE1572_5037	sce_4238	0.00E+00	0.00E+00
SCE1572_5037	sce_9607	0.00E+00	0.00E+00
SCE1572_5038	sce_4239	5.00E-63	5.00E-63
SCE1572_5040	sce_4240	1.00E-135	7.00E-145
SCE1572_5041	sce_4241	1.00E-91	4.00E-98
SCE1572_5042	sce_4242	0.00E+00	0.00E+00
SCE1572_5043	sce_4243	5.00E-58	9.00E-62
SCE1572_5044	sce_4244	3.00E-18	4.00E-14
SCE1572_5045	sce_4245	4.00E-93	1.00E-84
SCE1572_5047	sce_6904	8.00E-81	7.00E-72
SCE1572_505	sce_8661	7.00E-10	1.00E-06
SCE1572_5052	sce_522	7.00E-135	2.00E-126
SCE1572_5056	sce_7667	0.00E+00	0.00E+00
SCE1572_5057	sce_7841	3.00E-169	5.00E-175
SCE1572_5058	sce_4246	0.00E+00	0.00E+00
SCE1572_5059	sce_4247	6.00E-101	1.00E-98
SCE1572_5060	sce_4248	1.00E-171	1.00E-176
SCE1572_5061	sce_4250	4.00E-55	4.00E-55
SCE1572_5062	sce_4251	2.00E-139	5.00E-151
SCE1572_5063	sce_4252	1.00E-13	2.00E-21
SCE1572_5064	sce_4253	8.00E-143	2.00E-149

SCE1572_5065	sce_4254	2.00E-167	2.00E-170
SCE1572_5066	sce_4255	9.00E-162	1.00E-164
SCE1572_5067	sce_4256	2.00E-172	2.00E-172
SCE1572_5068	sce_4257	1.00E-144	0.00E+00
SCE1572_5069	sce_4258	0.00E+00	0.00E+00
SCE1572_507	sce_1673	1.00E-57	2.00E-63
SCE1572_5070	sce_4259	2.00E-153	2.00E-144
SCE1572_5071	sce_4260	0.00E+00	0.00E+00
SCE1572_5072	sce_4261	8.00E-136	5.00E-144
SCE1572_5073	sce_4262	3.00E-128	5.00E-131
SCE1572_5074	sce_4263	1.00E-34	1.00E-34
SCE1572_5076	sce_4265	0.00E+00	0.00E+00
SCE1572_5077	sce_4266	3.00E-90	3.00E-90
SCE1572_5078	sce_4267	0.00E+00	2.00E-178
SCE1572_5079	sce_4268	2.00E-64	3.00E-64
SCE1572_508	sce_1674	0.00E+00	0.00E+00
SCE1572_5080	sce_4269	0.00E+00	0.00E+00
SCE1572_5081	sce_4270	3.00E-39	3.00E-39
SCE1572_5082	sce_4271	5.00E-119	2.00E-118
SCE1572_5083	sce_4272	7.00E-130	5.00E-131
SCE1572_5084	sce_4273	1.00E-163	8.00E-164
SCE1572_5086	sce_4274	9.00E-27	8.00E-27
SCE1572_5087	sce_4275	2.00E-86	1.00E-93
SCE1572_5088	sce_4276	7.00E-160	8.00E-170
SCE1572_5089	sce_4277	3.00E-89	2.00E-89
SCE1572_509	sce_312	6.00E-94	3.00E-95
SCE1572_5090	sce_4278	0.00E+00	2.00E-171
SCE1572_5091	sce_4279	1.00E-172	2.00E-167
SCE1572_5093	sce_4280	8.00E-43	8.00E-43
SCE1572_5094	sce_4281	0.00E+00	0.00E+00
SCE1572_5095	sce_4282	0.00E+00	0.00E+00
SCE1572_5096	sce_4283	3.00E-139	3.00E-137
SCE1572_5097	sce_4284	1.00E-110	9.00E-110
SCE1572_5098	sce_4285	4.00E-155	2.00E-169
SCE1572_5099	sce_4286	1.00E-99	2.00E-104
SCE1572_510	sce_313	1.00E-157	9.00E-155
SCE1572_5100	sce_4287	5.00E-49	2.00E-48
SCE1572_5101	sce_4288	2.00E-111	3.00E-122
SCE1572_5102	sce_4289	0.00E+00	0.00E+00
SCE1572_5103	sce_4290	0.00E+00	0.00E+00
SCE1572_5104	sce_4291	4.00E-76	8.00E-64
SCE1572_5105	sce_4292	0.00E+00	0.00E+00
SCE1572_5106	sce_4293	4.00E-28	4.00E-28
SCE1572_5107	sce_4294	2.00E-97	1.00E-97
SCE1572_5108	sce_4295	1.00E-101	7.00E-95
SCE1572_511	sce_315	0.00E+00	0.00E+00
SCE1572_5110	sce_4297	0.00E+00	0.00E+00
SCE1572_5111	sce_4298	0.00E+00	0.00E+00
SCE1572_5112	sce_4299	3.00E-178	0.00E+00
SCE1572_5113	sce_4300	1.00E-69	5.00E-63
SCE1572_5114	sce_4301	0.00E+00	0.00E+00
SCE1572_5115	sce_4303	0.00E+00	4.00E-170
SCE1572_5116	sce_4304	2.00E-131	6.00E-146
SCE1572_5117	sce_4305	6.00E-80	2.00E-88
SCE1572_5118	sce_4306	4.00E-73	1.00E-63
SCE1572_5119	sce_4307	0.00E+00	0.00E+00
SCE1572_512	sce_317	3.00E-15	2.00E-12
SCE1572_5120	sce_4308	1.00E-169	2.00E-173
SCE1572_5121	sce_4309	0.00E+00	0.00E+00
SCE1572_5122	sce_4310	1.00E-69	1.00E-60
SCE1572_5123	sce_4311	4.00E-132	6.00E-139
SCE1572_5127	sce_4314	1.00E-180	0.00E+00
SCE1572_5128	sce_4315	2.00E-164	7.00E-179
SCE1572_513	sce_318	3.00E-131	1.00E-132
SCE1572_5130	sce_4317	6.00E-54	6.00E-54
SCE1572_5131	sce_4318	2.00E-53	5.00E-49
SCE1572_5132	sce_4319	5.00E-147	4.00E-154
SCE1572_5134	sce_4320	1.00E-25	2.00E-33
SCE1572_5135	sce_4321	1.00E-160	8.00E-161
SCE1572_5136	sce_4322	3.00E-139	2.00E-134
SCE1572_5137	sce_4323	5.00E-128	4.00E-141
SCE1572_514	sce_319	9.00E-88	2.00E-96
SCE1572_5141	sce_4324	0.00E+00	0.00E+00
SCE1572_5142	sce_4325	0.00E+00	3.00E-173
SCE1572_5145	sce_1505	2.00E-121	9.00E-116
SCE1572_5146	sce_3919	2.00E-53	9.00E-40

SCE1572_5147	sce_1503	4.00E-49	1.00E-69
SCE1572_5148	sce_4330	5.00E-95	6.00E-97
SCE1572_515	sce_321	5.00E-140	4.00E-140
SCE1572_5150	sce_4331	2.00E-86	3.00E-93
SCE1572_5154	sce_4332	4.00E-82	4.00E-82
SCE1572_5157	sce_4333	0.00E+00	0.00E+00
SCE1572_5159	sce_4334	1.00E-88	1.00E-88
SCE1572_5160	sce_4335	9.00E-132	6.00E-114
SCE1572_5164	sce_7843	2.00E-40	1.00E-37
SCE1572_5170	sce_4336	0.00E+00	0.00E+00
SCE1572_5171	sce_4337	7.00E-175	0.00E+00
SCE1572_5177	sce_4338	0.00E+00	0.00E+00
SCE1572_5178	sce_4339	0.00E+00	0.00E+00
SCE1572_5180	sce_4340	0.00E+00	0.00E+00
SCE1572_5181	sce_4342	4.00E-139	6.00E-142
SCE1572_5182	sce_4343	1.00E-112	9.00E-120
SCE1572_5183	sce_4344	4.00E-94	9.00E-94
SCE1572_5184	sce_4345	1.00E-114	5.00E-103
SCE1572_5185	sce_4346	5.00E-119	1.00E-119
SCE1572_5186	sce_4347	0.00E+00	0.00E+00
SCE1572_519	sce_2023	1.00E-68	3.00E-69
SCE1572_520	sce_322	6.00E-48	1.00E-50
SCE1572_5200	sce_4359	1.00E-39	6.00E-40
SCE1572_5202	sce_4360	2.00E-168	1.00E-167
SCE1572_5203	sce_4361	7.00E-21	5.00E-23
SCE1572_5204	sce_4362	2.00E-144	2.00E-154
SCE1572_5205	sce_4363	5.00E-137	2.00E-135
SCE1572_5206	sce_4364	1.00E-88	2.00E-94
SCE1572_5207	sce_4365	3.00E-86	6.00E-90
SCE1572_5208	sce_4366	3.00E-16	5.00E-26
SCE1572_5209	sce_4367	2.00E-15	9.00E-29
SCE1572_521	sce_323	2.00E-41	3.00E-43
SCE1572_5211	sce_4368	0.00E+00	0.00E+00
SCE1572_5212	sce_9054	3.00E-106	3.00E-106
SCE1572_5213	sce_4370	3.00E-162	2.00E-154
SCE1572_5214	sce_4371	3.00E-98	4.00E-90
SCE1572_5215	sce_4372	2.00E-142	2.00E-142
SCE1572_5217	sce_4373	0.00E+00	0.00E+00
SCE1572_522	sce_325	4.00E-103	4.00E-103
SCE1572_5220	sce_4374	3.00E-168	7.00E-86
SCE1572_5221	sce_4375	3.00E-25	1.00E-25
SCE1572_5222	sce_4377	0.00E+00	0.00E+00
SCE1572_5223	sce_4378	4.00E-173	6.00E-161
SCE1572_5224	sce_4379	3.00E-110	3.00E-101
SCE1572_5225	sce_4380	3.00E-11	1.00E-17
SCE1572_5226	sce_4381	2.00E-104	7.00E-105
SCE1572_5227	sce_4382	1.00E-07	1.00E-07
SCE1572_5228	sce_4383	7.00E-129	7.00E-129
SCE1572_5229	sce_4384	7.00E-49	7.00E-49
SCE1572_523	sce_326	2.00E-141	9.00E-141
SCE1572_5230	sce_4385	0.00E+00	0.00E+00
SCE1572_5231	sce_4386	0.00E+00	0.00E+00
SCE1572_5232	sce_4387	1.00E-70	1.00E-70
SCE1572_5233	sce_4388	3.00E-41	5.00E-41
SCE1572_5234	sce_4389	6.00E-63	6.00E-63
SCE1572_5235	sce_4390	0.00E+00	0.00E+00
SCE1572_5236	sce_4391	0.00E+00	0.00E+00
SCE1572_5237	sce_4392	1.00E-172	8.00E-173
SCE1572_5238	sce_4393	0.00E+00	0.00E+00
SCE1572_5239	sce_4394	2.00E-108	3.00E-124
SCE1572_524	sce_327	3.00E-91	1.00E-100
SCE1572_5240	sce_4395	5.00E-41	7.00E-45
SCE1572_5241	sce_4396	1.00E-69	3.00E-72
SCE1572_5242	sce_4397	3.00E-47	9.00E-48
SCE1572_5243	sce_4398	4.00E-24	5.00E-24
SCE1572_5244	sce_4399	3.00E-166	4.00E-170
SCE1572_5245	sce_4400	3.00E-93	6.00E-106
SCE1572_5246	sce_4402	2.00E-82	1.00E-82
SCE1572_5247	sce_4403	1.00E-54	2.00E-53
SCE1572_5248	sce_4404	2.00E-159	2.00E-172
SCE1572_5249	sce_4405	0.00E+00	0.00E+00
SCE1572_525	sce_328	4.00E-73	4.00E-73
SCE1572_5250	sce_4406	0.00E+00	0.00E+00
SCE1572_5251	sce_4407	4.00E-164	3.00E-170
SCE1572_5252	sce_4408	3.00E-116	1.00E-117
SCE1572_5254	sce_4409	1.00E-155	1.00E-173

SCE1572_5255	sce_4410	1.00E-09	2.00E-09
SCE1572_5256	sce_4411	6.00E-28	5.00E-31
SCE1572_5257	sce_4412	1.00E-98	2.00E-97
SCE1572_5259	sce_4413	3.00E-160	7.00E-163
SCE1572_526	sce_329	2.00E-172	3.00E-174
SCE1572_5260	sce_4415	7.00E-151	1.00E-162
SCE1572_5261	sce_4416	6.00E-71	6.00E-51
SCE1572_5264	sce_4418	4.00E-101	9.00E-102
SCE1572_5266	sce_4420	4.00E-163	4.00E-160
SCE1572_5267	sce_4421	0.00E+00	0.00E+00
SCE1572_5268	sce_4422	1.00E-50	6.00E-61
SCE1572_5269	sce_4423	0.00E+00	0.00E+00
SCE1572_527	sce_330	1.00E-76	2.00E-93
SCE1572_5270	sce_4424	2.00E-30	2.00E-30
SCE1572_5273	sce_4427	6.00E-23	2.00E-29
SCE1572_5276	sce_4428	1.00E-21	1.00E-18
SCE1572_5278	sce_4431	1.00E-133	2.00E-132
SCE1572_5279	sce_4432	0.00E+00	0.00E+00
SCE1572_528	sce_331	8.00E-74	1.00E-81
SCE1572_5280	sce_4433	2.00E-123	6.00E-126
SCE1572_5283	sce_4434	5.00E-100	5.00E-104
SCE1572_5284	sce_4435	6.00E-106	4.00E-106
SCE1572_5285	sce_4436	1.00E-21	3.00E-22
SCE1572_5286	sce_4439	1.00E-177	0.00E+00
SCE1572_5287	sce_4440	5.00E-24	5.00E-27
SCE1572_5288	sce_4441	0.00E+00	0.00E+00
SCE1572_529	sce_332	1.00E-20	2.00E-28
SCE1572_5290	sce_4444	0.00E+00	0.00E+00
SCE1572_5292	sce_4448	9.00E-76	2.00E-76
SCE1572_5295	sce_4451	7.00E-99	3.00E-104
SCE1572_5296	sce_9863	2.00E-20	2.00E-17
SCE1572_5297	sce_4452	6.00E-56	5.00E-49
SCE1572_5298	sce_4454	1.00E-106	3.00E-108
SCE1572_5299	sce_4455	0.00E+00	0.00E+00
SCE1572_53	sce_9404	0.00E+00	0.00E+00
SCE1572_530	sce_333	0.00E+00	0.00E+00
SCE1572_5304	sce_4456	6.00E-62	7.00E-62
SCE1572_5306	sce_1644	8.00E-125	8.00E-125
SCE1572_5307	sce_1643	1.00E-99	1.00E-99
SCE1572_5308	sce_1642	4.00E-139	6.00E-139
SCE1572_5309	sce_1641	1.00E-131	3.00E-132
SCE1572_531	sce_334	0.00E+00	0.00E+00
SCE1572_5310	sce_1640	1.00E-70	6.00E-64
SCE1572_5311	sce_1639	4.00E-63	3.00E-63
SCE1572_5315	sce_4457	4.00E-38	8.00E-37
SCE1572_532	sce_121	2.00E-98	4.00E-93
SCE1572_5321	sce_9369	4.00E-54	4.00E-54
SCE1572_5324	sce_4460	0.00E+00	0.00E+00
SCE1572_5325	sce_4462	6.00E-65	2.00E-64
SCE1572_5327	sce_4463	0.00E+00	0.00E+00
SCE1572_533	sce_339	2.00E-33	2.00E-36
SCE1572_5331	sce_4465	9.00E-130	3.00E-132
SCE1572_5332	sce_4466	0.00E+00	0.00E+00
SCE1572_5332	sce_7643	0.00E+00	0.00E+00
SCE1572_5333	sce_4467	1.00E-122	6.00E-147
SCE1572_5334	sce_2438	1.00E-164	6.00E-168
SCE1572_5335	sce_6710	0.00E+00	0.00E+00
SCE1572_5336	sce_4472	9.00E-97	2.00E-98
SCE1572_5337	sce_3082	0.00E+00	0.00E+00
SCE1572_5339	sce_3073	6.00E-32	1.00E-37
SCE1572_5340	sce_3074	0.00E+00	0.00E+00
SCE1572_5341	sce_3075	0.00E+00	0.00E+00
SCE1572_5341	sce_9735	0.00E+00	0.00E+00
SCE1572_5342	sce_3076	8.00E-63	3.00E-52
SCE1572_5345	sce_4471	1.00E-100	1.00E-100
SCE1572_5346	sce_4473	6.00E-25	9.00E-19
SCE1572_5347	sce_4474	6.00E-93	7.00E-102
SCE1572_5348	sce_4475	6.00E-153	1.00E-152
SCE1572_535	sce_341	0.00E+00	0.00E+00
SCE1572_5352	sce_4477	0.00E+00	0.00E+00
SCE1572_5353	sce_8197	4.00E-07	5.00E-07
SCE1572_5354	sce_4479	2.00E-81	7.00E-87
SCE1572_5355	sce_4481	3.00E-101	4.00E-101
SCE1572_5359	sce_4482	1.00E-79	8.00E-48
SCE1572_536	sce_342	2.00E-107	3.00E-101
SCE1572_5361	sce_4484	3.00E-141	3.00E-141

SCE1572_5362	sce_6192	2.00E-32	3.00E-42
SCE1572_5363	sce_6193	2.00E-106	4.00E-168
SCE1572_5365	sce_6194	1.00E-100	6.00E-102
SCE1572_5368	sce_4493	1.00E-58	1.00E-39
SCE1572_5369	sce_4494	0.00E+00	0.00E+00
SCE1572_537	sce_343	0.00E+00	0.00E+00
SCE1572_5371	sce_4495	0.00E+00	0.00E+00
SCE1572_5372	sce_7583	0.00E+00	0.00E+00
SCE1572_5373	sce_7582	3.00E-73	2.00E-73
SCE1572_5374	sce_4496	0.00E+00	0.00E+00
SCE1572_5375	sce_7141	6.00E-33	1.00E-26
SCE1572_5376	sce_4498	0.00E+00	0.00E+00
SCE1572_5377	sce_4499	0.00E+00	0.00E+00
SCE1572_5379	sce_4505	6.00E-144	6.00E-144
SCE1572_538	sce_344	8.00E-170	1.00E-173
SCE1572_5380	sce_4506	6.00E-135	3.00E-122
SCE1572_5381	sce_4507	0.00E+00	1.00E-166
SCE1572_5382	sce_4508	6.00E-170	3.00E-160
SCE1572_5384	sce_2062	4.00E-35	5.00E-34
SCE1572_5385	sce_4509	0.00E+00	0.00E+00
SCE1572_5386	sce_4510	3.00E-177	6.00E-171
SCE1572_5387	sce_4511	0.00E+00	0.00E+00
SCE1572_5388	sce_4512	1.00E-166	1.00E-163
SCE1572_5389	sce_4513	0.00E+00	0.00E+00
SCE1572_5389	sce_10204	0.00E+00	0.00E+00
SCE1572_5389	sce_388	0.00E+00	0.00E+00
SCE1572_5389	sce_5835	0.00E+00	0.00E+00
SCE1572_5389	sce_7640	0.00E+00	0.00E+00
SCE1572_5389	sce_3085	0.00E+00	0.00E+00
SCE1572_5389	sce_9557	0.00E+00	0.00E+00
SCE1572_5389	sce_7109	0.00E+00	0.00E+00
SCE1572_5389	sce_6535	0.00E+00	0.00E+00
SCE1572_5389	sce_3003	0.00E+00	0.00E+00
SCE1572_5389	sce_6093	0.00E+00	0.00E+00
SCE1572_5389	sce_6095	0.00E+00	0.00E+00
SCE1572_5389	sce_9920	0.00E+00	0.00E+00
SCE1572_5389	sce_6107	0.00E+00	0.00E+00
SCE1572_5389	sce_3917	0.00E+00	0.00E+00
SCE1572_5389	sce_6092	0.00E+00	0.00E+00
SCE1572_5389	sce_7661	0.00E+00	0.00E+00
SCE1572_5389	sce_8618	0.00E+00	0.00E+00
SCE1572_539	sce_345	4.00E-56	3.00E-50
SCE1572_5391	sce_4516	2.00E-162	2.00E-162
SCE1572_5392	sce_4517	3.00E-138	3.00E-138
SCE1572_5393	sce_4518	7.00E-149	7.00E-149
SCE1572_5394	sce_4519	0.00E+00	0.00E+00
SCE1572_54	sce_9403	2.00E-39	3.00E-48
SCE1572_540	sce_346	0.00E+00	0.00E+00
SCE1572_5400	sce_4522	0.00E+00	0.00E+00
SCE1572_5401	sce_4523	8.00E-38	1.00E-30
SCE1572_5402	sce_4524	7.00E-106	9.00E-105
SCE1572_5404	sce_4537	4.00E-60	4.00E-60
SCE1572_5405	sce_4538	2.00E-128	2.00E-128
SCE1572_5406	sce_4539	5.00E-62	9.00E-61
SCE1572_5408	sce_4540	6.00E-120	4.00E-103
SCE1572_541	sce_347	6.00E-90	1.00E-86
SCE1572_5412	sce_4562	0.00E+00	1.00E-176
SCE1572_5414	sce_4564	2.00E-122	3.00E-128
SCE1572_5415	sce_4565	0.00E+00	0.00E+00
SCE1572_5416	sce_4566	2.00E-113	6.00E-107
SCE1572_5417	sce_4567	3.00E-161	2.00E-159
SCE1572_5418	sce_10011	9.00E-38	2.00E-47
SCE1572_5419	sce_9329	6.00E-76	1.00E-62
SCE1572_542	sce_348	9.00E-120	7.00E-119
SCE1572_5420	sce_4568	7.00E-131	5.00E-139
SCE1572_5421	sce_4569	3.00E-76	1.00E-79
SCE1572_5423	sce_4573	0.00E+00	0.00E+00
SCE1572_5426	sce_1600	5.00E-13	3.00E-18
SCE1572_5427	sce_3045	1.00E-58	2.00E-59
SCE1572_5429	sce_4574	0.00E+00	0.00E+00
SCE1572_543	sce_349	4.00E-120	3.00E-100
SCE1572_5430	sce_7563	0.00E+00	0.00E+00
SCE1572_5434	sce_4578	0.00E+00	0.00E+00
SCE1572_544	sce_350	3.00E-57	3.00E-57
SCE1572_545	sce_352	4.00E-144	2.00E-145
SCE1572_5451	sce_5278	0.00E+00	0.00E+00

SCE1572_5452	sce_5276	0.00E+00	0.00E+00
SCE1572_5452	sce_2161	0.00E+00	0.00E+00
SCE1572_5452	sce_225	0.00E+00	0.00E+00
SCE1572_5452	sce_8357	0.00E+00	0.00E+00
SCE1572_5452	sce_6235	0.00E+00	0.00E+00
SCE1572_5468	sce_388	0.00E+00	0.00E+00
SCE1572_5468	sce_5835	0.00E+00	0.00E+00
SCE1572_5468	sce_7640	0.00E+00	0.00E+00
SCE1572_5468	sce_10204	0.00E+00	0.00E+00
SCE1572_5468	sce_9920	0.00E+00	0.00E+00
SCE1572_5468	sce_7661	0.00E+00	0.00E+00
SCE1572_5468	sce_3085	0.00E+00	0.00E+00
SCE1572_5468	sce_9557	0.00E+00	0.00E+00
SCE1572_5468	sce_3003	0.00E+00	0.00E+00
SCE1572_5468	sce_6092	0.00E+00	0.00E+00
SCE1572_5468	sce_4513	0.00E+00	0.00E+00
SCE1572_5468	sce_6095	0.00E+00	0.00E+00
SCE1572_5468	sce_7109	0.00E+00	0.00E+00
SCE1572_5468	sce_6094	0.00E+00	0.00E+00
SCE1572_5468	sce_6535	0.00E+00	0.00E+00
SCE1572_5468	sce_2065	0.00E+00	0.00E+00
SCE1572_5468	sce_6093	0.00E+00	0.00E+00
SCE1572_5468	sce_3917	0.00E+00	0.00E+00
SCE1572_5468	sce_6107	0.00E+00	0.00E+00
SCE1572_5468	sce_1398	0.00E+00	0.00E+00
SCE1572_5468	sce_8602	0.00E+00	0.00E+00
SCE1572_547	sce_353	2.00E-107	3.00E-115
SCE1572_5470	sce_4595	2.00E-52	1.00E-53
SCE1572_5475	sce_8779	5.00E-101	2.00E-84
SCE1572_5476	sce_8781	0.00E+00	0.00E+00
SCE1572_5477	sce_8782	2.00E-128	6.00E-153
SCE1572_548	sce_355	0.00E+00	0.00E+00
SCE1572_5480	sce_7640	0.00E+00	0.00E+00
SCE1572_5480	sce_388	0.00E+00	0.00E+00
SCE1572_5480	sce_5835	0.00E+00	0.00E+00
SCE1572_5480	sce_10204	0.00E+00	0.00E+00
SCE1572_5480	sce_3085	0.00E+00	0.00E+00
SCE1572_5480	sce_4513	0.00E+00	0.00E+00
SCE1572_5480	sce_9557	0.00E+00	0.00E+00
SCE1572_5480	sce_7109	0.00E+00	0.00E+00
SCE1572_5480	sce_9920	0.00E+00	0.00E+00
SCE1572_5480	sce_6107	0.00E+00	0.00E+00
SCE1572_5480	sce_3003	0.00E+00	0.00E+00
SCE1572_5480	sce_6535	0.00E+00	0.00E+00
SCE1572_5480	sce_6092	0.00E+00	0.00E+00
SCE1572_5480	sce_6093	0.00E+00	0.00E+00
SCE1572_5480	sce_7661	0.00E+00	0.00E+00
SCE1572_5480	sce_3917	0.00E+00	0.00E+00
SCE1572_5480	sce_6095	0.00E+00	0.00E+00
SCE1572_5480	sce_6094	0.00E+00	0.00E+00
SCE1572_5480	sce_2065	0.00E+00	0.00E+00
SCE1572_5480	sce_8618	0.00E+00	0.00E+00
SCE1572_5482	sce_5502	0.00E+00	0.00E+00
SCE1572_5482	sce_2641	0.00E+00	0.00E+00
SCE1572_5483	sce_5480	7.00E-121	5.00E-109
SCE1572_5484	sce_5479	3.00E-179	0.00E+00
SCE1572_5485	sce_7637	0.00E+00	0.00E+00
SCE1572_5485	sce_389	0.00E+00	0.00E+00
SCE1572_5485	sce_2666	0.00E+00	0.00E+00
SCE1572_5485	sce_9230	0.00E+00	0.00E+00
SCE1572_5485	sce_9237	0.00E+00	0.00E+00
SCE1572_5487	sce_4598	1.00E-112	2.00E-121
SCE1572_5488	sce_4599	2.00E-111	2.00E-110
SCE1572_55	sce_8347	0.00E+00	0.00E+00
SCE1572_55	sce_4610	0.00E+00	0.00E+00
SCE1572_5504	sce_2328	1.00E-21	3.00E-10
SCE1572_551	sce_357	2.00E-68	2.00E-67
SCE1572_5510	sce_2321	1.00E-14	1.00E-15
SCE1572_5512	sce_2319	4.00E-60	8.00E-62
SCE1572_552	sce_358	0.00E+00	0.00E+00
SCE1572_5522	sce_6361	6.00E-47	9.00E-56
SCE1572_5528	sce_7463	2.00E-37	6.00E-31
SCE1572_5529	sce_3646	9.00E-179	4.00E-179
SCE1572_5533	sce_3598	0.00E+00	0.00E+00
SCE1572_5536	sce_3597	0.00E+00	0.00E+00
SCE1572_5537	sce_3596	0.00E+00	0.00E+00

SCE1572_5538	sce_3595	0.00E+00	0.00E+00
SCE1572_5539	sce_3594	0.00E+00	0.00E+00
SCE1572_5540	sce_3593	4.00E-121	2.00E-128
SCE1572_5541	sce_3592	5.00E-89	5.00E-89
SCE1572_5542	sce_3590	0.00E+00	0.00E+00
SCE1572_5543	sce_3589	5.00E-38	5.00E-38
SCE1572_5544	sce_3588	9.00E-179	5.00E-179
SCE1572_556	sce_359	5.00E-93	7.00E-101
SCE1572_557	sce_360	0.00E+00	0.00E+00
SCE1572_5570	sce_7637	0.00E+00	0.00E+00
SCE1572_5570	sce_9230	0.00E+00	0.00E+00
SCE1572_5570	sce_389	0.00E+00	0.00E+00
SCE1572_5570	sce_2666	0.00E+00	0.00E+00
SCE1572_5570	sce_9237	0.00E+00	0.00E+00
SCE1572_5570	sce_9231	0.00E+00	0.00E+00
SCE1572_5571	sce_9230	0.00E+00	0.00E+00
SCE1572_5571	sce_7637	0.00E+00	0.00E+00
SCE1572_5571	sce_389	0.00E+00	0.00E+00
SCE1572_5571	sce_2666	0.00E+00	0.00E+00
SCE1572_5571	sce_9237	0.00E+00	0.00E+00
SCE1572_5571	sce_9231	0.00E+00	0.00E+00
SCE1572_5572	sce_9230	0.00E+00	0.00E+00
SCE1572_5572	sce_389	0.00E+00	0.00E+00
SCE1572_5572	sce_2666	0.00E+00	0.00E+00
SCE1572_5572	sce_7637	0.00E+00	0.00E+00
SCE1572_5572	sce_9237	0.00E+00	0.00E+00
SCE1572_5572	sce_9231	0.00E+00	0.00E+00
SCE1572_5579	sce_4645	0.00E+00	0.00E+00
SCE1572_558	sce_361	1.00E-106	2.00E-103
SCE1572_5580	sce_4646	1.00E-159	6.00E-165
SCE1572_5581	sce_4647	3.00E-144	1.00E-139
SCE1572_5582	sce_1162	3.00E-98	4.00E-104
SCE1572_5583	sce_1161	3.00E-133	4.00E-140
SCE1572_5584	sce_1160	1.00E-149	4.00E-160
SCE1572_5585	sce_4648	3.00E-111	2.00E-102
SCE1572_5587	sce_4650	1.00E-91	3.00E-95
SCE1572_559	sce_362	1.00E-52	1.00E-52
SCE1572_5592	sce_4656	0.00E+00	0.00E+00
SCE1572_5594	sce_4657	6.00E-142	7.00E-142
SCE1572_5596	sce_4658	0.00E+00	0.00E+00
SCE1572_5597	sce_4660	0.00E+00	0.00E+00
SCE1572_5598	sce_4661	2.00E-160	3.00E-171
SCE1572_5599	sce_5147	0.00E+00	0.00E+00
SCE1572_5599	sce_4662	0.00E+00	0.00E+00
SCE1572_560	sce_363	6.00E-176	6.00E-176
SCE1572_5600	sce_4663	0.00E+00	0.00E+00
SCE1572_5600	sce_5148	0.00E+00	0.00E+00
SCE1572_5601	sce_4665	0.00E+00	0.00E+00
SCE1572_5602	sce_4671	0.00E+00	0.00E+00
SCE1572_5606	sce_4684	4.00E-139	1.00E-147
SCE1572_5607	sce_6014	0.00E+00	0.00E+00
SCE1572_5608	sce_4685	6.00E-69	8.00E-63
SCE1572_5609	sce_4686	0.00E+00	0.00E+00
SCE1572_561	sce_364	1.00E-142	1.00E-143
SCE1572_5612	sce_5882	9.00E-60	6.00E-60
SCE1572_5614	sce_4701	2.00E-79	7.00E-78
SCE1572_5617	sce_4702	0.00E+00	1.00E-176
SCE1572_5619	sce_4704	1.00E-87	5.00E-90
SCE1572_562	sce_365	6.00E-130	9.00E-122
SCE1572_5622	sce_4705	0.00E+00	0.00E+00
SCE1572_5623	sce_4706	0.00E+00	0.00E+00
SCE1572_5625	sce_4707	2.00E-34	3.00E-27
SCE1572_563	sce_366	3.00E-96	1.00E-85
SCE1572_5630	sce_7538	0.00E+00	0.00E+00
SCE1572_5635	sce_8649	0.00E+00	0.00E+00
SCE1572_5635	sce_6571	0.00E+00	0.00E+00
SCE1572_5636	sce_8648	1.00E-141	1.00E-141
SCE1572_5637	sce_4709	0.00E+00	0.00E+00
SCE1572_5639	sce_4710	1.00E-92	5.00E-104
SCE1572_5640	sce_7661	0.00E+00	0.00E+00
SCE1572_5640	sce_8602	0.00E+00	0.00E+00
SCE1572_5640	sce_3917	0.00E+00	0.00E+00
SCE1572_5640	sce_6107	0.00E+00	0.00E+00
SCE1572_5640	sce_6092	0.00E+00	0.00E+00
SCE1572_5640	sce_6093	0.00E+00	0.00E+00
SCE1572_5640	sce_6094	0.00E+00	0.00E+00

SCE1572_5640	sce_6095	0.00E+00	0.00E+00
SCE1572_5640	sce_6535	0.00E+00	0.00E+00
SCE1572_5640	sce_388	0.00E+00	0.00E+00
SCE1572_5640	sce_10204	0.00E+00	0.00E+00
SCE1572_5640	sce_9920	0.00E+00	0.00E+00
SCE1572_5640	sce_3003	0.00E+00	0.00E+00
SCE1572_5640	sce_9557	0.00E+00	0.00E+00
SCE1572_5640	sce_5835	0.00E+00	0.00E+00
SCE1572_5640	sce_7640	0.00E+00	0.00E+00
SCE1572_5640	sce_2065	0.00E+00	0.00E+00
SCE1572_5640	sce_8618	0.00E+00	0.00E+00
SCE1572_5640	sce_4513	0.00E+00	0.00E+00
SCE1572_5640	sce_3085	0.00E+00	0.00E+00
SCE1572_5640	sce_7109	0.00E+00	0.00E+00
SCE1572_5649	sce_4711	0.00E+00	0.00E+00
SCE1572_5649	sce_3376	0.00E+00	0.00E+00
SCE1572_565	sce_368	7.00E-73	7.00E-73
SCE1572_5652	sce_9774	3.00E-134	8.00E-150
SCE1572_5654	sce_9776	0.00E+00	0.00E+00
SCE1572_5654	sce_5358	0.00E+00	0.00E+00
SCE1572_5655	sce_9777	0.00E+00	0.00E+00
SCE1572_5655	sce_5359	0.00E+00	0.00E+00
SCE1572_5656	sce_1130	5.00E-22	5.00E-55
SCE1572_5658	sce_4713	0.00E+00	0.00E+00
SCE1572_5659	sce_9663	0.00E+00	0.00E+00
SCE1572_5663	sce_4714	0.00E+00	0.00E+00
SCE1572_5664	sce_4715	2.00E-166	1.00E-155
SCE1572_5665	sce_4716	2.00E-62	2.00E-67
SCE1572_5666	sce_4717	5.00E-122	3.00E-130
SCE1572_5668	sce_4718	9.00E-29	9.00E-28
SCE1572_5669	sce_4719	4.00E-134	3.00E-139
SCE1572_567	sce_370	0.00E+00	0.00E+00
SCE1572_567	sce_41	0.00E+00	0.00E+00
SCE1572_5670	sce_4720	3.00E-176	5.00E-164
SCE1572_5671	sce_4721	1.00E-130	1.00E-130
SCE1572_5673	sce_4722	1.00E-103	7.00E-111
SCE1572_5675	sce_4727	1.00E-170	1.00E-152
SCE1572_5676	sce_4728	8.00E-45	1.00E-37
SCE1572_5677	sce_4729	5.00E-129	1.00E-125
SCE1572_5678	sce_4730	0.00E+00	4.00E-175
SCE1572_5679	sce_4731	0.00E+00	0.00E+00
SCE1572_568	sce_371	5.00E-48	3.00E-48
SCE1572_5680	sce_4732	0.00E+00	0.00E+00
SCE1572_5681	sce_4733	8.00E-30	9.00E-30
SCE1572_5682	sce_4734	8.00E-66	1.00E-62
SCE1572_5683	sce_4735	0.00E+00	0.00E+00
SCE1572_5684	sce_4736	1.00E-89	1.00E-89
SCE1572_5685	sce_4738	3.00E-66	3.00E-60
SCE1572_5686	sce_4739	1.00E-48	1.00E-48
SCE1572_5688	sce_4740	7.00E-99	7.00E-99
SCE1572_5689	sce_4741	1.00E-119	1.00E-119
SCE1572_569	sce_372	6.00E-20	5.00E-22
SCE1572_5690	sce_4742	8.00E-70	3.00E-77
SCE1572_5691	sce_4743	7.00E-149	3.00E-163
SCE1572_5692	sce_4744	0.00E+00	0.00E+00
SCE1572_5693	sce_4745	4.00E-96	7.00E-89
SCE1572_5694	sce_4746	0.00E+00	2.00E-173
SCE1572_5695	sce_9269	1.00E-46	1.00E-46
SCE1572_5697	sce_4750	0.00E+00	0.00E+00
SCE1572_5699	sce_4751	1.00E-92	5.00E-92
SCE1572_57	sce_6254	6.00E-92	5.00E-89
SCE1572_570	sce_804	2.00E-138	2.00E-141
SCE1572_5700	sce_4752	2.00E-64	2.00E-60
SCE1572_5701	sce_4753	3.00E-37	7.00E-44
SCE1572_5702	sce_4754	0.00E+00	0.00E+00
SCE1572_5703	sce_4755	0.00E+00	0.00E+00
SCE1572_5704	sce_4756	7.00E-146	7.00E-146
SCE1572_5705	sce_4757	9.00E-72	4.00E-58
SCE1572_5706	sce_4758	2.00E-97	2.00E-94
SCE1572_5707	sce_4759	6.00E-102	2.00E-102
SCE1572_5708	sce_4761	0.00E+00	0.00E+00
SCE1572_5709	sce_4762	0.00E+00	0.00E+00
SCE1572_5710	sce_4763	3.00E-11	1.00E-15
SCE1572_5711	sce_4764	0.00E+00	0.00E+00
SCE1572_5712	sce_4765	2.00E-55	2.00E-55
SCE1572_5713	sce_4766	4.00E-56	2.00E-56

SCE1572_5714	sce_4767	1.00E-104	4.00E-110
SCE1572_5715	sce_4768	1.00E-48	8.00E-49
SCE1572_5716	sce_4769	0.00E+00	0.00E+00
SCE1572_5716	sce_1353	0.00E+00	0.00E+00
SCE1572_5717	sce_4770	1.00E-48	1.00E-47
SCE1572_5718	sce_4771	4.00E-160	7.00E-160
SCE1572_5719	sce_4772	2.00E-115	2.00E-115
SCE1572_5720	sce_4773	2.00E-159	3.00E-159
SCE1572_5721	sce_4774	3.00E-166	3.00E-164
SCE1572_5722	sce_4775	8.00E-127	2.00E-129
SCE1572_5726	sce_4776	0.00E+00	0.00E+00
SCE1572_5727	sce_4778	0.00E+00	0.00E+00
SCE1572_5728	sce_4779	8.00E-106	4.00E-103
SCE1572_5729	sce_4780	1.00E-46	1.00E-50
SCE1572_573	sce_375	0.00E+00	0.00E+00
SCE1572_5731	sce_4781	6.00E-38	5.00E-46
SCE1572_5732	sce_4782	7.00E-94	2.00E-104
SCE1572_5733	sce_4783	8.00E-167	2.00E-169
SCE1572_5734	sce_4784	0.00E+00	0.00E+00
SCE1572_5735	sce_4785	0.00E+00	0.00E+00
SCE1572_5736	sce_4786	8.00E-48	2.00E-54
SCE1572_5737	sce_4788	1.00E-58	4.00E-54
SCE1572_5738	sce_4789	6.00E-45	2.00E-45
SCE1572_5739	sce_4790	2.00E-69	2.00E-70
SCE1572_574	sce_376	9.00E-108	1.00E-115
SCE1572_5740	sce_4791	4.00E-87	4.00E-87
SCE1572_5741	sce_4792	4.00E-137	6.00E-135
SCE1572_5742	sce_4793	2.00E-129	1.00E-116
SCE1572_5744	sce_4794	0.00E+00	0.00E+00
SCE1572_5745	sce_4795	2.00E-52	2.00E-52
SCE1572_5746	sce_4796	0.00E+00	0.00E+00
SCE1572_5747	sce_4797	1.00E-65	6.00E-91
SCE1572_5748	sce_4798	2.00E-165	3.00E-165
SCE1572_5749	sce_4799	0.00E+00	0.00E+00
SCE1572_575	sce_377	5.00E-22	5.00E-22
SCE1572_5750	sce_4800	2.00E-61	3.00E-72
SCE1572_5751	sce_4801	0.00E+00	0.00E+00
SCE1572_5752	sce_4803	1.00E-124	2.00E-112
SCE1572_5754	sce_4805	7.00E-121	4.00E-121
SCE1572_5755	sce_4806	0.00E+00	0.00E+00
SCE1572_5759	sce_4807	5.00E-147	6.00E-169
SCE1572_576	sce_381	0.00E+00	0.00E+00
SCE1572_5760	sce_4808	8.00E-134	7.00E-122
SCE1572_5761	sce_4809	2.00E-155	2.00E-154
SCE1572_5762	sce_4810	5.00E-125	2.00E-114
SCE1572_5764	sce_4811	8.00E-147	3.00E-146
SCE1572_5765	sce_4812	0.00E+00	0.00E+00
SCE1572_5766	sce_4813	0.00E+00	0.00E+00
SCE1572_5767	sce_4814	0.00E+00	0.00E+00
SCE1572_5768	sce_4815	0.00E+00	0.00E+00
SCE1572_5769	sce_4816	4.00E-177	4.00E-177
SCE1572_5770	sce_4817	0.00E+00	0.00E+00
SCE1572_5770	sce_9407	0.00E+00	0.00E+00
SCE1572_5770	sce_8451	0.00E+00	0.00E+00
SCE1572_5770	sce_3765	0.00E+00	0.00E+00
SCE1572_5772	sce_4818	0.00E+00	0.00E+00
SCE1572_5774	sce_4820	3.00E-13	1.00E-15
SCE1572_5775	sce_4821	0.00E+00	0.00E+00
SCE1572_5776	sce_4822	0.00E+00	0.00E+00
SCE1572_5777	sce_4823	9.00E-56	1.00E-55
SCE1572_5778	sce_4824	2.00E-108	2.00E-108
SCE1572_5779	sce_4825	5.00E-100	8.00E-135
SCE1572_5780	sce_4826	5.00E-117	1.00E-117
SCE1572_5781	sce_4827	0.00E+00	0.00E+00
SCE1572_5782	sce_4828	2.00E-30	6.00E-31
SCE1572_5783	sce_4830	4.00E-141	2.00E-160
SCE1572_5784	sce_4831	3.00E-73	1.00E-100
SCE1572_5785	sce_4832	1.00E-68	1.00E-70
SCE1572_5786	sce_4833	3.00E-47	8.00E-48
SCE1572_5787	sce_4834	5.00E-86	5.00E-86
SCE1572_5788	sce_4835	6.00E-98	3.00E-104
SCE1572_5789	sce_4836	0.00E+00	0.00E+00
SCE1572_579	sce_384	2.00E-57	1.00E-58
SCE1572_5790	sce_4837	1.00E-176	1.00E-176
SCE1572_5791	sce_4838	0.00E+00	0.00E+00
SCE1572_5792	sce_4839	7.00E-52	4.00E-54

SCE1572_5793	sce_4840	3.00E-108	9.00E-122
SCE1572_5794	sce_4841	1.00E-119	2.00E-121
SCE1572_5795	sce_4842	9.00E-80	1.00E-78
SCE1572_5796	sce_4843	2.00E-104	3.00E-110
SCE1572_5797	sce_4844	2.00E-146	3.00E-158
SCE1572_5798	sce_4846	6.00E-107	4.00E-103
SCE1572_5799	sce_4847	5.00E-137	2.00E-136
SCE1572_58	sce_6262	3.00E-124	9.00E-129
SCE1572_580	sce_385	1.00E-116	1.00E-125
SCE1572_5800	sce_4848	7.00E-180	0.00E+00
SCE1572_5801	sce_4849	8.00E-109	5.00E-120
SCE1572_5802	sce_4850	2.00E-124	3.00E-126
SCE1572_5803	sce_4851	2.00E-88	6.00E-84
SCE1572_5804	sce_4852	2.00E-41	2.00E-42
SCE1572_5806	sce_4854	2.00E-31	2.00E-31
SCE1572_5807	sce_4855	2.00E-73	2.00E-80
SCE1572_5808	sce_4856	4.00E-163	2.00E-163
SCE1572_5809	sce_4857	0.00E+00	0.00E+00
SCE1572_581	sce_386	3.00E-159	6.00E-174
SCE1572_5810	sce_4858	3.00E-64	3.00E-73
SCE1572_5811	sce_4859	0.00E+00	0.00E+00
SCE1572_5812	sce_4861	0.00E+00	0.00E+00
SCE1572_5812	sce_4106	0.00E+00	0.00E+00
SCE1572_5813	sce_4862	2.00E-170	4.00E-167
SCE1572_5814	sce_4863	3.00E-111	0.00E+00
SCE1572_5815	sce_4864	0.00E+00	0.00E+00
SCE1572_5816	sce_4865	2.00E-176	3.00E-169
SCE1572_5818	sce_9910	7.00E-118	6.00E-106
SCE1572_582	sce_392	0.00E+00	2.00E-180
SCE1572_5820	sce_10365	0.00E+00	0.00E+00
SCE1572_5820	sce_9503	0.00E+00	0.00E+00
SCE1572_5820	sce_9908	0.00E+00	0.00E+00
SCE1572_5823	sce_4866	2.00E-58	1.00E-58
SCE1572_5825	sce_4867	0.00E+00	0.00E+00
SCE1572_5826	sce_4868	3.00E-133	6.00E-142
SCE1572_5827	sce_4869	0.00E+00	2.00E-171
SCE1572_5828	sce_4870	4.00E-150	1.00E-156
SCE1572_583	sce_393	1.00E-104	2.00E-120
SCE1572_5831	sce_4872	8.00E-99	1.00E-76
SCE1572_5832	sce_4873	1.00E-147	6.00E-163
SCE1572_5833	sce_4874	0.00E+00	0.00E+00
SCE1572_5834	sce_4875	2.00E-45	2.00E-45
SCE1572_5835	sce_4876	1.00E-135	2.00E-135
SCE1572_5837	sce_4878	0.00E+00	0.00E+00
SCE1572_5838	sce_4879	1.00E-94	4.00E-88
SCE1572_584	sce_394	1.00E-168	6.00E-161
SCE1572_585	sce_395	0.00E+00	0.00E+00
SCE1572_5850	sce_160	2.00E-112	5.00E-108
SCE1572_5853	sce_2040	4.00E-08	1.00E-07
SCE1572_5854	sce_7025	3.00E-15	2.00E-26
SCE1572_5855	sce_3173	4.00E-59	4.00E-59
SCE1572_586	sce_396	0.00E+00	0.00E+00
SCE1572_5868	sce_4880	1.00E-112	2.00E-102
SCE1572_5869	sce_4881	7.00E-102	4.00E-102
SCE1572_587	sce_398	8.00E-180	0.00E+00
SCE1572_5870	sce_4882	1.00E-62	2.00E-63
SCE1572_5871	sce_4883	0.00E+00	0.00E+00
SCE1572_5872	sce_4885	0.00E+00	0.00E+00
SCE1572_5873	sce_4887	0.00E+00	0.00E+00
SCE1572_5874	sce_4888	5.00E-51	5.00E-51
SCE1572_5875	sce_4889	3.00E-41	1.00E-43
SCE1572_5876	sce_4890	7.00E-138	6.00E-143
SCE1572_5879	sce_4891	4.00E-68	7.00E-75
SCE1572_588	sce_399	2.00E-75	6.00E-83
SCE1572_5883	sce_7152	8.00E-69	7.00E-69
SCE1572_5884	sce_7151	0.00E+00	0.00E+00
SCE1572_5886	sce_7494	1.00E-83	7.00E-71
SCE1572_5887	sce_4892	4.00E-130	4.00E-133
SCE1572_5889	sce_4893	6.00E-15	4.00E-15
SCE1572_5890	sce_4895	3.00E-176	5.00E-177
SCE1572_5893	sce_4896	2.00E-150	1.00E-152
SCE1572_5894	sce_7535	9.00E-52	1.00E-47
SCE1572_5895	sce_7536	0.00E+00	0.00E+00
SCE1572_5896	sce_7537	0.00E+00	0.00E+00
SCE1572_5898	sce_4908	2.00E-28	1.00E-34
SCE1572_5899	sce_2489	4.00E-89	8.00E-87

SCE1572_59	sce_6258	3.00E-74	1.00E-90
SCE1572_5900	sce_2490	2.00E-98	2.00E-105
SCE1572_5906	sce_4909	0.00E+00	0.00E+00
SCE1572_5907	sce_4911	6.00E-58	6.00E-58
SCE1572_5908	sce_4912	0.00E+00	0.00E+00
SCE1572_5909	sce_4913	9.00E-70	9.00E-70
SCE1572_591	sce_400	5.00E-158	8.00E-146
SCE1572_5910	sce_4914	2.00E-121	4.00E-106
SCE1572_5911	sce_4915	0.00E+00	0.00E+00
SCE1572_5912	sce_4916	2.00E-71	5.00E-73
SCE1572_5913	sce_4917	2.00E-136	8.00E-141
SCE1572_5914	sce_4918	3.00E-130	5.00E-140
SCE1572_5915	sce_4919	0.00E+00	0.00E+00
SCE1572_5916	sce_4920	6.00E-68	1.00E-66
SCE1572_5917	sce_4921	0.00E+00	5.00E-173
SCE1572_5919	sce_4923	1.00E-178	1.00E-178
SCE1572_592	sce_401	4.00E-105	2.00E-112
SCE1572_5920	sce_4924	0.00E+00	0.00E+00
SCE1572_5922	sce_4925	3.00E-133	9.00E-155
SCE1572_5923	sce_4926	0.00E+00	0.00E+00
SCE1572_5924	sce_4927	4.00E-39	2.00E-58
SCE1572_5925	sce_4928	0.00E+00	0.00E+00
SCE1572_5926	sce_4929	0.00E+00	0.00E+00
SCE1572_5927	sce_4930	0.00E+00	0.00E+00
SCE1572_5928	sce_6137	9.00E-85	1.00E-84
SCE1572_5929	sce_6138	4.00E-63	7.00E-55
SCE1572_593	sce_402	0.00E+00	0.00E+00
SCE1572_5931	sce_4931	0.00E+00	0.00E+00
SCE1572_5932	sce_4932	0.00E+00	0.00E+00
SCE1572_5940	sce_2546	1.00E-08	7.00E-07
SCE1572_595	sce_404	2.00E-137	1.00E-138
SCE1572_596	sce_405	0.00E+00	0.00E+00
SCE1572_5973	sce_4934	9.00E-142	9.00E-133
SCE1572_5975	sce_4935	2.00E-132	2.00E-139
SCE1572_5976	sce_4936	2.00E-122	9.00E-141
SCE1572_5977	sce_4937	2.00E-113	1.00E-110
SCE1572_5978	sce_4938	0.00E+00	0.00E+00
SCE1572_5979	sce_4939	1.00E-116	1.00E-116
SCE1572_598	sce_406	6.00E-75	4.00E-83
SCE1572_5980	sce_4941	4.00E-169	3.00E-170
SCE1572_5981	sce_4942	2.00E-43	4.00E-43
SCE1572_5983	sce_2441	1.00E-06	2.00E-07
SCE1572_5990	sce_9404	0.00E+00	0.00E+00
SCE1572_5991	sce_4943	2.00E-151	2.00E-154
SCE1572_5992	sce_4944	1.00E-137	4.00E-149
SCE1572_5993	sce_4945	2.00E-101	3.00E-92
SCE1572_5994	sce_4946	0.00E+00	0.00E+00
SCE1572_5995	sce_4947	9.00E-155	9.00E-148
SCE1572_5996	sce_4948	7.00E-75	4.00E-74
SCE1572_5997	sce_4949	6.00E-137	3.00E-152
SCE1572_5998	sce_4950	5.00E-153	7.00E-162
SCE1572_5999	sce_4951	1.00E-154	1.00E-154
SCE1572_6	sce_6	0.00E+00	0.00E+00
SCE1572_60	sce_50	0.00E+00	0.00E+00
SCE1572_6000	sce_4952	3.00E-103	4.00E-117
SCE1572_6001	sce_4953	9.00E-37	2.00E-37
SCE1572_6002	sce_4954	8.00E-77	3.00E-79
SCE1572_6003	sce_4955	4.00E-124	2.00E-104
SCE1572_6004	sce_4956	2.00E-135	7.00E-136
SCE1572_6005	sce_4957	9.00E-160	5.00E-166
SCE1572_6007	sce_4958	2.00E-107	2.00E-109
SCE1572_6008	sce_4959	9.00E-83	2.00E-74
SCE1572_6009	sce_4960	8.00E-32	8.00E-32
SCE1572_601	sce_407	0.00E+00	0.00E+00
SCE1572_6010	sce_4961	3.00E-40	3.00E-40
SCE1572_6011	sce_4963	0.00E+00	0.00E+00
SCE1572_6012	sce_4964	5.00E-151	5.00E-170
SCE1572_6013	sce_4965	0.00E+00	0.00E+00
SCE1572_6014	sce_4966	1.00E-126	4.00E-120
SCE1572_6016	sce_4969	2.00E-90	2.00E-90
SCE1572_6017	sce_4970	0.00E+00	0.00E+00
SCE1572_6018	sce_4971	0.00E+00	0.00E+00
SCE1572_6019	sce_4972	1.00E-29	5.00E-29
SCE1572_602	sce_410	0.00E+00	0.00E+00
SCE1572_6020	sce_4973	3.00E-125	4.00E-125
SCE1572_6021	sce_4974	0.00E+00	0.00E+00

SCE1572_6023	sce_4976	0.00E+00	0.00E+00
SCE1572_6024	sce_4977	0.00E+00	0.00E+00
SCE1572_6025	sce_4978	1.00E-39	8.00E-57
SCE1572_6026	sce_4979	3.00E-121	4.00E-120
SCE1572_6027	sce_4980	2.00E-42	6.00E-51
SCE1572_6028	sce_4981	3.00E-73	2.00E-70
SCE1572_6029	sce_4982	0.00E+00	0.00E+00
SCE1572_603	sce_411	2.00E-117	5.00E-114
SCE1572_6030	sce_4983	1.00E-144	1.00E-136
SCE1572_6031	sce_4984	0.00E+00	0.00E+00
SCE1572_6032	sce_4985	3.00E-23	3.00E-23
SCE1572_6033	sce_4986	0.00E+00	0.00E+00
SCE1572_6034	sce_4987	0.00E+00	0.00E+00
SCE1572_6036	sce_4988	1.00E-14	9.00E-11
SCE1572_6037	sce_4989	9.00E-22	5.00E-14
SCE1572_6038	sce_4990	3.00E-146	4.00E-162
SCE1572_6039	sce_4991	8.00E-150	9.00E-161
SCE1572_604	sce_413	0.00E+00	0.00E+00
SCE1572_6040	sce_4992	0.00E+00	0.00E+00
SCE1572_6041	sce_4993	0.00E+00	0.00E+00
SCE1572_6042	sce_4994	4.00E-118	7.00E-117
SCE1572_6044	sce_4995	1.00E-42	2.00E-35
SCE1572_6045	sce_4996	2.00E-177	2.00E-178
SCE1572_6046	sce_4997	0.00E+00	0.00E+00
SCE1572_6047	sce_4999	3.00E-38	3.00E-39
SCE1572_6048	sce_5000	1.00E-109	5.00E-89
SCE1572_6049	sce_5001	1.00E-82	3.00E-70
SCE1572_605	sce_414	1.00E-69	8.00E-70
SCE1572_6051	sce_5002	2.00E-173	5.00E-174
SCE1572_6052	sce_5003	0.00E+00	0.00E+00
SCE1572_6053	sce_5004	2.00E-136	2.00E-144
SCE1572_6054	sce_5005	0.00E+00	0.00E+00
SCE1572_6055	sce_5006	0.00E+00	0.00E+00
SCE1572_6056	sce_5007	0.00E+00	0.00E+00
SCE1572_6057	sce_5008	2.00E-33	2.00E-25
SCE1572_6058	sce_5009	8.00E-106	5.00E-128
SCE1572_6059	sce_4871	3.00E-81	1.00E-89
SCE1572_606	sce_415	0.00E+00	0.00E+00
SCE1572_6061	sce_5011	4.00E-94	1.00E-92
SCE1572_6062	sce_5012	0.00E+00	0.00E+00
SCE1572_6064	sce_5013	2.00E-84	3.00E-91
SCE1572_6065	sce_5014	8.00E-17	4.00E-14
SCE1572_6066	sce_5015	4.00E-86	4.00E-70
SCE1572_6067	sce_5016	0.00E+00	0.00E+00
SCE1572_6068	sce_5017	5.00E-169	8.00E-174
SCE1572_6069	sce_5018	7.00E-81	1.00E-82
SCE1572_607	sce_416	1.00E-20	1.00E-20
SCE1572_6070	sce_5019	1.00E-61	7.00E-73
SCE1572_6074	sce_5020	0.00E+00	0.00E+00
SCE1572_6075	sce_5022	0.00E+00	0.00E+00
SCE1572_6076	sce_5023	7.00E-15	7.00E-15
SCE1572_6077	sce_5024	8.00E-35	8.00E-35
SCE1572_608	sce_418	0.00E+00	0.00E+00
SCE1572_6082	sce_244	6.00E-53	5.00E-52
SCE1572_6083	sce_3006	2.00E-126	3.00E-144
SCE1572_6086	sce_1876	7.00E-13	8.00E-13
SCE1572_6087	sce_5033	0.00E+00	0.00E+00
SCE1572_6087	sce_5602	0.00E+00	0.00E+00
SCE1572_6087	sce_3185	0.00E+00	0.00E+00
SCE1572_6088	sce_5035	3.00E-127	2.00E-118
SCE1572_6089	sce_5036	3.00E-39	8.00E-39
SCE1572_6090	sce_5037	2.00E-71	1.00E-66
SCE1572_6091	sce_5039	1.00E-32	1.00E-16
SCE1572_6094	sce_835	2.00E-128	7.00E-126
SCE1572_6096	sce_9920	0.00E+00	0.00E+00
SCE1572_6096	sce_7640	0.00E+00	0.00E+00
SCE1572_6097	sce_5042	1.00E-86	5.00E-77
SCE1572_6098	sce_5043	8.00E-140	5.00E-146
SCE1572_6099	sce_5045	1.00E-175	6.00E-174
SCE1572_61	sce_51	0.00E+00	0.00E+00
SCE1572_6100	sce_5047	0.00E+00	0.00E+00
SCE1572_6100	sce_8489	0.00E+00	0.00E+00
SCE1572_6101	sce_5048	0.00E+00	0.00E+00
SCE1572_6102	sce_5049	3.00E-83	3.00E-83
SCE1572_6103	sce_5050	9.00E-86	3.00E-83
SCE1572_6104	sce_5051	7.00E-153	6.00E-161

SCE1572_6105	sce_5052	1.00E-42	6.00E-43
SCE1572_6106	sce_5053	2.00E-101	1.00E-93
SCE1572_6107	sce_5054	7.00E-169	3.00E-159
SCE1572_6108	sce_5055	0.00E+00	0.00E+00
SCE1572_6109	sce_5056	3.00E-82	5.00E-81
SCE1572_611	sce_420	6.00E-99	1.00E-107
SCE1572_6110	sce_5057	3.00E-23	2.00E-22
SCE1572_6111	sce_5058	6.00E-94	6.00E-99
SCE1572_6112	sce_5059	4.00E-42	3.00E-63
SCE1572_6113	sce_5060	8.00E-107	2.00E-111
SCE1572_6114	sce_6885	1.00E-30	1.00E-28
SCE1572_6116	sce_2459	2.00E-97	6.00E-96
SCE1572_6118	sce_6665	7.00E-08	7.00E-08
SCE1572_6119	sce_5062	2.00E-112	2.00E-112
SCE1572_612	sce_421	1.00E-94	9.00E-95
SCE1572_6120	sce_5063	1.00E-81	2.00E-77
SCE1572_6121	sce_5064	2.00E-91	2.00E-130
SCE1572_6122	sce_5066	0.00E+00	0.00E+00
SCE1572_6123	sce_5068	0.00E+00	0.00E+00
SCE1572_6123	sce_5637	0.00E+00	0.00E+00
SCE1572_6124	sce_5069	3.00E-84	3.00E-84
SCE1572_6125	sce_5070	4.00E-133	4.00E-120
SCE1572_6126	sce_5071	1.00E-121	2.00E-118
SCE1572_6127	sce_5072	2.00E-172	0.00E+00
SCE1572_6128	sce_5073	0.00E+00	0.00E+00
SCE1572_6129	sce_5074	0.00E+00	0.00E+00
SCE1572_613	sce_422	4.00E-112	2.00E-103
SCE1572_6131	sce_5076	8.00E-44	8.00E-44
SCE1572_6133	sce_5077	0.00E+00	0.00E+00
SCE1572_6134	sce_5078	4.00E-42	2.00E-41
SCE1572_6135	sce_5079	0.00E+00	0.00E+00
SCE1572_6136	sce_5080	0.00E+00	0.00E+00
SCE1572_6137	sce_5081	2.00E-71	3.00E-72
SCE1572_6138	sce_7381	1.00E-120	1.00E-120
SCE1572_6139	sce_5082	0.00E+00	0.00E+00
SCE1572_614	sce_423	5.00E-78	8.00E-79
SCE1572_6140	sce_5083	0.00E+00	0.00E+00
SCE1572_6142	sce_5084	1.00E-120	9.00E-129
SCE1572_6145	sce_5088	7.00E-103	2.00E-116
SCE1572_6146	sce_1290	1.00E-37	3.00E-30
SCE1572_6151	sce_5091	0.00E+00	0.00E+00
SCE1572_6154	sce_5638	3.00E-69	4.00E-69
SCE1572_6155	sce_5094	2.00E-135	1.00E-136
SCE1572_6156	sce_5123	1.00E-75	2.00E-83
SCE1572_6158	sce_5128	8.00E-149	3.00E-137
SCE1572_6159	sce_5129	1.00E-169	2.00E-126
SCE1572_616	sce_424	0.00E+00	0.00E+00
SCE1572_6161	sce_5130	0.00E+00	0.00E+00
SCE1572_6162	sce_5131	0.00E+00	0.00E+00
SCE1572_6164	sce_5160	3.00E-100	2.00E-122
SCE1572_6165	sce_5161	0.00E+00	0.00E+00
SCE1572_6166	sce_5162	0.00E+00	3.00E-177
SCE1572_6167	sce_5163	1.00E-46	4.00E-51
SCE1572_6168	sce_5164	2.00E-161	3.00E-175
SCE1572_617	sce_425	8.00E-100	8.00E-100
SCE1572_6170	sce_5165	2.00E-76	2.00E-76
SCE1572_6171	sce_5166	0.00E+00	0.00E+00
SCE1572_6173	sce_335	4.00E-71	2.00E-64
SCE1572_6174	sce_336	7.00E-109	6.00E-115
SCE1572_6175	sce_5250	1.00E-138	8.00E-139
SCE1572_6176	sce_5171	2.00E-115	4.00E-124
SCE1572_6178	sce_5176	3.00E-102	7.00E-96
SCE1572_6179	sce_5177	5.00E-83	3.00E-101
SCE1572_618	sce_426	3.00E-116	8.00E-119
SCE1572_6180	sce_5178	0.00E+00	0.00E+00
SCE1572_6181	sce_5179	1.00E-101	1.00E-101
SCE1572_6184	sce_5320	5.00E-09	8.00E-10
SCE1572_6185	sce_5195	0.00E+00	0.00E+00
SCE1572_6186	sce_5196	2.00E-06	1.00E-18
SCE1572_6187	sce_5197	2.00E-67	3.00E-81
SCE1572_6188	sce_5199	2.00E-110	6.00E-109
SCE1572_619	sce_427	5.00E-89	4.00E-44
SCE1572_6191	sce_5200	7.00E-79	9.00E-83
SCE1572_6192	sce_5206	6.00E-77	9.00E-83
SCE1572_6195	sce_5207	6.00E-57	1.00E-54
SCE1572_6196	sce_5208	2.00E-35	3.00E-25

SCE1572_6197	sce_5209	7.00E-14	8.00E-18
SCE1572_6198	sce_5211	1.00E-116	2.00E-122
SCE1572_620	sce_428	0.00E+00	0.00E+00
SCE1572_6200	sce_5224	4.00E-144	2.00E-154
SCE1572_6201	sce_5225	0.00E+00	0.00E+00
SCE1572_6202	sce_5226	0.00E+00	0.00E+00
SCE1572_6202	sce_5403	0.00E+00	0.00E+00
SCE1572_6202	sce_1803	0.00E+00	0.00E+00
SCE1572_6202	sce_7969	0.00E+00	0.00E+00
SCE1572_6202	sce_7154	0.00E+00	0.00E+00
SCE1572_6202	sce_9665	0.00E+00	0.00E+00
SCE1572_6202	sce_5751	0.00E+00	0.00E+00
SCE1572_6202	sce_4073	0.00E+00	0.00E+00
SCE1572_6202	sce_5746	0.00E+00	0.00E+00
SCE1572_6202	sce_2053	0.00E+00	0.00E+00
SCE1572_6202	sce_9757	0.00E+00	0.00E+00
SCE1572_6202	sce_9954	0.00E+00	0.00E+00
SCE1572_6202	sce_3750	0.00E+00	0.00E+00
SCE1572_6202	sce_6712	0.00E+00	0.00E+00
SCE1572_6202	sce_5229	0.00E+00	0.00E+00
SCE1572_6203	sce_5228	4.00E-144	3.00E-135
SCE1572_6205	sce_2524	0.00E+00	0.00E+00
SCE1572_6206	sce_2525	0.00E+00	0.00E+00
SCE1572_621	sce_430	0.00E+00	0.00E+00
SCE1572_6210	sce_8592	0.00E+00	0.00E+00
SCE1572_6210	sce_3937	0.00E+00	0.00E+00
SCE1572_6213	sce_10331	4.00E-83	8.00E-81
SCE1572_6215	sce_5242	2.00E-135	1.00E-134
SCE1572_6217	sce_9321	2.00E-16	6.00E-18
SCE1572_6222	sce_2729	4.00E-51	5.00E-58
SCE1572_6224	sce_5247	1.00E-114	3.00E-121
SCE1572_6225	sce_5248	3.00E-82	1.00E-91
SCE1572_6227	sce_8772	4.00E-33	4.00E-42
SCE1572_623	sce_431	0.00E+00	0.00E+00
SCE1572_6231	sce_3674	3.00E-126	1.00E-128
SCE1572_6232	sce_3675	3.00E-165	2.00E-163
SCE1572_6234	sce_8040	8.00E-10	9.00E-11
SCE1572_6235	sce_10194	4.00E-48	1.00E-42
SCE1572_6236	sce_2008	2.00E-115	1.00E-115
SCE1572_6238	sce_5252	9.00E-122	1.00E-140
SCE1572_6241	sce_5253	2.00E-57	6.00E-58
SCE1572_6242	sce_5254	0.00E+00	8.00E-173
SCE1572_6246	sce_7862	5.00E-167	5.00E-165
SCE1572_6247	sce_614	9.00E-120	1.00E-109
SCE1572_6248	sce_7861	4.00E-56	4.00E-56
SCE1572_6252	sce_5264	0.00E+00	0.00E+00
SCE1572_6253	sce_5265	2.00E-97	1.00E-90
SCE1572_6254	sce_5266	0.00E+00	0.00E+00
SCE1572_6259	sce_5270	5.00E-47	2.00E-46
SCE1572_6260	sce_5271	9.00E-132	1.00E-132
SCE1572_6261	sce_5272	1.00E-58	1.00E-63
SCE1572_6262	sce_5273	0.00E+00	0.00E+00
SCE1572_6263	sce_5274	3.00E-179	0.00E+00
SCE1572_6264	sce_5275	3.00E-118	5.00E-121
SCE1572_6265	sce_5282	1.00E-75	1.00E-75
SCE1572_6266	sce_5284	2.00E-07	2.00E-06
SCE1572_6269	sce_5285	2.00E-56	4.00E-57
SCE1572_627	sce_10425	0.00E+00	0.00E+00
SCE1572_627	sce_7361	0.00E+00	0.00E+00
SCE1572_627	sce_3520	0.00E+00	0.00E+00
SCE1572_6270	sce_5287	3.00E-109	3.00E-109
SCE1572_6272	sce_5289	7.00E-169	2.00E-173
SCE1572_6273	sce_3740	0.00E+00	0.00E+00
SCE1572_6274	sce_3739	3.00E-71	3.00E-71
SCE1572_6275	sce_3738	0.00E+00	0.00E+00
SCE1572_6276	sce_3737	8.00E-96	6.00E-90
SCE1572_6278	sce_3669	2.00E-53	5.00E-60
SCE1572_6279	sce_5294	1.00E-43	6.00E-47
SCE1572_628	sce_436	2.00E-66	8.00E-74
SCE1572_6280	sce_5297	2.00E-66	1.00E-66
SCE1572_6281	sce_1108	0.00E+00	0.00E+00
SCE1572_6281	sce_9028	0.00E+00	0.00E+00
SCE1572_6283	sce_5305	7.00E-161	3.00E-174
SCE1572_6284	sce_5310	3.00E-108	3.00E-108
SCE1572_6285	sce_5311	1.00E-115	1.00E-115
SCE1572_6286	sce_5312	3.00E-81	1.00E-81

SCE1572_6287	sce_5313	1.00E-93	1.00E-99
SCE1572_6289	sce_5318	8.00E-84	1.00E-78
SCE1572_629	sce_437	0.00E+00	0.00E+00
SCE1572_6291	sce_5319	3.00E-73	1.00E-102
SCE1572_6292	sce_5321	9.00E-127	1.00E-131
SCE1572_6293	sce_5150	3.00E-148	5.00E-148
SCE1572_6296	sce_5322	4.00E-73	8.00E-69
SCE1572_6298	sce_5323	2.00E-123	4.00E-128
SCE1572_63	sce_54	0.00E+00	0.00E+00
SCE1572_630	sce_438	1.00E-134	1.00E-134
SCE1572_6300	sce_5324	2.00E-78	2.00E-80
SCE1572_6301	sce_7403	2.00E-43	1.00E-42
SCE1572_6302	sce_5329	0.00E+00	0.00E+00
SCE1572_6304	sce_5331	3.00E-39	3.00E-39
SCE1572_6305	sce_5332	6.00E-99	9.00E-100
SCE1572_6306	sce_5333	1.00E-116	1.00E-118
SCE1572_6307	sce_5334	5.00E-158	1.00E-163
SCE1572_6308	sce_5335	3.00E-133	3.00E-141
SCE1572_6309	sce_5336	7.00E-135	7.00E-135
SCE1572_631	sce_439	0.00E+00	0.00E+00
SCE1572_6310	sce_5337	3.00E-144	4.00E-145
SCE1572_6311	sce_5338	7.00E-141	7.00E-141
SCE1572_6313	sce_2034	2.00E-101	5.00E-105
SCE1572_6318	sce_5340	3.00E-62	8.00E-83
SCE1572_6319	sce_5341	3.00E-76	1.00E-74
SCE1572_632	sce_440	3.00E-162	2.00E-162
SCE1572_6321	sce_5342	3.00E-90	7.00E-94
SCE1572_6322	sce_5343	6.00E-65	1.00E-64
SCE1572_6323	sce_5344	0.00E+00	0.00E+00
SCE1572_6324	sce_5345	2.00E-147	2.00E-156
SCE1572_6325	sce_5346	1.00E-57	6.00E-43
SCE1572_6326	sce_5348	5.00E-51	5.00E-51
SCE1572_6327	sce_5349	1.00E-32	3.00E-33
SCE1572_6328	sce_5350	2.00E-113	2.00E-113
SCE1572_6329	sce_5351	0.00E+00	0.00E+00
SCE1572_633	sce_441	2.00E-135	8.00E-140
SCE1572_6330	sce_5352	0.00E+00	0.00E+00
SCE1572_6331	sce_5353	1.00E-64	1.00E-64
SCE1572_6332	sce_5354	7.00E-130	9.00E-140
SCE1572_6333	sce_5355	2.00E-142	3.00E-142
SCE1572_6334	sce_5357	2.00E-154	2.00E-164
SCE1572_6335	sce_5358	0.00E+00	0.00E+00
SCE1572_6335	sce_9776	0.00E+00	0.00E+00
SCE1572_6336	sce_5359	0.00E+00	0.00E+00
SCE1572_6336	sce_9777	0.00E+00	0.00E+00
SCE1572_6337	sce_5360	4.00E-96	4.00E-96
SCE1572_6338	sce_5361	2.00E-72	8.00E-64
SCE1572_6339	sce_5373	1.00E-147	1.00E-147
SCE1572_634	sce_442	0.00E+00	0.00E+00
SCE1572_6340	sce_5374	6.00E-46	3.00E-79
SCE1572_6341	sce_5375	6.00E-141	6.00E-141
SCE1572_6342	sce_5377	1.00E-172	0.00E+00
SCE1572_6343	sce_5378	0.00E+00	0.00E+00
SCE1572_6344	sce_5379	0.00E+00	0.00E+00
SCE1572_6345	sce_5380	2.00E-45	2.00E-50
SCE1572_6346	sce_5382	1.00E-150	1.00E-153
SCE1572_6347	sce_5383	3.00E-85	3.00E-85
SCE1572_635	sce_443	5.00E-95	5.00E-83
SCE1572_6351	sce_5387	3.00E-146	3.00E-139
SCE1572_6352	sce_5388	1.00E-63	9.00E-58
SCE1572_6353	sce_5389	7.00E-124	7.00E-124
SCE1572_6354	sce_5506	1.00E-56	2.00E-52
SCE1572_6356	sce_6038	1.00E-173	2.00E-170
SCE1572_6357	sce_5390	6.00E-158	6.00E-177
SCE1572_6359	sce_5391	7.00E-80	4.00E-69
SCE1572_636	sce_444	1.00E-97	1.00E-104
SCE1572_6360	sce_5392	3.00E-56	8.00E-50
SCE1572_6363	sce_5393	0.00E+00	0.00E+00
SCE1572_6364	sce_5394	5.00E-110	2.00E-126
SCE1572_6365	sce_5395	1.00E-168	6.00E-171
SCE1572_6366	sce_5396	2.00E-124	5.00E-74
SCE1572_6367	sce_5397	3.00E-79	7.00E-74
SCE1572_6368	sce_5398	0.00E+00	0.00E+00
SCE1572_637	sce_446	4.00E-90	4.00E-90
SCE1572_6370	sce_5400	9.00E-42	2.00E-45
SCE1572_6377	sce_5401	4.00E-95	7.00E-99

SCE1572_6378	sce_7567	3.00E-96	2.00E-96
SCE1572_638	sce_447	4.00E-108	1.00E-108
SCE1572_6381	sce_5402	4.00E-46	4.00E-46
SCE1572_6382	sce_5403	0.00E+00	0.00E+00
SCE1572_6382	sce_5226	0.00E+00	0.00E+00
SCE1572_6382	sce_7969	0.00E+00	0.00E+00
SCE1572_6382	sce_7154	0.00E+00	0.00E+00
SCE1572_6382	sce_5751	0.00E+00	0.00E+00
SCE1572_6382	sce_4073	0.00E+00	0.00E+00
SCE1572_6382	sce_9665	0.00E+00	0.00E+00
SCE1572_6382	sce_5746	0.00E+00	0.00E+00
SCE1572_6382	sce_2053	0.00E+00	0.00E+00
SCE1572_6382	sce_9954	0.00E+00	0.00E+00
SCE1572_6382	sce_1803	0.00E+00	0.00E+00
SCE1572_6382	sce_6712	0.00E+00	0.00E+00
SCE1572_6382	sce_3750	0.00E+00	0.00E+00
SCE1572_6382	sce_5229	0.00E+00	0.00E+00
SCE1572_6383	sce_5404	8.00E-12	8.00E-12
SCE1572_6384	sce_5405	7.00E-142	4.00E-140
SCE1572_6386	sce_5406	0.00E+00	0.00E+00
SCE1572_6387	sce_5407	0.00E+00	0.00E+00
SCE1572_6388	sce_5408	0.00E+00	0.00E+00
SCE1572_6389	sce_5409	0.00E+00	0.00E+00
SCE1572_639	sce_448	0.00E+00	0.00E+00
SCE1572_6390	sce_5410	1.00E-139	1.00E-139
SCE1572_6392	sce_5412	1.00E-110	1.00E-110
SCE1572_6393	sce_5413	5.00E-129	5.00E-129
SCE1572_6394	sce_5414	3.00E-58	6.00E-58
SCE1572_6396	sce_5415	0.00E+00	0.00E+00
SCE1572_6397	sce_5416	0.00E+00	0.00E+00
SCE1572_6398	sce_5417	3.00E-124	1.00E-130
SCE1572_6399	sce_5418	0.00E+00	0.00E+00
SCE1572_64	sce_55	1.00E-110	4.00E-103
SCE1572_640	sce_449	5.00E-50	8.00E-79
SCE1572_6400	sce_5419	0.00E+00	0.00E+00
SCE1572_6401	sce_5420	9.00E-07	9.00E-07
SCE1572_6402	sce_5422	0.00E+00	0.00E+00
SCE1572_6403	sce_5423	1.00E-123	7.00E-120
SCE1572_6404	sce_5424	2.00E-13	1.00E-27
SCE1572_6405	sce_5425	1.00E-98	4.00E-101
SCE1572_6406	sce_5426	2.00E-56	5.00E-75
SCE1572_6407	sce_5427	5.00E-140	7.00E-146
SCE1572_6408	sce_5428	4.00E-142	3.00E-134
SCE1572_6409	sce_5429	2.00E-13	3.00E-12
SCE1572_641	sce_450	3.00E-116	2.00E-112
SCE1572_6412	sce_5430	7.00E-103	8.00E-103
SCE1572_6413	sce_5431	1.00E-83	2.00E-83
SCE1572_6415	sce_5433	5.00E-128	2.00E-128
SCE1572_6416	sce_5434	3.00E-68	4.00E-51
SCE1572_6418	sce_5435	0.00E+00	0.00E+00
SCE1572_6419	sce_4723	3.00E-11	4.00E-18
SCE1572_642	sce_451	0.00E+00	0.00E+00
SCE1572_6420	sce_4725	0.00E+00	0.00E+00
SCE1572_6420	sce_5308	0.00E+00	0.00E+00
SCE1572_6422	sce_7652	1.00E-32	4.00E-24
SCE1572_6423	sce_3067	2.00E-134	2.00E-138
SCE1572_6425	sce_1172	5.00E-85	7.00E-76
SCE1572_6426	sce_1171	1.00E-47	7.00E-43
SCE1572_6428	sce_7206	1.00E-129	4.00E-145
SCE1572_6429	sce_2031	1.00E-144	8.00E-141
SCE1572_643	sce_452	4.00E-111	1.00E-98
SCE1572_6430	sce_6181	0.00E+00	0.00E+00
SCE1572_6431	sce_7564	8.00E-76	2.00E-73
SCE1572_6432	sce_2123	4.00E-75	3.00E-84
SCE1572_6433	sce_2124	2.00E-59	6.00E-58
SCE1572_6436	sce_3639	1.00E-109	9.00E-112
SCE1572_6437	sce_3640	8.00E-97	8.00E-97
SCE1572_644	sce_453	7.00E-56	2.00E-65
SCE1572_6445	sce_5449	5.00E-101	7.00E-106
SCE1572_6451	sce_8592	0.00E+00	0.00E+00
SCE1572_6451	sce_3937	0.00E+00	0.00E+00
SCE1572_6452	sce_3937	0.00E+00	0.00E+00
SCE1572_6452	sce_8592	0.00E+00	0.00E+00
SCE1572_6457	sce_5452	0.00E+00	0.00E+00
SCE1572_6464	sce_5437	7.00E-43	5.00E-40
SCE1572_6465	sce_5438	6.00E-93	3.00E-97

SCE1572_6466	sce_5439	7.00E-108	1.00E-107
SCE1572_6467	sce_5440	6.00E-150	6.00E-141
SCE1572_6468	sce_5441	0.00E+00	0.00E+00
SCE1572_6469	sce_5442	1.00E-174	7.00E-179
SCE1572_6470	sce_5443	2.00E-140	2.00E-140
SCE1572_6471	sce_5444	0.00E+00	2.00E-178
SCE1572_6472	sce_5445	2.00E-127	3.00E-127
SCE1572_6478	sce_2488	1.00E-61	7.00E-62
SCE1572_648	sce_455	0.00E+00	0.00E+00
SCE1572_6482	sce_5451	5.00E-170	2.00E-161
SCE1572_6484	sce_5454	5.00E-164	0.00E+00
SCE1572_6485	sce_5455	7.00E-93	5.00E-94
SCE1572_6486	sce_5456	4.00E-46	5.00E-43
SCE1572_6487	sce_5457	4.00E-59	9.00E-43
SCE1572_6488	sce_5458	2.00E-16	5.00E-13
SCE1572_6490	sce_2076	1.00E-67	2.00E-81
SCE1572_6492	sce_2078	1.00E-103	9.00E-124
SCE1572_6493	sce_2079	1.00E-101	2.00E-107
SCE1572_6498	sce_5461	0.00E+00	0.00E+00
SCE1572_6499	sce_9818	5.00E-08	4.00E-10
SCE1572_65	sce_56	2.00E-106	2.00E-102
SCE1572_650	sce_945	0.00E+00	0.00E+00
SCE1572_650	sce_457	0.00E+00	0.00E+00
SCE1572_6509	sce_3727	4.00E-59	2.00E-60
SCE1572_651	sce_458	3.00E-26	3.00E-26
SCE1572_6511	sce_9988	1.00E-85	4.00E-84
SCE1572_6512	sce_5465	0.00E+00	0.00E+00
SCE1572_6514	sce_5467	0.00E+00	0.00E+00
SCE1572_6515	sce_5468	4.00E-53	2.00E-74
SCE1572_6516	sce_5469	0.00E+00	0.00E+00
SCE1572_6517	sce_5471	3.00E-46	2.00E-62
SCE1572_652	sce_459	1.00E-55	2.00E-39
SCE1572_6520	sce_5493	2.00E-125	8.00E-100
SCE1572_6521	sce_5494	9.00E-28	9.00E-28
SCE1572_6524	sce_5495	7.00E-142	1.00E-141
SCE1572_6525	sce_1706	7.00E-67	2.00E-58
SCE1572_653	sce_460	6.00E-99	6.00E-99
SCE1572_6530	sce_9960	0.00E+00	0.00E+00
SCE1572_6531	sce_9961	0.00E+00	0.00E+00
SCE1572_6535	sce_9435	3.00E-73	2.00E-81
SCE1572_6541	sce_5497	1.00E-121	7.00E-118
SCE1572_6542	sce_5498	2.00E-141	2.00E-149
SCE1572_6543	sce_5499	1.00E-118	1.00E-118
SCE1572_6544	sce_5500	0.00E+00	0.00E+00
SCE1572_6545	sce_5501	2.00E-171	2.00E-143
SCE1572_6547	sce_9557	3.00E-169	0.00E+00
SCE1572_655	sce_461	2.00E-80	2.00E-80
SCE1572_6551	sce_5511	4.00E-170	0.00E+00
SCE1572_6556	sce_7099	1.00E-61	7.00E-66
SCE1572_6557	sce_5514	1.00E-134	1.00E-120
SCE1572_6559	sce_6328	4.00E-70	1.00E-69
SCE1572_656	sce_462	9.00E-111	2.00E-111
SCE1572_657	sce_463	1.00E-94	6.00E-85
SCE1572_6570	sce_6017	2.00E-64	4.00E-50
SCE1572_6572	sce_9878	2.00E-86	2.00E-86
SCE1572_6574	sce_8429	0.00E+00	0.00E+00
SCE1572_6575	sce_5136	3.00E-22	8.00E-23
SCE1572_6576	sce_9599	2.00E-108	2.00E-108
SCE1572_6577	sce_9598	5.00E-142	2.00E-141
SCE1572_6578	sce_1328	7.00E-162	3.00E-164
SCE1572_6579	sce_9849	9.00E-64	3.00E-65
SCE1572_658	sce_464	4.00E-47	5.00E-47
SCE1572_6581	sce_6235	0.00E+00	0.00E+00
SCE1572_6581	sce_2161	0.00E+00	0.00E+00
SCE1572_6581	sce_225	0.00E+00	0.00E+00
SCE1572_6581	sce_8357	0.00E+00	0.00E+00
SCE1572_6581	sce_5276	0.00E+00	0.00E+00
SCE1572_6582	sce_5517	1.00E-111	2.00E-120
SCE1572_6585	sce_7661	0.00E+00	0.00E+00
SCE1572_6585	sce_6093	0.00E+00	0.00E+00
SCE1572_6585	sce_6092	0.00E+00	0.00E+00
SCE1572_6585	sce_6095	0.00E+00	0.00E+00
SCE1572_6585	sce_3917	0.00E+00	0.00E+00
SCE1572_6585	sce_6107	0.00E+00	0.00E+00
SCE1572_6585	sce_6094	0.00E+00	0.00E+00
SCE1572_6585	sce_10204	0.00E+00	0.00E+00

SCE1572_6585	sce_9920	0.00E+00	0.00E+00
SCE1572_6585	sce_6535	0.00E+00	0.00E+00
SCE1572_6585	sce_388	0.00E+00	0.00E+00
SCE1572_6585	sce_3003	0.00E+00	0.00E+00
SCE1572_6585	sce_7640	0.00E+00	0.00E+00
SCE1572_6585	sce_9557	0.00E+00	0.00E+00
SCE1572_6585	sce_5835	0.00E+00	0.00E+00
SCE1572_6585	sce_4513	0.00E+00	0.00E+00
SCE1572_6585	sce_8618	0.00E+00	0.00E+00
SCE1572_6587	sce_9236	0.00E+00	0.00E+00
SCE1572_6587	sce_389	0.00E+00	0.00E+00
SCE1572_6588	sce_389	0.00E+00	0.00E+00
SCE1572_6588	sce_7637	0.00E+00	0.00E+00
SCE1572_6588	sce_2666	0.00E+00	0.00E+00
SCE1572_6588	sce_9237	0.00E+00	0.00E+00
SCE1572_659	sce_465	4.00E-24	4.00E-24
SCE1572_6596	sce_5518	4.00E-23	2.00E-38
SCE1572_6597	sce_5519	4.00E-52	2.00E-56
SCE1572_6598	sce_5520	2.00E-107	5.00E-107
SCE1572_6599	sce_5522	0.00E+00	0.00E+00
SCE1572_66	sce_58	0.00E+00	0.00E+00
SCE1572_660	sce_466	0.00E+00	0.00E+00
SCE1572_6600	sce_5523	0.00E+00	0.00E+00
SCE1572_6601	sce_5524	0.00E+00	0.00E+00
SCE1572_6601	sce_9469	0.00E+00	0.00E+00
SCE1572_6602	sce_5526	3.00E-72	3.00E-97
SCE1572_6603	sce_5527	3.00E-121	3.00E-126
SCE1572_6605	sce_2518	4.00E-26	5.00E-23
SCE1572_6609	sce_8361	3.00E-09	8.00E-10
SCE1572_661	sce_467	3.00E-09	4.00E-15
SCE1572_6610	sce_803	6.00E-37	1.00E-47
SCE1572_6614	sce_5529	3.00E-152	1.00E-148
SCE1572_6616	sce_5530	0.00E+00	0.00E+00
SCE1572_6617	sce_7059	2.00E-47	9.00E-53
SCE1572_6619	sce_7066	2.00E-19	2.00E-29
SCE1572_662	sce_468	0.00E+00	0.00E+00
SCE1572_6624	sce_169	2.00E-22	1.00E-24
SCE1572_6625	sce_9401	3.00E-41	2.00E-43
SCE1572_6629	sce_3602	8.00E-63	6.00E-61
SCE1572_6630	sce_3603	9.00E-63	1.00E-63
SCE1572_6634	sce_9373	0.00E+00	0.00E+00
SCE1572_6634	sce_4062	0.00E+00	0.00E+00
SCE1572_6637	sce_5710	0.00E+00	0.00E+00
SCE1572_664	sce_469	0.00E+00	0.00E+00
SCE1572_6640	sce_5538	0.00E+00	0.00E+00
SCE1572_6641	sce_5539	6.00E-122	2.00E-116
SCE1572_6642	sce_5540	4.00E-73	1.00E-77
SCE1572_6643	sce_5541	2.00E-177	1.00E-177
SCE1572_6645	sce_1705	3.00E-86	6.00E-98
SCE1572_6646	sce_2536	7.00E-93	8.00E-93
SCE1572_665	sce_470	1.00E-78	3.00E-93
SCE1572_6650	sce_1704	2.00E-133	2.00E-134
SCE1572_6652	sce_7114	3.00E-48	8.00E-49
SCE1572_6654	sce_5546	0.00E+00	0.00E+00
SCE1572_6654	sce_10036	0.00E+00	0.00E+00
SCE1572_6655	sce_5551	1.00E-20	2.00E-44
SCE1572_6657	sce_3612	0.00E+00	0.00E+00
SCE1572_666	sce_471	8.00E-147	4.00E-150
SCE1572_6663	sce_5556	2.00E-116	2.00E-101
SCE1572_6665	sce_5554	7.00E-176	1.00E-154
SCE1572_6666	sce_5555	0.00E+00	0.00E+00
SCE1572_6668	sce_9809	2.00E-154	2.00E-171
SCE1572_6669	sce_9810	3.00E-96	7.00E-97
SCE1572_667	sce_472	0.00E+00	0.00E+00
SCE1572_6670	sce_9811	1.00E-56	9.00E-43
SCE1572_6671	sce_5917	5.00E-62	3.00E-62
SCE1572_6673	sce_5558	1.00E-30	7.00E-38
SCE1572_6676	sce_5560	7.00E-88	5.00E-95
SCE1572_6677	sce_5181	2.00E-94	1.00E-82
SCE1572_6678	sce_5561	2.00E-92	8.00E-93
SCE1572_668	sce_473	1.00E-116	2.00E-112
SCE1572_6680	sce_5562	2.00E-84	5.00E-90
SCE1572_6681	sce_5563	7.00E-103	8.00E-128
SCE1572_6683	sce_5564	2.00E-166	2.00E-159
SCE1572_6684	sce_5568	2.00E-172	2.00E-179
SCE1572_6687	sce_5569	2.00E-66	2.00E-66

SCE1572_6688	sce_5086	0.00E+00	0.00E+00
SCE1572_6688	sce_5085	0.00E+00	0.00E+00
SCE1572_6688	sce_6701	0.00E+00	0.00E+00
SCE1572_6688	sce_3793	0.00E+00	0.00E+00
SCE1572_669	sce_474	0.00E+00	0.00E+00
SCE1572_6691	sce_10016	3.00E-24	3.00E-24
SCE1572_6694	sce_2414	3.00E-131	1.00E-122
SCE1572_6695	sce_5574	5.00E-162	2.00E-171
SCE1572_6696	sce_5575	3.00E-89	1.00E-100
SCE1572_6697	sce_5576	9.00E-102	9.00E-102
SCE1572_67	sce_60	1.00E-133	8.00E-122
SCE1572_670	sce_475	3.00E-160	4.00E-164
SCE1572_6700	sce_2475	7.00E-145	1.00E-152
SCE1572_6701	sce_3424	0.00E+00	0.00E+00
SCE1572_6704	sce_5577	2.00E-66	1.00E-77
SCE1572_6705	sce_5578	0.00E+00	0.00E+00
SCE1572_6705	sce_1392	0.00E+00	0.00E+00
SCE1572_6706	sce_5579	7.00E-89	7.00E-89
SCE1572_6708	sce_5580	1.00E-132	1.00E-130
SCE1572_6709	sce_5581	9.00E-180	8.00E-180
SCE1572_671	sce_476	2.00E-145	8.00E-167
SCE1572_6710	sce_5582	0.00E+00	0.00E+00
SCE1572_6710	sce_8116	0.00E+00	0.00E+00
SCE1572_6711	sce_5583	2.00E-101	2.00E-101
SCE1572_6712	sce_5584	0.00E+00	0.00E+00
SCE1572_6713	sce_5585	4.00E-94	3.00E-96
SCE1572_6714	sce_5586	0.00E+00	0.00E+00
SCE1572_6715	sce_5587	0.00E+00	0.00E+00
SCE1572_6716	sce_5588	0.00E+00	0.00E+00
SCE1572_6717	sce_5589	9.00E-105	1.00E-97
SCE1572_6719	sce_5590	3.00E-145	3.00E-140
SCE1572_672	sce_478	7.00E-53	2.00E-83
SCE1572_6720	sce_5592	8.00E-78	3.00E-69
SCE1572_6721	sce_5593	1.00E-60	3.00E-60
SCE1572_6722	sce_5594	9.00E-74	9.00E-74
SCE1572_6723	sce_5595	0.00E+00	0.00E+00
SCE1572_6724	sce_5596	9.00E-172	0.00E+00
SCE1572_6725	sce_5597	4.00E-103	2.00E-102
SCE1572_6726	sce_5598	4.00E-85	3.00E-85
SCE1572_6727	sce_5599	5.00E-82	3.00E-86
SCE1572_6728	sce_5600	8.00E-54	2.00E-38
SCE1572_6729	sce_5601	6.00E-13	5.00E-15
SCE1572_6730	sce_5602	0.00E+00	0.00E+00
SCE1572_6730	sce_3185	0.00E+00	0.00E+00
SCE1572_6730	sce_5033	0.00E+00	0.00E+00
SCE1572_6732	sce_5604	6.00E-124	2.00E-127
SCE1572_6733	sce_5605	7.00E-152	2.00E-145
SCE1572_6734	sce_5606	3.00E-114	1.00E-102
SCE1572_6735	sce_5607	0.00E+00	0.00E+00
SCE1572_6736	sce_5608	1.00E-153	1.00E-153
SCE1572_6737	sce_5609	4.00E-84	1.00E-83
SCE1572_6738	sce_5610	4.00E-96	8.00E-112
SCE1572_6740	sce_1003	0.00E+00	0.00E+00
SCE1572_6742	sce_5614	4.00E-130	2.00E-140
SCE1572_6743	sce_5615	5.00E-12	3.00E-14
SCE1572_6744	sce_5616	5.00E-80	3.00E-66
SCE1572_6750	sce_5627	4.00E-169	3.00E-178
SCE1572_6755	sce_10428	4.00E-43	9.00E-43
SCE1572_6756	sce_10429	4.00E-54	9.00E-54
SCE1572_6757	sce_10430	5.00E-116	5.00E-116
SCE1572_6759	sce_5628	2.00E-54	3.00E-62
SCE1572_676	sce_480	4.00E-78	8.00E-81
SCE1572_6760	sce_1338	0.00E+00	0.00E+00
SCE1572_6760	sce_9335	0.00E+00	0.00E+00
SCE1572_6764	sce_5629	0.00E+00	0.00E+00
SCE1572_6768	sce_5447	7.00E-20	2.00E-14
SCE1572_6769	sce_5632	0.00E+00	0.00E+00
SCE1572_677	sce_481	0.00E+00	0.00E+00
SCE1572_6770	sce_5634	2.00E-06	1.00E-06
SCE1572_6771	sce_5635	4.00E-23	3.00E-08
SCE1572_6773	sce_5637	0.00E+00	0.00E+00
SCE1572_6776	sce_5639	0.00E+00	0.00E+00
SCE1572_6776	sce_9625	0.00E+00	0.00E+00
SCE1572_6776	sce_5292	0.00E+00	0.00E+00
SCE1572_6777	sce_5640	9.00E-10	8.00E-10
SCE1572_6778	sce_5641	2.00E-90	6.00E-100

SCE1572_6779	sce_5642	7.00E-87	1.00E-87
SCE1572_678	sce_482	0.00E+00	0.00E+00
SCE1572_6781	sce_5643	3.00E-50	1.00E-41
SCE1572_6782	sce_5644	1.00E-42	3.00E-40
SCE1572_6783	sce_5645	1.00E-73	1.00E-73
SCE1572_6787	sce_5646	4.00E-111	3.00E-113
SCE1572_6788	sce_5647	9.00E-129	2.00E-126
SCE1572_6789	sce_5648	0.00E+00	0.00E+00
SCE1572_679	sce_483	8.00E-72	8.00E-72
SCE1572_6792	sce_5650	0.00E+00	0.00E+00
SCE1572_6793	sce_5652	0.00E+00	0.00E+00
SCE1572_6794	sce_5653	1.00E-113	4.00E-117
SCE1572_6795	sce_5654	0.00E+00	0.00E+00
SCE1572_6796	sce_5655	1.00E-35	2.00E-22
SCE1572_6797	sce_5656	1.00E-169	1.00E-169
SCE1572_6798	sce_5657	0.00E+00	0.00E+00
SCE1572_6799	sce_5658	5.00E-50	3.00E-78
SCE1572_68	sce_61	0.00E+00	0.00E+00
SCE1572_6800	sce_5660	0.00E+00	0.00E+00
SCE1572_6802	sce_5662	0.00E+00	0.00E+00
SCE1572_6803	sce_5663	1.00E-111	8.00E-102
SCE1572_6804	sce_5664	3.00E-106	3.00E-110
SCE1572_6806	sce_5666	0.00E+00	0.00E+00
SCE1572_6807	sce_5667	2.00E-133	2.00E-133
SCE1572_6808	sce_5668	5.00E-134	9.00E-143
SCE1572_6809	sce_5669	0.00E+00	0.00E+00
SCE1572_681	sce_484	2.00E-77	7.00E-90
SCE1572_6810	sce_5670	0.00E+00	0.00E+00
SCE1572_6811	sce_5671	3.00E-134	5.00E-141
SCE1572_6812	sce_5672	4.00E-24	5.00E-37
SCE1572_6813	sce_5673	0.00E+00	0.00E+00
SCE1572_6814	sce_5675	0.00E+00	0.00E+00
SCE1572_6815	sce_5677	0.00E+00	0.00E+00
SCE1572_6816	sce_5678	0.00E+00	0.00E+00
SCE1572_6817	sce_5679	9.00E-55	5.00E-55
SCE1572_6818	sce_5680	4.00E-60	3.00E-60
SCE1572_6819	sce_5681	1.00E-97	4.00E-101
SCE1572_682	sce_485	0.00E+00	0.00E+00
SCE1572_6820	sce_5682	2.00E-35	3.00E-34
SCE1572_6821	sce_5683	1.00E-116	2.00E-107
SCE1572_6822	sce_5684	6.00E-79	8.00E-80
SCE1572_6823	sce_5685	0.00E+00	0.00E+00
SCE1572_6824	sce_5686	4.00E-124	4.00E-118
SCE1572_6826	sce_5687	4.00E-63	1.00E-62
SCE1572_6827	sce_5688	0.00E+00	0.00E+00
SCE1572_6828	sce_5689	0.00E+00	0.00E+00
SCE1572_683	sce_486	8.00E-47	9.00E-47
SCE1572_6831	sce_5691	0.00E+00	0.00E+00
SCE1572_6832	sce_5694	5.00E-87	8.00E-88
SCE1572_6833	sce_5695	1.00E-146	1.00E-162
SCE1572_6835	sce_5696	1.00E-93	1.00E-93
SCE1572_6837	sce_5697	1.00E-73	8.00E-74
SCE1572_6838	sce_5698	3.00E-69	4.00E-69
SCE1572_6839	sce_5699	2.00E-169	0.00E+00
SCE1572_684	sce_487	1.00E-156	2.00E-147
SCE1572_6840	sce_5700	2.00E-93	1.00E-93
SCE1572_6841	sce_5701	0.00E+00	0.00E+00
SCE1572_6842	sce_5702	1.00E-147	2.00E-146
SCE1572_6843	sce_5703	6.00E-104	2.00E-112
SCE1572_6844	sce_5704	0.00E+00	0.00E+00
SCE1572_6845	sce_5705	6.00E-78	3.00E-86
SCE1572_6846	sce_5706	2.00E-99	2.00E-99
SCE1572_6847	sce_5707	0.00E+00	1.00E-130
SCE1572_6848	sce_5709	0.00E+00	1.00E-168
SCE1572_6849	sce_5711	0.00E+00	0.00E+00
SCE1572_6850	sce_5712	4.00E-85	4.00E-66
SCE1572_6851	sce_5713	3.00E-27	2.00E-30
SCE1572_6853	sce_5714	0.00E+00	0.00E+00
SCE1572_6854	sce_5715	7.00E-79	1.00E-79
SCE1572_6855	sce_5716	6.00E-30	7.00E-30
SCE1572_6856	sce_5717	5.00E-94	5.00E-94
SCE1572_6857	sce_5718	1.00E-97	2.00E-102
SCE1572_6858	sce_5719	2.00E-168	1.00E-157
SCE1572_6859	sce_5720	2.00E-51	4.00E-51
SCE1572_686	sce_488	0.00E+00	0.00E+00
SCE1572_6860	sce_5721	0.00E+00	0.00E+00

SCE1572_6860	sce_8732	0.00E+00	0.00E+00
SCE1572_6861	sce_5723	8.00E-23	1.00E-22
SCE1572_6862	sce_5724	0.00E+00	0.00E+00
SCE1572_6863	sce_5725	2.00E-165	6.00E-171
SCE1572_6865	sce_5728	0.00E+00	0.00E+00
SCE1572_6866	sce_5729	0.00E+00	0.00E+00
SCE1572_6867	sce_5730	0.00E+00	0.00E+00
SCE1572_6868	sce_5731	2.00E-95	3.00E-90
SCE1572_6869	sce_5732	9.00E-51	8.00E-58
SCE1572_687	sce_489	8.00E-53	1.00E-49
SCE1572_6870	sce_5733	7.00E-176	2.00E-169
SCE1572_6871	sce_5734	2.00E-100	2.00E-96
SCE1572_6872	sce_5735	1.00E-162	9.00E-163
SCE1572_6873	sce_5737	6.00E-148	1.00E-156
SCE1572_6874	sce_5738	7.00E-130	0.00E+00
SCE1572_6875	sce_5739	7.00E-16	4.00E-17
SCE1572_6876	sce_5741	2.00E-45	4.00E-41
SCE1572_6877	sce_5742	0.00E+00	0.00E+00
SCE1572_6878	sce_5743	4.00E-113	6.00E-118
SCE1572_6879	sce_5744	0.00E+00	0.00E+00
SCE1572_688	sce_490	6.00E-123	4.00E-118
SCE1572_6883	sce_5745	4.00E-120	3.00E-117
SCE1572_6886	sce_9736	1.00E-60	5.00E-61
SCE1572_689	sce_491	3.00E-172	0.00E+00
SCE1572_6892	sce_5751	0.00E+00	0.00E+00
SCE1572_6892	sce_5746	0.00E+00	0.00E+00
SCE1572_6892	sce_9665	0.00E+00	0.00E+00
SCE1572_6892	sce_5403	0.00E+00	0.00E+00
SCE1572_6892	sce_5226	0.00E+00	0.00E+00
SCE1572_6892	sce_1803	0.00E+00	0.00E+00
SCE1572_6892	sce_7154	0.00E+00	0.00E+00
SCE1572_6892	sce_7969	0.00E+00	0.00E+00
SCE1572_6892	sce_2053	0.00E+00	0.00E+00
SCE1572_6893	sce_5746	0.00E+00	0.00E+00
SCE1572_6893	sce_9665	0.00E+00	0.00E+00
SCE1572_6893	sce_5751	0.00E+00	0.00E+00
SCE1572_6893	sce_5403	0.00E+00	0.00E+00
SCE1572_6893	sce_5226	0.00E+00	0.00E+00
SCE1572_6893	sce_7969	0.00E+00	0.00E+00
SCE1572_6893	sce_2053	0.00E+00	0.00E+00
SCE1572_6893	sce_1803	0.00E+00	0.00E+00
SCE1572_6893	sce_4073	0.00E+00	0.00E+00
SCE1572_6893	sce_9954	0.00E+00	0.00E+00
SCE1572_6893	sce_3750	0.00E+00	0.00E+00
SCE1572_6894	sce_5751	0.00E+00	0.00E+00
SCE1572_6894	sce_5746	0.00E+00	0.00E+00
SCE1572_6894	sce_9665	0.00E+00	0.00E+00
SCE1572_6894	sce_5226	0.00E+00	0.00E+00
SCE1572_6894	sce_5403	0.00E+00	0.00E+00
SCE1572_6894	sce_7969	0.00E+00	0.00E+00
SCE1572_6894	sce_4073	0.00E+00	0.00E+00
SCE1572_6894	sce_7154	0.00E+00	0.00E+00
SCE1572_6894	sce_2053	0.00E+00	0.00E+00
SCE1572_6894	sce_9954	0.00E+00	0.00E+00
SCE1572_6894	sce_9757	0.00E+00	0.00E+00
SCE1572_6894	sce_6712	0.00E+00	0.00E+00
SCE1572_6894	sce_3750	0.00E+00	0.00E+00
SCE1572_6894	sce_1803	0.00E+00	0.00E+00
SCE1572_6898	sce_5752	0.00E+00	0.00E+00
SCE1572_6899	sce_5753	3.00E-127	1.00E-118
SCE1572_69	sce_62	3.00E-21	2.00E-12
SCE1572_690	sce_492	4.00E-58	1.00E-58
SCE1572_6903	sce_2563	5.00E-44	2.00E-37
SCE1572_691	sce_494	4.00E-151	8.00E-155
SCE1572_6910	sce_9236	0.00E+00	0.00E+00
SCE1572_6910	sce_389	0.00E+00	0.00E+00
SCE1572_6915	sce_5756	0.00E+00	0.00E+00
SCE1572_6916	sce_5761	5.00E-143	6.00E-143
SCE1572_6917	sce_8380	3.00E-64	3.00E-48
SCE1572_6918	sce_5762	5.00E-68	5.00E-68
SCE1572_6919	sce_8379	2.00E-89	1.00E-97
SCE1572_692	sce_495	6.00E-42	6.00E-42
SCE1572_6921	sce_2065	0.00E+00	0.00E+00
SCE1572_6921	sce_8618	0.00E+00	0.00E+00
SCE1572_6921	sce_10204	0.00E+00	0.00E+00
SCE1572_6921	sce_6092	0.00E+00	0.00E+00

SCE1572_6921	sce_6535	0.00E+00	0.00E+00
SCE1572_6921	sce_3003	0.00E+00	0.00E+00
SCE1572_6921	sce_6095	0.00E+00	0.00E+00
SCE1572_6921	sce_7661	0.00E+00	0.00E+00
SCE1572_6921	sce_388	0.00E+00	0.00E+00
SCE1572_6921	sce_7640	0.00E+00	0.00E+00
SCE1572_6921	sce_4513	0.00E+00	0.00E+00
SCE1572_6921	sce_9557	0.00E+00	0.00E+00
SCE1572_6921	sce_5835	0.00E+00	0.00E+00
SCE1572_6922	sce_1259	1.00E-22	5.00E-27
SCE1572_6923	sce_805	0.00E+00	0.00E+00
SCE1572_6923	sce_229	0.00E+00	0.00E+00
SCE1572_6929	sce_5770	5.00E-15	1.00E-21
SCE1572_693	sce_496	0.00E+00	0.00E+00
SCE1572_6932	sce_5763	1.00E-76	1.00E-76
SCE1572_6937	sce_5766	4.00E-41	2.00E-43
SCE1572_6939	sce_5767	4.00E-121	9.00E-127
SCE1572_694	sce_9364	2.00E-47	8.00E-48
SCE1572_6940	sce_5768	3.00E-51	2.00E-53
SCE1572_6941	sce_5769	0.00E+00	0.00E+00
SCE1572_6942	sce_1680	0.00E+00	0.00E+00
SCE1572_6946	sce_5528	6.00E-65	6.00E-65
SCE1572_6947	sce_5773	3.00E-94	5.00E-113
SCE1572_6948	sce_5835	0.00E+00	0.00E+00
SCE1572_6948	sce_388	0.00E+00	0.00E+00
SCE1572_6948	sce_7640	0.00E+00	0.00E+00
SCE1572_6948	sce_3085	0.00E+00	0.00E+00
SCE1572_6948	sce_7109	0.00E+00	0.00E+00
SCE1572_6948	sce_3003	0.00E+00	0.00E+00
SCE1572_6948	sce_10204	0.00E+00	0.00E+00
SCE1572_6948	sce_4513	0.00E+00	0.00E+00
SCE1572_695	sce_9358	3.00E-66	7.00E-71
SCE1572_6953	sce_2385	1.00E-37	1.00E-38
SCE1572_6955	sce_5883	5.00E-75	5.00E-65
SCE1572_6956	sce_177	2.00E-15	4.00E-12
SCE1572_6960	sce_5775	0.00E+00	0.00E+00
SCE1572_6962	sce_5776	3.00E-77	7.00E-77
SCE1572_6963	sce_5777	0.00E+00	0.00E+00
SCE1572_6964	sce_5778	0.00E+00	0.00E+00
SCE1572_6965	sce_5779	2.00E-125	2.00E-125
SCE1572_6966	sce_5780	0.00E+00	0.00E+00
SCE1572_6967	sce_5781	0.00E+00	0.00E+00
SCE1572_6968	sce_5782	1.00E-147	2.00E-135
SCE1572_6969	sce_5783	1.00E-66	9.00E-55
SCE1572_6970	sce_5784	4.00E-36	1.00E-39
SCE1572_6974	sce_5787	0.00E+00	0.00E+00
SCE1572_6974	sce_1723	0.00E+00	0.00E+00
SCE1572_6974	sce_774	0.00E+00	0.00E+00
SCE1572_6975	sce_5788	4.00E-102	4.00E-114
SCE1572_6976	sce_5789	1.00E-156	1.00E-163
SCE1572_6978	sce_5790	6.00E-86	4.00E-85
SCE1572_6980	sce_5791	2.00E-123	1.00E-123
SCE1572_6981	sce_5794	2.00E-77	2.00E-77
SCE1572_6983	sce_5797	0.00E+00	0.00E+00
SCE1572_6983	sce_3612	0.00E+00	0.00E+00
SCE1572_6983	sce_886	0.00E+00	0.00E+00
SCE1572_6983	sce_248	0.00E+00	0.00E+00
SCE1572_6984	sce_5799	2.00E-40	1.00E-35
SCE1572_6985	sce_5800	0.00E+00	0.00E+00
SCE1572_6988	sce_5805	3.00E-106	3.00E-98
SCE1572_6991	sce_5807	7.00E-180	2.00E-170
SCE1572_6992	sce_100	2.00E-129	2.00E-136
SCE1572_6993	sce_5811	9.00E-108	3.00E-123
SCE1572_6994	sce_5812	3.00E-112	2.00E-111
SCE1572_6995	sce_5813	6.00E-98	4.00E-86
SCE1572_6996	sce_5815	4.00E-76	6.00E-76
SCE1572_7	sce_7	1.00E-88	1.00E-88
SCE1572_70	sce_63	2.00E-151	6.00E-152
SCE1572_7003	sce_3413	0.00E+00	0.00E+00
SCE1572_7004	sce_5821	6.00E-86	2.00E-89
SCE1572_7005	sce_5822	6.00E-64	2.00E-57
SCE1572_7008	sce_6818	4.00E-08	4.00E-08
SCE1572_7009	sce_9483	1.00E-101	1.00E-113
SCE1572_701	sce_497	0.00E+00	0.00E+00
SCE1572_7011	sce_9481	9.00E-135	3.00E-124
SCE1572_7015	sce_9479	1.00E-29	9.00E-34

SCE1572_702	sce_498	1.00E-24	3.00E-23
SCE1572_7022	sce_6228	0.00E+00	0.00E+00
SCE1572_7022	sce_6833	0.00E+00	0.00E+00
SCE1572_7022	sce_6827	0.00E+00	0.00E+00
SCE1572_7022	sce_10159	0.00E+00	0.00E+00
SCE1572_7022	sce_7513	0.00E+00	0.00E+00
SCE1572_7022	sce_7408	0.00E+00	0.00E+00
SCE1572_7022	sce_6876	0.00E+00	0.00E+00
SCE1572_7022	sce_6836	0.00E+00	0.00E+00
SCE1572_7022	sce_2543	0.00E+00	0.00E+00
SCE1572_7026	sce_5823	0.00E+00	0.00E+00
SCE1572_7027	sce_5824	0.00E+00	0.00E+00
SCE1572_7027	sce_5830	0.00E+00	0.00E+00
SCE1572_7027	sce_2940	0.00E+00	0.00E+00
SCE1572_7027	sce_5827	0.00E+00	0.00E+00
SCE1572_7027	sce_2589	0.00E+00	0.00E+00
SCE1572_7029	sce_5827	0.00E+00	0.00E+00
SCE1572_7029	sce_5830	0.00E+00	0.00E+00
SCE1572_7029	sce_5824	0.00E+00	0.00E+00
SCE1572_7029	sce_2940	0.00E+00	0.00E+00
SCE1572_7029	sce_2589	0.00E+00	0.00E+00
SCE1572_703	sce_500	1.00E-17	9.00E-31
SCE1572_7030	sce_5828	7.00E-57	7.00E-57
SCE1572_7032	sce_5830	0.00E+00	0.00E+00
SCE1572_7032	sce_5827	0.00E+00	0.00E+00
SCE1572_7032	sce_5824	0.00E+00	0.00E+00
SCE1572_7032	sce_2940	0.00E+00	0.00E+00
SCE1572_7032	sce_2589	0.00E+00	0.00E+00
SCE1572_7033	sce_5831	2.00E-63	2.00E-63
SCE1572_7034	sce_5832	2.00E-83	2.00E-70
SCE1572_7035	sce_5833	0.00E+00	0.00E+00
SCE1572_7036	sce_5834	2.00E-97	2.00E-97
SCE1572_704	sce_501	0.00E+00	0.00E+00
SCE1572_7041	sce_5839	5.00E-70	4.00E-79
SCE1572_7044	sce_9896	3.00E-106	3.00E-115
SCE1572_705	sce_502	0.00E+00	0.00E+00
SCE1572_706	sce_503	3.00E-40	9.00E-50
SCE1572_7063	sce_7667	0.00E+00	0.00E+00
SCE1572_7068	sce_5840	0.00E+00	0.00E+00
SCE1572_7069	sce_1257	9.00E-50	3.00E-54
SCE1572_707	sce_504	0.00E+00	0.00E+00
SCE1572_7072	sce_5849	0.00E+00	0.00E+00
SCE1572_7074	sce_5850	1.00E-72	1.00E-72
SCE1572_7075	sce_5851	1.00E-117	8.00E-117
SCE1572_7076	sce_5854	0.00E+00	0.00E+00
SCE1572_7079	sce_5855	6.00E-55	6.00E-55
SCE1572_708	sce_505	2.00E-92	2.00E-92
SCE1572_7080	sce_5856	0.00E+00	0.00E+00
SCE1572_7081	sce_5857	9.00E-152	4.00E-153
SCE1572_7082	sce_5858	2.00E-81	3.00E-76
SCE1572_7083	sce_5859	5.00E-72	3.00E-84
SCE1572_7084	sce_5860	1.00E-167	2.00E-159
SCE1572_7087	sce_9904	1.00E-85	3.00E-80
SCE1572_7091	sce_5861	0.00E+00	0.00E+00
SCE1572_7095	sce_5863	4.00E-45	7.00E-43
SCE1572_7096	sce_1560	0.00E+00	0.00E+00
SCE1572_7097	sce_1561	3.00E-112	3.00E-105
SCE1572_7098	sce_1562	2.00E-115	2.00E-114
SCE1572_71	sce_64	6.00E-100	2.00E-102
SCE1572_710	sce_507	2.00E-50	2.00E-50
SCE1572_7100	sce_5868	9.00E-66	3.00E-58
SCE1572_7101	sce_5869	0.00E+00	0.00E+00
SCE1572_7102	sce_5870	0.00E+00	0.00E+00
SCE1572_7104	sce_5836	0.00E+00	0.00E+00
SCE1572_7105	sce_5837	0.00E+00	0.00E+00
SCE1572_7109	sce_1435	6.00E-36	6.00E-36
SCE1572_711	sce_508	7.00E-59	4.00E-67
SCE1572_7114	sce_7551	1.00E-115	5.00E-107
SCE1572_7118	sce_9883	4.00E-77	3.00E-93
SCE1572_7122	sce_5871	0.00E+00	0.00E+00
SCE1572_7124	sce_5872	0.00E+00	0.00E+00
SCE1572_7125	sce_5874	0.00E+00	0.00E+00
SCE1572_713	sce_509	0.00E+00	0.00E+00
SCE1572_7131	sce_8424	5.00E-23	7.00E-15
SCE1572_7132	sce_3684	5.00E-85	5.00E-85
SCE1572_7133	sce_5482	0.00E+00	0.00E+00

SCE1572_7134	sce_6324	5.00E-50	3.00E-50
SCE1572_7135	sce_5876	5.00E-167	2.00E-173
SCE1572_7136	sce_5877	6.00E-130	2.00E-140
SCE1572_7143	sce_301	2.00E-69	4.00E-66
SCE1572_7144	sce_5878	4.00E-66	7.00E-74
SCE1572_7146	sce_5879	2.00E-57	1.00E-57
SCE1572_7147	sce_5880	2.00E-158	5.00E-168
SCE1572_7148	sce_5885	1.00E-110	1.00E-73
SCE1572_7149	sce_5886	4.00E-67	1.00E-67
SCE1572_7150	sce_5887	4.00E-98	2.00E-98
SCE1572_7151	sce_5889	2.00E-31	1.00E-32
SCE1572_7152	sce_5890	2.00E-126	3.00E-122
SCE1572_7153	sce_9846	2.00E-115	2.00E-128
SCE1572_7155	sce_5893	3.00E-160	1.00E-149
SCE1572_7157	sce_1355	7.00E-23	2.00E-25
SCE1572_7159	sce_5894	6.00E-59	2.00E-59
SCE1572_7161	sce_5895	3.00E-73	4.00E-73
SCE1572_7162	sce_5896	3.00E-171	3.00E-171
SCE1572_7163	sce_5897	2.00E-163	3.00E-155
SCE1572_7164	sce_3432	1.00E-28	2.00E-37
SCE1572_7170	sce_5899	0.00E+00	0.00E+00
SCE1572_7171	sce_5900	3.00E-28	3.00E-28
SCE1572_7172	sce_5901	1.00E-80	2.00E-80
SCE1572_7173	sce_5902	0.00E+00	0.00E+00
SCE1572_7175	sce_5904	0.00E+00	0.00E+00
SCE1572_7176	sce_5905	1.00E-176	1.00E-163
SCE1572_7178	sce_2175	0.00E+00	0.00E+00
SCE1572_7181	sce_6290	2.00E-18	2.00E-18
SCE1572_7182	sce_1246	2.00E-159	6.00E-160
SCE1572_7183	sce_1245	2.00E-118	2.00E-115
SCE1572_7185	sce_5906	3.00E-62	5.00E-71
SCE1572_7186	sce_5907	0.00E+00	0.00E+00
SCE1572_7187	sce_5908	0.00E+00	0.00E+00
SCE1572_7188	sce_5909	0.00E+00	0.00E+00
SCE1572_7189	sce_1882	2.00E-129	1.00E-118
SCE1572_7190	sce_1881	2.00E-122	3.00E-114
SCE1572_7191	sce_5914	5.00E-117	9.00E-118
SCE1572_7193	sce_5915	0.00E+00	0.00E+00
SCE1572_7199	sce_8384	3.00E-134	3.00E-134
SCE1572_72	sce_65	2.00E-153	6.00E-148
SCE1572_7200	sce_5918	0.00E+00	0.00E+00
SCE1572_7201	sce_5919	9.00E-139	7.00E-138
SCE1572_7206	sce_5924	6.00E-101	3.00E-101
SCE1572_7207	sce_5926	4.00E-79	2.00E-93
SCE1572_7208	sce_5920	7.00E-73	3.00E-76
SCE1572_7209	sce_5936	1.00E-146	3.00E-147
SCE1572_7210	sce_5937	3.00E-37	2.00E-35
SCE1572_7211	sce_5938	8.00E-160	1.00E-170
SCE1572_7212	sce_5939	1.00E-10	1.00E-10
SCE1572_7214	sce_5941	3.00E-105	1.00E-110
SCE1572_7216	sce_5944	2.00E-133	2.00E-139
SCE1572_7219	sce_5945	2.00E-90	9.00E-93
SCE1572_7220	sce_5946	1.00E-10	1.00E-28
SCE1572_7222	sce_5947	7.00E-154	4.00E-152
SCE1572_7223	sce_5948	0.00E+00	0.00E+00
SCE1572_7224	sce_5950	6.00E-176	6.00E-176
SCE1572_7225	sce_5951	2.00E-173	7.00E-175
SCE1572_7226	sce_5952	2.00E-129	2.00E-112
SCE1572_7227	sce_5953	3.00E-56	7.00E-66
SCE1572_7228	sce_5954	3.00E-88	3.00E-86
SCE1572_7229	sce_5955	2.00E-128	3.00E-138
SCE1572_7233	sce_8264	3.00E-66	3.00E-78
SCE1572_7234	sce_5956	2.00E-77	5.00E-75
SCE1572_7235	sce_5957	0.00E+00	0.00E+00
SCE1572_7236	sce_5958	0.00E+00	0.00E+00
SCE1572_7237	sce_5960	0.00E+00	0.00E+00
SCE1572_7238	sce_5962	0.00E+00	0.00E+00
SCE1572_7240	sce_5963	4.00E-117	1.00E-106
SCE1572_7241	sce_7661	0.00E+00	0.00E+00
SCE1572_7241	sce_6107	0.00E+00	0.00E+00
SCE1572_7241	sce_3917	0.00E+00	0.00E+00
SCE1572_7241	sce_6093	0.00E+00	0.00E+00
SCE1572_7241	sce_3003	0.00E+00	0.00E+00
SCE1572_7241	sce_6535	0.00E+00	0.00E+00
SCE1572_7241	sce_6092	0.00E+00	0.00E+00
SCE1572_7241	sce_388	0.00E+00	0.00E+00

SCE1572_7241	sce_6094	0.00E+00	0.00E+00
SCE1572_7241	sce_8602	0.00E+00	0.00E+00
SCE1572_7241	sce_6095	0.00E+00	0.00E+00
SCE1572_7241	sce_9920	0.00E+00	0.00E+00
SCE1572_7241	sce_7640	0.00E+00	0.00E+00
SCE1572_7241	sce_5835	0.00E+00	0.00E+00
SCE1572_7241	sce_10204	0.00E+00	0.00E+00
SCE1572_7241	sce_8618	0.00E+00	0.00E+00
SCE1572_7242	sce_5964	0.00E+00	0.00E+00
SCE1572_7243	sce_5965	0.00E+00	0.00E+00
SCE1572_7245	sce_5966	2.00E-47	2.00E-46
SCE1572_7246	sce_5968	6.00E-126	6.00E-126
SCE1572_7248	sce_5969	1.00E-35	1.00E-35
SCE1572_7249	sce_2378	0.00E+00	0.00E+00
SCE1572_725	sce_9985	5.00E-42	2.00E-27
SCE1572_7250	sce_2378	0.00E+00	0.00E+00
SCE1572_7252	sce_2379	6.00E-177	4.00E-171
SCE1572_7253	sce_2380	1.00E-60	9.00E-61
SCE1572_7254	sce_2382	3.00E-65	7.00E-34
SCE1572_7255	sce_5970	0.00E+00	0.00E+00
SCE1572_7256	sce_5971	1.00E-158	3.00E-177
SCE1572_7257	sce_5537	1.00E-30	4.00E-33
SCE1572_7258	sce_5972	4.00E-147	4.00E-147
SCE1572_726	sce_527	0.00E+00	0.00E+00
SCE1572_7260	sce_5973	3.00E-103	2.00E-111
SCE1572_7263	sce_5978	3.00E-97	4.00E-84
SCE1572_7265	sce_5979	0.00E+00	0.00E+00
SCE1572_7268	sce_5980	0.00E+00	0.00E+00
SCE1572_7270	sce_5982	0.00E+00	0.00E+00
SCE1572_7271	sce_6026	4.00E-68	3.00E-63
SCE1572_7272	sce_6025	1.00E-65	3.00E-73
SCE1572_7275	sce_6816	7.00E-76	1.00E-84
SCE1572_7277	sce_7693	2.00E-19	2.00E-19
SCE1572_7279	sce_629	2.00E-06	3.00E-06
SCE1572_728	sce_528	2.00E-158	1.00E-157
SCE1572_7280	sce_5983	1.00E-82	8.00E-65
SCE1572_7281	sce_5984	3.00E-79	1.00E-72
SCE1572_7282	sce_5986	7.00E-125	6.00E-129
SCE1572_7283	sce_5987	0.00E+00	0.00E+00
SCE1572_7285	sce_5988	0.00E+00	0.00E+00
SCE1572_7286	sce_5989	0.00E+00	0.00E+00
SCE1572_7287	sce_5990	3.00E-62	2.00E-62
SCE1572_7288	sce_6130	0.00E+00	0.00E+00
SCE1572_729	sce_529	3.00E-86	2.00E-81
SCE1572_7290	sce_5991	2.00E-120	2.00E-128
SCE1572_7296	sce_6071	8.00E-138	7.00E-142
SCE1572_7297	sce_6072	3.00E-34	5.00E-34
SCE1572_7298	sce_6074	0.00E+00	0.00E+00
SCE1572_7299	sce_3003	0.00E+00	0.00E+00
SCE1572_7299	sce_9920	0.00E+00	0.00E+00
SCE1572_7299	sce_6535	0.00E+00	0.00E+00
SCE1572_7299	sce_388	0.00E+00	0.00E+00
SCE1572_7299	sce_5835	0.00E+00	0.00E+00
SCE1572_7299	sce_9557	0.00E+00	0.00E+00
SCE1572_7299	sce_10204	0.00E+00	0.00E+00
SCE1572_7299	sce_7640	0.00E+00	0.00E+00
SCE1572_7299	sce_4513	0.00E+00	0.00E+00
SCE1572_7299	sce_3085	0.00E+00	0.00E+00
SCE1572_7299	sce_3917	0.00E+00	0.00E+00
SCE1572_7299	sce_6093	0.00E+00	0.00E+00
SCE1572_7299	sce_6092	0.00E+00	0.00E+00
SCE1572_7299	sce_6107	0.00E+00	0.00E+00
SCE1572_7299	sce_7661	0.00E+00	0.00E+00
SCE1572_7299	sce_8618	0.00E+00	0.00E+00
SCE1572_7299	sce_6095	0.00E+00	0.00E+00
SCE1572_7299	sce_2065	0.00E+00	0.00E+00
SCE1572_7299	sce_6094	0.00E+00	0.00E+00
SCE1572_7299	sce_7109	0.00E+00	0.00E+00
SCE1572_73	sce_66	0.00E+00	0.00E+00
SCE1572_730	sce_532	0.00E+00	0.00E+00
SCE1572_7300	sce_3004	3.00E-82	3.00E-82
SCE1572_7302	sce_2466	3.00E-09	1.00E-09
SCE1572_7303	sce_5169	1.00E-92	4.00E-95
SCE1572_7304	sce_5168	3.00E-119	1.00E-121
SCE1572_7305	sce_2493	9.00E-138	5.00E-144
SCE1572_7308	sce_10414	1.00E-14	7.00E-14

SCE1572_7309	sce_4708	0.00E+00	0.00E+00
SCE1572_7309	sce_6222	0.00E+00	0.00E+00
SCE1572_731	sce_533	2.00E-113	5.00E-116
SCE1572_7310	sce_6091	3.00E-141	5.00E-155
SCE1572_7311	sce_6095	0.00E+00	0.00E+00
SCE1572_7311	sce_6093	0.00E+00	0.00E+00
SCE1572_7311	sce_6092	0.00E+00	0.00E+00
SCE1572_7311	sce_6094	0.00E+00	0.00E+00
SCE1572_7311	sce_6107	0.00E+00	0.00E+00
SCE1572_7311	sce_3917	0.00E+00	0.00E+00
SCE1572_7311	sce_7661	0.00E+00	0.00E+00
SCE1572_7311	sce_388	0.00E+00	0.00E+00
SCE1572_7311	sce_9920	0.00E+00	0.00E+00
SCE1572_7311	sce_6535	0.00E+00	0.00E+00
SCE1572_7311	sce_4513	0.00E+00	0.00E+00
SCE1572_7311	sce_3003	0.00E+00	0.00E+00
SCE1572_7311	sce_9557	0.00E+00	0.00E+00
SCE1572_7311	sce_10204	0.00E+00	0.00E+00
SCE1572_7311	sce_8602	0.00E+00	0.00E+00
SCE1572_7311	sce_7640	0.00E+00	0.00E+00
SCE1572_7311	sce_5835	0.00E+00	0.00E+00
SCE1572_7319	sce_6109	4.00E-70	7.00E-67
SCE1572_732	sce_534	3.00E-180	8.00E-179
SCE1572_7321	sce_6110	4.00E-18	4.00E-18
SCE1572_7323	sce_6111	5.00E-38	8.00E-37
SCE1572_7325	sce_6112	6.00E-179	3.00E-178
SCE1572_7326	sce_6113	7.00E-159	1.00E-145
SCE1572_7327	sce_6114	0.00E+00	0.00E+00
SCE1572_7327	sce_6541	0.00E+00	0.00E+00
SCE1572_7329	sce_6116	2.00E-60	2.00E-55
SCE1572_733	sce_536	3.00E-121	2.00E-131
SCE1572_7330	sce_6117	0.00E+00	0.00E+00
SCE1572_7332	sce_6119	0.00E+00	0.00E+00
SCE1572_7334	sce_6120	0.00E+00	0.00E+00
SCE1572_7336	sce_6121	0.00E+00	0.00E+00
SCE1572_7337	sce_6122	0.00E+00	0.00E+00
SCE1572_7338	sce_6123	6.00E-163	7.00E-164
SCE1572_7339	sce_6124	7.00E-47	7.00E-49
SCE1572_734	sce_537	0.00E+00	0.00E+00
SCE1572_7340	sce_6125	2.00E-102	2.00E-101
SCE1572_7341	sce_6126	0.00E+00	0.00E+00
SCE1572_7342	sce_6127	0.00E+00	0.00E+00
SCE1572_7343	sce_4461	0.00E+00	0.00E+00
SCE1572_7348	sce_6131	2.00E-88	3.00E-88
SCE1572_7349	sce_6132	3.00E-58	1.00E-64
SCE1572_735	sce_538	9.00E-88	4.00E-101
SCE1572_7350	sce_6133	4.00E-56	2.00E-75
SCE1572_7351	sce_6134	5.00E-77	3.00E-54
SCE1572_7352	sce_6135	6.00E-115	2.00E-116
SCE1572_7357	sce_6139	3.00E-113	3.00E-129
SCE1572_7358	sce_6140	8.00E-137	8.00E-137
SCE1572_736	sce_539	2.00E-102	7.00E-105
SCE1572_7360	sce_6141	0.00E+00	0.00E+00
SCE1572_7361	sce_6142	6.00E-108	2.00E-90
SCE1572_7363	sce_6144	0.00E+00	0.00E+00
SCE1572_7364	sce_6145	0.00E+00	0.00E+00
SCE1572_7365	sce_2350	0.00E+00	0.00E+00
SCE1572_7366	sce_6146	6.00E-160	7.00E-163
SCE1572_7367	sce_6147	0.00E+00	0.00E+00
SCE1572_7368	sce_6148	2.00E-74	1.00E-82
SCE1572_7369	sce_6149	1.00E-30	1.00E-33
SCE1572_7370	sce_6150	0.00E+00	0.00E+00
SCE1572_7371	sce_6151	2.00E-46	1.00E-52
SCE1572_7372	sce_6153	0.00E+00	0.00E+00
SCE1572_7377	sce_6154	0.00E+00	0.00E+00
SCE1572_7379	sce_7617	7.00E-31	3.00E-31
SCE1572_738	sce_540	1.00E-171	3.00E-166
SCE1572_7380	sce_868	1.00E-179	0.00E+00
SCE1572_7384	sce_2434	9.00E-168	6.00E-161
SCE1572_7387	sce_6155	2.00E-160	2.00E-160
SCE1572_7388	sce_6156	6.00E-155	8.00E-150
SCE1572_739	sce_541	0.00E+00	0.00E+00
SCE1572_7390	sce_7829	6.00E-49	3.00E-50
SCE1572_7391	sce_7831	1.00E-149	4.00E-152
SCE1572_7393	sce_7832	5.00E-60	4.00E-60
SCE1572_7394	sce_7833	3.00E-74	3.00E-76

SCE1572_7395	sce_7834	1.00E-103	1.00E-96
SCE1572_7396	sce_6157	9.00E-92	1.00E-89
SCE1572_7397	sce_6158	5.00E-126	1.00E-125
SCE1572_7398	sce_6159	0.00E+00	0.00E+00
SCE1572_7399	sce_6161	1.00E-66	2.00E-51
SCE1572_74	sce_67	5.00E-119	4.00E-106
SCE1572_740	sce_542	3.00E-54	4.00E-42
SCE1572_7400	sce_6162	4.00E-141	9.00E-142
SCE1572_7403	sce_6163	5.00E-151	2.00E-150
SCE1572_7404	sce_6164	6.00E-67	5.00E-67
SCE1572_7405	sce_6165	8.00E-73	1.00E-72
SCE1572_7406	sce_6166	2.00E-88	2.00E-71
SCE1572_7408	sce_6167	7.00E-110	7.00E-110
SCE1572_741	sce_543	8.00E-85	1.00E-96
SCE1572_7411	sce_6169	4.00E-58	6.00E-61
SCE1572_7412	sce_6170	1.00E-165	1.00E-161
SCE1572_7413	sce_6171	0.00E+00	0.00E+00
SCE1572_7416	sce_6172	6.00E-140	8.00E-151
SCE1572_7417	sce_6173	0.00E+00	0.00E+00
SCE1572_742	sce_544	4.00E-156	4.00E-157
SCE1572_7420	sce_6175	1.00E-127	3.00E-132
SCE1572_7423	sce_6176	3.00E-39	1.00E-46
SCE1572_7424	sce_6177	2.00E-127	2.00E-127
SCE1572_7425	sce_6178	2.00E-76	2.00E-74
SCE1572_7427	sce_5086	0.00E+00	0.00E+00
SCE1572_7427	sce_5085	0.00E+00	0.00E+00
SCE1572_7427	sce_6701	0.00E+00	0.00E+00
SCE1572_7427	sce_3793	0.00E+00	0.00E+00
SCE1572_7428	sce_6179	0.00E+00	0.00E+00
SCE1572_7429	sce_6180	2.00E-153	5.00E-147
SCE1572_743	sce_545	5.00E-85	2.00E-110
SCE1572_7430	sce_3028	0.00E+00	0.00E+00
SCE1572_7431	sce_4600	5.00E-96	3.00E-109
SCE1572_7432	sce_6207	5.00E-66	5.00E-69
SCE1572_7433	sce_6208	1.00E-34	3.00E-35
SCE1572_7434	sce_6211	2.00E-43	2.00E-43
SCE1572_7435	sce_6212	1.00E-177	0.00E+00
SCE1572_7436	sce_8363	6.00E-29	2.00E-29
SCE1572_7438	sce_6218	4.00E-105	3.00E-106
SCE1572_7443	sce_5549	7.00E-66	2.00E-65
SCE1572_7446	sce_6219	2.00E-32	2.00E-32
SCE1572_7448	sce_6220	0.00E+00	0.00E+00
SCE1572_7449	sce_6221	0.00E+00	0.00E+00
SCE1572_745	sce_548	0.00E+00	0.00E+00
SCE1572_7453	sce_6235	0.00E+00	0.00E+00
SCE1572_7453	sce_2161	0.00E+00	0.00E+00
SCE1572_7453	sce_225	0.00E+00	0.00E+00
SCE1572_7453	sce_8357	0.00E+00	0.00E+00
SCE1572_7453	sce_5276	0.00E+00	0.00E+00
SCE1572_7454	sce_6238	4.00E-106	6.00E-96
SCE1572_7455	sce_6239	3.00E-159	1.00E-149
SCE1572_7456	sce_6240	0.00E+00	3.00E-174
SCE1572_7457	sce_6241	4.00E-73	8.00E-85
SCE1572_7458	sce_6242	4.00E-160	3.00E-163
SCE1572_746	sce_549	0.00E+00	0.00E+00
SCE1572_746	sce_3665	0.00E+00	0.00E+00
SCE1572_7460	sce_6244	4.00E-25	2.00E-30
SCE1572_7461	sce_6246	0.00E+00	0.00E+00
SCE1572_7462	sce_6247	4.00E-126	3.00E-126
SCE1572_7463	sce_6248	3.00E-150	1.00E-161
SCE1572_7464	sce_6249	1.00E-115	3.00E-112
SCE1572_7465	sce_6250	2.00E-107	1.00E-107
SCE1572_7467	sce_6263	4.00E-176	3.00E-176
SCE1572_7468	sce_6264	4.00E-23	5.00E-24
SCE1572_7469	sce_6265	8.00E-136	5.00E-139
SCE1572_7470	sce_6266	2.00E-118	8.00E-116
SCE1572_7472	sce_6268	8.00E-102	8.00E-102
SCE1572_7473	sce_6269	0.00E+00	0.00E+00
SCE1572_7474	sce_6270	5.00E-94	5.00E-94
SCE1572_7475	sce_6271	0.00E+00	0.00E+00
SCE1572_7477	sce_6273	3.00E-120	9.00E-136
SCE1572_7478	sce_6275	4.00E-67	5.00E-67
SCE1572_7479	sce_6276	0.00E+00	0.00E+00
SCE1572_748	sce_551	2.00E-75	8.00E-76
SCE1572_7480	sce_6278	4.00E-65	1.00E-66
SCE1572_7481	sce_6279	7.00E-12	5.00E-12

SCE1572_7482	sce_6280	8.00E-18	1.00E-24
SCE1572_7483	sce_6281	0.00E+00	0.00E+00
SCE1572_7483	sce_9625	0.00E+00	0.00E+00
SCE1572_7483	sce_5292	0.00E+00	0.00E+00
SCE1572_7485	sce_6306	0.00E+00	0.00E+00
SCE1572_7487	sce_615	0.00E+00	0.00E+00
SCE1572_749	sce_4659	4.00E-105	2.00E-102
SCE1572_7492	sce_7123	1.00E-42	8.00E-42
SCE1572_7493	sce_6083	3.00E-175	2.00E-159
SCE1572_75	sce_68	1.00E-79	1.00E-81
SCE1572_750	sce_552	0.00E+00	0.00E+00
SCE1572_7500	sce_6307	2.00E-60	1.00E-60
SCE1572_7501	sce_6309	9.00E-155	1.00E-154
SCE1572_7502	sce_6310	9.00E-92	9.00E-95
SCE1572_7503	sce_6313	9.00E-157	1.00E-156
SCE1572_7504	sce_6314	3.00E-40	1.00E-44
SCE1572_7505	sce_6315	3.00E-176	3.00E-177
SCE1572_7506	sce_8592	0.00E+00	0.00E+00
SCE1572_7506	sce_3937	0.00E+00	0.00E+00
SCE1572_7508	sce_3937	0.00E+00	0.00E+00
SCE1572_751	sce_553	1.00E-85	2.00E-84
SCE1572_7515	sce_6331	3.00E-50	5.00E-59
SCE1572_7516	sce_6332	4.00E-45	7.00E-41
SCE1572_7517	sce_6344	0.00E+00	0.00E+00
SCE1572_7518	sce_6345	0.00E+00	0.00E+00
SCE1572_7519	sce_6346	5.00E-81	5.00E-81
SCE1572_752	sce_554	7.00E-25	9.00E-29
SCE1572_7520	sce_6347	4.00E-83	1.00E-83
SCE1572_7524	sce_6348	1.00E-45	2.00E-45
SCE1572_7525	sce_6349	0.00E+00	0.00E+00
SCE1572_7527	sce_6351	6.00E-173	2.00E-177
SCE1572_7528	sce_6352	0.00E+00	0.00E+00
SCE1572_753	sce_555	7.00E-126	3.00E-126
SCE1572_7530	sce_6375	3.00E-149	9.00E-150
SCE1572_7531	sce_6373	1.00E-08	4.00E-09
SCE1572_7534	sce_6381	2.00E-21	1.00E-20
SCE1572_7535	sce_6382	0.00E+00	0.00E+00
SCE1572_7536	sce_6383	0.00E+00	0.00E+00
SCE1572_7537	sce_6401	5.00E-27	5.00E-28
SCE1572_7538	sce_6403	0.00E+00	0.00E+00
SCE1572_7539	sce_6404	2.00E-23	3.00E-32
SCE1572_754	sce_556	8.00E-168	8.00E-168
SCE1572_7540	sce_6405	3.00E-177	8.00E-176
SCE1572_7541	sce_6406	1.00E-50	7.00E-45
SCE1572_7542	sce_6407	1.00E-74	4.00E-82
SCE1572_7543	sce_6408	0.00E+00	0.00E+00
SCE1572_7544	sce_6409	7.00E-78	3.00E-95
SCE1572_7545	sce_9213	0.00E+00	0.00E+00
SCE1572_7546	sce_9212	0.00E+00	0.00E+00
SCE1572_7547	sce_9211	7.00E-158	5.00E-153
SCE1572_7548	sce_9210	5.00E-147	8.00E-154
SCE1572_7549	sce_5154	0.00E+00	0.00E+00
SCE1572_755	sce_557	4.00E-37	2.00E-33
SCE1572_7550	sce_5153	0.00E+00	0.00E+00
SCE1572_7552	sce_6410	2.00E-97	2.00E-77
SCE1572_7553	sce_6411	3.00E-66	3.00E-66
SCE1572_7554	sce_6412	1.00E-94	8.00E-106
SCE1572_7555	sce_6413	8.00E-45	2.00E-69
SCE1572_7557	sce_6415	3.00E-74	4.00E-82
SCE1572_7559	sce_3680	0.00E+00	0.00E+00
SCE1572_7560	sce_9660	3.00E-165	2.00E-178
SCE1572_7562	sce_1286	2.00E-30	5.00E-39
SCE1572_7563	sce_5995	0.00E+00	0.00E+00
SCE1572_7566	sce_6421	8.00E-62	2.00E-62
SCE1572_7567	sce_6422	3.00E-103	7.00E-68
SCE1572_7568	sce_6423	2.00E-107	3.00E-105
SCE1572_7569	sce_6424	0.00E+00	0.00E+00
SCE1572_757	sce_558	1.00E-121	1.00E-121
SCE1572_7571	sce_6425	0.00E+00	0.00E+00
SCE1572_7572	sce_6427	4.00E-29	6.00E-20
SCE1572_7573	sce_6428	2.00E-111	1.00E-109
SCE1572_7574	sce_6429	1.00E-96	5.00E-106
SCE1572_7575	sce_6430	0.00E+00	0.00E+00
SCE1572_7576	sce_6431	0.00E+00	0.00E+00
SCE1572_7577	sce_6432	4.00E-76	4.00E-76
SCE1572_7578	sce_6433	2.00E-50	2.00E-50

SCE1572_7579	sce_6435	0.00E+00	0.00E+00
SCE1572_758	sce_559	3.00E-93	1.00E-103
SCE1572_7580	sce_6436	6.00E-132	1.00E-145
SCE1572_7581	sce_6437	0.00E+00	0.00E+00
SCE1572_7581	sce_831	0.00E+00	0.00E+00
SCE1572_7582	sce_6438	2.00E-87	2.00E-94
SCE1572_7583	sce_6439	2.00E-149	2.00E-156
SCE1572_7584	sce_6440	2.00E-16	2.00E-16
SCE1572_7585	sce_6441	3.00E-46	3.00E-46
SCE1572_7586	sce_6442	9.00E-53	6.00E-51
SCE1572_7587	sce_6443	3.00E-161	3.00E-159
SCE1572_7588	sce_6444	5.00E-78	8.00E-79
SCE1572_7589	sce_6448	1.00E-138	1.00E-138
SCE1572_759	sce_560	4.00E-93	4.00E-93
SCE1572_7590	sce_6449	0.00E+00	0.00E+00
SCE1572_7591	sce_6450	0.00E+00	0.00E+00
SCE1572_7592	sce_6451	0.00E+00	0.00E+00
SCE1572_7593	sce_6452	0.00E+00	0.00E+00
SCE1572_7594	sce_6453	2.00E-43	2.00E-43
SCE1572_7595	sce_6454	6.00E-165	4.00E-165
SCE1572_7596	sce_6455	0.00E+00	8.00E-154
SCE1572_7597	sce_6456	7.00E-73	7.00E-73
SCE1572_7599	sce_6458	3.00E-51	4.00E-52
SCE1572_760	sce_561	0.00E+00	0.00E+00
SCE1572_7600	sce_6461	9.00E-124	4.00E-142
SCE1572_7601	sce_6462	6.00E-178	5.00E-176
SCE1572_7602	sce_6463	0.00E+00	0.00E+00
SCE1572_7603	sce_6464	0.00E+00	0.00E+00
SCE1572_7604	sce_6465	1.00E-64	6.00E-65
SCE1572_7606	sce_9946	1.00E-160	1.00E-175
SCE1572_7607	sce_6467	5.00E-109	6.00E-107
SCE1572_7609	sce_6470	4.00E-56	2.00E-49
SCE1572_761	sce_562	1.00E-95	3.00E-101
SCE1572_7610	sce_6471	4.00E-117	4.00E-117
SCE1572_7611	sce_6472	1.00E-61	2.00E-61
SCE1572_7612	sce_6473	0.00E+00	0.00E+00
SCE1572_7613	sce_6475	8.00E-88	6.00E-84
SCE1572_7614	sce_6476	2.00E-114	4.00E-117
SCE1572_7617	sce_6477	2.00E-134	2.00E-134
SCE1572_7618	sce_6478	5.00E-120	5.00E-120
SCE1572_7619	sce_6479	3.00E-157	3.00E-157
SCE1572_762	sce_92	0.00E+00	0.00E+00
SCE1572_7620	sce_6480	1.00E-149	9.00E-158
SCE1572_7621	sce_6481	1.00E-139	3.00E-127
SCE1572_7622	sce_6482	0.00E+00	0.00E+00
SCE1572_7623	sce_6483	1.00E-151	9.00E-144
SCE1572_7624	sce_6484	0.00E+00	0.00E+00
SCE1572_7625	sce_6486	0.00E+00	0.00E+00
SCE1572_7626	sce_6487	3.00E-78	1.00E-77
SCE1572_7627	sce_7919	1.00E-09	6.00E-10
SCE1572_7628	sce_6488	6.00E-139	4.00E-140
SCE1572_7629	sce_6489	1.00E-126	1.00E-126
SCE1572_7630	sce_6491	2.00E-56	8.00E-50
SCE1572_7631	sce_6492	2.00E-53	4.00E-56
SCE1572_7632	sce_6493	0.00E+00	0.00E+00
SCE1572_7634	sce_6494	3.00E-75	6.00E-74
SCE1572_7635	sce_6495	0.00E+00	0.00E+00
SCE1572_7636	sce_6496	1.00E-71	1.00E-81
SCE1572_7637	sce_6497	0.00E+00	0.00E+00
SCE1572_7638	sce_6498	3.00E-108	6.00E-116
SCE1572_7639	sce_6499	0.00E+00	0.00E+00
SCE1572_764	sce_563	0.00E+00	0.00E+00
SCE1572_7640	sce_6500	2.00E-72	9.00E-71
SCE1572_7641	sce_6501	0.00E+00	0.00E+00
SCE1572_7641	sce_736	0.00E+00	0.00E+00
SCE1572_7643	sce_6502	0.00E+00	1.00E-179
SCE1572_7644	sce_6504	0.00E+00	0.00E+00
SCE1572_7645	sce_6505	0.00E+00	0.00E+00
SCE1572_7646	sce_6506	0.00E+00	0.00E+00
SCE1572_7647	sce_9653	0.00E+00	0.00E+00
SCE1572_7648	sce_2926	0.00E+00	0.00E+00
SCE1572_765	sce_2573	3.00E-26	2.00E-26
SCE1572_7654	sce_8663	3.00E-12	7.00E-10
SCE1572_7655	sce_8662	1.00E-41	2.00E-24
SCE1572_7659	sce_9782	8.00E-45	2.00E-45
SCE1572_7662	sce_9807	1.00E-60	9.00E-69

SCE1572_7665	sce_1677	3.00E-127	2.00E-126
SCE1572_7667	sce_2109	2.00E-12	6.00E-13
SCE1572_767	sce_565	4.00E-147	7.00E-133
SCE1572_7677	sce_6508	0.00E+00	0.00E+00
SCE1572_7678	sce_6509	2.00E-55	2.00E-55
SCE1572_7679	sce_6510	5.00E-81	4.00E-80
SCE1572_768	sce_566	5.00E-180	1.00E-163
SCE1572_7680	sce_6511	3.00E-127	1.00E-134
SCE1572_7681	sce_6512	7.00E-129	3.00E-147
SCE1572_7682	sce_6513	1.00E-76	4.00E-82
SCE1572_7683	sce_6514	0.00E+00	0.00E+00
SCE1572_7685	sce_6515	5.00E-34	4.00E-63
SCE1572_7687	sce_6516	3.00E-117	5.00E-126
SCE1572_769	sce_567	1.00E-52	7.00E-56
SCE1572_7691	sce_6517	3.00E-71	7.00E-70
SCE1572_7692	sce_6518	6.00E-160	3.00E-173
SCE1572_7693	sce_6519	4.00E-116	3.00E-120
SCE1572_7694	sce_6520	0.00E+00	0.00E+00
SCE1572_7695	sce_6521	7.00E-83	2.00E-101
SCE1572_7696	sce_6522	2.00E-117	2.00E-120
SCE1572_7697	sce_6523	4.00E-176	1.00E-172
SCE1572_7698	sce_6524	6.00E-145	3.00E-150
SCE1572_7699	sce_6525	1.00E-124	7.00E-125
SCE1572_77	sce_69	1.00E-169	9.00E-177
SCE1572_770	sce_568	8.00E-101	8.00E-101
SCE1572_7700	sce_6526	2.00E-165	7.00E-159
SCE1572_7701	sce_6527	5.00E-27	1.00E-29
SCE1572_7702	sce_6528	0.00E+00	0.00E+00
SCE1572_7703	sce_6536	4.00E-113	2.00E-121
SCE1572_7704	sce_6537	1.00E-37	3.00E-34
SCE1572_7706	sce_6538	0.00E+00	0.00E+00
SCE1572_7707	sce_6539	0.00E+00	0.00E+00
SCE1572_7709	sce_6541	0.00E+00	0.00E+00
SCE1572_7709	sce_6114	0.00E+00	0.00E+00
SCE1572_771	sce_569	1.00E-157	2.00E-139
SCE1572_7710	sce_6542	2.00E-84	2.00E-88
SCE1572_7711	sce_6543	1.00E-71	3.00E-70
SCE1572_7712	sce_6544	3.00E-120	5.00E-153
SCE1572_7713	sce_6545	1.00E-63	5.00E-62
SCE1572_7718	sce_6546	1.00E-139	1.00E-162
SCE1572_7719	sce_6547	4.00E-97	1.00E-99
SCE1572_772	sce_570	4.00E-129	2.00E-135
SCE1572_7720	sce_6548	1.00E-139	8.00E-142
SCE1572_7723	sce_6549	4.00E-153	3.00E-147
SCE1572_7724	sce_6550	0.00E+00	0.00E+00
SCE1572_7725	sce_6551	0.00E+00	0.00E+00
SCE1572_7726	sce_6552	2.00E-59	3.00E-59
SCE1572_7727	sce_6553	0.00E+00	0.00E+00
SCE1572_7729	sce_6554	1.00E-76	1.00E-85
SCE1572_773	sce_571	2.00E-97	5.00E-100
SCE1572_7730	sce_6555	3.00E-102	1.00E-99
SCE1572_7731	sce_6556	9.00E-51	9.00E-51
SCE1572_7732	sce_6557	5.00E-82	2.00E-67
SCE1572_7733	sce_6558	8.00E-111	4.00E-102
SCE1572_7735	sce_6560	1.00E-51	1.00E-51
SCE1572_7736	sce_6561	4.00E-107	2.00E-113
SCE1572_7737	sce_6562	6.00E-51	2.00E-64
SCE1572_7738	sce_6563	2.00E-130	0.00E+00
SCE1572_774	sce_572	0.00E+00	0.00E+00
SCE1572_7740	sce_6564	0.00E+00	0.00E+00
SCE1572_7741	sce_6565	3.00E-162	1.00E-158
SCE1572_7742	sce_6566	0.00E+00	0.00E+00
SCE1572_7743	sce_6567	9.00E-138	4.00E-135
SCE1572_7744	sce_6568	1.00E-155	1.00E-155
SCE1572_7745	sce_6569	6.00E-51	2.00E-48
SCE1572_7746	sce_6571	0.00E+00	0.00E+00
SCE1572_7746	sce_8649	0.00E+00	0.00E+00
SCE1572_7747	sce_6572	5.00E-139	5.00E-139
SCE1572_775	sce_573	6.00E-54	4.00E-54
SCE1572_7758	sce_6574	3.00E-76	1.00E-89
SCE1572_7759	sce_6577	0.00E+00	0.00E+00
SCE1572_7760	sce_6578	0.00E+00	0.00E+00
SCE1572_7761	sce_6579	1.00E-51	2.00E-50
SCE1572_7765	sce_6583	1.00E-46	1.00E-46
SCE1572_7766	sce_6584	0.00E+00	0.00E+00
SCE1572_7768	sce_6585	0.00E+00	0.00E+00

SCE1572_7768	sce_900	0.00E+00	0.00E+00
SCE1572_7769	sce_6586	3.00E-134	3.00E-144
SCE1572_777	sce_574	6.00E-98	1.00E-96
SCE1572_7770	sce_6587	0.00E+00	0.00E+00
SCE1572_7771	sce_6588	1.00E-127	1.00E-115
SCE1572_7772	sce_6589	2.00E-47	7.00E-41
SCE1572_7773	sce_6590	1.00E-51	1.00E-51
SCE1572_7777	sce_512	1.00E-75	8.00E-77
SCE1572_778	sce_575	1.00E-150	3.00E-144
SCE1572_7784	sce_518	5.00E-155	7.00E-144
SCE1572_779	sce_576	0.00E+00	0.00E+00
SCE1572_7790	sce_6596	4.00E-19	3.00E-18
SCE1572_7791	sce_6597	3.00E-114	8.00E-116
SCE1572_7792	sce_6598	8.00E-74	8.00E-74
SCE1572_7793	sce_6599	0.00E+00	0.00E+00
SCE1572_7794	sce_6600	4.00E-64	8.00E-77
SCE1572_7795	sce_6601	8.00E-114	2.00E-125
SCE1572_7798	sce_6602	2.00E-08	6.00E-16
SCE1572_78	sce_70	2.00E-58	4.00E-59
SCE1572_7800	sce_6605	0.00E+00	0.00E+00
SCE1572_7801	sce_6606	0.00E+00	0.00E+00
SCE1572_7802	sce_6607	7.00E-106	4.00E-108
SCE1572_7803	sce_6608	8.00E-16	3.00E-09
SCE1572_7804	sce_6609	0.00E+00	0.00E+00
SCE1572_7805	sce_6610	0.00E+00	0.00E+00
SCE1572_7806	sce_6611	1.00E-164	5.00E-165
SCE1572_7807	sce_6612	0.00E+00	0.00E+00
SCE1572_7808	sce_6613	0.00E+00	0.00E+00
SCE1572_7810	sce_6614	1.00E-58	5.00E-58
SCE1572_7811	sce_6615	3.00E-108	3.00E-108
SCE1572_7812	sce_6616	2.00E-103	2.00E-103
SCE1572_7814	sce_6617	3.00E-47	2.00E-47
SCE1572_7815	sce_6618	0.00E+00	0.00E+00
SCE1572_7815	sce_3121	0.00E+00	0.00E+00
SCE1572_7816	sce_6619	3.00E-89	3.00E-89
SCE1572_7817	sce_6620	3.00E-96	3.00E-96
SCE1572_7818	sce_6621	5.00E-107	6.00E-123
SCE1572_7819	sce_6622	0.00E+00	0.00E+00
SCE1572_782	sce_577	5.00E-99	4.00E-97
SCE1572_7820	sce_6623	3.00E-141	1.00E-139
SCE1572_7821	sce_6624	1.00E-85	3.00E-92
SCE1572_7822	sce_6625	8.00E-162	5.00E-162
SCE1572_7823	sce_6626	8.00E-56	5.00E-56
SCE1572_7824	sce_6627	3.00E-169	0.00E+00
SCE1572_7825	sce_6628	6.00E-115	1.00E-114
SCE1572_7826	sce_6629	0.00E+00	0.00E+00
SCE1572_7827	sce_6630	9.00E-49	8.00E-38
SCE1572_7828	sce_6631	6.00E-151	6.00E-151
SCE1572_7829	sce_6632	1.00E-98	5.00E-100
SCE1572_783	sce_578	0.00E+00	0.00E+00
SCE1572_7830	sce_6633	0.00E+00	0.00E+00
SCE1572_7831	sce_6634	1.00E-154	1.00E-154
SCE1572_7832	sce_6635	5.00E-82	5.00E-82
SCE1572_7833	sce_6636	0.00E+00	0.00E+00
SCE1572_7834	sce_6637	1.00E-08	4.00E-08
SCE1572_7835	sce_6638	5.00E-92	5.00E-92
SCE1572_7836	sce_6639	5.00E-159	1.00E-151
SCE1572_7837	sce_6640	4.00E-35	4.00E-35
SCE1572_7838	sce_6641	4.00E-127	9.00E-102
SCE1572_7839	sce_6642	3.00E-87	7.00E-71
SCE1572_784	sce_579	4.00E-149	4.00E-135
SCE1572_7840	sce_6644	0.00E+00	0.00E+00
SCE1572_7841	sce_6645	3.00E-77	6.00E-77
SCE1572_7842	sce_6646	1.00E-94	1.00E-97
SCE1572_7843	sce_6647	0.00E+00	0.00E+00
SCE1572_7844	sce_6648	5.00E-92	6.00E-92
SCE1572_7845	sce_6649	3.00E-164	1.00E-159
SCE1572_7846	sce_6650	3.00E-36	5.00E-26
SCE1572_7847	sce_6651	3.00E-39	3.00E-39
SCE1572_7848	sce_6652	0.00E+00	0.00E+00
SCE1572_7849	sce_6653	0.00E+00	0.00E+00
SCE1572_785	sce_581	1.00E-165	2.00E-176
SCE1572_7850	sce_6654	0.00E+00	0.00E+00
SCE1572_7851	sce_6655	1.00E-98	1.00E-98
SCE1572_7853	sce_6656	3.00E-112	3.00E-112
SCE1572_7854	sce_6657	2.00E-109	1.00E-98

SCE1572_7855	sce_6658	2.00E-79	4.00E-72
SCE1572_7856	sce_6659	0.00E+00	0.00E+00
SCE1572_7857	sce_6660	4.00E-166	2.00E-158
SCE1572_7858	sce_6661	0.00E+00	0.00E+00
SCE1572_7862	sce_6662	3.00E-170	1.00E-174
SCE1572_7863	sce_6663	7.00E-50	7.00E-50
SCE1572_7864	sce_6664	3.00E-100	3.00E-100
SCE1572_7865	sce_6667	1.00E-151	1.00E-165
SCE1572_7866	sce_6668	0.00E+00	7.00E-180
SCE1572_7867	sce_6669	1.00E-47	1.00E-50
SCE1572_7868	sce_6670	5.00E-154	3.00E-134
SCE1572_7869	sce_6671	2.00E-161	2.00E-160
SCE1572_787	sce_582	0.00E+00	0.00E+00
SCE1572_7870	sce_6672	5.00E-87	3.00E-90
SCE1572_7871	sce_6673	4.00E-123	3.00E-134
SCE1572_7872	sce_6674	0.00E+00	0.00E+00
SCE1572_7873	sce_6675	1.00E-82	1.00E-82
SCE1572_7874	sce_6676	2.00E-52	3.00E-54
SCE1572_7875	sce_6678	6.00E-125	3.00E-129
SCE1572_7876	sce_6679	2.00E-127	2.00E-127
SCE1572_7877	sce_6680	7.00E-127	7.00E-127
SCE1572_7878	sce_6681	3.00E-131	4.00E-137
SCE1572_7879	sce_6682	0.00E+00	0.00E+00
SCE1572_788	sce_583	0.00E+00	0.00E+00
SCE1572_7880	sce_6684	3.00E-42	2.00E-42
SCE1572_7881	sce_6685	0.00E+00	0.00E+00
SCE1572_7883	sce_6688	1.00E-96	1.00E-99
SCE1572_7884	sce_6689	0.00E+00	0.00E+00
SCE1572_7884	sce_5475	0.00E+00	0.00E+00
SCE1572_7885	sce_5474	0.00E+00	0.00E+00
SCE1572_7885	sce_6690	0.00E+00	0.00E+00
SCE1572_7886	sce_6691	5.00E-48	5.00E-48
SCE1572_7887	sce_6692	0.00E+00	0.00E+00
SCE1572_7888	sce_6693	0.00E+00	0.00E+00
SCE1572_7889	sce_6694	2.00E-137	2.00E-138
SCE1572_7890	sce_6695	7.00E-55	5.00E-40
SCE1572_7893	sce_2085	1.00E-14	9.00E-15
SCE1572_7896	sce_6697	3.00E-41	3.00E-41
SCE1572_7898	sce_6698	6.00E-102	2.00E-120
SCE1572_7902	sce_6705	6.00E-72	2.00E-72
SCE1572_7903	sce_6706	9.00E-70	2.00E-71
SCE1572_7904	sce_6707	6.00E-115	2.00E-114
SCE1572_7907	sce_4677	1.00E-108	2.00E-108
SCE1572_791	sce_584	0.00E+00	0.00E+00
SCE1572_7910	sce_4620	3.00E-12	6.00E-12
SCE1572_7911	sce_1247	4.00E-70	1.00E-68
SCE1572_7912	sce_7656	2.00E-32	6.00E-29
SCE1572_7914	sce_6714	1.00E-126	6.00E-128
SCE1572_7916	sce_6716	0.00E+00	0.00E+00
SCE1572_7917	sce_6717	8.00E-153	1.00E-161
SCE1572_7918	sce_6718	0.00E+00	0.00E+00
SCE1572_7919	sce_6718	0.00E+00	0.00E+00
SCE1572_792	sce_585	3.00E-53	7.00E-48
SCE1572_7920	sce_6718	0.00E+00	0.00E+00
SCE1572_7922	sce_6719	1.00E-168	1.00E-160
SCE1572_7923	sce_6720	3.00E-55	1.00E-49
SCE1572_7924	sce_6721	0.00E+00	0.00E+00
SCE1572_7925	sce_6722	7.00E-42	4.00E-56
SCE1572_7927	sce_6723	0.00E+00	0.00E+00
SCE1572_7928	sce_6724	0.00E+00	0.00E+00
SCE1572_7929	sce_6725	3.00E-109	3.00E-109
SCE1572_793	sce_586	3.00E-108	3.00E-108
SCE1572_7930	sce_6726	3.00E-158	8.00E-158
SCE1572_7931	sce_6729	0.00E+00	0.00E+00
SCE1572_7932	sce_6730	0.00E+00	0.00E+00
SCE1572_7935	sce_6733	0.00E+00	0.00E+00
SCE1572_7936	sce_6734	1.00E-112	6.00E-126
SCE1572_7937	sce_6735	1.00E-103	2.00E-130
SCE1572_7938	sce_6736	4.00E-160	6.00E-162
SCE1572_7939	sce_6737	9.00E-120	3.00E-103
SCE1572_794	sce_587	6.00E-113	6.00E-113
SCE1572_7940	sce_6738	0.00E+00	0.00E+00
SCE1572_7941	sce_6739	5.00E-122	9.00E-123
SCE1572_7942	sce_6742	0.00E+00	0.00E+00
SCE1572_7943	sce_6743	9.00E-104	9.00E-104
SCE1572_7944	sce_6744	1.00E-58	1.00E-63

SCE1572_7949	sce_6745	2.00E-110	1.00E-121
SCE1572_795	sce_588	0.00E+00	0.00E+00
SCE1572_7950	sce_6746	4.00E-172	9.00E-179
SCE1572_7951	sce_6747	1.00E-82	1.00E-82
SCE1572_7952	sce_6748	0.00E+00	0.00E+00
SCE1572_7953	sce_6750	0.00E+00	0.00E+00
SCE1572_7955	sce_6751	2.00E-157	9.00E-160
SCE1572_7956	sce_6752	2.00E-118	5.00E-110
SCE1572_7957	sce_6753	9.00E-89	1.00E-100
SCE1572_7958	sce_4490	1.00E-74	1.00E-74
SCE1572_7959	sce_6754	2.00E-59	2.00E-59
SCE1572_796	sce_589	0.00E+00	0.00E+00
SCE1572_7960	sce_2029	9.00E-123	1.00E-122
SCE1572_7962	sce_6756	0.00E+00	0.00E+00
SCE1572_7963	sce_6757	0.00E+00	0.00E+00
SCE1572_7965	sce_6764	3.00E-33	2.00E-38
SCE1572_7966	sce_6765	6.00E-65	8.00E-63
SCE1572_7968	sce_6766	8.00E-111	3.00E-98
SCE1572_797	sce_590	0.00E+00	0.00E+00
SCE1572_7970	sce_6769	2.00E-126	1.00E-125
SCE1572_7971	sce_6770	6.00E-76	6.00E-86
SCE1572_7972	sce_6771	1.00E-76	5.00E-90
SCE1572_7973	sce_6772	1.00E-49	1.00E-51
SCE1572_7974	sce_6773	3.00E-150	6.00E-153
SCE1572_7975	sce_7456	5.00E-94	2.00E-97
SCE1572_7976	sce_7452	7.00E-29	1.00E-29
SCE1572_7978	sce_9166	1.00E-141	3.00E-145
SCE1572_798	sce_591	7.00E-125	7.00E-125
SCE1572_7984	sce_6787	2.00E-155	3.00E-151
SCE1572_7985	sce_6788	0.00E+00	0.00E+00
SCE1572_7986	sce_6789	1.00E-109	5.00E-106
SCE1572_7987	sce_6790	2.00E-99	3.00E-111
SCE1572_799	sce_592	0.00E+00	0.00E+00
SCE1572_7991	sce_6792	8.00E-102	3.00E-102
SCE1572_7993	sce_6793	6.00E-155	9.00E-179
SCE1572_7997	sce_6225	2.00E-118	5.00E-99
SCE1572_7998	sce_6226	0.00E+00	0.00E+00
SCE1572_8	sce_8	9.00E-170	1.00E-169
SCE1572_8001	sce_9566	0.00E+00	0.00E+00
SCE1572_8003	sce_9566	0.00E+00	0.00E+00
SCE1572_8003	sce_7530	0.00E+00	0.00E+00
SCE1572_8005	sce_6795	0.00E+00	0.00E+00
SCE1572_8007	sce_6796	2.00E-139	4.00E-138
SCE1572_8008	sce_6797	0.00E+00	0.00E+00
SCE1572_8009	sce_6798	3.00E-142	3.00E-142
SCE1572_801	sce_593	1.00E-127	2.00E-118
SCE1572_8010	sce_6800	2.00E-117	8.00E-119
SCE1572_8011	sce_6801	2.00E-141	7.00E-156
SCE1572_8013	sce_6802	0.00E+00	0.00E+00
SCE1572_8014	sce_6803	0.00E+00	0.00E+00
SCE1572_8015	sce_6804	3.00E-46	6.00E-46
SCE1572_8016	sce_6805	9.00E-22	2.00E-24
SCE1572_8018	sce_6807	1.00E-66	7.00E-67
SCE1572_8019	sce_1676	0.00E+00	0.00E+00
SCE1572_802	sce_594	0.00E+00	0.00E+00
SCE1572_8020	sce_6833	0.00E+00	0.00E+00
SCE1572_8020	sce_6827	0.00E+00	0.00E+00
SCE1572_8020	sce_6228	0.00E+00	0.00E+00
SCE1572_8020	sce_10159	0.00E+00	0.00E+00
SCE1572_8020	sce_7513	0.00E+00	0.00E+00
SCE1572_8020	sce_7408	0.00E+00	0.00E+00
SCE1572_8020	sce_6876	0.00E+00	0.00E+00
SCE1572_8020	sce_6836	0.00E+00	0.00E+00
SCE1572_8020	sce_2543	0.00E+00	0.00E+00
SCE1572_8020	sce_7036	0.00E+00	0.00E+00
SCE1572_8021	sce_7413	3.00E-69	3.00E-69
SCE1572_8022	sce_7414	3.00E-39	3.00E-45
SCE1572_8030	sce_304	1.00E-163	3.00E-165
SCE1572_8034	sce_9170	6.00E-53	1.00E-52
SCE1572_8035	sce_9171	2.00E-137	5.00E-142
SCE1572_8039	sce_9793	2.00E-70	3.00E-61
SCE1572_8040	sce_10359	4.00E-88	3.00E-86
SCE1572_8041	sce_6872	8.00E-80	2.00E-91
SCE1572_8042	sce_6877	6.00E-45	3.00E-49
SCE1572_8043	sce_6809	4.00E-12	4.00E-12
SCE1572_8068	sce_7404	0.00E+00	0.00E+00

SCE1572_8072	sce_9803	1.00E-36	3.00E-37
SCE1572_8073	sce_9804	2.00E-34	2.00E-29
SCE1572_8074	sce_1036	4.00E-18	2.00E-16
SCE1572_8082	sce_6847	5.00E-13	5.00E-13
SCE1572_8083	sce_6849	2.00E-31	7.00E-38
SCE1572_8089	sce_5122	6.00E-149	1.00E-160
SCE1572_809	sce_225	0.00E+00	0.00E+00
SCE1572_809	sce_6235	0.00E+00	0.00E+00
SCE1572_809	sce_2161	0.00E+00	0.00E+00
SCE1572_809	sce_8357	0.00E+00	0.00E+00
SCE1572_809	sce_5276	0.00E+00	0.00E+00
SCE1572_8090	sce_5121	0.00E+00	0.00E+00
SCE1572_8091	sce_5118	2.00E-50	8.00E-50
SCE1572_8092	sce_5117	4.00E-45	1.00E-51
SCE1572_8093	sce_5116	0.00E+00	0.00E+00
SCE1572_8094	sce_5115	3.00E-133	1.00E-135
SCE1572_8095	sce_5114	6.00E-125	6.00E-125
SCE1572_8096	sce_5113	6.00E-72	6.00E-72
SCE1572_8097	sce_5112	0.00E+00	0.00E+00
SCE1572_8098	sce_9043	6.00E-26	9.00E-34
SCE1572_8099	sce_9044	0.00E+00	0.00E+00
SCE1572_810	sce_6107	0.00E+00	0.00E+00
SCE1572_810	sce_3917	0.00E+00	0.00E+00
SCE1572_810	sce_6093	0.00E+00	0.00E+00
SCE1572_810	sce_6094	0.00E+00	0.00E+00
SCE1572_810	sce_6092	0.00E+00	0.00E+00
SCE1572_810	sce_7661	0.00E+00	0.00E+00
SCE1572_810	sce_6095	0.00E+00	0.00E+00
SCE1572_810	sce_2065	0.00E+00	0.00E+00
SCE1572_810	sce_8618	0.00E+00	0.00E+00
SCE1572_8100	sce_5110	0.00E+00	0.00E+00
SCE1572_8115	sce_5109	2.00E-100	2.00E-101
SCE1572_8126	sce_5107	0.00E+00	0.00E+00
SCE1572_8127	sce_9045	0.00E+00	0.00E+00
SCE1572_8133	sce_5552	2.00E-23	7.00E-23
SCE1572_8137	sce_861	5.00E-10	1.00E-09
SCE1572_8146	sce_3042	4.00E-52	6.00E-47
SCE1572_8176	sce_5106	6.00E-81	1.00E-93
SCE1572_8190	sce_4070	4.00E-147	2.00E-147
SCE1572_8203	sce_6891	7.00E-28	3.00E-27
SCE1572_8206	sce_8351	3.00E-130	2.00E-143
SCE1572_8207	sce_8350	1.00E-56	2.00E-60
SCE1572_8208	sce_8348	5.00E-17	7.00E-18
SCE1572_8209	sce_8347	0.00E+00	0.00E+00
SCE1572_8209	sce_4610	0.00E+00	0.00E+00
SCE1572_8210	sce_8346	6.00E-110	7.00E-117
SCE1572_8211	sce_8345	0.00E+00	0.00E+00
SCE1572_8212	sce_8344	4.00E-54	8.00E-43
SCE1572_8213	sce_8343	0.00E+00	0.00E+00
SCE1572_8214	sce_8342	0.00E+00	0.00E+00
SCE1572_8220	sce_4907	3.00E-52	1.00E-48
SCE1572_8222	sce_7070	5.00E-169	6.00E-175
SCE1572_8225	sce_6785	6.00E-86	1.00E-96
SCE1572_8226	sce_6895	1.00E-87	4.00E-101
SCE1572_8227	sce_6896	3.00E-125	1.00E-123
SCE1572_8228	sce_6897	0.00E+00	0.00E+00
SCE1572_8229	sce_6899	2.00E-121	2.00E-142
SCE1572_823	sce_631	4.00E-68	8.00E-69
SCE1572_8230	sce_6900	5.00E-156	2.00E-167
SCE1572_8231	sce_6901	0.00E+00	0.00E+00
SCE1572_8232	sce_6902	2.00E-78	5.00E-74
SCE1572_8233	sce_6905	1.00E-175	1.00E-167
SCE1572_8235	sce_6906	6.00E-36	2.00E-29
SCE1572_8236	sce_6907	3.00E-74	2.00E-74
SCE1572_8237	sce_6908	1.00E-66	5.00E-62
SCE1572_8238	sce_6909	1.00E-96	4.00E-97
SCE1572_8239	sce_6910	5.00E-132	7.00E-136
SCE1572_824	sce_5835	0.00E+00	0.00E+00
SCE1572_824	sce_10204	0.00E+00	0.00E+00
SCE1572_824	sce_388	0.00E+00	0.00E+00
SCE1572_824	sce_7640	0.00E+00	0.00E+00
SCE1572_824	sce_4513	0.00E+00	0.00E+00
SCE1572_824	sce_3085	0.00E+00	0.00E+00
SCE1572_824	sce_7109	0.00E+00	0.00E+00
SCE1572_824	sce_3003	0.00E+00	0.00E+00
SCE1572_824	sce_9557	0.00E+00	0.00E+00

SCE1572_824	sce_6535	0.00E+00	0.00E+00
SCE1572_824	sce_6092	0.00E+00	0.00E+00
SCE1572_824	sce_7661	0.00E+00	0.00E+00
SCE1572_8240	sce_6911	8.00E-103	3.00E-113
SCE1572_8241	sce_6912	0.00E+00	0.00E+00
SCE1572_8242	sce_6913	4.00E-44	2.00E-39
SCE1572_8243	sce_6914	1.00E-169	1.00E-169
SCE1572_8244	sce_6915	2.00E-95	2.00E-95
SCE1572_8245	sce_6916	0.00E+00	0.00E+00
SCE1572_8246	sce_6917	1.00E-73	3.00E-73
SCE1572_8247	sce_6918	4.00E-107	2.00E-102
SCE1572_8248	sce_6919	4.00E-77	2.00E-85
SCE1572_8249	sce_6920	0.00E+00	0.00E+00
SCE1572_825	sce_621	0.00E+00	0.00E+00
SCE1572_8250	sce_6921	0.00E+00	0.00E+00
SCE1572_8251	sce_6922	2.00E-45	5.00E-71
SCE1572_8252	sce_6923	0.00E+00	0.00E+00
SCE1572_8253	sce_6924	3.00E-157	0.00E+00
SCE1572_8254	sce_6925	3.00E-133	1.00E-139
SCE1572_8255	sce_6926	0.00E+00	0.00E+00
SCE1572_8256	sce_6927	3.00E-79	9.00E-75
SCE1572_8257	sce_6928	3.00E-108	7.00E-108
SCE1572_8258	sce_6929	2.00E-118	2.00E-118
SCE1572_8259	sce_6930	0.00E+00	0.00E+00
SCE1572_826	sce_622	0.00E+00	0.00E+00
SCE1572_8260	sce_6931	4.00E-136	2.00E-154
SCE1572_8261	sce_6932	2.00E-110	1.00E-124
SCE1572_8262	sce_6933	0.00E+00	0.00E+00
SCE1572_8263	sce_6934	0.00E+00	0.00E+00
SCE1572_8264	sce_6935	2.00E-104	1.00E-103
SCE1572_8265	sce_6936	2.00E-69	8.00E-87
SCE1572_8266	sce_6937	0.00E+00	0.00E+00
SCE1572_8268	sce_6317	3.00E-11	9.00E-11
SCE1572_8269	sce_6318	0.00E+00	0.00E+00
SCE1572_827	sce_623	4.00E-106	1.00E-112
SCE1572_8270	sce_6319	0.00E+00	0.00E+00
SCE1572_8271	sce_6940	1.00E-136	8.00E-111
SCE1572_8272	sce_6941	3.00E-88	3.00E-89
SCE1572_8273	sce_6942	1.00E-98	1.00E-98
SCE1572_8274	sce_7920	4.00E-19	4.00E-33
SCE1572_8275	sce_6944	0.00E+00	5.00E-107
SCE1572_8276	sce_6945	6.00E-114	9.00E-113
SCE1572_8277	sce_6946	6.00E-87	8.00E-85
SCE1572_8278	sce_6947	3.00E-149	4.00E-158
SCE1572_8279	sce_6948	3.00E-72	1.00E-75
SCE1572_828	sce_624	4.00E-42	1.00E-43
SCE1572_8280	sce_6949	0.00E+00	0.00E+00
SCE1572_8281	sce_6950	2.00E-169	4.00E-169
SCE1572_8282	sce_6951	0.00E+00	0.00E+00
SCE1572_8283	sce_6316	1.00E-49	5.00E-43
SCE1572_8284	sce_611	3.00E-07	8.00E-13
SCE1572_8285	sce_613	2.00E-54	6.00E-58
SCE1572_8286	sce_6953	9.00E-84	1.00E-83
SCE1572_8287	sce_6954	1.00E-161	1.00E-166
SCE1572_8289	sce_6053	3.00E-25	5.00E-31
SCE1572_829	sce_625	3.00E-83	5.00E-86
SCE1572_8291	sce_9526	7.00E-12	3.00E-13
SCE1572_8294	sce_6979	7.00E-91	6.00E-95
SCE1572_8295	sce_6980	4.00E-115	4.00E-115
SCE1572_8296	sce_6981	2.00E-105	4.00E-98
SCE1572_8297	sce_6982	0.00E+00	0.00E+00
SCE1572_8298	sce_6983	0.00E+00	0.00E+00
SCE1572_830	sce_626	7.00E-66	2.00E-67
SCE1572_8301	sce_6985	0.00E+00	0.00E+00
SCE1572_8302	sce_6986	7.00E-83	1.00E-73
SCE1572_8303	sce_6987	1.00E-43	1.00E-53
SCE1572_8304	sce_6988	3.00E-64	1.00E-68
SCE1572_8305	sce_6989	2.00E-106	2.00E-106
SCE1572_8306	sce_6990	4.00E-26	4.00E-26
SCE1572_8307	sce_1129	7.00E-16	1.00E-16
SCE1572_8309	sce_6991	1.00E-124	5.00E-150
SCE1572_8310	sce_6992	4.00E-159	2.00E-160
SCE1572_8311	sce_6993	0.00E+00	1.00E-104
SCE1572_8312	sce_6994	4.00E-93	4.00E-93
SCE1572_8313	sce_6995	1.00E-172	2.00E-169
SCE1572_8314	sce_6996	0.00E+00	0.00E+00

SCE1572_8315	sce_6997	2.00E-60	2.00E-71
SCE1572_8316	sce_6998	2.00E-165	4.00E-173
SCE1572_8317	sce_6999	2.00E-111	2.00E-120
SCE1572_8318	sce_7000	7.00E-156	4.00E-133
SCE1572_8319	sce_7002	0.00E+00	0.00E+00
SCE1572_8320	sce_7003	3.00E-47	2.00E-61
SCE1572_8321	sce_7004	0.00E+00	0.00E+00
SCE1572_8322	sce_7006	1.00E-105	4.00E-105
SCE1572_8323	sce_7007	3.00E-118	3.00E-118
SCE1572_8324	sce_7008	2.00E-127	7.00E-118
SCE1572_8325	sce_7009	0.00E+00	1.00E-166
SCE1572_8326	sce_7010	0.00E+00	0.00E+00
SCE1572_8327	sce_7011	0.00E+00	2.00E-180
SCE1572_8329	sce_7012	0.00E+00	0.00E+00
SCE1572_8330	sce_7013	0.00E+00	0.00E+00
SCE1572_8331	sce_7014	9.00E-148	4.00E-150
SCE1572_8332	sce_7015	6.00E-85	1.00E-85
SCE1572_8333	sce_7016	6.00E-48	6.00E-48
SCE1572_8336	sce_1676	0.00E+00	0.00E+00
SCE1572_8337	sce_304	2.00E-155	3.00E-165
SCE1572_8338	sce_9167	7.00E-22	1.00E-21
SCE1572_8343	sce_3174	8.00E-28	2.00E-18
SCE1572_8347	sce_9482	5.00E-100	7.00E-129
SCE1572_8349	sce_9480	2.00E-49	2.00E-50
SCE1572_835	sce_633	6.00E-28	5.00E-29
SCE1572_8355	sce_6226	0.00E+00	0.00E+00
SCE1572_836	sce_634	0.00E+00	0.00E+00
SCE1572_8367	sce_2167	1.00E-126	2.00E-121
SCE1572_8368	sce_2165	4.00E-124	7.00E-110
SCE1572_8369	sce_7077	5.00E-151	8.00E-156
SCE1572_8370	sce_7078	1.00E-96	2.00E-112
SCE1572_8371	sce_7079	5.00E-84	1.00E-77
SCE1572_8372	sce_7080	7.00E-60	1.00E-59
SCE1572_8373	sce_7085	0.00E+00	0.00E+00
SCE1572_8374	sce_7086	6.00E-83	6.00E-83
SCE1572_8375	sce_7087	5.00E-93	3.00E-86
SCE1572_8378	sce_7088	2.00E-116	1.00E-127
SCE1572_8379	sce_7089	2.00E-108	2.00E-90
SCE1572_838	sce_635	1.00E-122	2.00E-108
SCE1572_8380	sce_7090	4.00E-43	9.00E-45
SCE1572_8381	sce_7144	0.00E+00	0.00E+00
SCE1572_8385	sce_8592	0.00E+00	0.00E+00
SCE1572_8385	sce_3937	0.00E+00	0.00E+00
SCE1572_8386	sce_1676	0.00E+00	0.00E+00
SCE1572_8388	sce_7378	3.00E-73	2.00E-86
SCE1572_8389	sce_8819	3.00E-73	1.00E-76
SCE1572_839	sce_636	2.00E-65	7.00E-57
SCE1572_8391	sce_7154	0.00E+00	0.00E+00
SCE1572_8391	sce_5403	0.00E+00	0.00E+00
SCE1572_8391	sce_7969	0.00E+00	0.00E+00
SCE1572_8391	sce_5226	0.00E+00	0.00E+00
SCE1572_8391	sce_1803	0.00E+00	0.00E+00
SCE1572_8391	sce_9665	0.00E+00	0.00E+00
SCE1572_8391	sce_5751	0.00E+00	0.00E+00
SCE1572_8391	sce_4073	0.00E+00	0.00E+00
SCE1572_8391	sce_5746	0.00E+00	0.00E+00
SCE1572_8391	sce_2053	0.00E+00	0.00E+00
SCE1572_8391	sce_9954	0.00E+00	0.00E+00
SCE1572_8391	sce_9757	0.00E+00	0.00E+00
SCE1572_8391	sce_3750	0.00E+00	0.00E+00
SCE1572_8391	sce_6712	0.00E+00	0.00E+00
SCE1572_8394	sce_7155	0.00E+00	0.00E+00
SCE1572_8395	sce_7156	0.00E+00	0.00E+00
SCE1572_8396	sce_7157	2.00E-45	2.00E-52
SCE1572_8397	sce_7158	0.00E+00	0.00E+00
SCE1572_8399	sce_7159	0.00E+00	0.00E+00
SCE1572_84	sce_71	4.00E-54	3.00E-65
SCE1572_840	sce_637	0.00E+00	0.00E+00
SCE1572_8400	sce_7160	0.00E+00	0.00E+00
SCE1572_8401	sce_7161	0.00E+00	0.00E+00
SCE1572_8402	sce_7162	2.00E-168	2.00E-168
SCE1572_8403	sce_7163	0.00E+00	0.00E+00
SCE1572_8405	sce_7164	1.00E-63	1.00E-63
SCE1572_8406	sce_7165	0.00E+00	3.00E-146
SCE1572_8407	sce_7166	4.00E-40	1.00E-49
SCE1572_8408	sce_7167	0.00E+00	0.00E+00

SCE1572_841	sce_5488	2.00E-170	1.00E-168
SCE1572_8410	sce_7169	1.00E-108	2.00E-110
SCE1572_8411	sce_7170	2.00E-46	1.00E-47
SCE1572_8412	sce_7171	0.00E+00	0.00E+00
SCE1572_8413	sce_7172	5.00E-112	2.00E-119
SCE1572_8414	sce_7173	0.00E+00	0.00E+00
SCE1572_8415	sce_7176	0.00E+00	0.00E+00
SCE1572_8416	sce_7177	6.00E-102	4.00E-110
SCE1572_8417	sce_7178	7.00E-152	3.00E-135
SCE1572_8418	sce_7179	4.00E-79	2.00E-82
SCE1572_8420	sce_7180	1.00E-66	1.00E-66
SCE1572_8421	sce_7181	4.00E-178	3.00E-153
SCE1572_8422	sce_7182	4.00E-132	2.00E-136
SCE1572_8423	sce_7183	0.00E+00	0.00E+00
SCE1572_8425	sce_7186	6.00E-66	4.00E-66
SCE1572_8427	sce_7187	5.00E-12	5.00E-12
SCE1572_8428	sce_7189	4.00E-46	1.00E-32
SCE1572_8429	sce_7190	2.00E-178	0.00E+00
SCE1572_843	sce_639	0.00E+00	0.00E+00
SCE1572_8431	sce_7191	2.00E-91	2.00E-91
SCE1572_8432	sce_7192	8.00E-35	1.00E-37
SCE1572_8434	sce_7193	0.00E+00	0.00E+00
SCE1572_8436	sce_7196	1.00E-169	7.00E-166
SCE1572_8438	sce_7197	5.00E-107	2.00E-107
SCE1572_8439	sce_7198	4.00E-112	1.00E-111
SCE1572_844	sce_640	0.00E+00	0.00E+00
SCE1572_8440	sce_7199	1.00E-146	3.00E-138
SCE1572_8441	sce_7201	1.00E-177	1.00E-177
SCE1572_8442	sce_7202	1.00E-54	1.00E-59
SCE1572_8443	sce_7203	6.00E-83	8.00E-76
SCE1572_8444	sce_7204	2.00E-67	3.00E-60
SCE1572_8445	sce_7205	4.00E-37	7.00E-49
SCE1572_8448	sce_3793	0.00E+00	0.00E+00
SCE1572_8448	sce_7069	0.00E+00	0.00E+00
SCE1572_8448	sce_5086	0.00E+00	0.00E+00
SCE1572_8448	sce_5085	0.00E+00	0.00E+00
SCE1572_8448	sce_6701	0.00E+00	0.00E+00
SCE1572_8449	sce_7207	1.00E-71	2.00E-77
SCE1572_8450	sce_7208	2.00E-111	4.00E-105
SCE1572_8451	sce_7211	2.00E-96	6.00E-102
SCE1572_8452	sce_7212	7.00E-66	1.00E-72
SCE1572_8453	sce_7213	1.00E-55	3.00E-59
SCE1572_8454	sce_7214	1.00E-117	5.00E-128
SCE1572_8455	sce_7215	2.00E-122	4.00E-137
SCE1572_8456	sce_7216	2.00E-107	4.00E-118
SCE1572_8457	sce_7217	0.00E+00	0.00E+00
SCE1572_8458	sce_7218	0.00E+00	0.00E+00
SCE1572_8459	sce_7219	1.00E-84	3.00E-93
SCE1572_8460	sce_7220	4.00E-136	1.00E-124
SCE1572_8461	sce_7221	1.00E-34	1.00E-34
SCE1572_8462	sce_7222	1.00E-76	5.00E-82
SCE1572_8463	sce_7224	0.00E+00	0.00E+00
SCE1572_8464	sce_7225	6.00E-15	6.00E-15
SCE1572_8466	sce_7226	5.00E-70	3.00E-68
SCE1572_8467	sce_7227	7.00E-56	5.00E-73
SCE1572_8468	sce_7228	0.00E+00	0.00E+00
SCE1572_8469	sce_7230	4.00E-11	3.00E-12
SCE1572_847	sce_4968	0.00E+00	0.00E+00
SCE1572_8470	sce_7231	1.00E-159	2.00E-168
SCE1572_8471	sce_7232	0.00E+00	0.00E+00
SCE1572_8472	sce_7234	5.00E-93	6.00E-69
SCE1572_8473	sce_7236	7.00E-75	4.00E-74
SCE1572_8474	sce_7237	0.00E+00	0.00E+00
SCE1572_8476	sce_7238	5.00E-114	3.00E-124
SCE1572_8477	sce_7239	0.00E+00	0.00E+00
SCE1572_8477	sce_9431	0.00E+00	0.00E+00
SCE1572_8478	sce_7243	1.00E-44	3.00E-59
SCE1572_8479	sce_7244	1.00E-140	6.00E-155
SCE1572_848	sce_4641	0.00E+00	0.00E+00
SCE1572_848	sce_4638	0.00E+00	0.00E+00
SCE1572_848	sce_3575	0.00E+00	0.00E+00
SCE1572_848	sce_3577	0.00E+00	0.00E+00
SCE1572_848	sce_3580	0.00E+00	0.00E+00
SCE1572_8480	sce_7245	0.00E+00	0.00E+00
SCE1572_8481	sce_7246	7.00E-66	4.00E-73
SCE1572_8482	sce_7248	8.00E-35	8.00E-35

SCE1572_8483	sce_7249	0.00E+00	0.00E+00
SCE1572_8484	sce_7251	5.00E-39	5.00E-46
SCE1572_8485	sce_7252	3.00E-121	2.00E-166
SCE1572_8488	sce_7254	0.00E+00	0.00E+00
SCE1572_8489	sce_7256	2.00E-123	1.00E-131
SCE1572_849	sce_4638	0.00E+00	0.00E+00
SCE1572_849	sce_4641	0.00E+00	0.00E+00
SCE1572_849	sce_4640	0.00E+00	0.00E+00
SCE1572_8490	sce_7257	3.00E-154	1.00E-149
SCE1572_8491	sce_7258	1.00E-106	9.00E-98
SCE1572_8492	sce_7260	0.00E+00	0.00E+00
SCE1572_8493	sce_7261	2.00E-73	4.00E-76
SCE1572_8494	sce_7262	7.00E-112	6.00E-83
SCE1572_8495	sce_3750	0.00E+00	0.00E+00
SCE1572_8495	sce_5229	0.00E+00	0.00E+00
SCE1572_8495	sce_6712	0.00E+00	0.00E+00
SCE1572_85	sce_73	0.00E+00	0.00E+00
SCE1572_850	sce_3577	0.00E+00	0.00E+00
SCE1572_850	sce_3575	0.00E+00	0.00E+00
SCE1572_8503	sce_4101	2.00E-37	7.00E-38
SCE1572_8504	sce_4100	2.00E-116	9.00E-124
SCE1572_8505	sce_4099	1.00E-98	1.00E-105
SCE1572_8506	sce_4098	1.00E-82	7.00E-91
SCE1572_8507	sce_7263	1.00E-109	7.00E-108
SCE1572_8509	sce_9501	2.00E-164	0.00E+00
SCE1572_851	sce_3575	0.00E+00	0.00E+00
SCE1572_851	sce_3576	0.00E+00	8.00E-172
SCE1572_851	sce_3580	0.00E+00	0.00E+00
SCE1572_851	sce_3577	0.00E+00	0.00E+00
SCE1572_851	sce_3579	0.00E+00	0.00E+00
SCE1572_8510	sce_8461	2.00E-67	2.00E-67
SCE1572_8511	sce_9502	2.00E-153	2.00E-153
SCE1572_8517	sce_10363	5.00E-113	9.00E-111
SCE1572_8518	sce_7264	5.00E-129	5.00E-129
SCE1572_8519	sce_7265	2.00E-39	5.00E-39
SCE1572_8520	sce_7266	8.00E-55	4.00E-67
SCE1572_8522	sce_7267	0.00E+00	0.00E+00
SCE1572_8524	sce_7268	1.00E-176	2.00E-147
SCE1572_8525	sce_7269	9.00E-50	9.00E-50
SCE1572_8526	sce_7270	2.00E-73	5.00E-66
SCE1572_8528	sce_7272	2.00E-173	4.00E-174
SCE1572_8529	sce_7273	0.00E+00	0.00E+00
SCE1572_8530	sce_7274	3.00E-169	1.00E-164
SCE1572_8531	sce_7275	6.00E-100	3.00E-107
SCE1572_8533	sce_7276	5.00E-173	2.00E-147
SCE1572_8534	sce_7277	3.00E-165	4.00E-165
SCE1572_8535	sce_7278	2.00E-173	2.00E-173
SCE1572_8536	sce_7279	1.00E-107	2.00E-119
SCE1572_8537	sce_7280	1.00E-169	1.00E-178
SCE1572_8538	sce_7281	1.00E-172	1.00E-171
SCE1572_8539	sce_7282	7.00E-116	1.00E-118
SCE1572_8540	sce_7283	0.00E+00	0.00E+00
SCE1572_8541	sce_7285	3.00E-96	3.00E-96
SCE1572_8542	sce_7286	3.00E-90	5.00E-99
SCE1572_8543	sce_7288	2.00E-146	1.00E-146
SCE1572_8545	sce_3427	0.00E+00	0.00E+00
SCE1572_8546	sce_3426	0.00E+00	0.00E+00
SCE1572_8547	sce_3425	2.00E-128	2.00E-135
SCE1572_8548	sce_3424	0.00E+00	0.00E+00
SCE1572_8549	sce_7289	0.00E+00	0.00E+00
SCE1572_8550	sce_7290	7.00E-146	3.00E-152
SCE1572_8551	sce_7291	8.00E-173	5.00E-166
SCE1572_8552	sce_7292	4.00E-75	4.00E-72
SCE1572_8553	sce_7293	3.00E-94	1.00E-91
SCE1572_8554	sce_7294	0.00E+00	0.00E+00
SCE1572_8555	sce_7295	3.00E-54	3.00E-54
SCE1572_8556	sce_7296	2.00E-123	8.00E-147
SCE1572_8557	sce_7297	0.00E+00	0.00E+00
SCE1572_8558	sce_7298	0.00E+00	0.00E+00
SCE1572_8560	sce_6041	3.00E-85	3.00E-85
SCE1572_8561	sce_7304	0.00E+00	0.00E+00
SCE1572_8561	sce_6101	0.00E+00	0.00E+00
SCE1572_8565	sce_4641	0.00E+00	0.00E+00
SCE1572_8566	sce_7306	2.00E-86	4.00E-82
SCE1572_8567	sce_7307	0.00E+00	0.00E+00
SCE1572_8568	sce_7308	4.00E-86	4.00E-88

SCE1572_8578	sce_1676	0.00E+00	0.00E+00
SCE1572_8581	sce_9566	0.00E+00	0.00E+00
SCE1572_8581	sce_7530	0.00E+00	0.00E+00
SCE1572_8583	sce_9567	0.00E+00	0.00E+00
SCE1572_8583	sce_7528	0.00E+00	5.00E-175
SCE1572_8585	sce_7312	7.00E-56	4.00E-58
SCE1572_8588	sce_7314	1.00E-107	2.00E-107
SCE1572_8589	sce_7315	2.00E-67	6.00E-67
SCE1572_8590	sce_7317	2.00E-13	6.00E-20
SCE1572_8594	sce_6282	0.00E+00	0.00E+00
SCE1572_860	sce_3570	3.00E-10	3.00E-10
SCE1572_8601	sce_8200	9.00E-22	2.00E-22
SCE1572_8603	sce_7319	3.00E-167	2.00E-167
SCE1572_8604	sce_7320	0.00E+00	0.00E+00
SCE1572_8605	sce_7321	6.00E-101	2.00E-100
SCE1572_8607	sce_7322	0.00E+00	0.00E+00
SCE1572_8608	sce_7323	3.00E-53	1.00E-57
SCE1572_861	sce_3567	2.00E-35	2.00E-35
SCE1572_8610	sce_7325	2.00E-161	3.00E-161
SCE1572_8611	sce_7326	0.00E+00	0.00E+00
SCE1572_8613	sce_7332	8.00E-126	9.00E-126
SCE1572_8614	sce_7334	2.00E-117	8.00E-97
SCE1572_8615	sce_7335	0.00E+00	0.00E+00
SCE1572_8616	sce_7336	3.00E-136	3.00E-134
SCE1572_8617	sce_7337	4.00E-118	5.00E-121
SCE1572_8618	sce_7338	2.00E-158	8.00E-166
SCE1572_8619	sce_7339	4.00E-61	3.00E-55
SCE1572_8620	sce_7341	5.00E-76	8.00E-74
SCE1572_8621	sce_7343	0.00E+00	0.00E+00
SCE1572_8622	sce_7344	3.00E-72	7.00E-68
SCE1572_8623	sce_7345	3.00E-112	1.00E-117
SCE1572_8624	sce_7346	2.00E-24	2.00E-31
SCE1572_8625	sce_7347	0.00E+00	0.00E+00
SCE1572_8627	sce_7348	1.00E-125	5.00E-118
SCE1572_8628	sce_7349	2.00E-115	2.00E-115
SCE1572_8629	sce_6206	2.00E-48	2.00E-44
SCE1572_8630	sce_7350	3.00E-55	3.00E-39
SCE1572_8632	sce_7352	4.00E-61	9.00E-61
SCE1572_8635	sce_4902	1.00E-62	5.00E-58
SCE1572_8637	sce_7353	1.00E-180	0.00E+00
SCE1572_8638	sce_7355	3.00E-47	5.00E-52
SCE1572_8639	sce_7358	0.00E+00	4.00E-172
SCE1572_864	sce_3568	5.00E-105	1.00E-104
SCE1572_8640	sce_7359	3.00E-105	9.00E-107
SCE1572_8641	sce_7360	5.00E-108	6.00E-106
SCE1572_8643	sce_7362	4.00E-98	6.00E-97
SCE1572_8644	sce_7363	7.00E-149	7.00E-146
SCE1572_8646	sce_7364	9.00E-55	2.00E-47
SCE1572_8650	sce_7366	4.00E-161	5.00E-163
SCE1572_8652	sce_7367	1.00E-149	1.00E-127
SCE1572_8653	sce_7368	2.00E-127	1.00E-140
SCE1572_8654	sce_7369	0.00E+00	0.00E+00
SCE1572_8655	sce_7370	0.00E+00	0.00E+00
SCE1572_8656	sce_7371	5.00E-67	7.00E-68
SCE1572_8657	sce_7372	1.00E-28	1.00E-28
SCE1572_8658	sce_7373	9.00E-82	2.00E-80
SCE1572_8659	sce_7374	3.00E-51	5.00E-48
SCE1572_8661	sce_7375	0.00E+00	0.00E+00
SCE1572_8663	sce_7393	2.00E-109	2.00E-109
SCE1572_8664	sce_7395	2.00E-59	1.00E-65
SCE1572_8668	sce_2495	2.00E-71	1.00E-61
SCE1572_8669	sce_6970	6.00E-17	6.00E-17
SCE1572_867	sce_641	2.00E-98	2.00E-96
SCE1572_8671	sce_6971	1.00E-19	2.00E-25
SCE1572_8672	sce_3730	9.00E-66	2.00E-76
SCE1572_8674	sce_2613	7.00E-67	1.00E-63
SCE1572_8676	sce_7073	1.00E-28	2.00E-28
SCE1572_868	sce_642	3.00E-180	0.00E+00
SCE1572_8680	sce_7433	3.00E-118	3.00E-118
SCE1572_8681	sce_3735	4.00E-103	2.00E-108
SCE1572_8682	sce_6962	4.00E-11	9.00E-15
SCE1572_8684	sce_1255	1.00E-30	1.00E-30
SCE1572_8685	sce_7426	1.00E-165	1.00E-165
SCE1572_8687	sce_7428	3.00E-84	9.00E-90
SCE1572_8688	sce_4940	4.00E-106	2.00E-105
SCE1572_8689	sce_8360	5.00E-79	4.00E-76

SCE1572_8691	sce_8694	0.00E+00	0.00E+00
SCE1572_8691	sce_9974	0.00E+00	0.00E+00
SCE1572_8691	sce_9973	0.00E+00	0.00E+00
SCE1572_8691	sce_2645	0.00E+00	0.00E+00
SCE1572_8694	sce_7435	0.00E+00	0.00E+00
SCE1572_8695	sce_7436	1.00E-179	2.00E-169
SCE1572_8698	sce_7465	0.00E+00	0.00E+00
SCE1572_8698	sce_774	0.00E+00	0.00E+00
SCE1572_8698	sce_1723	0.00E+00	0.00E+00
SCE1572_8698	sce_5787	0.00E+00	0.00E+00
SCE1572_8698	sce_9954	0.00E+00	0.00E+00
SCE1572_8698	sce_5229	0.00E+00	0.00E+00
SCE1572_8698	sce_6712	0.00E+00	0.00E+00
SCE1572_8698	sce_3750	0.00E+00	0.00E+00
SCE1572_87	sce_74	2.00E-102	9.00E-97
SCE1572_870	sce_645	6.00E-100	2.00E-94
SCE1572_8700	sce_7466	3.00E-150	9.00E-152
SCE1572_8702	sce_7467	6.00E-61	1.00E-56
SCE1572_8703	sce_7473	9.00E-117	3.00E-126
SCE1572_8704	sce_7474	4.00E-40	2.00E-43
SCE1572_8709	sce_7477	4.00E-33	4.00E-37
SCE1572_871	sce_646	0.00E+00	0.00E+00
SCE1572_8710	sce_851	1.00E-67	1.00E-67
SCE1572_8711	sce_852	1.00E-65	1.00E-65
SCE1572_8712	sce_7522	2.00E-148	2.00E-148
SCE1572_8713	sce_7523	4.00E-167	6.00E-170
SCE1572_8714	sce_7524	4.00E-171	4.00E-177
SCE1572_8715	sce_7525	1.00E-129	1.00E-129
SCE1572_8716	sce_7526	2.00E-134	2.00E-169
SCE1572_8719	sce_5999	3.00E-105	1.00E-105
SCE1572_872	sce_647	0.00E+00	0.00E+00
SCE1572_8722	sce_5994	5.00E-49	2.00E-44
SCE1572_8729	sce_7527	2.00E-68	1.00E-83
SCE1572_8730	sce_7540	8.00E-131	2.00E-130
SCE1572_8731	sce_7541	3.00E-70	7.00E-63
SCE1572_8733	sce_828	3.00E-110	2.00E-120
SCE1572_8734	sce_829	4.00E-84	6.00E-73
SCE1572_8737	sce_7545	4.00E-158	2.00E-162
SCE1572_8738	sce_7546	5.00E-125	2.00E-129
SCE1572_8739	sce_7547	6.00E-152	4.00E-171
SCE1572_874	sce_648	5.00E-60	1.00E-57
SCE1572_8740	sce_7557	6.00E-126	6.00E-122
SCE1572_8741	sce_7559	1.00E-152	4.00E-162
SCE1572_8748	sce_7571	0.00E+00	0.00E+00
SCE1572_8749	sce_7572	0.00E+00	6.00E-177
SCE1572_8756	sce_7575	0.00E+00	0.00E+00
SCE1572_8757	sce_7576	1.00E-50	8.00E-64
SCE1572_8758	sce_7578	0.00E+00	3.00E-180
SCE1572_8759	sce_7579	0.00E+00	3.00E-177
SCE1572_876	sce_649	2.00E-61	2.00E-61
SCE1572_8760	sce_2037	0.00E+00	0.00E+00
SCE1572_8764	sce_1676	0.00E+00	0.00E+00
SCE1572_8766	sce_8672	7.00E-40	4.00E-31
SCE1572_8767	sce_9806	6.00E-63	9.00E-74
SCE1572_8767	sce_6808	6.00E-63	9.00E-74
SCE1572_877	sce_651	1.00E-75	1.00E-75
SCE1572_8777	sce_7787	2.00E-81	5.00E-94
SCE1572_8779	sce_7584	1.00E-64	1.00E-64
SCE1572_878	sce_653	5.00E-60	2.00E-57
SCE1572_8781	sce_7585	0.00E+00	0.00E+00
SCE1572_8782	sce_7586	8.00E-155	2.00E-145
SCE1572_8783	sce_7587	0.00E+00	0.00E+00
SCE1572_8784	sce_7588	1.00E-140	4.00E-140
SCE1572_8785	sce_7589	3.00E-166	6.00E-169
SCE1572_8786	sce_7590	0.00E+00	0.00E+00
SCE1572_8787	sce_7591	3.00E-176	0.00E+00
SCE1572_8789	sce_7592	9.00E-164	3.00E-173
SCE1572_879	sce_654	9.00E-144	6.00E-130
SCE1572_8790	sce_2005	0.00E+00	0.00E+00
SCE1572_8791	sce_1526	7.00E-17	1.00E-16
SCE1572_8792	sce_5303	3.00E-97	3.00E-97
SCE1572_8795	sce_8795	2.00E-33	9.00E-28
SCE1572_88	sce_75	2.00E-152	2.00E-152
SCE1572_880	sce_655	0.00E+00	0.00E+00
SCE1572_8803	sce_1534	4.00E-65	5.00E-71
SCE1572_8804	sce_1535	6.00E-71	2.00E-71

SCE1572_881	sce_656	1.00E-121	2.00E-96
SCE1572_8811	sce_2112	6.00E-52	3.00E-51
SCE1572_8812	sce_7084	3.00E-88	4.00E-88
SCE1572_8814	sce_2393	1.00E-16	7.00E-17
SCE1572_8815	sce_7043	6.00E-34	6.00E-34
SCE1572_8816	sce_9596	2.00E-147	2.00E-147
SCE1572_8818	sce_9597	1.00E-105	1.00E-105
SCE1572_882	sce_657	0.00E+00	0.00E+00
SCE1572_8824	sce_2109	2.00E-12	6.00E-13
SCE1572_8826	sce_515	1.00E-167	3.00E-168
SCE1572_8834	sce_6701	0.00E+00	0.00E+00
SCE1572_8834	sce_5086	0.00E+00	0.00E+00
SCE1572_8834	sce_5085	0.00E+00	0.00E+00
SCE1572_8834	sce_3793	0.00E+00	0.00E+00
SCE1572_884	sce_658	1.00E-57	1.00E-57
SCE1572_8841	sce_2158	4.00E-132	6.00E-129
SCE1572_8845	sce_7604	9.00E-145	1.00E-146
SCE1572_8846	sce_7605	4.00E-129	2.00E-125
SCE1572_8848	sce_7607	8.00E-161	8.00E-163
SCE1572_8849	sce_7610	2.00E-156	3.00E-152
SCE1572_885	sce_659	5.00E-134	1.00E-134
SCE1572_8850	sce_7611	1.00E-106	2.00E-86
SCE1572_8852	sce_7620	0.00E+00	0.00E+00
SCE1572_8854	sce_7621	8.00E-140	2.00E-139
SCE1572_8855	sce_7622	2.00E-84	2.00E-84
SCE1572_8856	sce_7624	1.00E-90	2.00E-90
SCE1572_8857	sce_7625	5.00E-69	5.00E-69
SCE1572_8858	sce_7626	0.00E+00	0.00E+00
SCE1572_8859	sce_7627	0.00E+00	0.00E+00
SCE1572_886	sce_660	0.00E+00	0.00E+00
SCE1572_8861	sce_8755	0.00E+00	0.00E+00
SCE1572_8861	sce_7818	0.00E+00	0.00E+00
SCE1572_8861	sce_7814	0.00E+00	0.00E+00
SCE1572_8861	sce_7815	0.00E+00	0.00E+00
SCE1572_8861	sce_8753	0.00E+00	0.00E+00
SCE1572_8865	sce_6039	7.00E-151	6.00E-151
SCE1572_8866	sce_6040	2.00E-166	2.00E-169
SCE1572_8868	sce_1251	4.00E-11	2.00E-13
SCE1572_8869	sce_7628	3.00E-135	2.00E-126
SCE1572_887	sce_661	3.00E-140	3.00E-140
SCE1572_8871	sce_7630	0.00E+00	0.00E+00
SCE1572_8872	sce_7631	2.00E-111	1.00E-111
SCE1572_8873	sce_1062	1.00E-171	0.00E+00
SCE1572_8876	sce_7661	0.00E+00	0.00E+00
SCE1572_8876	sce_6092	0.00E+00	0.00E+00
SCE1572_8876	sce_6095	0.00E+00	0.00E+00
SCE1572_8876	sce_3917	0.00E+00	0.00E+00
SCE1572_8876	sce_6107	0.00E+00	0.00E+00
SCE1572_8876	sce_6093	0.00E+00	0.00E+00
SCE1572_8876	sce_6094	0.00E+00	0.00E+00
SCE1572_8876	sce_388	0.00E+00	0.00E+00
SCE1572_8876	sce_6535	0.00E+00	0.00E+00
SCE1572_8876	sce_9920	0.00E+00	0.00E+00
SCE1572_8876	sce_10204	0.00E+00	0.00E+00
SCE1572_8876	sce_7640	0.00E+00	0.00E+00
SCE1572_8876	sce_5835	0.00E+00	0.00E+00
SCE1572_8876	sce_4513	0.00E+00	0.00E+00
SCE1572_8876	sce_9557	0.00E+00	0.00E+00
SCE1572_8876	sce_1398	0.00E+00	0.00E+00
SCE1572_8876	sce_3003	0.00E+00	0.00E+00
SCE1572_8876	sce_8602	0.00E+00	0.00E+00
SCE1572_8876	sce_8618	0.00E+00	0.00E+00
SCE1572_8878	sce_7515	5.00E-94	4.00E-57
SCE1572_8880	sce_7519	6.00E-117	3.00E-105
SCE1572_8881	sce_9791	4.00E-135	3.00E-140
SCE1572_8883	sce_10354	8.00E-63	3.00E-63
SCE1572_8886	sce_7657	0.00E+00	0.00E+00
SCE1572_8886	sce_3491	0.00E+00	0.00E+00
SCE1572_8886	sce_6712	0.00E+00	0.00E+00
SCE1572_8886	sce_9954	0.00E+00	0.00E+00
SCE1572_8887	sce_7658	3.00E-21	2.00E-19
SCE1572_8888	sce_7659	1.00E-144	1.00E-121
SCE1572_889	sce_662	0.00E+00	0.00E+00
SCE1572_8890	sce_6214	5.00E-96	1.00E-96
SCE1572_8894	sce_7664	0.00E+00	0.00E+00
SCE1572_8896	sce_7666	4.00E-55	8.00E-62

SCE1572_8897	sce_7673	0.00E+00	0.00E+00
SCE1572_8897	sce_7667	0.00E+00	0.00E+00
SCE1572_89	sce_76	0.00E+00	0.00E+00
SCE1572_890	sce_663	0.00E+00	1.00E-179
SCE1572_8900	sce_7675	8.00E-170	1.00E-177
SCE1572_8901	sce_7677	0.00E+00	0.00E+00
SCE1572_8902	sce_3000	2.00E-126	5.00E-140
SCE1572_8904	sce_7678	2.00E-143	7.00E-144
SCE1572_8905	sce_7689	3.00E-78	2.00E-78
SCE1572_8909	sce_7691	2.00E-117	6.00E-123
SCE1572_891	sce_664	0.00E+00	0.00E+00
SCE1572_8910	sce_7692	8.00E-69	3.00E-69
SCE1572_8911	sce_7695	5.00E-98	3.00E-102
SCE1572_8914	sce_7696	2.00E-107	2.00E-103
SCE1572_8917	sce_6286	1.00E-94	4.00E-73
SCE1572_8918	sce_6287	5.00E-62	5.00E-74
SCE1572_8919	sce_6288	1.00E-45	1.00E-45
SCE1572_8920	sce_864	0.00E+00	0.00E+00
SCE1572_8929	sce_7697	2.00E-47	2.00E-47
SCE1572_893	sce_666	3.00E-92	5.00E-85
SCE1572_8931	sce_7698	3.00E-136	2.00E-120
SCE1572_8932	sce_7699	0.00E+00	0.00E+00
SCE1572_8933	sce_7700	7.00E-113	1.00E-109
SCE1572_8934	sce_7701	2.00E-86	7.00E-71
SCE1572_8935	sce_7702	5.00E-77	3.00E-78
SCE1572_8937	sce_7703	0.00E+00	0.00E+00
SCE1572_8938	sce_7704	1.00E-22	5.00E-23
SCE1572_8939	sce_7705	6.00E-95	7.00E-95
SCE1572_894	sce_667	0.00E+00	0.00E+00
SCE1572_8940	sce_7706	6.00E-168	0.00E+00
SCE1572_8941	sce_7708	2.00E-140	3.00E-149
SCE1572_8942	sce_7709	2.00E-168	2.00E-168
SCE1572_8943	sce_7710	1.00E-42	3.00E-45
SCE1572_8945	sce_7712	2.00E-117	2.00E-117
SCE1572_8946	sce_7713	0.00E+00	0.00E+00
SCE1572_8947	sce_7714	0.00E+00	0.00E+00
SCE1572_8948	sce_7715	6.00E-25	2.00E-25
SCE1572_8949	sce_7717	1.00E-68	3.00E-78
SCE1572_895	sce_668	7.00E-110	4.00E-129
SCE1572_8952	sce_7718	4.00E-81	4.00E-62
SCE1572_8953	sce_7719	5.00E-16	8.00E-15
SCE1572_8954	sce_7720	5.00E-142	4.00E-144
SCE1572_8958	sce_7721	0.00E+00	0.00E+00
SCE1572_8962	sce_309	3.00E-74	1.00E-79
SCE1572_8963	sce_7725	2.00E-84	4.00E-90
SCE1572_8964	sce_7726	4.00E-108	4.00E-116
SCE1572_8965	sce_7727	0.00E+00	0.00E+00
SCE1572_8968	sce_7729	0.00E+00	0.00E+00
SCE1572_8969	sce_7730	3.00E-101	3.00E-97
SCE1572_8970	sce_7731	1.00E-173	9.00E-167
SCE1572_8971	sce_7733	0.00E+00	0.00E+00
SCE1572_8975	sce_7734	2.00E-135	3.00E-128
SCE1572_8976	sce_7735	3.00E-47	3.00E-50
SCE1572_8977	sce_7736	1.00E-63	1.00E-63
SCE1572_8978	sce_7737	9.00E-176	8.00E-166
SCE1572_8979	sce_7738	6.00E-15	4.00E-14
SCE1572_898	sce_670	8.00E-99	3.00E-116
SCE1572_8980	sce_10099	9.00E-46	5.00E-46
SCE1572_8984	sce_7750	7.00E-139	6.00E-156
SCE1572_8987	sce_7751	2.00E-154	1.00E-173
SCE1572_8989	sce_7752	0.00E+00	0.00E+00
SCE1572_8990	sce_7753	6.00E-53	4.00E-51
SCE1572_8991	sce_7756	1.00E-36	6.00E-37
SCE1572_8992	sce_7757	1.00E-122	6.00E-123
SCE1572_8993	sce_7758	0.00E+00	0.00E+00
SCE1572_8994	sce_7759	4.00E-104	2.00E-109
SCE1572_8995	sce_7760	5.00E-56	2.00E-55
SCE1572_8996	sce_7761	1.00E-47	4.00E-41
SCE1572_8997	sce_7762	1.00E-129	1.00E-127
SCE1572_8998	sce_7763	0.00E+00	0.00E+00
SCE1572_8999	sce_7764	4.00E-88	2.00E-88
SCE1572_9	sce_9	3.00E-110	3.00E-111
SCE1572_90	sce_77	0.00E+00	0.00E+00
SCE1572_900	sce_671	0.00E+00	0.00E+00
SCE1572_9000	sce_7765	4.00E-118	4.00E-118
SCE1572_9001	sce_7766	2.00E-67	6.00E-68

SCE1572_9002	sce_7767	6.00E-42	6.00E-42
SCE1572_9003	sce_7768	2.00E-78	2.00E-78
SCE1572_9004	sce_7769	1.00E-103	3.00E-117
SCE1572_9005	sce_7770	2.00E-08	1.00E-10
SCE1572_9006	sce_7771	0.00E+00	0.00E+00
SCE1572_9007	sce_7772	3.00E-39	2.00E-30
SCE1572_9008	sce_7773	0.00E+00	0.00E+00
SCE1572_901	sce_672	2.00E-128	3.00E-136
SCE1572_9012	sce_7774	2.00E-135	3.00E-127
SCE1572_902	sce_674	4.00E-25	1.00E-22
SCE1572_9021	sce_7776	4.00E-115	7.00E-105
SCE1572_9022	sce_7777	7.00E-159	4.00E-150
SCE1572_9023	sce_7778	0.00E+00	0.00E+00
SCE1572_9024	sce_7779	1.00E-40	1.00E-40
SCE1572_9025	sce_7780	8.00E-130	5.00E-130
SCE1572_9026	sce_7781	6.00E-153	2.00E-141
SCE1572_9027	sce_7782	0.00E+00	0.00E+00
SCE1572_9028	sce_7783	3.00E-165	2.00E-158
SCE1572_9029	sce_7784	7.00E-94	3.00E-95
SCE1572_903	sce_6084	6.00E-92	3.00E-88
SCE1572_9030	sce_7785	2.00E-116	2.00E-91
SCE1572_9031	sce_7786	0.00E+00	0.00E+00
SCE1572_9032	sce_7361	0.00E+00	0.00E+00
SCE1572_9032	sce_10425	0.00E+00	0.00E+00
SCE1572_9032	sce_3520	0.00E+00	0.00E+00
SCE1572_9033	sce_7791	2.00E-106	8.00E-90
SCE1572_9034	sce_7792	1.00E-80	6.00E-84
SCE1572_9035	sce_7793	7.00E-111	1.00E-105
SCE1572_9036	sce_7794	0.00E+00	0.00E+00
SCE1572_9037	sce_7796	0.00E+00	0.00E+00
SCE1572_9038	sce_7798	1.00E-48	2.00E-44
SCE1572_9039	sce_7799	2.00E-162	1.00E-155
SCE1572_904	sce_676	6.00E-145	5.00E-145
SCE1572_9040	sce_7800	9.00E-113	5.00E-123
SCE1572_9041	sce_7801	2.00E-176	6.00E-170
SCE1572_9042	sce_7802	5.00E-47	4.00E-50
SCE1572_9043	sce_7804	8.00E-76	2.00E-127
SCE1572_9044	sce_7805	0.00E+00	0.00E+00
SCE1572_9046	sce_7806	0.00E+00	0.00E+00
SCE1572_9047	sce_7807	1.00E-19	3.00E-20
SCE1572_9048	sce_7808	3.00E-54	2.00E-47
SCE1572_9049	sce_7809	0.00E+00	0.00E+00
SCE1572_905	sce_677	6.00E-124	3.00E-154
SCE1572_9050	sce_7810	5.00E-71	1.00E-77
SCE1572_9051	sce_7812	1.00E-75	2.00E-75
SCE1572_9053	sce_7818	0.00E+00	0.00E+00
SCE1572_9053	sce_7814	0.00E+00	0.00E+00
SCE1572_9053	sce_8755	0.00E+00	0.00E+00
SCE1572_9053	sce_7815	0.00E+00	0.00E+00
SCE1572_9053	sce_8753	0.00E+00	0.00E+00
SCE1572_9054	sce_7818	0.00E+00	0.00E+00
SCE1572_9055	sce_7819	5.00E-86	2.00E-97
SCE1572_9056	sce_1376	4.00E-69	1.00E-61
SCE1572_906	sce_678	3.00E-97	4.00E-97
SCE1572_9065	sce_7821	0.00E+00	0.00E+00
SCE1572_9067	sce_7822	3.00E-136	3.00E-157
SCE1572_9068	sce_7823	1.00E-111	6.00E-111
SCE1572_9069	sce_7824	4.00E-109	9.00E-121
SCE1572_907	sce_680	3.00E-89	3.00E-89
SCE1572_9070	sce_7825	0.00E+00	0.00E+00
SCE1572_9071	sce_7827	1.00E-179	0.00E+00
SCE1572_9072	sce_7828	4.00E-81	1.00E-88
SCE1572_9073	sce_7385	3.00E-169	2.00E-166
SCE1572_9074	sce_7837	3.00E-171	2.00E-167
SCE1572_9075	sce_3793	0.00E+00	0.00E+00
SCE1572_9075	sce_7069	0.00E+00	0.00E+00
SCE1572_9075	sce_5086	0.00E+00	0.00E+00
SCE1572_9075	sce_6701	0.00E+00	0.00E+00
SCE1572_9075	sce_5085	0.00E+00	0.00E+00
SCE1572_9077	sce_2024	0.00E+00	0.00E+00
SCE1572_9078	sce_2025	1.00E-65	1.00E-66
SCE1572_908	sce_681	9.00E-68	9.00E-78
SCE1572_9082	sce_7846	2.00E-79	1.00E-69
SCE1572_9083	sce_7847	9.00E-174	3.00E-167
SCE1572_9084	sce_7849	0.00E+00	0.00E+00
SCE1572_9087	sce_7850	0.00E+00	0.00E+00

SCE1572_9088	sce_7851	1.00E-47	2.00E-47
SCE1572_9089	sce_7867	0.00E+00	0.00E+00
SCE1572_9090	sce_7869	1.00E-69	4.00E-62
SCE1572_9092	sce_7876	0.00E+00	0.00E+00
SCE1572_9093	sce_7877	3.00E-21	5.00E-08
SCE1572_9094	sce_7878	5.00E-102	2.00E-108
SCE1572_9095	sce_7879	2.00E-135	1.00E-134
SCE1572_9096	sce_7880	2.00E-50	3.00E-44
SCE1572_9099	sce_7886	8.00E-123	3.00E-133
SCE1572_91	sce_79	3.00E-109	3.00E-109
SCE1572_910	sce_2065	0.00E+00	0.00E+00
SCE1572_910	sce_8618	0.00E+00	0.00E+00
SCE1572_910	sce_3917	0.00E+00	0.00E+00
SCE1572_910	sce_6107	0.00E+00	0.00E+00
SCE1572_910	sce_6092	0.00E+00	0.00E+00
SCE1572_910	sce_6094	0.00E+00	0.00E+00
SCE1572_910	sce_6095	0.00E+00	0.00E+00
SCE1572_910	sce_6093	0.00E+00	0.00E+00
SCE1572_910	sce_7661	0.00E+00	0.00E+00
SCE1572_910	sce_6535	0.00E+00	0.00E+00
SCE1572_910	sce_3003	0.00E+00	0.00E+00
SCE1572_910	sce_10204	0.00E+00	0.00E+00
SCE1572_910	sce_4513	0.00E+00	0.00E+00
SCE1572_910	sce_388	0.00E+00	0.00E+00
SCE1572_910	sce_7640	0.00E+00	0.00E+00
SCE1572_910	sce_9557	0.00E+00	0.00E+00
SCE1572_910	sce_5835	0.00E+00	0.00E+00
SCE1572_9100	sce_7882	5.00E-87	2.00E-88
SCE1572_9101	sce_7883	0.00E+00	0.00E+00
SCE1572_9103	sce_7884	0.00E+00	0.00E+00
SCE1572_9104	sce_7887	0.00E+00	0.00E+00
SCE1572_9105	sce_7888	2.00E-139	1.00E-131
SCE1572_9107	sce_7889	4.00E-53	2.00E-55
SCE1572_9108	sce_7890	6.00E-50	3.00E-51
SCE1572_9109	sce_7891	7.00E-79	2.00E-82
SCE1572_9110	sce_7892	0.00E+00	0.00E+00
SCE1572_9111	sce_7893	4.00E-77	3.00E-79
SCE1572_9112	sce_7894	8.00E-137	7.00E-135
SCE1572_9113	sce_7895	5.00E-71	5.00E-71
SCE1572_9115	sce_7897	2.00E-44	2.00E-44
SCE1572_9116	sce_7898	0.00E+00	0.00E+00
SCE1572_9117	sce_7899	0.00E+00	0.00E+00
SCE1572_912	sce_682	0.00E+00	0.00E+00
SCE1572_9123	sce_7901	2.00E-100	2.00E-100
SCE1572_9124	sce_7902	0.00E+00	0.00E+00
SCE1572_9125	sce_7903	2.00E-72	2.00E-66
SCE1572_9126	sce_7904	7.00E-71	2.00E-58
SCE1572_9127	sce_7905	4.00E-116	2.00E-117
SCE1572_9128	sce_7906	2.00E-73	2.00E-73
SCE1572_9129	sce_7907	0.00E+00	0.00E+00
SCE1572_913	sce_683	8.00E-59	1.00E-61
SCE1572_9130	sce_7908	5.00E-66	4.00E-62
SCE1572_9131	sce_7909	5.00E-26	2.00E-27
SCE1572_9132	sce_7910	1.00E-131	8.00E-132
SCE1572_9134	sce_7912	8.00E-89	9.00E-43
SCE1572_9135	sce_7913	1.00E-68	1.00E-56
SCE1572_9136	sce_7914	2.00E-79	2.00E-68
SCE1572_914	sce_684	2.00E-44	2.00E-44
SCE1572_9143	sce_8592	0.00E+00	0.00E+00
SCE1572_9143	sce_3937	0.00E+00	0.00E+00
SCE1572_9144	sce_7942	1.00E-108	6.00E-109
SCE1572_9145	sce_7943	7.00E-138	5.00E-139
SCE1572_9147	sce_7944	7.00E-128	7.00E-128
SCE1572_9148	sce_7945	0.00E+00	0.00E+00
SCE1572_9149	sce_7946	1.00E-104	1.00E-104
SCE1572_9150	sce_7947	1.00E-51	5.00E-31
SCE1572_9151	sce_7948	3.00E-50	6.00E-45
SCE1572_9152	sce_7949	0.00E+00	0.00E+00
SCE1572_9153	sce_7951	6.00E-83	6.00E-83
SCE1572_9154	sce_7952	0.00E+00	0.00E+00
SCE1572_9155	sce_7953	2.00E-172	1.00E-165
SCE1572_9156	sce_7954	0.00E+00	0.00E+00
SCE1572_9157	sce_7955	0.00E+00	0.00E+00
SCE1572_9158	sce_7956	5.00E-74	7.00E-65
SCE1572_9160	sce_7957	0.00E+00	0.00E+00
SCE1572_9161	sce_7958	3.00E-114	7.00E-119

SCE1572_917	sce_685	0.00E+00	0.00E+00
SCE1572_9171	sce_7098	6.00E-20	6.00E-27
SCE1572_9173	sce_7964	4.00E-40	5.00E-43
SCE1572_9174	sce_7965	8.00E-148	2.00E-158
SCE1572_9175	sce_7967	1.00E-107	1.00E-95
SCE1572_9177	sce_7969	0.00E+00	0.00E+00
SCE1572_9177	sce_5403	0.00E+00	0.00E+00
SCE1572_9178	sce_2177	8.00E-44	2.00E-55
SCE1572_9179	sce_7971	2.00E-87	1.00E-87
SCE1572_918	sce_686	0.00E+00	0.00E+00
SCE1572_9181	sce_7972	0.00E+00	0.00E+00
SCE1572_9187	sce_7985	7.00E-124	3.00E-123
SCE1572_9188	sce_7986	0.00E+00	0.00E+00
SCE1572_9189	sce_4575	5.00E-12	1.00E-12
SCE1572_919	sce_687	1.00E-72	1.00E-72
SCE1572_9190	sce_7987	1.00E-112	9.00E-113
SCE1572_9191	sce_7988	5.00E-55	5.00E-55
SCE1572_9192	sce_7386	0.00E+00	0.00E+00
SCE1572_9193	sce_7387	2.00E-55	2.00E-52
SCE1572_9194	sce_7388	7.00E-72	1.00E-75
SCE1572_9195	sce_7389	4.00E-88	5.00E-91
SCE1572_9196	sce_7990	2.00E-158	2.00E-159
SCE1572_9197	sce_1285	4.00E-56	3.00E-52
SCE1572_9198	sce_7991	7.00E-128	4.00E-131
SCE1572_9199	sce_7992	3.00E-139	3.00E-139
SCE1572_92	sce_81	0.00E+00	0.00E+00
SCE1572_9200	sce_7998	3.00E-15	4.00E-14
SCE1572_9202	sce_8000	0.00E+00	0.00E+00
SCE1572_9203	sce_8001	2.00E-114	3.00E-112
SCE1572_9204	sce_8002	3.00E-118	1.00E-120
SCE1572_9205	sce_8003	6.00E-72	2.00E-64
SCE1572_9206	sce_8004	3.00E-168	5.00E-167
SCE1572_9209	sce_8010	0.00E+00	0.00E+00
SCE1572_921	sce_688	0.00E+00	0.00E+00
SCE1572_9210	sce_8011	0.00E+00	0.00E+00
SCE1572_9211	sce_8012	1.00E-157	2.00E-156
SCE1572_9212	sce_8013	6.00E-159	3.00E-160
SCE1572_9214	sce_8014	0.00E+00	0.00E+00
SCE1572_9215	sce_8015	4.00E-42	2.00E-44
SCE1572_9216	sce_8016	0.00E+00	0.00E+00
SCE1572_9217	sce_8019	2.00E-180	0.00E+00
SCE1572_922	sce_689	7.00E-39	9.00E-35
SCE1572_9220	sce_8022	0.00E+00	0.00E+00
SCE1572_9222	sce_9351	6.00E-76	5.00E-76
SCE1572_9223	sce_7616	7.00E-80	7.00E-80
SCE1572_923	sce_690	2.00E-125	2.00E-106
SCE1572_9235	sce_4668	3.00E-45	7.00E-38
SCE1572_9236	sce_4669	3.00E-20	4.00E-25
SCE1572_924	sce_691	3.00E-68	4.00E-68
SCE1572_9248	sce_8023	0.00E+00	0.00E+00
SCE1572_9249	sce_8024	2.00E-168	1.00E-168
SCE1572_925	sce_692	2.00E-113	1.00E-104
SCE1572_9250	sce_8026	0.00E+00	0.00E+00
SCE1572_9251	sce_8027	0.00E+00	0.00E+00
SCE1572_9253	sce_8028	2.00E-108	2.00E-121
SCE1572_9254	sce_8029	2.00E-175	0.00E+00
SCE1572_9255	sce_8030	2.00E-157	1.00E-157
SCE1572_9256	sce_8031	8.00E-19	7.00E-16
SCE1572_9258	sce_8033	9.00E-151	4.00E-153
SCE1572_9259	sce_8034	2.00E-175	9.00E-166
SCE1572_926	sce_694	6.00E-131	1.00E-132
SCE1572_9260	sce_8035	0.00E+00	6.00E-176
SCE1572_9261	sce_8039	7.00E-33	3.00E-35
SCE1572_9262	sce_8041	3.00E-63	3.00E-63
SCE1572_9263	sce_8042	1.00E-50	1.00E-50
SCE1572_9264	sce_8043	8.00E-124	9.00E-132
SCE1572_9265	sce_8044	0.00E+00	0.00E+00
SCE1572_9266	sce_8047	1.00E-180	1.00E-141
SCE1572_9268	sce_8049	7.00E-137	2.00E-143
SCE1572_9269	sce_8050	3.00E-96	4.00E-109
SCE1572_927	sce_695	3.00E-109	6.00E-124
SCE1572_9272	sce_8052	7.00E-105	1.00E-101
SCE1572_9273	sce_8053	2.00E-136	8.00E-125
SCE1572_9274	sce_8054	0.00E+00	0.00E+00
SCE1572_9275	sce_8055	0.00E+00	0.00E+00
SCE1572_9276	sce_8056	0.00E+00	0.00E+00

SCE1572_9277	sce_8057	7.00E-168	4.00E-149
SCE1572_9278	sce_8058	8.00E-46	5.00E-25
SCE1572_9279	sce_8059	2.00E-89	3.00E-126
SCE1572_928	sce_696	6.00E-98	2.00E-105
SCE1572_9280	sce_8060	4.00E-22	2.00E-47
SCE1572_9281	sce_8061	2.00E-96	3.00E-97
SCE1572_9282	sce_8062	2.00E-115	2.00E-135
SCE1572_9283	sce_8064	1.00E-79	1.00E-79
SCE1572_9285	sce_8065	1.00E-58	3.00E-51
SCE1572_9286	sce_8067	2.00E-164	5.00E-163
SCE1572_9287	sce_8068	0.00E+00	0.00E+00
SCE1572_9288	sce_8069	4.00E-45	4.00E-45
SCE1572_929	sce_697	1.00E-157	8.00E-155
SCE1572_9290	sce_8070	0.00E+00	0.00E+00
SCE1572_9291	sce_8071	1.00E-104	1.00E-104
SCE1572_9292	sce_8072	0.00E+00	0.00E+00
SCE1572_9293	sce_8073	0.00E+00	0.00E+00
SCE1572_9294	sce_8074	0.00E+00	1.00E-179
SCE1572_9295	sce_8075	0.00E+00	0.00E+00
SCE1572_9296	sce_8076	4.00E-177	4.00E-170
SCE1572_9297	sce_8077	1.00E-67	8.00E-76
SCE1572_9298	sce_8078	1.00E-67	2.00E-71
SCE1572_9299	sce_8079	1.00E-92	2.00E-101
SCE1572_93	sce_82	0.00E+00	0.00E+00
SCE1572_930	sce_698	2.00E-95	2.00E-95
SCE1572_9300	sce_8080	7.00E-167	3.00E-166
SCE1572_9301	sce_8081	4.00E-42	8.00E-41
SCE1572_9302	sce_8082	5.00E-30	5.00E-30
SCE1572_9303	sce_8083	1.00E-75	1.00E-75
SCE1572_9304	sce_8084	0.00E+00	0.00E+00
SCE1572_9305	sce_8085	6.00E-118	2.00E-129
SCE1572_9306	sce_8086	7.00E-172	0.00E+00
SCE1572_9307	sce_8087	0.00E+00	0.00E+00
SCE1572_9308	sce_8088	0.00E+00	0.00E+00
SCE1572_931	sce_699	4.00E-100	4.00E-100
SCE1572_9310	sce_8089	0.00E+00	0.00E+00
SCE1572_9311	sce_8090	1.00E-163	6.00E-168
SCE1572_9312	sce_8092	2.00E-89	3.00E-89
SCE1572_9313	sce_8093	4.00E-80	2.00E-78
SCE1572_9314	sce_8094	0.00E+00	0.00E+00
SCE1572_9315	sce_8095	2.00E-113	8.00E-136
SCE1572_9316	sce_8096	1.00E-135	4.00E-143
SCE1572_9317	sce_8097	4.00E-174	0.00E+00
SCE1572_9318	sce_8099	0.00E+00	0.00E+00
SCE1572_9318	sce_6289	0.00E+00	0.00E+00
SCE1572_9320	sce_8101	2.00E-144	2.00E-144
SCE1572_9321	sce_8102	7.00E-38	2.00E-32
SCE1572_9322	sce_8103	2.00E-61	2.00E-61
SCE1572_9323	sce_8104	0.00E+00	0.00E+00
SCE1572_9324	sce_8105	0.00E+00	0.00E+00
SCE1572_9326	sce_8106	6.00E-53	6.00E-48
SCE1572_9327	sce_8107	1.00E-24	1.00E-24
SCE1572_9328	sce_8108	0.00E+00	0.00E+00
SCE1572_933	sce_701	0.00E+00	0.00E+00
SCE1572_9330	sce_8109	5.00E-89	5.00E-89
SCE1572_9331	sce_8110	3.00E-112	3.00E-112
SCE1572_9333	sce_8112	2.00E-70	1.00E-74
SCE1572_9334	sce_8113	0.00E+00	0.00E+00
SCE1572_9335	sce_8114	3.00E-158	3.00E-145
SCE1572_9336	sce_8115	5.00E-72	6.00E-79
SCE1572_9337	sce_8116	0.00E+00	0.00E+00
SCE1572_9337	sce_5582	0.00E+00	0.00E+00
SCE1572_9338	sce_8117	3.00E-66	2.00E-66
SCE1572_9339	sce_8118	5.00E-25	5.00E-31
SCE1572_934	sce_702	1.00E-148	2.00E-142
SCE1572_9340	sce_8119	1.00E-133	1.00E-133
SCE1572_9341	sce_8120	1.00E-144	3.00E-138
SCE1572_9342	sce_8121	9.00E-39	9.00E-39
SCE1572_9343	sce_8123	3.00E-131	2.00E-132
SCE1572_9344	sce_8124	7.00E-157	2.00E-148
SCE1572_9345	sce_8125	0.00E+00	0.00E+00
SCE1572_9346	sce_8126	0.00E+00	0.00E+00
SCE1572_9349	sce_8130	3.00E-18	1.00E-22
SCE1572_935	sce_703	5.00E-139	6.00E-139
SCE1572_9350	sce_8131	0.00E+00	0.00E+00
SCE1572_9351	sce_8132	7.00E-62	7.00E-62

SCE1572_9352	sce_8133	4.00E-102	2.00E-109
SCE1572_9353	sce_8134	0.00E+00	0.00E+00
SCE1572_9354	sce_8135	1.00E-84	7.00E-85
SCE1572_9355	sce_8136	3.00E-62	2.00E-62
SCE1572_9356	sce_8137	0.00E+00	0.00E+00
SCE1572_9357	sce_8138	0.00E+00	0.00E+00
SCE1572_9358	sce_8139	1.00E-131	4.00E-135
SCE1572_9359	sce_8140	6.00E-167	4.00E-167
SCE1572_936	sce_704	2.00E-112	1.00E-123
SCE1572_9360	sce_8141	2.00E-177	2.00E-173
SCE1572_9362	sce_8142	0.00E+00	0.00E+00
SCE1572_9364	sce_8144	0.00E+00	0.00E+00
SCE1572_9365	sce_8145	0.00E+00	0.00E+00
SCE1572_9366	sce_8146	2.00E-78	1.00E-87
SCE1572_9367	sce_8147	0.00E+00	0.00E+00
SCE1572_9368	sce_8148	2.00E-64	1.00E-53
SCE1572_9369	sce_8149	8.00E-13	8.00E-13
SCE1572_937	sce_705	4.00E-71	4.00E-66
SCE1572_9370	sce_8150	8.00E-124	2.00E-120
SCE1572_9371	sce_8151	5.00E-39	9.00E-47
SCE1572_9372	sce_8152	2.00E-168	2.00E-169
SCE1572_9373	sce_8153	5.00E-89	1.00E-95
SCE1572_9374	sce_8155	2.00E-172	4.00E-177
SCE1572_9376	sce_8157	3.00E-20	3.00E-20
SCE1572_9377	sce_8158	1.00E-67	5.00E-78
SCE1572_9379	sce_8159	0.00E+00	0.00E+00
SCE1572_938	sce_706	2.00E-22	6.00E-14
SCE1572_9380	sce_8160	7.00E-27	4.00E-44
SCE1572_9381	sce_8161	0.00E+00	0.00E+00
SCE1572_9383	sce_8162	0.00E+00	0.00E+00
SCE1572_9384	sce_8164	4.00E-76	3.00E-83
SCE1572_9385	sce_8165	2.00E-15	2.00E-23
SCE1572_9386	sce_8166	2.00E-155	2.00E-174
SCE1572_9387	sce_8167	0.00E+00	0.00E+00
SCE1572_9388	sce_8168	8.00E-88	2.00E-87
SCE1572_9389	sce_8169	0.00E+00	0.00E+00
SCE1572_939	sce_707	1.00E-107	3.00E-111
SCE1572_9390	sce_8170	1.00E-155	4.00E-155
SCE1572_9391	sce_8171	1.00E-34	1.00E-43
SCE1572_9393	sce_8172	2.00E-72	3.00E-64
SCE1572_9394	sce_8173	2.00E-150	4.00E-164
SCE1572_9395	sce_8174	7.00E-22	7.00E-20
SCE1572_9396	sce_8175	0.00E+00	0.00E+00
SCE1572_9397	sce_8179	2.00E-83	3.00E-84
SCE1572_9398	sce_8180	4.00E-115	8.00E-108
SCE1572_9399	sce_8181	8.00E-150	6.00E-149
SCE1572_94	sce_83	3.00E-46	3.00E-46
SCE1572_940	sce_708	0.00E+00	0.00E+00
SCE1572_9400	sce_8182	8.00E-36	1.00E-44
SCE1572_9401	sce_8183	1.00E-58	2.00E-58
SCE1572_9402	sce_8184	0.00E+00	0.00E+00
SCE1572_9403	sce_8185	1.00E-148	1.00E-140
SCE1572_9404	sce_8186	1.00E-106	6.00E-102
SCE1572_9407	sce_8189	0.00E+00	0.00E+00
SCE1572_9408	sce_8190	1.00E-85	6.00E-93
SCE1572_9409	sce_8191	0.00E+00	0.00E+00
SCE1572_941	sce_709	5.00E-29	2.00E-29
SCE1572_9410	sce_8192	0.00E+00	0.00E+00
SCE1572_9411	sce_8193	2.00E-147	1.00E-144
SCE1572_9412	sce_8195	7.00E-150	2.00E-147
SCE1572_9413	sce_8196	1.00E-154	5.00E-173
SCE1572_9414	sce_8198	0.00E+00	0.00E+00
SCE1572_9416	sce_8203	1.00E-69	1.00E-69
SCE1572_9417	sce_8204	0.00E+00	0.00E+00
SCE1572_9418	sce_8205	2.00E-72	2.00E-72
SCE1572_9419	sce_8206	4.00E-70	2.00E-70
SCE1572_9420	sce_8207	1.00E-61	4.00E-66
SCE1572_9421	sce_8208	0.00E+00	0.00E+00
SCE1572_9422	sce_8209	6.00E-53	7.00E-53
SCE1572_9423	sce_8210	9.00E-26	1.00E-24
SCE1572_9424	sce_8211	3.00E-66	5.00E-73
SCE1572_9425	sce_8212	2.00E-82	7.00E-89
SCE1572_9426	sce_8213	2.00E-105	1.00E-103
SCE1572_9427	sce_8214	1.00E-29	4.00E-24
SCE1572_9428	sce_8215	0.00E+00	0.00E+00
SCE1572_9430	sce_8219	1.00E-113	1.00E-123

SCE1572_9431	sce_8220	3.00E-104	1.00E-108
SCE1572_9432	sce_8221	2.00E-135	8.00E-126
SCE1572_9433	sce_8222	1.00E-24	1.00E-24
SCE1572_9434	sce_8223	1.00E-139	3.00E-136
SCE1572_9435	sce_8224	0.00E+00	0.00E+00
SCE1572_9436	sce_8225	0.00E+00	0.00E+00
SCE1572_9437	sce_8226	2.00E-35	2.00E-35
SCE1572_9439	sce_8227	2.00E-107	2.00E-117
SCE1572_9440	sce_8228	4.00E-153	4.00E-138
SCE1572_9441	sce_8229	4.00E-134	3.00E-133
SCE1572_9442	sce_8230	5.00E-53	4.00E-53
SCE1572_9443	sce_6393	2.00E-07	3.00E-12
SCE1572_9444	sce_1718	0.00E+00	0.00E+00
SCE1572_9446	sce_8233	6.00E-43	8.00E-50
SCE1572_9448	sce_8235	0.00E+00	5.00E-179
SCE1572_9449	sce_8236	8.00E-156	2.00E-155
SCE1572_945	sce_710	5.00E-72	3.00E-74
SCE1572_9450	sce_8237	5.00E-171	5.00E-171
SCE1572_9451	sce_8238	2.00E-15	5.00E-15
SCE1572_9452	sce_8239	2.00E-96	8.00E-86
SCE1572_9458	sce_8241	7.00E-58	4.00E-58
SCE1572_946	sce_711	6.00E-101	1.00E-91
SCE1572_9460	sce_8242	2.00E-138	2.00E-163
SCE1572_9461	sce_8243	6.00E-116	2.00E-113
SCE1572_9462	sce_8244	9.00E-54	3.00E-60
SCE1572_9464	sce_8245	3.00E-163	3.00E-161
SCE1572_9466	sce_8247	0.00E+00	9.00E-175
SCE1572_9467	sce_8248	3.00E-110	6.00E-155
SCE1572_9468	sce_8249	0.00E+00	0.00E+00
SCE1572_9469	sce_8250	0.00E+00	0.00E+00
SCE1572_9470	sce_8251	1.00E-67	2.00E-70
SCE1572_9473	sce_8252	3.00E-137	2.00E-139
SCE1572_9474	sce_8253	1.00E-179	4.00E-180
SCE1572_9475	sce_8254	7.00E-145	7.00E-149
SCE1572_9476	sce_8255	8.00E-77	1.00E-86
SCE1572_9478	sce_8265	6.00E-152	8.00E-151
SCE1572_9479	sce_8266	9.00E-132	6.00E-119
SCE1572_9480	sce_8267	5.00E-60	3.00E-47
SCE1572_9481	sce_8268	0.00E+00	0.00E+00
SCE1572_9482	sce_8269	1.00E-59	5.00E-69
SCE1572_9483	sce_8270	6.00E-171	0.00E+00
SCE1572_9484	sce_8271	3.00E-142	3.00E-142
SCE1572_9485	sce_8272	8.00E-73	7.00E-58
SCE1572_9486	sce_8273	2.00E-177	2.00E-177
SCE1572_9487	sce_2574	2.00E-09	3.00E-09
SCE1572_9488	sce_8274	1.00E-164	0.00E+00
SCE1572_9489	sce_8275	0.00E+00	0.00E+00
SCE1572_949	sce_2338	2.00E-62	4.00E-63
SCE1572_9490	sce_8276	4.00E-175	3.00E-178
SCE1572_9492	sce_8277	0.00E+00	0.00E+00
SCE1572_9494	sce_8280	4.00E-51	2.00E-62
SCE1572_9495	sce_8281	1.00E-172	2.00E-171
SCE1572_9496	sce_8282	3.00E-122	9.00E-126
SCE1572_9497	sce_8283	0.00E+00	0.00E+00
SCE1572_9498	sce_8284	8.00E-65	8.00E-65
SCE1572_9499	sce_8285	6.00E-69	6.00E-69
SCE1572_95	sce_84	9.00E-06	2.00E-06
SCE1572_9500	sce_8286	0.00E+00	0.00E+00
SCE1572_9501	sce_8287	0.00E+00	0.00E+00
SCE1572_9502	sce_8288	2.00E-127	1.00E-118
SCE1572_9503	sce_8290	5.00E-43	3.00E-43
SCE1572_9504	sce_8291	5.00E-156	1.00E-157
SCE1572_9505	sce_8292	3.00E-148	5.00E-160
SCE1572_9506	sce_8293	0.00E+00	0.00E+00
SCE1572_9507	sce_8294	2.00E-93	3.00E-102
SCE1572_9508	sce_8295	3.00E-149	6.00E-159
SCE1572_9509	sce_8296	5.00E-29	1.00E-26
SCE1572_9510	sce_8297	1.00E-15	5.00E-16
SCE1572_9512	sce_8299	0.00E+00	0.00E+00
SCE1572_9513	sce_8300	1.00E-114	3.00E-124
SCE1572_9514	sce_8301	4.00E-101	4.00E-101
SCE1572_9515	sce_8302	4.00E-179	0.00E+00
SCE1572_9516	sce_8303	2.00E-56	3.00E-50
SCE1572_9517	sce_8304	0.00E+00	0.00E+00
SCE1572_9518	sce_8305	1.00E-97	1.00E-117
SCE1572_9520	sce_3738	0.00E+00	0.00E+00

SCE1572_9523	sce_8308	4.00E-65	2.00E-65
SCE1572_9524	sce_8309	2.00E-84	1.00E-91
SCE1572_9526	sce_8310	0.00E+00	0.00E+00
SCE1572_9527	sce_8311	1.00E-26	5.00E-29
SCE1572_9528	sce_8312	0.00E+00	0.00E+00
SCE1572_9529	sce_8313	0.00E+00	0.00E+00
SCE1572_953	sce_716	1.00E-36	7.00E-37
SCE1572_9530	sce_8314	0.00E+00	0.00E+00
SCE1572_9531	sce_8315	0.00E+00	0.00E+00
SCE1572_9532	sce_8316	4.00E-69	2.00E-69
SCE1572_9533	sce_8317	0.00E+00	0.00E+00
SCE1572_9534	sce_8318	0.00E+00	0.00E+00
SCE1572_9536	sce_8319	0.00E+00	0.00E+00
SCE1572_9537	sce_8320	2.00E-23	7.00E-25
SCE1572_9538	sce_8321	2.00E-63	3.00E-63
SCE1572_9541	sce_8323	9.00E-110	1.00E-139
SCE1572_9542	sce_8324	2.00E-48	2.00E-50
SCE1572_9543	sce_8325	3.00E-131	1.00E-119
SCE1572_9547	sce_2969	0.00E+00	0.00E+00
SCE1572_9549	sce_8328	1.00E-128	1.00E-128
SCE1572_9550	sce_8329	3.00E-59	3.00E-59
SCE1572_9551	sce_8330	3.00E-107	5.00E-98
SCE1572_9554	sce_8631	6.00E-14	1.00E-14
SCE1572_9555	sce_8332	7.00E-93	7.00E-95
SCE1572_9556	sce_8333	0.00E+00	0.00E+00
SCE1572_9557	sce_8334	3.00E-131	1.00E-130
SCE1572_9560	sce_8336	5.00E-134	6.00E-135
SCE1572_9561	sce_8337	0.00E+00	0.00E+00
SCE1572_9563	sce_8352	3.00E-177	4.00E-169
SCE1572_9564	sce_8353	2.00E-97	5.00E-92
SCE1572_9565	sce_8354	3.00E-149	5.00E-166
SCE1572_9568	sce_136	0.00E+00	0.00E+00
SCE1572_957	sce_719	0.00E+00	0.00E+00
SCE1572_9570	sce_7661	0.00E+00	0.00E+00
SCE1572_9570	sce_10204	0.00E+00	0.00E+00
SCE1572_9570	sce_3917	0.00E+00	0.00E+00
SCE1572_9570	sce_6095	0.00E+00	0.00E+00
SCE1572_9570	sce_6093	0.00E+00	0.00E+00
SCE1572_9570	sce_6092	0.00E+00	0.00E+00
SCE1572_9570	sce_6094	0.00E+00	0.00E+00
SCE1572_9570	sce_6107	0.00E+00	0.00E+00
SCE1572_9570	sce_6535	0.00E+00	0.00E+00
SCE1572_9570	sce_388	0.00E+00	0.00E+00
SCE1572_9570	sce_9920	0.00E+00	0.00E+00
SCE1572_9570	sce_9557	0.00E+00	0.00E+00
SCE1572_9570	sce_3003	0.00E+00	0.00E+00
SCE1572_9570	sce_7640	0.00E+00	0.00E+00
SCE1572_9570	sce_5835	0.00E+00	0.00E+00
SCE1572_9570	sce_8602	0.00E+00	0.00E+00
SCE1572_9570	sce_8618	0.00E+00	0.00E+00
SCE1572_9570	sce_2065	0.00E+00	0.00E+00
SCE1572_9570	sce_4513	0.00E+00	0.00E+00
SCE1572_9570	sce_3085	0.00E+00	0.00E+00
SCE1572_9571	sce_8364	1.00E-154	8.00E-174
SCE1572_9572	sce_8365	1.00E-18	2.00E-19
SCE1572_9573	sce_8366	9.00E-57	3.00E-78
SCE1572_9574	sce_8367	4.00E-37	3.00E-32
SCE1572_9576	sce_8368	2.00E-56	2.00E-53
SCE1572_9577	sce_8369	1.00E-85	1.00E-68
SCE1572_9579	sce_4341	2.00E-169	4.00E-161
SCE1572_958	sce_720	3.00E-71	2.00E-74
SCE1572_959	sce_721	0.00E+00	0.00E+00
SCE1572_9597	sce_9238	0.00E+00	0.00E+00
SCE1572_9597	sce_4347	0.00E+00	0.00E+00
SCE1572_9598	sce_4593	0.00E+00	0.00E+00
SCE1572_960	sce_722	6.00E-88	6.00E-88
SCE1572_9601	sce_9564	0.00E+00	0.00E+00
SCE1572_9603	sce_8430	9.00E-110	1.00E-108
SCE1572_961	sce_723	7.00E-66	2.00E-65
SCE1572_9617	sce_8431	6.00E-139	8.00E-138
SCE1572_9618	sce_8432	3.00E-92	4.00E-102
SCE1572_962	sce_724	0.00E+00	0.00E+00
SCE1572_9621	sce_8435	0.00E+00	0.00E+00
SCE1572_9622	sce_8435	3.00E-176	0.00E+00
SCE1572_9623	sce_8436	0.00E+00	0.00E+00
SCE1572_9624	sce_8437	0.00E+00	0.00E+00

SCE1572_9625	sce_8438	0.00E+00	0.00E+00
SCE1572_9627	sce_2065	0.00E+00	0.00E+00
SCE1572_9627	sce_8618	0.00E+00	0.00E+00
SCE1572_9628	sce_1657	4.00E-159	2.00E-161
SCE1572_9629	sce_8441	0.00E+00	0.00E+00
SCE1572_963	sce_725	0.00E+00	0.00E+00
SCE1572_9631	sce_8443	6.00E-32	1.00E-31
SCE1572_9632	sce_8444	1.00E-109	3.00E-112
SCE1572_9633	sce_8445	0.00E+00	0.00E+00
SCE1572_9634	sce_8446	0.00E+00	0.00E+00
SCE1572_9635	sce_8447	3.00E-109	2.00E-113
SCE1572_9636	sce_8448	0.00E+00	0.00E+00
SCE1572_9637	sce_8449	1.00E-33	2.00E-28
SCE1572_9638	sce_8450	3.00E-80	1.00E-93
SCE1572_964	sce_726	2.00E-68	8.00E-71
SCE1572_9640	sce_8451	0.00E+00	0.00E+00
SCE1572_9640	sce_9407	0.00E+00	0.00E+00
SCE1572_9640	sce_4817	0.00E+00	0.00E+00
SCE1572_9640	sce_3765	0.00E+00	0.00E+00
SCE1572_9641	sce_8452	0.00E+00	0.00E+00
SCE1572_9642	sce_8453	5.00E-42	4.00E-56
SCE1572_9643	sce_8454	0.00E+00	0.00E+00
SCE1572_9644	sce_8455	1.00E-49	2.00E-50
SCE1572_9645	sce_8456	2.00E-41	2.00E-41
SCE1572_9646	sce_8457	0.00E+00	0.00E+00
SCE1572_9647	sce_8458	0.00E+00	0.00E+00
SCE1572_9648	sce_8459	2.00E-32	8.00E-25
SCE1572_9652	sce_10366	4.00E-92	3.00E-85
SCE1572_9653	sce_9503	0.00E+00	0.00E+00
SCE1572_9653	sce_10365	0.00E+00	0.00E+00
SCE1572_9653	sce_9908	0.00E+00	0.00E+00
SCE1572_9656	sce_8463	3.00E-101	8.00E-102
SCE1572_9657	sce_8464	3.00E-162	1.00E-157
SCE1572_9658	sce_8465	0.00E+00	0.00E+00
SCE1572_9659	sce_8466	0.00E+00	0.00E+00
SCE1572_9660	sce_8467	8.00E-121	3.00E-121
SCE1572_9661	sce_8469	2.00E-119	2.00E-119
SCE1572_9662	sce_8470	3.00E-74	6.00E-97
SCE1572_9663	sce_8471	1.00E-164	3.00E-157
SCE1572_9664	sce_8472	1.00E-42	4.00E-24
SCE1572_9665	sce_8473	3.00E-64	5.00E-62
SCE1572_9666	sce_8474	3.00E-24	5.00E-33
SCE1572_9667	sce_8475	2.00E-127	2.00E-127
SCE1572_9668	sce_8476	1.00E-139	3.00E-136
SCE1572_9669	sce_8477	2.00E-88	4.00E-95
SCE1572_9670	sce_8478	0.00E+00	0.00E+00
SCE1572_9671	sce_8479	3.00E-74	7.00E-73
SCE1572_9672	sce_8480	1.00E-166	4.00E-160
SCE1572_9673	sce_8481	1.00E-84	5.00E-89
SCE1572_9674	sce_8483	0.00E+00	0.00E+00
SCE1572_9675	sce_8484	0.00E+00	0.00E+00
SCE1572_9676	sce_8485	2.00E-28	8.00E-35
SCE1572_9677	sce_8486	3.00E-146	2.00E-134
SCE1572_9678	sce_8487	1.00E-121	5.00E-132
SCE1572_9679	sce_8488	3.00E-104	3.00E-103
SCE1572_968	sce_728	8.00E-79	2.00E-79
SCE1572_9680	sce_8489	0.00E+00	0.00E+00
SCE1572_9681	sce_8490	0.00E+00	0.00E+00
SCE1572_9682	sce_8491	4.00E-109	7.00E-115
SCE1572_9683	sce_8492	2.00E-109	2.00E-109
SCE1572_9684	sce_8493	0.00E+00	2.00E-172
SCE1572_9685	sce_8494	2.00E-32	3.00E-51
SCE1572_9686	sce_8495	2.00E-127	2.00E-134
SCE1572_9687	sce_8496	4.00E-168	4.00E-174
SCE1572_9688	sce_8497	0.00E+00	0.00E+00
SCE1572_9689	sce_8498	7.00E-129	9.00E-118
SCE1572_969	sce_729	2.00E-109	4.00E-117
SCE1572_9690	sce_8499	8.00E-49	7.00E-52
SCE1572_9691	sce_8500	0.00E+00	0.00E+00
SCE1572_9692	sce_8501	8.00E-76	7.00E-78
SCE1572_9693	sce_8502	0.00E+00	0.00E+00
SCE1572_9694	sce_8504	0.00E+00	0.00E+00
SCE1572_9696	sce_8505	0.00E+00	0.00E+00
SCE1572_9697	sce_8506	2.00E-108	2.00E-107
SCE1572_9698	sce_8507	2.00E-128	3.00E-129
SCE1572_9699	sce_8508	3.00E-145	8.00E-148

SCE1572_970	sce_730	2.00E-30	1.00E-30
SCE1572_9700	sce_8509	9.00E-76	9.00E-71
SCE1572_9702	sce_8510	2.00E-81	1.00E-80
SCE1572_9704	sce_8511	9.00E-122	2.00E-133
SCE1572_9705	sce_8512	1.00E-68	2.00E-61
SCE1572_9708	sce_8513	0.00E+00	0.00E+00
SCE1572_9709	sce_8514	0.00E+00	0.00E+00
SCE1572_971	sce_731	6.00E-107	9.00E-111
SCE1572_9710	sce_8515	0.00E+00	0.00E+00
SCE1572_9711	sce_8516	0.00E+00	0.00E+00
SCE1572_9713	sce_8517	2.00E-86	9.00E-91
SCE1572_9714	sce_8518	1.00E-51	1.00E-51
SCE1572_9715	sce_8520	3.00E-44	1.00E-74
SCE1572_9716	sce_8521	5.00E-55	3.00E-60
SCE1572_9717	sce_8522	4.00E-160	4.00E-160
SCE1572_9718	sce_8523	0.00E+00	0.00E+00
SCE1572_972	sce_732	0.00E+00	0.00E+00
SCE1572_9720	sce_8524	0.00E+00	0.00E+00
SCE1572_9721	sce_8525	9.00E-144	4.00E-146
SCE1572_9722	sce_8526	2.00E-175	1.00E-175
SCE1572_9725	sce_8528	1.00E-96	2.00E-78
SCE1572_9726	sce_8529	4.00E-63	2.00E-64
SCE1572_9728	sce_8530	5.00E-155	5.00E-155
SCE1572_9729	sce_8531	6.00E-141	9.00E-162
SCE1572_973	sce_733	1.00E-90	9.00E-91
SCE1572_9730	sce_8532	3.00E-94	2.00E-103
SCE1572_9731	sce_8533	0.00E+00	0.00E+00
SCE1572_9732	sce_8534	0.00E+00	0.00E+00
SCE1572_9733	sce_8535	1.00E-153	2.00E-148
SCE1572_9734	sce_8536	1.00E-100	2.00E-100
SCE1572_9735	sce_8537	4.00E-171	0.00E+00
SCE1572_9736	sce_8538	6.00E-28	1.00E-06
SCE1572_9737	sce_8539	0.00E+00	0.00E+00
SCE1572_9738	sce_8540	4.00E-128	8.00E-123
SCE1572_9740	sce_8541	2.00E-126	1.00E-137
SCE1572_9741	sce_8542	0.00E+00	0.00E+00
SCE1572_9742	sce_8543	0.00E+00	0.00E+00
SCE1572_9743	sce_8544	4.00E-179	4.00E-179
SCE1572_9744	sce_8545	5.00E-105	2.00E-137
SCE1572_9746	sce_8547	3.00E-34	1.00E-30
SCE1572_9747	sce_8548	3.00E-87	1.00E-104
SCE1572_9748	sce_8549	0.00E+00	0.00E+00
SCE1572_9749	sce_8550	2.00E-109	2.00E-109
SCE1572_975	sce_734	1.00E-87	9.00E-80
SCE1572_9750	sce_8551	1.00E-140	1.00E-148
SCE1572_9757	sce_8552	3.00E-111	8.00E-105
SCE1572_9758	sce_8553	0.00E+00	0.00E+00
SCE1572_9759	sce_8555	7.00E-147	7.00E-158
SCE1572_9760	sce_8556	0.00E+00	0.00E+00
SCE1572_9761	sce_8557	7.00E-42	8.00E-42
SCE1572_9762	sce_8558	6.00E-86	1.00E-120
SCE1572_9763	sce_7918	7.00E-83	1.00E-83
SCE1572_9764	sce_7917	2.00E-82	8.00E-141
SCE1572_9765	sce_8560	4.00E-109	5.00E-113
SCE1572_9766	sce_8561	0.00E+00	0.00E+00
SCE1572_9767	sce_8562	2.00E-103	2.00E-103
SCE1572_9768	sce_8564	4.00E-134	8.00E-106
SCE1572_9769	sce_8565	3.00E-47	5.00E-51
SCE1572_977	sce_736	0.00E+00	0.00E+00
SCE1572_977	sce_6501	0.00E+00	0.00E+00
SCE1572_9770	sce_8566	0.00E+00	0.00E+00
SCE1572_9771	sce_8567	2.00E-53	2.00E-53
SCE1572_9772	sce_8568	2.00E-63	2.00E-63
SCE1572_9773	sce_8569	3.00E-178	3.00E-178
SCE1572_9774	sce_8570	0.00E+00	0.00E+00
SCE1572_9776	sce_8571	2.00E-140	4.00E-133
SCE1572_9777	sce_8572	6.00E-29	8.00E-33
SCE1572_9778	sce_8574	0.00E+00	0.00E+00
SCE1572_9779	sce_8575	2.00E-18	4.00E-11
SCE1572_978	sce_737	3.00E-100	6.00E-107
SCE1572_9781	sce_8576	2.00E-71	2.00E-69
SCE1572_9782	sce_8577	1.00E-25	2.00E-32
SCE1572_9783	sce_8578	8.00E-158	1.00E-154
SCE1572_9784	sce_8580	6.00E-79	4.00E-71
SCE1572_9786	sce_8584	6.00E-145	7.00E-146
SCE1572_9787	sce_8585	2.00E-169	0.00E+00

SCE1572_9788	sce_8586	2.00E-125	2.00E-117
SCE1572_9789	sce_8587	4.00E-153	1.00E-149
SCE1572_979	sce_739	5.00E-23	4.00E-23
SCE1572_9790	sce_220	1.00E-40	9.00E-54
SCE1572_9792	sce_7989	4.00E-79	7.00E-82
SCE1572_9793	sce_8605	0.00E+00	0.00E+00
SCE1572_9794	sce_8606	0.00E+00	0.00E+00
SCE1572_9796	sce_8607	2.00E-101	3.00E-103
SCE1572_9797	sce_8608	2.00E-10	1.00E-10
SCE1572_9798	sce_8609	1.00E-21	5.00E-18
SCE1572_9799	sce_8610	1.00E-40	8.00E-41
SCE1572_980	sce_740	1.00E-123	1.00E-123
SCE1572_9805	sce_8612	1.00E-169	5.00E-178
SCE1572_9807	sce_8613	5.00E-41	4.00E-41
SCE1572_9808	sce_8614	0.00E+00	0.00E+00
SCE1572_9809	sce_8615	2.00E-85	5.00E-104
SCE1572_981	sce_741	0.00E+00	0.00E+00
SCE1572_9810	sce_8616	5.00E-170	1.00E-174
SCE1572_9812	sce_8618	0.00E+00	0.00E+00
SCE1572_9812	sce_2065	0.00E+00	0.00E+00
SCE1572_9812	sce_6535	0.00E+00	0.00E+00
SCE1572_9812	sce_6092	0.00E+00	0.00E+00
SCE1572_9812	sce_6095	0.00E+00	0.00E+00
SCE1572_9812	sce_3003	0.00E+00	0.00E+00
SCE1572_9812	sce_7661	0.00E+00	0.00E+00
SCE1572_9812	sce_9557	0.00E+00	0.00E+00
SCE1572_9812	sce_10204	0.00E+00	0.00E+00
SCE1572_9812	sce_5835	0.00E+00	0.00E+00
SCE1572_9812	sce_388	0.00E+00	0.00E+00
SCE1572_9812	sce_7640	0.00E+00	0.00E+00
SCE1572_9812	sce_4513	0.00E+00	0.00E+00
SCE1572_9813	sce_8619	5.00E-53	6.00E-48
SCE1572_9814	sce_8620	8.00E-58	2.00E-52
SCE1572_9816	sce_8621	1.00E-112	4.00E-122
SCE1572_9819	sce_3896	4.00E-08	5.00E-14
SCE1572_9820	sce_8622	4.00E-110	7.00E-105
SCE1572_9821	sce_8623	8.00E-129	4.00E-131
SCE1572_9822	sce_8624	3.00E-129	5.00E-132
SCE1572_9823	sce_7836	1.00E-66	2.00E-60
SCE1572_983	sce_742	0.00E+00	0.00E+00
SCE1572_9835	sce_7133	1.00E-84	4.00E-87
SCE1572_9836	sce_8655	2.00E-151	2.00E-151
SCE1572_9837	sce_8650	5.00E-118	2.00E-118
SCE1572_984	sce_744	1.00E-159	1.00E-159
SCE1572_9846	sce_7504	5.00E-14	7.00E-06
SCE1572_9847	sce_8635	0.00E+00	0.00E+00
SCE1572_9849	sce_8638	8.00E-156	4.00E-157
SCE1572_985	sce_745	0.00E+00	0.00E+00
SCE1572_9850	sce_6016	6.00E-14	5.00E-14
SCE1572_9853	sce_8645	2.00E-168	2.00E-171
SCE1572_9854	sce_8657	0.00E+00	0.00E+00
SCE1572_9855	sce_8658	0.00E+00	0.00E+00
SCE1572_9856	sce_2572	9.00E-90	8.00E-90
SCE1572_9857	sce_8667	0.00E+00	0.00E+00
SCE1572_9858	sce_8668	7.00E-77	3.00E-98
SCE1572_9859	sce_8669	0.00E+00	0.00E+00
SCE1572_986	sce_747	0.00E+00	0.00E+00
SCE1572_9860	sce_6222	0.00E+00	0.00E+00
SCE1572_9860	sce_4708	0.00E+00	0.00E+00
SCE1572_9863	sce_10287	1.00E-10	2.00E-12
SCE1572_9864	sce_8675	1.00E-100	7.00E-101
SCE1572_9865	sce_8676	0.00E+00	0.00E+00
SCE1572_9866	sce_8677	0.00E+00	0.00E+00
SCE1572_9867	sce_8678	2.00E-119	4.00E-104
SCE1572_9868	sce_8679	1.00E-125	6.00E-142
SCE1572_9869	sce_8680	1.00E-166	1.00E-164
SCE1572_987	sce_749	2.00E-180	0.00E+00
SCE1572_9870	sce_8681	1.00E-69	3.00E-68
SCE1572_9871	sce_8682	2.00E-161	0.00E+00
SCE1572_9872	sce_8685	2.00E-111	2.00E-111
SCE1572_9873	sce_9440	9.00E-94	1.00E-93
SCE1572_9874	sce_9439	4.00E-132	6.00E-123
SCE1572_9876	sce_8692	1.00E-34	1.00E-35
SCE1572_9880	sce_5093	2.00E-32	9.00E-20
SCE1572_9882	sce_2092	3.00E-100	3.00E-100
SCE1572_9885	sce_8696	7.00E-154	2.00E-154

SCE1572_9886	sce_8697	0.00E+00	0.00E+00
SCE1572_9887	sce_8698	2.00E-122	2.00E-118
SCE1572_9888	sce_8699	0.00E+00	0.00E+00
SCE1572_9889	sce_8700	0.00E+00	0.00E+00
SCE1572_9890	sce_8701	0.00E+00	0.00E+00
SCE1572_9892	sce_8703	5.00E-07	1.00E-11
SCE1572_9893	sce_8704	0.00E+00	0.00E+00
SCE1572_9894	sce_8705	2.00E-06	8.00E-15
SCE1572_9895	sce_8706	0.00E+00	0.00E+00
SCE1572_9898	sce_8708	2.00E-83	2.00E-83
SCE1572_9899	sce_8709	6.00E-79	8.00E-80
SCE1572_990	sce_750	0.00E+00	0.00E+00
SCE1572_9900	sce_8710	0.00E+00	0.00E+00
SCE1572_9902	sce_8712	4.00E-136	9.00E-127
SCE1572_9903	sce_8713	0.00E+00	0.00E+00
SCE1572_9904	sce_7561	1.00E-30	1.00E-31
SCE1572_9907	sce_8718	0.00E+00	0.00E+00
SCE1572_9908	sce_8719	0.00E+00	0.00E+00
SCE1572_9910	sce_8720	8.00E-91	6.00E-93
SCE1572_9911	sce_8721	1.00E-140	1.00E-142
SCE1572_9912	sce_8722	4.00E-47	5.00E-44
SCE1572_9914	sce_8724	8.00E-26	8.00E-29
SCE1572_9915	sce_8725	0.00E+00	0.00E+00
SCE1572_9916	sce_8726	0.00E+00	0.00E+00
SCE1572_9917	sce_8727	3.00E-97	7.00E-97
SCE1572_992	sce_752	0.00E+00	0.00E+00
SCE1572_9920	sce_3794	0.00E+00	0.00E+00
SCE1572_9921	sce_8730	0.00E+00	0.00E+00
SCE1572_9923	sce_8731	0.00E+00	0.00E+00
SCE1572_9924	sce_8732	0.00E+00	0.00E+00
SCE1572_9924	sce_5721	0.00E+00	0.00E+00
SCE1572_9926	sce_8733	1.00E-61	9.00E-63
SCE1572_9927	sce_8734	0.00E+00	0.00E+00
SCE1572_9928	sce_8735	3.00E-50	6.00E-40
SCE1572_993	sce_753	4.00E-105	3.00E-116
SCE1572_9930	sce_8737	0.00E+00	0.00E+00
SCE1572_9931	sce_8738	4.00E-64	5.00E-71
SCE1572_9935	sce_8739	1.00E-34	2.00E-42
SCE1572_9936	sce_8740	6.00E-96	1.00E-95
SCE1572_9939	sce_6035	1.00E-71	3.00E-80
SCE1572_9942	sce_8792	2.00E-167	2.00E-167
SCE1572_9943	sce_8791	3.00E-70	2.00E-69
SCE1572_9946	sce_3894	5.00E-20	4.00E-16
SCE1572_9949	sce_6031	2.00E-46	2.00E-46
SCE1572_9950	sce_8747	2.00E-160	3.00E-150
SCE1572_9951	sce_8748	1.00E-110	1.00E-102
SCE1572_9952	sce_8750	2.00E-124	1.00E-133
SCE1572_9953	sce_7598	0.00E+00	0.00E+00
SCE1572_9954	sce_7599	0.00E+00	0.00E+00
SCE1572_9955	sce_7600	5.00E-150	2.00E-144
SCE1572_9956	sce_7601	2.00E-100	2.00E-100
SCE1572_9957	sce_7602	3.00E-22	3.00E-22
SCE1572_9958	sce_7603	8.00E-62	1.00E-65
SCE1572_9959	sce_9664	0.00E+00	0.00E+00
SCE1572_996	sce_754	0.00E+00	0.00E+00
SCE1572_9960	sce_9662	0.00E+00	0.00E+00
SCE1572_9963	sce_8755	0.00E+00	0.00E+00
SCE1572_9963	sce_8753	0.00E+00	0.00E+00
SCE1572_9963	sce_7818	0.00E+00	0.00E+00
SCE1572_9963	sce_7815	0.00E+00	0.00E+00
SCE1572_9964	sce_8755	0.00E+00	0.00E+00
SCE1572_9964	sce_7818	0.00E+00	0.00E+00
SCE1572_9964	sce_8753	0.00E+00	0.00E+00
SCE1572_9964	sce_7814	0.00E+00	0.00E+00
SCE1572_9964	sce_7815	0.00E+00	0.00E+00
SCE1572_9966	sce_3146	3.00E-10	4.00E-12
SCE1572_9967	sce_8760	0.00E+00	8.00E-173
SCE1572_9968	sce_8762	2.00E-43	6.00E-52
SCE1572_9969	sce_8763	1.00E-69	2.00E-75
SCE1572_997	sce_755	3.00E-115	6.00E-112
SCE1572_9970	sce_8765	6.00E-41	6.00E-44
SCE1572_9971	sce_8766	5.00E-133	6.00E-133
SCE1572_9972	sce_8767	3.00E-111	2.00E-107
SCE1572_9973	sce_8768	0.00E+00	0.00E+00
SCE1572_9974	sce_8770	9.00E-109	5.00E-93
SCE1572_9975	sce_8771	0.00E+00	0.00E+00

SCE1572_9977	sce_8773	8.00E-165	2.00E-167
SCE1572_998	sce_756	9.00E-149	3.00E-153
SCE1572_9980	sce_8776	4.00E-101	1.00E-110
SCE1572_9981	sce_8777	0.00E+00	0.00E+00
SCE1572_9982	sce_8778	0.00E+00	0.00E+00
SCE1572_9983	sce_7926	0.00E+00	0.00E+00
SCE1572_9984	sce_8784	9.00E-157	5.00E-157
SCE1572_9985	sce_5809	9.00E-49	2.00E-52
SCE1572_9986	sce_5808	4.00E-152	4.00E-146
SCE1572_9987	sce_8796	2.00E-137	7.00E-137
SCE1572_9988	sce_8797	1.00E-57	2.00E-56
SCE1572_9989	sce_8798	0.00E+00	0.00E+00
SCE1572_9990	sce_8799	3.00E-71	2.00E-109
SCE1572_9991	sce_8803	0.00E+00	0.00E+00
SCE1572_9996	sce_8812	0.00E+00	3.00E-164
SCE1572_9997	sce_8818	0.00E+00	0.00E+00
SCE1572_9998	sce_8820	2.00E-122	1.00E-124
SCE1572_9999	sce_8821	1.00E-127	5.00E-135

TableS8 A comparison of KEGG categories for So0157-2 and So ce56

Presence*	Category	Subcategory	Subsystem	Role	Organism A	Active in organism A	Organism B	Active in organism B
A and B	Amino Acids and Derivatives	Alanine, serine, and glycine	Alanine biosynthesis	Alanine racemase (EC 5.1.1.1)	SCE1572_9334	yes	sce_8113	yes
A and B	Amino Acids and Derivatives	Alanine, serine, and glycine	Alanine biosynthesis	Branched-chain amino acid aminotransferase (EC 2.6.1.42)	SCE1572_354	yes	sce_195	yes
A and B	Amino Acids and Derivatives	Alanine, serine, and glycine	Alanine biosynthesis	Chaperone protein HscA	SCE1572_9421	yes	sce_8208	yes
A and B	Amino Acids and Derivatives	Alanine, serine, and glycine	Alanine biosynthesis	Cysteine desulfurase (EC 2.8.1.7)	SCE1572_1872	yes	sce_1345	yes
A and B	Amino Acids and Derivatives	Alanine, serine, and glycine	Alanine biosynthesis	Cysteine desulfurase (EC 2.8.1.7), IscS subfamily	SCE1572_9417	yes	sce_8204	yes
A and B	Amino Acids and Derivatives	Alanine, serine, and glycine	Alanine biosynthesis	Cysteine desulfurase (EC 2.8.1.7), SufS subfamily	SCE1572_10250	yes	sce_5192	yes
A and B	Amino Acids and Derivatives	Alanine, serine, and glycine	Alanine biosynthesis	Ferredoxin, 2Fe-2S	SCE1572_9111	yes	sce_7893	yes
A and B	Amino Acids and Derivatives	Alanine, serine, and glycine	Alanine biosynthesis	Iron binding protein IscA for iron-sulfur cluster assembly	SCE1572_9419	yes	sce_8206	yes
A and B	Amino Acids and Derivatives	Alanine, serine, and glycine	Alanine biosynthesis	Iron-sulfur cluster assembly scaffold protein IscU	SCE1572_9418	yes	sce_8205	yes
A and B	Amino Acids and Derivatives	Alanine, serine, and glycine	Alanine biosynthesis	Iron-sulfur cluster regulator IscR	SCE1572_7588	yes	sce_6444	yes
A and B	Amino Acids and Derivatives	Alanine, serine, and glycine	Alanine biosynthesis	Valine--pyruvate aminotransferase (EC 2.6.1.66)	SCE1572_1439	yes	sce_6322	yes
A and B	Amino Acids and Derivatives	Alanine, serine, and glycine	Glycine Biosynthesis	2-amino-3-ketobutyrate coenzyme A ligase (EC 2.3.1.29)	SCE1572_9365	yes	sce_8145	yes
A and B	Amino Acids and Derivatives	Alanine, serine, and glycine	Glycine Biosynthesis	L-threonine 3-dehydrogenase (EC 1.1.1.103)	SCE1572_9364	yes	sce_8144	yes
A and B	Amino Acids and Derivatives	Alanine, serine, and glycine	Glycine Biosynthesis	Low-specificity L-threonine aldolase (EC 4.1.2.5)	SCE1572_2736	yes	sce_2275	yes
A and B	Amino Acids and Derivatives	Alanine, serine, and glycine	Glycine Biosynthesis	Serine hydroxymethyltransferase (EC 2.1.2.1)	SCE1572_8655	yes	sce_7370	yes
A and B	Amino Acids and Derivatives	Alanine, serine, and glycine	Glycine and Serine Utilization	2-amino-3-ketobutyrate coenzyme A ligase (EC 2.3.1.29)	SCE1572_9365	yes	sce_8145	yes
A and B	Amino Acids and Derivatives	Alanine, serine, and glycine	Glycine and Serine Utilization	Aminomethyltransferase (glycine cleavage system T protein) (EC 2.1.2.	SCE1572_2704	yes	sce_2242	yes
A and B	Amino Acids and Derivatives	Alanine, serine, and glycine	Glycine and Serine Utilization	Cystathionine beta-synthase (EC 4.2.1.22)	SCE1572_693	yes	sce_496	yes
A and B	Amino Acids and Derivatives	Alanine, serine, and glycine	Glycine and Serine Utilization	Cystathionine gamma-lyase (EC 4.4.1.1)	SCE1572_164	yes	sce_6912	yes
A and B	Amino Acids and Derivatives	Alanine, serine, and glycine	Glycine and Serine Utilization	D-3-phosphoglycerate dehydrogenase (EC 1.1.1.95)	SCE1572_1389	yes	sce_1692	yes
A and B	Amino Acids and Derivatives	Alanine, serine, and glycine	Glycine and Serine Utilization	Glycine cleavage system H protein	SCE1572_2703	yes	sce_2241	yes
A and B	Amino Acids and Derivatives	Alanine, serine, and glycine	Glycine and Serine Utilization	Glycine dehydrogenase [decarboxylating] (glycine cleavage system P1 [	SCE1572_2702	yes	sce_2240	yes
A and B	Amino Acids and Derivatives	Alanine, serine, and glycine	Glycine and Serine Utilization	Glycine dehydrogenase [decarboxylating] (glycine cleavage system P2 [	SCE1572_8088	yes	sce_10237	yes
A and B	Amino Acids and Derivatives	Alanine, serine, and glycine	Glycine and Serine Utilization	L-serine dehydratase (EC 4.3.1.17)	SCE1572_2497	yes	sce_2075	yes
A and B	Amino Acids and Derivatives	Alanine, serine, and glycine	Glycine and Serine Utilization	Phosphoserine aminotransferase (EC 2.6.1.52)	SCE1572_5915	yes	sce_4919	yes
A and B	Amino Acids and Derivatives	Alanine, serine, and glycine	Glycine and Serine Utilization	Phosphoserine phosphatase (EC 3.1.3.3)	SCE1572_985	yes	sce_745	yes
A and B	Amino Acids and Derivatives	Alanine, serine, and glycine	Glycine and Serine Utilization	Serine hydroxymethyltransferase (EC 2.1.2.1)	SCE1572_8655	yes	sce_7370	yes
A and B	Amino Acids and Derivatives	Alanine, serine, and glycine	Glycine and Serine Utilization	Seryl-tRNA synthetase (EC 6.1.1.11)	SCE1572_10462	yes	sce_9101	yes
A and B	Amino Acids and Derivatives	Alanine, serine, and glycine	Glycine and Serine Utilization	Threonine dehydratase, catabolic (EC 4.3.1.19)	SCE1572_5116	yes	sce_4304	yes
A and B	Amino Acids and Derivatives	Alanine, serine, and glycine	Glycine cleavage system	Aminomethyltransferase (glycine cleavage system T protein) (EC 2.1.2.	SCE1572_2704	yes	sce_2242	yes
A and B	Amino Acids and Derivatives	Alanine, serine, and glycine	Glycine cleavage system	Glycine cleavage system H protein	SCE1572_2703	yes	sce_2241	yes
A and B	Amino Acids and Derivatives	Alanine, serine, and glycine	Glycine cleavage system	Glycine cleavage system transcriptional activator GcvA	SCE1572_1245	yes	sce_5041	yes
A and B	Amino Acids and Derivatives	Alanine, serine, and glycine	Glycine cleavage system	Glycine dehydrogenase [decarboxylating] (glycine cleavage system P1 [	SCE1572_2702	yes	sce_2240	yes
A and B	Amino Acids and Derivatives	Alanine, serine, and glycine	Glycine cleavage system	Glycine dehydrogenase [decarboxylating] (glycine cleavage system P2 [	SCE1572_8088	yes	sce_10237	yes
A and B	Amino Acids and Derivatives	Alanine, serine, and glycine	Serine Biosynthesis	D-3-phosphoglycerate dehydrogenase (EC 1.1.1.95)	SCE1572_1389	yes	sce_1692	yes
A and B	Amino Acids and Derivatives	Alanine, serine, and glycine	Serine Biosynthesis	Phosphoserine aminotransferase (EC 2.6.1.52)	SCE1572_5915	yes	sce_4919	yes
A and B	Amino Acids and Derivatives	Alanine, serine, and glycine	Serine Biosynthesis	Phosphoserine phosphatase (EC 3.1.3.3)	SCE1572_985	yes	sce_745	yes
A and B	Amino Acids and Derivatives	Alanine, serine, and glycine	Serine Biosynthesis	Serine hydroxymethyltransferase (EC 2.1.2.1)	SCE1572_8655	yes	sce_7370	yes
B	Amino Acids and Derivatives	Alanine, serine, and glycine	Glycine cleavage system	Glycine cleavage system transcriptional antiactivator GcvR		no	sce_9889	yes
A and B	Amino Acids and Derivatives	Arginine; urea cycle, polyamines	Arginine Biosynthesis -- gjo	Acetylglutamate kinase (EC 2.7.2.8)	SCE1572_92	yes	sce_81	yes
A and B	Amino Acids and Derivatives	Arginine; urea cycle, polyamines	Arginine Biosynthesis -- gjo	Argininosuccinate lyase (EC 4.3.2.1)	SCE1572_2405	yes	sce_1959	yes
A and B	Amino Acids and Derivatives	Arginine; urea cycle, polyamines	Arginine Biosynthesis -- gjo	Argininosuccinate synthase (EC 6.3.4.5)	SCE1572_6795	yes	sce_5654	yes
A and B	Amino Acids and Derivatives	Arginine; urea cycle, polyamines	Arginine Biosynthesis -- gjo	Glutamate N-acetyltransferase (EC 2.3.1.35)	SCE1572_3887	yes	sce_3211	yes
A and B	Amino Acids and Derivatives	Arginine; urea cycle, polyamines	Arginine Biosynthesis -- gjo	N-acetyl-gamma-glutamyl-phosphate reductase (EC 1.2.1.38)	SCE1572_6683	yes	sce_5564	yes
A and B	Amino Acids and Derivatives	Arginine; urea cycle, polyamines	Arginine Biosynthesis -- gjo	N-acetylglutamate synthase (EC 2.3.1.1)	SCE1572_3887	yes	sce_3211	yes
A and B	Amino Acids and Derivatives	Arginine; urea cycle, polyamines	Arginine Biosynthesis -- gjo	N-acetylornithine carbamoyltransferase (EC 2.1.3.9)	SCE1572_93	yes	sce_82	yes
A and B	Amino Acids and Derivatives	Arginine; urea cycle, polyamines	Arginine Biosynthesis -- gjo	N-succinyl-L,L-diaminopimelate desuccinylase (EC 3.5.1.18)	SCE1572_3985	yes	sce_3316	yes
A and B	Amino Acids and Derivatives	Arginine; urea cycle, polyamines	Arginine Biosynthesis -- gjo	Ornithine carbamoyltransferase (EC 2.1.3.3)	SCE1572_9212	yes	sce_8013	yes
A and B	Amino Acids and Derivatives	Arginine; urea cycle, polyamines	Arginine Biosynthesis extended	Acetylglutamate kinase (EC 2.7.2.8)	SCE1572_92	yes	sce_81	yes
A and B	Amino Acids and Derivatives	Arginine; urea cycle, polyamines	Arginine Biosynthesis extended	Argininosuccinate lyase (EC 4.3.2.1)	SCE1572_2405	yes	sce_1959	yes
A and B	Amino Acids and Derivatives	Arginine; urea cycle, polyamines	Arginine Biosynthesis extended	Argininosuccinate synthase (EC 6.3.4.5)	SCE1572_6795	yes	sce_5654	yes
A and B	Amino Acids and Derivatives	Arginine; urea cycle, polyamines	Arginine Biosynthesis extended	Glutamate N-acetyltransferase (EC 2.3.1.35)	SCE1572_3887	yes	sce_3211	yes
A and B	Amino Acids and Derivatives	Arginine; urea cycle, polyamines	Arginine Biosynthesis extended	N-acetyl-gamma-glutamyl-phosphate reductase (EC 1.2.1.38)	SCE1572_6683	yes	sce_5564	yes
A and B	Amino Acids and Derivatives	Arginine; urea cycle, polyamines	Arginine Biosynthesis extended	N-acetylglutamate synthase (EC 2.3.1.1)	SCE1572_3887	yes	sce_3211	yes
A and B	Amino Acids and Derivatives	Arginine; urea cycle, polyamines	Arginine Biosynthesis extended	N-acetylornithine carbamoyltransferase (EC 2.1.3.9)	SCE1572_93	yes	sce_82	yes
A and B	Amino Acids and Derivatives	Arginine; urea cycle, polyamines	Arginine Biosynthesis extended	N-succinyl-L,L-diaminopimelate desuccinylase (EC 3.5.1.18)	SCE1572_3985	yes	sce_3316	yes
A and B	Amino Acids and Derivatives	Arginine; urea cycle, polyamines	Arginine Biosynthesis extended	Ornithine carbamoyltransferase (EC 2.1.3.3)	SCE1572_9212	yes	sce_8013	yes
A and B	Amino Acids and Derivatives	Arginine; urea cycle, polyamines	Arginine and Ornithine Degradation	Agmatinase (EC 3.5.3.11)	SCE1572_47	yes	sce_46	yes
A and B	Amino Acids and Derivatives	Arginine; urea cycle, polyamines	Arginine and Ornithine Degradation	Arginine N-succinyltransferase (EC 2.3.1.109)	SCE1572_2280	yes	sce_1826	yes
A and B	Amino Acids and Derivatives	Arginine; urea cycle, polyamines	Arginine and Ornithine Degradation	Arginine decarboxylase (EC 4.1.1.19)	SCE1572_8115	yes	sce_93	yes
A and B	Amino Acids and Derivatives	Arginine; urea cycle, polyamines	Arginine and Ornithine Degradation	Biosynthetic arginine decarboxylase (EC 4.1.1.19)	SCE1572_3252	yes	sce_2748	yes
A and B	Amino Acids and Derivatives	Arginine; urea cycle, polyamines	Arginine and Ornithine Degradation	Delta-1-pyrroline-5-carboxylate dehydrogenase (EC 1.5.1.12)	SCE1572_6031	yes	sce_4984	yes
A and B	Amino Acids and Derivatives	Arginine; urea cycle, polyamines	Arginine and Ornithine Degradation	N-carbamoylputrescine amidase (3.5.1.53)	SCE1572_8402	yes	sce_7162	yes
A and B	Amino Acids and Derivatives	Arginine; urea cycle, polyamines	Arginine and Ornithine Degradation	NADP-specific glutamate dehydrogenase (EC 1.4.1.4)	SCE1572_1504	yes	sce_6937	yes
A and B	Amino Acids and Derivatives	Arginine; urea cycle, polyamines	Arginine and Ornithine Degradation	Ornithine carbamoyltransferase (EC 2.1.3.3)	SCE1572_9212	yes	sce_8013	yes
A and B	Amino Acids and Derivatives	Arginine; urea cycle, polyamines	Arginine and Ornithine Degradation	Ornithine cyclodeaminase (EC 4.3.1.12)	SCE1572_3173	yes	sce_2667	yes
A and B	Amino Acids and Derivatives	Arginine; urea cycle, polyamines	Arginine and Ornithine Degradation	Succinylglutamic semialdehyde dehydrogenase (EC 1.2.1.71)	SCE1572_1008	yes	sce_761	yes
A and B	Amino Acids and Derivatives	Arginine; urea cycle, polyamines	Arginine and Ornithine Degradation	Succinylornithine transaminase (EC 2.6.1.81)	SCE1572_5	yes	sce_5	yes
A and B	Amino Acids and Derivatives	Arginine; urea cycle, polyamines	Polyamine Metabolism	ABC transporter, periplasmic spermidine putrescine-binding protein Pot	SCE1572_11387	yes	sce_10223	yes
A and B	Amino Acids and Derivatives	Arginine; urea cycle, polyamines	Polyamine Metabolism	Agmatinase (EC 3.5.3.11)	SCE1572_47	yes	sce_46	yes
A and B	Amino Acids and Derivatives	Arginine; urea cycle, polyamines	Polyamine Metabolism	Arginine decarboxylase (EC 4.1.1.19)	SCE1572_8115	yes	sce_93	yes
A and B	Amino Acids and Derivatives	Arginine; urea cycle, polyamines	Polyamine Metabolism	Biosynthetic arginine decarboxylase (EC 4.1.1.19)	SCE1572_3252	yes	sce_2748	yes
A and B	Amino Acids and Derivatives	Arginine; urea cycle, polyamines	Polyamine Metabolism	N-carbamoylputrescine amidase (3.5.1.53)	SCE1572_8402	yes	sce_7162	yes
A and B	Amino Acids and Derivatives	Arginine; urea cycle, polyamines	Polyamine Metabolism	Putrescine transport ATP-binding protein PotA (TC 3.A.1.11.1)	SCE1572_11392	yes	sce_10231	yes
A and B	Amino Acids and Derivatives	Arginine; urea cycle, polyamines	Polyamine Metabolism	S-adenosylmethionine decarboxylase proenzyme (EC 4.1.1.50), prokar	SCE1572_7524	yes	sce_6348	yes
A and B	Amino Acids and Derivatives	Arginine; urea cycle, polyamines	Polyamine Metabolism	Spermidine Putrescine ABC transporter permease component PotB (TC	SCE1572_11391	yes	sce_10230	yes
A and B	Amino Acids and Derivatives	Arginine; urea cycle, polyamines	Polyamine Metabolism	Spermidine Putrescine ABC transporter permease component potC (TC	SCE1572_11390	yes	sce_10229	yes
A and B	Amino Acids and Derivatives	Arginine; urea cycle, polyamines	Polyamine Metabolism	Spermidine synthase (EC 2.5.1.16)	SCE1572_507	yes	sce_1673	yes
A and B	Amino Acids and Derivatives	Arginine; urea cycle, polyamines	Urea carboxylase and Allophanate hydrolase cluster	Urea carboxylase (EC 6.3.4.6)	SCE1572_6544	yes	sce_5500	yes
A and B	Amino Acids and Derivatives	Arginine; urea cycle, polyamines	Urea decomposition	Nickel transporter UreH	SCE1572_1803	yes	sce_6510	yes
A and B	Amino Acids and Derivatives	Arginine; urea cycle, polyamines	Urea decomposition	Urea ABC transporter, ATPase protein UrtD	SCE1572_7464	yes	sce_6249	yes
A and B	Amino Acids and Derivatives	Arginine; urea cycle, polyamines	Urea decomposition	Urea ABC transporter, ATPase protein UrtE	SCE1572_7465	yes	sce_6250	yes
A and B	Amino Acids and Derivatives	Arginine; urea cycle, polyamines	Urea decomposition	Urea ABC transporter, permease protein UrtB	SCE1572_7462	yes	sce_6247	yes
A and B	Amino Acids and Derivatives	Arginine; urea cycle, polyamines	Urea decomposition	Urea ABC transporter, permease protein UrtC	SCE1572_7463	yes	sce_6248	yes
A and B	Amino Acids and Derivatives	Arginine; urea cycle, polyamines	Urea decomposition	Urea ABC transporter, substrate binding protein UrtA	SCE1572_7461	yes	sce_6246	yes
A and B	Amino Acids and Derivatives	Arginine; urea cycle, polyamines	Urea decomposition	Urea carboxylase (EC 6.3.4.6)	SCE1572_6544	yes	sce_5500	yes
A and B	Amino Acids and Derivatives	Arginine; urea cycle, polyamines	Urea decomposition	Urea carboxylase-related ABC transporter, ATPase protein	SCE1572_4525	yes	sce_3784	yes
A and B	Amino Acids and Derivatives	Arginine; urea cycle, polyamines	Urea decomposition	Urea carboxylase-related ABC transporter, periplasmic substrate-bindin	SCE1572_4524	yes	sce_2159	yes
A and B	Amino Acids and Derivatives	Arginine; urea cycle, polyamines	Urea decomposition	Urea carboxylase-related ABC transporter, permease protein	SCE1572_4526	yes	sce_3785	yes
A and B	Amino Acids and Derivatives	Arginine; urea cycle, polyamines	Urea decomposition	Urea carboxylase-related amino acid permease	SCE1572_6545	yes	sce_5501	yes
A and B	Amino Acids and Derivatives	Arginine; urea cycle, polyamines	Urea decomposition	Urea carboxylase-related aminomethyltransferase (EC 2.1.2.10)	SCE1572_6542	yes	sce_5498	yes
A and B	Amino Acids and Derivatives	Arginine; urea cycle, polyamines	Urea decomposition	Urease accessory protein UreD	SCE1572_3069	yes	sce_7327	yes
A and B	Amino Acids and Derivatives	Arginine; urea cycle, polyamines	Urea decomposition	Urease accessory protein UreF	SCE1572_3073	yes	sce_7330	yes
A and B	Amino Acids and Derivatives	Arginine; urea cycle, polyamines	Urea decomposition	Urease accessory protein UreG	SCE1572_3074	yes	sce_7331	yes
A and B	Amino Acids and Derivatives	Arginine; urea cycle, polyamines	Urea decomposition	Urease alpha subunit (EC 3.5.1.5)	SCE1572_3071	yes	sce_7329	yes
A and B	Amino Acids and Derivatives	Arginine; urea cycle, polyamines	Urea decomposition	Urease gamma subunit (EC 3.5.1.5)	SCE1572_3070	yes	sce_7328	yes
A	Amino Acids and Derivatives	Arginine; urea cycle, polyamines	Arginine Biosynthesis -- gjo	Acetylornithine aminotransferase (EC 2.6.1.11)	SCE1572_10277	yes		no
A	Amino Acids and Derivatives	Arginine; urea cycle, polyamines	Arginine Biosynthesis -- gjo	Acetylornithine deacetylase (EC 3.5.1.16)	SCE1572_5459	yes		no
A	Amino Acids and Derivatives	Arginine; urea cycle, polyamines	Cyanophycin Metabolism	Cyanophycin synthase (EC 6.3.2.29)(EC 6.3.2.30)	SCE1572_9183	yes		no
A	Amino Acids and Derivatives	Arginine; urea cycle, polyamines	Cyanophycin Metabolism	Cyanophycinase (EC 3.4.15.6)	SCE1572_9182	yes		no
B	Amino Acids and Derivatives	Arginine; urea cycle, polyamines	Arginine and Ornithine Degradation	Ornithine decarboxylase (EC 4.1.1.17)		no	sce_93	yes
A and B	Amino Acids and Derivatives	Aromatic amino acids and derivatives	Aromatic amino acid degradation	4-hydroxyphenylpyruvate dioxygenase (EC 1.13.11.27)	SCE1572_4318	yes	sce_5475	yes
A and B	Amino Acids and Derivatives	Aromatic amino acids and derivatives	Aromatic amino acid degradation	5-carboxymethyl-2-hydroxymuconate semialdehyde dehydrogenase (E	SCE1572_11413	yes	sce_10270	yes
A and B	Amino Acids and Derivatives	Aromatic amino acids and derivatives	Aromatic amino acid degradation	Aromatic-L-amino-acid decarboxylase (EC 4.1.1.28)	SCE1572_1105	yes	sce_2563	yes
A and B	Amino Acids and Derivatives	Aromatic amino acids and derivatives	Aromatic amino acid degradation	Kynurenine formamidase, bacterial (EC 3.5.1.9)	SCE1572_4334	yes	sce_1451	yes
A and B	Amino Acids and Derivatives	Aromatic amino acids and derivatives	Aromatic amino acid degradation	Tryptophan 2,3-dioxygenase (EC 1.13.11.11)	SCE1572_10736	yes	sce_9394	yes
A and B	Amino Acids and Derivatives	Aromatic amino acids and derivatives	Chorismate: Intermediate for synthesis of Tryptophan, PAPA a	Anthranilate phosphoribosyltransferase (EC 2.4.2.18)	SCE1572_1717	yes	sce_1230	yes
A and B	Amino Acids and Derivatives	Aromatic amino acids and derivatives	Chorismate: Intermediate for synthesis of Tryptophan, PAPA a	Anthranilate synthase, amidotransferase component (EC 4.1.3.27)	SCE1572_266	yes	sce_5652	yes
A and B	Amino Acids and Derivatives	Aromatic amino acids and derivatives	Chorismate: Intermediate for synthesis of Tryptophan, PAPA a	Anthranilate synthase, aminase component (EC 4.1.3.27)	SCE1572_6793	yes	sce_5652	yes
A and B	Amino Acids and Derivatives	Aromatic amino acids and derivatives	Chorismate: Intermediate for synthesis of Tryptophan, PAPA a	Indole-3-glycerol phosphate synthase (EC 4.1.1.48)	SCE1572_1718	yes	sce_1231	yes
A and B	Amino Acids and Derivatives	Aromatic amino acids and derivatives	Chorismate: Intermediate for synthesis of Tryptophan, PAPA a	Isochorismatase (EC 3.3.2.1)	SCE1572_5977	yes	sce_4937	yes
A and B	Amino Acids and Derivatives	Aromatic amino acids and derivatives	Chorismate: Intermediate for synthesis of Tryptophan, PAPA a	Isochorismatase (EC 3.3.2.1) of siderophore biosynthesis	SCE1572_5181	yes	sce_4342	yes
A and B	Amino Acids and Derivatives	Aromatic amino acids and derivatives	Chorismate: Intermediate for synthesis of Tryptophan, PAPA a	Isochorismate synthase (EC 5.4.4.2) of siderophore biosynthesis	SCE1572_5180	yes	sce_4340	yes
A and B	Amino Acids and Derivatives	Aromatic amino acids and derivatives	Chorismate: Intermediate for synthesis of Tryptophan, PAPA a	Para-aminobenzoate synthase, aminase component (EC 2.6.1.85)	SCE1572_265	yes	sce_697	yes
A and B	Amino Acids and Derivatives	Aromatic amino acids and derivatives	Chorismate: Intermediate for synthesis of Tryptophan, PAPA a	Phosphoribosylanthranilate isomerase (EC 5.3.1.24)	SCE1572_1718	yes	sce_1231	yes
A and B	Amino Acids and Derivatives	Aromatic amino acids and derivatives	Chorismate: Intermediate for synthesis of Tryptophan, PAPA a	Phosphoribosylformimino-5-aminoimidazole carboxamide ribotide isom	SCE1572_3830	yes	sce_3157	yes
A and B	Amino Acids and Derivatives	Aromatic amino acids and derivatives	Chorismate: Intermediate for synthesis of Tryptophan, PAPA a	Tryptophan synthase alpha chain (EC 4.2.1.20)	SCE1572_1720	yes	sce_1233	yes
A and B	Amino Acids and Derivatives	Aromatic amino acids and derivatives	Chorismate: Intermediate for synthesis of Tryptophan, PAPA a	Tryptophan synthase beta chain (EC 4.2.1.20)	SCE1572_1719	yes	sce_1232	yes
A and B	Amino Acids and Derivatives	Aromatic amino acids and derivatives	Chorismate Synthesis	2-keto-3-deoxy-D-arabino-heptulosonate-7-phosphate synthase I alpha	SCE1572_3307	yes	sce_2798	yes
A and B	Amino Acids and Derivatives	Aromatic amino acids and derivatives	Chorismate Synthesis	2-keto-3-deoxy-D-arabino-heptulosonate-7-phosphate synthase II (EC	SCE1572_4918	yes	sce_4129	yes
A and B	Amino Acids and Derivatives	Aromatic amino acids and derivatives	Chorismate Synthesis	3-dehydroquinate dehydratase I (EC 4.2.1.10)	SCE1572_5233	yes	sce_4388	yes
A and B	Amino Acids and Derivatives	Aromatic amino acids and derivatives	Chorismate Synthesis	3-dehydroquinate synthase (EC 4.2.3.4)	SCE1572_1355	yes	sce_980	yes
A and B	Amino Acids and Derivatives	Aromatic amino acids and derivatives	Chorismate Synthesis	5-Enolpyruvylshikimate-3-phosphate synthase (EC 2.5.1.19)	SCE1572_4080	yes	sce_3406	yes
A and B	Amino Acids and Derivatives	Aromatic amino acids and derivatives	Chorismate Synthesis	Chorismate mutase I (EC 5.4.99.5)	SCE1572_1885	yes	sce_1401	yes
A and B	Amino Acids and Derivatives	Aromatic amino acids and derivatives	Chorismate Synthesis	Chorismate synthase (EC 4.2.3.5)	SCE1572_6397	yes	sce_5416	yes
A and B	Amino Acids and Derivatives	Aromatic amino acids and derivatives	Chorismate Synthesis	Prephenate and/or arogenate dehydrogenase (unknown specificity) (EC	SCE1572_1885	yes	sce_1401	yes
A and B	Amino Acids and Derivatives	Aromatic amino acids and derivatives	Chorismate Synthesis	Prephenate dehydratase (EC 4.2.1.51)	SCE1572_2392	yes	sce_1945	yes
A and B	Amino Acids and Derivatives	Aromatic amino acids and derivatives	Chorismate Synthesis	Shikimate 5-dehydrogenase I alpha (EC 1.1.1.25)	SCE1572_3015	yes	sce_2476	yes
A and B	Amino Acids and Derivatives	Aromatic amino acids and derivatives	Chorismate Synthesis	Shikimate 5-dehydrogenase I gamma (EC 1.1.1.25)	SCE1572_2281	yes	sce_1827	yes
A and B	Amino Acids and Derivatives	Aromatic amino acids and derivatives	Chorismate Synthesis	Shikimate kinase I (EC 2.7.1.71)	SCE1572_6396	yes	sce_5415	yes
A and B	Amino Acids and Derivatives	Aromatic amino acids and derivatives	Common Pathway For Synthesis of Aromatic Compounds (DA	2-keto-3-deoxy-D-arabino-heptulosonate-7-phosphate synthase I alpha	SCE1572_3307	yes	sce_2798	yes
A and B	Amino Acids and Derivatives	Aromatic amino acids and derivatives	Common Pathway For Synthesis of Aromatic Compounds (DA	2-keto-3-deoxy-D-arabino-heptulosonate-7-phosphate synthase II (EC	SCE1572_4918	yes	sce_4129	yes
A and B	Amino Acids and Derivatives	Aromatic amino acids and derivatives	Common Pathway For Synthesis of Aromatic Compounds (DA	3-dehydroquinate dehydratase I (EC 4.2.1.10)	SCE1572_5233	yes	sce_4388	yes
A and B	Amino Acids and Derivatives	Aromatic amino acids and derivatives	Common Pathway For Synthesis of Aromatic Compounds (DA	3-dehydroquinate synthase (EC 4.2.3.4)	SCE1572_1355	yes	sce_980	yes
A and B	Amino Acids and Derivatives	Aromatic amino acids and derivatives	Common Pathway For Synthesis of Aromatic Compounds (DA	5-Enolpyruvylshikimate-3-phosphate synthase (EC 2.5.1.19)	SCE1572_4080	yes	sce_3406	yes
A and B	Amino Acids and Derivatives	Aromatic amino acids and derivatives	Common Pathway For Synthesis of Aromatic Compounds (DA	Chorismate synthase (EC 4.2.3.5)	SCE1572_6397	yes	sce_5416	yes
A and B	Amino Acids and Derivatives	Aromatic amino acids and derivatives	Common Pathway For Synthesis of Aromatic Compounds (DA	Shikimate 5-dehydrogenase I alpha (EC 1.1.1.25)	SCE1572_3015	yes	sce_2476	yes
A and B	Amino Acids and Derivatives	Aromatic amino acids and derivatives	Common Pathway For Synthesis of Aromatic Compounds (DA	Shikimate 5-dehydrogenase I gamma (EC 1.1.1.25)	SCE1572_2281	yes	sce_1827	yes
A and B	Amino Acids and Derivatives	Aromatic amino acids and derivatives	Common Pathway For Synthesis of Aromatic Compounds (DA	Shikimate kinase I (EC 2.7.1.71)	SCE1572_6396	yes	sce_5415	yes
A and B	Amino Acids and Derivatives	Aromatic amino acids and derivatives	Phenylalanine and Tyrosine Branches from Chorismate	Biosynthetic Aromatic amino acid aminotransferase beta (EC 2.6.1.57)	SCE1572_4073	yes	sce_3398	yes
A and B	Amino Acids and Derivatives	Aromatic amino acids and derivatives	Phenylalanine and Tyrosine Branches from Chorismate	Chorismate mutase I (EC 5.4.99.5)	SCE1572_1885	yes	sce_1401	yes
A and B	Amino Acids and Derivatives	Aromatic amino acids and derivatives	Phenylalanine and Tyrosine Branches from Chorismate	Prephenate and/or arogenate dehydrogenase (unknown specificity) (EC	SCE1572_1885	yes	sce_1401	yes
A and B	Amino Acids and Derivatives	Aromatic amino acids and derivatives	Phenylalanine and Tyrosine Branches from Chorismate	Prephenate dehydratase (EC 4.2.1.51)	SCE1572_2392	yes	sce_1945	yes
A and B	Amino Acids and Derivatives	Aromatic amino acids and derivatives	Tryptophan synthesis	Anthranilate phosphoribosyltransferase (EC 2.4.2.18)	SCE1572_1717	yes	sce_1230	yes
A and B	Amino Acids and Derivatives	Aromatic amino acids and derivatives	Tryptophan synthesis	Anthranilate synthase, amidotransferase component (EC 4.1.3.27)	SCE1572_266	yes	sce_5652	yes
A and B	Amino Acids and Derivatives	Aromatic amino acids and derivatives	Tryptophan synthesis	Anthranilate synthase, aminase component (EC 4.1.3.27)	SCE1572_6793	yes	sce_5652	yes
A and B	Amino Acids and Derivatives	Aromatic amino acids and derivatives	Tryptophan synthesis	Indole-3-glycerol phosphate synthase (EC 4.1.1.48)	SCE1572_1718	yes	sce_1231	yes
A and B	Amino Acids and Derivatives	Aromatic amino acids and derivatives	Tryptophan synthesis	Phosphoribosylanthranilate isomerase (EC 5.3.1.24)	SCE1572_1718	yes	sce_1231	yes
A and B	Amino Acids and Derivatives	Aromatic amino acids and derivatives	Tryptophan synthesis	Tryptophan synthase alpha chain (EC 4.2.1.20)	SCE1572_1720	yes	sce_1233	yes
A and B	Amino Acids and Derivatives	Aromatic amino acids and derivatives	Tryptophan synthesis	Tryptophan synthase beta chain (EC 4.2.1.20)	SCE1572_1719	yes	sce_1232	yes
B	Amino Acids and Derivatives	Aromatic amino acids and derivatives	Aromatic amino acid degradation	Phenylalanine-4-hydroxylase (EC 1.14.16.1)		no	sce_5898	yes
B	Amino Acids and Derivatives	Aromatic amino acids and derivatives	Aromatic amino acid degradation	Tryptophan 2-monooxygenase (EC 1.13.12.3)		no	sce_10227	yes
B	Amino Acids and Derivatives	Aromatic amino acids and derivatives	Chorismate: Intermediate for synthesis of Tryptophan, PAPA a	Para-aminobenzoate synthase, amidotransferase component (EC 2.6.1.85)		no	sce_5651	yes
A and B	Amino Acids and Derivatives	Branched-chain amino acids	Branched-Chain Amino Acid Biosynthesis	(R)-citramalate synthase (EC 2.3.1.182)	SCE1572_4977	yes	sce_4186	yes
A and B	Amino Acids and Derivatives	Branched-chain amino acids	Branched-Chain Amino Acid Biosynthesis	2-isopropylmalate synthase (EC 2.3.3.13)	SCE1572_4976	yes	sce_4185	yes
A and B	Amino Acids and Derivatives	Branched-chain amino acids	Branched-Chain Amino Acid Biosynthesis	3-isopropylmalate dehydratase large subunit (EC 4.2.1.33)	SCE1572_108	yes	sce_96	yes
A and B	Amino Acids and Derivatives	Branched-chain amino acids	Branched-Chain Amino Acid Biosynthesis	3-isopropylmalate dehydratase small subunit (EC 4.2.1.33)	SCE1572_107	yes	sce_95	yes
A and B	Amino Acids and Derivatives	Branched-chain amino acids	Branched-Chain Amino Acid Biosynthesis	3-isopropylmalate dehydrogenase (EC 1.1.1.85)	SCE1572_4978	yes	sce_4187	yes
A and B	Amino Acids and Derivatives	Branched-chain amino acids	Branched-Chain Amino Acid Biosynthesis	Acetolactate synthase large subunit (EC 2.2.1.6)	SCE1572_311	yes	sce_4182	yes
A and B	Amino Acids and Derivatives	Branched-chain amino acids	Branched-Chain Amino Acid Biosynthesis	Acetolactate synthase small subunit (EC 2.2.1.6)	SCE1572_4974	yes	sce_4183	yes
A and B	Amino Acids and Derivatives	Branched-chain amino acids	Branched-Chain Amino Acid Biosynthesis	Branched-chain amino acid aminotransferase (EC 2.6.1.42)	SCE1572_354	yes	sce_195	yes
A and B	Amino Acids and Derivatives	Branched-chain amino acids	Branched-Chain Amino Acid Biosynthesis	Dihydroxy-acid dehydratase (EC 4.2.1.9)	SCE1572_7255	yes	sce_1693	yes
A and B	Amino Acids and Derivatives	Branched-chain amino acids	Branched-Chain Amino Acid Biosynthesis	Ketol-acid reductoisomerase (EC 1.1.1.86)	SCE1572_4975	yes	sce_4184	yes

A and B	Amino Acids and Derivatives	Branched-chain amino acids	Branched-Chain Amino Acid Biosynthesis	Leucine dehydrogenase (EC 1.4.1.9)	SCE1572_8281	yes	sce_6950	yes
A and B	Amino Acids and Derivatives	Branched-chain amino acids	Branched-Chain Amino Acid Biosynthesis	Threonine dehydratase, catabolic (EC 4.3.1.19)	SCE1572_5116	yes	sce_4304	yes
A and B	Amino Acids and Derivatives	Branched-chain amino acids	Branched-Chain Amino Acid Biosynthesis	Valine--pyruvate aminotransferase (EC 2.6.1.66)	SCE1572_1439	yes	sce_6322	yes
A and B	Amino Acids and Derivatives	Branched-chain amino acids	HMG-CoA	Biotin carboxyl carrier protein of methylcrotonyl-CoA carboxylase	SCE1572_4933	yes	sce_4144	yes
A and B	Amino Acids and Derivatives	Branched-chain amino acids	HMG-CoA	Branched-chain alpha-keto acid dehydrogenase, E1 component, alpha s	SCE1572_2201	yes	sce_1748	yes
A and B	Amino Acids and Derivatives	Branched-chain amino acids	HMG-CoA	Branched-chain alpha-keto acid dehydrogenase, E1 component, beta su	SCE1572_2200	yes	sce_1747	yes
A and B	Amino Acids and Derivatives	Branched-chain amino acids	HMG-CoA	Branched-chain amino acid aminotransferase (EC 2.6.1.42)	SCE1572_354	yes	sce_195	yes
A and B	Amino Acids and Derivatives	Branched-chain amino acids	HMG-CoA	Dihydrolipoamide acyltransferase component of branched-chain alpha-l	SCE1572_2199	yes	sce_1746	yes
A and B	Amino Acids and Derivatives	Branched-chain amino acids	HMG-CoA	Hydroxymethylglutaryl-CoA lyase (EC 4.1.3.4)	SCE1572_5790	yes	sce_4837	yes
A and B	Amino Acids and Derivatives	Branched-chain amino acids	HMG-CoA	Hydroxymethylglutaryl-CoA reductase (EC 1.1.1.34)	SCE1572_4053	yes	sce_3379	yes
A and B	Amino Acids and Derivatives	Branched-chain amino acids	HMG-CoA	Hydroxymethylglutaryl-CoA synthase (EC 2.3.3.10)	SCE1572_864	yes	sce_3568	yes
A and B	Amino Acids and Derivatives	Branched-chain amino acids	HMG-CoA	Isovaleryl-CoA dehydrogenase (EC 1.3.99.10)	SCE1572_8971	yes	sce_7733	yes
A and B	Amino Acids and Derivatives	Branched-chain amino acids	HMG-CoA	Leucine dehydrogenase (EC 1.4.1.9)	SCE1572_8281	yes	sce_6950	yes
A and B	Amino Acids and Derivatives	Branched-chain amino acids	HMG-CoA	Methylcrotonyl-CoA carboxylase biotin-containing subunit (EC 6.4.1.4)	SCE1572_2729	yes	sce_2267	yes
A and B	Amino Acids and Derivatives	Branched-chain amino acids	HMG-CoA	Methylcrotonyl-CoA carboxylase carboxyl transferase subunit (EC 6.4.	SCE1572_3419	yes	sce_2906	yes
A and B	Amino Acids and Derivatives	Branched-chain amino acids	HMG-CoA	Methylglutaconyl-CoA hydratase (EC 4.2.1.18)	SCE1572_861	yes	sce_1987	yes
A and B	Amino Acids and Derivatives	Branched-chain amino acids	HMG-CoA	Succinyl-CoA:3-ketoacid-coenzyme A transferase subunit A (EC 2.8.3.	SCE1572_7617	yes	sce_6477	yes
A and B	Amino Acids and Derivatives	Branched-chain amino acids	HMG-CoA	Succinyl-CoA:3-ketoacid-coenzyme A transferase subunit B (EC 2.8.3.	SCE1572_7618	yes	sce_6478	yes
A and B	Amino Acids and Derivatives	Branched-chain amino acids	HMG CoA Synthesis	Biotin carboxyl carrier protein of methylcrotonyl-CoA carboxylase	SCE1572_4933	yes	sce_4144	yes
A and B	Amino Acids and Derivatives	Branched-chain amino acids	HMG CoA Synthesis	Hydroxymethylglutaryl-CoA lyase (EC 4.1.3.4)	SCE1572_5790	yes	sce_4837	yes
A and B	Amino Acids and Derivatives	Branched-chain amino acids	HMG CoA Synthesis	Isovaleryl-CoA dehydrogenase (EC 1.3.99.10)	SCE1572_8971	yes	sce_7733	yes
A and B	Amino Acids and Derivatives	Branched-chain amino acids	HMG CoA Synthesis	Methylcrotonyl-CoA carboxylase biotin-containing subunit (EC 6.4.1.4)	SCE1572_2729	yes	sce_2267	yes
A and B	Amino Acids and Derivatives	Branched-chain amino acids	HMG CoA Synthesis	Methylcrotonyl-CoA carboxylase carboxyl transferase subunit (EC 6.4.	SCE1572_3419	yes	sce_2906	yes
A and B	Amino Acids and Derivatives	Branched-chain amino acids	HMG CoA Synthesis	Methylglutaconyl-CoA hydratase (EC 4.2.1.18)	SCE1572_861	yes	sce_1987	yes
A and B	Amino Acids and Derivatives	Branched-chain amino acids	Isoleucine degradation	3-hydroxyacyl-CoA dehydrogenase (EC 1.1.1.35)	SCE1572_60	yes	sce_50	yes
A and B	Amino Acids and Derivatives	Branched-chain amino acids	Isoleucine degradation	3-ketoacyl-CoA thiolase (EC 2.3.1.16)	SCE1572_61	yes	sce_51	yes
A and B	Amino Acids and Derivatives	Branched-chain amino acids	Isoleucine degradation	Acyl-CoA dehydrogenase, short-chain specific (EC 1.3.99.2)	SCE1572_1837	yes	sce_1299	yes
A and B	Amino Acids and Derivatives	Branched-chain amino acids	Isoleucine degradation	Branched-chain alpha-keto acid dehydrogenase, E1 component, alpha s	SCE1572_2201	yes	sce_1748	yes
A and B	Amino Acids and Derivatives	Branched-chain amino acids	Isoleucine degradation	Branched-chain alpha-keto acid dehydrogenase, E1 component, beta su	SCE1572_2200	yes	sce_1747	yes
A and B	Amino Acids and Derivatives	Branched-chain amino acids	Isoleucine degradation	Branched-chain amino acid aminotransferase (EC 2.6.1.42)	SCE1572_354	yes	sce_195	yes
A and B	Amino Acids and Derivatives	Branched-chain amino acids	Isoleucine degradation	Butyryl-CoA dehydrogenase (EC 1.3.99.2)	SCE1572_1711	yes	sce_1225	yes
A and B	Amino Acids and Derivatives	Branched-chain amino acids	Isoleucine degradation	Dihydrolipoamide acyltransferase component of branched-chain alpha-l	SCE1572_2199	yes	sce_1746	yes
A and B	Amino Acids and Derivatives	Branched-chain amino acids	Isoleucine degradation	Enoyl-CoA hydratase (EC 4.2.1.17)	SCE1572_60	yes	sce_50	yes
A and B	Amino Acids and Derivatives	Branched-chain amino acids	Isoleucine degradation	Enoyl-CoA hydratase [isoleucine degradation] (EC 4.2.1.17)	SCE1572_9647	yes	sce_8458	yes
A and B	Amino Acids and Derivatives	Branched-chain amino acids	Isoleucine degradation	Transcriptional regulator BkdR of isoleucine and valine catabolism oper	SCE1572_3384	yes	sce_2871	yes
A and B	Amino Acids and Derivatives	Branched-chain amino acids	Leucine Biosynthesis	2-isopropylmalate synthase (EC 2.3.3.13)	SCE1572_4976	yes	sce_4185	yes
A and B	Amino Acids and Derivatives	Branched-chain amino acids	Leucine Biosynthesis	3-isopropylmalate dehydratase large subunit (EC 4.2.1.33)	SCE1572_108	yes	sce_96	yes
A and B	Amino Acids and Derivatives	Branched-chain amino acids	Leucine Biosynthesis	3-isopropylmalate dehydratase small subunit (EC 4.2.1.33)	SCE1572_107	yes	sce_95	yes
A and B	Amino Acids and Derivatives	Branched-chain amino acids	Leucine Biosynthesis	3-isopropylmalate dehydrogenase (EC 1.1.1.85)	SCE1572_4978	yes	sce_4187	yes
A and B	Amino Acids and Derivatives	Branched-chain amino acids	Leucine Biosynthesis	Branched-chain amino acid aminotransferase (EC 2.6.1.42)	SCE1572_354	yes	sce_195	yes
A and B	Amino Acids and Derivatives	Branched-chain amino acids	Leucine Degradation and HMG-CoA Metabolism	Biotin carboxyl carrier protein of methylcrotonyl-CoA carboxylase	SCE1572_4933	yes	sce_4144	yes
A and B	Amino Acids and Derivatives	Branched-chain amino acids	Leucine Degradation and HMG-CoA Metabolism	Branched-chain alpha-keto acid dehydrogenase, E1 component, alpha s	SCE1572_2201	yes	sce_1748	yes
A and B	Amino Acids and Derivatives	Branched-chain amino acids	Leucine Degradation and HMG-CoA Metabolism	Branched-chain alpha-keto acid dehydrogenase, E1 component, beta su	SCE1572_2200	yes	sce_1747	yes
A and B	Amino Acids and Derivatives	Branched-chain amino acids	Leucine Degradation and HMG-CoA Metabolism	Branched-chain amino acid aminotransferase (EC 2.6.1.42)	SCE1572_354	yes	sce_195	yes
A and B	Amino Acids and Derivatives	Branched-chain amino acids	Leucine Degradation and HMG-CoA Metabolism	Dihydrolipoamide acyltransferase component of branched-chain alpha-l	SCE1572_2199	yes	sce_1746	yes
A and B	Amino Acids and Derivatives	Branched-chain amino acids	Leucine Degradation and HMG-CoA Metabolism	Hydroxymethylglutaryl-CoA lyase (EC 4.1.3.4)	SCE1572_5790	yes	sce_4837	yes
A and B	Amino Acids and Derivatives	Branched-chain amino acids	Leucine Degradation and HMG-CoA Metabolism	Hydroxymethylglutaryl-CoA reductase (EC 1.1.1.34)	SCE1572_4053	yes	sce_3379	yes
A and B	Amino Acids and Derivatives	Branched-chain amino acids	Leucine Degradation and HMG-CoA Metabolism	Hydroxymethylglutaryl-CoA synthase (EC 2.3.3.10)	SCE1572_864	yes	sce_3568	yes
A and B	Amino Acids and Derivatives	Branched-chain amino acids	Leucine Degradation and HMG-CoA Metabolism	Isovaleryl-CoA dehydrogenase (EC 1.3.99.10)	SCE1572_8971	yes	sce_7733	yes
A and B	Amino Acids and Derivatives	Branched-chain amino acids	Leucine Degradation and HMG-CoA Metabolism	Leucine dehydrogenase (EC 1.4.1.9)	SCE1572_8281	yes	sce_6950	yes
A and B	Amino Acids and Derivatives	Branched-chain amino acids	Leucine Degradation and HMG-CoA Metabolism	Methylcrotonyl-CoA carboxylase biotin-containing subunit (EC 6.4.1.4)	SCE1572_2729	yes	sce_2267	yes
A and B	Amino Acids and Derivatives	Branched-chain amino acids	Leucine Degradation and HMG-CoA Metabolism	Methylcrotonyl-CoA carboxylase carboxyl transferase subunit (EC 6.4.	SCE1572_3419	yes	sce_2906	yes
A and B	Amino Acids and Derivatives	Branched-chain amino acids	Leucine Degradation and HMG-CoA Metabolism	Methylglutaconyl-CoA hydratase (EC 4.2.1.18)	SCE1572_861	yes	sce_1987	yes
A and B	Amino Acids and Derivatives	Branched-chain amino acids	Leucine Degradation and HMG-CoA Metabolism	Succinyl-CoA:3-ketoacid-coenzyme A transferase subunit A (EC 2.8.3.	SCE1572_7617	yes	sce_6477	yes
A and B	Amino Acids and Derivatives	Branched-chain amino acids	Leucine Degradation and HMG-CoA Metabolism	Succinyl-CoA:3-ketoacid-coenzyme A transferase subunit B (EC 2.8.3.	SCE1572_7618	yes	sce_6478	yes
A and B	Amino Acids and Derivatives	Branched-chain amino acids	Valine degradation	3-hydroxyacyl-CoA dehydrogenase (EC 1.1.1.35)	SCE1572_60	yes	sce_50	yes
A and B	Amino Acids and Derivatives	Branched-chain amino acids	Valine degradation	Branched-chain alpha-keto acid dehydrogenase, E1 component, alpha s	SCE1572_2201	yes	sce_1748	yes
A and B	Amino Acids and Derivatives	Branched-chain amino acids	Valine degradation	Branched-chain alpha-keto acid dehydrogenase, E1 component, beta su	SCE1572_2200	yes	sce_1747	yes
A and B	Amino Acids and Derivatives	Branched-chain amino acids	Valine degradation	Branched-chain amino acid aminotransferase (EC 2.6.1.42)	SCE1572_354	yes	sce_195	yes
A and B	Amino Acids and Derivatives	Branched-chain amino acids	Valine degradation	Butyryl-CoA dehydrogenase (EC 1.3.99.2)	SCE1572_1711	yes	sce_1225	yes
A and B	Amino Acids and Derivatives	Branched-chain amino acids	Valine degradation	Dihydrolipoamide acyltransferase component of branched-chain alpha-l	SCE1572_2199	yes	sce_1746	yes
A and B	Amino Acids and Derivatives	Branched-chain amino acids	Valine degradation	Enoyl-CoA hydratase (EC 4.2.1.17)	SCE1572_60	yes	sce_50	yes
A and B	Amino Acids and Derivatives	Branched-chain amino acids	Valine degradation	Methylmalonate-semialdehyde dehydrogenase (EC 1.2.1.27)	SCE1572_5449	yes	sce_7163	yes
A and B	Amino Acids and Derivatives	Branched-chain amino acids	Valine degradation	Transcriptional regulator BkdR of isoleucine and valine catabolism oper	SCE1572_3384	yes	sce_2871	yes
B	Amino Acids and Derivatives	Branched-chain amino acids	Branched-Chain Amino Acid Biosynthesis	Leucine-responsive regulatory protein, regulator for leucine (or lrp) regulon and high-affinity branch		no	sce_6297	yes
A and B	Amino Acids and Derivatives	Glutamine, glutamate, aspartate, asparagine; ar	Glutamate and Aspartate uptake in Bacteria	Asparagine synthetase [glutamine-hydrolyzing] (EC 6.3.5.4)	SCE1572_5095	yes	sce_2297	yes
A and B	Amino Acids and Derivatives	Glutamine, glutamate, aspartate, asparagine; ar	Glutamate and Aspartate uptake in Bacteria	Sodium/glutamate symport protein	SCE1572_4740	yes	sce_3961	yes
A and B	Amino Acids and Derivatives	Glutamine, glutamate, aspartate, asparagine; ar	Glutamate dehydrogenases	NAD-specific glutamate dehydrogenase (EC 1.4.1.2)	SCE1572_5775	yes	sce_4821	yes
A and B	Amino Acids and Derivatives	Glutamine, glutamate, aspartate, asparagine; ar	Glutamate dehydrogenases	NADP-specific glutamate dehydrogenase (EC 1.4.1.4)	SCE1572_1504	yes	sce_6937	yes
A and B	Amino Acids and Derivatives	Glutamine, glutamate, aspartate, asparagine; ar	Glutamine, Glutamate, Aspartate and Asparagine Biosynthesis	Asparagine synthetase [glutamine-hydrolyzing] (EC 6.3.5.4)	SCE1572_5095	yes	sce_2297	yes
A and B	Amino Acids and Derivatives	Glutamine, glutamate, aspartate, asparagine; ar	Glutamine, Glutamate, Aspartate and Asparagine Biosynthesis	Aspartate aminotransferase (EC 2.6.1.1)	SCE1572_689	yes	sce_1273	yes
A and B	Amino Acids and Derivatives	Glutamine, glutamate, aspartate, asparagine; ar	Glutamine, Glutamate, Aspartate and Asparagine Biosynthesis	Aspartate ammonia-lyase (EC 4.3.1.1)	SCE1572_545	yes	sce_352	yes
A and B	Amino Acids and Derivatives	Glutamine, glutamate, aspartate, asparagine; ar	Glutamine, Glutamate, Aspartate and Asparagine Biosynthesis	Glutamate racemase (EC 5.1.1.3)	SCE1572_284	yes	sce_5417	yes
A and B	Amino Acids and Derivatives	Glutamine, glutamate, aspartate, asparagine; ar	Glutamine, Glutamate, Aspartate and Asparagine Biosynthesis	Glutamate synthase [NADPH] large chain (EC 1.4.1.13)	SCE1572_6809	yes	sce_5669	yes
A and B	Amino Acids and Derivatives	Glutamine, glutamate, aspartate, asparagine; ar	Glutamine, Glutamate, Aspartate and Asparagine Biosynthesis	Glutamate synthase [NADPH] small chain (EC 1.4.1.13)	SCE1572_5230	yes	sce_4385	yes
A and B	Amino Acids and Derivatives	Glutamine, glutamate, aspartate, asparagine; ar	Glutamine, Glutamate, Aspartate and Asparagine Biosynthesis	Glutamine synthetase type I (EC 6.3.1.2)	SCE1572_9287	yes	sce_8068	yes
A and B	Amino Acids and Derivatives	Glutamine, glutamate, aspartate, asparagine; ar	Glutamine, Glutamate, Aspartate and Asparagine Biosynthesis	L-asparaginase (EC 3.5.1.1)	SCE1572_4078	yes	sce_3404	yes
A and B	Amino Acids and Derivatives	Glutamine, glutamate, aspartate, asparagine; ar	Glutamine, Glutamate, Aspartate and Asparagine Biosynthesis	NAD-specific glutamate dehydrogenase (EC 1.4.1.2)	SCE1572_5775	yes	sce_4821	yes
A and B	Amino Acids and Derivatives	Glutamine, glutamate, aspartate, asparagine; ar	Glutamine, Glutamate, Aspartate and Asparagine Biosynthesis	NADP-specific glutamate dehydrogenase (EC 1.4.1.4)	SCE1572_1504	yes	sce_6937	yes
A and B	Amino Acids and Derivatives	Glutamine, glutamate, aspartate, asparagine; ar	Glutamine, Glutamate, Aspartate and Asparagine Biosynthesis	glutamine synthetase family protein	SCE1572_5267	yes	sce_4421	yes
A and B	Amino Acids and Derivatives	Glutamine, glutamate, aspartate, asparagine; ar	Glutamine synthetases	Glutamine synthetase type I (EC 6.3.1.2)	SCE1572_9287	yes	sce_8068	yes
A	Amino Acids and Derivatives	Glutamine, glutamate, aspartate, asparagine; ar	Glutamine, Glutamate, Aspartate and Asparagine Biosynthesis	Aspartate racemase (EC 5.1.1.13)	SCE1572_8667	yes		no
A and B	Amino Acids and Derivatives	Histidine Metabolism	Histidine Biosynthesis	ATP phosphoribosyltransferase (EC 2.4.2.17)	SCE1572_11377	yes	sce_10213	yes
A and B	Amino Acids and Derivatives	Histidine Metabolism	Histidine Biosynthesis	ATP phosphoribosyltransferase regulatory subunit (EC 2.4.2.17)	SCE1572_11192	yes	sce_9931	yes
A and B	Amino Acids and Derivatives	Histidine Metabolism	Histidine Biosynthesis	Histidinol dehydrogenase (EC 1.1.1.23)	SCE1572_3264	yes	sce_2759	yes
A and B	Amino Acids and Derivatives	Histidine Metabolism	Histidine Biosynthesis	Histidinol-phosphate aminotransferase (EC 2.6.1.9)	SCE1572_5675	yes	sce_4727	yes
A and B	Amino Acids and Derivatives	Histidine Metabolism	Histidine Biosynthesis	Imidazole glycerol phosphate synthase amidotransferase subunit (EC 2.	SCE1572_3850	yes	sce_3168	yes
A and B	Amino Acids and Derivatives	Histidine Metabolism	Histidine Biosynthesis	Imidazole glycerol phosphate synthase cyclase subunit (EC 4.1.3.-)	SCE1572_7771	yes	sce_6588	yes
A and B	Amino Acids and Derivatives	Histidine Metabolism	Histidine Biosynthesis	Imidazoleglycerol-phosphate dehydratase (EC 4.2.1.19)	SCE1572_3851	yes	sce_3169	yes
A and B	Amino Acids and Derivatives	Histidine Metabolism	Histidine Biosynthesis	Phosphoribosyl-AMP cyclohydrolase (EC 3.5.4.19)	SCE1572_641	yes	sce_450	yes
A and B	Amino Acids and Derivatives	Histidine Metabolism	Histidine Biosynthesis	Phosphoribosyl-ATP pyrophosphatase (EC 3.6.1.31)	SCE1572_641	yes	sce_450	yes
A and B	Amino Acids and Derivatives	Histidine Metabolism	Histidine Biosynthesis	Phosphoribosylformimino-5-aminoimidazole carboxamide ribotide isom	SCE1572_3830	yes	sce_3157	yes
A and B	Amino Acids and Derivatives	Histidine Metabolism	Histidine Degradation	Formiminoglutamic iminohydrolase (EC 3.5.3.13)	SCE1572_1551	yes	sce_1078	yes
A and B	Amino Acids and Derivatives	Histidine Metabolism	Histidine Degradation	Histidine ammonia-lyase (EC 4.3.1.3)	SCE1572_8014	yes	sce_6803	yes
A and B	Amino Acids and Derivatives	Histidine Metabolism	Histidine Degradation	Imidazolonepropionase (EC 3.5.2.7)	SCE1572_5759	yes	sce_4807	yes
A and B	Amino Acids and Derivatives	Histidine Metabolism	Histidine Degradation	N-formylglutamate deformylase (EC 3.5.1.68)	SCE1572_5999	yes	sce_4951	yes
A and B	Amino Acids and Derivatives	Histidine Metabolism	Histidine Degradation	Urocanate hydratase (EC 4.2.1.49)	SCE1572_10157	yes	sce_8960	yes
A and B	Amino Acids and Derivatives	Lysine, threonine, methionine, and cysteine	Cysteine Biosynthesis	Adenylylsulfate kinase (EC 2.7.1.25)	SCE1572_7923	yes	sce_6720	yes
A and B	Amino Acids and Derivatives	Lysine, threonine, methionine, and cysteine	Cysteine Biosynthesis	Cystathionine beta-synthase (EC 4.2.1.22)	SCE1572_693	yes	sce_496	yes
A and B	Amino Acids and Derivatives	Lysine, threonine, methionine, and cysteine	Cysteine Biosynthesis	Cystathionine gamma-lyase (EC 4.4.1.1)	SCE1572_164	yes	sce_6912	yes
A and B	Amino Acids and Derivatives	Lysine, threonine, methionine, and cysteine	Cysteine Biosynthesis	Cysteine synthase (EC 2.5.1.47)	SCE1572_3174	yes	sce_3947	yes
A and B	Amino Acids and Derivatives	Lysine, threonine, methionine, and cysteine	Cysteine Biosynthesis	Phosphoadenylyl-sulfate reductase [thioredoxin] (EC 1.8.4.8)	SCE1572_10665	yes	sce_9273	yes
A and B	Amino Acids and Derivatives	Lysine, threonine, methionine, and cysteine	Cysteine Biosynthesis	Serine acetyltransferase (EC 2.3.1.30)	SCE1572_4722	yes	sce_3948	yes
A and B	Amino Acids and Derivatives	Lysine, threonine, methionine, and cysteine	Cysteine Biosynthesis	Sulfate and thiosulfate binding protein CysP	SCE1572_560	yes	sce_363	yes
A and B	Amino Acids and Derivatives	Lysine, threonine, methionine, and cysteine	Cysteine Biosynthesis	Sulfate and thiosulfate import ATP-binding protein CysA (EC 3.6.3.25)	SCE1572_9474	yes	sce_8253	yes
A and B	Amino Acids and Derivatives	Lysine, threonine, methionine, and cysteine	Cysteine Biosynthesis	Sulfate transport system permease protein CysT	SCE1572_561	yes	sce_364	yes
A and B	Amino Acids and Derivatives	Lysine, threonine, methionine, and cysteine	Cysteine Biosynthesis	Sulfate transport system permease protein CysW	SCE1572_562	yes	sce_365	yes
A and B	Amino Acids and Derivatives	Lysine, threonine, methionine, and cysteine	Cysteine Biosynthesis	Sulfite reductase [NADPH] hemoprotein beta-component (EC 1.8.1.2)	SCE1572_9694	yes	sce_8504	yes
A and B	Amino Acids and Derivatives	Lysine, threonine, methionine, and cysteine	Lysine Biosynthesis DAP Pathway	2,3,4,5-tetrahydropyridine-2,6-dicarboxylate N-acetyltransferase (EC 2	SCE1572_9431	yes	sce_8220	yes
A and B	Amino Acids and Derivatives	Lysine, threonine, methionine, and cysteine	Lysine Biosynthesis DAP Pathway	2,3,4,5-tetrahydropyridine-2,6-dicarboxylate N-succinyltransferase (EC	SCE1572_3984	yes	sce_3315	yes
A and B	Amino Acids and Derivatives	Lysine, threonine, methionine, and cysteine	Lysine Biosynthesis DAP Pathway	Aspartate-semialdehyde dehydrogenase (EC 1.2.1.11)	SCE1572_7286	yes	sce_5989	yes
A and B	Amino Acids and Derivatives	Lysine, threonine, methionine, and cysteine	Lysine Biosynthesis DAP Pathway	Aspartokinase (EC 2.7.2.4)	SCE1572_3345	yes	sce_2841	yes
A and B	Amino Acids and Derivatives	Lysine, threonine, methionine, and cysteine	Lysine Biosynthesis DAP Pathway	Diaminopimelate decarboxylase (EC 4.1.1.20)	SCE1572_7449	yes	sce_6221	yes
A and B	Amino Acids and Derivatives	Lysine, threonine, methionine, and cysteine	Lysine Biosynthesis DAP Pathway	Diaminopimelate epimerase (EC 5.1.1.7)	SCE1572_10476	yes	sce_9121	yes
A and B	Amino Acids and Derivatives	Lysine, threonine, methionine, and cysteine	Lysine Biosynthesis DAP Pathway	Dihydrodipicolinate reductase (EC 1.3.1.26)	SCE1572_10180	yes	sce_8979	yes
A and B	Amino Acids and Derivatives	Lysine, threonine, methionine, and cysteine	Lysine Biosynthesis DAP Pathway	Dihydrodipicolinate synthase (EC 4.2.1.52)	SCE1572_807	yes	sce_8978	yes
A and B	Amino Acids and Derivatives	Lysine, threonine, methionine, and cysteine	Lysine Biosynthesis DAP Pathway	N-acetyl-L,L-diaminopimelate deacetylase (EC 3.5.1.47)	SCE1572_6684	yes	sce_2663	yes
A and B	Amino Acids and Derivatives	Lysine, threonine, methionine, and cysteine	Lysine Biosynthesis DAP Pathway	N-succinyl-L,L-diaminopimelate desuccinylase (EC 3.5.1.18)	SCE1572_3985	yes	sce_3316	yes
A and B	Amino Acids and Derivatives	Lysine, threonine, methionine, and cysteine	Lysine Biosynthesis DAP Pathway, GJO scratch	2,3,4,5-tetrahydropyridine-2,6-dicarboxylate N-acetyltransferase (EC 2	SCE1572_9431	yes	sce_8220	yes
A and B	Amino Acids and Derivatives	Lysine, threonine, methionine, and cysteine	Lysine Biosynthesis DAP Pathway, GJO scratch	2,3,4,5-tetrahydropyridine-2,6-dicarboxylate N-succinyltransferase (EC	SCE1572_3984	yes	sce_3315	yes
A and B	Amino Acids and Derivatives	Lysine, threonine, methionine, and cysteine	Lysine Biosynthesis DAP Pathway, GJO scratch	Aspartate-semialdehyde dehydrogenase (EC 1.2.1.11)	SCE1572_7286	yes	sce_5989	yes
A and B	Amino Acids and Derivatives	Lysine, threonine, methionine, and cysteine	Lysine Biosynthesis DAP Pathway, GJO scratch	Aspartokinase (EC 2.7.2.4)	SCE1572_3345	yes	sce_2841	yes
A and B	Amino Acids and Derivatives	Lysine, threonine, methionine, and cysteine	Lysine Biosynthesis DAP Pathway, GJO scratch	Diaminopimelate decarboxylase (EC 4.1.1.20)	SCE1572_7449	yes	sce_6221	yes
A and B	Amino Acids and Derivatives	Lysine, threonine, methionine, and cysteine	Lysine Biosynthesis DAP Pathway, GJO scratch	Diaminopimelate epimerase (EC 5.1.1.7)	SCE1572_10476	yes	sce_9121	yes
A and B	Amino Acids and Derivatives	Lysine, threonine, methionine, and cysteine	Lysine Biosynthesis DAP Pathway, GJO scratch	Dihydrodipicolinate reductase (EC 1.3.1.26)	SCE1572_10180	yes	sce_8979	yes
A and B	Amino Acids and Derivatives	Lysine, threonine, methionine, and cysteine	Lysine Biosynthesis DAP Pathway, GJO scratch	Dihydrodipicolinate synthase (EC 4.2.1.52)	SCE1572_807	yes	sce_8978	yes
A and B	Amino Acids and Derivatives	Lysine, threonine, methionine, and cysteine	Lysine Biosynthesis DAP Pathway, GJO scratch	N-acetyl-L,L-diaminopimelate deacetylase (EC 3.5.1.47)	SCE1572_6684	yes	sce_2663	yes
A and B	Amino Acids and Derivatives	Lysine, threonine, methionine, and cysteine	Lysine Biosynthesis DAP Pathway, GJO scratch	N-succinyl-L,L-diaminopimelate desuccinylase (EC 3.5.1.18)	SCE1572_3985	yes	sce_3316	yes
A and B	Amino Acids and Derivatives	Lysine, threonine, methionine, and cysteine	Lysine fermentation	3,5-diaminohexanoate dehydrogenase (EC 1.4.1.11)	SCE1572_3262	yes	sce_2757	yes
A and B	Amino Acids and Derivatives	Lysine, threonine, methionine, and cysteine	Lysine fermentation	3-aminobutyryl-CoA ammonia-lyase (EC 4.3.1.14)	SCE1572_8263	yes	sce_6934	yes
A and B	Amino Acids and Derivatives	Lysine, threonine, methionine, and cysteine	Lysine fermentation	3-keto-5-aminohexanoate cleavage enzyme	SCE1572_8263	yes	sce_6934	yes
A and B	Amino Acids and Derivatives	Lysine, threonine, methionine, and cysteine	Lysine fermentation	Acetyl-CoA acetyltransferase (EC 2.3.1.9)	SCE1572_61	yes	sce_51	yes
A and B	Amino Acids and Derivatives	Lysine, threonine, methionine, and cysteine	Lysine fermentation	Butyryl-CoA dehydrogenase (EC 1.3.99.2)	SCE1572_1711	yes	sce_1225	yes
A and B	Amino Acids and Derivatives	Lysine, threonine, methionine, and cysteine	Lysine fermentation	Electron transfer flavoprotein, alpha subunit	SCE1572_934	yes	sce_702	yes
A and B	Amino Acids and Derivatives	Lysine, threonine, methionine, and cysteine	Lysine fermentation	Electron transfer flavoprotein, beta subunit	SCE1572_935	yes	sce_703	yes
A and B	Amino Acids and Derivatives	Lysine, threonine, methionine, and cysteine	Lysine fermentation	L-beta-lysine 5,6-aminomutase alpha subunit (EC 5.4.3.3)	SCE1572_3913	yes	sce_3243	yes
A and B	Amino Acids and Derivatives	Lysine, threonine, methionine, and cysteine	Lysine fermentation	L-beta-lysine 5,6-aminomutase beta subunit (EC 5.4.3.3)	SCE1572_3914	yes	sce_3245	yes
A and B	Amino Acids and Derivatives	Lysine, threonine, methionine, and cysteine	Lysine fermentation	Lysine 2,3-aminomutase (EC 5.4.3.2)	SCE1572_3246	yes	sce_2741	yes
A and B	Amino Acids and Derivatives	Lysine, threonine, methionine, and cysteine	Methionine Biosynthesis	5,10-methylenetetrahydrofolate reductase (EC 1.5.1.20)	SCE1572_7802	yes	sce_6607	yes
A and B	Amino Acids and Derivatives	Lysine, threonine, methionine, and cysteine	Methionine Biosynthesis	5-methyltetrahydrofolate--homocysteine methyltransferase (EC 2.1.1.1	SCE1572_5635	yes	sce_5820	yes
A and B	Amino Acids and Derivatives	Lysine, threonine, methionine, and cysteine	Methionine Biosynthesis	Adenosylhomocysteinase (EC 3.3.1.1)	SCE1572_722	yes	sce_1791	yes
A and B	Amino Acids and Derivatives	Lysine, threonine, methionine, and cysteine	Methionine Biosynthesis	Cystathionine beta-synthase (EC 4.2.1.22)	SCE1572_693	yes	sce_496	yes
A and B	Amino Acids and Derivatives	Lysine, threonine, methionine, and cysteine	Methionine Biosynthesis	Cystathionine gamma-lyase (EC 4.4.1.1)	SCE1572_164	yes	sce_6912	yes
A and B	Amino Acids and Derivatives	Lysine, threonine, methionine, and cysteine	Methionine Biosynthesis	Cystathionine gamma-synthase (EC 2.5.1.48)	SCE1572_9693	yes	sce_8502	yes
A and B	Amino Acids and Derivatives	Lysine, threonine, methionine, and cysteine	Methionine Biosynthesis	Cysteine synthase (EC 2.5.1.47)	SCE1572_3174	yes	sce_3947	yes
A and B	Amino Acids and Derivatives	Lysine, threonine, methionine, and cysteine	Methionine Biosynthesis	Homocysteine S-methyltransferase (EC 2.1.1.10)	SCE1572_758	yes	sce_559	yes
A and B	Amino Acids and Derivatives	Lysine, threonine, methionine, and cysteine	Methionine Biosynthesis	Homoserine dehydrogenase (EC 1.1.1.3)	SCE1572_6399	yes	sce_5418	yes
A and B	Amino Acids and Derivatives	Lysine, threonine, methionine, and cysteine	Methionine Biosynthesis	Homoserine kinase (EC 2.7.1.39)	SCE1572_633	yes	sce_441	yes
A and B	Amino Acids and Derivatives	Lysine, threonine, methionine, and cysteine	Methionine Biosynthesis	Methionine ABC transporter ATP-binding protein	SCE1572_1100	yes	sce_3521	yes
A and B	Amino Acids and Derivatives	Lysine, threonine, methionine, and cysteine	Methionine Biosynthesis	O-acetylhomoserine sulfhydrylase (EC 2.5.1.49)	SCE1572_7144	yes	sce_5878	yes
A and B	Amino Acids and Derivatives	Lysine, threonine, methionine, and cysteine	Methionine Biosynthesis	O-succinylhomoserine sulfhydrylase (EC 2.5.1.48)	SCE1572_7144	yes	sce_5878	yes
A and B	Amino Acids and Derivatives	Lysine, threonine, methionine, and cysteine	Methionine Biosynthesis	S-adenosylmethionine synthetase (EC 2.5.1.6)	SCE1572_9402	yes	sce_8184	yes
A and B	Amino Acids and Derivatives	Lysine, threonine, methionine, and cysteine	Methionine Biosynthesis	Serine acetyltransferase (EC 2.3.1.30)	SCE1572_4722	yes	sce_3948	yes
A and B	Amino Acids and Derivatives	Lysine, threonine, methionine, and cysteine	Methionine Salvage	1,2-dihydroxy-3-keto-5-methylthiopentene dioxygenase (EC 1.13.11.5	SCE1572_2153	yes	sce_1721	yes
A and B	Amino Acids and Derivatives	Lysine, threonine, methionine, and cysteine	Methionine Salvage	2,3-diketo-5-methylthiopentyl-1-phosphate enolase-phosphatase (EC 3.	SCE1572_2154	yes	sce_1722	yes
A and B	Amino Acids and Derivatives	Lysine, threonine, methionine, and cysteine	Methionine Salvage	2-hydroxy-3-keto-5-methylthiopentenyl-1-phosphate phosphatase relat	SCE1572_3842	yes	sce_3165	yes
A and B	Amino Acids and Derivatives	Lysine, threonine, methionine, and cysteine	Methionine Salvage	5'-methylthioadenosine phosphorylase (EC 2.4.2.28)	SCE1572_5013	yes	sce_4215	yes
A and B	Amino Acids and Derivatives	Lysine, threonine, methionine, and cysteine	Methionine Salvage	Methylthioribose-1-phosphate isomerase (EC 5.3.1.23)	SCE1572_2204	yes	sce_1751	yes
A and B	Amino Acids and Derivatives	Lysine, threonine, methionine, and cysteine	Methionine Salvage	Methylthioribulose-1-phosphate dehydratase (EC 4.2.1.109)	SCE1572_2152	yes	sce_1720	yes
A and B	Amino Acids and Derivatives	Lysine, threonine, methionine, and cysteine	S-methylmethionine	Homocysteine S-methyltransferase (EC 2.1.1.10)	SCE1572_758	yes	sce_559	yes
A and B	Amino Acids and Derivatives	Lysine, threonine, methionine, and cysteine	Threonine and Homoserine Biosynthesis	Aspartate aminotransferase (EC 2.6.1.1)	SCE1572_689	yes	sce_1273	yes
A and B	Amino Acids and Derivatives	Lysine, threonine, methionine, and cysteine	Threonine and Homoserine Biosynthesis	Aspartate-semialdehyde dehydrogenase (EC 1.2.1.11)	SCE1572_7286	yes	sce_5989	yes
A and B	Amino Acids and Derivatives	Lysine, threonine, methionine, and cysteine	Threonine and Homoserine Biosynthesis	Aspartokinase (EC 2.7.2.4)	SCE1572_3345	yes	sce_2841	yes
A and B	Amino Acids and Derivatives	Lysine, threonine, methionine, and cysteine	Threonine and Homoserine Biosynthesis	Homoserine dehydrogenase (EC 1.1.1.3)	SCE1572_6399	yes	sce_5418	yes
A and B	Amino Acids and Derivatives	Lysine, threonine, methionine, and cysteine	Threonine and Homoserine Biosynthesis	Homoserine kinase (EC 2.7.1.39)	SCE1572_633	yes	sce_441	yes
A and B	Amino Acids and Derivatives	Lysine, threonine, methionine, and cysteine	Threonine and Homoserine Biosynthesis	Threonine synthase (EC 4.2.3.1)	SCE1572_11433	yes	sce_10289	yes

A and B	Amino Acids and Derivatives	Lysine, threonine, methionine, and cysteine	Threonine degradation	L-threonine 3-dehydrogenase (EC 1.1.1.103)	SCE1572_9364	yes	sce_8144	yes
A and B	Amino Acids and Derivatives	Lysine, threonine, methionine, and cysteine	Threonine degradation	Low-specificity L-threonine aldolase (EC 4.1.2.5)	SCE1572_2736	yes	sce_2275	yes
A and B	Amino Acids and Derivatives	Lysine, threonine, methionine, and cysteine	Threonine degradation	Threonine dehydratase, catabolic (EC 4.3.1.19)	SCE1572_5116	yes	sce_4304	yes
A and B	Amino Acids and Derivatives	Lysine, threonine, methionine, and cysteine	Threonine degradation	Threonine dehydrogenase and related Zn-dependent dehydrogenases	SCE1572_42	yes	sce_534	yes
A	Amino Acids and Derivatives	Lysine, threonine, methionine, and cysteine	Cysteine Biosynthesis	Sulfate permease	SCE1572_6929	yes		no
A	Amino Acids and Derivatives	Lysine, threonine, methionine, and cysteine	Methionine Biosynthesis	5-methyltetrahydropteroyltriglutamate--homocysteine methyltransferase	SCE1572_3532	yes		no
A	Amino Acids and Derivatives	Lysine, threonine, methionine, and cysteine	Methionine Biosynthesis	Methionine ABC transporter permease protein	SCE1572_1101	yes		no
A	Amino Acids and Derivatives	Lysine, threonine, methionine, and cysteine	Methionine Biosynthesis	Methionine ABC transporter substrate-binding protein	SCE1572_1102	yes		no
B	Amino Acids and Derivatives	Lysine, threonine, methionine, and cysteine	Cysteine Biosynthesis	Cys regulon transcriptional activator CysB		no	sce_2533	yes
B	Amino Acids and Derivatives	Lysine, threonine, methionine, and cysteine	Lysine degradation	Lysine decarboxylase (EC 4.1.1.18)		no	sce_93	yes
B	Amino Acids and Derivatives	Lysine, threonine, methionine, and cysteine	Methionine Biosynthesis	Predicted regulator of methionine metabolism, ArsR family		no	sce_3330	yes
A and B	Amino Acids and Derivatives	Proline and 4-hydroxyproline	A Hypothetical Protein Related to Proline Metabolism	Hypothetical protein YggS, proline synthase co-transcribed bacterial ho	SCE1572_10504	yes	sce_9152	yes
A and B	Amino Acids and Derivatives	Proline and 4-hydroxyproline	A Hypothetical Protein Related to Proline Metabolism	Pyrroline-5-carboxylate reductase (EC 1.5.1.2)	SCE1572_7743	yes	sce_6567	yes
A and B	Amino Acids and Derivatives	Proline and 4-hydroxyproline	Proline, 4-hydroxyproline uptake and utilization	D-amino-acid oxidase (EC 1.4.3.3)	SCE1572_7258	yes	sce_5972	yes
A and B	Amino Acids and Derivatives	Proline and 4-hydroxyproline	Proline, 4-hydroxyproline uptake and utilization	Delta-1-pyrroline-5-carboxylate dehydrogenase (EC 1.5.1.12)	SCE1572_6031	yes	sce_4984	yes
A and B	Amino Acids and Derivatives	Proline and 4-hydroxyproline	Proline, 4-hydroxyproline uptake and utilization	Ketoglutarate semialdehyde dehydrogenase (EC 1.2.1.26)	SCE1572_10398	yes	sce_9215	yes
A and B	Amino Acids and Derivatives	Proline and 4-hydroxyproline	Proline, 4-hydroxyproline uptake and utilization	Proline iminopeptidase (EC 3.4.11.5)	SCE1572_7176	yes	sce_5905	yes
A and B	Amino Acids and Derivatives	Proline and 4-hydroxyproline	Proline Synthesis	Gamma-glutamyl phosphate reductase (EC 1.2.1.41)	SCE1572_4908	yes	sce_4118	yes
A and B	Amino Acids and Derivatives	Proline and 4-hydroxyproline	Proline Synthesis	Glutamate 5-kinase (EC 2.7.2.11)	SCE1572_10703	yes	sce_9312	yes
A and B	Amino Acids and Derivatives	Proline and 4-hydroxyproline	Proline Synthesis	NADP-specific glutamate dehydrogenase (EC 1.4.1.4)	SCE1572_1504	yes	sce_6937	yes
A and B	Amino Acids and Derivatives	Proline and 4-hydroxyproline	Proline Synthesis	Pyrroline-5-carboxylate reductase (EC 1.5.1.2)	SCE1572_7743	yes	sce_6567	yes
A and B	Amino Acids and Derivatives	Proline and 4-hydroxyproline	Proline Synthesis	RNA-binding C-terminal domain PUA	SCE1572_10703	yes	sce_9312	yes
A	Amino Acids and Derivatives	Proline and 4-hydroxyproline	Proline, 4-hydroxyproline uptake and utilization	D-proline reductase, 23 kDa subunit (EC 1.21.4.1)	SCE1572_8108	yes		no
A	Amino Acids and Derivatives	Proline and 4-hydroxyproline	Proline, 4-hydroxyproline uptake and utilization	D-proline reductase, 26 kDa subunit (EC 1.21.4.1)	SCE1572_8107	yes		no
A	Amino Acids and Derivatives	Proline and 4-hydroxyproline	Proline, 4-hydroxyproline uptake and utilization	D-proline reductase, 45 kDa subunit (EC 1.21.4.1)	SCE1572_8108	yes		no
A and B	Amino Acids and Derivatives	no subcategory	Creatine and Creatinine Degradation	Creatinine amidohydrolase (EC 3.5.2.10)	SCE1572_2672	yes	sce_2221	yes
A and B	Amino Acids and Derivatives	no subcategory	Creatine and Creatinine Degradation	N-methylhydantoinase (ATP-hydrolyzing) (EC 3.5.2.14)	SCE1572_3451	yes	sce_2943	yes
A and B	Amino Acids and Derivatives	no subcategory	Creatine and Creatinine Degradation	N-methylhydantoinase A (EC 3.5.2.14)	SCE1572_3453	yes	sce_2945	yes
A and B	Amino Acids and Derivatives	no subcategory	Creatine and Creatinine Degradation	N-methylhydantoinase B (EC 3.5.2.14)	SCE1572_3452	yes	sce_2944	yes
B	Amino Acids and Derivatives	no subcategory	Creatine and Creatinine Degradation	Cytosine deaminase (EC 3.5.4.1)		no	sce_4620	yes
A	Carbohydrates	Aminosugars	Chitin and N-acetylglucosamine utilization	Glucosamine-6-phosphate deaminase [isomerizing], alternative (EC 3.5.	SCE1572_3562	yes		no
A	Carbohydrates	Aminosugars	Chitin and N-acetylglucosamine utilization	N-acetylglucosamine related transporter, NagX	SCE1572_7992	yes		no
A	Carbohydrates	Aminosugars	Chitin and N-acetylglucosamine utilization	N-acetylglucosamine-6-phosphate deacetylase (EC 3.5.1.25)	SCE1572_3561	yes		no
A	Carbohydrates	Aminosugars	Chitin and N-acetylglucosamine utilization	PTS system, N-acetylglucosamine-specific IIB component (EC 2.7.1.69	SCE1572_3560	yes		no
A	Carbohydrates	Aminosugars	Chitin and N-acetylglucosamine utilization	PTS system, N-acetylglucosamine-specific IIC component (EC 2.7.1.69	SCE1572_3560	yes		no
A and B	Carbohydrates	Central carbohydrate metabolism	Dehydrogenase complexes	2-oxoglutarate dehydrogenase E1 component (EC 1.2.4.2)	SCE1572_2352	yes	sce_1318	yes
A and B	Carbohydrates	Central carbohydrate metabolism	Dehydrogenase complexes	Branched-chain alpha-keto acid dehydrogenase, E1 component, alpha s	SCE1572_2201	yes	sce_1748	yes
A and B	Carbohydrates	Central carbohydrate metabolism	Dehydrogenase complexes	Branched-chain alpha-keto acid dehydrogenase, E1 component, beta su	SCE1572_2200	yes	sce_1747	yes
A and B	Carbohydrates	Central carbohydrate metabolism	Dehydrogenase complexes	Cytosol aminopeptidase PepA (EC 3.4.11.1)	SCE1572_3407	yes	sce_2894	yes
A and B	Carbohydrates	Central carbohydrate metabolism	Dehydrogenase complexes	Dihydrolipoamide acetyltransferase component of pyruvate dehydrogen	SCE1572_2006	yes	sce_1142	yes
A and B	Carbohydrates	Central carbohydrate metabolism	Dehydrogenase complexes	Dihydrolipoamide acyltransferase component of branched-chain alpha-l	SCE1572_2199	yes	sce_1746	yes
A and B	Carbohydrates	Central carbohydrate metabolism	Dehydrogenase complexes	Dihydrolipoamide succinyltransferase component (E2) of 2-oxoglutarat	SCE1572_828	yes	sce_1318	yes
A and B	Carbohydrates	Central carbohydrate metabolism	Dehydrogenase complexes	Pyruvate dehydrogenase E1 component alpha subunit (EC 1.2.4.1)	SCE1572_5066	yes	sce_4255	yes
A and B	Carbohydrates	Central carbohydrate metabolism	Dehydrogenase complexes	Pyruvate dehydrogenase E1 component beta subunit (EC 1.2.4.1)	SCE1572_5067	yes	sce_4256	yes
A and B	Carbohydrates	Central carbohydrate metabolism	Dihydroxyacetone kinases	DHA-specific EI component	SCE1572_3297	yes	sce_2789	yes
A and B	Carbohydrates	Central carbohydrate metabolism	Dihydroxyacetone kinases	DHA-specific IIA component	SCE1572_3297	yes	sce_2789	yes
A and B	Carbohydrates	Central carbohydrate metabolism	Dihydroxyacetone kinases	DHA-specific phosphocarrier protein HPr	SCE1572_3297	yes	sce_2789	yes
A and B	Carbohydrates	Central carbohydrate metabolism	Dihydroxyacetone kinases	Phosphoenolpyruvate-dihydroxyacetone phosphotransferase (EC 2.7.1.	SCE1572_3298	yes	sce_2790	yes
A and B	Carbohydrates	Central carbohydrate metabolism	Dihydroxyacetone kinases	Phosphoenolpyruvate-dihydroxyacetone phosphotransferase (EC 2.7.1.	SCE1572_3299	yes	sce_2791	yes
A and B	Carbohydrates	Central carbohydrate metabolism	Dihydroxyacetone kinases	Phosphoenolpyruvate-dihydroxyacetone phosphotransferase (EC 2.7.1.	SCE1572_3297	yes	sce_2789	yes
A and B	Carbohydrates	Central carbohydrate metabolism	Entner-Doudoroff Pathway	2,3-bisphosphoglycerate-independent phosphoglycerate mutase (EC 5.4	SCE1572_1634	yes	sce_1168	yes
A and B	Carbohydrates	Central carbohydrate metabolism	Entner-Doudoroff Pathway	2-dehydro-3-deoxygluconate kinase (EC 2.7.1.45)	SCE1572_4552	yes	sce_1380	yes
A and B	Carbohydrates	Central carbohydrate metabolism	Entner-Doudoroff Pathway	2-dehydro-3-deoxyphosphogluconate aldolase (EC 4.1.2.14)	SCE1572_4553	yes	sce_1379	yes
A and B	Carbohydrates	Central carbohydrate metabolism	Entner-Doudoroff Pathway	6-phosphogluconolactonase (EC 3.1.1.31), eukaryotic type	SCE1572_5831	yes	sce_4872	yes
A and B	Carbohydrates	Central carbohydrate metabolism	Entner-Doudoroff Pathway	D-glycerate 2-kinase (EC 2.7.1.-)	SCE1572_4036	yes	sce_3364	yes
A and B	Carbohydrates	Central carbohydrate metabolism	Entner-Doudoroff Pathway	Enolase (EC 4.2.1.11)	SCE1572_9808	yes	sce_8614	yes
A and B	Carbohydrates	Central carbohydrate metabolism	Entner-Doudoroff Pathway	Glucokinase (EC 2.7.1.2)	SCE1572_5828	yes	sce_4870	yes
A and B	Carbohydrates	Central carbohydrate metabolism	Entner-Doudoroff Pathway	Gluconokinase (EC 2.7.1.12)	SCE1572_1893	yes	sce_1407	yes
A and B	Carbohydrates	Central carbohydrate metabolism	Entner-Doudoroff Pathway	Gluconolactonase (EC 3.1.1.17)	SCE1572_7960	yes	sce_2029	yes
A and B	Carbohydrates	Central carbohydrate metabolism	Entner-Doudoroff Pathway	Glucose 1-dehydrogenase (EC 1.1.1.47)	SCE1572_1769	yes	sce_1694	yes
A and B	Carbohydrates	Central carbohydrate metabolism	Entner-Doudoroff Pathway	Glucose-6-phosphate 1-dehydrogenase (EC 1.1.1.49)	SCE1572_5833	yes	sce_4874	yes
A and B	Carbohydrates	Central carbohydrate metabolism	Entner-Doudoroff Pathway	Hexokinase (EC 2.7.1.1)	SCE1572_7950	yes	sce_6746	yes
A and B	Carbohydrates	Central carbohydrate metabolism	Entner-Doudoroff Pathway	NAD-dependent glyceraldehyde-3-phosphate dehydrogenase (EC 1.2.1	SCE1572_6421	yes	sce_8225	yes
A and B	Carbohydrates	Central carbohydrate metabolism	Entner-Doudoroff Pathway	Phosphoglycerate kinase (EC 2.7.2.3)	SCE1572_6890	yes	sce_8224	yes
A and B	Carbohydrates	Central carbohydrate metabolism	Entner-Doudoroff Pathway	Polyphosphate glucokinase (EC 2.7.1.63)	SCE1572_1006	yes	sce_759	yes
A and B	Carbohydrates	Central carbohydrate metabolism	Entner-Doudoroff Pathway	Pyruvate kinase (EC 2.7.1.40)	SCE1572_6128	yes	sce_5073	yes
A and B	Carbohydrates	Central carbohydrate metabolism	Glycolate, glyoxylate interconversions	Glycolate dehydrogenase (EC 1.1.99.14), subunit GlcD	SCE1572_6827	yes	sce_5688	yes
A and B	Carbohydrates	Central carbohydrate metabolism	Glycolate, glyoxylate interconversions	Glycolate oxidase (EC 1.1.3.15)	SCE1572_9787	yes	sce_8585	yes
A and B	Carbohydrates	Central carbohydrate metabolism	Glycolate, glyoxylate interconversions	Phosphoglycolate phosphatase (EC 3.1.3.18)	SCE1572_11194	yes	sce_8372	yes
A and B	Carbohydrates	Central carbohydrate metabolism	Glycolysis and Gluconeogenesis	2,3-bisphosphoglycerate-independent phosphoglycerate mutase (EC 5.4	SCE1572_1634	yes	sce_1168	yes
A and B	Carbohydrates	Central carbohydrate metabolism	Glycolysis and Gluconeogenesis	6-phosphofructokinase (EC 2.7.1.11)	SCE1572_4596	yes	sce_3833	yes
A and B	Carbohydrates	Central carbohydrate metabolism	Glycolysis and Gluconeogenesis	Enolase (EC 4.2.1.11)	SCE1572_9808	yes	sce_8614	yes
A and B	Carbohydrates	Central carbohydrate metabolism	Glycolysis and Gluconeogenesis	Fructose-1,6-bisphosphatase, Bacillus type (EC 3.1.3.11)	SCE1572_10951	yes	sce_9561	yes
A and B	Carbohydrates	Central carbohydrate metabolism	Glycolysis and Gluconeogenesis	Fructose-1,6-bisphosphatase, type I (EC 3.1.3.11)	SCE1572_3405	yes	sce_2892	yes
A and B	Carbohydrates	Central carbohydrate metabolism	Glycolysis and Gluconeogenesis	Fructose-bisphosphate aldolase class I (EC 4.1.2.13)	SCE1572_3406	yes	sce_2893	yes
A and B	Carbohydrates	Central carbohydrate metabolism	Glycolysis and Gluconeogenesis	Fructose-bisphosphate aldolase class II (EC 4.1.2.13)	SCE1572_2567	yes	sce_2139	yes
A and B	Carbohydrates	Central carbohydrate metabolism	Glycolysis and Gluconeogenesis	Glucokinase (EC 2.7.1.2)	SCE1572_5828	yes	sce_4870	yes
A and B	Carbohydrates	Central carbohydrate metabolism	Glycolysis and Gluconeogenesis	Glucose-6-phosphate isomerase (EC 5.3.1.9)	SCE1572_7517	yes	sce_6344	yes
A and B	Carbohydrates	Central carbohydrate metabolism	Glycolysis and Gluconeogenesis	Hexokinase (EC 2.7.1.1)	SCE1572_7950	yes	sce_6746	yes
A and B	Carbohydrates	Central carbohydrate metabolism	Glycolysis and Gluconeogenesis	NAD-dependent glyceraldehyde-3-phosphate dehydrogenase (EC 1.2.1	SCE1572_6421	yes	sce_8225	yes
A and B	Carbohydrates	Central carbohydrate metabolism	Glycolysis and Gluconeogenesis	Phosphoenolpyruvate synthase (EC 2.7.9.2)	SCE1572_1055	yes	sce_4687	yes
A and B	Carbohydrates	Central carbohydrate metabolism	Glycolysis and Gluconeogenesis	Phosphoglycerate kinase (EC 2.7.2.3)	SCE1572_6890	yes	sce_8224	yes
A and B	Carbohydrates	Central carbohydrate metabolism	Glycolysis and Gluconeogenesis	Polyphosphate glucokinase (EC 2.7.1.63)	SCE1572_1006	yes	sce_759	yes
A and B	Carbohydrates	Central carbohydrate metabolism	Glycolysis and Gluconeogenesis	Pyrophosphate--fructose 6-phosphate 1-phosphotransferase, alpha subu	SCE1572_2888	yes	sce_2511	yes
A and B	Carbohydrates	Central carbohydrate metabolism	Glycolysis and Gluconeogenesis	Pyruvate kinase (EC 2.7.1.40)	SCE1572_6128	yes	sce_5073	yes
A and B	Carbohydrates	Central carbohydrate metabolism	Glycolysis and Gluconeogenesis	Pyruvate,phosphate dikinase (EC 2.7.9.1)	SCE1572_9634	yes	sce_8446	yes
A and B	Carbohydrates	Central carbohydrate metabolism	Glycolysis and Gluconeogenesis	Triosephosphate isomerase (EC 5.3.1.1)	SCE1572_9434	yes	sce_8223	yes
A and B	Carbohydrates	Central carbohydrate metabolism	Glyoxylate bypass	Aconitate hydratase (EC 4.2.1.3)	SCE1572_5235	yes	sce_4390	yes
A and B	Carbohydrates	Central carbohydrate metabolism	Glyoxylate bypass	Citrate synthase (si) (EC 2.3.3.1)	SCE1572_2382	yes	sce_1933	yes
A and B	Carbohydrates	Central carbohydrate metabolism	Glyoxylate bypass	Isocitrate lyase (EC 4.1.3.1)	SCE1572_11040	yes	sce_9752	yes
A and B	Carbohydrates	Central carbohydrate metabolism	Glyoxylate bypass	Malate dehydrogenase (EC 1.1.1.37)	SCE1572_1660	yes	sce_1175	yes
A and B	Carbohydrates	Central carbohydrate metabolism	Glyoxylate bypass	Malate synthase (EC 2.3.3.9)	SCE1572_11039	yes	sce_9751	yes
A and B	Carbohydrates	Central carbohydrate metabolism	Methylglyoxal Metabolism	Aldehyde dehydrogenase (EC 1.2.1.3)	SCE1572_4022	yes	sce_2406	yes
A and B	Carbohydrates	Central carbohydrate metabolism	Methylglyoxal Metabolism	Hydroxyacylglutathione hydrolase (EC 3.1.2.6)	SCE1572_10143	yes	sce_8946	yes
A and B	Carbohydrates	Central carbohydrate metabolism	Methylglyoxal Metabolism	Lactoylglutathione lyase (EC 4.4.1.5)	SCE1572_4796	yes	sce_4011	yes
A and B	Carbohydrates	Central carbohydrate metabolism	Pentose phosphate pathway	6-phosphogluconate dehydrogenase, decarboxylating (EC 1.1.1.44)	SCE1572_4750	yes	sce_3971	yes
A and B	Carbohydrates	Central carbohydrate metabolism	Pentose phosphate pathway	6-phosphogluconolactonase (EC 3.1.1.31), eukaryotic type	SCE1572_5831	yes	sce_4872	yes
A and B	Carbohydrates	Central carbohydrate metabolism	Pentose phosphate pathway	Glucose-6-phosphate 1-dehydrogenase (EC 1.1.1.49)	SCE1572_5833	yes	sce_4874	yes
A and B	Carbohydrates	Central carbohydrate metabolism	Pentose phosphate pathway	Ribose 5-phosphate isomerase A (EC 5.3.1.6)	SCE1572_2404	yes	sce_1958	yes
A and B	Carbohydrates	Central carbohydrate metabolism	Pentose phosphate pathway	Ribose-phosphate pyrophosphokinase (EC 2.7.6.1)	SCE1572_8154	yes	sce_7319	yes
A and B	Carbohydrates	Central carbohydrate metabolism	Pentose phosphate pathway	Ribulose-phosphate 3-epimerase (EC 5.1.3.1)	SCE1572_10509	yes	sce_9159	yes
A and B	Carbohydrates	Central carbohydrate metabolism	Pentose phosphate pathway	Transaldolase (EC 2.2.1.2)	SCE1572_29	yes	sce_31	yes
A and B	Carbohydrates	Central carbohydrate metabolism	Pentose phosphate pathway	Transketolase (EC 2.2.1.1)	SCE1572_5327	yes	sce_4463	yes
A and B	Carbohydrates	Central carbohydrate metabolism	Pyruvate:ferredoxin oxidoreductase	Pyruvate-flavodoxin oxidoreductase (EC 1.2.7.-)	SCE1572_6058	yes	sce_5009	yes
A and B	Carbohydrates	Central carbohydrate metabolism	Pyruvate Alanine Serine Interconversions	Alanine dehydrogenase (EC 1.4.1.1)	SCE1572_10473	yes	sce_9118	yes
A and B	Carbohydrates	Central carbohydrate metabolism	Pyruvate Alanine Serine Interconversions	Alanine racemase (EC 5.1.1.1)	SCE1572_9334	yes	sce_8113	yes
A and B	Carbohydrates	Central carbohydrate metabolism	Pyruvate Alanine Serine Interconversions	Branched-chain amino acid aminotransferase (EC 2.6.1.42)	SCE1572_354	yes	sce_195	yes
A and B	Carbohydrates	Central carbohydrate metabolism	Pyruvate Alanine Serine Interconversions	L-serine dehydratase (EC 4.3.1.17)	SCE1572_2497	yes	sce_2075	yes
A and B	Carbohydrates	Central carbohydrate metabolism	Pyruvate Alanine Serine Interconversions	Omega-amino acid--pyruvate aminotransferase (EC 2.6.1.18)	SCE1572_8423	yes	sce_7183	yes
A and B	Carbohydrates	Central carbohydrate metabolism	Pyruvate Alanine Serine Interconversions	Valine--pyruvate aminotransferase (EC 2.6.1.66)	SCE1572_1439	yes	sce_6322	yes
A and B	Carbohydrates	Central carbohydrate metabolism	Pyruvate metabolism I: anaplerotic reactions, PEP	Malate permease	SCE1572_5325	yes	sce_4462	yes
A and B	Carbohydrates	Central carbohydrate metabolism	Pyruvate metabolism I: anaplerotic reactions, PEP	Phosphoenolpyruvate carboxykinase [GTP] (EC 4.1.1.32)	SCE1572_4050	yes	sce_3376	yes
A and B	Carbohydrates	Central carbohydrate metabolism	Pyruvate metabolism I: anaplerotic reactions, PEP	Phosphoenolpyruvate carboxylase (EC 4.1.1.31)	SCE1572_4598	yes	sce_3835	yes
A and B	Carbohydrates	Central carbohydrate metabolism	Pyruvate metabolism I: anaplerotic reactions, PEP	Phosphoenolpyruvate synthase (EC 2.7.9.2)	SCE1572_1055	yes	sce_4687	yes
A and B	Carbohydrates	Central carbohydrate metabolism	Pyruvate metabolism I: anaplerotic reactions, PEP	Pyruvate kinase (EC 2.7.1.40)	SCE1572_6128	yes	sce_5073	yes
A and B	Carbohydrates	Central carbohydrate metabolism	Pyruvate metabolism I: anaplerotic reactions, PEP	Pyruvate,phosphate dikinase (EC 2.7.9.1)	SCE1572_9634	yes	sce_8446	yes
A and B	Carbohydrates	Central carbohydrate metabolism	Pyruvate metabolism II: acetyl-CoA, acetogenesis from pyruvat	Acetyl-CoA synthetase (ADP-forming) alpha and beta chains, putative	SCE1572_5434	yes	sce_4578	yes
A and B	Carbohydrates	Central carbohydrate metabolism	Pyruvate metabolism II: acetyl-CoA, acetogenesis from pyruvat	Acetyl-coenzyme A synthetase (EC 6.2.1.1)	SCE1572_8163	yes	sce_8866	yes
A and B	Carbohydrates	Central carbohydrate metabolism	Pyruvate metabolism II: acetyl-CoA, acetogenesis from pyruvat	Acylphosphate phosphohydrolase (EC 3.6.1.7), putative	SCE1572_10056	yes	sce_8873	yes
A and B	Carbohydrates	Central carbohydrate metabolism	Pyruvate metabolism II: acetyl-CoA, acetogenesis from pyruvat	Aldehyde dehydrogenase (EC 1.2.1.3)	SCE1572_4022	yes	sce_2406	yes
A and B	Carbohydrates	Central carbohydrate metabolism	Pyruvate metabolism II: acetyl-CoA, acetogenesis from pyruvat	Dihydrolipoamide acetyltransferase component of pyruvate dehydrogen	SCE1572_2006	yes	sce_1142	yes
A and B	Carbohydrates	Central carbohydrate metabolism	Pyruvate metabolism II: acetyl-CoA, acetogenesis from pyruvat	Dihydrolipoamide dehydrogenase of pyruvate dehydrogenase complex (	SCE1572_10146	yes	sce_8949	yes
A and B	Carbohydrates	Central carbohydrate metabolism	Pyruvate metabolism II: acetyl-CoA, acetogenesis from pyruvat	NAD-dependent protein deacetylase of SIR2 family	SCE1572_901	yes	sce_672	yes
A and B	Carbohydrates	Central carbohydrate metabolism	Pyruvate metabolism II: acetyl-CoA, acetogenesis from pyruvat	Pyruvate dehydrogenase E1 component alpha subunit (EC 1.2.4.1)	SCE1572_5066	yes	sce_4255	yes
A and B	Carbohydrates	Central carbohydrate metabolism	Pyruvate metabolism II: acetyl-CoA, acetogenesis from pyruvat	Pyruvate dehydrogenase E1 component beta subunit (EC 1.2.4.1)	SCE1572_5067	yes	sce_4256	yes
A and B	Carbohydrates	Central carbohydrate metabolism	TCA Cycle	2-oxoglutarate dehydrogenase E1 component (EC 1.2.4.2)	SCE1572_2352	yes	sce_1318	yes
A and B	Carbohydrates	Central carbohydrate metabolism	TCA Cycle	Aconitate hydratase (EC 4.2.1.3)	SCE1572_5235	yes	sce_4390	yes
A and B	Carbohydrates	Central carbohydrate metabolism	TCA Cycle	Citrate synthase (si) (EC 2.3.3.1)	SCE1572_2382	yes	sce_1933	yes
A and B	Carbohydrates	Central carbohydrate metabolism	TCA Cycle	Dihydrolipoamide dehydrogenase of pyruvate dehydrogenase complex (	SCE1572_10146	yes	sce_8949	yes
A and B	Carbohydrates	Central carbohydrate metabolism	TCA Cycle	Dihydrolipoamide succinyltransferase component (E2) of 2-oxoglutarat	SCE1572_828	yes	sce_1318	yes
A and B	Carbohydrates	Central carbohydrate metabolism	TCA Cycle	Fumarate hydratase class I, aerobic (EC 4.2.1.2)	SCE1572_593	yes	sce_402	yes
A and B	Carbohydrates	Central carbohydrate metabolism	TCA Cycle	Fumarate hydratase class II (EC 4.2.1.2)	SCE1572_7239	yes	sce_10177	yes
A and B	Carbohydrates	Central carbohydrate metabolism	TCA Cycle	Isocitrate dehydrogenase [NADP] (EC 1.1.1.42)	SCE1572_1627	yes	sce_7633	yes
A and B	Carbohydrates	Central carbohydrate metabolism	TCA Cycle	Isocitrate dehydrogenase [NAD] (EC 1.1.1.41)	SCE1572_7602	yes	sce_6463	yes
A and B	Carbohydrates	Central carbohydrate metabolism	TCA Cycle	Malate dehydrogenase (EC 1.1.1.37)	SCE1572_1660	yes	sce_1175	yes
A and B	Carbohydrates	Central carbohydrate metabolism	TCA Cycle	Succinate dehydrogenase flavoprotein subunit (EC 1.3.99.1)	SCE1572_8615	yes	sce_3094	yes
A and B	Carbohydrates	Central carbohydrate metabolism	TCA Cycle	Succinate dehydrogenase iron-sulfur protein (EC 1.3.99.1)	SCE1572_8616	yes	sce_7336	yes
A and B	Carbohydrates	Central carbohydrate metabolism	TCA Cycle	Succinyl-CoA ligase [ADP-forming] alpha chain (EC 6.2.1.5)	SCE1572_11373	yes	sce_10210	yes
A and B	Carbohydrates	Central carbohydrate metabolism	TCA Cycle	Succinyl-CoA ligase [ADP-forming] beta chain (EC 6.2.1.5)	SCE1572_3507	yes	sce_2990	yes
A	Carbohydrates	Central carbohydrate metabolism	Entner-Doudoroff Pathway	6-phosphogluconolactonase (EC 3.1.1.31)	SCE1572_8633	yes		no
B	Carbohydrates	Central carbohydrate metabolism	Glycolate, glyoxylate interconversions	Glycolate dehydrogenase (EC 1.1.99.14), iron-sulfur subunit GlcF		no	sce_1682	yes
B	Carbohydrates	Central carbohydrate metabolism	Pyruvate metabolism I: anaplerotic reactions, PEP	NAD-dependent malic enzyme (EC 1.1.1.38)		no	sce_6274	yes
A and B	Carbohydrates	Di- and oligosaccharides	Lactose and Galactose Uptake and Utilization	Beta-galactosidase (EC 3.2.1.23)	SCE1572_6701	yes	sce_3424	yes
A and B	Carbohydrates	Di- and oligosaccharides	Lactose and Galactose Uptake and Utilization	Galactokinase (EC 2.7.1.6)	SCE1572_6700	yes	sce_2475	yes
A and B	Carbohydrates	Di- and oligosaccharides	Lactose and Galactose Uptake and Utilization	UDP-glucose 4-epimerase (EC 5.1.3.2)	SCE1572_1369	yes	sce_995	yes
A and B	Carbohydrates	Di- and oligosaccharides	Lactose utilization	Beta-galactosidase (EC 3.2.1.23)	SCE1572_6701	yes	sce_3424	yes
A and B	Carbohydrates	Di- and oligosaccharides	Lactose utilization	Galactoside O-acetyltransferase (EC 2.3.1.18)	SCE1572_8804	yes	sce_1535	yes
A and B	Carbohydrates	Di- and oligosaccharides	Maltose and Maltodextrin Utilization	4-alpha-glucanotransferase (amylomaltase) (EC 2.4.1.25)	SCE1572_586	yes	sce_396	yes
A and B	Carbohydrates	Di- and oligosaccharides	Maltose and Maltodextrin Utilization	Alpha-glucosidase (EC 3.2.1.20)	SCE1572_440	yes	sce_3862	yes
A and B	Carbohydrates	Di- and oligosaccharides	Maltose and Maltodextrin Utilization	Beta-phosphoglucomutase (EC 5.4.2.6)	SCE1572_11165	yes	sce_9891	yes
A and B	Carbohydrates	Di- and oligosaccharides	Maltose and Maltodextrin Utilization	Glucoamylase (EC 3.2.1.3)	SCE1572_5385	yes	sce_1738	yes
A and B	Carbohydrates	Di- and oligosaccharides	Maltose and Maltodextrin Utilization	Glycogen phosphorylase (EC 2.4.1.1)	SCE1572_5906	yes	sce_4909	yes
A and B	Carbohydrates	Di- and oligosaccharides	Maltose and Maltodextrin Utilization	Malto-oligosyltrehalose synthase (EC 5.4.99.15)	SCE1572_3181	yes	sce_2671	yes
A and B	Carbohydrates	Di- and oligosaccharides	Maltose and Maltodextrin Utilization	Maltodextrin glucosidase (EC 3.2.1.20)	SCE1572_762	yes	sce_92	yes
A and B	Carbohydrates	Di- and oligosaccharides	Maltose and Maltodextrin Utilization	Maltose O-acetyltransferase (EC 2.3.1.79)	SCE1572_8623	yes	sce_7345	yes
A and B	Carbohydrates	Di- and oligosaccharides	Maltose and Maltodextrin Utilization	Maltose phosphorylase (EC 2.4.1.8)	SCE1572_10442	yes	sce_9083	yes
A and B	Carbohydrates	Di- and oligosaccharides	Maltose and Maltodextrin Utilization	Maltose/maltodextrin ABC transporter, permease protein MalF	SCE1572_6788	yes	sce_5647	yes
A and B	Carbohydrates	Di- and oligosaccharides	Maltose and Maltodextrin Utilization	Maltose/maltodextrin ABC transporter, permease protein MalG	SCE1572_1171	yes	sce_5646	yes
A and B	Carbohydrates	Di- and oligosaccharides	Maltose and Maltodextrin Utilization	Maltose/maltodextrin ABC transporter, substrate binding periplasmic pr	SCE1572_6789	yes	sce_5648	yes
A and B	Carbohydrates	Di- and oligosaccharides	Trehalose Biosynthesis	1,4-alpha-glucan (glycogen) branching enzyme, GH-13-type (EC 2.4.1.	SCE1572_6074	yes	sce_5020	yes
A and B	Carbohydrates	Di- and oligosaccharides	Trehalose Biosynthesis	Alpha,alpha-trehalose-phosphate synthase [UDP-forming] (EC 2.4.1.15	SCE1572_746	yes	sce_549	yes
A and B	Carbohydrates	Di- and oligosaccharides	Trehalose Biosynthesis	Glucoamylase (EC 3.2.1.3)	SCE1572_5385	yes	sce_1738	yes
A and B	Carbohydrates	Di- and oligosaccharides	Trehalose Biosynthesis	Glycogen debranching enzyme (EC 3.2.1.-)	SCE1572_1926	yes	sce_1444	yes
A and B	Carbohydrates	Di- and oligosaccharides	Trehalose Biosynthesis	Malto-oligosyltrehalose synthase (EC 5.4.99.15)	SCE1572_3181	yes	sce_2671	yes
A and B	Carbohydrates	Di- and oligosaccharides	Trehalose Biosynthesis	Malto-oligosyltrehalose trehalohydrolase (EC 3.2.1.141)	SCE1572_3182	yes	sce_2672	yes
A and B	Carbohydrates	Di- and oligosaccharides	Trehalose Biosynthesis	Trehalose phosphorylase (EC 2.4.1.64)	SCE1572_10442	yes	sce_9083	yes

A and B	Carbohydrates	Di- and oligosaccharides	Trehalose Biosynthesis	Trehalose synthase (EC 5.4.99.16)	SCE1572_601	yes	sce_407	yes
A and B	Carbohydrates	Di- and oligosaccharides	Trehalose Biosynthesis	Trehalose-6-phosphate phosphatase (EC 3.1.3.12)	SCE1572_746	yes	sce_549	yes
A and B	Carbohydrates	Di- and oligosaccharides	Trehalose Uptake and Utilization	Beta-phosphoglucomutase (EC 5.4.2.6)	SCE1572_11165	yes	sce_9891	yes
A and B	Carbohydrates	Di- and oligosaccharides	Trehalose Uptake and Utilization	Glucose/mannose:H symporter GlcP	SCE1572_10391	yes	sce_9046	yes
A and B	Carbohydrates	Di- and oligosaccharides	Trehalose Uptake and Utilization	Trehalose phosphorylase (EC 2.4.1.64)	SCE1572_10442	yes	sce_9083	yes
A	Carbohydrates	Di- and oligosaccharides	Lactose and Galactose Uptake and Utilization	Galactose/methyl galactoside ABC transport system, ATP-binding prote	SCE1572_212	yes		no
A	Carbohydrates	Di- and oligosaccharides	Lactose and Galactose Uptake and Utilization	Galactose/methyl galactoside ABC transport system, permease protein M	SCE1572_211	yes		no
A	Carbohydrates	Di- and oligosaccharides	Trehalose Uptake and Utilization	PTS system, glucose-specific IIA component (EC 2.7.1.69)	SCE1572_3559	yes		no
B	Carbohydrates	Di- and oligosaccharides	Lactose and Galactose Uptake and Utilization	Aldose 1-epimerase (EC 5.1.3.3)		no	sce_9185	yes
B	Carbohydrates	Di- and oligosaccharides	Maltose and Maltodextrin Utilization	Maltose/maltodextrin transport ATP-binding protein MalK (EC 3.6.3.19)		no	sce_3429	yes
B	Carbohydrates	Di- and oligosaccharides	Maltose and Maltodextrin Utilization	Multiple sugar ABC transporter, ATP-binding protein		no	sce_3429	yes
B	Carbohydrates	Di- and oligosaccharides	Maltose and Maltodextrin Utilization	Periplasmic alpha-amylase (EC 3.2.1.1)		no	sce_7664	yes
A and B	Carbohydrates	Fermentation	Acetoin, butanediol metabolism	Acetolactate synthase large subunit (EC 2.2.1.6)	SCE1572_311	yes	sce_4182	yes
A and B	Carbohydrates	Fermentation	Acetoin, butanediol metabolism	Acetolactate synthase small subunit (EC 2.2.1.6)	SCE1572_4974	yes	sce_4183	yes
A and B	Carbohydrates	Fermentation	Acetyl-CoA fermentation to Butyrate	3-hydroxyacyl-CoA dehydrogenase (EC 1.1.1.35)	SCE1572_60	yes	sce_50	yes
A and B	Carbohydrates	Fermentation	Acetyl-CoA fermentation to Butyrate	3-hydroxybutyryl-CoA dehydratase (EC 4.2.1.55)	SCE1572_463	yes	sce_277	yes
A and B	Carbohydrates	Fermentation	Acetyl-CoA fermentation to Butyrate	3-hydroxybutyryl-CoA dehydrogenase (EC 1.1.1.157)	SCE1572_466	yes	sce_278	yes
A and B	Carbohydrates	Fermentation	Acetyl-CoA fermentation to Butyrate	3-hydroxybutyryl-CoA epimerase (EC 5.1.2.3)	SCE1572_60	yes	sce_50	yes
A and B	Carbohydrates	Fermentation	Acetyl-CoA fermentation to Butyrate	Acetyl-CoA acetyltransferase (EC 2.3.1.9)	SCE1572_61	yes	sce_51	yes
[truncated: 1,675,641 more chars]
